# Dissecting contributions of individual systemic inflammatory response syndrome criteria from a prospective algorithm to the prediction and diagnosis of sepsis in a polytrauma cohort

**DOI:** 10.3389/fmed.2023.1227031

**Published:** 2023-07-31

**Authors:** Roman Schefzik, Bianka Hahn, Verena Schneider-Lindner

**Affiliations:** Department of Anesthesiology and Surgical Intensive Care Medicine, Medical Faculty Mannheim, Heidelberg University, Mannheim, Germany

**Keywords:** diagnosis, intensive care medicine, logistic regression, polytrauma, prediction, sepsis, systemic inflammatory response syndrome, weighting

## Abstract

**Background:**

Sepsis is the leading cause of death in intensive care units (ICUs), and its timely detection and treatment improve clinical outcome and survival. Systemic inflammatory response syndrome (SIRS) refers to the concurrent fulfillment of at least two out of the following four clinical criteria: tachycardia, tachypnea, abnormal body temperature, and abnormal leukocyte count. While SIRS was controversially abandoned from the current sepsis definition, a dynamic SIRS representation still has potential for sepsis prediction and diagnosis.

**Objective:**

We retrospectively elucidate the individual contributions of the SIRS criteria in a polytrauma cohort from the post-surgical ICU of University Medical Center Mannheim (Germany).

**Methods:**

We used a dynamic and prospective SIRS algorithm tailored to the ICU setting by accounting for catecholamine therapy and mechanical ventilation. Two clinically relevant tasks are considered: (i) sepsis prediction using the first 24 h after admission to our ICU, and (ii) sepsis diagnosis using the last 24 h before sepsis onset and a time point of comparable ICU treatment duration for controls, respectively. We determine the importance of individual SIRS criteria by systematically varying criteria weights when summarizing the SIRS algorithm output with SIRS descriptors and assessing the classification performance of the resulting logistic regression models using a specifically developed ranking score.

**Results:**

Our models perform better for the diagnosis than the prediction task (maximum AUROC 0.816 vs. 0.693). Risk models containing only the SIRS level average mostly show reasonable performance across criteria weights, with prediction and diagnosis AUROCs ranging from 0.455 (weight on leukocyte criterion only) to 0.693 and 0.619 to 0.800, respectively. For sepsis prediction, temperature and tachypnea are the most important SIRS criteria, whereas the leukocytes criterion is least important and potentially even counterproductive. For sepsis diagnosis, all SIRS criteria are relevant, with the temperature criterion being most influential.

**Conclusion:**

SIRS is relevant for sepsis prediction and diagnosis in polytrauma, and no criterion should a priori be omitted. Hence, the original expert-defined SIRS criteria are valid, capturing important sepsis risk determinants. Our prospective SIRS algorithm provides dynamic determination of SIRS criteria and descriptors, allowing their integration in sepsis risk models also in other settings.

## 1. Introduction

Sepsis (1) is the leading cause of death in intensive care units (ICUs) and has an immense medical, societal and economic relevance (2, 3). As each hour of delayed effective antibiotic treatment increases mortality, timely detection and treatment of sepsis are crucial and improve clinical outcome and survival (4). On the other hand, unnecessary antibiotic treatment due to a wrong sepsis diagnosis may contribute to antimicrobial resistance (5, 6). Overall, prediction and early detection of sepsis are still challenging in the absence of suitable biomarkers and a gold-standard diagnostic test.

Sepsis originally had been defined as the systemic inflammatory response syndrome (SIRS) due to an infection (7), commonly referred to as Sepsis-1. In contrast, the latest consensus definition of sepsis, commonly referred to as Sepsis-3, specifies sepsis as a life-threatening organ dysfunction, caused by a dysregulated host response to an infection (8, 9). In particular, Sepsis-3 is not based on the SIRS concept anymore. However, this is controversially discussed, especially as Sepsis-3 has been developed as epidemiological measure of sepsis incidence rather than to support early detection of sepsis (10). SIRS remains an important predictor of sepsis (11) and is still a relevant topic of current research, see, e.g., (12), (13), or (14).

SIRS can arise due to various causes and includes, but is not limited to, more than one of the following four clinical manifestations (7): tachycardia (TC), tachypnea (TP), abnormal body temperature (Tem), and abnormal leukocyte (while blood cell) count (Leu). While originally not limited to those, SIRS is generally operationalized as meeting at least two out of the above four criteria concurrently. Previously, SIRS has typically been determined at time points in spot check evaluations only. Moreover, in ICU settings, tachypnea and tachycardia may be masked by interventions like mechanical ventilation and catecholamine therapy, respectively, which are not accounted for by the traditional SIRS definition. A first attempt to resolve these issues is given by the SIRS algorithm introduced in (15). However, this algorithm partly has a retrospective design, which impedes a real-time application for prediction at the bedside in a clinical decision support system (16). While the influence of individual SIRS criteria on ICU mortality has already been examined (17, 18), corresponding investigations in the context of sepsis are still lacking to our knowledge.

Patients with polytrauma, defined as multiple, potentially lethal injuries in typically more than one body region (19), are at high risk of sepsis (20–22). Although biomarkers for sepsis specifically for polytrauma patients have been proposed (23–25), the lack of valid, clinically applicable sepsis biomarkers also pertains to these patients. As polytrauma patients often develop SIRS due to a trauma-induced inflammatory response (26), they represent a population of high interest for research on SIRS and sepsis alike.

Therefore, we here report a detailed analysis of the role and relevance of the four SIRS criteria for sepsis prediction and diagnosis in a cohort of polytrauma patients. Our aim is to determine whether some of the four SIRS criteria have a more pronounced influence than others in the context of two distinct, clinically relevant tasks, namely (i) sepsis prediction, considering the first 24 h after ICU admission, and (ii) sepsis diagnosis, considering the last 24 h before sepsis onset. We also investigate how the results for different weighting schemes relate to those for the hitherto common usage of SIRS, i.e., a scenario of equal weighting. For this, we introduce a novel adaptation of the approach by (15), for providing a time-dependent summary of SIRS criteria as SIRS descriptors. Our enhanced algorithm, referred to as SIRS Prospective, uses a dynamic, time interval-based concept of SIRS specifically tailored to the ICU treatment context by explicitly accounting for catecholamine therapy and mechanical ventilation. While applied to electronic medical records in retrospective studies here, our SIRS algorithm itself is designed in a prospective manner and generally suitable for analyses of real-time data streams, e.g., in clinical decision support systems.

## 2. Methods and data

### 2.1. Polytrauma cohort and sepsis (outcome) definition

In our retrospective studies, we consider a polytrauma cohort based on data from the post-surgical ICU of the University Medical Centre Mannheim, Germany, where the cohort at hand has been identified as follows. Among all valid admissions to the ICU from April 2006 to December 2016, we first selected those that had free text entries corresponding to the German expressions for “fall”, “fracture”, “accident”, or “trauma” in their electronic medical record. From the remaining patients, we then kept those in our cohort that had recent injuries in more than one body region as admission reason and had an injury severity score greater than 15. These patients were defined as having polytrauma (27). We further reduced this cohort by excluding patients that were younger than 16 years. Moreover, as the focus of our studies is the first ICU stay after trauma incidence, we only included this period in our analyses. We also excluded patients (i) whose day of trauma onset is more than 1 day before the start of the ICU stay, (ii) whose first period of ICU stay is less than 24 h, or (iii) for which the difference between the start of the ICU stay and the first sepsis diagnosis time point is less than 24 h.

In this context, the trauma onset day has been determined using both a computational and manual screening of patient anamnesis and hospital records, and the time point of sepsis treatment initiation was taken as the sepsis onset time point. Further, we here defined the starting point of the ICU stay (ICU admission time point) as the first chart time with a valid heart rate or peripheral, pulsoxymetrically measured oxygen saturation (SpO_2_) value. The end of a patient's ICU stay is determined in a similar way.

Sepsis as the outcome in our studies was defined based on clinical validation in our paper as follows: The ICU electronic medical records were comprehensively reviewed by experienced intensive care physicians, who manually searched for entries related to sepsis-specific antibacterial treatment. Patients with a corresponding entry were defined as having sepsis. Moreover, the corresponding sepsis diagnosis time was defined as the time of the first order of the antibacterial in the electronic medical record.

Using the above selection strategy and sepsis definition, our final polytrauma cohort consists of 415 encounters in total, 143 of which developed sepsis (34%), and 272 of which did not develop sepsis (66%). Basic characteristics of the patients in our polytrauma cohort are summarized in Table 1.

**Table 1:** Patient characteristics for our polytrauma cohort consisting of in total 415 patients, where the data is represented in the form mean ± sd or n (% of N), and *p*-values for comparisons between the sepsis and no sepsis group are derived using t-tests or χ^2^-tests, respectively.

**Characteristic**	**Sepsis (N = 143)**	**No sepsis (N = 272)**	***P*-value**
**Basic characteristics**			
Age (in years)	50.9 ± 19.8	48.7 ± 20.0	0.2888
Men	118 (82.5%)	190 (69.9%)	0.0051
ICU length-of-stay (in days)	25.4 ± 17.8	8.5 ± 6.8	< 0.0001
ICU mortality	28 (19.6%)	20 (7.4%)	0.0002
**Acute condition**			
Glasgow Coma Scale	9.7 ± 5.0	11.2 ± 4.5	0.0022
Simplified Acute Physiology Score II	33.6 ± 10.5	27.4 ± 9.0	< 0.0001
	[8 (5.6%) missing]	[18 (6.6%) missing]	
Injury Severity Score	35.7 ± 8.6	32.3 ± 8.1	0.0002
AIS abdomen	1.7 ± 1.8	1.2 ± 1.6	0.0101
AIS extremities	2.5 ± 1.5	2.3 ± 1.5	0.4483
AIS face	1.1 ± 1.3	1.1 ± 1.4	0.6443
AIS head	2.4 ± 1.9	2.2 ± 1.9	0.2988
AIS thorax	3.0 ± 1.5	2.7 ± 1.5	0.0521
AIS soft tissue	2.0 ± 0.8	1.9 ± 0.8	0.0943
**Chronic condition on admission**			
Alcoholism	27 (18.9%)	27 (9.9%)	0.0100
Cardiovascular diseases	33 (23.1%)	26 (9.6%)	0.0002
Diabetes	17 (11.9%)	19 (7.0%)	0.0917
Respiratory diseases	7 (4.9%)	7 (2.6%)	0.2549^*^

AIS, abbreviated injury scale; ICU, intensive care unit. ^*^P-value derived using Fisher's exact test.

### 2.2. Prospective SIRS algorithm

We introduce an algorithm to adequately describe the SIRS phenomenon and determine the validity of the individual SIRS criteria, namely tachycardia (TC), tachypnea (TP), abnormal body temperature (Tem), and abnormal leukocyte (white blood cell) count (Leu). In particular, the presented algorithm allows for a dynamic description of SIRS and is specifically tailored to the setting of an ICU by explicitly accounting for ICU-specific catecholamine therapies and mechanical ventilation when evaluating tachycardia and tachypnea, respectively, in contrast to the original SIRS definition by (7). First attempts into this direction have been made by (15). However, their algorithm is partly designed in a retrospective manner, in that the validity of some of the SIRS criteria at a fixed time point is determined by taking account of future values. This consequently hampers a real-time clinical application of their tool. To address this shortcoming, we here adapt the SIRS algorithm of (15) by developing it from a retrospective to a prospective tool, which allows for the evaluation of SIRS criteria at a time point of interest without the need to consider future events. As part of this, we provide a simplification and harmonization of the previous algorithm rules in (15) by putting them in a general overarching frame which is then specifically elaborated for the individual SIRS criteria (Table 2).

**Table 2:** Prospective SIRS algorithm rules.

**SIRS criterion**	**Subcriterion**	**(Sub)criterion fulfilled if**	**Maximum validity length**
Tachycardia (TC) ^*^	(i) heart rate η (ii) catecholamine dose *d*, *d* = *d*_Nor_+*d*_Adr_+*d*_Dob_	η>90/min*d*>0μg/min	0.5 h1 h
Tachypnea (TP) ^*^	(i) respiratory rate ν † (ii) PaCO_2_ ρ † (iii) expired minute volume EMV	ν>20/min ρ < 32 mmHg EMV >0 L/min	0.5 h § 8 h § 1 h
Temperature (Tem) ϑ		ϑ∉[36, 38]°C	4 h
Leukocyte count (Leu) *l*		*l*∉[4000, 12000]/μL	24 h

The conditions for the fulfillment of a SIRS (sub)criterion (3rd column) are taken from (7) (except from catecholamine dose and EMV), while the maximum validity lengths (4th column) are determined based on clinical expertise. Adr, adrenaline; Dob, dobutamine; Nor, noradrenaline; PaCO_2_, partial pressure of carbon dioxide in arterial blood.

^*^TC/TP criterion is fulfilled if at least one of the respective subcriteria is fulfilled.

†Measurement only valid if there is no EMV record > 0 within the preceding hour.

§Possibly, a subsequent EMV record > 0 ends validity earlier.

The general rationale of our novel, prospective SIRS algorithm, referred to as SIRS Prospective, essentially consists of two steps. First, for each measurement of an involved vital or laboratory parameter, we check whether the respective value deviates from a pre-defined range indicating typical measurements that are clinically to be expected for healthy people (7). If this is the case, then the corresponding SIRS criterion is considered to be fulfilled, and criterion validity starts with the chart time of the measurement. Second, SIRS criterion validity is designed to last until a corresponding subsequent measurement is charted, leading to a re-evaluation of criterion validity according to the first step. However, maximum validity intervals for SIRS criteria are implemented, depending on the considered variable and determined in accordance with clinical expertise. In addition, possible interplays between variables for the tachypnea criterion are explicitly accounted for. The explicit rules of the SIRS Prospective algorithm for the four individual SIRS criteria are as follows:

#### 2.2.1. Tachycardia criterion (TC)

The TC criterion is fulfilled if at least one of the following two subcriteria is fulfilled: (i) the heart rate criterion or (ii) the catecholamine criterion.

*(i) Heart rate criterion*. Each heart rate record > 90 beats per minute starts criterion validity for 30 min if no measurement ≤ 90 beats per minute ends criterion validity earlier.

*(ii) Catecholamine criterion*. Here, we consider a patient to receive a catecholamine therapy if doses of adrenaline, noradrenaline or dobutamine are administered. Each dose record > 0 μg/min of adrenaline, noradrenaline or dobutamine starts criterion validity for 30 min.

#### 2.2.2. Tachypnea criterion (TP)

The TP criterion is fulfilled if at least one of the following three subcriteria is fulfilled: (i) the EMV (expired minute volume) criterion indicating mechanical ventilation, (ii) the respiratory rate criterion or (iii) the PaCO_2_ (partial pressure of carbon dioxide in arterial blood) criterion.

*(i) EMV (expired minute volume)/mechanical ventilation criterion*. Here, we evaluate the presence of mechanical ventilation of a patient by considering records of the EMV. Each EMV record > 0 L starts criterion validity for 1 h.

*(ii) Respiratory rate criterion*. Each respiratory rate record > 20 breaths per minute without an EMV record > 0 L within the preceding hour starts criterion validity for 30 min unless a subsequent respiratory rate record ≤ 20 breaths per minute or an EMV record > 0 L ends criterion validity earlier.

*(iii) PaCO*_2_ *(partial pressure of carbon dioxide in arterial blood) criterion*. Each PaCO_2_ record < 32mmHg without an EMV record > 0 L within the preceding hour starts criterion validity for 8 h unless a subsequent PaCO_2_ record ≥ 32 mmHg or an EMV record > 0 L ends the criterion validity earlier.

#### 2.2.3. Temperature criterion (Tem)

Each temperature record < 36°C or > 38°C starts criterion validity for 4 h if no measurement ≥ 36°C or ≤ 38°C ends validity earlier.

Note that temperature records ≤ 29°C are excluded from our analyses here, as these likely mirror a wrong recording of ambient temperature, e.g., due to sensor dislocation. Similarly, also values ≥ 42.5°C are excluded from our studies.

#### 2.2.4. Leukocytes criterion (Leu)

Each leukocyte count < 4000/μL or > 12000/μL starts criterion validity for 24 h if no measurement that lies within the interval [4000, 12000]/μL ends validity earlier.

Note that we here do *not* additionally consider a further subcriterion from the original SIRS definition by (7) which suggests the validity of the leukocyte criterion if there exist > 10% immature (band) forms.

The rules of our prospective SIRS algorithm and their overarching frame as presented above are summarized in Table 2. At this point, we emphasize again that our algorithm itself, as indicated by its name, has a prospective design, but the studies in which it will be applied here have a retrospecive design.

### 2.3. SIRS levels and weighting schemes for SIRS criteria

Traditionally, the SIRS level λ^*^ at a given time point is defined as the number of SIRS criteria (out of the criteria TC, TP, Tem, and Leu) that are fulfilled concurrently, and SIRS is diagnosed if at least two out of the four SIRS criteria are fulfilled simultaneously. To investigate the individual contributions of the four SIRS criteria in the context of sepsis prediction and diagnosis, we here adapt the SIRS level concept to the needs for our analyses. Non-technically speaking, we assign weights *w*_TC_, *w*_TP_, *w*_Tem_, and *w*_Leu_, respectively, between 0 and 1 to each SIRS criterion. We then define the SIRS level λ at a given time point as the sum of the weights of the fulfilled criteria.

**Figure 1:**
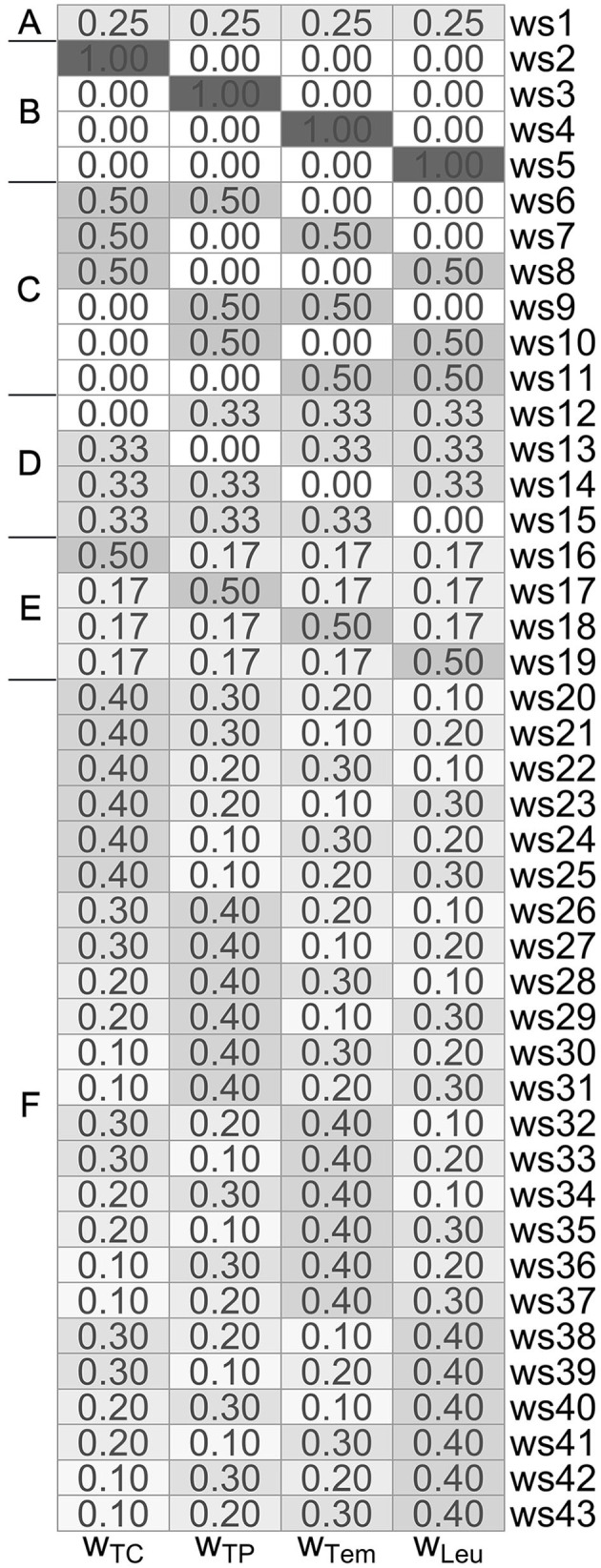
Overview of the differently composed (type **A–F**) weighting schemes ws1-ws43, consisting of corresponding weights *w*_TC_, *w*_TP_, *w*_Tem_, and *w*_Leu_ for the tachycardia, tachypnea, temperature and leukocytes criterion, respectively.

Specifically, in our studies, we employ different weighting schemes (i.e., weights *w*_TC_, *w*_TP_, *w*_Tem_, *w*_Leu_) for the four SIRS criteria as summarized in Figure 1, where we consider differently composed weightings of the following types:

- Type A: all four SIRS criteria have equal weight of 1/4 each,
- Type B: exactly one SIRS criterion has full weight of 1, and the remaining three have zero weight,
- Type C: exactly two SIRS criteria have a weight of 1/2 each, and the remaining two have zero weight,
- Type D: exactly three SIRS criteria have a weight of 1/3 each, and the remaining one has zero weight,
- Type E: exactly one SIRS criterion has a dominant high weight of 1/2, and the remaining three have a weight of 1/6 each,
- Type F: the weights of the SIRS criteria are gradually varying, attaining values of 0.4, 0.3, 0.2, and 0.1.

In what follows, we formalize and describe our approach in a more technical framework. In a given minute ℓ, the SIRS level $\lambda^{*}:=\lambda_{\ell }^{*}$ is traditionally derived by


$$\begin{array}{l} \lambda^{*}:={𝟙}_{\mathrm{TC}}+{𝟙}_{\mathrm{TP}}+{𝟙}_{\mathrm{Tem}}+{𝟙}_{\mathrm{Leu}}\in \;\left\{0,1,2,3,4\right\}\;, \end{array}$$

where


$${𝟙}_{c}:=\{\begin{array}{ll} 1, & \text{ criterion }c\text{ fulfilled}\\ 0, & \text{ criterion }c\text{ not fulfilled} \end{array}$$

for criterion *c* ∈ {TC, TP, Tem, Leu}, and SIRS is then diagnosed if λ^*^ ≥ 2. Thus, the SIRS level λ^*^ can only attain a discrete spectrum of values (namely 0, 1, 2, 3 or 4).

To adapt this concept to the setting of our analyses, we now introduce non-negative weights


$$\begin{array}{l} w_{\mathrm{TC}},\;w_{\mathrm{TP}},\;w_{\mathrm{Tem}},\;w_{\mathrm{Leu}}\geq 0 \end{array}$$

for the four SIRS criteria, where we follow the common definition of weights assuming that


$$\begin{array}{l} w_{\mathrm{TC}}+w_{\mathrm{TP}}+w_{\mathrm{Tem}}+w_{\mathrm{Leu}}=1. \end{array}$$

We then define the SIRS level λ: = λ_ℓ_ in a given minute ℓ as follows:


$$\begin{array}{l} \lambda :=w_{\mathrm{TC}}{𝟙}_{\mathrm{TC}}+w_{\mathrm{TP}}{𝟙}_{\mathrm{TP}}+w_{\mathrm{Tem}}{𝟙}_{\mathrm{Tem}}+w_{\mathrm{Leu}}{𝟙}_{\mathrm{Leu}}\in \left[0,1\right]. \end{array}$$

In this definition here, the SIRS level λ can attain values in [0, 1], and we can therefore think of λ as a SIRS intensity level here. Thus, we proceed from the traditional consideration of SIRS levels λ^*^ ∈ {0, 1, 2, 3, 4} (with SIRS diagnosis if λ^*^ ≥ 2) to a SIRS level spectrum λ ∈ [0, 1], with values of λ depending on the choice of the weights.

Despite of our aim to investigate the effect of giving different weights to the four SIRS criteria, the common concept of equal weighting (Type A) used in weighting scheme 1 (ws1) from Figure 1 still has our specific focus for several reasons. First, we will use it as a (traditional) reference approach against which we compare the other weighting schemes. Second, the equal weighting scheme allows for a straightforward interpretation, as it (is the only weighting scheme that) directly relates to the number of fulfilled SIRS criteria. Specifically, for ws1, a λ value of 0, 0.25, 0.5, 0.75 and 1 corresponds to the concurrent fulfillment of 0, 1, 2, 3 and 4 SIRS criteria, respectively. In particular, in the framework of our formulation here, in the common case of equal weighting *w*_TC_ = *w*_TP_ = *w*_Tem_ = *w*_Leu_: = 1/4 (ws1), we have a SIRS diagnosis if


$$\begin{array}{l} \lambda =\frac{1}{4}\left({𝟙}_{\mathrm{TC}}+{𝟙}_{\mathrm{TP}}+{𝟙}_{\mathrm{Tem}}+{𝟙}_{\mathrm{Leu}}\right)\geq \frac{1}{2}. \end{array}$$

Analogously, the threshold value of 0.5 for λ could also be applied to the other weighting scheme settings, and we may generally think of a SIRS diagnosis if λ ≥ 0.5 in our new framework.

### 2.4. SIRS descriptors

We here introduce three summary measures Λ, Δ and *C*, which we use as SIRS descriptors (15) to describe the output of our SIRS algorithm over a pre-defined time period consisting of *L* consecutive minutes:

- Λ refers to the average SIRS level over the considered time period,
- Δ refers to the SIRS level trend in the time period, comparing the levels of the first and the last (*L*-th) considered minutes, and
- *C* refers to the number of changes in the SIRS level, reflecting the degree of SIRS level fluctuation within the time period.

The SIRS descriptors Λ, Δ and *C* represent intuitive and well-established measures. For instance, the mean (Λ) has been shown to be a powerful time series summary statistics for clinical disease prediction tasks in a recent study by (28). While additional summary measures are available (29), we do not use them here, as initial considerations did not yield any benefit.

**Figure 2:**
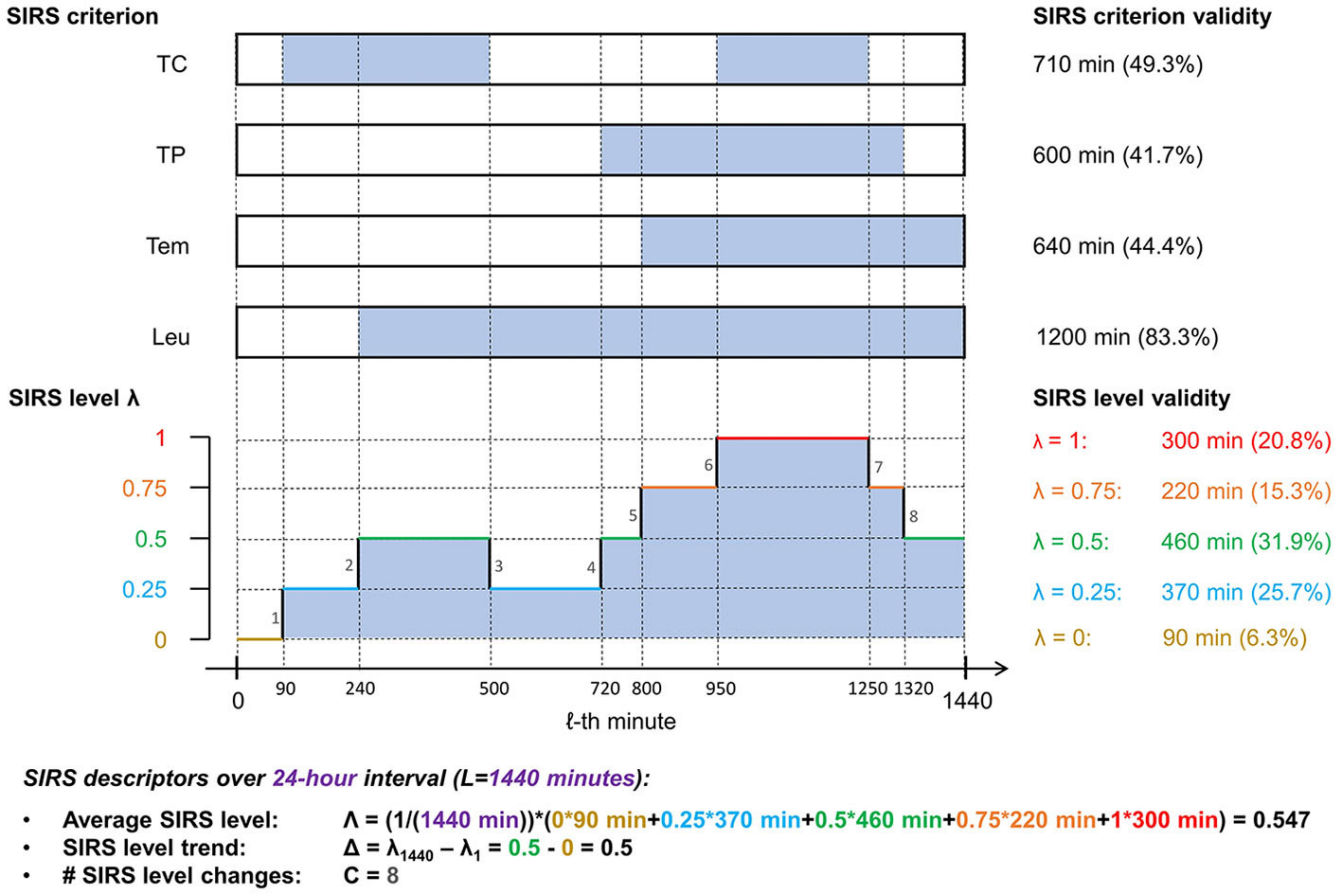
Illustration of the SIRS algorithm output and the corresponding summarizing SIRS descriptors for a fictitious encounter over an observation period of 24 h (*L* = 1, 440 min), using exemplarily the equal weighting scheme (ws1), which gives a weight of 0.25 to each of the four SIRS criteria (TC, tachycardia; TP, tachypnea; Tem, temperature; Leu, leukocytes): At the top, time spans of validity of a respective SIRS criterion are indicated by filled sections in the corresponding boxes. Below, the corresponding SIRS levels λ for each minute of the observation period are shown. Note that in the specific present case of equal weighting of the SIRS criteria, the SIRS levels λ are directly related to the number of concomitantly fulfilled SIRS criteria (in that a λ value of 0, 0.25, 0.5, 0.75, and 1 corresponds to the concurrent fulfillment of 0, 1, 2, 3, and 4 SIRS criteria, respectively), thus reflecting the traditional view of the SIRS phenomenon.

An illustration of the SIRS algorithm output and the descriptors is given in Figure 2.

Technically, if λ_1_, λ_2_, …, λ_*L*_ ∈ [0, 1] denotes a series of *L* (time-ordered) SIRS levels, such that level λ_1_ is valid in the first considered minute, level λ_2_ is valid in the second considered minute, …, level λ_*L*_ is valid in the last (*L*-th) considered minute, then

- $\Lambda :=\frac{1}{L}\sum_{\ell =1}^{L}\lambda_{\ell }\in [0,1],$,
- Δ: = λ_*L*_ − λ_1_ ∈ [−1, 1], and
- $C:=\sum_{\ell =1}^{L-1}{𝟙}_{\left\{\lambda_{\ell }\neq \lambda_{\ell +1}\right\}}\in \{0,1,\ldots ,L-1\}$.

In our analyses, we focus on 24-h periods comprising *L*: = 1, 440 min, by considering the first 24 h after ICU admission (prediction task) and the last 24 h before an index (sepsis) time point (diagnosis task), respectively.

### 2.5. Sepsis prediction and diagnosis tasks

Based on the output by the SIRS Prospective algorithm, we calculate the SIRS descriptors (average SIRS level Λ, SIRS level trend Δ, number of changes in SIRS level *C*) for the different weighting schemes and evaluate their discriminative performance by using them for the clinically relevant tasks of sepsis prediction and sepsis diagnosis, respectively (15):

1. Sepsis *prediction* refers to the time period of the first 24 h after ICU admission, with the aim of predicting the development of sepsis during a patient's further ICU stay. Here, the distinction between case and control group is made irrespective of the follow-up time. That is, we take the 143 encounters developing sepsis at any point during their time in the ICU as the cases (referred to as the sepsis group throughout the paper) and the 272 encounters not developing sepsis during that time as the controls (referred to as the no sepsis group throughout the paper).
2. Sepsis *diagnosis* refers to the time period of the last 24 h before an index time point, which corresponds to
  - the sepsis onset (time point) for patients developing sepsis (case group), and
  - a time point of comparable ICU treatment duration in a control group,

for comparison of both groups. Here, we also take the 143 encounters developing sepsis as the cases (referred to as the sepsis group throughout the paper). However, the controls (referred to as the control group throughout the paper) are derived by matching encounters to the sepsis cases according to their ICU length-of-stay in a nested case-control study design (30). Specifically, for each septic encounter, controls are identified as all ICU admissions of the cohort treated in the ICU for at least as long as the given septic patient, independent of a possible later development of sepsis. This way, we construct risk sets, each consisting of a septic encounter and all admissions with matching on length-of-stay as controls, so that the total number of controls is much greater than the number of admissions. In each risk set, we then calculate the index time for each control as the sum of the ICU admission time and treatment duration of its corresponding septic encounter. Using this strategy for our diagnosis task, we finally end up with in total 29721 controls derived from the risk sets, noting that two septic patients have an identical length-of-stay in the ICU until the sepsis onset and thus an identical risk set, such that we have 143 septic patients in 142 strata.

### 2.6. Models and evaluation techniques

We first employ basic tools from descriptive statistics to summarize the simultaneous fulfillment of the four SIRS criteria as well as the distributions and properties of the SIRS descriptors Λ, Δ, and *C* for each of our weighting schemes in the prediction and diagnosis task, respectively. For comparisons between the sepsis and no sepsis/control groups in this context, the two-sided Wilcoxon rank sum test is used to check for significant differences with respect to the mean. Additionally, we employ the waddR tool (31) based on Wasserstein distances to test for corresponding differences between whole distributions (location and variability) of the SIRS descriptors.

To investigate the impact of the output of our SIRS algorithm on sepsis prediction and diagnosis, for each of our weighting schemes, we consider the following different logistic regression models with a binary response variable *S* indicating whether sepsis occurs or not, and the derived SIRS descriptors Λ, Δ and *C* as predictor variables (and also an intercept term, which we omit in the formulas for convenience):


$$\begin{array}{l} S\sim \Lambda ,\;S\sim \Delta ,\;S\sim C,\;S\sim \Lambda +\Delta ,\;S\sim \Lambda +C,\;S\sim \Delta +C\text{ and }S\sim \Lambda +\Delta +C. \end{array}$$

For model validation, we here use 10-fold cross validation.

We mainly evaluate the discriminative performance of our logistic regression models using area under the receiver operating characteristic curve (AUROC) values, basically balancing sensitivity and specificity. However, for the sake of completeness, we also separately assess sensitivity and specificity of the models, where the predicted sepsis probabilities yielding the maximum sum of sensitivity and specificity from the ROC curve data are employed as model-based cutoffs here. However, we note that in principle the choice of probability threshold based on clinical expertise (32) is important for decision making and model calibration (33).

Additionally, we evaluate the predictive performance of our models by looking at calibration, see, e.g., (34) for an overview. In particular, we consider both the slopes and the intercepts of probability calibration plots (35, 36). Here, reference targets for a good performance are a slope value of 1 and an intercept value of 0, i.e., the calibration plot should be a curve close to the diagonal, such that predicted risks correspond well to observed proportions. As performance evaluation measures for calibration, we consider the distance of the calibration slope to the reference value of 1, referred to as DistSlope, as well as the distance of the calibration intercept to the reference value of 0, referred to as DistIntercept.

Both models and weighting schemes are assessed based on their relative performance to their competitors, where a performance/importance ranking is made using a specifically proposed ranking score as a quantitative scoring system (Supplementary Section 1 in Supplementary material 1).

We here focus on logistic regression models since these are quite basic, clear and easy to communicate to practitioners. Moreover, it has been witnessed that there is virtually no benefit of using more complex machine learning methods over logistic regression (37). In particular, logistic regression has shown good calibration compared to other approaches (38).

Despite an existent imbalance between the sizes of the sepsis and no sepsis/control groups, in particular for the diagnosis task, we do not use imbalance correction approaches (such as weighted logistic regression) in our models here, as it has been shown by (39) that such methods typically do not improve AUROC values (which are our main evaluation measure here) and even deteriorate calibration in terms of the slope and intercept of probability calibration plots, compared to uncorrected data. Initial tests for the diagnosis task (not explicitly shown) suggest that these findings are confirmed for our polytrauma cohort data.

For the prediction task, we additionally consider Cox proportional hazards (PH) models (40) for time-to-event analysis, where the event in our setting corresponds to the sepsis time point. Specifically, for each weighting scheme, we consider a multivariable Cox PH model (*T, S*) ~ Λ + Δ + *C*, with the SIRS descriptors Λ, Δ and *C* being derived based on the first 24 h after ICU admission. Here, *T* refers to the time-to-event, i.e., the time to sepsis onset (*S* = 1; 143 septic encounters) or the end of the ICU stay (*S* = 0; 272 non-septic encounters).

### 2.7. Reference algorithms

To highlight the benefit of our novel, prospective SIRS algorithm introduced above, referred to as *SIRS Prospective*, we compare its results to those obtained by the following related reference algorithms:

- *SIRS Conventional*: This very basic algorithm uses the traditional SIRS definition and predicts sepsis when ≥ 2 SIRS criteria are concurrently fulfilled for ≥ 1 min in the considered time period. Transferred to the context of the paper here, sepsis is predicted when the SIRS level λ is ≥ 0.5 for ≥ 1 min.
- *SIRS Non-ICU*: This algorithm corresponds to the SIRS Prospective algorithm, but *without* accounting for catecholamine therapy and ventilatory support in the tachycardia and tachypnea criteria, respectively. Thus, it can be seen as variant of our SIRS algorithm in a non-ICU-specific setting. In particular, for the temperature and leukocytes criteria, the rules of the SIRS Non-ICU algorithm exactly correspond to those of the SIRS Prospective algorithm. This implies that the results for these two algorithms coincide when weight is only given to the temperature and/or leukocytes criteria (i.e., for weighting schemes ws4, ws5, and ws11; Figure 1).
- *SIRS Retrospective*: This algorithm is a former, retrospective version of our SIRS algorithm, which had been introduced in (15). While SIRS Prospective takes measurements at face value, SIRS Retrospective is a more conservative approach, which is more strict to allow SIRS criteria to be fulfilled. In particular, SIRS Retrospective partly makes use of future measurement values to derive the validity of SIRS criteria and thus is not suitable for a potential real-time application, in contrast to the SIRS Prospective approach.

## 3. Results

### 3.1. Prediction task

#### 3.1.1. Basic analyses and descriptive statistics

**Figure 3:**
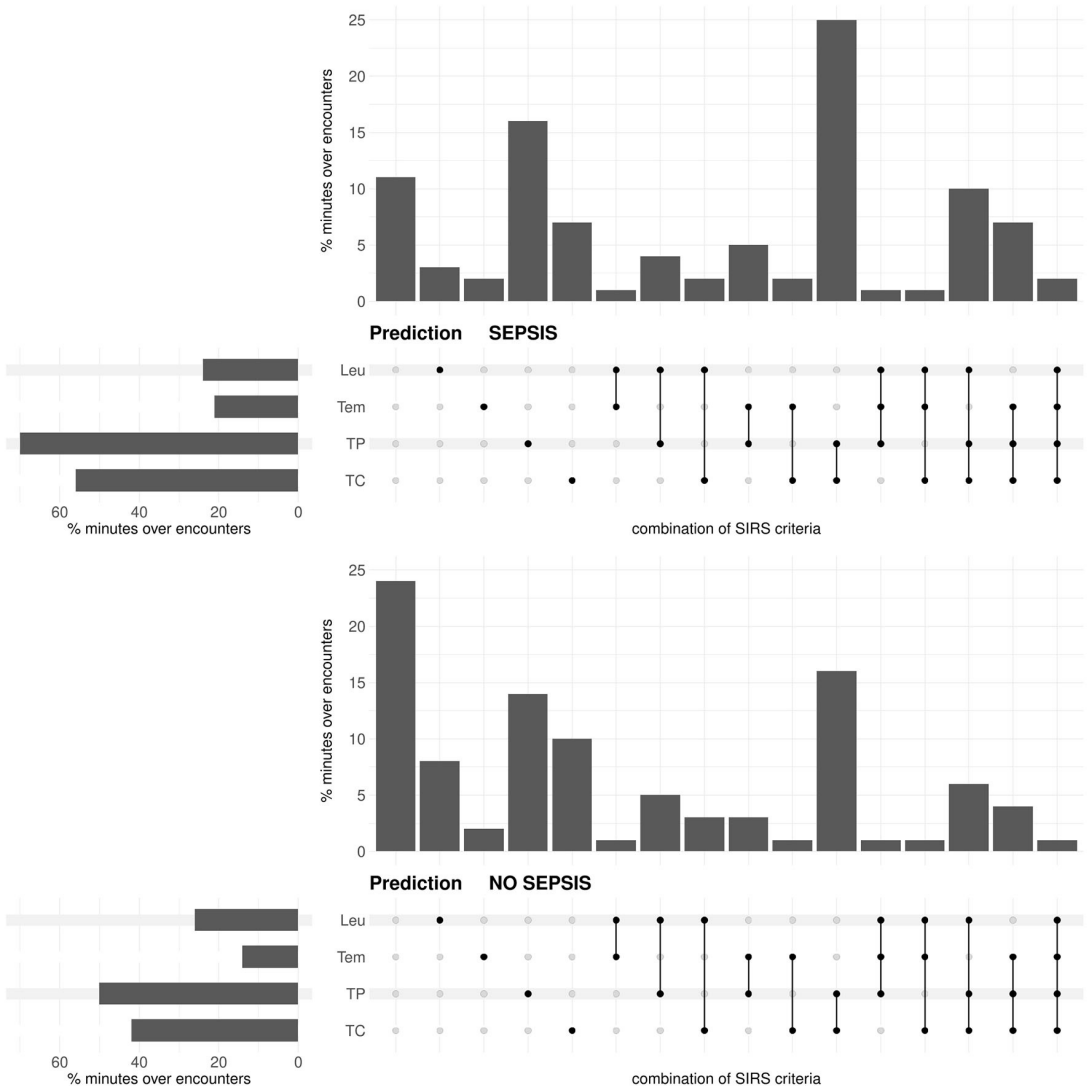
Prediction task: Upset plot-like figure relating to the frequencies of fulfilled SIRS criteria (TC, tachycardia; TP, tachypnea; Tem, temperature; Leu, leukocytes; indicated by the black dots), represented in the form of percentages of minutes over encounters, i.e., as percentages of in total 143 × 1440 = 205920 min for the sepsis group and 272 × 1440 = 391680 min for the no sepsis group, respectively.

In the prediction task, for both the sepsis and the no sepsis group, the tachypnea and the tachycardia criteria are fulfilled most often, then the leukocytes and the temperature criteria (Figure 3). The situation that either only the tachypnea or only the tachycardia or only the temperature criterion is fulfilled occurs more frequently in the sepsis group, whereas the situation that only the leukocytes criterion is fulfilled occurs more frequently in the no sepsis group (Figure 3; respective *P*-values *P* < 0.0001 from χ^2^-tests). Meaningfully, the situation that there is no fulfilled SIRS criterion clearly occurs more frequently in the no sepsis group (Figure 3; *P* < 0.0001 from a χ^2^-test). The simultaneous fulfillment of only the tachypnea and the tachycardia criterion constitutes the most blatant example where the occurrence is higher in the sepsis group than in the no sepsis group (Figure 3; *P* < 0.0001 from a χ^2^-test).

For the standard equal weighting scheme (ws1), summaries of the distributions of the SIRS descriptor values Λ, Δ and *C* for the sepsis and no sepsis groups in the prediction task are given in Table 3. Corresponding boxplots for all weighting schemes can be found in Supplementary material 1 (Supplementary Figures 1–3), accompanied by results for the respective Wilcoxon rank sum tests (Supplementary Figure 4 in Supplementary material 1) and the alternative waddR tool [(31); Supplementary Figure 5 in Supplementary material 1]. The values of the SIRS descriptor Λ are significantly different between the sepsis group and the no sepsis group (namely, significantly greater for the sepsis group) for all weighting schemes except for ws5, giving weight to the leukocytes criterion only. The values of the SIRS descriptor Δ are significantly different between the sepsis group and the no sepsis group (namely, significantly greater for the sepsis group) for a bit less than 1/4 of the weighting schemes. In particular, significant differences in terms of Δ (i.e., for ws3, ws6, ws14, ws17, ws21, ws23, ws26-ws29) appear to be mainly driven by a high weight on the tachypnea criterion. The values of the SIRS descriptor *C* are significantly different between the sepsis group and the no sepsis group for most (84%) weighting schemes. In case of a significant difference, the values for *C* are greater in the no sepsis group for all weighting schemes except for ws4 and ws11, for which *C* is significantly greater in the sepsis group. Hence, significantly greater values of *C* for the sepsis group appear to be driven by the temperature criterion, whereas significantly greater values of *C* for the no sepsis group appear to be driven by the tachycardia and tachypnea criteria. Note that the only weighting scheme for which all three SIRS descriptors are not significantly different between the sepsis and the no sepsis group is ws5.

**Table 3:** Prediction and diagnosis task: Mean ± sd of the SIRS descriptors Λ, Δ, and *C* for the equal weighting scheme (ws1) for the sepsis and no sepsis/control group, where the *P*-values for comparison are derived using a two-sided Wilcoxon rank sum test.

		**Sepsis**	**No sepsis/ Controls**	***P*-value**
	Λ	0.43 ± 0.16	0.33 ± 0.18	< 0.0001
Prediction	Δ	0.17 ± 0.33	0.10 ± 0.33	0.0831
	*C*	14.1 ± 8.9	16.8 ± 10.5	0.0219
	Λ	0.53 ± 0.19	0.32 ± 0.20	< 0.0001
Diagnosis	Δ	0.13 ± 0.27	0.00 ± 0.24	< 0.0001
	*C*	13.0 ± 10.7	14.1 ± 10.3	0.0796

#### 3.1.2. Model and weighting scheme performances

In the univariable logistic regression model *S* ~ Λ, Λ is a significant predictor for all weighting schemes except for ws5 and ws11, in which the leukocytes criterion has a high weight (Supplementary Figure 6 in Supplementary material 1) [e.g., for ws1: odds ratio (OR): 2.27 [95% confidence interval (CI): 1.69–3.09] for 1/4-unit change; *P* < 0.0001].

In the *S* ~ Δ model, Δ is a significant predictor for weighting schemes in which the tachycardia and/or the tachypnea criterion have a high weight (Supplementary Figure 6 in Supplementary material 1) [e.g., for ws1: OR: 1.15 [95% CI: 0.99–1.35] for 1/4-unit change; *P* = 0.0702].

In the *S* ~ *C* model, *C* is a significant predictor for all but five weighting schemes (Supplementary Figure 6 in Supplementary material 1) [e.g., for ws1: OR: 0.97 [95% CI: 0.95-0.99]; *P* = 0.0111].

In the multivariable logistic regression model *S* ~ Λ + Δ + *C*, Λ is a significant predictor for all weighting schemes except for ws4, ws5 and ws11, in which the tachypnea and the tachycardia criteria have zero weight and the temperature and/or the leukocytes criteria a high weight (Supplementary Figure 7 in Supplementary material 1). Moreover, Δ is only a significant predictor for ws4, in which all weight is given to the temperature criterion, and *C* typically is a significant predictor for those weighting schemes in which the tachypnea criterion has zero or a low weight (Supplementary Figure 7 in Supplementary material 1).

**Figure 4:**
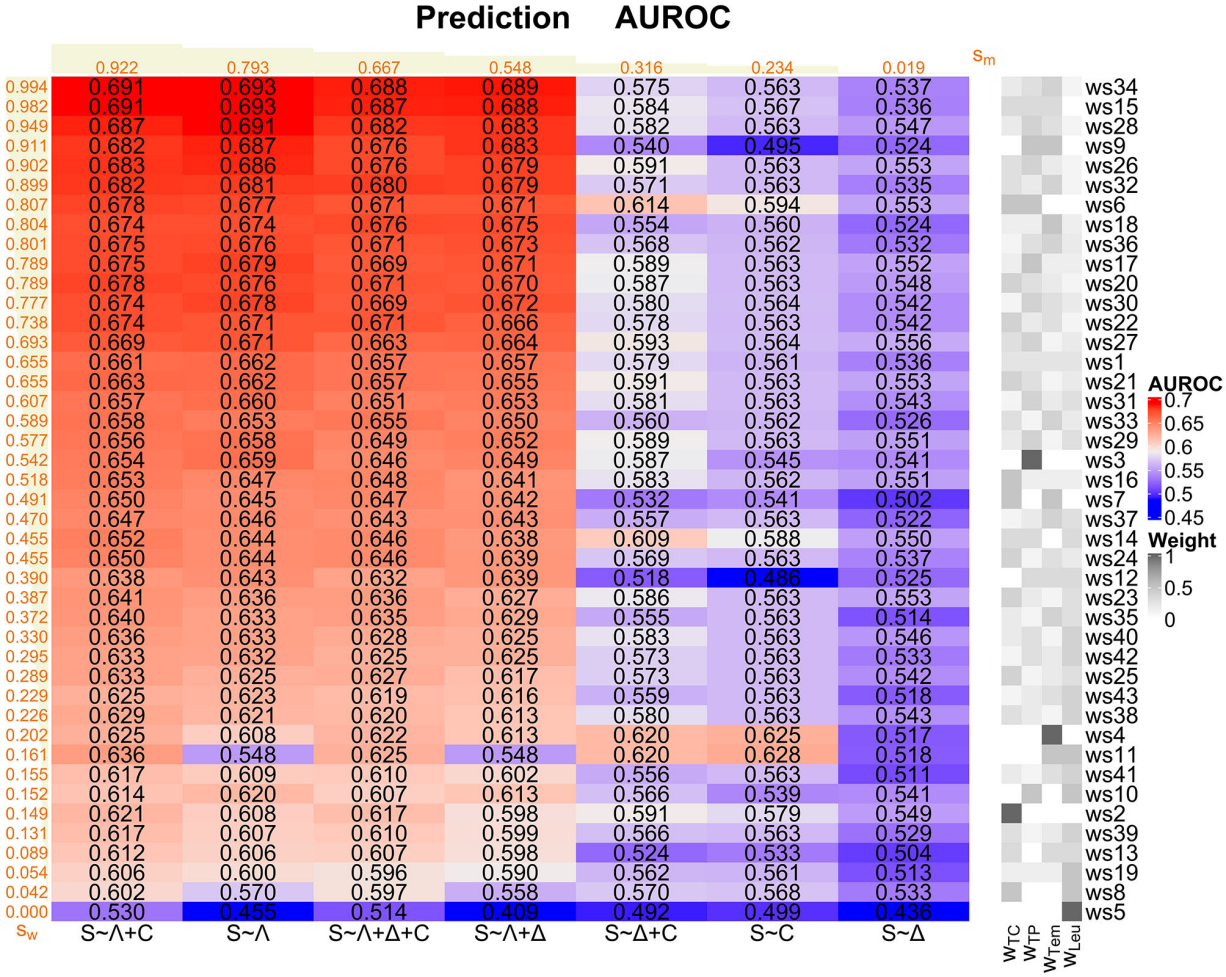
Prediction task: Overview of the AUROC values of the weighting schemes (ws1-ws43) for each considered logistic regression model (*S* ~ Λ, *S* ~ Δ, *S* ~ *C, S* ~ Λ + Δ, *S* ~ Λ + *C, S* ~ Δ + *C*, and *S* ~ Λ + Δ + *C*). Weighting schemes (rows) and models (columns) are decreasingly ordered according to their corresponding performances with respect to the ranking score values *s*_*w*_ and *s*_*m*_ (displayed in orange; derived as described in Supplementary Section 1 in Supplementary material 1) from the top to the bottom and from the left to the right, respectively. We emphasize that the ranking scores for the weighting schemes are derived using the four best-performing models (i.e., *S* ~ Λ + *C, S* ~ Λ, *S* ~ Λ + Δ + *C*, and *S* ~ Λ + Δ) only. For convenience, the compositions of the weighting schemes with respect to the four SIRS criteria (i.e., the weights *w*_TC_, *w*_TP_, *w*_Tem_, and *w*_Leu_ for the tachycardia, tachypnea, temperature, and leukocytes criterion, respectively) are indicated on the right-hand side of the overall plot, mirroring the specifications in Figure 1.

For each of our 7 logistic regression models and each of our 43 weighting schemes, we consider the corresponding AUROC values as main performance measures here (Figure 4 and Supplementary Figure 8 in Supplementary material 1). In the prediction task, we generally observe not that high AUROC values, with a maximum of 0.693 for the *S* ~ Λ model for ws15 and ws34, respectively.

The models *S* ~ Λ + *C*, *S* ~ Λ, *S* ~ Λ + Δ + *C* and *S* ~ Λ + Δ globally perform well over all weighting schemes, mirrored by the corresponding ranking score (Supplementary Section 1 in Supplementary material 1) values *s*_*m*_ in Figure 4. In contrast, models *S* ~ Δ + *C* and *S* ~ *C* perform rather badly, and model *S* ~ Δ clearly worst. Overall, Λ appears to be the most relevant SIRS descriptor and should be included in a potential sepsis prediction model, followed by *C*. In contrast, the descriptor Δ isolatedly performs badly and does not clearly contribute to a performance improvement, or even deteriorates performance, when considering it in combinations with Λ and *C* (compare the ranking scores *s*_*m*_ of (i) *S* ~ Λ vs. *S* ~ Λ + Δ, (ii) *S* ~ *C* vs. *S* ~ Δ + *C* and (iii) *S* ~ Λ + *C* vs. *S* ~ Λ + Δ + *C* in Figure 4).

When assessing the global performance of specific weighting schemes over the logistic regression models, we meaningfully restrict our attention here to the four best-performing models *S* ~ Λ, *S* ~ Λ + Δ + *C*, *S* ~ Λ + Δ and *S* ~ Λ + *C* figured out before. Weighting schemes with a good performance are in particular ws34, ws15, ws28, ws9, ws26, ws32, ws6, ws18 and ws36 (ranking scores *s*_*w*_ in Figure 4). These weighting schemes typically have a high weight on the tachypnea and the temperature criteria and a zero or low weight on the leukocytes criterion, and they all outperform the standard equal weighting scheme ws1 in terms of the AUROC values (Figure 4 and Supplementary Figure 8 in Supplementary material 1). Weighting schemes with a poor performance are in particular ws5, ws8, ws19, ws13, ws39, ws2, ws10, ws41 and ws11 (ranking scores *s*_*w*_ in Figure 4), where ws5 (weight only on leukocytes criterion) performs worst for all of the four best-performing models. These weighting schemes typically have a low weight on the temperature and the tachypnea criterion and a high weight on the leukocytes criterion, and they all perform worse than the standard equal weighting scheme ws1 in terms of the AUROC values (Figure 4 and Supplementary Figure 8 in Supplementary material 1).

In conclusion, based on our AUROC analyses and supported by additional results regarding sensitivity, specificity and calibration as alternative performance measures (Supplementary Figures 9–12 in Supplementary material 1) as well as Cox PH models (next subsubsection), *S* ~ Λ is our preferred model for the prediction task. Moreover, tachypnea and temperature constitute the most important SIRS criteria in the prediction task, followed by the tachycardia criterion, whereas the leukocytes criterion is by far the least important one and can even be counterproductive. Hence, the leukocytes criterion should not be given a high or prominent weight for the prediction task, or it may even be omitted completely.

#### 3.1.3. Cox PH models

The results for the multivariable Cox PH model (*T, S*) ~ Λ + Δ + *C* for time-to-event analysis, where the sepsis onset is the event here, basically confirm that Λ is by far the most important predictor in the prediction task. In particular, for all weighting schemes except for ws4, ws5, ws8 and ws11, holding the other covariates constant, a higher value of Λ is associated with an increased sepsis risk and a shorter time-to-event (Supplementary Figures 13, 14 in Supplementary material 1). In contrast, Δ and *C*, respectively, are essentially associated with no effect (Supplementary Figures 13, 14 in Supplementary material 1). Finally, the multivariable Cox PH model is globally statistically significant (i.e., the omnibus null hypothesis that all SIRS descriptor coefficients are zero is rejected) for all weighting schemes except for ws5, ws8, ws10 and ws19 (Supplementary Figure 15 in Supplementary material 1). Compared to the (time-independent) multivariable logistic regression model *S* ~ Λ + Δ + *C* (Supplementary Figure 7 in Supplementary material 1), the (time-dependent) multivariable Cox PH model (*T, S*) ~ Λ + Δ + *C* (Supplementary Figure 14 in Supplementary material 1) exhibits similar results with respect to Λ and Δ. In contrast, the significance of *C* that is present for some of the weighting schemes in the logistic regression model essentially vanishes for the Cox PH model. This underpins our choice of *S* ~ Λ as the preferred model for the prediction task.

### 3.2. Diagnosis task

#### 3.2.1. Basic analyses and descriptive statistics

**Figure 5:**
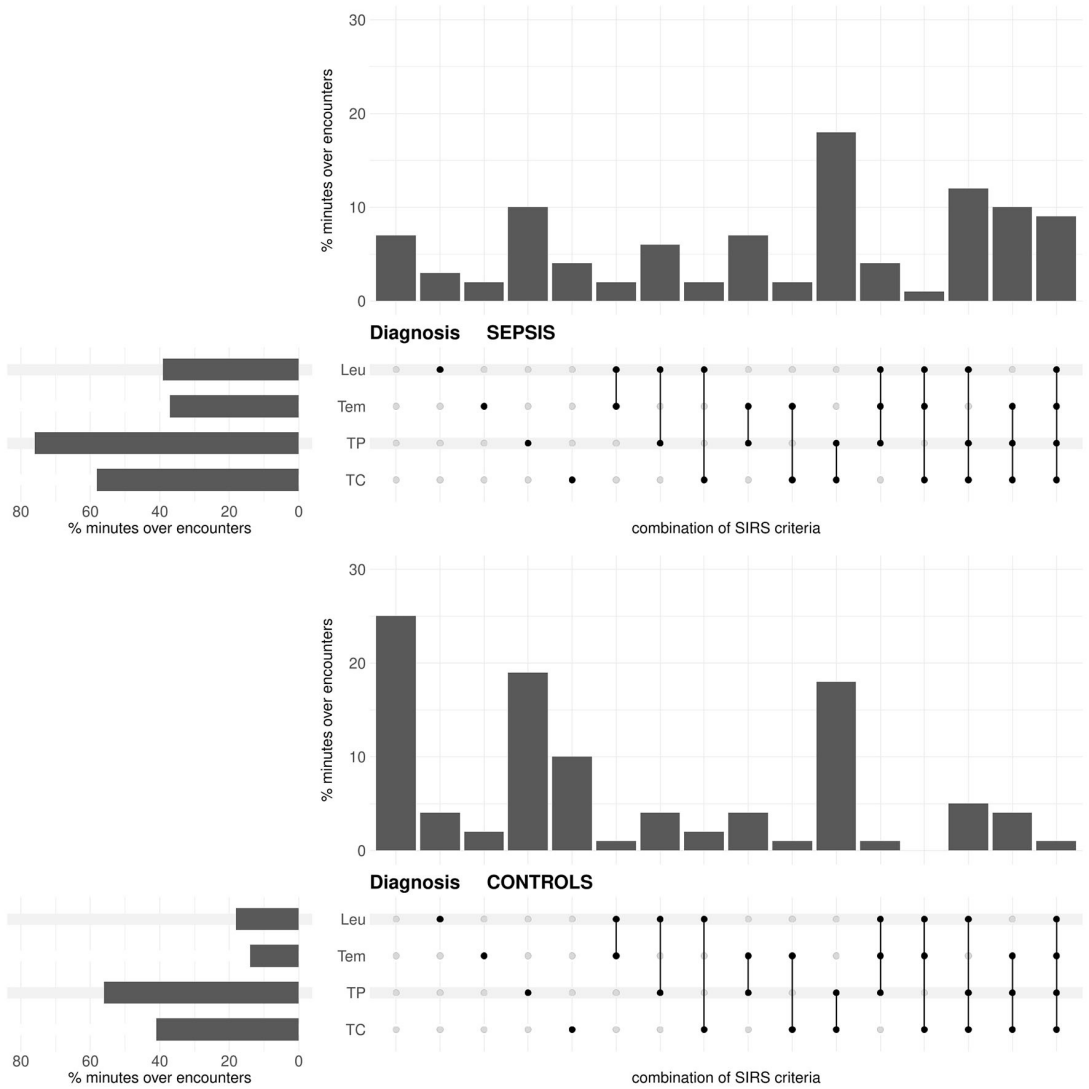
Diagnosis task: Upset plot-like figure relating to the frequencies of fulfilled SIRS criteria (TC, tachycardia; TP, tachypnea; Tem, temperature; Leu, leukocytes; indicated by the black dots), represented in the form of percentages of minutes over encounters, i.e., as percentages of in total 143 × 1440 = 205920 min for the sepsis group and 29721 × 1440 = 42798240 min for the no sepsis group, respectively.

In the diagnosis task, for both the sepsis and the control group, the tachypnea and the tachycardia criteria are fulfilled most often, then the leukocytes and the temperature criteria (Figure 5). The occurrence of each criterion is clearly more frequent in the sepsis group than in the control group (Figure 5; respective *P*-values *P* < 0.0001 from χ^2^-tests). Compared to the prediction task (Figure 3), the temperature and the leukocytes criteria are more often fulfilled for the sepsis group in the diagnosis task (respective *P*-values *P* < 0.0001 from χ^2^-tests). In the diagnosis task, the occurrence of no fulfilled criterion is meaningfully more frequent in the control group than in the sepsis group (~ 25% vs. ~ 7% in Figure 5; *P* < 0.0001 from a χ^2^-test). In contrast, in the sepsis group, there are more occurrences of three or more fulfilled SIRS criteria simultaneously than in the control group (*P* < 0.0001 from a χ^2^-test).

For the standard equal weighting scheme (ws1), summaries of the distributions of the SIRS descriptor values Λ, Δ and *C* for the sepsis and control groups in the diagnosis task are given in Table 3. Corresponding boxplots for all weighting schemes can be found in Supplementary material 1 (Supplementary Figures 16–18), accompanied by results for the respective Wilcoxon rank sum (Supplementary Figure 19 in Supplementary material 1) and waddR (Supplementary Figure 20 in Supplementary material 1) tests. The values of the SIRS descriptor Λ are significantly different between the sepsis and the control group (namely, significantly greater for the sepsis group) for all weighting schemes. The values of the SIRS descriptor Δ are significantly different between the sepsis and the control group (namely, significantly greater for the sepsis group) for all weighting schemes except for ws2, ws3 and ws6, putting weight exclusively on the tachycardia and/or tachypnea criteria. Thus, significant differences for Δ appear to be mainly driven by the temperature and the leukocytes criteria. The values of the SIRS descriptor *C* are significantly different between the sepsis and the control group for 8 (19%) weighting schemes only. In case of significantly greater values of *C* for the sepsis group (ws4, ws5, ws11), these differences are mainly driven by the temperature and leukocytes criteria. In contrast, in case of significantly greater values of *C* for the control group (ws3, ws6, ws10, ws14, ws15), these differences are mainly driven by the tachypnea criterion.

#### 3.2.2. Model and weighting scheme performances

In the univariable logistic regression model *S* ~ Λ, Λ is a significant predictor for all weighting schemes (Supplementary Figure 21 in Supplementary material 1) [e.g., for ws1: OR: 3.36 [95% CI: 2.75–4.12] for 1/4-unit change; *P* < 0.0001].

In the *S* ~ Δ model, Δ is a significant predictor for all weighting schemes except for ws2, ws3 and ws6, giving weight exclusively to the tachycardia and/or tachypnea criteria (Supplementary Figure 21 in Supplementary material 1) [e.g., for ws1: OR: 1.69 [95% CI: 1.43-1.99] for 1/4-unit change; *P* < 0.0001]. Thus, *S* ~ Δ is useful only if the temperature and the leukocytes criteria are considered.

In the *S* ~ *C* model, *C* is a significant predictor for ≈16% of the weighting schemes only (Supplementary Figure 21 in Supplementary material 1) [e.g., for ws1: OR: 0.99 [95% CI: 0.97-1.01]; *P* = 0.1994].

In the multivariable logistic regression model *S* ~ Λ + Δ + *C*, Λ is a significant predictor for all weighting schemes. This also holds for Δ, except for ws2, ws3 and ws6. In contrast, *C* is a significant predictor for ≈14% of the weighting schemes only (Supplementary Figure 22 in Supplementary material 1).

**Figure 6:**
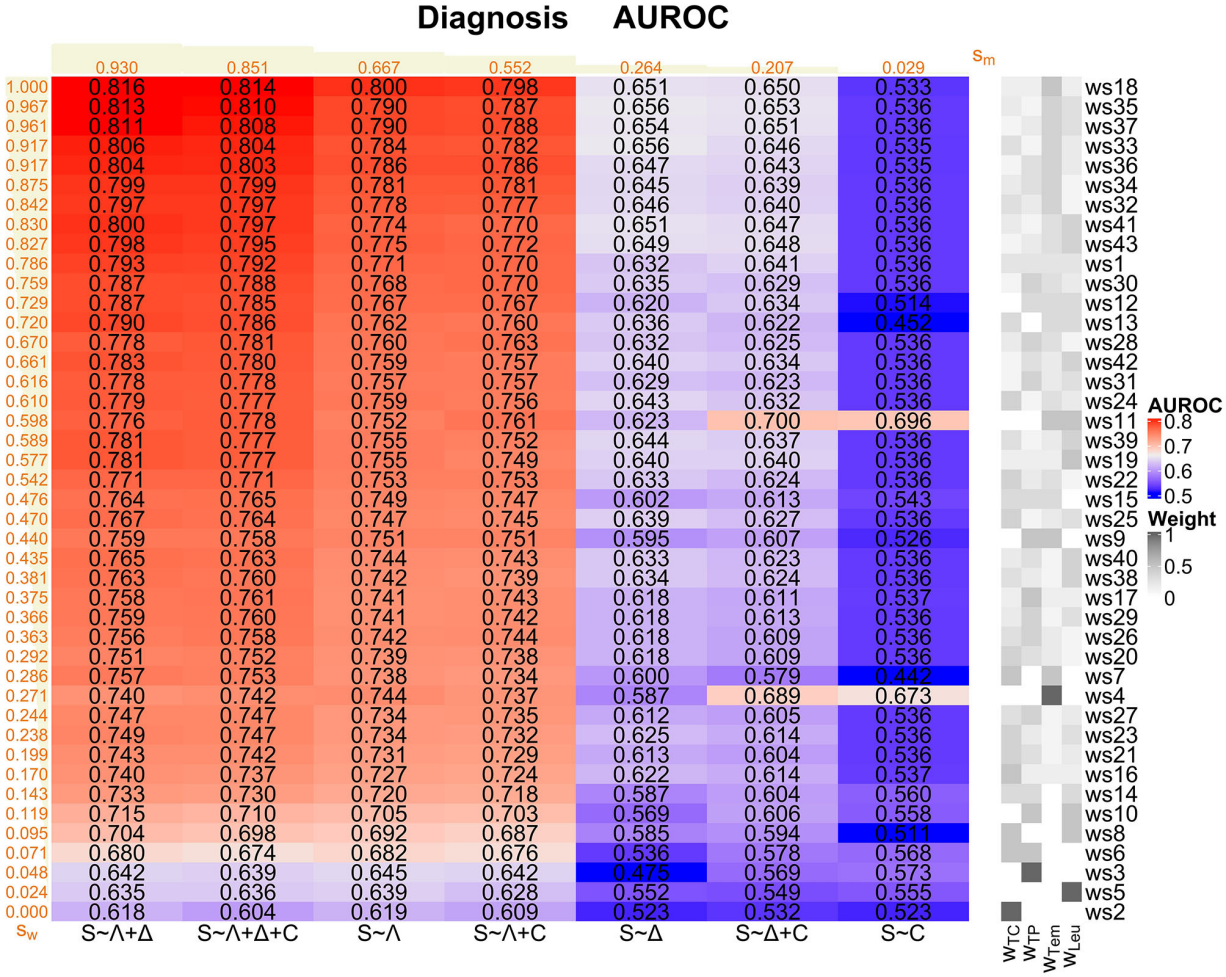
Diagnosis task: Overview of the AUROC values of the weighting schemes (ws1-ws43) for each considered logistic regression model (*S* ~ Λ, *S* ~ Δ, *S* ~ *C, S* ~ Λ + Δ, *S* ~ Λ + *C, S* ~ Δ + *C*, and *S* ~ Λ + Δ + *C*). Weighting schemes (rows) and models (columns) are decreasingly ordered according to their corresponding performances with respect to the ranking score values *s*_*w*_ and *s*_*m*_ (displayed in orange; derived as described in Supplementary Section 1 in Supplementary material 1) from the top to the bottom and from the left to the right, respectively. We emphasize that the ranking scores for the weighting schemes are derived using the four best-performing models (i.e., *S* ~ Λ + Δ, *S* ~ Λ + Δ + *C, S* ~ Λ, and *S* ~ Λ + *C*) only. For convenience, the compositions of the weighting schemes with respect to the four SIRS criteria (i.e., the weights *w*_TC_, *w*_TP_, *w*_Tem_ and *w*_Leu_ for the tachycardia, tachypnea, temperature, and leukocytes criterion, respectively) are indicated on the right-hand side of the overall plot, mirroring the specifications in Figure 1.

Considering again AUROC values as the main performance measures (Figure 6, Supplementary Figure 23 in Supplementary material 1), in the diagnosis task, there is a maximum AUROC of 0.816 for the *S* ~ Λ + Δ model for ws18, giving individually high weight to the temperature criterion and equal positive weight to the remaining criteria.

The models *S* ~ Λ + Δ, *S* ~ Λ + Δ + *C*, *S* ~ Λ and *S* ~ Λ + *C* globally perform well, where *S* ~ Λ + Δ has the highest ranking score of all considered models (Figure 6). In contrast, models *S* ~ Δ and *S* ~ Δ + *C* perform a bit weaker, and model *S* ~ *C* clearly worst. Overall, Λ appears to be the most relevant SIRS descriptor and should be included in a potential sepsis diagnosis model, followed by Δ. In contrast, the descriptor *C* isolatedly performs badly and even deteriorates performance when considering it in combinations with Λ and Δ (compare the ranking scores of (i) *S* ~ Λ vs. *S* ~ Λ + *C*, (ii) *S* ~ Δ vs. *S* ~ Δ + *C* and (iii) *S* ~ Λ + Δ vs. *S* ~ Λ + Δ + *C*; Figure 6).

When evaluating the global performance of specific weighting schemes over the logistic regression models, we again meaningfully restrict our attention to the four best-performing models *S* ~ Λ + Δ, *S* ~ Λ + Δ + *C*, *S* ~ Λ and *S* ~ Λ + *C*. Weighting schemes with a good performance are in particular ws18, ws35, ws37, ws33, ws36, ws34, ws32, ws41, and ws43 (Figure 6). These weighting schemes consider all SIRS criteria simultaneously, but give a higher weight to the temperature criterion. In particular, the AUROC values corresponding to these weighting schemes are larger than the AUROC values for the standard equal weighting scheme ws1, which itself already performs well (Supplementary Figure 23 in Supplementary material 1). Notably, ws18 performs best for all of the four best-performing models (ranking score *s*_ws18_ = 1; Figure 6). Weighting schemes with a poor performance are in particular ws2, ws5, ws3, ws6, ws8 and ws10 (Figure 6), where ws2, with weight only on the tachycardia criterion, performs worst for all of the four best-performing models (ranking score *s*_ws2_ = 0; Figure 6). These weighting schemes have zero weight on the temperature criterion, and weight is given only to one or two SIRS criteria simultaneously. All these weighting schemes perform worse than the standard equal weighting scheme ws1 in terms of the AUROC values (Figure 6 and Supplementary Figure 23 in Supplementary material 1).

In conclusion, based on our AUROC analyses and underpinned by additional results regarding sensitivity, specificity and calibration as alternative performance measures (Supplementary Figures 24–27 in Supplementary material 1), *S* ~ Λ + Δ is our preferred model for the diagnosis task. Moreover, accounting for all SIRS criteria simultaneously is important in the diagnosis task, while temperature by far clearly constitutes the most important SIRS criterion.

### 3.3. Comparison of prospective SIRS algorithm to reference approaches

**Figure 7:**
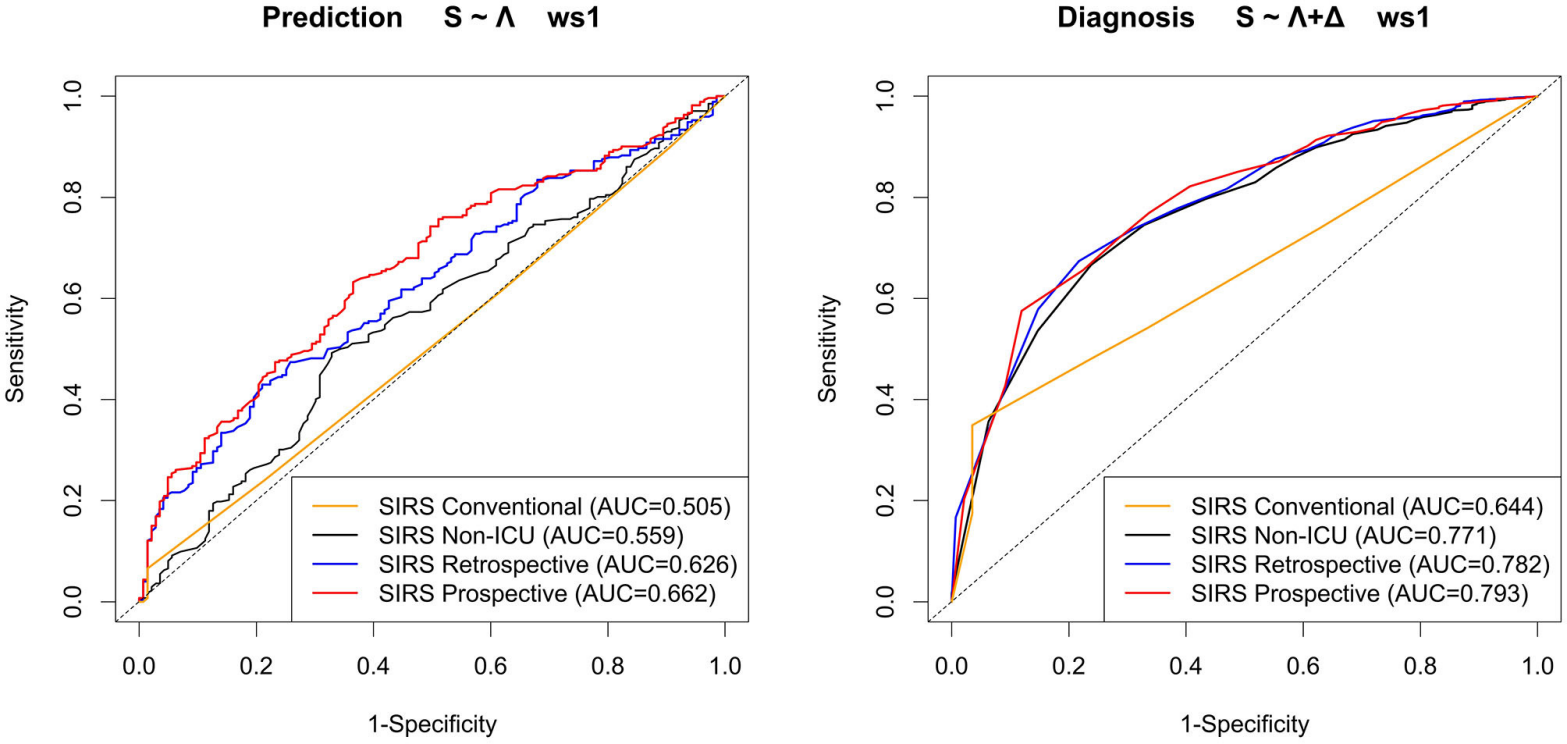
ROC curves and corresponding AUROC (AUC) values for the models *S* ~ Λ for the prediction task and *S* ~ Λ + Δ for the diagnosis task, respectively, with respect to weighting scheme ws1 (equal weight for all SIRS criteria) for the SIRS algorithm versions Conventional, Non-ICU, Retrospective, and Prospective.

We now compare our hitherto results based on the prospective SIRS algorithm (SIRS Prospective) to those obtained by the related reference algorithms SIRS Conventional, SIRS Non-ICU and SIRS Retrospective, respectively. For convenience, in the main text, we exemplarily only focus on the results for the (overall well-performing) standard equal weighting scheme (ws1) considering all SIRS criteria and our preferred models *S* ~ Λ for the prediction task and *S* ~ Λ + Δ for the diagnosis task, respectively (Figure 7 and Table 4).

**Table 4:** Performance measures for the models *S* ~ Λ for the prediction task and *S* ~ Λ + Δ for the diagnosis task, respectively, with respect to weighting scheme ws1 (equal weight for all SIRS criteria) for the SIRS algorithm versions Conventional, Non-ICU, Retrospective and Prospective.

		**Prediction**	**Diagnosis**
		*S* ~ Λ	*S* ~ Λ + Δ
		**ws1**	**ws1**
Sensitivity	SIRS Conventional	0.986	0.965
	SIRS Non-ICU	0.671	0.762
	SIRS Retrospective	0.790	0.783
	SIRS Prospective	0.636	0.881
Specificity	SIRS Conventional	0.066	0.349
	SIRS Non-ICU	0.493	0.666
	SIRS Retrospective	0.430	0.674
	SIRS Prospective	0.632	0.575
DistSlope	SIRS Conventional	0.267	0.019
	SIRS Non-ICU	0.159	0.020
	SIRS Retrospective	0.043	0.014
	SIRS Prospective	0.022	0.010
DistIntercept	SIRS Conventional	0.168	5.341
	SIRS Non-ICU	0.101	0.096
	SIRS Retrospective	0.026	0.067
	SIRS Prospective	0.012	0.046

Here, DistSlope refers to the distance of the calibration slope to the reference value of 1 (indicating good calibration), and DistIntercept to the distance of the calibration intercept to the reference value of 0 (indicating good calibration).

Overall, the corresponding AUROC values in the diagnosis task are clearly higher than those in the prediction task, thus indicating a better discriminative performance of the algorithms and models near to the index (sepsis) time point (Figure 7). For both tasks, SIRS Prospective has the best AUROC, followed by SIRS Retrospective and then SIRS Non-ICU (Figure 7). SIRS Conventional has the worst AUROC (Figure 7), showing indeed high sensitivity, but by far weakest specificity (Table 4). While there is a quite clear AUROC performance order for the prediction task, the differences between the AUROC values of SIRS Non-ICU, SIRS Retrospective and SIRS Prospective become smaller for the diagnosis task. The algorithm performance rankings with respect to the AUROC values basically also hold in terms of the calibration measures DistSlope and DistIntercept (Table 4). In a nutshell, our results suggest superiority of the SIRS Prospective algorithm compared to the reference approaches and thus confirm its meaningfulness.

For the sake of completeness, all ROC curves and AUROC values of the four considered algorithms in our 301 considered scenarios (43 weighting schemes × 7 logistic regression models) are provided in Supplementary material 2 (prediction task) and Supplementary material 3 (diagnosis task), respectively. Essentially, the results described before continue to hold for well-performing models and weighting schemes according to Figure 4 (prediction) and Figure 6 (diagnosis).

## 4. Discussion

The principal goal of our study was to investigate the importance of each of the four SIRS criteria for sepsis prediction and diagnosis in a polytrauma cohort from an ICU. For both tasks, we assessed sepsis classification performance of regression models from systematically varied weights of the SIRS criteria. For each weighting scheme, criteria were determined and summarized as SIRS descriptors Λ (average SIRS level), Δ (SIRS level trend) and *C* (number of changes in SIRS level) with a novel prospective SIRS algorithm. The resulting regression models were compared with a specifically devised scoring system facilitating objective identification of the optimal SIRS criteria weights and SIRS descriptor-based classification models. The SIRS descriptor Λ is of greatest importance for AUROC-based sepsis classification in both tasks. For diagnosis, Δ was relevant as additional parameter. Combining our findings for sepsis prediction and diagnosis suggests that the importance of individual SIRS criteria changes over ICU treatment time. Thus, we support the a priori consideration of all SIRS criteria in a given sepsis risk model. For both tasks, a dynamic ICU-specific representation of systemic inflammation with our prospective SIRS algorithm was superior to static consideration of SIRS with a conventional SIRS algorithm version.

Our novel prospective algorithm captures the SIRS phenomenon in a time-dependent fashion. It has a conceptually clear overarching framework, which basically relies on (i) assessment of the observed parameters regarding the SIRS criteria thresholds based on (7) complemented by ICU-specific adjustments and (ii) the assignment of clinically plausible duration intervals for each parameter (Table 2). By accounting for catecholamine therapy and mechanical ventilation, we have specifically tailored our SIRS algorithm to the ICU; however, due to the modular design, a SIRS algorithm for non-ICU settings is readily available. Thanks to its strictly prospective implementation, our SIRS algorithm is potentially applicable in real-time in a data-driven clinical decision support system (16). While several artificial intelligence and machine learning-based approaches have already been proposed in the context of sepsis prediction (41–45, among others), the translation of such techniques is still in its infancy, and the tools typically are not operationally used yet (46). This may be for instance due to a skepticism of clinicians toward too complex, possibly black box algorithms. In this light, we believe that our conceptually simple, comprehensible and interpretable SIRS algorithm has high potential for application in clinical practice. Generally, the dynamization accomplished by our algorithm for SIRS may also serve as a template for capturing other time-dependent clinical phenomena.

Despite using an explicit dynamic modeling approach, the focus of our analyses was still on the three descriptors average SIRS level Λ, SIRS level trend Δ and number of changes in SIRS level *C*, which summarize the output of our SIRS algorithm over specified time intervals, here 24 h. However, a more detailed consideration of the temporal evolution, e.g., in minute resolution, is also possible. Respective initial inspections of group average trajectories of the SIRS level λ are consistent with our findings for 24-h intervals (Supplementary Figures 28–31 and Supplementary Section 3 in Supplementary material 1).

Our SIRS descriptor-based models in general perform better for the sepsis diagnosis than for the prediction task with respect to AUROC and other alternative metrics. This intuitively makes sense, as the diagnosis task is performed closer to the relevant index (sepsis) time point. This temporal association supports the validity of SIRS, particularly our dynamic representation, as an acute, sepsis-related concept. Our results of the prediction task nevertheless suggest that sepsis predcition based on SIRS criteria within 24 h after ICU admission may be meaningful, but is not overly powerful.

Overall, for both prediction and diagnosis, we found that the average SIRS level Λ is a good classifier. This is in line with recent results from the literature, in which the mean has been shown to be a powerful time series summary statistics for clinical disease prediction tasks (28). The other SIRS descriptors Δ and *C* may be useful for classification as well, but apparently should be considered and interpreted *together* with the values of Λ. Interestingly, the importance of Δ and *C* appears to change, depending on the task. In particular, while *C* appears to be more important than Δ for prediction, the opposite holds for diagnosis: While *C* is less important here, the SIRS level trend Δ apparently becomes more relevant, which is in line with results by (15). This reflects that close to the time of sepsis diagnosis a positive trend in SIRS level is present, which additionally supports the validity of the SIRS concept for sepsis diagnosis and generally the operationalization of SIRS with dynamic SIRS descriptors. An explanation for the behavior of Δ may be that, in the prediction setting, it is comparatively probable to observe a non-negative trend in both the sepsis and the no sepsis groups. This is because the different parameters required for SIRS descriptor determination are generally not likely to be measured and recorded at exactly the same minute with reference to the ICU admission time point. This technical delay in data acquisition may not only cover meaningful group differences in Δ, but may also non-differentially inflate *C*. This likely at least partly causes the initial strong upward slope of both group SIRS level λ averages within the first 120 min of admission (Supplementary Figure 29 in Supplementary material 1). In contrast, as patients have already stayed at the ICU for some time, Δ and *C* are not likely to be affected this way when used for sepsis diagnosis and consistently no initial slope in group averages of λ is present (Supplementary Figure 31 in Supplementary material 1).

Regarding the performance of the different weighting schemes over our logistic regression models, for the sepsis prediction task, temperature and tachypnea constitute the most important SIRS criteria, followed by the tachycardia criterion. In contrast, the leukocytes criterion should not be given a high or leading weight for the prediction task and may even be omitted completely. On the other hand, for the diagnosis task, the temperature criterion is most prominent when distinguishing between the sepsis and the control group, which is consistent with results by (47). Remarkably, also the leukocytes criterion plays a much more important role for diagnosis, and thus, its relevance strongly differs between the prediction and diagnosis tasks. In the prediction task, the leukocytes criterion is clearly not suitable for distinguishing between the sepsis and no sepsis group. Here, a fulfillment of the leukocytes criterion (i.e., leukocytosis) likely occurs as a result of physical and emotional stress shortly after the (poly)trauma in a transient process that is not related to bone marrow production or the release of band cells or other immature cells (48). Hence, the leukocytes criterion is likely not a suitable predictor for a later development of sepsis at the ICU admission stage shortly after trauma. This can be related to results by (49), who showed that variations in leukocytes count in trauma patients at admission are not beneficial in predicting the need for therapeutic interventions such as volume resuscitation, transfusion or surgery. However, the leukocytes criterion obviously becomes more important in the course of ICU treatment and is much more able to contribute to the distinction between the sepsis and control groups at a later stage than at admission, as witnessed by the results for the diagnosis task shortly before the index (sepsis) time point.

In the diagnosis task, the *joint* fulfillment of SIRS criteria (i.e., the interplay between the criteria), with a specific focus on the temperature criterion, is very important to distinguish the sepsis from the control group. In particular, the more one approaches the index (sepsis) time point the more important the joint consideration of all four SIRS criteria appears to become, in that a joint fulfillment of three to four SIRS criteria points at the development of sepsis, while no or only one fulfilled SIRS criterion points at no (impending) sepsis.

Overall, no weighting scheme performs best for both tasks and for all considered models, and weighting schemes may in particular show different performances at different points during treatment time (here, prediction and diagnosis). Hence, consistent with the dynamic nature of systemic inflammation, the individual importance of the four SIRS criteria for sepsis prediction may change over treatment time as observed in our study. Thus, no SIRS criterion should a priori be omitted in a sepsis risk model. In particular, the standard equal weighting scheme ws1 performs quite well in both the prediction (Supplementary Figure 8 in Supplementary material 1) and especially the diagnosis task (Supplementary Figure 23 in Supplementary material 1) compared to the other weighting schemes. Hence, ws1 is a reasonable overall compromise, which underpins the validity of the original expert definition of SIRS (7). All in all, our findings of a dynamic role of the SIRS criteria further support the validity of our approach to capturing SIRS.

To highlight the benefits of our novel SIRS Prospective algorithm, we exemplarily compared its performance to that of three reference approaches for the overall well-performing equal weighting scheme ws1 for our preferred models *S* ~ Λ (prediction) and *S* ~ Λ + Δ (diagnosis), respectively. For both the prediction and the diagnosis tasks, SIRS Prospective clearly outperforms the non-dynamic SIRS Conventional approach, that is indeed highly sensitive, but suffers from a lack of specificity, which drastically limits the usefulness of this very basic approach. This confirms that a dynamic modeling of the SIRS phenomenon as used in SIRS Prospective is essential. Moreover, SIRS Prospective overall outperforms the SIRS Non-ICU algorithm, which by construction corresponds to SIRS Prospective but does not account for catecholamine therapy and mechanical ventilation when determining the validity of the tachycardia and tachypnea criteria, respectively. Hence, our results underline that accounting for ICU-specific interventions is beneficial in our settings. Lastly, while SIRS Retrospective and SIRS Prospective overall perform similarly well, the latter performs slightly better in terms of AUROC and calibration metrics. Moreover, SIRS Prospective is conceptually simpler, easier to implement, and has the advantage of not having to possibly rely on future values to determine the validity of a SIRS criterion, such that in principle, it could be applied in real-time in a clinical decision support system, unlike SIRS Retrospective. In a nutshell, we have shown the superiority of the SIRS Prospective algorithm compared to the reference approaches and in particular that accounting for catecholamine therapy and mechanical ventilation as well as dynamic aspects makes sense in our ICU setting considering critically ill polytrauma patients.

Finally, we emphasize again that our results here hold for a specific cohort of polytrauma patients and are additionally limited by the single-center design of our study. It remains to be investigated whether the results are confirmed also in other patient groups and settings.

## 5. Conclusion

Overall, our novel prospective SIRS algorithm provides a conceptually simple, yet promising tool that we have used for sepsis prediction (using data from the first 24 h after ICU admission) and diagnosis (using data from the last 24 h prior to the index/sepsis time point) in an ICU polytrauma cohort. For these applications, our SIRS algorithm typically outperforms reference algorithm versions. Moreover, the results obtained by our algorithm reveal the importance and contribution of the four SIRS criteria in our considered settings, using different weighting schemes and logistic regression models including several summarizing SIRS descriptors. In particular, in the sepsis prediction task, temperature and tachypnea turn out to be the most important SIRS criteria, while the leukocytes criterion is clearly the least relevant one. In contrast, in the sepsis diagnosis task, temperature turns out to be the most important SIRS criterion, and a joint consideration of all four SIRS criteria becomes essential. From a modeling point of view, in particular the average SIRS level Λ proves to be an important predictor that should be included in any sepsis prediction or diagnosis model. The SIRS level trend Δ that is additionally proposed for sepsis diagnosis models highlights the acute change in patient state, associated with impending sepsis.

### 5.1. Implications and recommendations for translational research

We in what follows summarize general implications and recommendations for translational research on sepsis with respect to SIRS and its criteria which can be derived from our cohort of ICU polytrauma patients.

- Despite not being present anymore in the latest consensus definition of sepsis, SIRS remains an important concept in the context of sepsis prediction and diagnosis.
- We recommend a dynamic, prospective description of the SIRS phenomenon, as e.g., provided by our SIRS Prospective algorithm, to exploit the potential of SIRS for sepsis prediction and diagnosis, as well as to allow for real-time applications in clinical decision support systems.
- When considering SIRS in ICU settings, we recommend to account for the ICU-specific interventions of catecholamine therapy and mechanical ventilation when assessing whether the tachycardia and tachypnea SIRS criteria, respectively, are fulfilled.
- The importance of the four individual SIRS criteria for sepsis prediction may change over treatment time, reflecting the evolving clinical patient state. Thus, all SIRS criteria from the original expert definition (7) are potentially important and should be monitored, and none should be omitted a priori.
- In particular, the temperature criterion overall appears to play a prominent role. Therefore, monitoring a patient's temperature appears a simple yet efficient measure for early detection of sepsis.

## 6. Outlook and future work

While the prospective SIRS algorithm introduced here appears to be a well-performing and promising tool, there are plenty of opportunities for further development of the algorithm. For instance, further rules from subgroup analyses or patient stratification could be derived (e.g., taking account of etiology or specifically tailored relevant patient subgroups) that could be implemented “on top” of the current algorithm rules, favored by the modular design of the algorithm.

Moreover, one may rethink the thresholding approach that is used to define the range of “normal” values and thus to derive the validity of the SIRS criteria. As the threshold values that are currently employed stem from the original work by (7) from the early 1990s, they could be re-evaluated, and it may be checked whether the choice of other thresholds would be more appropriate, see, e.g., (50) in the context of temperature. Ideally, a relaxation or even the complete abolition of the thresholding strategy to describe the SIRS phenomenon should be a major aim for future work, e.g., by developing a dynamic, continuous “SIRS state”, or the like.

We here only considered our three SIRS descriptors in the logistic regression models for sepsis prediction and diagnosis. However, also other clinical, laboratory or demographic parameters can be included into the models. Likewise, the output and descriptors, respectively, of our SIRS algorithm could be included in more comprehensive sepsis risk models, e.g., as a part of a multifactorial algorithm with the long-term aim to provide a final sepsis/SIRS score for clinical decision support.

Moreover, we stress that our studies here have been performed using a polytrauma cohort, comprising quite specifically selected patents from the ICU. However, similar analyses can readily be conducted for cohorts consisting of more general, unselected patients. One example may be the cohort based on the ground truth for sepsis questionnaire introduced in (51), in which the sepsis time point can be derived using labels assigned by clinical experts.

Likewise, in future work, one could consider alternative (i) SIRS descriptors, which may more comprehensively describe and summarize the time dynamics in the SIRS algorithm (52), and (ii) evaluation tools, such as NetBenefit (53) as a metric for clinical utility.

Furthermore, a network-based approach to the evolution of SIRS in the context of organ systems could be taken with the network analysis methods recently proposed in (54).

Finally, our SIRS algorithm can potentially be used in other contexts apart from sepsis prediction and diagnosis, e.g., for other complications, and the dynamic time series concept can likely be transferred to other settings and application areas with possibly different time resolutions.

## Software usage

The SIRS algorithm variants have been implemented in SAS v9.4 (SAS Institute, Cary, NC). The statistical analyses and evaluations have been performed using SAS v9.4 and the R language and environment for statistical computing (55).

## Data availability statement

The datasets presented in this article are not readily available because due to data protection reasons, the raw patient data cannot be made publicly available. Aggregated data as well as code for the SIRS algorithm may be made available upon reasonable request. Requests to access the datasets should be directed to RS, roman.schefzik@zi-mannheim.de.

## Ethics statement

The studies involving human participants were reviewed and approved by Medical Ethics Commission II of the Medical Faculty Mannheim, Heidelberg University (2016-840R-MA). Written informed consent from the participants' legal guardian/next of kin was not required to participate in this study in accordance with the national legislation and the institutional requirements.

## Author contributions

RS and VS-L: methods and study conceptualization, writing, editing, and interpretation of results. RS and BH: implementation and analyses. BH: data preparation and curation. RS: figures. All authors contributed to the article and approved the submitted version.
