# Supplementary material for: Dissecting contributions of individual systemic inflammatory response syndrome criteria from a prospective algorithm to the prediction and diagnosis of sepsis in a polytrauma cohort
Source: Front Med (Lausanne). 2023 Jul 31;10:1227031. doi: 10.3389/fmed.2023.1227031 (PMC10424878; doi:10.3389/fmed.2023.1227031)
Supplement: Supplementary file 1 [file Data_Sheet_1.pdf]

Supplementary Material 1

Dissecting contributions of individual systemic inflammatory response syndrome criteria from a prospective algorithm to the prediction and diagnosis of sepsis in a polytrauma cohort

Roman Schefzik, Bianka Hahn and Verena Schneider-Lindner

Contents

S1 Supplementary methods: Ranking scores **2**

S2 Supplementary results for the SIRS Prospective algorithm **4**
S2.1 Prediction task 4
S2.2 Diagnosis task 17

S3 Supplementary results for the SIRS Prospective algorithm: Initial inspection of temporal evolution **28**
S3.1 Prediction task 28
S3.2 Diagnosis task 30

S1 Supplementary methods: Ranking scores

We here introduce a quantitative scoring strategy to rank the performances of (i) specific models over several weighting schemes and (ii) specific weighting schemes over several models, respectively, which is specifically tailored to the setting of our analyses, but analogously could be applied also to other contexts.

Let $\mathcal{M}$ with $\#\mathcal{M} = M$ be the set of the considered models, and let $\mathcal{W}$ with $\#\mathcal{W} = W$ be the set of the considered weighting schemes. Moreover, let E be a performance evaluation measure, and let e_m^w be the corresponding value of E for model m and weighting scheme w.

For a fixed model $m \in \mathcal{M}$, we define a ranking score s_m by

$$s_m := \frac{1}{W(M-1)} \sum_{w=1}^{W} (\mathrm{rk}(e_m^w) - 1) \in [0, 1],$$

where for a fixed weighting scheme $w \in \mathcal{W}$, $\mathrm{rk}(e_m^w)$ denotes the the rank of e_m^w in $\{e_1^w, \ldots, e_M^w\}$. Basically, s_m can be regarded as an average rank score which is suitably standardized to ensure that its values lie within the unit interval $[0, 1]$, allowing for a straightforward interpretation and possible comparisons. The specific interpretation of the score s_m depends on whether the underlying performance evaluation measure E is positively oriented (i.e., the larger the value the better the performance) or negatively oriented (i.e., the smaller the value the better the performance). Specifically, if E is positively oriented, s_m is also positively oriented (i.e., the larger the value of s_m the better the performance of model m in terms of the performance evaluation measure E over all considered weighting schemes), where a value of exactly zero for model m indicates that m performs worst for all weighting schemes, while a value of exactly one for model m indicates that m performs best for all weighting schemes. Analogously, if E is negatively oriented, s_m is also negatively oriented (i.e., the smaller the value of s_m the better the performance of model m in terms of the performance evaluation measure E over all considered weighting schemes), where a value of exactly one for model m indicates that m performs worst for all weighting schemes, while a value of exactly zero for model m indicates that m performs best for all weighting schemes.

In the same spirit, for a fixed weighting scheme $w \in \mathcal{W}$, we define a ranking score s_w by

$$s_w := \frac{1}{M(W-1)} \sum_{m=1}^{M} (\mathrm{rk}(e_m^w) - 1) \in [0, 1],$$

where for a fixed model $m \in \mathcal{M}$, $\mathrm{rk}(e_m^w)$ denotes the the rank of e_m^w in $\{e_m^1, \ldots, e_m^W\}$. Basically, s_w can be regarded as an average rank score which is suitably standardized to ensure that its values lie within the unit interval $[0, 1]$, allowing for a straightforward interpretation and possible comparisons. The specific interpretation of the score s_w depends on whether the underlying performance evaluation measure E is positively oriented (i.e., the larger the value the better the performance) or negatively oriented (i.e., the smaller the value the better the performance). Specifically, if E is positively oriented, s_w is also positively oriented (i.e., the larger the value of s_w the better the performance of weighting scheme w in terms of the performance evaluation measure E over all considered models), where a value of exactly zero for weighting scheme w indicates that w performs worst for all models, while a value of exactly one for weighting scheme w indicates that w performs best for all models. Analogously,

if E is negatively oriented, s_w is also negatively oriented (i.e., the smaller the value of s_w the better the performance of weighting scheme w in terms of the performance evaluation measure E over all considered models), where a value of exactly one for weighting scheme w indicates that w performs worst for all models, while a value of exactly zero for weighting scheme w indicates that w performs best for all models.
Note that as the scores s_m and s_w are solely rank-based by construction, it is recommended to consider them *together* with the actual performance evaluation measure values for a comprehensive interpretation of the results.
In our analyses,

- we specifically focus on AUROC, sensitivity and specificity as positively oriented performance evaluation measures E, as well as on DistSlope (i.e., the distance of the calibration slope to the reference value of 1 indicating good calibration) and DistIntercept (i.e., the distance of the calibration intercept to the reference value of 0 indicating good calibration) as negatively oriented performance evaluation measures E.

- during the calculation process of the ranks, possible ties are resolved by assigning the corresponding average ranks here (however, alternative methods for dealing with ties, such as randomly resolving them, are in principle also possible).

- when deriving the scores s_m, we use $\mathcal{M} := \{S \sim \Lambda, S \sim \Delta, S \sim C, S \sim \Lambda + \Delta + C, S \sim \Lambda + \Delta, S \sim \Lambda + C, S \sim \Delta + C\}$ (with notation as explained in the main text) with $\#\mathcal{M} = 7$ as the set of models, and $\mathcal{W} := \{\text{ws1}, \dots, \text{ws43}\}$ with $\#\mathcal{W} = 43$ as the set of weighting schemes.

- when deriving the scores s_w, we also take $\mathcal{W} := \{\text{ws1}, \dots, \text{ws43}\}$ with $\#\mathcal{W} = 43$ as the set of weighting schemes, but for a better meaningfulness only consider the respective four best-performing models for each considered performance evaluation measure, i.e., $\#\mathcal{M} = 4$. In particular, when focusing on the central AUROC values as performance evaluation measures in Figures 4 and 6, respectively, in the main text, we only consider the models $\mathcal{M} := \{S \sim \Lambda, S \sim \Lambda + \Delta + C, S \sim \Lambda + \Delta, S \sim \Lambda + C\}$ for the derivation of the s_w's, as these turn out to perform clearly better than the remaining models $S \sim \Delta, S \sim C$ and $S \sim \Delta + C$. However, all observations and conclusions basically continue to hold when considering the ranking scores based on all 7 models (results not explicitly shown).

S2 Supplementary results for the SIRS Prospective algorithm

S2.1 Prediction task

	**Prediction**		Diagnosis	
Boxplots for SIRS descriptor Λ	**Figure S1**	**p. 5**	Figure S16	p. 18
Boxplots for SIRS descriptor Δ	**Figure S2**	**p. 6**	Figure S17	p. 19
Boxplots for SIRS descriptor C	**Figure S3**	**p. 7**	Figure S18	p. 20
Wilcoxon rank sum test	**Figure S4**	**p. 8**	Figure S19	p. 21
waddR test	**Figure S5**	**p. 8**	Figure S20	p. 21
Univariable logistic regression	**Figure S6**	**p. 9**	Figure S21	p. 22
Multivariable logistic regression	**Figure S7**	**p. 9**	Figure S22	p. 22
AUROC values: Comparison to equal weighting	**Figure S8**	**p. 10**	Figure S23	p. 23
Sensitivity	**Figure S9**	**p. 11**	Figure S24	p. 24
Specificity	**Figure S10**	**p. 12**	Figure S25	p. 25
Calibration slope	**Figure S11**	**p. 13**	Figure S26	p. 26
Calibration intercept	**Figure S12**	**p. 14**	Figure S27	p. 27
Hazard ratios for Λ, Δ and C in multiv. Cox model	**Figure S13**	**p. 15**		
Multivariable Cox model	**Figure S14**	**p. 16**		
Omnibus Cox model	**Figure S15**	**p. 16**		

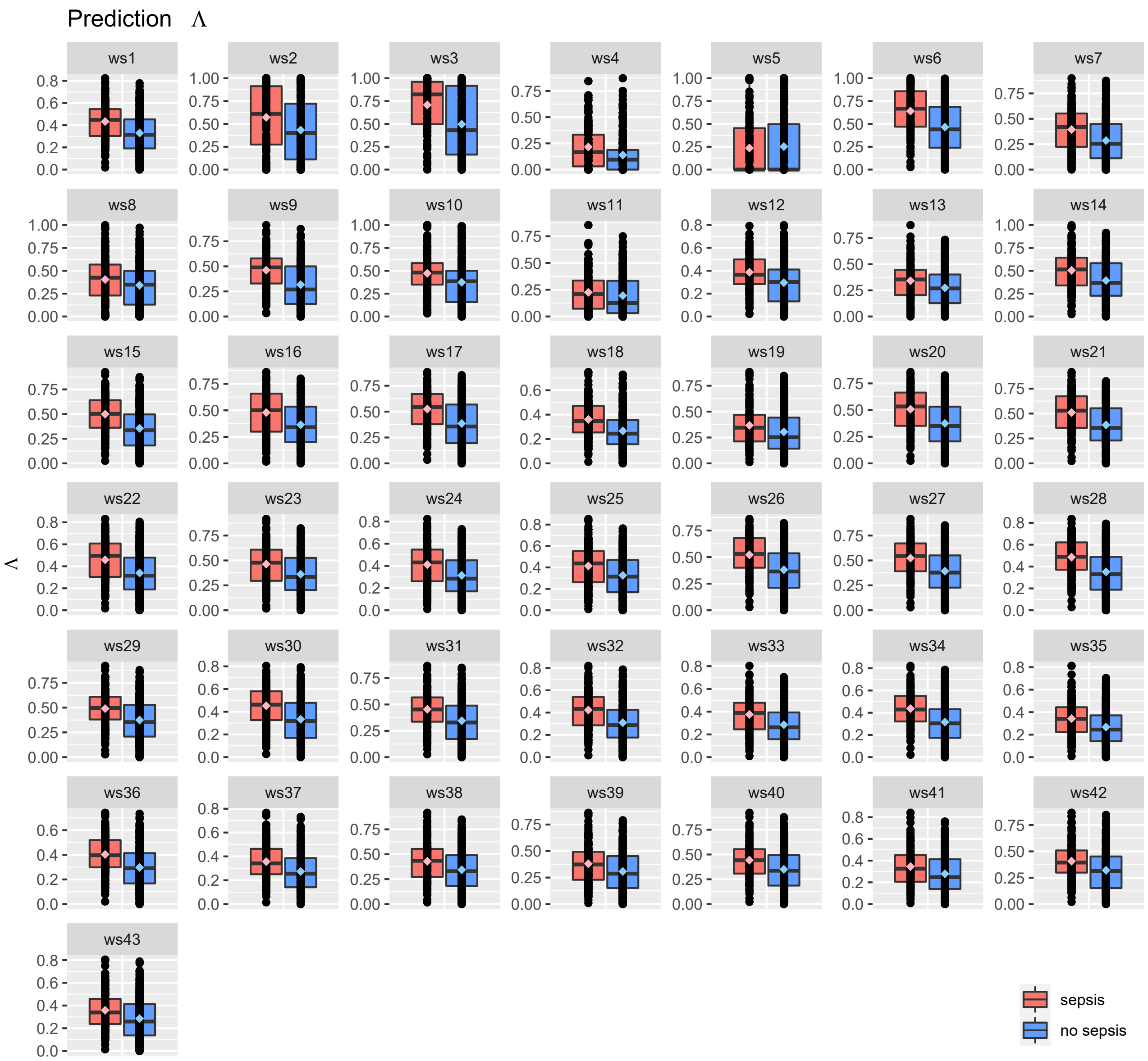

Figure S1: Prediction task: Boxplots representing the distributions of the SIRS descriptor Λ (average SIRS level) for the sepsis and no sepsis group, respectively, for the different weighting schemes ws1 to ws43. Corresponding means are indicated by diamond symbols.

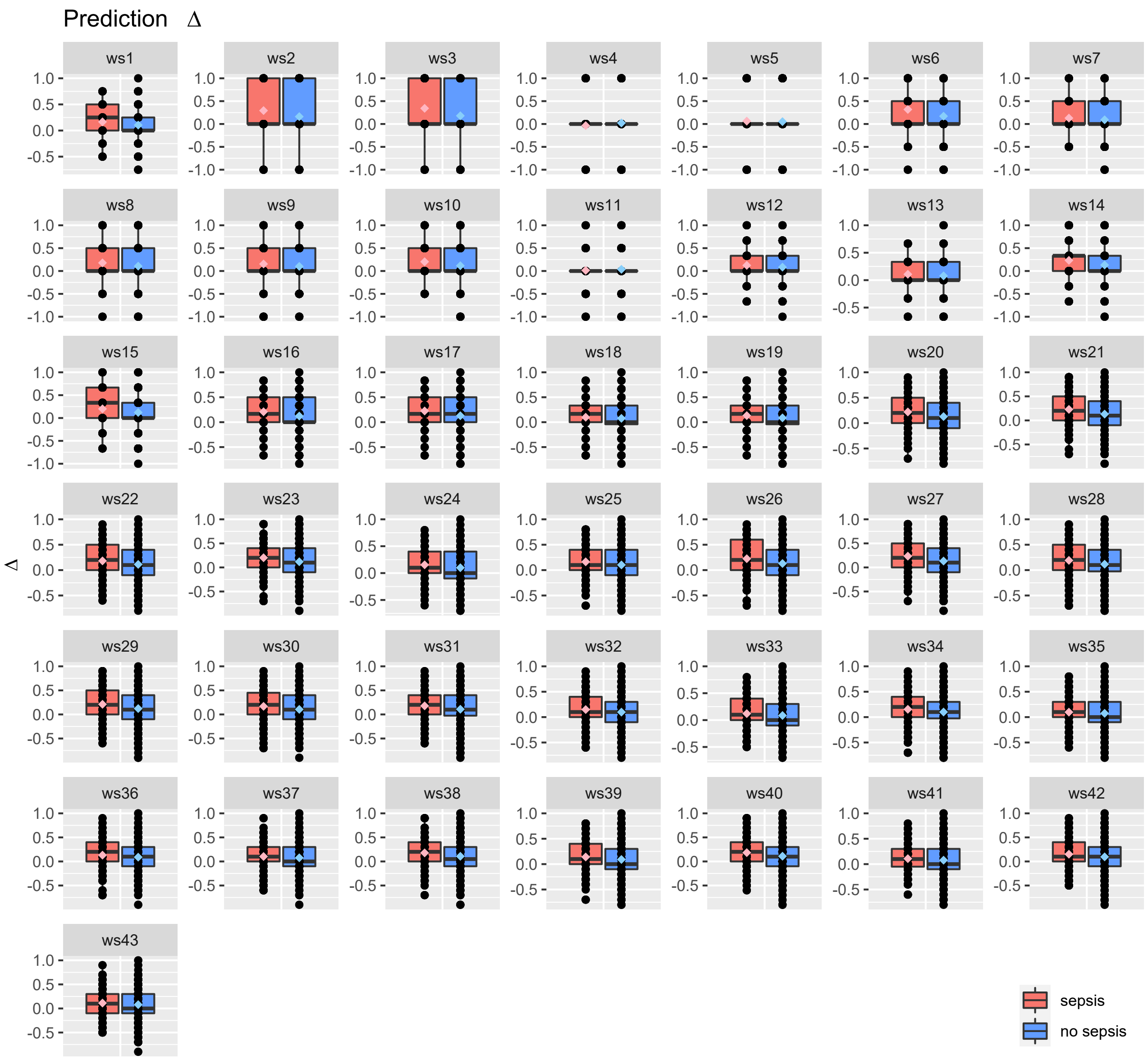

Figure S2: Prediction task: Boxplots representing the distributions of the SIRS descriptor Δ (SIRS level trend) for the sepsis and no sepsis group, respectively, for the different weighting schemes ws1 to ws43. Corresponding means are indicated by diamond symbols.

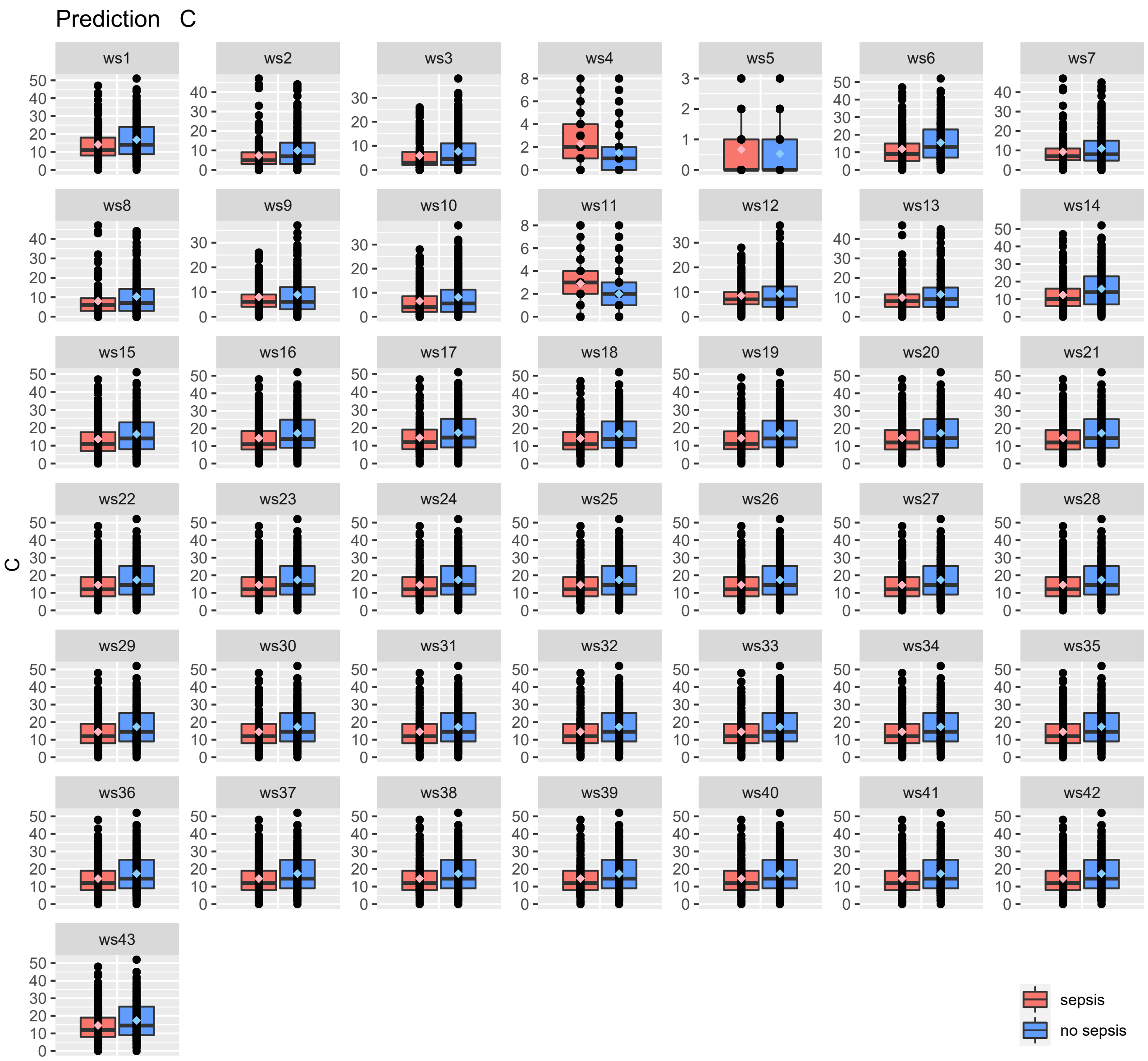

Figure S3: Prediction task: Boxplots representing the distributions of the SIRS descriptor C (number of changes in SIRS level) for the sepsis and no sepsis group, respectively, for the different weighting schemes ws1 to ws43. Corresponding means are indicated by diamond symbols.

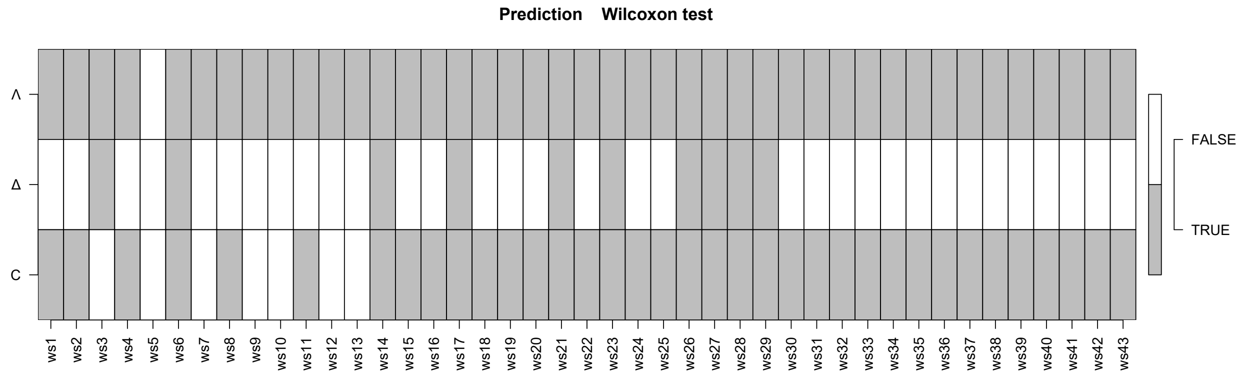

Figure S4: Prediction task: For each of the SIRS descriptors Λ, Δ and C and the different weighting schemes ws1 to ws43, results for the Wilcoxon rank sum test at a 5% significance level to check whether the descriptor value for the sepsis group is significantly different from the descriptor value for the no sepsis group (TRUE) or not (FALSE): The corresponding cell is colored in grey if the null hypothesis H_0 of no difference can be rejected in favor of the alternative H_1 that there is a difference, and the cell is white if H_0 cannot be rejected.

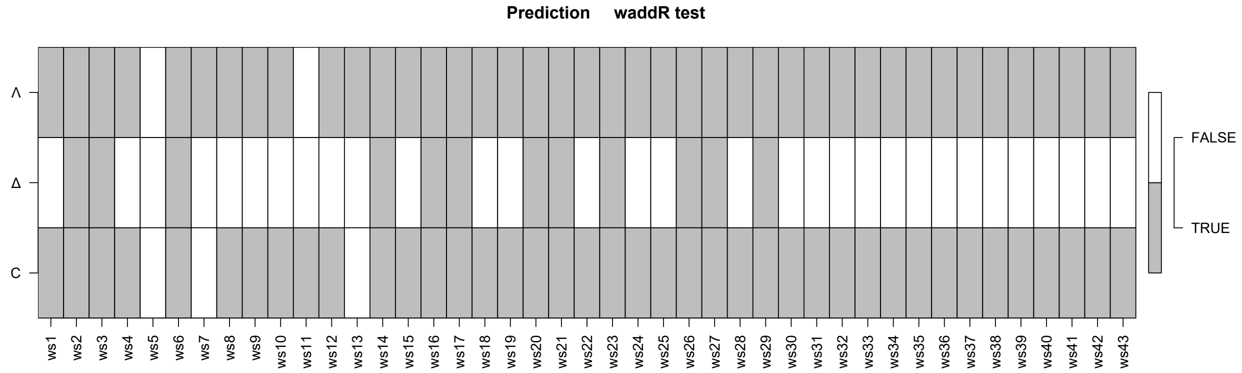

Figure S5: Prediction task: For each of the SIRS descriptors Λ, Δ and C and the different weighting schemes ws1 to ws43, results for the waddR test [1] at a 5% significance level to check whether the distribution of the descriptor values for the sepsis group is significantly different from the distribution of the descriptor values for the no sepsis group (TRUE) or not (FALSE): The corresponding cell is colored in grey if the null hypothesis H_0 of no difference can be rejected in favor of the alternative H_1 that there is a difference, and the cell is white if H_0 cannot be rejected.

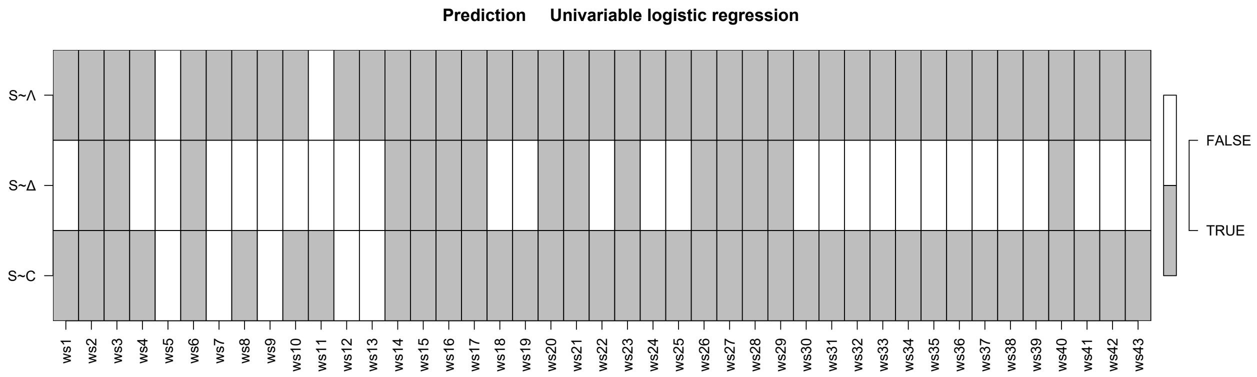

Figure S6: Prediction task: Overview map indicating whether for a specific weighting scheme ws1 to ws43 the SIRS descriptor Λ, Δ and C is a significant predictor (TRUE) or not (FALSE) in the corresponding univariable logistic regression model $S \sim \Lambda$, $S \sim \Delta$ and $S \sim C$, respectively, for a significance level of 5%

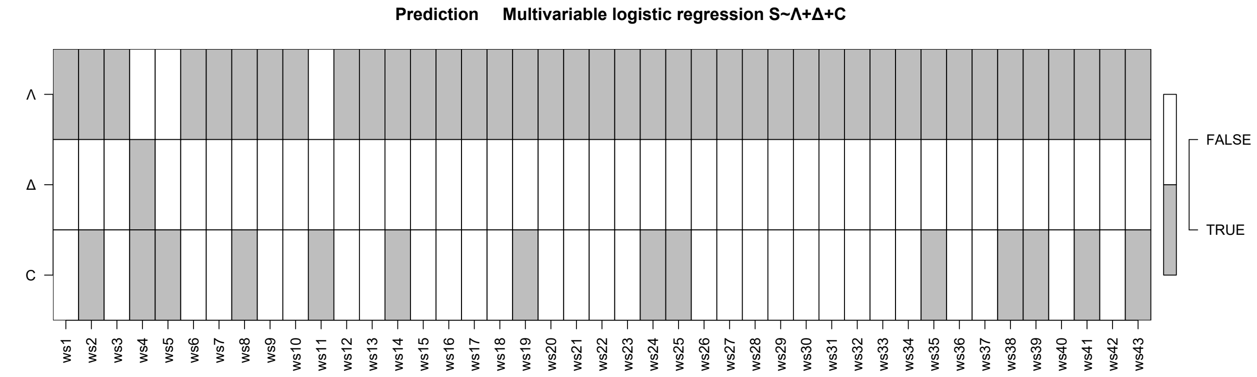

Figure S7: Prediction task: Overview map indicating whether for a specific weighting scheme ws1 to ws43 the SIRS descriptors Λ, Δ and C are significant predictors (TRUE) or not (FALSE) in the multivariable logistic regression model $S \sim \Lambda + \Delta + C$, for a significance level of 5%

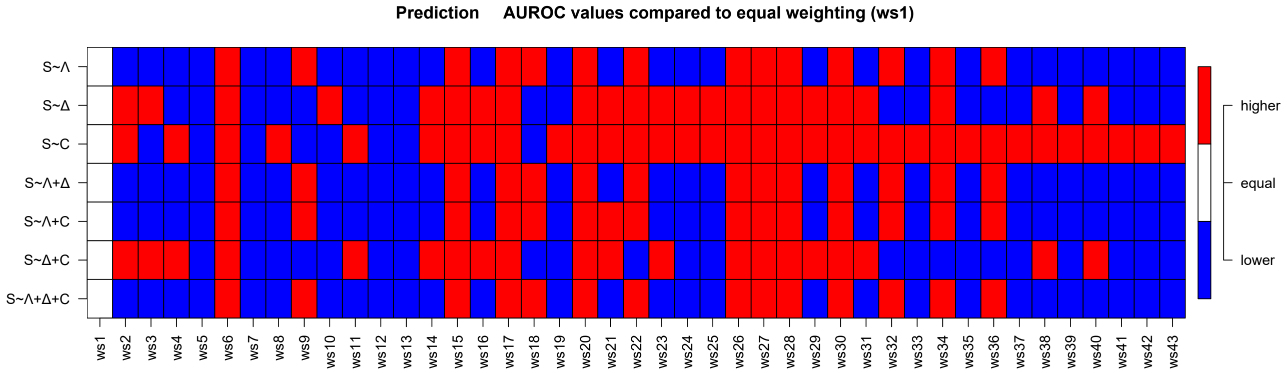

Figure S8: Prediction task: Overview of the AUROC values of the weighting schemes ws1 to ws43 in comparison to the equal weighting scheme (ws1) for each considered logistic regression model. This figure accompanies Figure 4 from the main text.

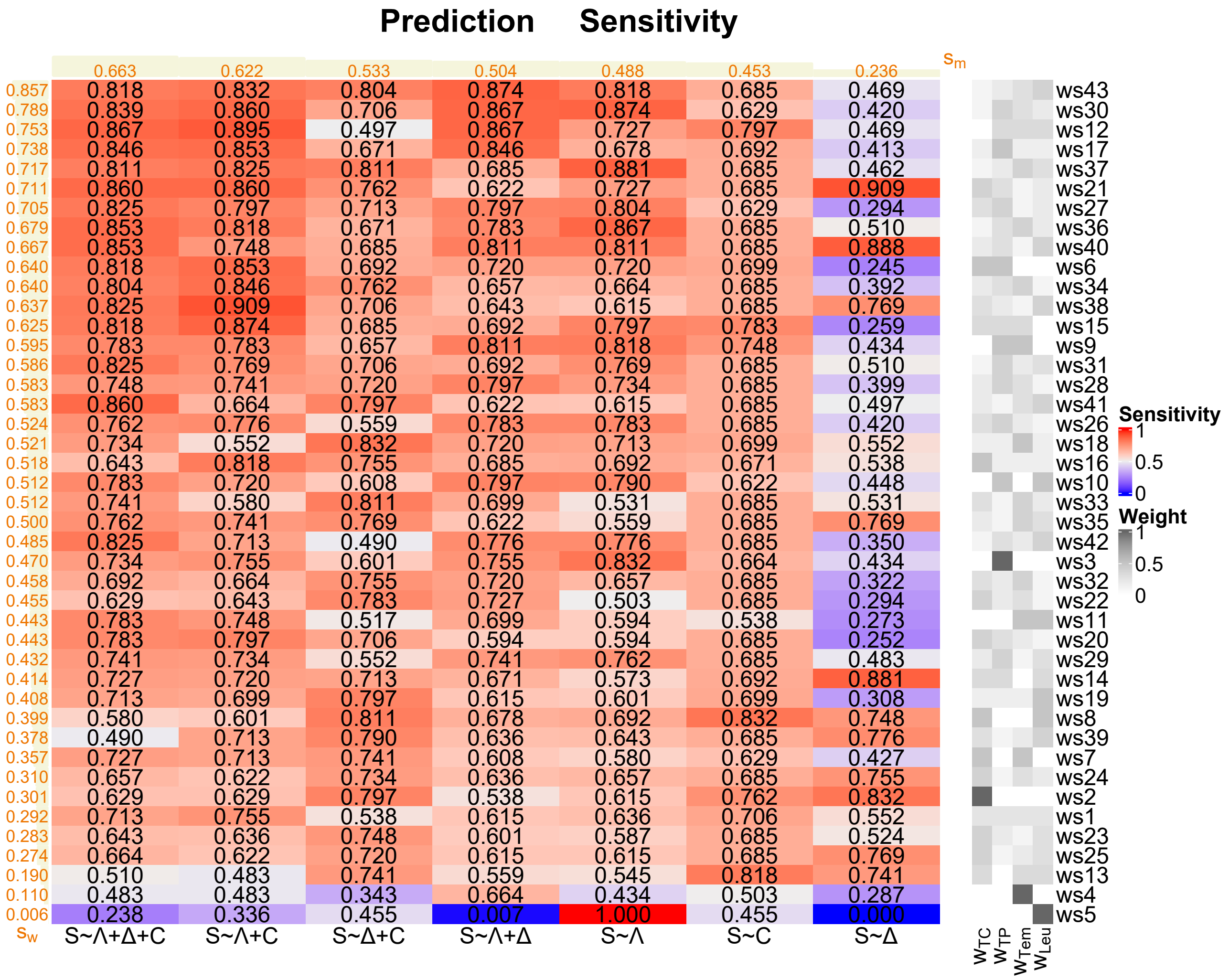

Figure S9: Prediction task: Overview of the sensitivity values of the weighting schemes (ws1-ws43) for each considered logistic regression model ($S \sim \Lambda$, $S \sim \Delta$, $S \sim C$, $S \sim \Lambda + \Delta$, $S \sim \Lambda + C$, $S \sim \Delta + C$ and $S \sim \Lambda + \Delta + C$). Weighting schemes (rows) and models (columns) are decreasingly ordered according to their corresponding performances with respect to the ranking score values s_w and s_m (displayed in orange; derived as described in Section S1) from the top to the bottom and from the left to the right, respectively. For convenience, the compositions of the weighting schemes with respect to the four SIRS criteria (i.e., the weights w_{TC}, w_{TP}, w_{Tem} and w_{Leu} for the tachycardia, tachypnea, temperature and leukocytes criterion, respectively) are indicated on the right-hand side of the overall plot, mirroring the specifications in Figure 1.

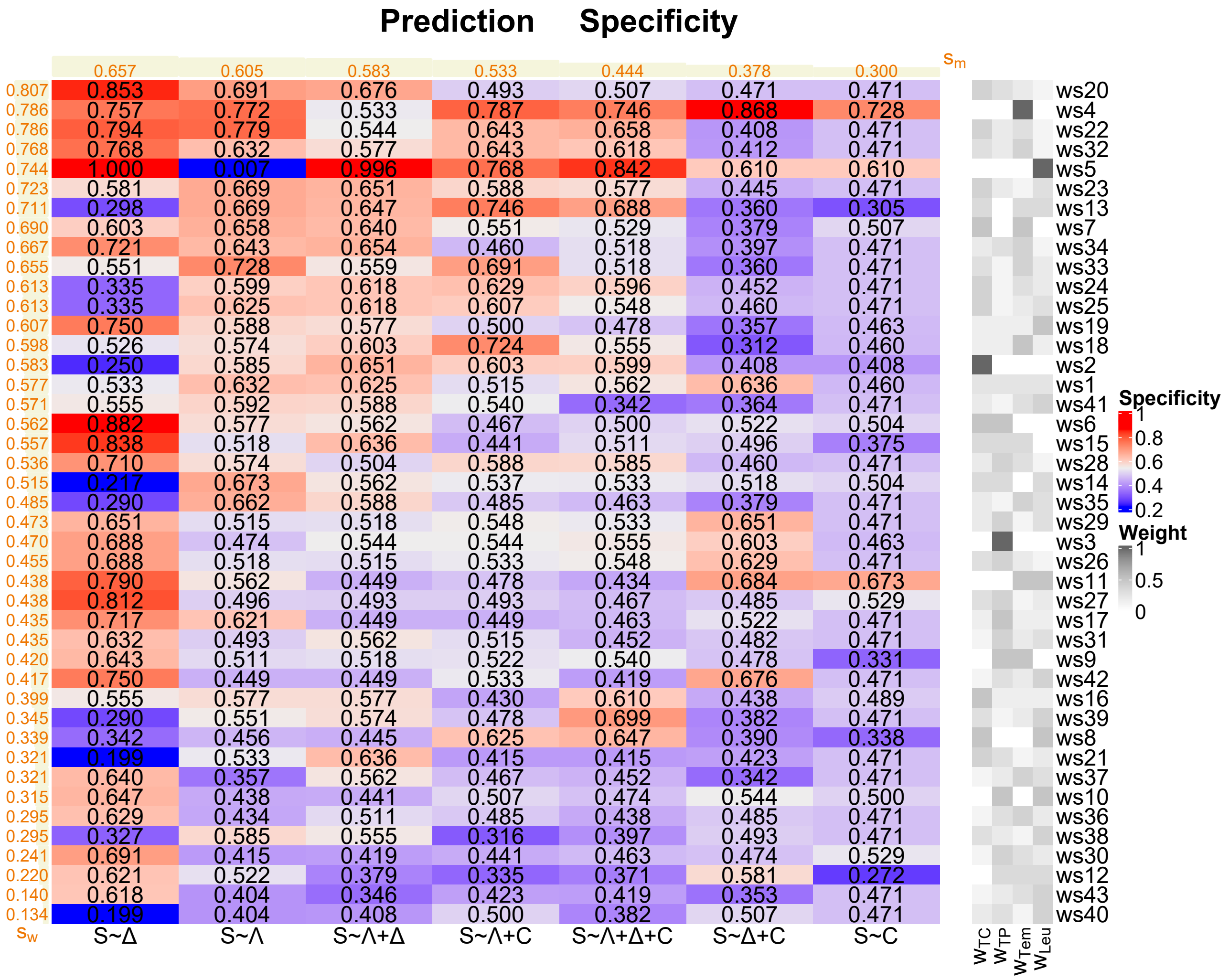

Figure S10: Prediction task: Overview of the specificity values of the weighting schemes (ws1-ws43) for each considered logistic regression model ($S \sim \Lambda$, $S \sim \Delta$, $S \sim C$, $S \sim \Lambda + \Delta$, $S \sim \Lambda + C$, $S \sim \Delta + C$ and $S \sim \Lambda + \Delta + C$). Weighting schemes (rows) and models (columns) are decreasingly ordered according to their corresponding performances with respect to the ranking score values s_w and s_m (displayed in orange; derived as described in Section S1) from the top to the bottom and from the left to the right, respectively. For convenience, the compositions of the weighting schemes with respect to the four SIRS criteria (i.e., the weights w_{TC}, w_{TP}, w_{Tem} and w_{Leu} for the tachycardia, tachypnea, temperature and leukocytes criterion, respectively) are indicated on the right-hand side of the overall plot, mirroring the specifications in Figure 1.

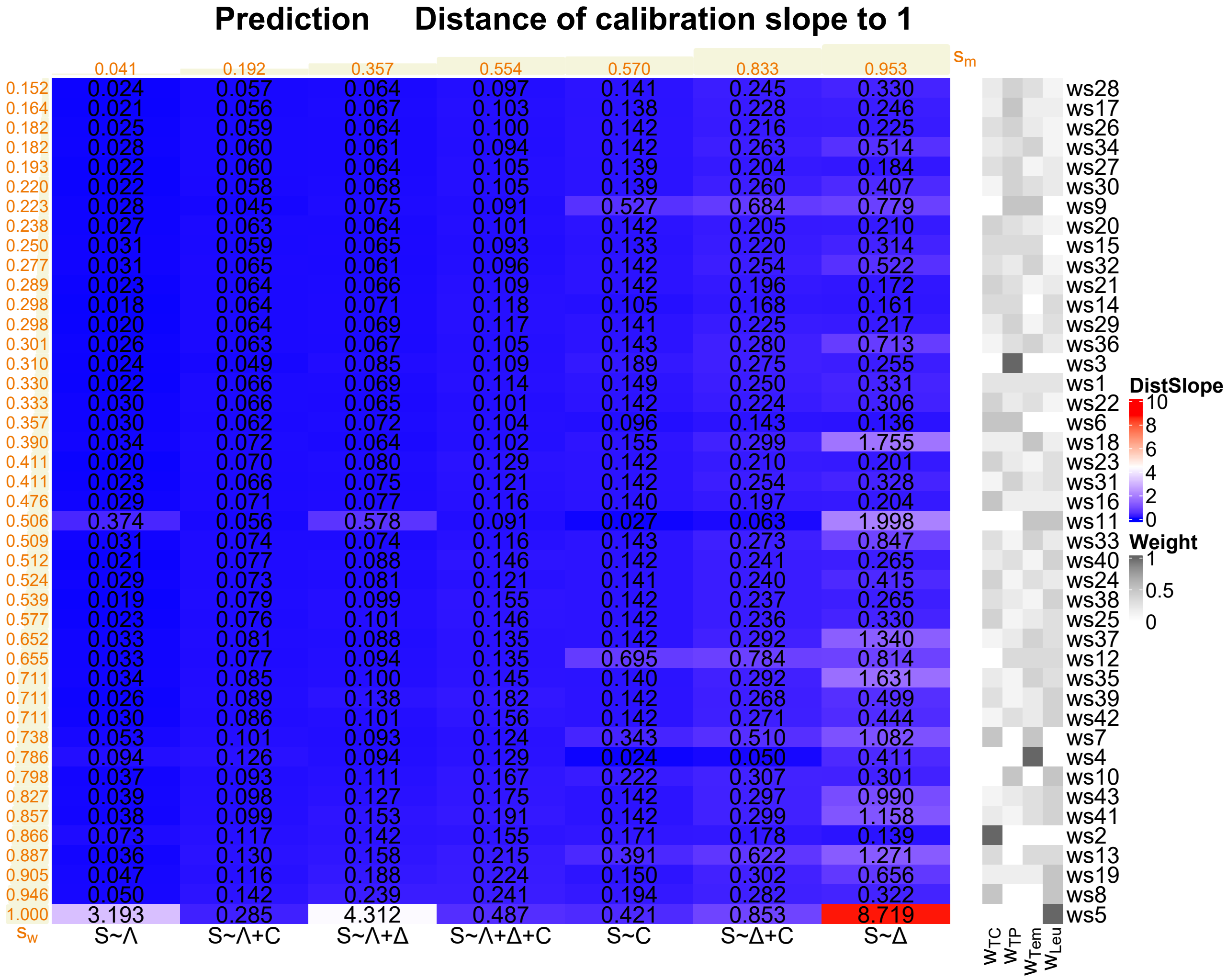

Figure S11: Prediction task: Overview of the distances of the calibration slope to the reference value of 1 (indicating good calibration; DistSlope) of the weighting schemes (ws1-ws43) for each considered logistic regression model ($S \sim \Lambda$, $S \sim \Delta$, $S \sim C$, $S \sim \Lambda + \Delta$, $S \sim \Lambda + C$, $S \sim \Delta + C$ and $S \sim \Lambda + \Delta + C$). Weighting schemes (rows) and models (columns) are decreasingly ordered according to their corresponding performances with respect to the ranking score values s_w and s_m (displayed in orange; derived as described in Section S1) from the top to the bottom and from the left to the right, respectively. Note that here, the performance measures are negatively oriented (i.e., the smaller the value the better the performance). For convenience, the compositions of the weighting schemes with respect to the four SIRS criteria (i.e., the weights w_{TC}, w_{TP}, w_{Tem} and w_{Leu} for the tachycardia, tachypnea, temperature and leukocytes criterion, respectively) are indicated on the right-hand side of the overall plot, mirroring the specifications in Figure 1.

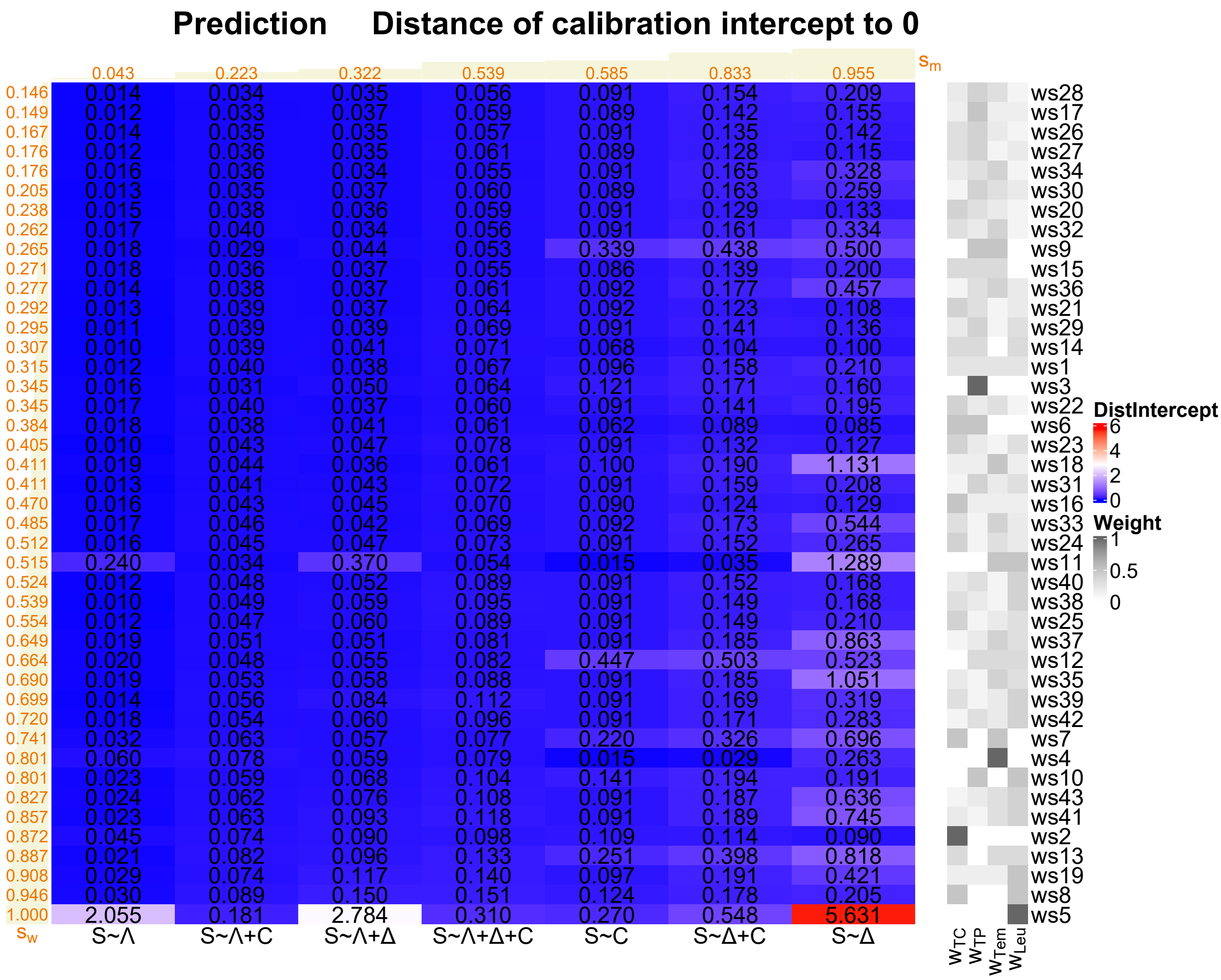

Figure S12: Prediction task: Overview of the distances of the calibration intercept to the reference value of 0 (indicating good calibration; DistIntercept) of the weighting schemes (ws1-ws43) for each considered logistic regression model ($S \sim \Lambda$, $S \sim \Delta$, $S \sim C$, $S \sim \Lambda{+}\Delta$, $S \sim \Lambda{+}C$, $S \sim \Delta{+}C$ and $S \sim \Lambda{+}\Delta{+}C$). Weighting schemes (rows) and models (columns) are decreasingly ordered according to their corresponding performances with respect to the ranking score values s_w and s_m (displayed in orange; derived as described in Section S1) from the top to the bottom and from the left to the right, respectively. Note that here, the performance measures are negatively oriented (i.e., the smaller the value the better the performance). For convenience, the compositions of the weighting schemes with respect to the four SIRS criteria (i.e., the weights w_{TC}, w_{TP}, w_{Tem} and w_{Leu} for the tachycardia, tachypnea, temperature and leukocytes criterion, respectively) are indicated on the right-hand side of the overall plot, mirroring the specifications in Figure 1.

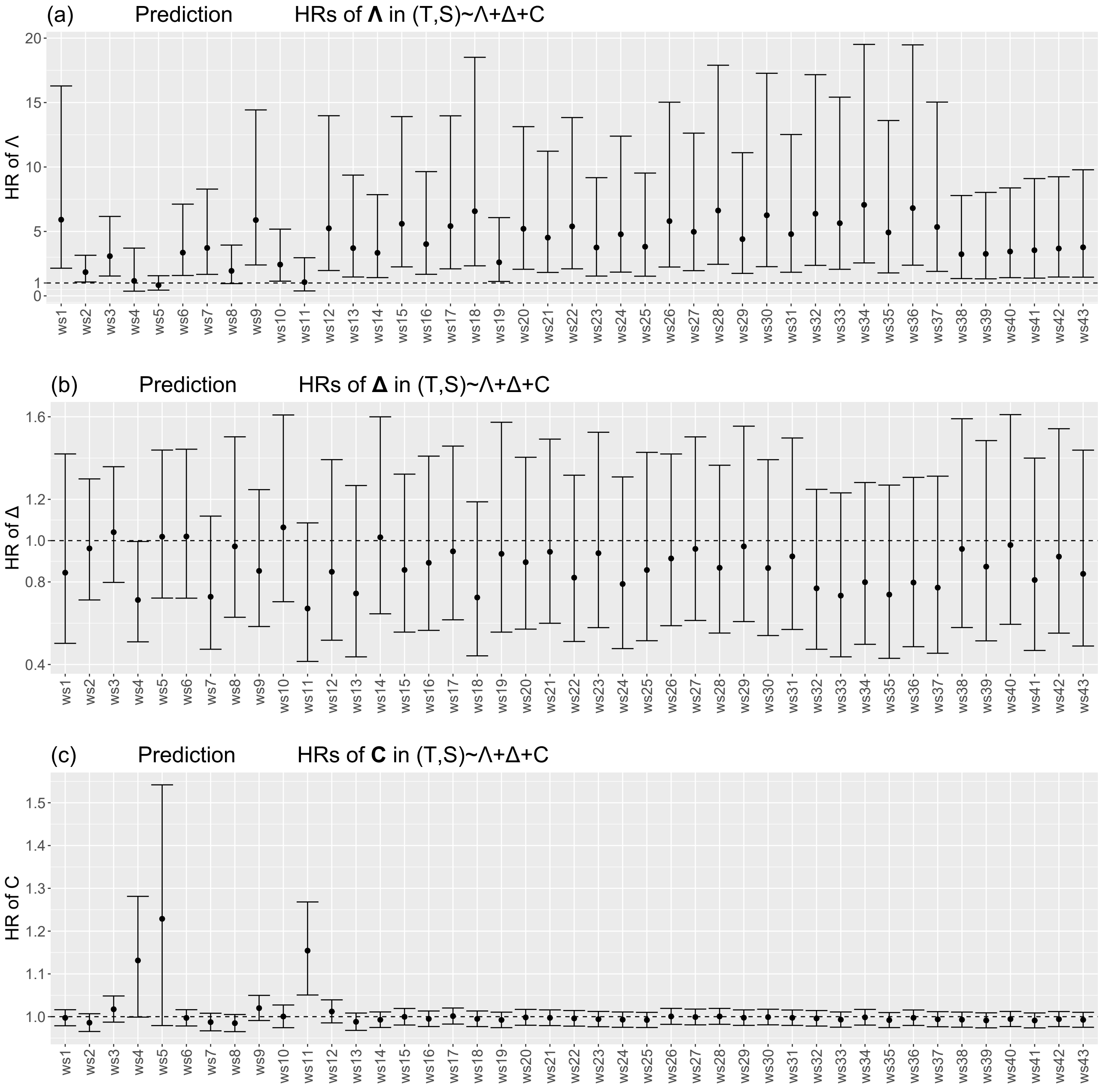

Figure S13: Prediction task: Hazard ratios (HRs) with 95% confidence intervals of the SIRS descriptors (a) Λ, (b) Δ and (c) C in the multivariable Cox proportional hazards model $(T, S) \sim \Lambda + \Delta + C$ for the different weighting schemes ws1 to ws43

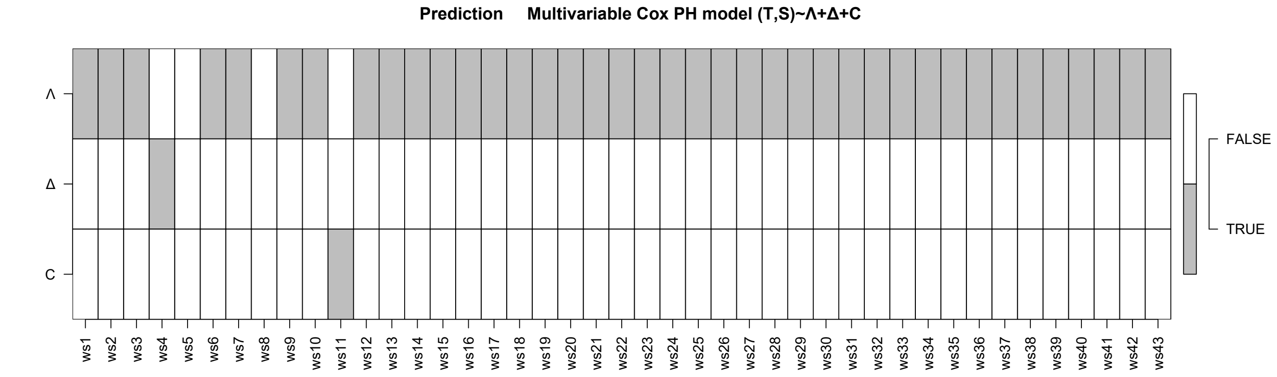

Figure S14: Prediction task: Overview map indicating whether for a specific weighting scheme ws1 to ws43 the SIRS descriptors Λ, Δ and C are significant predictors (TRUE) or not (FALSE) in the multivariable Cox proportional hazards model $(T, S) \sim \Lambda + \Delta + C$, for a significance level of 5%

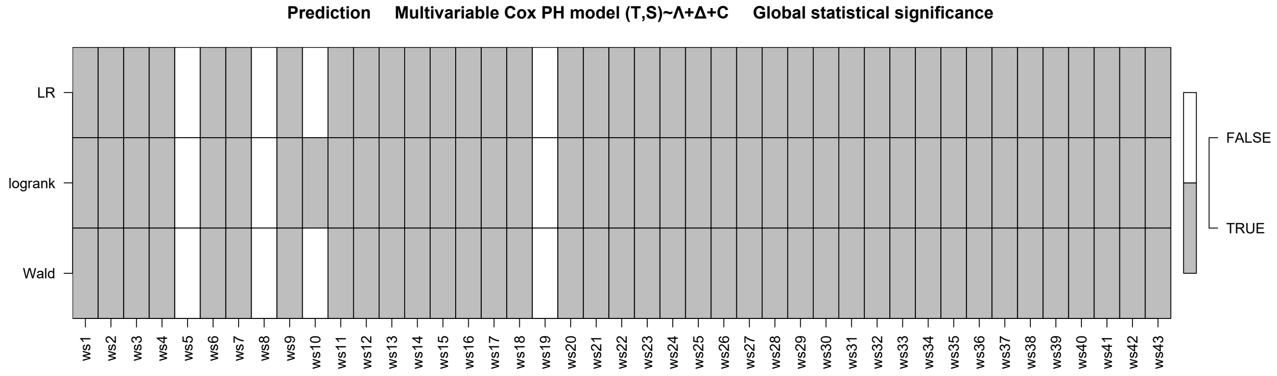

Figure S15: Prediction task: Overview map indicating whether for a specific weighting scheme ws1 to ws43 the multivariable Cox proportional hazards model $(T, S) \sim \Lambda + \Delta + C$ is globally statistically significant (TRUE) or not (FALSE) for a significance level of 5% (i.e., whether the omnibus null hypothesis that all SIRS descriptor coefficients are zero can be rejected or not), considering likelihood-ratio (LR), score logrank (logrank) and Wald tests, respectively

S2.2 Diagnosis task

	Prediction		**Diagnosis**	
Boxplots for SIRS descriptor Λ	Figure S1	p. 5	**Figure S16**	**p. 18**
Boxplots for SIRS descriptor Δ	Figure S2	p. 6	**Figure S17**	**p. 19**
Boxplots for SIRS descriptor C	Figure S3	p. 7	**Figure S18**	**p. 20**
Wilcoxon rank sum test	Figure S4	p. 8	**Figure S19**	**p. 21**
waddR test	Figure S5	p. 8	**Figure S20**	**p. 21**
Univariable logistic regression	Figure S6	p. 9	**Figure S21**	**p. 22**
Multivariable logistic regression	Figure S7	p. 9	**Figure S22**	**p. 22**
AUROC values: Comparison to equal weighting	Figure S8	p. 10	**Figure S23**	**p. 23**
Sensitivity	Figure S9	p. 11	**Figure S24**	**p. 24**
Specificity	Figure S10	p. 12	**Figure S25**	**p. 25**
Calibration slope	Figure S11	p. 13	**Figure S26**	**p. 26**
Calibration intercept	Figure S12	p. 14	**Figure S27**	**p. 27**
Hazard ratios for Λ, Δ and C in multiv. Cox model	Figure S13	p. 15		
Multivariable Cox model	Figure S14	p. 16		
Omnibus Cox model	Figure S15	p. 16		

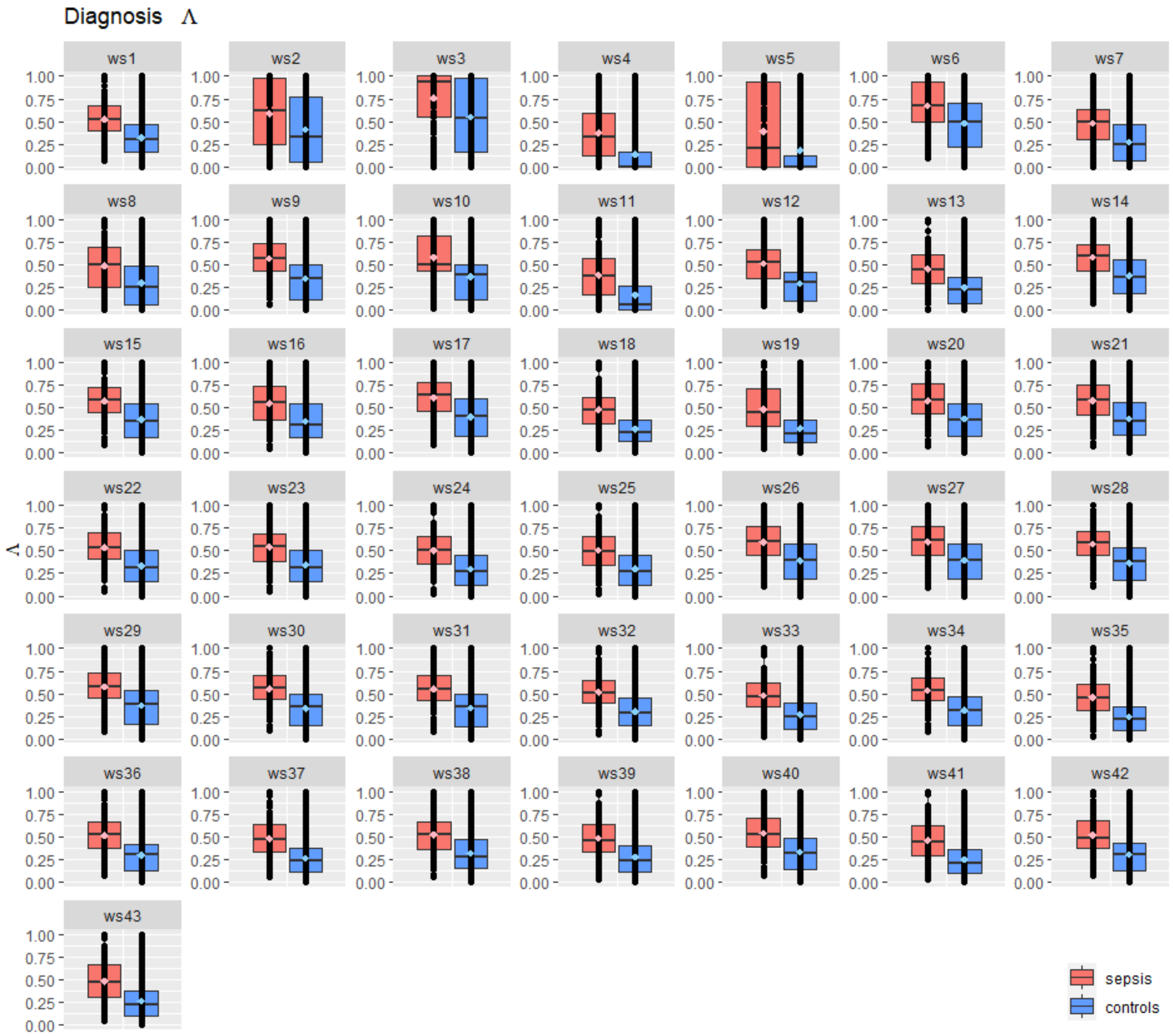

Figure S16: Diagnosis task: Boxplots representing the distributions of the SIRS descriptor Λ (average SIRS level) for the sepsis and control group, respectively, for the different weighting schemes ws1 to ws43. Corresponding means are indicated by diamond symbols.

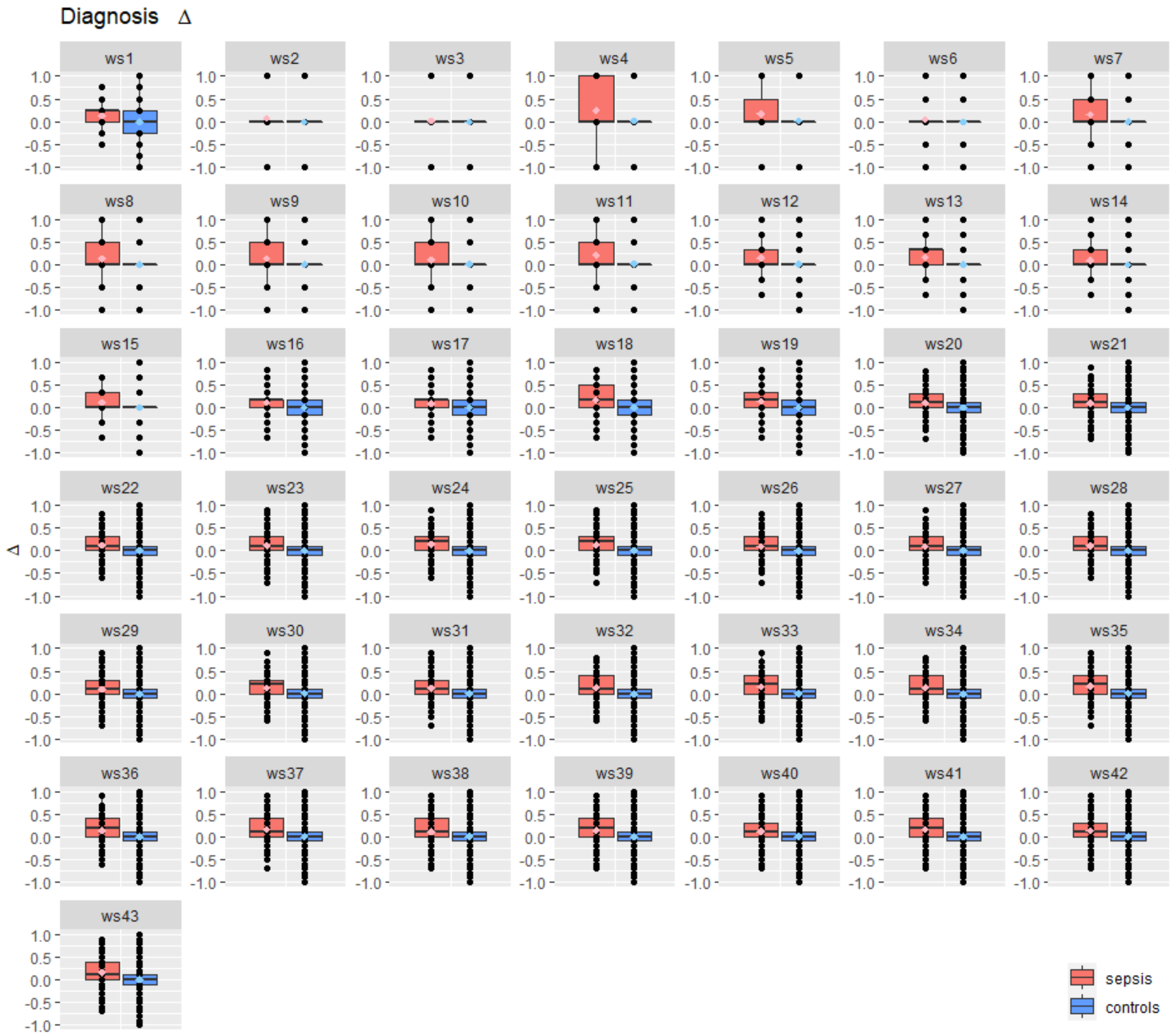

Figure S17: Diagnosis task: Boxplots representing the distributions of the SIRS descriptor Δ (SIRS level trend) for the sepsis and control group, respectively, for the different weighting schemes ws1 to ws43. Corresponding means are indicated by diamond symbols.

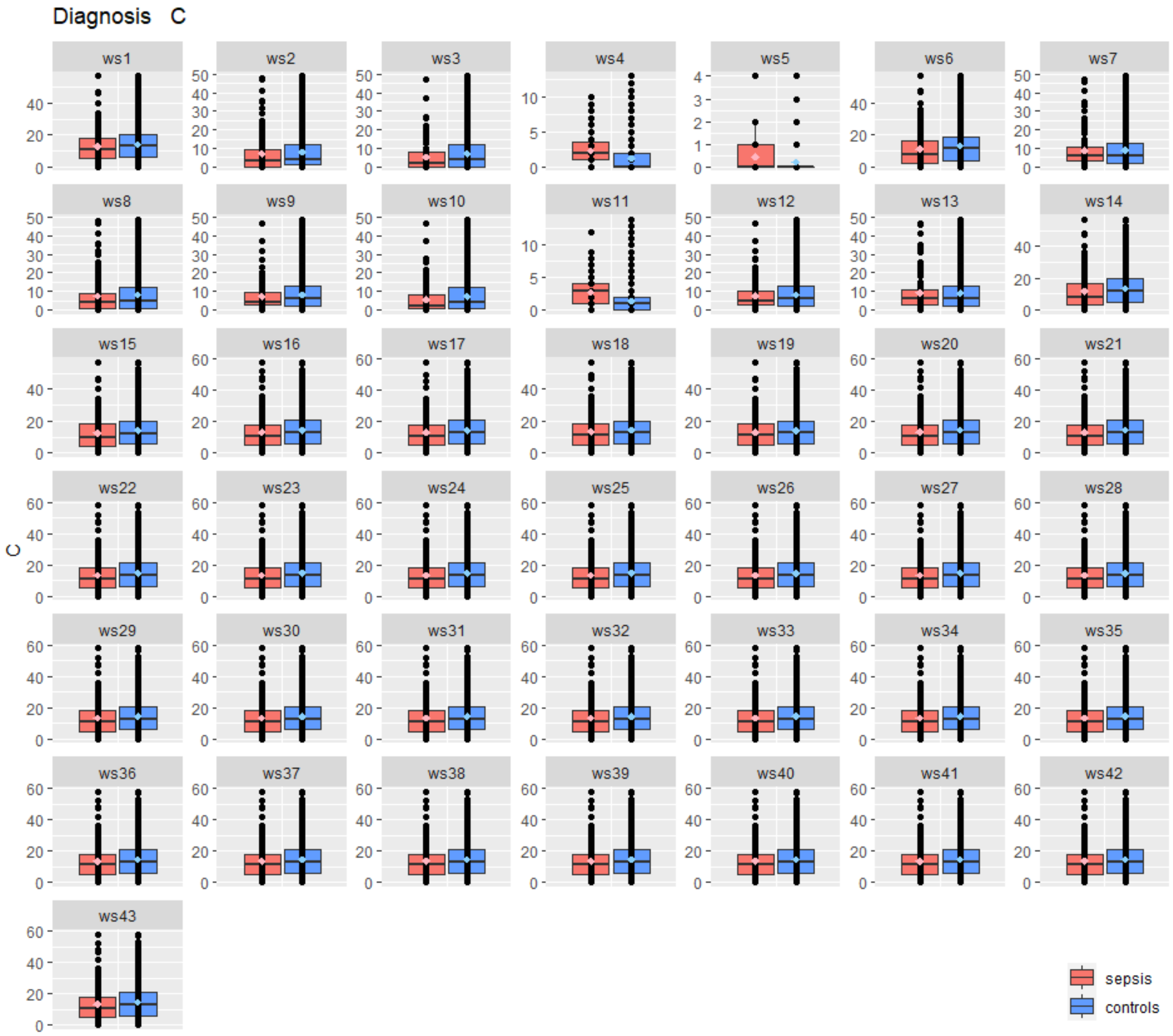

Figure S18: Diagnosis task: Boxplots representing the distributions of the SIRS descriptor C (number of changes in SIRS level) for the sepsis and control group, respectively, for the different weighting schemes ws1 to ws43. Corresponding means are indicated by diamond symbols.

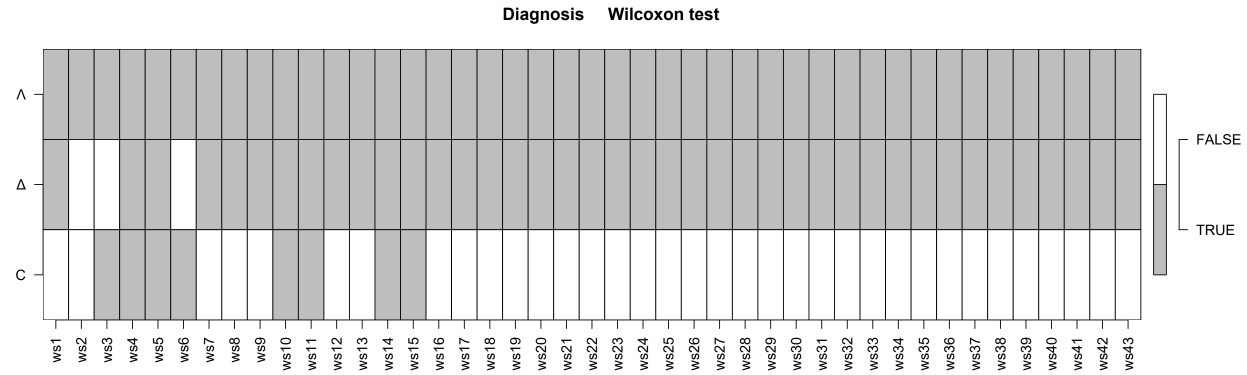

Figure S19: Diagnosis task: For each of the SIRS descriptors Λ, Δ and C and the different weighting schemes ws1 to ws43, results for the Wilcoxon rank sum test at a 5% significance level to check whether the descriptor value for the sepsis group is significantly different from the descriptor value for the control group (TRUE) or not (FALSE): The corresponding cell is colored in grey if the null hypothesis H_0 of no difference can be rejected in favor of the alternative H_1 that there is a difference, and the cell is white if H_0 cannot be rejected.

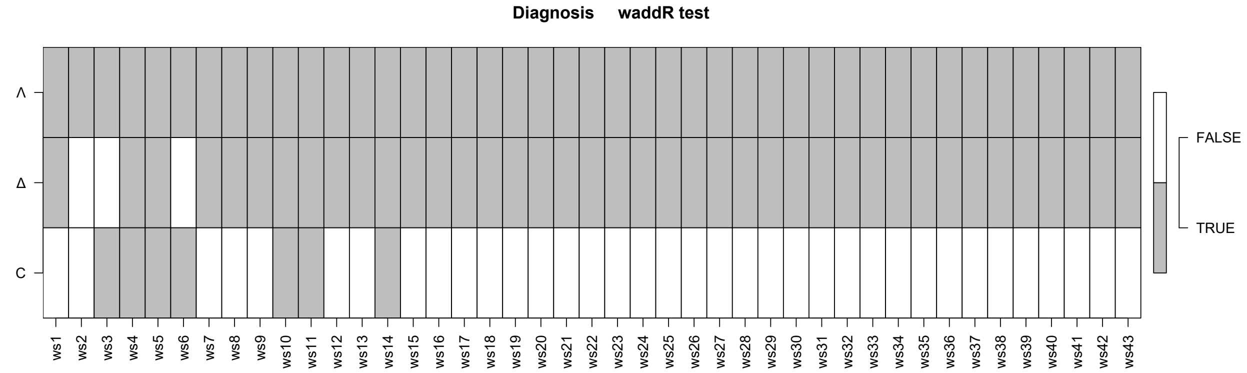

Figure S20: Diagnosis task: For each of the SIRS descriptors Λ, Δ and C and the different weighting schemes ws1 to ws43, results for the waddR test [1] at a 5% significance level to check whether the distribution of the descriptor values for the sepsis group is significantly different from the distribution of the descriptor values for the control group (TRUE) or not (FALSE): The corresponding cell is colored in grey if the null hypothesis H_0 of no difference can be rejected in favor of the alternative H_1 that there is a difference, and the cell is white if H_0 cannot be rejected.

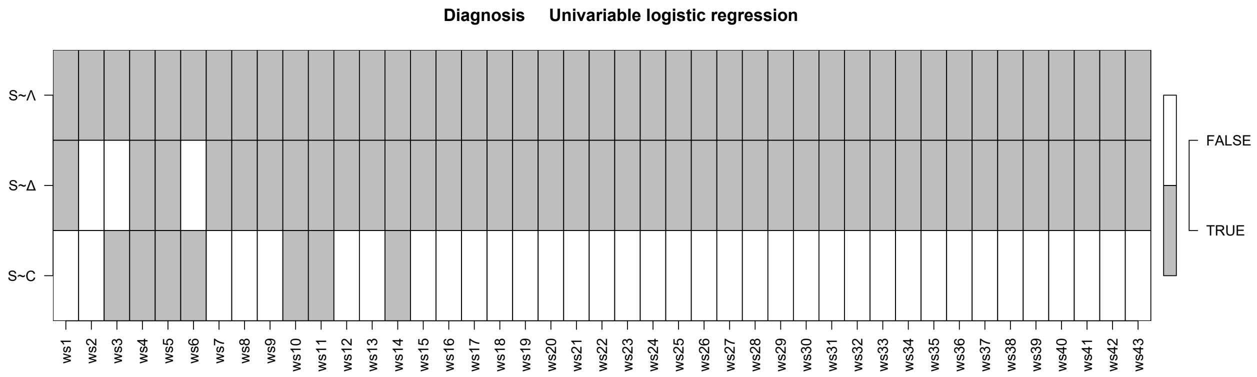

Figure S21: Diagnosis task: Overview map indicating whether for a specific weighting scheme ws1 to ws43 the SIRS descriptor Λ, Δ and C is a significant predictor (TRUE) or not (FALSE) in the corresponding univariable logistic regression model $S \sim \Lambda$, $S \sim \Delta$ and $S \sim C$, respectively, for a significance level of 5%

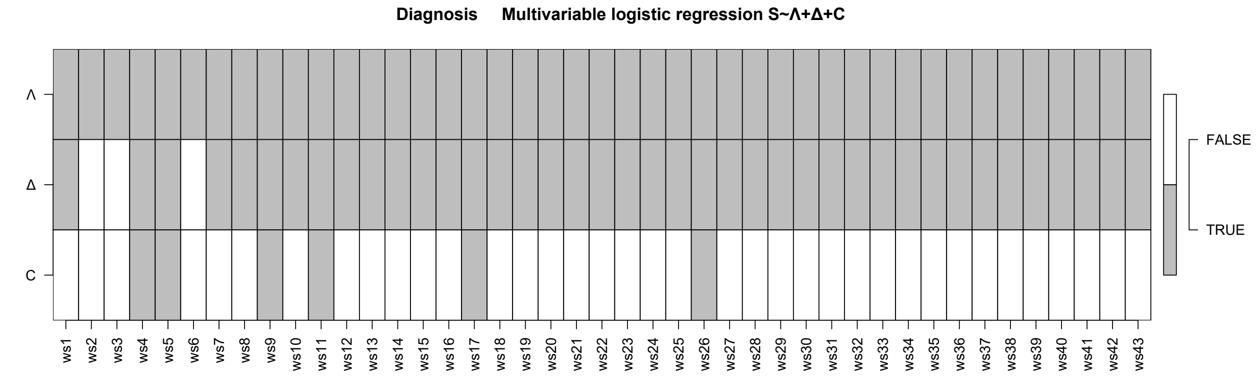

Figure S22: Diagnosis task: Overview map indicating whether for a specific weighting scheme ws1 to ws43 the SIRS descriptors Λ, Δ and C are significant predictors (TRUE) or not (FALSE) in the multivariable logistic regression model $S \sim \Lambda + \Delta + C$, for a significance level of 5%

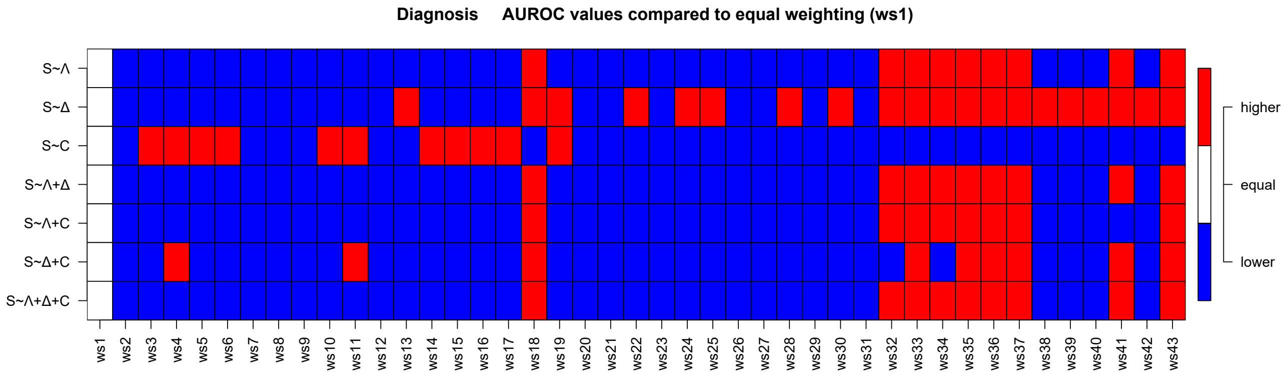

Figure S23: Diagnosis task: Overview of the AUROC values of the weighting schemes ws1 to ws43 in comparison to the equal weighting scheme (ws1) for each considered logistic regression model. This figure accompanies Figure 6 from the main text.

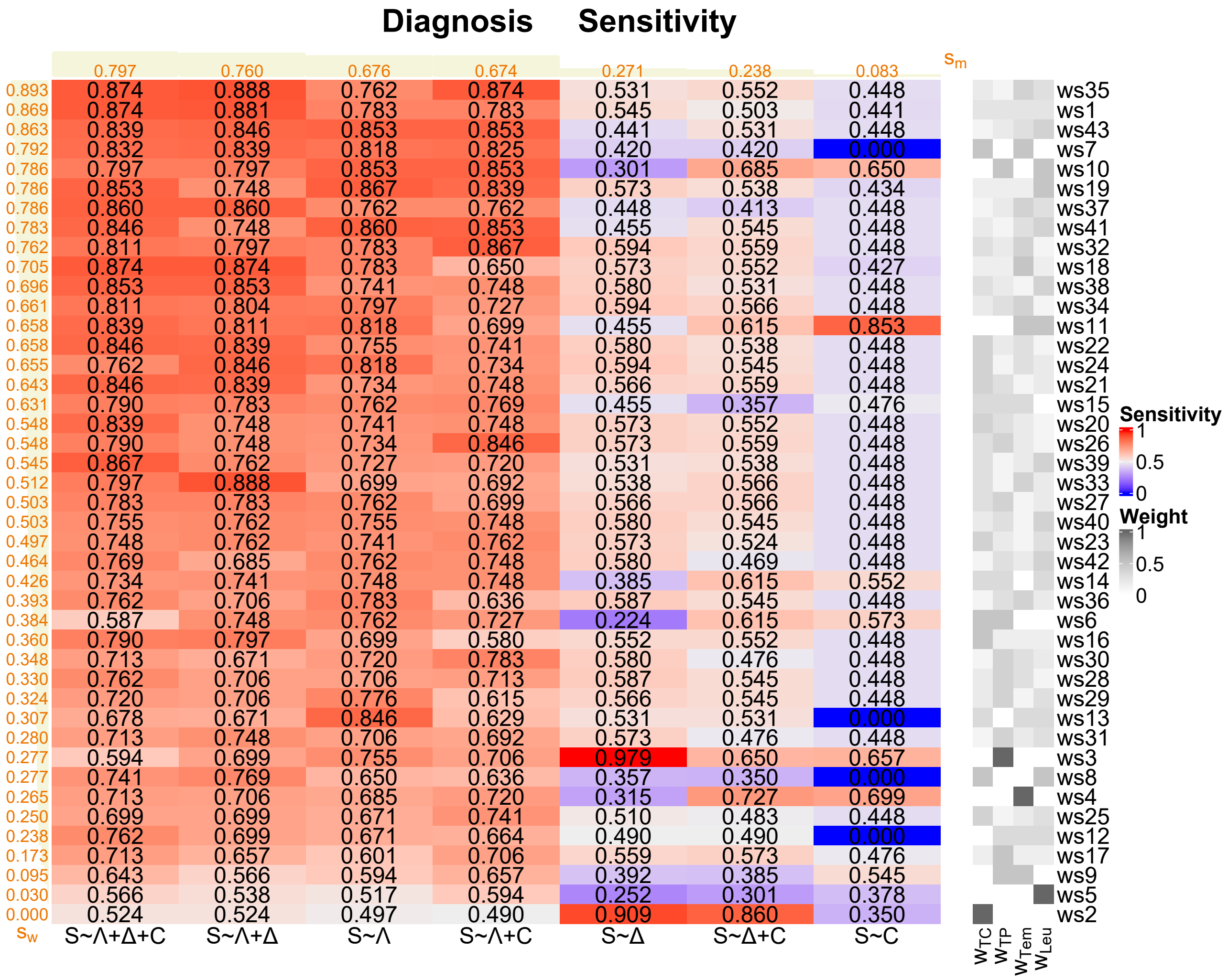

Figure S24: Diagnosis task: Overview of the sensitivity values of the weighting schemes (ws1-ws43) for each considered logistic regression model ($S \sim \Lambda$, $S \sim \Delta$, $S \sim C$, $S \sim \Lambda + \Delta$, $S \sim \Lambda + C$, $S \sim \Delta + C$ and $S \sim \Lambda + \Delta + C$) Weighting schemes (rows) and models (columns) are decreasingly ordered according to their corresponding performances with respect to the ranking score values s_w and s_m (displayed in orange; derived as described in Section S1) from the top to the bottom and from the left to the right, respectively. For convenience, the compositions of the weighting schemes with respect to the four SIRS criteria (i.e., the weights w_{TC}, w_{TP}, w_{Tem} and w_{Leu} for the tachycardia, tachypnea, temperature and leukocytes criterion, respectively) are indicated on the right-hand side of the overall plot, mirroring the specifications in Figure 1.

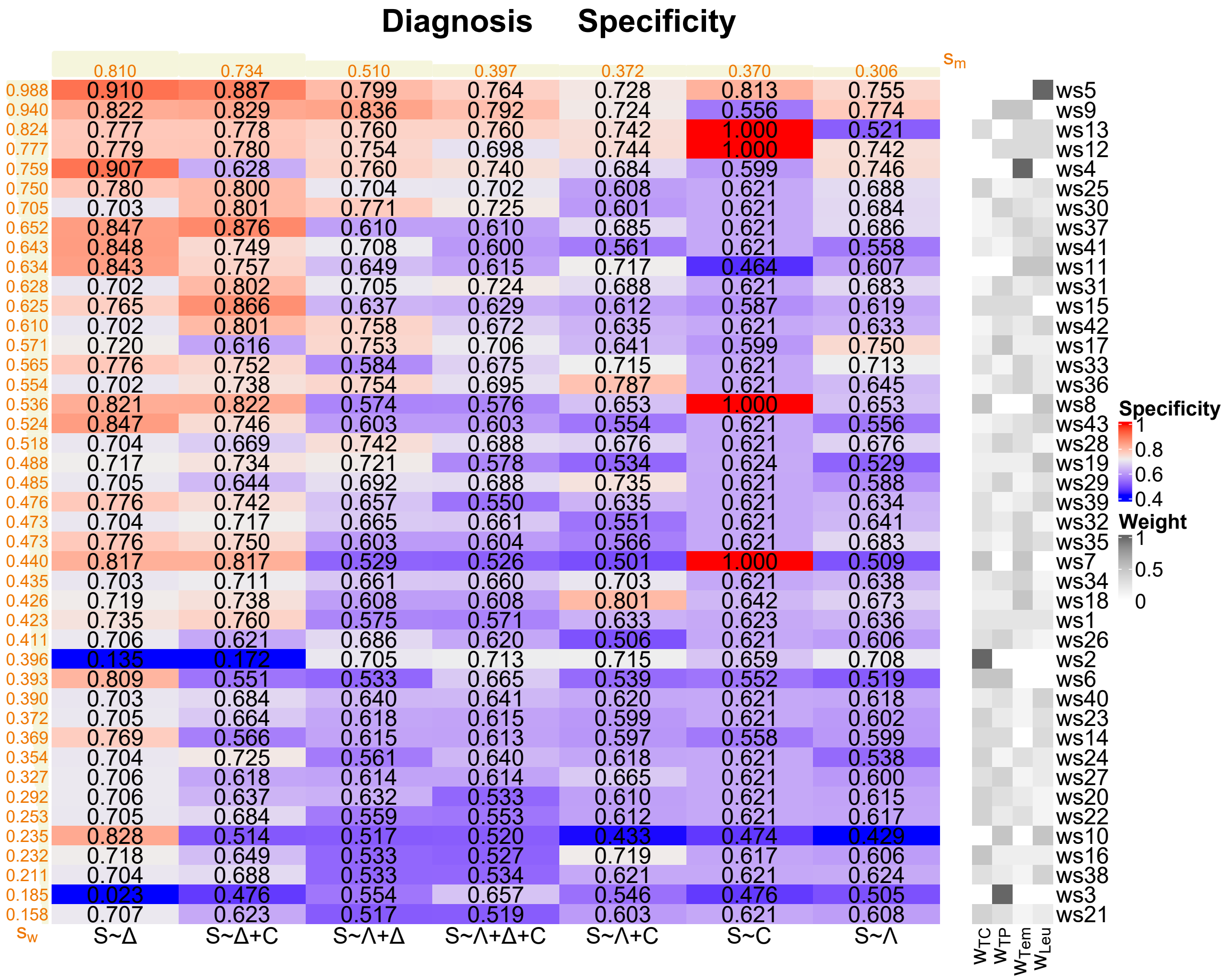

Figure S25: Diagnosis task: Overview of the specificity values of the weighting schemes (ws1-ws43) for each considered logistic regression model ($S \sim \Lambda$, $S \sim \Delta$, $S \sim C$, $S \sim \Lambda + \Delta$, $S \sim \Lambda + C$, $S \sim \Delta + C$ and $S \sim \Lambda + \Delta + C$). Weighting schemes (rows) and models (columns) are decreasingly ordered according to their corresponding performances with respect to the ranking score values s_w and s_m (displayed in orange; derived as described in Section S1) from the top to the bottom and from the left to the right, respectively. For convenience, the compositions of the weighting schemes with respect to the four SIRS criteria (i.e., the weights w_{TC}, w_{TP}, w_{Tem} and w_{Leu} for the tachycardia, tachypnea, temperature and leukocytes criterion, respectively) are indicated on the right-hand side of the overall plot, mirroring the specifications in Figure 1.

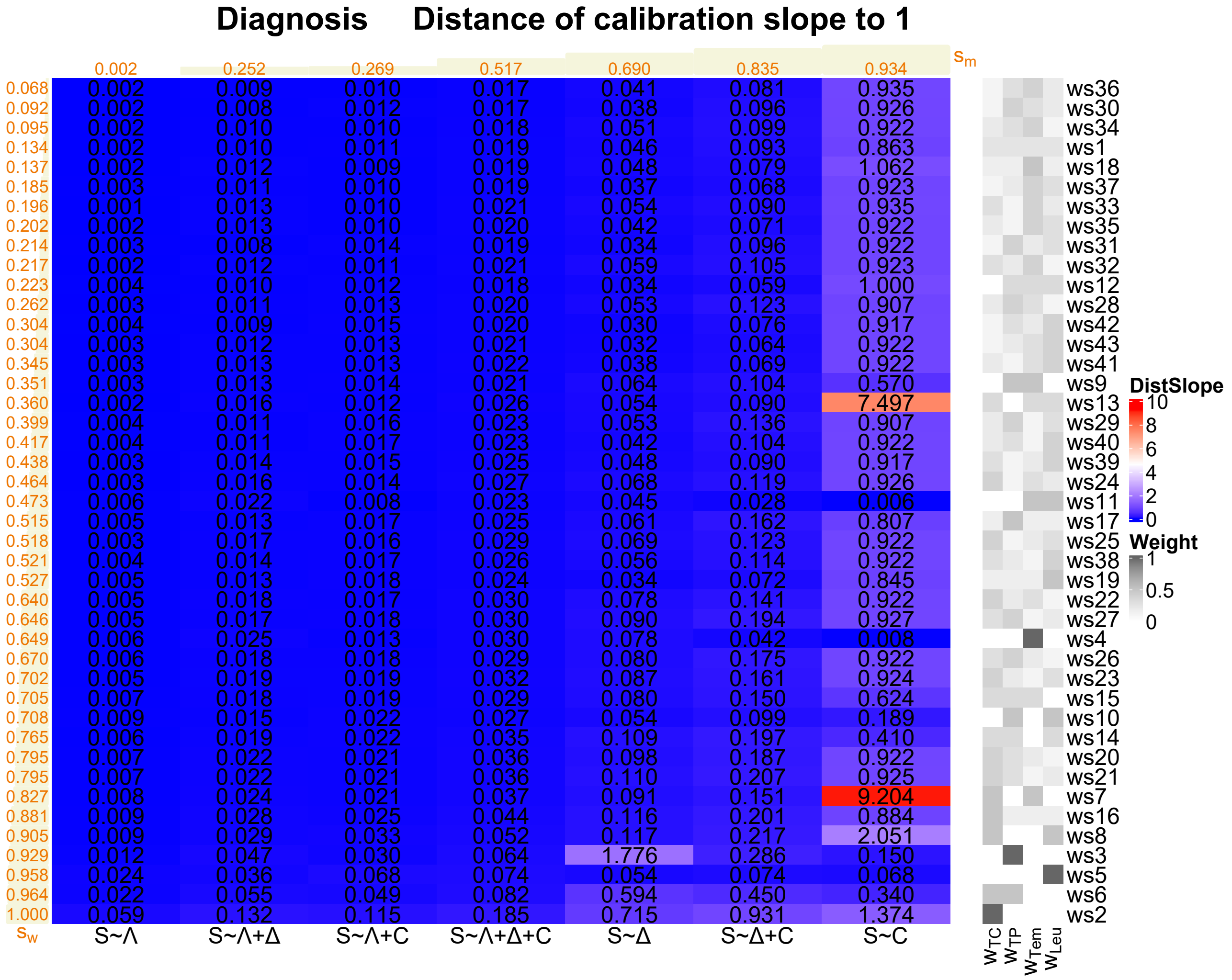

Figure S26: Diagnosis task: Overview of the distances of the calibration slope to the reference value of 1 (indicating good calibration; DistSlope) of the weighting schemes (ws1-ws43) for each considered logistic regression model ($S \sim \Lambda$, $S \sim \Delta$, $S \sim C$, $S \sim \Lambda + \Delta$, $S \sim \Lambda + C$, $S \sim \Delta + C$ and $S \sim \Lambda + \Delta + C$). Weighting schemes (rows) and models (columns) are decreasingly ordered according to their corresponding performances with respect to the ranking score values s_w and s_m (displayed in orange; derived as described in Section S1) from the top to the bottom and from the left to the right, respectively. Note that here, the performance measures are negatively oriented (i.e., the smaller the value the better the performance). For convenience, the compositions of the weighting schemes with respect to the four SIRS criteria (i.e., the weights w_{TC}, w_{TP}, w_{Tem} and w_{Leu} for the tachycardia, tachypnea, temperature and leukocytes criterion, respectively) are indicated on the right-hand side of the overall plot, mirroring the specifications in Figure 1.

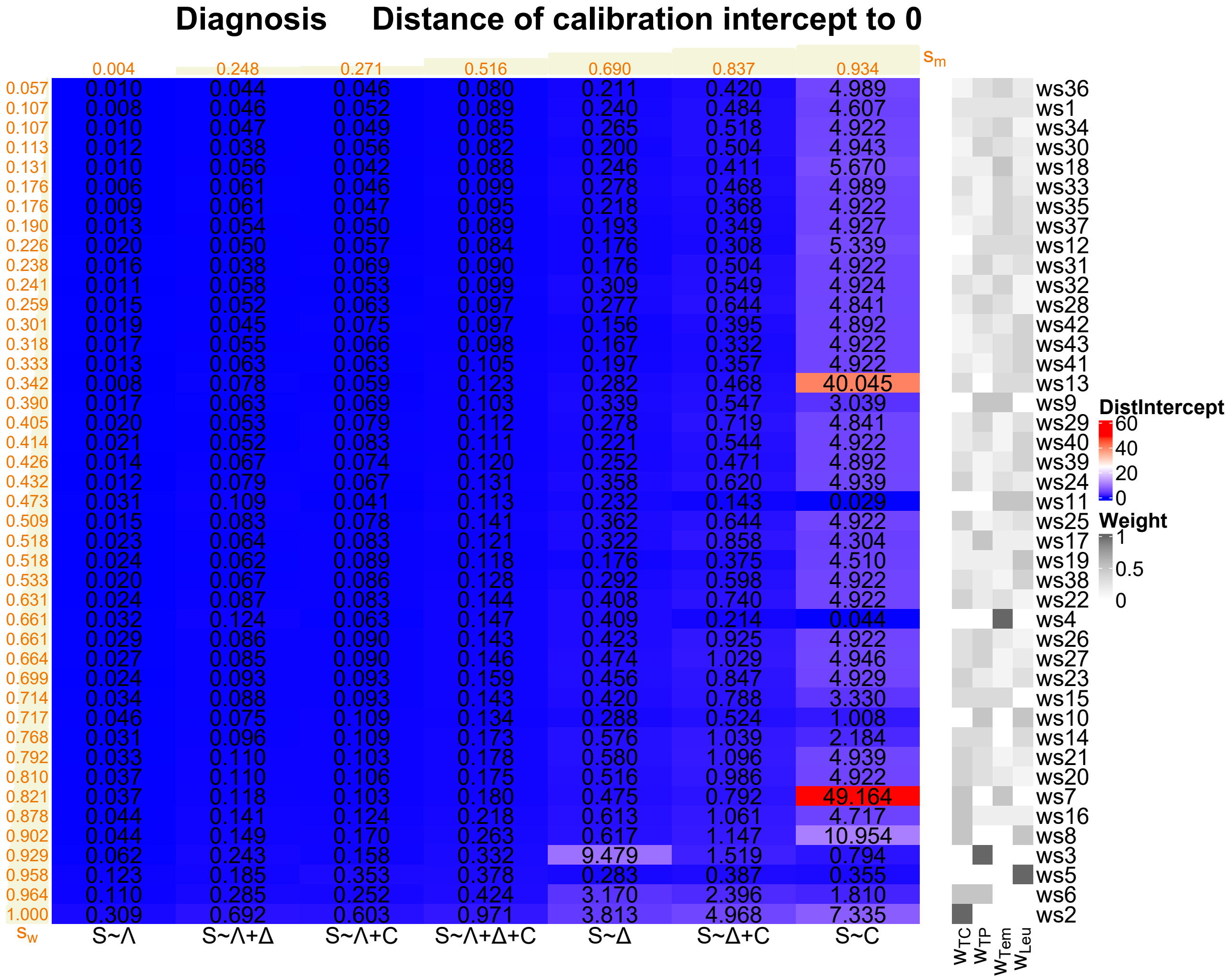

Figure S27: Diagnosis task: Overview of the distances of the calibration intercept to the reference value of 0 (indicating good calibration; DistIntercept) of the weighting schemes (ws1-ws43) for each considered logistic regression model ($S \sim \Lambda$, $S \sim \Delta$, $S \sim C$, $S \sim \Lambda{+}\Delta$, $S \sim \Lambda{+}C$, $S \sim \Delta{+}C$ and $S \sim \Lambda{+}\Delta{+}C$). Weighting schemes (rows) and models (columns) are decreasingly ordered according to their corresponding performances with respect to the ranking score values s_w and s_m (displayed in orange; derived as described in Section S1) from the top to the bottom and from the left to the right, respectively. Note that here, the performance measures are negatively oriented (i.e., the smaller the value the better the performance). For convenience, the compositions of the weighting schemes with respect to the four SIRS criteria (i.e., the weights w_{TC}, w_{TP}, w_{Tem} and w_{Leu} for the tachycardia, tachypnea, temperature and leukocytes criterion, respectively) are indicated on the right-hand side of the overall plot, mirroring the specifications in Figure 1.

S3 Supplementary results for the SIRS Prospective algorithm: Initial inspection of temporal evolution

S3.1 Prediction task

During the first 24 hours after ICU admission, more encounters fulfil the tachypnea or the tachycardia criterion in the sepsis group than in the no sepsis group, whereas the temporal evolution of the fulfillment of the leukocytes and temperature criteria is rather similar for the sepsis and the no sepsis group (Figure S28).
Based on a visual inspection of Figure S29, the average SIRS levels over encounters for the sepsis group remain higher than those for the no sepsis group throughout the 1440 minutes for all weighting schemes, except for ws5 (giving weight only to the leukocytes criterion). However, the difference appears to be not that pronounced for those schemes giving high weight to the leukocytes criterion and zero or low weight to the temperature and/or the tachypnea criterion (e.g. for ws8, ws10, ws11 or ws19). In particular, the behavior of the time series for ws5 in Figure S29 mirrors well the bad performance of ws5 in terms of AUROC values shown in Figure 4 in the main text. In contrast, rather obvious differences based on visual inspection appear to exist for those weighting schemes giving high weight to the temperature and/or tachypnea criterion and zero or low weight to the leukocytes criterion (e.g. for ws3, ws4, ws6, ws18, ws26, ws32 or ws34).

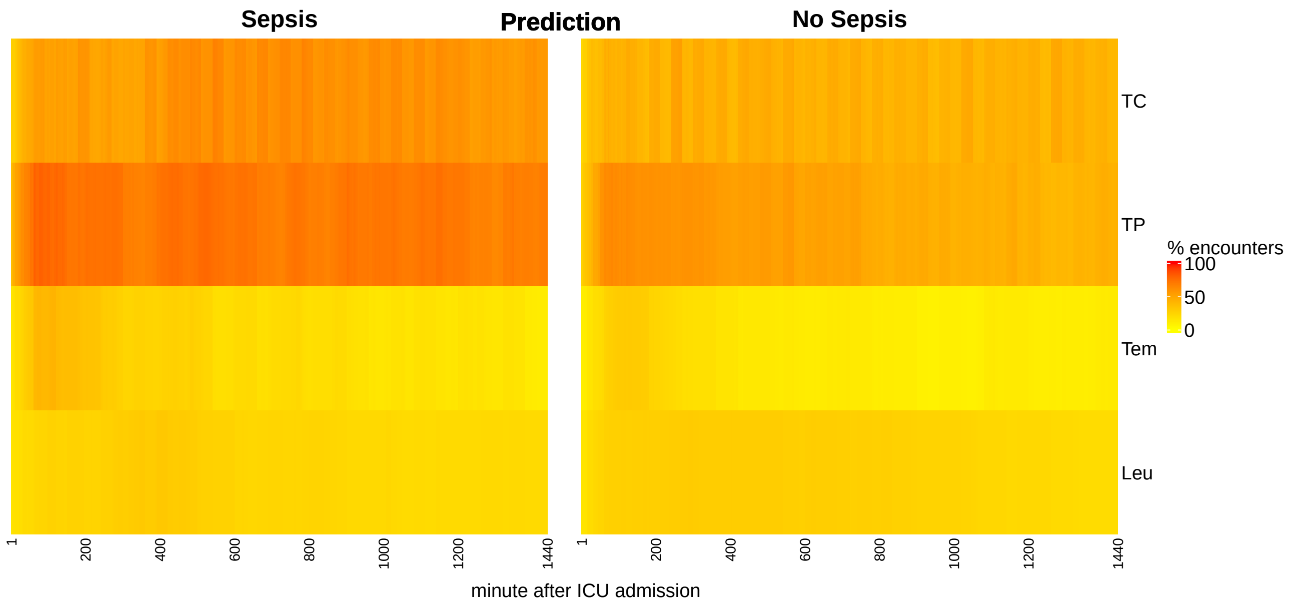

Figure S28: Prediction task: Temporal evolution of the percentages of encounters with sepsis or no sepsis/controls, respectively, fulfilling the SIRS criteria (tachycardia, TC; tachypnea, TP; temperature, Tem; leukocytes, Leu)

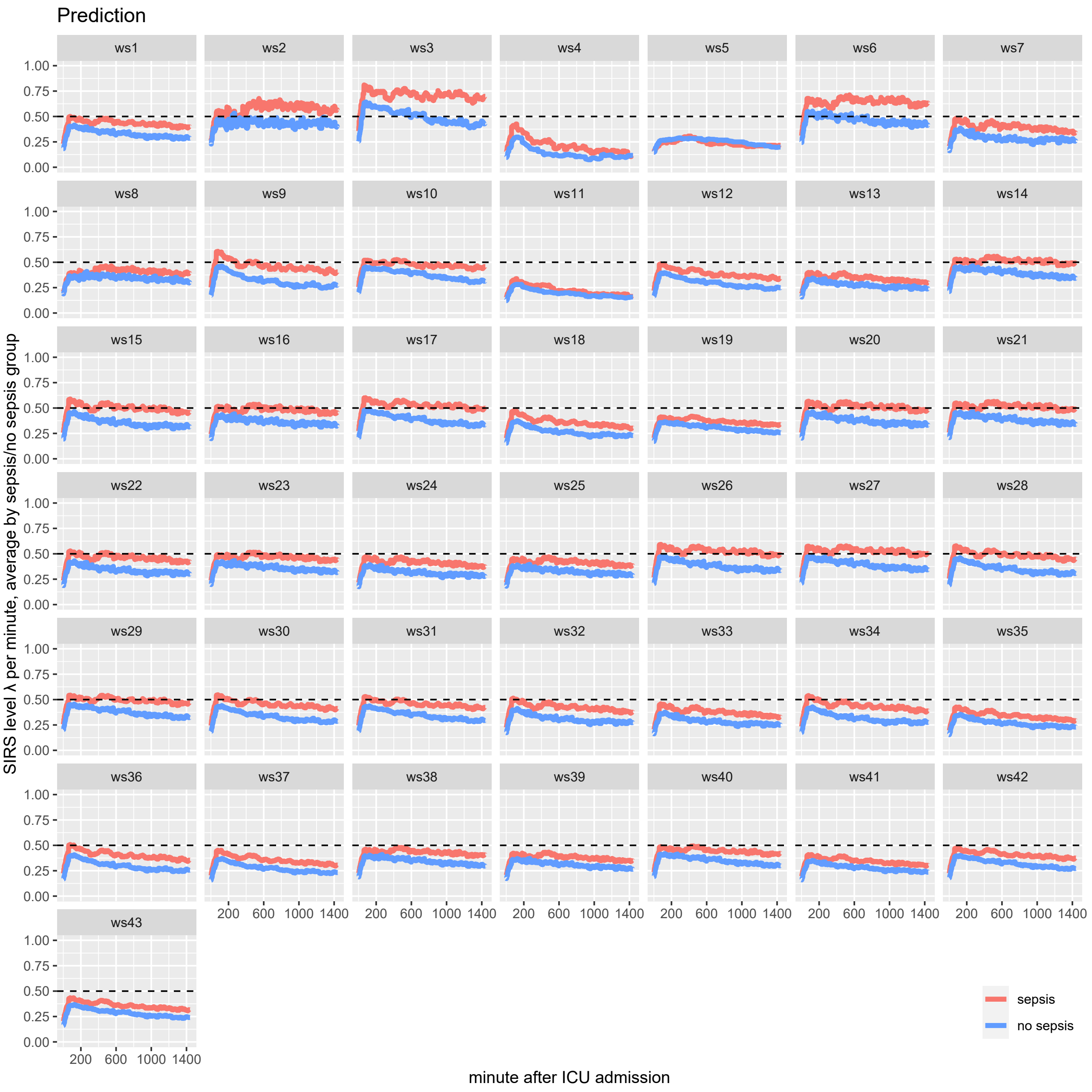

Figure S29: Prediction task: SIRS level λ per minute for minutes 1 to 1440 after ICU admission, average by the sepsis and no sepsis group, respectively, for each of the weighting schemes ws1 to ws43

S3.2 Diagnosis task

During the last 24 hours before index (sepsis) time, there are throughout more encounters fulfilling a respective SIRS criterion in the sepsis group than in the control group, where the difference is pronounced for the temperature and leukocytes criteria, medium for the tachypnea criterion and rather small for the tachycardia criterion (Figure S30). Comparing the prediction and the diagnosis task, the differences in occurrences between the sepsis and the control group get bigger during the course of the ICU stay for the temperature and the leukocytes criteria, in that there are more septic encounters fulfilling these criteria. On the contrary, the differences in occurrences between the sepsis and the control group change only marginally during the course of the ICU stay for the tachycardia and the tachypnea criteria (Figures S28 and S30).
In the diagnosis task, the average SIRS levels over encounters for the sepsis group are higher than those for the control group throughout all weighting schemes (visual inspection of Figure S31). In the sepsis group, the average SIRS levels over encounters increase over time when approaching the sepsis (index) time point, whereas the average SIRS level over encounters is virtually more or less constant in the control group (Figure S31).

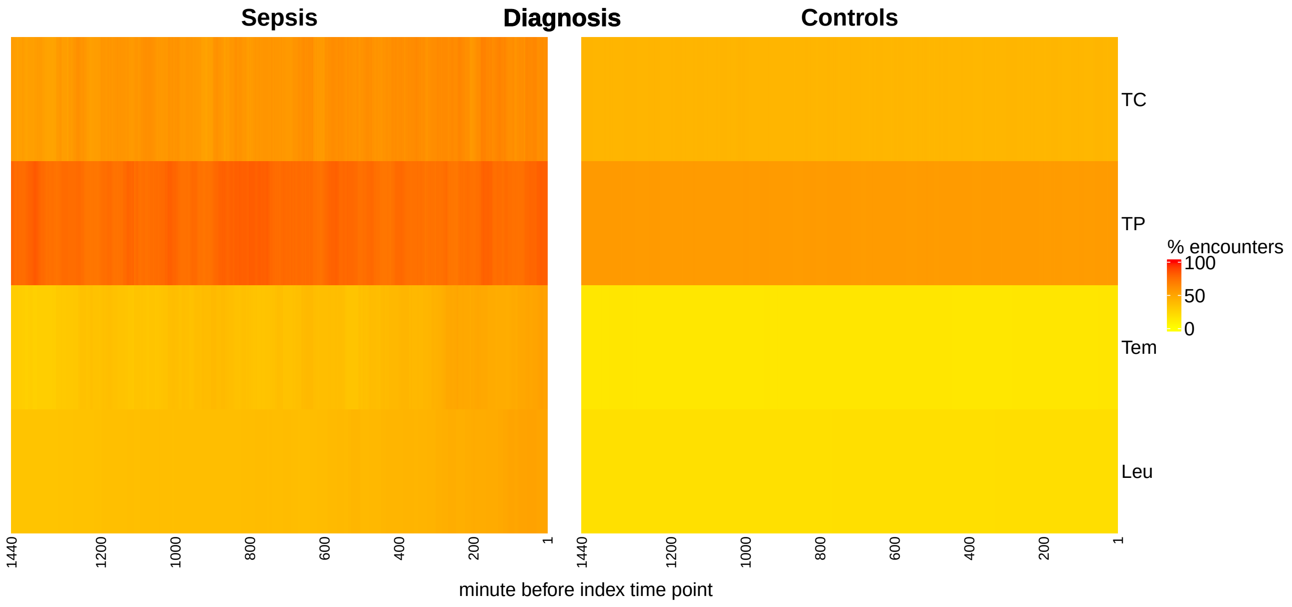

Figure S30: Diagnosis task: Temporal evolution of the percentages of encounters with sepsis or no sepsis/controls, respectively, fulfilling the SIRS criteria (tachycardia, TC; tachypnea, TP; temperature, Tem; leukocytes, Leu)

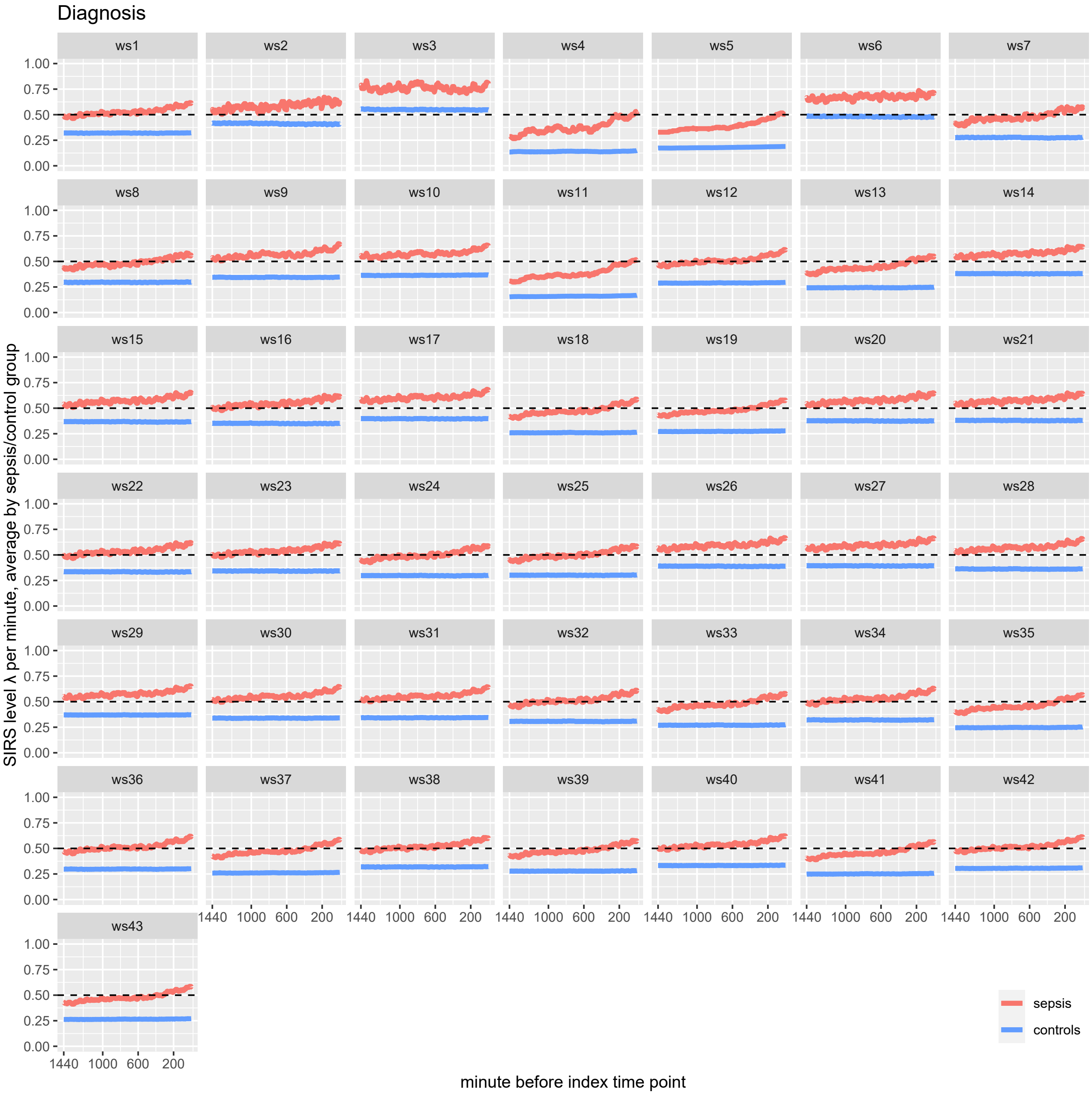

Figure S31: Diagnosis task: SIRS level λ per minute for minutes 1 to 1440 before index/sepsis time point, average by the sepsis and control group, respectively, for each of the weighting schemes ws1 to ws43

References

[1] R. Schefzik, J. Flesch, and A. Goncalves. Fast identification of differential distributions in single-cell RNA-sequencing data with waddR. *Bioinformatics*, 37:3204–3211, 2021.
