# Supplementary material for: Dissecting contributions of individual systemic inflammatory response syndrome criteria from a prospective algorithm to the prediction and diagnosis of sepsis in a polytrauma cohort
Source: Front Med (Lausanne). 2023 Jul 31;10:1227031. doi: 10.3389/fmed.2023.1227031 (PMC10424878; doi:10.3389/fmed.2023.1227031)
Supplement: Supplementary file 2 [file Data_Sheet_2.pdf]

Supplementary Material 2

Dissecting contributions of individual systemic inflammatory response syndrome criteria from a prospective algorithm to the prediction and diagnosis of sepsis in a polytrauma cohort

Roman Schefzik, Bianka Hahn and Verena Schneider-Lindner

Here, we expand on the comparison of the four algorithms SIRS Conventional, SIRS Non-ICU, SIRS Retrospective and SIRS Prospective for the sepsis **prediction** task from Figure 7 in the main text and provide the corresponding ROC curves and AUROC values for all our 301 considered scenarios (43 weighting schemes $\times$ 7 logistic regression models). In particular, we consider the weighting schemes ws1 to ws43 and the models $S \sim \Lambda$, $S \sim \Delta$, $S \sim C$, $S \sim \Lambda + \Delta$, $S \sim \Lambda + C$, $S \sim \Delta + C$ and $S \sim \Lambda + \Delta + C$ as described in the main text.

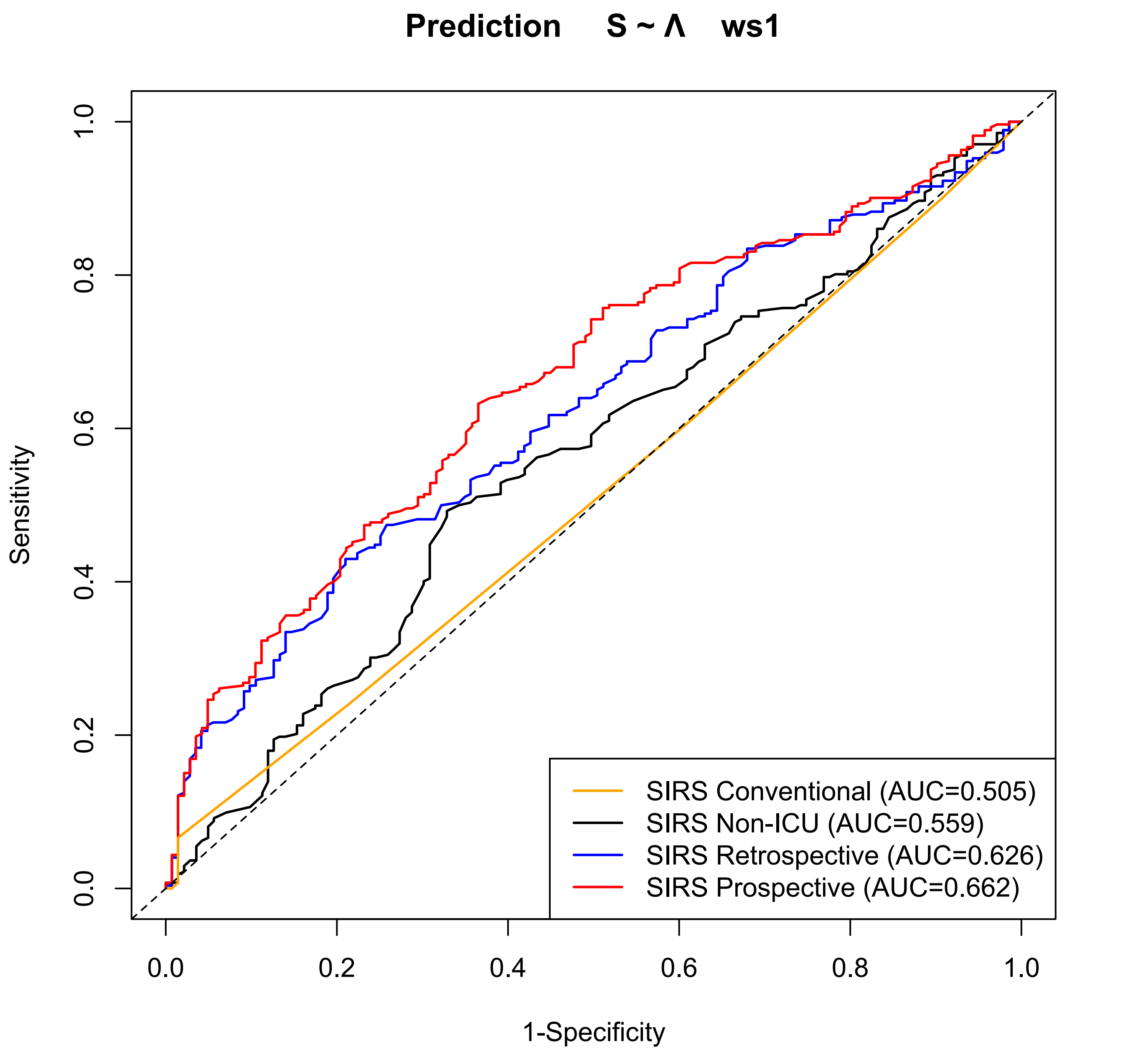

Prediction S ~ Λ ws1
Sensitivity
1-Specificity
0.0
0.2
0.4
0.6
0.8
1.0
0.0
0.2
0.4
0.6
0.8
1.0
SIRS Conventional (AUC=0.505)
SIRS Non-ICU (AUC=0.559)
SIRS Retrospective (AUC=0.626)
SIRS Prospective (AUC=0.662)

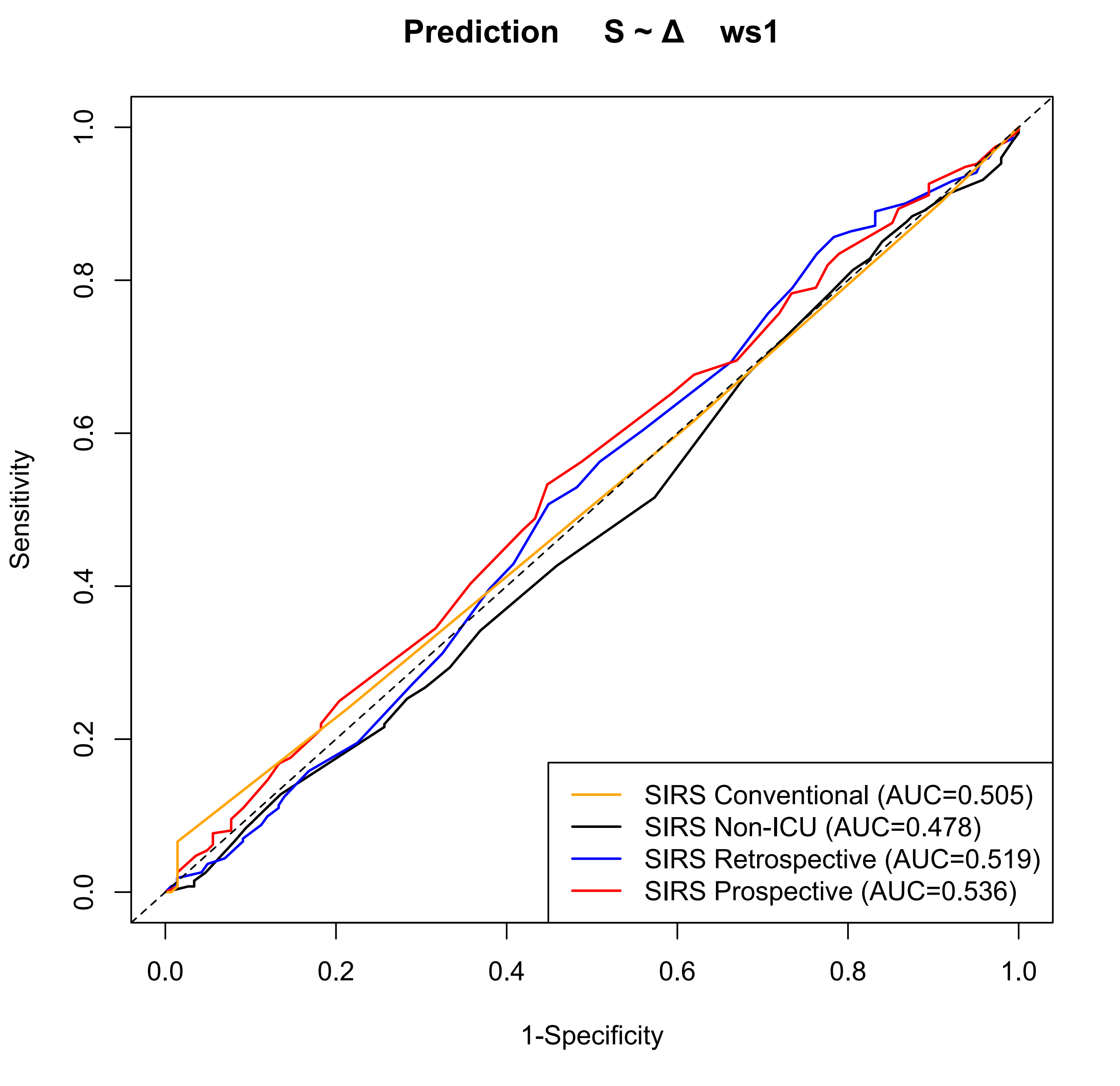

Prediction S ~ Δ ws1
Sensitivity
1-Specificity
0.0
0.2
0.4
0.6
0.8
1.0
SIRS Conventional (AUC=0.505)
SIRS Non-ICU (AUC=0.478)
SIRS Retrospective (AUC=0.519)
SIRS Prospective (AUC=0.536)

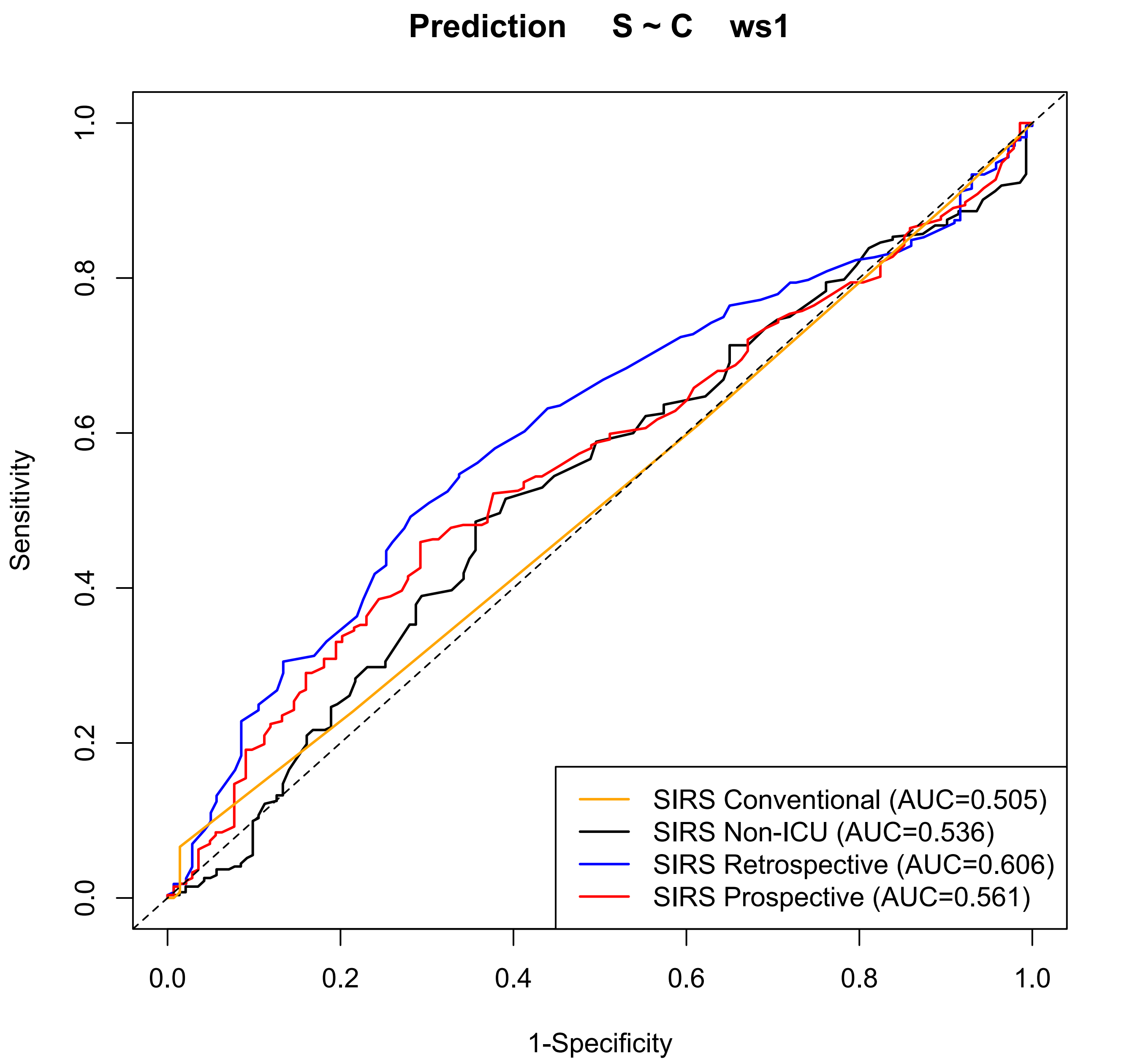
Prediction S ~ C ws1
Sensitivity
1-Specificity
0.0
0.2
0.4
0.6
0.8
1.0
SIRS Conventional (AUC=0.505)
SIRS Non-ICU (AUC=0.536)
SIRS Retrospective (AUC=0.606)
SIRS Prospective (AUC=0.561)

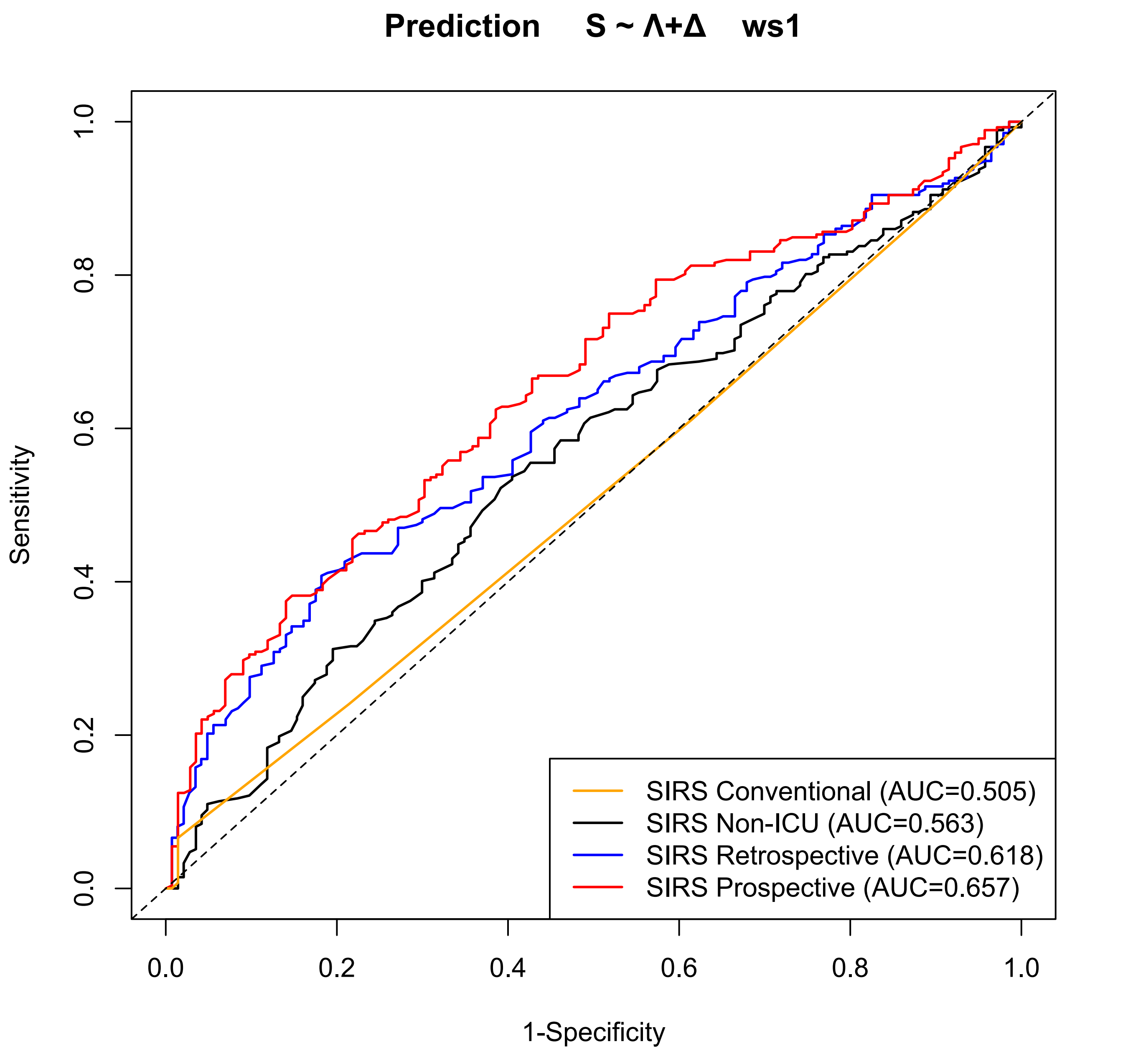

Prediction S ~ Λ+Δ ws1
Sensitivity
1-Specificity
0.0
0.2
0.4
0.6
0.8
1.0
SIRS Conventional (AUC=0.505)
SIRS Non-ICU (AUC=0.563)
SIRS Retrospective (AUC=0.618)
SIRS Prospective (AUC=0.657)

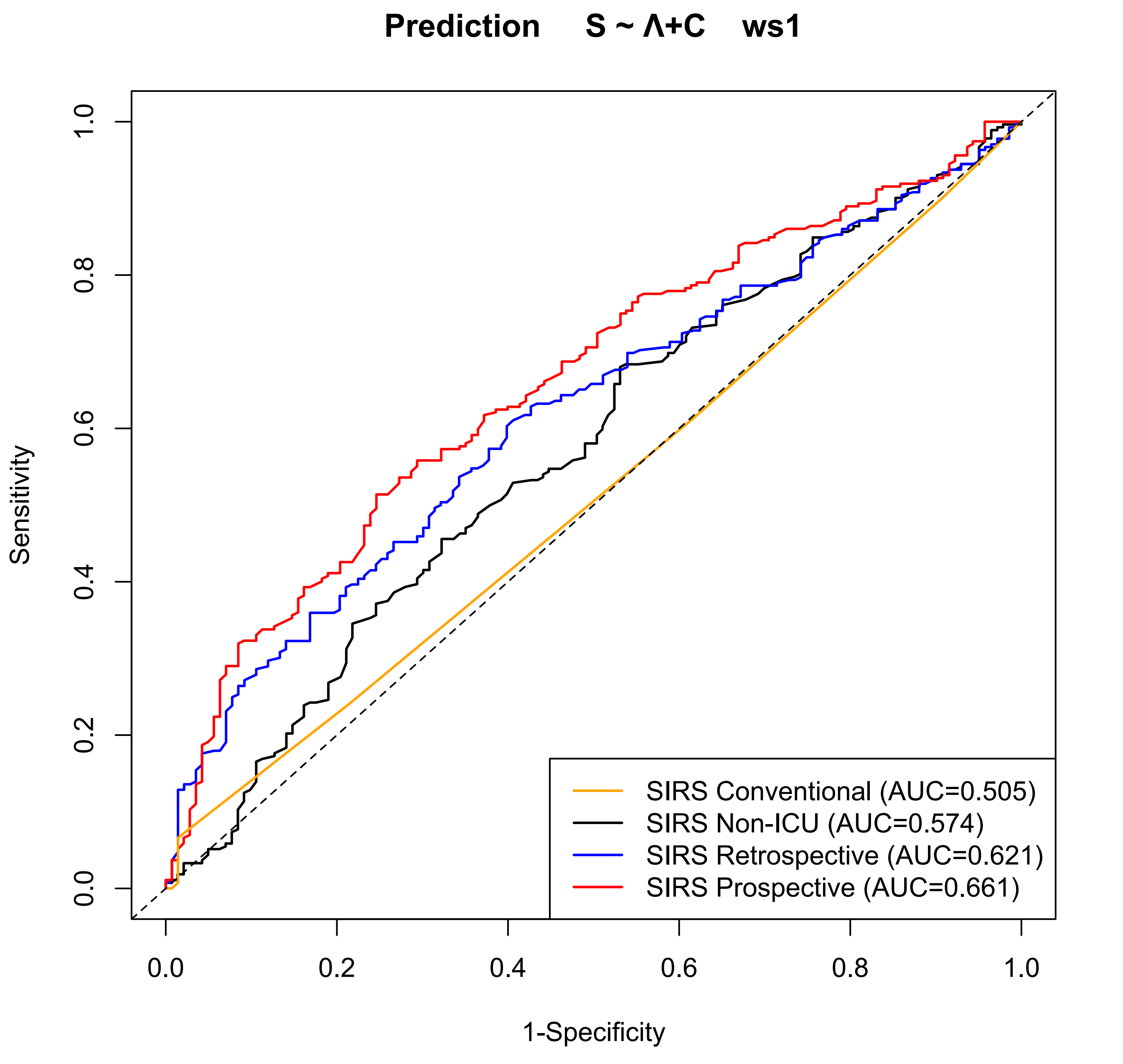

Prediction S ~ Λ+C ws1
Sensitivity
1-Specificity
0.0
0.2
0.4
0.6
0.8
1.0
SIRS Conventional (AUC=0.505)
SIRS Non-ICU (AUC=0.574)
SIRS Retrospective (AUC=0.621)
SIRS Prospective (AUC=0.661)

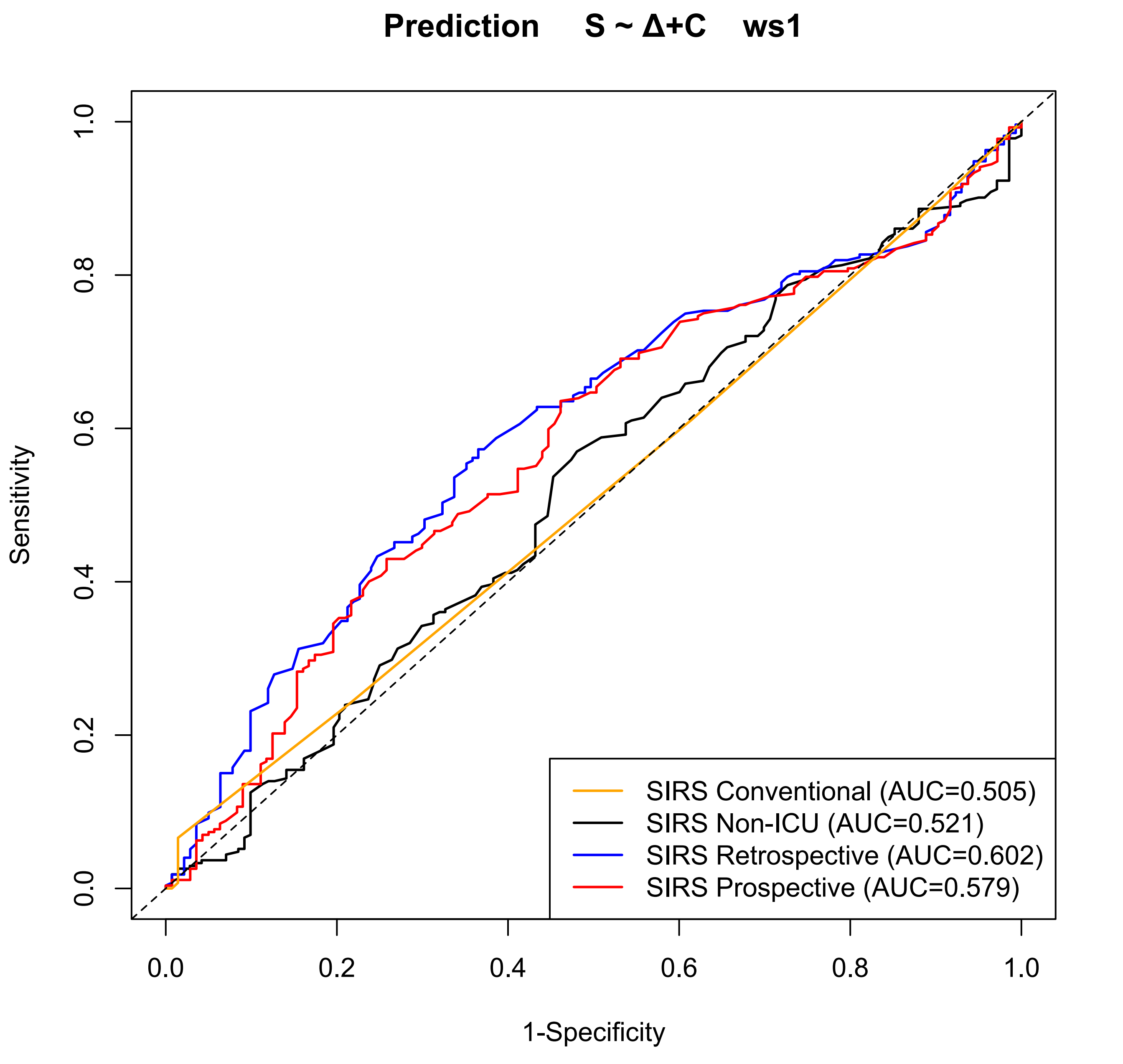

Prediction S ~ Δ+C ws1
Sensitivity
1-Specificity
0.0
0.2
0.4
0.6
0.8
1.0
0.0
0.2
0.4
0.6
0.8
1.0
SIRS Conventional (AUC=0.505)
SIRS Non-ICU (AUC=0.521)
SIRS Retrospective (AUC=0.602)
SIRS Prospective (AUC=0.579)

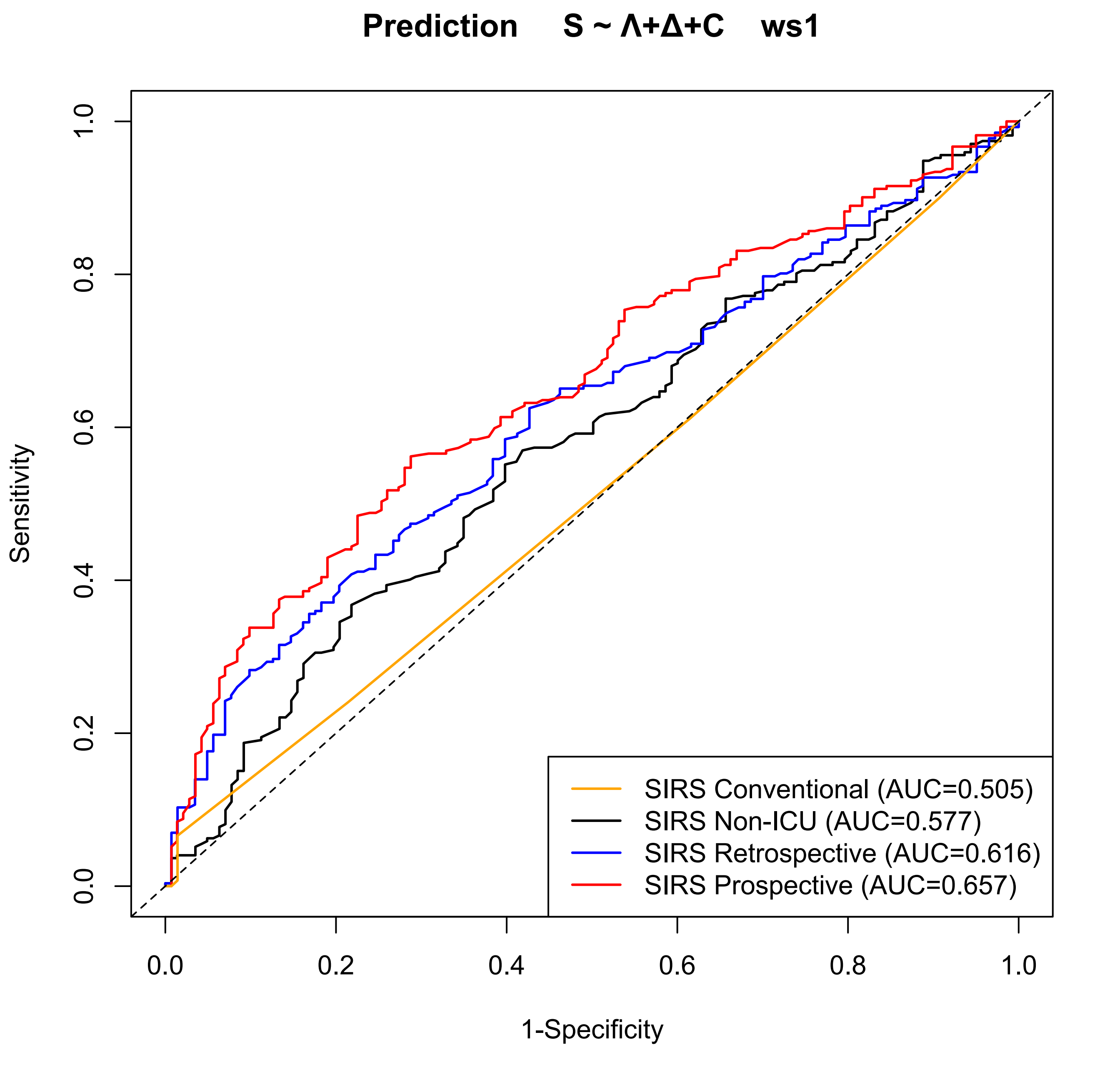

Prediction S ~ Λ+Δ+C ws1
Sensitivity
1-Specificity
0.0
0.2
0.4
0.6
0.8
1.0
SIRS Conventional (AUC=0.505)
SIRS Non-ICU (AUC=0.577)
SIRS Retrospective (AUC=0.616)
SIRS Prospective (AUC=0.657)

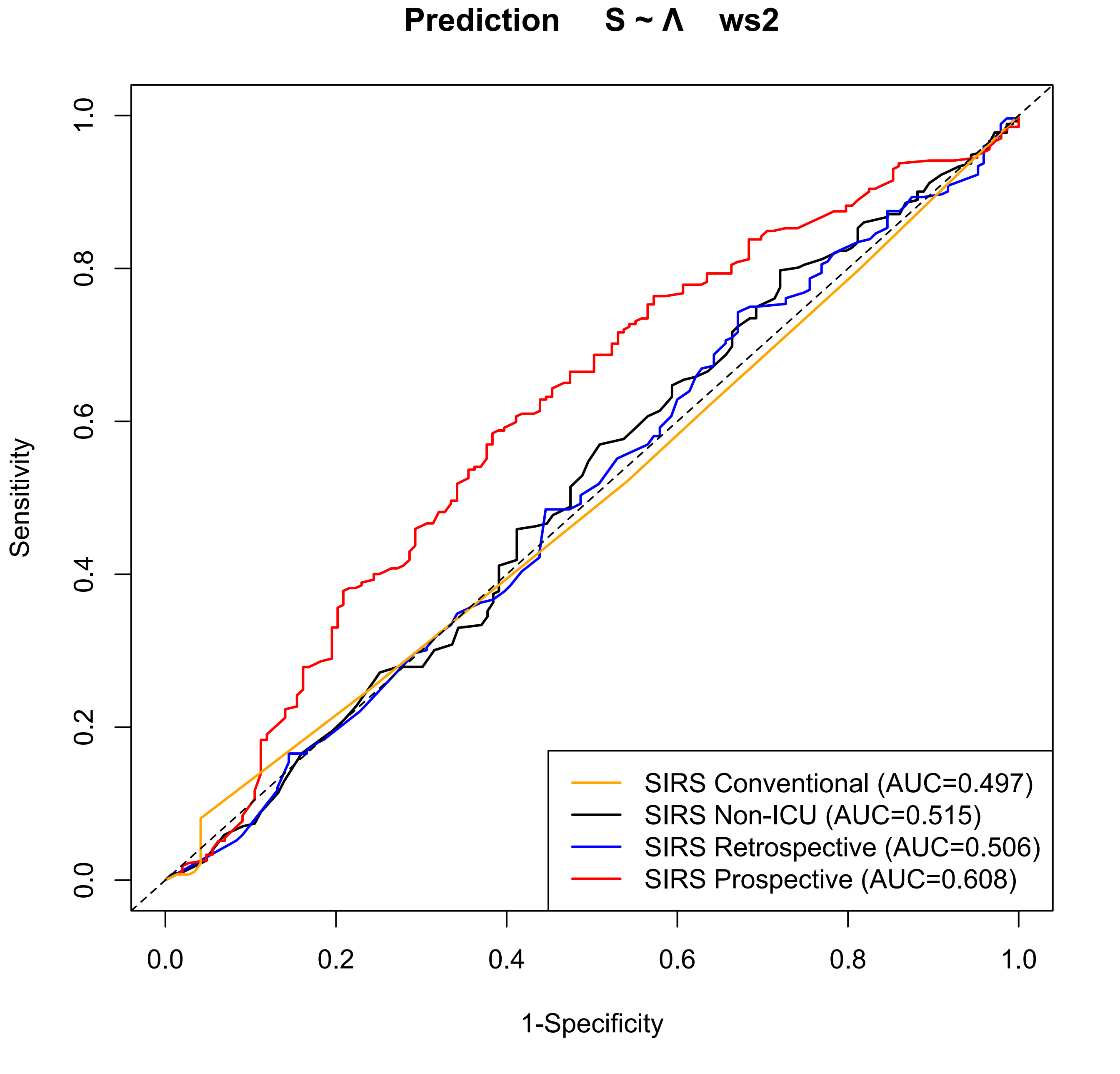
Prediction S ~ Λ ws2
Sensitivity
1-Specificity
0.0
0.2
0.4
0.6
0.8
1.0
SIRS Conventional (AUC=0.497)
SIRS Non-ICU (AUC=0.515)
SIRS Retrospective (AUC=0.506)
SIRS Prospective (AUC=0.608)

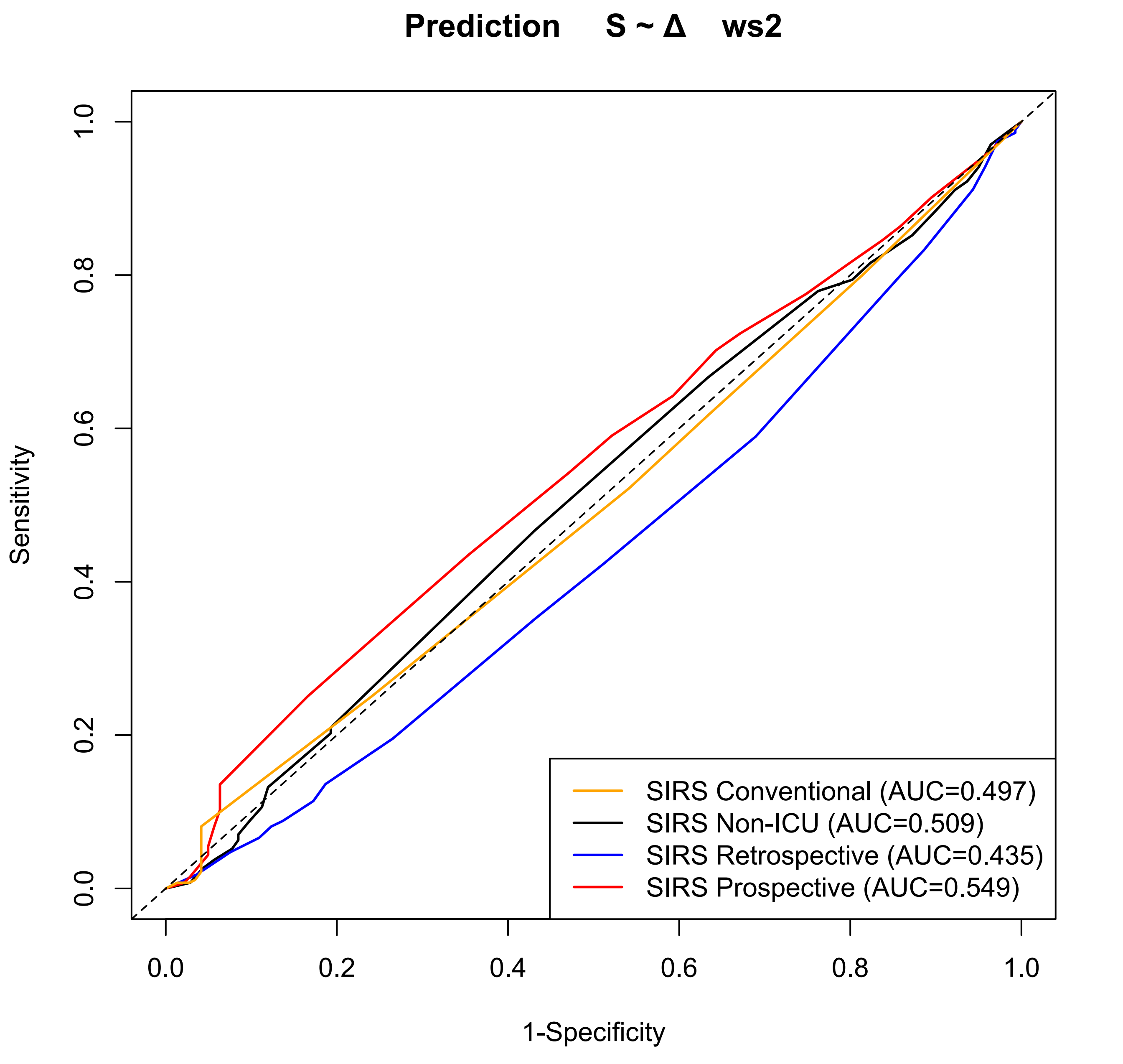

Prediction S ~ Δ ws2
Sensitivity
1-Specificity
0.0
0.2
0.4
0.6
0.8
1.0
SIRS Conventional (AUC=0.497)
SIRS Non-ICU (AUC=0.509)
SIRS Retrospective (AUC=0.435)
SIRS Prospective (AUC=0.549)

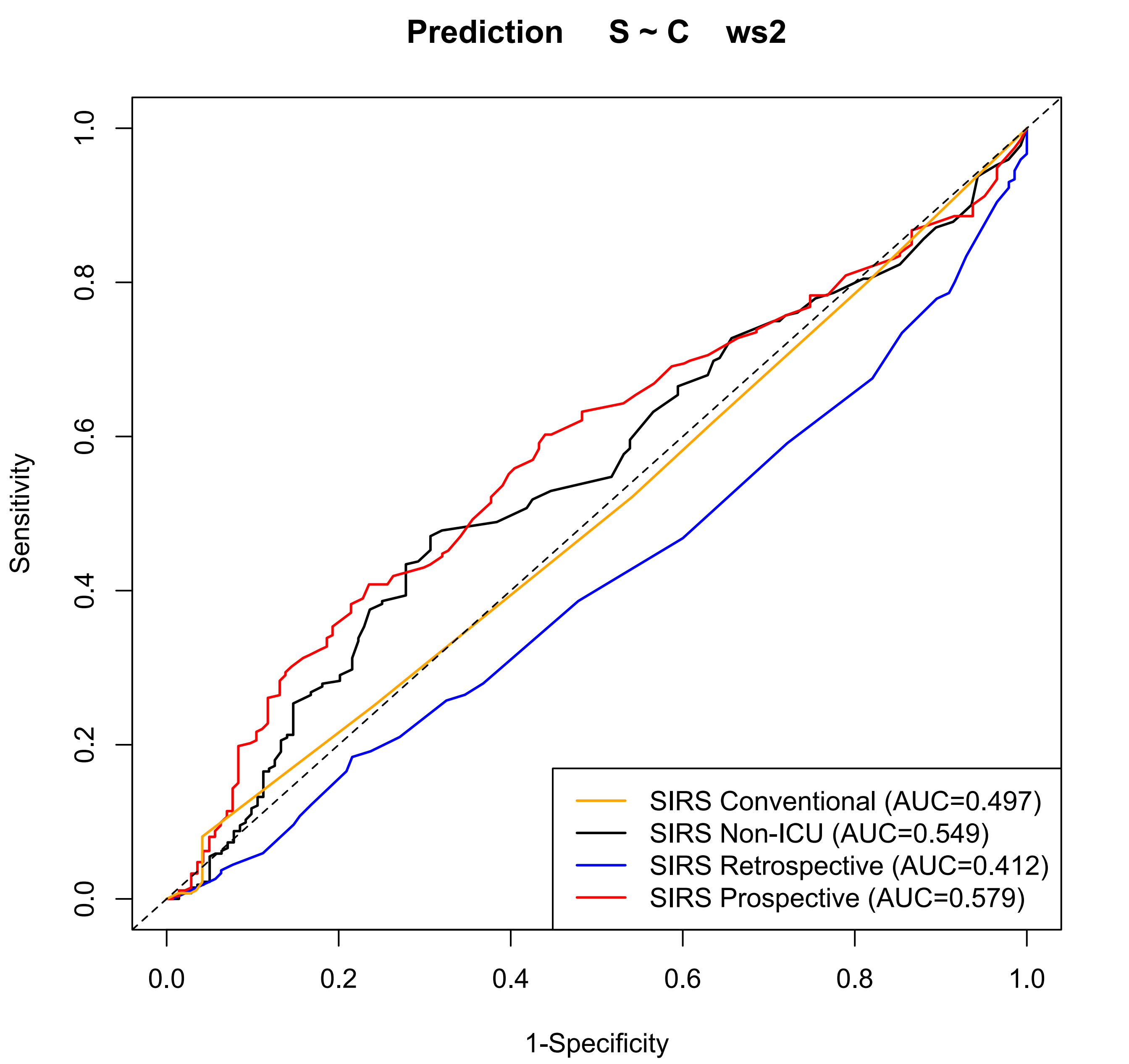

Prediction S ~ C ws2
Sensitivity
1-Specificity
0.0
0.2
0.4
0.6
0.8
1.0
SIRS Conventional (AUC=0.497)
SIRS Non-ICU (AUC=0.549)
SIRS Retrospective (AUC=0.412)
SIRS Prospective (AUC=0.579)

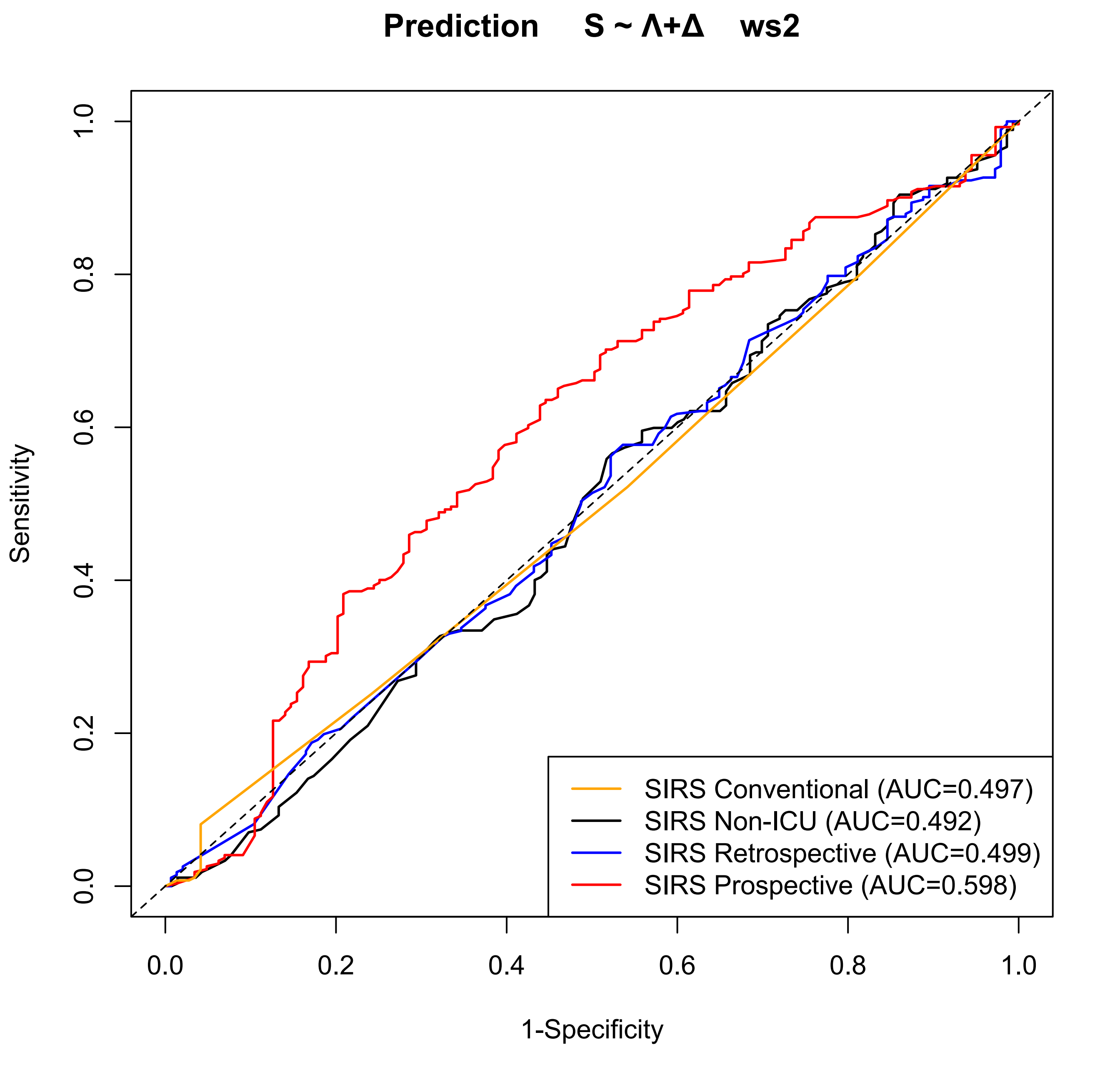

Prediction S ~ Λ+Δ ws2
Sensitivity
1-Specificity
0.0
0.2
0.4
0.6
0.8
1.0
SIRS Conventional (AUC=0.497)
SIRS Non-ICU (AUC=0.492)
SIRS Retrospective (AUC=0.499)
SIRS Prospective (AUC=0.598)

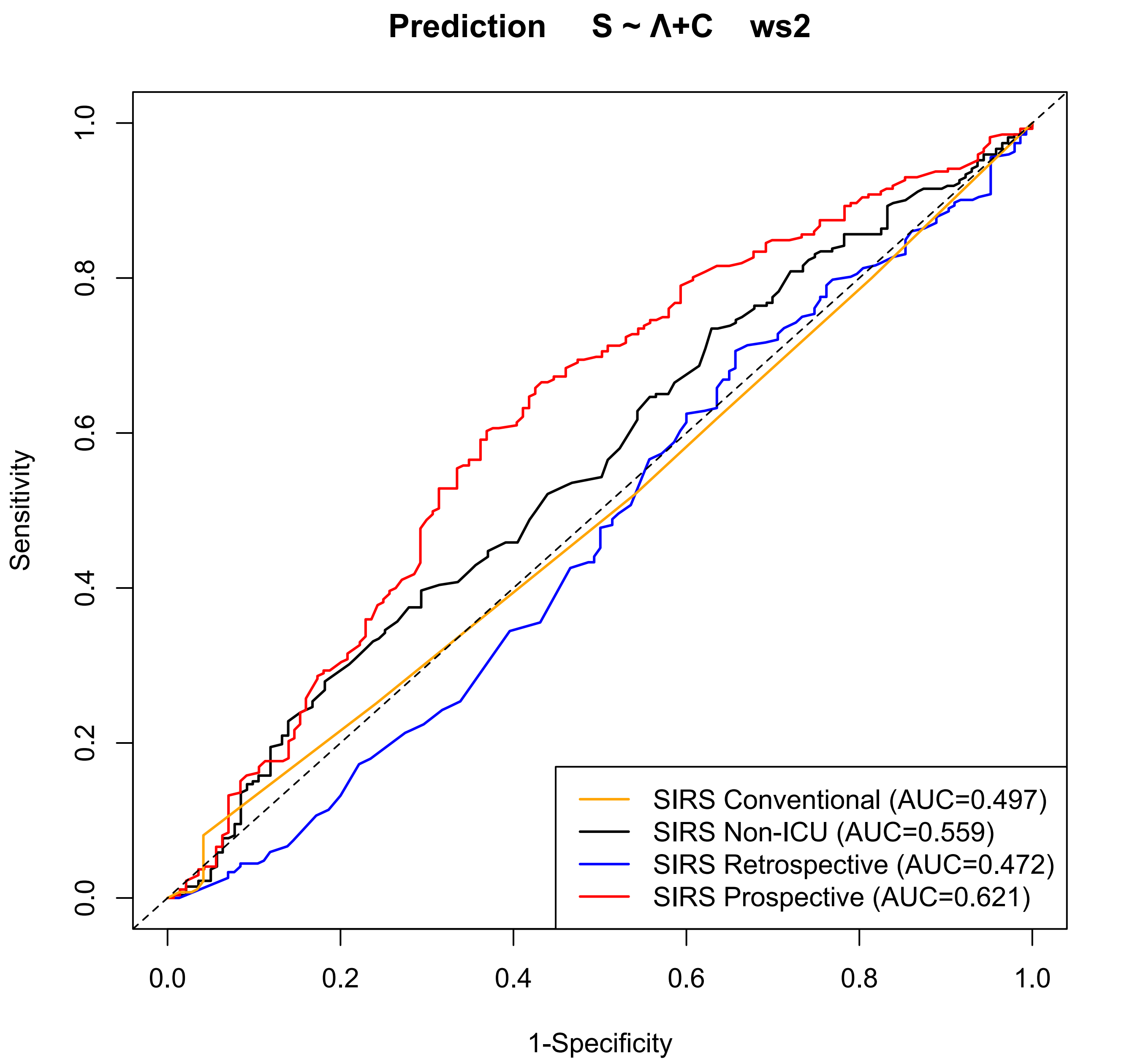
Prediction S ~ Λ+C ws2
Sensitivity
1-Specificity
0.0
0.2
0.4
0.6
0.8
1.0
SIRS Conventional (AUC=0.497)
SIRS Non-ICU (AUC=0.559)
SIRS Retrospective (AUC=0.472)
SIRS Prospective (AUC=0.621)

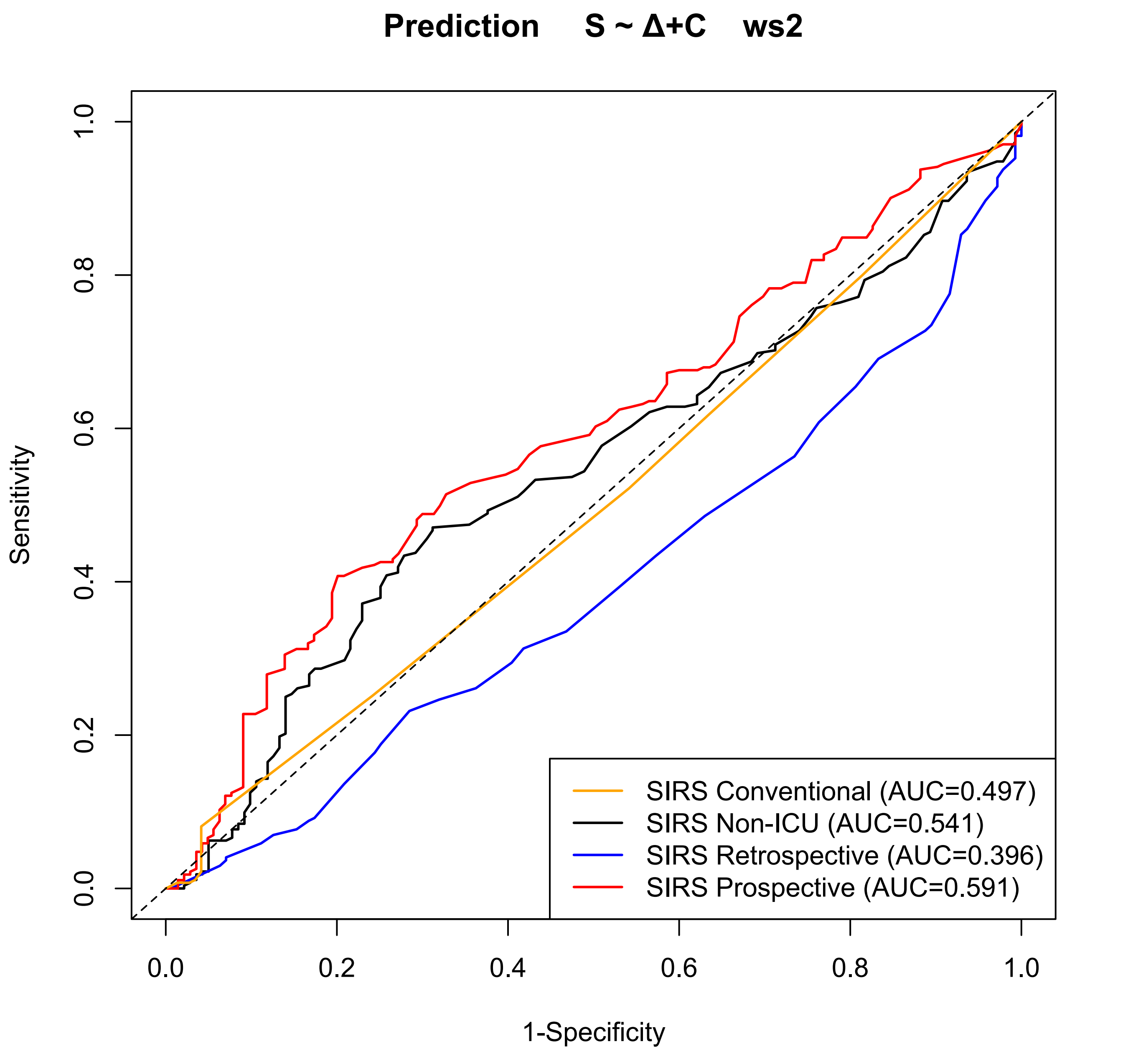

Prediction S ~ Δ+C ws2
Sensitivity
1-Specificity
0.0
0.2
0.4
0.6
0.8
1.0
SIRS Conventional (AUC=0.497)
SIRS Non-ICU (AUC=0.541)
SIRS Retrospective (AUC=0.396)
SIRS Prospective (AUC=0.591)

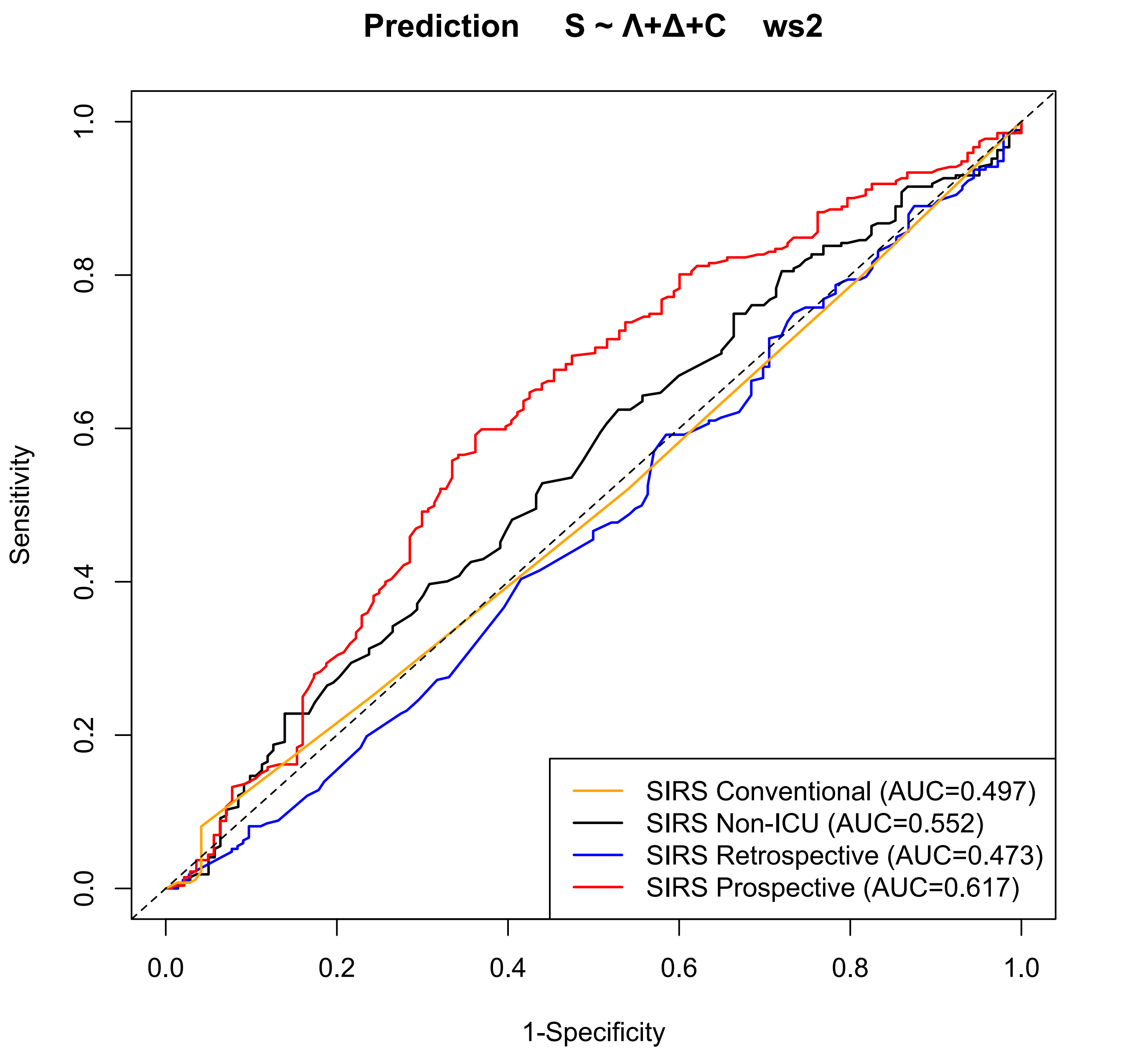
Prediction S ~ Λ+Δ+C ws2
Sensitivity
1-Specificity
0.0
0.2
0.4
0.6
0.8
1.0
SIRS Conventional (AUC=0.497)
SIRS Non-ICU (AUC=0.552)
SIRS Retrospective (AUC=0.473)
SIRS Prospective (AUC=0.617)

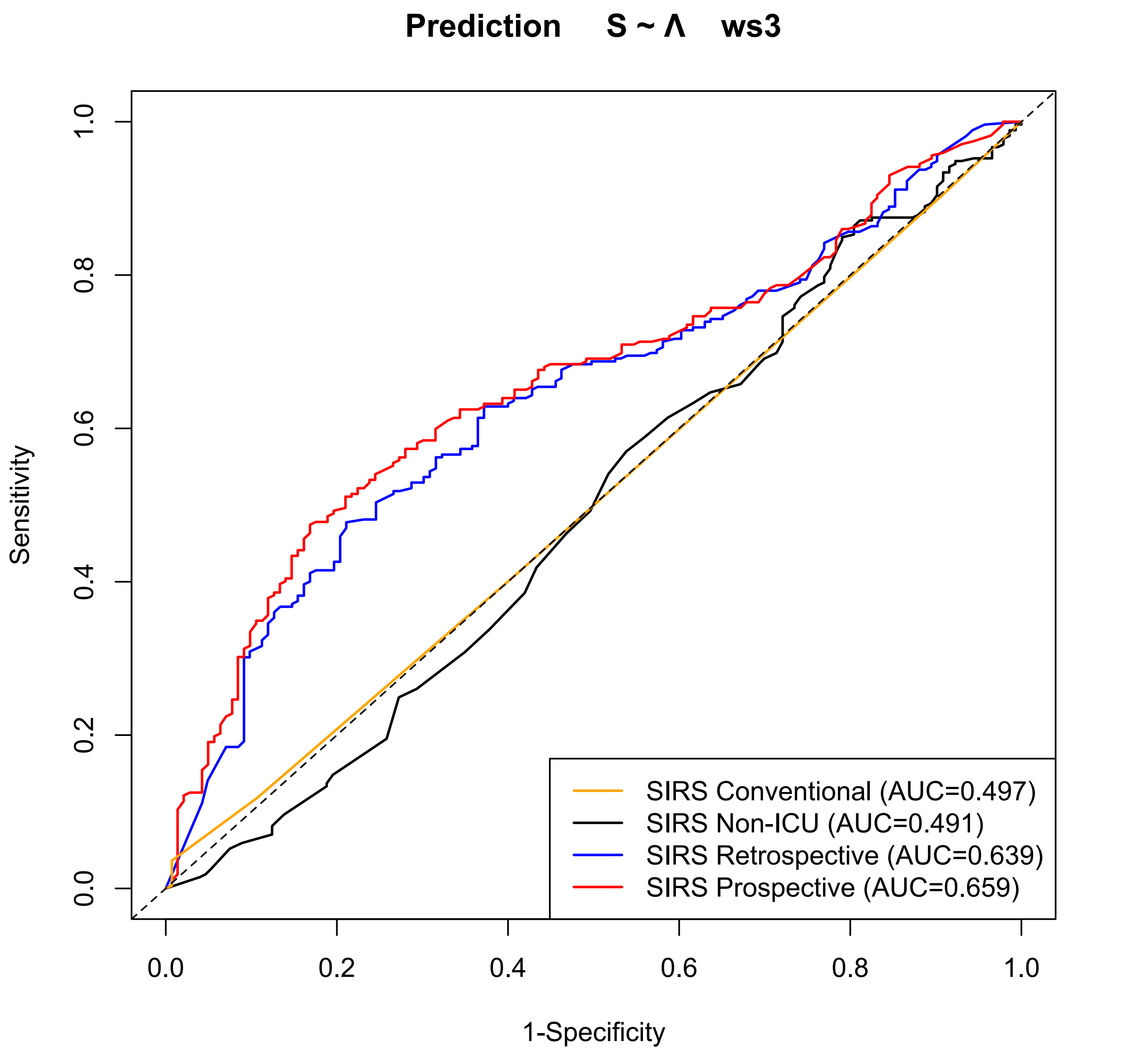

Prediction S ~ Λ ws3
Sensitivity
1-Specificity
0.0
0.2
0.4
0.6
0.8
1.0
0.0
0.2
0.4
0.6
0.8
1.0
SIRS Conventional (AUC=0.497)
SIRS Non-ICU (AUC=0.491)
SIRS Retrospective (AUC=0.639)
SIRS Prospective (AUC=0.659)

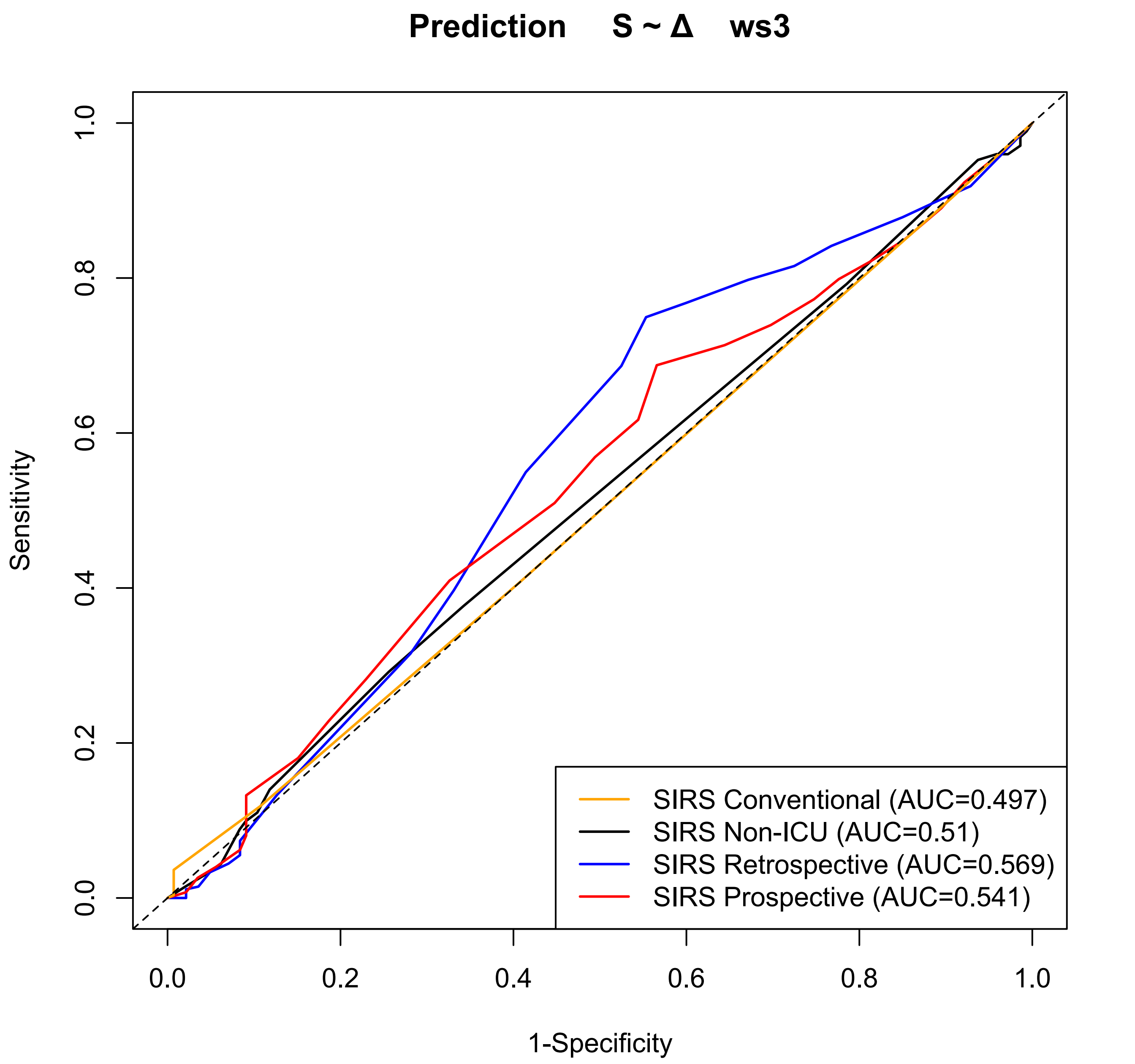
Prediction S ~ Δ ws3
Sensitivity
1-Specificity
0.0
0.2
0.4
0.6
0.8
1.0
SIRS Conventional (AUC=0.497)
SIRS Non-ICU (AUC=0.51)
SIRS Retrospective (AUC=0.569)
SIRS Prospective (AUC=0.541)

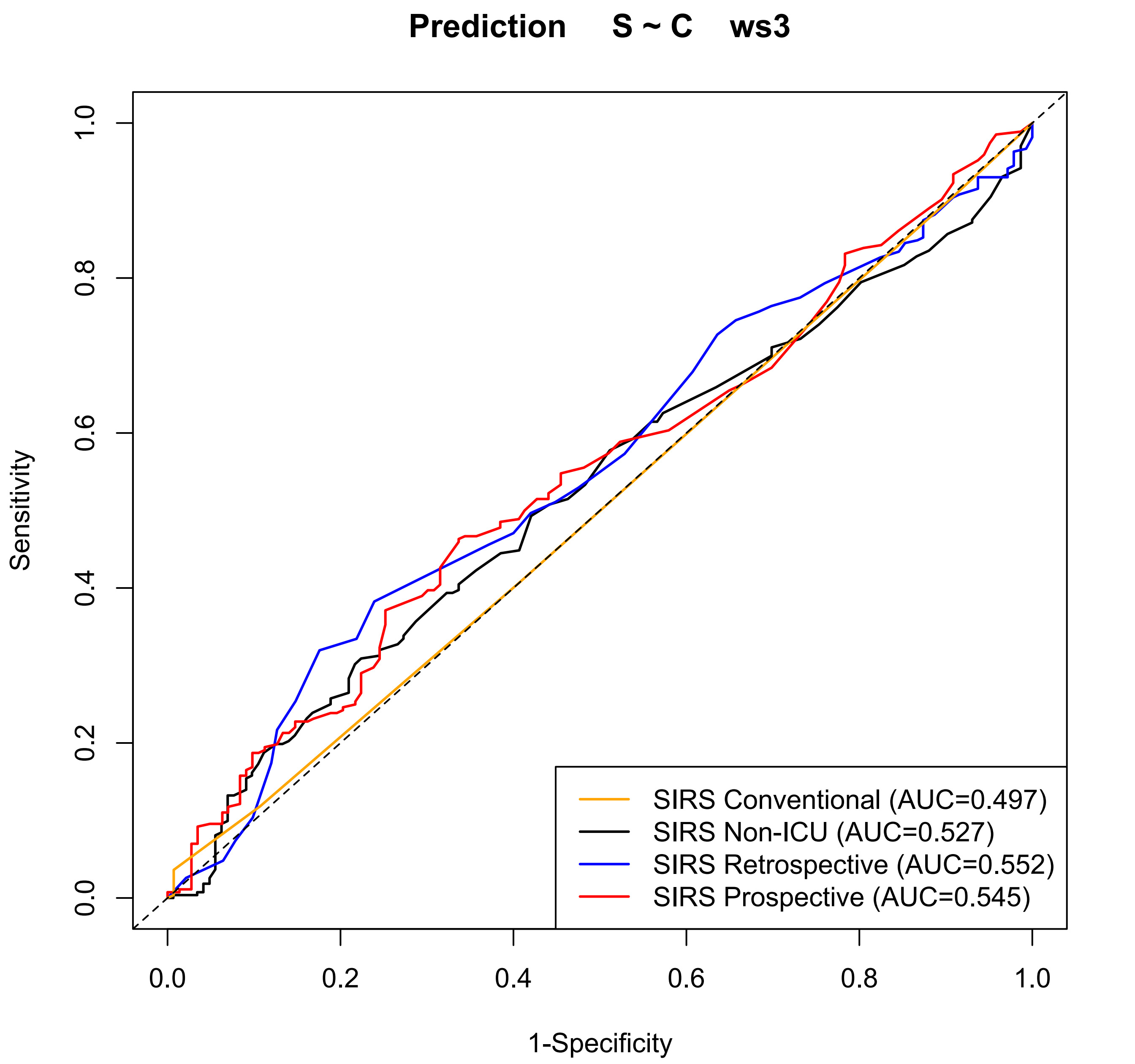
Prediction S ~ C ws3
Sensitivity
1-Specificity
0.0
0.2
0.4
0.6
0.8
1.0
0.0
0.2
0.4
0.6
0.8
1.0
SIRS Conventional (AUC=0.497)
SIRS Non-ICU (AUC=0.527)
SIRS Retrospective (AUC=0.552)
SIRS Prospective (AUC=0.545)

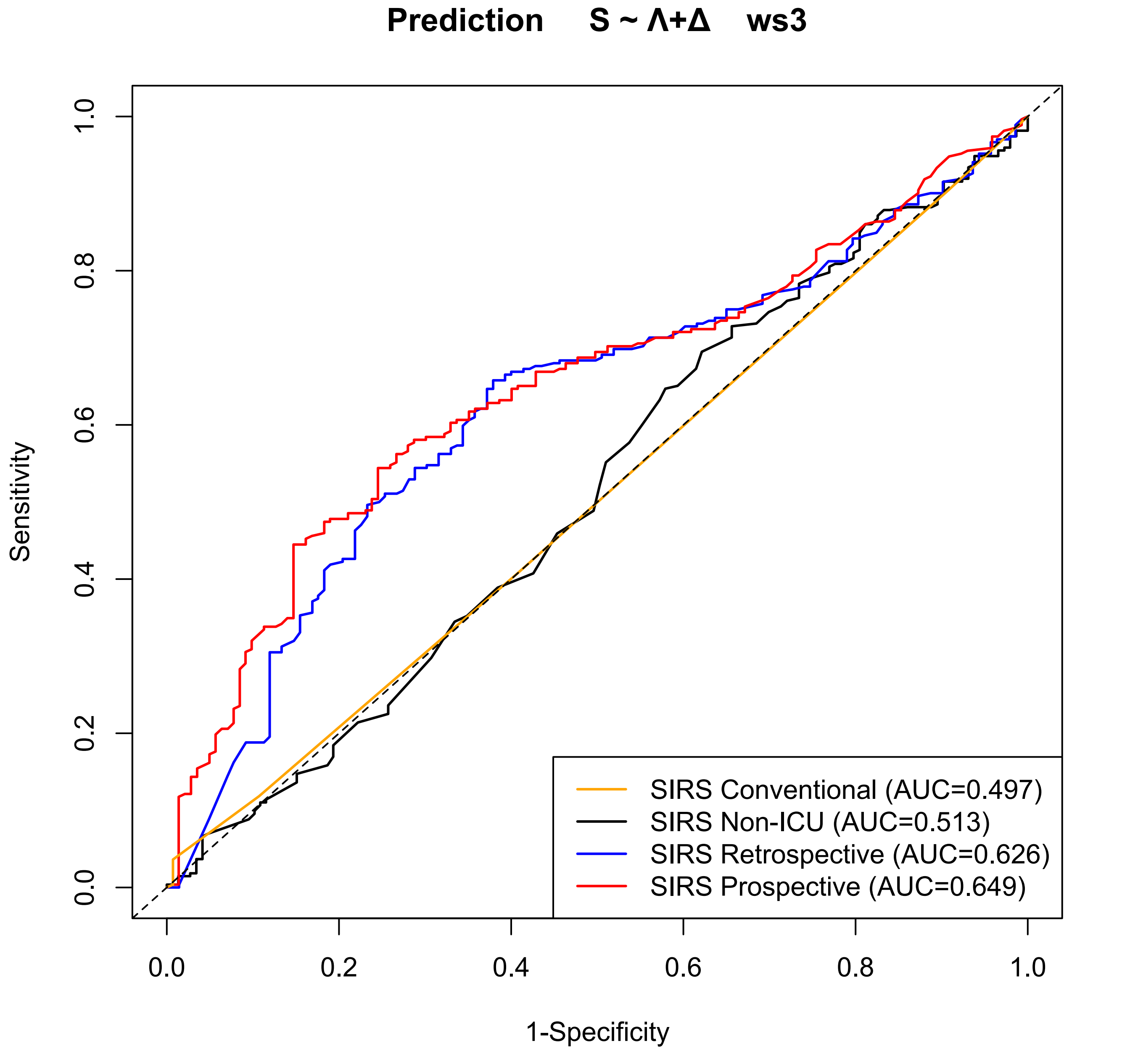

Prediction S ~ Λ+Δ ws3
Sensitivity
1-Specificity
0.0
0.2
0.4
0.6
0.8
1.0
SIRS Conventional (AUC=0.497)
SIRS Non-ICU (AUC=0.513)
SIRS Retrospective (AUC=0.626)
SIRS Prospective (AUC=0.649)

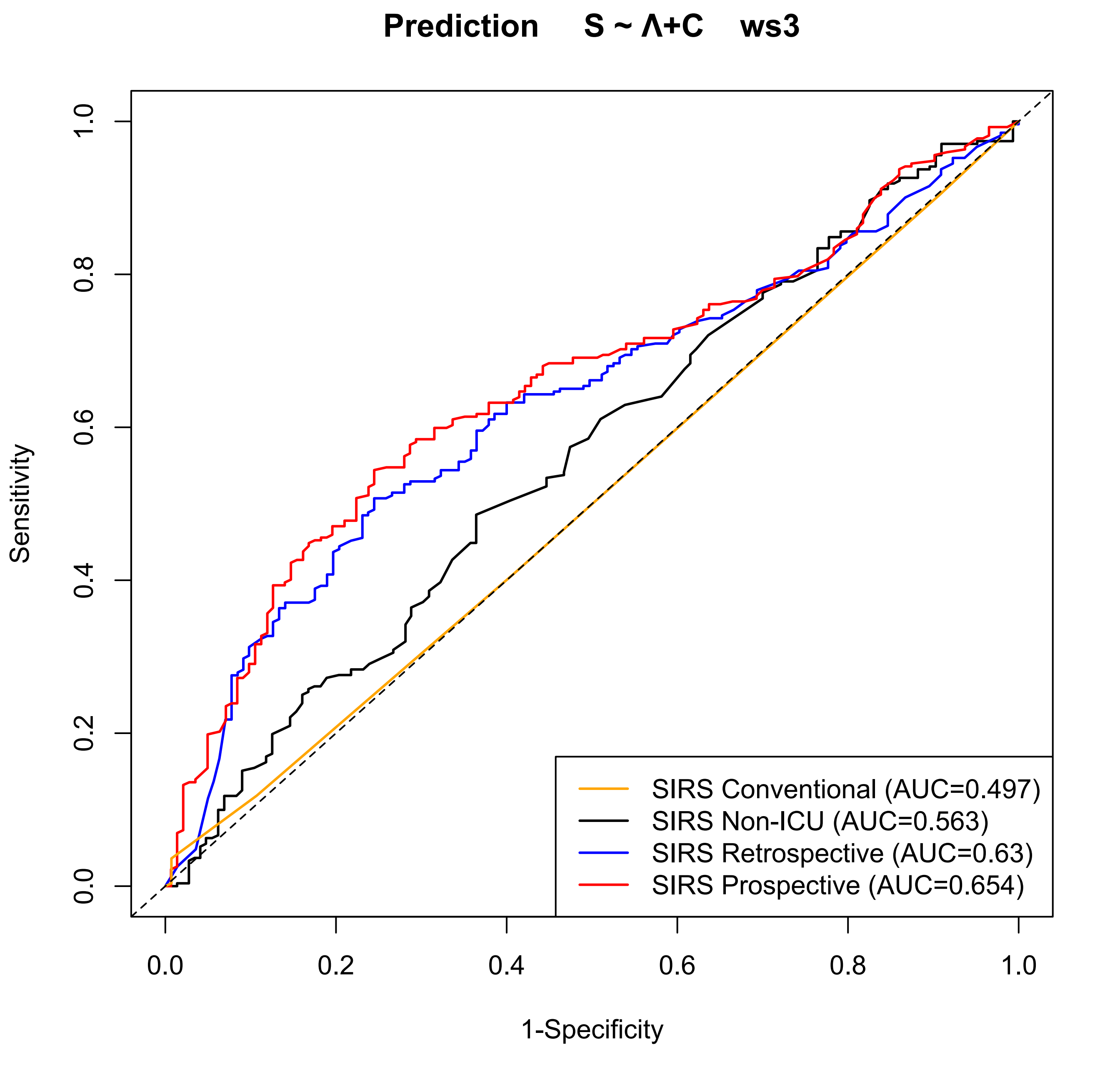
Prediction S ~ Λ+C ws3
Sensitivity
1-Specificity
0.0
0.2
0.4
0.6
0.8
1.0
SIRS Conventional (AUC=0.497)
SIRS Non-ICU (AUC=0.563)
SIRS Retrospective (AUC=0.63)
SIRS Prospective (AUC=0.654)

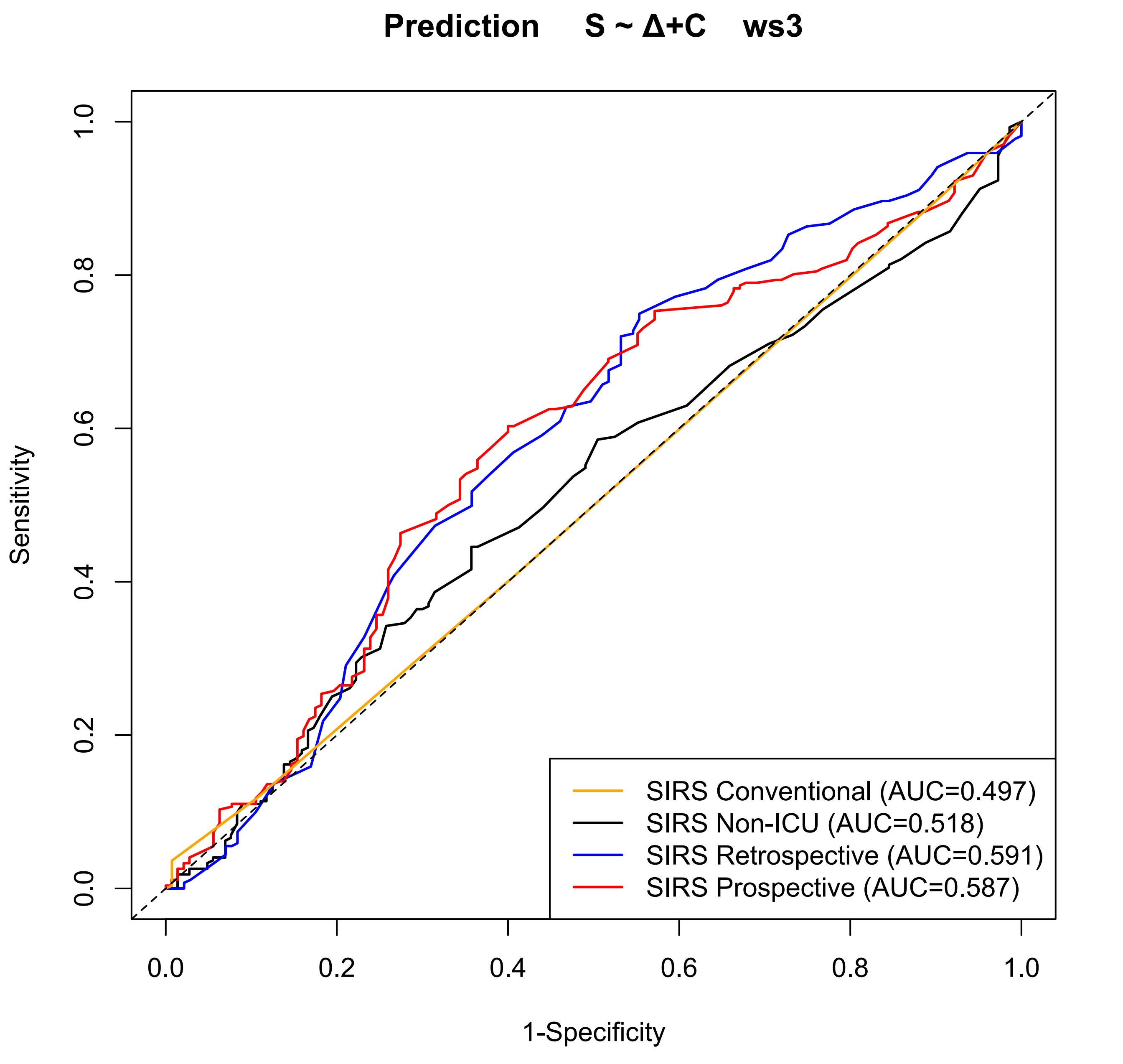

Prediction S ~ Δ+C ws3
Sensitivity
1-Specificity
0.0
0.2
0.4
0.6
0.8
1.0
SIRS Conventional (AUC=0.497)
SIRS Non-ICU (AUC=0.518)
SIRS Retrospective (AUC=0.591)
SIRS Prospective (AUC=0.587)

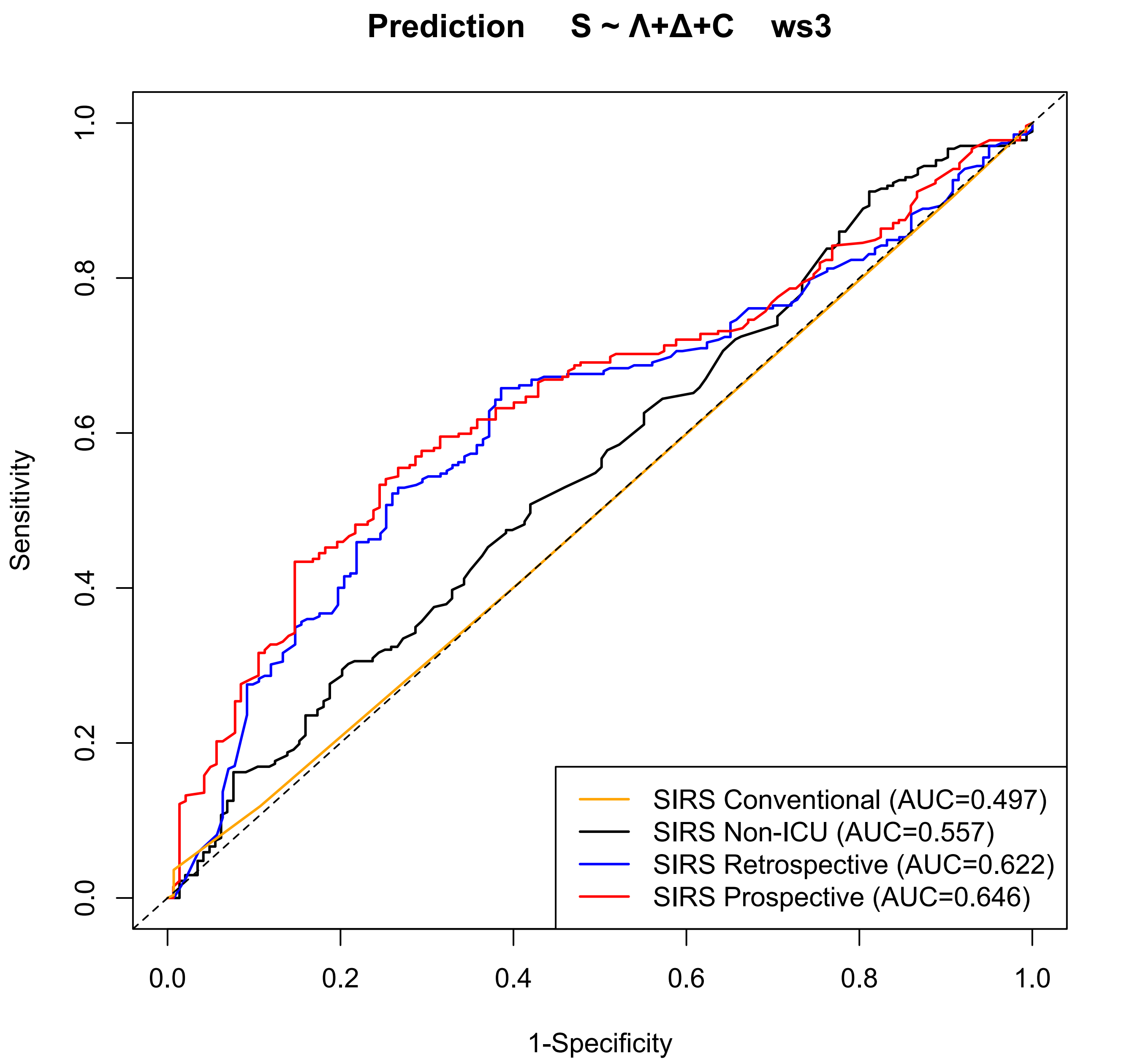

Prediction S ~ Λ+Δ+C ws3
Sensitivity
1-Specificity
0.0
0.2
0.4
0.6
0.8
1.0
SIRS Conventional (AUC=0.497)
SIRS Non-ICU (AUC=0.557)
SIRS Retrospective (AUC=0.622)
SIRS Prospective (AUC=0.646)

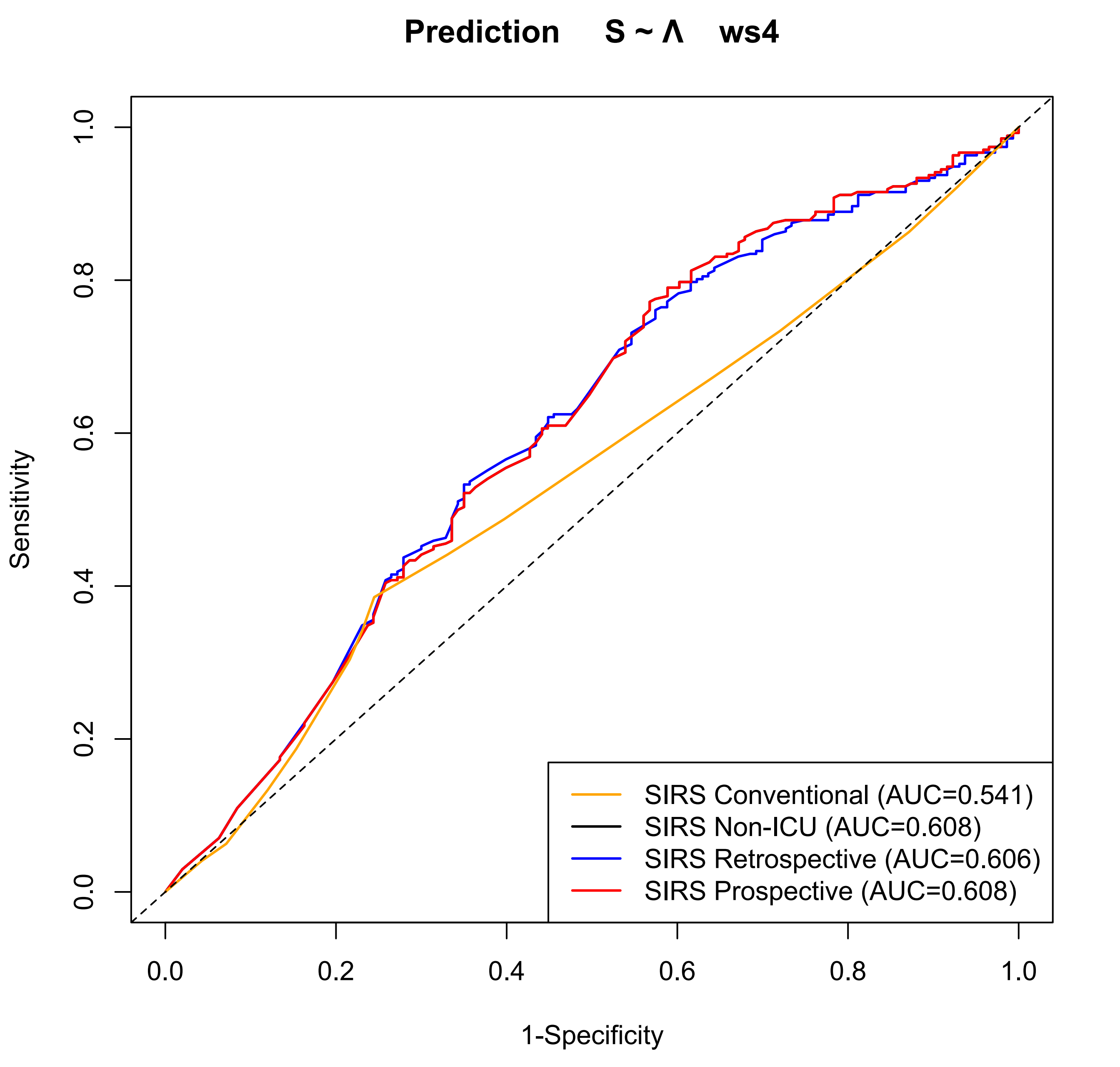

Prediction S ~ Λ ws4
Sensitivity
1-Specificity
0.0
0.2
0.4
0.6
0.8
1.0
0.0
0.2
0.4
0.6
0.8
1.0
SIRS Conventional (AUC=0.541)
SIRS Non-ICU (AUC=0.608)
SIRS Retrospective (AUC=0.606)
SIRS Prospective (AUC=0.608)

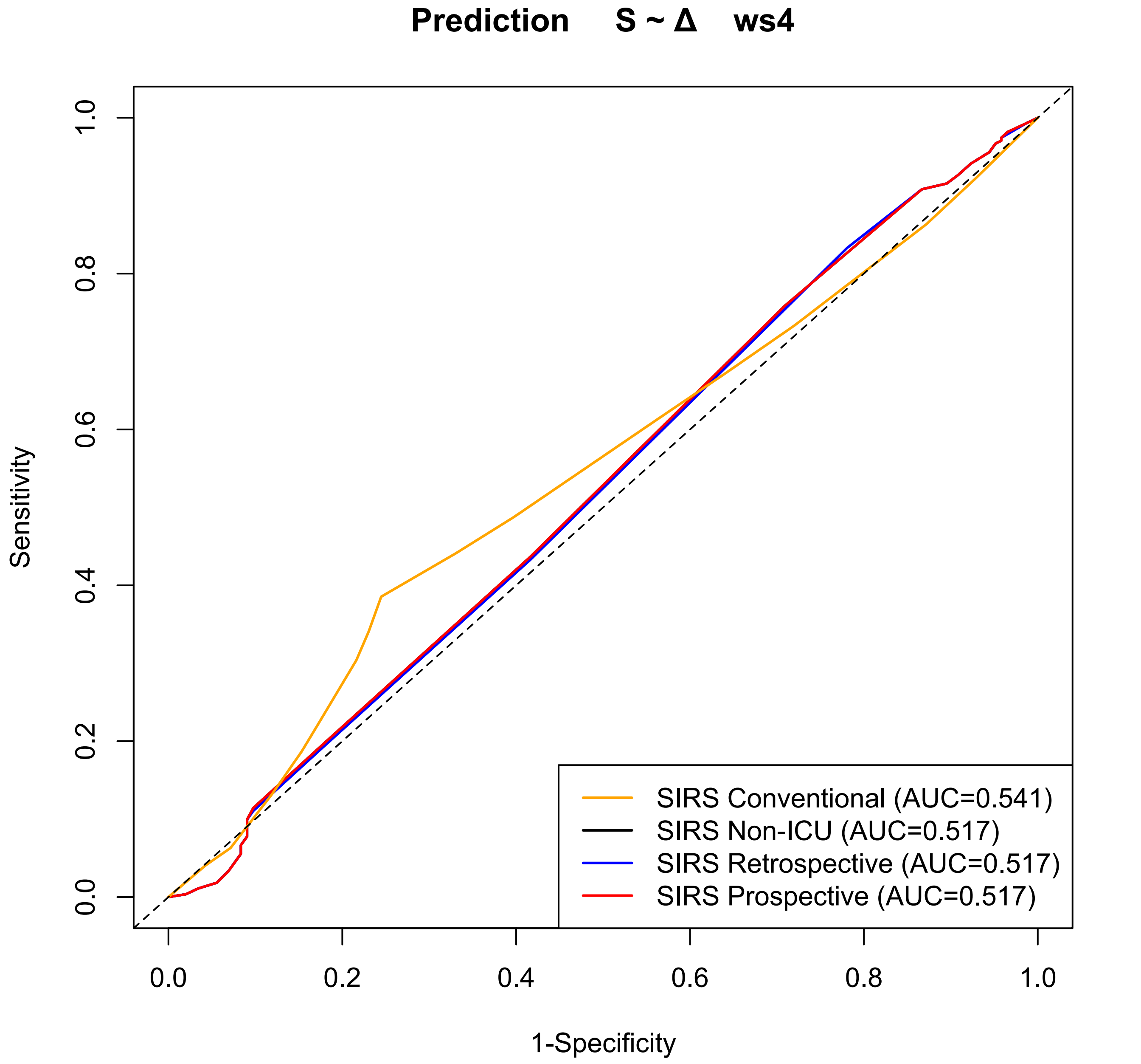

Prediction S ~ Δ ws4
Sensitivity
1-Specificity
0.0
0.2
0.4
0.6
0.8
1.0
0.0
0.2
0.4
0.6
0.8
1.0
SIRS Conventional (AUC=0.541)
SIRS Non-ICU (AUC=0.517)
SIRS Retrospective (AUC=0.517)
SIRS Prospective (AUC=0.517)

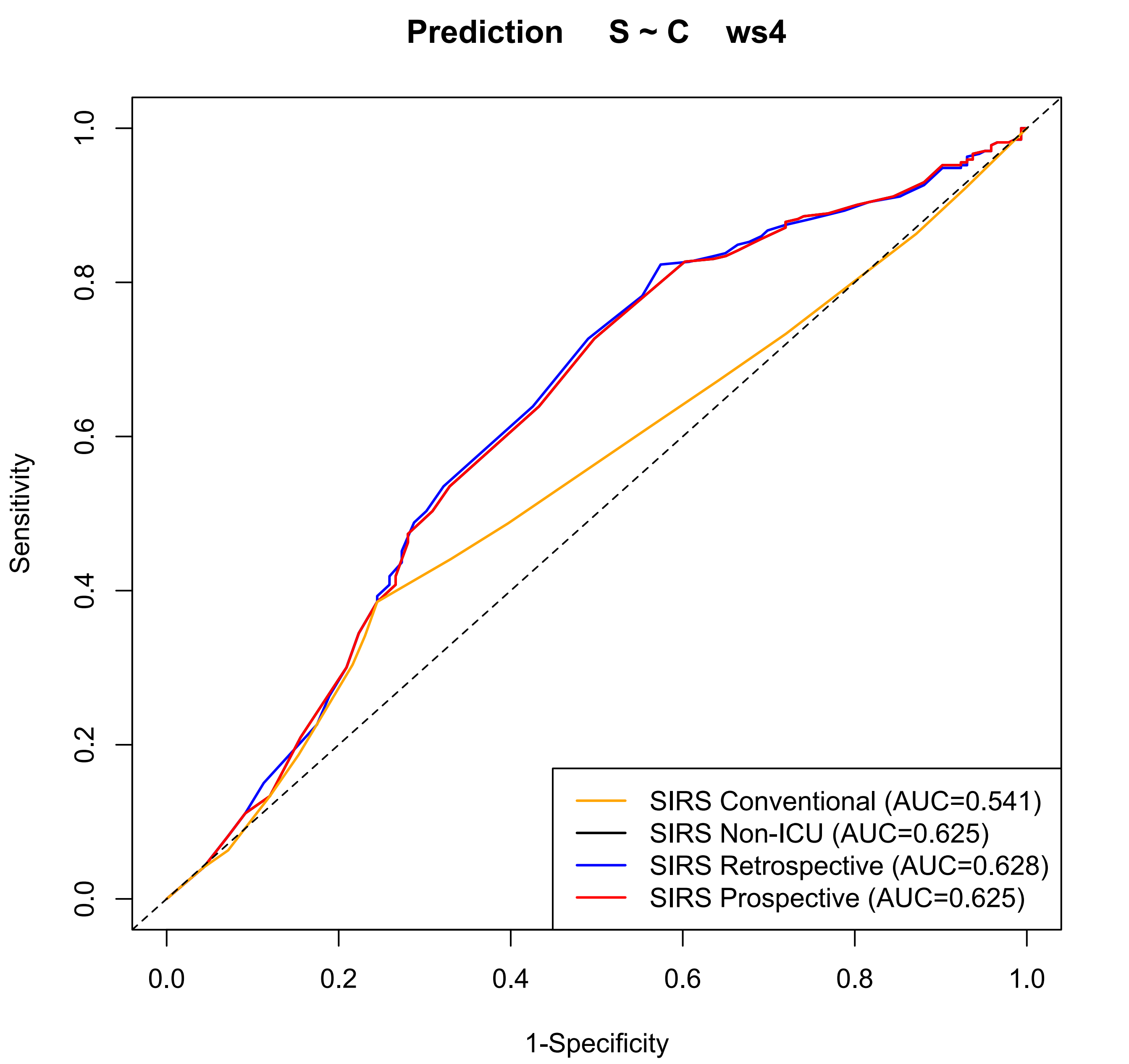

Prediction S ~ C ws4
Sensitivity
1-Specificity
0.0
0.2
0.4
0.6
0.8
1.0
SIRS Conventional (AUC=0.541)
SIRS Non-ICU (AUC=0.625)
SIRS Retrospective (AUC=0.628)
SIRS Prospective (AUC=0.625)

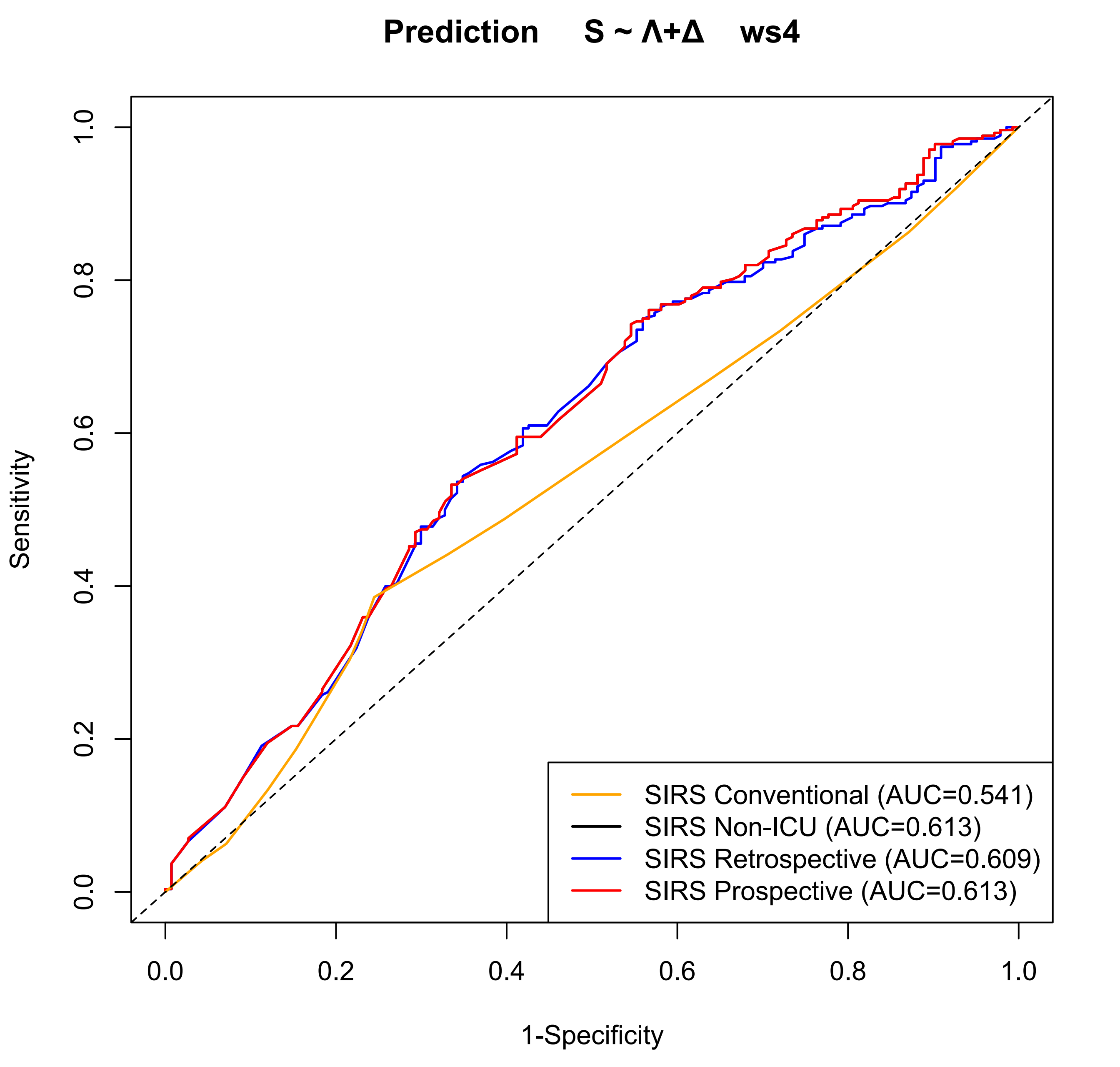
Prediction S ~ Λ+Δ ws4
Sensitivity
1-Specificity
0.0
0.2
0.4
0.6
0.8
1.0
0.0
0.2
0.4
0.6
0.8
1.0
SIRS Conventional (AUC=0.541)
SIRS Non-ICU (AUC=0.613)
SIRS Retrospective (AUC=0.609)
SIRS Prospective (AUC=0.613)

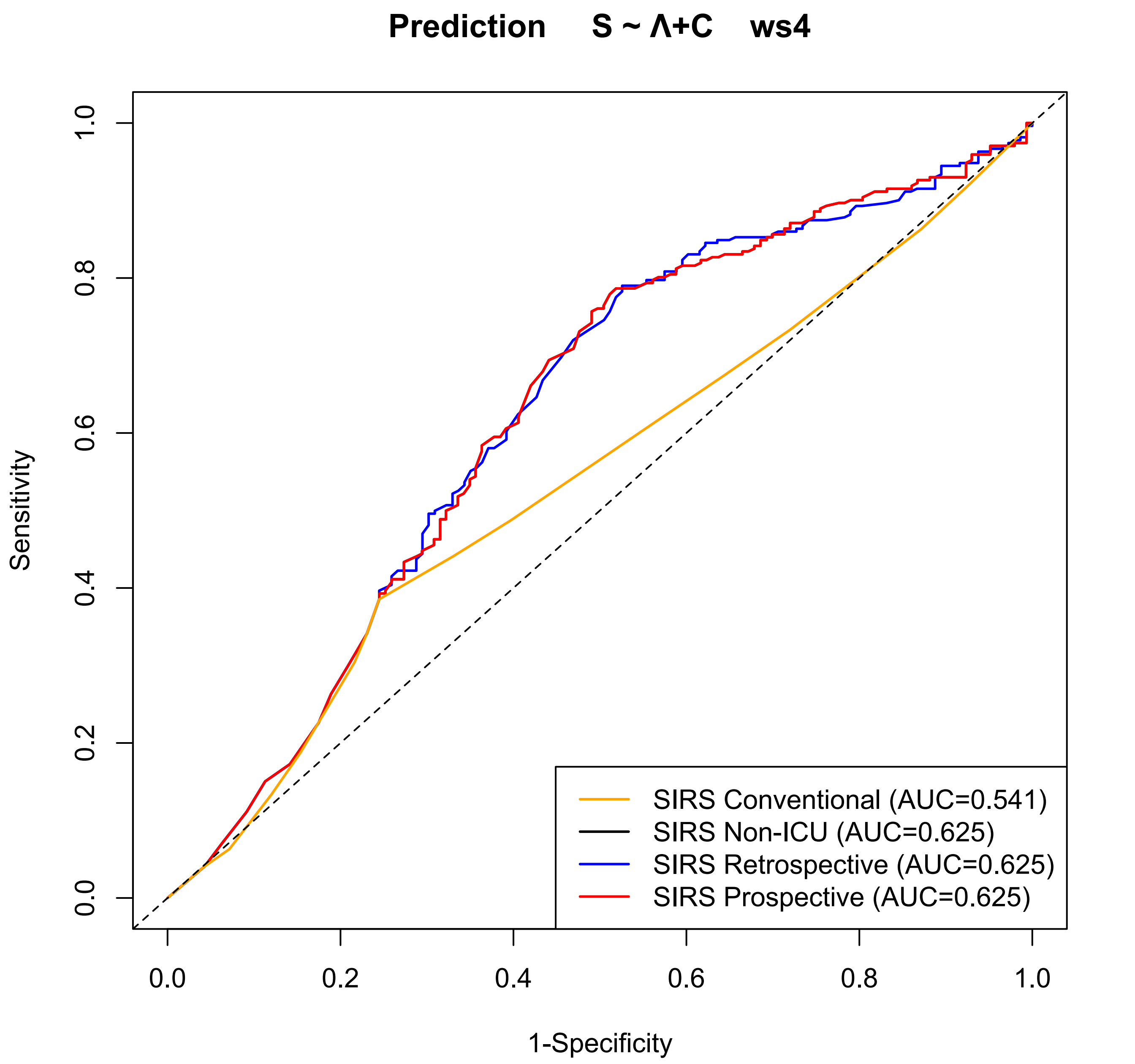
Prediction S ~ Λ+C ws4
Sensitivity
1-Specificity
0.0
0.2
0.4
0.6
0.8
1.0
0.0
0.2
0.4
0.6
0.8
1.0
SIRS Conventional (AUC=0.541)
SIRS Non-ICU (AUC=0.625)
SIRS Retrospective (AUC=0.625)
SIRS Prospective (AUC=0.625)

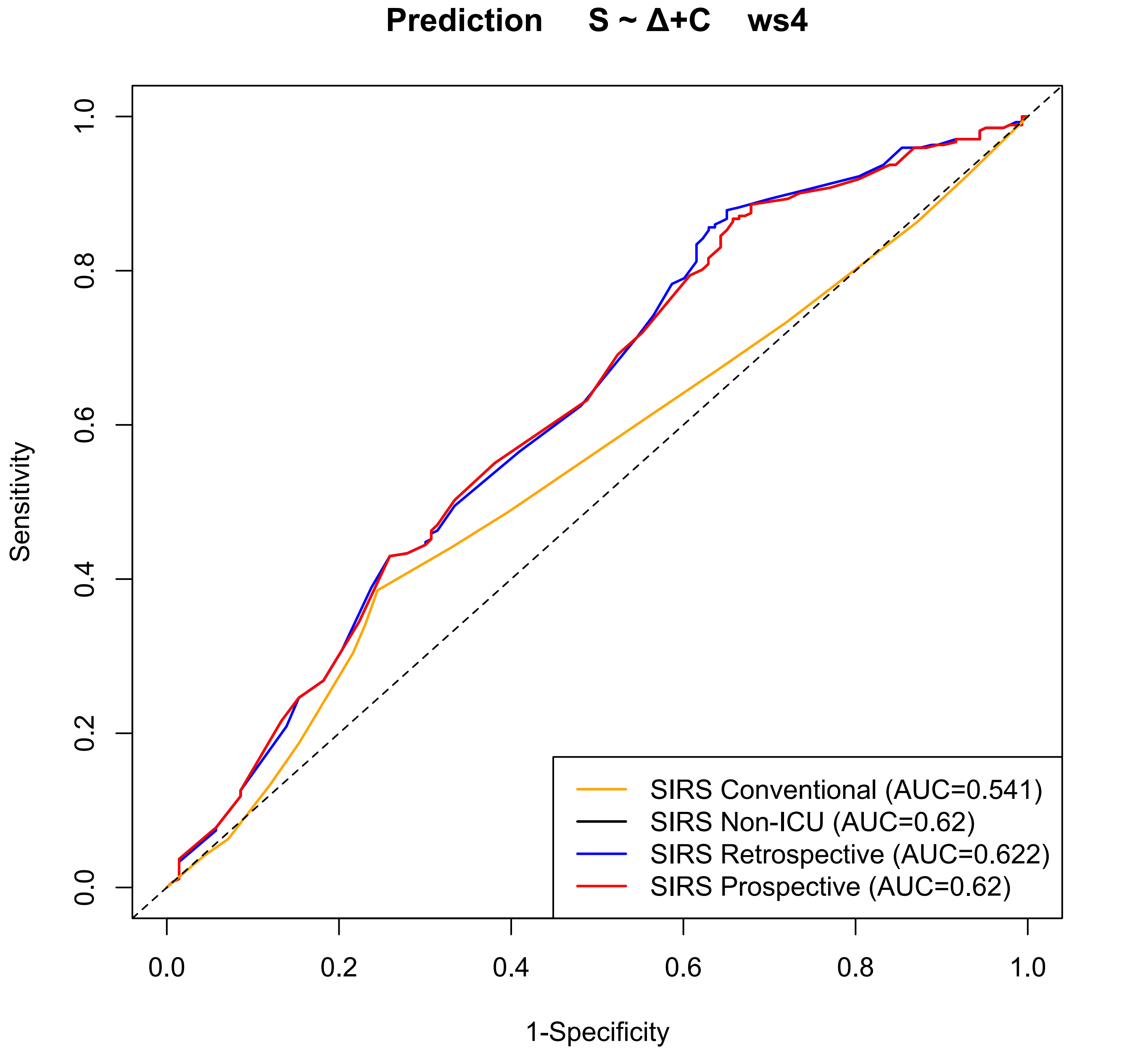
Prediction S ~ Δ+C ws4
Sensitivity
1-Specificity
0.0
0.2
0.4
0.6
0.8
1.0
SIRS Conventional (AUC=0.541)
SIRS Non-ICU (AUC=0.62)
SIRS Retrospective (AUC=0.622)
SIRS Prospective (AUC=0.62)

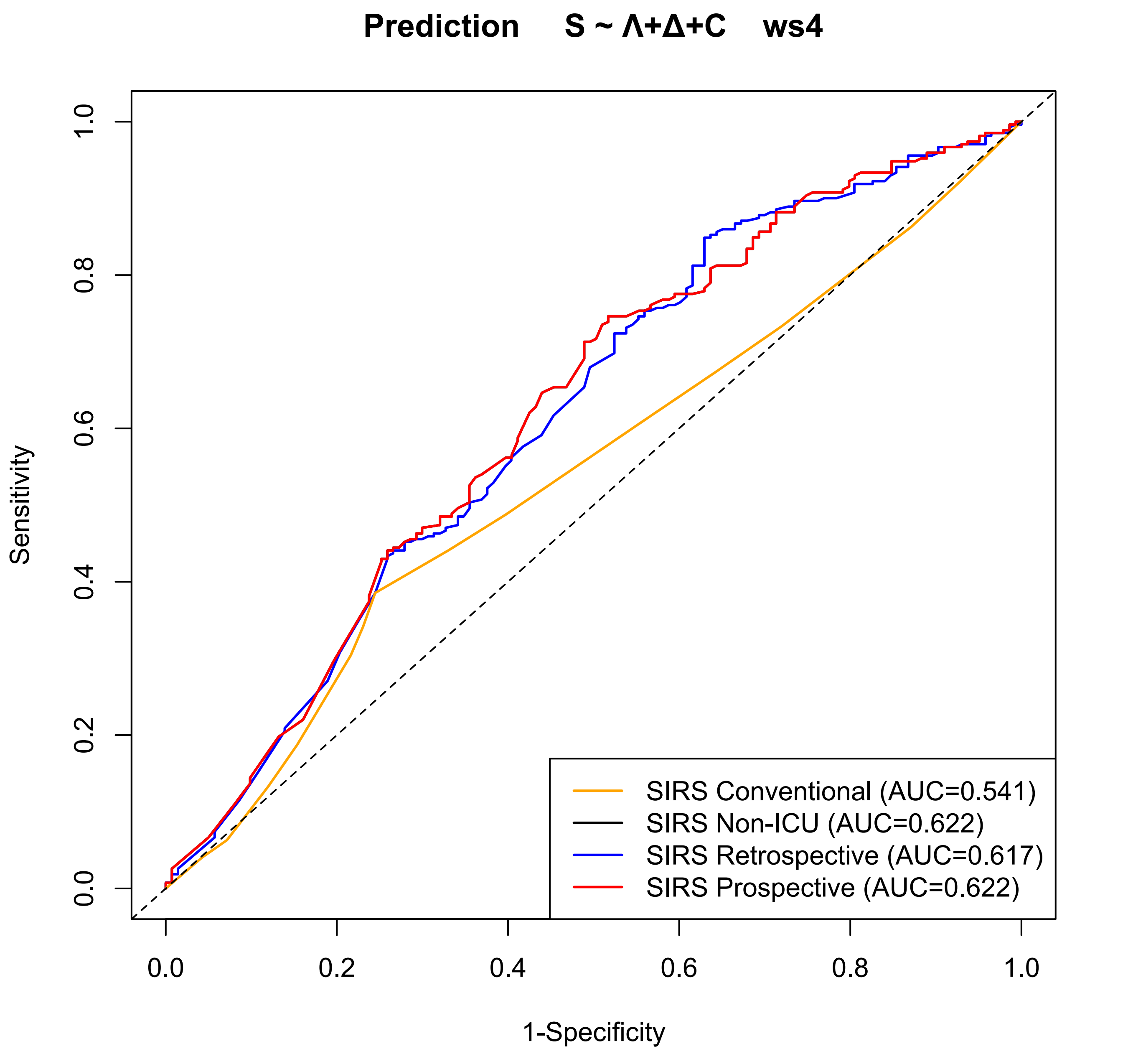

Prediction S ~ Λ+Δ+C ws4
Sensitivity
1-Specificity
0.0
0.2
0.4
0.6
0.8
1.0
SIRS Conventional (AUC=0.541)
SIRS Non-ICU (AUC=0.622)
SIRS Retrospective (AUC=0.617)
SIRS Prospective (AUC=0.622)

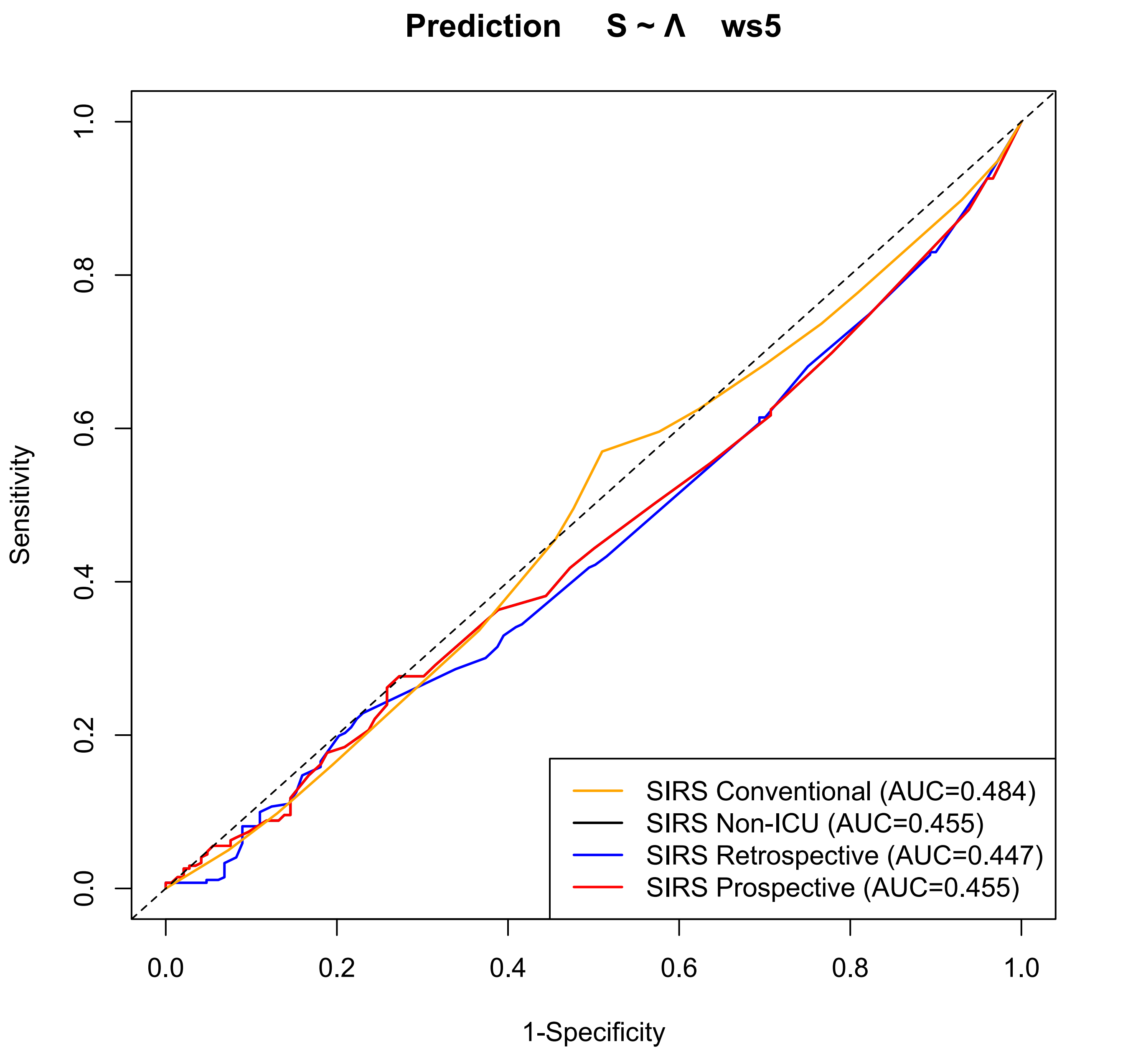
Prediction S ~ Λ ws5
Sensitivity
1-Specificity
0.0
0.2
0.4
0.6
0.8
1.0
0.0
0.2
0.4
0.6
0.8
1.0
SIRS Conventional (AUC=0.484)
SIRS Non-ICU (AUC=0.455)
SIRS Retrospective (AUC=0.447)
SIRS Prospective (AUC=0.455)

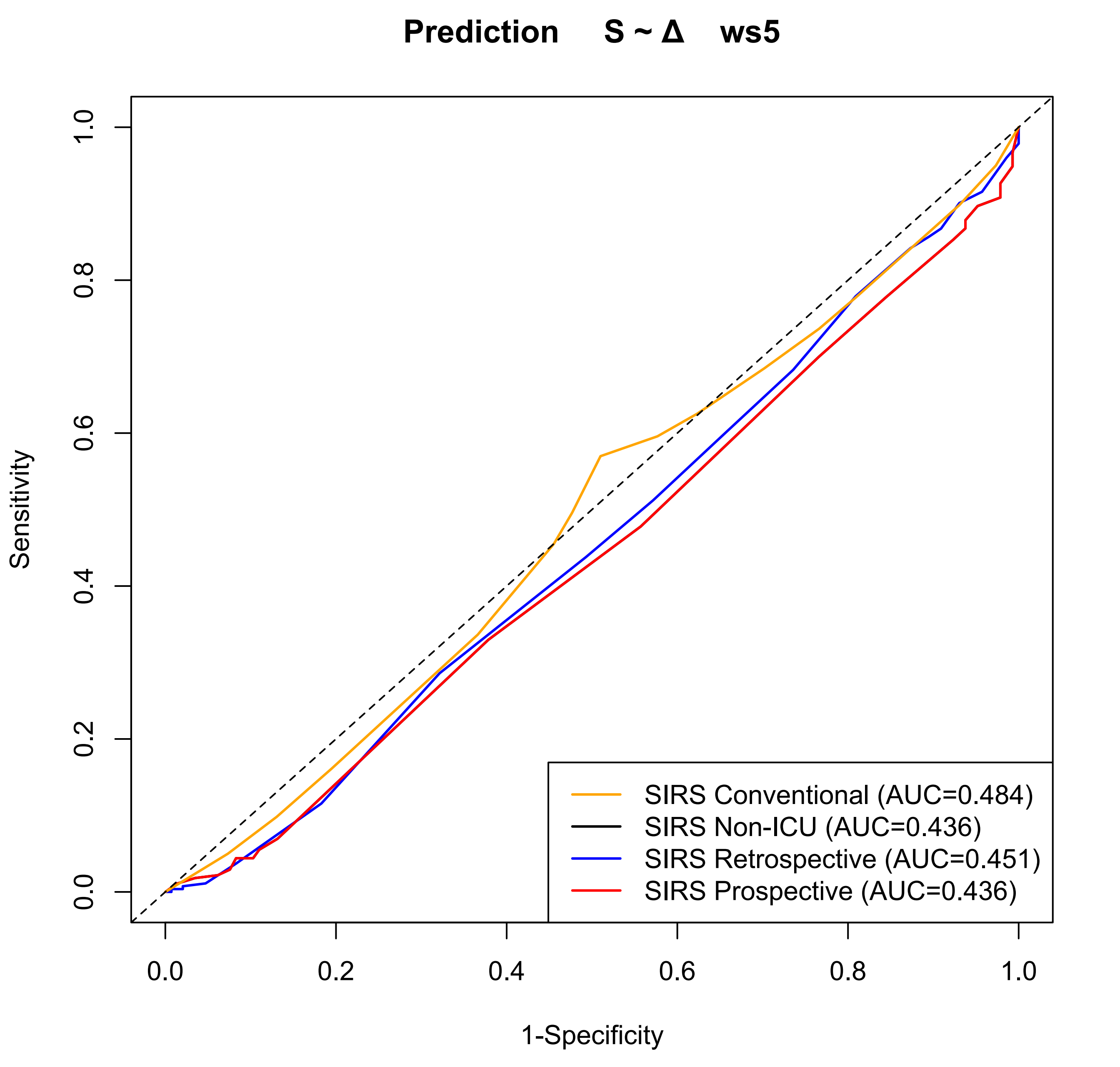

Prediction S ~ Δ ws5
Sensitivity
1-Specificity
0.0
0.2
0.4
0.6
0.8
1.0
0.0
0.2
0.4
0.6
0.8
1.0
SIRS Conventional (AUC=0.484)
SIRS Non-ICU (AUC=0.436)
SIRS Retrospective (AUC=0.451)
SIRS Prospective (AUC=0.436)

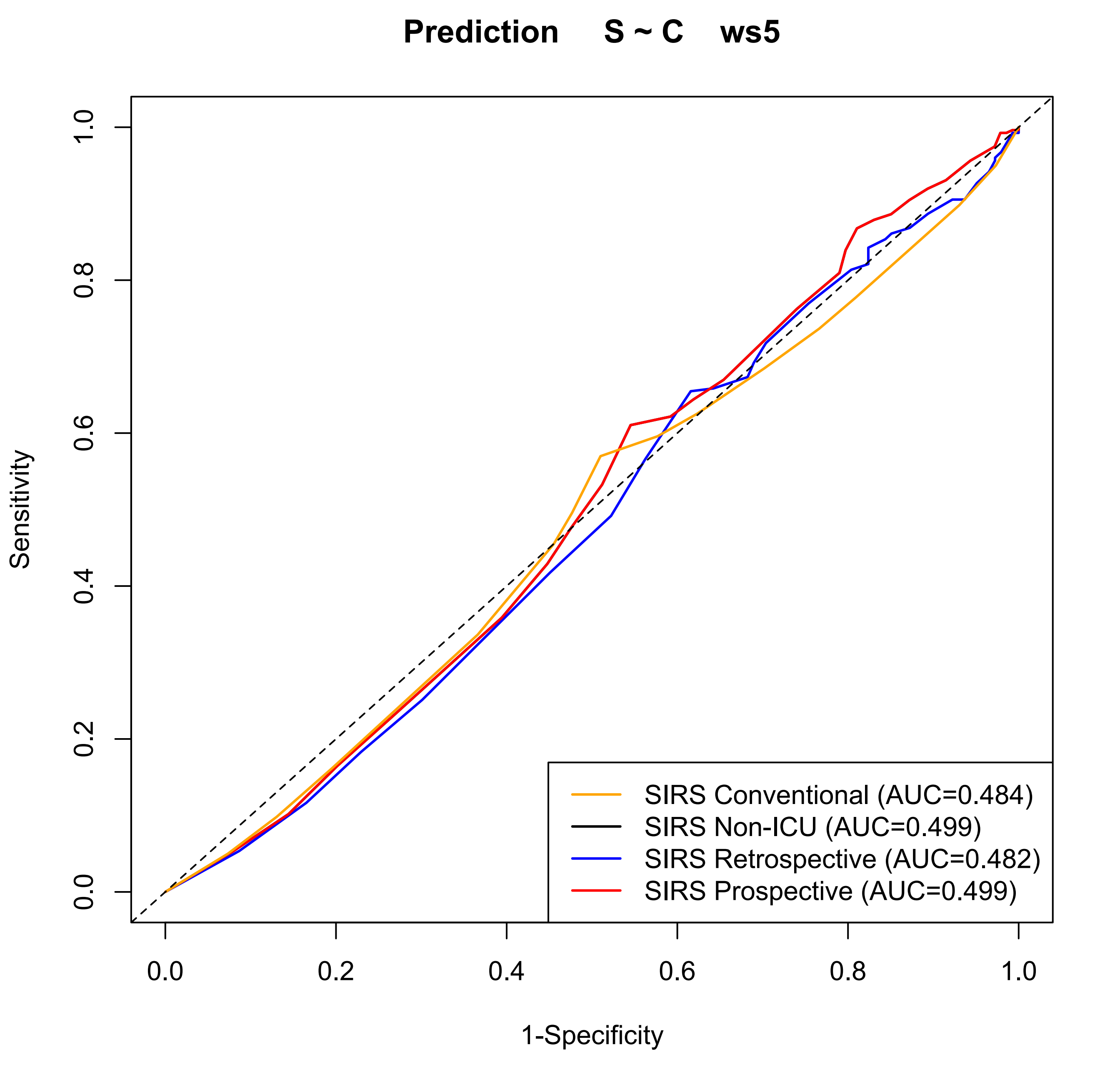

Prediction S ~ C ws5
Sensitivity
1-Specificity
0.0
0.2
0.4
0.6
0.8
1.0
SIRS Conventional (AUC=0.484)
SIRS Non-ICU (AUC=0.499)
SIRS Retrospective (AUC=0.482)
SIRS Prospective (AUC=0.499)

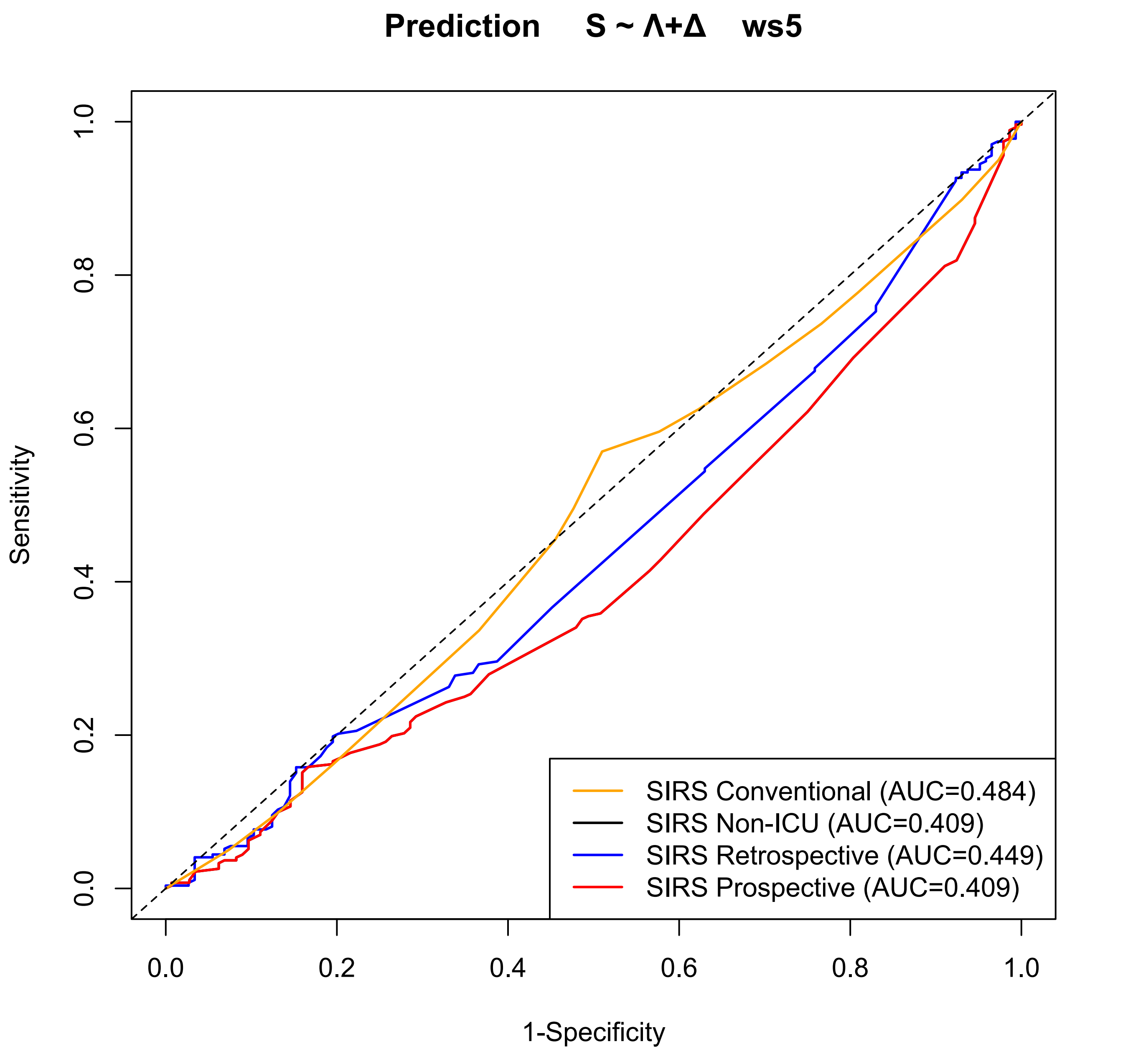
Prediction S ~ Λ+Δ ws5
Sensitivity
1-Specificity
0.0
0.2
0.4
0.6
0.8
1.0
SIRS Conventional (AUC=0.484)
SIRS Non-ICU (AUC=0.409)
SIRS Retrospective (AUC=0.449)
SIRS Prospective (AUC=0.409)

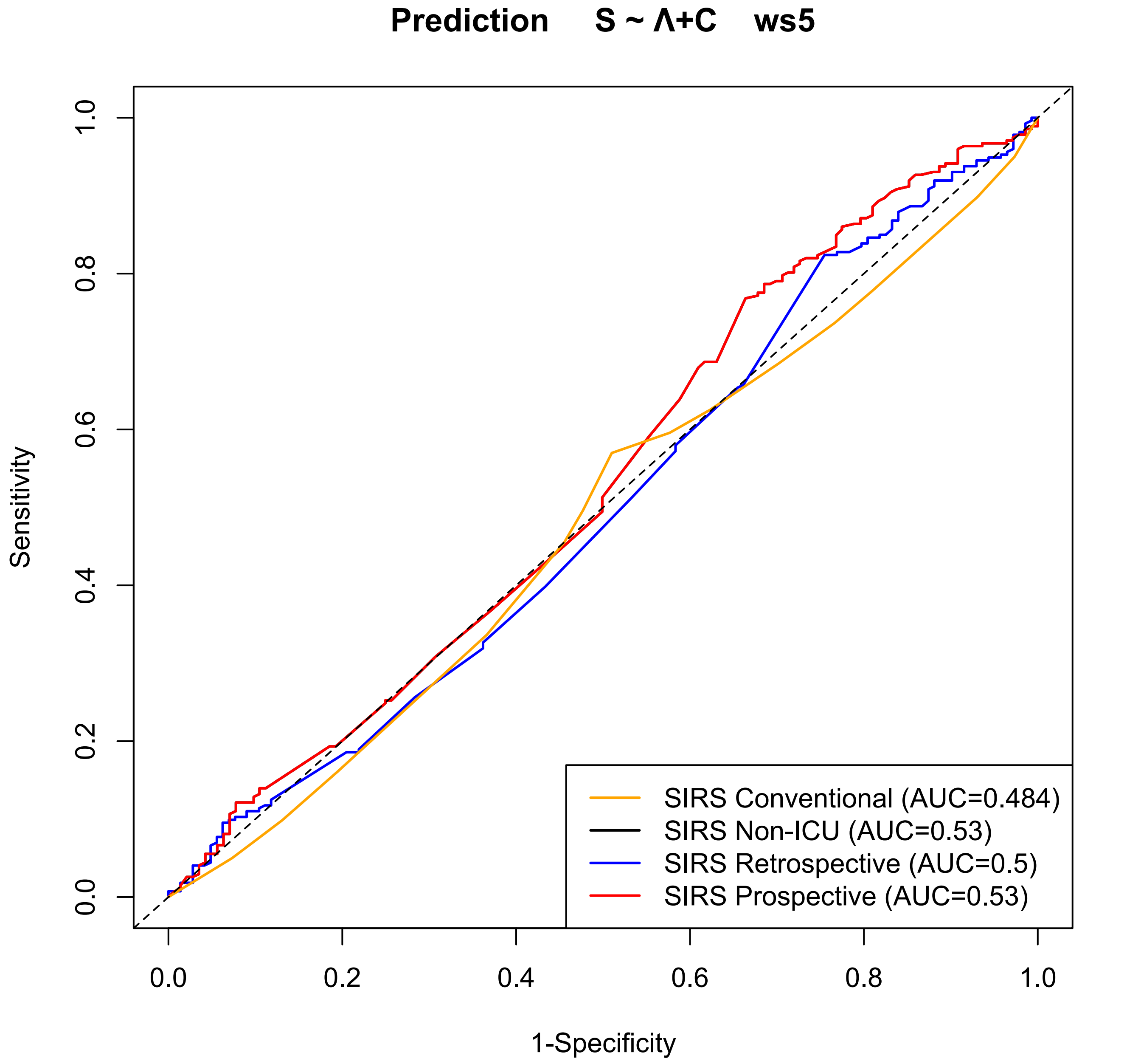

Prediction S ~ Λ+C ws5
Sensitivity
1-Specificity
0.0
0.2
0.4
0.6
0.8
1.0
SIRS Conventional (AUC=0.484)
SIRS Non-ICU (AUC=0.53)
SIRS Retrospective (AUC=0.5)
SIRS Prospective (AUC=0.53)

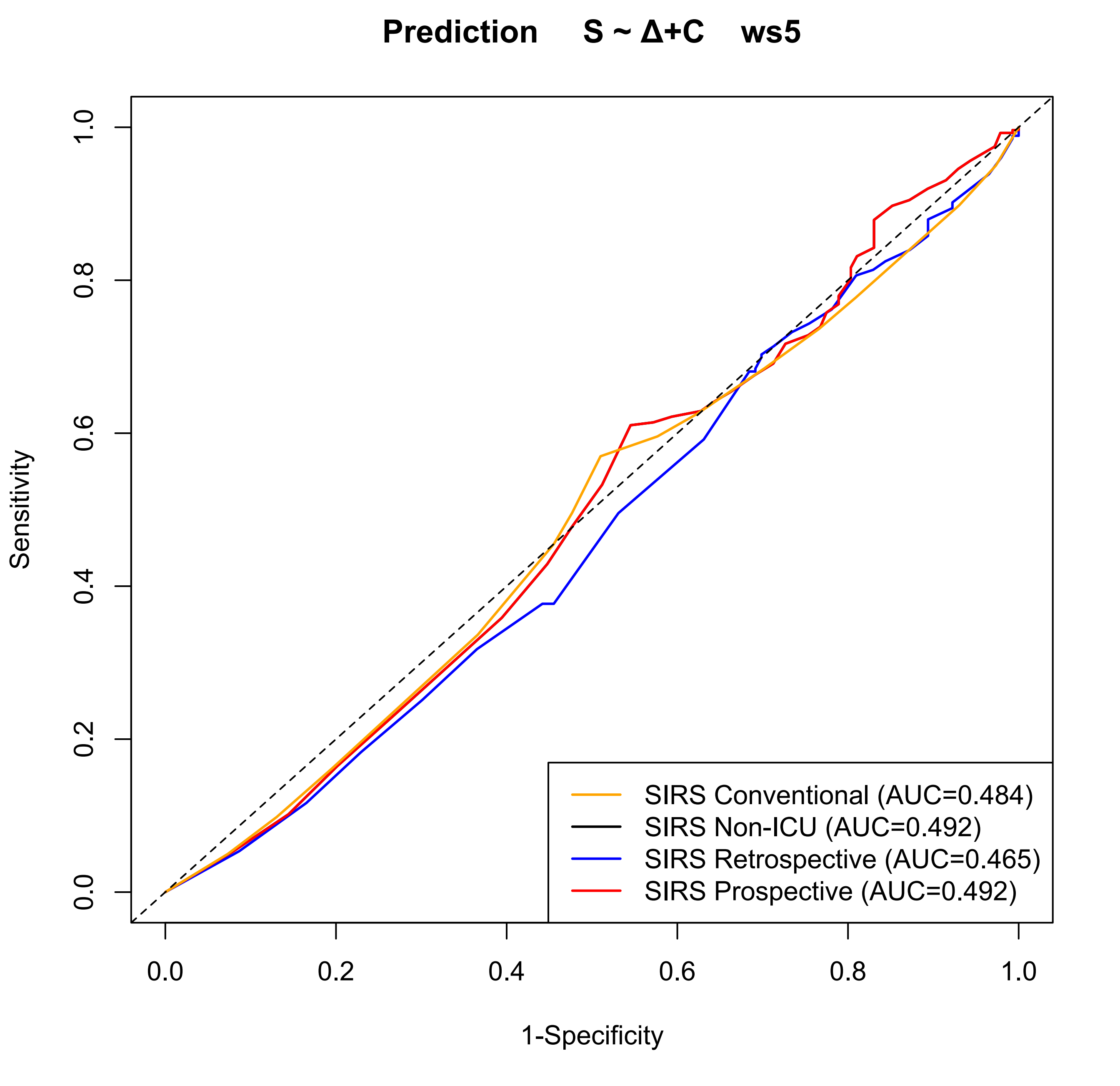
Prediction S ~ Δ+C ws5
Sensitivity
1-Specificity
0.0
0.2
0.4
0.6
0.8
1.0
SIRS Conventional (AUC=0.484)
SIRS Non-ICU (AUC=0.492)
SIRS Retrospective (AUC=0.465)
SIRS Prospective (AUC=0.492)

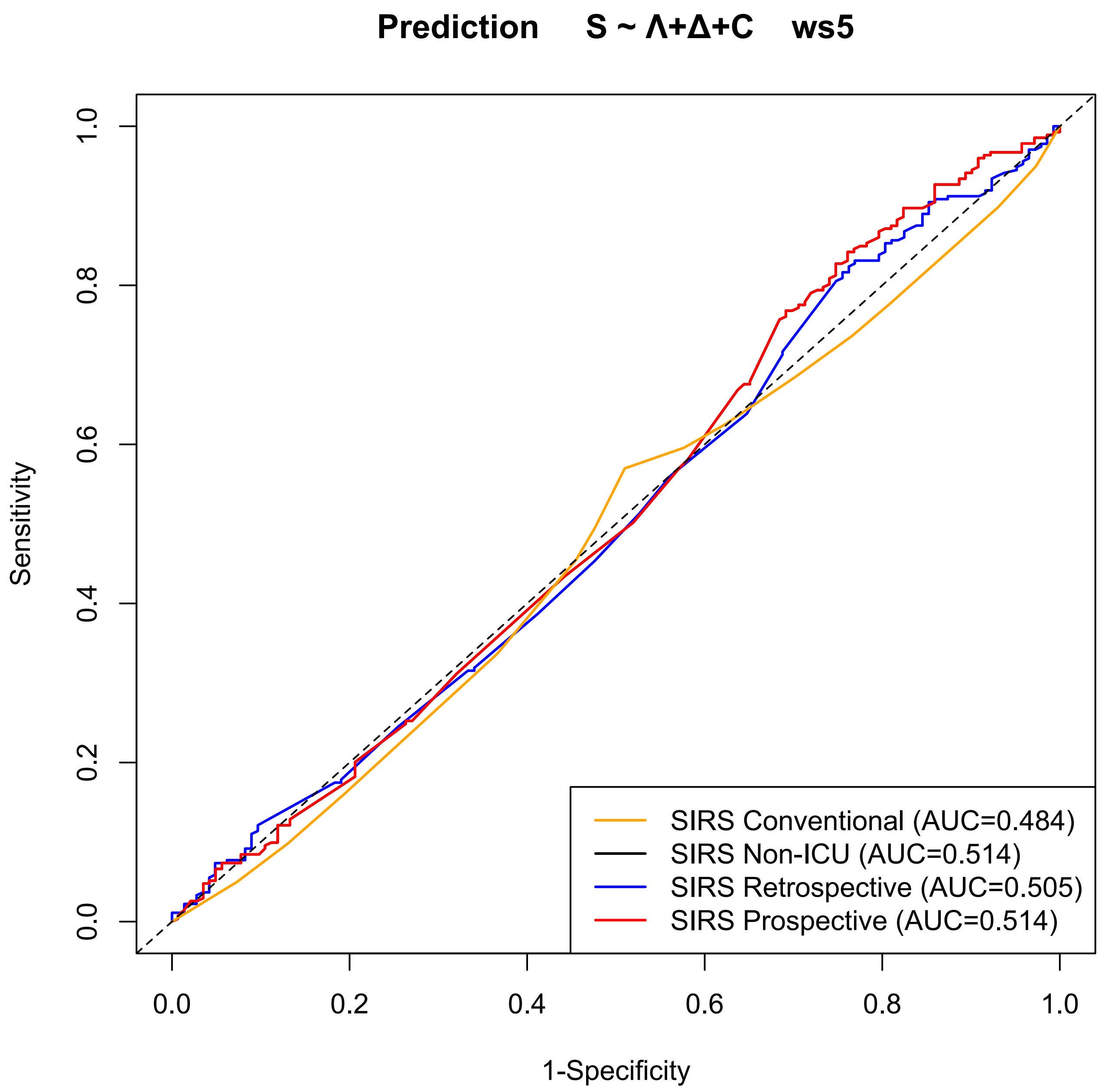
Prediction S ~ Λ+Δ+C ws5
Sensitivity
1-Specificity
0.0
0.2
0.4
0.6
0.8
1.0
SIRS Conventional (AUC=0.484)
SIRS Non-ICU (AUC=0.514)
SIRS Retrospective (AUC=0.505)
SIRS Prospective (AUC=0.514)

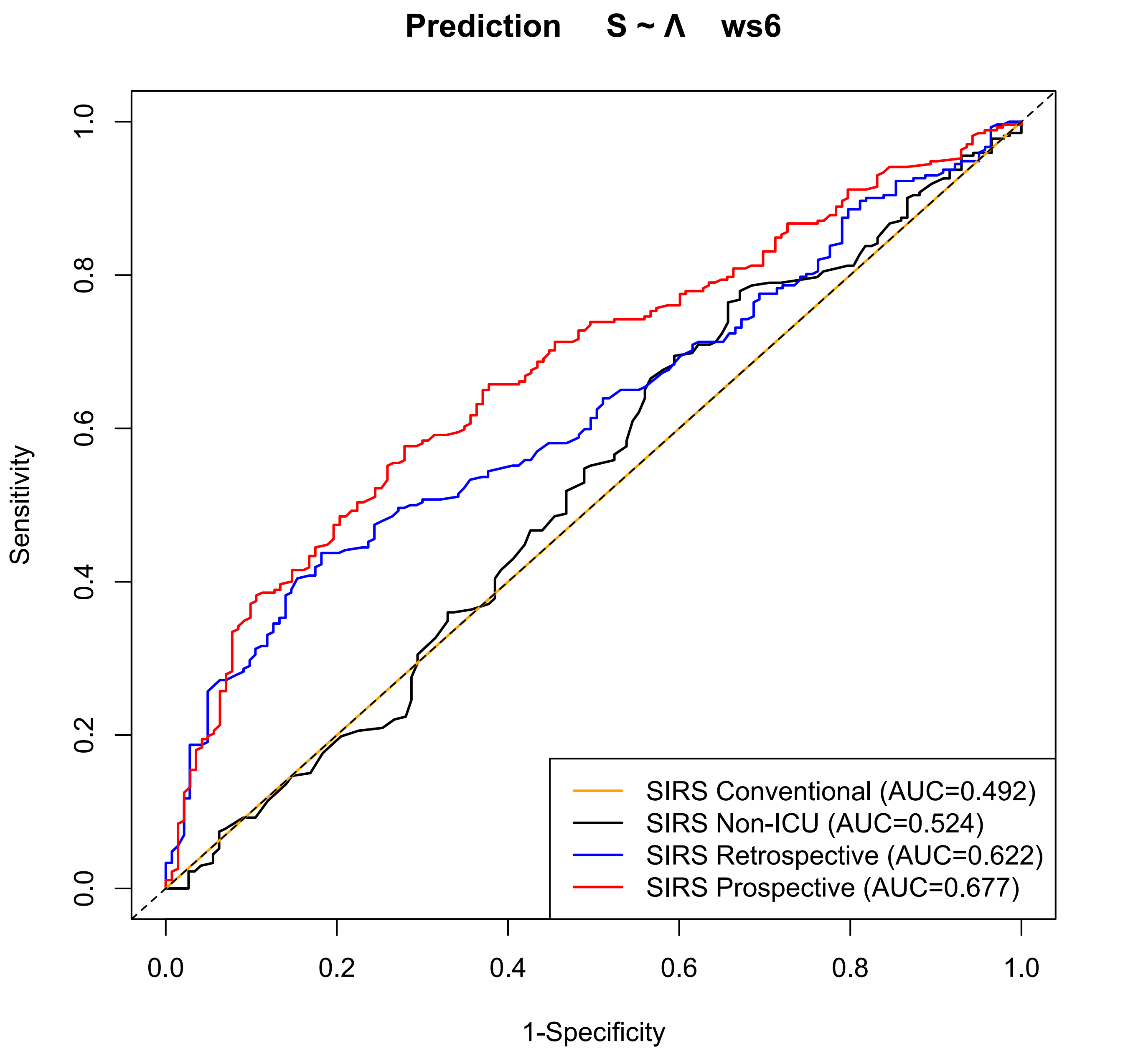

Prediction S ~ Λ ws6
Sensitivity
1-Specificity
0.0
0.2
0.4
0.6
0.8
1.0
SIRS Conventional (AUC=0.492)
SIRS Non-ICU (AUC=0.524)
SIRS Retrospective (AUC=0.622)
SIRS Prospective (AUC=0.677)

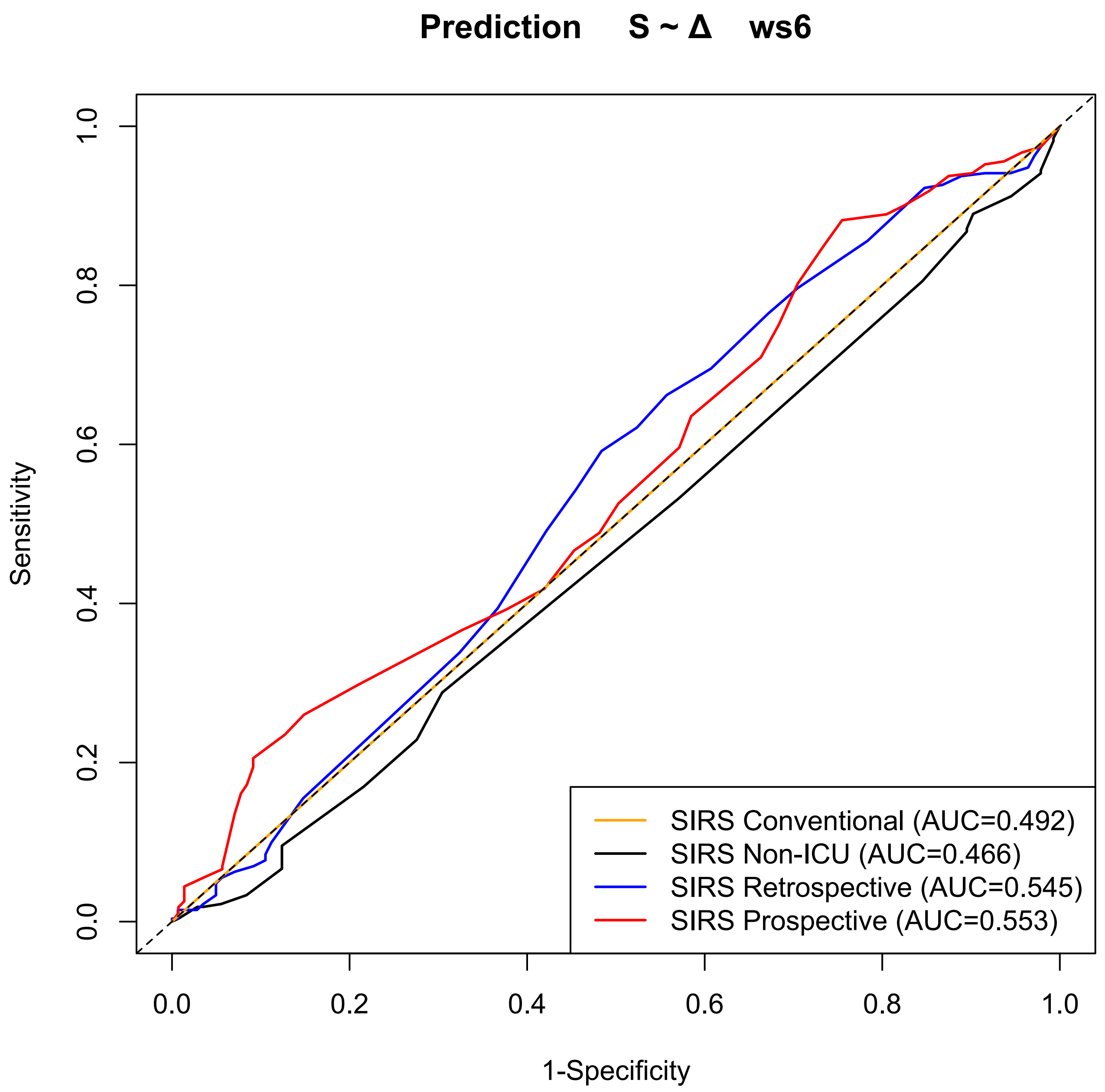

Prediction S ~ Δ ws6
Sensitivity
1-Specificity
0.0
0.2
0.4
0.6
0.8
1.0
SIRS Conventional (AUC=0.492)
SIRS Non-ICU (AUC=0.466)
SIRS Retrospective (AUC=0.545)
SIRS Prospective (AUC=0.553)

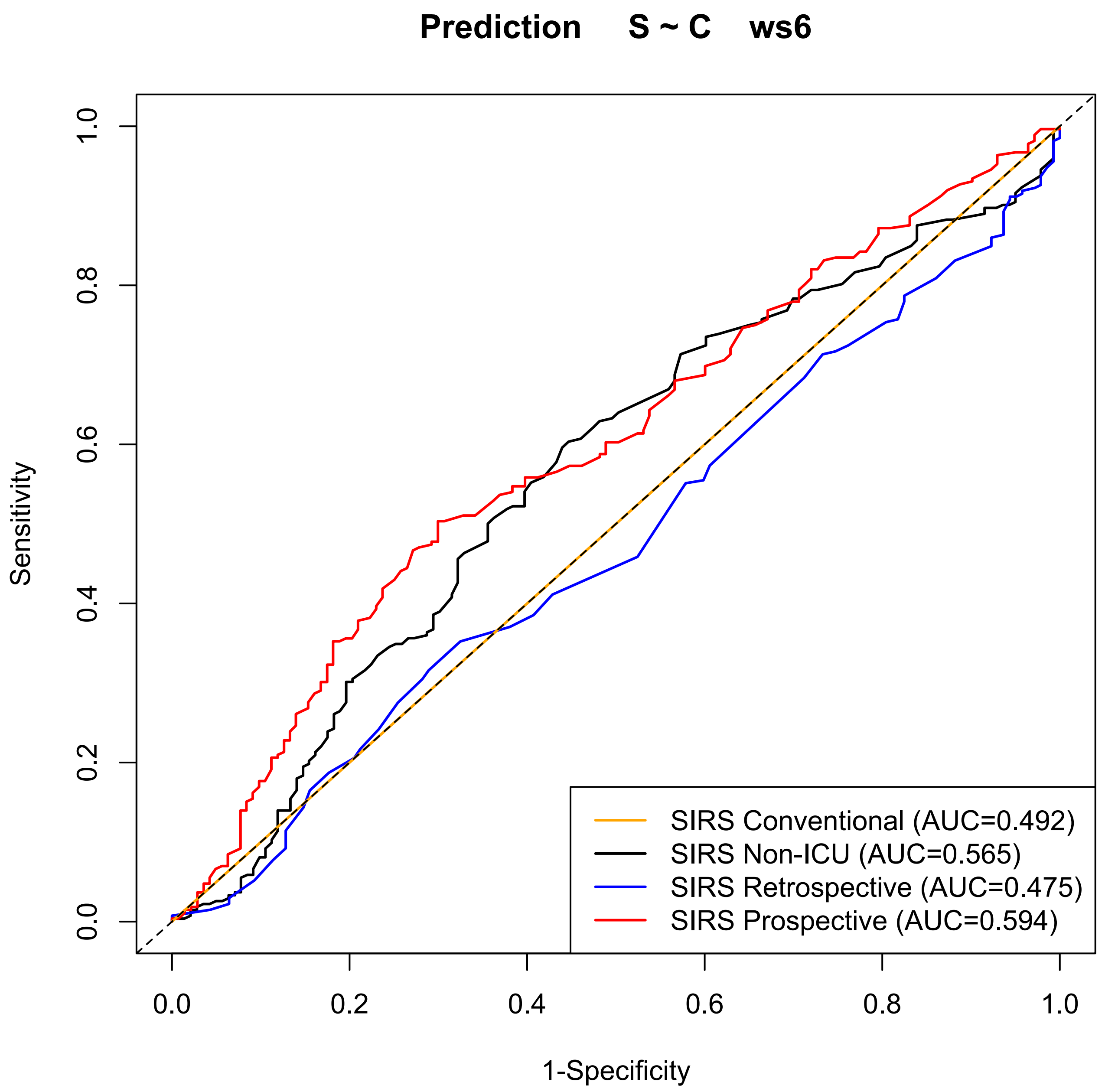

Prediction S ~ C ws6
Sensitivity
1-Specificity
0.0
0.2
0.4
0.6
0.8
1.0
SIRS Conventional (AUC=0.492)
SIRS Non-ICU (AUC=0.565)
SIRS Retrospective (AUC=0.475)
SIRS Prospective (AUC=0.594)

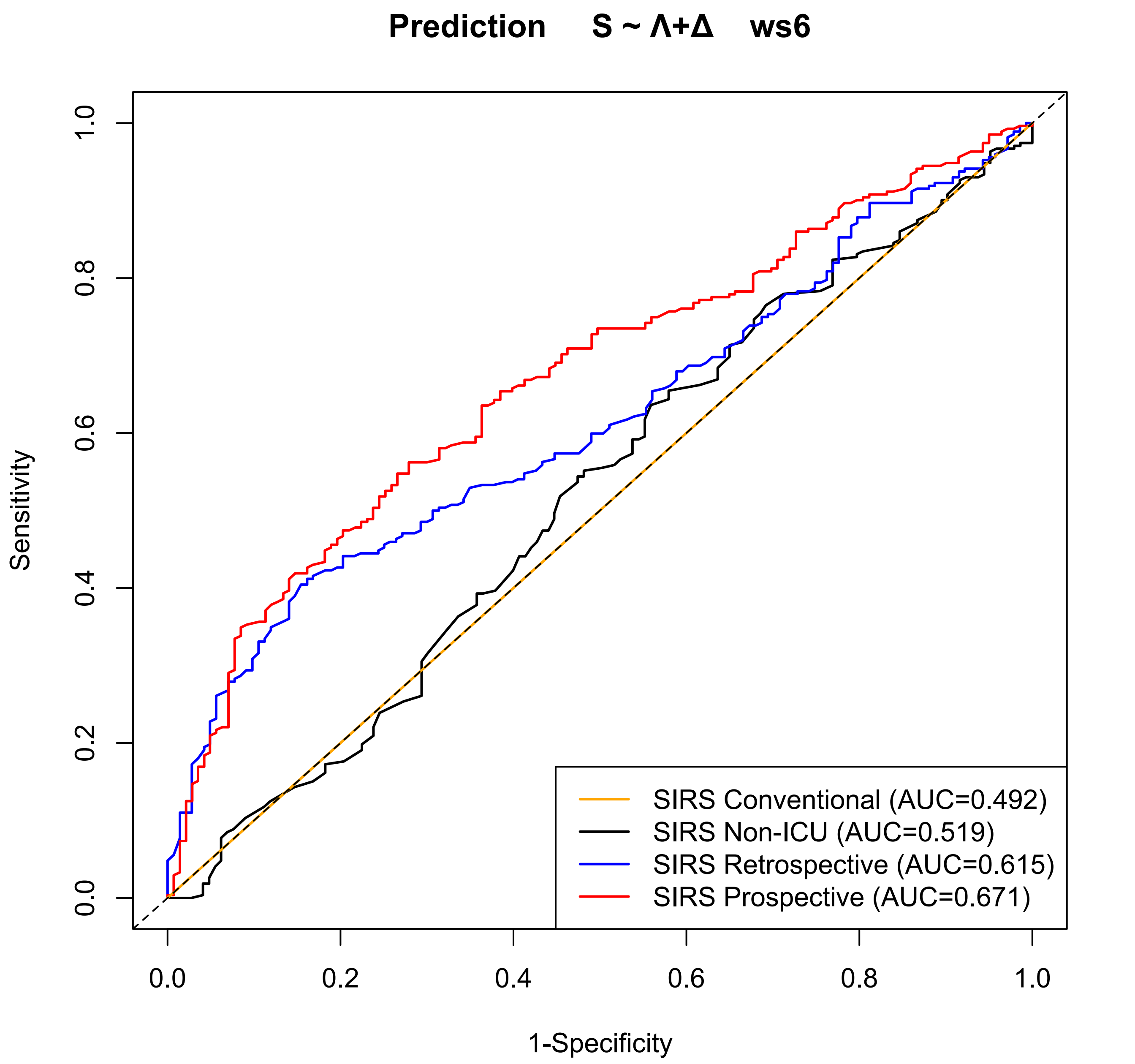

Prediction S ~ Λ+Δ ws6
Sensitivity
1-Specificity
0.0
0.2
0.4
0.6
0.8
1.0
SIRS Conventional (AUC=0.492)
SIRS Non-ICU (AUC=0.519)
SIRS Retrospective (AUC=0.615)
SIRS Prospective (AUC=0.671)

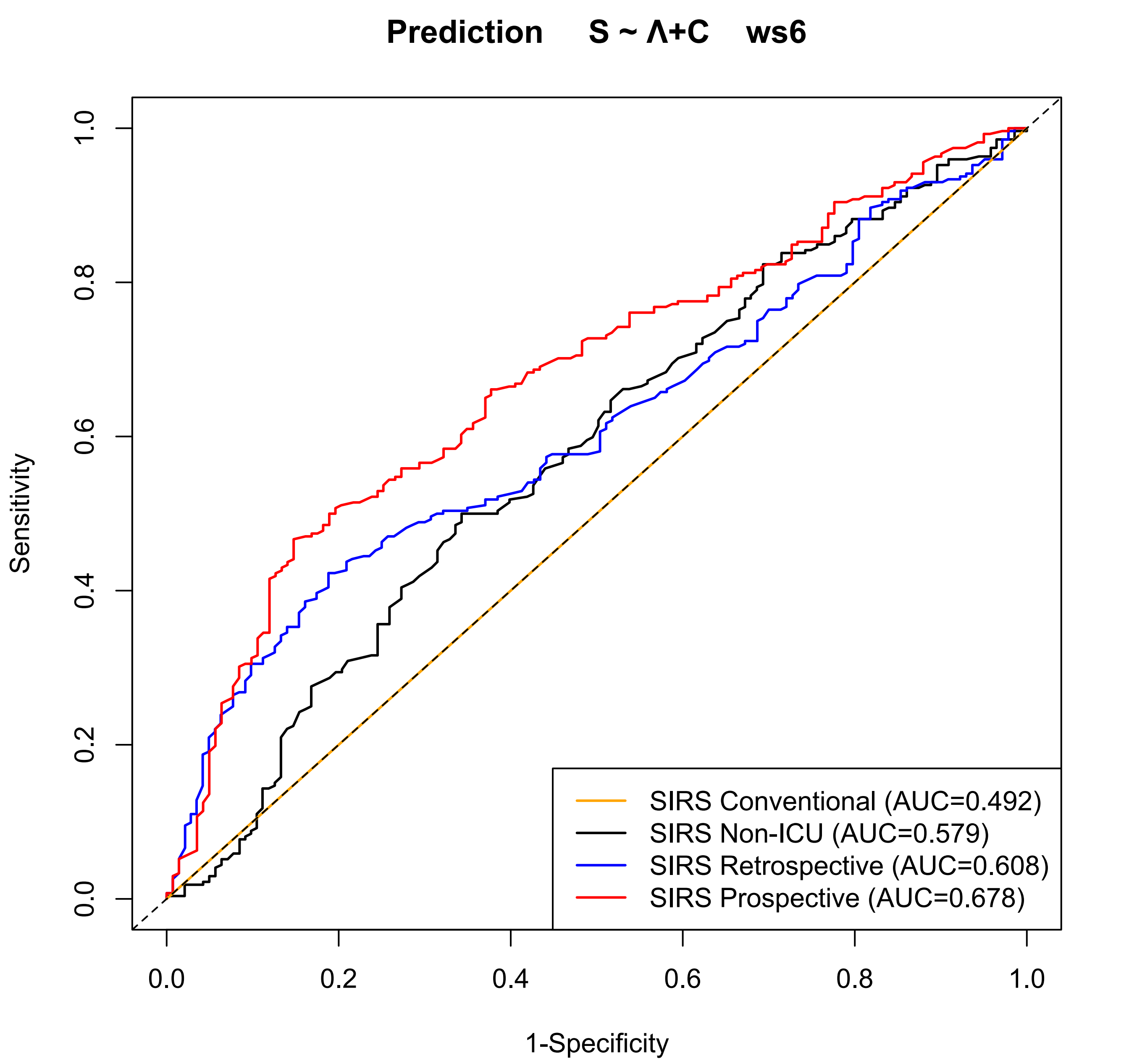

Prediction S ~ Λ+C ws6
Sensitivity
1-Specificity
0.0
0.2
0.4
0.6
0.8
1.0
0.0
0.2
0.4
0.6
0.8
1.0
SIRS Conventional (AUC=0.492)
SIRS Non-ICU (AUC=0.579)
SIRS Retrospective (AUC=0.608)
SIRS Prospective (AUC=0.678)

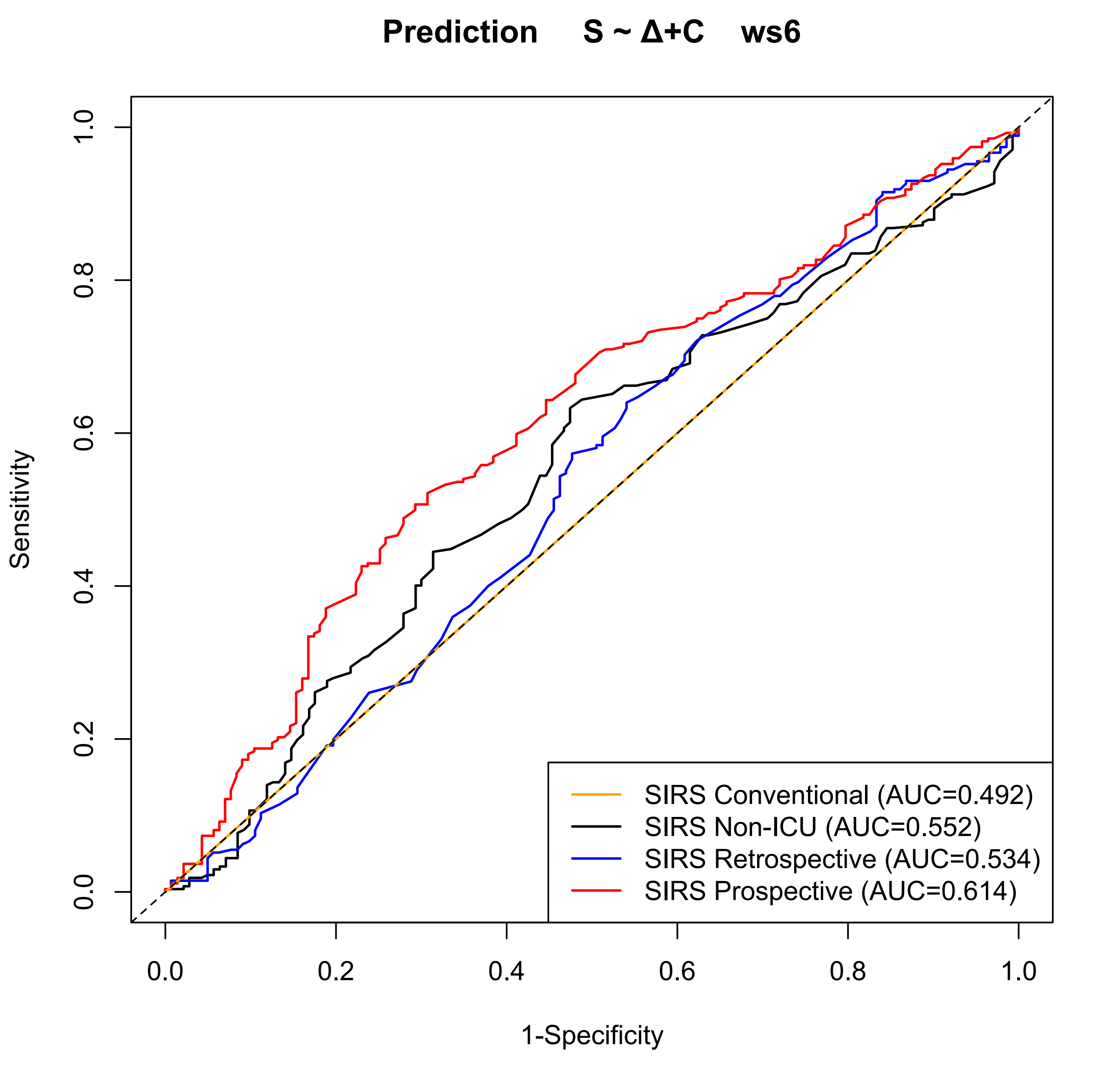

Prediction S ~ Δ+C ws6
Sensitivity
1-Specificity
0.0
0.2
0.4
0.6
0.8
1.0
0.0
0.2
0.4
0.6
0.8
1.0
SIRS Conventional (AUC=0.492)
SIRS Non-ICU (AUC=0.552)
SIRS Retrospective (AUC=0.534)
SIRS Prospective (AUC=0.614)

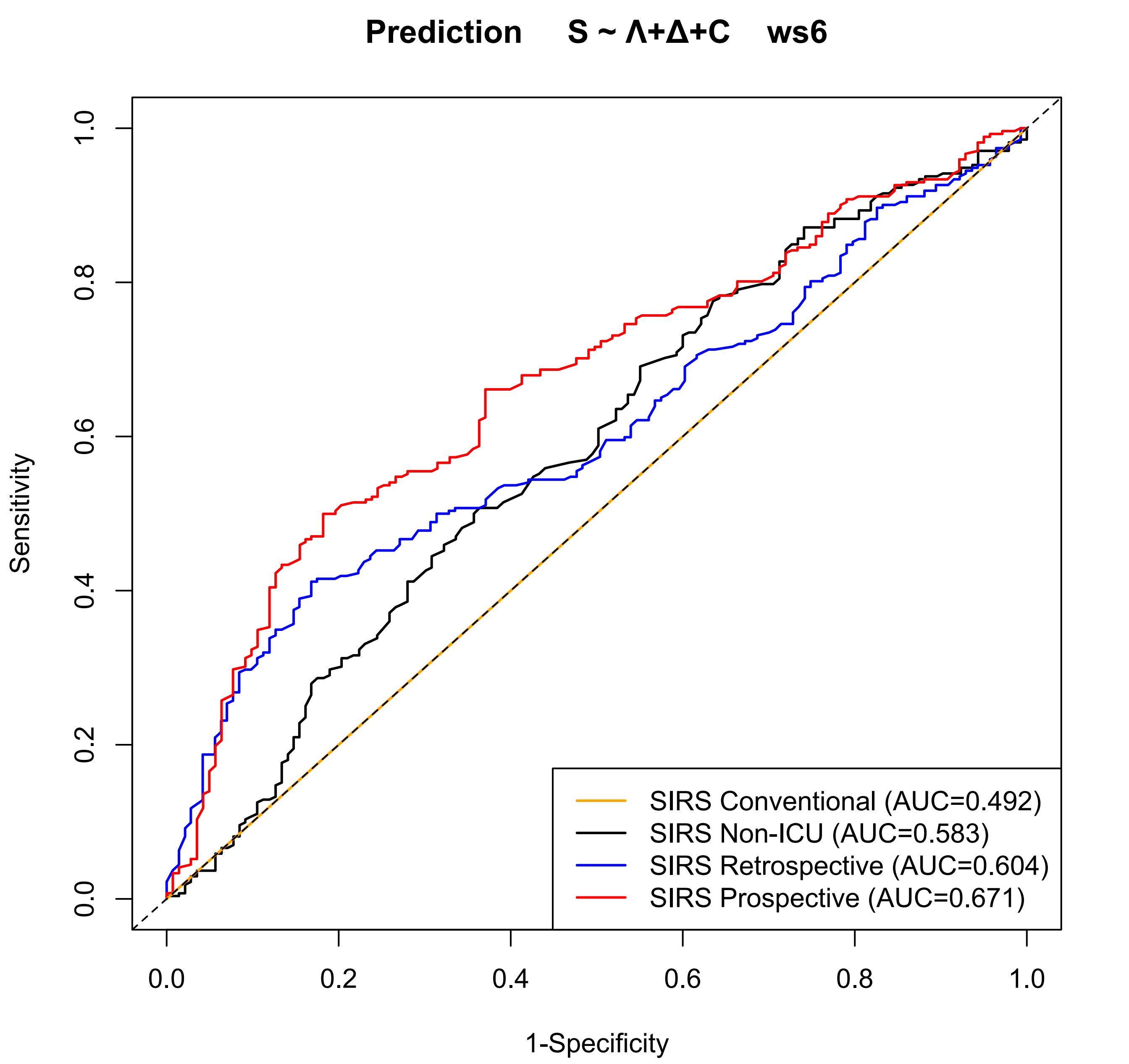

Prediction S ~ Λ+Δ+C ws6
Sensitivity
1-Specificity
0.0
0.2
0.4
0.6
0.8
1.0
SIRS Conventional (AUC=0.492)
SIRS Non-ICU (AUC=0.583)
SIRS Retrospective (AUC=0.604)
SIRS Prospective (AUC=0.671)

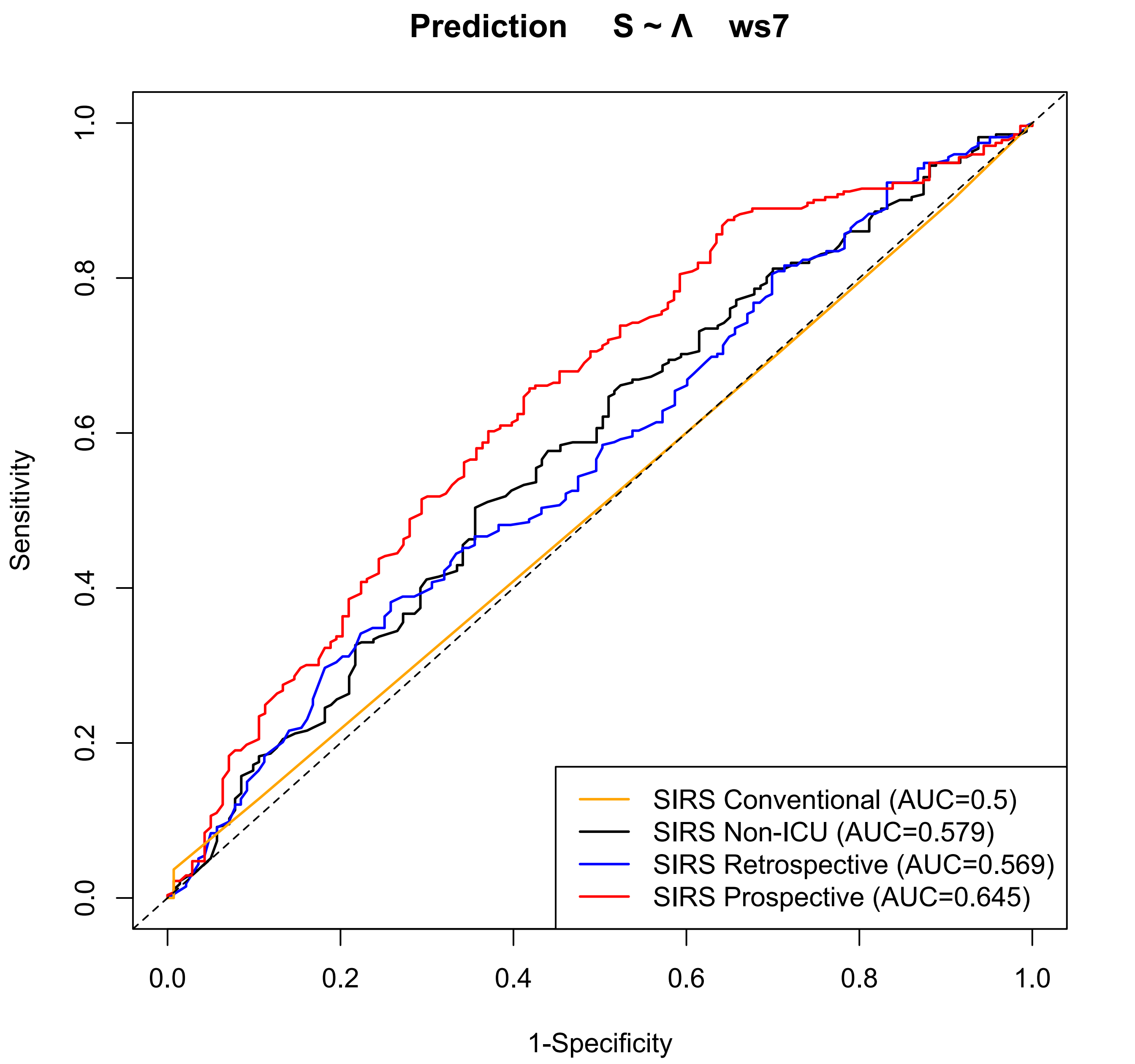

Prediction S ~ Λ ws7
Sensitivity
1-Specificity
0.0
0.2
0.4
0.6
0.8
1.0
SIRS Conventional (AUC=0.5)
SIRS Non-ICU (AUC=0.579)
SIRS Retrospective (AUC=0.569)
SIRS Prospective (AUC=0.645)

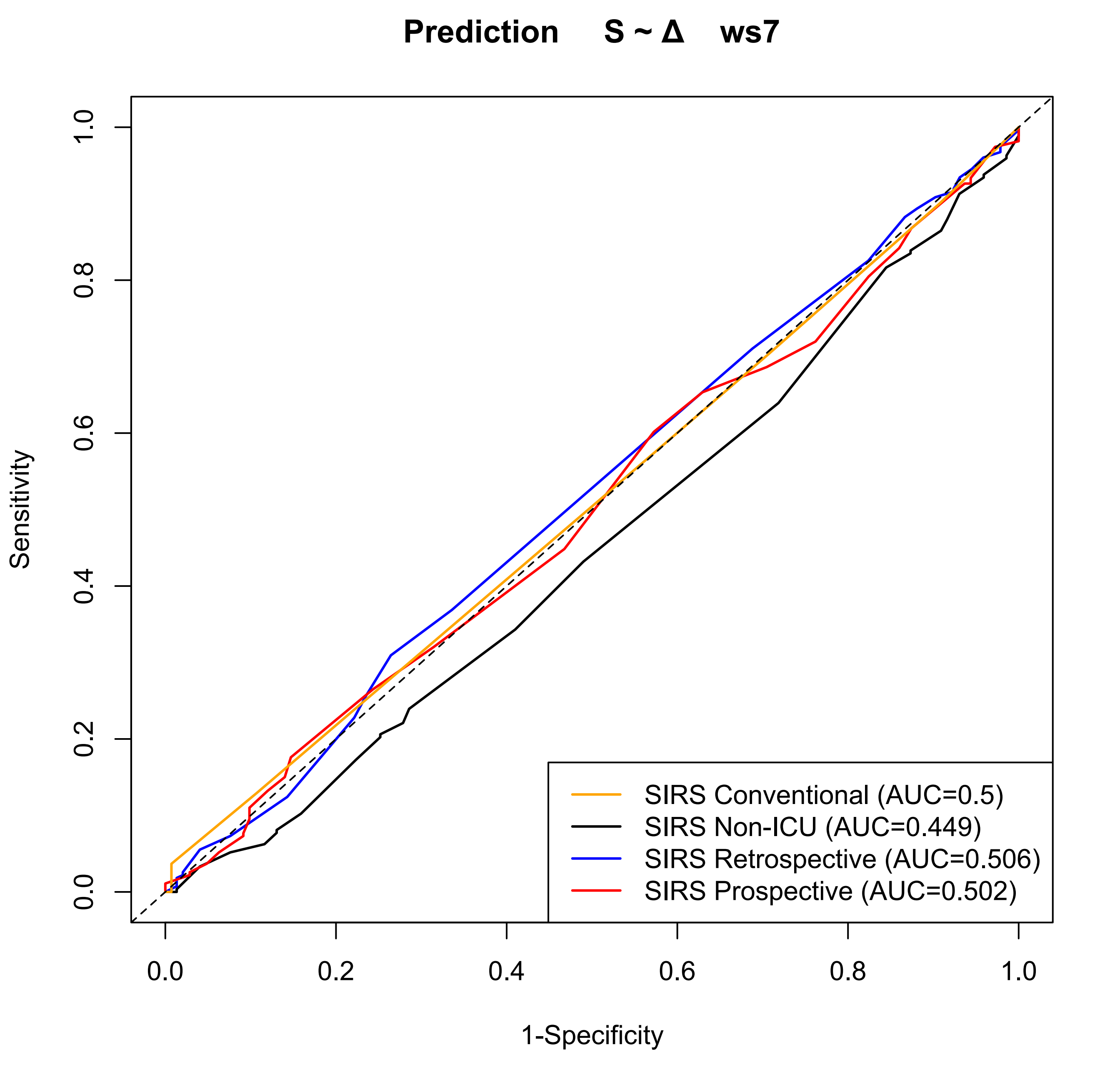

Prediction S ~ Δ ws7
Sensitivity
1-Specificity
0.0
0.2
0.4
0.6
0.8
1.0
SIRS Conventional (AUC=0.5)
SIRS Non-ICU (AUC=0.449)
SIRS Retrospective (AUC=0.506)
SIRS Prospective (AUC=0.502)

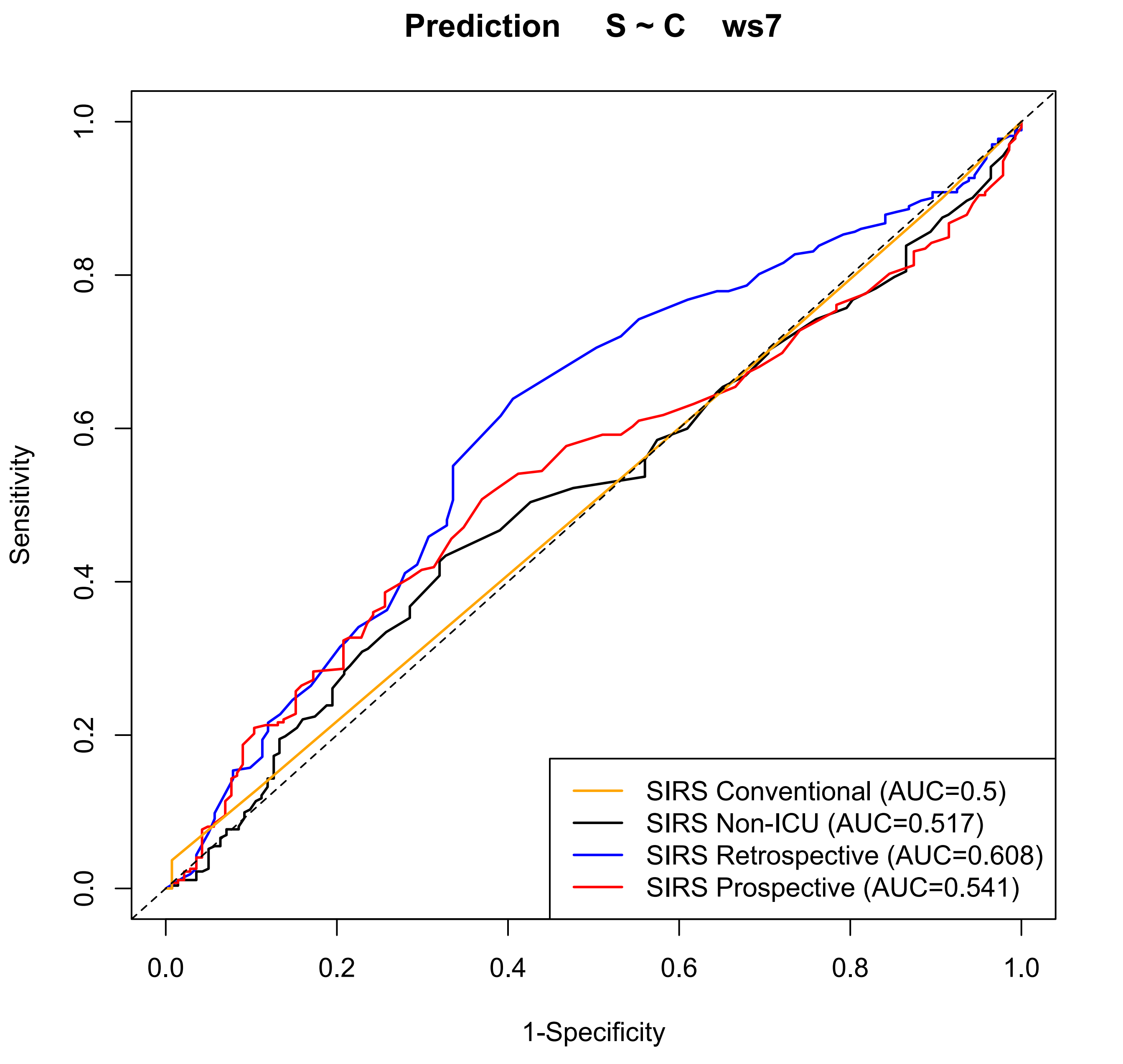

Prediction S ~ C ws7
Sensitivity
1-Specificity
0.0
0.2
0.4
0.6
0.8
1.0
0.0
0.2
0.4
0.6
0.8
1.0
SIRS Conventional (AUC=0.5)
SIRS Non-ICU (AUC=0.517)
SIRS Retrospective (AUC=0.608)
SIRS Prospective (AUC=0.541)

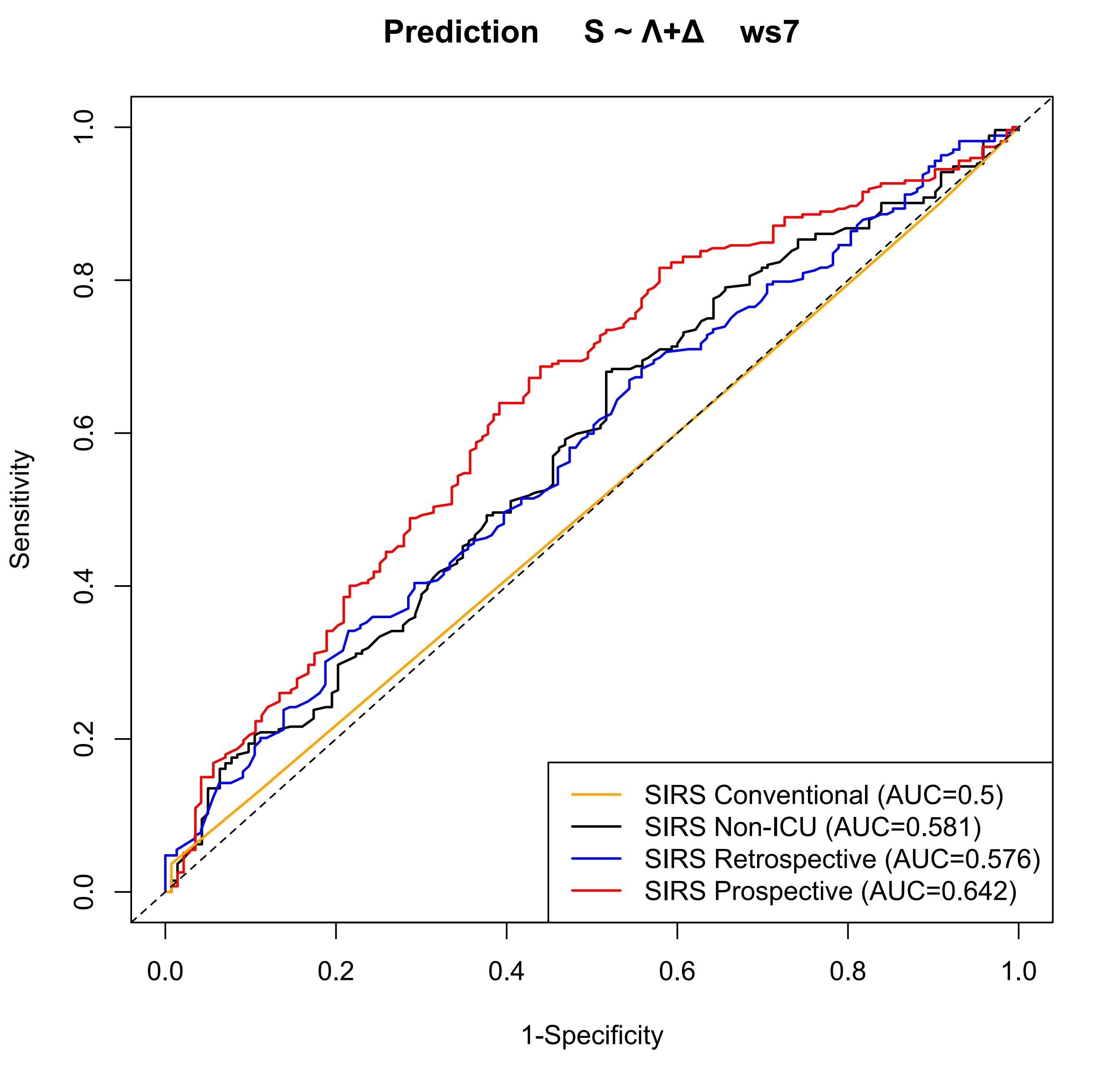

Prediction S ~ Λ+Δ ws7
Sensitivity
1-Specificity
0.0
0.2
0.4
0.6
0.8
1.0
SIRS Conventional (AUC=0.5)
SIRS Non-ICU (AUC=0.581)
SIRS Retrospective (AUC=0.576)
SIRS Prospective (AUC=0.642)

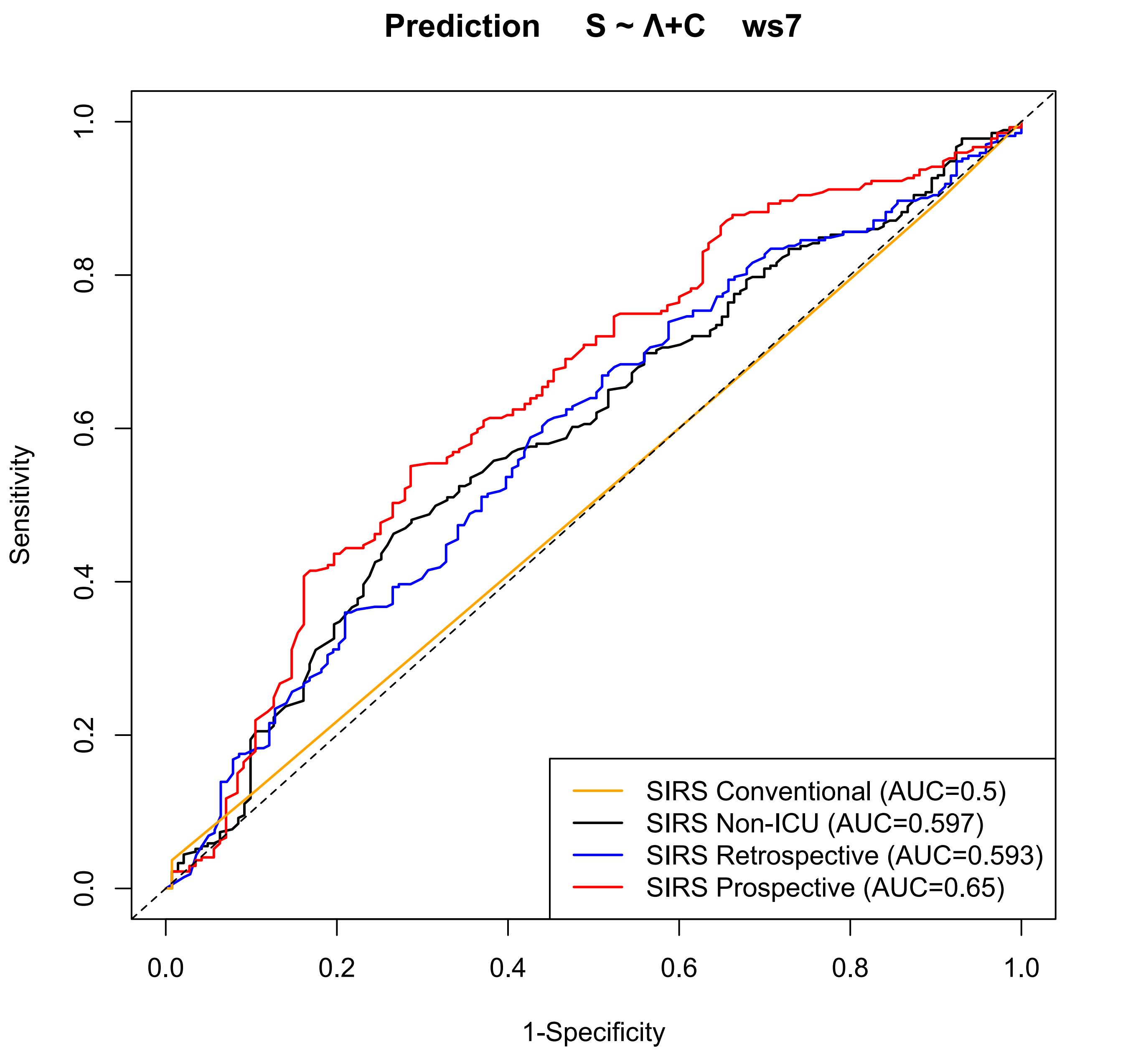

Prediction S ~ Λ+C ws7
Sensitivity
1-Specificity
0.0
0.2
0.4
0.6
0.8
1.0
0.0
0.2
0.4
0.6
0.8
1.0
SIRS Conventional (AUC=0.5)
SIRS Non-ICU (AUC=0.597)
SIRS Retrospective (AUC=0.593)
SIRS Prospective (AUC=0.65)

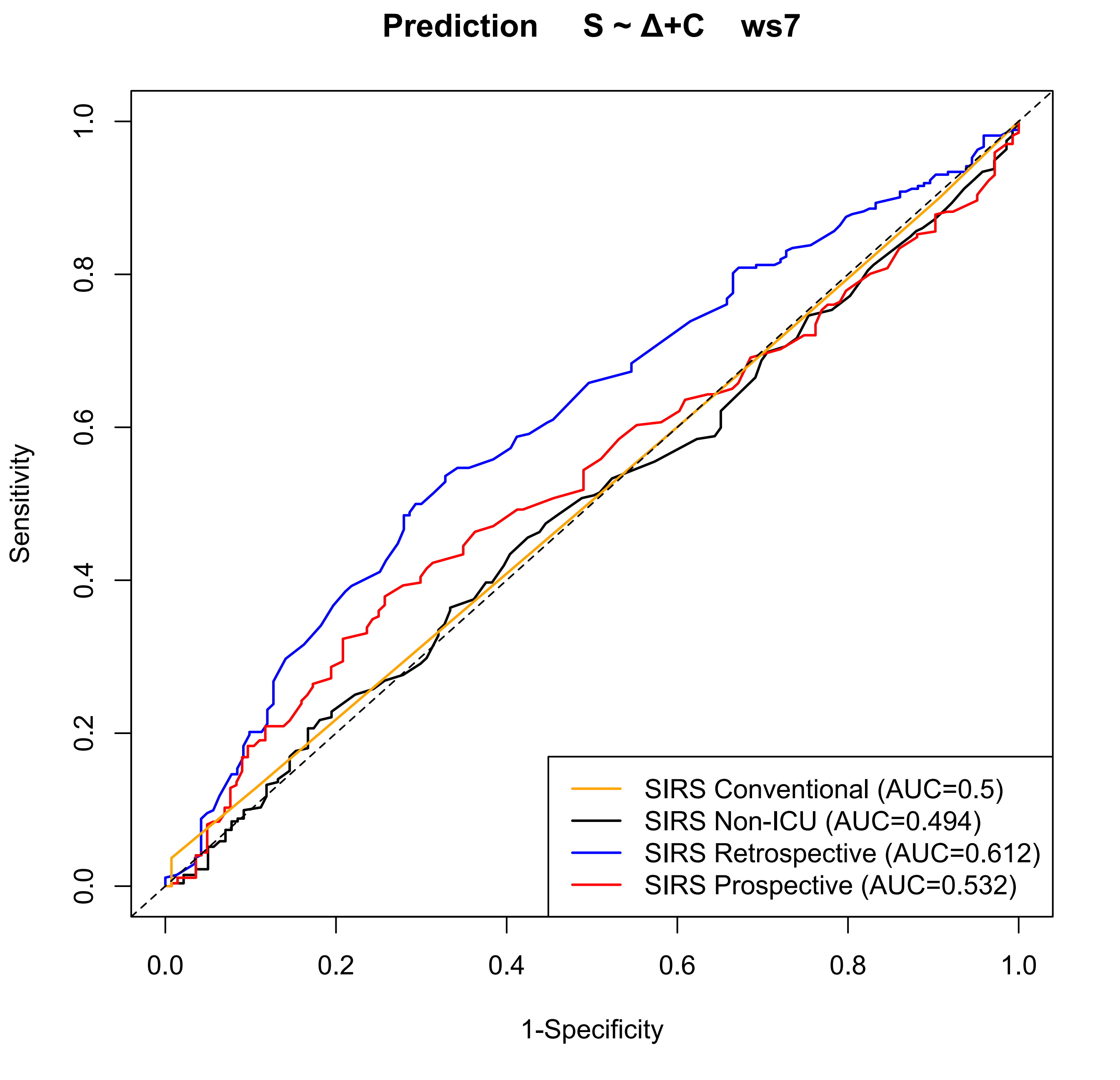

Prediction S ~ Δ+C ws7
Sensitivity
1-Specificity
0.0
0.2
0.4
0.6
0.8
1.0
SIRS Conventional (AUC=0.5)
SIRS Non-ICU (AUC=0.494)
SIRS Retrospective (AUC=0.612)
SIRS Prospective (AUC=0.532)

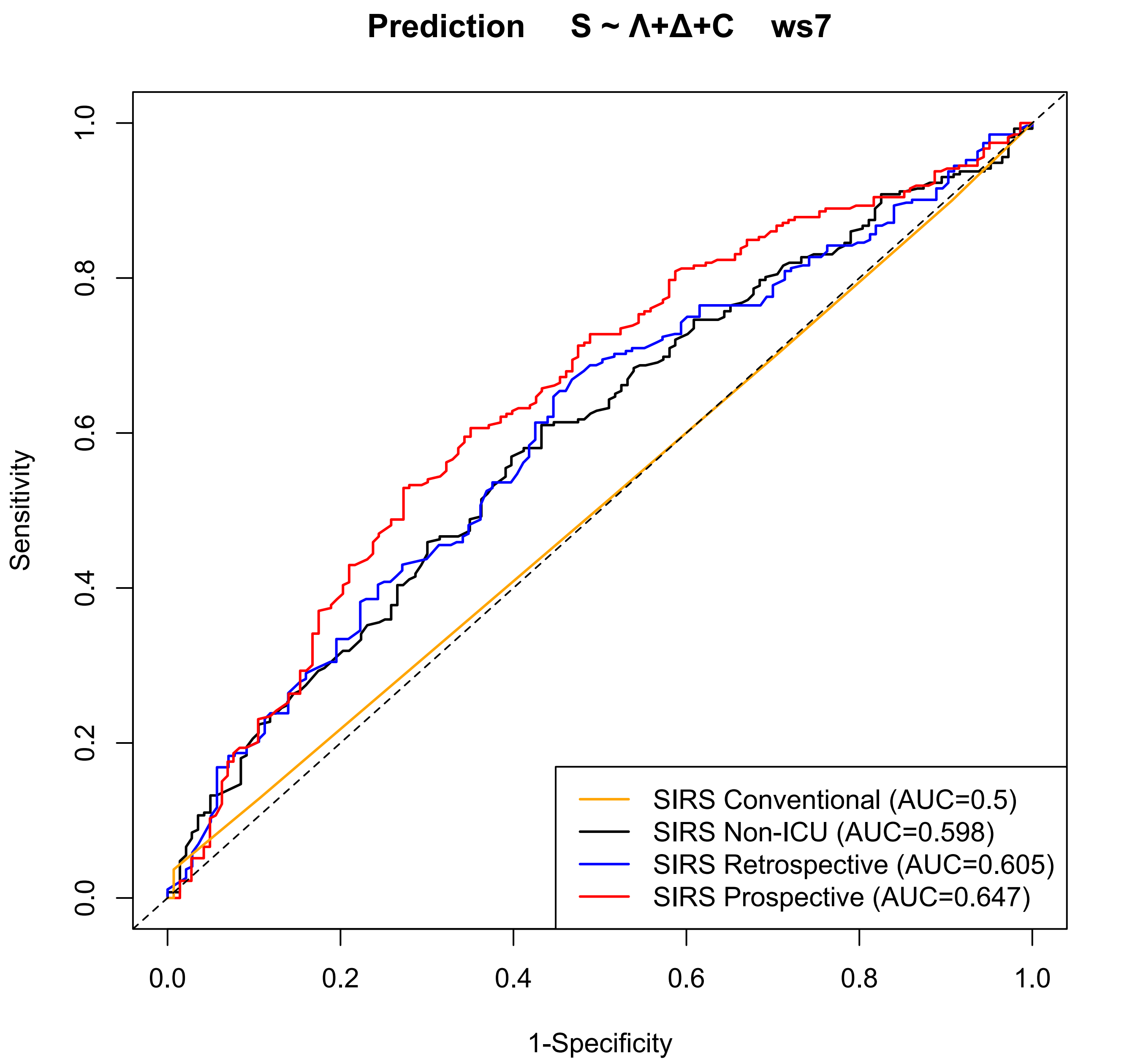
Prediction S ~ Λ+Δ+C ws7
Sensitivity
1-Specificity
0.0
0.2
0.4
0.6
0.8
1.0
SIRS Conventional (AUC=0.5)
SIRS Non-ICU (AUC=0.598)
SIRS Retrospective (AUC=0.605)
SIRS Prospective (AUC=0.647)

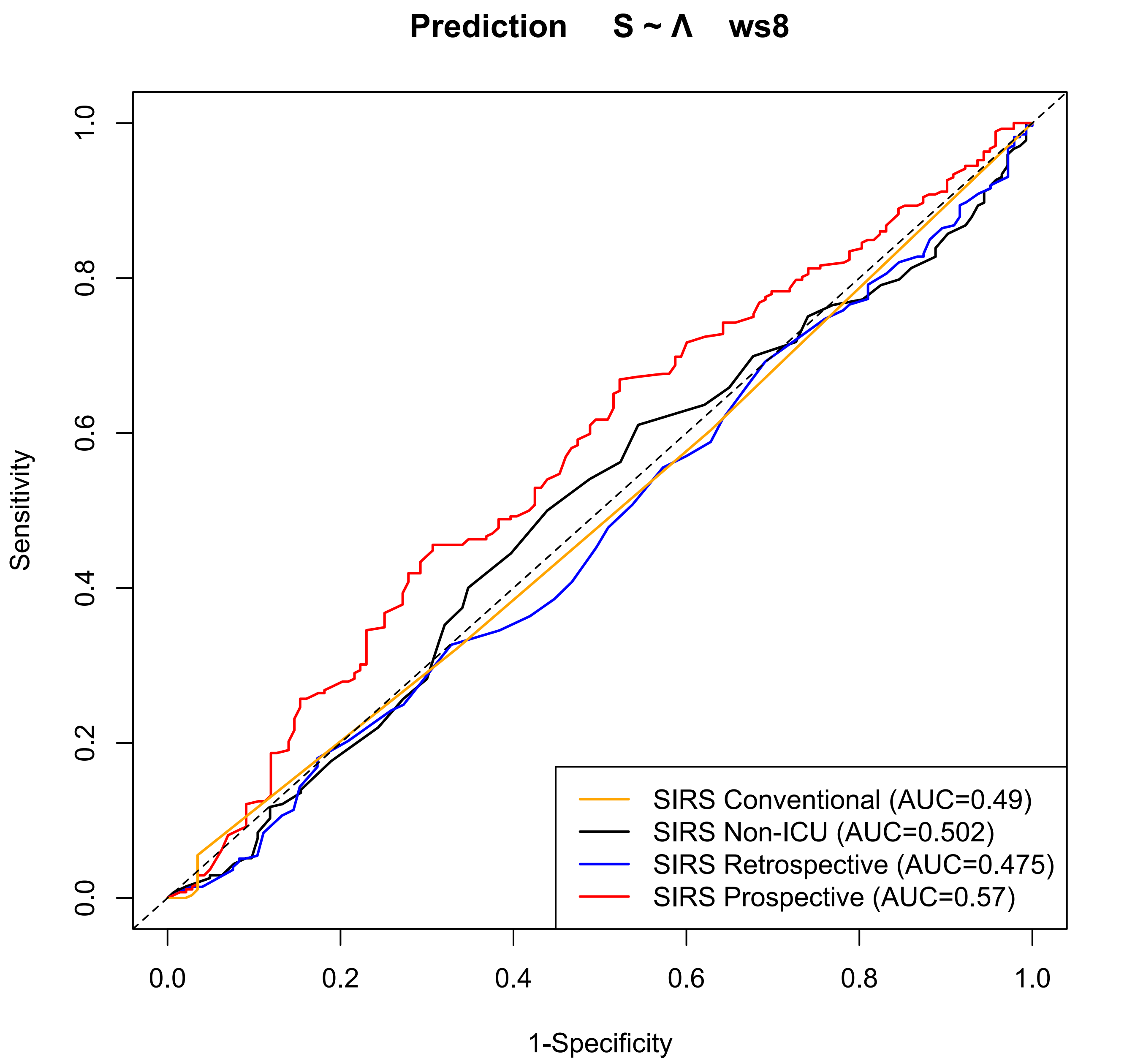

Prediction S ~ Λ ws8
Sensitivity
1-Specificity
0.0
0.2
0.4
0.6
0.8
1.0
SIRS Conventional (AUC=0.49)
SIRS Non-ICU (AUC=0.502)
SIRS Retrospective (AUC=0.475)
SIRS Prospective (AUC=0.57)

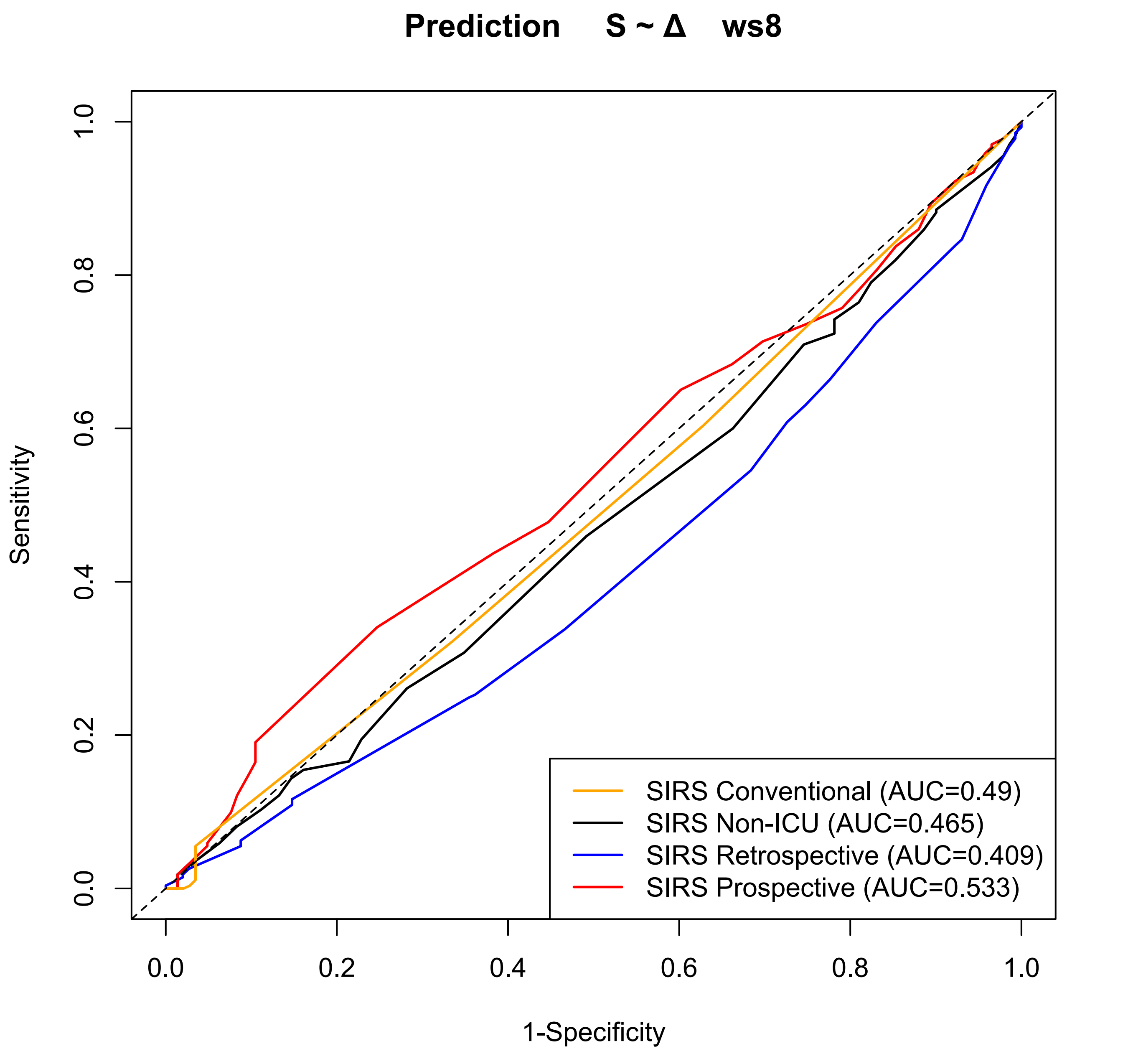

Prediction S ~ Δ ws8
Sensitivity
1-Specificity
0.0
0.2
0.4
0.6
0.8
1.0
SIRS Conventional (AUC=0.49)
SIRS Non-ICU (AUC=0.465)
SIRS Retrospective (AUC=0.409)
SIRS Prospective (AUC=0.533)

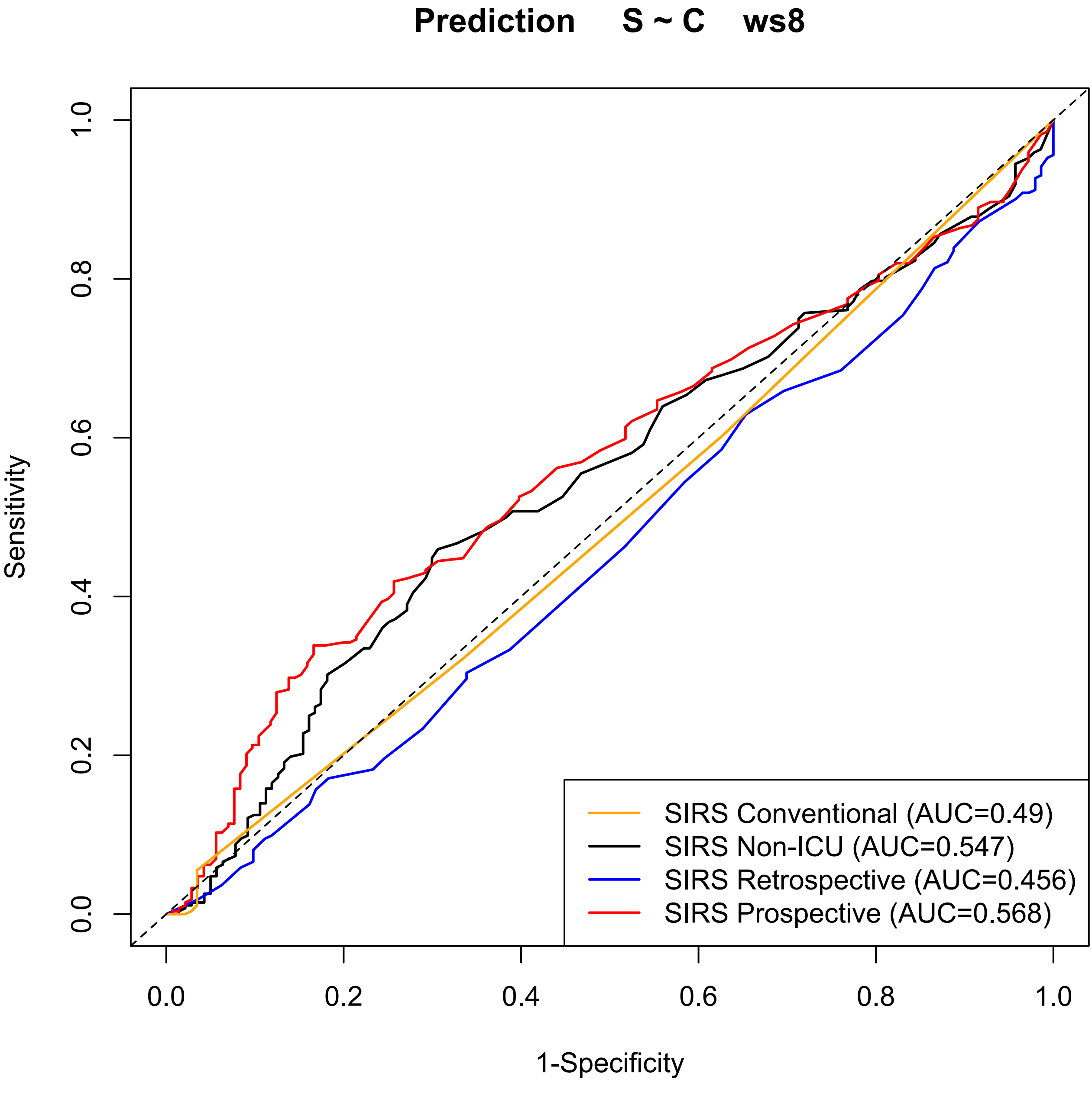

Prediction S ~ C ws8
Sensitivity
1-Specificity
0.0
0.2
0.4
0.6
0.8
1.0
SIRS Conventional (AUC=0.49)
SIRS Non-ICU (AUC=0.547)
SIRS Retrospective (AUC=0.456)
SIRS Prospective (AUC=0.568)

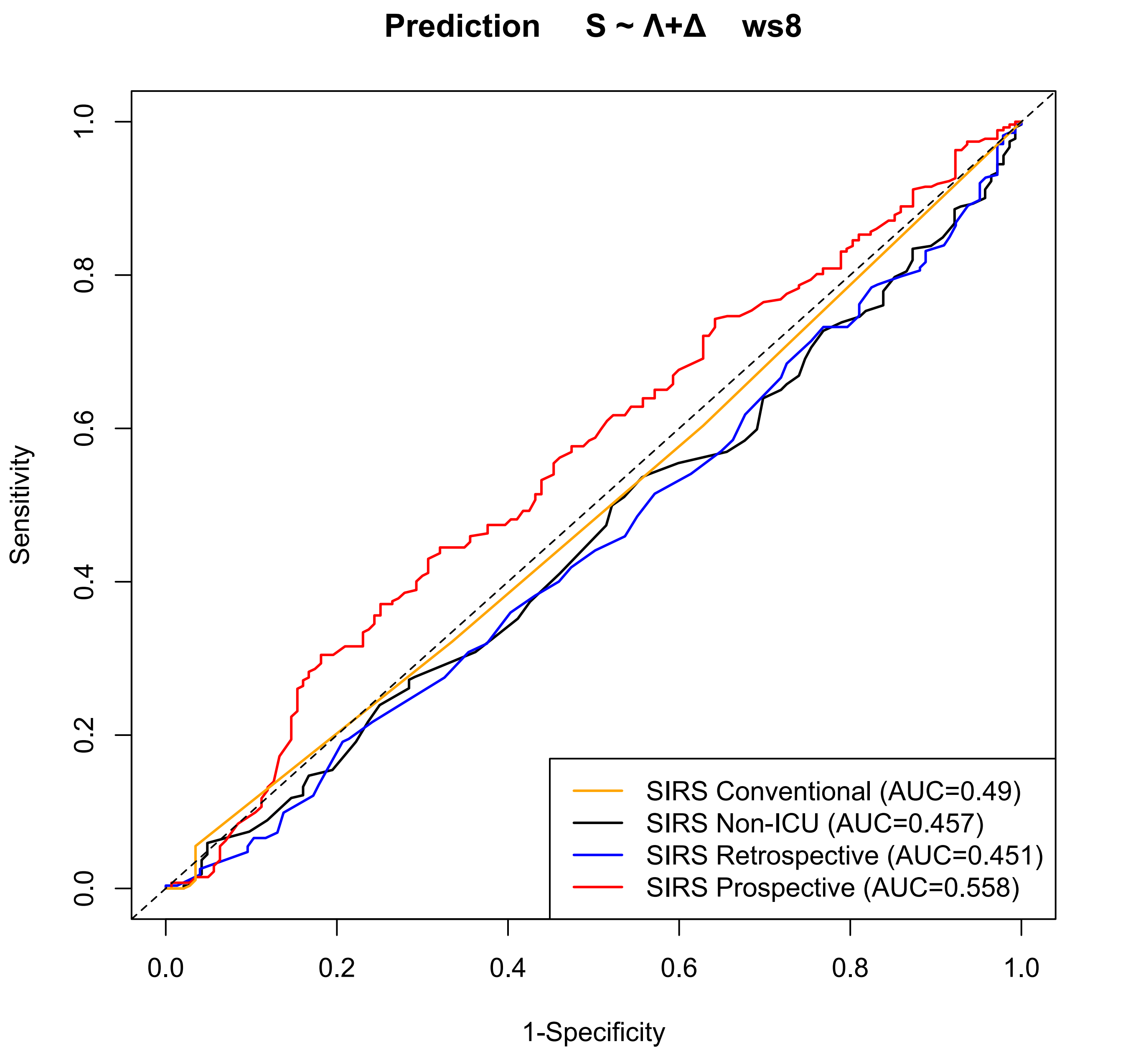

Prediction S ~ Λ+Δ ws8
1.0
0.8
0.6
0.4
0.2
0.0
Sensitivity
0.0
0.2
0.4
0.6
0.8
1.0
1-Specificity
SIRS Conventional (AUC=0.49)
SIRS Non-ICU (AUC=0.457)
SIRS Retrospective (AUC=0.451)
SIRS Prospective (AUC=0.558)

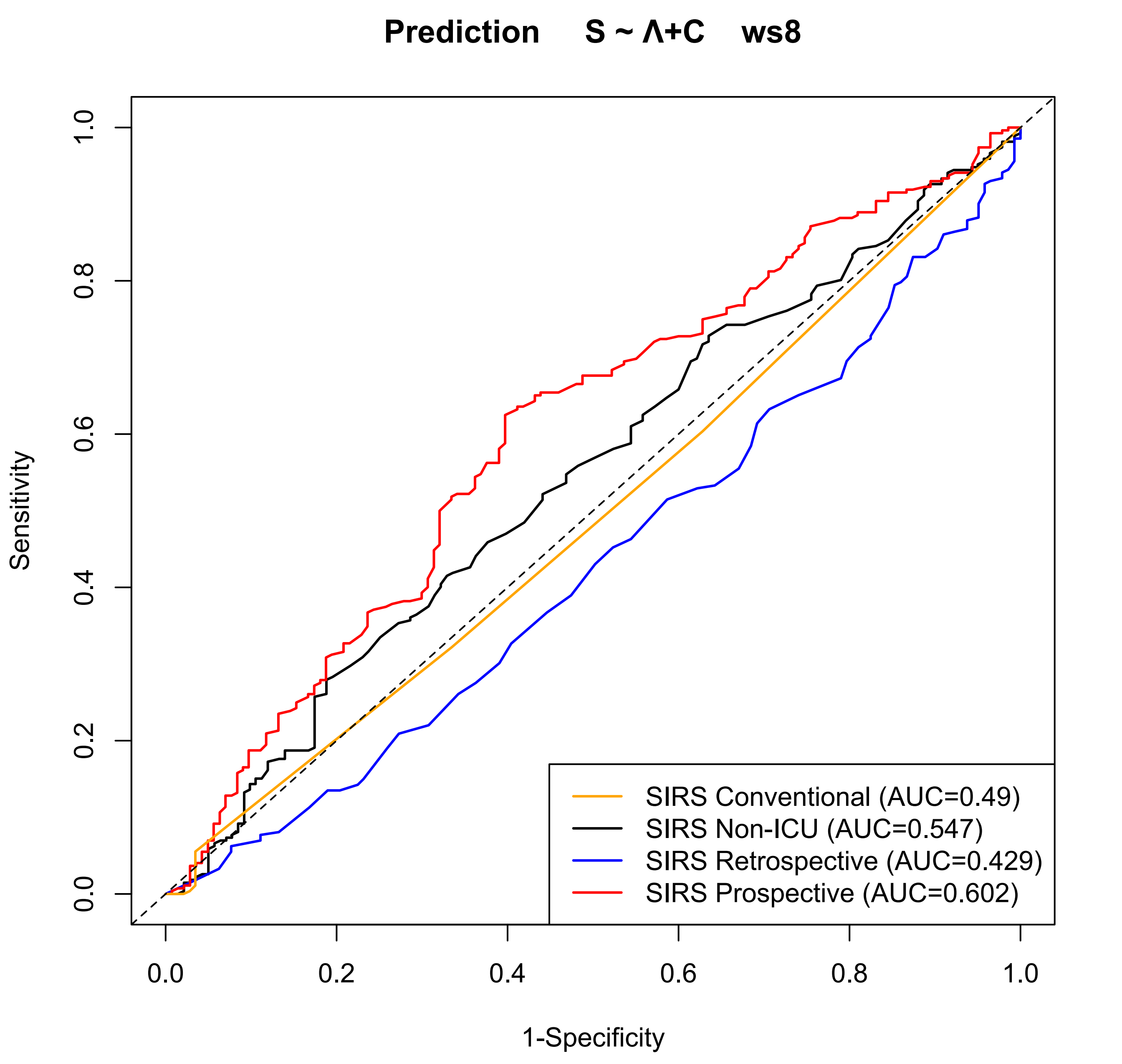
Prediction S ~ Λ+C ws8
Sensitivity
1-Specificity
0.0
0.2
0.4
0.6
0.8
1.0
0.0
0.2
0.4
0.6
0.8
1.0
SIRS Conventional (AUC=0.49)
SIRS Non-ICU (AUC=0.547)
SIRS Retrospective (AUC=0.429)
SIRS Prospective (AUC=0.602)

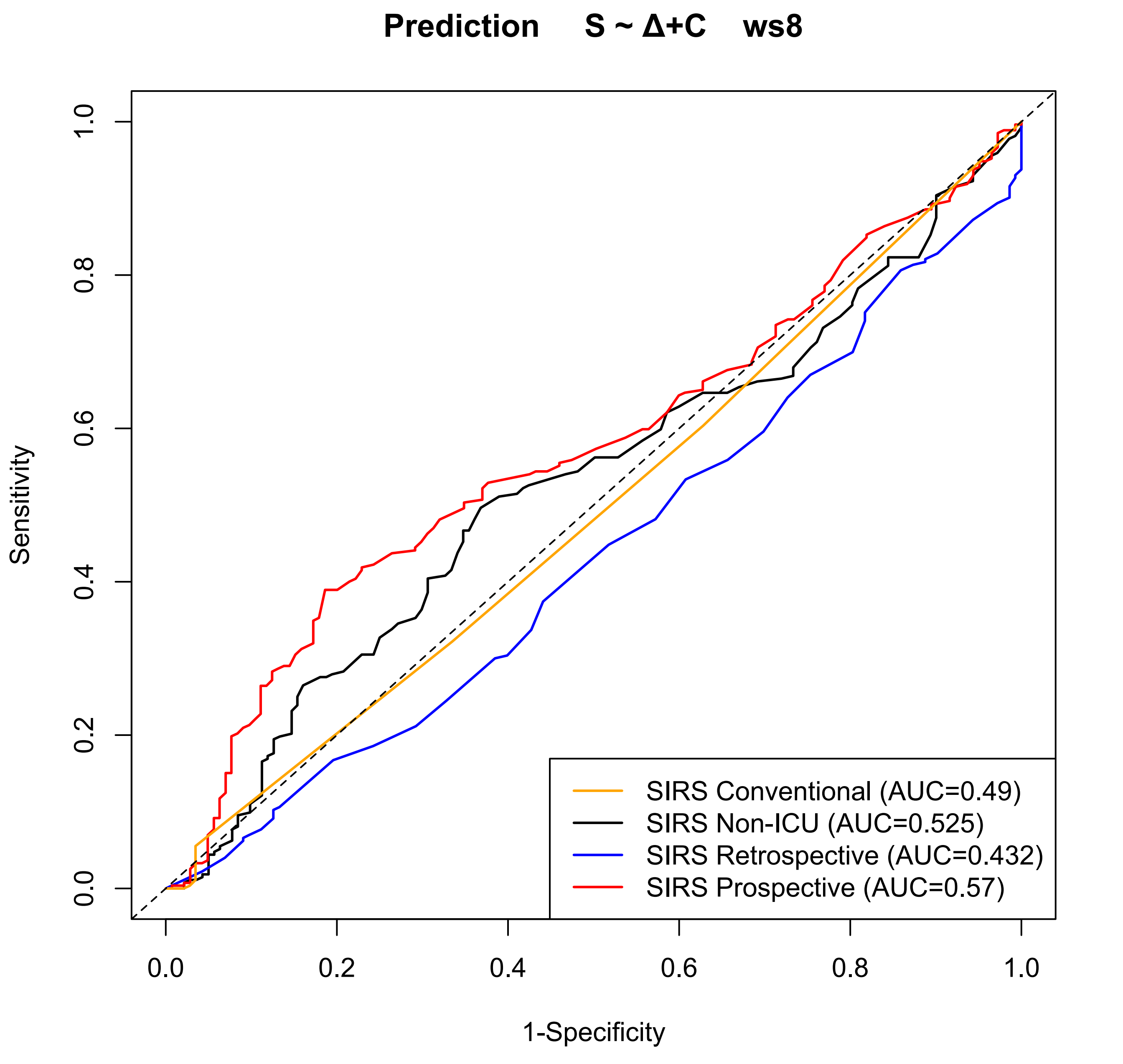
Prediction S ~ Δ+C ws8
Sensitivity
1-Specificity
0.0
0.2
0.4
0.6
0.8
1.0
SIRS Conventional (AUC=0.49)
SIRS Non-ICU (AUC=0.525)
SIRS Retrospective (AUC=0.432)
SIRS Prospective (AUC=0.57)

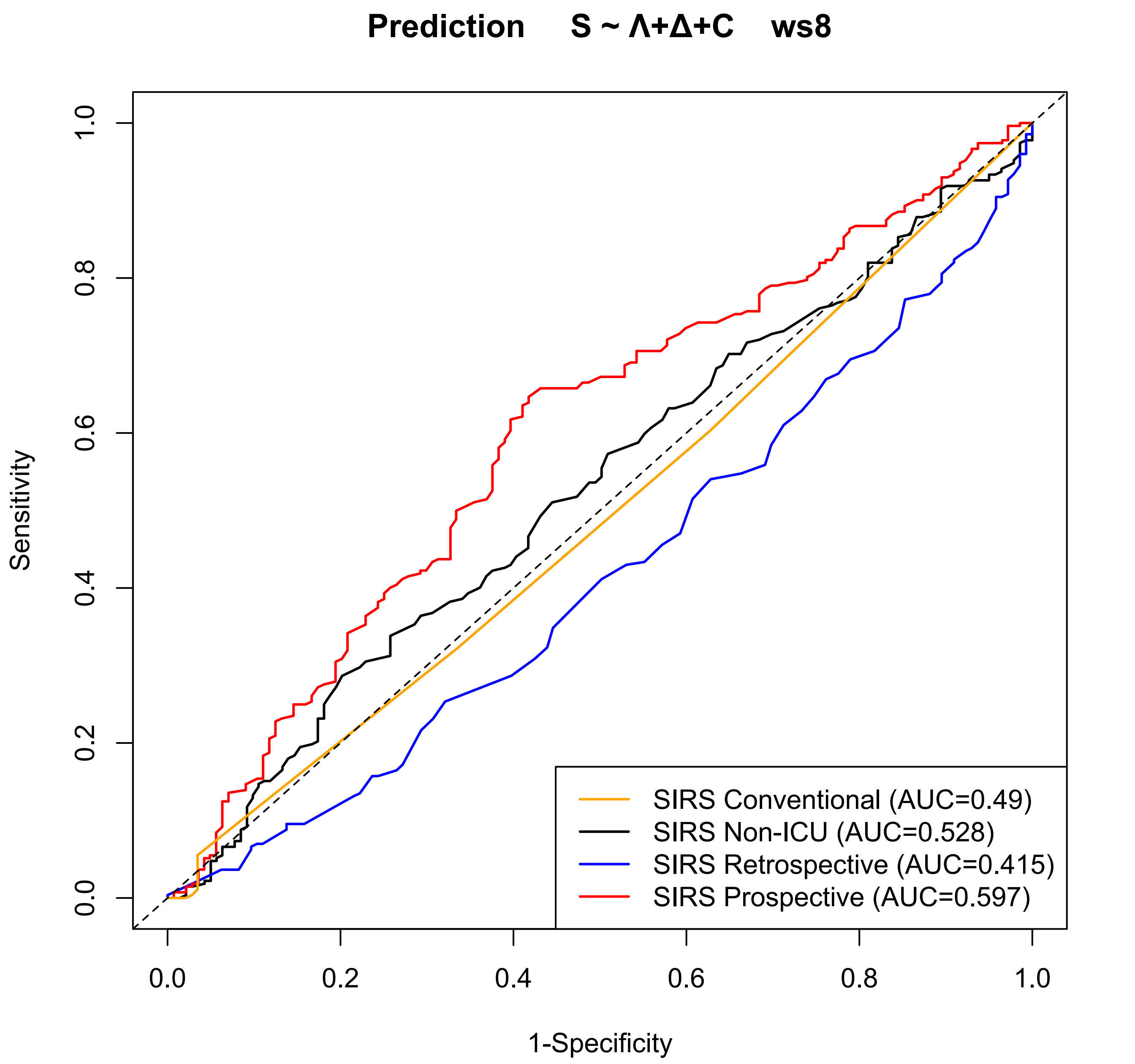
Prediction S ~ Λ+Δ+C ws8
Sensitivity
1-Specificity
0.0
0.2
0.4
0.6
0.8
1.0
SIRS Conventional (AUC=0.49)
SIRS Non-ICU (AUC=0.528)
SIRS Retrospective (AUC=0.415)
SIRS Prospective (AUC=0.597)

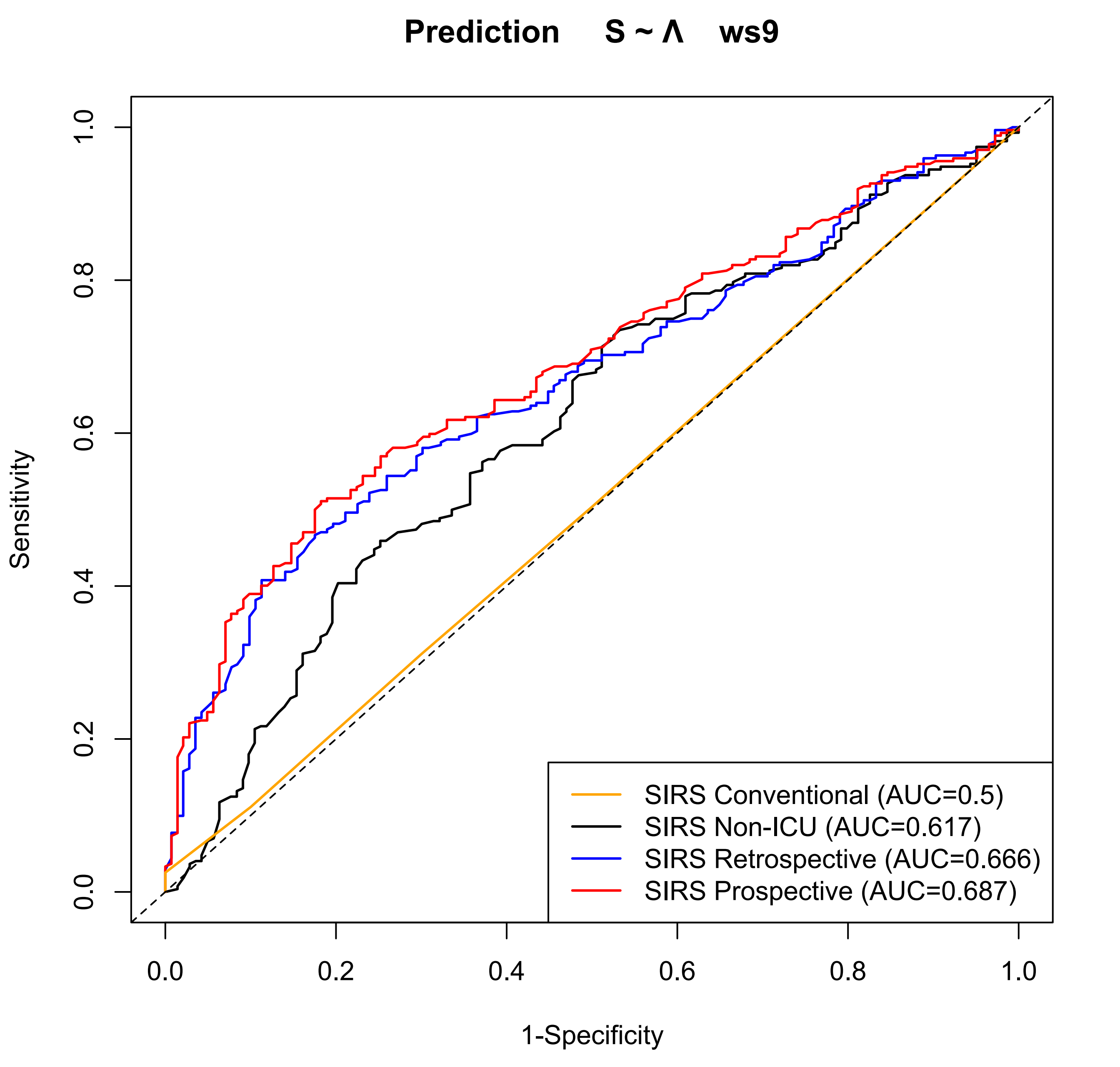
Prediction S ~ Λ ws9
Sensitivity
1-Specificity
0.0
0.2
0.4
0.6
0.8
1.0
SIRS Conventional (AUC=0.5)
SIRS Non-ICU (AUC=0.617)
SIRS Retrospective (AUC=0.666)
SIRS Prospective (AUC=0.687)

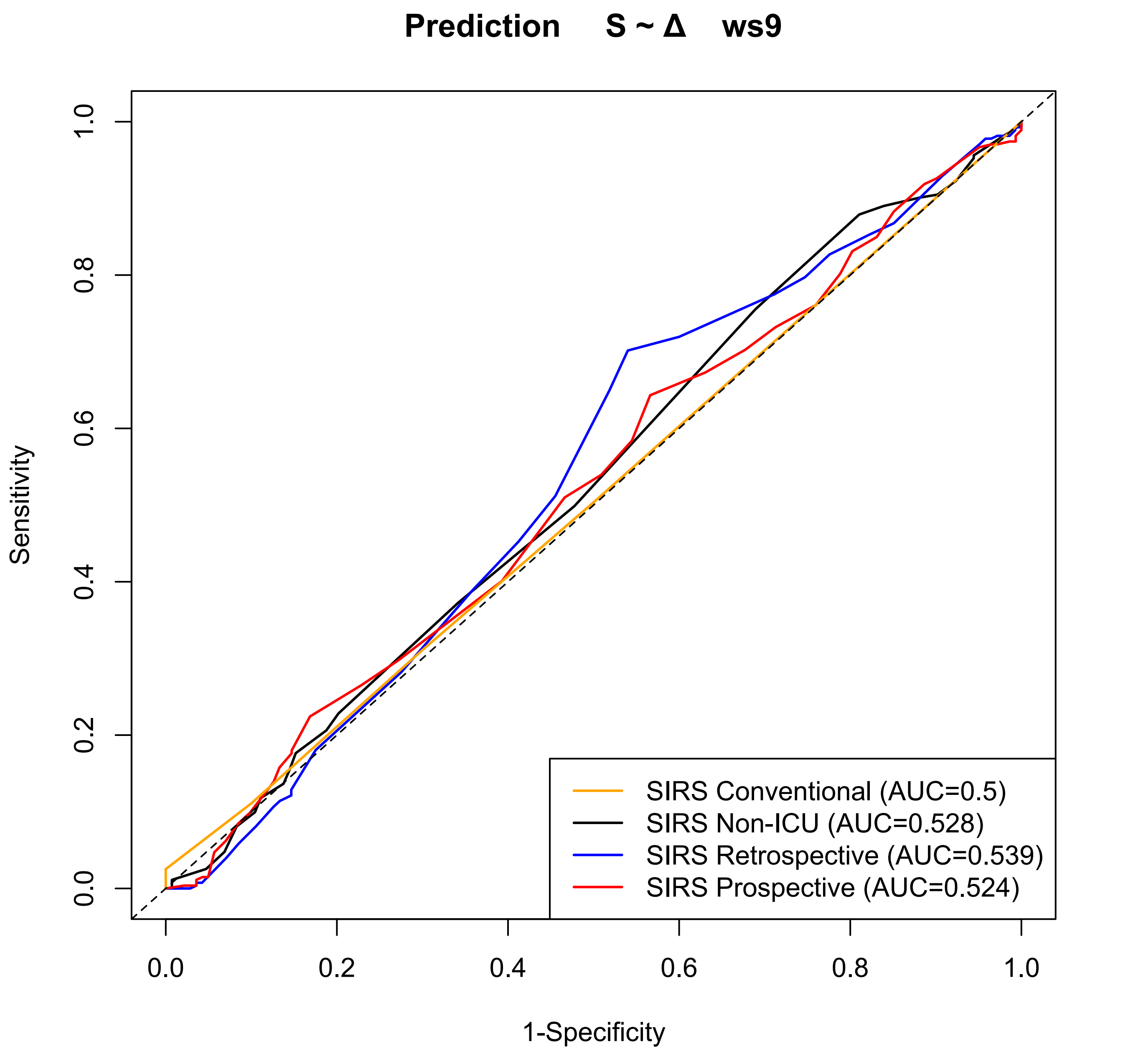

Prediction S ~ Δ ws9
Sensitivity
1-Specificity
0.0
0.2
0.4
0.6
0.8
1.0
SIRS Conventional (AUC=0.5)
SIRS Non-ICU (AUC=0.528)
SIRS Retrospective (AUC=0.539)
SIRS Prospective (AUC=0.524)

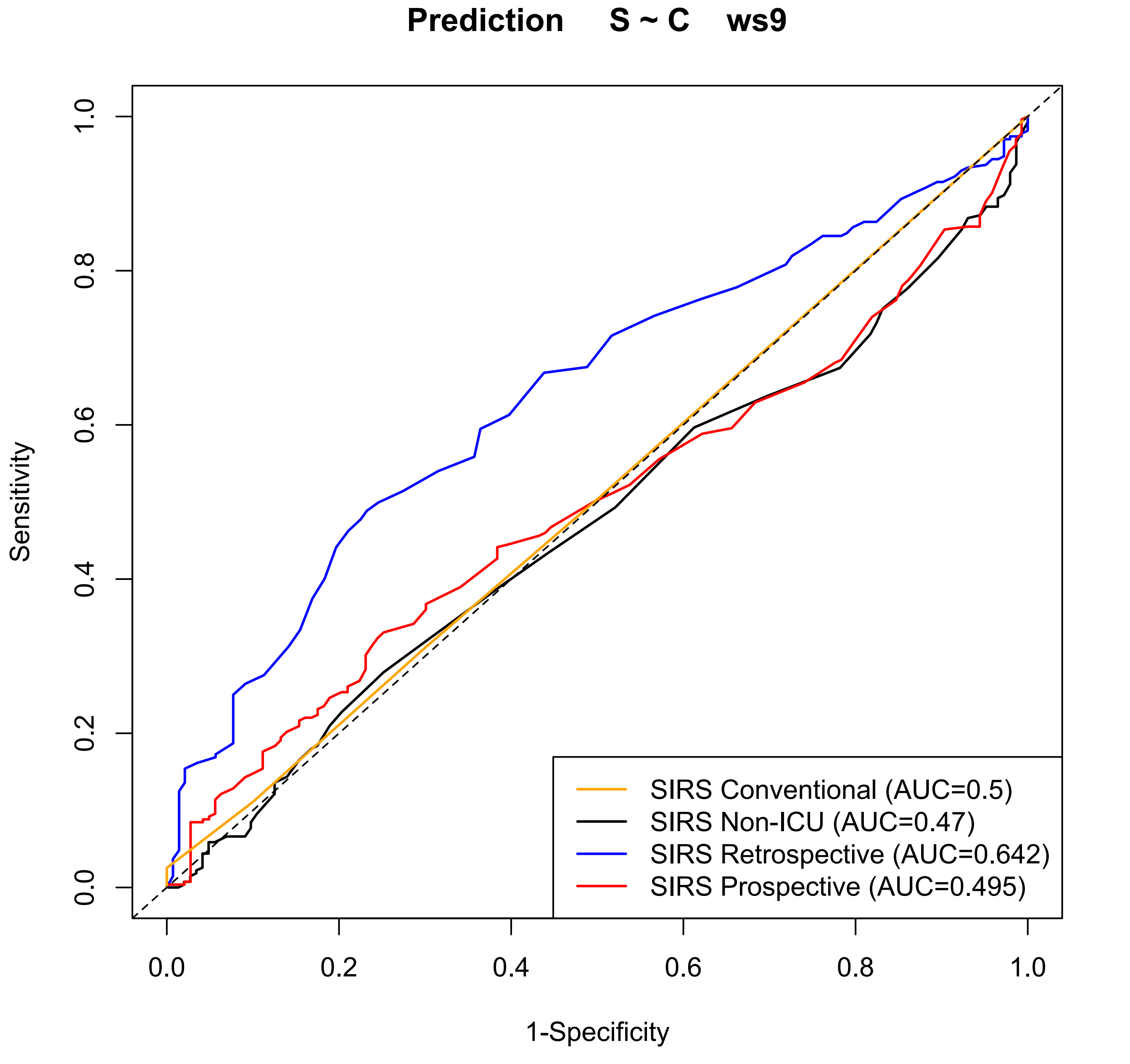
Prediction S ~ C ws9
Sensitivity
1-Specificity
0.0
0.2
0.4
0.6
0.8
1.0
SIRS Conventional (AUC=0.5)
SIRS Non-ICU (AUC=0.47)
SIRS Retrospective (AUC=0.642)
SIRS Prospective (AUC=0.495)

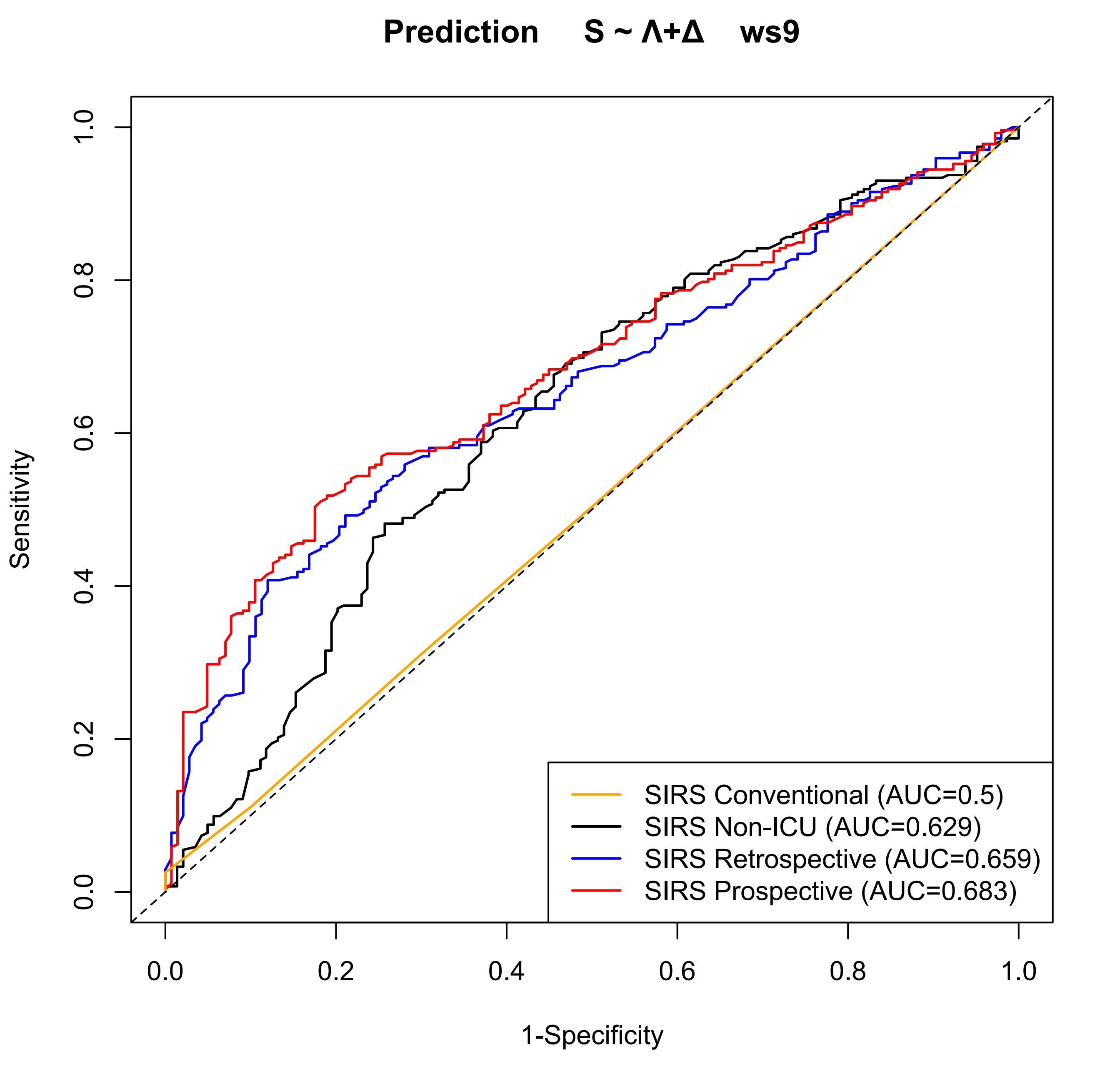

Prediction S ~ Λ+Δ ws9
Sensitivity
1-Specificity
0.0
0.2
0.4
0.6
0.8
1.0
SIRS Conventional (AUC=0.5)
SIRS Non-ICU (AUC=0.629)
SIRS Retrospective (AUC=0.659)
SIRS Prospective (AUC=0.683)

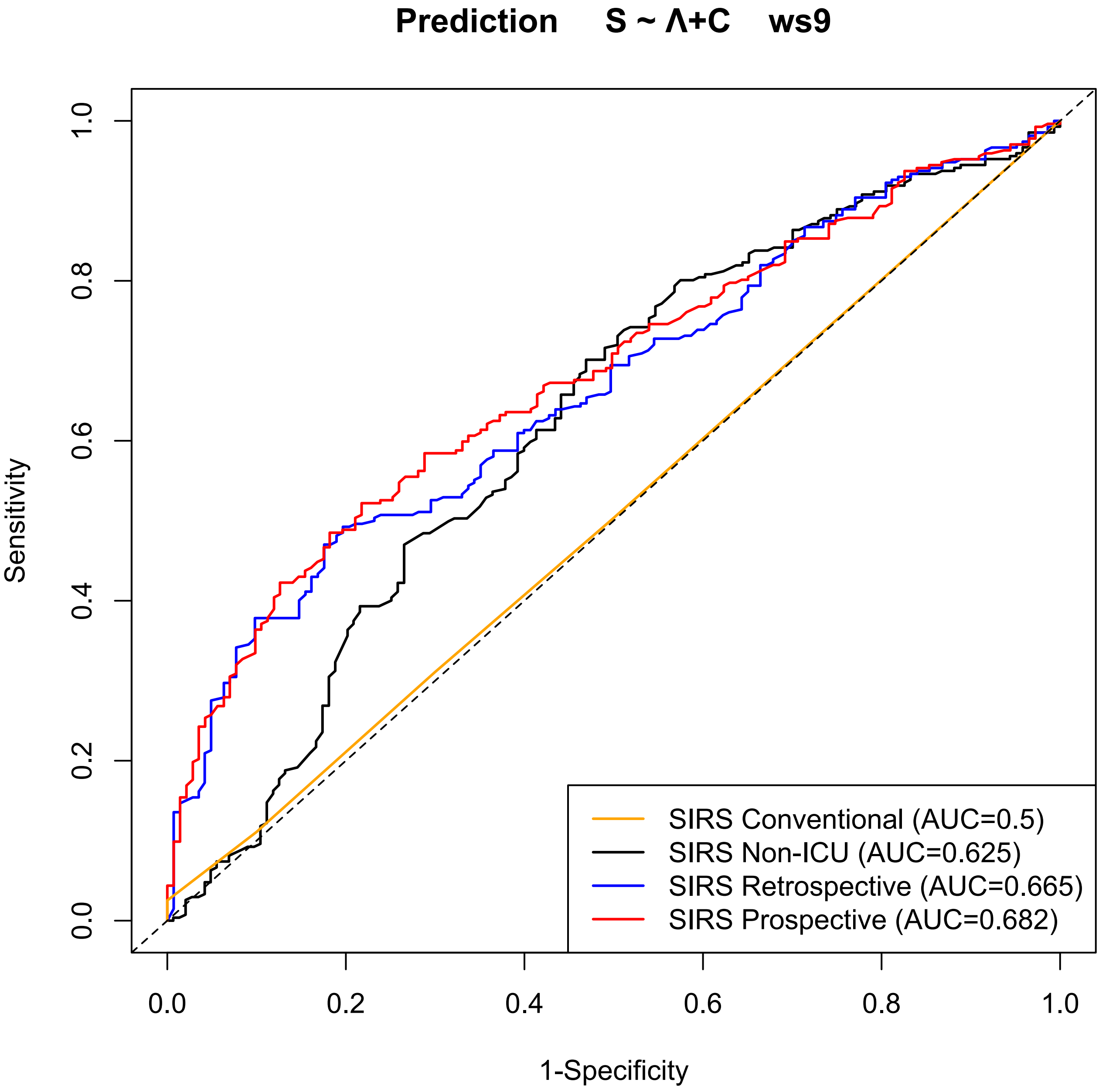
Prediction S ~ Λ+C ws9
Sensitivity
1-Specificity
0.0
0.2
0.4
0.6
0.8
1.0
SIRS Conventional (AUC=0.5)
SIRS Non-ICU (AUC=0.625)
SIRS Retrospective (AUC=0.665)
SIRS Prospective (AUC=0.682)

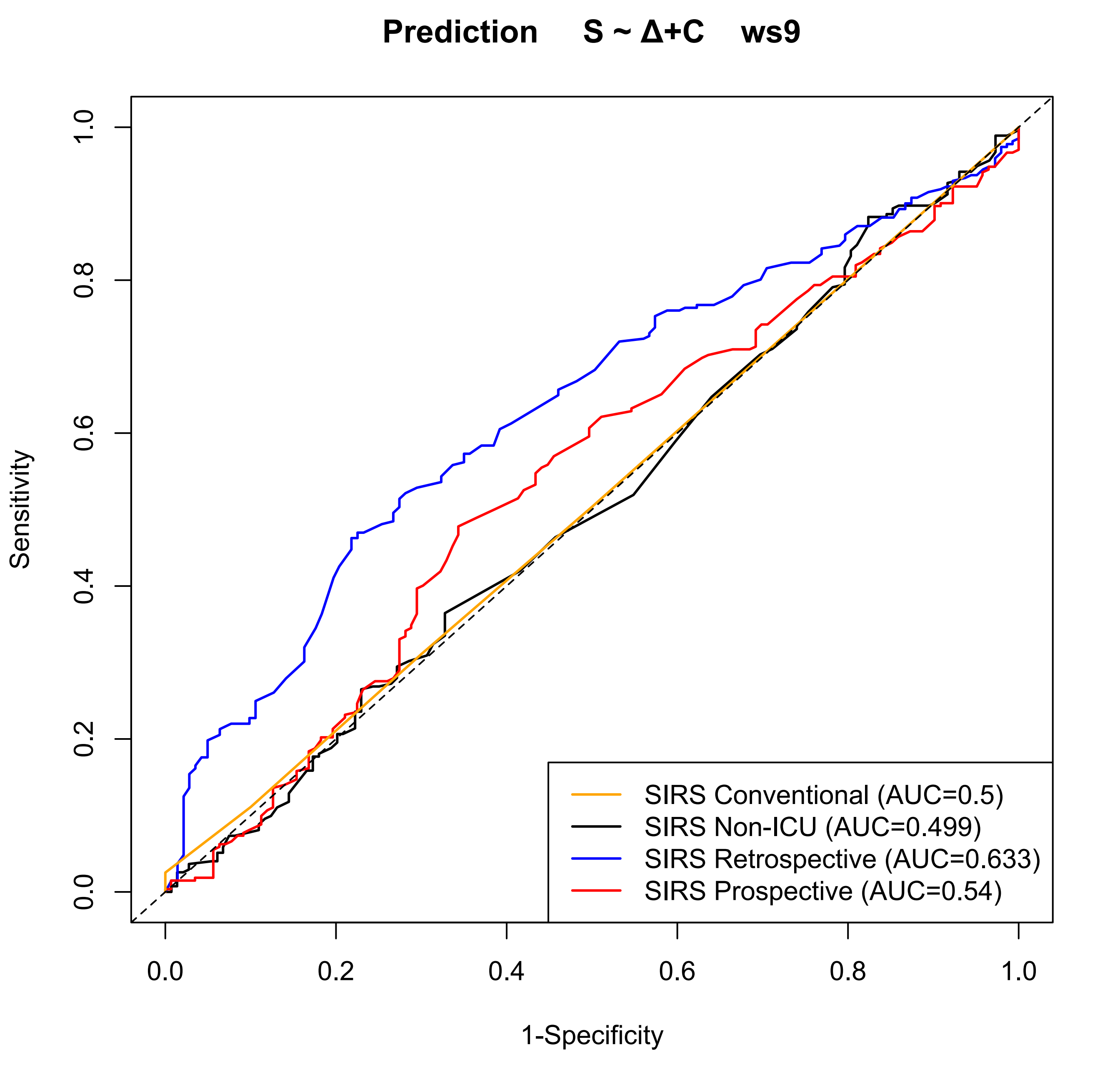

Prediction S ~ Δ+C ws9
Sensitivity
1-Specificity
0.0
0.2
0.4
0.6
0.8
1.0
SIRS Conventional (AUC=0.5)
SIRS Non-ICU (AUC=0.499)
SIRS Retrospective (AUC=0.633)
SIRS Prospective (AUC=0.54)

Prediction S ~ Λ+Δ+C ws9

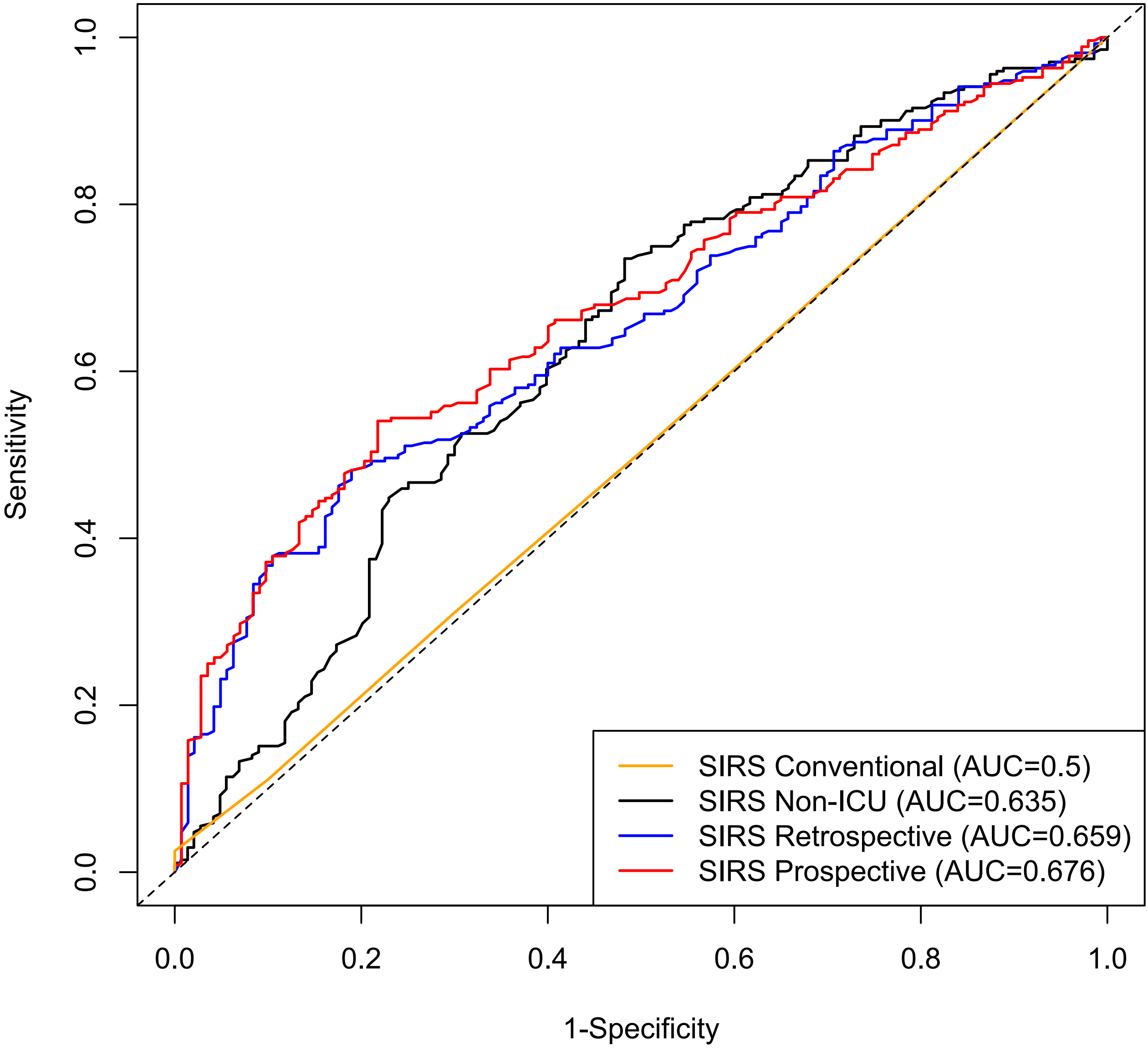

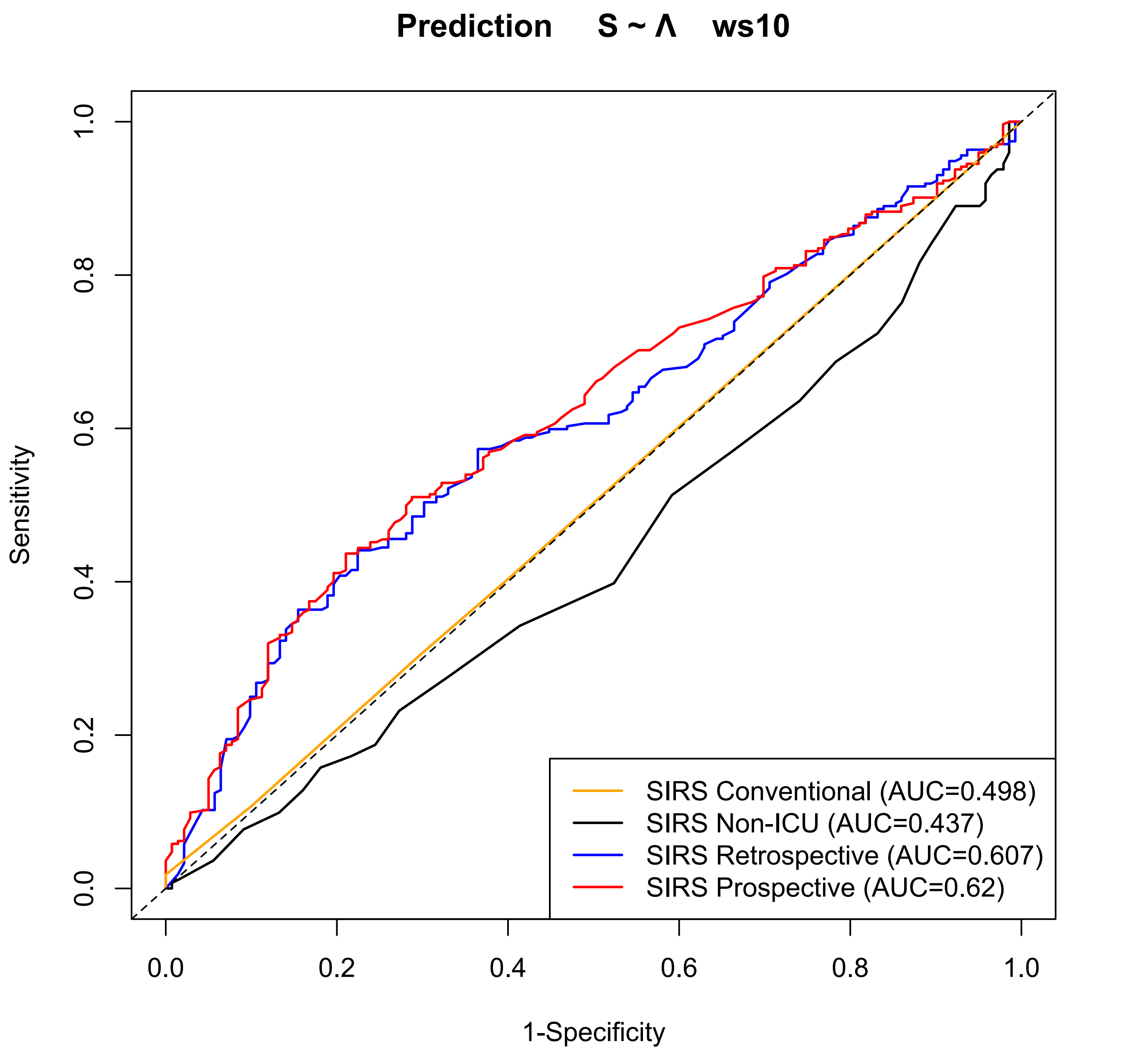

Prediction S ~ Λ ws10
Sensitivity
1-Specificity
0.0
0.2
0.4
0.6
0.8
1.0
SIRS Conventional (AUC=0.498)
SIRS Non-ICU (AUC=0.437)
SIRS Retrospective (AUC=0.607)
SIRS Prospective (AUC=0.62)

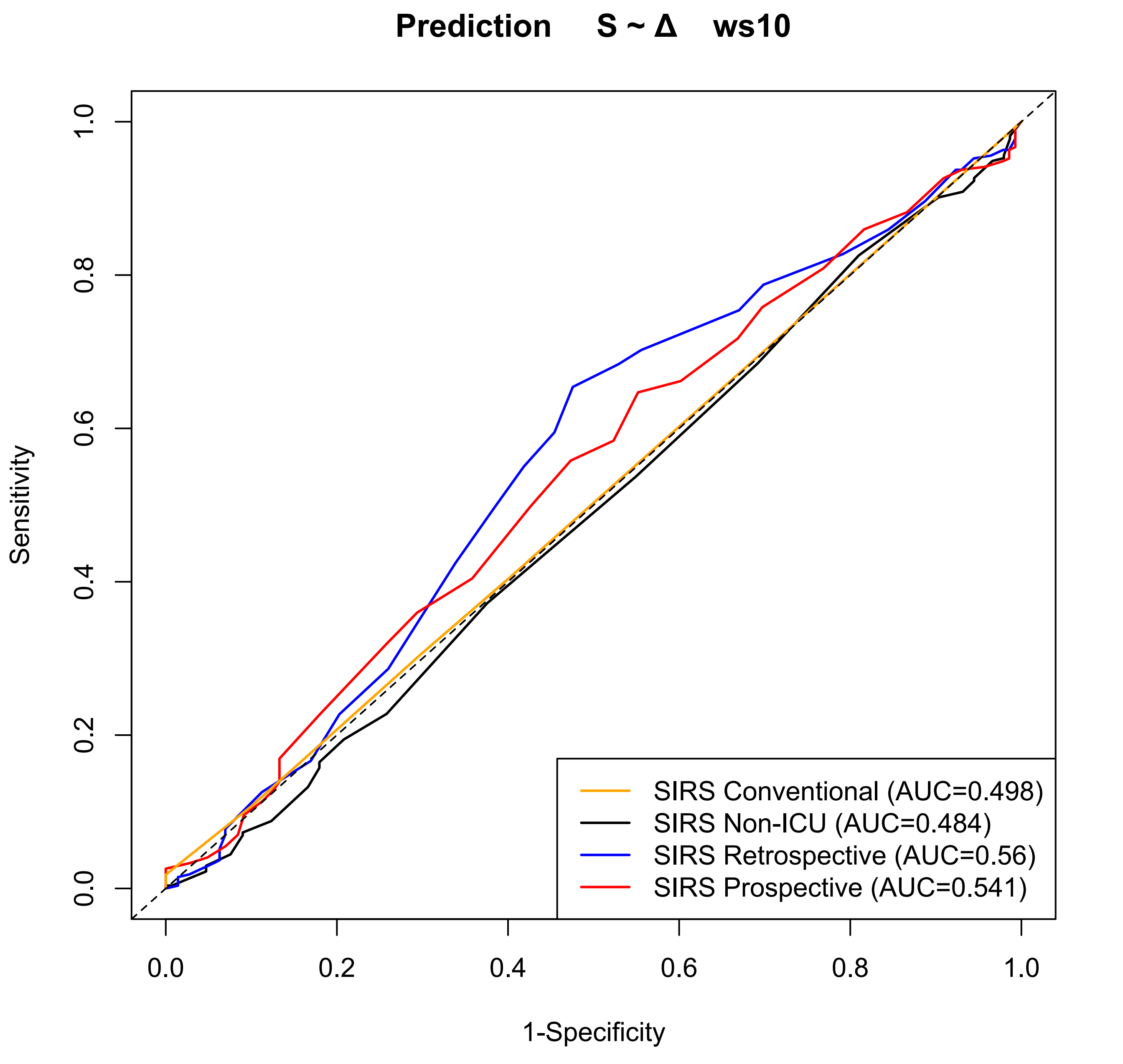

Prediction S ~ Δ ws10
Sensitivity
1-Specificity
0.0
0.2
0.4
0.6
0.8
1.0
0.0
0.2
0.4
0.6
0.8
1.0
SIRS Conventional (AUC=0.498)
SIRS Non-ICU (AUC=0.484)
SIRS Retrospective (AUC=0.56)
SIRS Prospective (AUC=0.541)

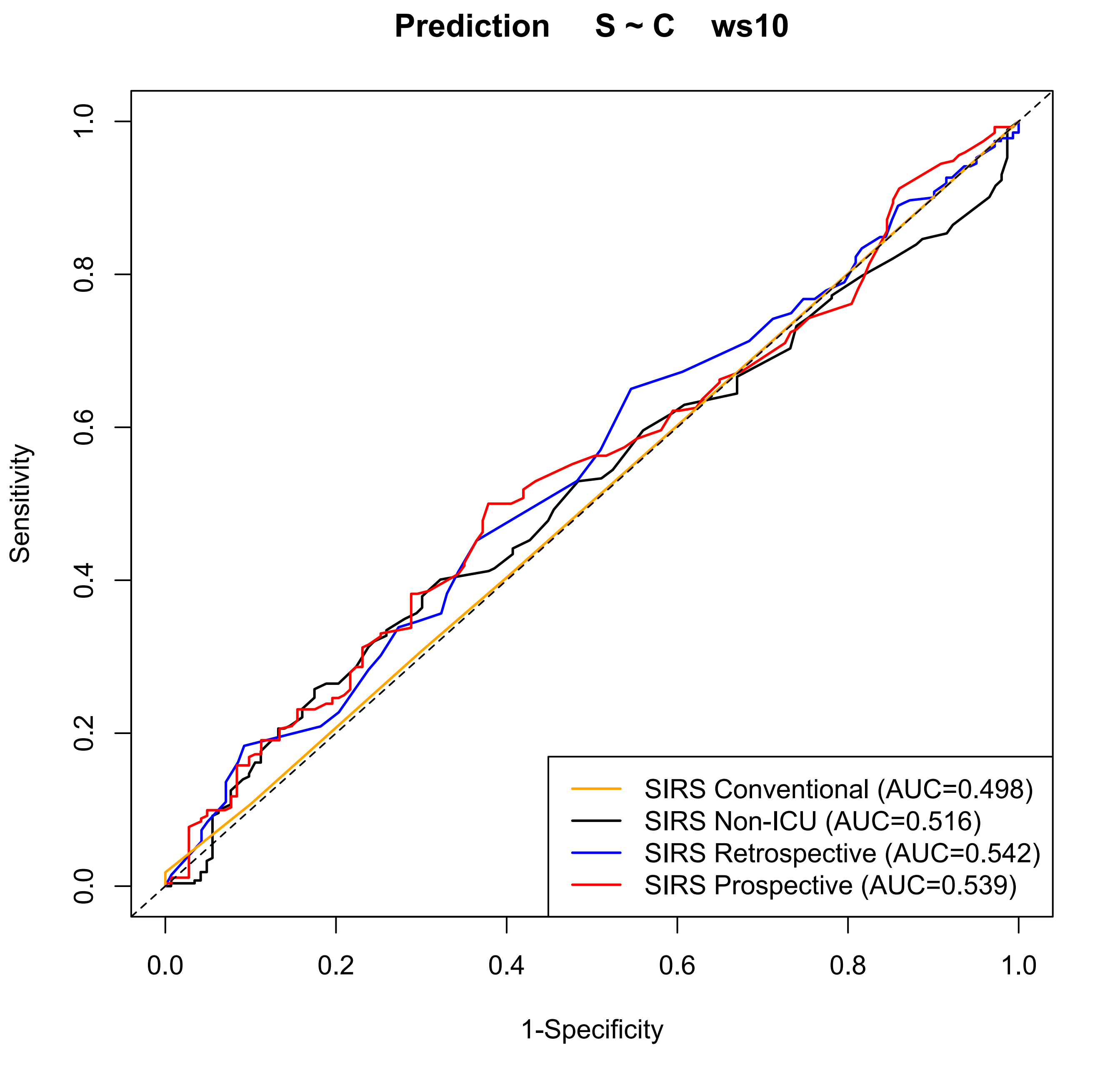

Prediction S ~ C ws10
Sensitivity
1-Specificity
0.0
0.2
0.4
0.6
0.8
1.0
SIRS Conventional (AUC=0.498)
SIRS Non-ICU (AUC=0.516)
SIRS Retrospective (AUC=0.542)
SIRS Prospective (AUC=0.539)

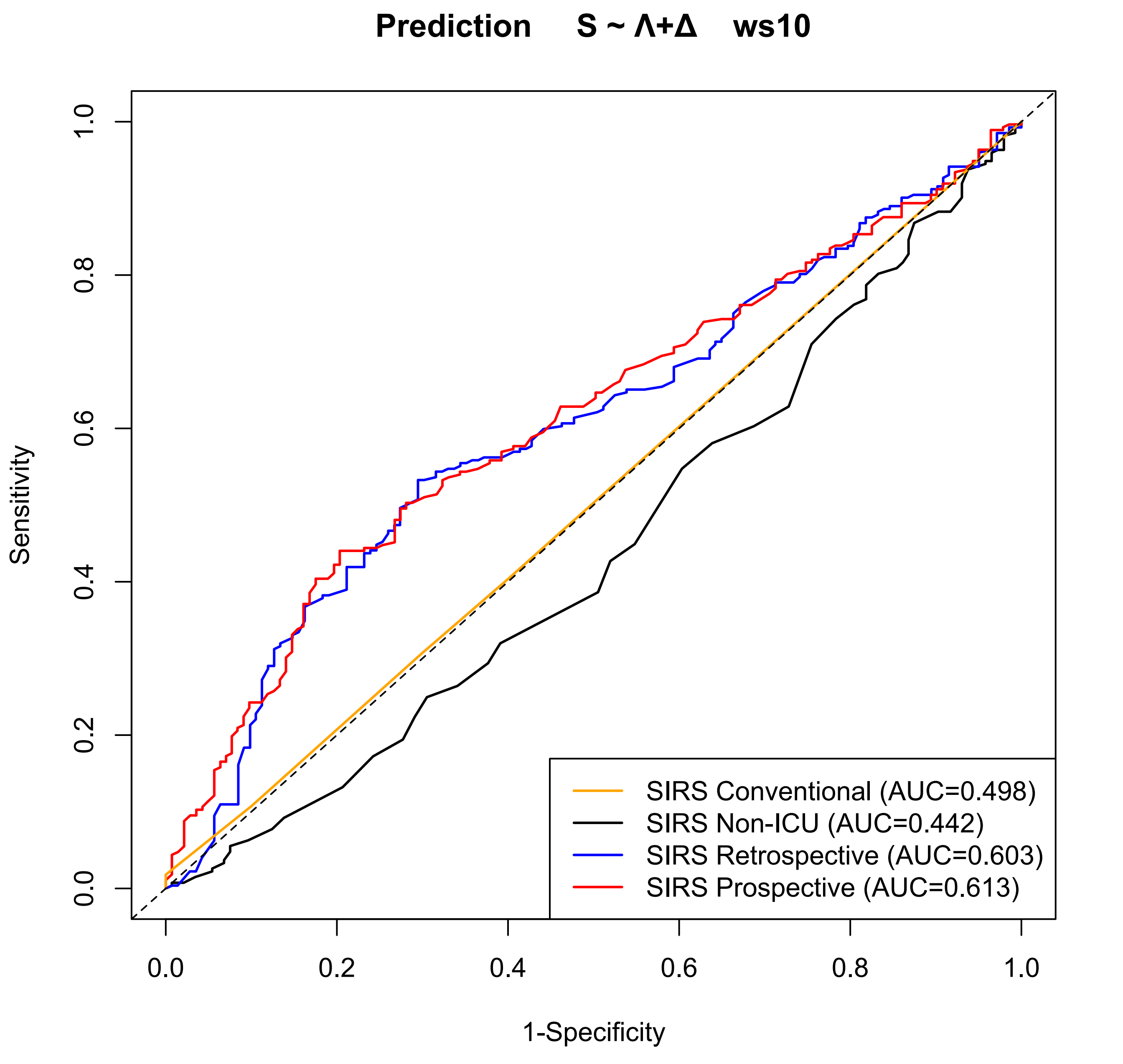
Prediction S ~ Λ+Δ ws10
Sensitivity
1-Specificity
0.0
0.2
0.4
0.6
0.8
1.0
SIRS Conventional (AUC=0.498)
SIRS Non-ICU (AUC=0.442)
SIRS Retrospective (AUC=0.603)
SIRS Prospective (AUC=0.613)

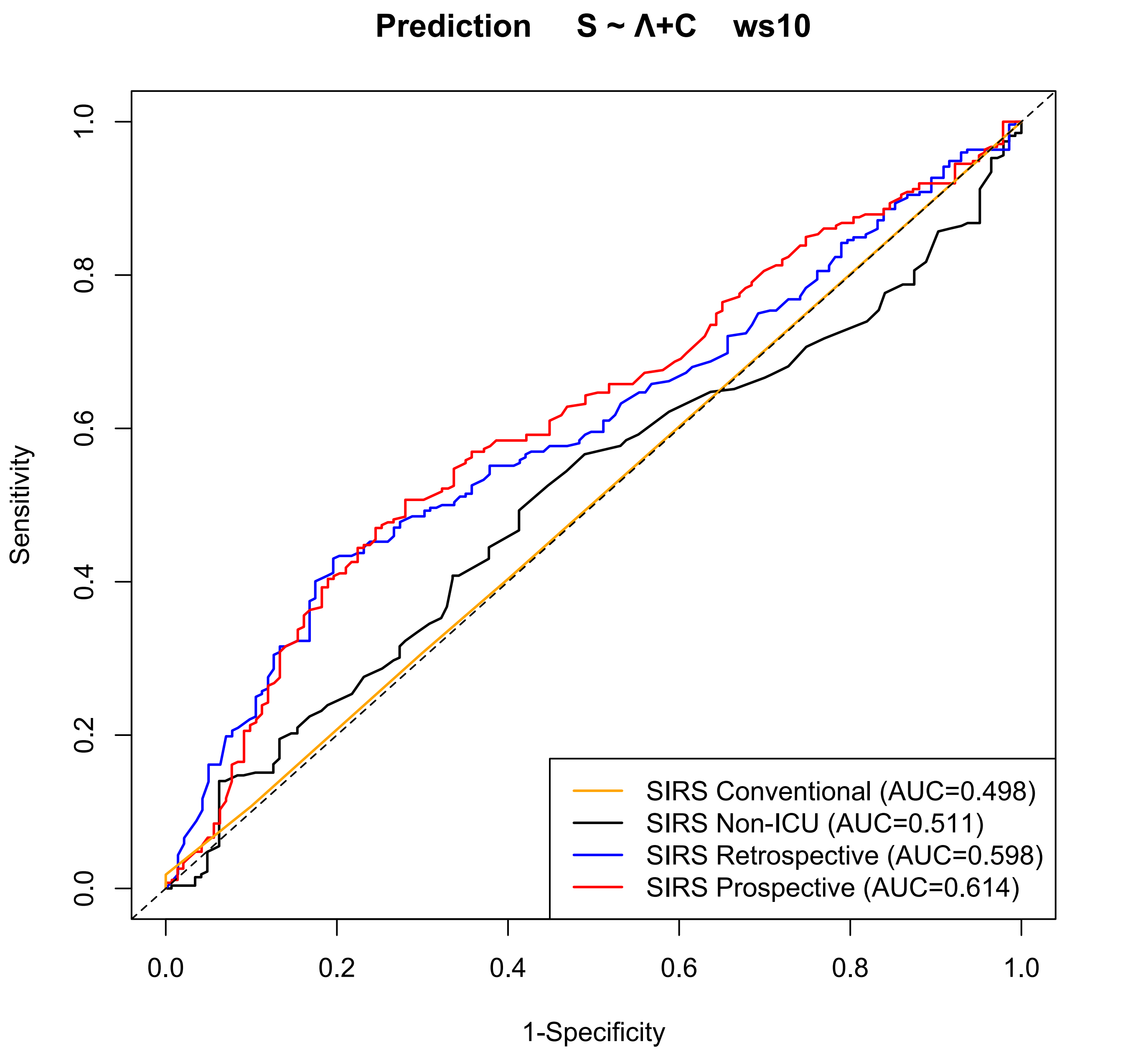

Prediction S ~ Λ+C ws10
Sensitivity
1-Specificity
0.0
0.2
0.4
0.6
0.8
1.0
0.0
0.2
0.4
0.6
0.8
1.0
SIRS Conventional (AUC=0.498)
SIRS Non-ICU (AUC=0.511)
SIRS Retrospective (AUC=0.598)
SIRS Prospective (AUC=0.614)

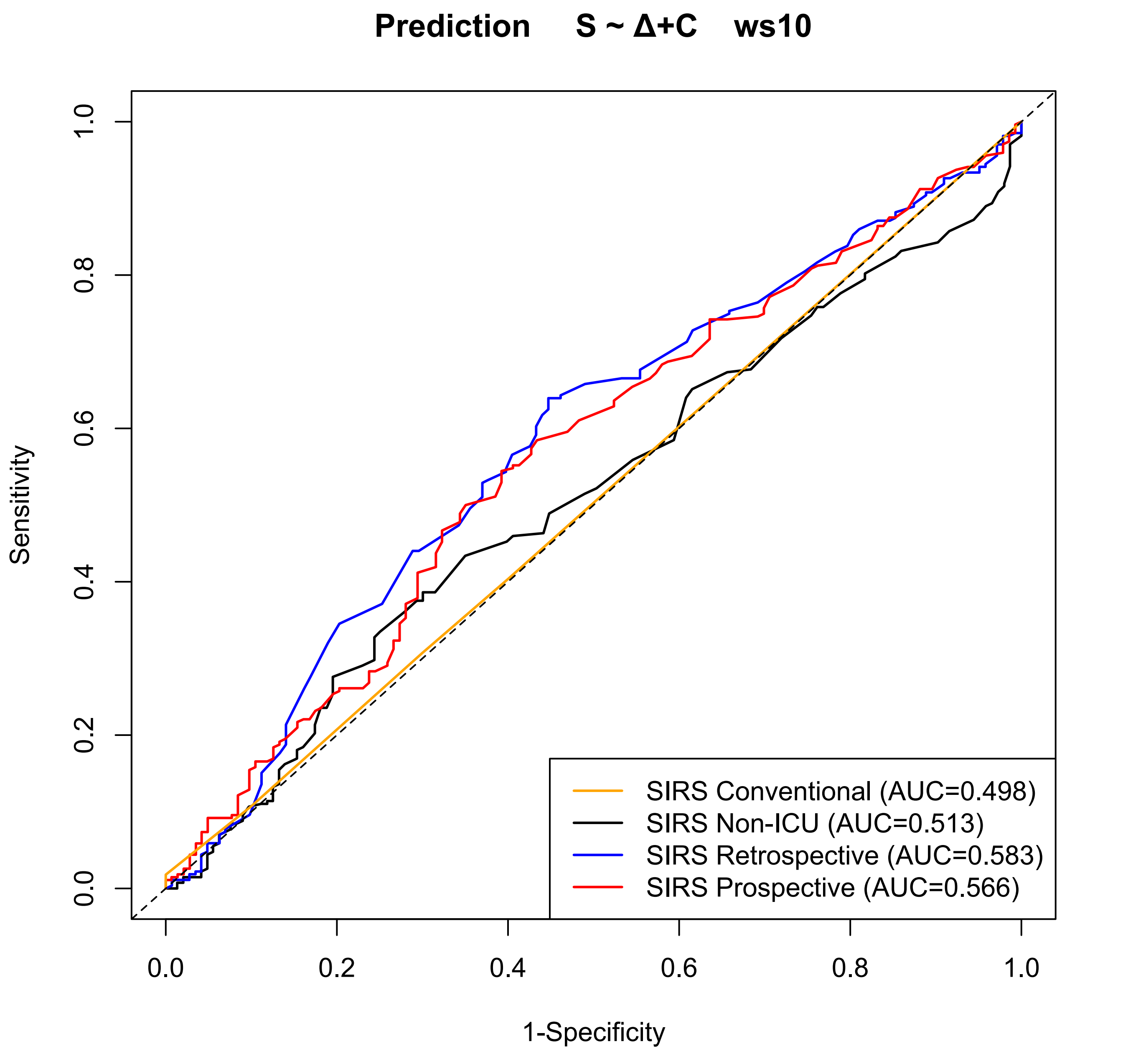

Prediction S ~ Δ+C ws10
Sensitivity
1-Specificity
0.0
0.2
0.4
0.6
0.8
1.0
SIRS Conventional (AUC=0.498)
SIRS Non-ICU (AUC=0.513)
SIRS Retrospective (AUC=0.583)
SIRS Prospective (AUC=0.566)

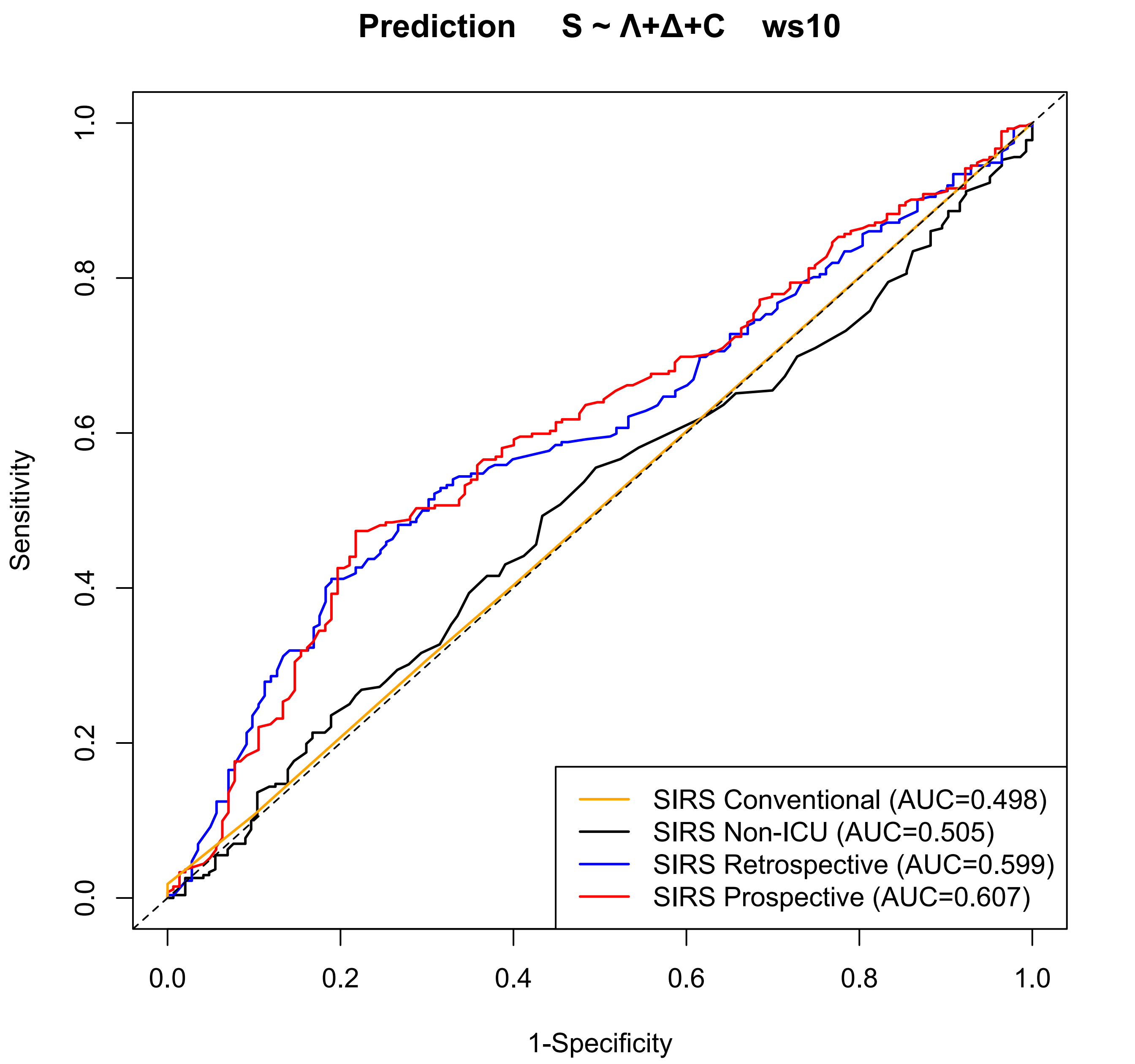
Prediction S ~ Λ+Δ+C ws10
Sensitivity
1-Specificity
0.0
0.2
0.4
0.6
0.8
1.0
SIRS Conventional (AUC=0.498)
SIRS Non-ICU (AUC=0.505)
SIRS Retrospective (AUC=0.599)
SIRS Prospective (AUC=0.607)

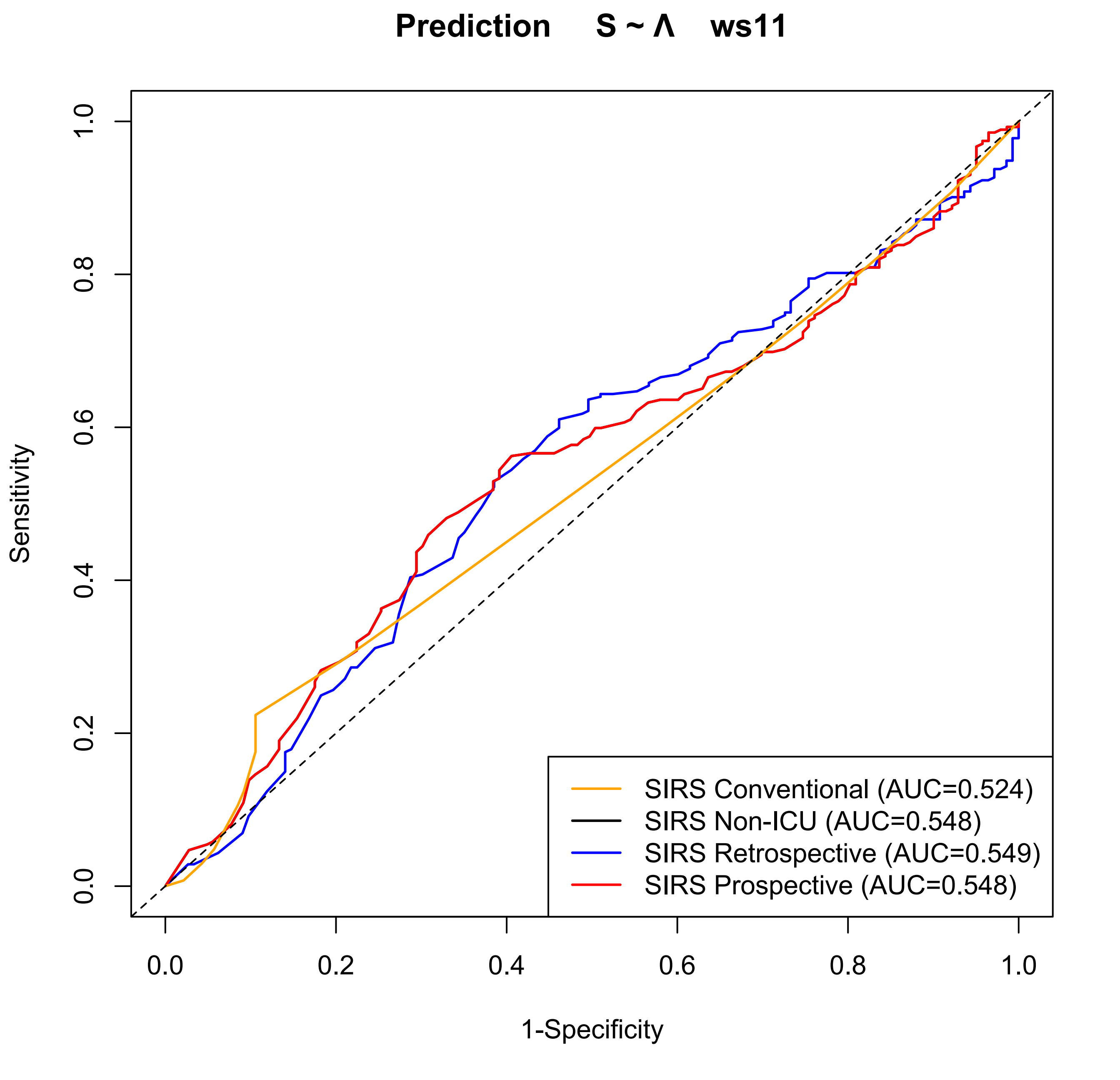
Prediction S ~ Λ ws11
Sensitivity
1-Specificity
0.0
0.2
0.4
0.6
0.8
1.0
SIRS Conventional (AUC=0.524)
SIRS Non-ICU (AUC=0.548)
SIRS Retrospective (AUC=0.549)
SIRS Prospective (AUC=0.548)

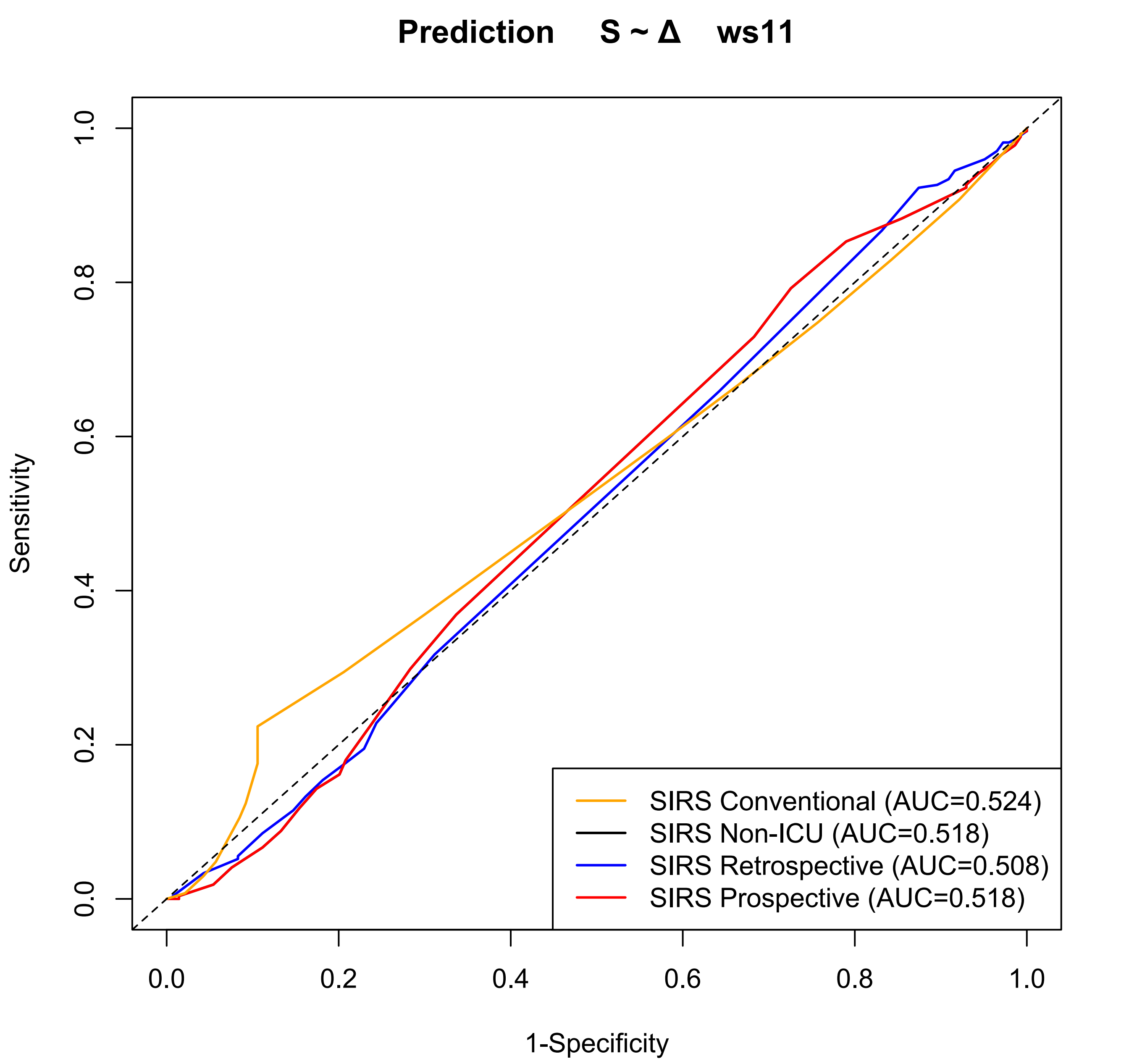
Prediction S ~ Δ ws11
Sensitivity
1-Specificity
0.0
0.2
0.4
0.6
0.8
1.0
SIRS Conventional (AUC=0.524)
SIRS Non-ICU (AUC=0.518)
SIRS Retrospective (AUC=0.508)
SIRS Prospective (AUC=0.518)

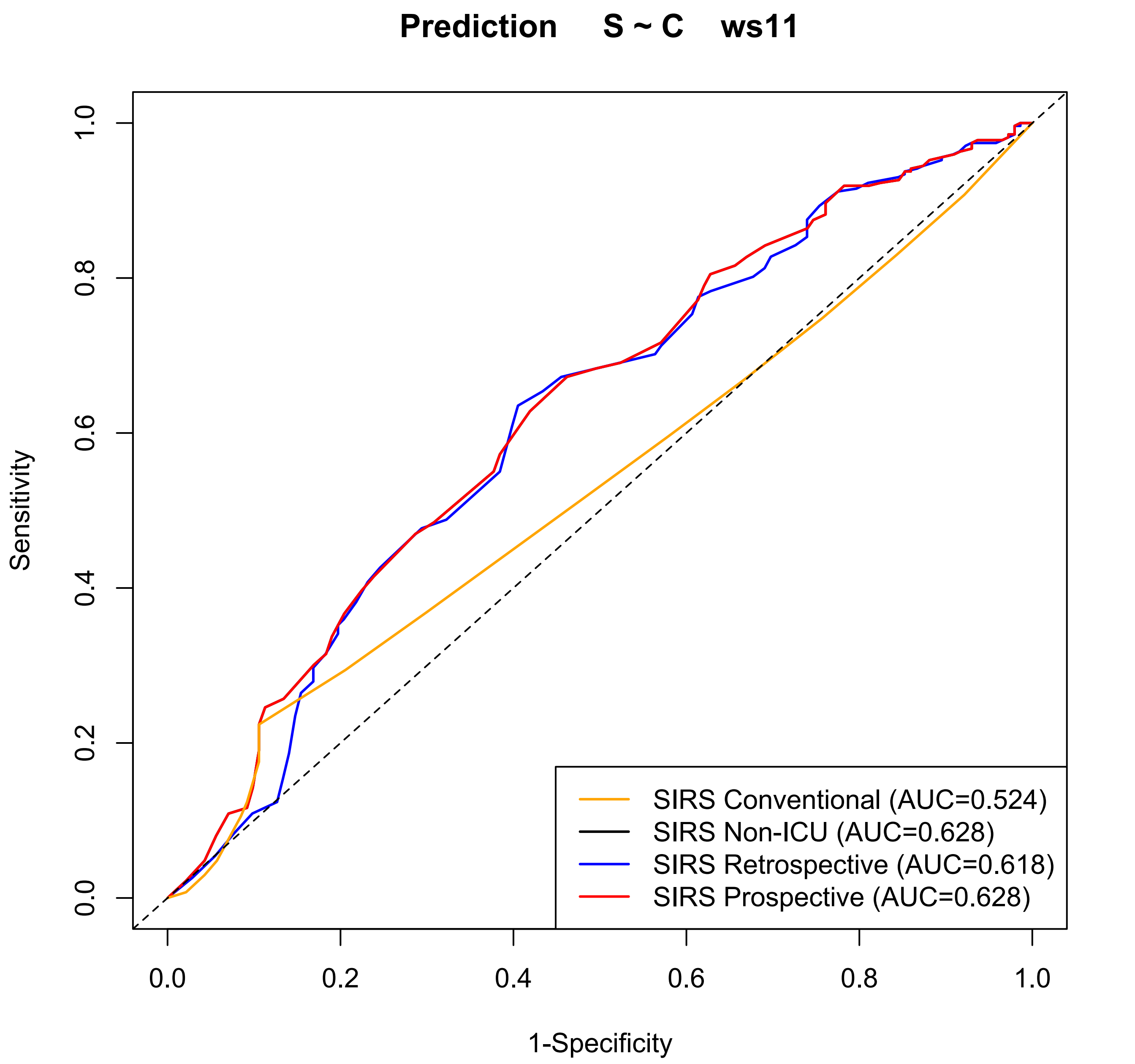

Prediction S ~ C ws11
Sensitivity
1-Specificity
0.0
0.2
0.4
0.6
0.8
1.0
SIRS Conventional (AUC=0.524)
SIRS Non-ICU (AUC=0.628)
SIRS Retrospective (AUC=0.618)
SIRS Prospective (AUC=0.628)

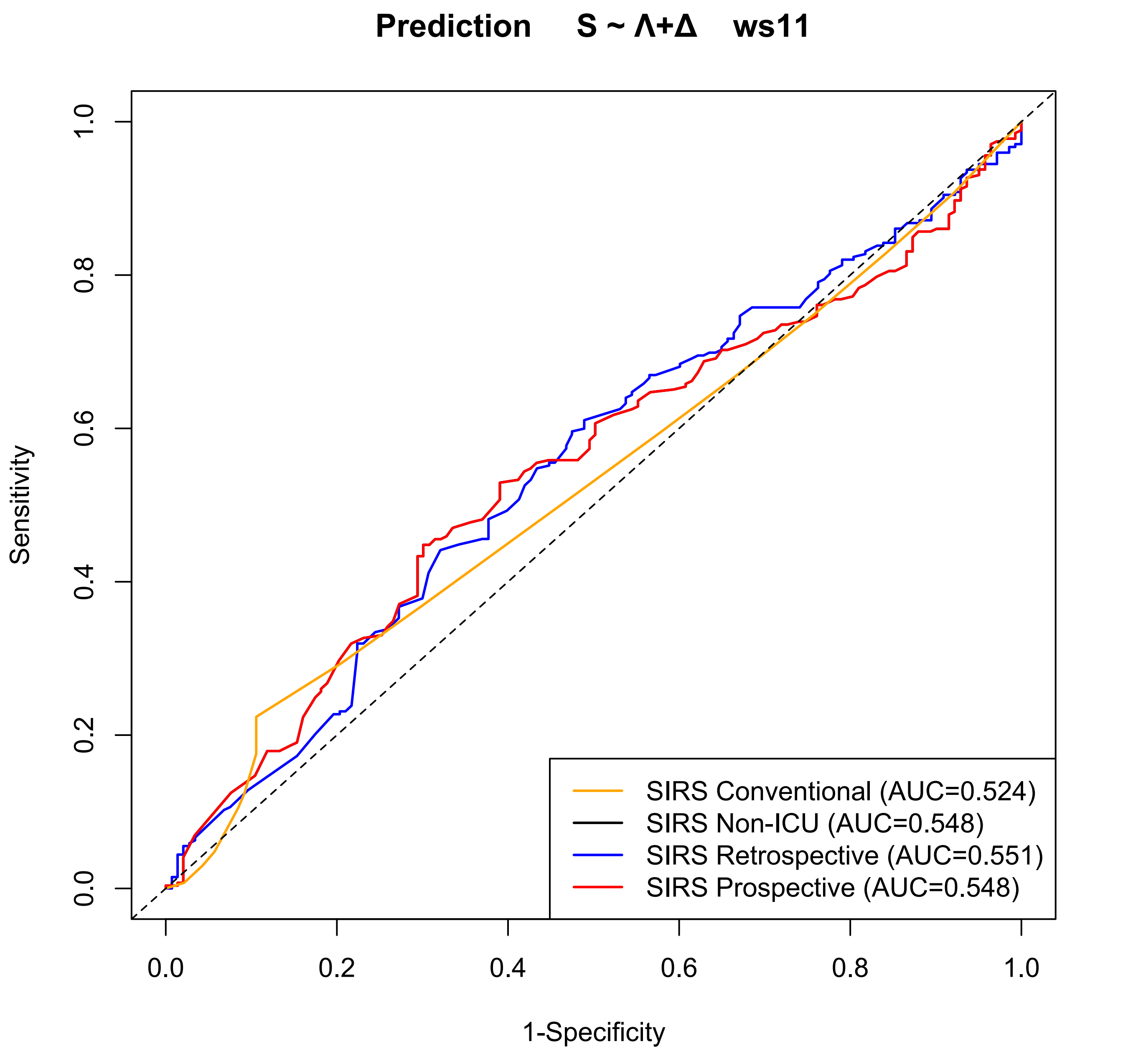

Prediction S ~ Λ+Δ ws11
Sensitivity
1-Specificity
0.0
0.2
0.4
0.6
0.8
1.0
SIRS Conventional (AUC=0.524)
SIRS Non-ICU (AUC=0.548)
SIRS Retrospective (AUC=0.551)
SIRS Prospective (AUC=0.548)

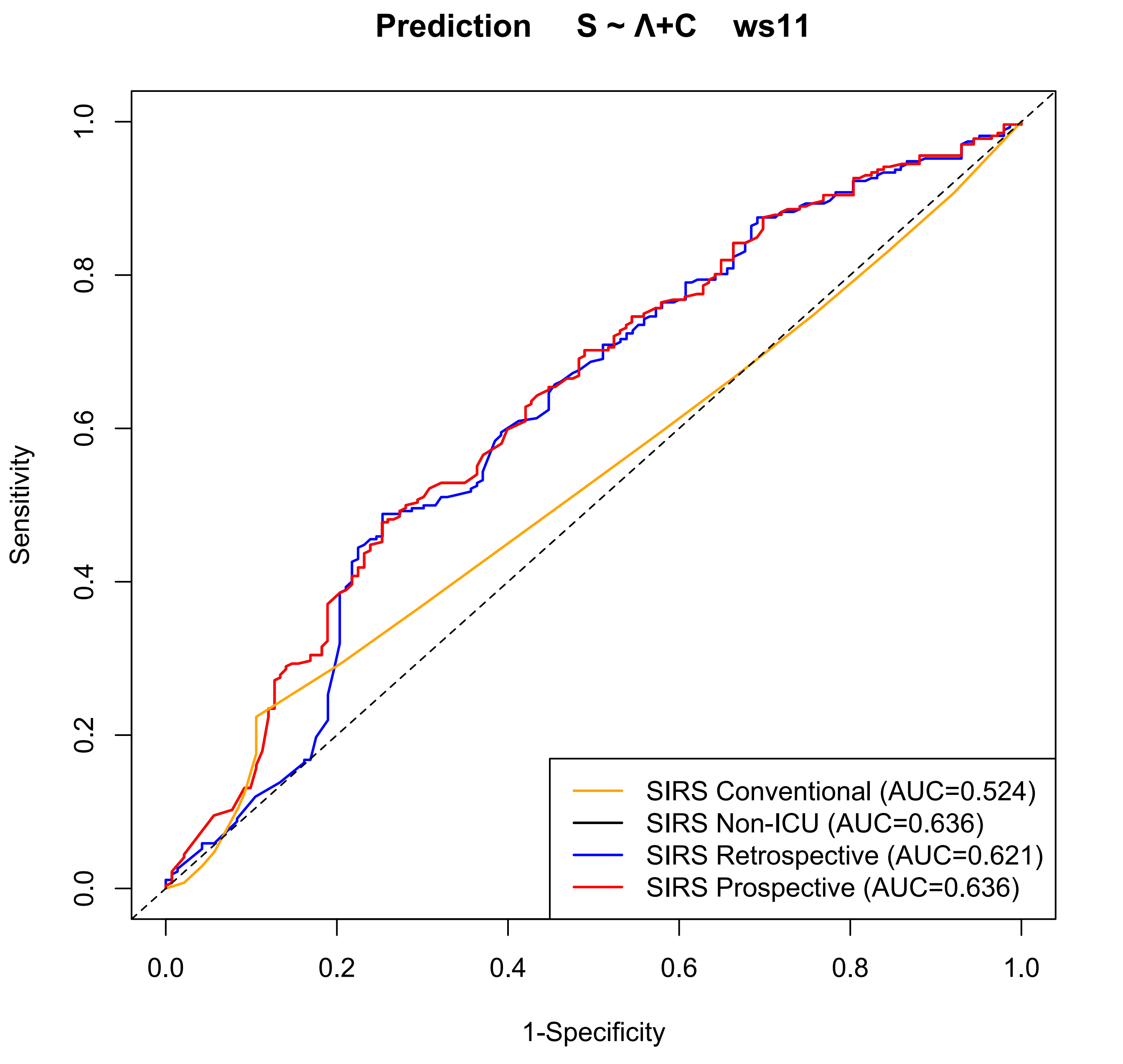

Prediction S ~ Λ+C ws11
Sensitivity
1-Specificity
0.0
0.2
0.4
0.6
0.8
1.0
SIRS Conventional (AUC=0.524)
SIRS Non-ICU (AUC=0.636)
SIRS Retrospective (AUC=0.621)
SIRS Prospective (AUC=0.636)

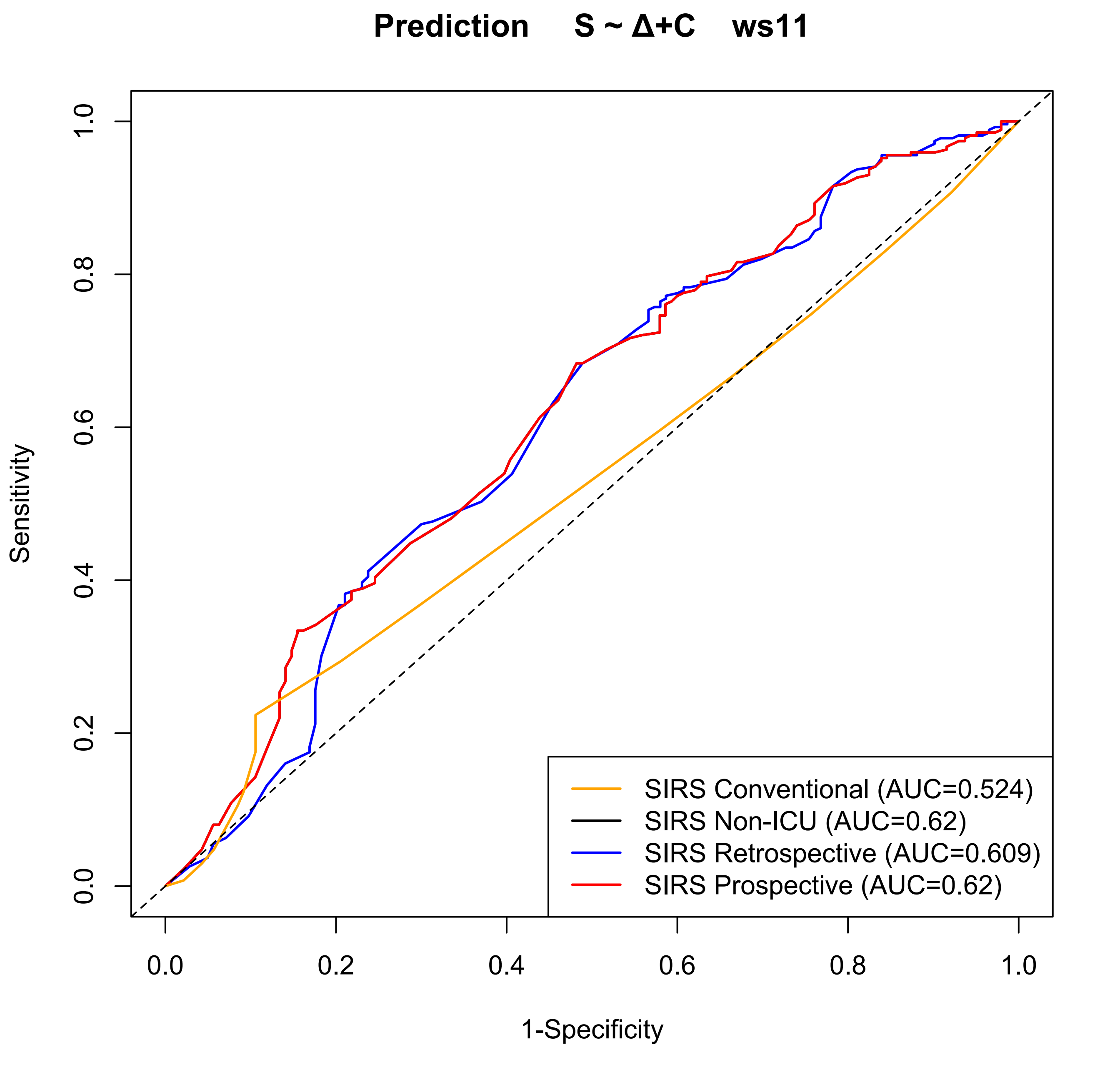
Prediction S ~ Δ+C ws11
Sensitivity
1-Specificity
0.0
0.2
0.4
0.6
0.8
1.0
SIRS Conventional (AUC=0.524)
SIRS Non-ICU (AUC=0.62)
SIRS Retrospective (AUC=0.609)
SIRS Prospective (AUC=0.62)

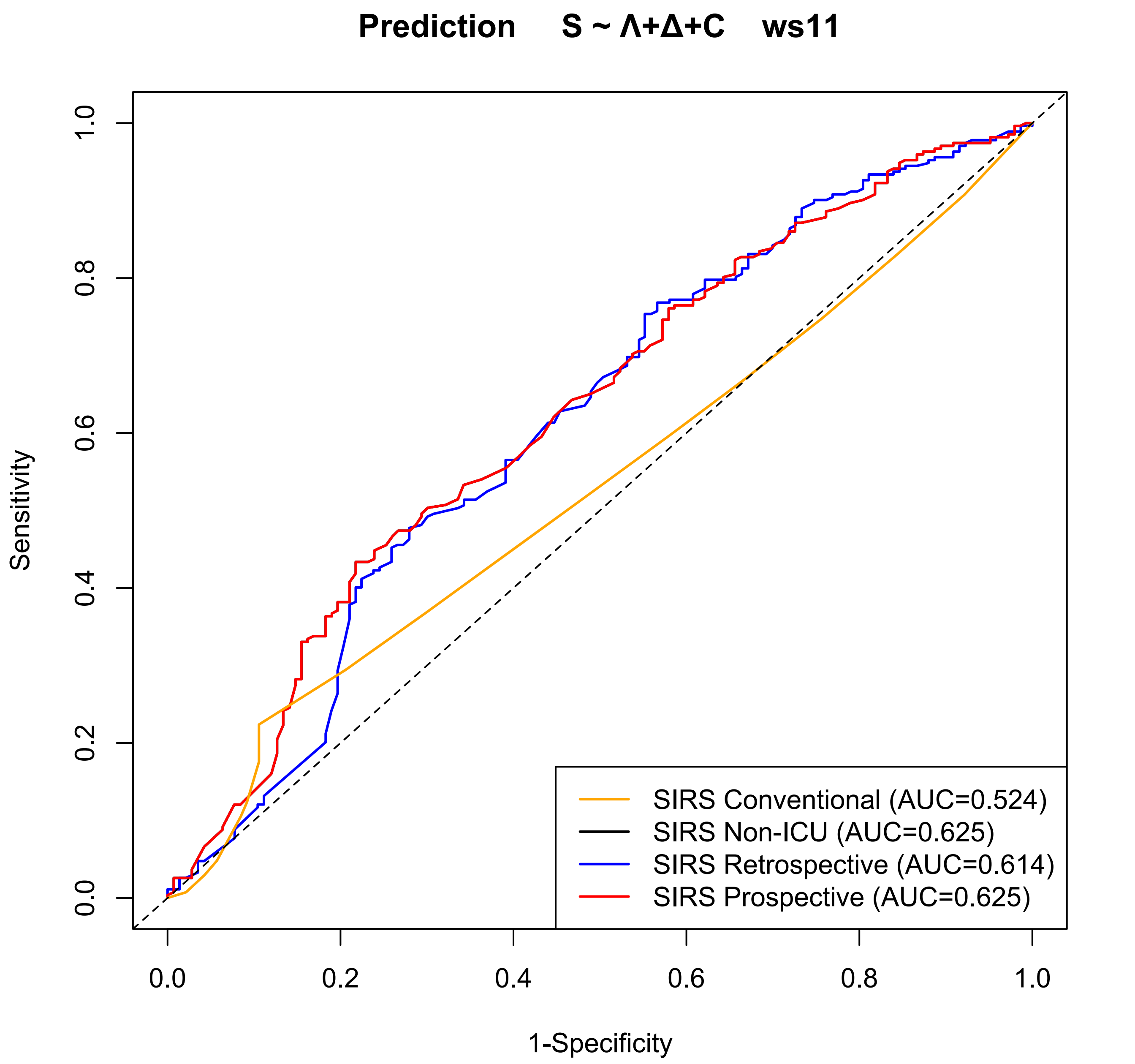

Prediction S ~ Λ+Δ+C ws11
Sensitivity
1-Specificity
0.0
0.2
0.4
0.6
0.8
1.0
SIRS Conventional (AUC=0.524)
SIRS Non-ICU (AUC=0.625)
SIRS Retrospective (AUC=0.614)
SIRS Prospective (AUC=0.625)

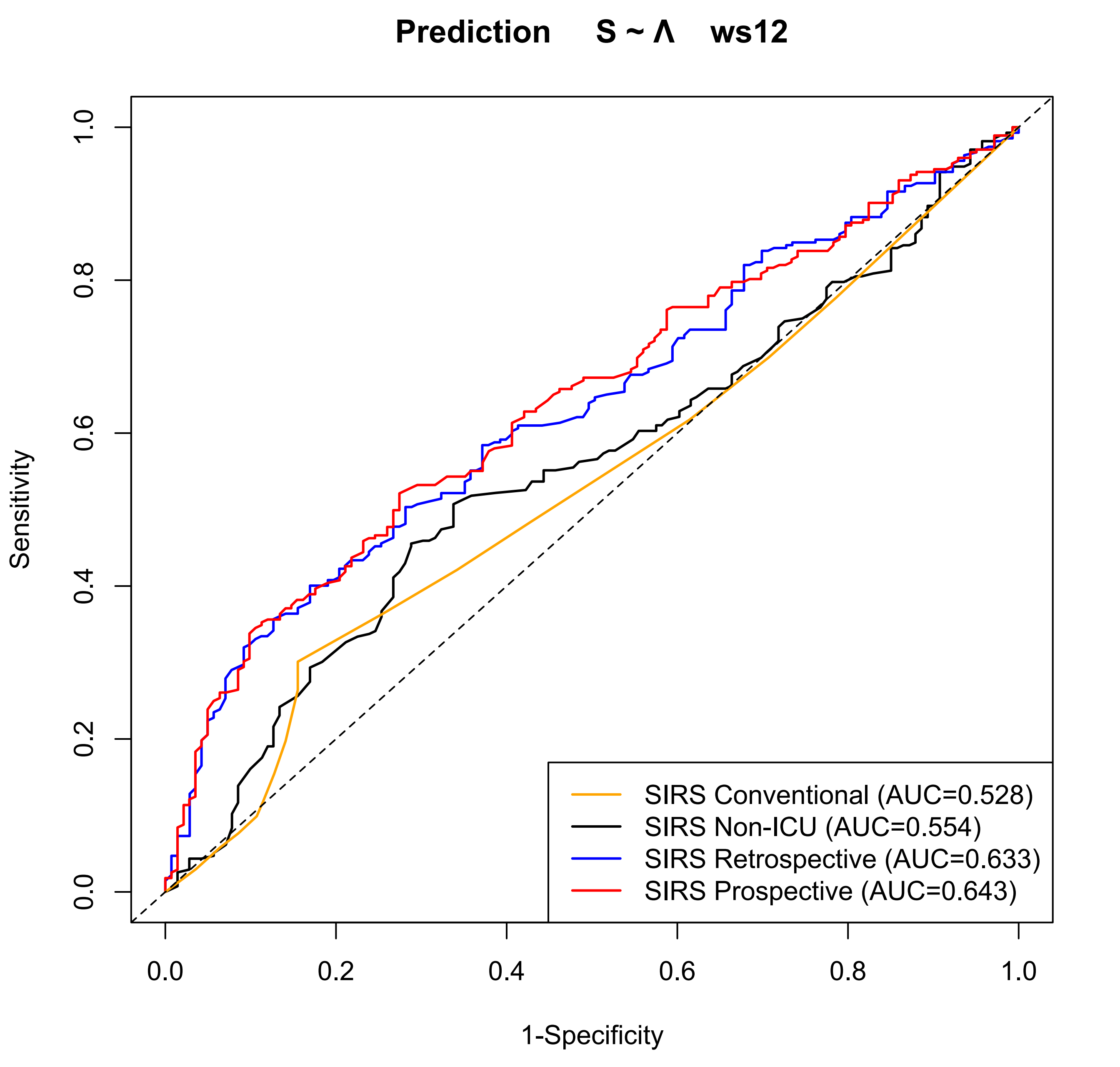

Prediction S ~ Λ ws12
Sensitivity
1-Specificity
0.0
0.2
0.4
0.6
0.8
1.0
SIRS Conventional (AUC=0.528)
SIRS Non-ICU (AUC=0.554)
SIRS Retrospective (AUC=0.633)
SIRS Prospective (AUC=0.643)

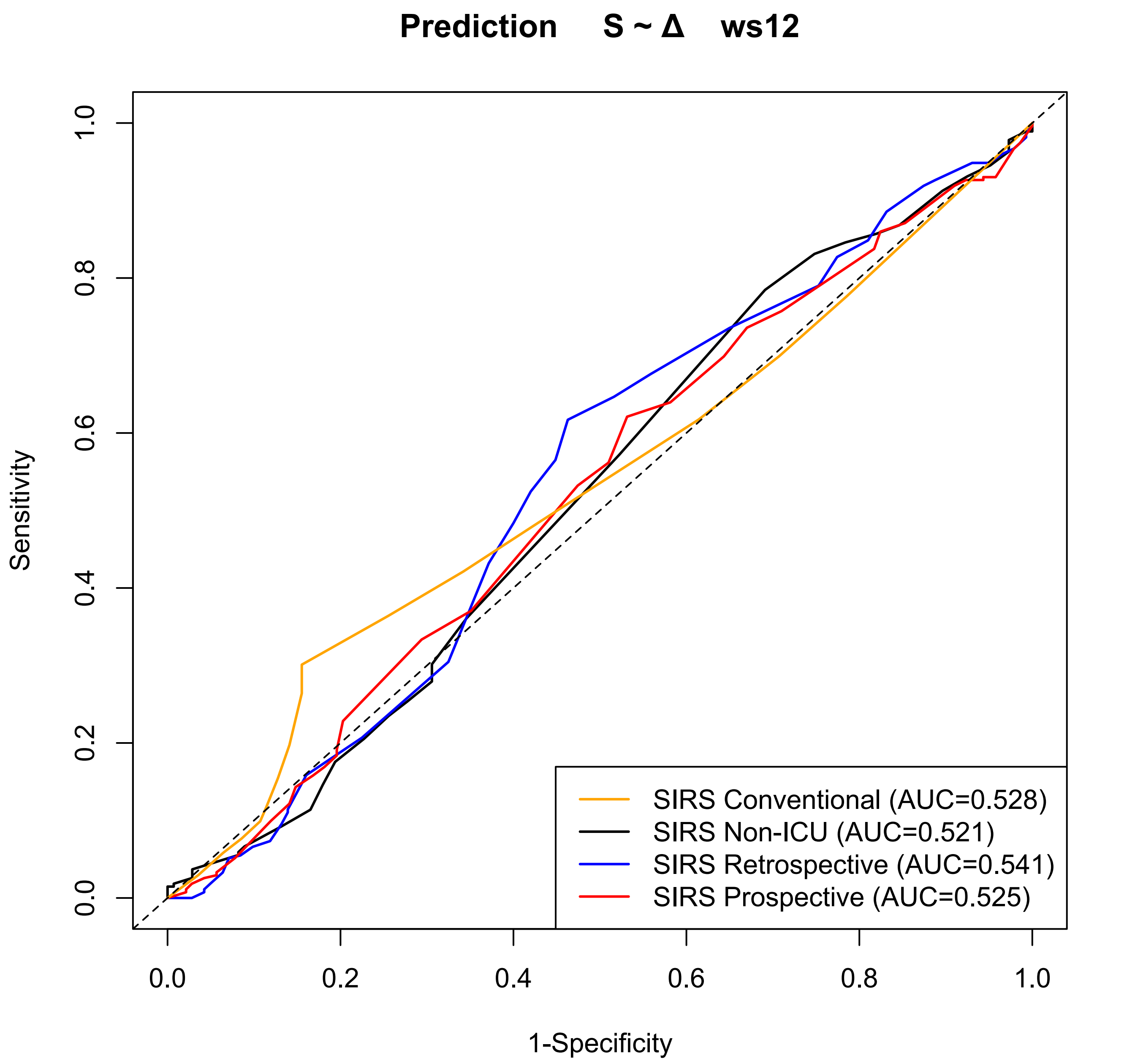

Prediction S ~ Δ ws12
Sensitivity
1-Specificity
0.0
0.2
0.4
0.6
0.8
1.0
SIRS Conventional (AUC=0.528)
SIRS Non-ICU (AUC=0.521)
SIRS Retrospective (AUC=0.541)
SIRS Prospective (AUC=0.525)

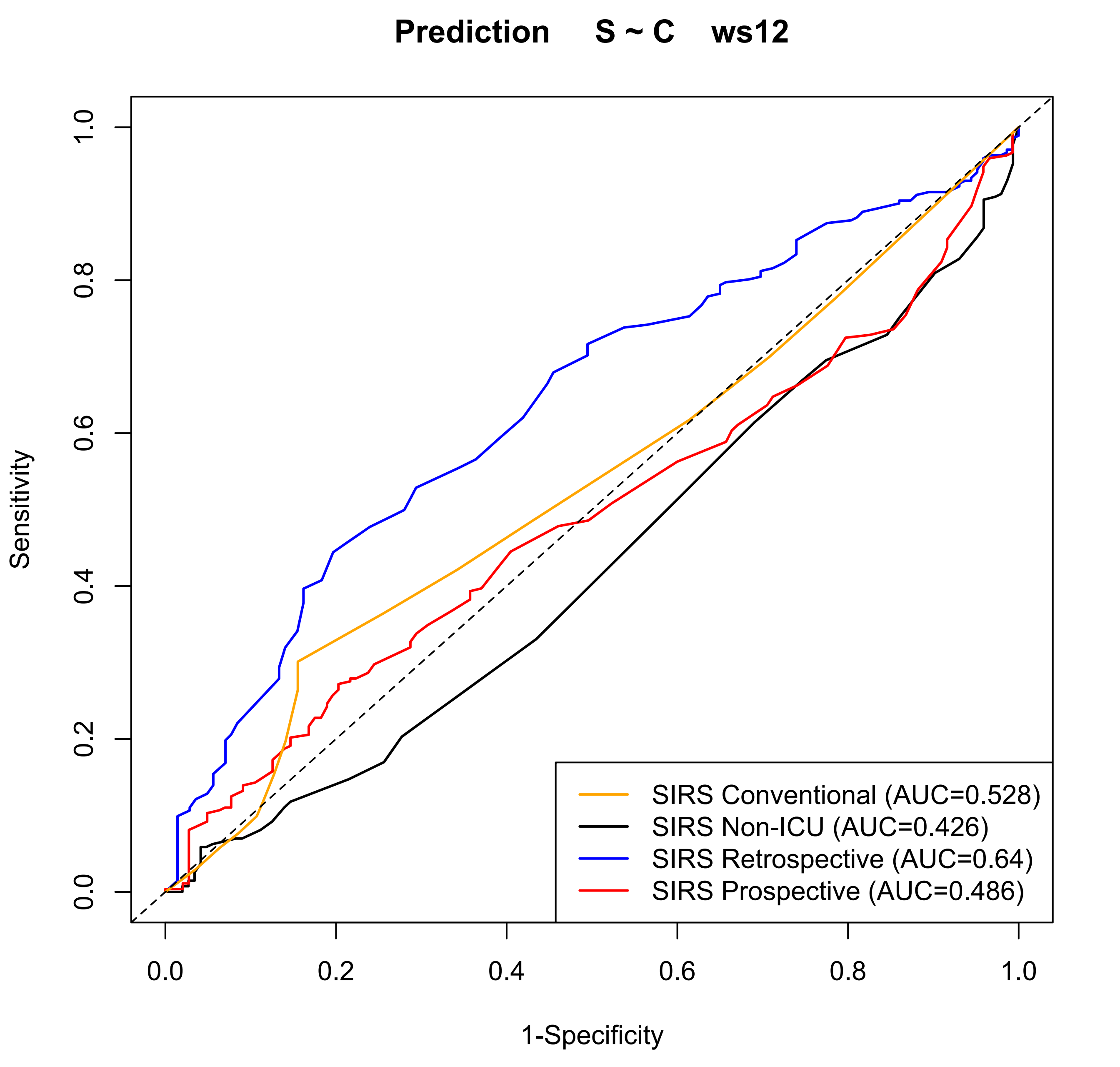
Prediction S ~ C ws12
Sensitivity
1-Specificity
0.0
0.2
0.4
0.6
0.8
1.0
0.0
0.2
0.4
0.6
0.8
1.0
SIRS Conventional (AUC=0.528)
SIRS Non-ICU (AUC=0.426)
SIRS Retrospective (AUC=0.64)
SIRS Prospective (AUC=0.486)

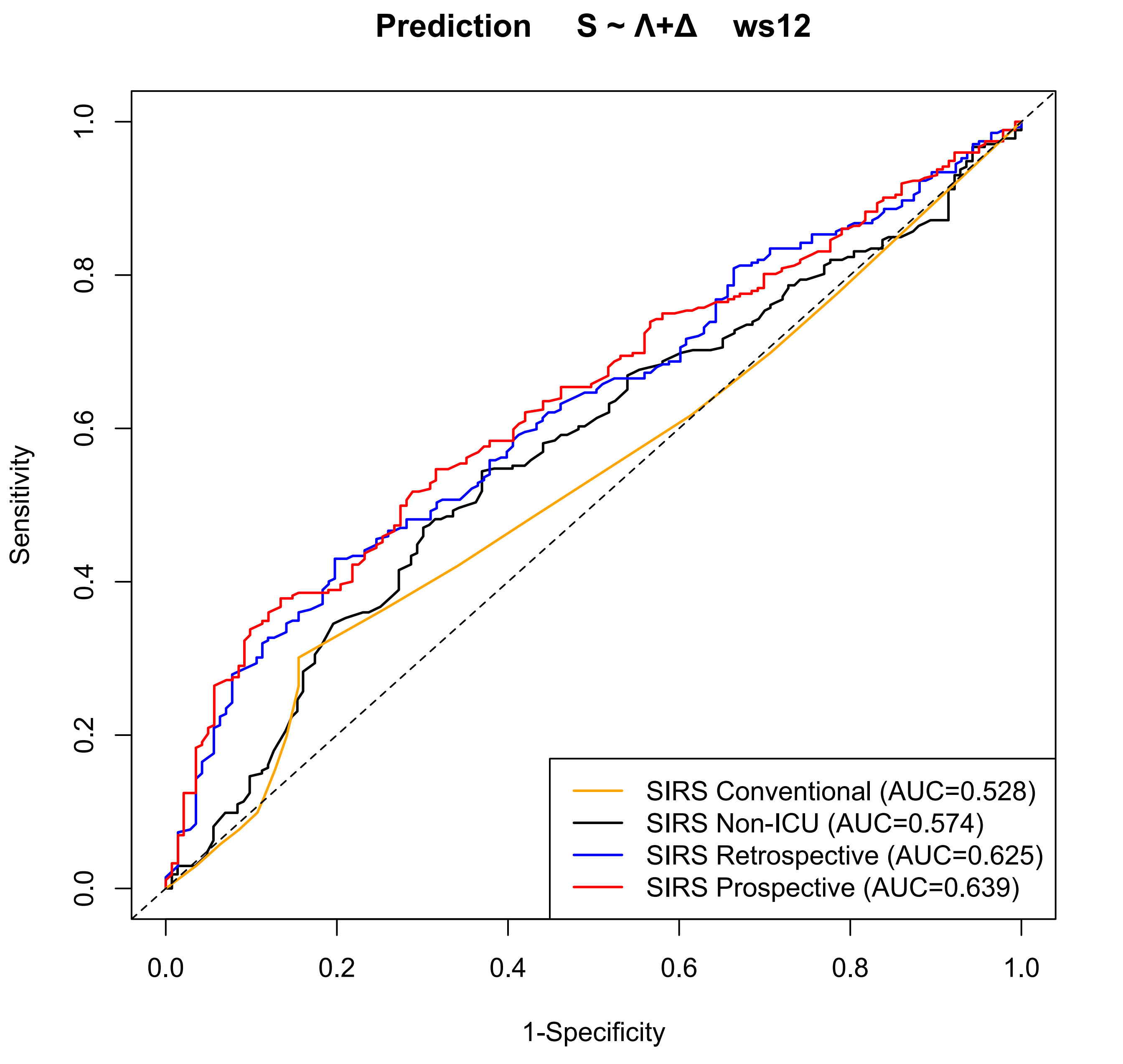

Prediction S ~ Λ+Δ ws12
Sensitivity
1-Specificity
0.0
0.2
0.4
0.6
0.8
1.0
SIRS Conventional (AUC=0.528)
SIRS Non-ICU (AUC=0.574)
SIRS Retrospective (AUC=0.625)
SIRS Prospective (AUC=0.639)

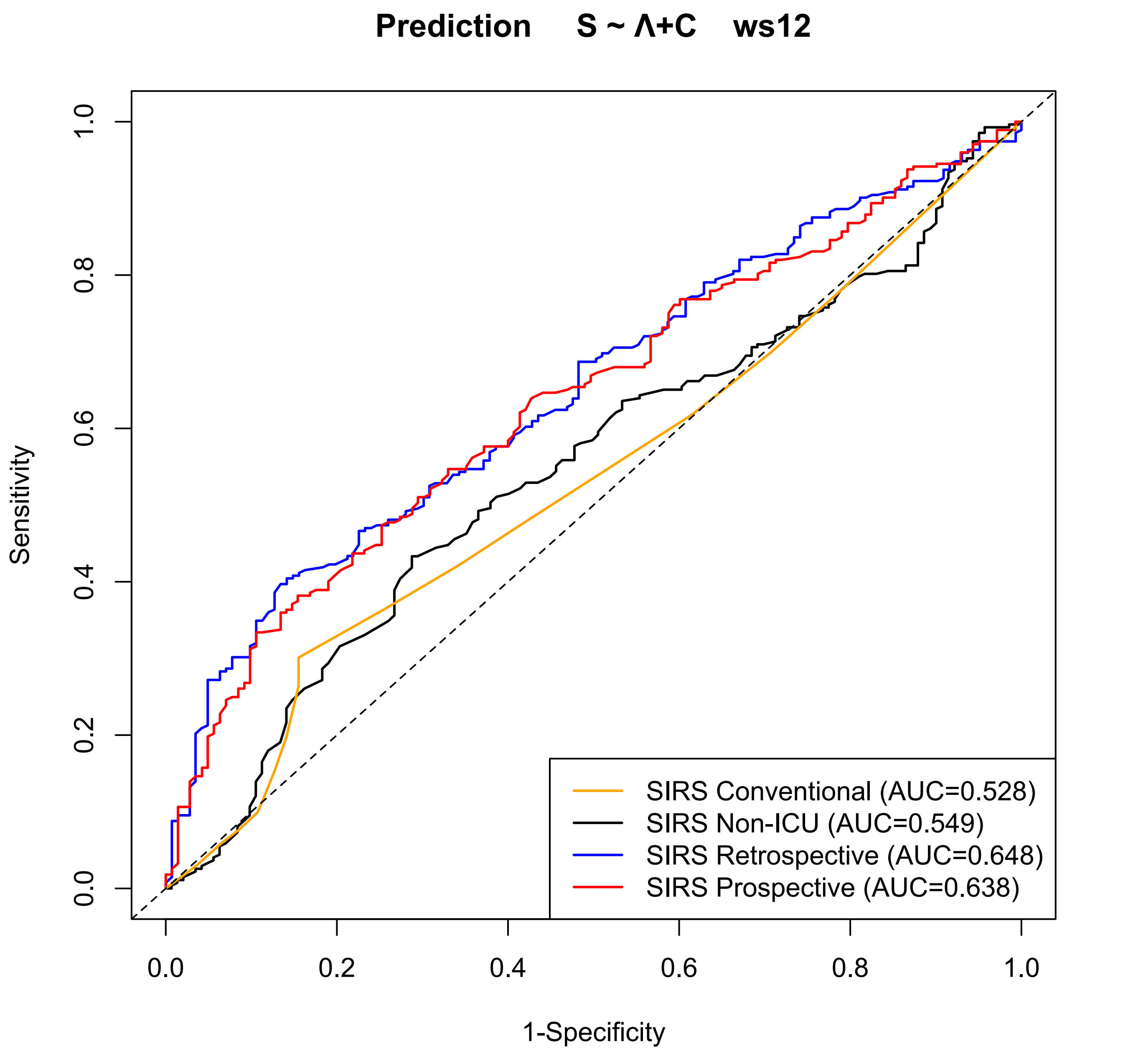

Prediction S ~ Λ+C ws12
Sensitivity
1-Specificity
0.0
0.2
0.4
0.6
0.8
1.0
SIRS Conventional (AUC=0.528)
SIRS Non-ICU (AUC=0.549)
SIRS Retrospective (AUC=0.648)
SIRS Prospective (AUC=0.638)

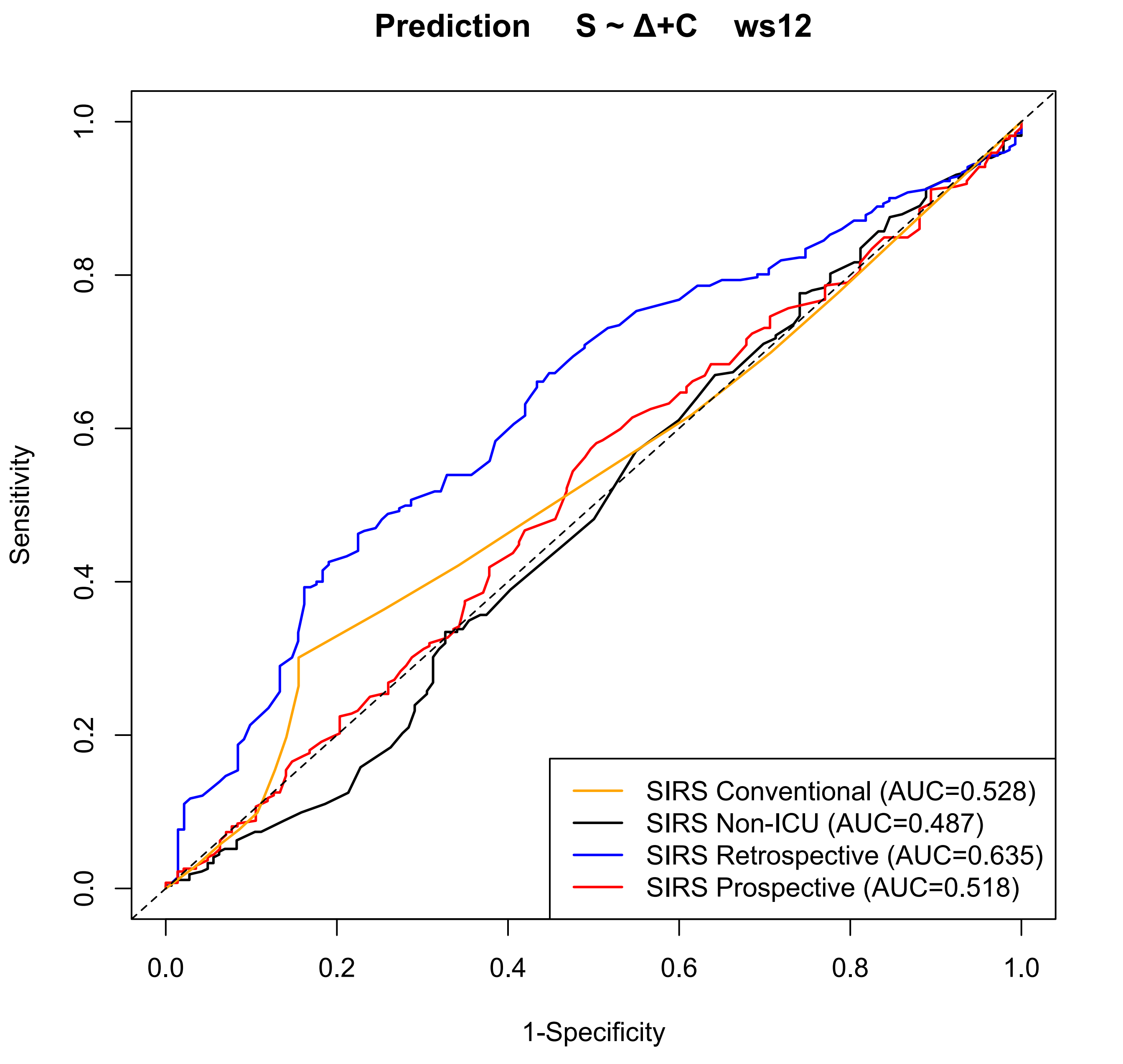

Prediction S ~ Δ+C ws12
Sensitivity
1-Specificity
0.0
0.2
0.4
0.6
0.8
1.0
SIRS Conventional (AUC=0.528)
SIRS Non-ICU (AUC=0.487)
SIRS Retrospective (AUC=0.635)
SIRS Prospective (AUC=0.518)

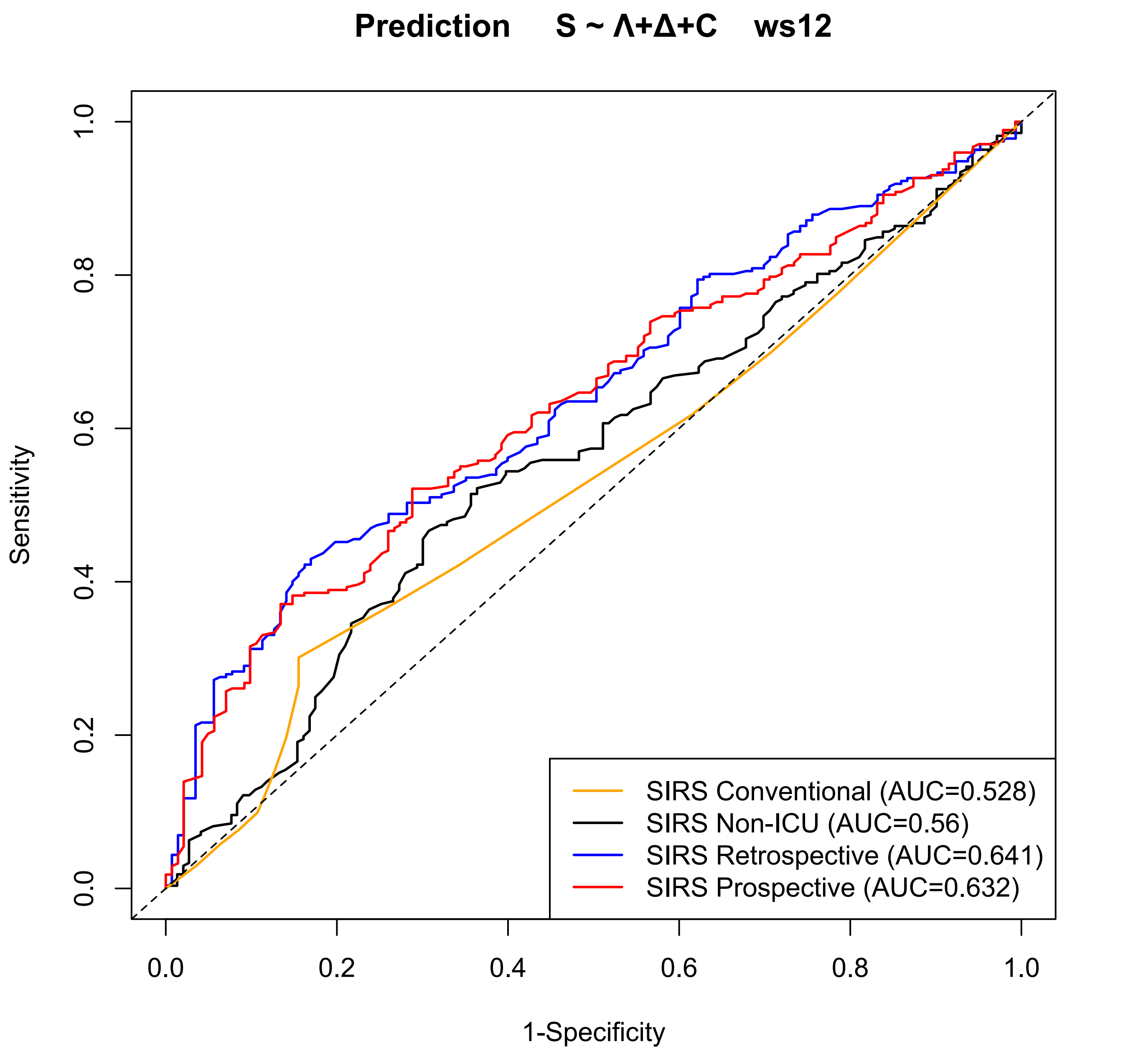

Prediction S ~ Λ+Δ+C ws12
Sensitivity
1-Specificity
0.0
0.2
0.4
0.6
0.8
1.0
SIRS Conventional (AUC=0.528)
SIRS Non-ICU (AUC=0.56)
SIRS Retrospective (AUC=0.641)
SIRS Prospective (AUC=0.632)

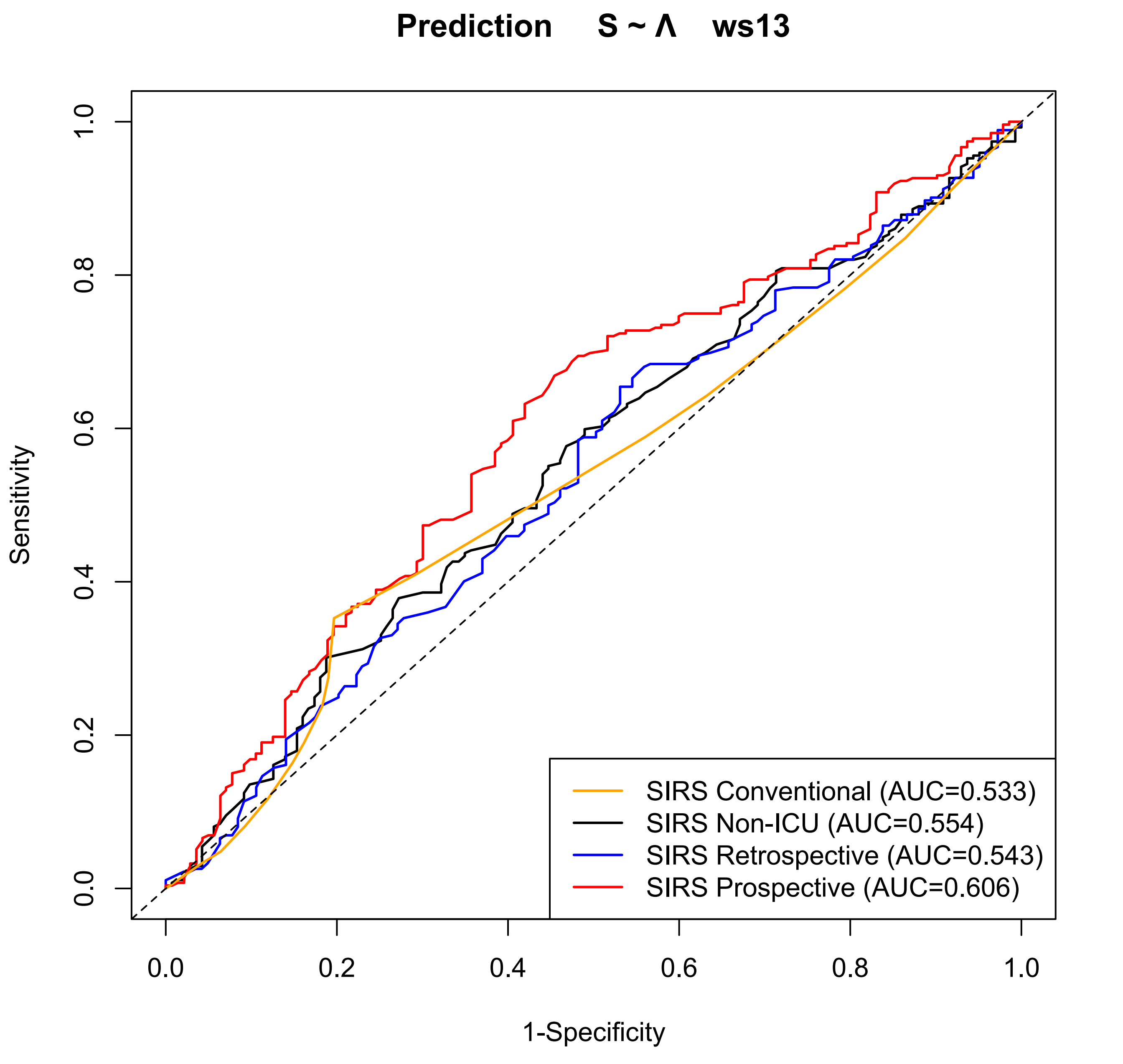

Prediction S ~ Λ ws13
Sensitivity
1-Specificity
0.0
0.2
0.4
0.6
0.8
1.0
SIRS Conventional (AUC=0.533)
SIRS Non-ICU (AUC=0.554)
SIRS Retrospective (AUC=0.543)
SIRS Prospective (AUC=0.606)

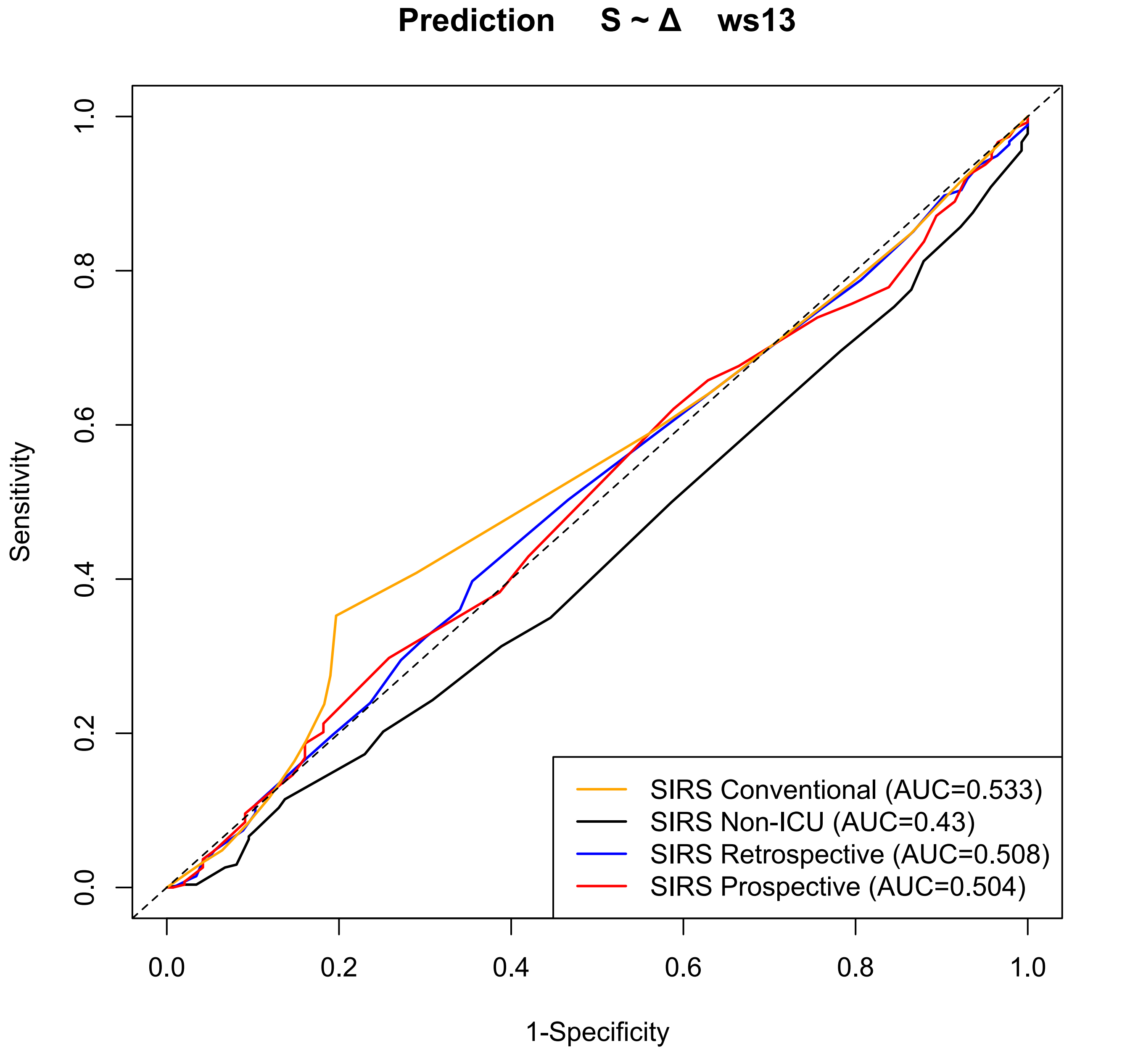
Prediction S ~ Δ ws13
Sensitivity
1-Specificity
0.0
0.2
0.4
0.6
0.8
1.0
SIRS Conventional (AUC=0.533)
SIRS Non-ICU (AUC=0.43)
SIRS Retrospective (AUC=0.508)
SIRS Prospective (AUC=0.504)

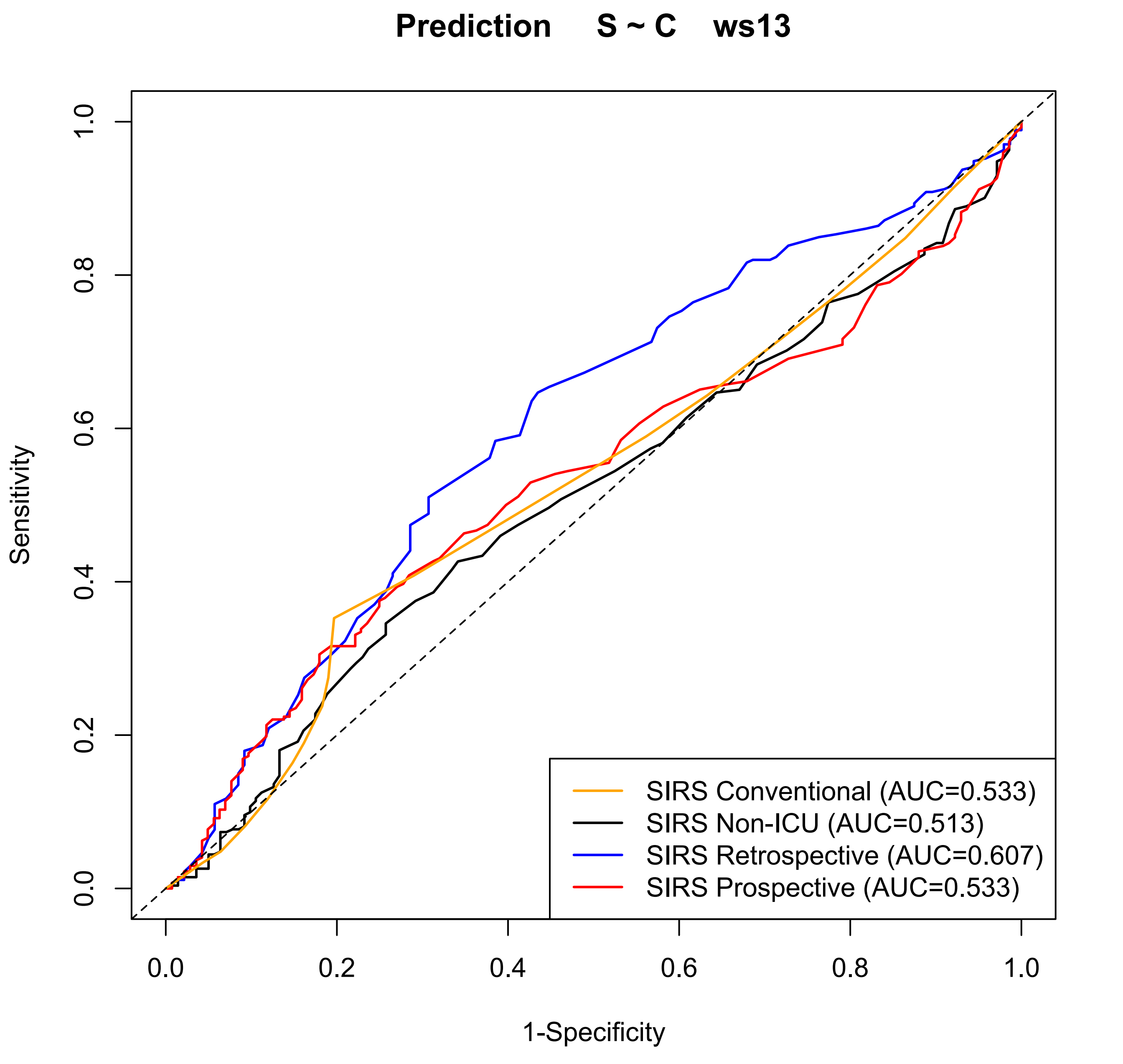

Prediction S ~ C ws13
Sensitivity
1-Specificity
0.0
0.2
0.4
0.6
0.8
1.0
SIRS Conventional (AUC=0.533)
SIRS Non-ICU (AUC=0.513)
SIRS Retrospective (AUC=0.607)
SIRS Prospective (AUC=0.533)

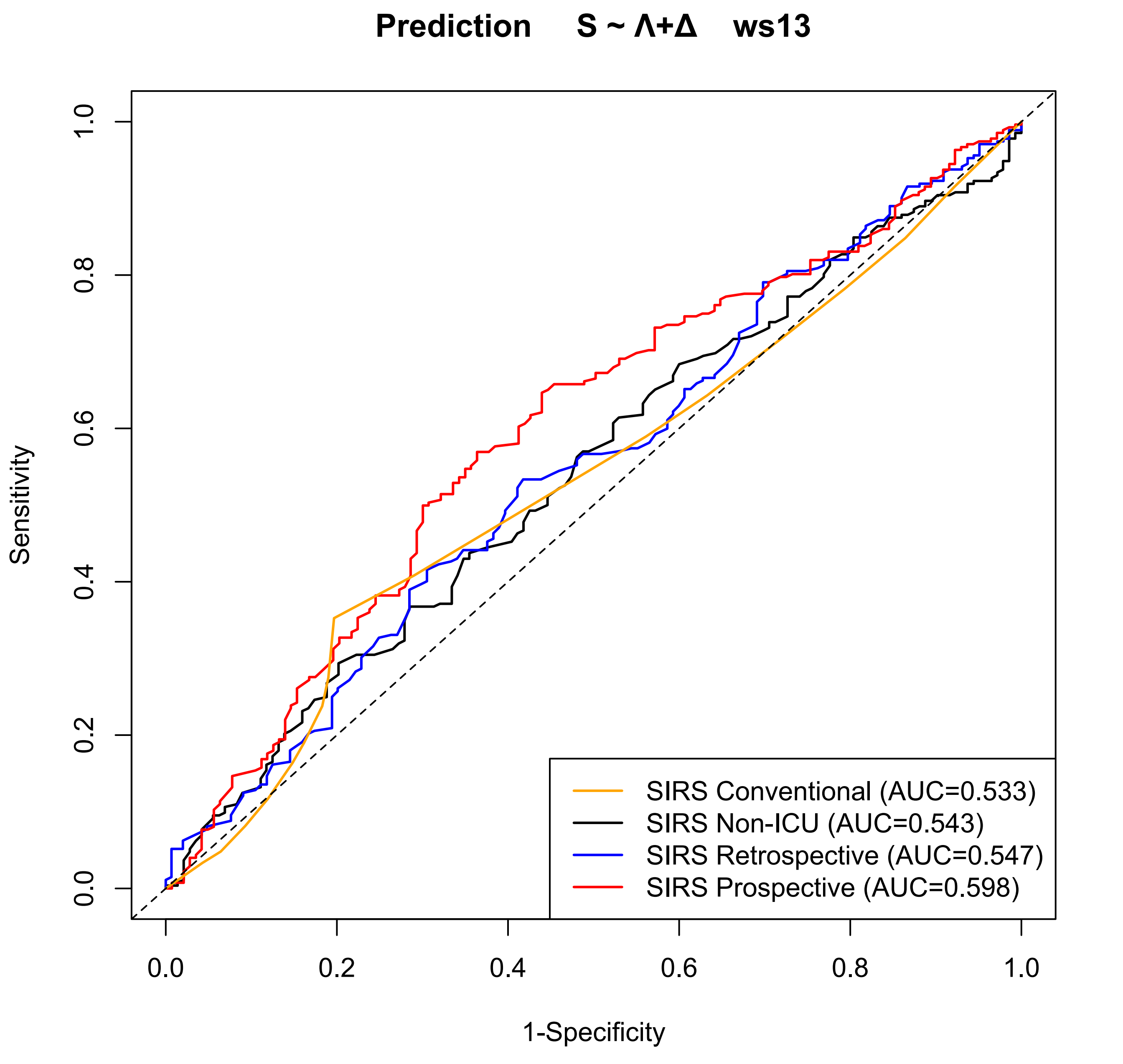

Prediction S ~ Λ+Δ ws13
Sensitivity
1-Specificity
0.0
0.2
0.4
0.6
0.8
1.0
0.0
0.2
0.4
0.6
0.8
1.0
SIRS Conventional (AUC=0.533)
SIRS Non-ICU (AUC=0.543)
SIRS Retrospective (AUC=0.547)
SIRS Prospective (AUC=0.598)

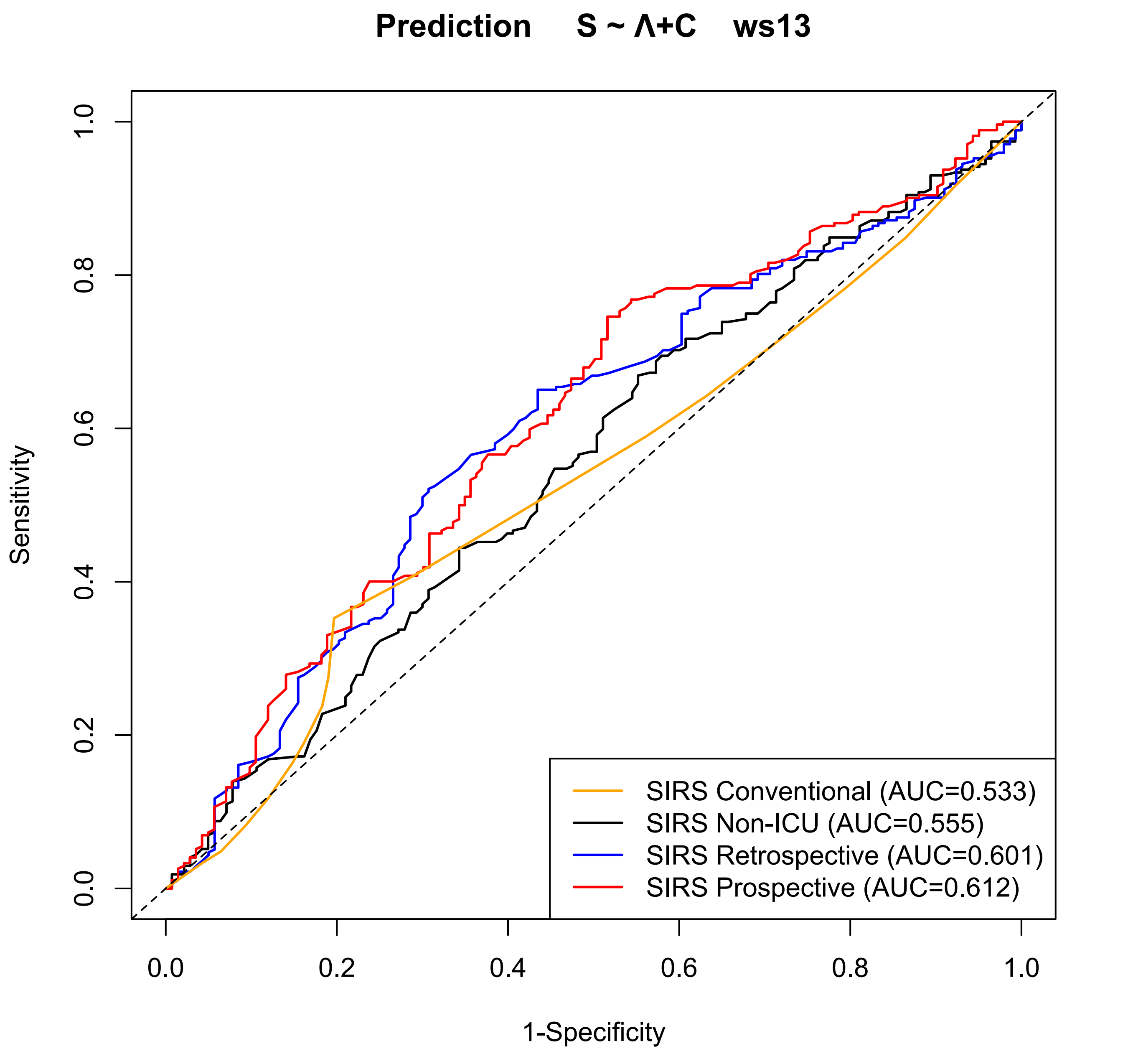

Prediction S ~ Λ+C ws13
Sensitivity
1-Specificity
0.0
0.2
0.4
0.6
0.8
1.0
0.0
0.2
0.4
0.6
0.8
1.0
SIRS Conventional (AUC=0.533)
SIRS Non-ICU (AUC=0.555)
SIRS Retrospective (AUC=0.601)
SIRS Prospective (AUC=0.612)

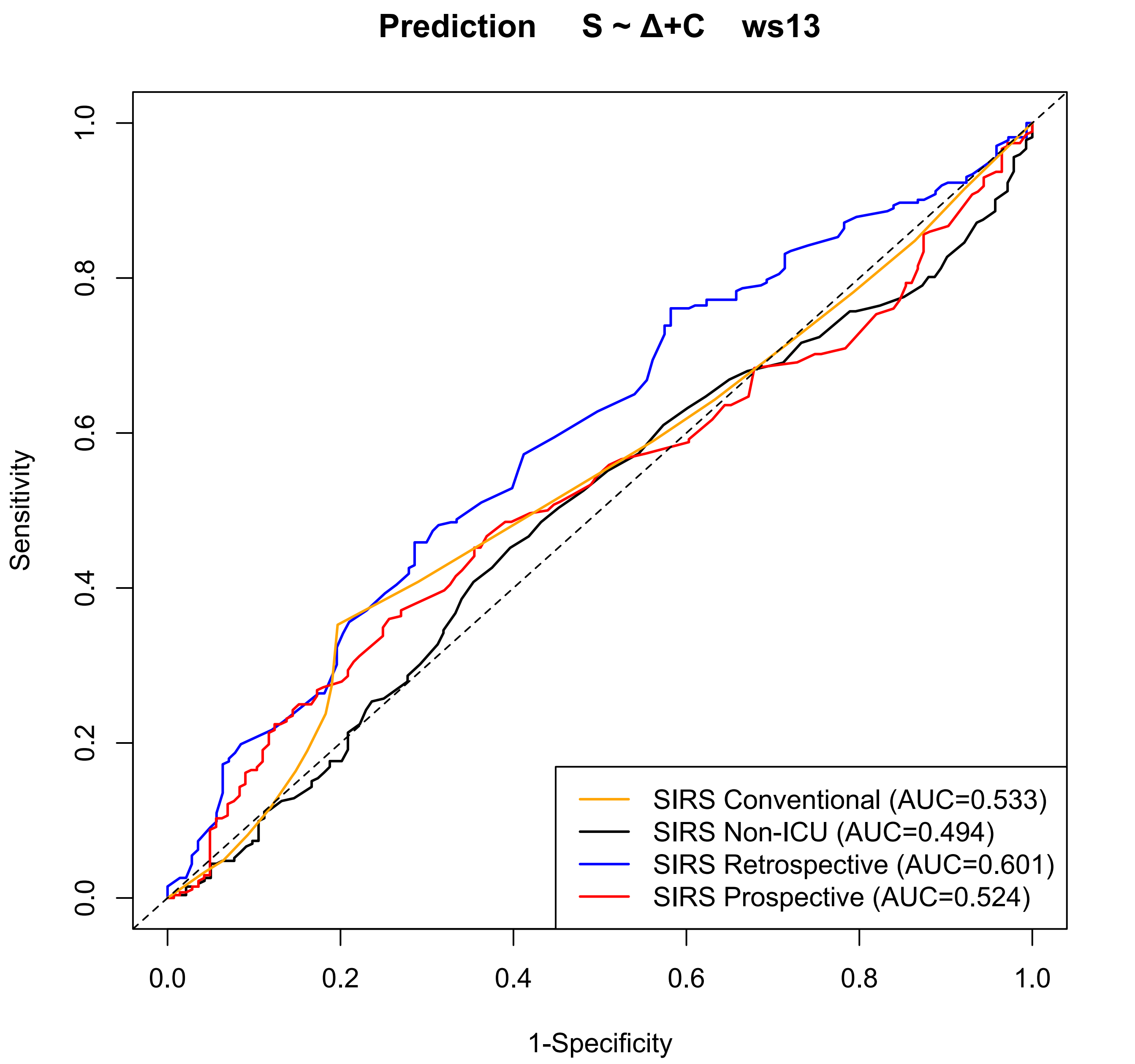

Prediction S ~ Δ+C ws13
Sensitivity
1-Specificity
0.0
0.2
0.4
0.6
0.8
1.0
SIRS Conventional (AUC=0.533)
SIRS Non-ICU (AUC=0.494)
SIRS Retrospective (AUC=0.601)
SIRS Prospective (AUC=0.524)

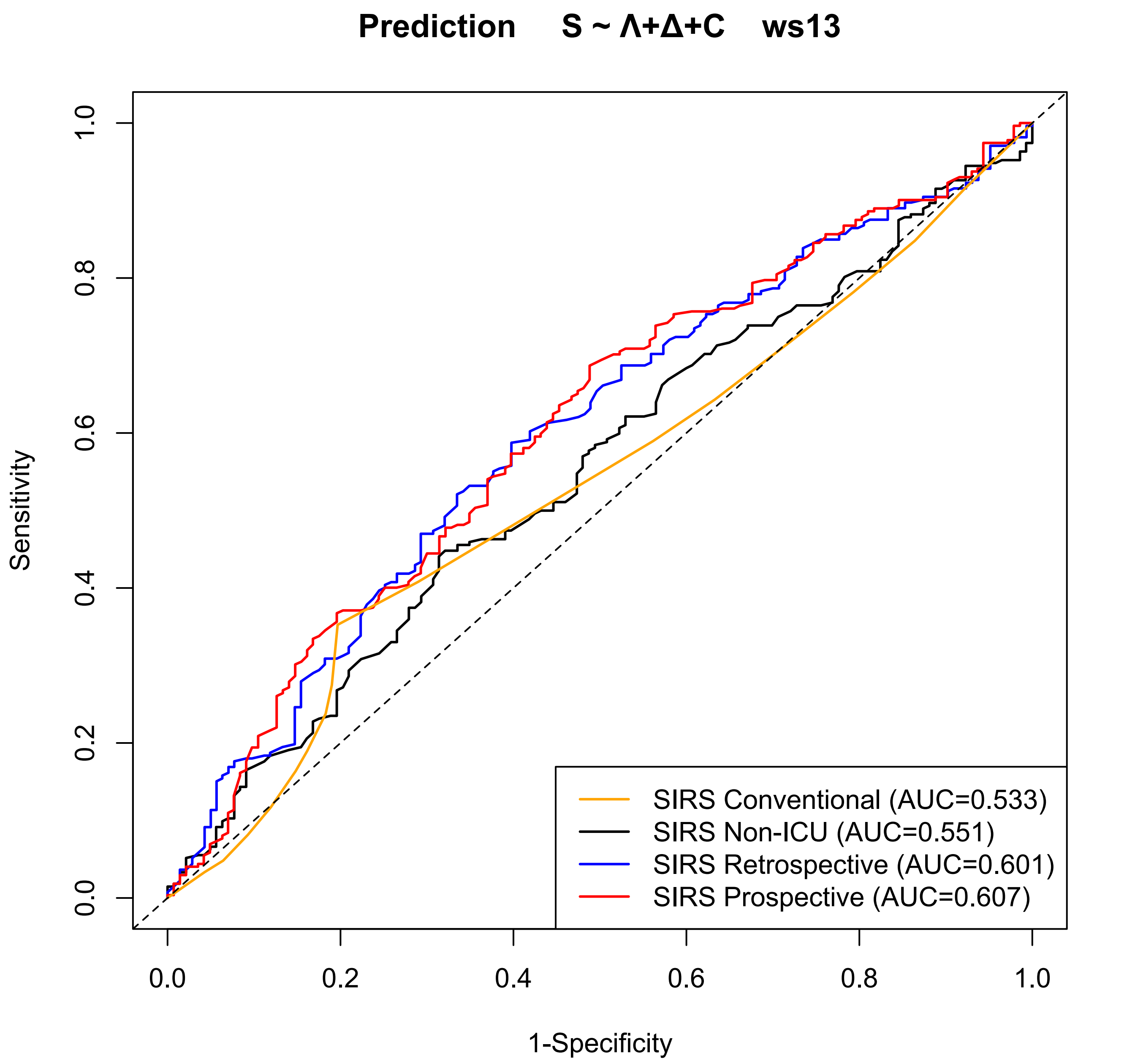

Prediction S ~ Λ+Δ+C ws13
Sensitivity
1-Specificity
0.0
0.2
0.4
0.6
0.8
1.0
SIRS Conventional (AUC=0.533)
SIRS Non-ICU (AUC=0.551)
SIRS Retrospective (AUC=0.601)
SIRS Prospective (AUC=0.607)

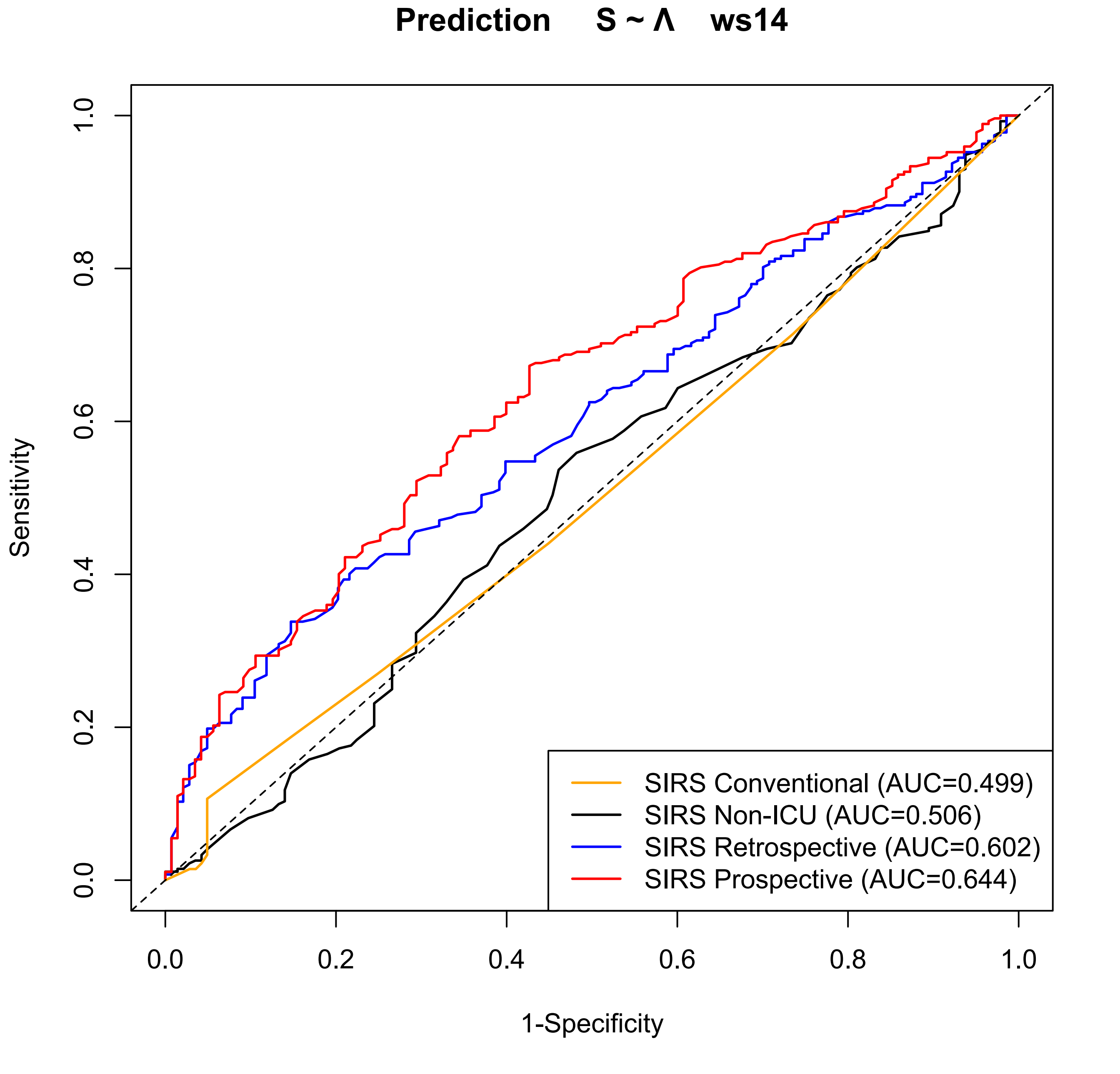

Prediction S ~ Λ ws14
Sensitivity
1-Specificity
0.0
0.2
0.4
0.6
0.8
1.0
SIRS Conventional (AUC=0.499)
SIRS Non-ICU (AUC=0.506)
SIRS Retrospective (AUC=0.602)
SIRS Prospective (AUC=0.644)

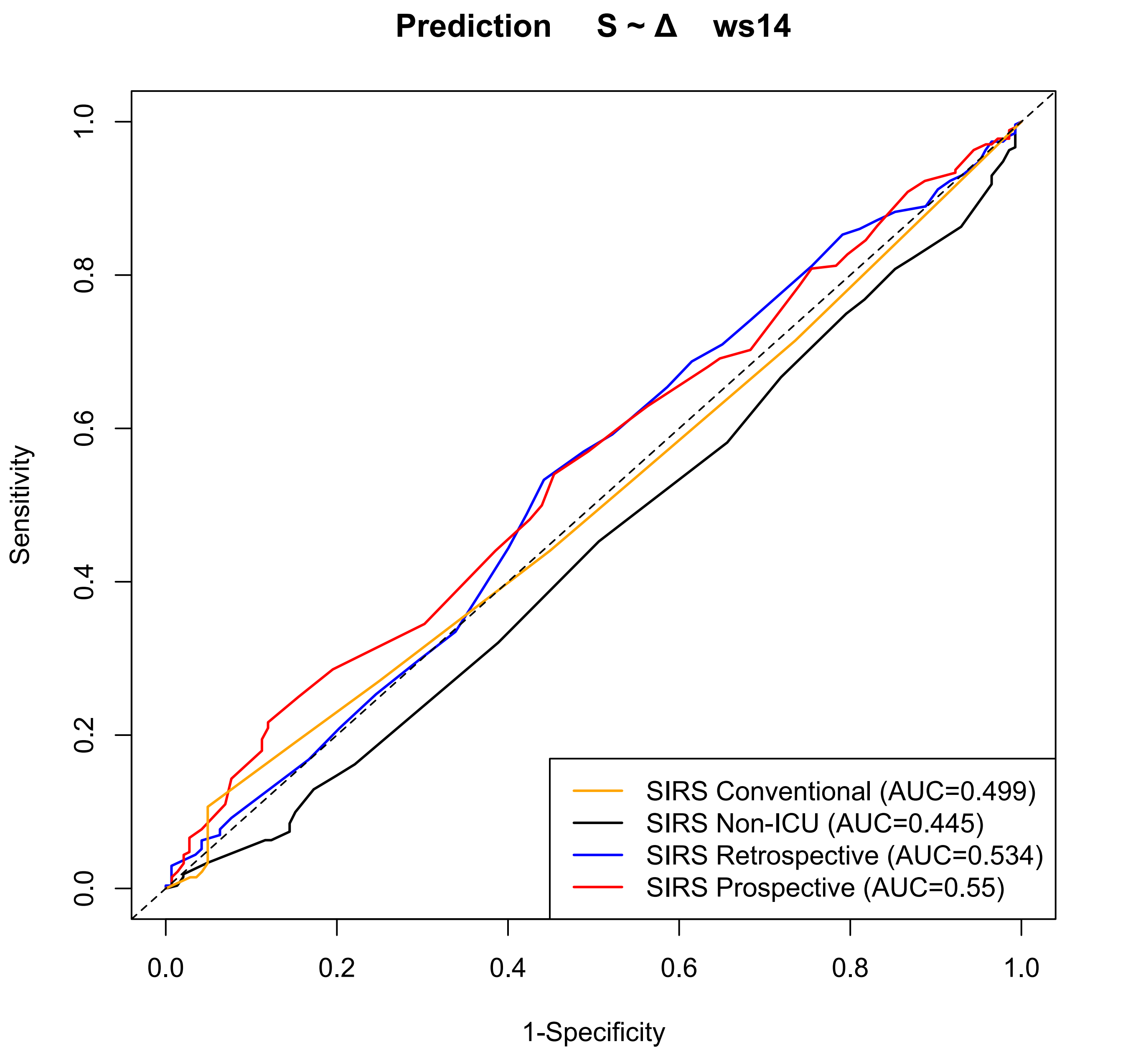

Prediction S ~ Δ ws14
Sensitivity
1-Specificity
0.0
0.2
0.4
0.6
0.8
1.0
SIRS Conventional (AUC=0.499)
SIRS Non-ICU (AUC=0.445)
SIRS Retrospective (AUC=0.534)
SIRS Prospective (AUC=0.55)

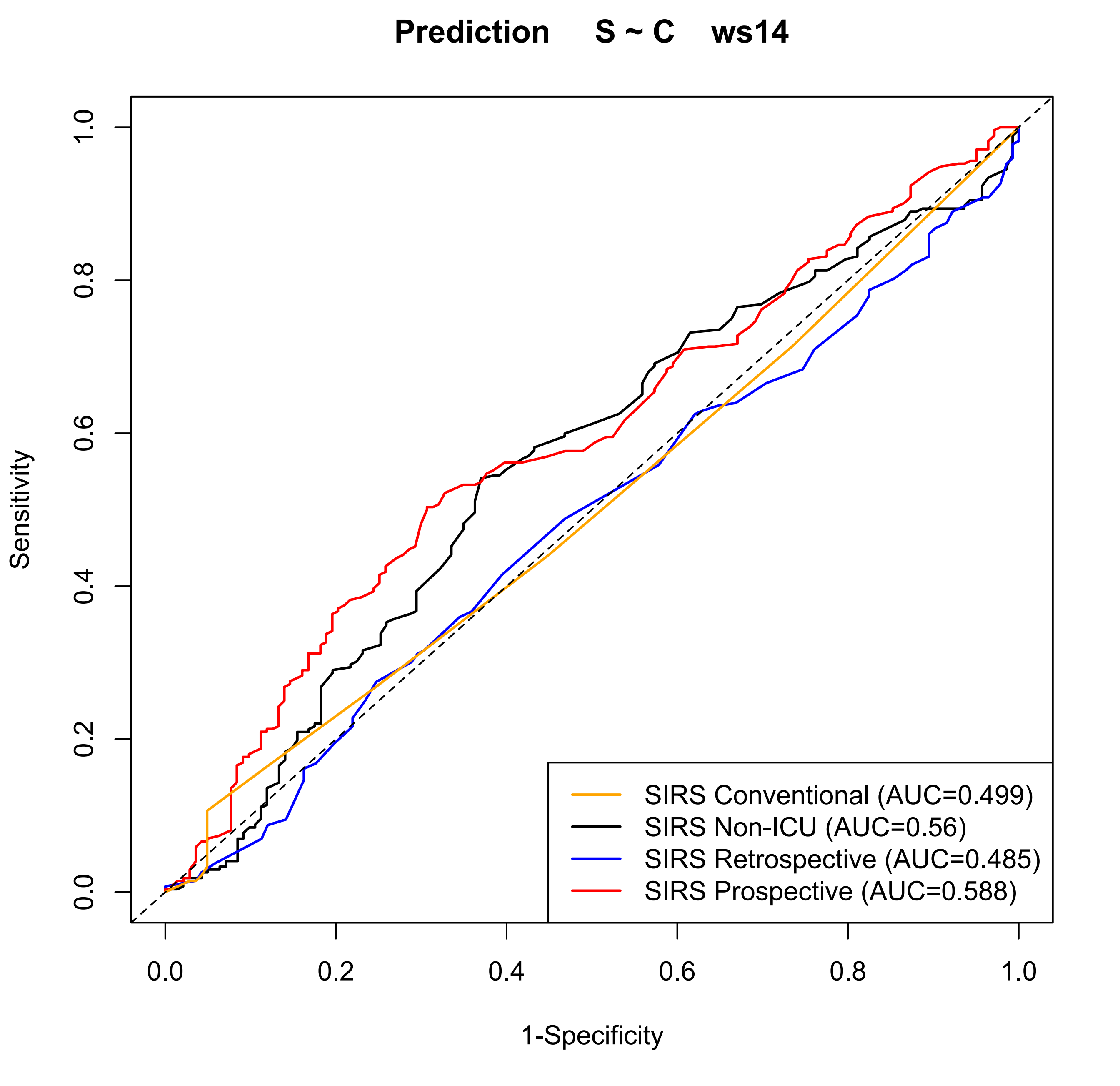

Prediction S ~ C ws14
Sensitivity
1-Specificity
0.0
0.2
0.4
0.6
0.8
1.0
SIRS Conventional (AUC=0.499)
SIRS Non-ICU (AUC=0.56)
SIRS Retrospective (AUC=0.485)
SIRS Prospective (AUC=0.588)

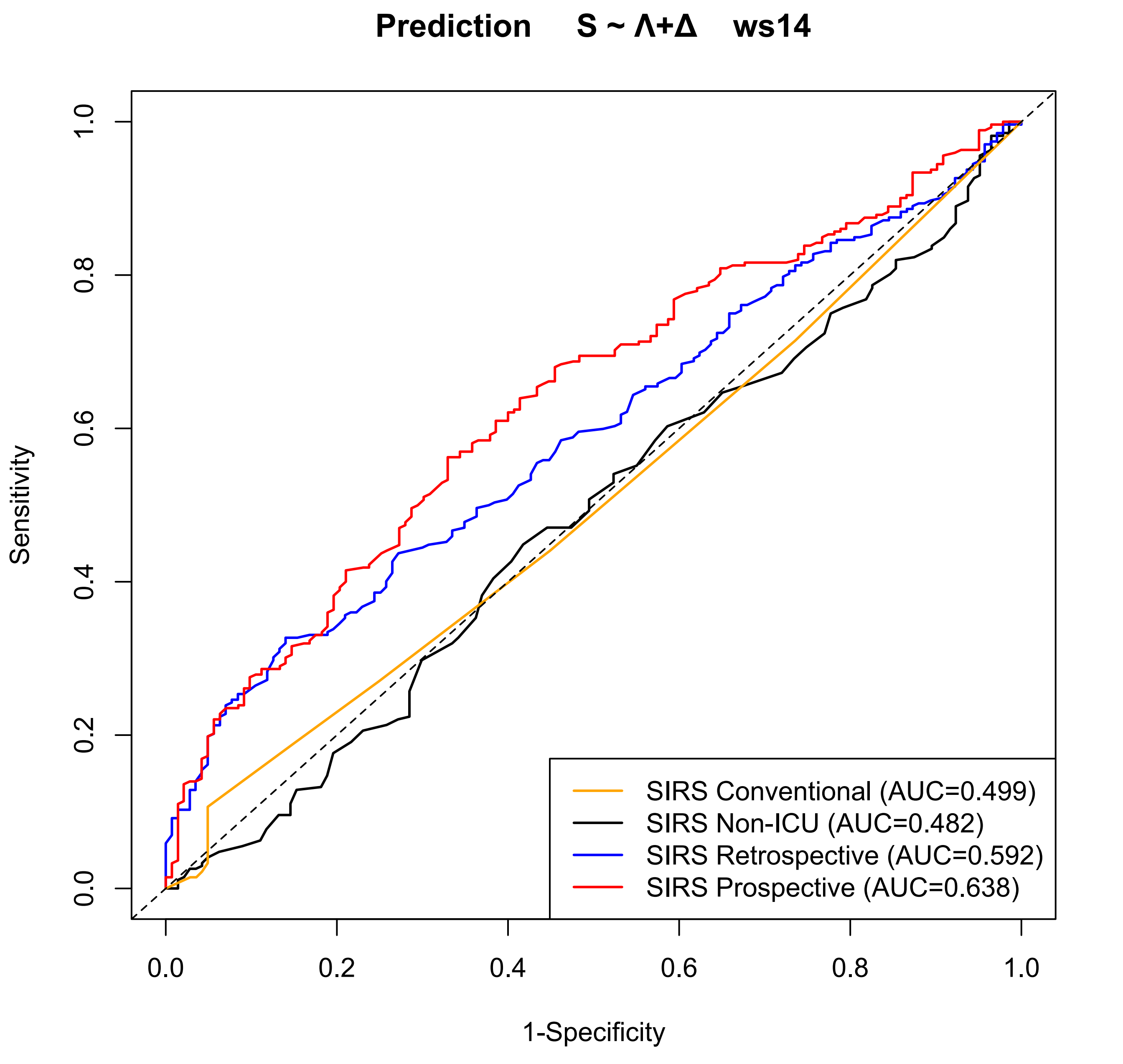
Prediction S ~ Λ+Δ ws14
Sensitivity
1-Specificity
0.0
0.2
0.4
0.6
0.8
1.0
SIRS Conventional (AUC=0.499)
SIRS Non-ICU (AUC=0.482)
SIRS Retrospective (AUC=0.592)
SIRS Prospective (AUC=0.638)

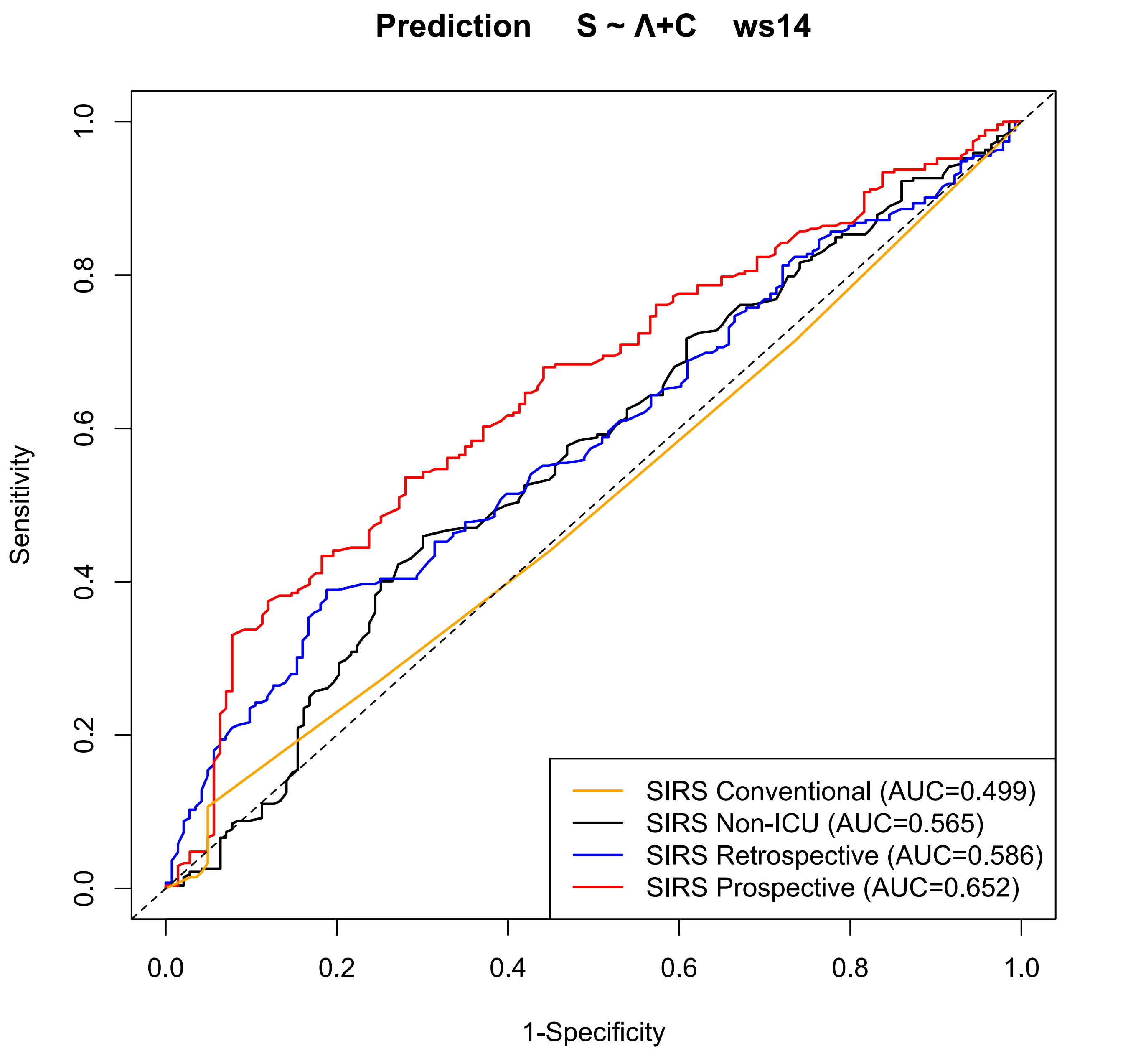
Prediction S ~ Λ+C ws14
Sensitivity
1-Specificity
0.0
0.2
0.4
0.6
0.8
1.0
0.0
0.2
0.4
0.6
0.8
1.0
SIRS Conventional (AUC=0.499)
SIRS Non-ICU (AUC=0.565)
SIRS Retrospective (AUC=0.586)
SIRS Prospective (AUC=0.652)

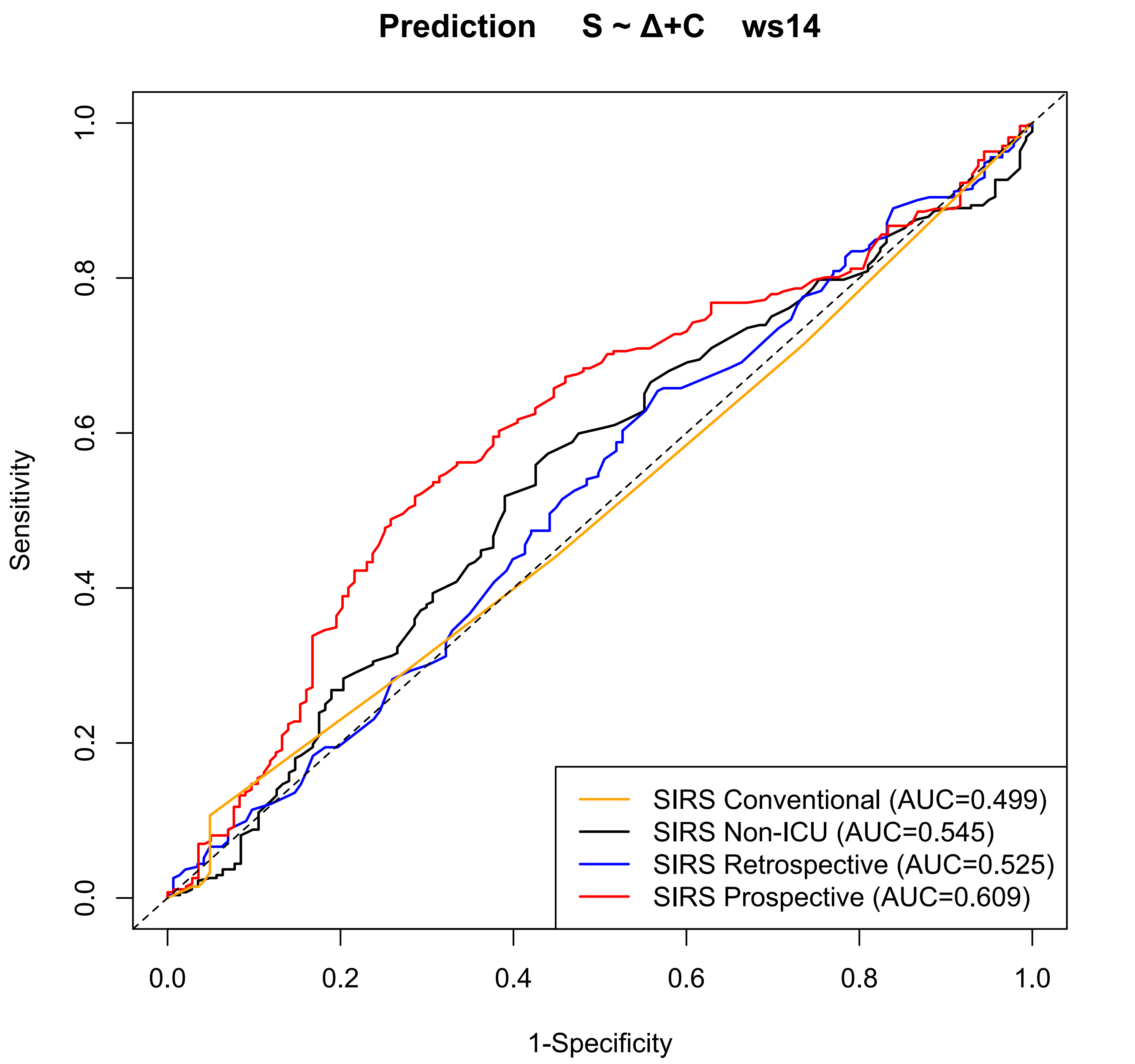
Prediction S ~ Δ+C ws14
Sensitivity
1-Specificity
0.0
0.2
0.4
0.6
0.8
1.0
SIRS Conventional (AUC=0.499)
SIRS Non-ICU (AUC=0.545)
SIRS Retrospective (AUC=0.525)
SIRS Prospective (AUC=0.609)

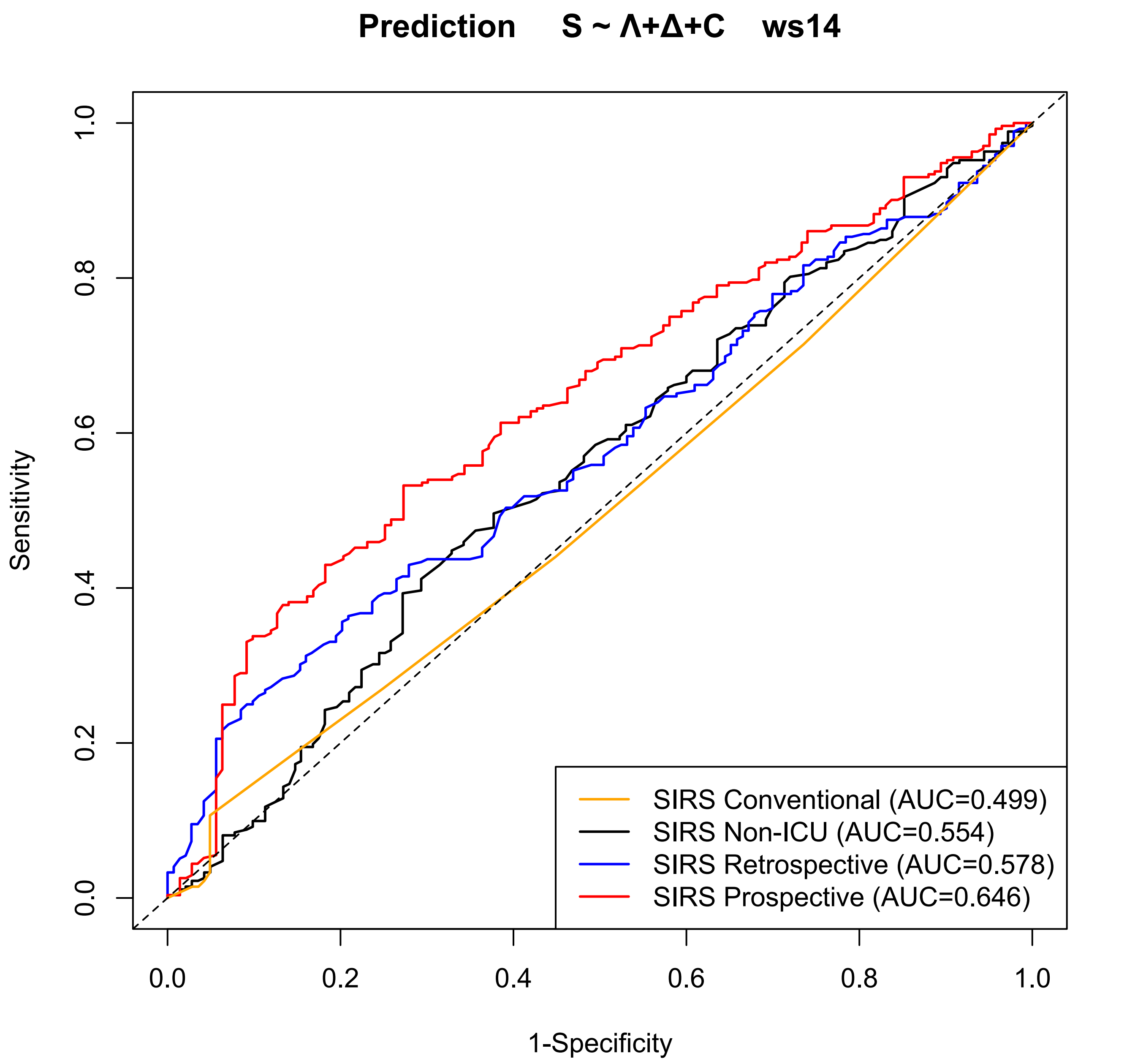

Prediction S ~ Λ+Δ+C ws14
Sensitivity
1-Specificity
0.0
0.2
0.4
0.6
0.8
1.0
SIRS Conventional (AUC=0.499)
SIRS Non-ICU (AUC=0.554)
SIRS Retrospective (AUC=0.578)
SIRS Prospective (AUC=0.646)

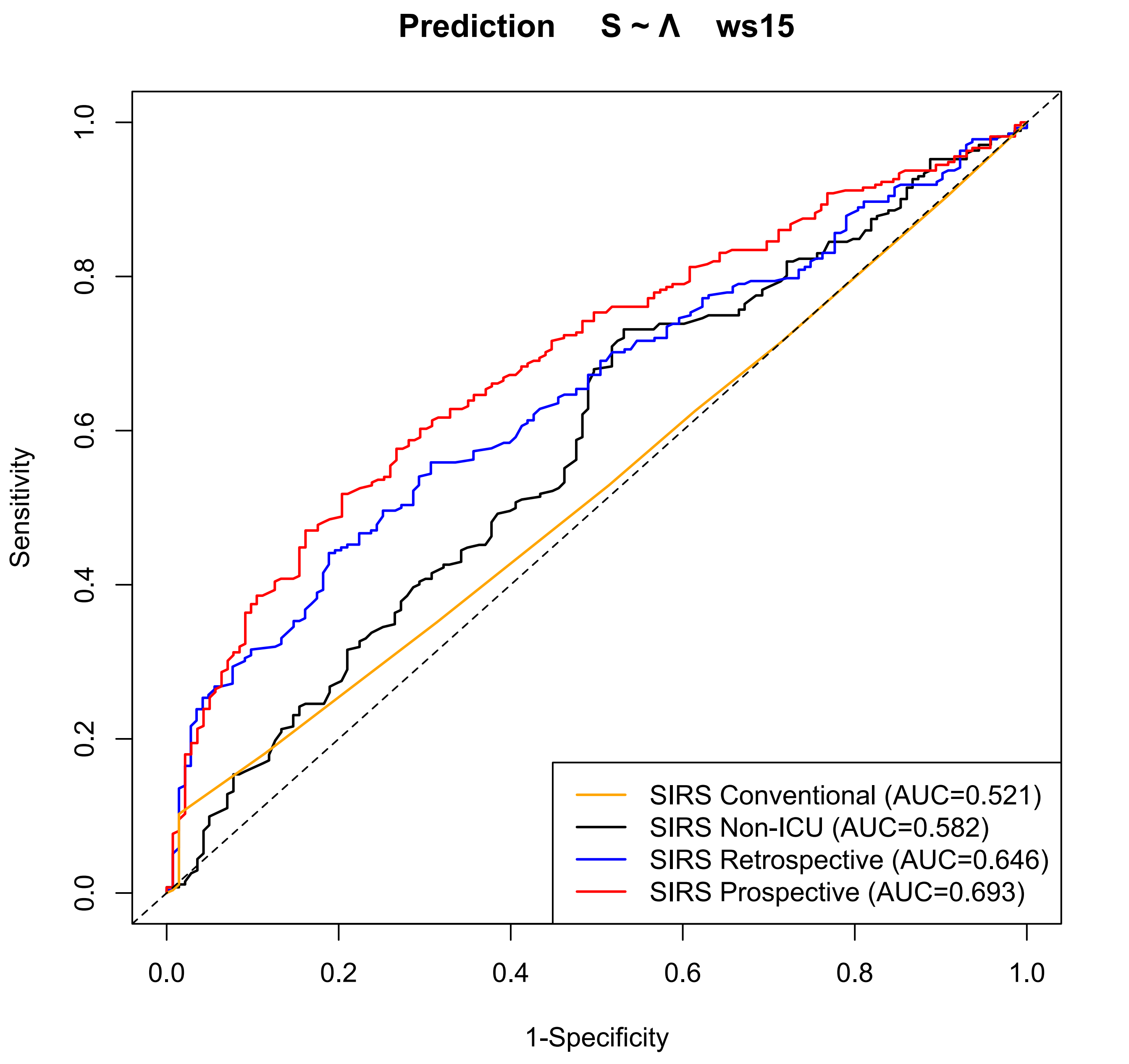

Prediction S ~ Λ ws15
Sensitivity
1-Specificity
0.0
0.2
0.4
0.6
0.8
1.0
0.0
0.2
0.4
0.6
0.8
1.0
SIRS Conventional (AUC=0.521)
SIRS Non-ICU (AUC=0.582)
SIRS Retrospective (AUC=0.646)
SIRS Prospective (AUC=0.693)

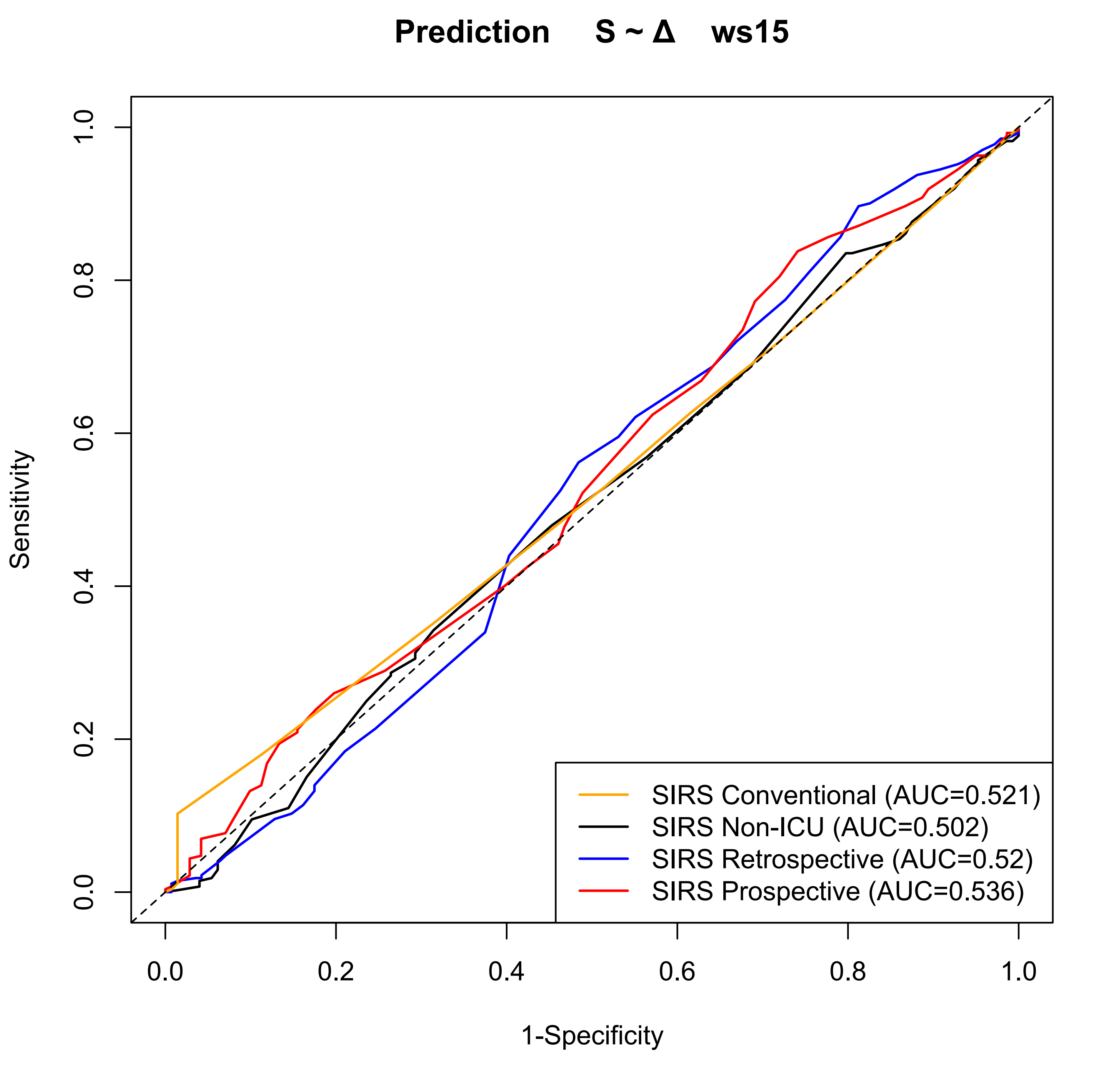

Prediction S ~ Δ ws15
Sensitivity
1-Specificity
0.0
0.2
0.4
0.6
0.8
1.0
SIRS Conventional (AUC=0.521)
SIRS Non-ICU (AUC=0.502)
SIRS Retrospective (AUC=0.52)
SIRS Prospective (AUC=0.536)

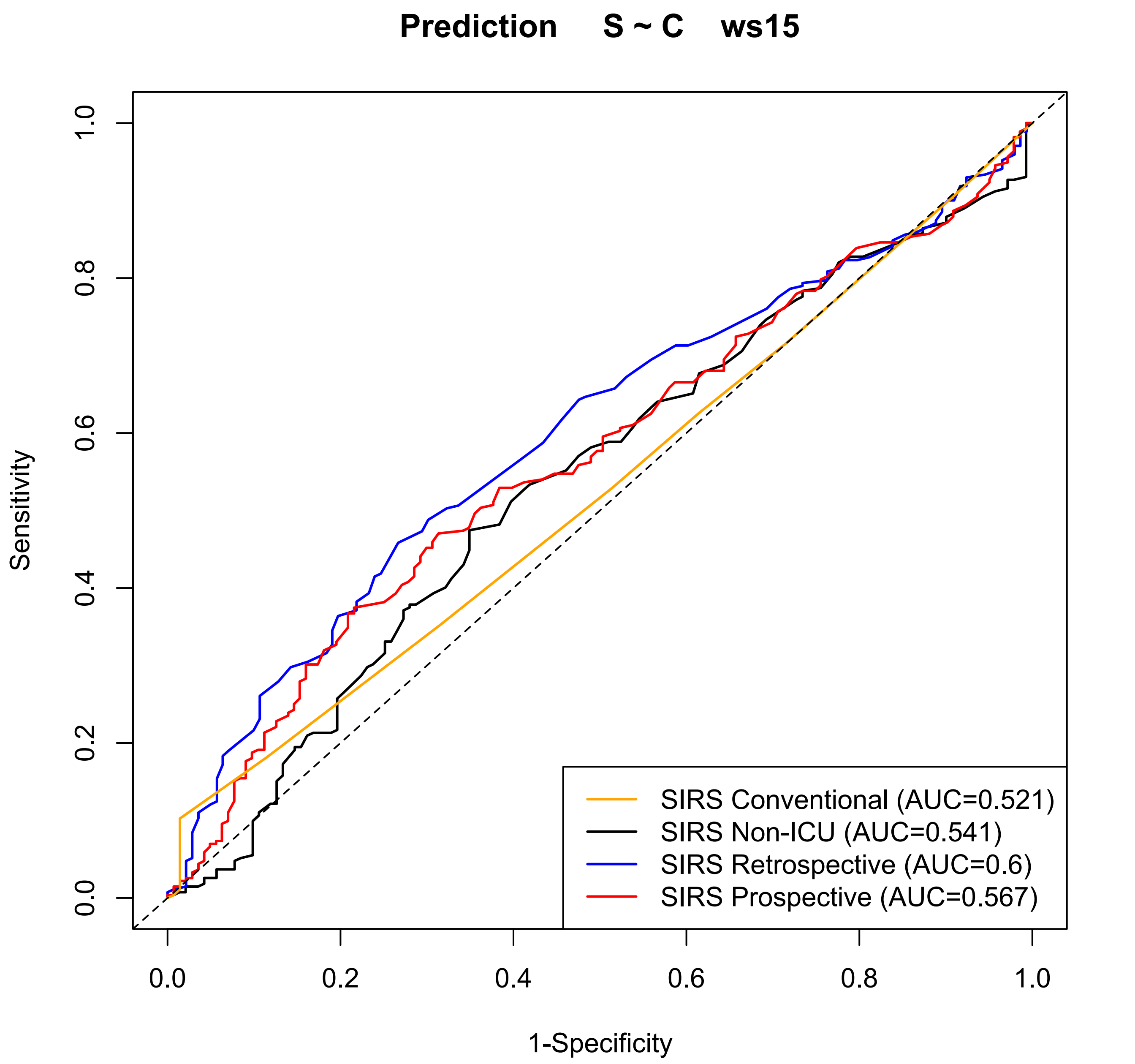

Prediction S ~ C ws15
Sensitivity
1-Specificity
0.0
0.2
0.4
0.6
0.8
1.0
SIRS Conventional (AUC=0.521)
SIRS Non-ICU (AUC=0.541)
SIRS Retrospective (AUC=0.6)
SIRS Prospective (AUC=0.567)

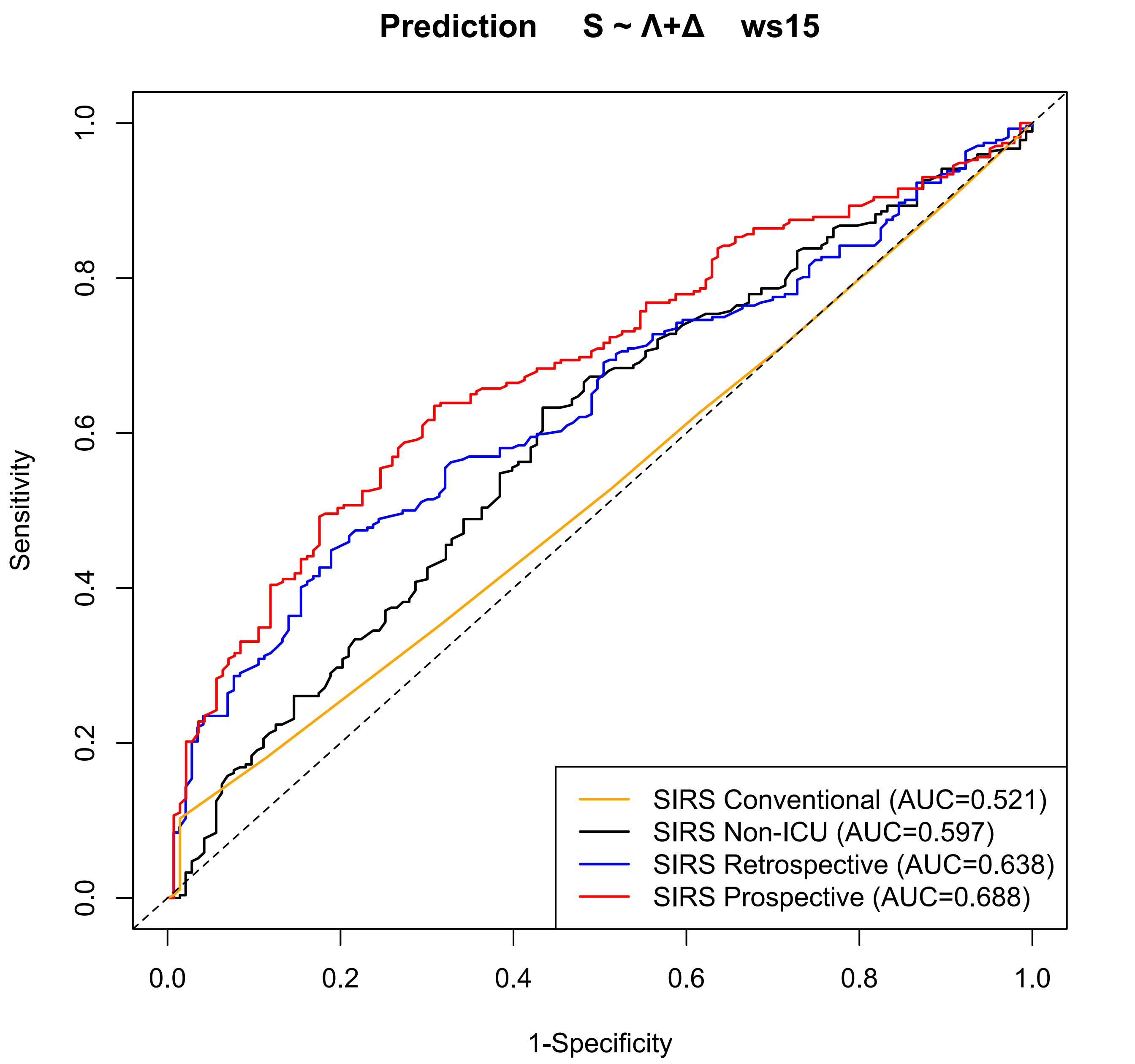

Prediction S ~ Λ+Δ ws15
Sensitivity
1-Specificity
0.0
0.2
0.4
0.6
0.8
1.0
SIRS Conventional (AUC=0.521)
SIRS Non-ICU (AUC=0.597)
SIRS Retrospective (AUC=0.638)
SIRS Prospective (AUC=0.688)

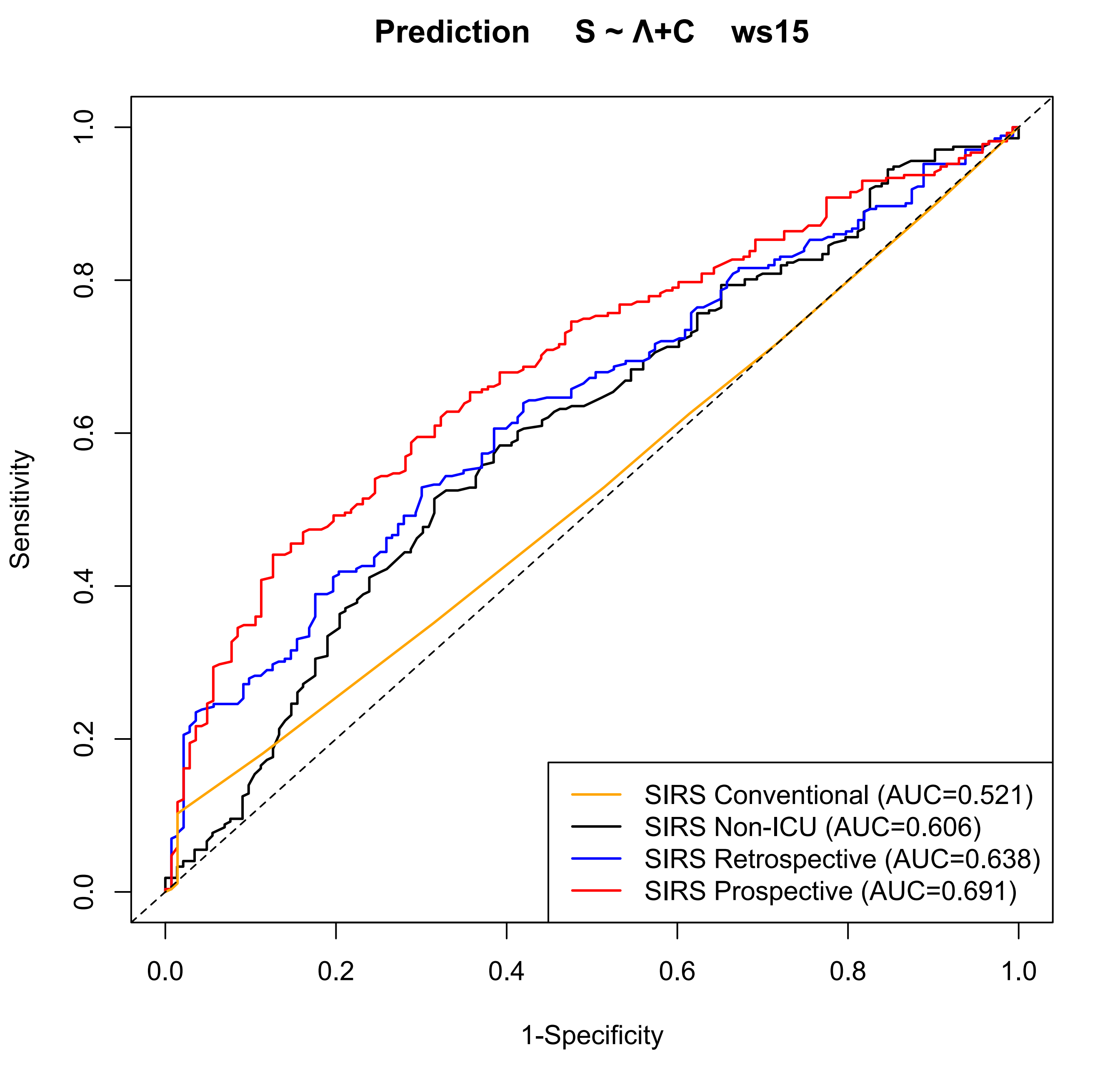
Prediction S ~ Λ+C ws15
Sensitivity
1-Specificity
0.0
0.2
0.4
0.6
0.8
1.0
0.0
0.2
0.4
0.6
0.8
1.0
SIRS Conventional (AUC=0.521)
SIRS Non-ICU (AUC=0.606)
SIRS Retrospective (AUC=0.638)
SIRS Prospective (AUC=0.691)

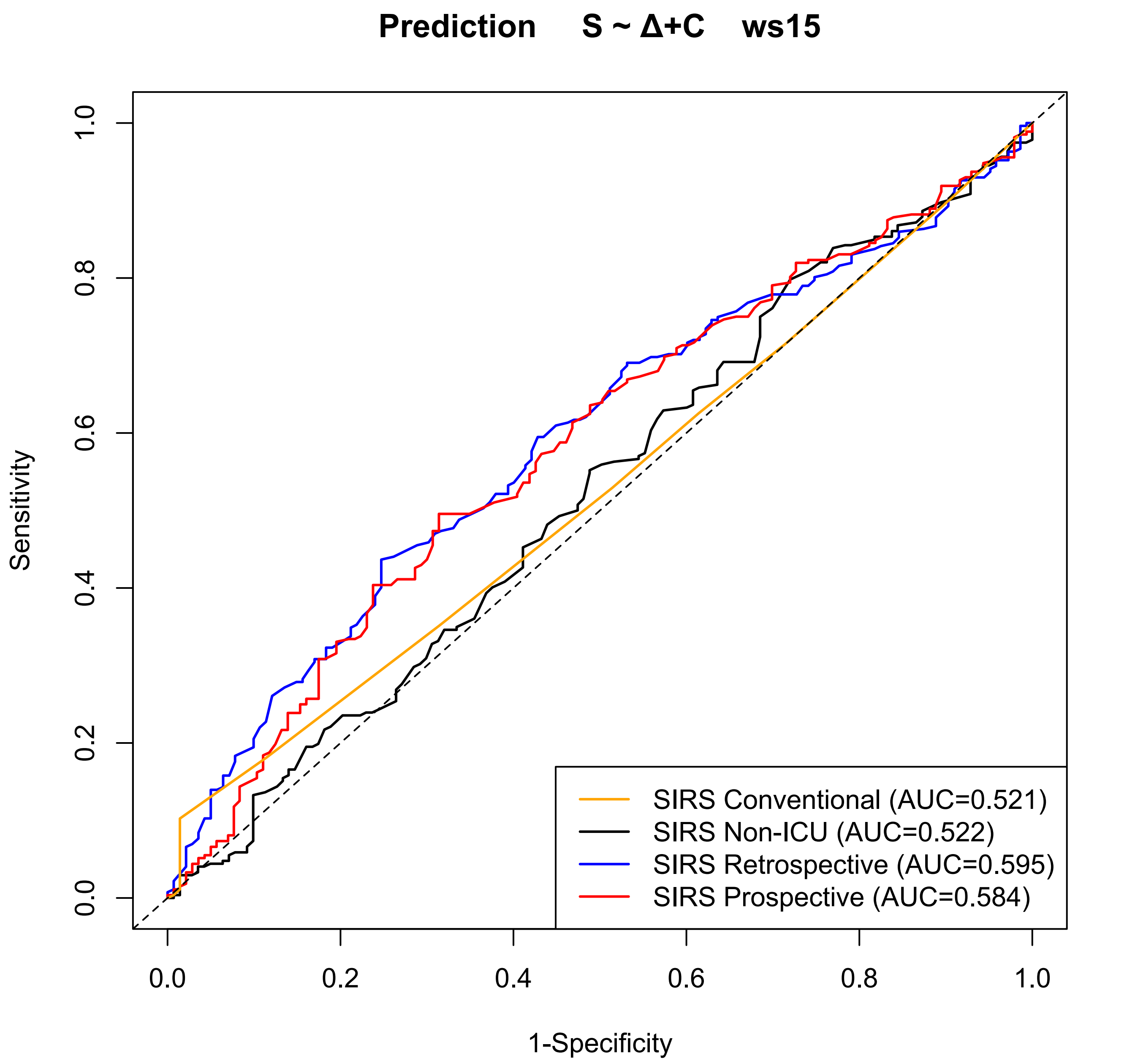

Prediction S ~ Δ+C ws15
Sensitivity
1-Specificity
0.0
0.2
0.4
0.6
0.8
1.0
SIRS Conventional (AUC=0.521)
SIRS Non-ICU (AUC=0.522)
SIRS Retrospective (AUC=0.595)
SIRS Prospective (AUC=0.584)

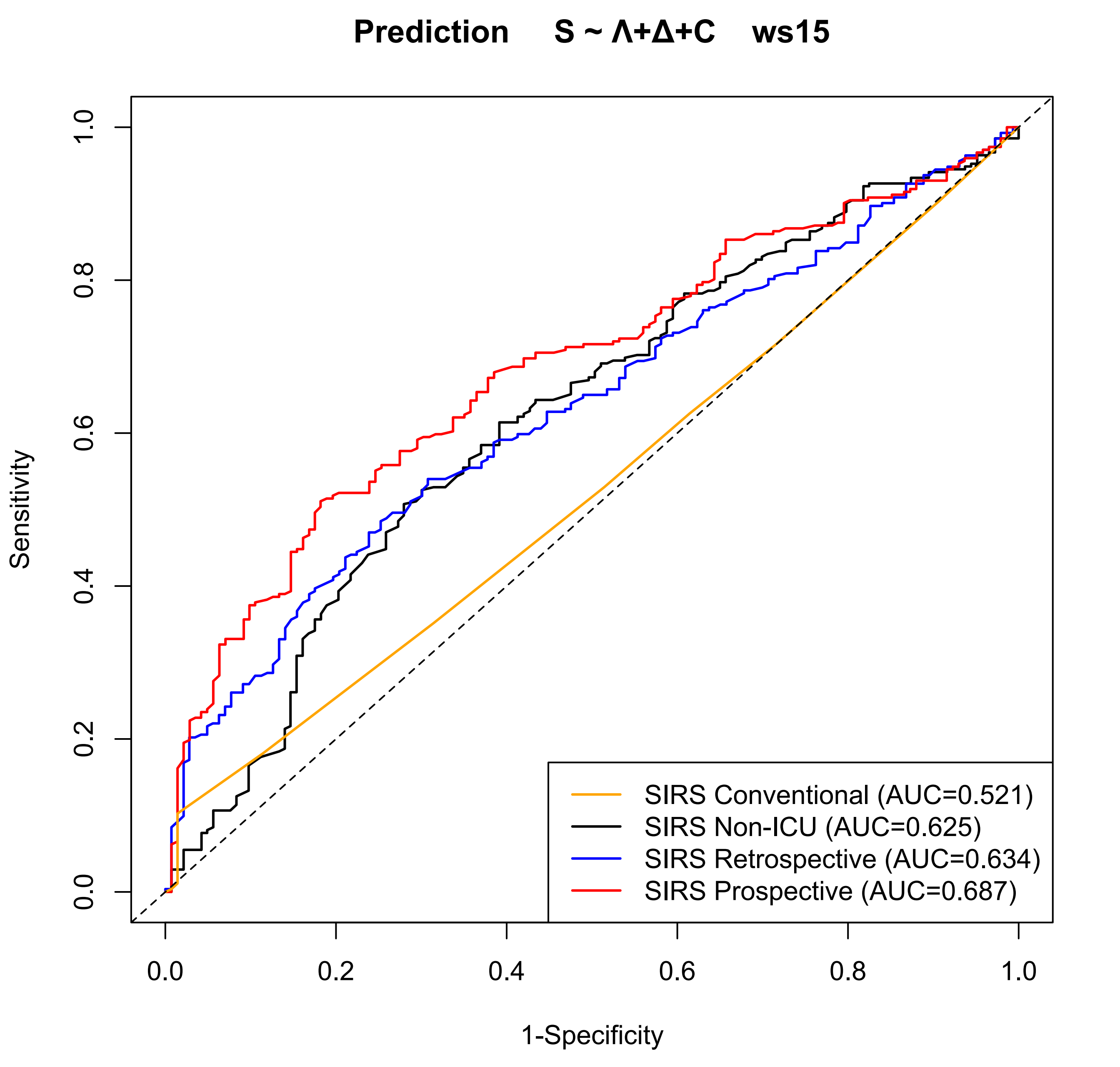
Prediction S ~ Λ+Δ+C ws15
Sensitivity
1-Specificity
0.0
0.2
0.4
0.6
0.8
1.0
SIRS Conventional (AUC=0.521)
SIRS Non-ICU (AUC=0.625)
SIRS Retrospective (AUC=0.634)
SIRS Prospective (AUC=0.687)

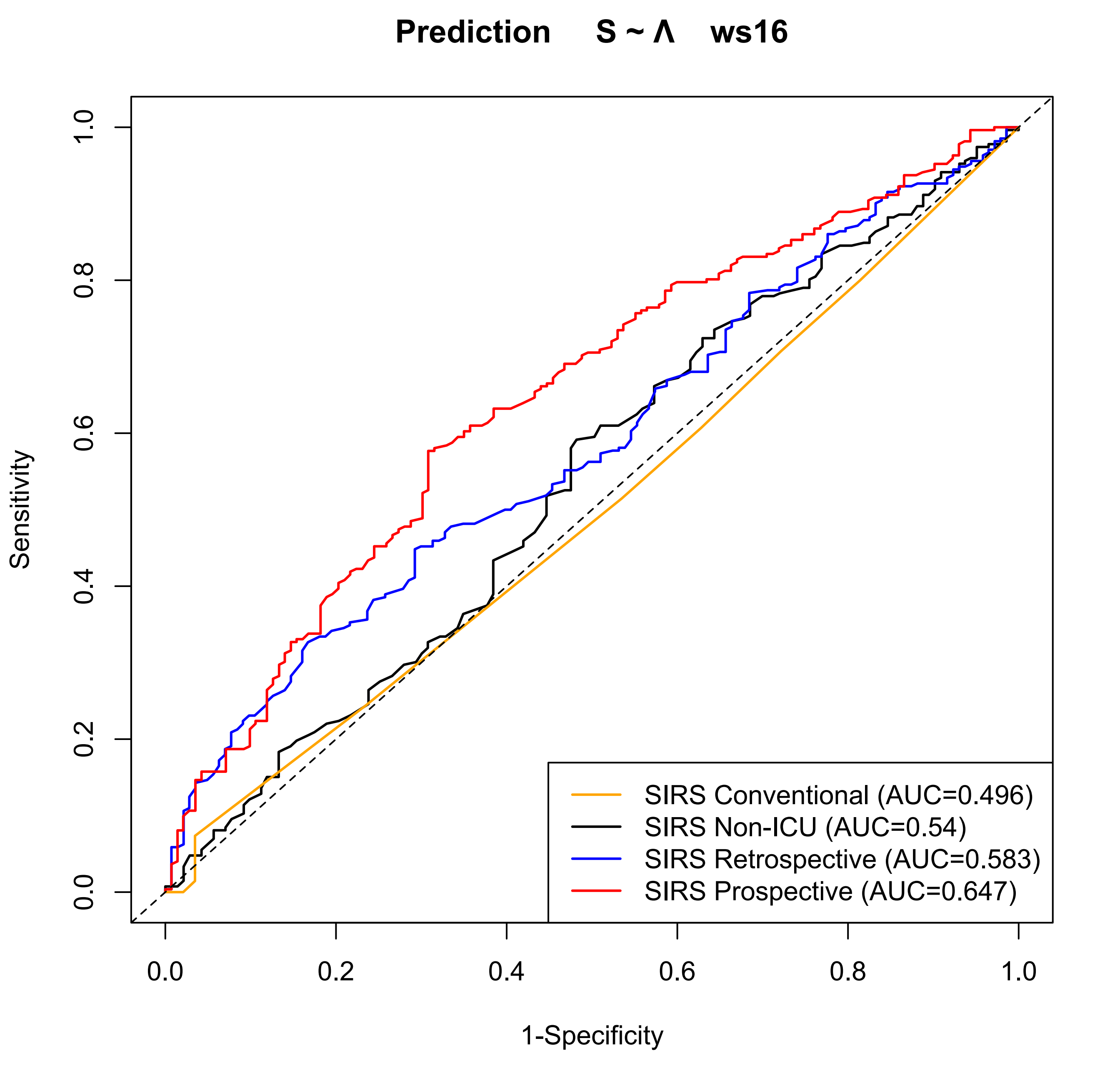
Prediction S ~ Λ ws16
Sensitivity
1-Specificity
0.0
0.2
0.4
0.6
0.8
1.0
SIRS Conventional (AUC=0.496)
SIRS Non-ICU (AUC=0.54)
SIRS Retrospective (AUC=0.583)
SIRS Prospective (AUC=0.647)

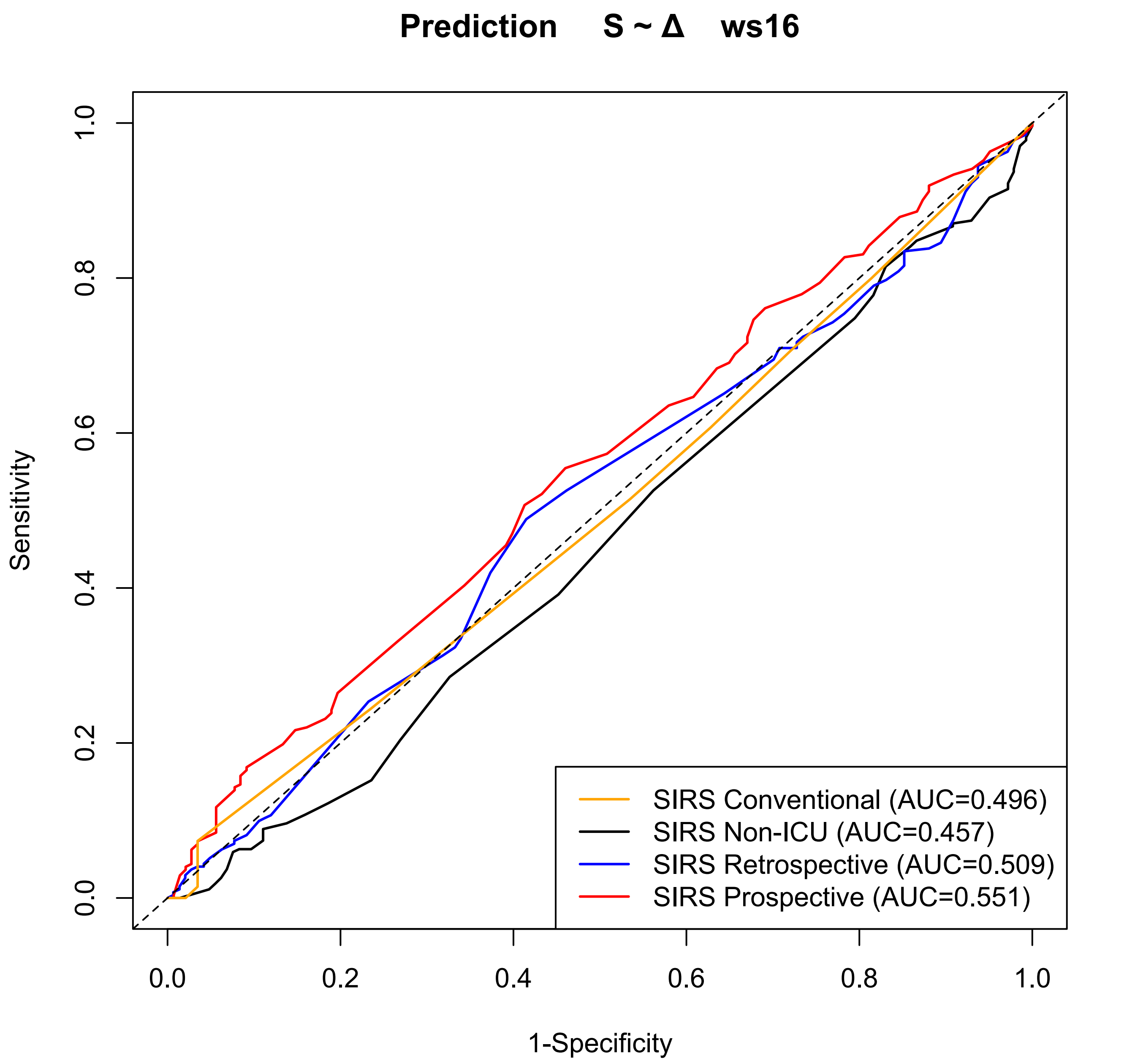
Prediction S ~ Δ ws16
Sensitivity
1-Specificity
0.0
0.2
0.4
0.6
0.8
1.0
SIRS Conventional (AUC=0.496)
SIRS Non-ICU (AUC=0.457)
SIRS Retrospective (AUC=0.509)
SIRS Prospective (AUC=0.551)

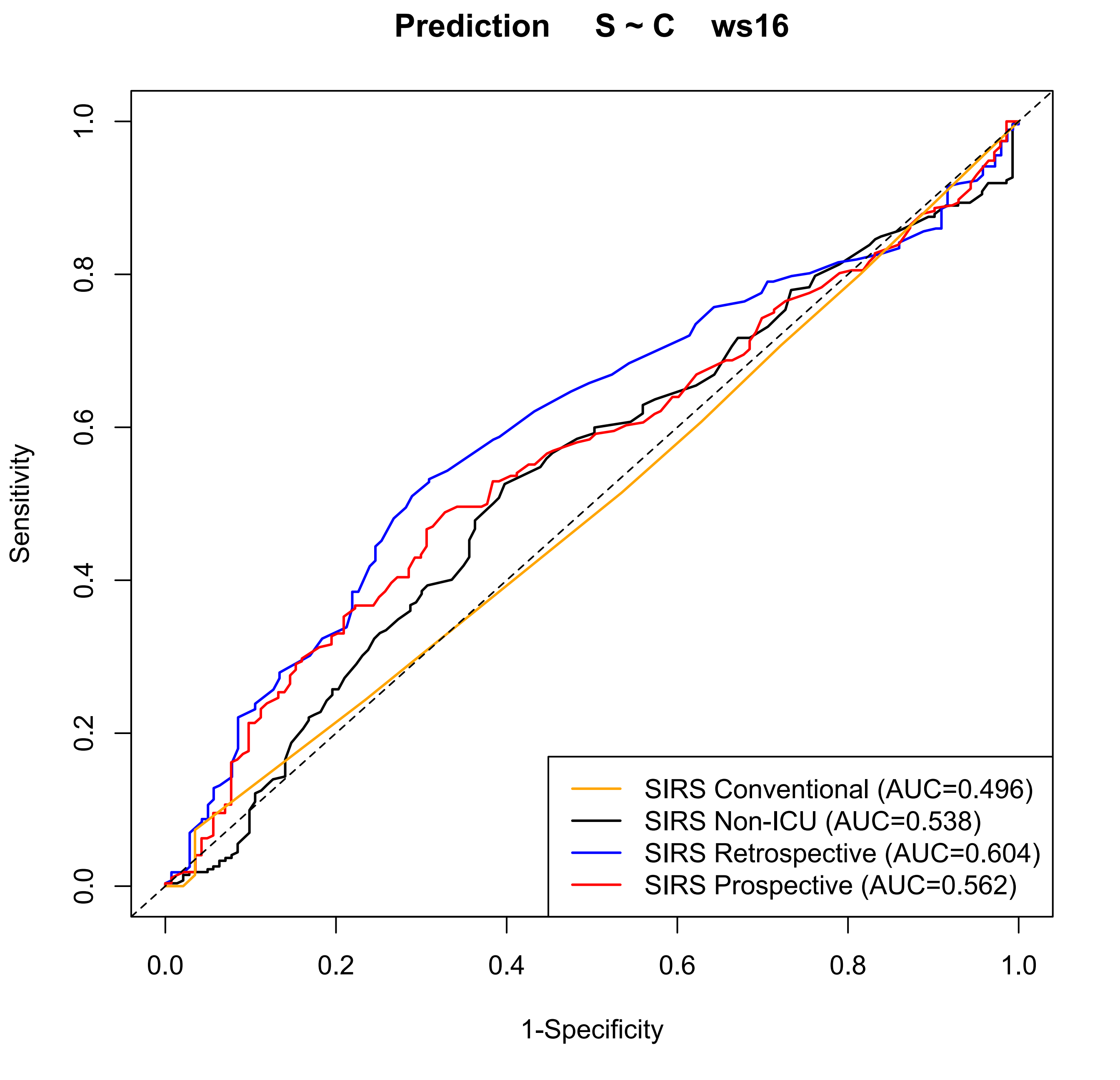

Prediction S ~ C ws16
Sensitivity
1-Specificity
0.0
0.2
0.4
0.6
0.8
1.0
SIRS Conventional (AUC=0.496)
SIRS Non-ICU (AUC=0.538)
SIRS Retrospective (AUC=0.604)
SIRS Prospective (AUC=0.562)

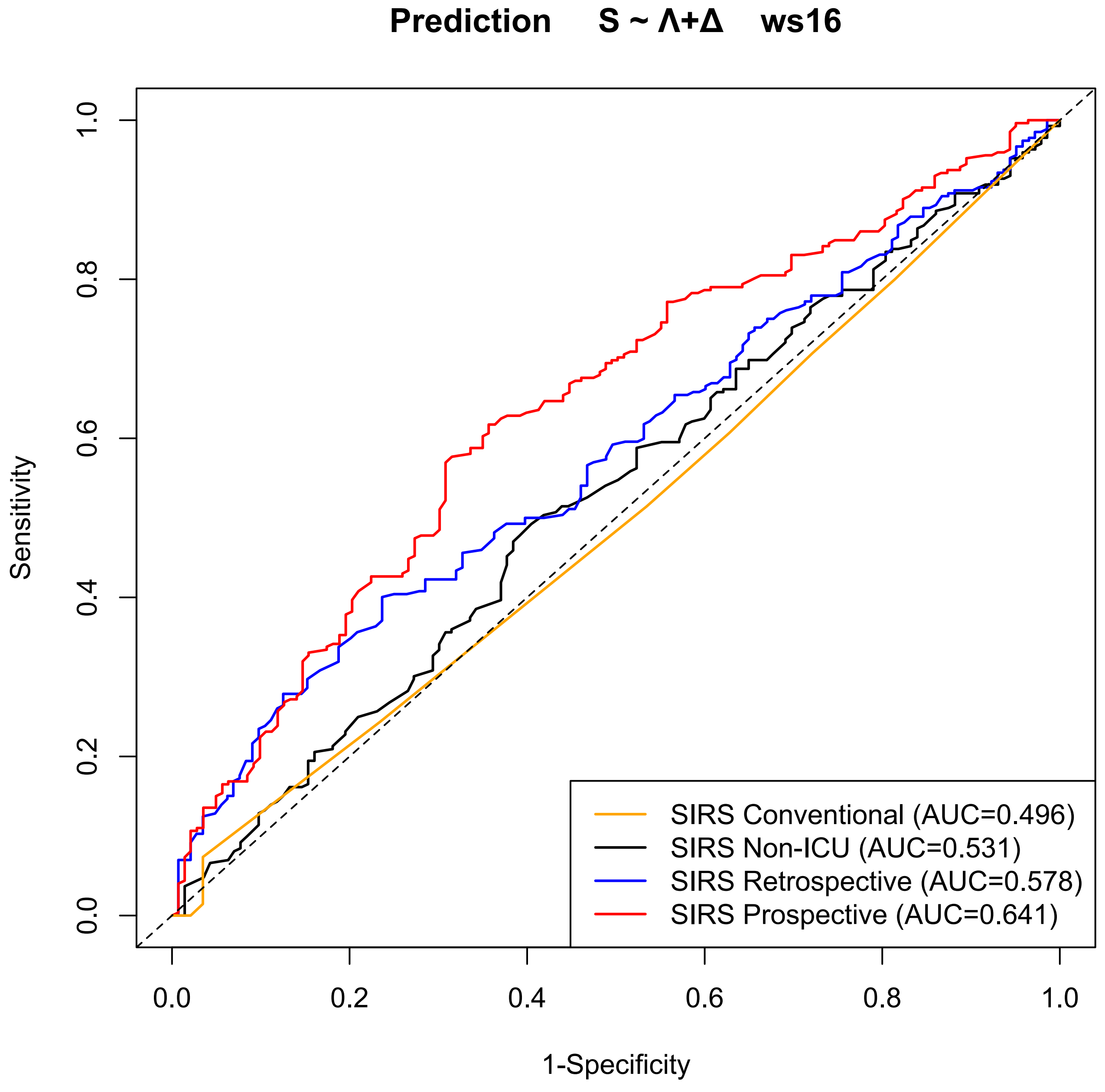

Prediction S ~ Λ+Δ ws16
Sensitivity
1-Specificity
0.0
0.2
0.4
0.6
0.8
1.0
0.0
0.2
0.4
0.6
0.8
1.0
SIRS Conventional (AUC=0.496)
SIRS Non-ICU (AUC=0.531)
SIRS Retrospective (AUC=0.578)
SIRS Prospective (AUC=0.641)

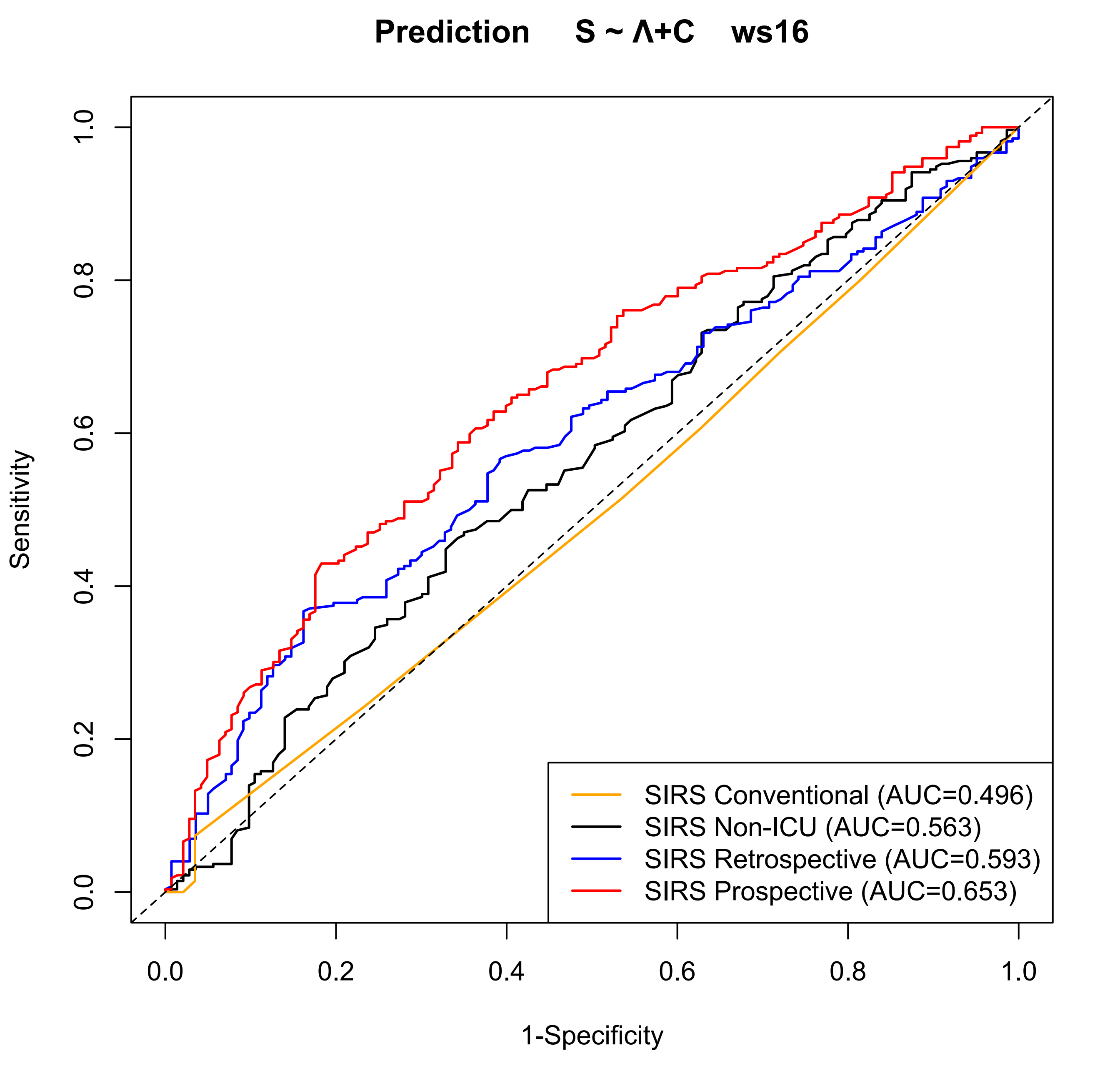

Prediction S ~ Λ+C ws16
Sensitivity
1-Specificity
0.0
0.2
0.4
0.6
0.8
1.0
0.0
0.2
0.4
0.6
0.8
1.0
SIRS Conventional (AUC=0.496)
SIRS Non-ICU (AUC=0.563)
SIRS Retrospective (AUC=0.593)
SIRS Prospective (AUC=0.653)

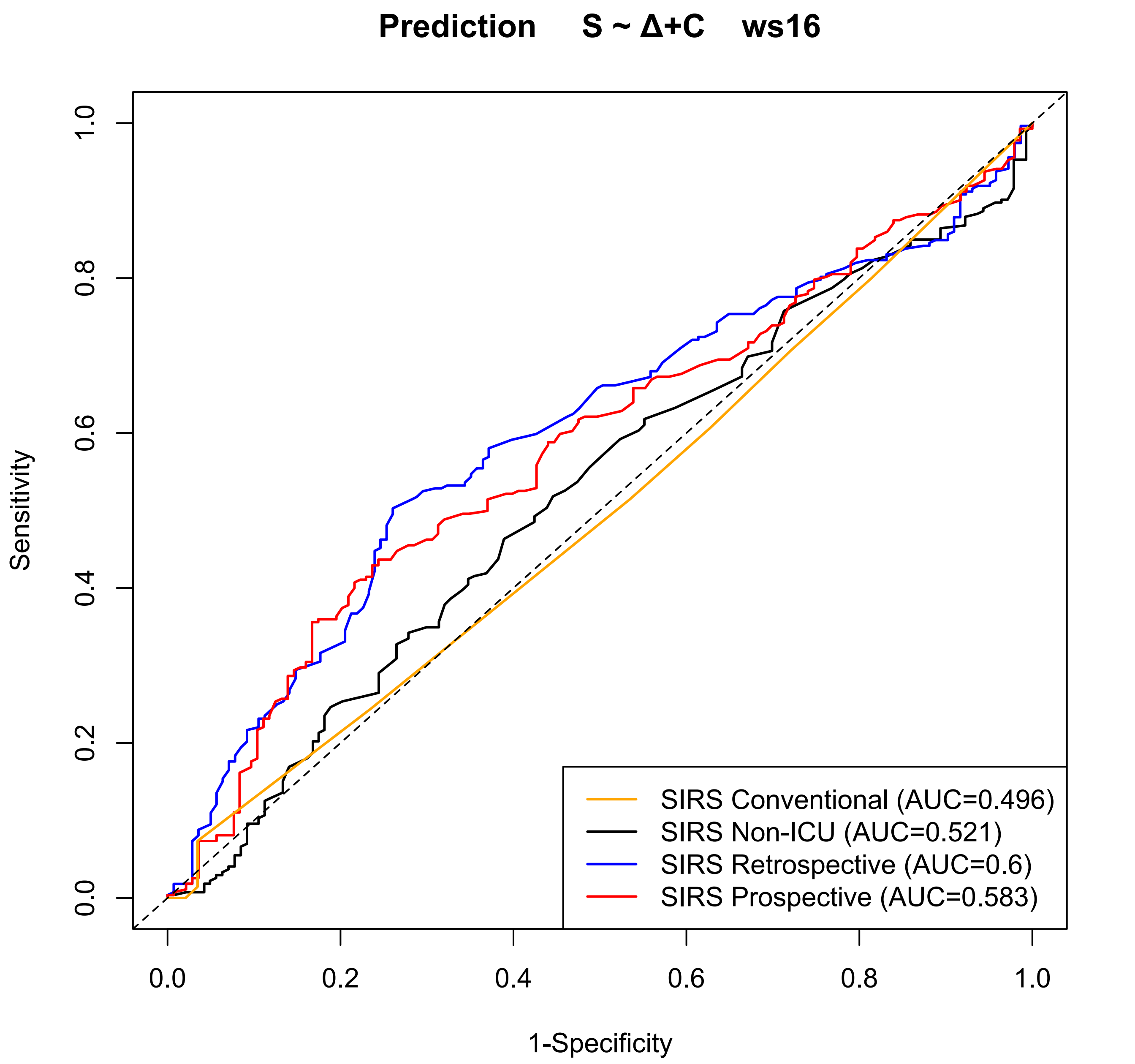

Prediction S ~ Δ+C ws16
Sensitivity
1-Specificity
0.0
0.2
0.4
0.6
0.8
1.0
SIRS Conventional (AUC=0.496)
SIRS Non-ICU (AUC=0.521)
SIRS Retrospective (AUC=0.6)
SIRS Prospective (AUC=0.583)

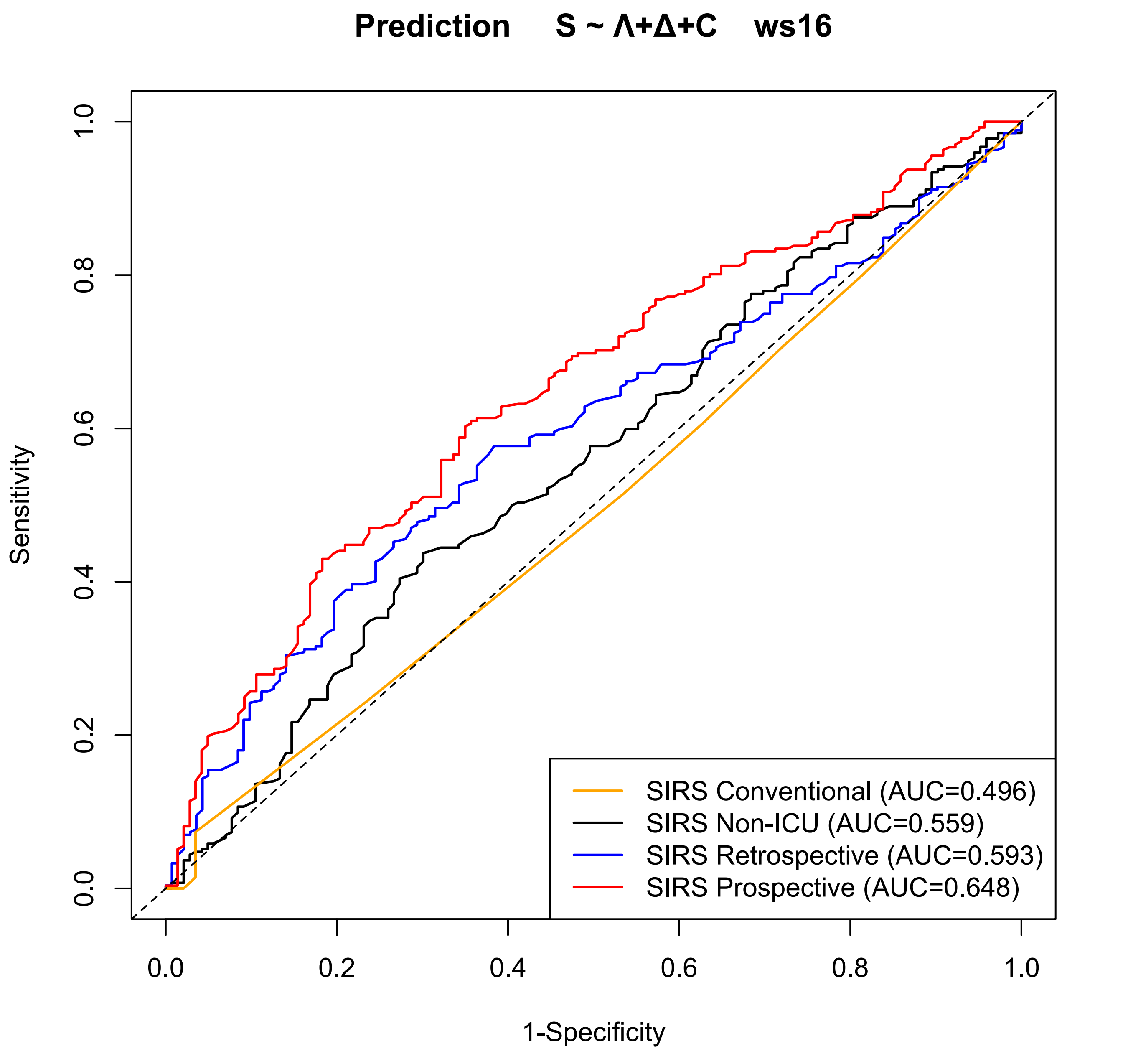

Prediction S ~ Λ+Δ+C ws16
Sensitivity
1-Specificity
0.0
0.2
0.4
0.6
0.8
1.0
SIRS Conventional (AUC=0.496)
SIRS Non-ICU (AUC=0.559)
SIRS Retrospective (AUC=0.593)
SIRS Prospective (AUC=0.648)

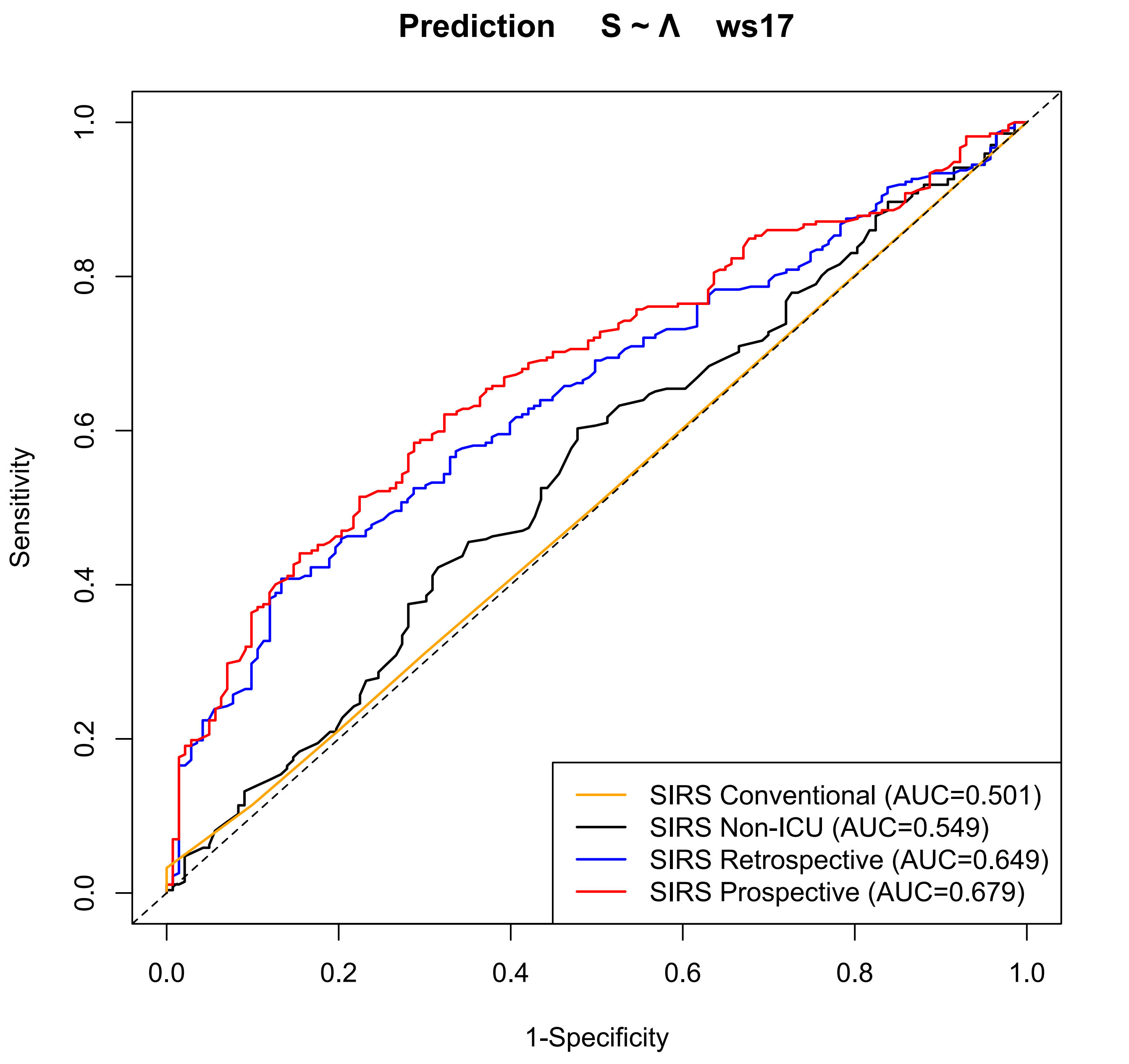
Prediction S ~ Λ ws17
Sensitivity
1-Specificity
0.0
0.2
0.4
0.6
0.8
1.0
SIRS Conventional (AUC=0.501)
SIRS Non-ICU (AUC=0.549)
SIRS Retrospective (AUC=0.649)
SIRS Prospective (AUC=0.679)

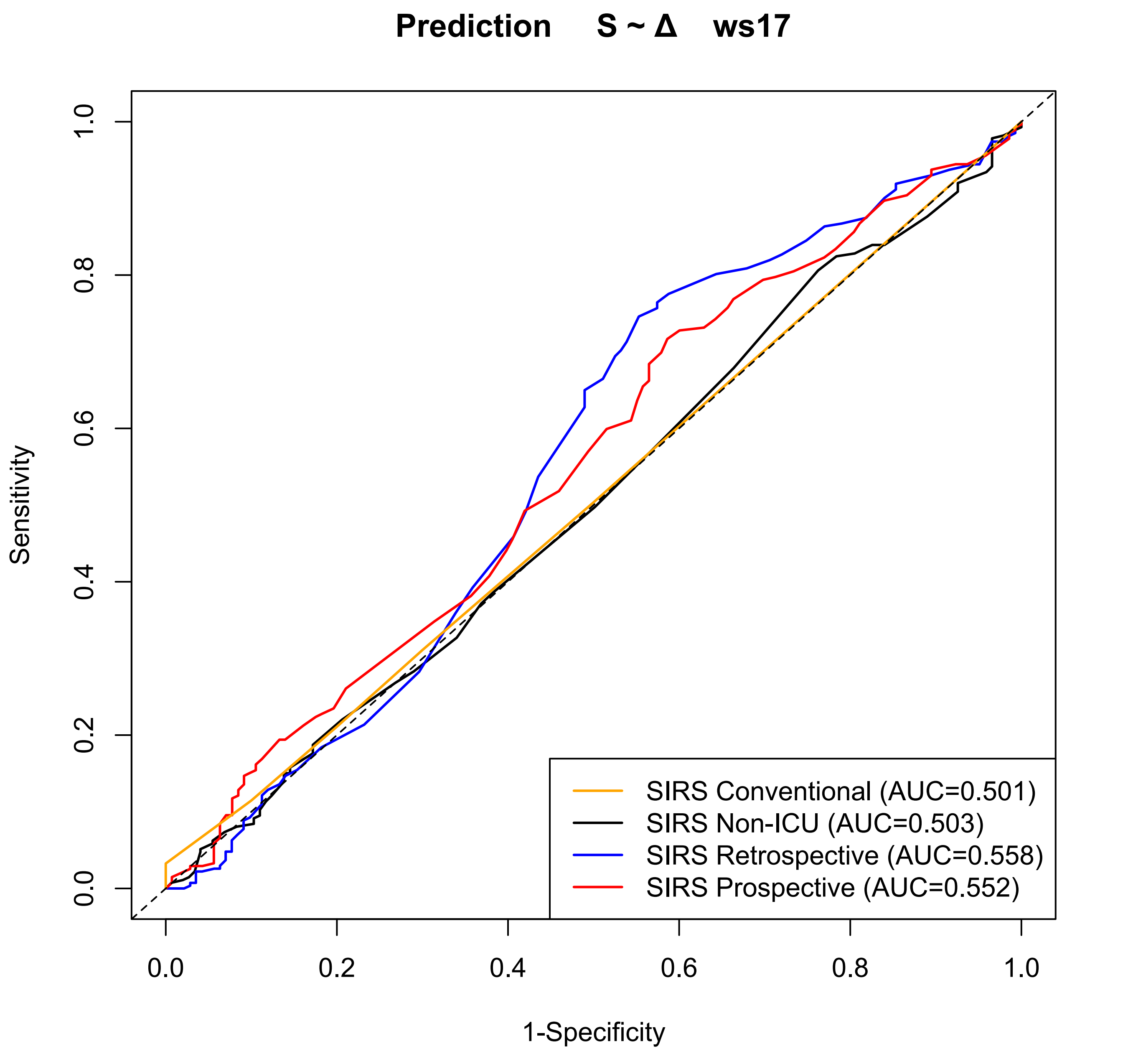

Prediction S ~ Δ ws17
Sensitivity
1-Specificity
0.0
0.2
0.4
0.6
0.8
1.0
SIRS Conventional (AUC=0.501)
SIRS Non-ICU (AUC=0.503)
SIRS Retrospective (AUC=0.558)
SIRS Prospective (AUC=0.552)

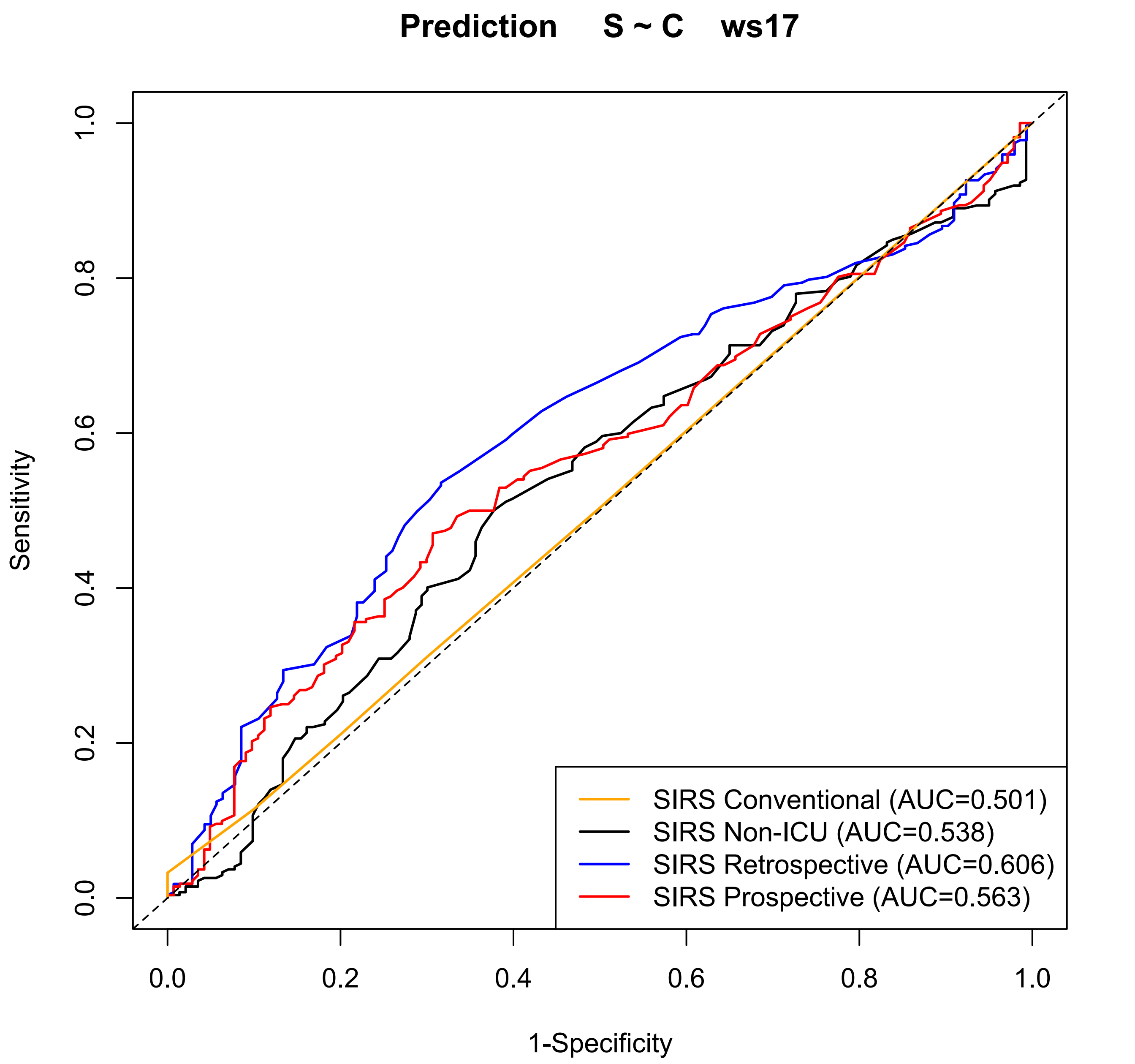

Prediction S ~ C ws17
Sensitivity
1-Specificity
0.0
0.2
0.4
0.6
0.8
1.0
SIRS Conventional (AUC=0.501)
SIRS Non-ICU (AUC=0.538)
SIRS Retrospective (AUC=0.606)
SIRS Prospective (AUC=0.563)

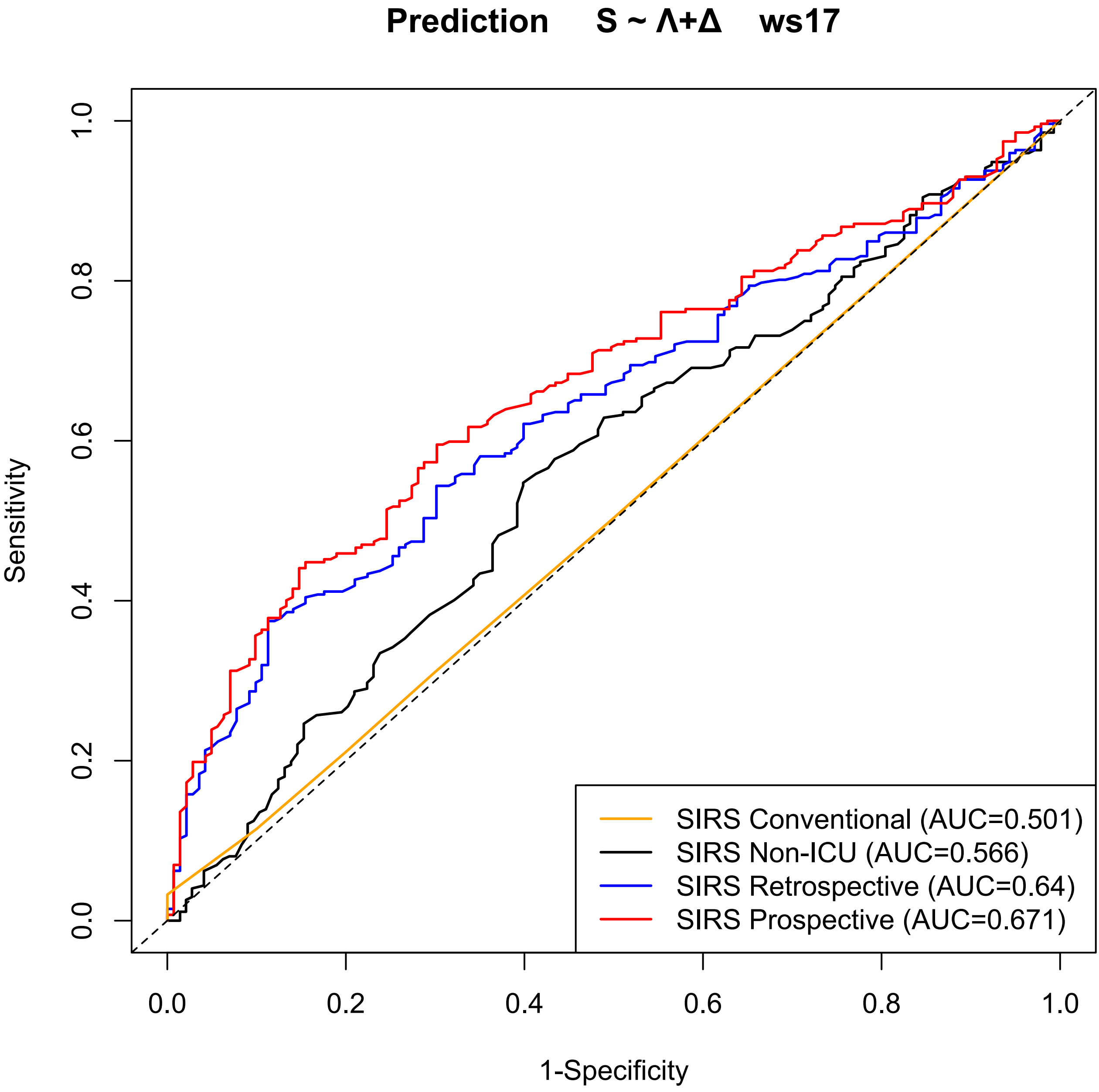

Prediction S ~ Λ+Δ ws17
Sensitivity
1-Specificity
0.0
0.2
0.4
0.6
0.8
1.0
0.0
0.2
0.4
0.6
0.8
1.0
SIRS Conventional (AUC=0.501)
SIRS Non-ICU (AUC=0.566)
SIRS Retrospective (AUC=0.64)
SIRS Prospective (AUC=0.671)

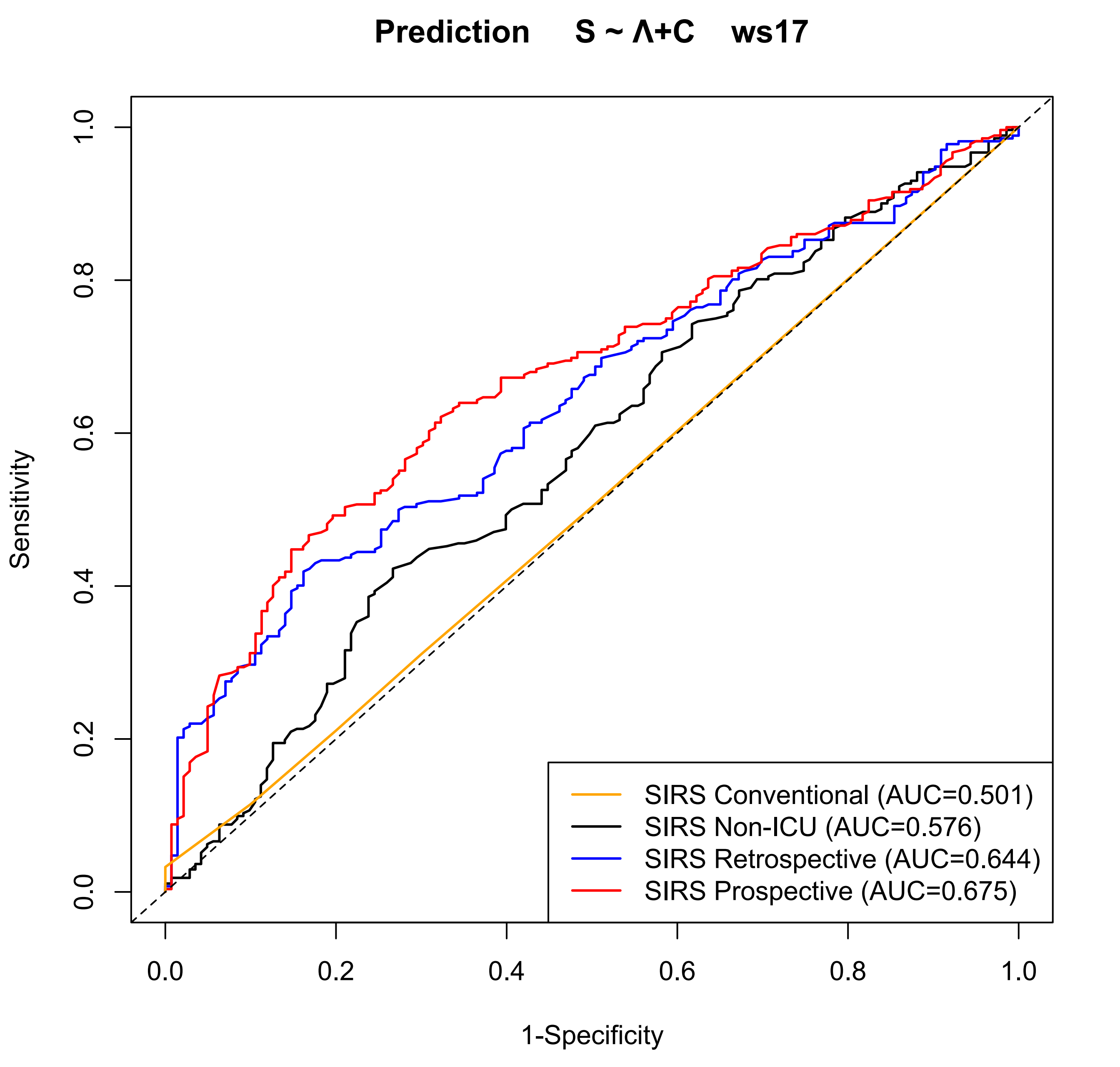

Prediction S ~ Λ+C ws17
Sensitivity
1-Specificity
0.0
0.2
0.4
0.6
0.8
1.0
SIRS Conventional (AUC=0.501)
SIRS Non-ICU (AUC=0.576)
SIRS Retrospective (AUC=0.644)
SIRS Prospective (AUC=0.675)

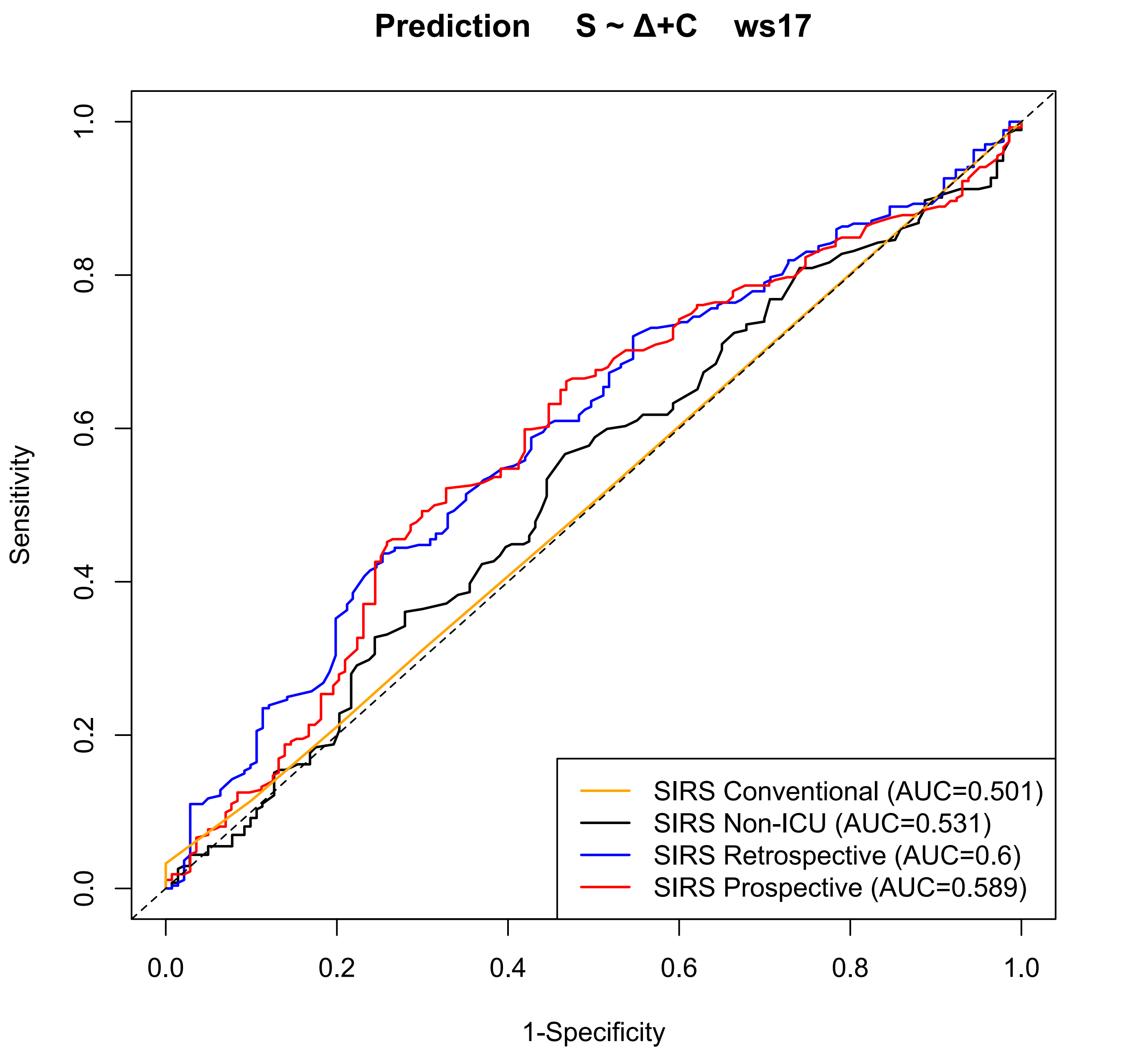

Prediction S ~ Δ+C ws17
Sensitivity
1-Specificity
0.0
0.2
0.4
0.6
0.8
1.0
0.0
0.2
0.4
0.6
0.8
1.0
SIRS Conventional (AUC=0.501)
SIRS Non-ICU (AUC=0.531)
SIRS Retrospective (AUC=0.6)
SIRS Prospective (AUC=0.589)

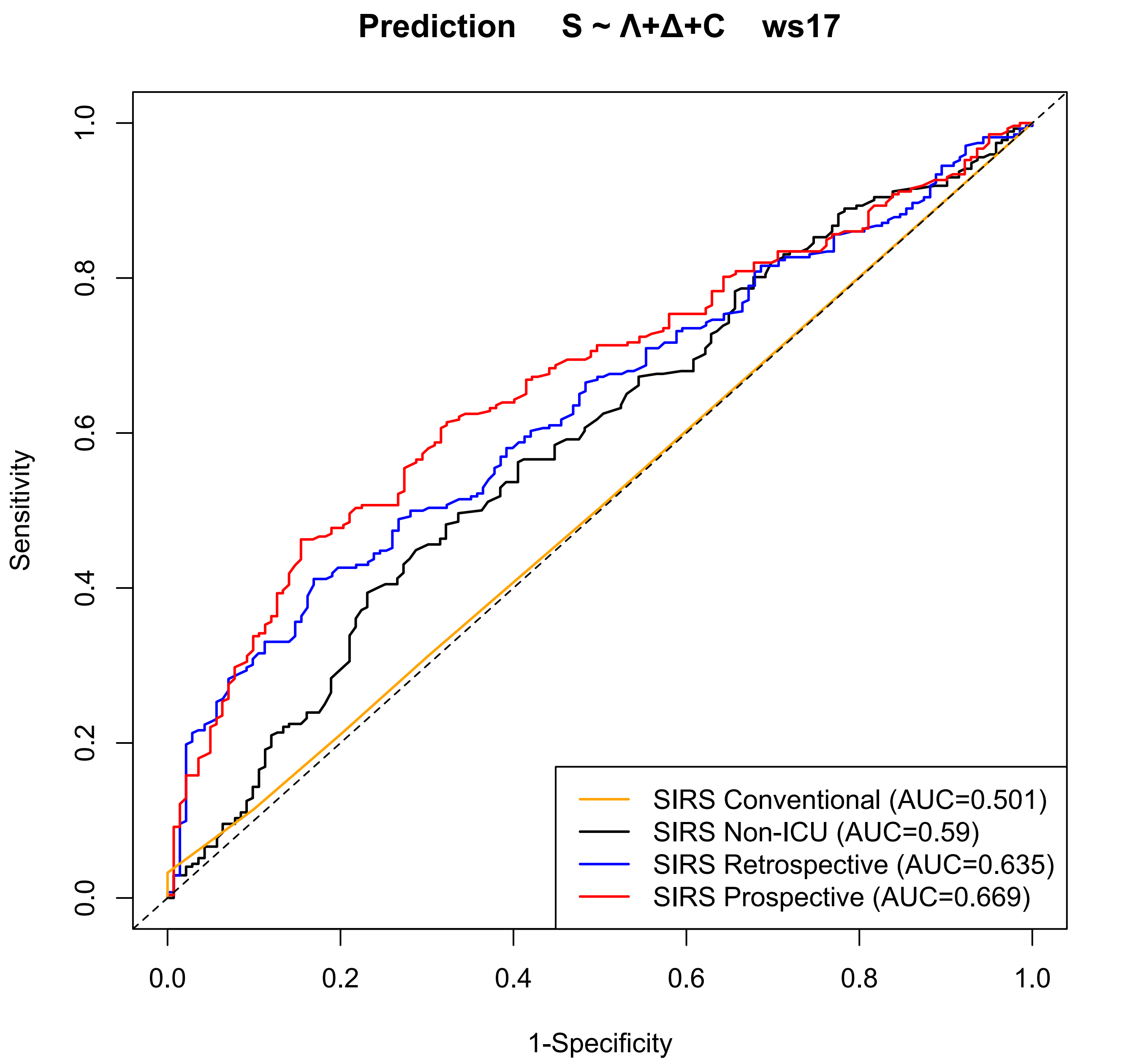

Prediction S ~ Λ+Δ+C ws17
Sensitivity
1-Specificity
0.0
0.2
0.4
0.6
0.8
1.0
SIRS Conventional (AUC=0.501)
SIRS Non-ICU (AUC=0.59)
SIRS Retrospective (AUC=0.635)
SIRS Prospective (AUC=0.669)

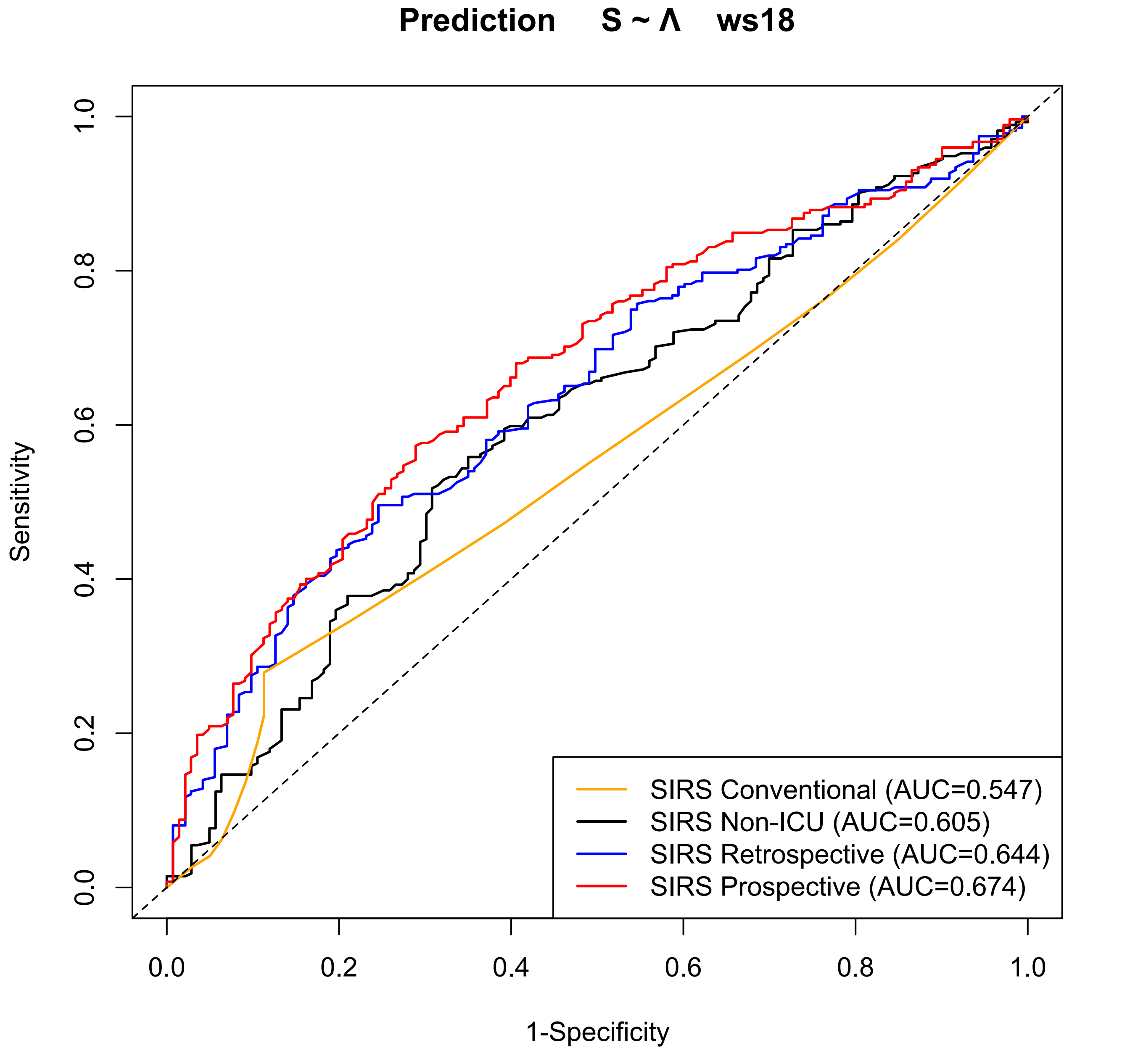
Prediction S ~ Λ ws18
Sensitivity
1-Specificity
0.0
0.2
0.4
0.6
0.8
1.0
SIRS Conventional (AUC=0.547)
SIRS Non-ICU (AUC=0.605)
SIRS Retrospective (AUC=0.644)
SIRS Prospective (AUC=0.674)

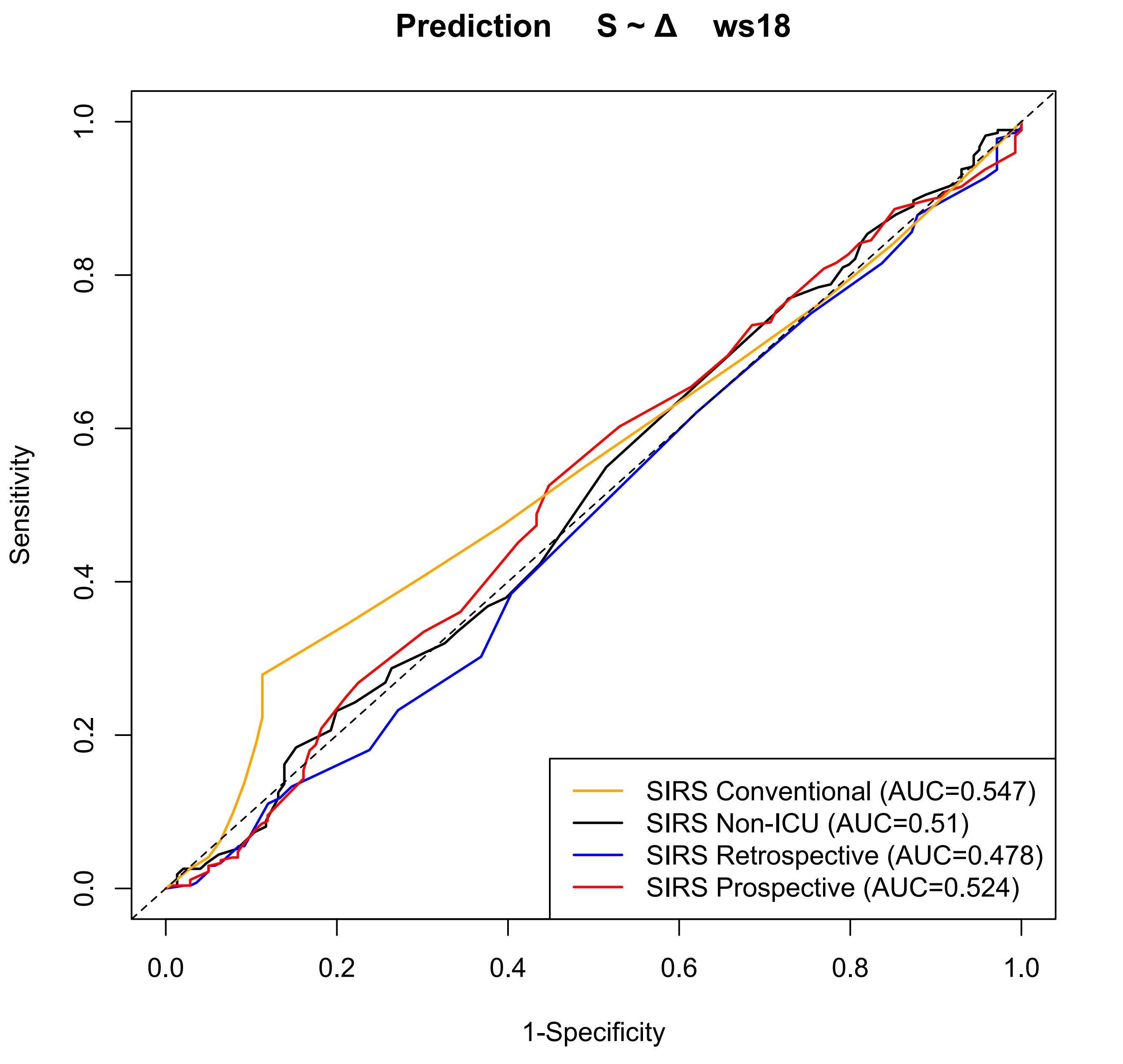

Prediction S ~ Δ ws18
Sensitivity
1-Specificity
0.0
0.2
0.4
0.6
0.8
1.0
0.0
0.2
0.4
0.6
0.8
1.0
SIRS Conventional (AUC=0.547)
SIRS Non-ICU (AUC=0.51)
SIRS Retrospective (AUC=0.478)
SIRS Prospective (AUC=0.524)

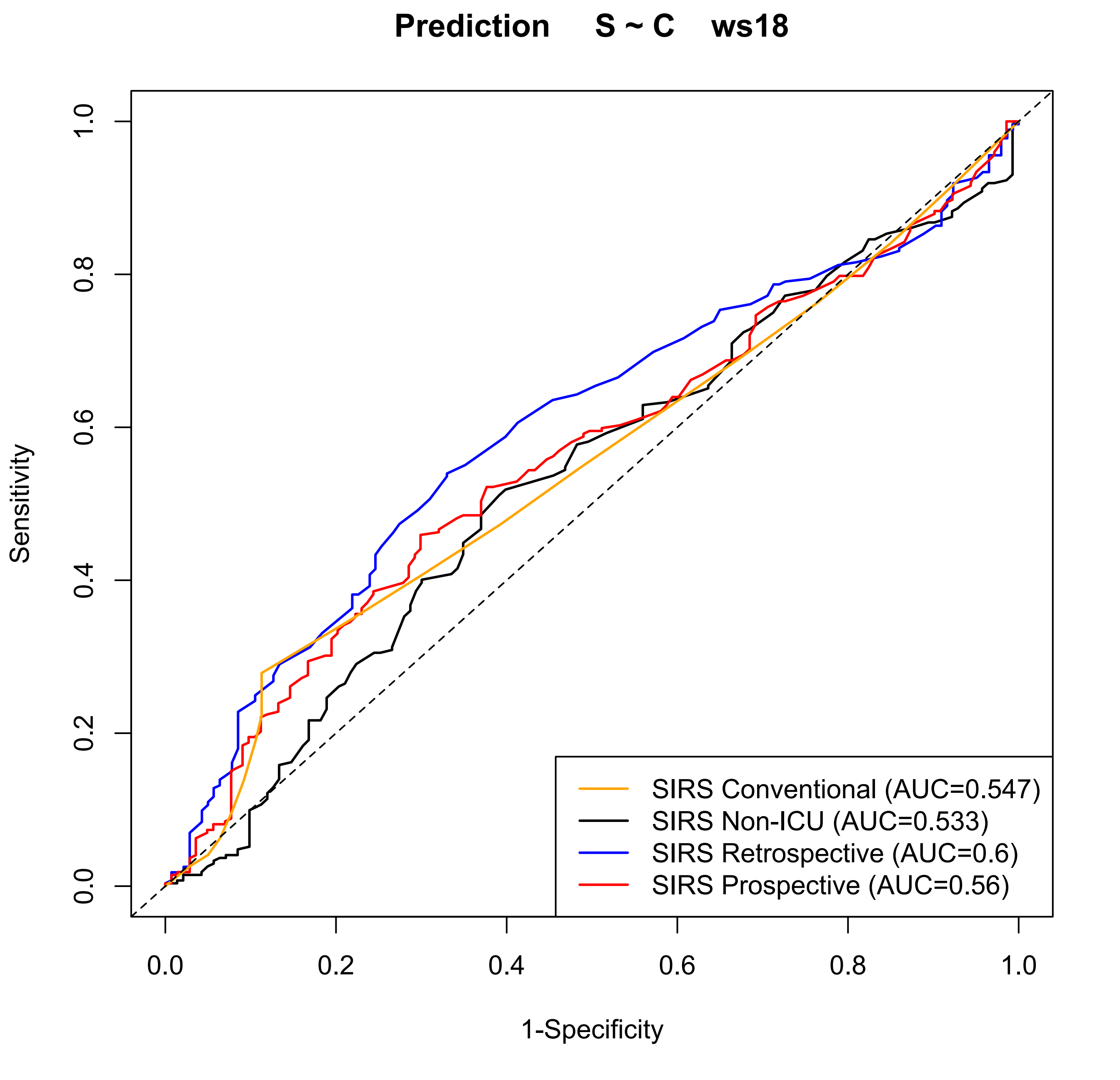

Prediction S ~ C ws18
Sensitivity
1-Specificity
0.0
0.2
0.4
0.6
0.8
1.0
SIRS Conventional (AUC=0.547)
SIRS Non-ICU (AUC=0.533)
SIRS Retrospective (AUC=0.6)
SIRS Prospective (AUC=0.56)

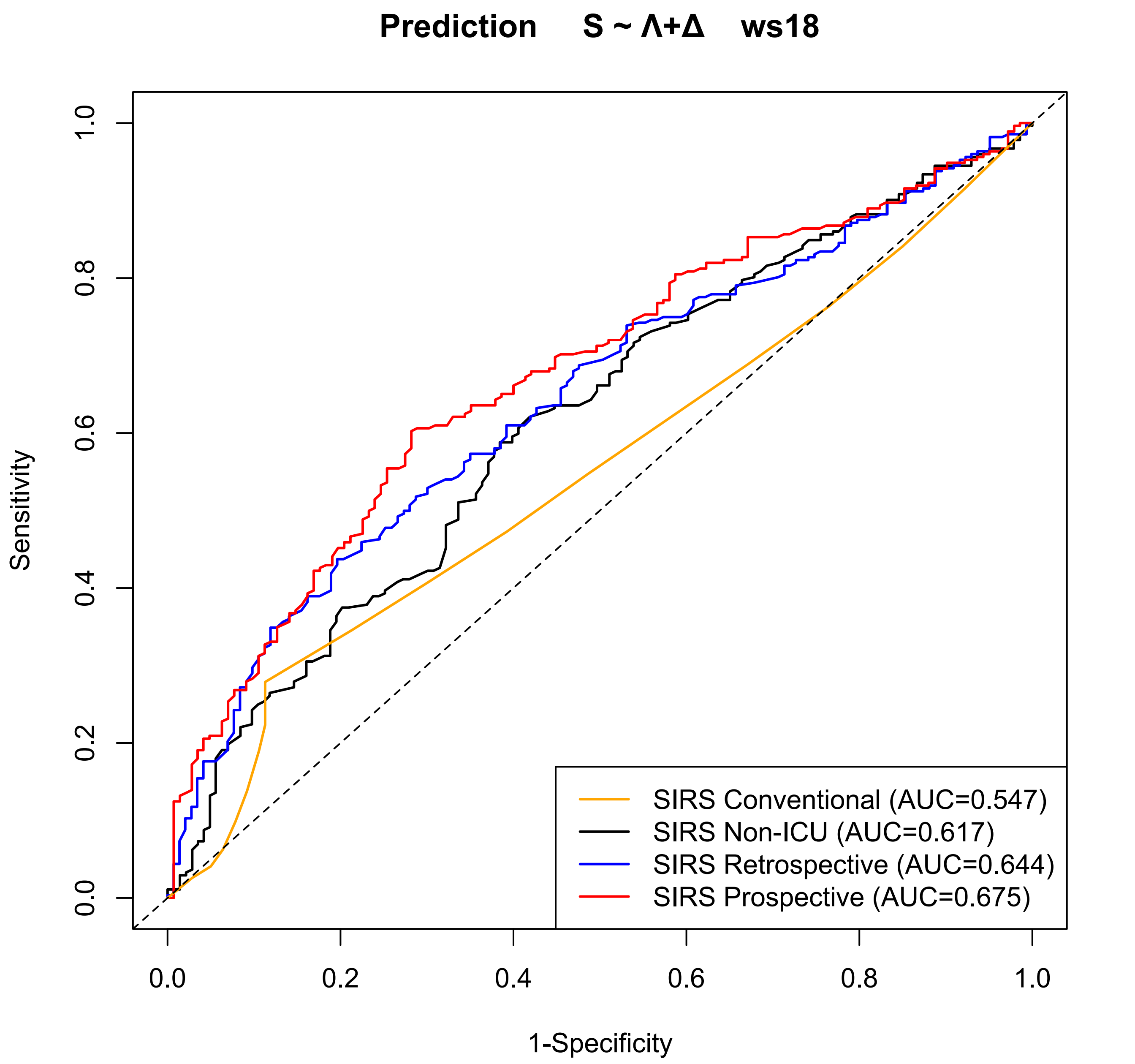

Prediction S ~ Λ+Δ ws18
Sensitivity
1-Specificity
0.0
0.2
0.4
0.6
0.8
1.0
SIRS Conventional (AUC=0.547)
SIRS Non-ICU (AUC=0.617)
SIRS Retrospective (AUC=0.644)
SIRS Prospective (AUC=0.675)

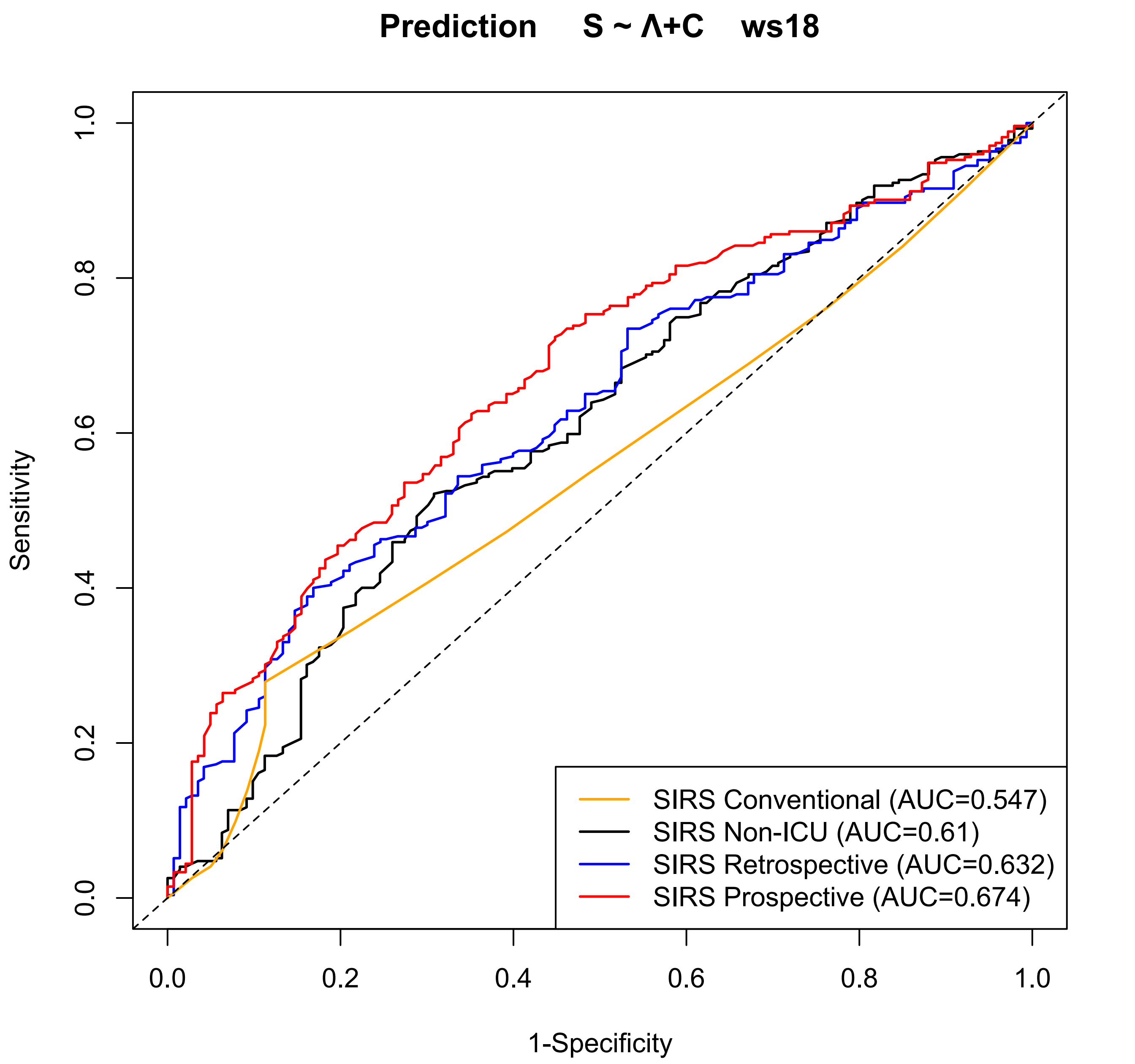
Prediction S ~ Λ+C ws18
Sensitivity
1-Specificity
0.0
0.2
0.4
0.6
0.8
1.0
SIRS Conventional (AUC=0.547)
SIRS Non-ICU (AUC=0.61)
SIRS Retrospective (AUC=0.632)
SIRS Prospective (AUC=0.674)

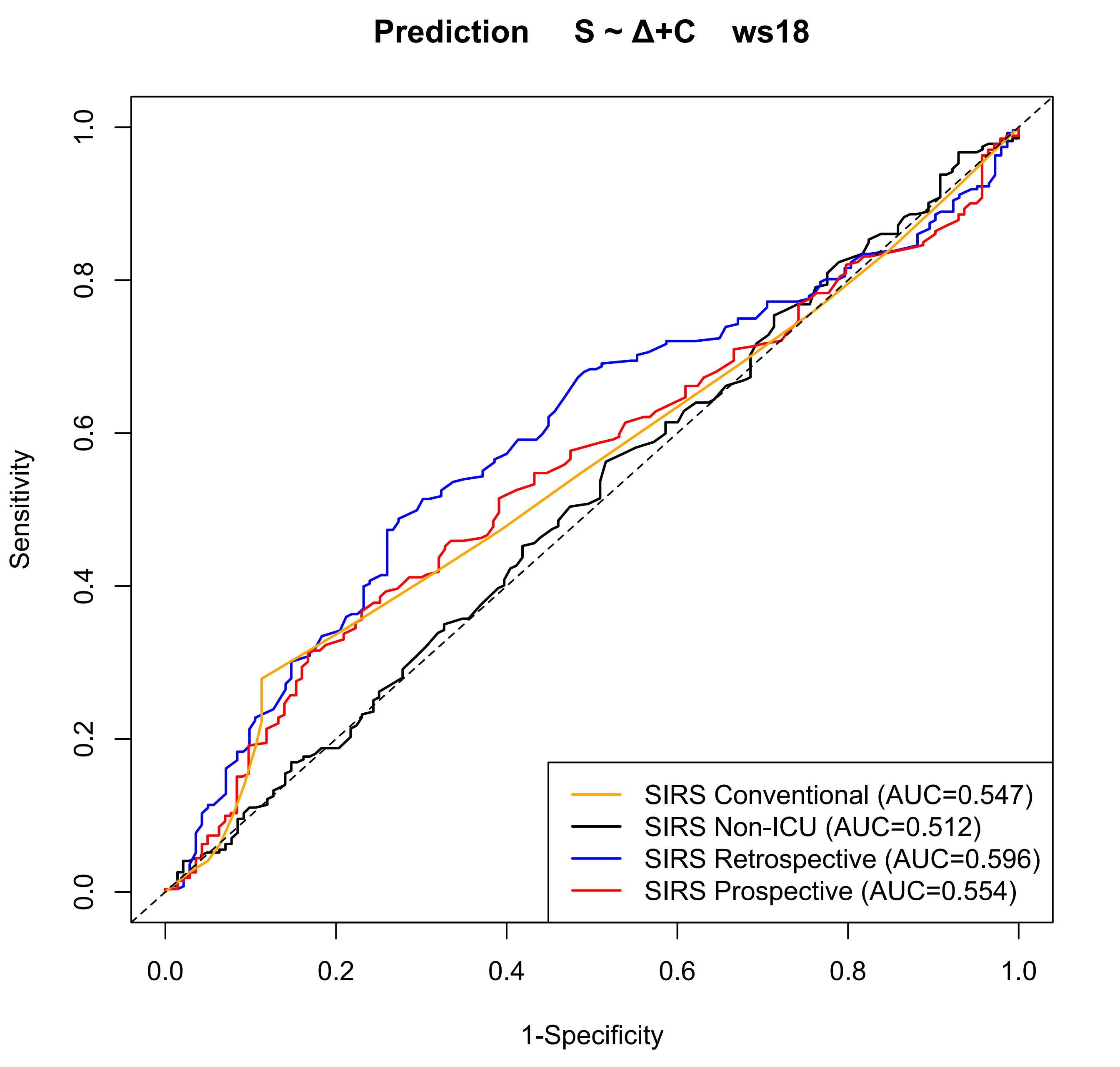

Prediction S ~ Δ+C ws18
Sensitivity
1-Specificity
0.0
0.2
0.4
0.6
0.8
1.0
SIRS Conventional (AUC=0.547)
SIRS Non-ICU (AUC=0.512)
SIRS Retrospective (AUC=0.596)
SIRS Prospective (AUC=0.554)

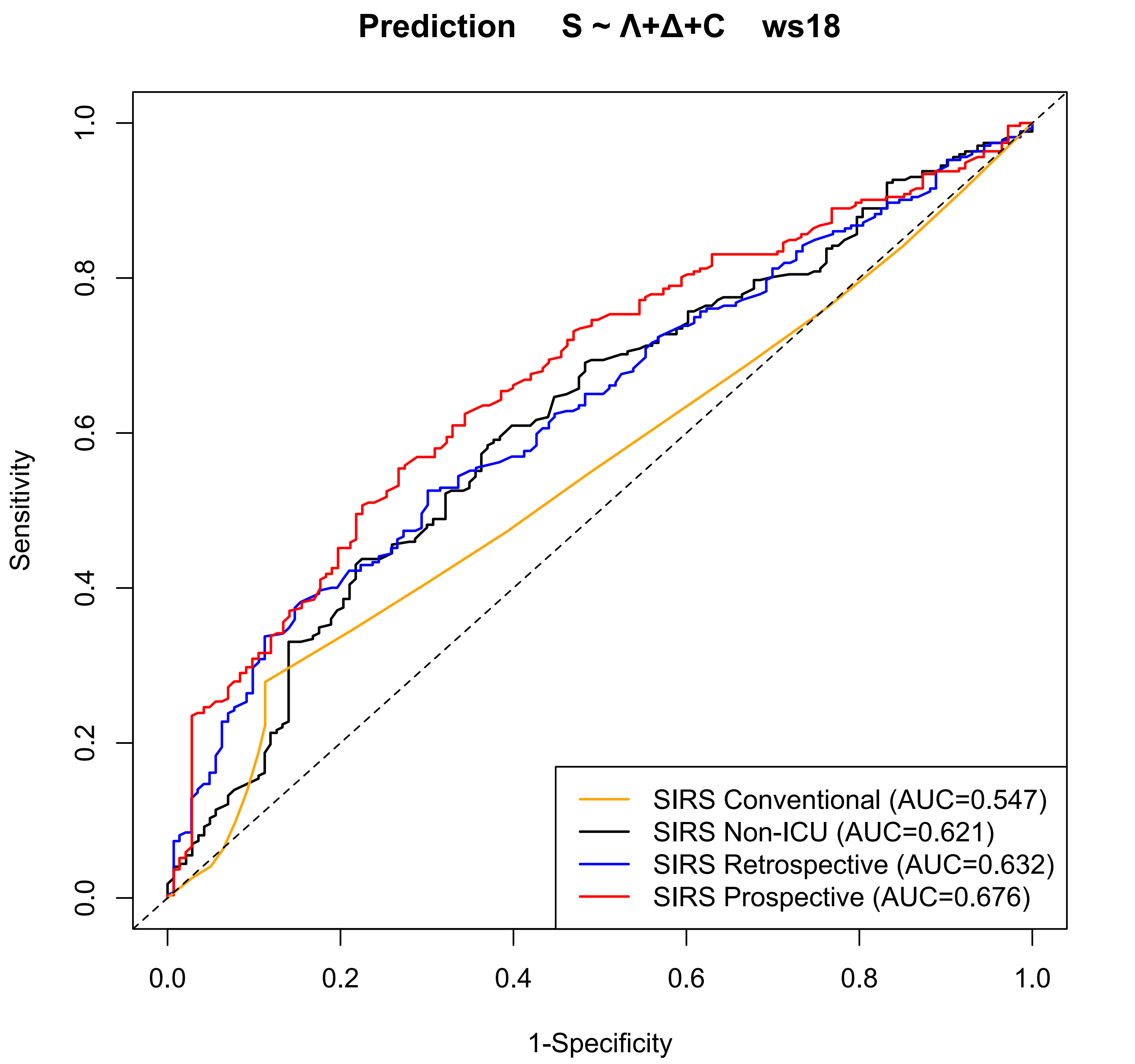

Prediction S ~ Λ+Δ+C ws18
Sensitivity
1-Specificity
0.0
0.2
0.4
0.6
0.8
1.0
SIRS Conventional (AUC=0.547)
SIRS Non-ICU (AUC=0.621)
SIRS Retrospective (AUC=0.632)
SIRS Prospective (AUC=0.676)

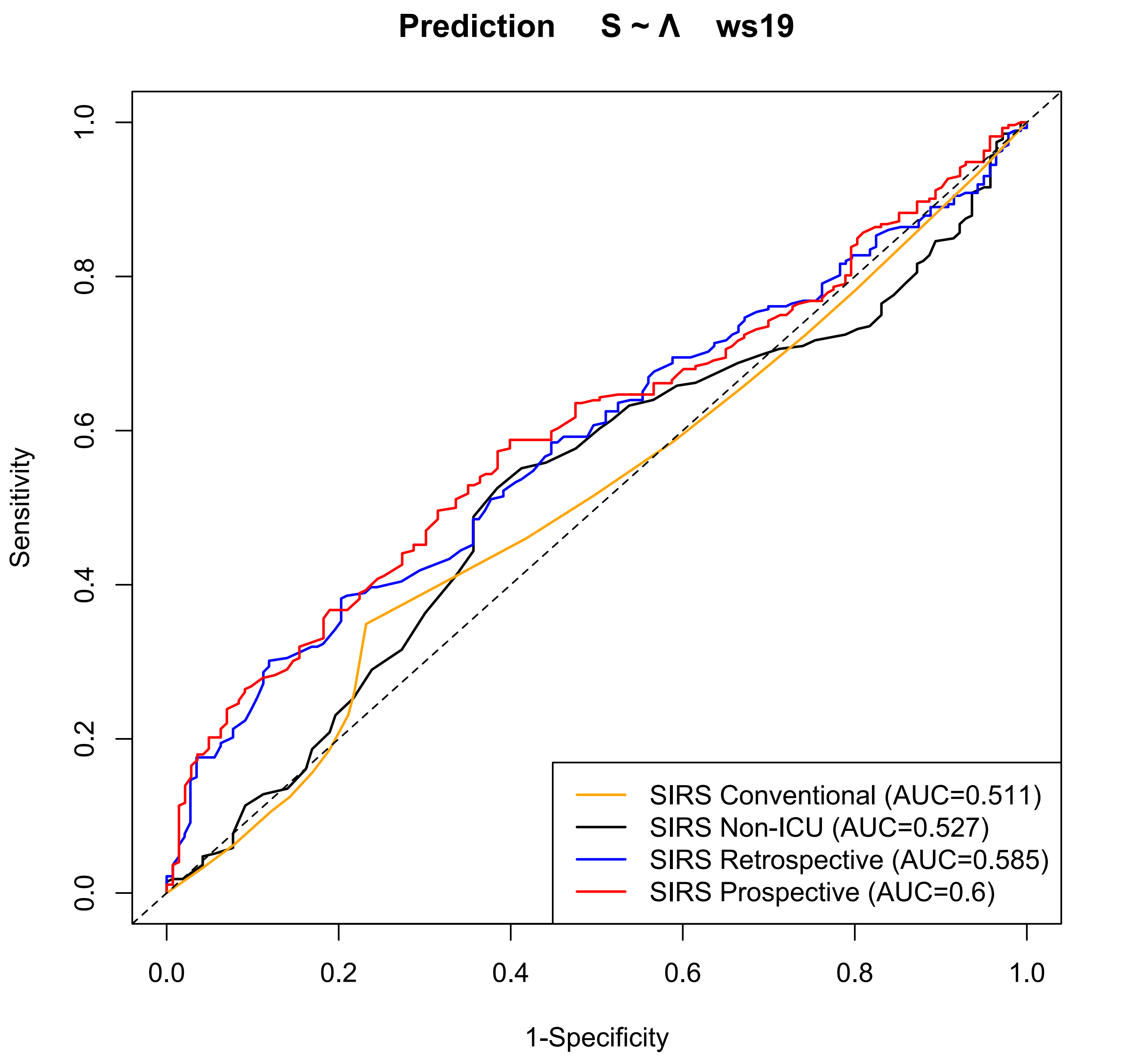

Prediction S ~ Λ ws19
Sensitivity
1-Specificity
0.0
0.2
0.4
0.6
0.8
1.0
SIRS Conventional (AUC=0.511)
SIRS Non-ICU (AUC=0.527)
SIRS Retrospective (AUC=0.585)
SIRS Prospective (AUC=0.6)

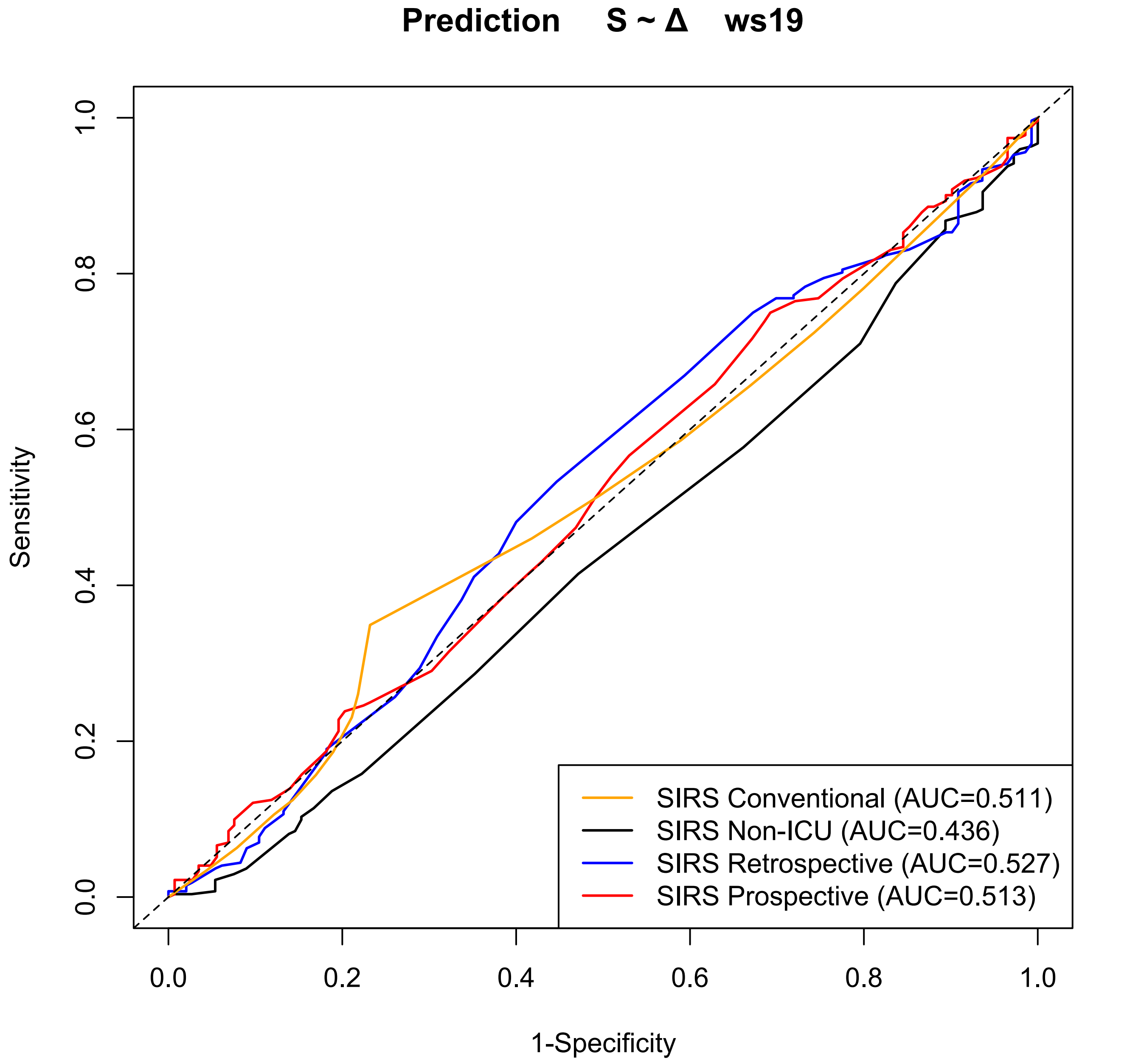

Prediction S ~ Δ ws19
Sensitivity
1-Specificity
0.0
0.2
0.4
0.6
0.8
1.0
SIRS Conventional (AUC=0.511)
SIRS Non-ICU (AUC=0.436)
SIRS Retrospective (AUC=0.527)
SIRS Prospective (AUC=0.513)

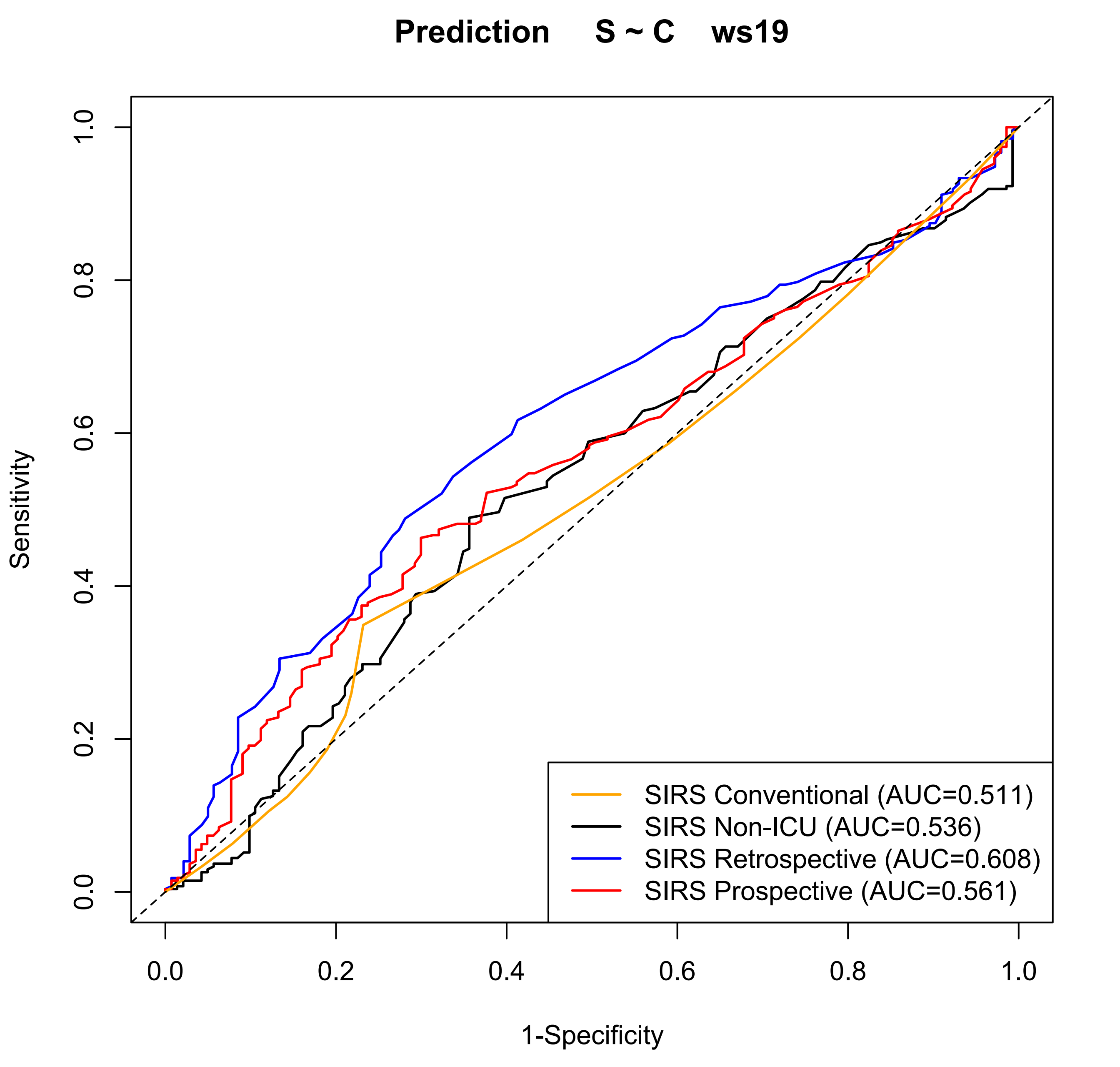

Prediction S ~ C ws19
Sensitivity
1-Specificity
0.0
0.2
0.4
0.6
0.8
1.0
0.0
0.2
0.4
0.6
0.8
1.0
SIRS Conventional (AUC=0.511)
SIRS Non-ICU (AUC=0.536)
SIRS Retrospective (AUC=0.608)
SIRS Prospective (AUC=0.561)

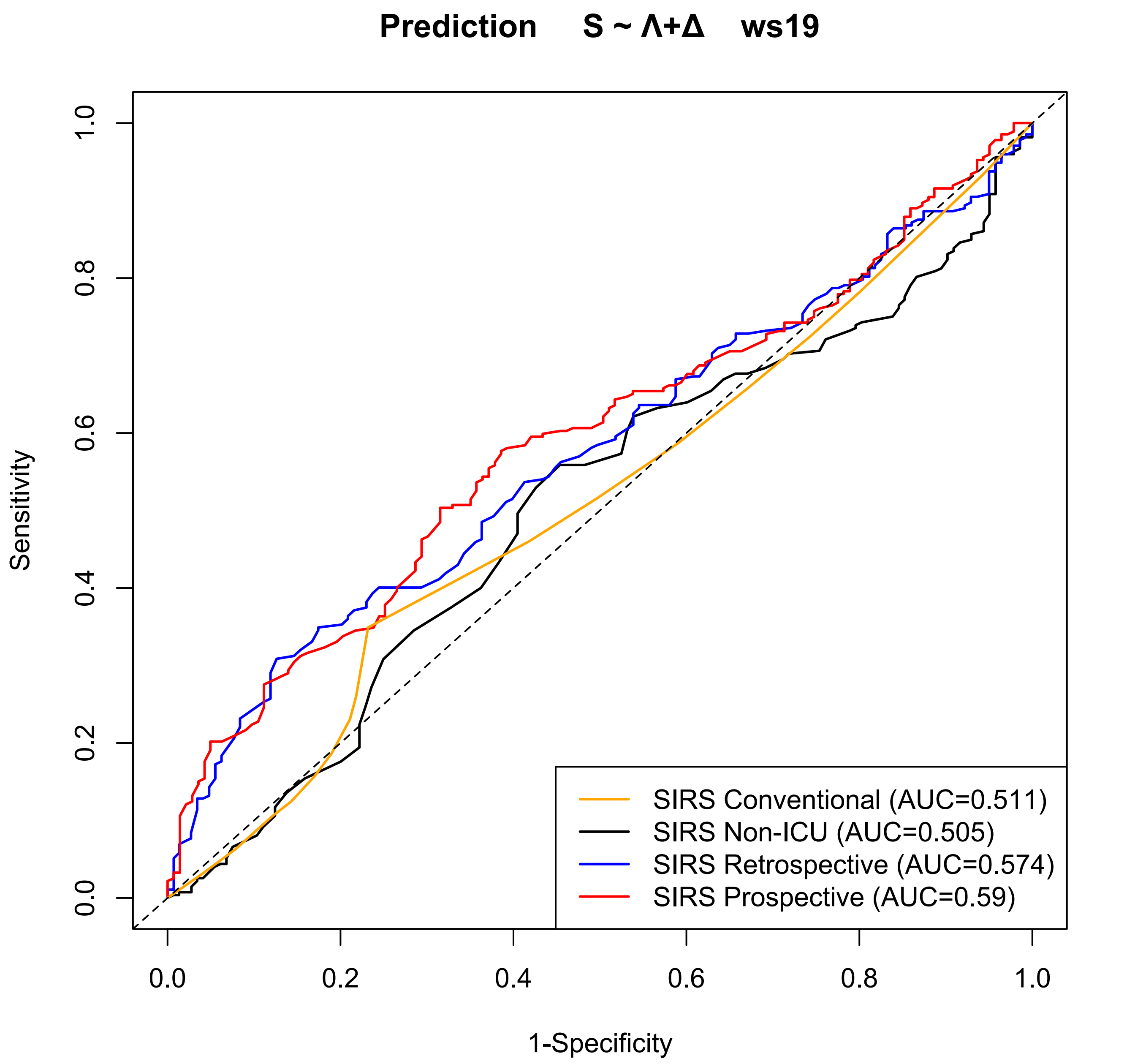

Prediction S ~ Λ+Δ ws19
Sensitivity
1-Specificity
0.0
0.2
0.4
0.6
0.8
1.0
SIRS Conventional (AUC=0.511)
SIRS Non-ICU (AUC=0.505)
SIRS Retrospective (AUC=0.574)
SIRS Prospective (AUC=0.59)

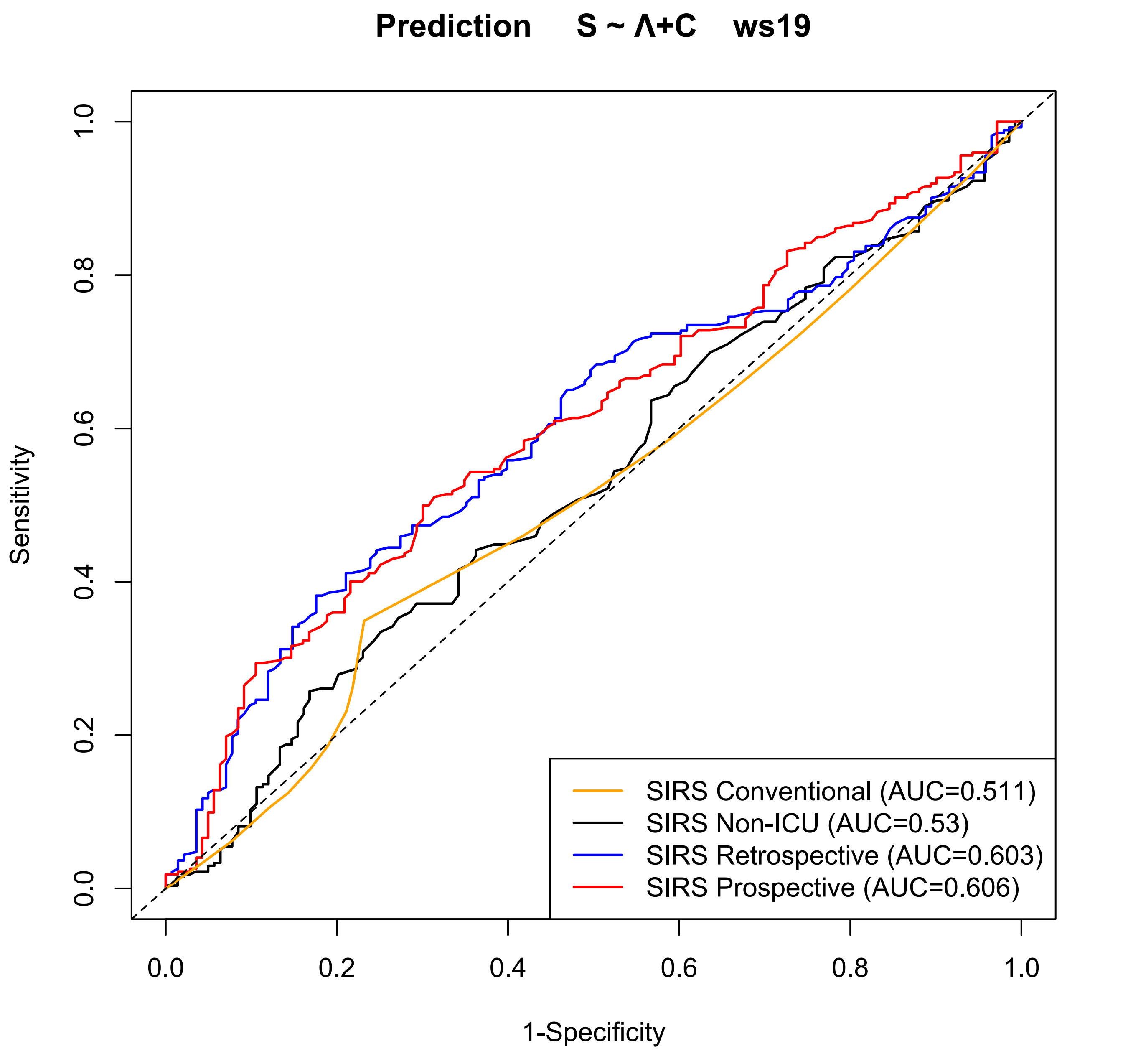

Prediction S ~ Λ+C ws19
Sensitivity
1-Specificity
0.0
0.2
0.4
0.6
0.8
1.0
SIRS Conventional (AUC=0.511)
SIRS Non-ICU (AUC=0.53)
SIRS Retrospective (AUC=0.603)
SIRS Prospective (AUC=0.606)

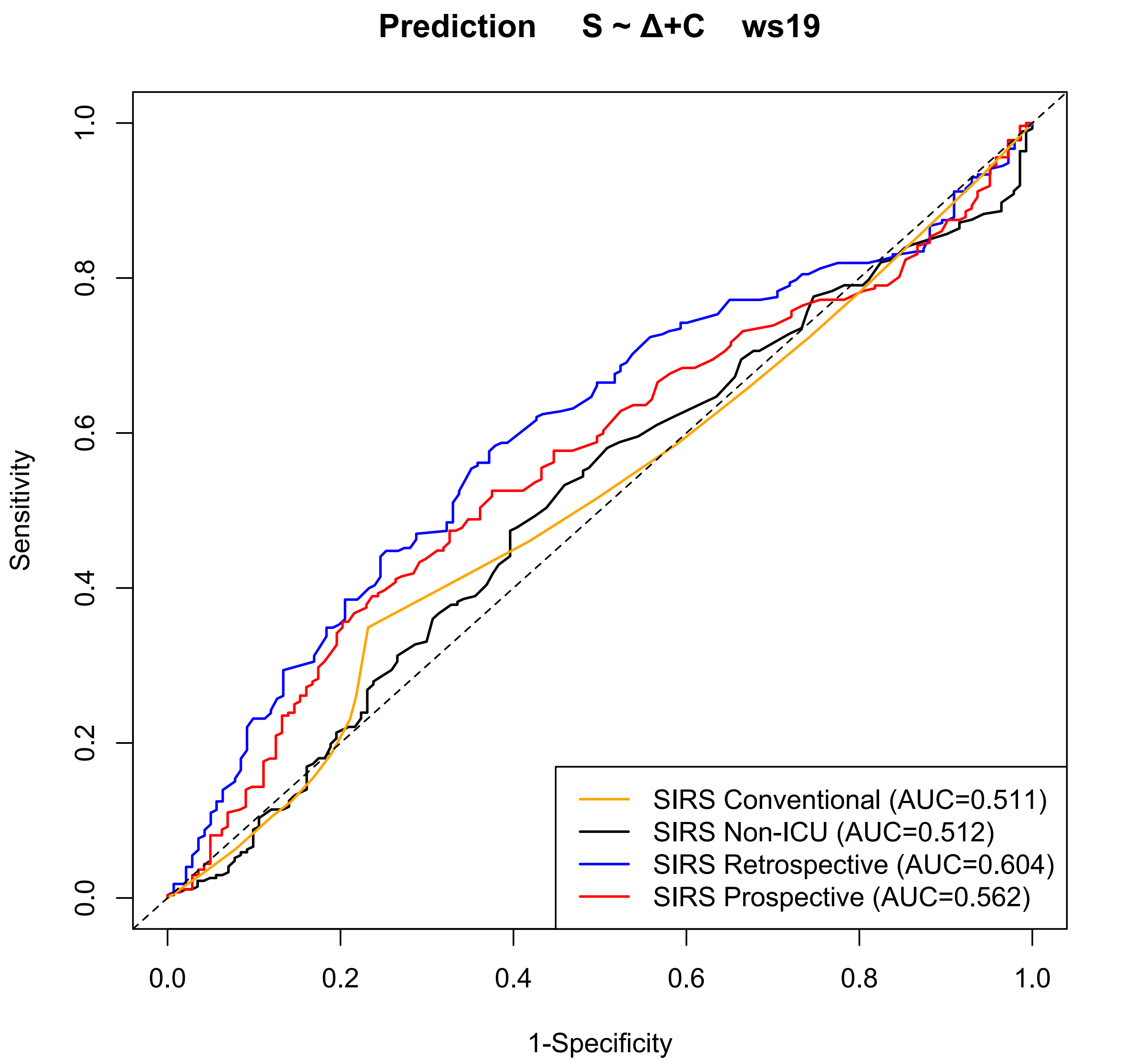

Prediction S ~ Δ+C ws19
Sensitivity
1-Specificity
0.0
0.2
0.4
0.6
0.8
1.0
0.0
0.2
0.4
0.6
0.8
1.0
SIRS Conventional (AUC=0.511)
SIRS Non-ICU (AUC=0.512)
SIRS Retrospective (AUC=0.604)
SIRS Prospective (AUC=0.562)

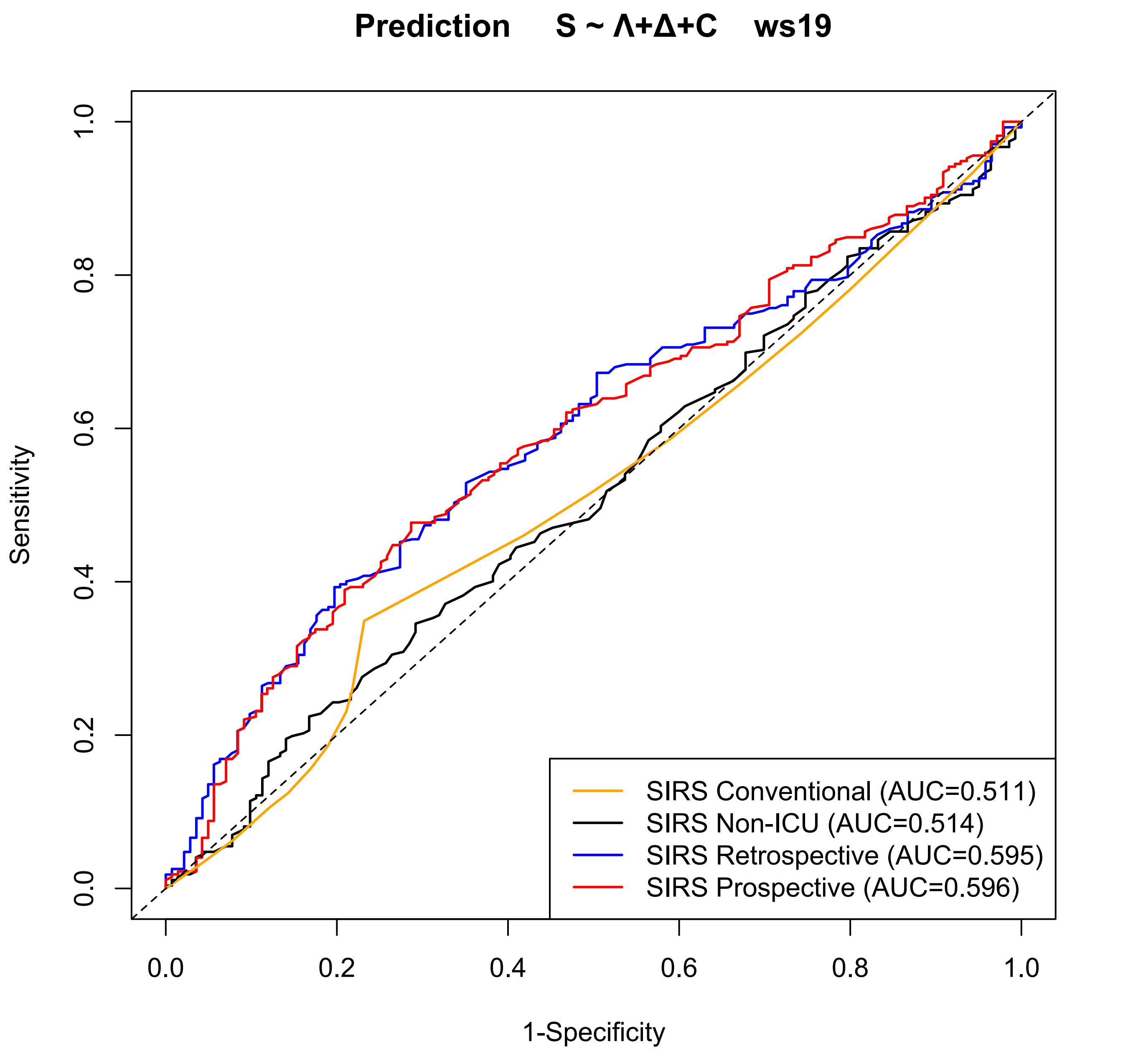

Prediction S ~ Λ+Δ+C ws19
Sensitivity
1-Specificity
0.0
0.2
0.4
0.6
0.8
1.0
SIRS Conventional (AUC=0.511)
SIRS Non-ICU (AUC=0.514)
SIRS Retrospective (AUC=0.595)
SIRS Prospective (AUC=0.596)

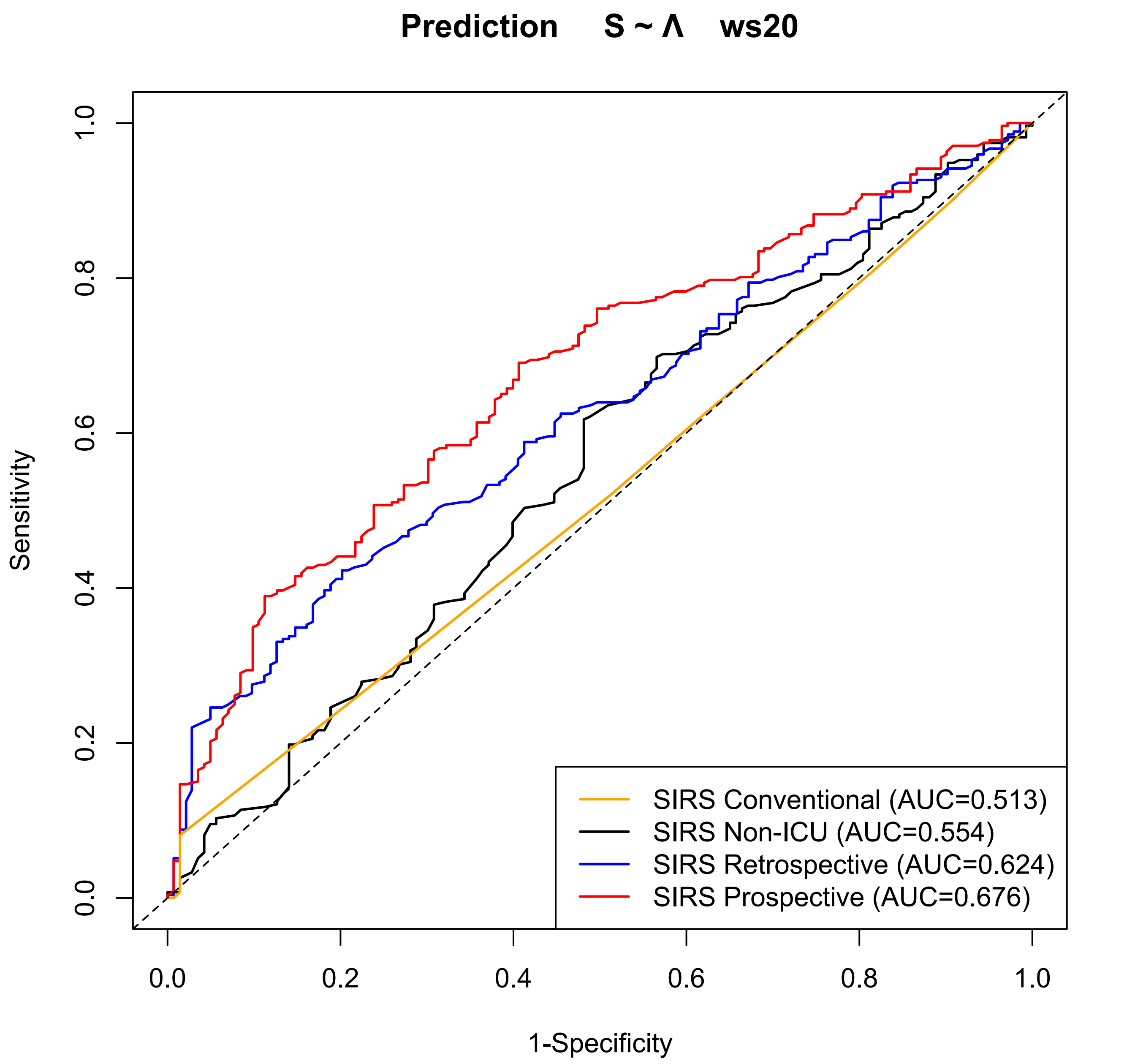

Prediction S ~ Λ ws20
Sensitivity
1-Specificity
0.0
0.2
0.4
0.6
0.8
1.0
SIRS Conventional (AUC=0.513)
SIRS Non-ICU (AUC=0.554)
SIRS Retrospective (AUC=0.624)
SIRS Prospective (AUC=0.676)

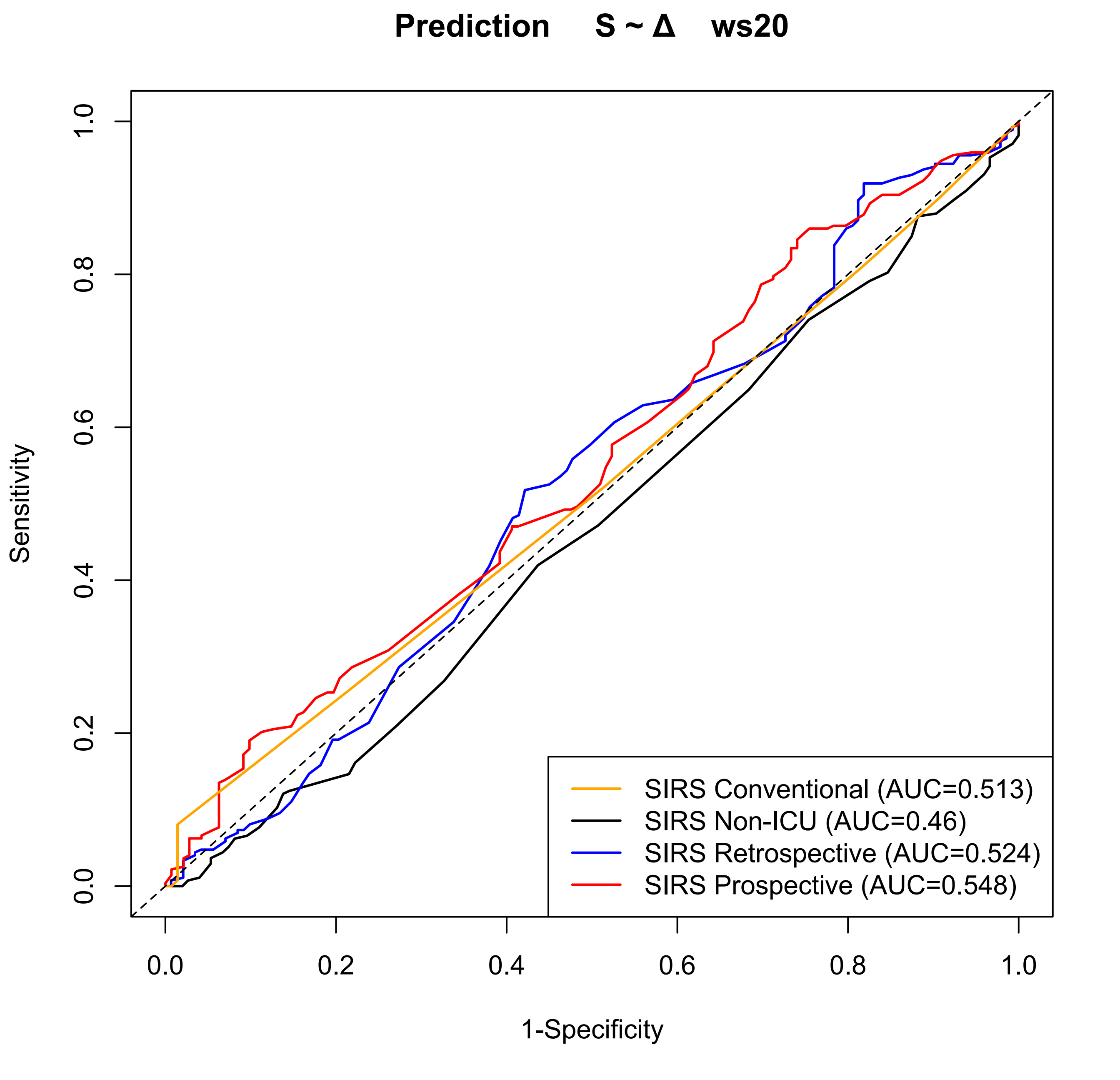

Prediction S ~ Δ ws20
Sensitivity
1-Specificity
0.0
0.2
0.4
0.6
0.8
1.0
SIRS Conventional (AUC=0.513)
SIRS Non-ICU (AUC=0.46)
SIRS Retrospective (AUC=0.524)
SIRS Prospective (AUC=0.548)

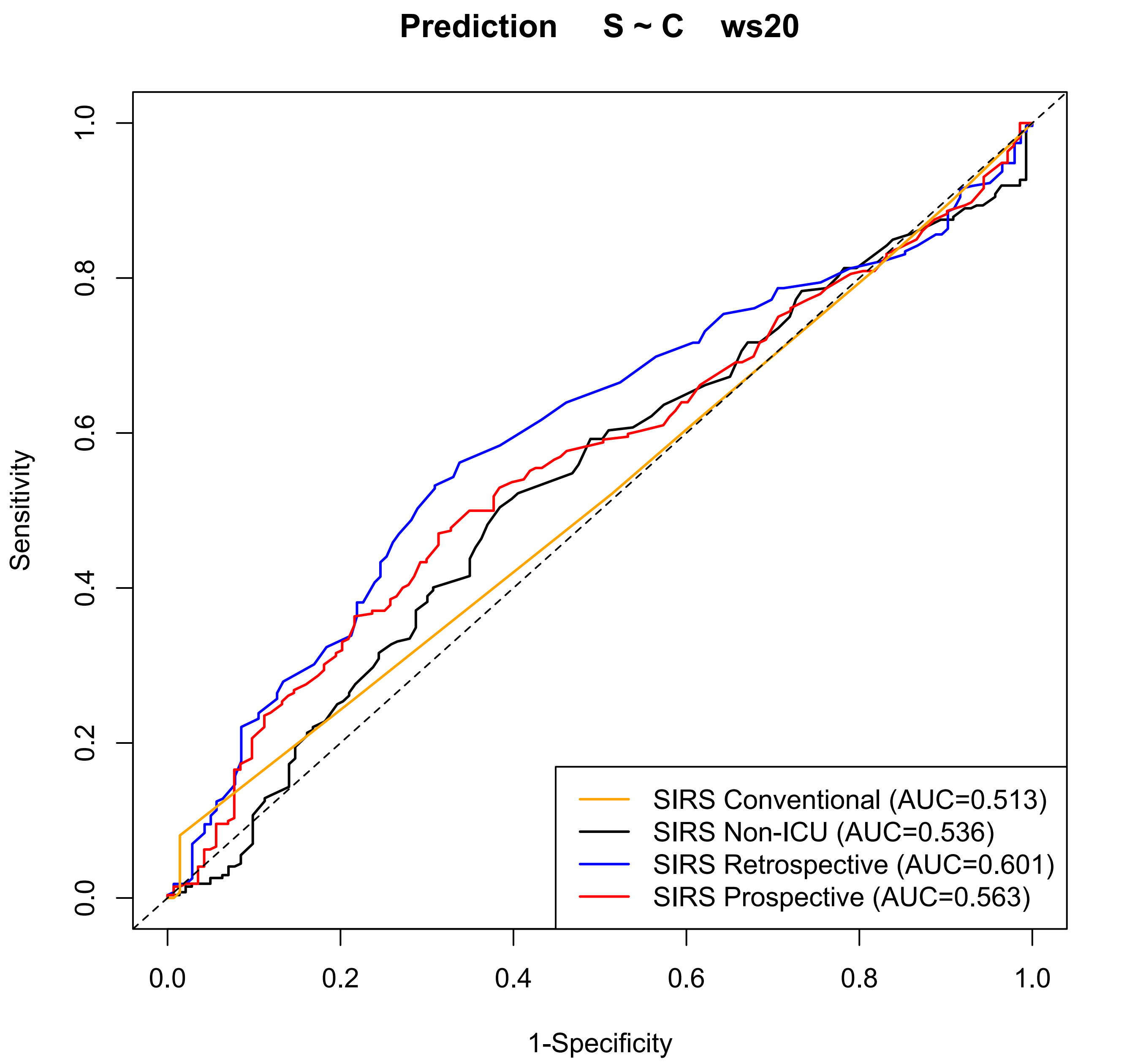

Prediction S ~ C ws20
Sensitivity
1-Specificity
0.0
0.2
0.4
0.6
0.8
1.0
SIRS Conventional (AUC=0.513)
SIRS Non-ICU (AUC=0.536)
SIRS Retrospective (AUC=0.601)
SIRS Prospective (AUC=0.563)

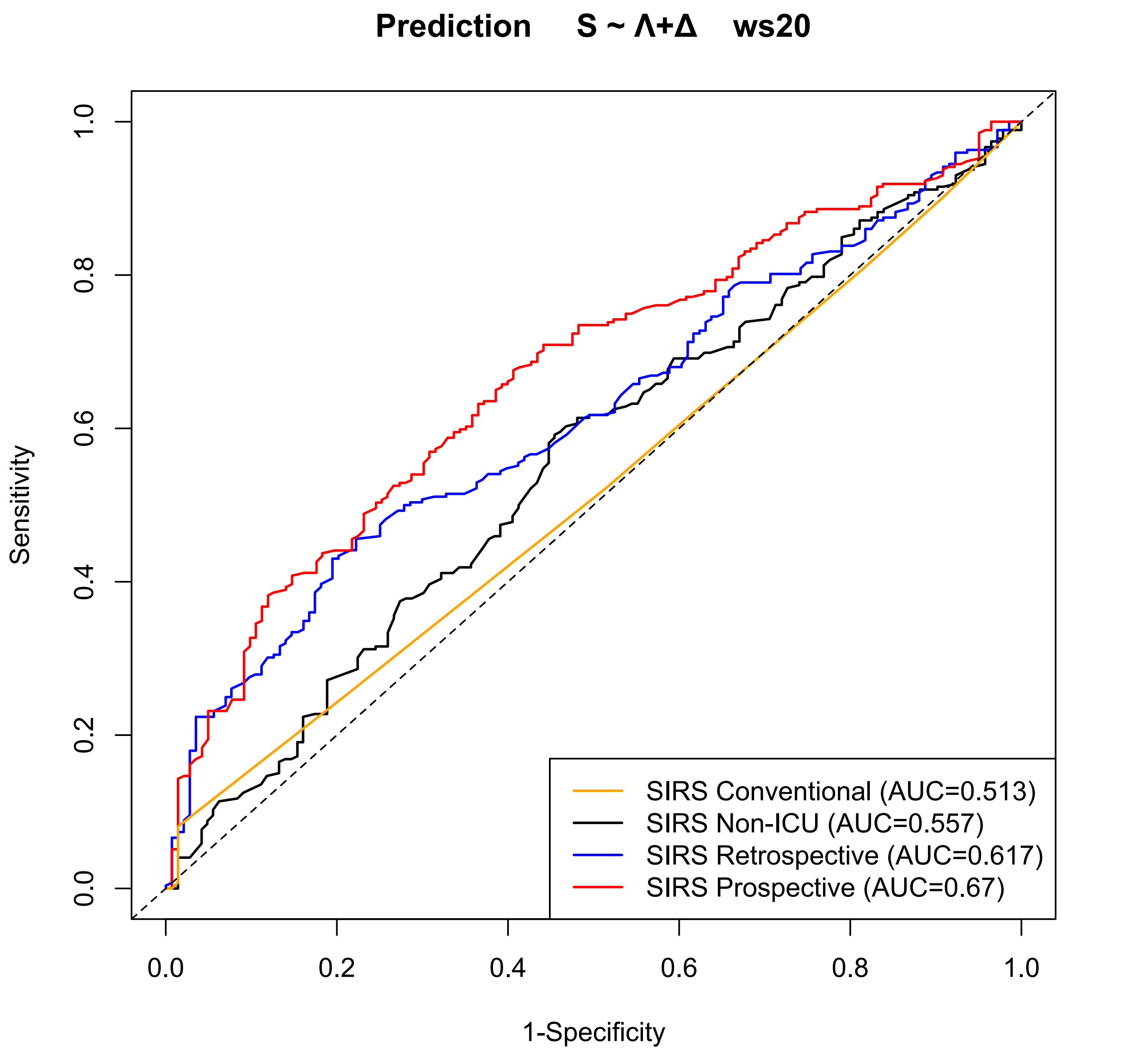
Prediction S ~ Λ+Δ ws20
Sensitivity
1-Specificity
0.0
0.2
0.4
0.6
0.8
1.0
SIRS Conventional (AUC=0.513)
SIRS Non-ICU (AUC=0.557)
SIRS Retrospective (AUC=0.617)
SIRS Prospective (AUC=0.67)

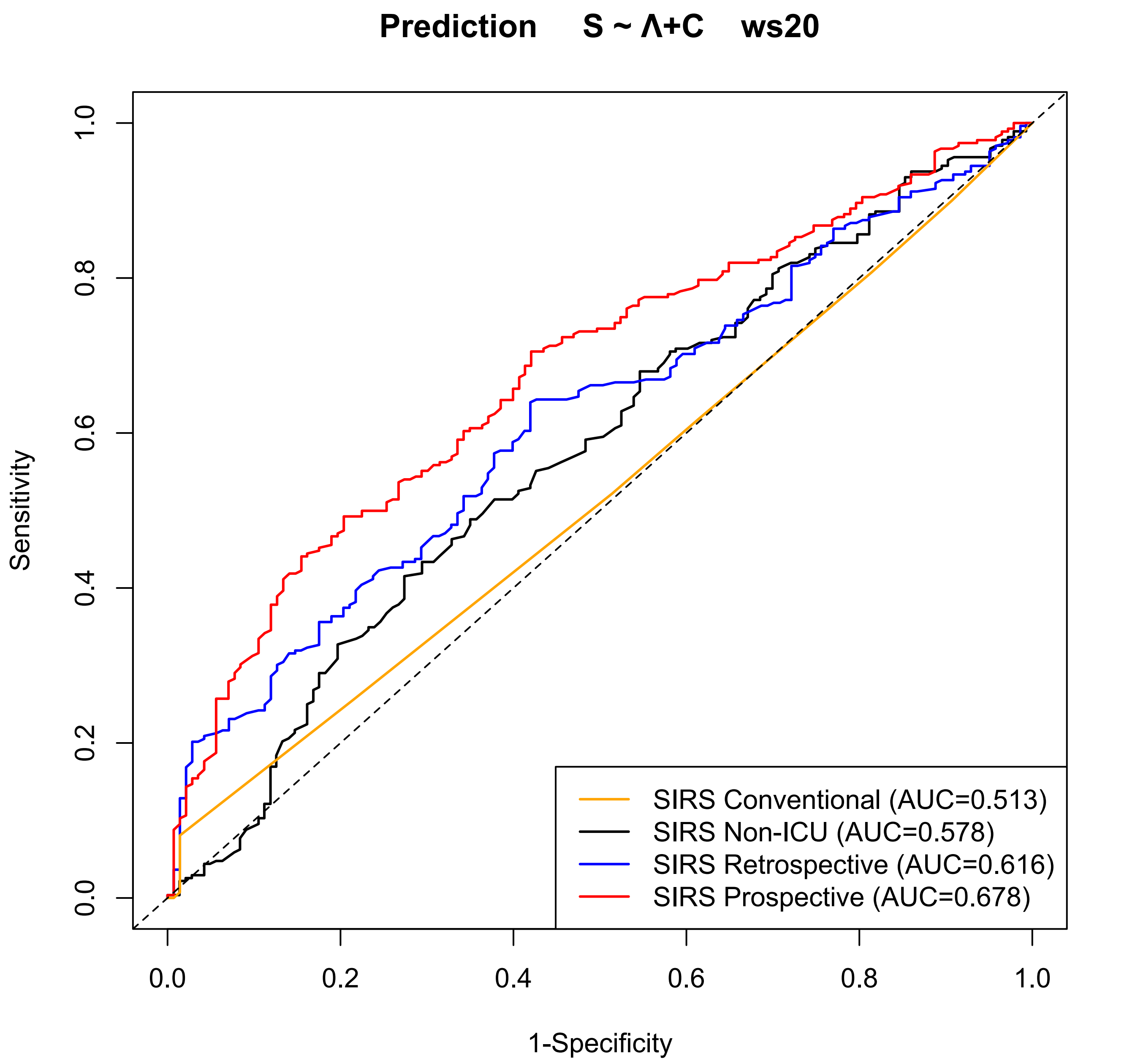

Prediction S ~ Λ+C ws20
Sensitivity
1-Specificity
0.0
0.2
0.4
0.6
0.8
1.0
SIRS Conventional (AUC=0.513)
SIRS Non-ICU (AUC=0.578)
SIRS Retrospective (AUC=0.616)
SIRS Prospective (AUC=0.678)

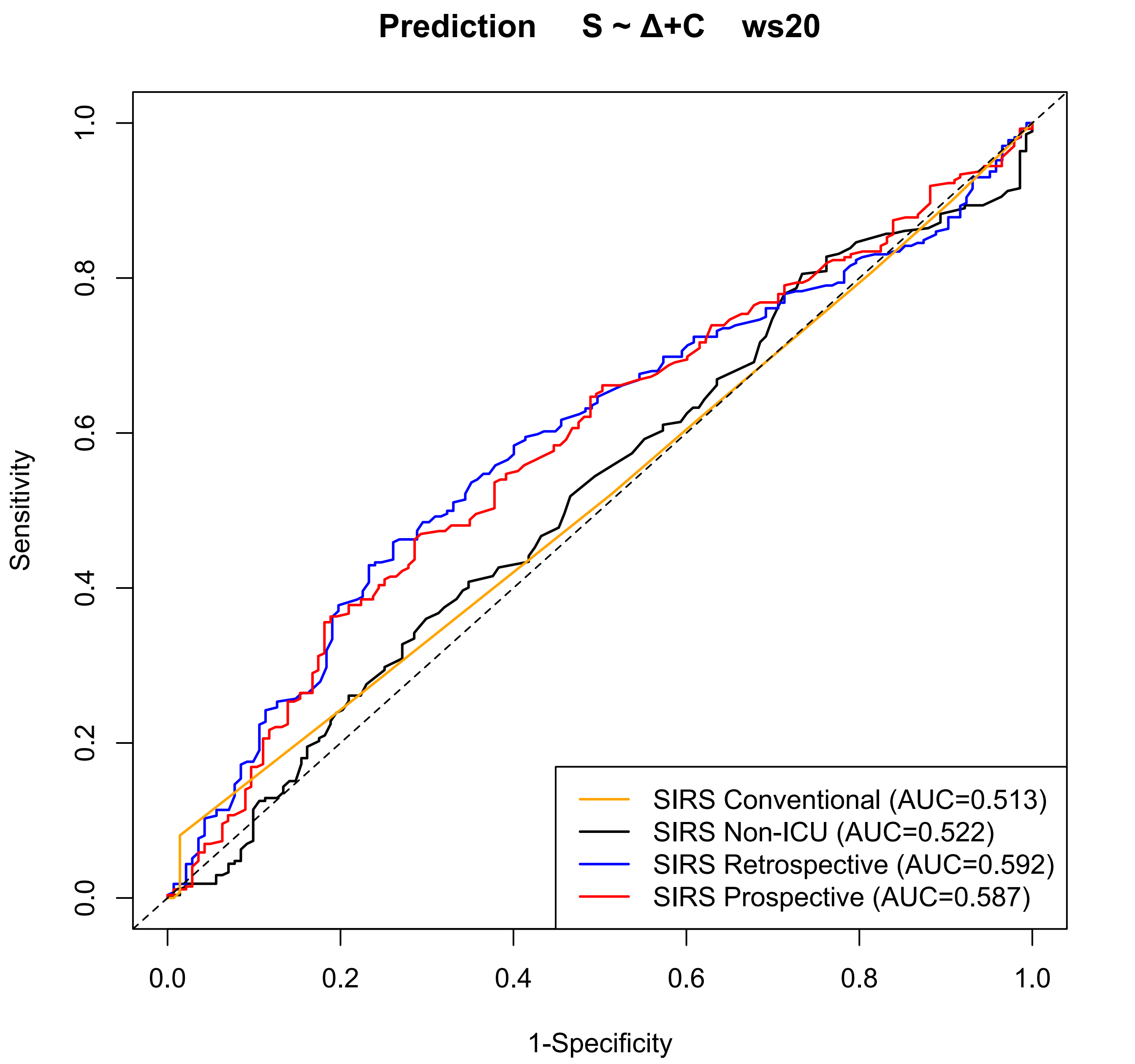

Prediction S ~ Δ+C ws20
Sensitivity
1-Specificity
0.0
0.2
0.4
0.6
0.8
1.0
SIRS Conventional (AUC=0.513)
SIRS Non-ICU (AUC=0.522)
SIRS Retrospective (AUC=0.592)
SIRS Prospective (AUC=0.587)

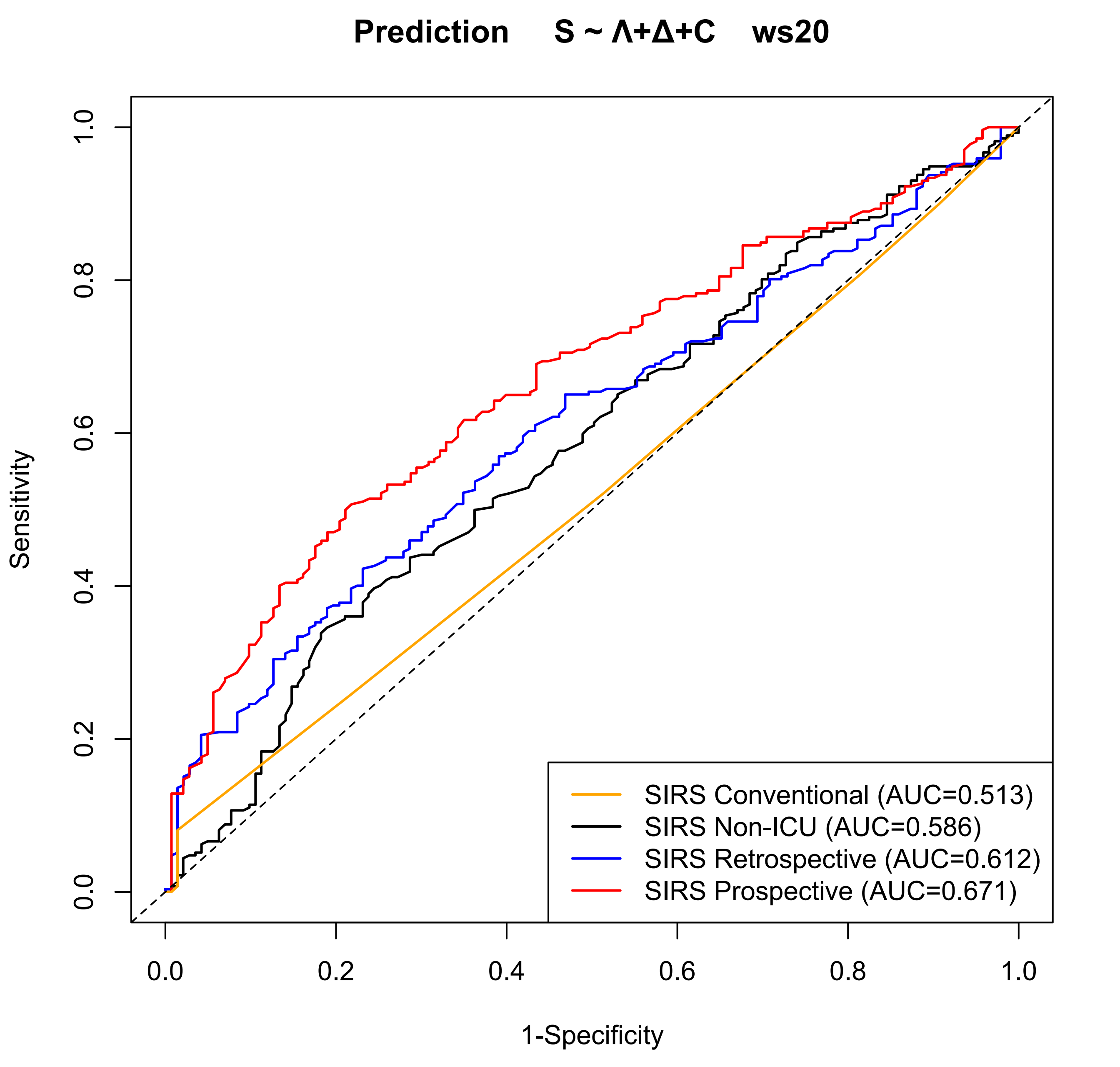

Prediction S ~ Λ+Δ+C ws20
Sensitivity
1-Specificity
0.0
0.2
0.4
0.6
0.8
1.0
0.0
0.2
0.4
0.6
0.8
1.0
SIRS Conventional (AUC=0.513)
SIRS Non-ICU (AUC=0.586)
SIRS Retrospective (AUC=0.612)
SIRS Prospective (AUC=0.671)

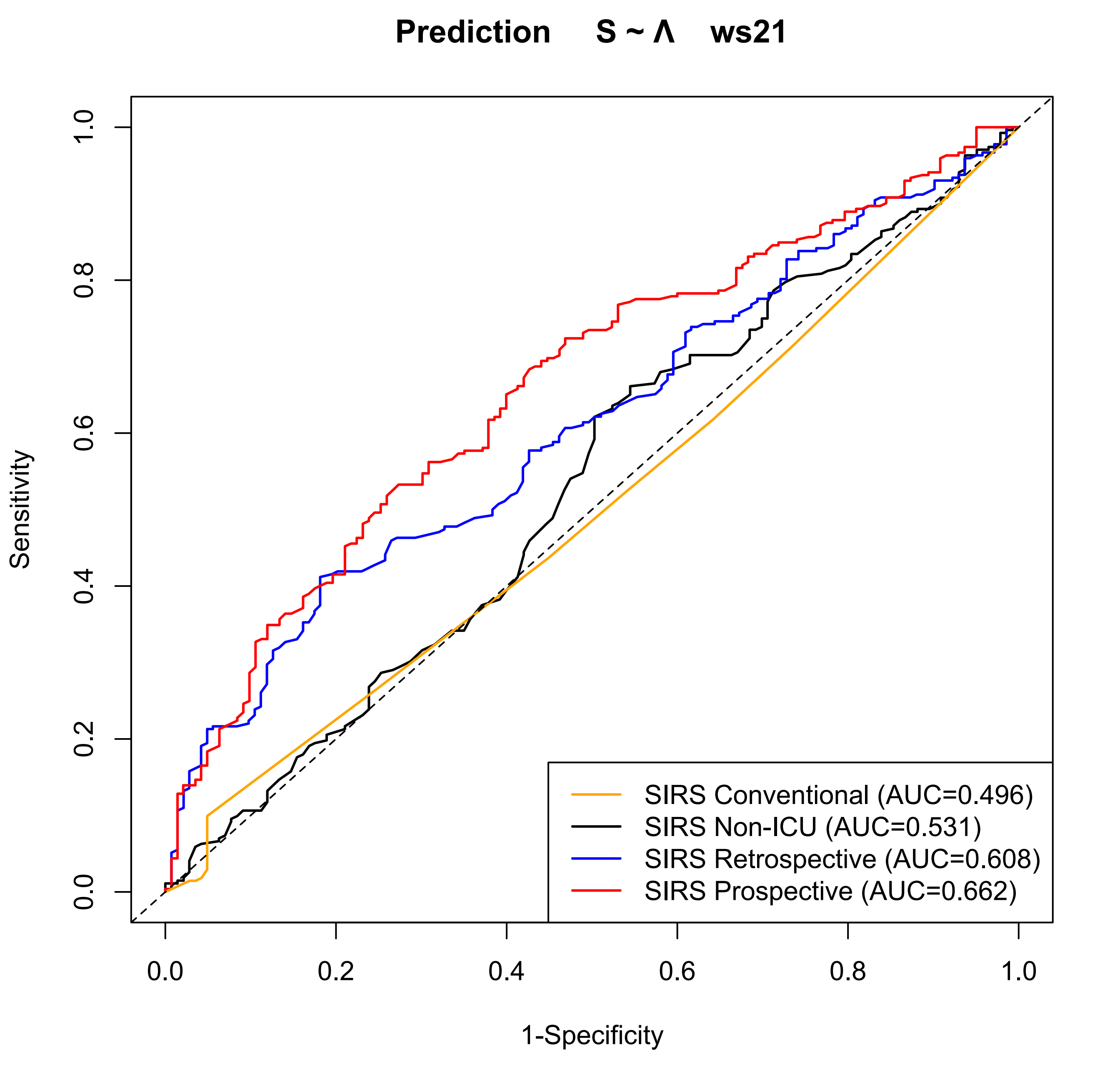

Prediction S ~ Λ ws21
Sensitivity
1-Specificity
0.0
0.2
0.4
0.6
0.8
1.0
0.0
0.2
0.4
0.6
0.8
1.0
SIRS Conventional (AUC=0.496)
SIRS Non-ICU (AUC=0.531)
SIRS Retrospective (AUC=0.608)
SIRS Prospective (AUC=0.662)

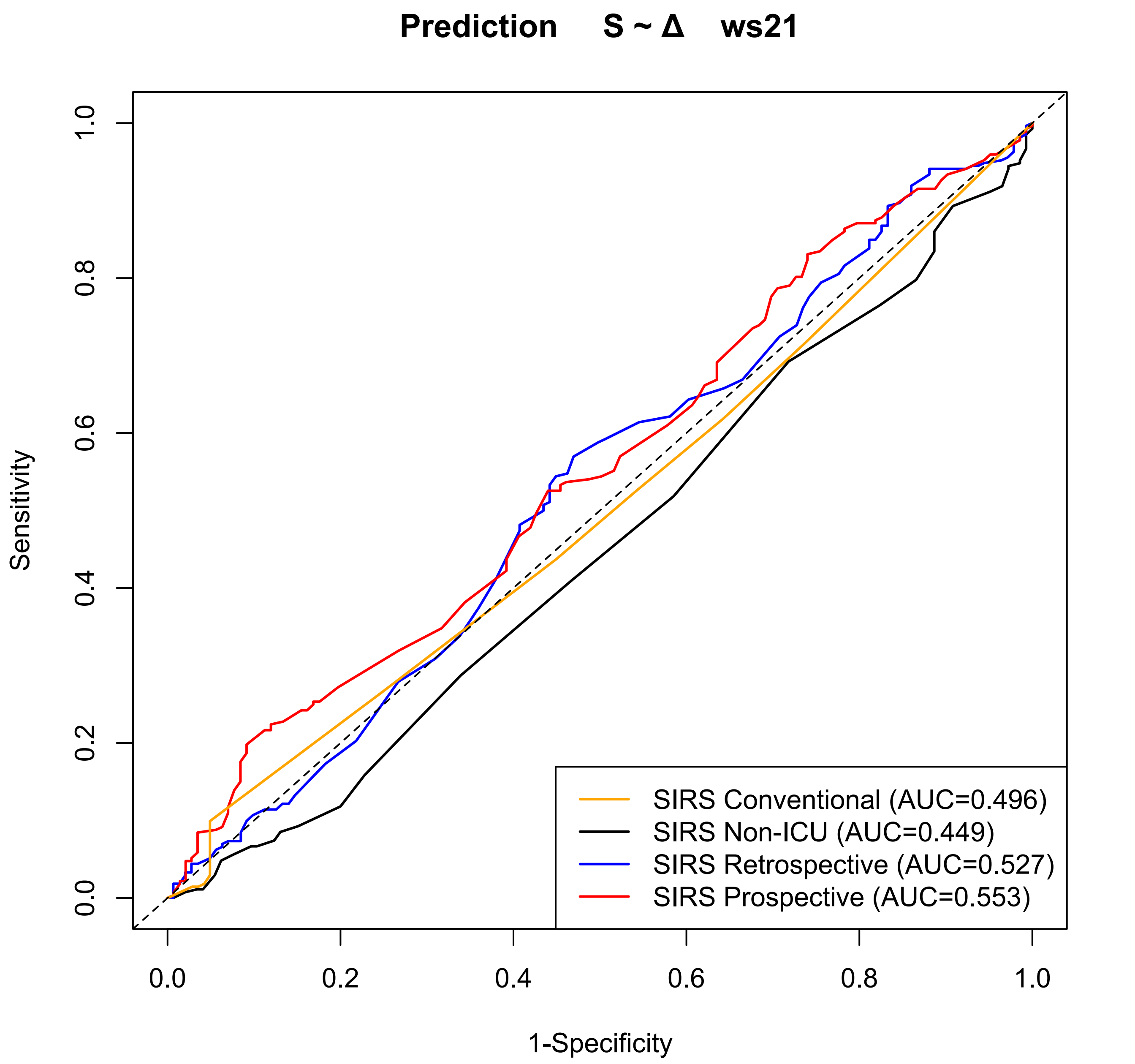

Prediction S ~ Δ ws21
Sensitivity
1-Specificity
0.0
0.2
0.4
0.6
0.8
1.0
0.0
0.2
0.4
0.6
0.8
1.0
SIRS Conventional (AUC=0.496)
SIRS Non-ICU (AUC=0.449)
SIRS Retrospective (AUC=0.527)
SIRS Prospective (AUC=0.553)

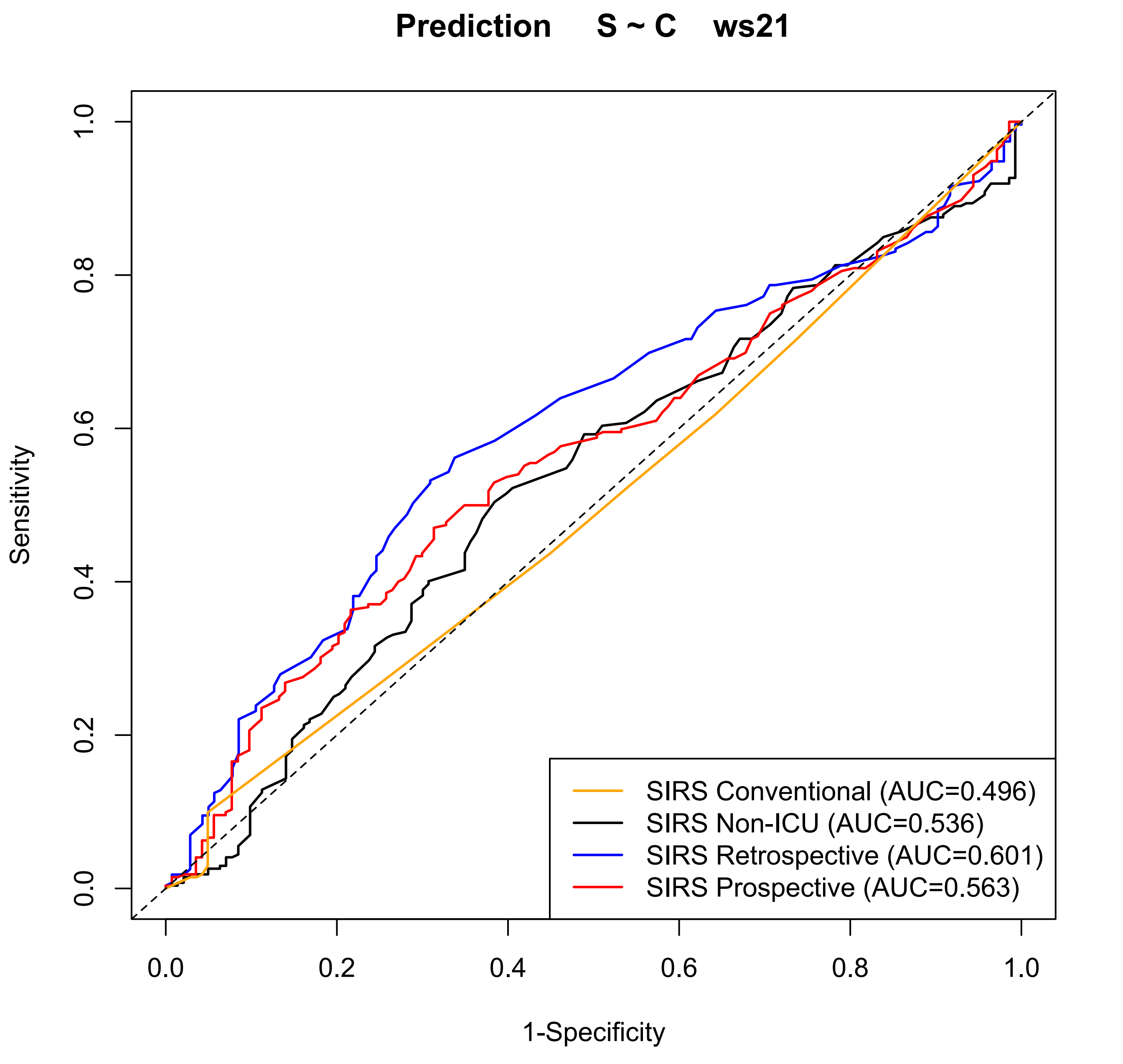
Prediction S ~ C ws21
Sensitivity
1-Specificity
0.0
0.2
0.4
0.6
0.8
1.0
SIRS Conventional (AUC=0.496)
SIRS Non-ICU (AUC=0.536)
SIRS Retrospective (AUC=0.601)
SIRS Prospective (AUC=0.563)

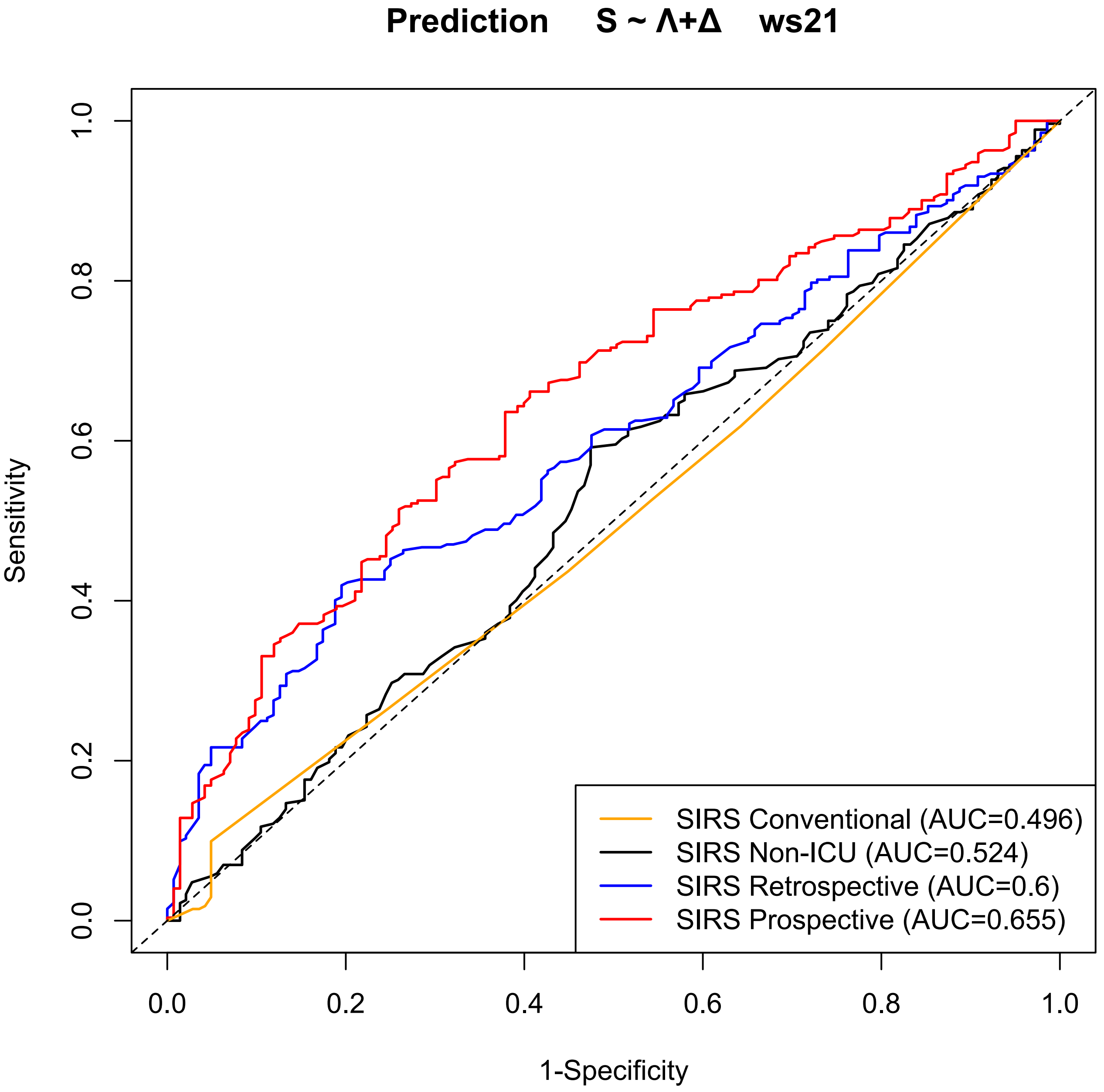

Prediction S ~ Λ+Δ ws21
Sensitivity
1-Specificity
0.0
0.2
0.4
0.6
0.8
1.0
SIRS Conventional (AUC=0.496)
SIRS Non-ICU (AUC=0.524)
SIRS Retrospective (AUC=0.6)
SIRS Prospective (AUC=0.655)

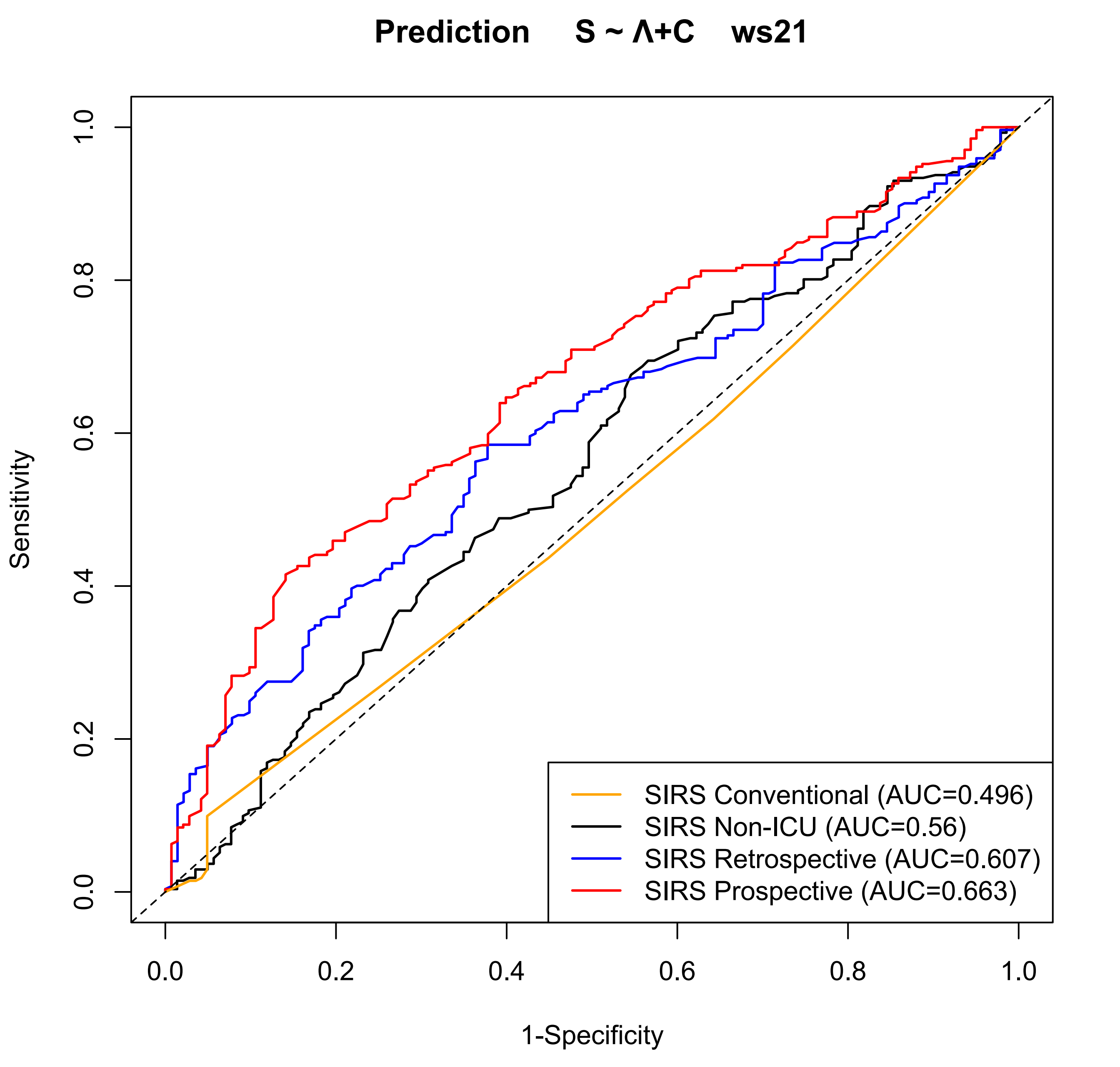

Prediction S ~ Λ+C ws21
Sensitivity
1-Specificity
0.0
0.2
0.4
0.6
0.8
1.0
0.0
0.2
0.4
0.6
0.8
1.0
SIRS Conventional (AUC=0.496)
SIRS Non-ICU (AUC=0.56)
SIRS Retrospective (AUC=0.607)
SIRS Prospective (AUC=0.663)

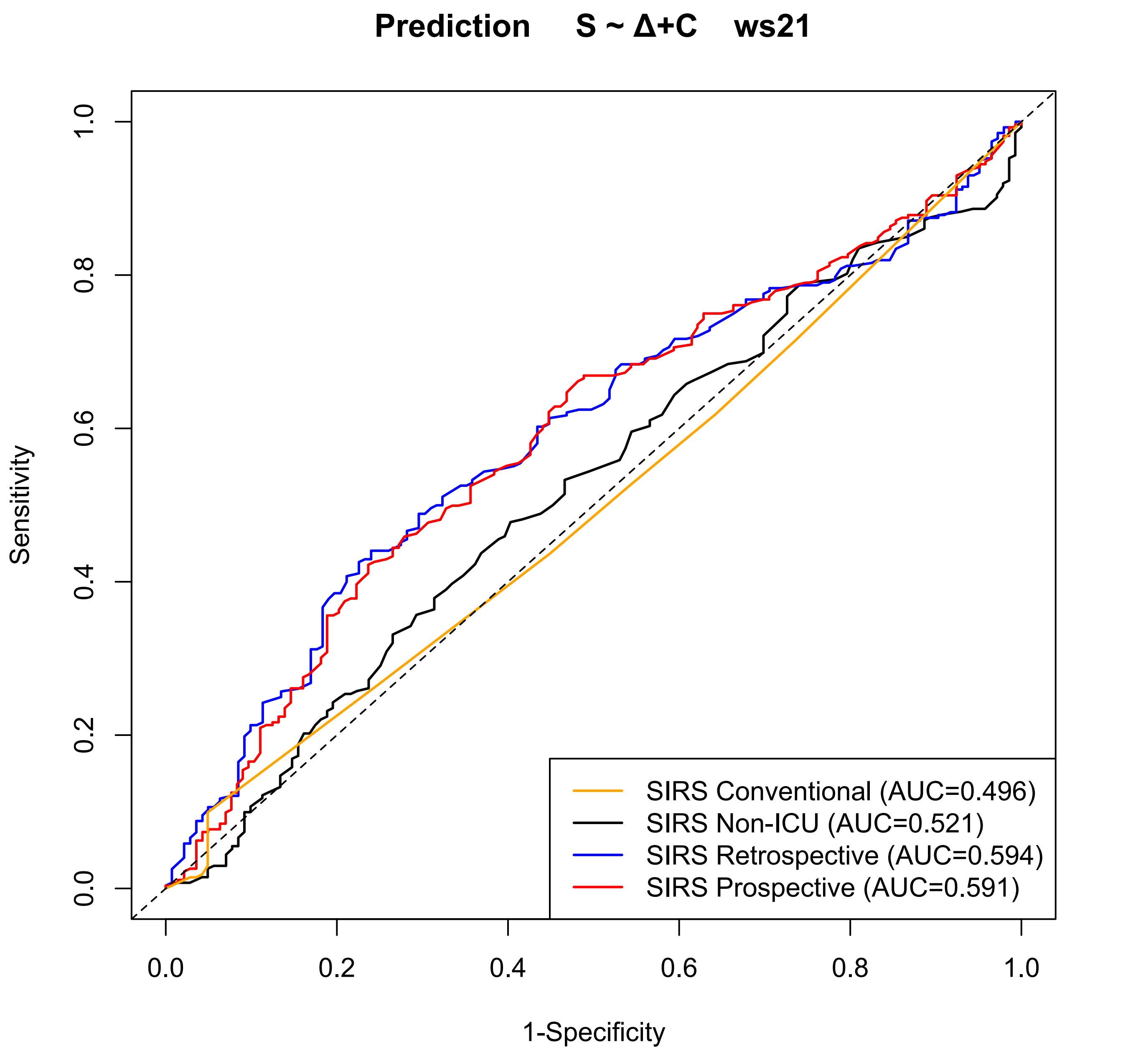

Prediction S ~ Δ+C ws21
Sensitivity
1-Specificity
0.0
0.2
0.4
0.6
0.8
1.0
0.0
0.2
0.4
0.6
0.8
1.0
SIRS Conventional (AUC=0.496)
SIRS Non-ICU (AUC=0.521)
SIRS Retrospective (AUC=0.594)
SIRS Prospective (AUC=0.591)

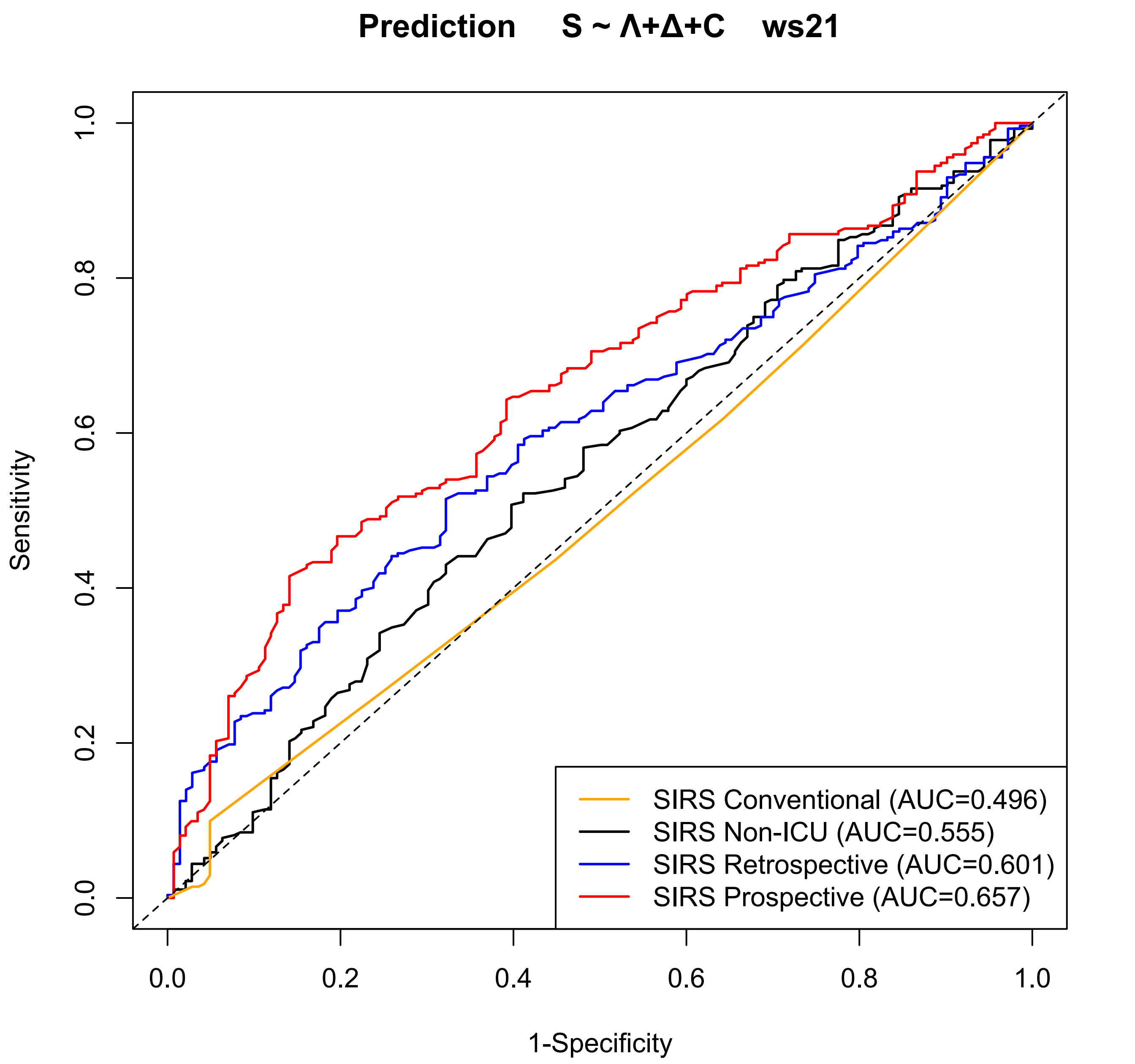

Prediction S ~ Λ+Δ+C ws21
Sensitivity
1-Specificity
0.0
0.2
0.4
0.6
0.8
1.0
0.0
0.2
0.4
0.6
0.8
1.0
SIRS Conventional (AUC=0.496)
SIRS Non-ICU (AUC=0.555)
SIRS Retrospective (AUC=0.601)
SIRS Prospective (AUC=0.657)

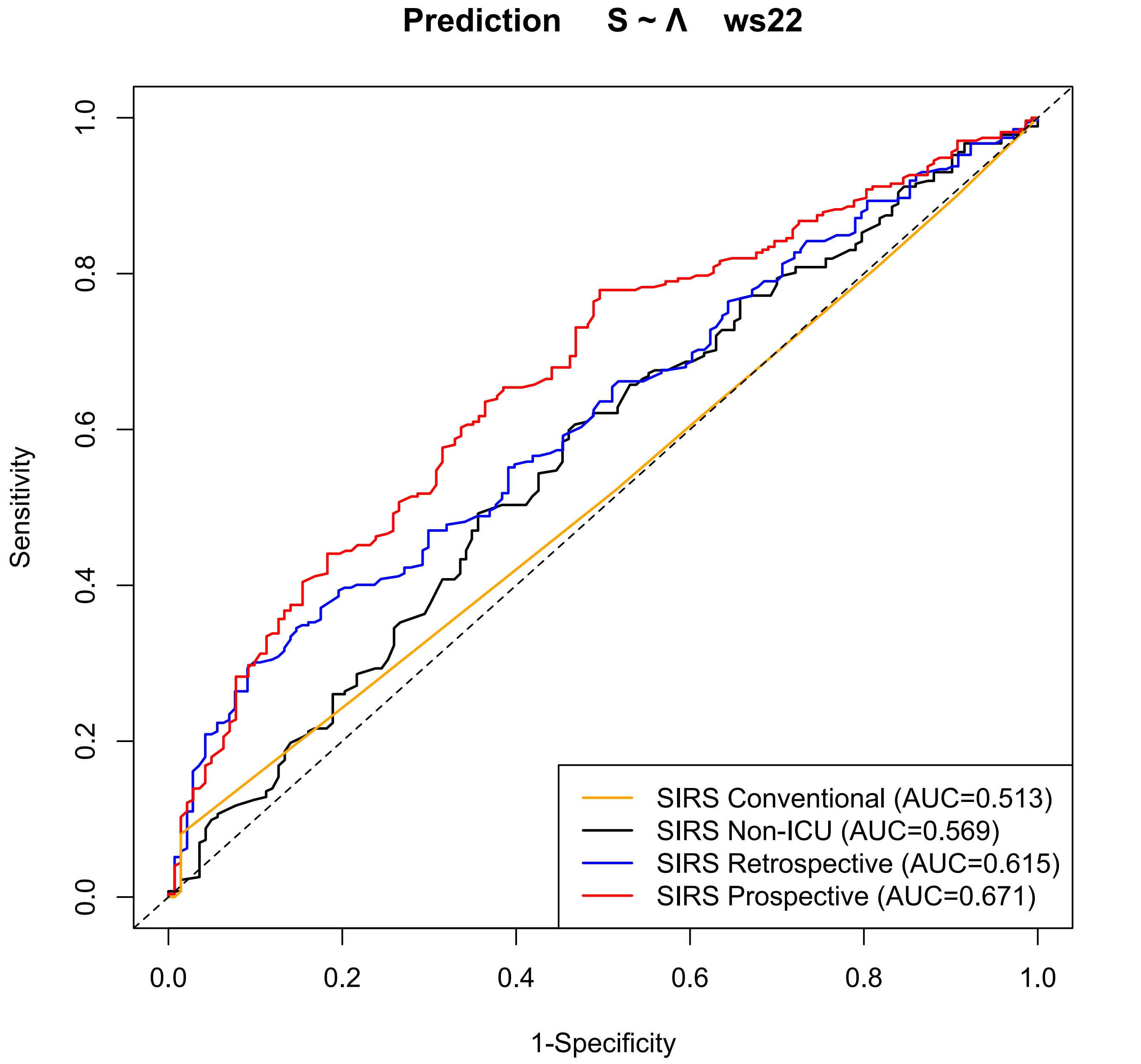

Prediction S ~ Λ ws22
Sensitivity
1-Specificity
0.0
0.2
0.4
0.6
0.8
1.0
SIRS Conventional (AUC=0.513)
SIRS Non-ICU (AUC=0.569)
SIRS Retrospective (AUC=0.615)
SIRS Prospective (AUC=0.671)

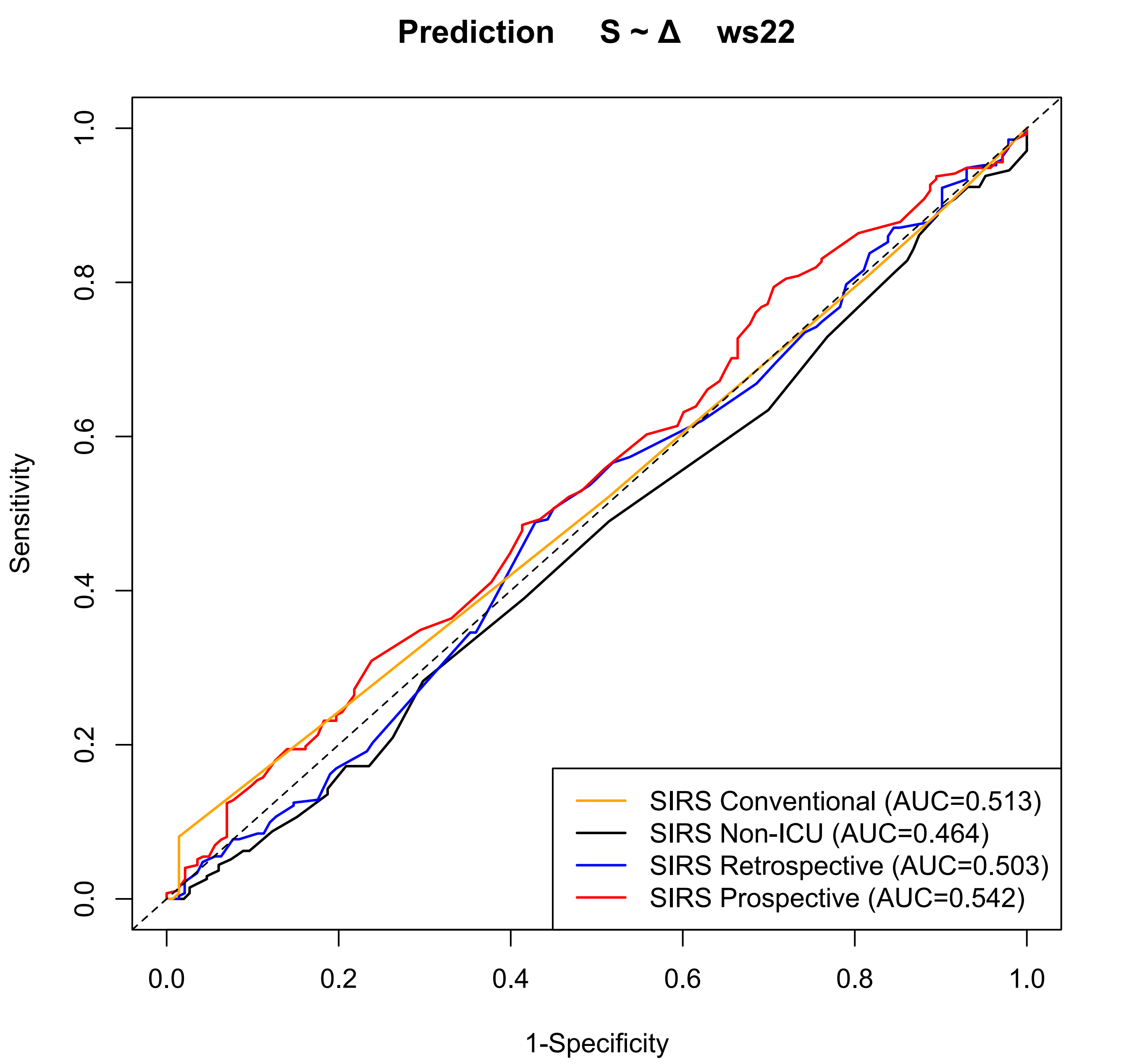

Prediction S ~ Δ ws22
Sensitivity
1-Specificity
0.0
0.2
0.4
0.6
0.8
1.0
SIRS Conventional (AUC=0.513)
SIRS Non-ICU (AUC=0.464)
SIRS Retrospective (AUC=0.503)
SIRS Prospective (AUC=0.542)

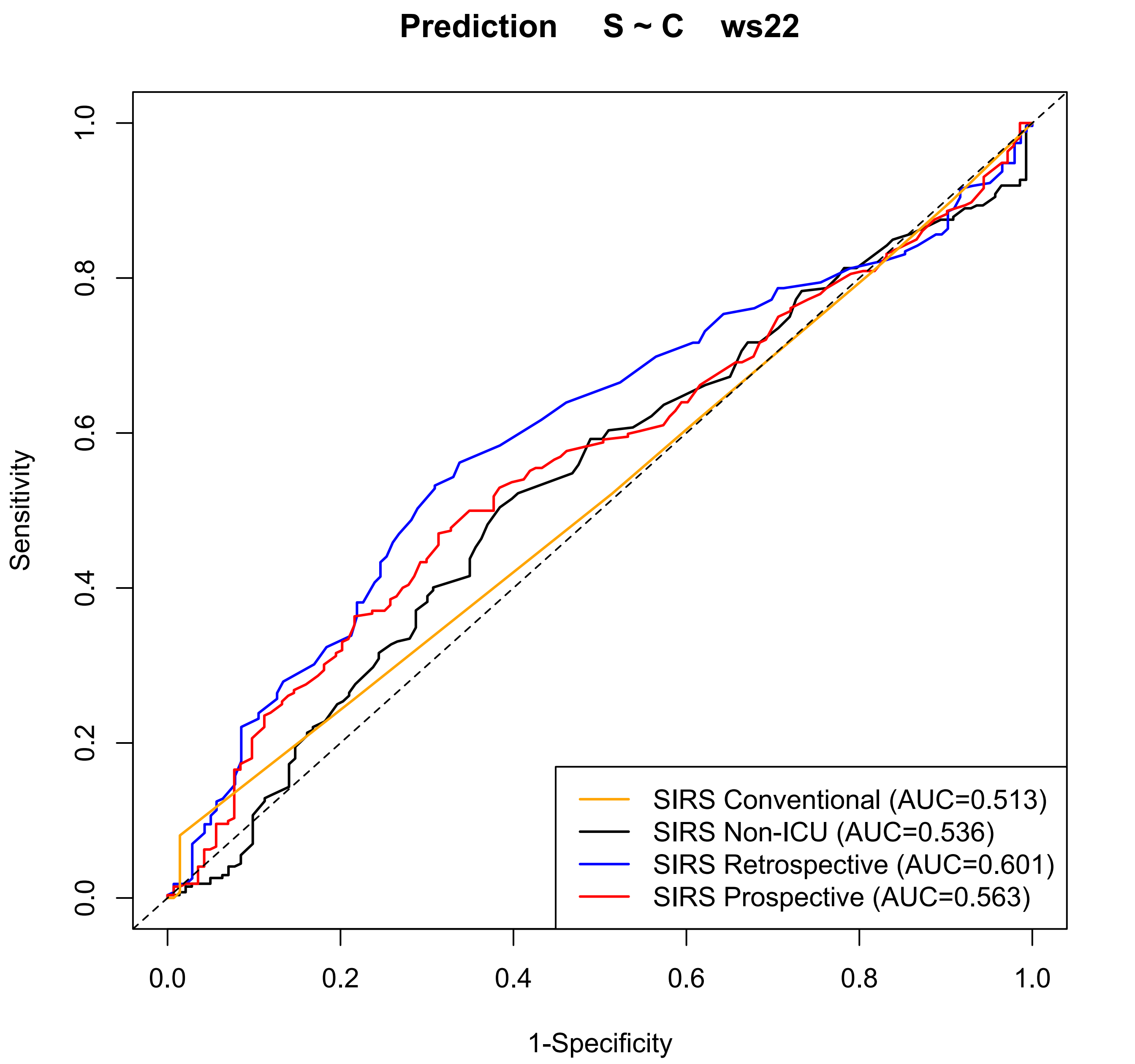

Prediction S ~ C ws22
Sensitivity
1-Specificity
0.0
0.2
0.4
0.6
0.8
1.0
SIRS Conventional (AUC=0.513)
SIRS Non-ICU (AUC=0.536)
SIRS Retrospective (AUC=0.601)
SIRS Prospective (AUC=0.563)

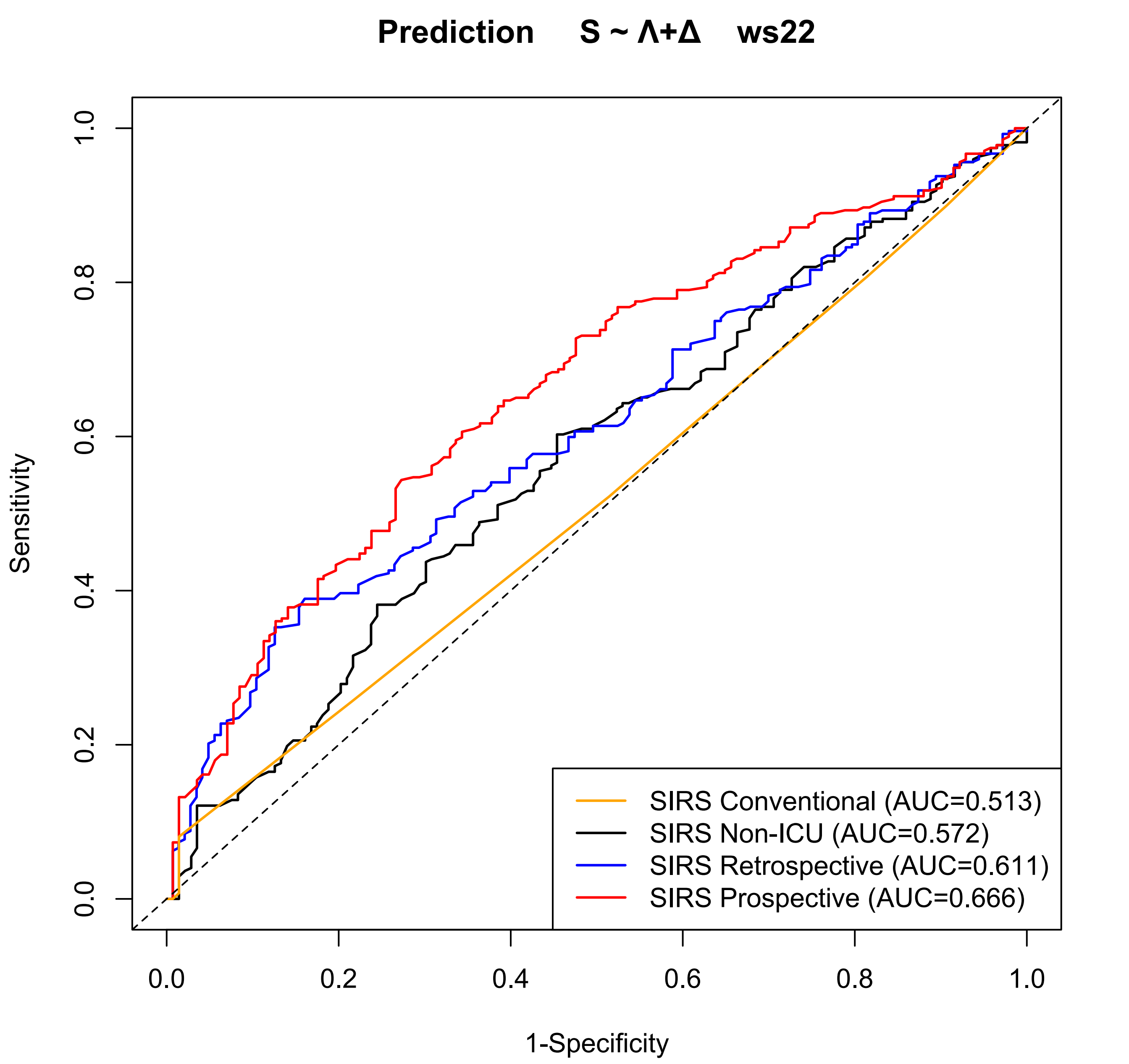
Prediction S ~ Λ+Δ ws22
Sensitivity
1-Specificity
0.0
0.2
0.4
0.6
0.8
1.0
SIRS Conventional (AUC=0.513)
SIRS Non-ICU (AUC=0.572)
SIRS Retrospective (AUC=0.611)
SIRS Prospective (AUC=0.666)

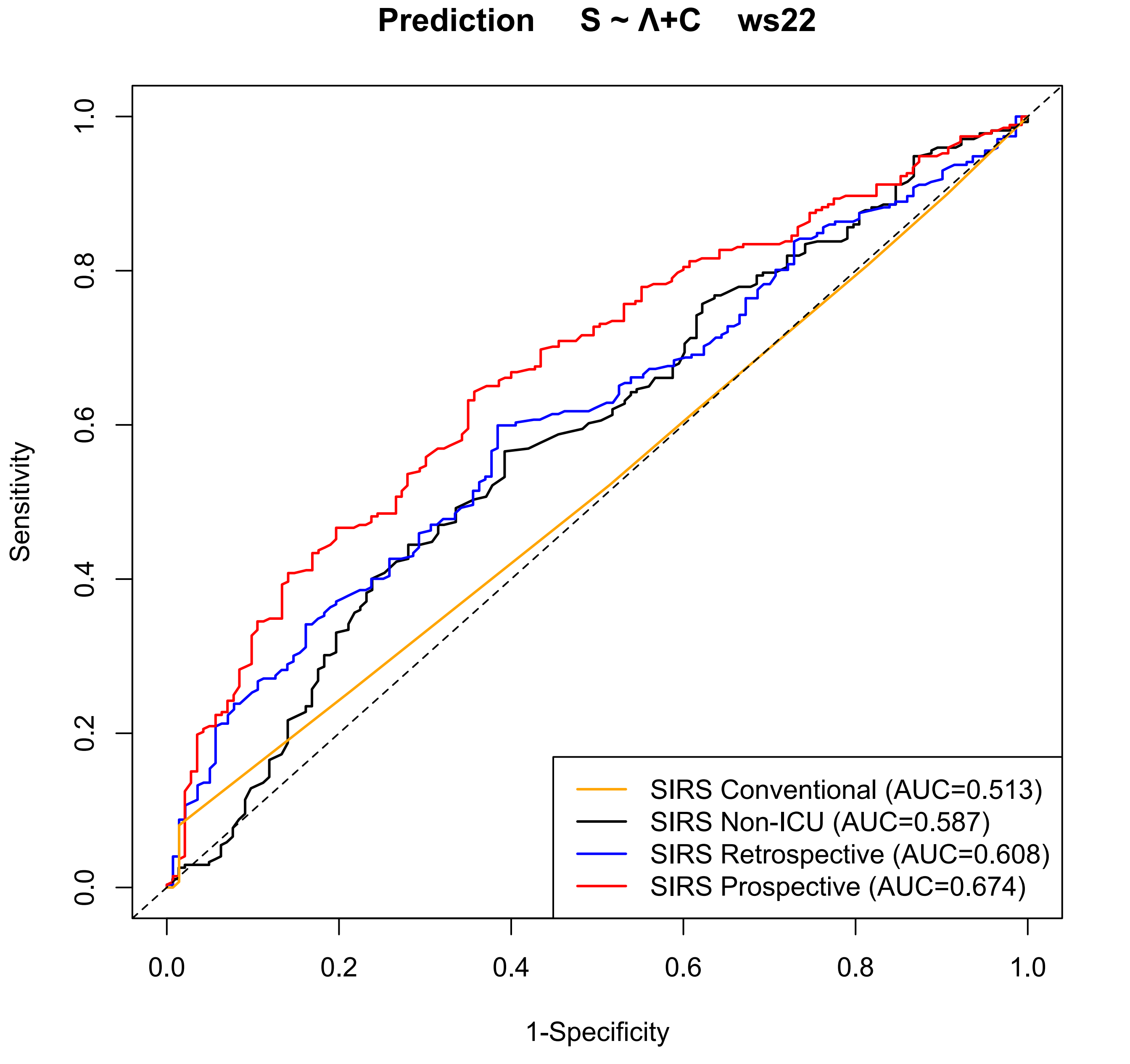

Prediction S ~ Λ+C ws22
Sensitivity
1-Specificity
0.0
0.2
0.4
0.6
0.8
1.0
0.0
0.2
0.4
0.6
0.8
1.0
SIRS Conventional (AUC=0.513)
SIRS Non-ICU (AUC=0.587)
SIRS Retrospective (AUC=0.608)
SIRS Prospective (AUC=0.674)

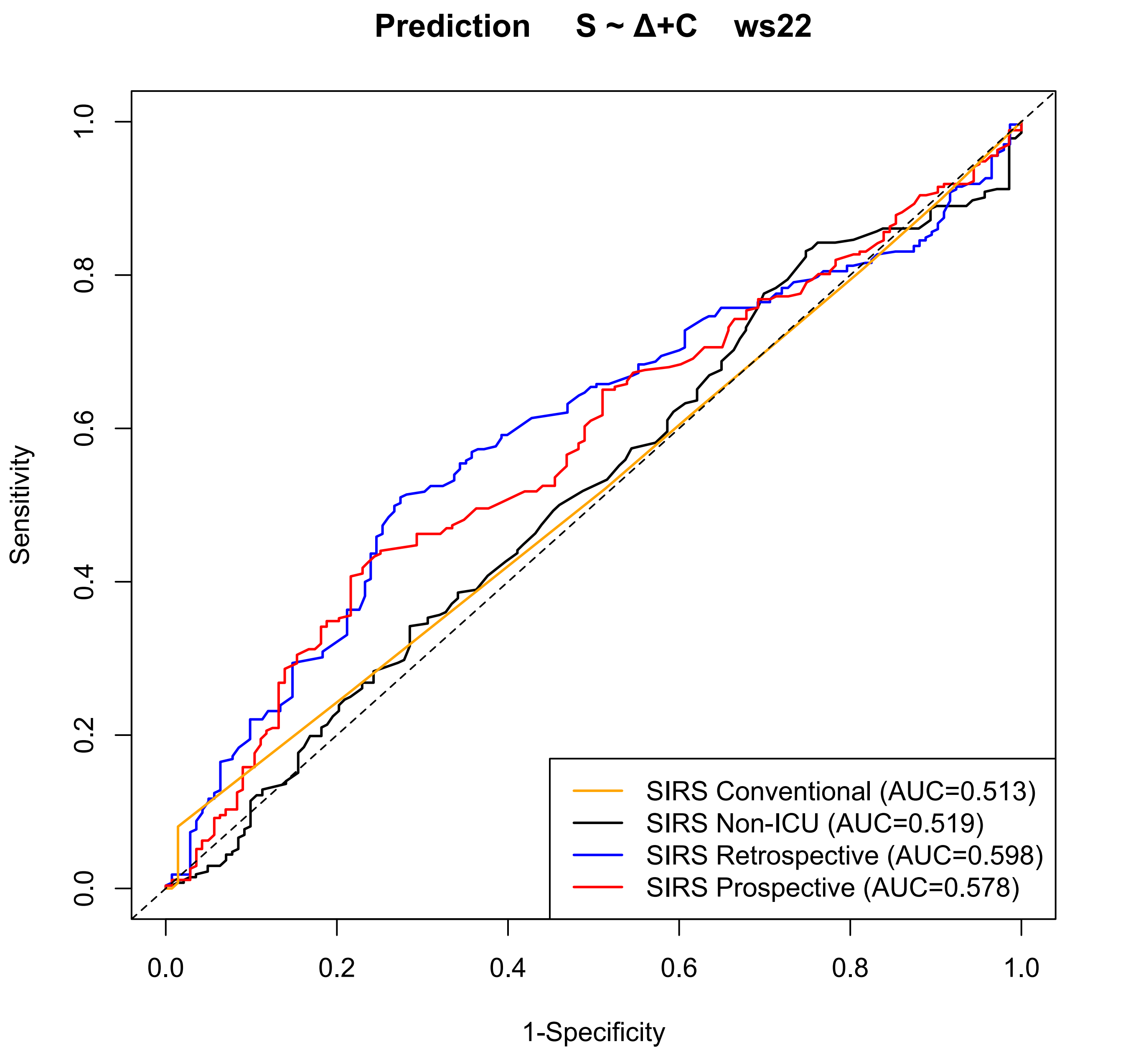

Prediction S ~ Δ+C ws22
Sensitivity
1-Specificity
0.0
0.2
0.4
0.6
0.8
1.0
SIRS Conventional (AUC=0.513)
SIRS Non-ICU (AUC=0.519)
SIRS Retrospective (AUC=0.598)
SIRS Prospective (AUC=0.578)

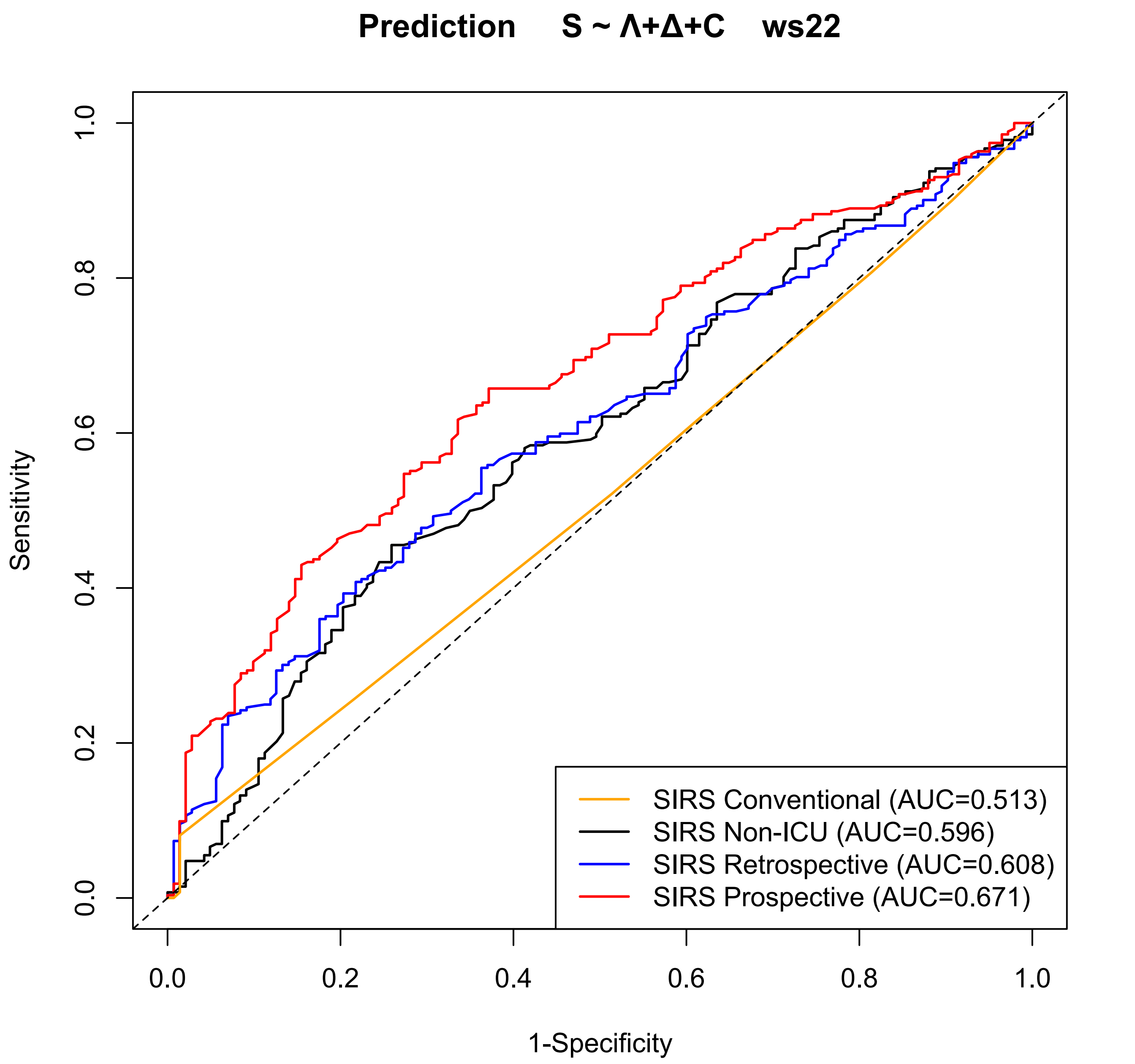

Prediction S ~ Λ+Δ+C ws22
Sensitivity
1-Specificity
0.0
0.2
0.4
0.6
0.8
1.0
0.0
0.2
0.4
0.6
0.8
1.0
SIRS Conventional (AUC=0.513)
SIRS Non-ICU (AUC=0.596)
SIRS Retrospective (AUC=0.608)
SIRS Prospective (AUC=0.671)

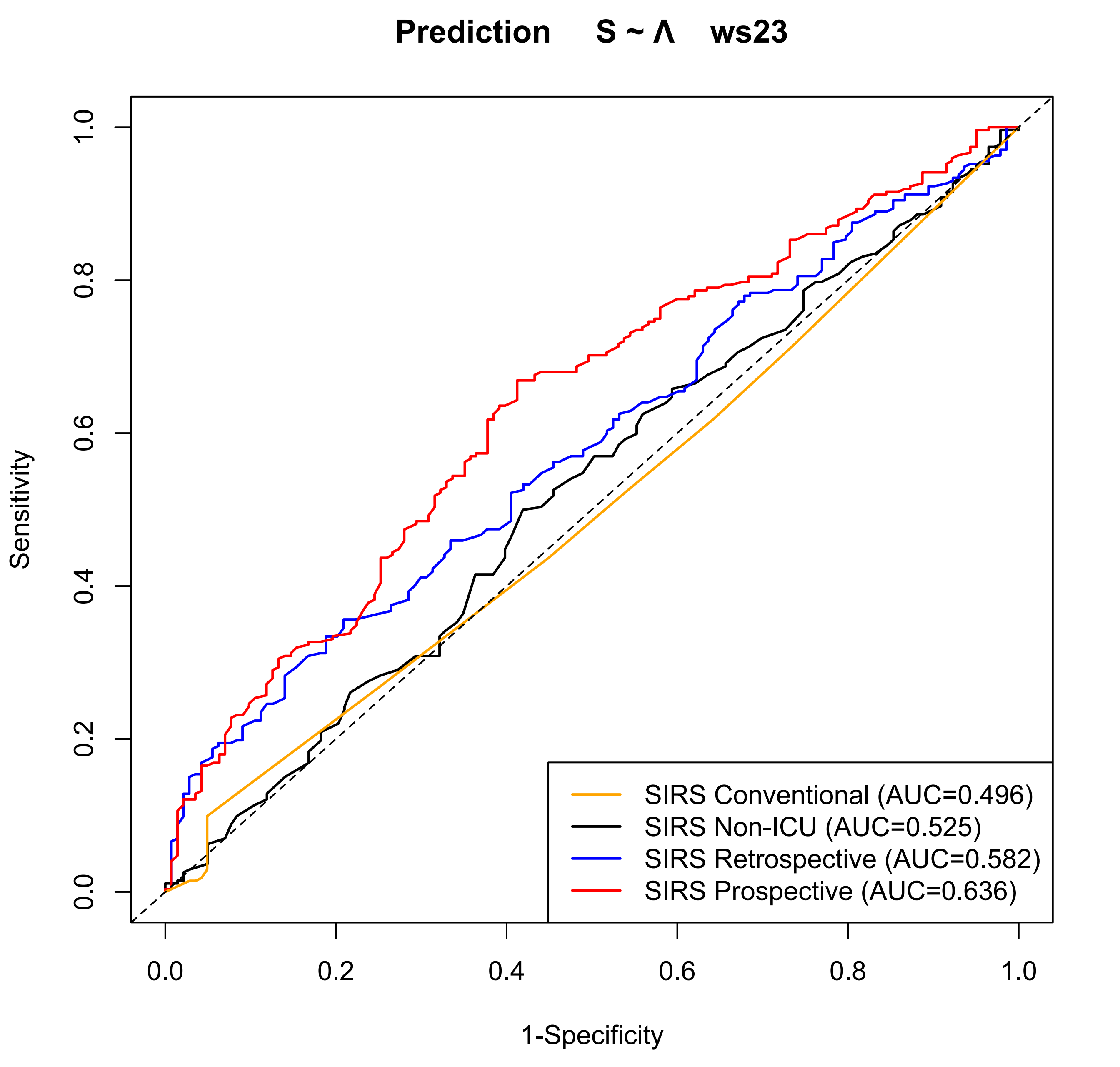
Prediction S ~ Λ ws23
Sensitivity
1-Specificity
0.0
0.2
0.4
0.6
0.8
1.0
0.0
0.2
0.4
0.6
0.8
1.0
SIRS Conventional (AUC=0.496)
SIRS Non-ICU (AUC=0.525)
SIRS Retrospective (AUC=0.582)
SIRS Prospective (AUC=0.636)

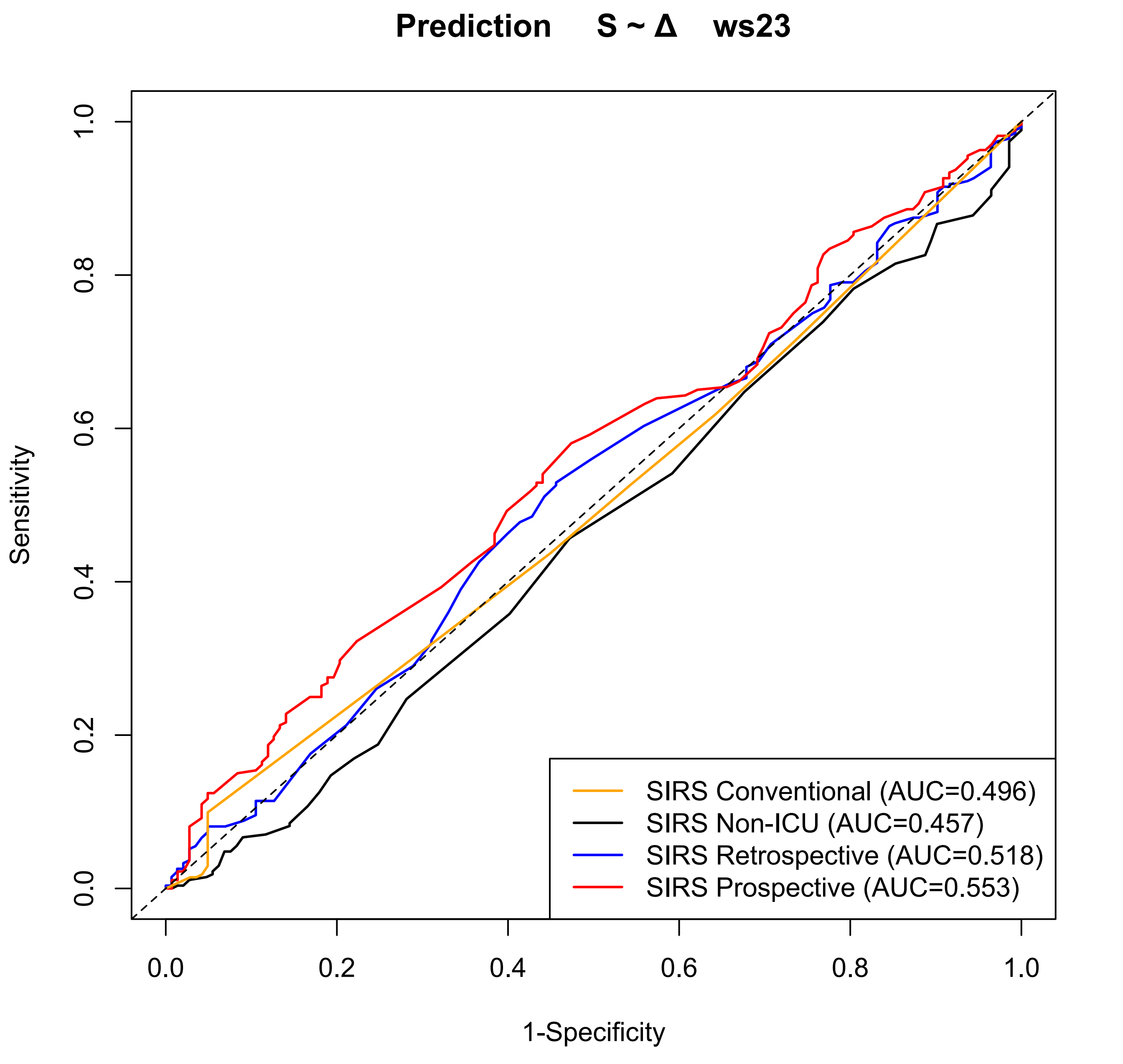

Prediction S ~ Δ ws23
Sensitivity
1-Specificity
0.0
0.2
0.4
0.6
0.8
1.0
SIRS Conventional (AUC=0.496)
SIRS Non-ICU (AUC=0.457)
SIRS Retrospective (AUC=0.518)
SIRS Prospective (AUC=0.553)

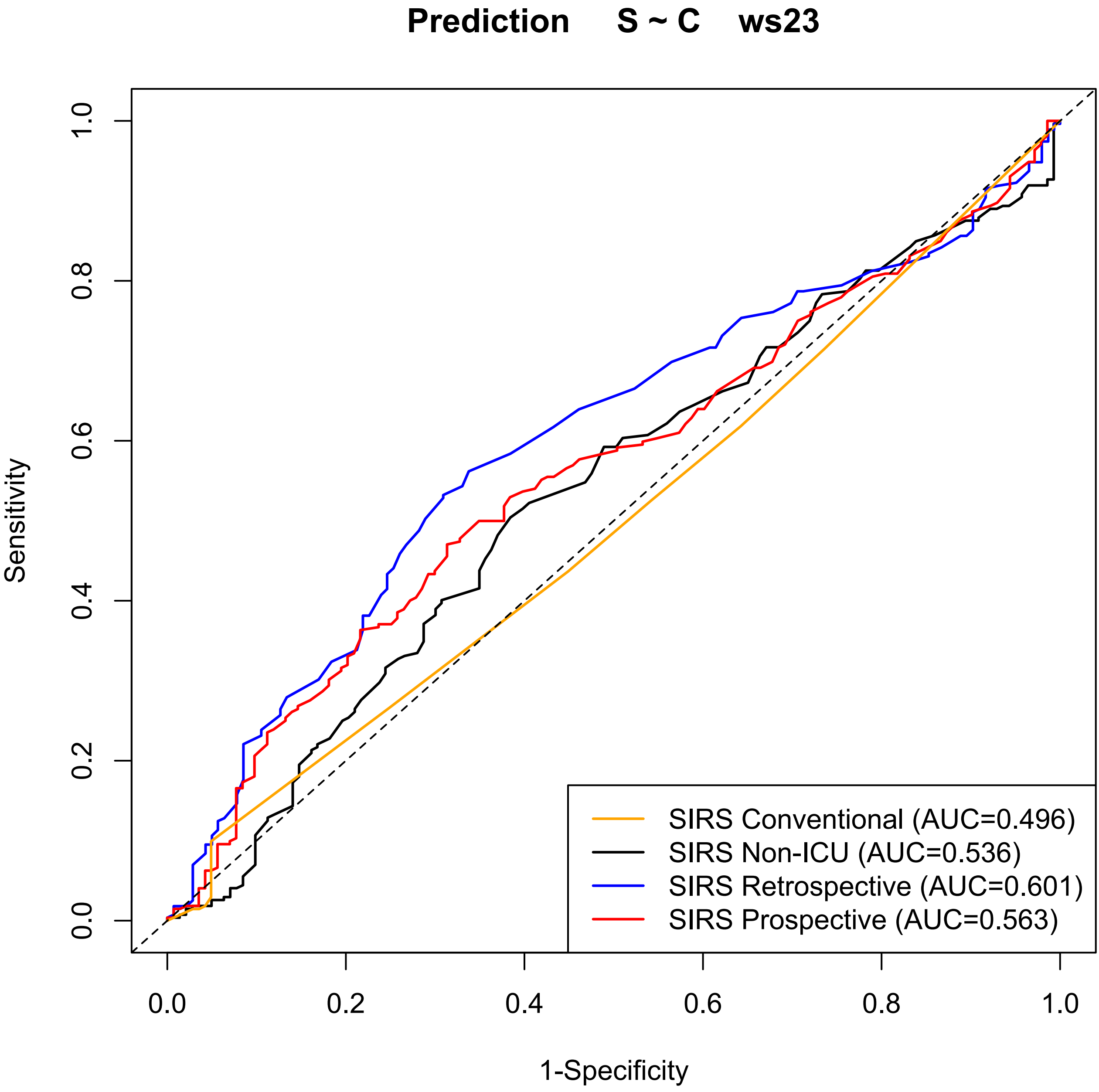

Prediction S ~ C ws23
Sensitivity
1-Specificity
0.0
0.2
0.4
0.6
0.8
1.0
SIRS Conventional (AUC=0.496)
SIRS Non-ICU (AUC=0.536)
SIRS Retrospective (AUC=0.601)
SIRS Prospective (AUC=0.563)

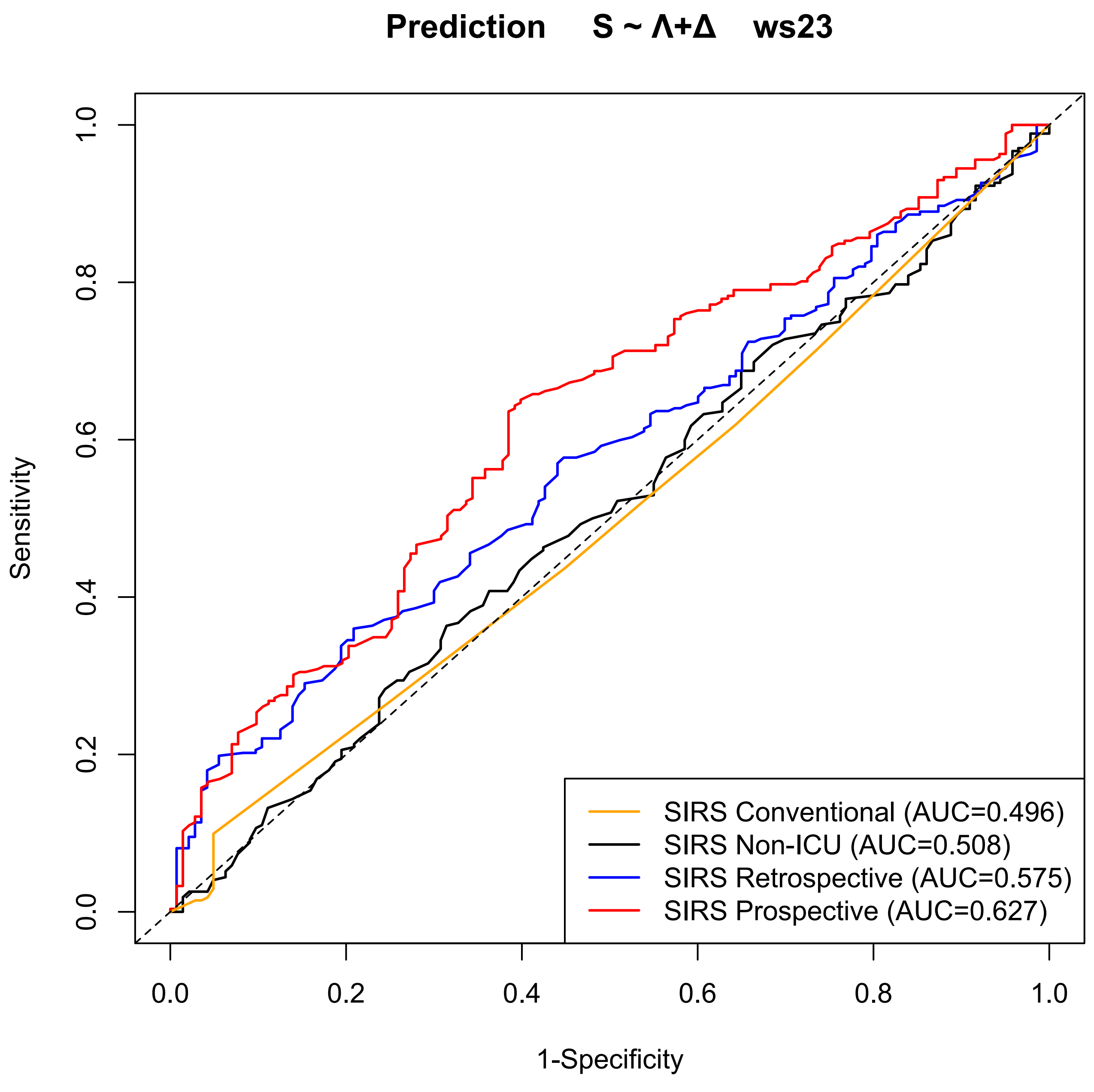

Prediction S ~ Λ+Δ ws23
Sensitivity
1-Specificity
0.0
0.2
0.4
0.6
0.8
1.0
SIRS Conventional (AUC=0.496)
SIRS Non-ICU (AUC=0.508)
SIRS Retrospective (AUC=0.575)
SIRS Prospective (AUC=0.627)

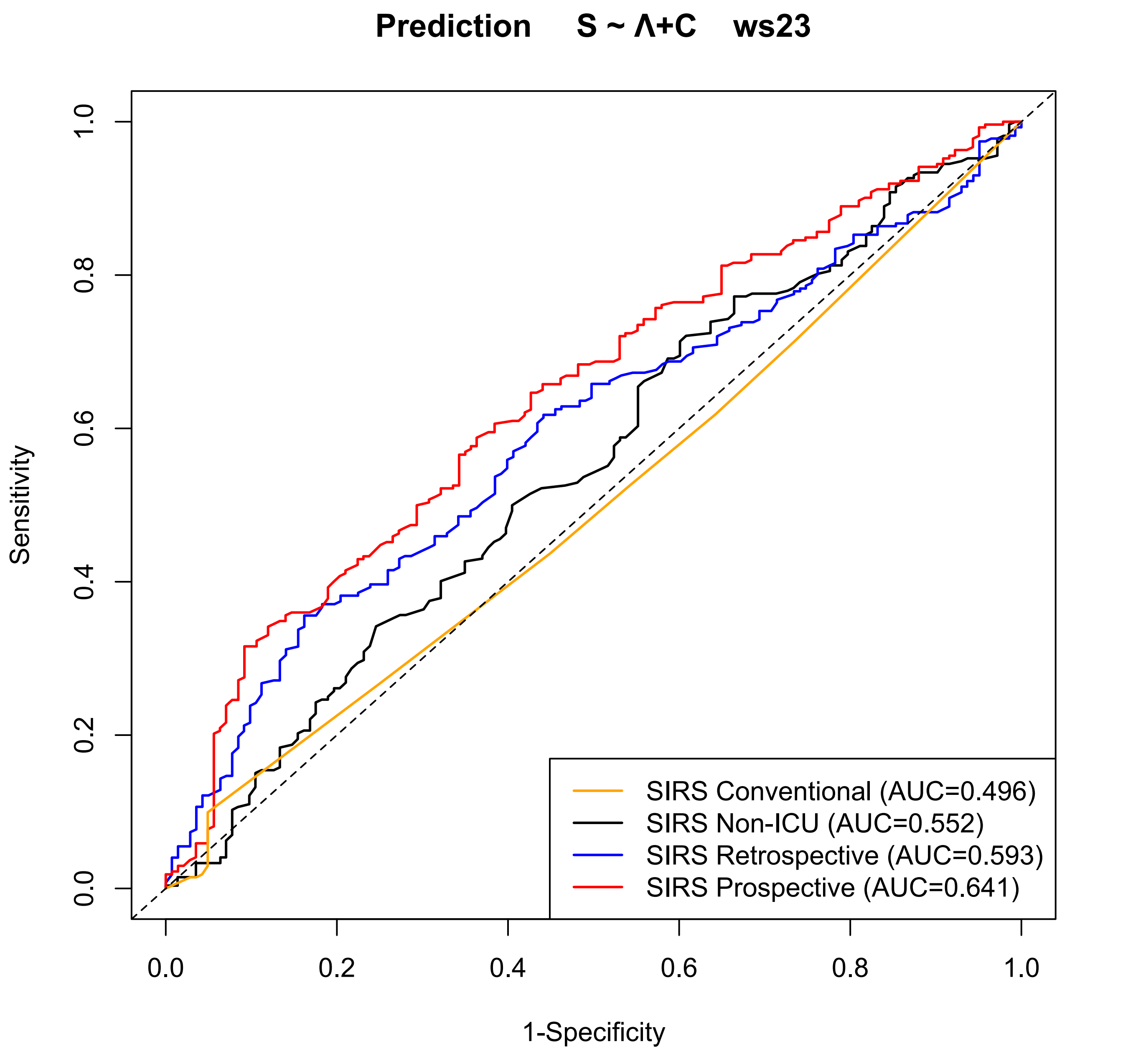

Prediction S ~ Λ+C ws23
Sensitivity
1-Specificity
0.0
0.2
0.4
0.6
0.8
1.0
SIRS Conventional (AUC=0.496)
SIRS Non-ICU (AUC=0.552)
SIRS Retrospective (AUC=0.593)
SIRS Prospective (AUC=0.641)

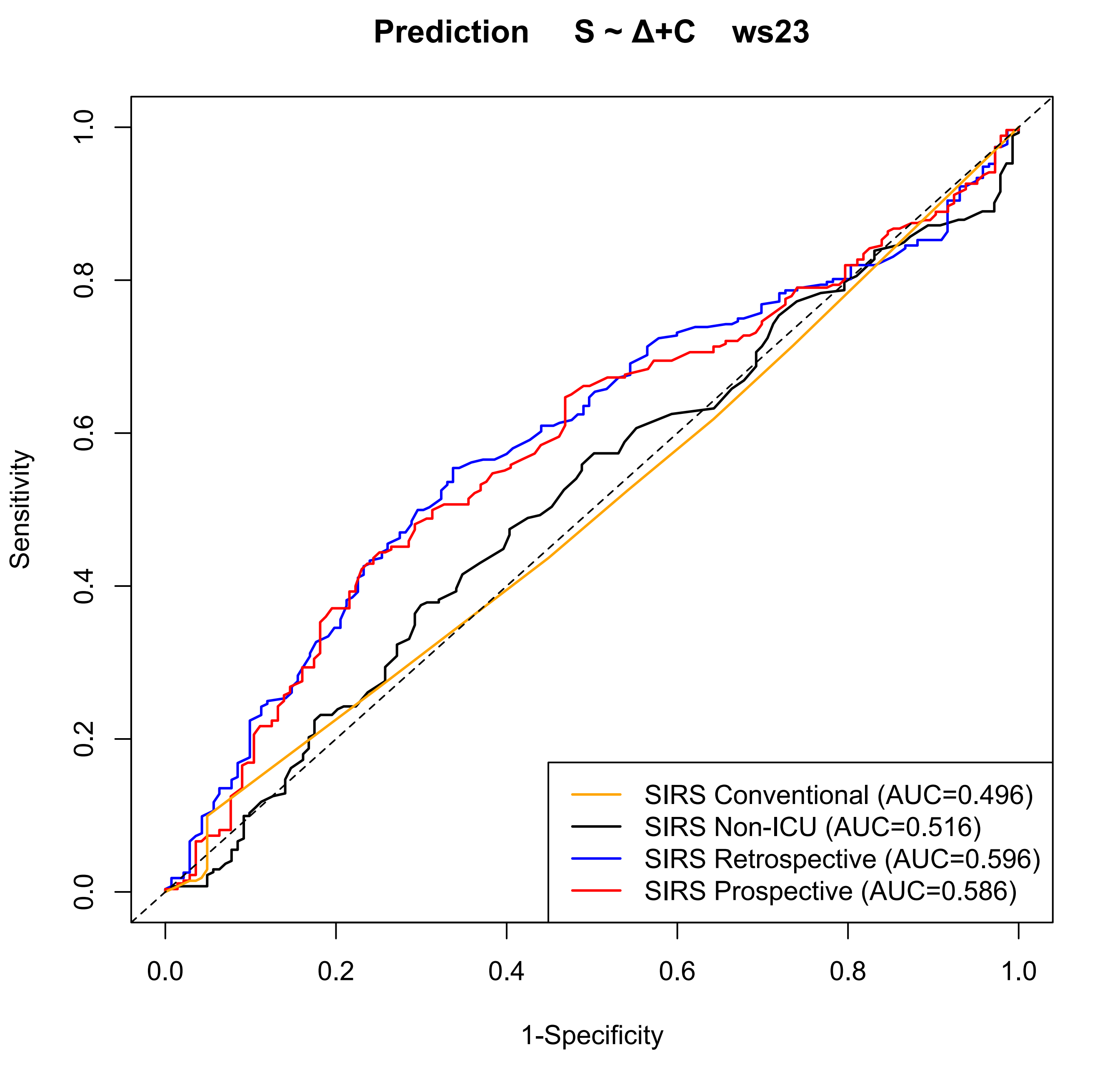

Prediction S ~ Δ+C ws23
Sensitivity
1-Specificity
0.0
0.2
0.4
0.6
0.8
1.0
SIRS Conventional (AUC=0.496)
SIRS Non-ICU (AUC=0.516)
SIRS Retrospective (AUC=0.596)
SIRS Prospective (AUC=0.586)

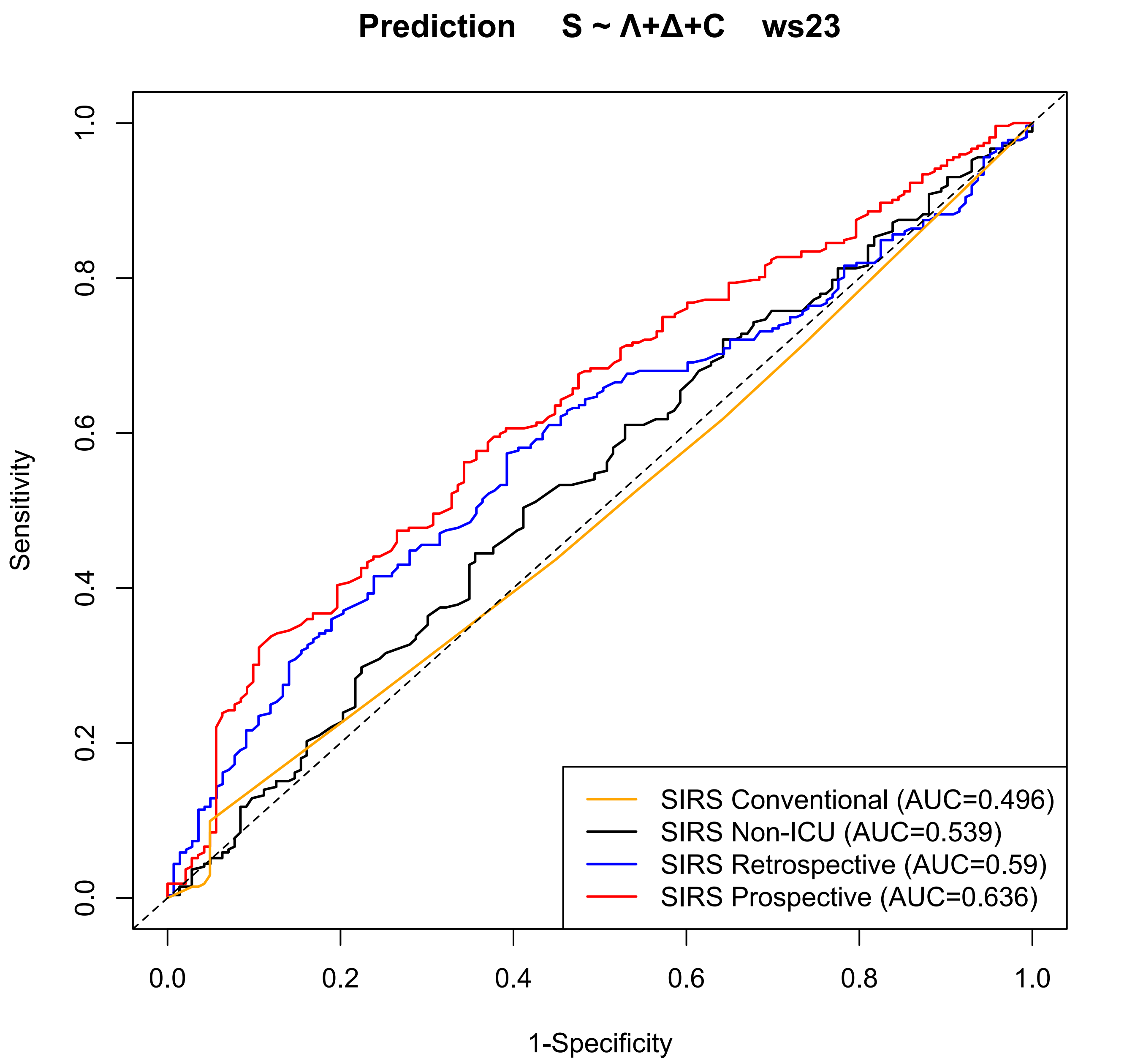

Prediction S ~ Λ+Δ+C ws23
Sensitivity
1-Specificity
0.0
0.2
0.4
0.6
0.8
1.0
0.0
0.2
0.4
0.6
0.8
1.0
SIRS Conventional (AUC=0.496)
SIRS Non-ICU (AUC=0.539)
SIRS Retrospective (AUC=0.59)
SIRS Prospective (AUC=0.636)

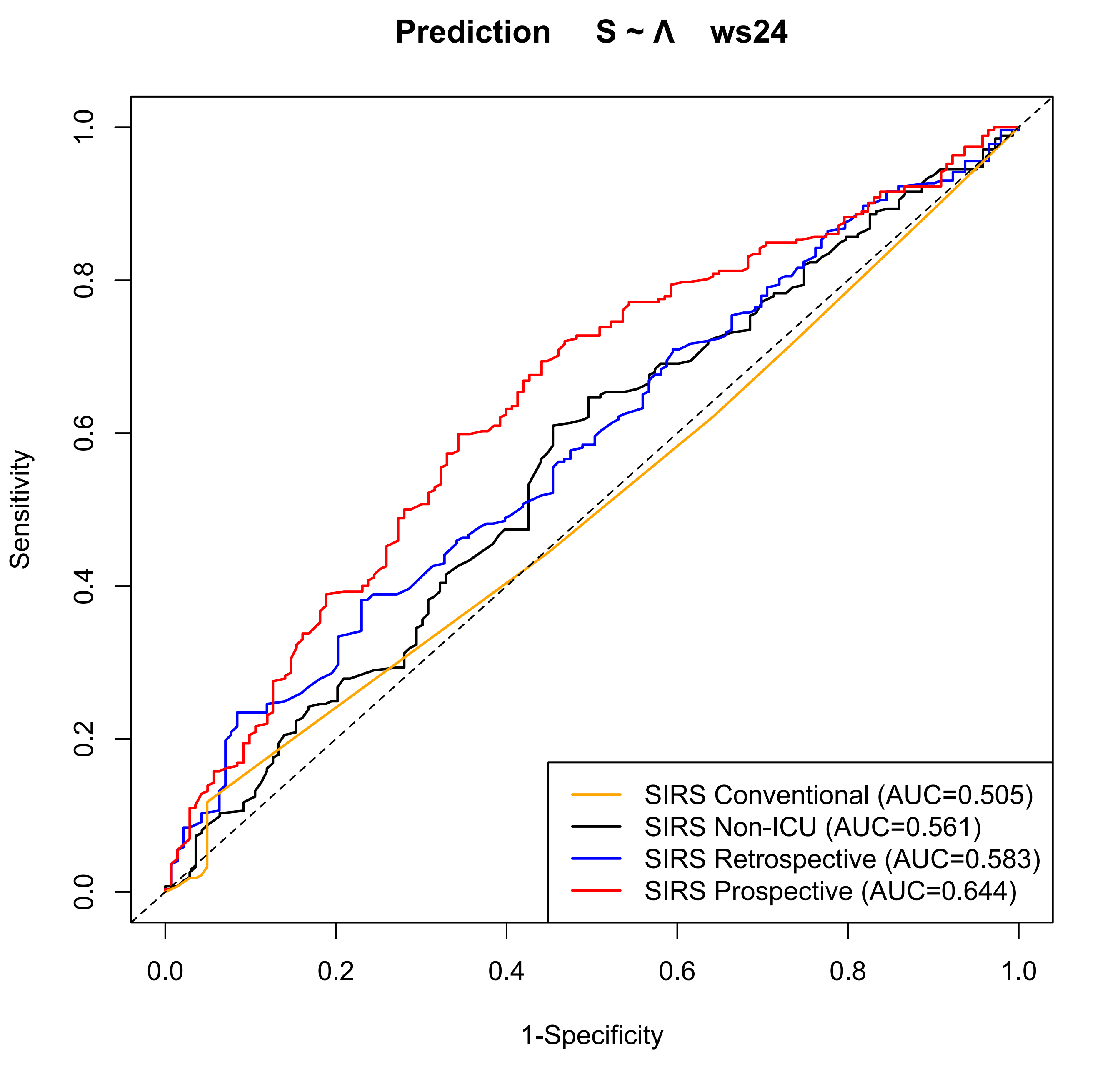
Prediction S ~ Λ ws24
Sensitivity
1-Specificity
0.0
0.2
0.4
0.6
0.8
1.0
SIRS Conventional (AUC=0.505)
SIRS Non-ICU (AUC=0.561)
SIRS Retrospective (AUC=0.583)
SIRS Prospective (AUC=0.644)

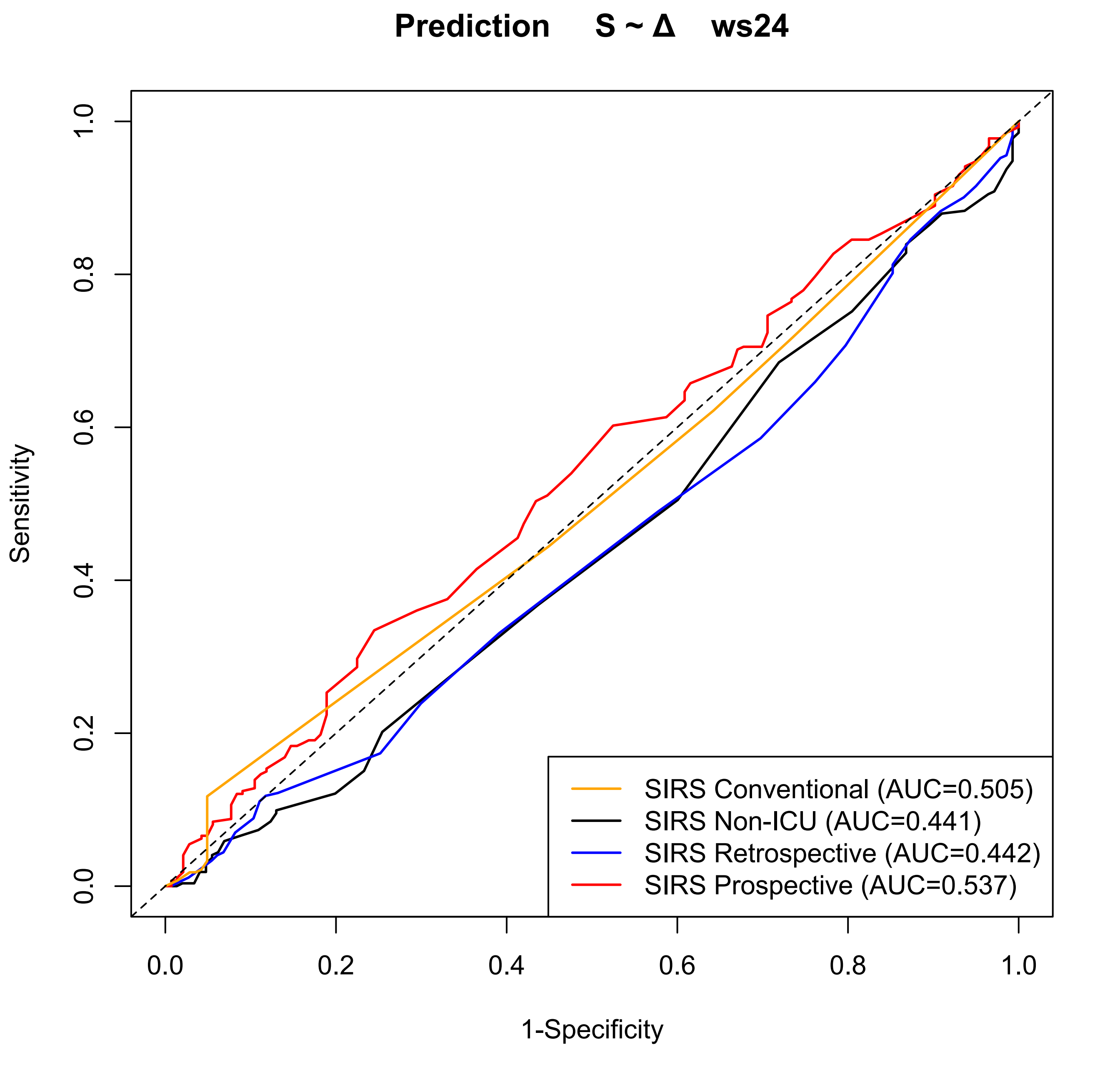

Prediction S ~ Δ ws24
Sensitivity
1-Specificity
0.0
0.2
0.4
0.6
0.8
1.0
SIRS Conventional (AUC=0.505)
SIRS Non-ICU (AUC=0.441)
SIRS Retrospective (AUC=0.442)
SIRS Prospective (AUC=0.537)

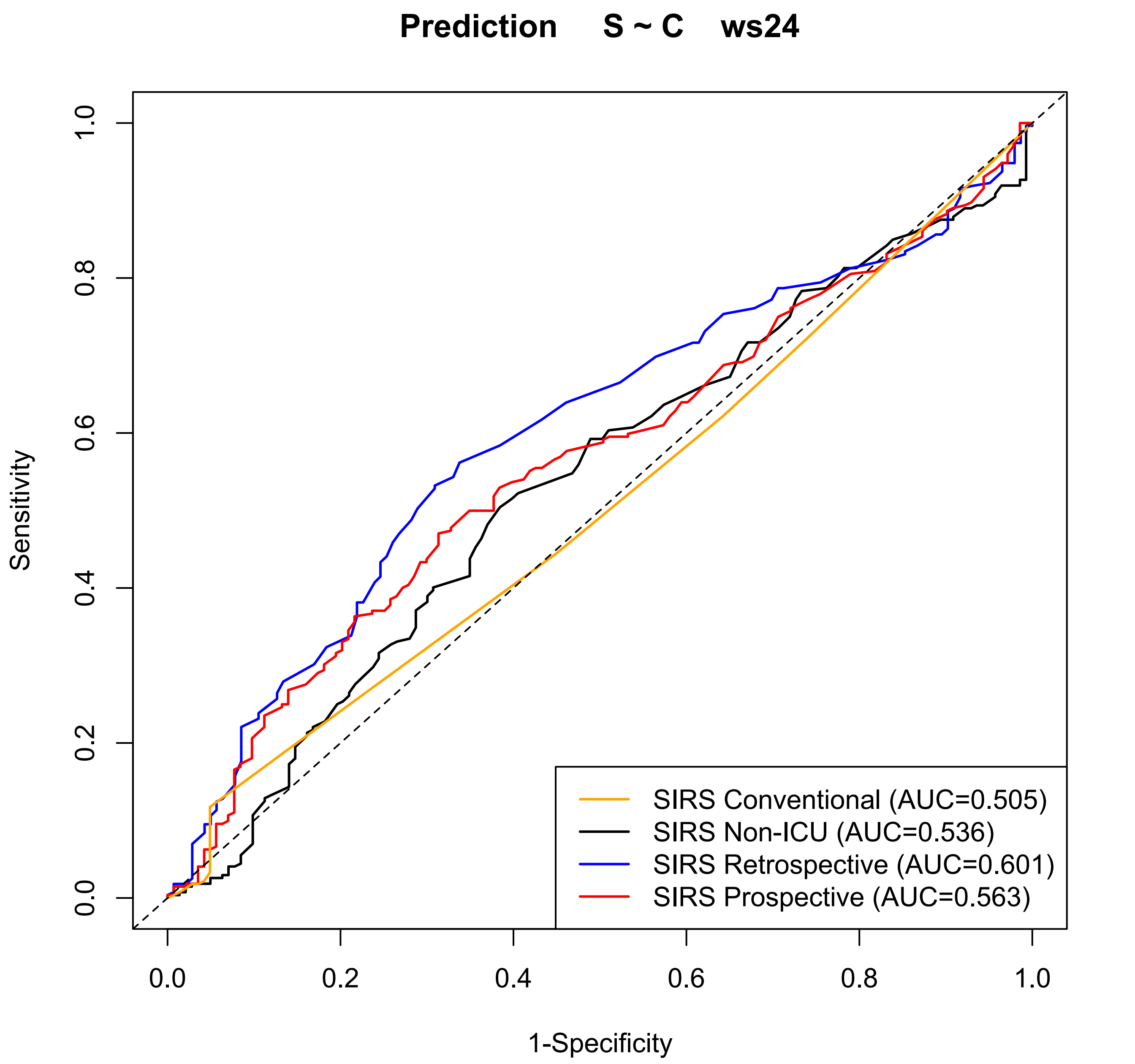

Prediction S ~ C ws24
Sensitivity
1-Specificity
0.0
0.2
0.4
0.6
0.8
1.0
SIRS Conventional (AUC=0.505)
SIRS Non-ICU (AUC=0.536)
SIRS Retrospective (AUC=0.601)
SIRS Prospective (AUC=0.563)

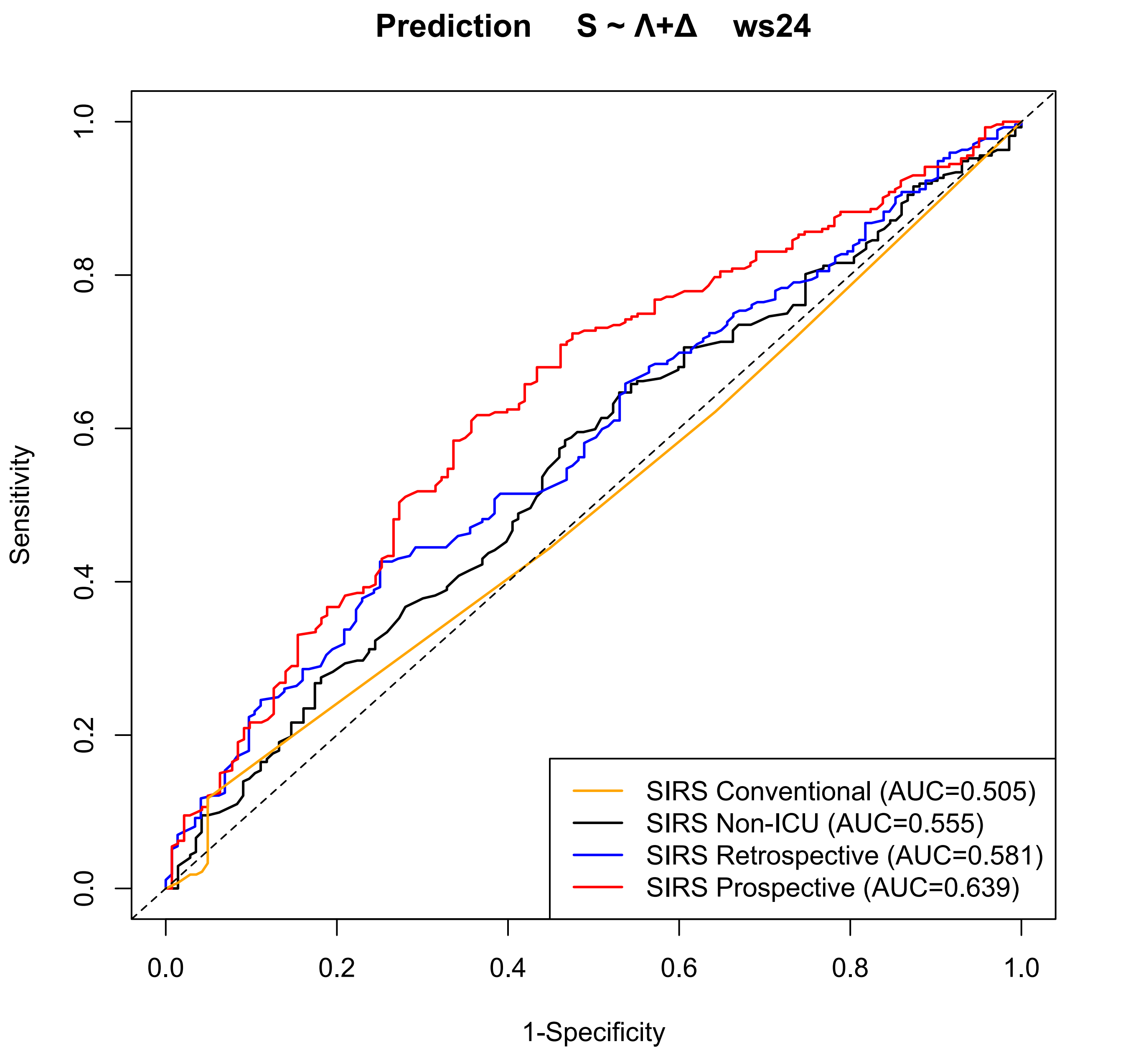

Prediction S ~ Λ+Δ ws24
Sensitivity
1-Specificity
0.0
0.2
0.4
0.6
0.8
1.0
SIRS Conventional (AUC=0.505)
SIRS Non-ICU (AUC=0.555)
SIRS Retrospective (AUC=0.581)
SIRS Prospective (AUC=0.639)

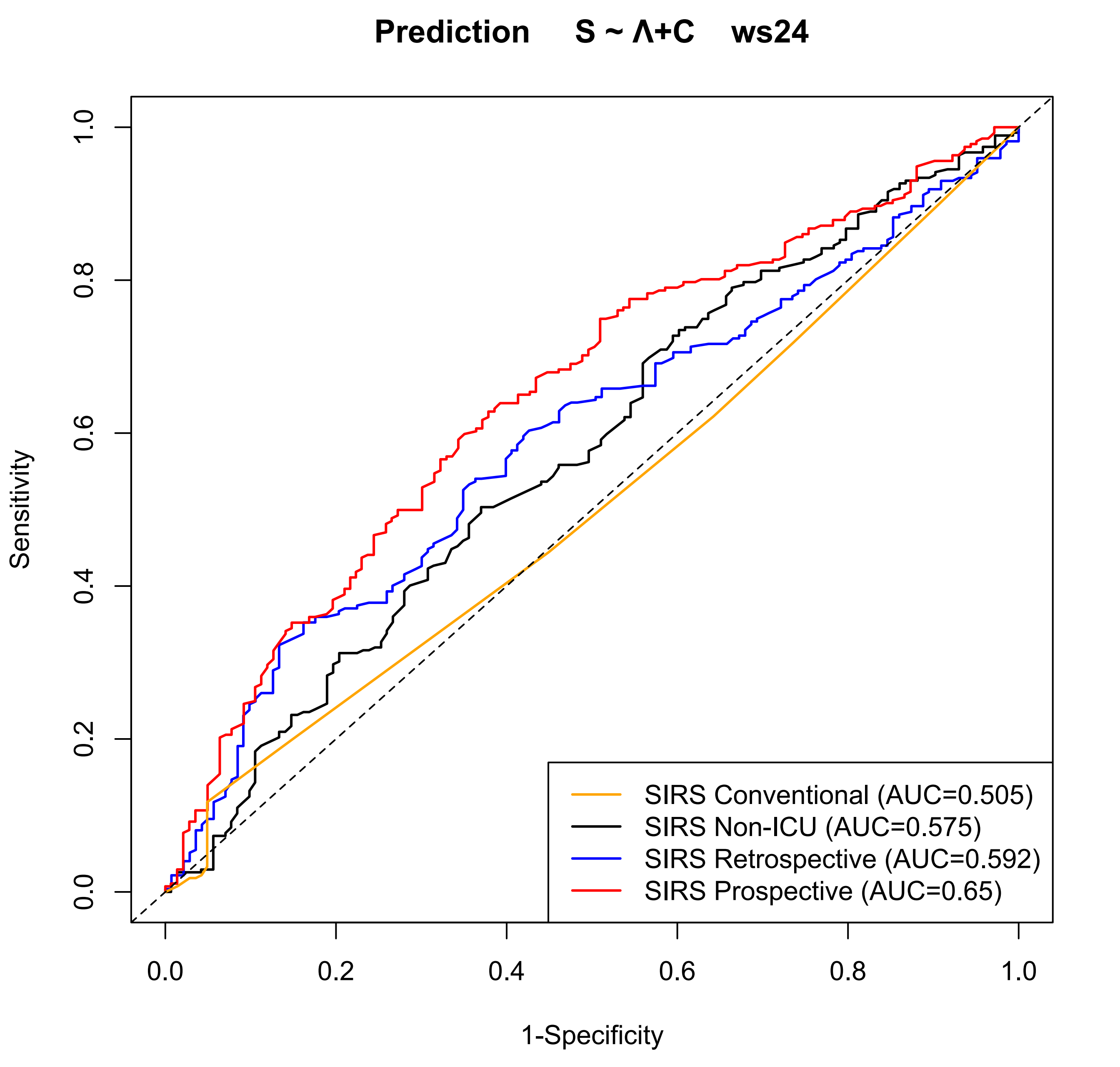

Prediction S ~ Λ+C ws24
Sensitivity
1-Specificity
0.0
0.2
0.4
0.6
0.8
1.0
SIRS Conventional (AUC=0.505)
SIRS Non-ICU (AUC=0.575)
SIRS Retrospective (AUC=0.592)
SIRS Prospective (AUC=0.65)

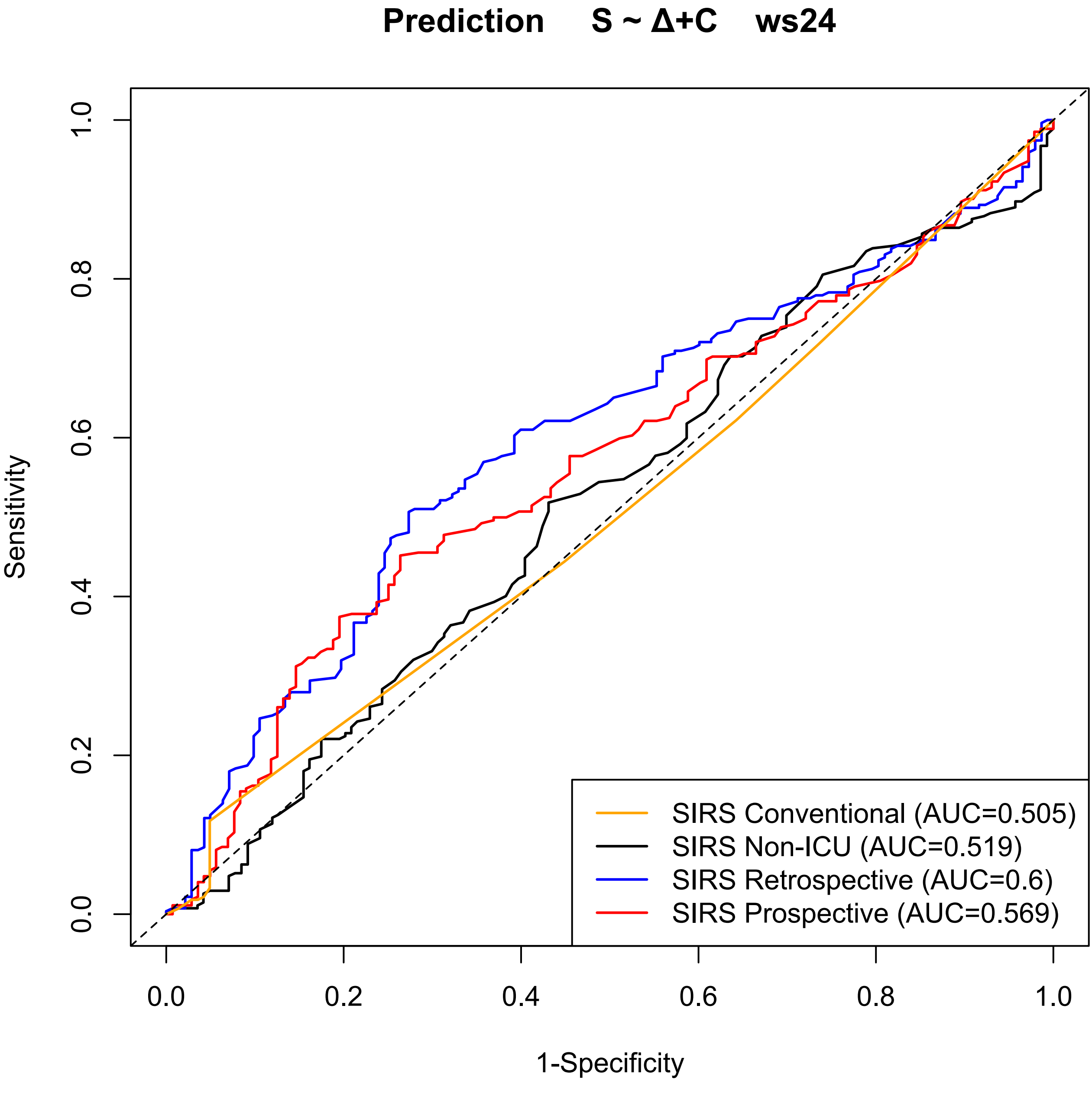
Prediction S ~ Δ+C ws24
Sensitivity
1-Specificity
0.0
0.2
0.4
0.6
0.8
1.0
SIRS Conventional (AUC=0.505)
SIRS Non-ICU (AUC=0.519)
SIRS Retrospective (AUC=0.6)
SIRS Prospective (AUC=0.569)

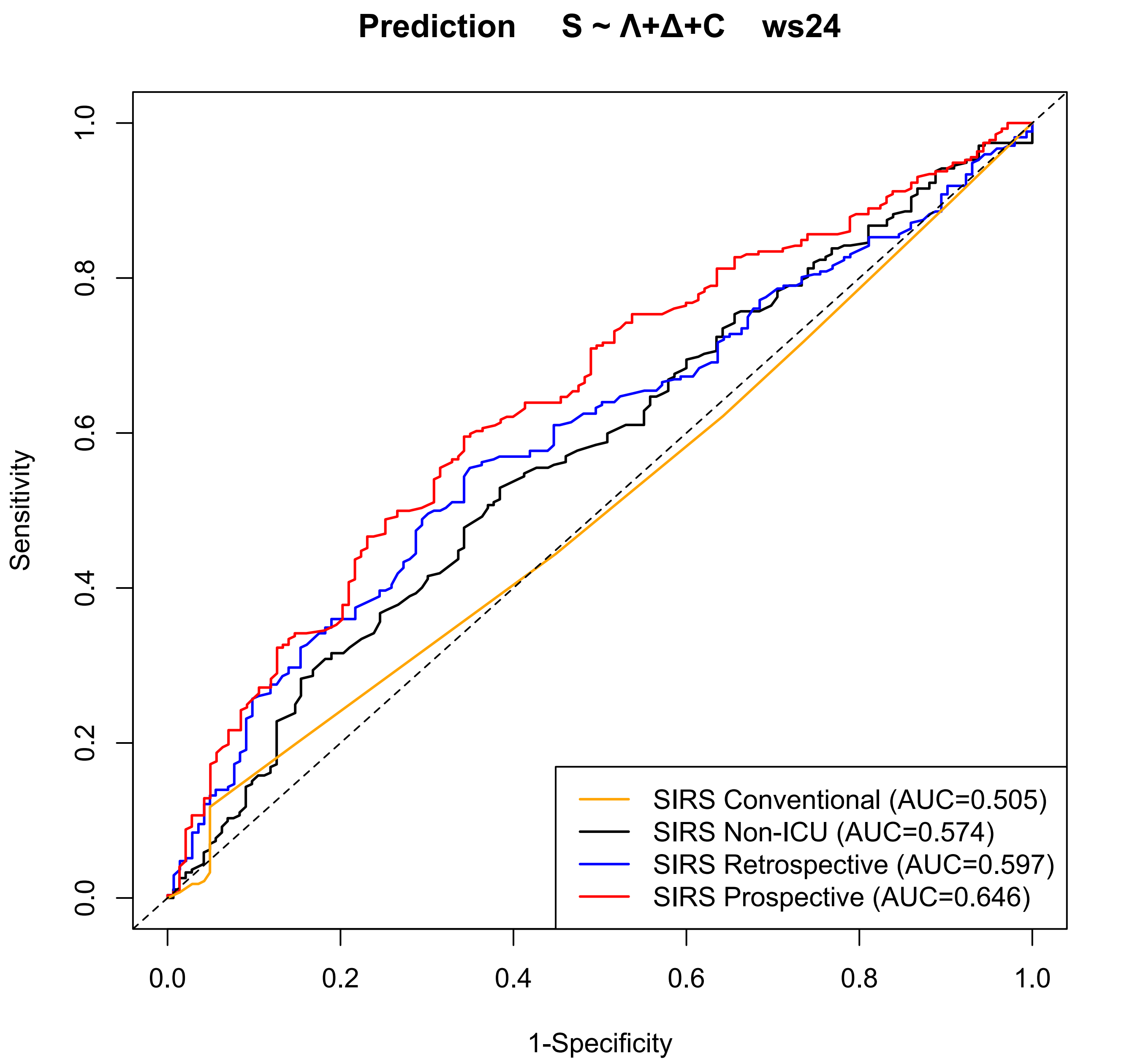

Prediction S ~ Λ+Δ+C ws24
Sensitivity
1-Specificity
0.0
0.2
0.4
0.6
0.8
1.0
SIRS Conventional (AUC=0.505)
SIRS Non-ICU (AUC=0.574)
SIRS Retrospective (AUC=0.597)
SIRS Prospective (AUC=0.646)

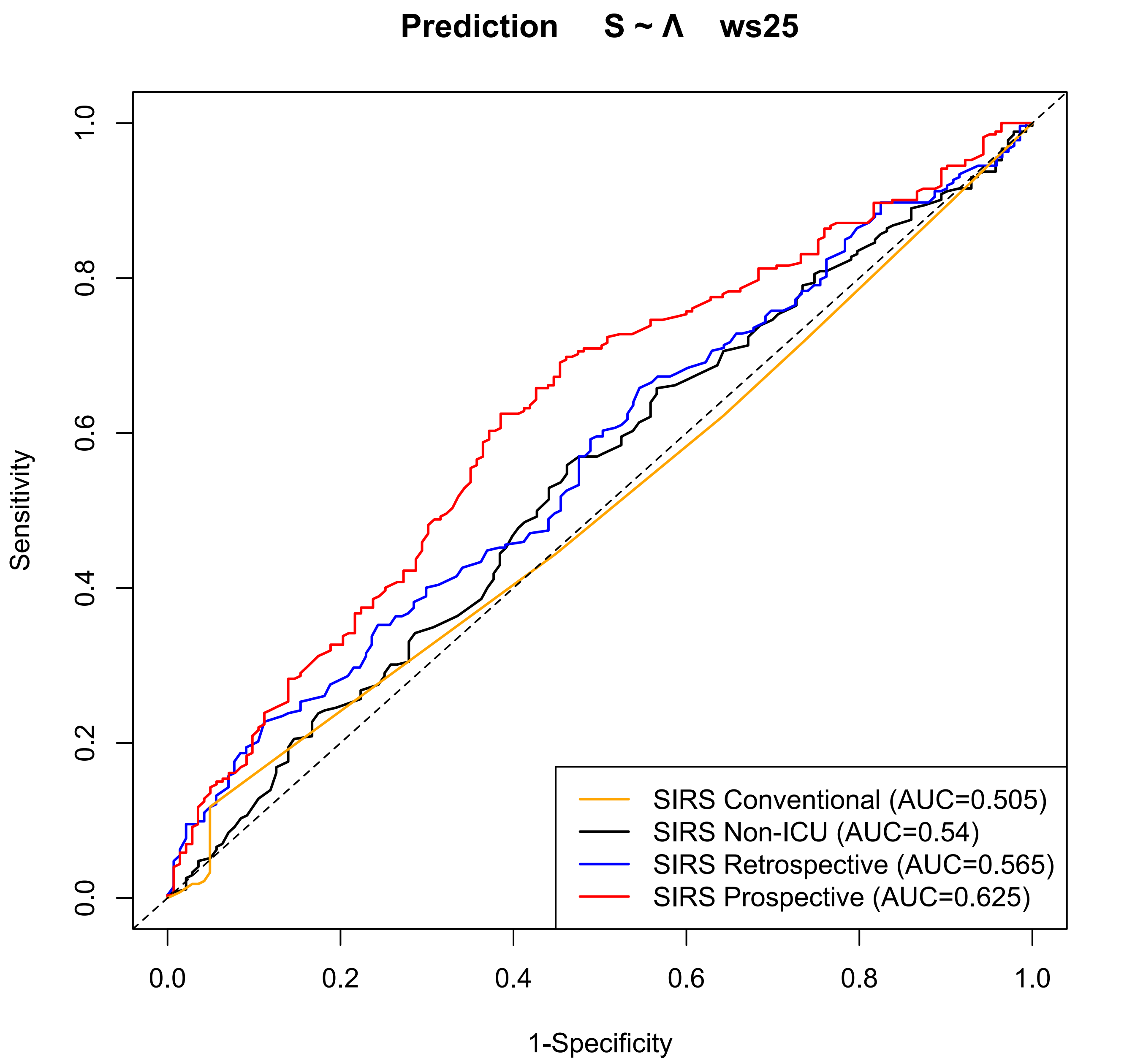
Prediction S ~ Λ ws25
Sensitivity
1-Specificity
0.0
0.2
0.4
0.6
0.8
1.0
0.0
0.2
0.4
0.6
0.8
1.0
SIRS Conventional (AUC=0.505)
SIRS Non-ICU (AUC=0.54)
SIRS Retrospective (AUC=0.565)
SIRS Prospective (AUC=0.625)

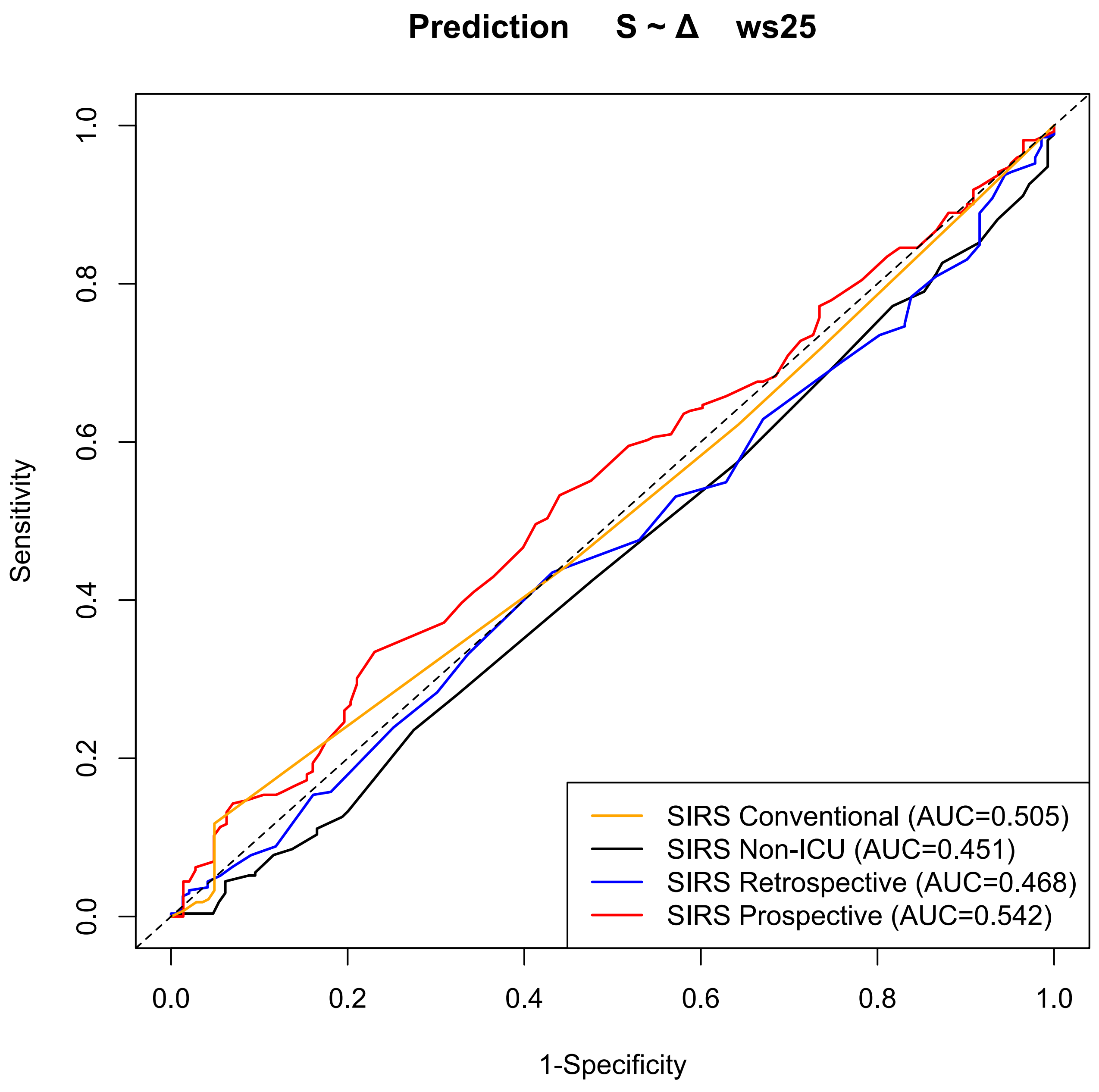

Prediction S ~ Δ ws25
Sensitivity
1-Specificity
0.0
0.2
0.4
0.6
0.8
1.0
0.0
0.2
0.4
0.6
0.8
1.0
SIRS Conventional (AUC=0.505)
SIRS Non-ICU (AUC=0.451)
SIRS Retrospective (AUC=0.468)
SIRS Prospective (AUC=0.542)

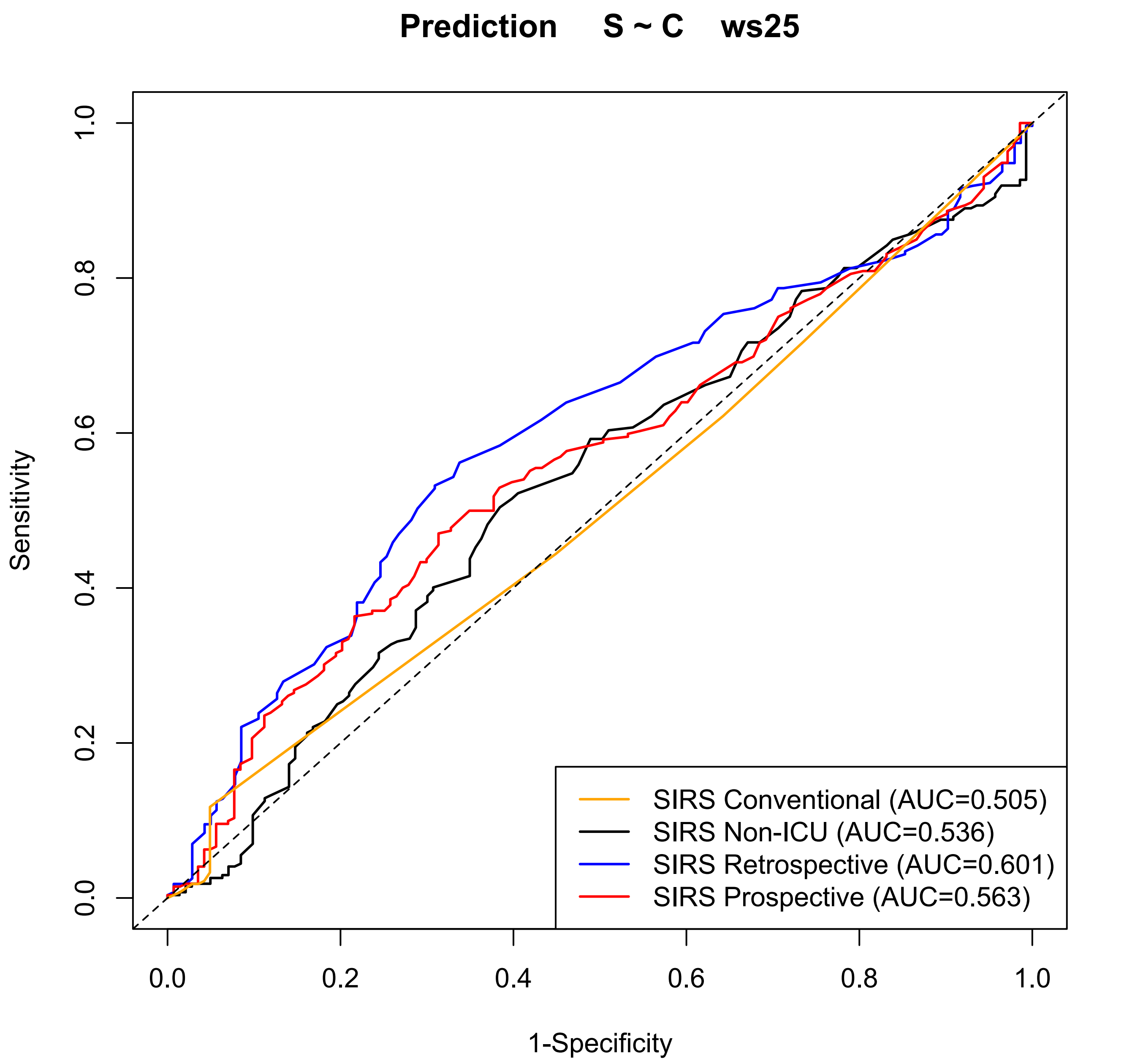

Prediction S ~ C ws25
Sensitivity
1-Specificity
0.0
0.2
0.4
0.6
0.8
1.0
SIRS Conventional (AUC=0.505)
SIRS Non-ICU (AUC=0.536)
SIRS Retrospective (AUC=0.601)
SIRS Prospective (AUC=0.563)

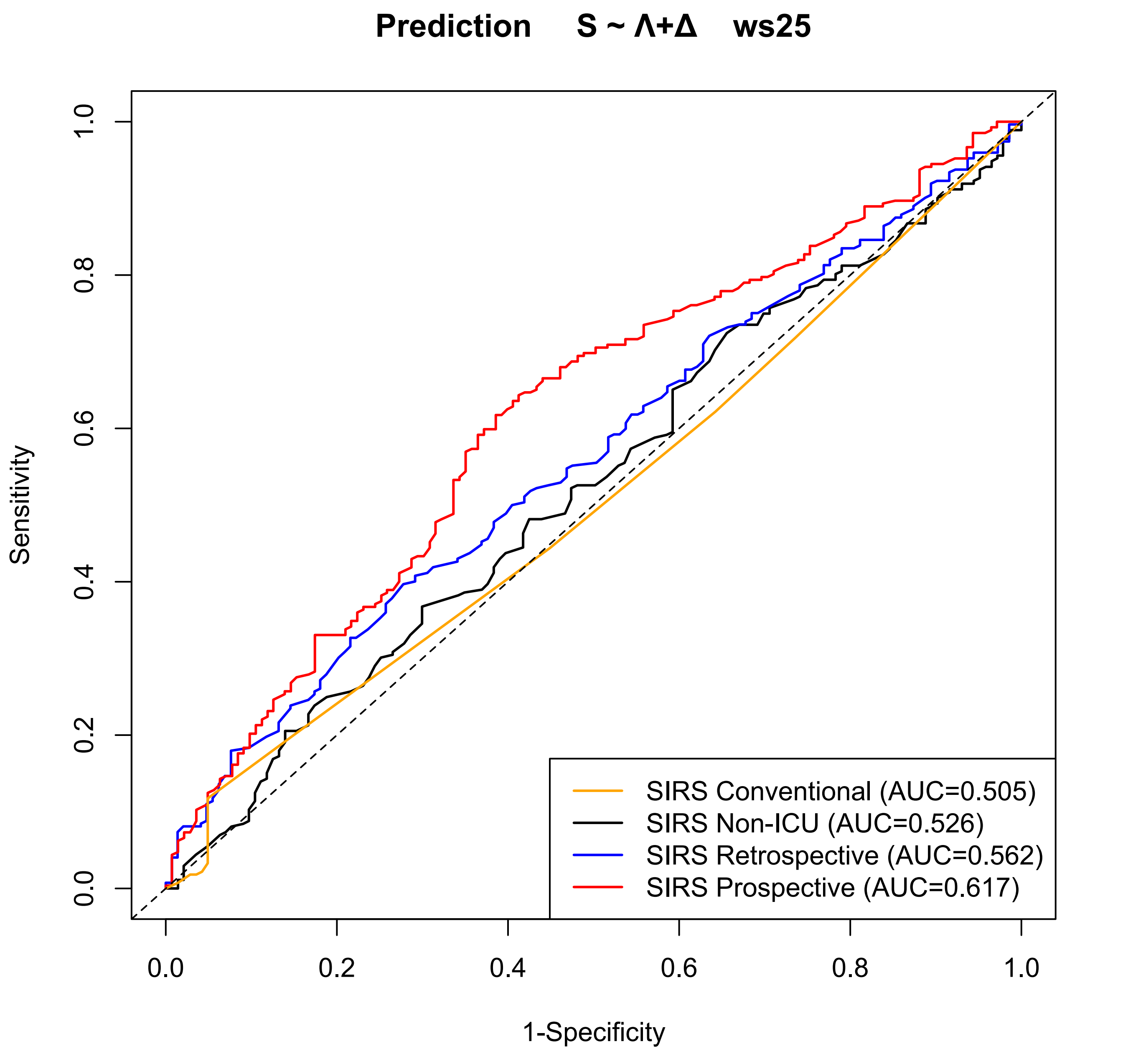

Prediction S ~ Λ+Δ ws25
Sensitivity
1-Specificity
0.0
0.2
0.4
0.6
0.8
1.0
SIRS Conventional (AUC=0.505)
SIRS Non-ICU (AUC=0.526)
SIRS Retrospective (AUC=0.562)
SIRS Prospective (AUC=0.617)

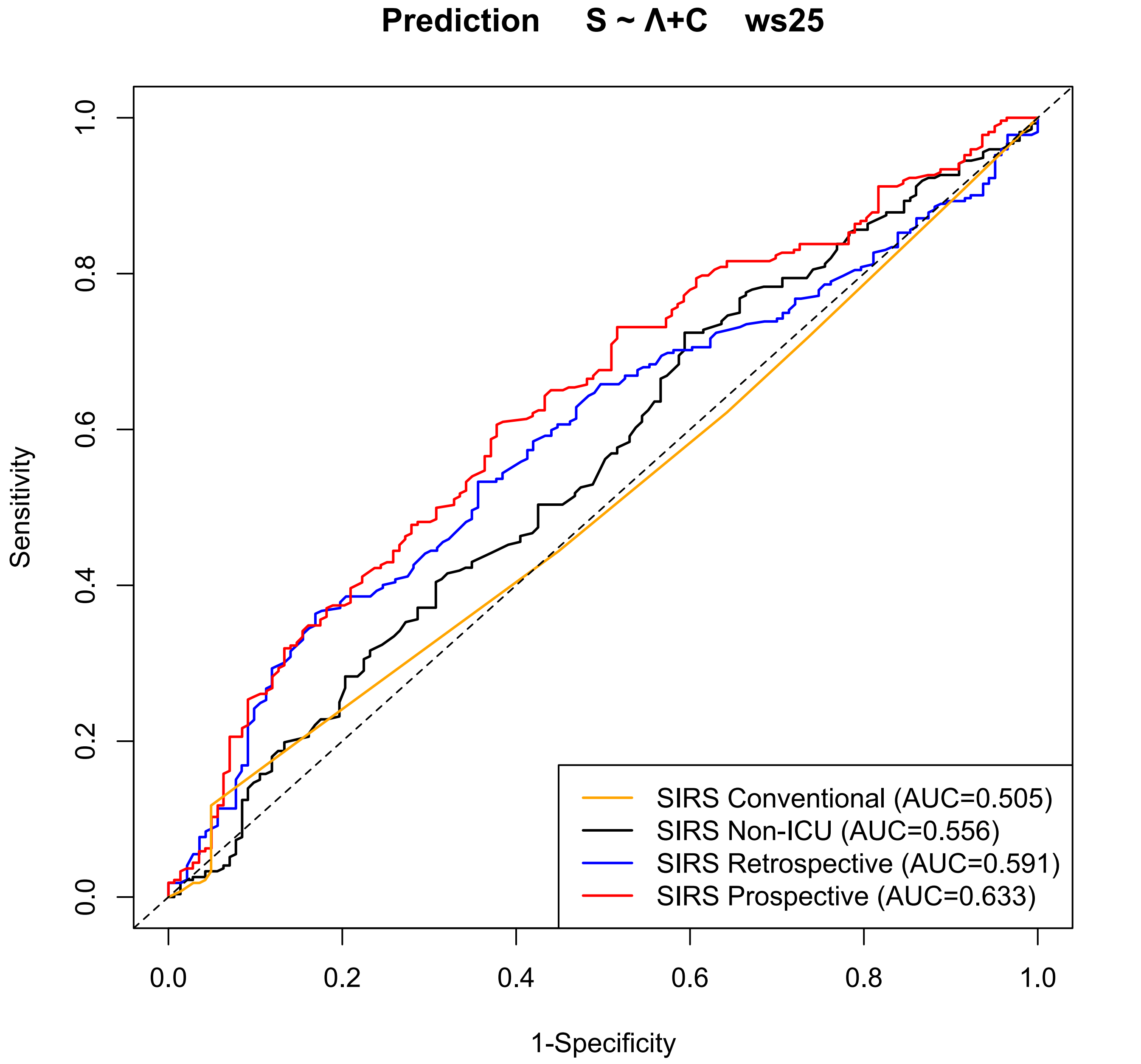
Prediction S ~ Λ+C ws25
Sensitivity
1-Specificity
0.0
0.2
0.4
0.6
0.8
1.0
0.0
0.2
0.4
0.6
0.8
1.0
SIRS Conventional (AUC=0.505)
SIRS Non-ICU (AUC=0.556)
SIRS Retrospective (AUC=0.591)
SIRS Prospective (AUC=0.633)

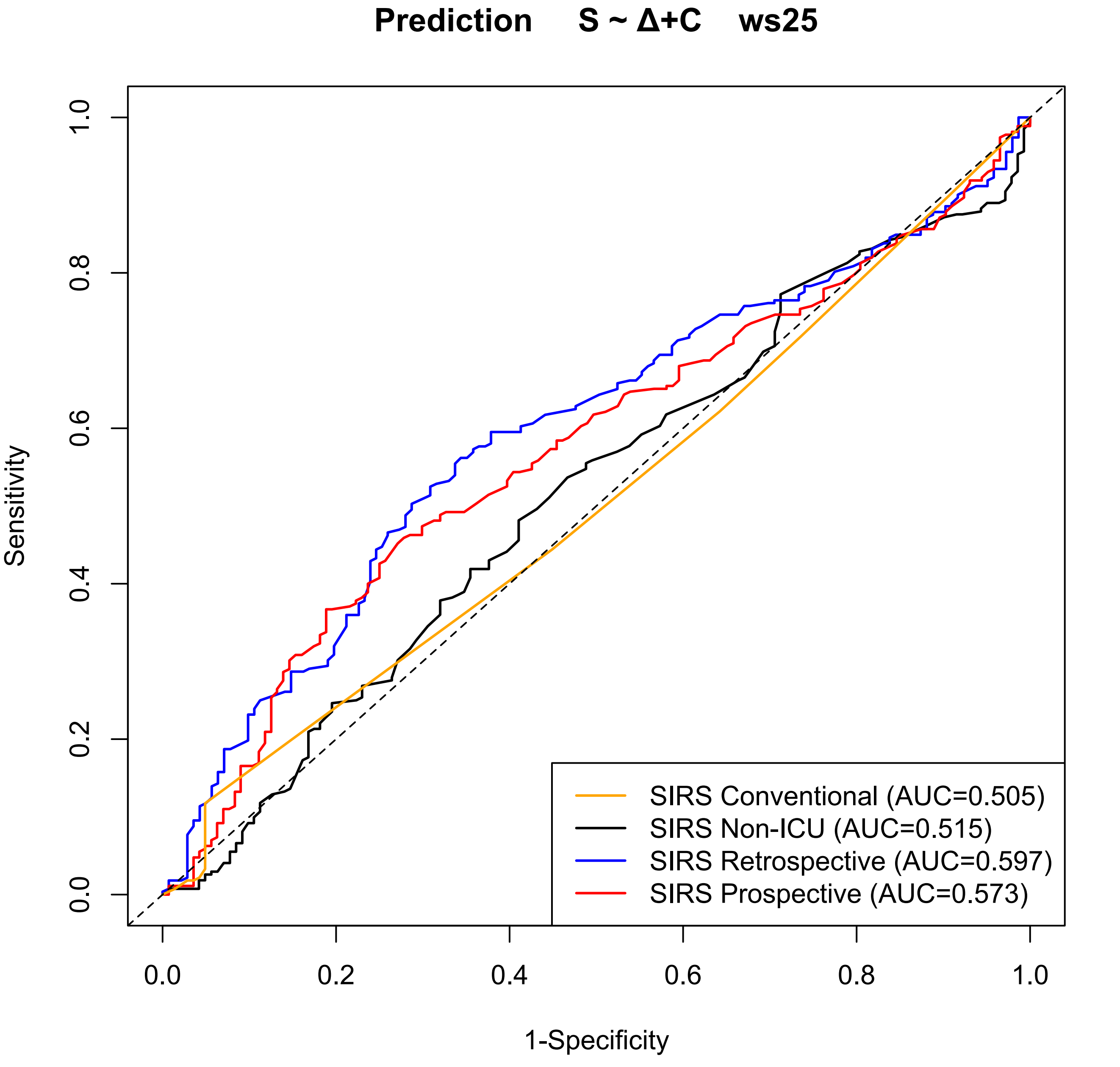

Prediction S ~ Δ+C ws25
Sensitivity
1-Specificity
0.0
0.2
0.4
0.6
0.8
1.0
0.0
0.2
0.4
0.6
0.8
1.0
SIRS Conventional (AUC=0.505)
SIRS Non-ICU (AUC=0.515)
SIRS Retrospective (AUC=0.597)
SIRS Prospective (AUC=0.573)

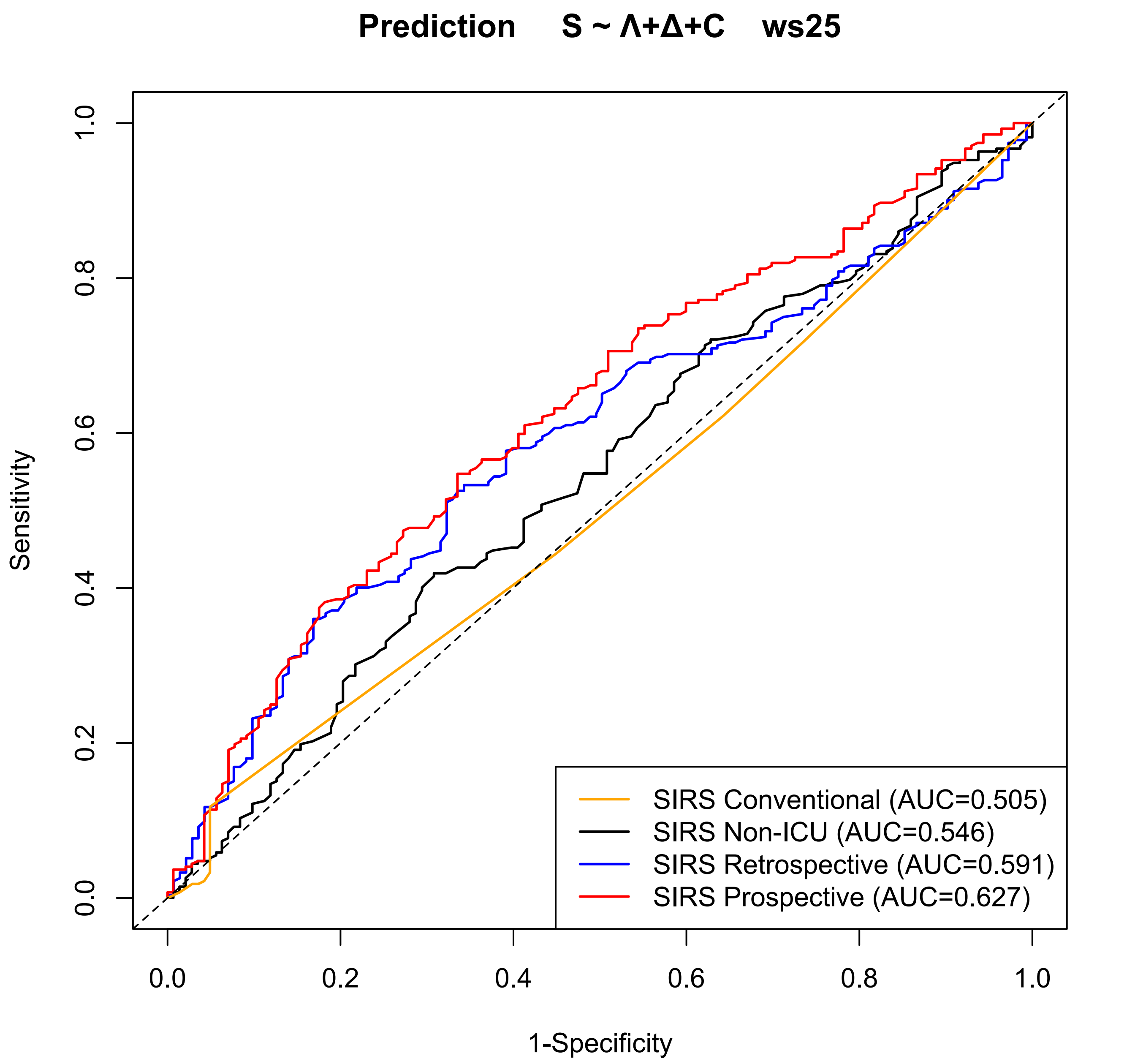
Prediction S ~ Λ+Δ+C ws25
Sensitivity
1-Specificity
0.0
0.2
0.4
0.6
0.8
1.0
SIRS Conventional (AUC=0.505)
SIRS Non-ICU (AUC=0.546)
SIRS Retrospective (AUC=0.591)
SIRS Prospective (AUC=0.627)

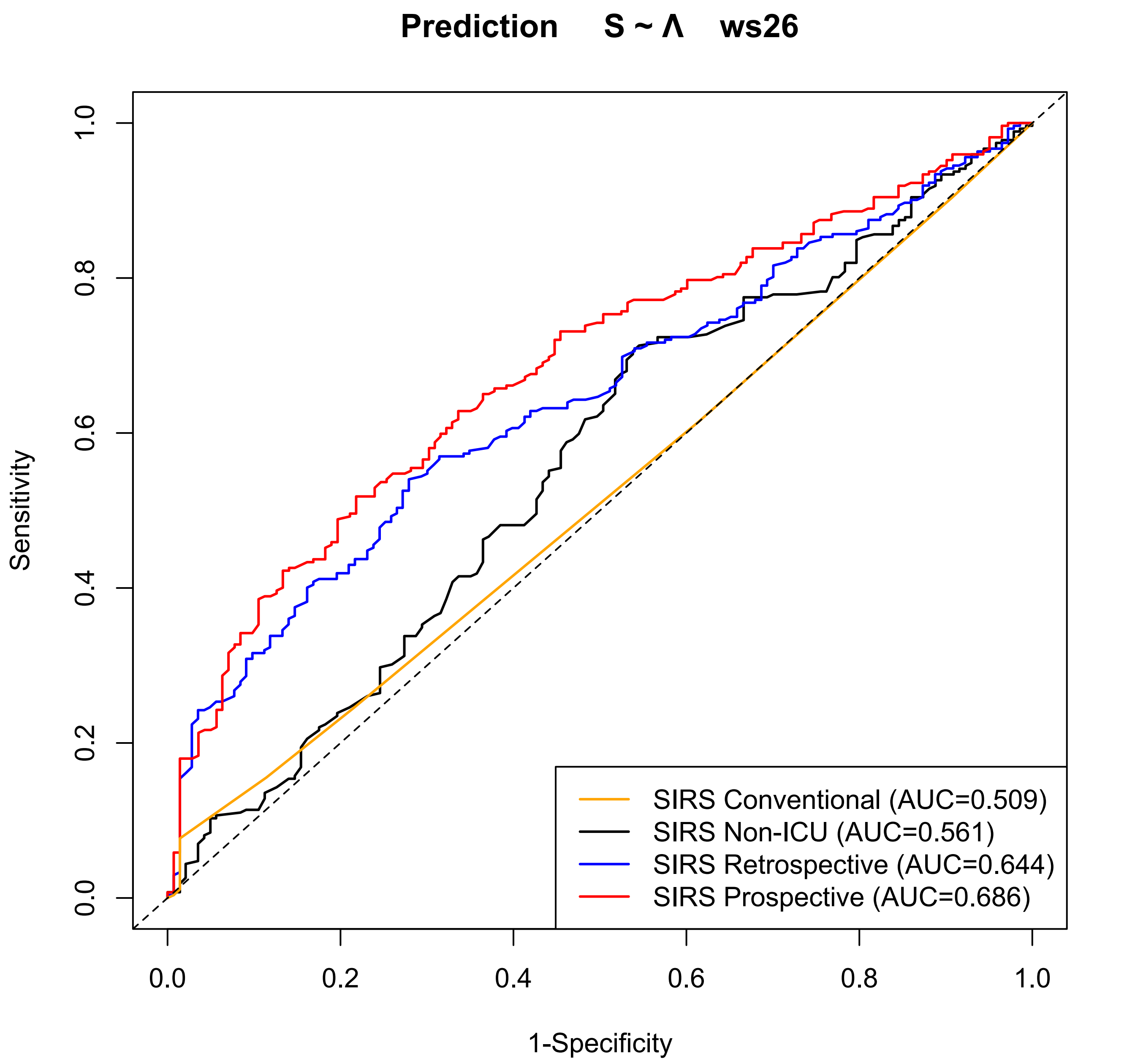
Prediction S ~ Λ ws26
Sensitivity
1-Specificity
0.0
0.2
0.4
0.6
0.8
1.0
SIRS Conventional (AUC=0.509)
SIRS Non-ICU (AUC=0.561)
SIRS Retrospective (AUC=0.644)
SIRS Prospective (AUC=0.686)

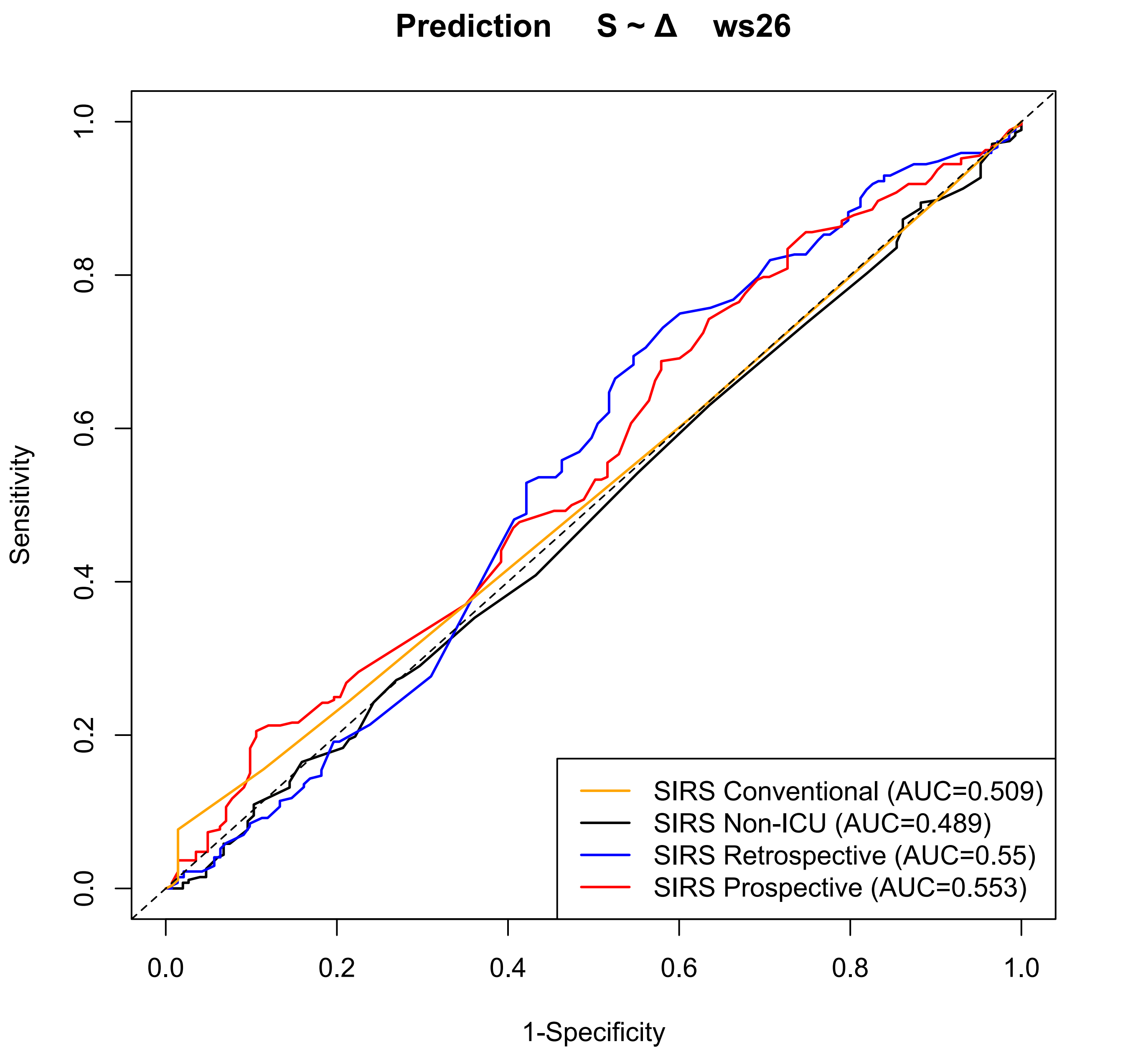

Prediction S ~ Δ ws26
Sensitivity
1-Specificity
0.0
0.2
0.4
0.6
0.8
1.0
0.0
0.2
0.4
0.6
0.8
1.0
SIRS Conventional (AUC=0.509)
SIRS Non-ICU (AUC=0.489)
SIRS Retrospective (AUC=0.55)
SIRS Prospective (AUC=0.553)

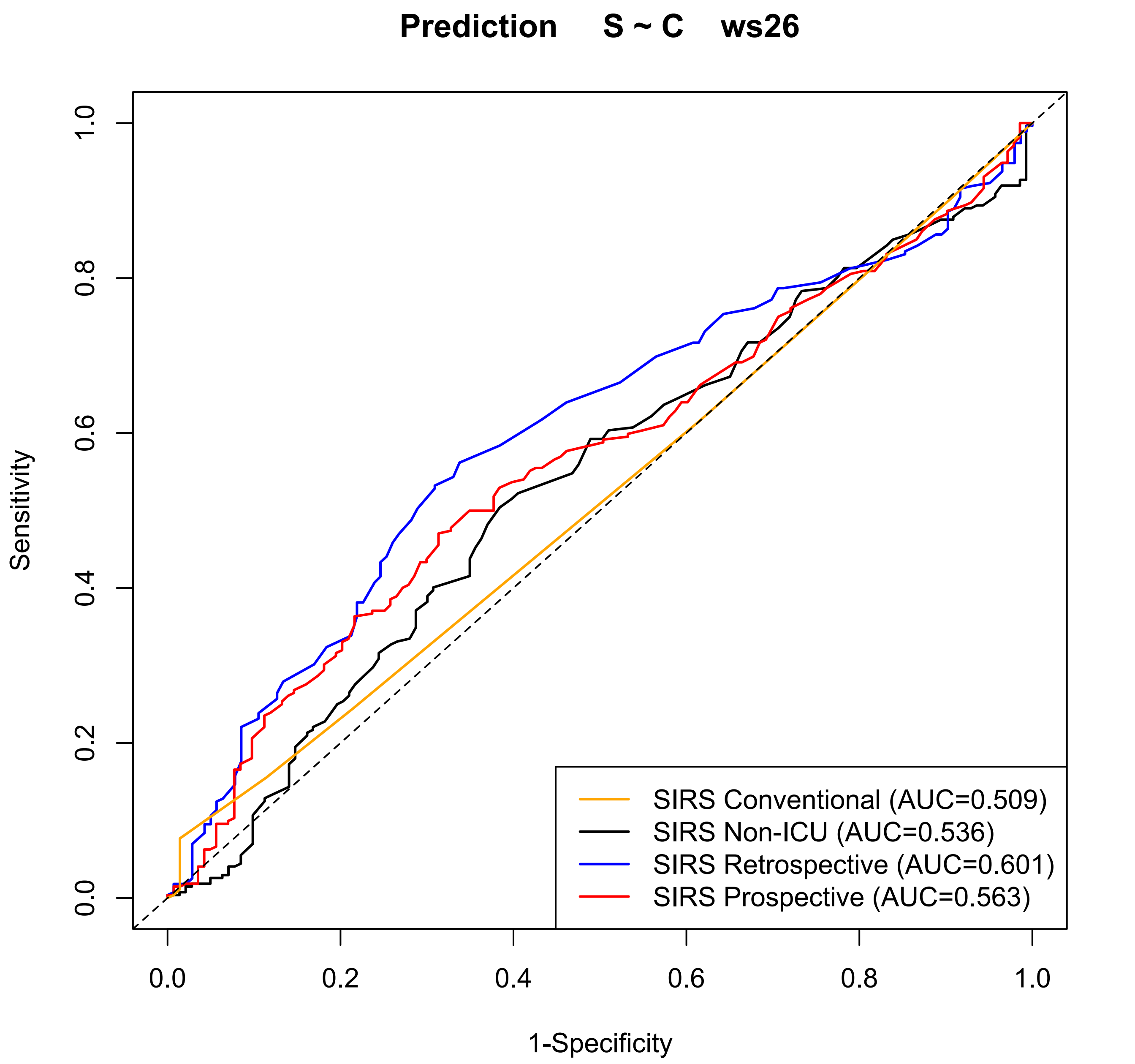

Prediction S ~ C ws26
Sensitivity
1-Specificity
0.0
0.2
0.4
0.6
0.8
1.0
SIRS Conventional (AUC=0.509)
SIRS Non-ICU (AUC=0.536)
SIRS Retrospective (AUC=0.601)
SIRS Prospective (AUC=0.563)

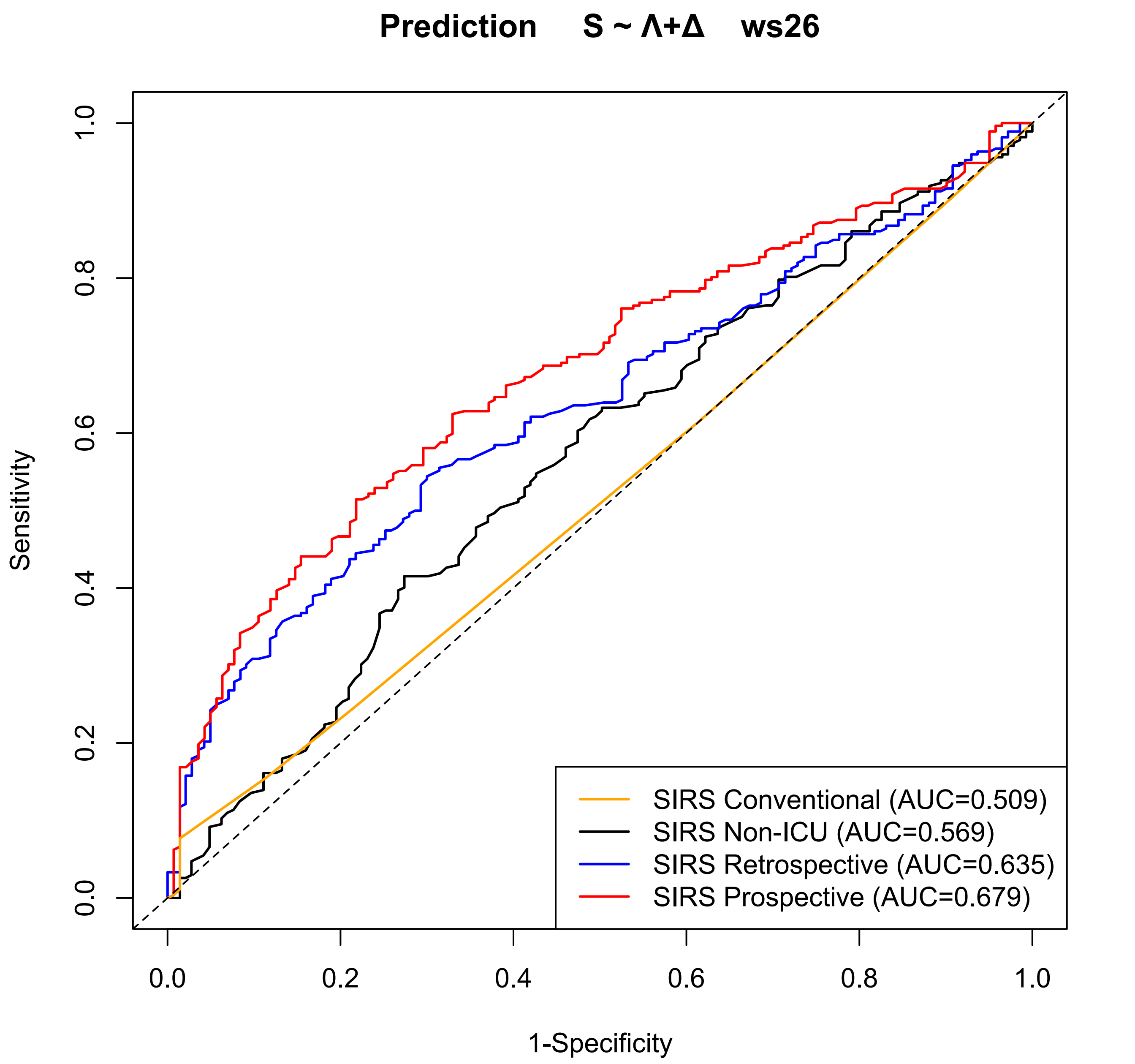

Prediction S ~ Λ+Δ ws26
Sensitivity
1-Specificity
0.0
0.2
0.4
0.6
0.8
1.0
SIRS Conventional (AUC=0.509)
SIRS Non-ICU (AUC=0.569)
SIRS Retrospective (AUC=0.635)
SIRS Prospective (AUC=0.679)

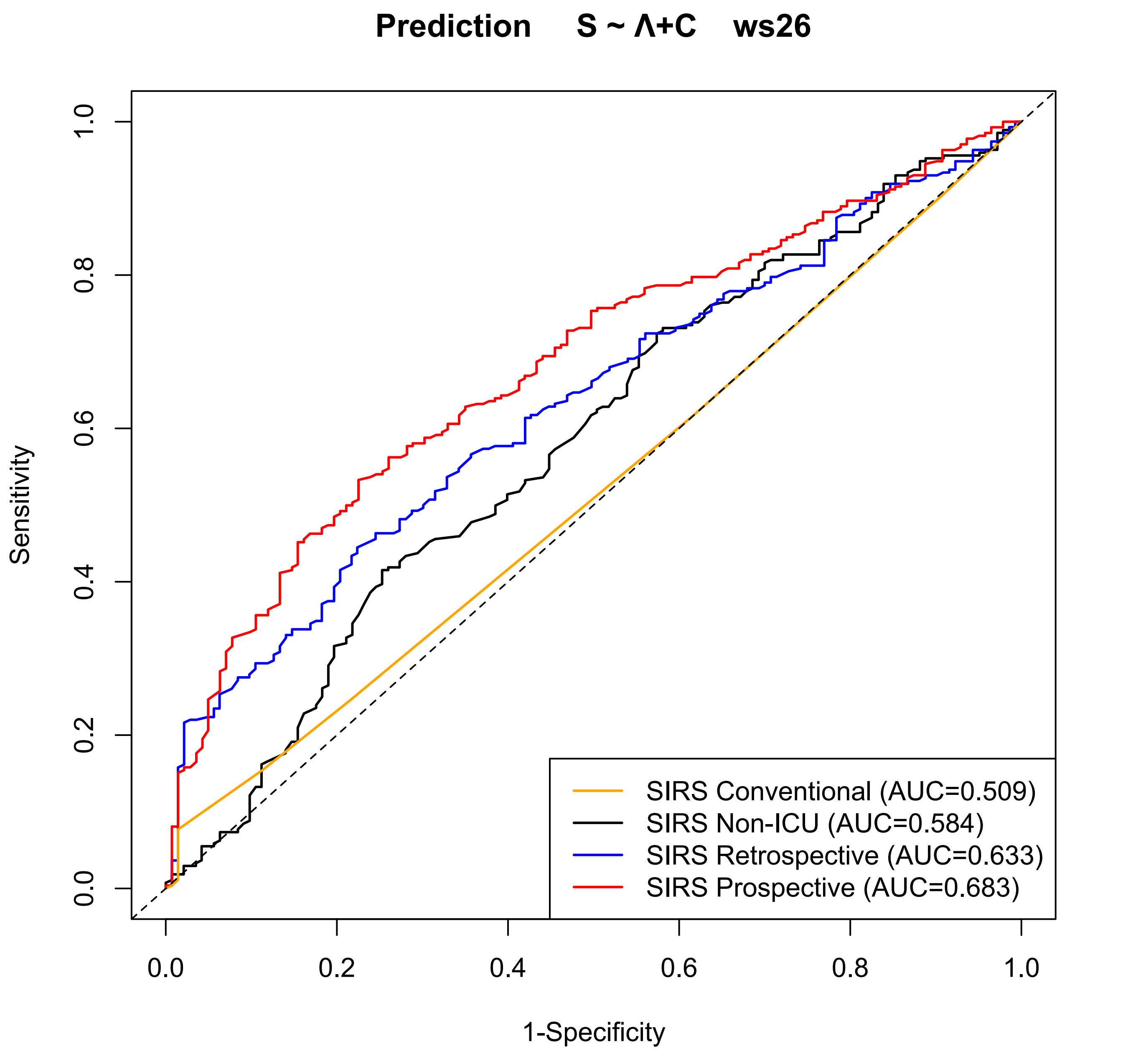

Prediction S ~ Λ+C ws26
Sensitivity
1-Specificity
0.0
0.2
0.4
0.6
0.8
1.0
0.0
0.2
0.4
0.6
0.8
1.0
SIRS Conventional (AUC=0.509)
SIRS Non-ICU (AUC=0.584)
SIRS Retrospective (AUC=0.633)
SIRS Prospective (AUC=0.683)

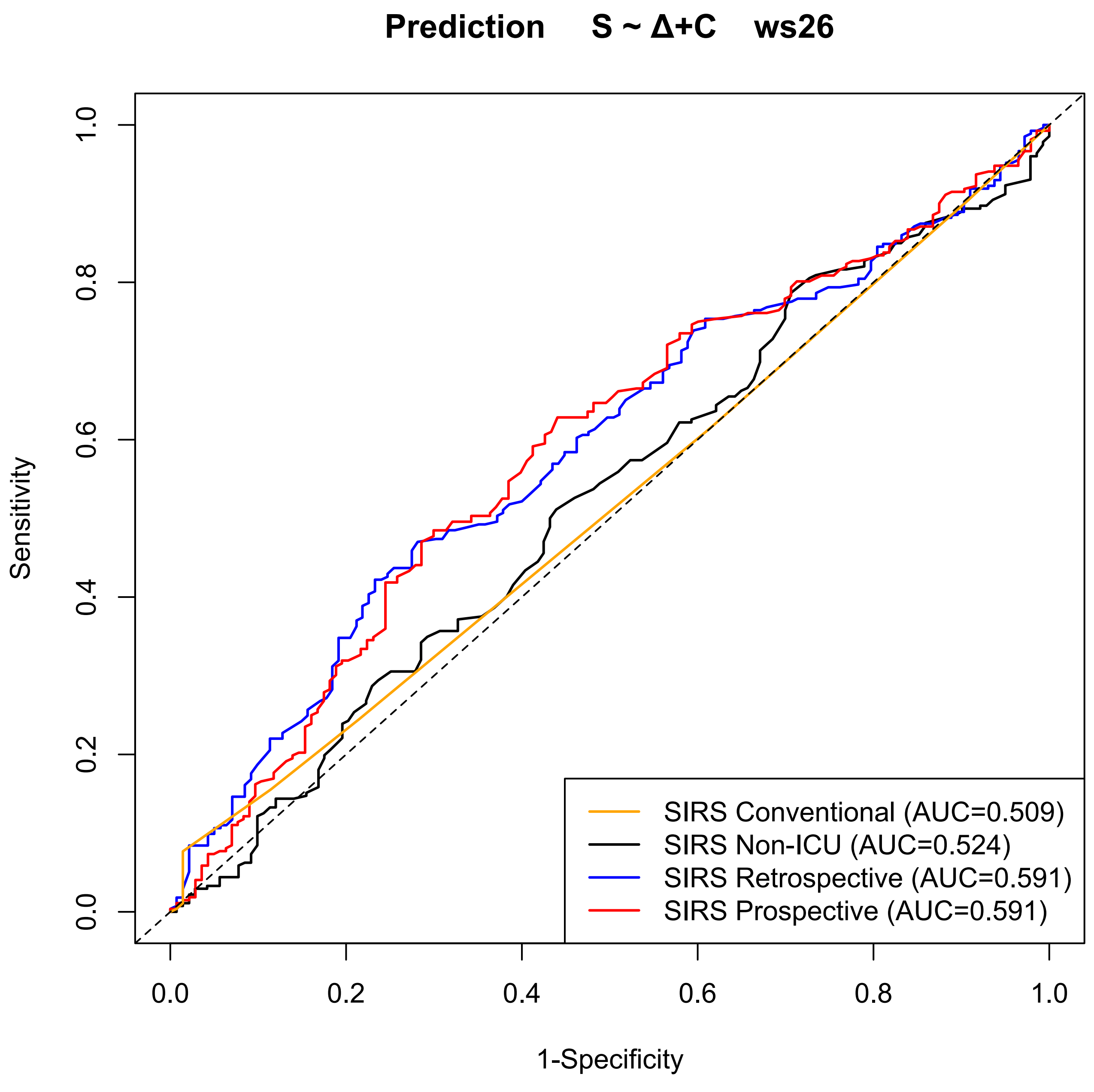

Prediction S ~ Δ+C ws26
Sensitivity
1-Specificity
0.0
0.2
0.4
0.6
0.8
1.0
SIRS Conventional (AUC=0.509)
SIRS Non-ICU (AUC=0.524)
SIRS Retrospective (AUC=0.591)
SIRS Prospective (AUC=0.591)

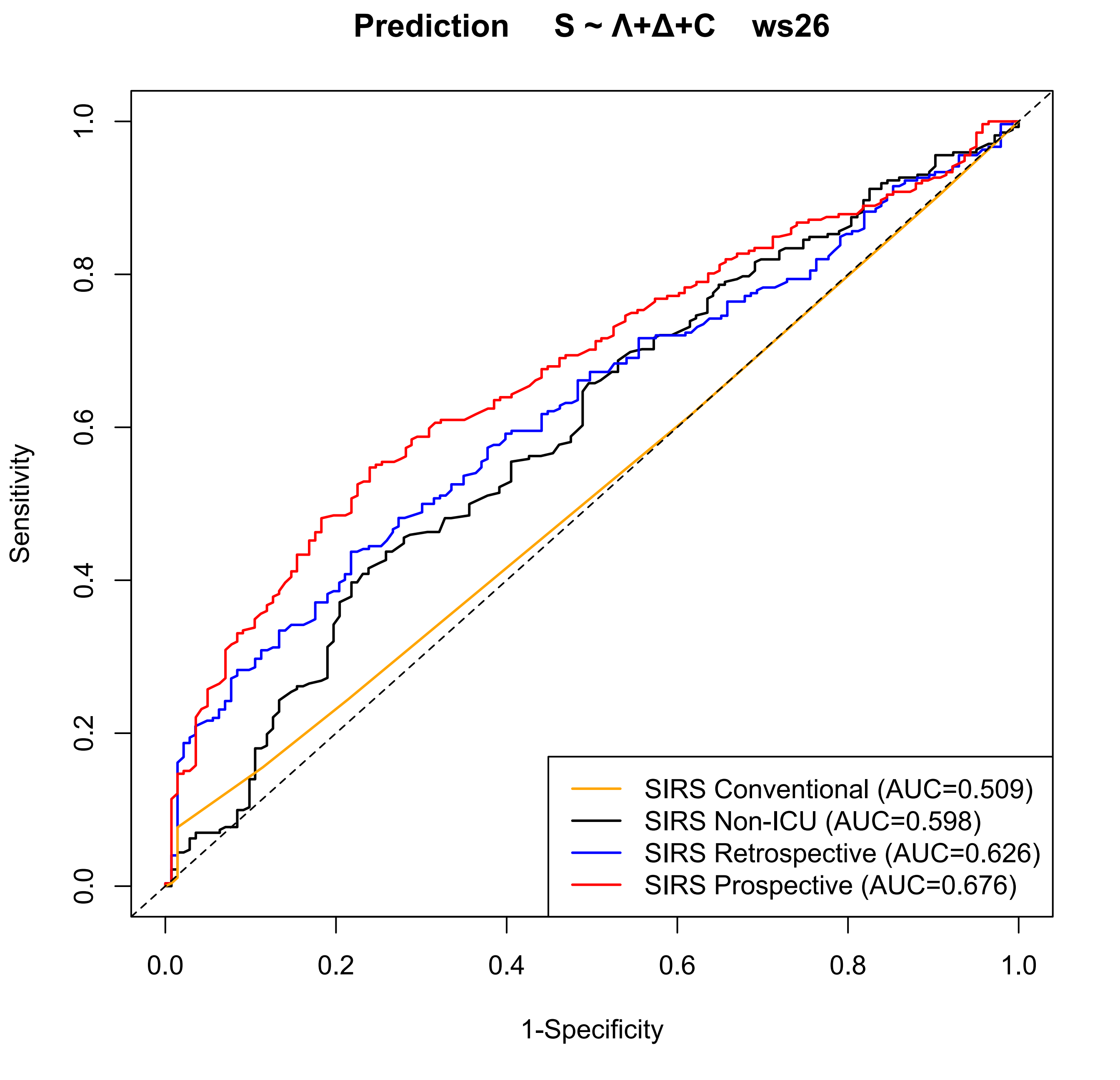

Prediction S ~ Λ+Δ+C ws26
Sensitivity
1-Specificity
0.0
0.2
0.4
0.6
0.8
1.0
SIRS Conventional (AUC=0.509)
SIRS Non-ICU (AUC=0.598)
SIRS Retrospective (AUC=0.626)
SIRS Prospective (AUC=0.676)

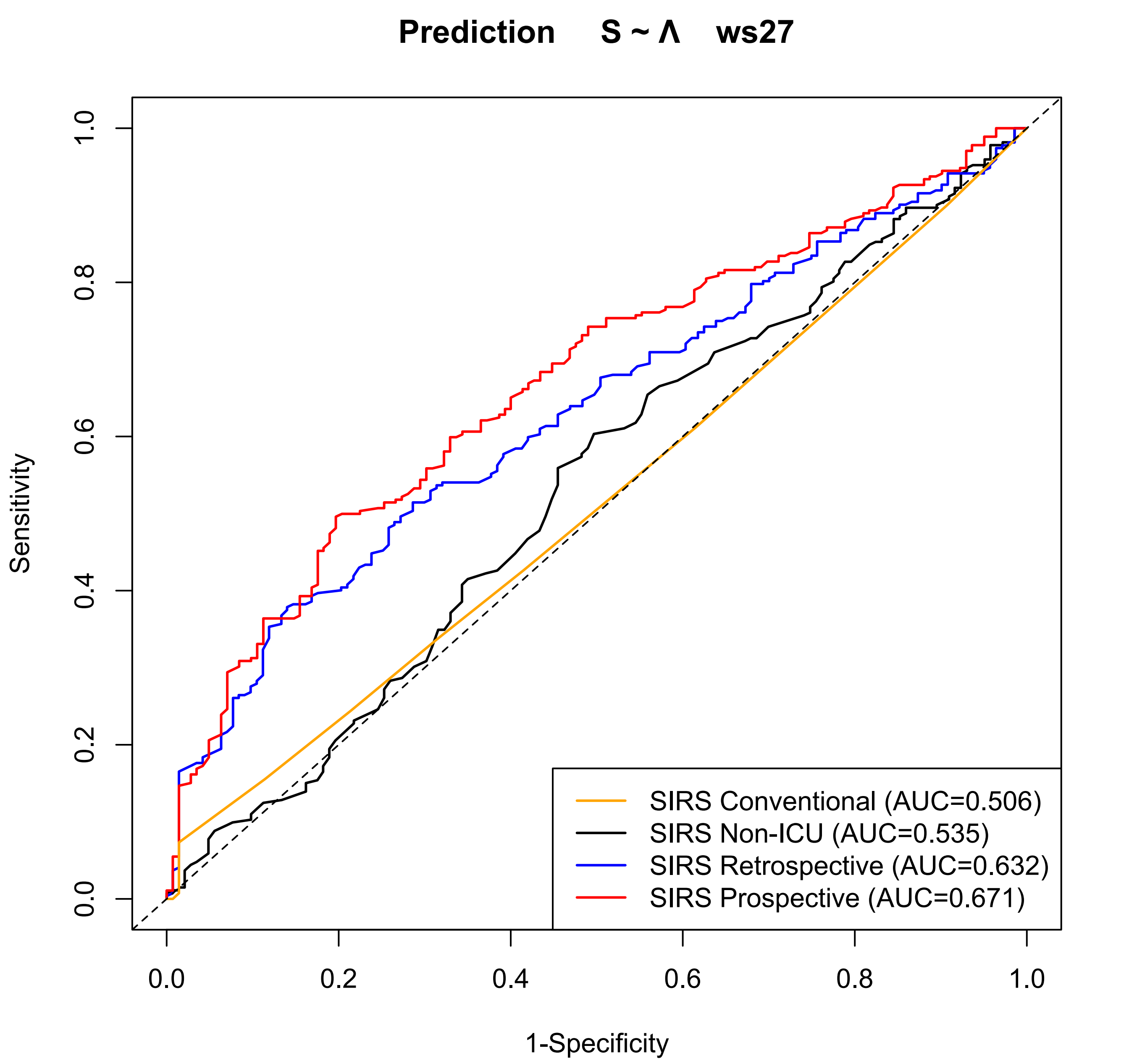

Prediction S ~ Λ ws27
Sensitivity
1-Specificity
0.0
0.2
0.4
0.6
0.8
1.0
0.0
0.2
0.4
0.6
0.8
1.0
SIRS Conventional (AUC=0.506)
SIRS Non-ICU (AUC=0.535)
SIRS Retrospective (AUC=0.632)
SIRS Prospective (AUC=0.671)

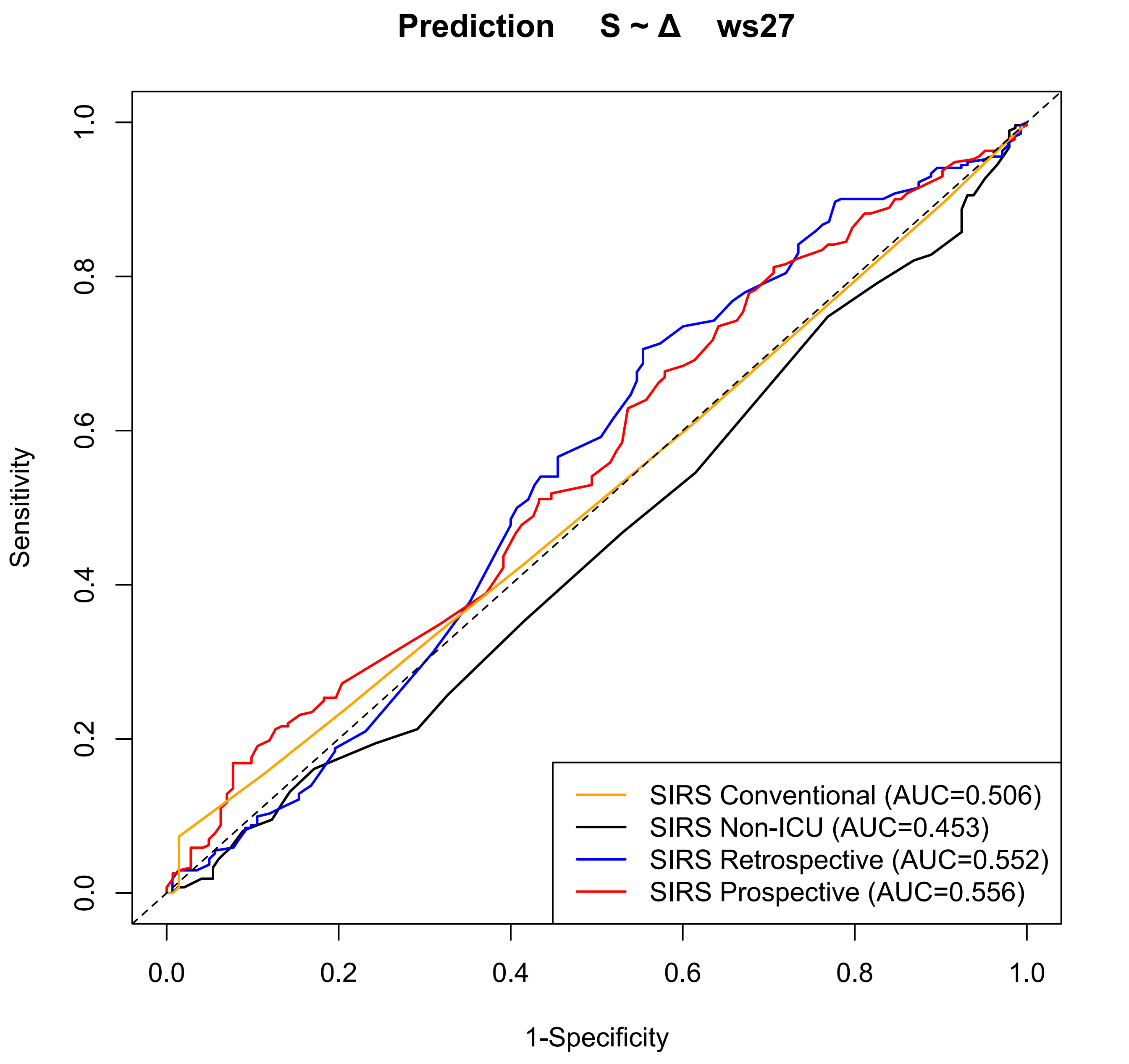

Prediction S ~ Δ ws27
Sensitivity
1-Specificity
0.0
0.2
0.4
0.6
0.8
1.0
SIRS Conventional (AUC=0.506)
SIRS Non-ICU (AUC=0.453)
SIRS Retrospective (AUC=0.552)
SIRS Prospective (AUC=0.556)

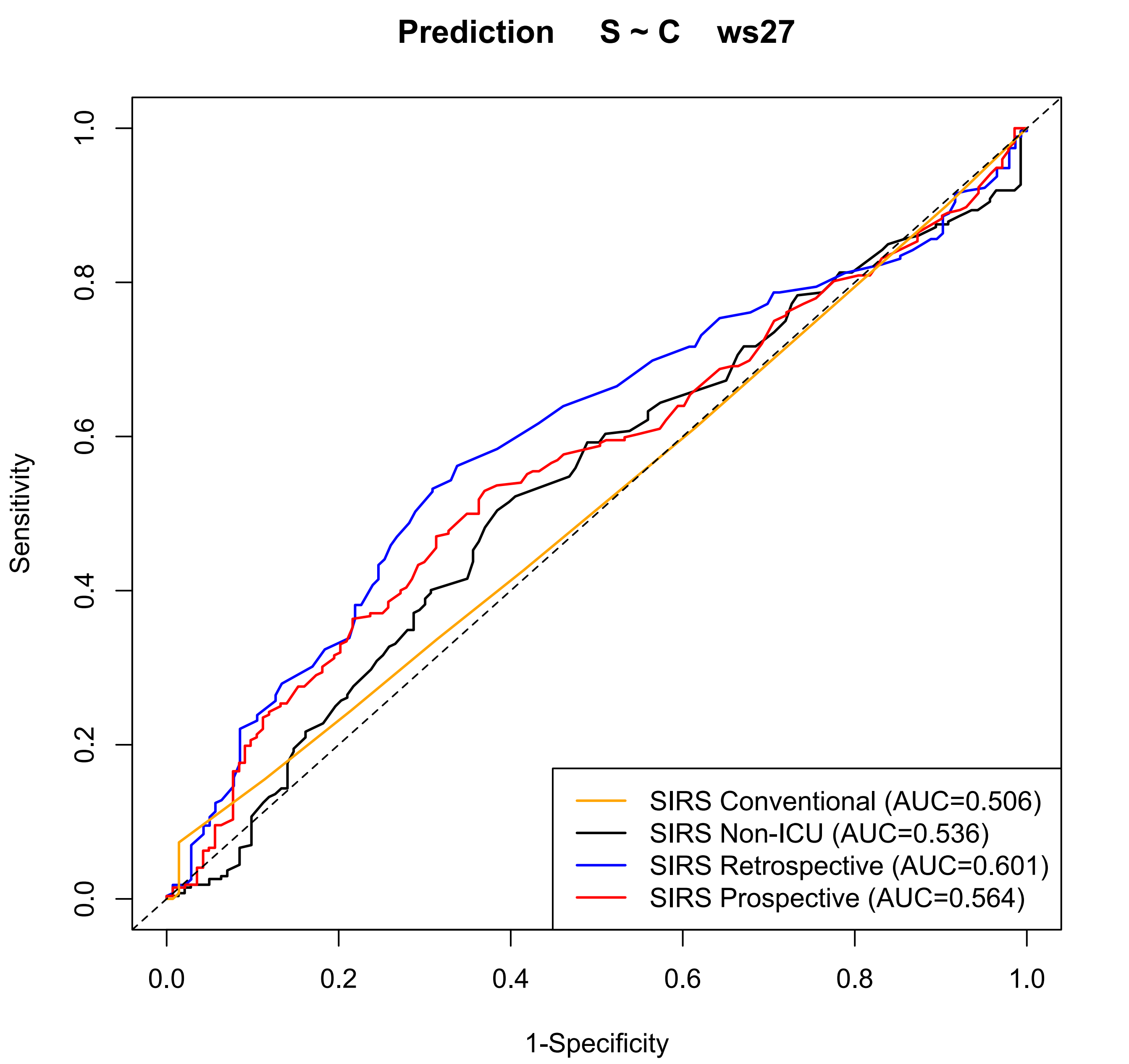

Prediction S ~ C ws27
Sensitivity
1-Specificity
0.0
0.2
0.4
0.6
0.8
1.0
0.0
0.2
0.4
0.6
0.8
1.0
SIRS Conventional (AUC=0.506)
SIRS Non-ICU (AUC=0.536)
SIRS Retrospective (AUC=0.601)
SIRS Prospective (AUC=0.564)

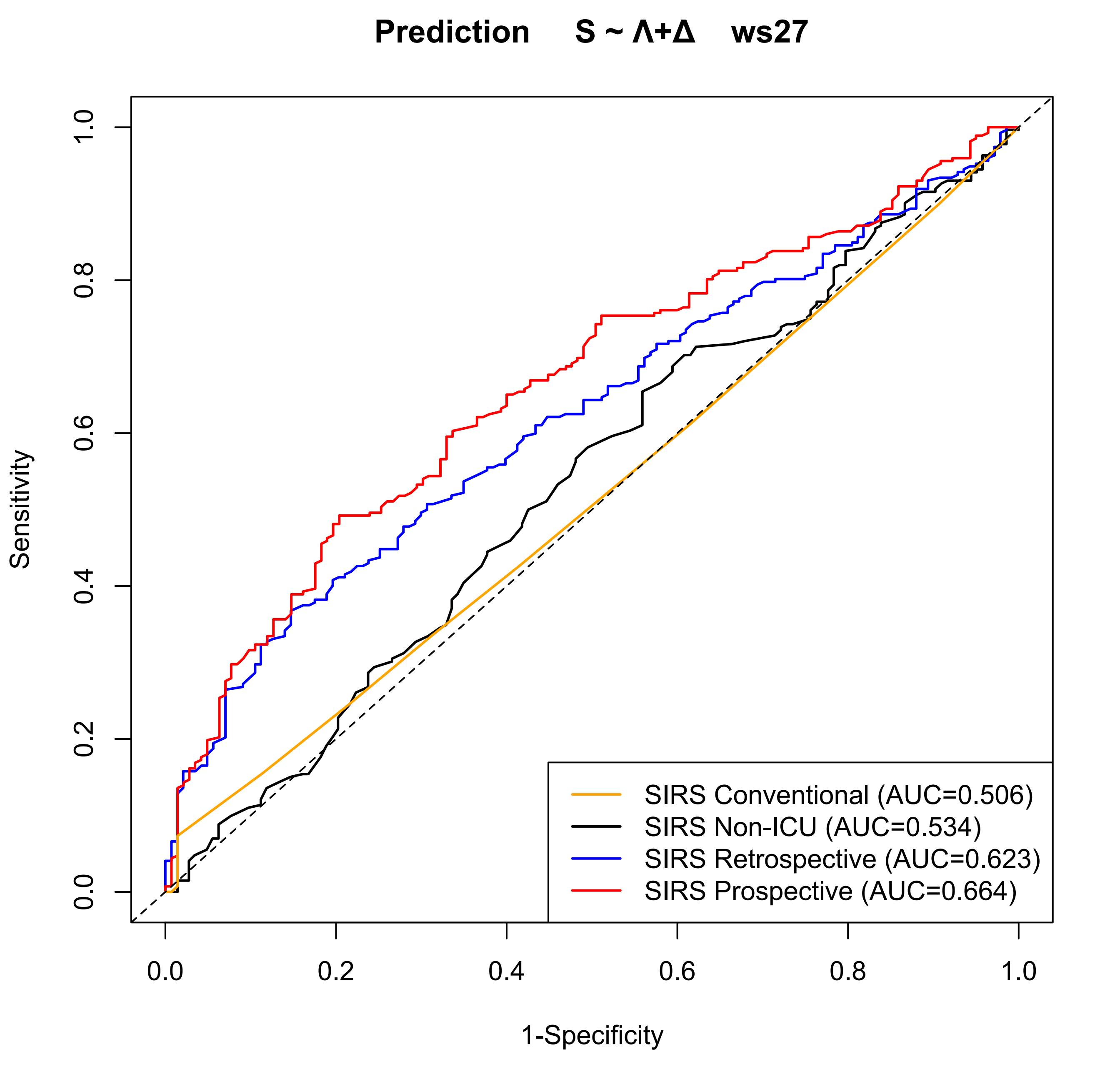

Prediction S ~ Λ+Δ ws27
Sensitivity
1-Specificity
0.0
0.2
0.4
0.6
0.8
1.0
SIRS Conventional (AUC=0.506)
SIRS Non-ICU (AUC=0.534)
SIRS Retrospective (AUC=0.623)
SIRS Prospective (AUC=0.664)

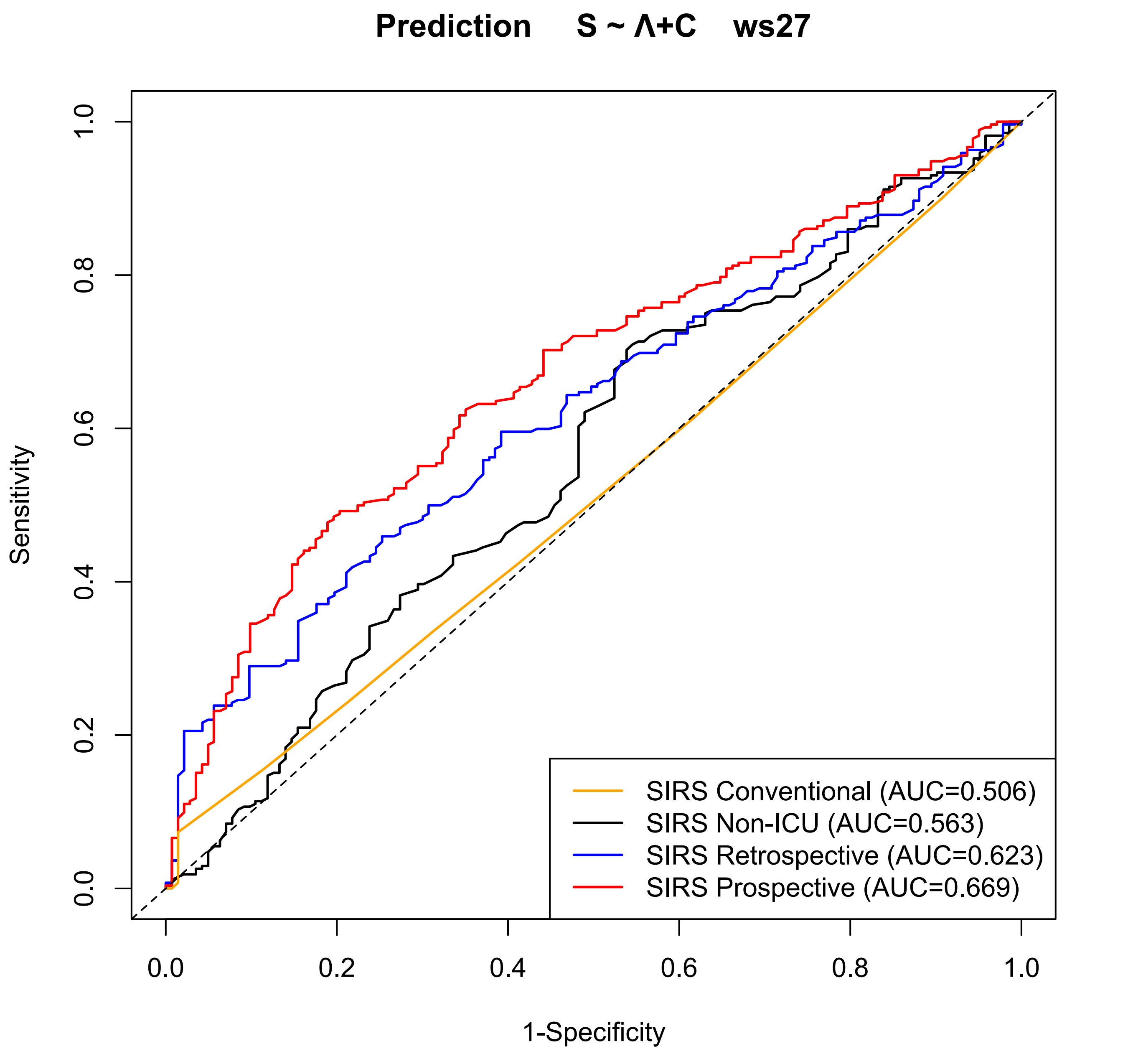

Prediction S ~ Λ+C ws27
Sensitivity
1-Specificity
0.0
0.2
0.4
0.6
0.8
1.0
SIRS Conventional (AUC=0.506)
SIRS Non-ICU (AUC=0.563)
SIRS Retrospective (AUC=0.623)
SIRS Prospective (AUC=0.669)

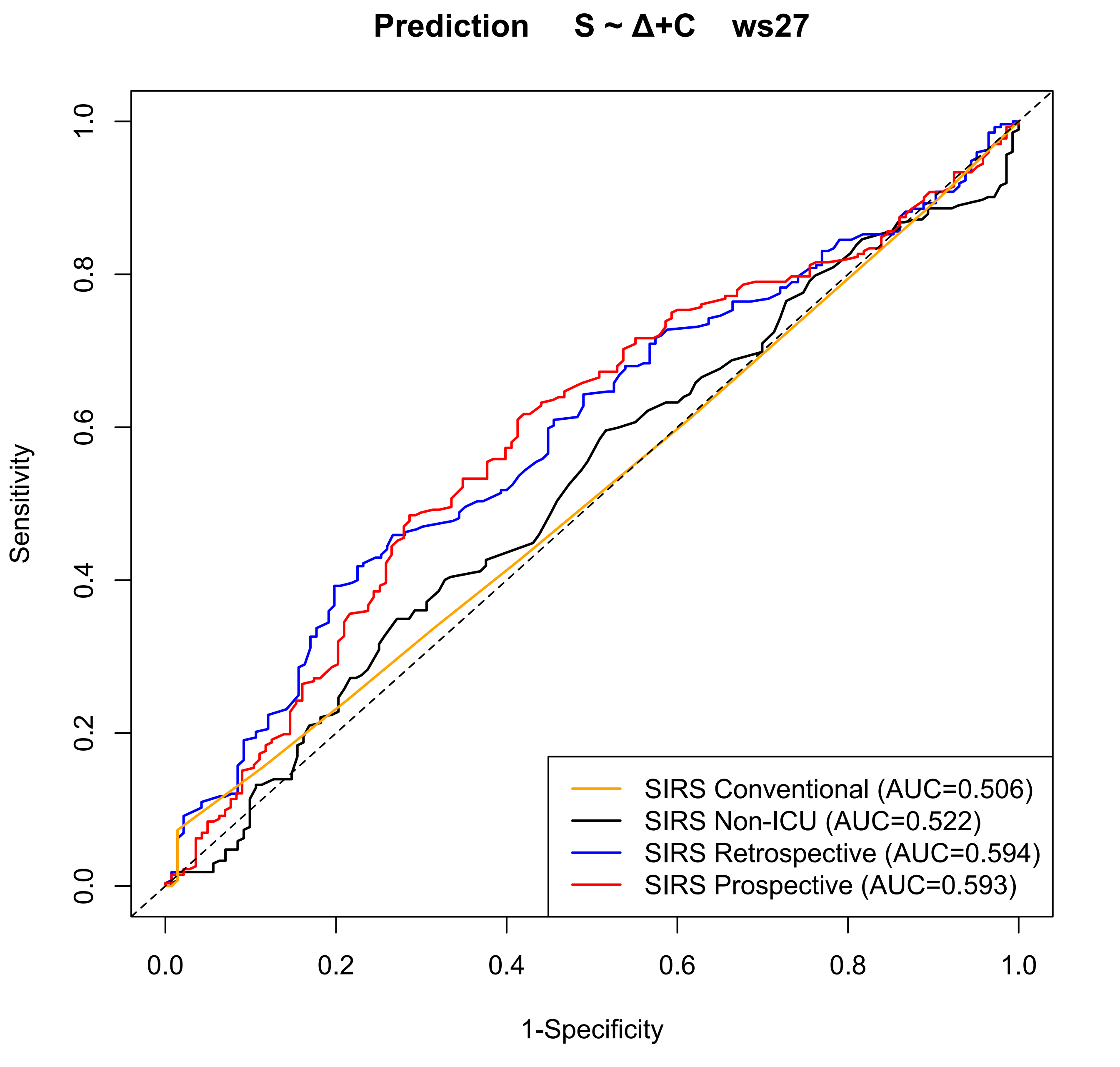

Prediction S ~ Δ+C ws27
Sensitivity
1-Specificity
0.0
0.2
0.4
0.6
0.8
1.0
0.0
0.2
0.4
0.6
0.8
1.0
SIRS Conventional (AUC=0.506)
SIRS Non-ICU (AUC=0.522)
SIRS Retrospective (AUC=0.594)
SIRS Prospective (AUC=0.593)

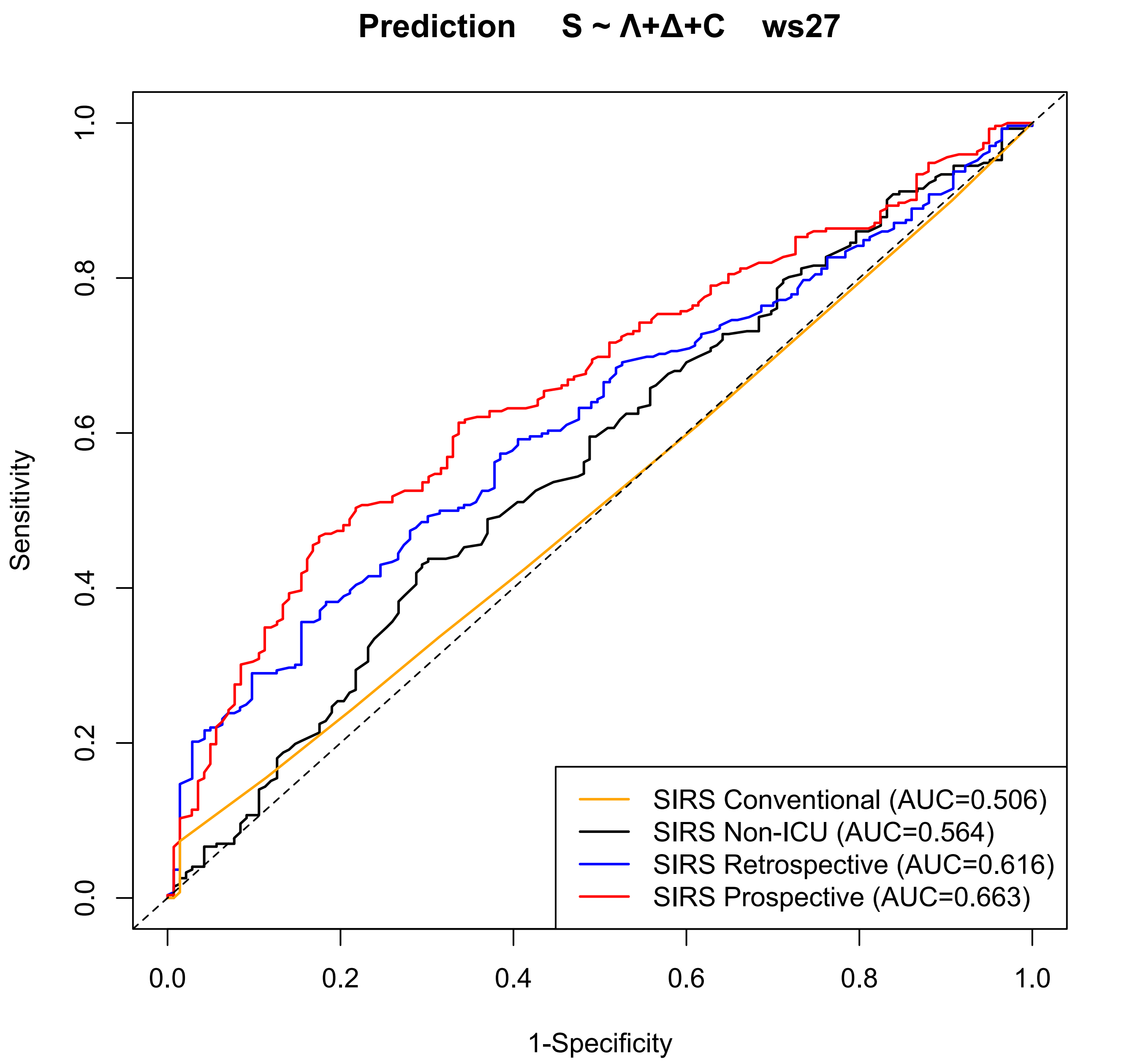

Prediction S ~ Λ+Δ+C ws27
Sensitivity
1-Specificity
0.0
0.2
0.4
0.6
0.8
1.0
SIRS Conventional (AUC=0.506)
SIRS Non-ICU (AUC=0.564)
SIRS Retrospective (AUC=0.616)
SIRS Prospective (AUC=0.663)

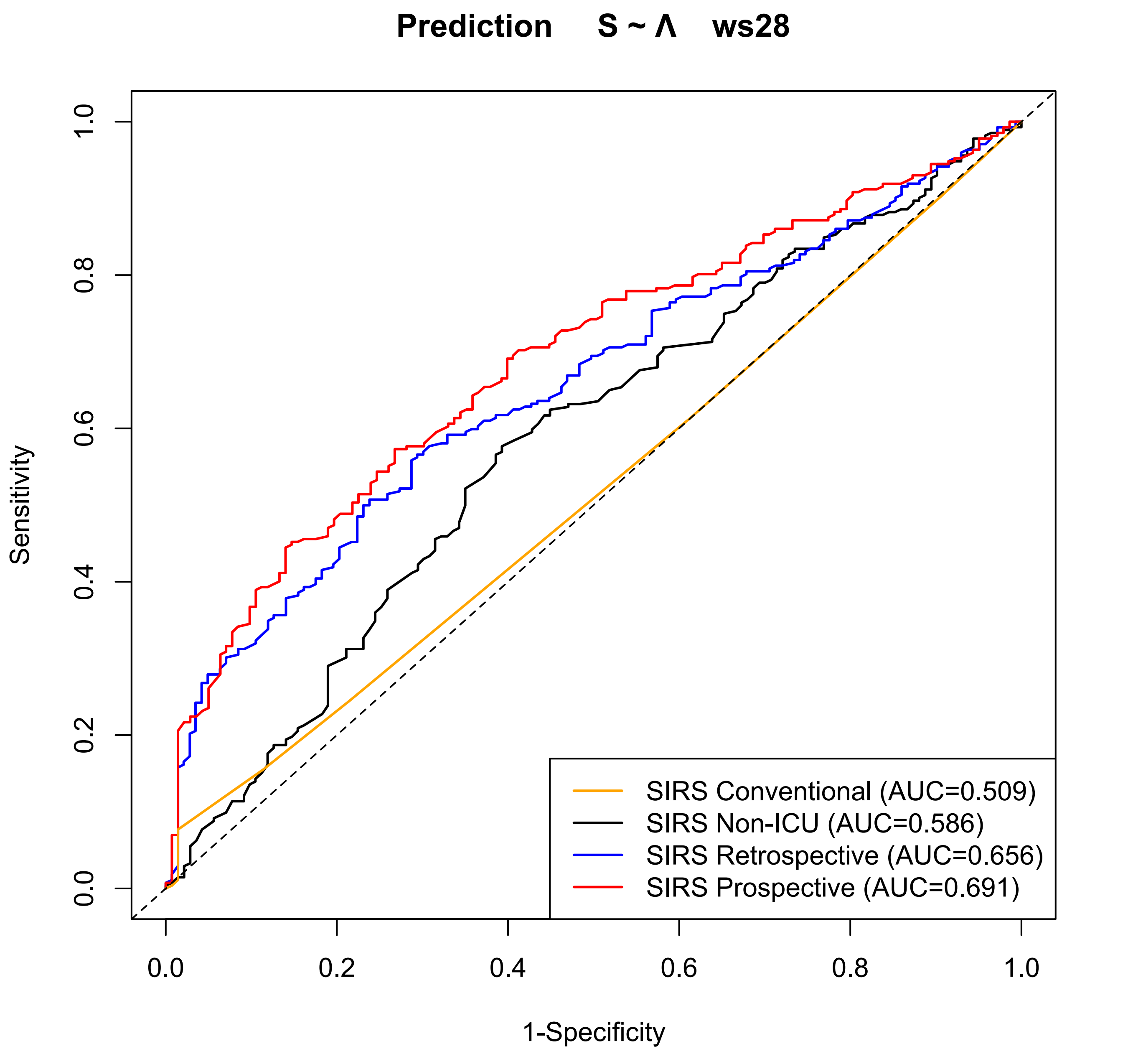
Prediction S ~ Λ ws28
Sensitivity
1-Specificity
0.0
0.2
0.4
0.6
0.8
1.0
SIRS Conventional (AUC=0.509)
SIRS Non-ICU (AUC=0.586)
SIRS Retrospective (AUC=0.656)
SIRS Prospective (AUC=0.691)

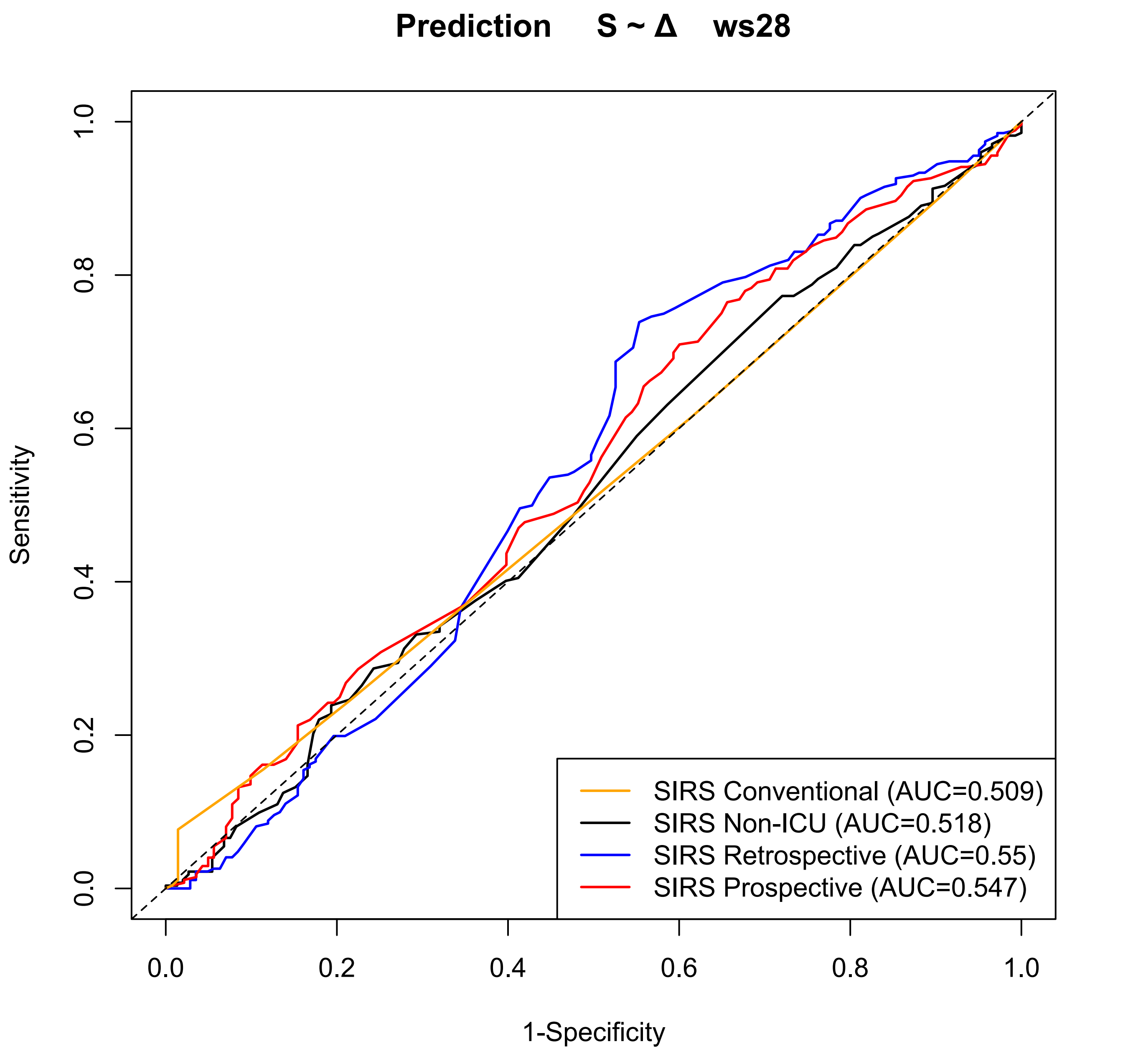

Prediction S ~ Δ ws28
Sensitivity
1-Specificity
0.0
0.2
0.4
0.6
0.8
1.0
SIRS Conventional (AUC=0.509)
SIRS Non-ICU (AUC=0.518)
SIRS Retrospective (AUC=0.55)
SIRS Prospective (AUC=0.547)

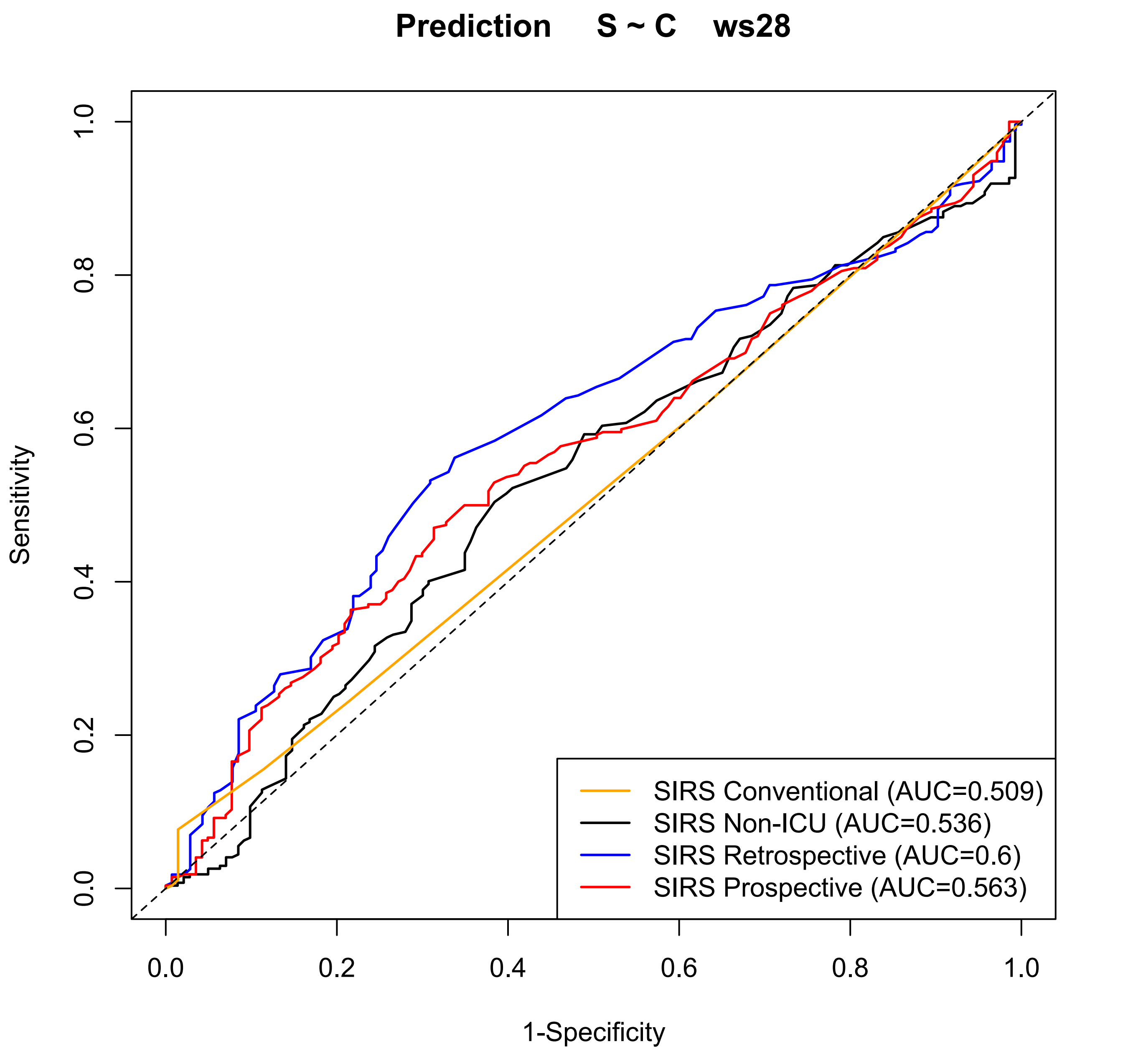

Prediction S ~ C ws28
Sensitivity
1-Specificity
0.0
0.2
0.4
0.6
0.8
1.0
SIRS Conventional (AUC=0.509)
SIRS Non-ICU (AUC=0.536)
SIRS Retrospective (AUC=0.6)
SIRS Prospective (AUC=0.563)

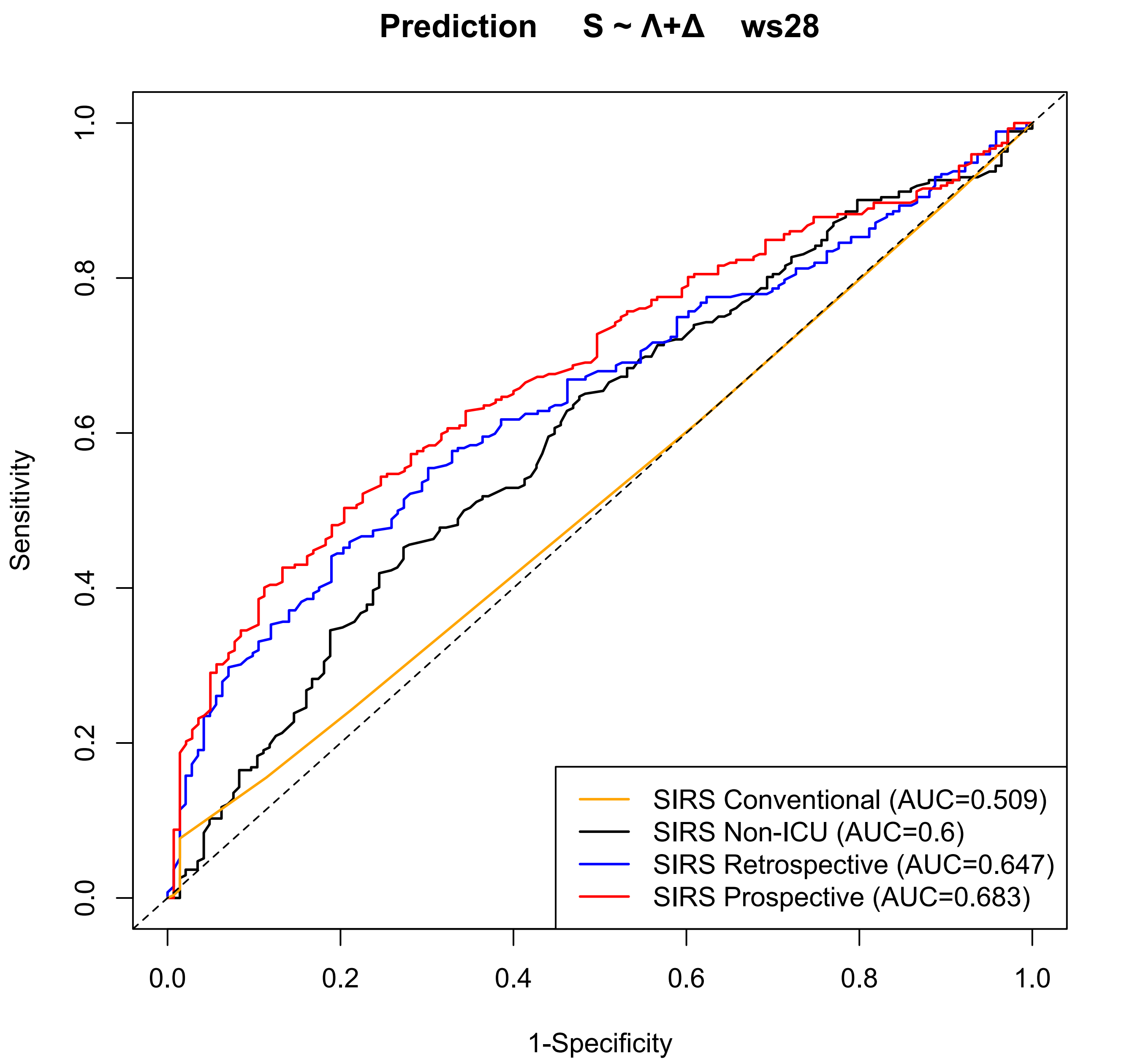

Prediction S ~ Λ+Δ ws28
Sensitivity
1-Specificity
0.0
0.2
0.4
0.6
0.8
1.0
SIRS Conventional (AUC=0.509)
SIRS Non-ICU (AUC=0.6)
SIRS Retrospective (AUC=0.647)
SIRS Prospective (AUC=0.683)

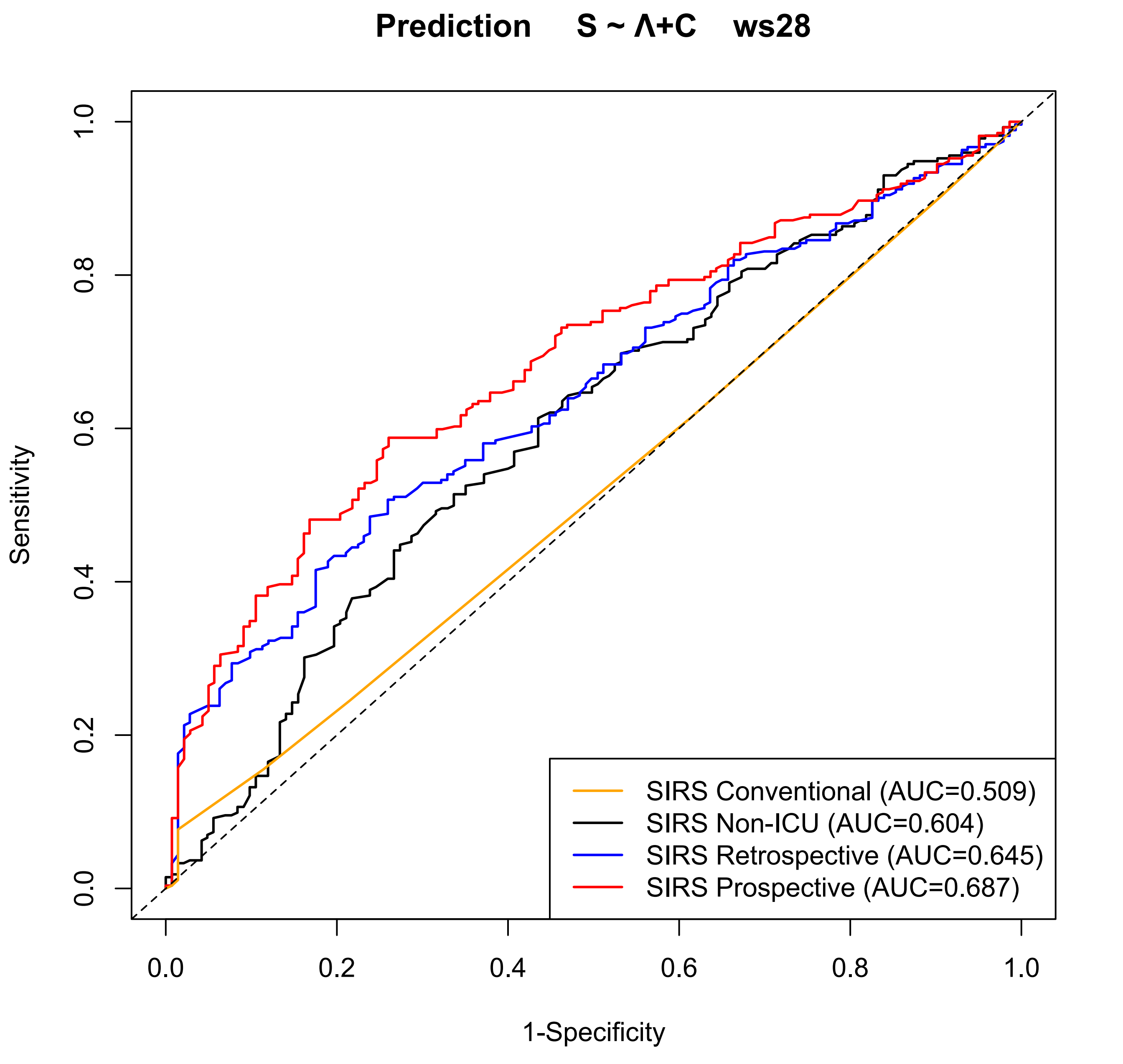

Prediction S ~ Λ+C ws28
Sensitivity
1-Specificity
0.0
0.2
0.4
0.6
0.8
1.0
0.0
0.2
0.4
0.6
0.8
1.0
SIRS Conventional (AUC=0.509)
SIRS Non-ICU (AUC=0.604)
SIRS Retrospective (AUC=0.645)
SIRS Prospective (AUC=0.687)

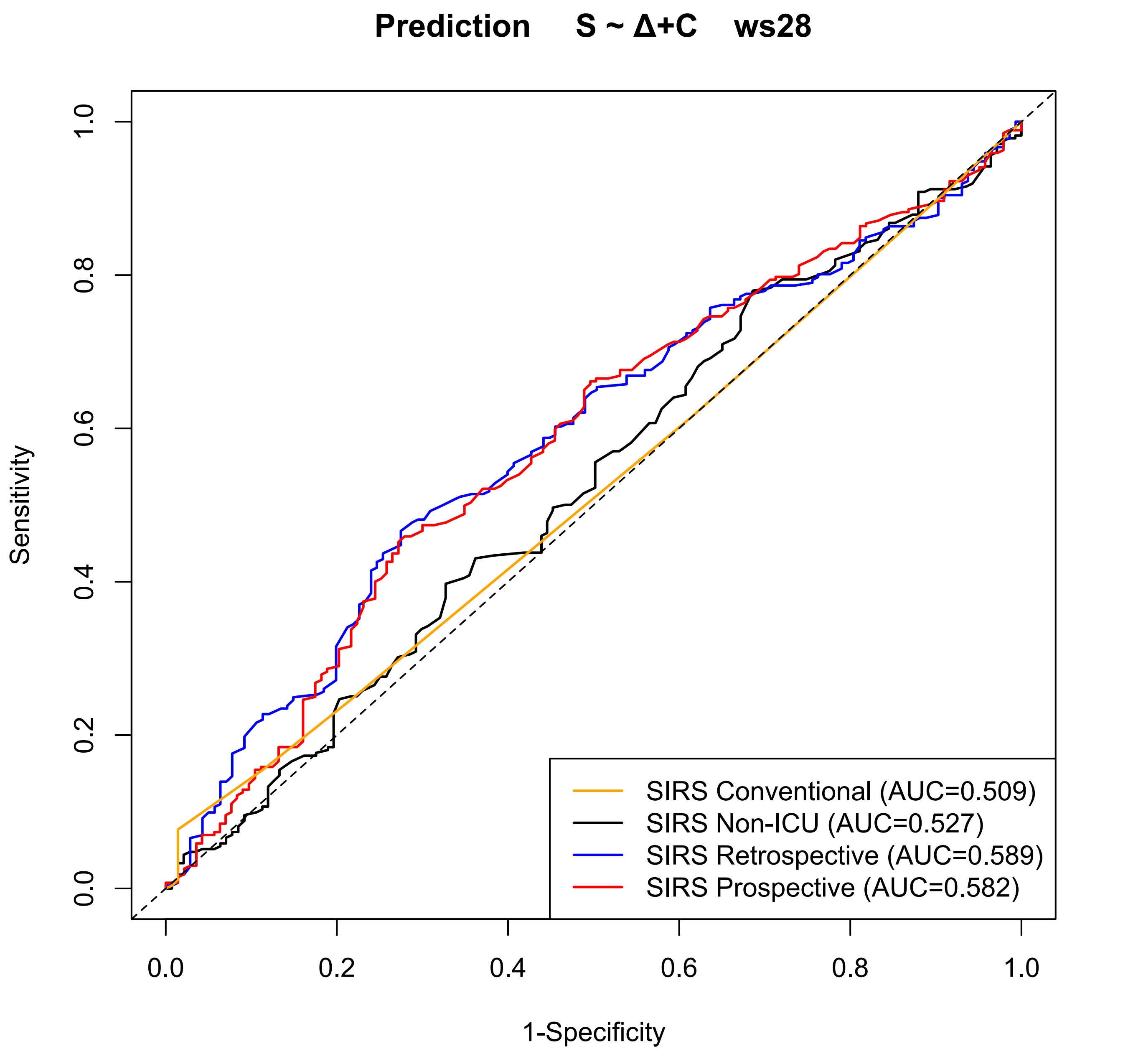

Prediction S ~ Δ+C ws28
Sensitivity
1-Specificity
0.0
0.2
0.4
0.6
0.8
1.0
SIRS Conventional (AUC=0.509)
SIRS Non-ICU (AUC=0.527)
SIRS Retrospective (AUC=0.589)
SIRS Prospective (AUC=0.582)

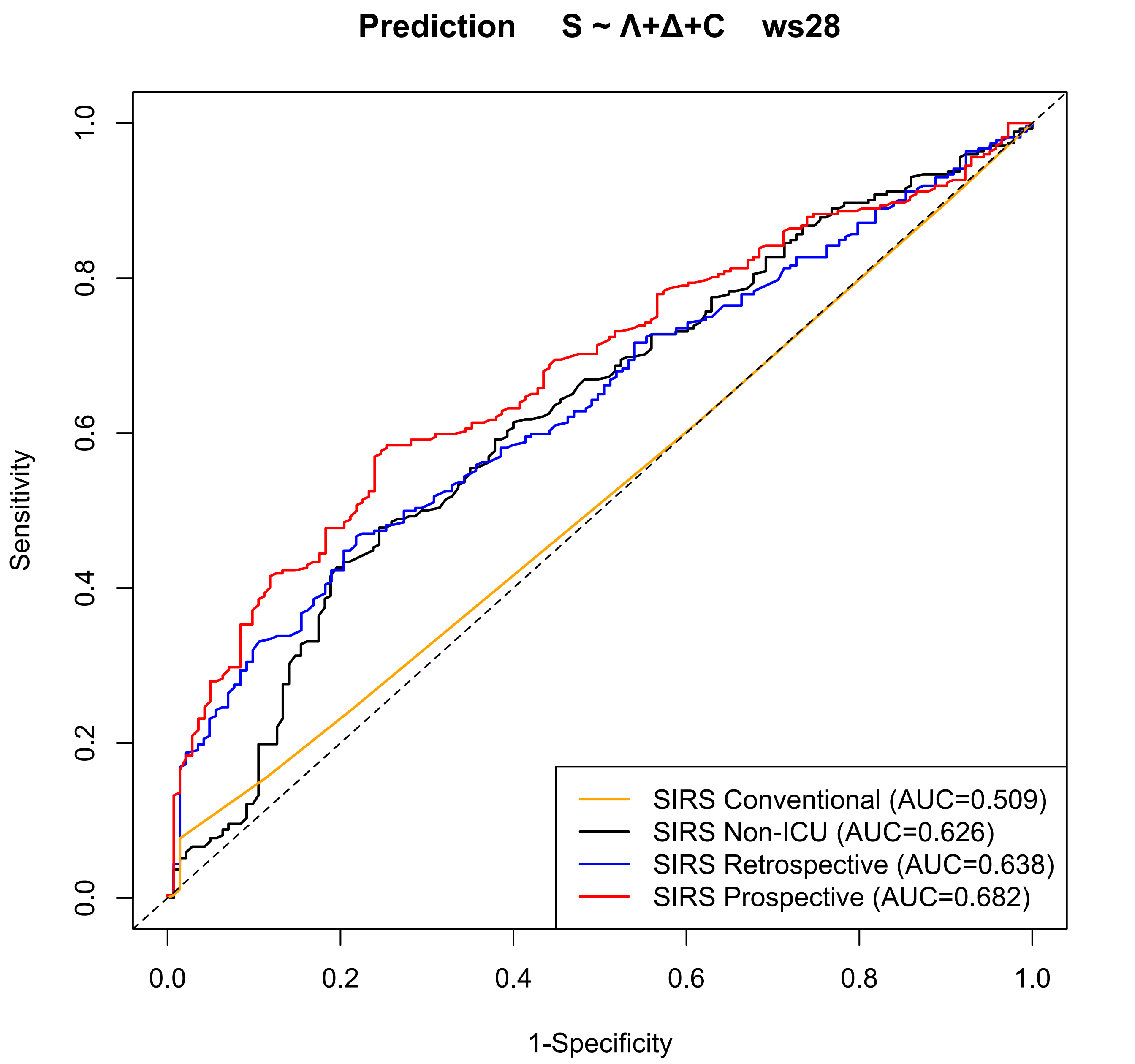

Prediction S ~ Λ+Δ+C ws28
Sensitivity
1-Specificity
0.0
0.2
0.4
0.6
0.8
1.0
0.0
0.2
0.4
0.6
0.8
1.0
SIRS Conventional (AUC=0.509)
SIRS Non-ICU (AUC=0.626)
SIRS Retrospective (AUC=0.638)
SIRS Prospective (AUC=0.682)

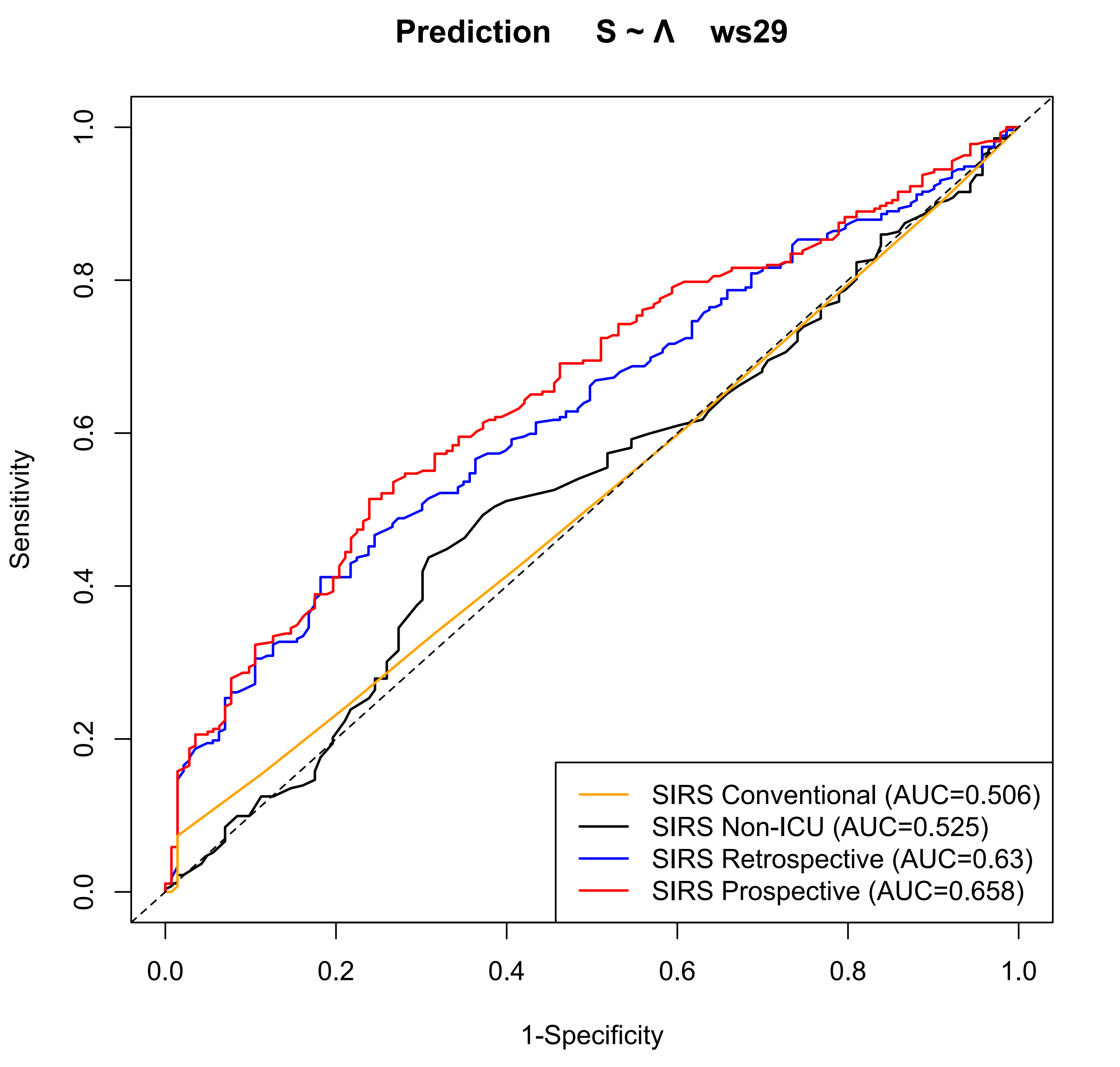
Prediction S ~ Λ ws29
Sensitivity
1-Specificity
0.0
0.2
0.4
0.6
0.8
1.0
SIRS Conventional (AUC=0.506)
SIRS Non-ICU (AUC=0.525)
SIRS Retrospective (AUC=0.63)
SIRS Prospective (AUC=0.658)

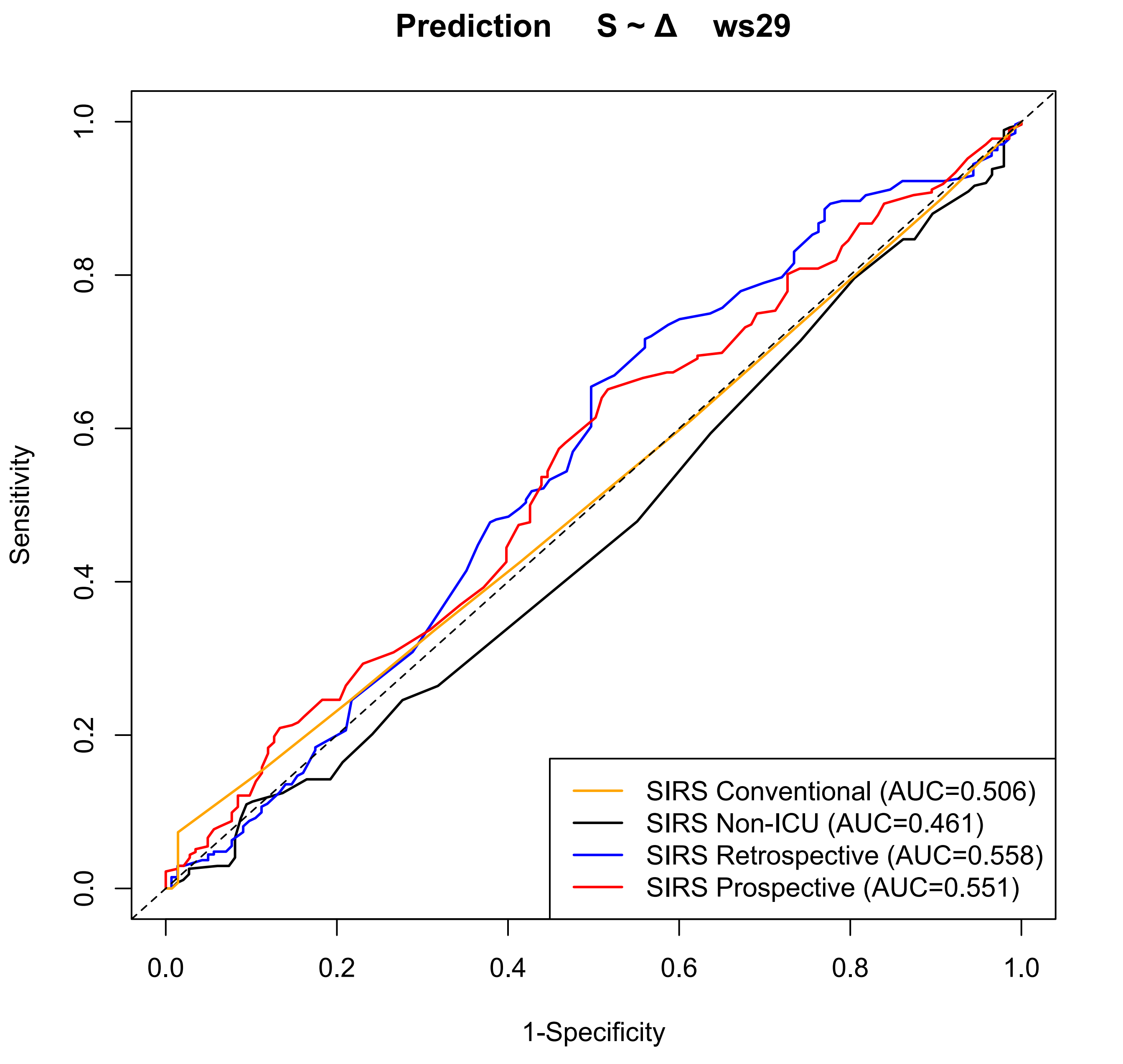
Prediction S ~ Δ ws29
Sensitivity
1-Specificity
0.0
0.2
0.4
0.6
0.8
1.0
SIRS Conventional (AUC=0.506)
SIRS Non-ICU (AUC=0.461)
SIRS Retrospective (AUC=0.558)
SIRS Prospective (AUC=0.551)

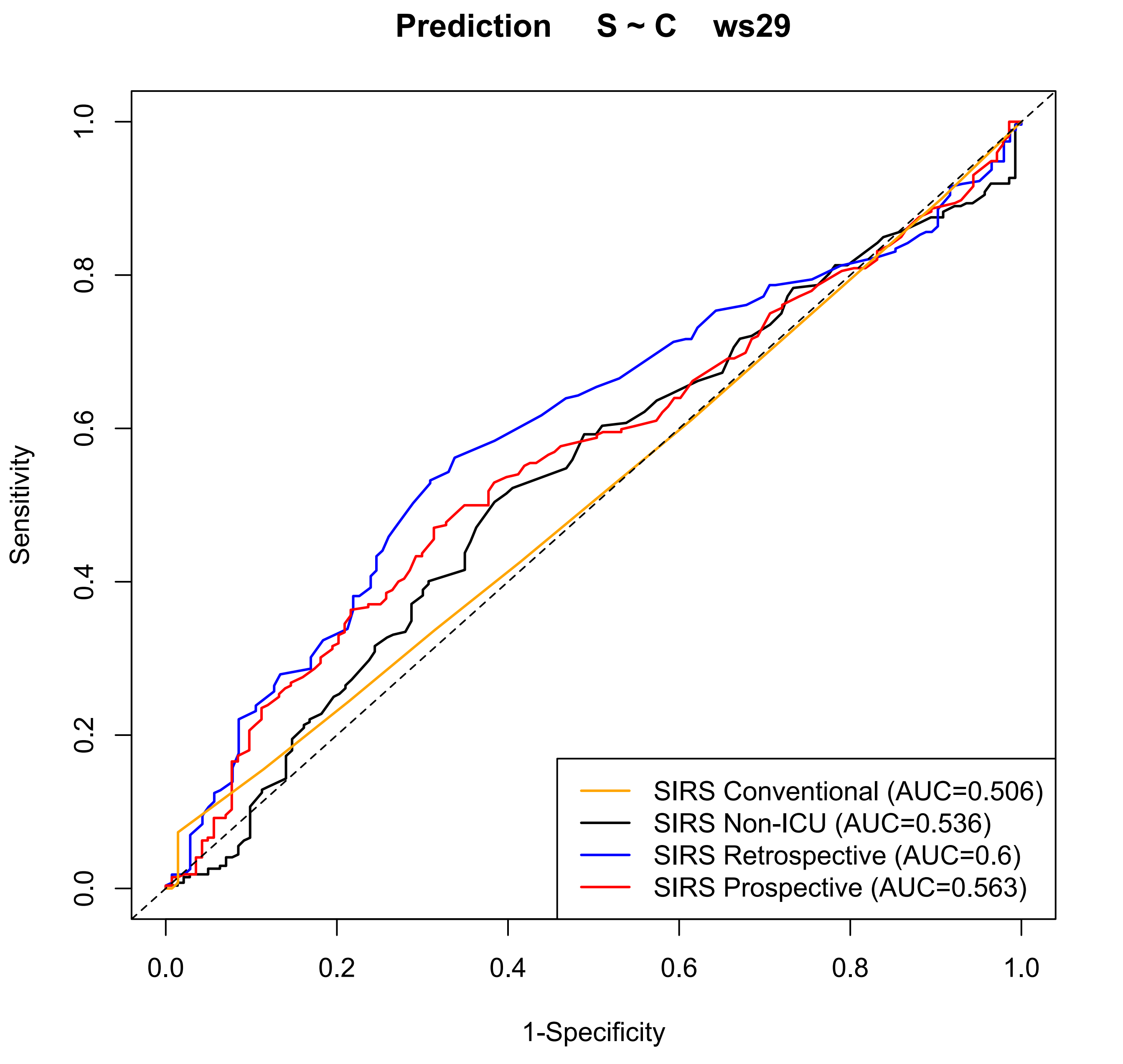

Prediction S ~ C ws29
Sensitivity
1-Specificity
0.0
0.2
0.4
0.6
0.8
1.0
SIRS Conventional (AUC=0.506)
SIRS Non-ICU (AUC=0.536)
SIRS Retrospective (AUC=0.6)
SIRS Prospective (AUC=0.563)

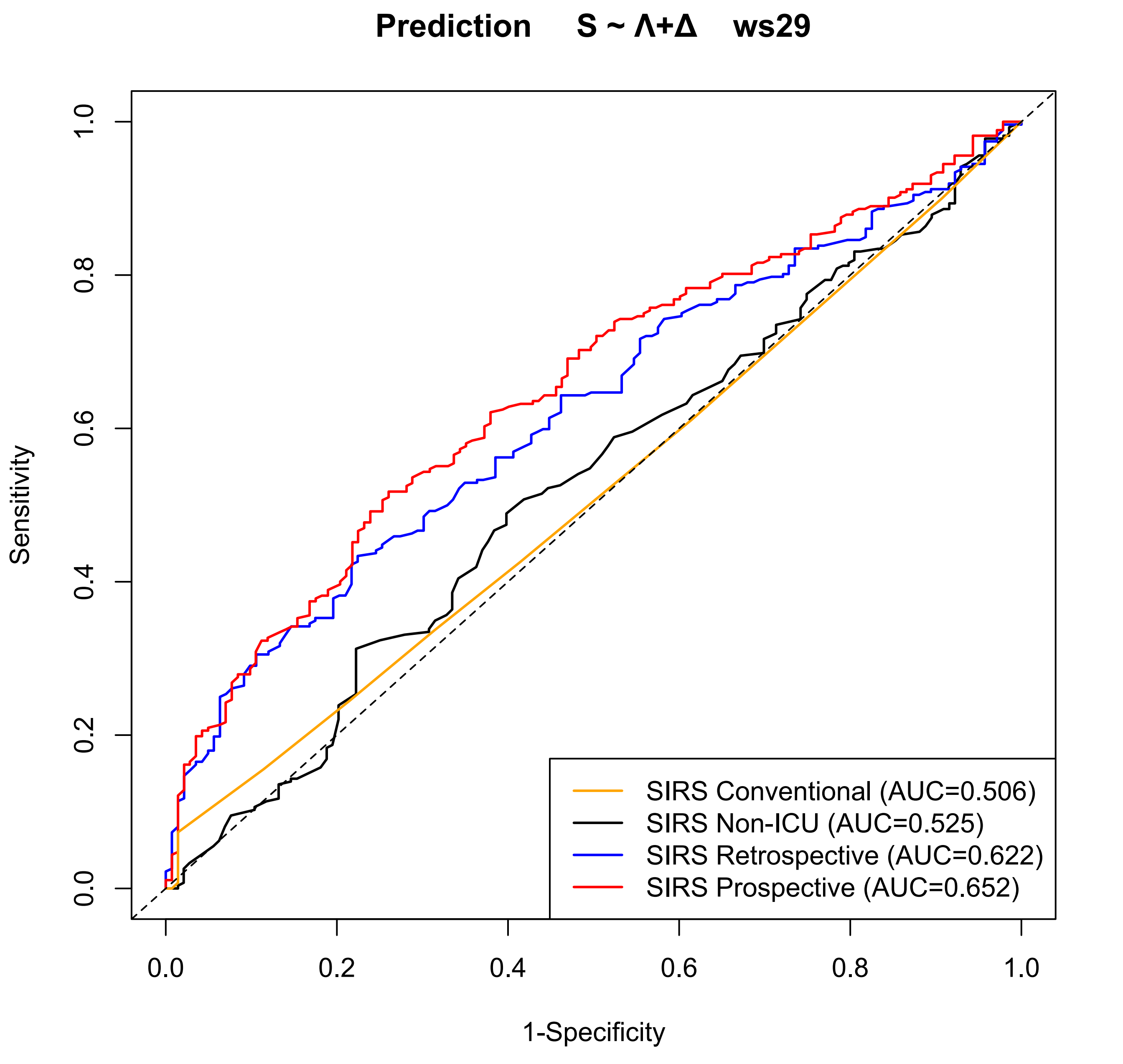

Prediction S ~ Λ+Δ ws29
Sensitivity
1-Specificity
0.0
0.2
0.4
0.6
0.8
1.0
SIRS Conventional (AUC=0.506)
SIRS Non-ICU (AUC=0.525)
SIRS Retrospective (AUC=0.622)
SIRS Prospective (AUC=0.652)

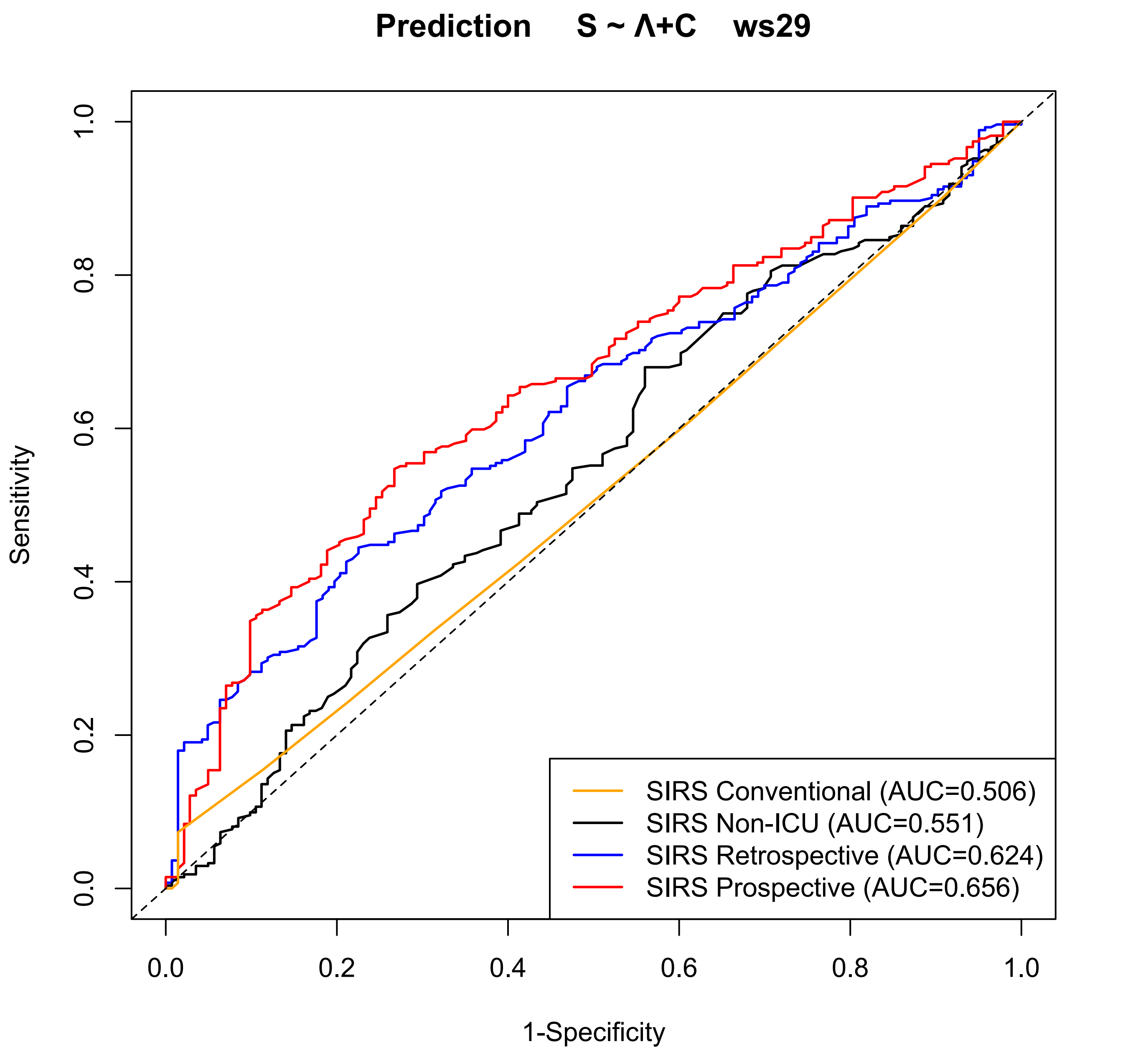

Prediction S ~ Λ+C ws29
Sensitivity
1-Specificity
0.0
0.2
0.4
0.6
0.8
1.0
0.0
0.2
0.4
0.6
0.8
1.0
SIRS Conventional (AUC=0.506)
SIRS Non-ICU (AUC=0.551)
SIRS Retrospective (AUC=0.624)
SIRS Prospective (AUC=0.656)

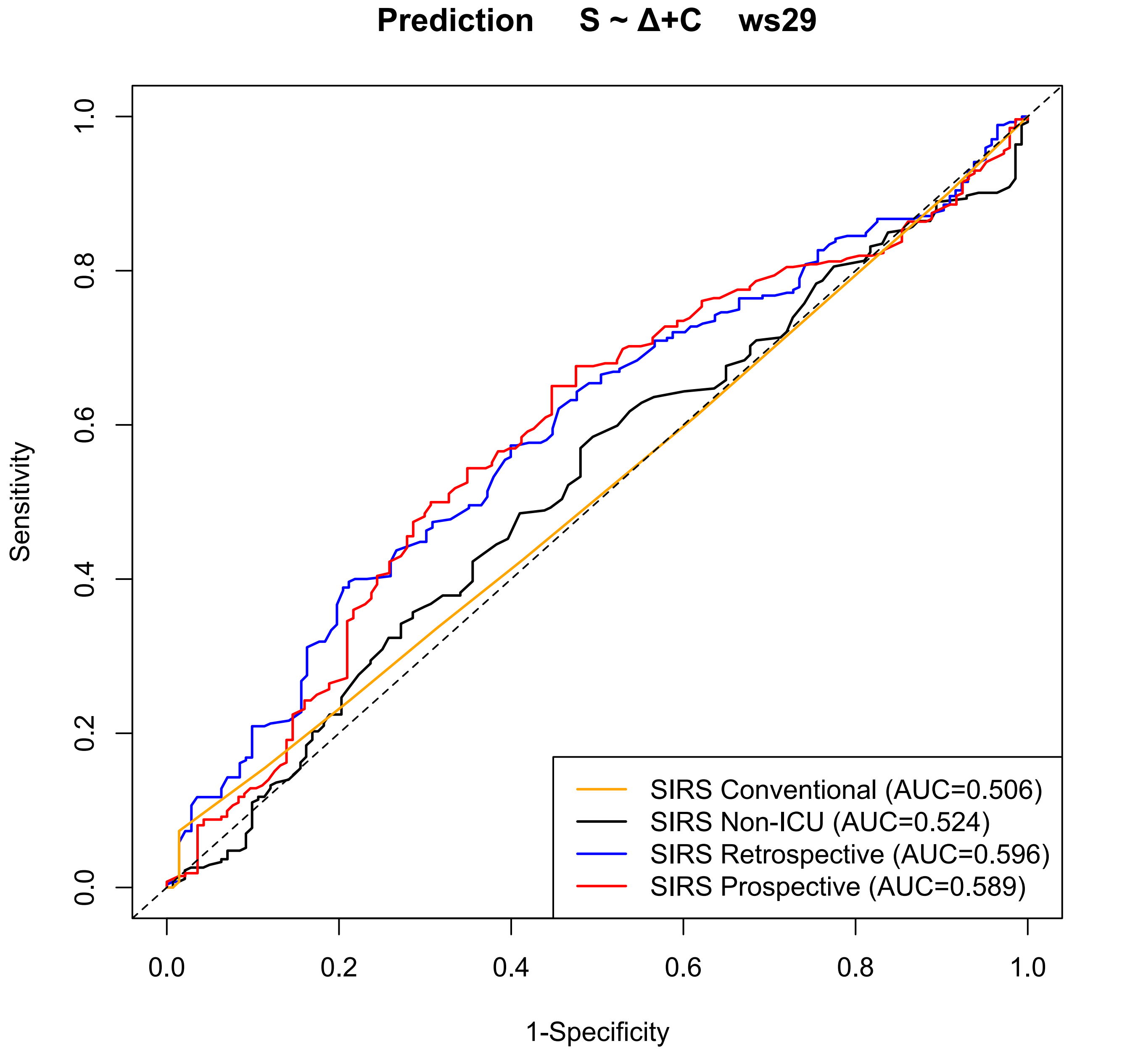
Prediction S ~ Δ+C ws29
Sensitivity
1-Specificity
0.0
0.2
0.4
0.6
0.8
1.0
0.0
0.2
0.4
0.6
0.8
1.0
SIRS Conventional (AUC=0.506)
SIRS Non-ICU (AUC=0.524)
SIRS Retrospective (AUC=0.596)
SIRS Prospective (AUC=0.589)

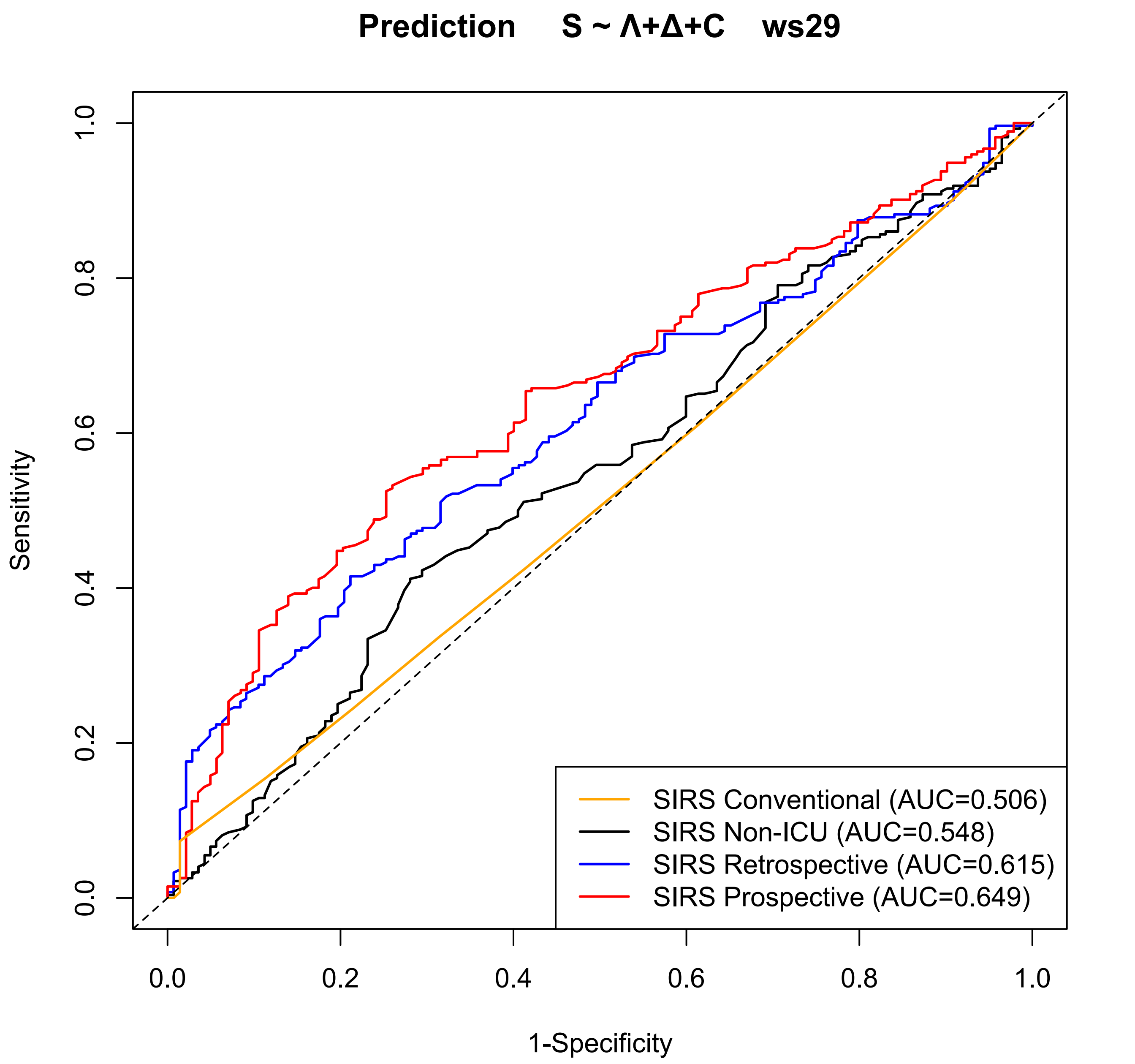

Prediction S ~ Λ+Δ+C ws29
Sensitivity
1-Specificity
0.0
0.2
0.4
0.6
0.8
1.0
0.0
0.2
0.4
0.6
0.8
1.0
SIRS Conventional (AUC=0.506)
SIRS Non-ICU (AUC=0.548)
SIRS Retrospective (AUC=0.615)
SIRS Prospective (AUC=0.649)

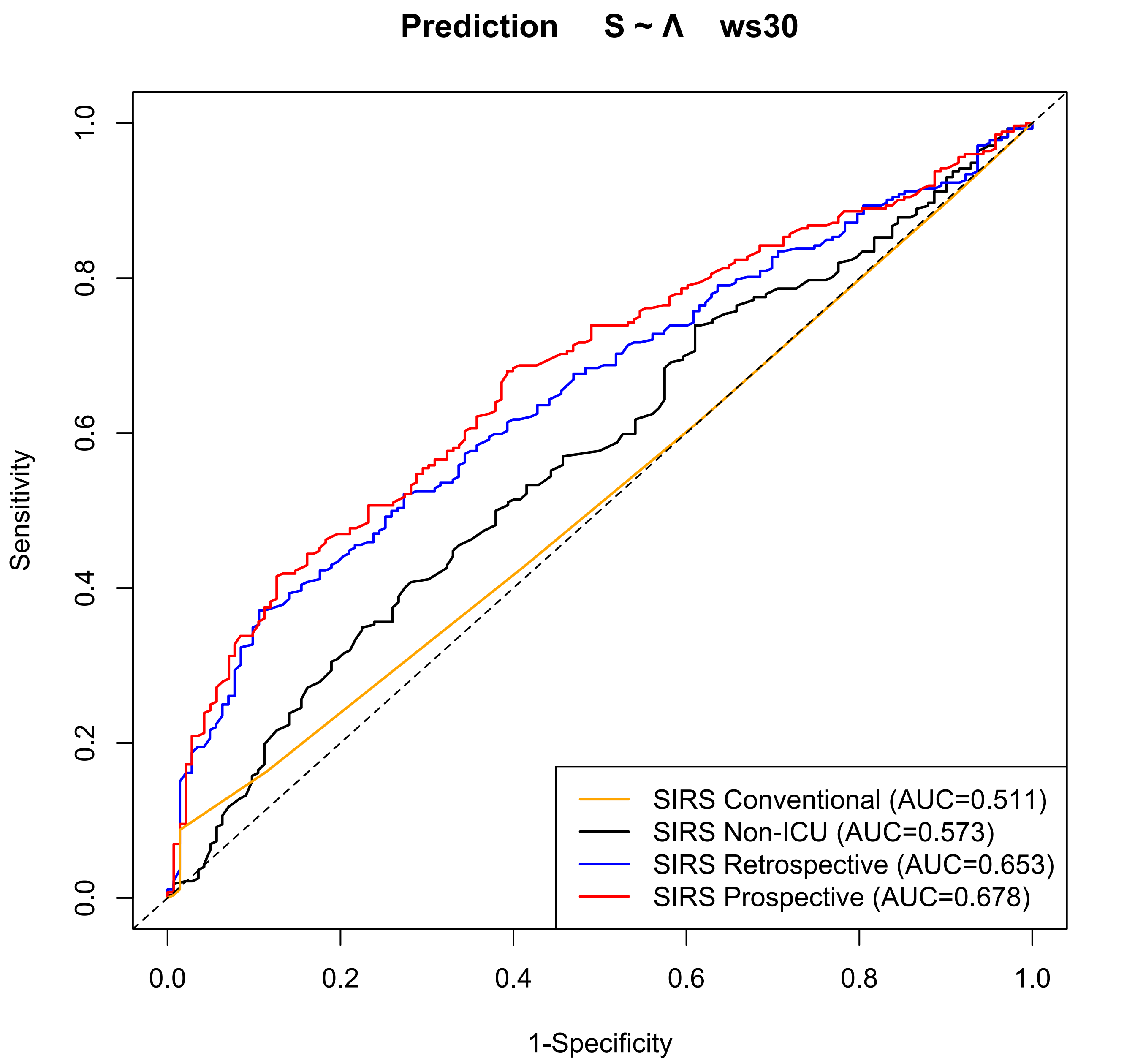

Prediction S ~ Λ ws30
Sensitivity
1-Specificity
0.0
0.2
0.4
0.6
0.8
1.0
SIRS Conventional (AUC=0.511)
SIRS Non-ICU (AUC=0.573)
SIRS Retrospective (AUC=0.653)
SIRS Prospective (AUC=0.678)

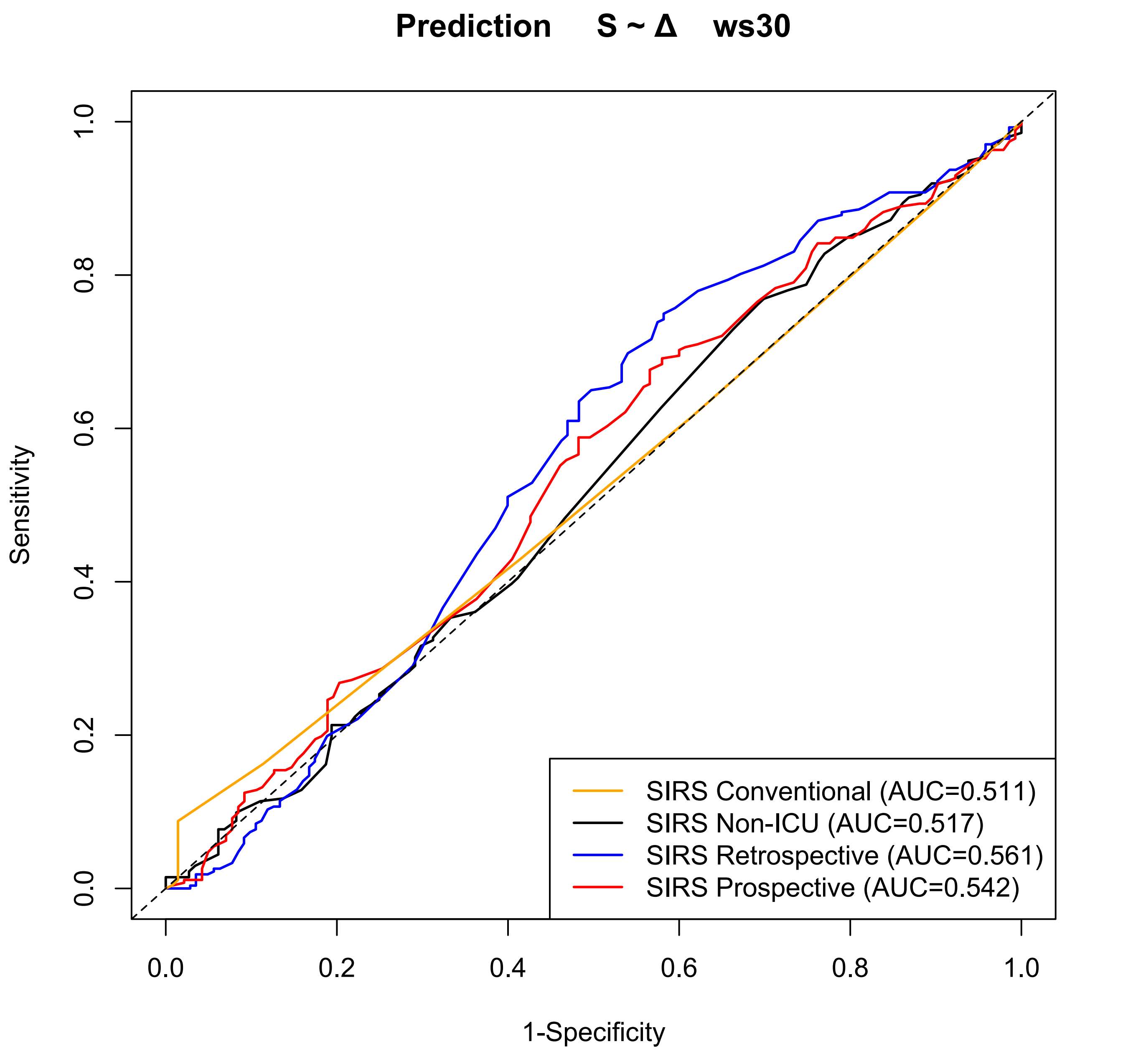

Prediction S ~ Δ ws30
Sensitivity
1-Specificity
0.0
0.2
0.4
0.6
0.8
1.0
SIRS Conventional (AUC=0.511)
SIRS Non-ICU (AUC=0.517)
SIRS Retrospective (AUC=0.561)
SIRS Prospective (AUC=0.542)

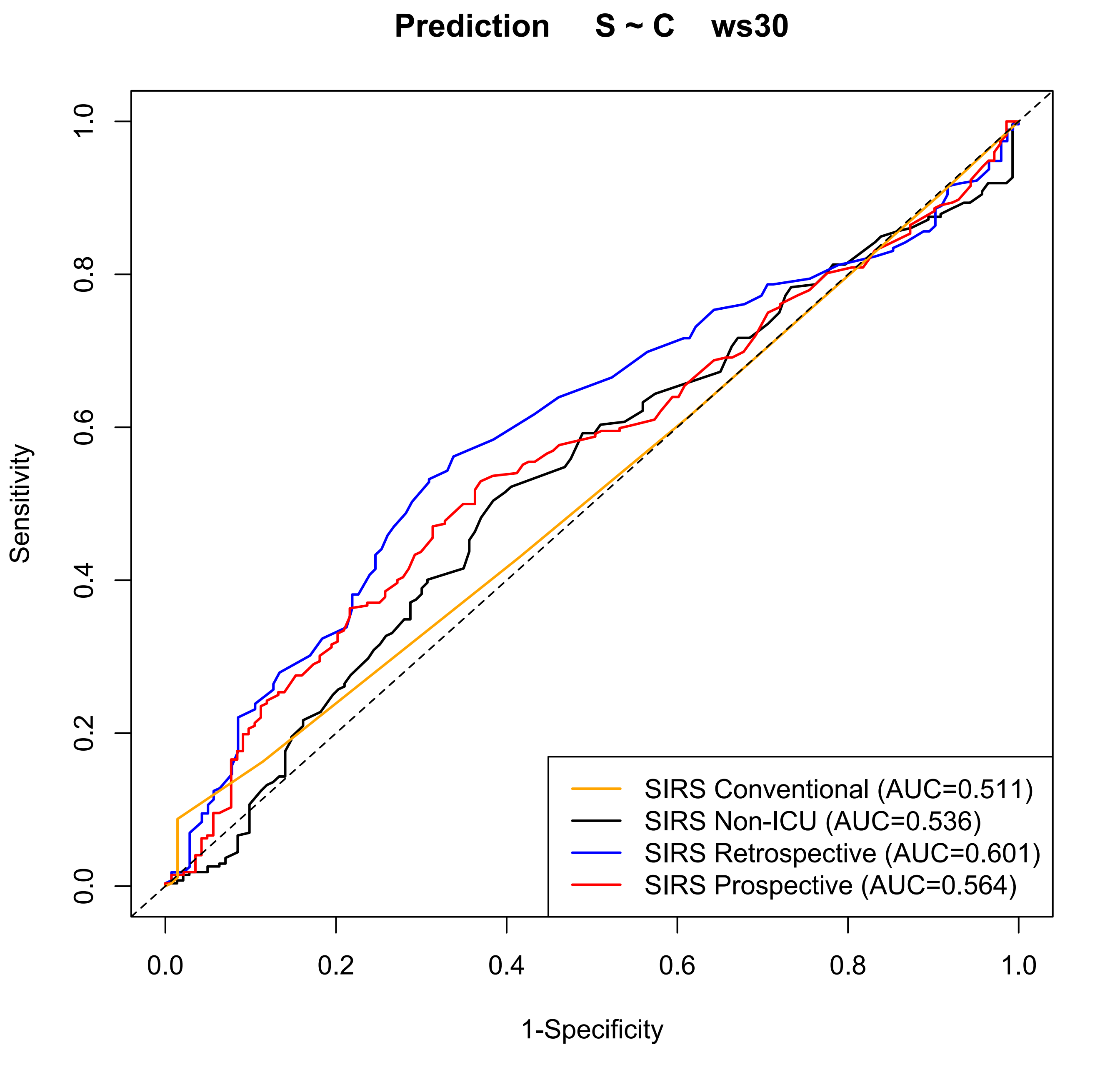

Prediction S ~ C ws30
Sensitivity
1-Specificity
0.0
0.2
0.4
0.6
0.8
1.0
0.0
0.2
0.4
0.6
0.8
1.0
SIRS Conventional (AUC=0.511)
SIRS Non-ICU (AUC=0.536)
SIRS Retrospective (AUC=0.601)
SIRS Prospective (AUC=0.564)

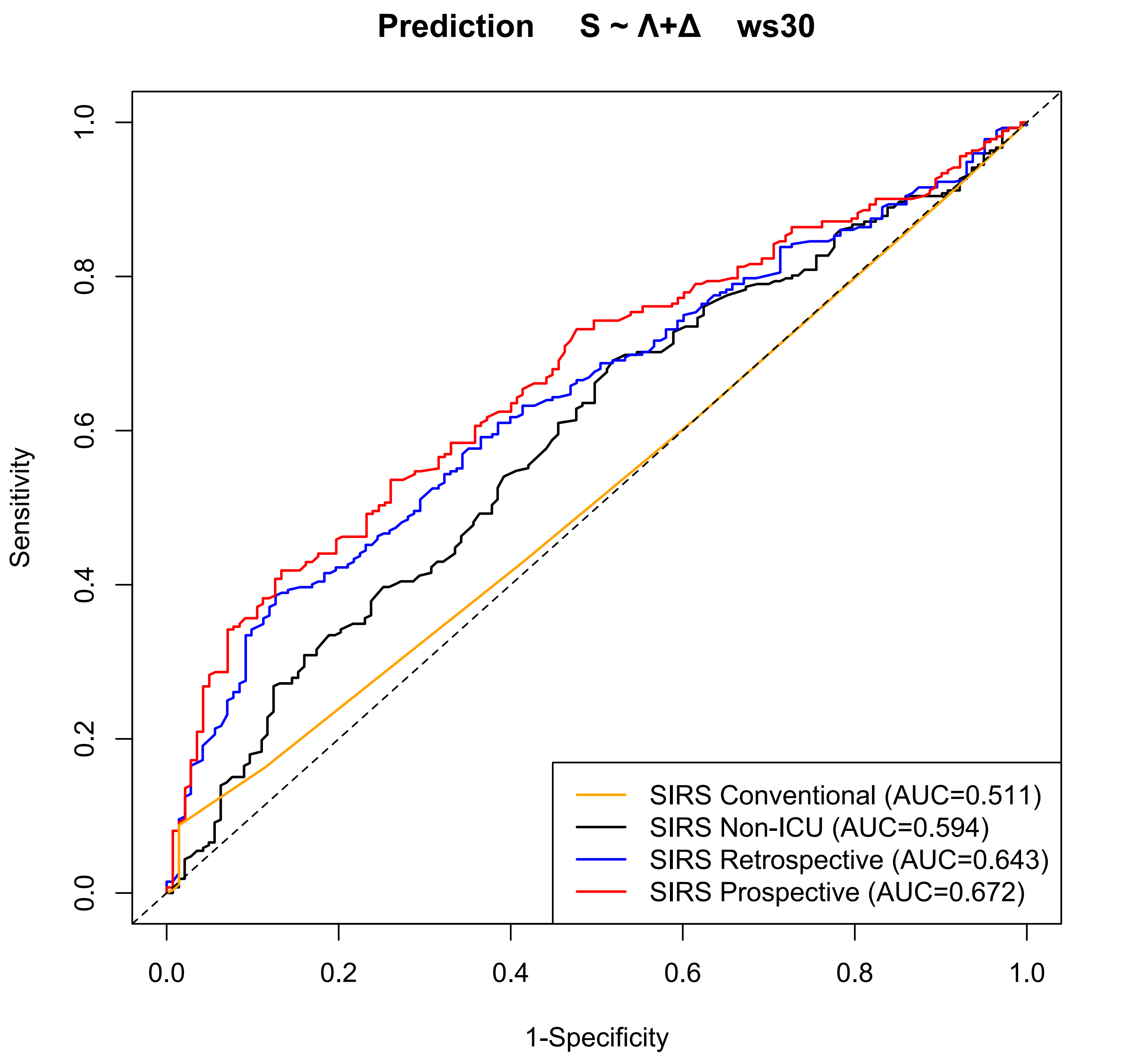

Prediction S ~ Λ+Δ ws30
Sensitivity
1-Specificity
0.0
0.2
0.4
0.6
0.8
1.0
SIRS Conventional (AUC=0.511)
SIRS Non-ICU (AUC=0.594)
SIRS Retrospective (AUC=0.643)
SIRS Prospective (AUC=0.672)

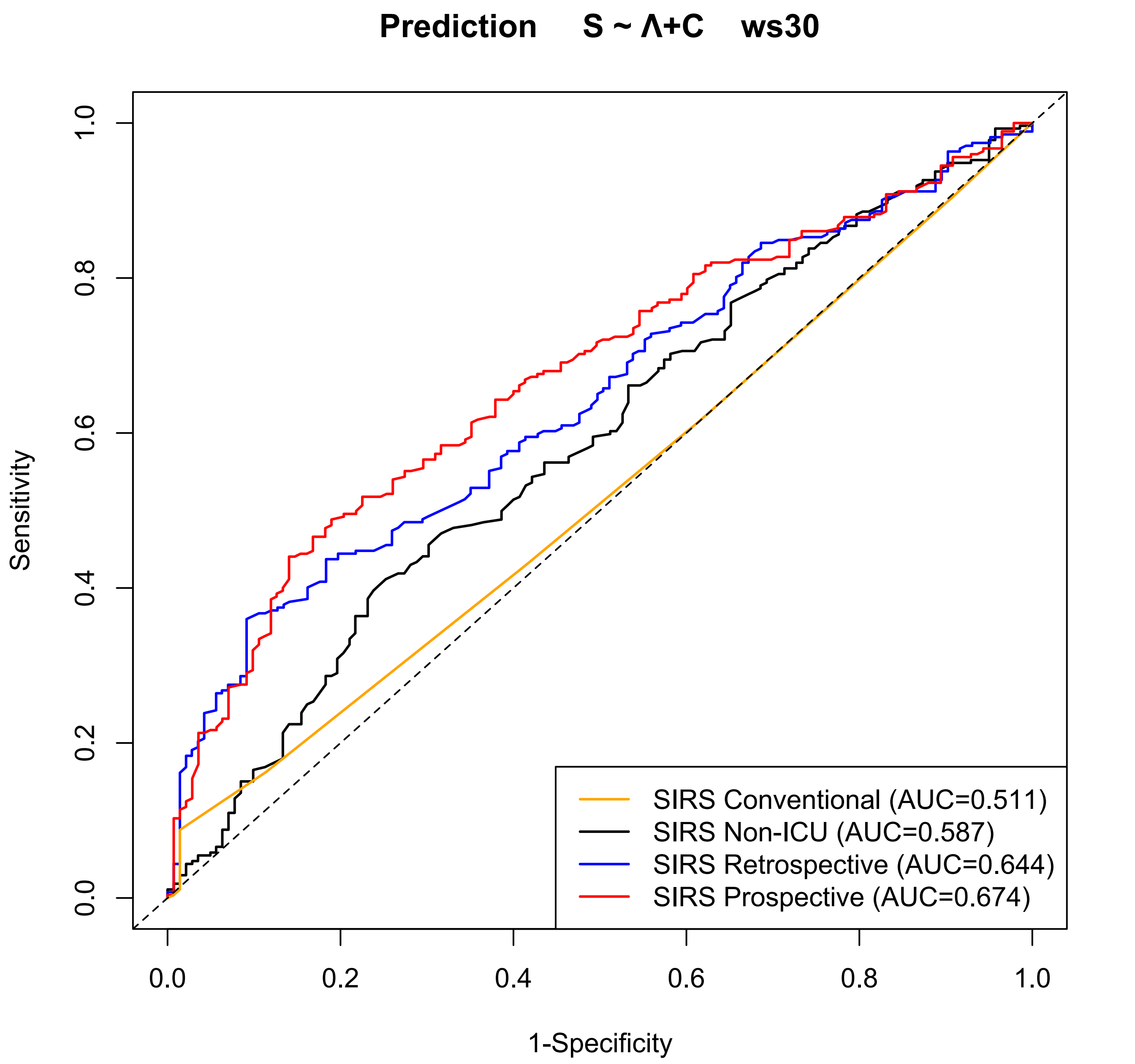

Prediction S ~ Λ+C ws30
Sensitivity
1-Specificity
0.0
0.2
0.4
0.6
0.8
1.0
0.0
0.2
0.4
0.6
0.8
1.0
SIRS Conventional (AUC=0.511)
SIRS Non-ICU (AUC=0.587)
SIRS Retrospective (AUC=0.644)
SIRS Prospective (AUC=0.674)

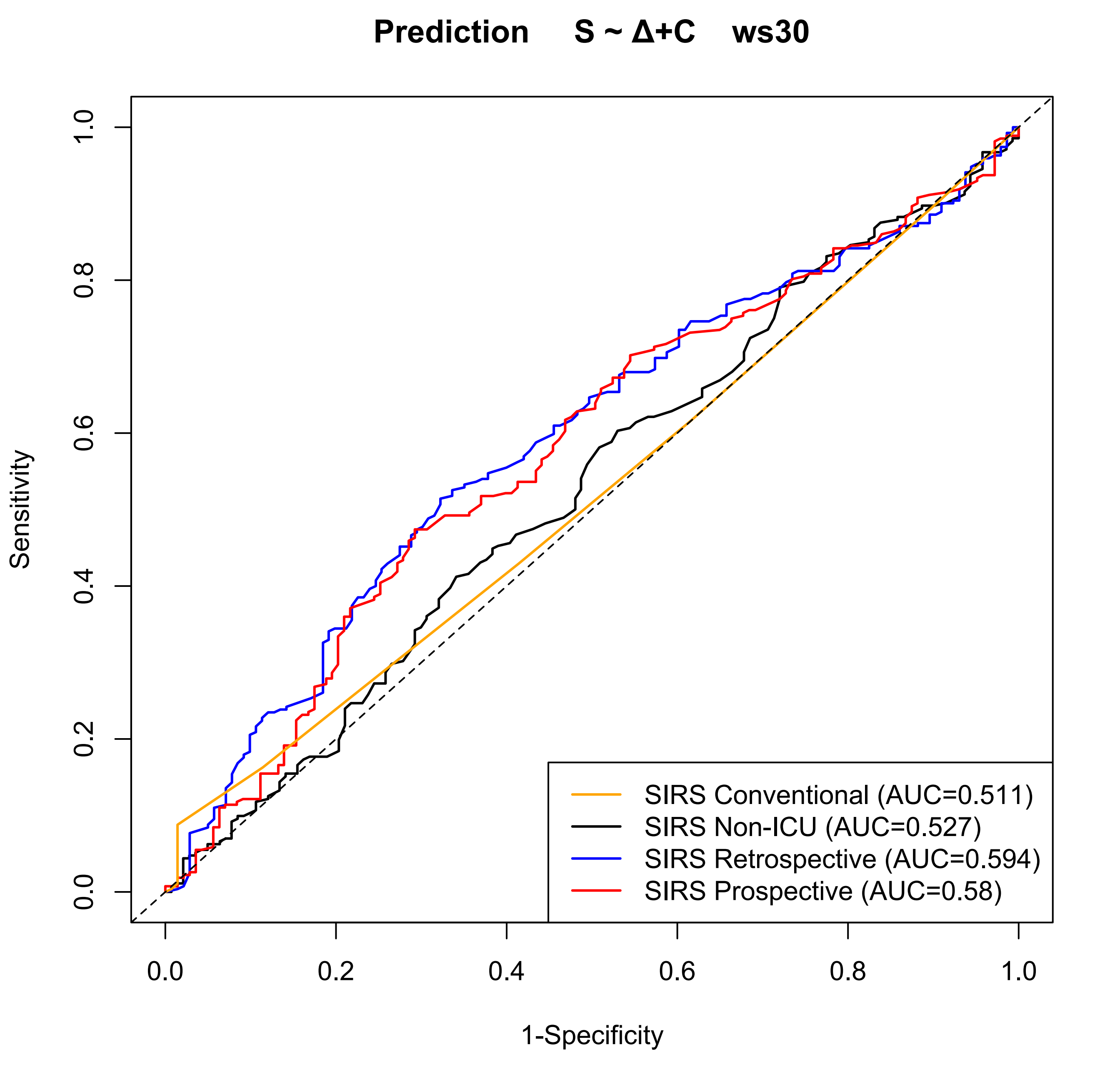

Prediction S ~ Δ+C ws30
Sensitivity
1-Specificity
0.0
0.2
0.4
0.6
0.8
1.0
0.0
0.2
0.4
0.6
0.8
1.0
SIRS Conventional (AUC=0.511)
SIRS Non-ICU (AUC=0.527)
SIRS Retrospective (AUC=0.594)
SIRS Prospective (AUC=0.58)

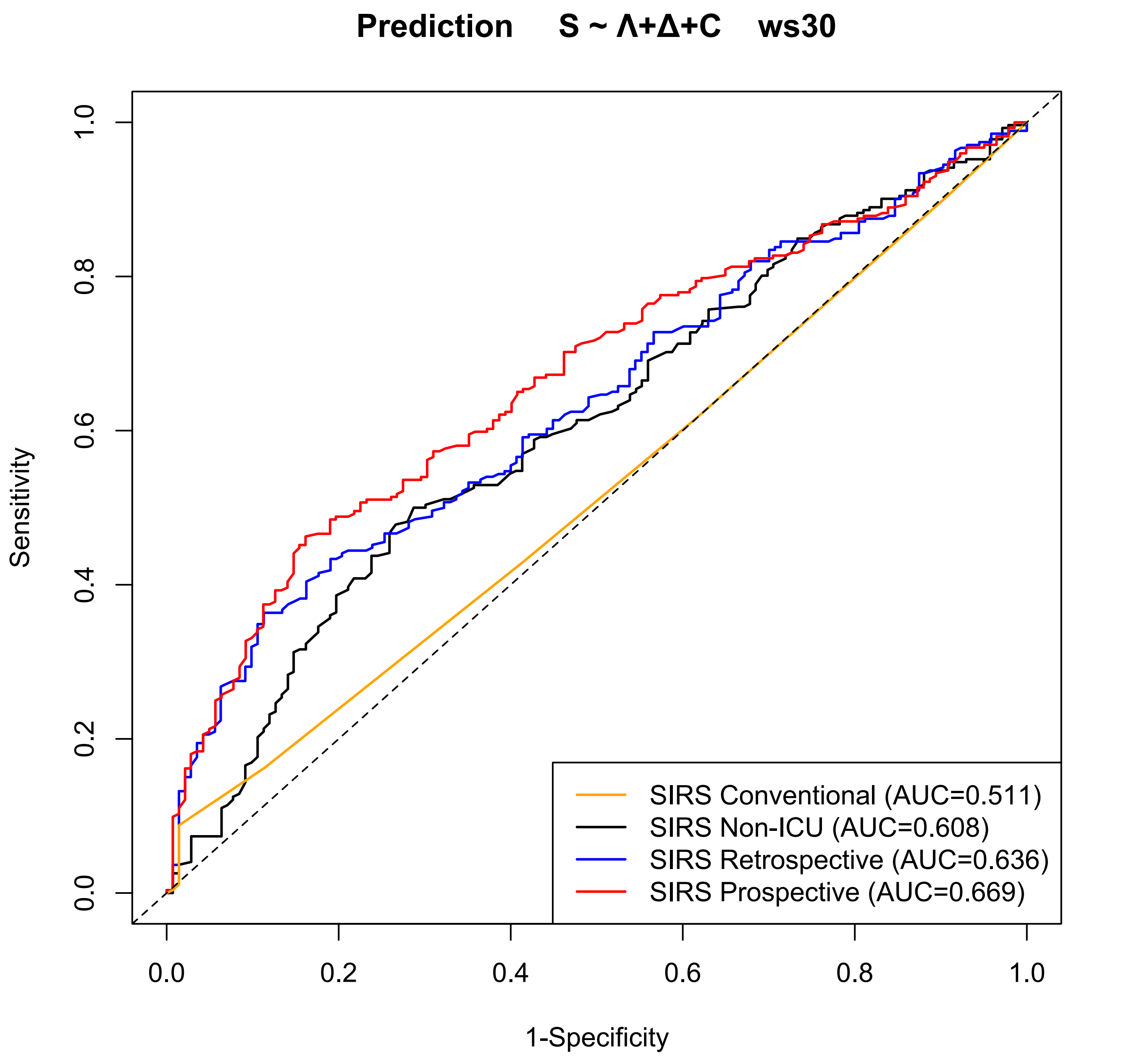
Prediction S ~ Λ+Δ+C ws30
Sensitivity
1-Specificity
0.0
0.2
0.4
0.6
0.8
1.0
0.0
0.2
0.4
0.6
0.8
1.0
SIRS Conventional (AUC=0.511)
SIRS Non-ICU (AUC=0.608)
SIRS Retrospective (AUC=0.636)
SIRS Prospective (AUC=0.669)

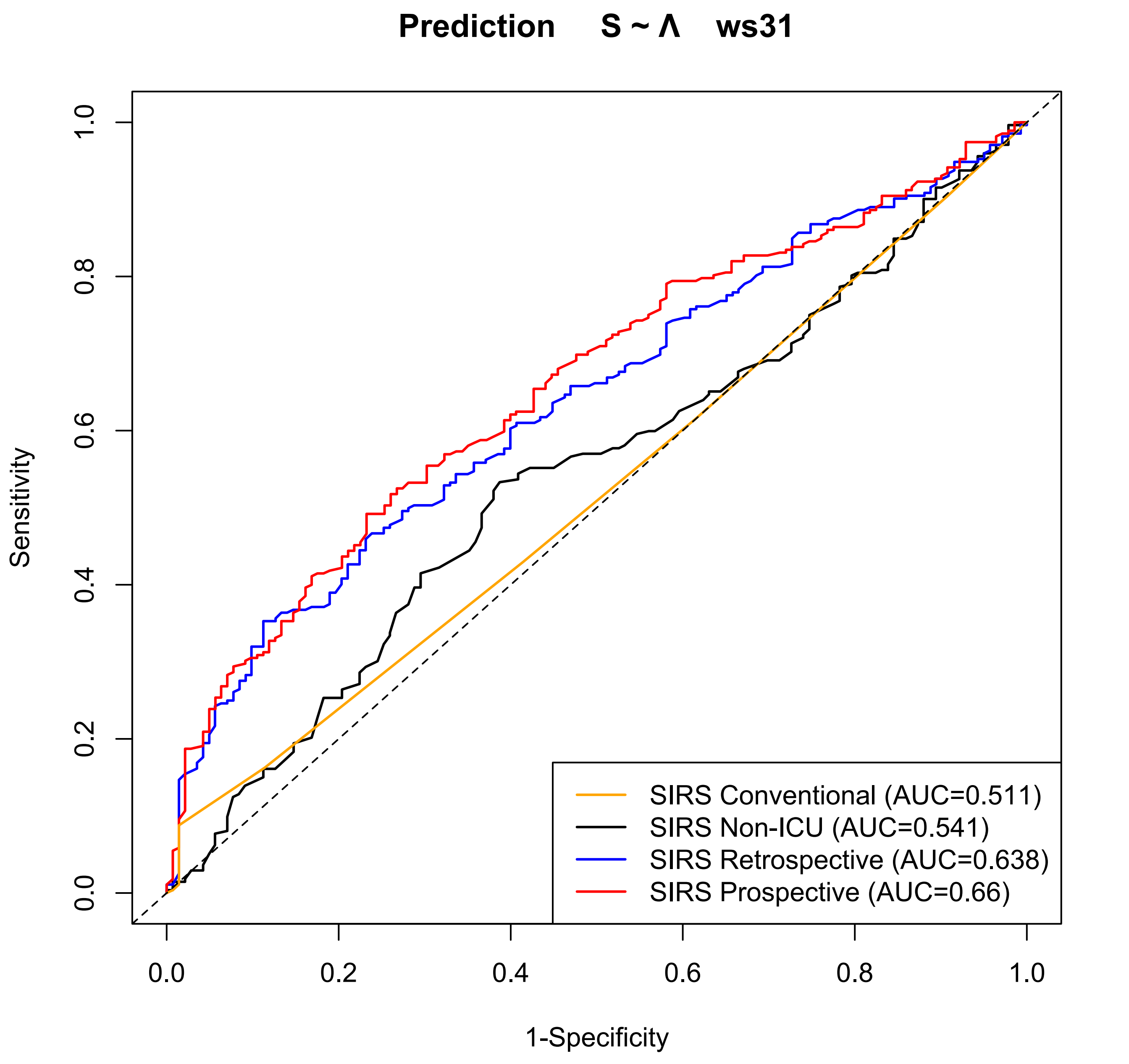
Prediction S ~ Λ ws31
Sensitivity
1-Specificity
0.0
0.2
0.4
0.6
0.8
1.0
SIRS Conventional (AUC=0.511)
SIRS Non-ICU (AUC=0.541)
SIRS Retrospective (AUC=0.638)
SIRS Prospective (AUC=0.66)

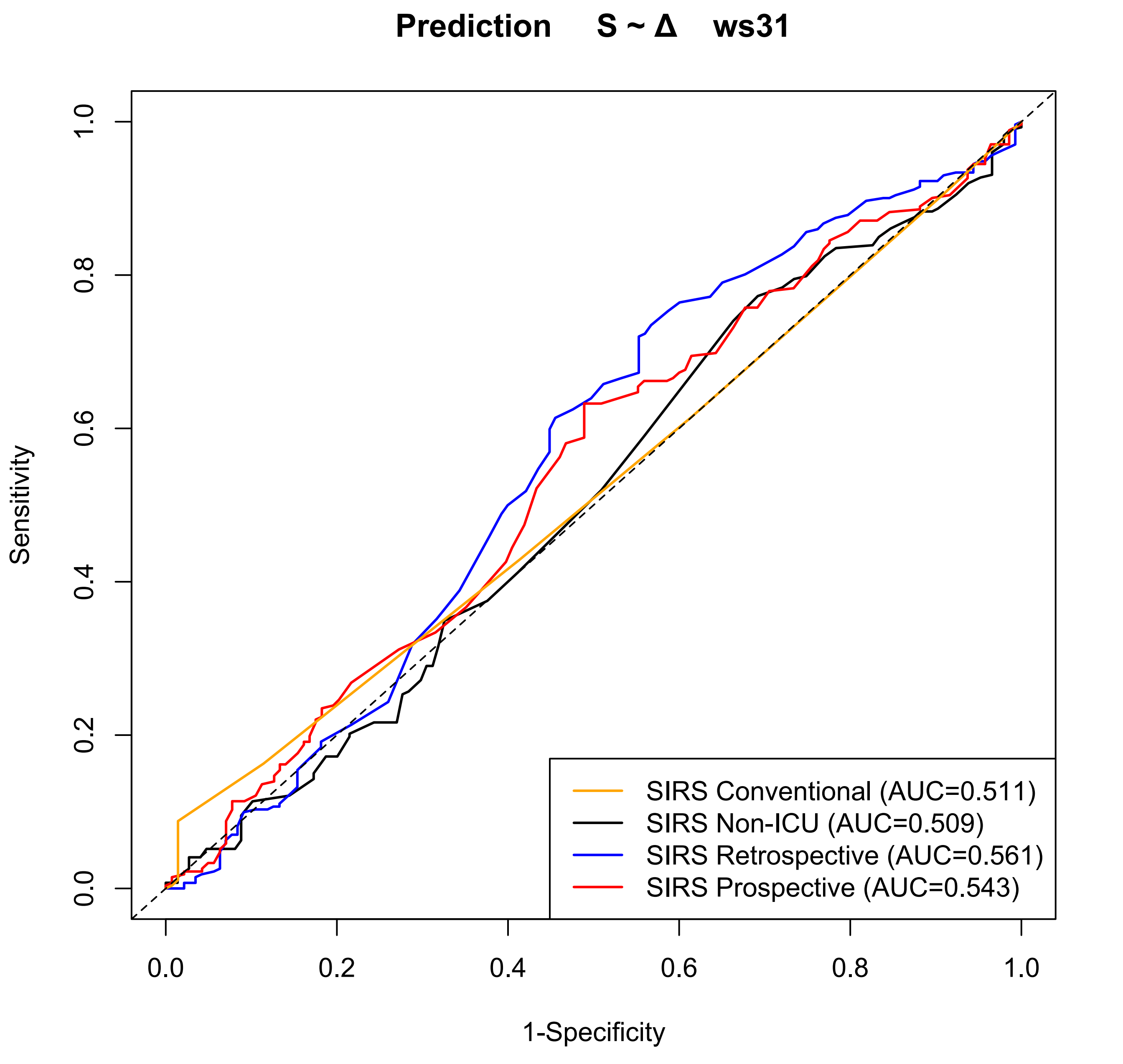

Prediction S ~ Δ ws31
Sensitivity
1-Specificity
0.0
0.2
0.4
0.6
0.8
1.0
SIRS Conventional (AUC=0.511)
SIRS Non-ICU (AUC=0.509)
SIRS Retrospective (AUC=0.561)
SIRS Prospective (AUC=0.543)

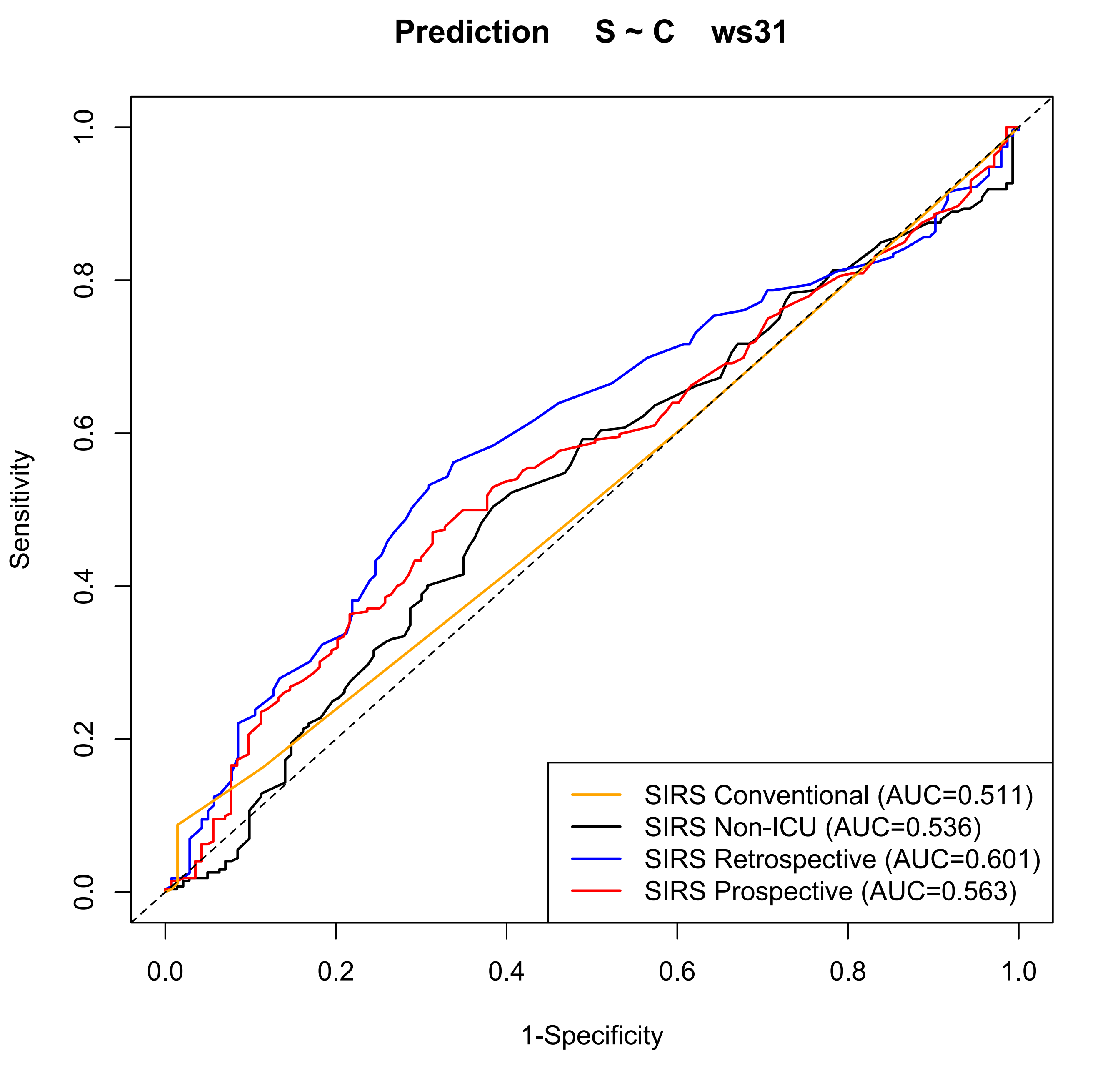

Prediction S ~ C ws31
Sensitivity
1-Specificity
0.0
0.2
0.4
0.6
0.8
1.0
SIRS Conventional (AUC=0.511)
SIRS Non-ICU (AUC=0.536)
SIRS Retrospective (AUC=0.601)
SIRS Prospective (AUC=0.563)

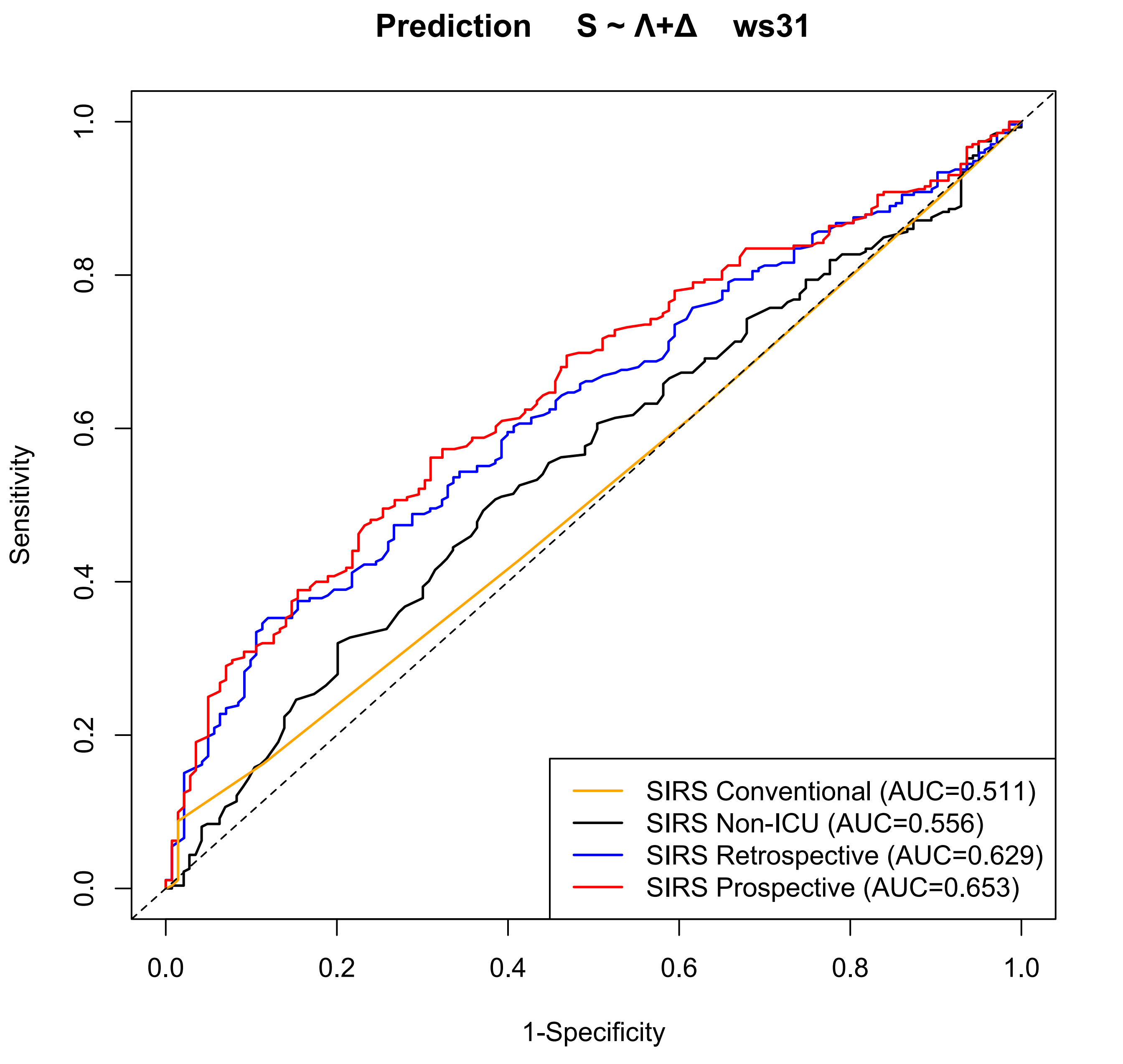
Prediction S ~ Λ+Δ ws31
Sensitivity
1-Specificity
0.0
0.2
0.4
0.6
0.8
1.0
SIRS Conventional (AUC=0.511)
SIRS Non-ICU (AUC=0.556)
SIRS Retrospective (AUC=0.629)
SIRS Prospective (AUC=0.653)

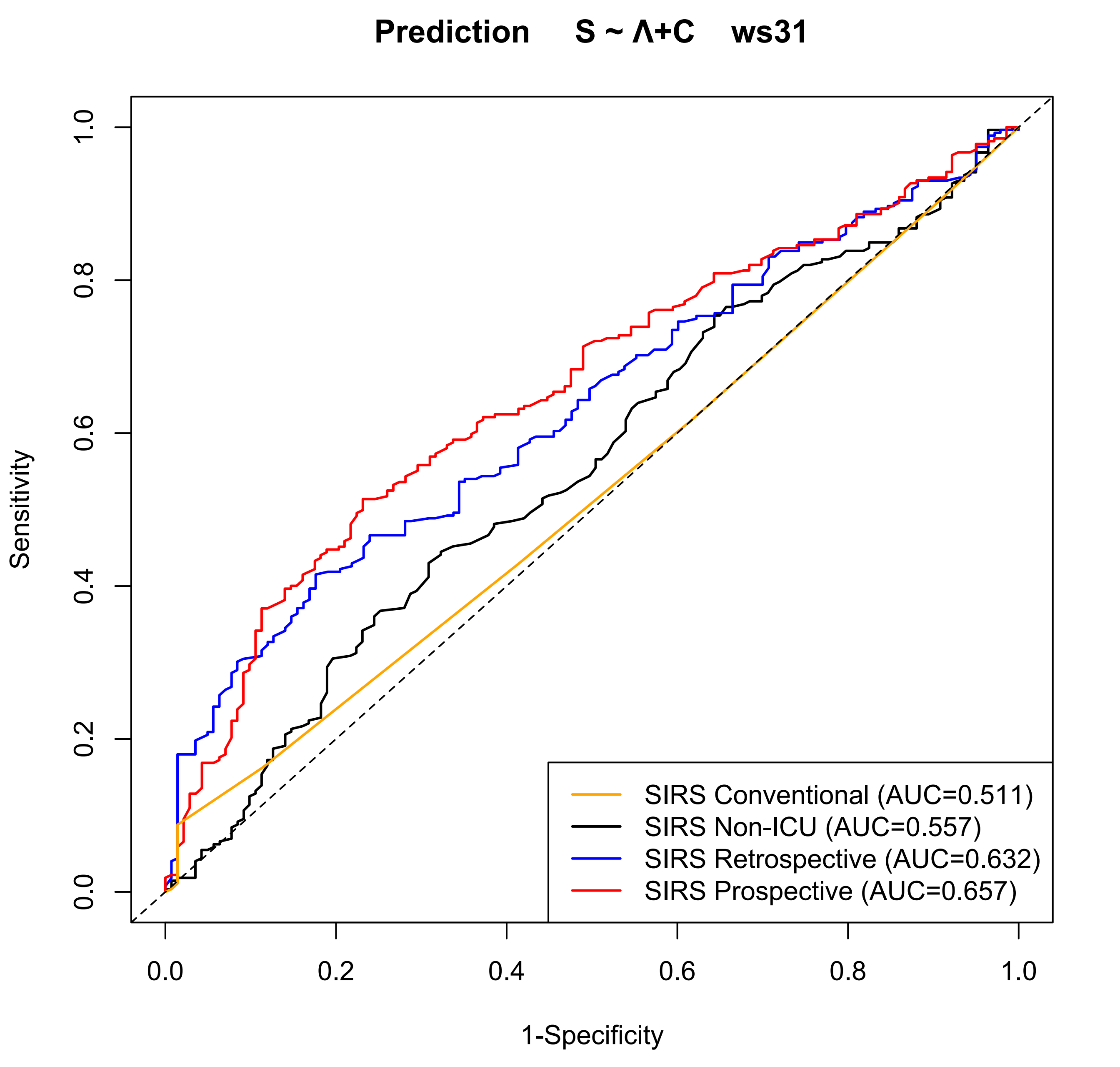

Prediction S ~ Λ+C ws31
Sensitivity
1-Specificity
0.0
0.2
0.4
0.6
0.8
1.0
SIRS Conventional (AUC=0.511)
SIRS Non-ICU (AUC=0.557)
SIRS Retrospective (AUC=0.632)
SIRS Prospective (AUC=0.657)

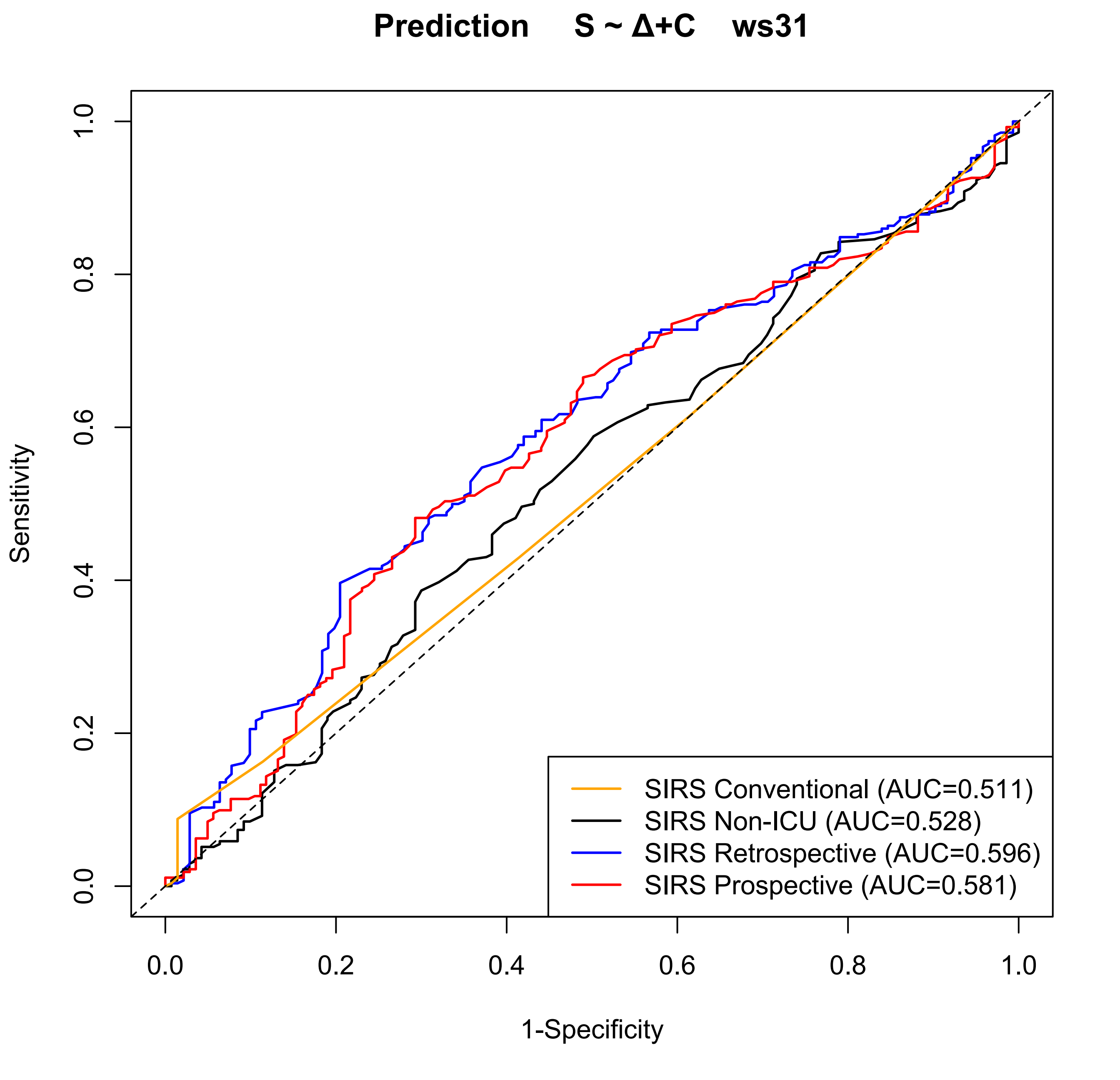
Prediction S ~ Δ+C ws31
Sensitivity
1-Specificity
0.0
0.2
0.4
0.6
0.8
1.0
SIRS Conventional (AUC=0.511)
SIRS Non-ICU (AUC=0.528)
SIRS Retrospective (AUC=0.596)
SIRS Prospective (AUC=0.581)

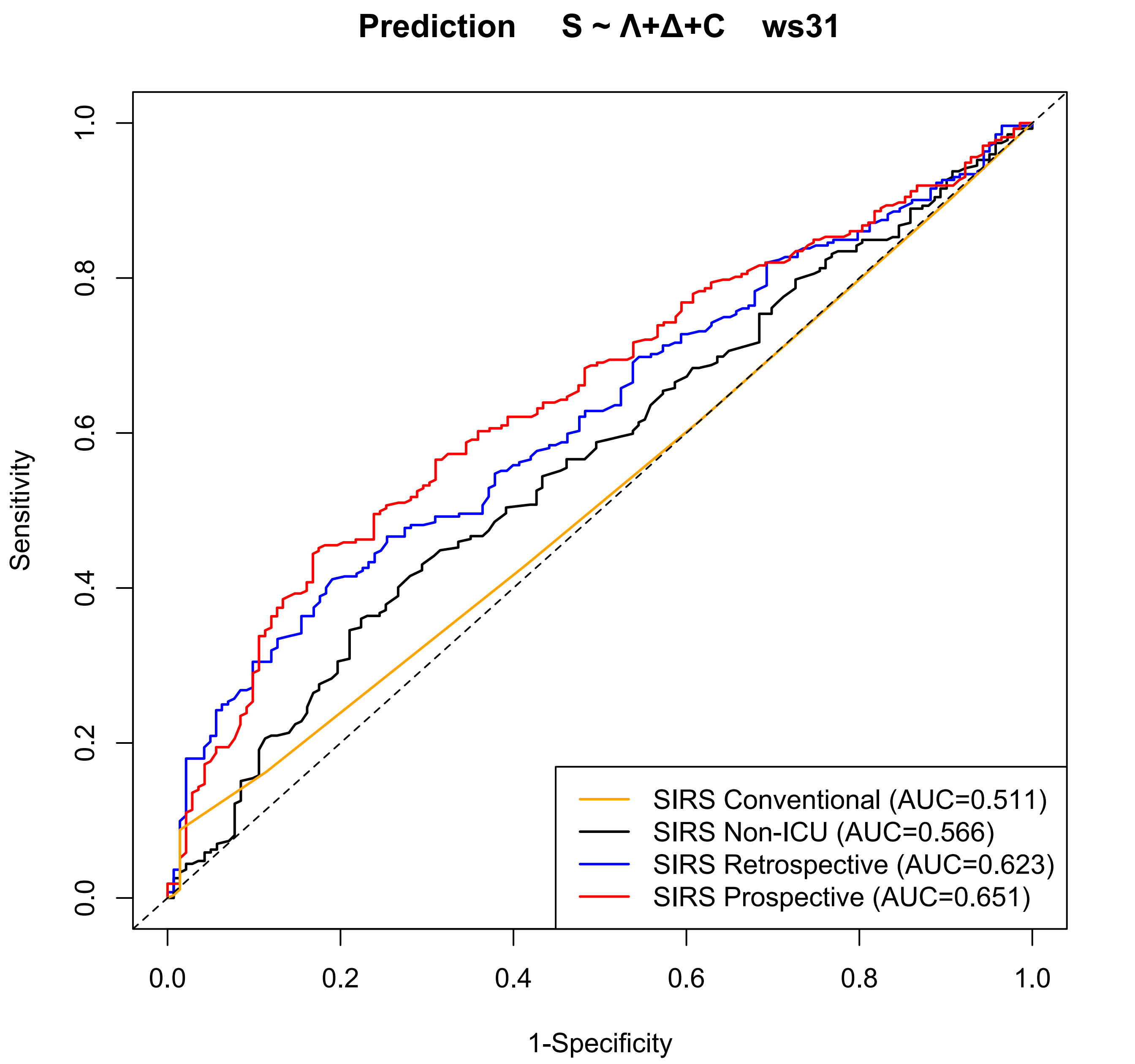

Prediction S ~ Λ+Δ+C ws31
Sensitivity
1-Specificity
0.0
0.2
0.4
0.6
0.8
1.0
SIRS Conventional (AUC=0.511)
SIRS Non-ICU (AUC=0.566)
SIRS Retrospective (AUC=0.623)
SIRS Prospective (AUC=0.651)

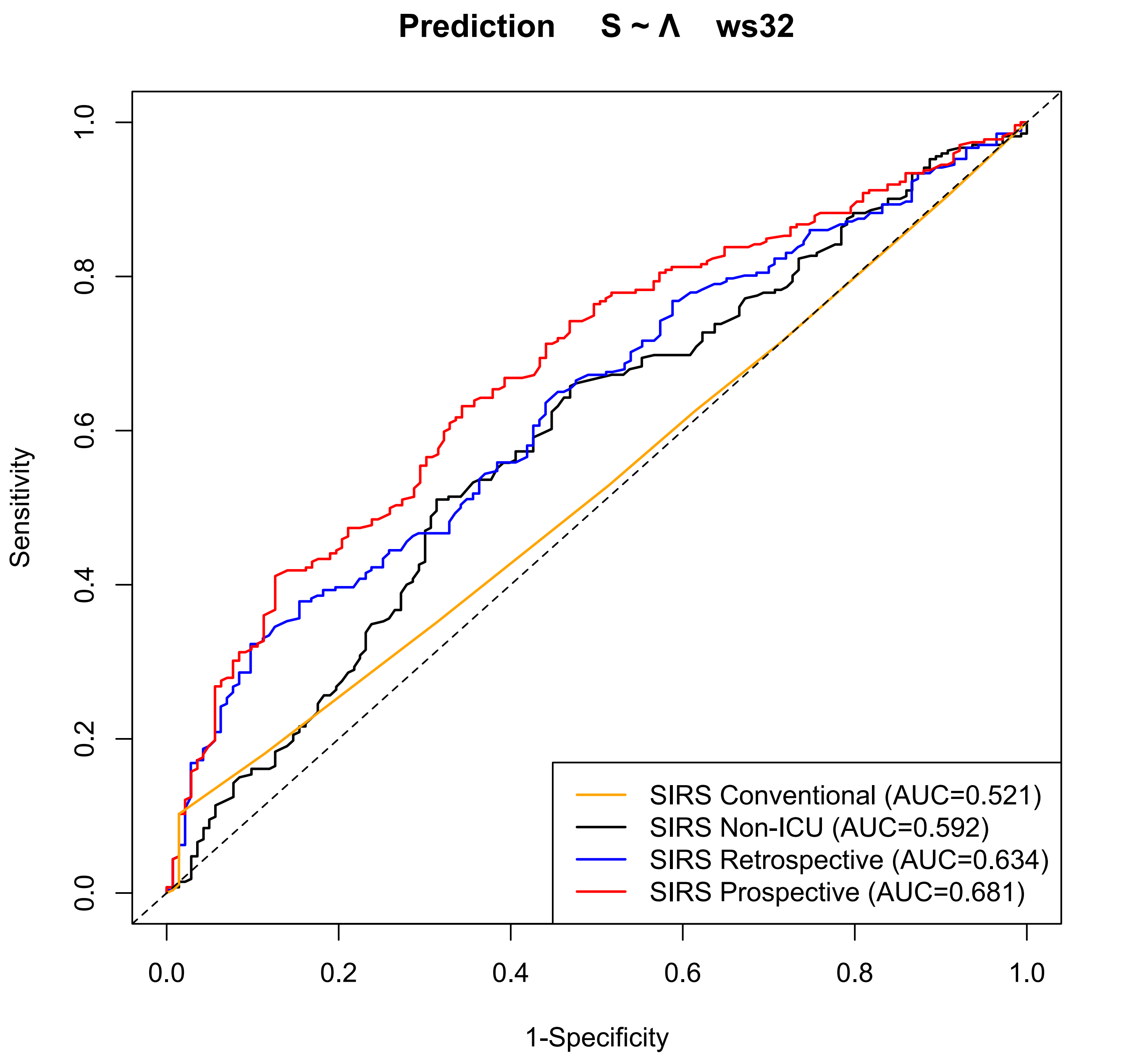

Prediction S ~ Λ ws32
Sensitivity
1-Specificity
0.0
0.2
0.4
0.6
0.8
1.0
SIRS Conventional (AUC=0.521)
SIRS Non-ICU (AUC=0.592)
SIRS Retrospective (AUC=0.634)
SIRS Prospective (AUC=0.681)

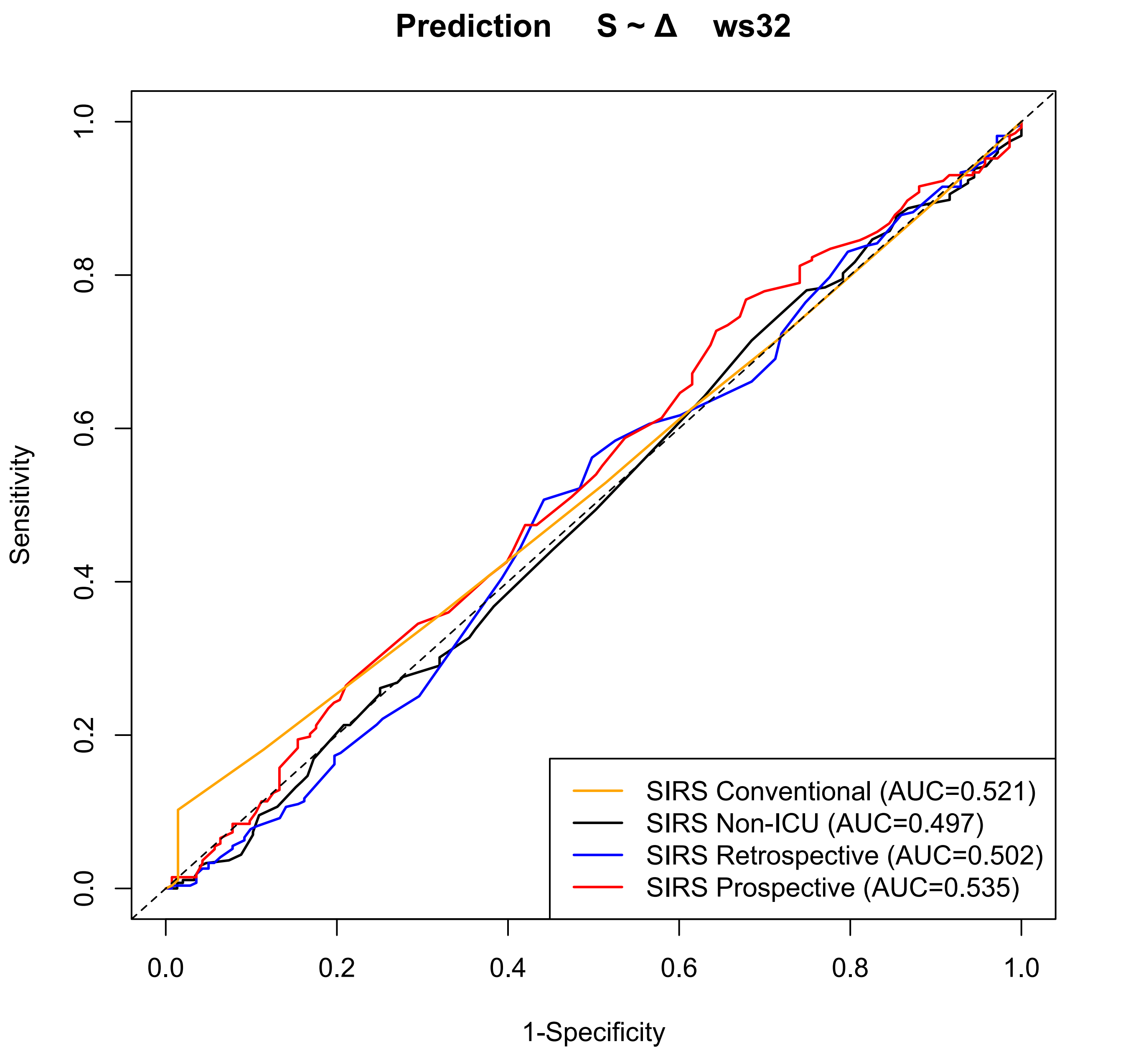

Prediction S ~ Δ ws32
Sensitivity
1-Specificity
0.0
0.2
0.4
0.6
0.8
1.0
0.0
0.2
0.4
0.6
0.8
1.0
SIRS Conventional (AUC=0.521)
SIRS Non-ICU (AUC=0.497)
SIRS Retrospective (AUC=0.502)
SIRS Prospective (AUC=0.535)

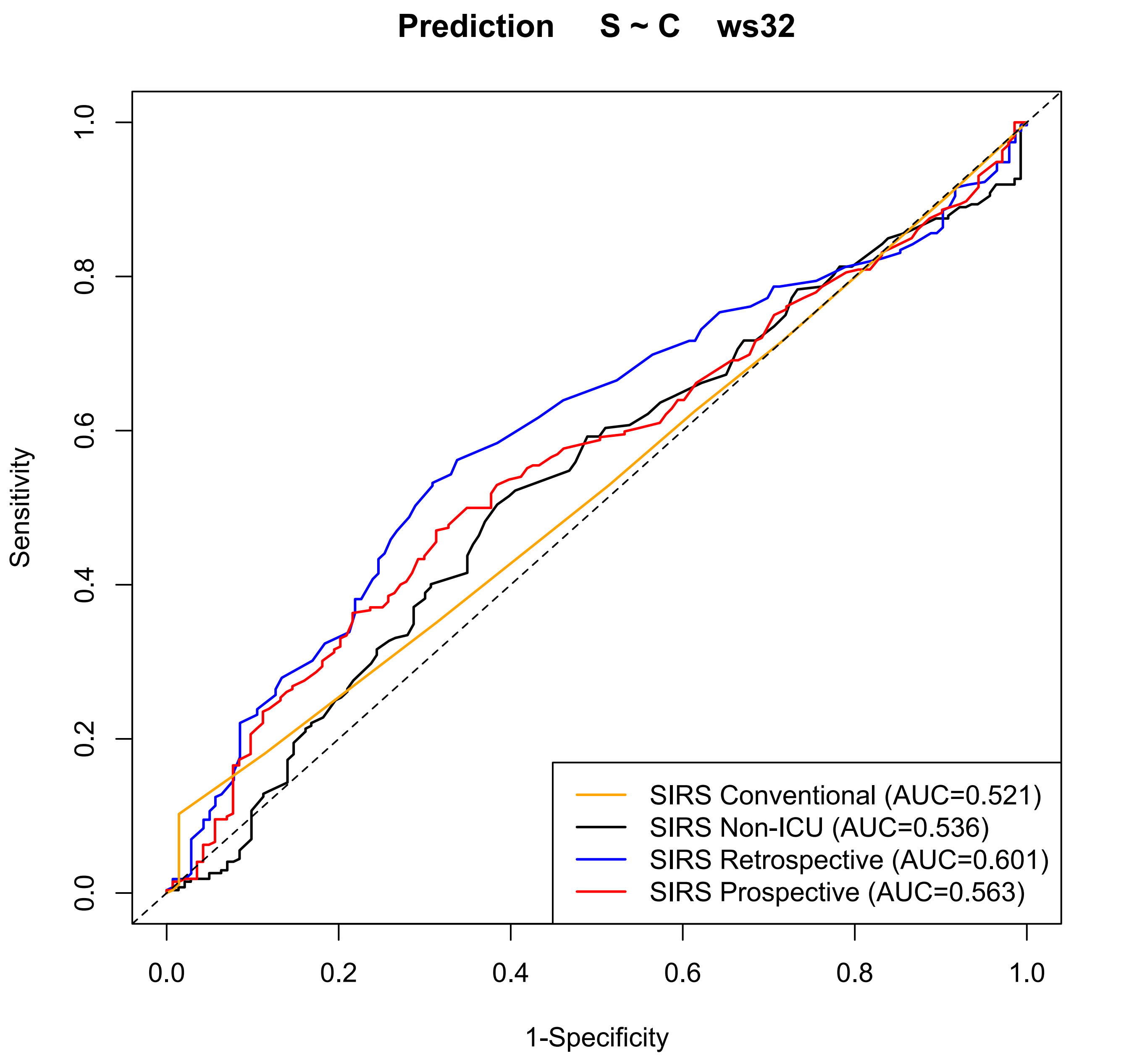

Prediction S ~ C ws32
Sensitivity
1-Specificity
0.0
0.2
0.4
0.6
0.8
1.0
SIRS Conventional (AUC=0.521)
SIRS Non-ICU (AUC=0.536)
SIRS Retrospective (AUC=0.601)
SIRS Prospective (AUC=0.563)

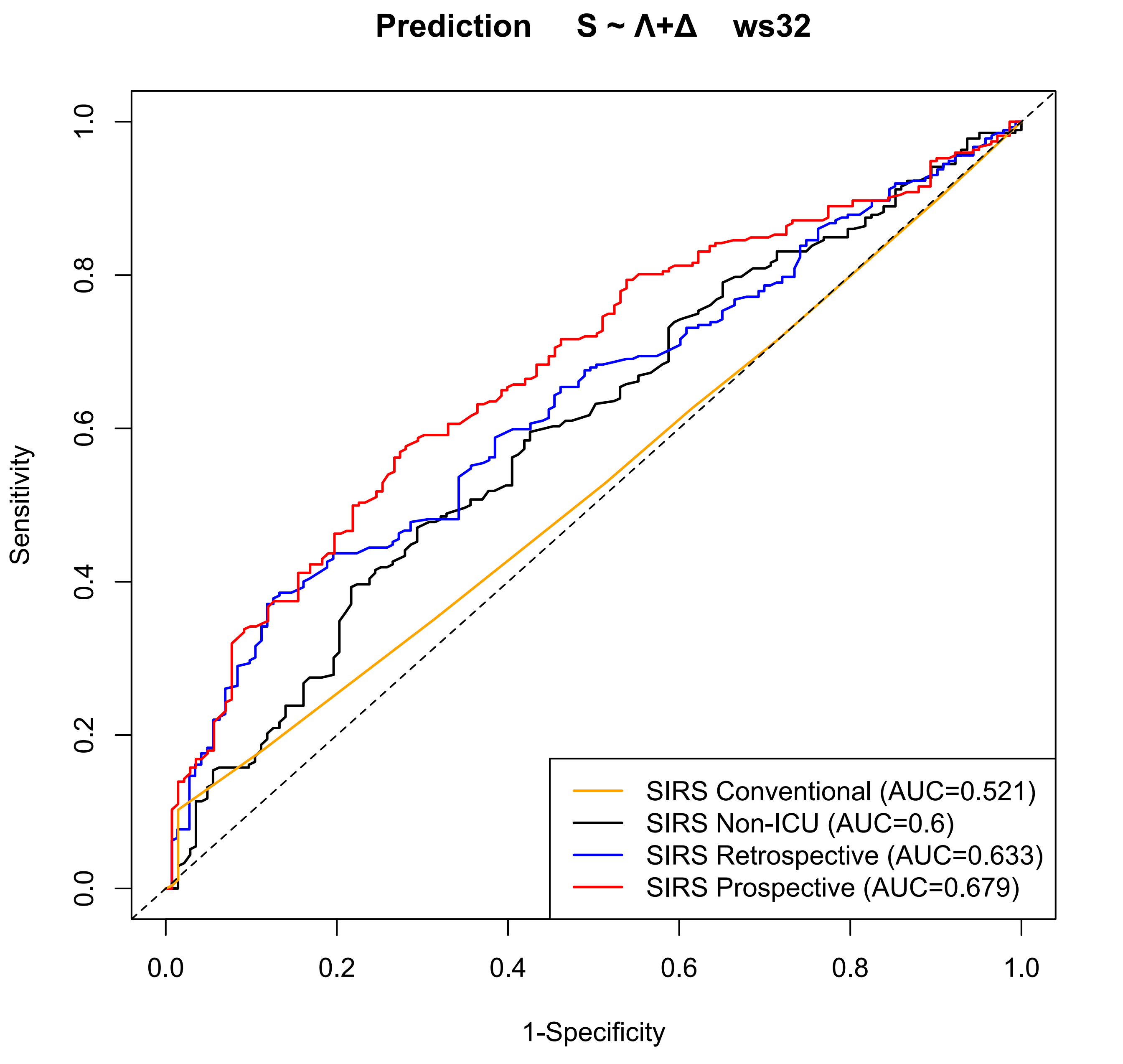

Prediction S ~ Λ+Δ ws32
Sensitivity
1-Specificity
0.0
0.2
0.4
0.6
0.8
1.0
SIRS Conventional (AUC=0.521)
SIRS Non-ICU (AUC=0.6)
SIRS Retrospective (AUC=0.633)
SIRS Prospective (AUC=0.679)

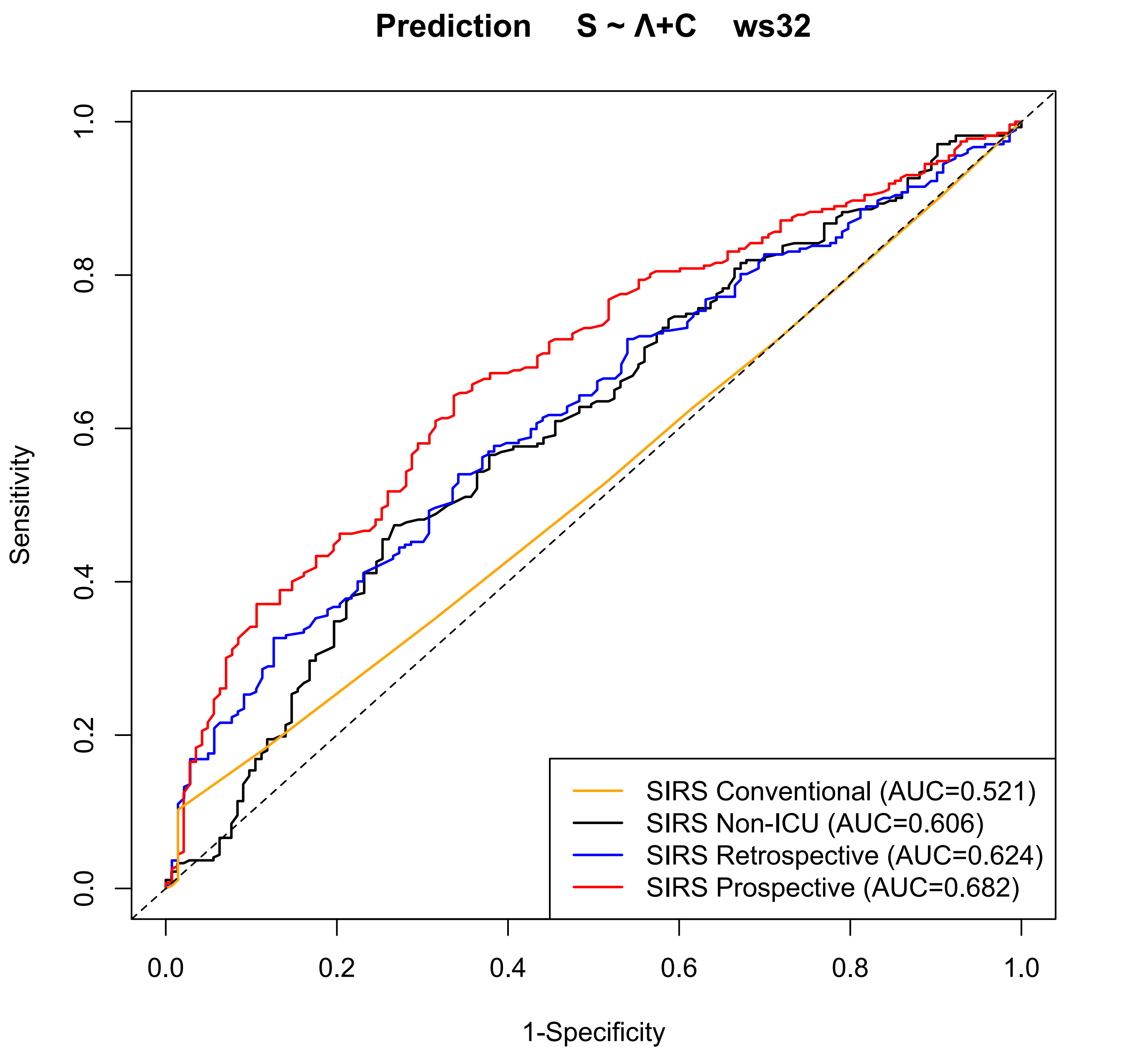
Prediction S ~ Λ+C ws32
Sensitivity
1-Specificity
0.0
0.2
0.4
0.6
0.8
1.0
SIRS Conventional (AUC=0.521)
SIRS Non-ICU (AUC=0.606)
SIRS Retrospective (AUC=0.624)
SIRS Prospective (AUC=0.682)

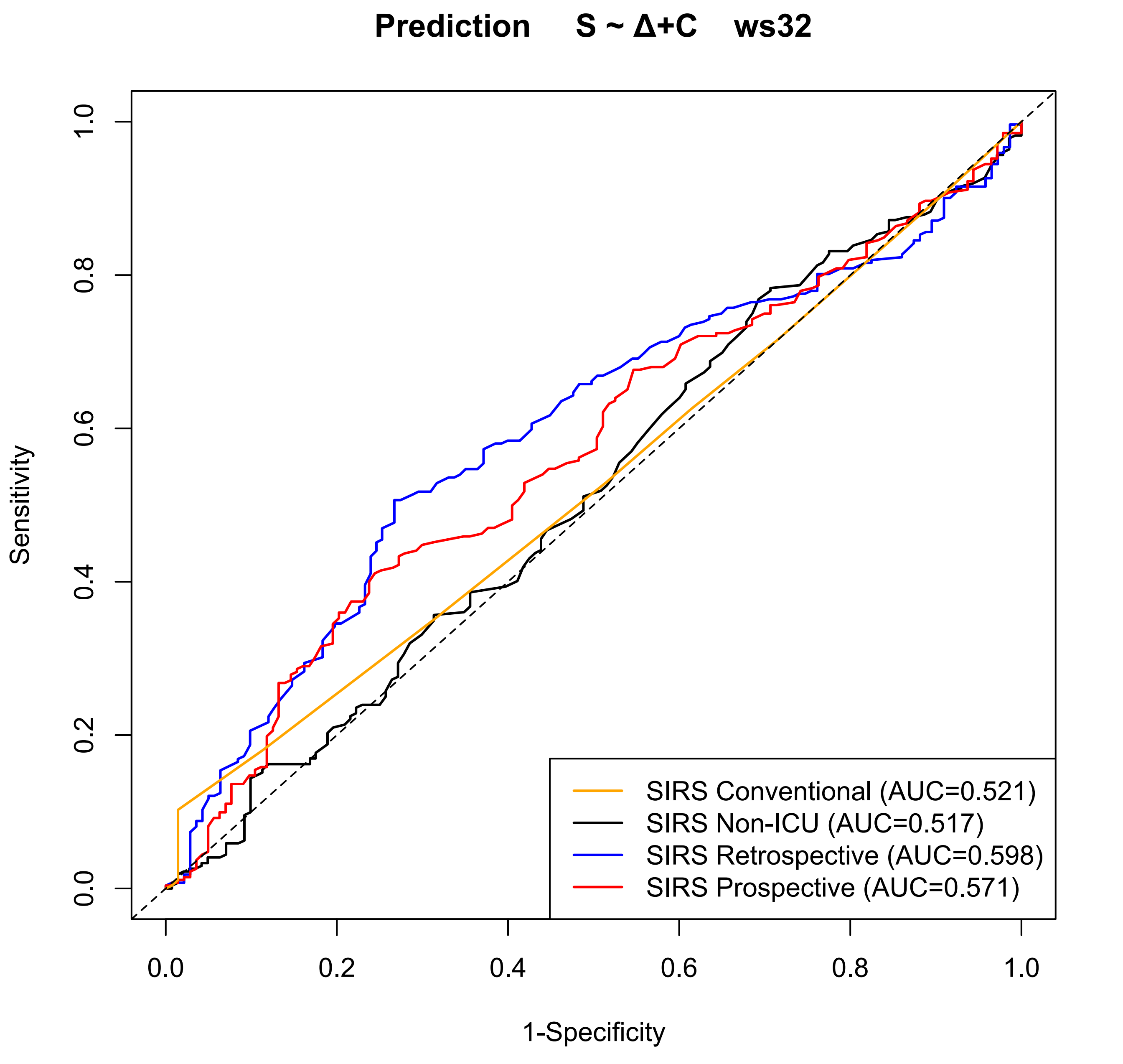
Prediction S ~ Δ+C ws32
Sensitivity
1-Specificity
0.0
0.2
0.4
0.6
0.8
1.0
SIRS Conventional (AUC=0.521)
SIRS Non-ICU (AUC=0.517)
SIRS Retrospective (AUC=0.598)
SIRS Prospective (AUC=0.571)

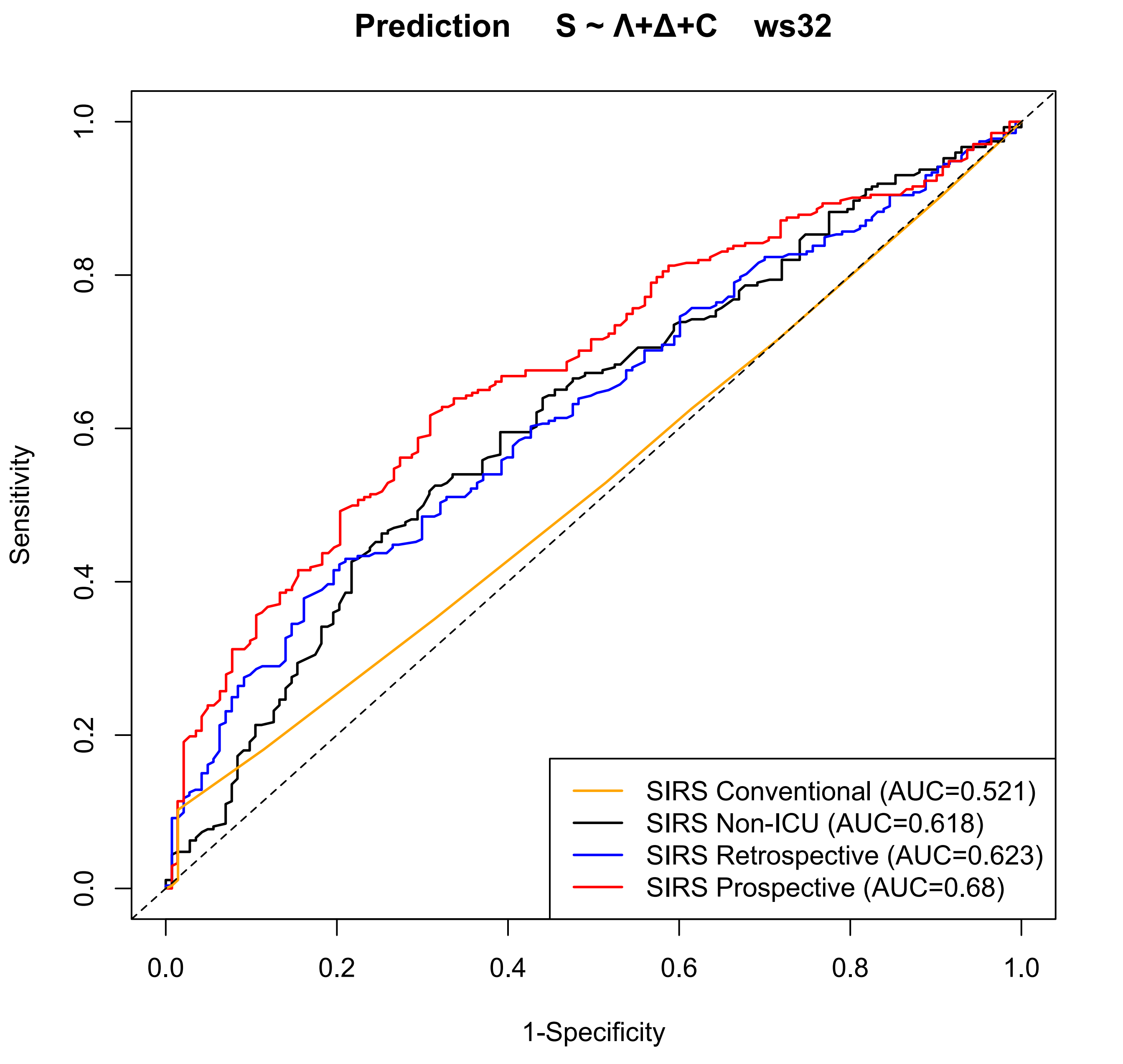

Prediction S ~ Λ+Δ+C ws32
Sensitivity
1-Specificity
0.0
0.2
0.4
0.6
0.8
1.0
0.0
0.2
0.4
0.6
0.8
1.0
SIRS Conventional (AUC=0.521)
SIRS Non-ICU (AUC=0.618)
SIRS Retrospective (AUC=0.623)
SIRS Prospective (AUC=0.68)

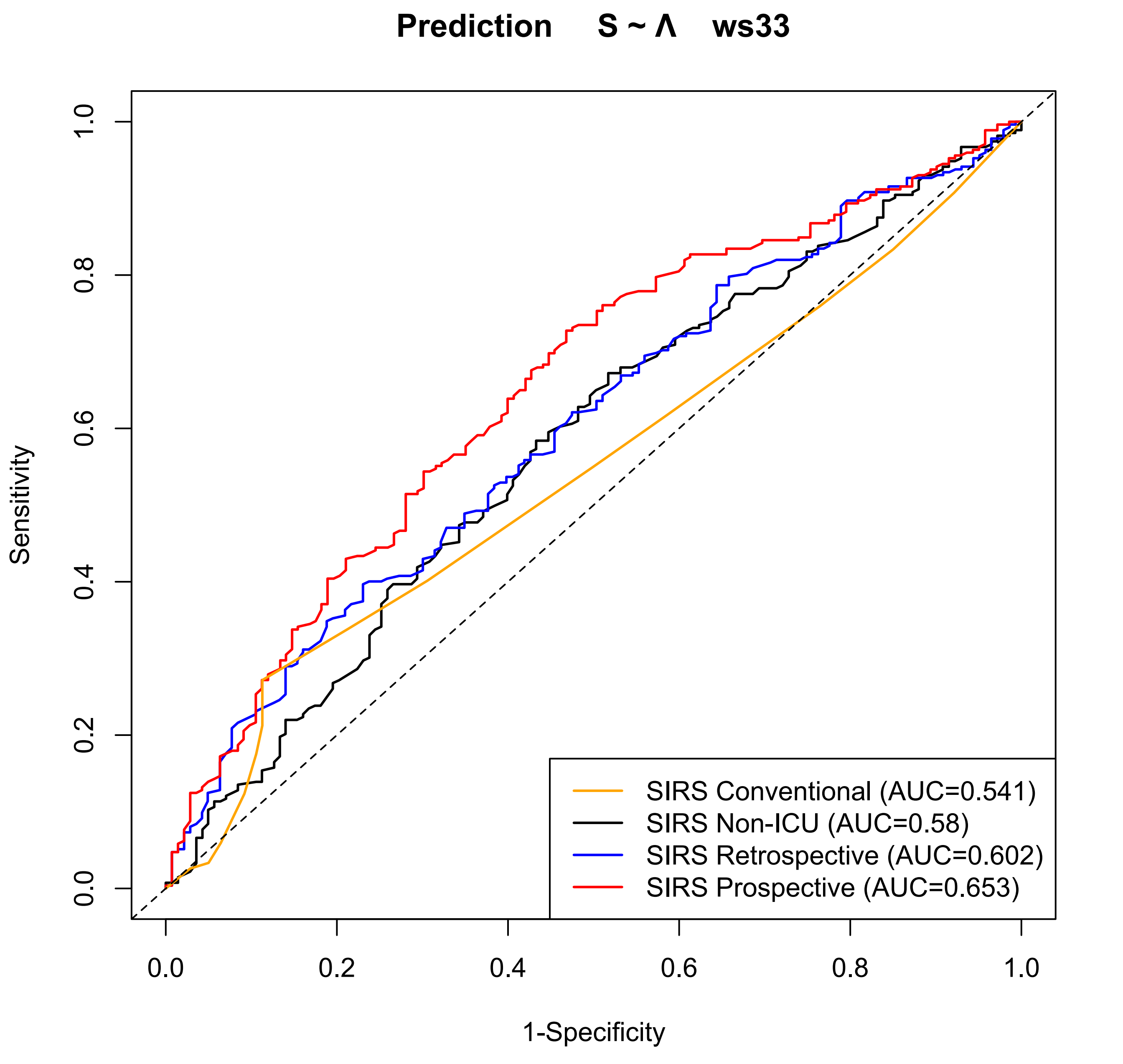

Prediction S ~ Λ ws33
Sensitivity
1-Specificity
0.0
0.2
0.4
0.6
0.8
1.0
SIRS Conventional (AUC=0.541)
SIRS Non-ICU (AUC=0.58)
SIRS Retrospective (AUC=0.602)
SIRS Prospective (AUC=0.653)

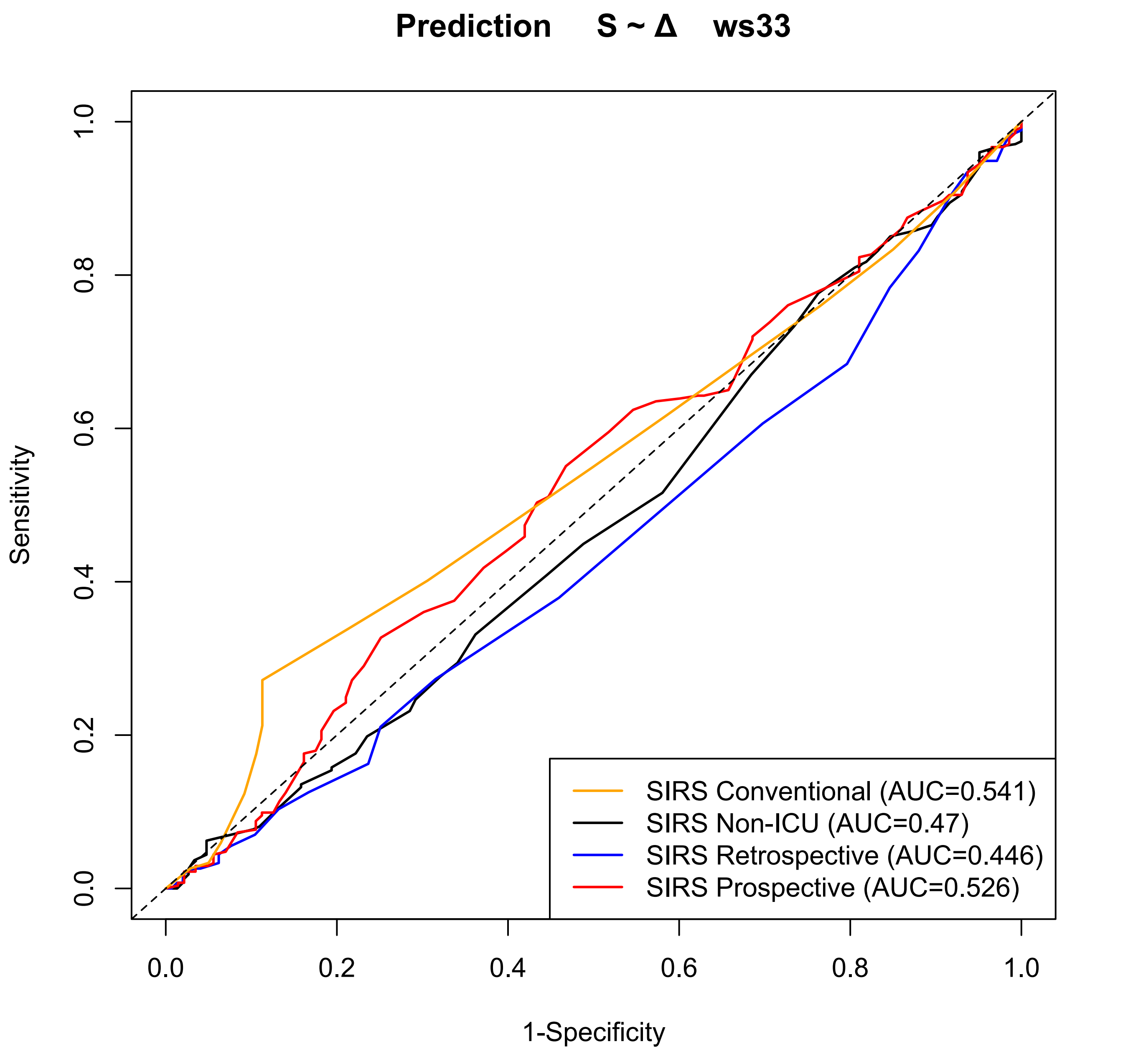
Prediction S ~ Δ ws33
Sensitivity
1-Specificity
0.0
0.2
0.4
0.6
0.8
1.0
SIRS Conventional (AUC=0.541)
SIRS Non-ICU (AUC=0.47)
SIRS Retrospective (AUC=0.446)
SIRS Prospective (AUC=0.526)

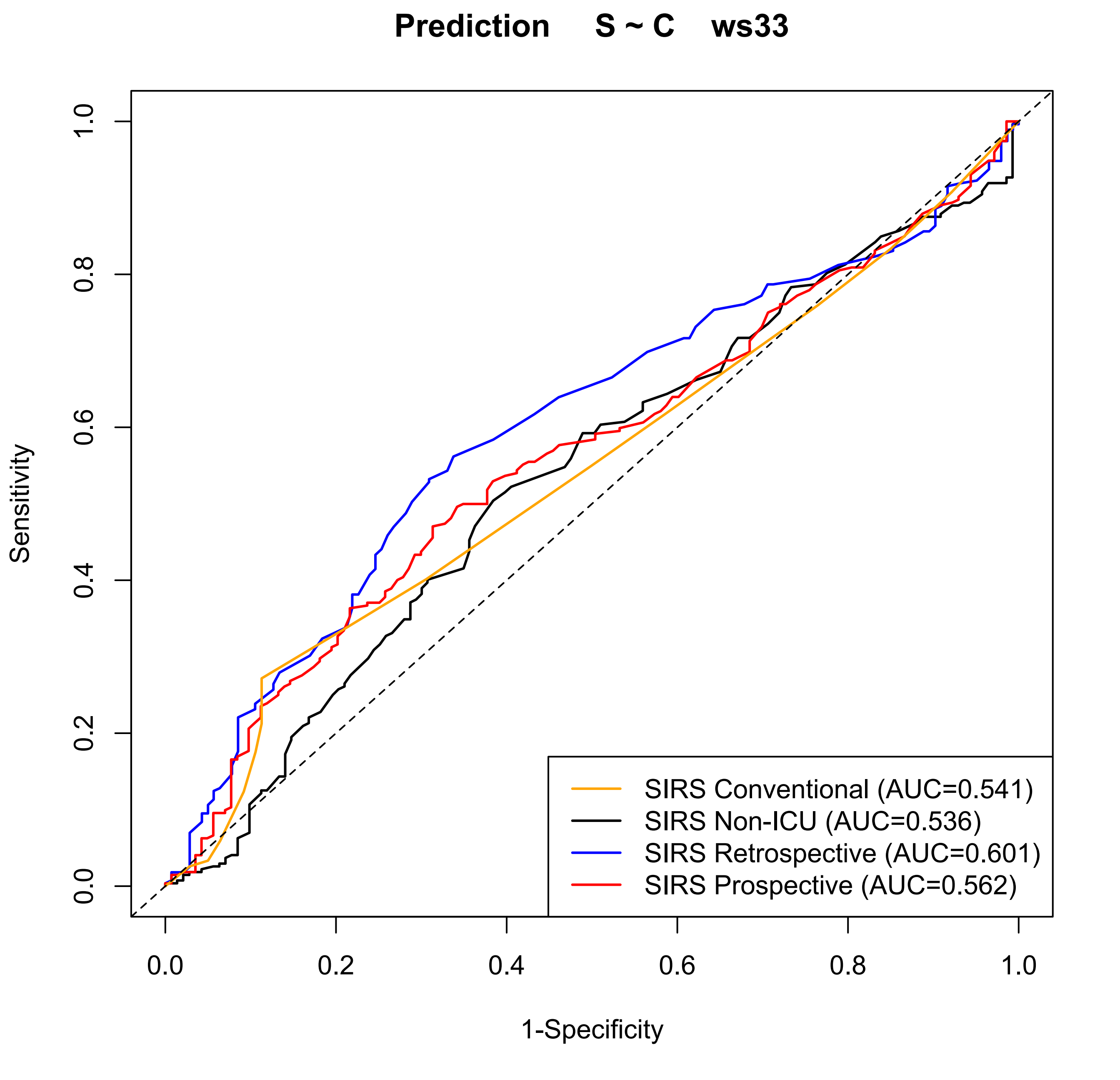

Prediction S ~ C ws33
Sensitivity
1-Specificity
0.0
0.2
0.4
0.6
0.8
1.0
SIRS Conventional (AUC=0.541)
SIRS Non-ICU (AUC=0.536)
SIRS Retrospective (AUC=0.601)
SIRS Prospective (AUC=0.562)

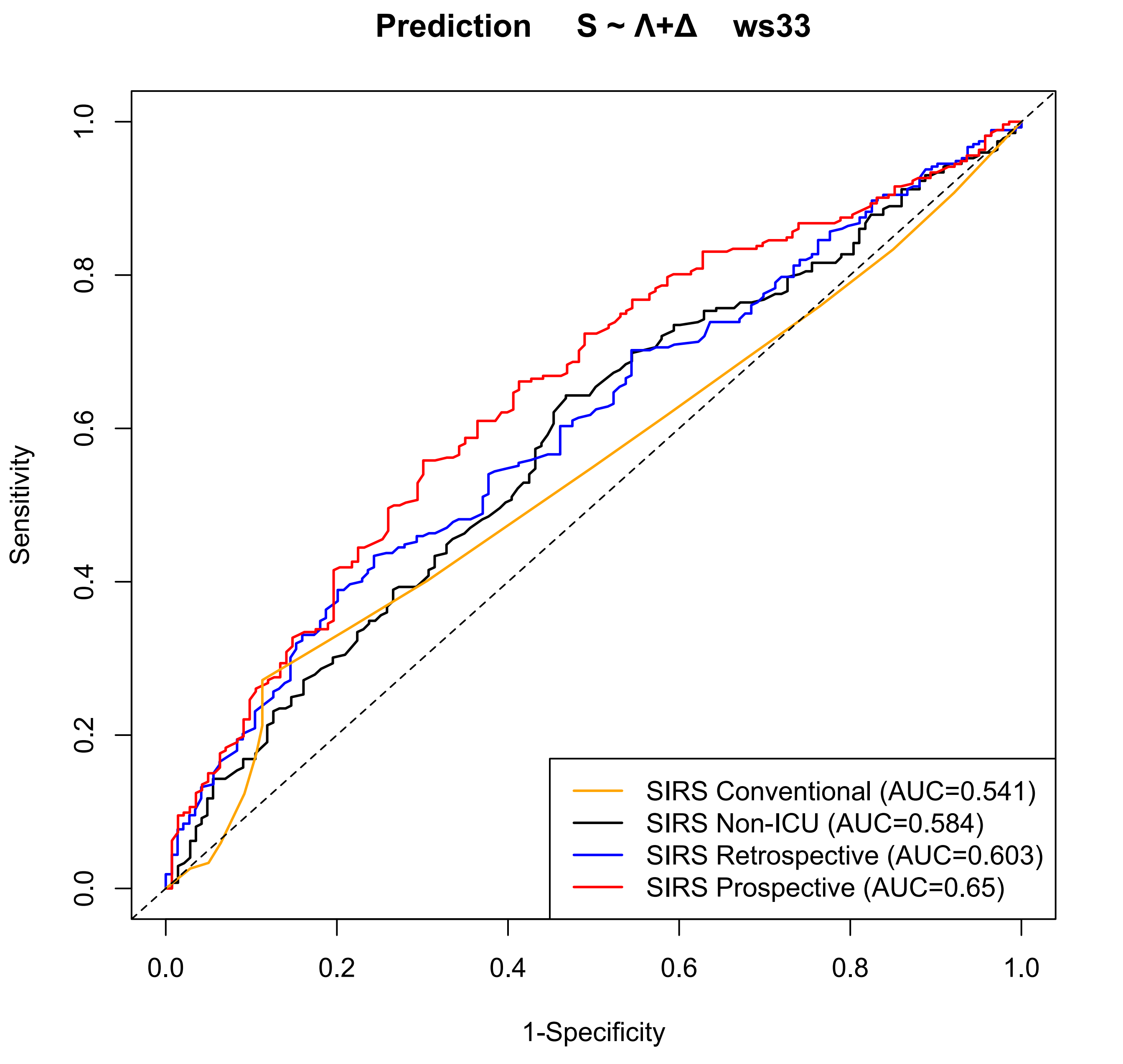
Prediction S ~ Λ+Δ ws33
Sensitivity
1-Specificity
0.0
0.2
0.4
0.6
0.8
1.0
0.0
0.2
0.4
0.6
0.8
1.0
SIRS Conventional (AUC=0.541)
SIRS Non-ICU (AUC=0.584)
SIRS Retrospective (AUC=0.603)
SIRS Prospective (AUC=0.65)

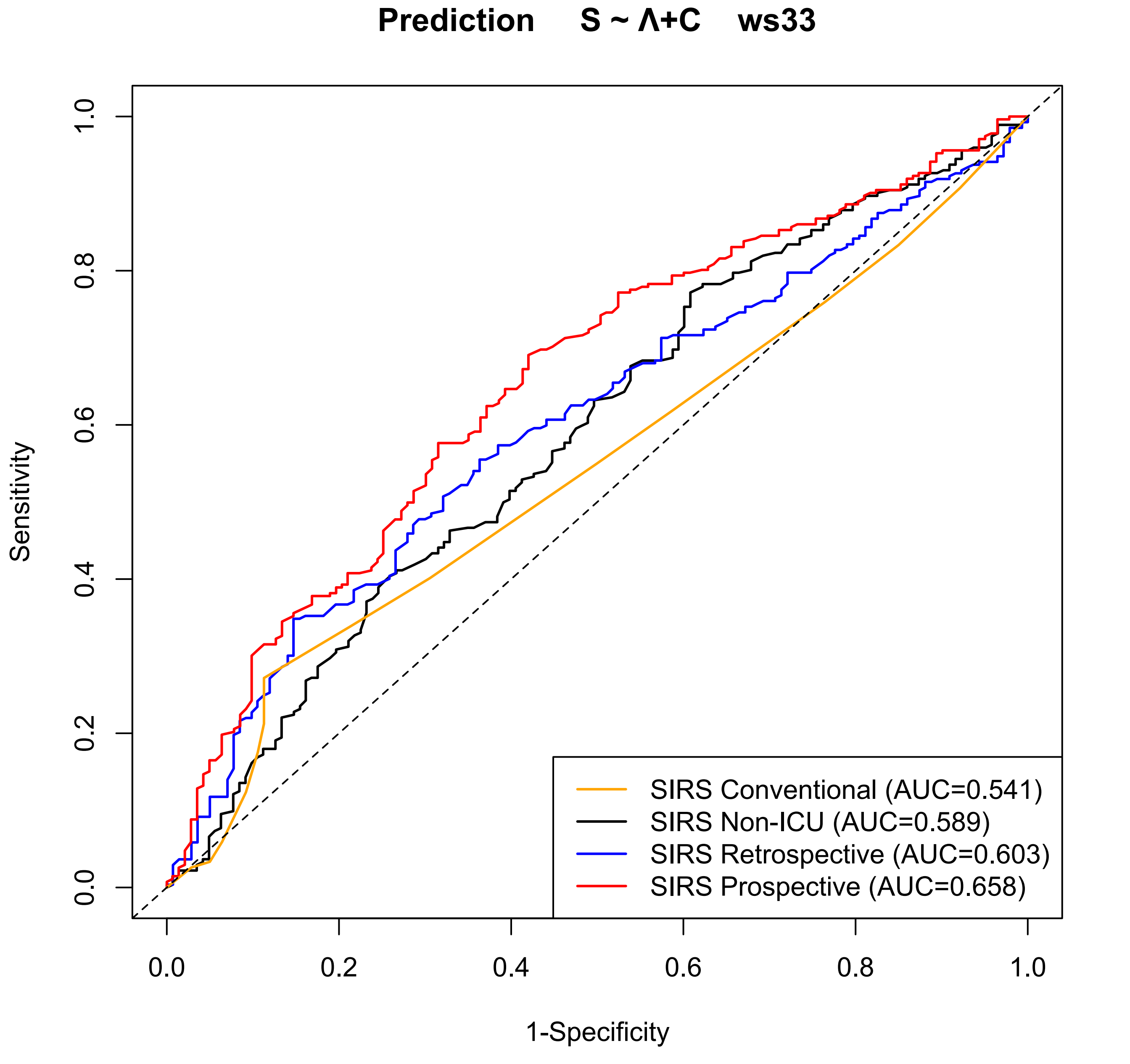

Prediction S ~ Λ+C ws33
Sensitivity
1-Specificity
0.0
0.2
0.4
0.6
0.8
1.0
0.0
0.2
0.4
0.6
0.8
1.0
SIRS Conventional (AUC=0.541)
SIRS Non-ICU (AUC=0.589)
SIRS Retrospective (AUC=0.603)
SIRS Prospective (AUC=0.658)

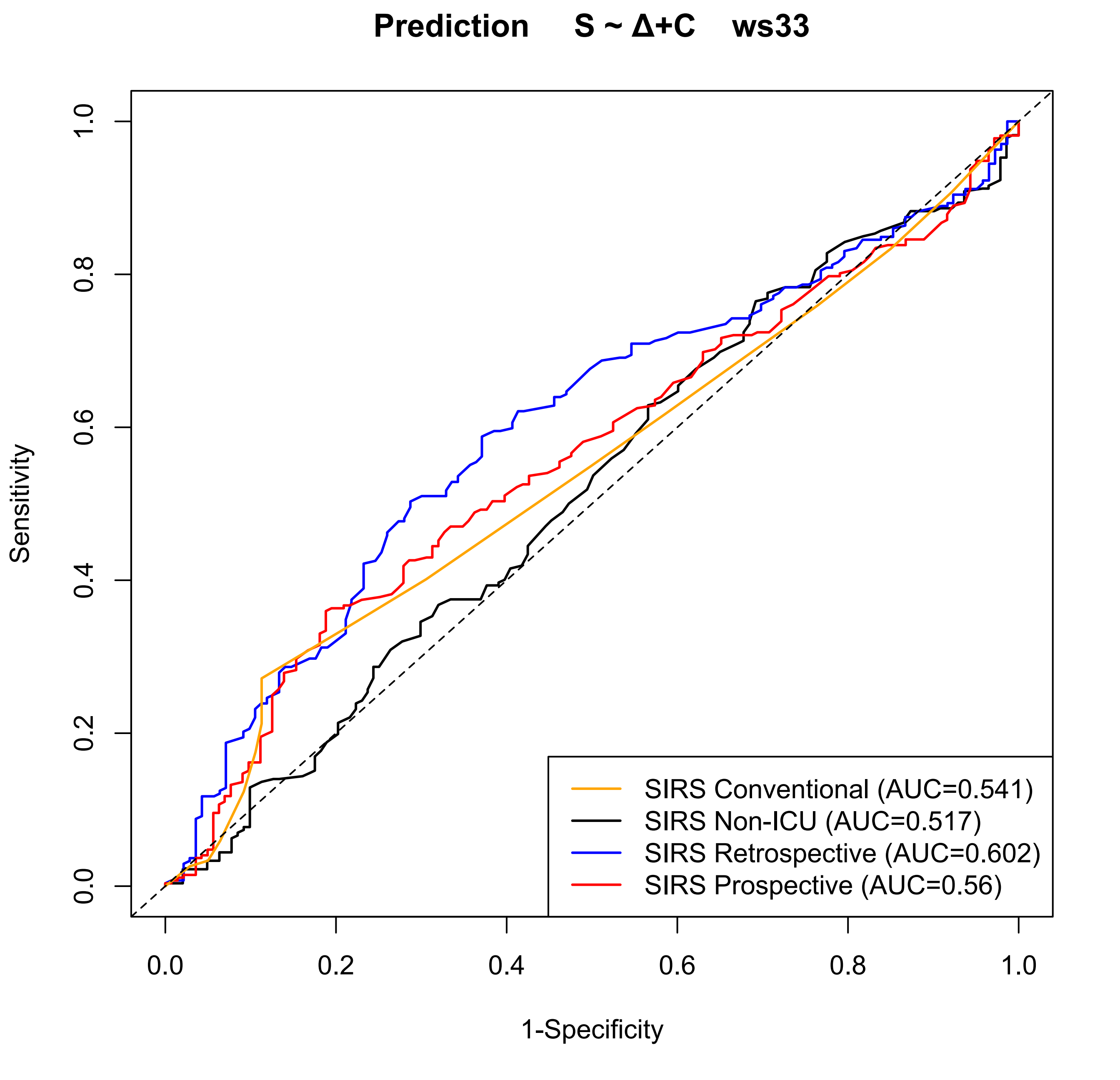

Prediction S ~ Δ+C ws33
Sensitivity
1-Specificity
0.0
0.2
0.4
0.6
0.8
1.0
0.0
0.2
0.4
0.6
0.8
1.0
SIRS Conventional (AUC=0.541)
SIRS Non-ICU (AUC=0.517)
SIRS Retrospective (AUC=0.602)
SIRS Prospective (AUC=0.56)

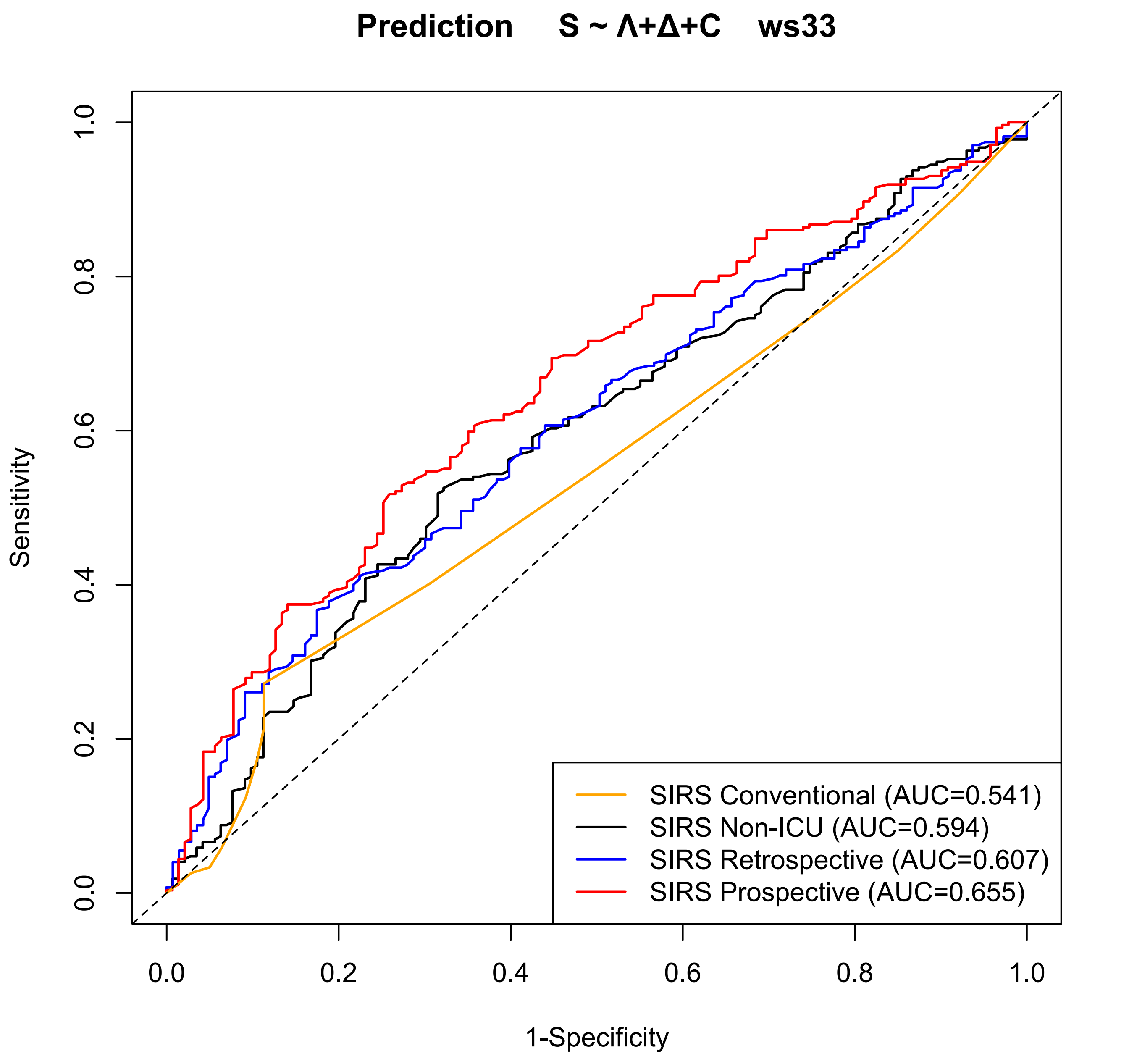

Prediction S ~ Λ+Δ+C ws33
Sensitivity
1-Specificity
0.0
0.2
0.4
0.6
0.8
1.0
0.0
0.2
0.4
0.6
0.8
1.0
SIRS Conventional (AUC=0.541)
SIRS Non-ICU (AUC=0.594)
SIRS Retrospective (AUC=0.607)
SIRS Prospective (AUC=0.655)

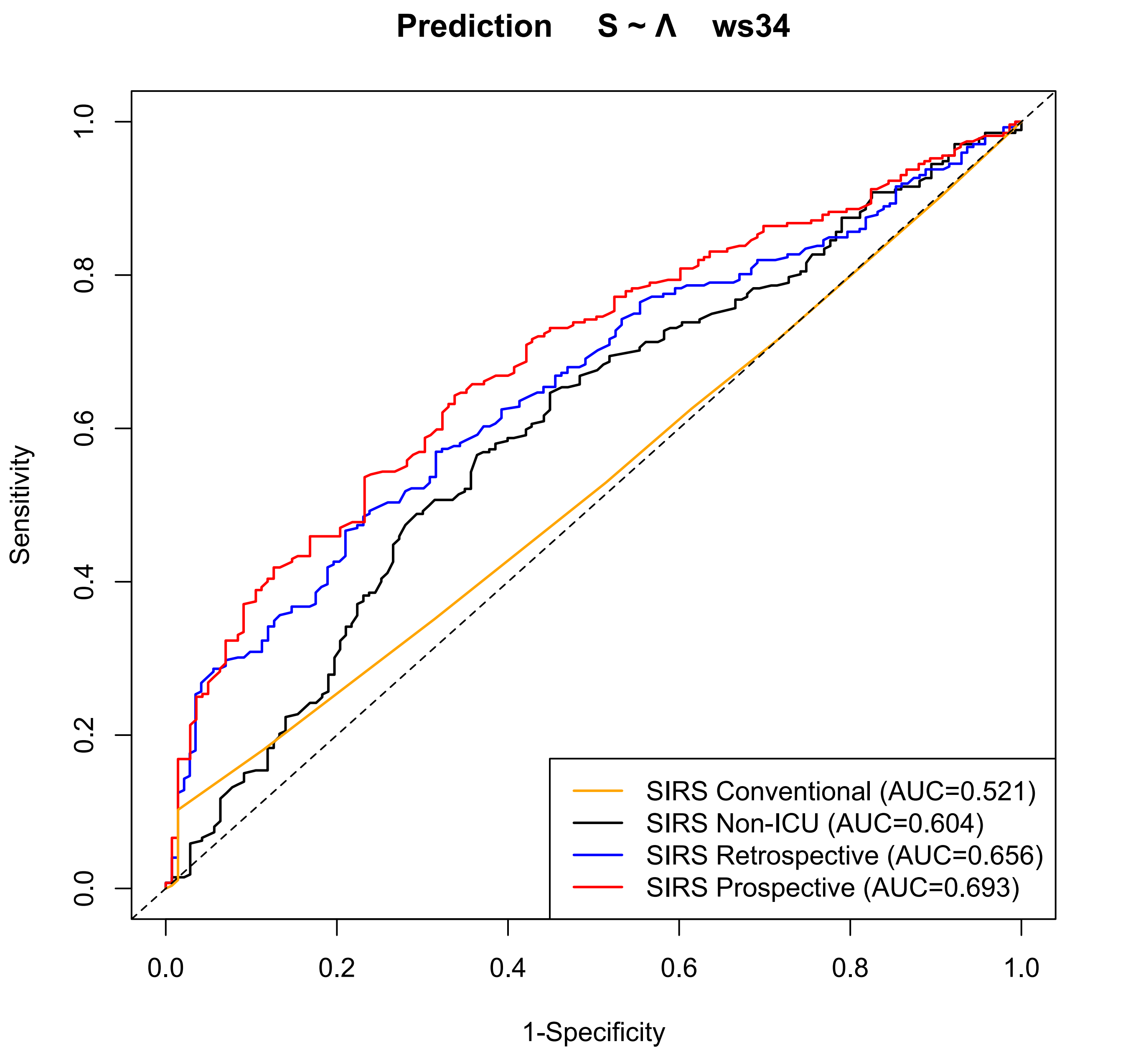
Prediction S ~ Λ ws34
Sensitivity
1-Specificity
0.0
0.2
0.4
0.6
0.8
1.0
SIRS Conventional (AUC=0.521)
SIRS Non-ICU (AUC=0.604)
SIRS Retrospective (AUC=0.656)
SIRS Prospective (AUC=0.693)

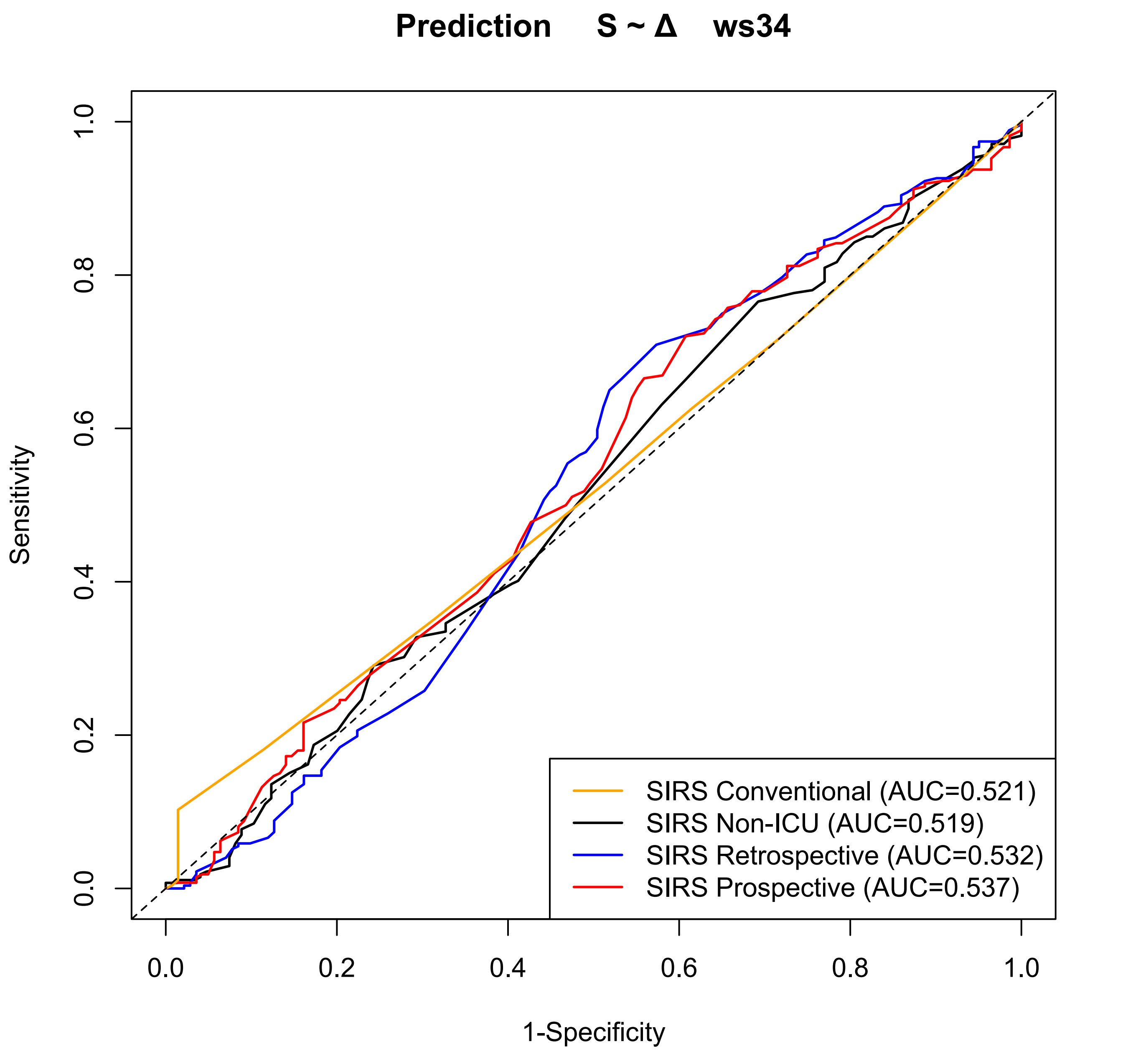
Prediction S ~ Δ ws34
Sensitivity
1-Specificity
0.0
0.2
0.4
0.6
0.8
1.0
SIRS Conventional (AUC=0.521)
SIRS Non-ICU (AUC=0.519)
SIRS Retrospective (AUC=0.532)
SIRS Prospective (AUC=0.537)

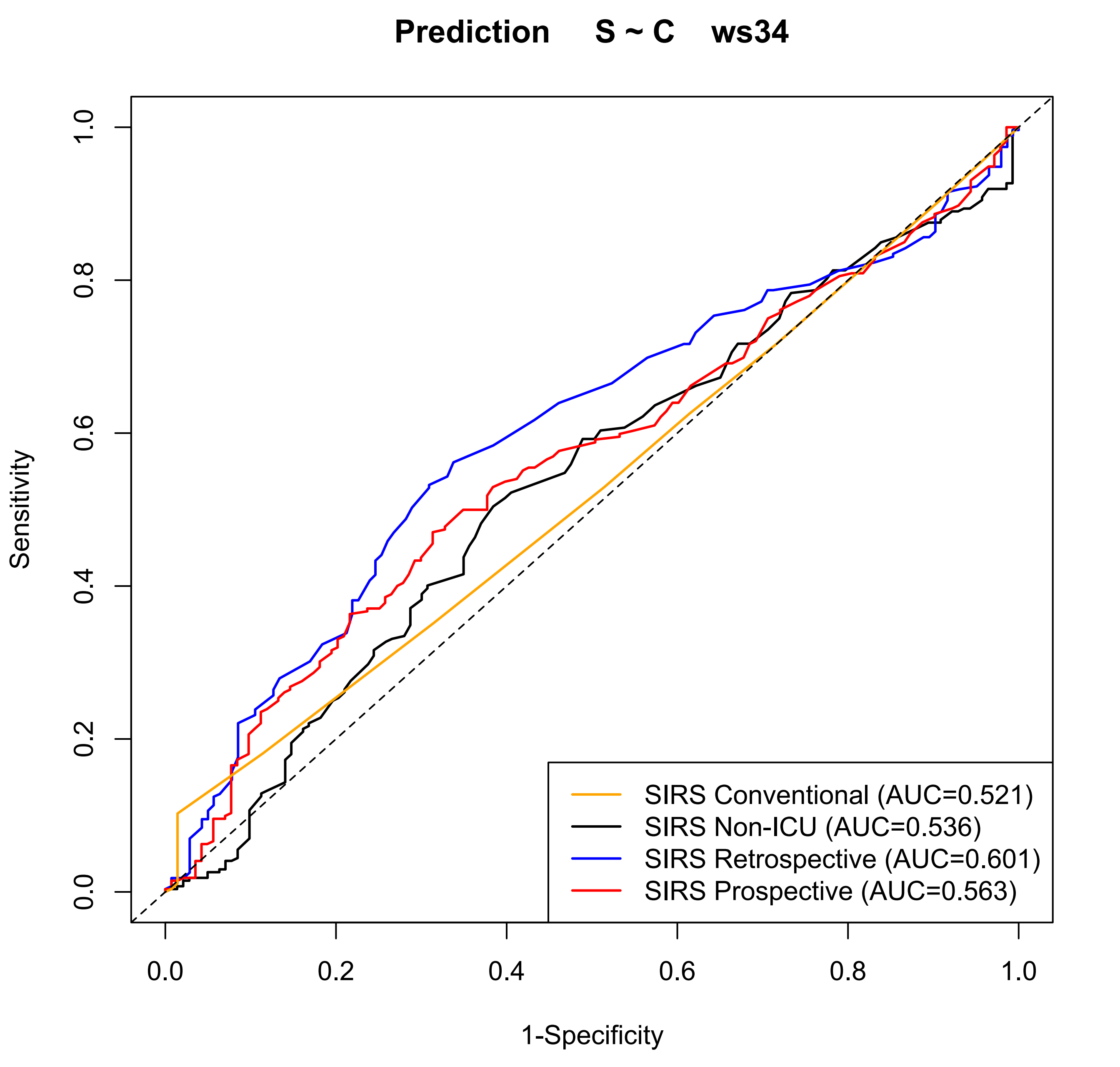

Prediction S ~ C ws34
Sensitivity
1-Specificity
0.0
0.2
0.4
0.6
0.8
1.0
0.0
0.2
0.4
0.6
0.8
1.0
SIRS Conventional (AUC=0.521)
SIRS Non-ICU (AUC=0.536)
SIRS Retrospective (AUC=0.601)
SIRS Prospective (AUC=0.563)

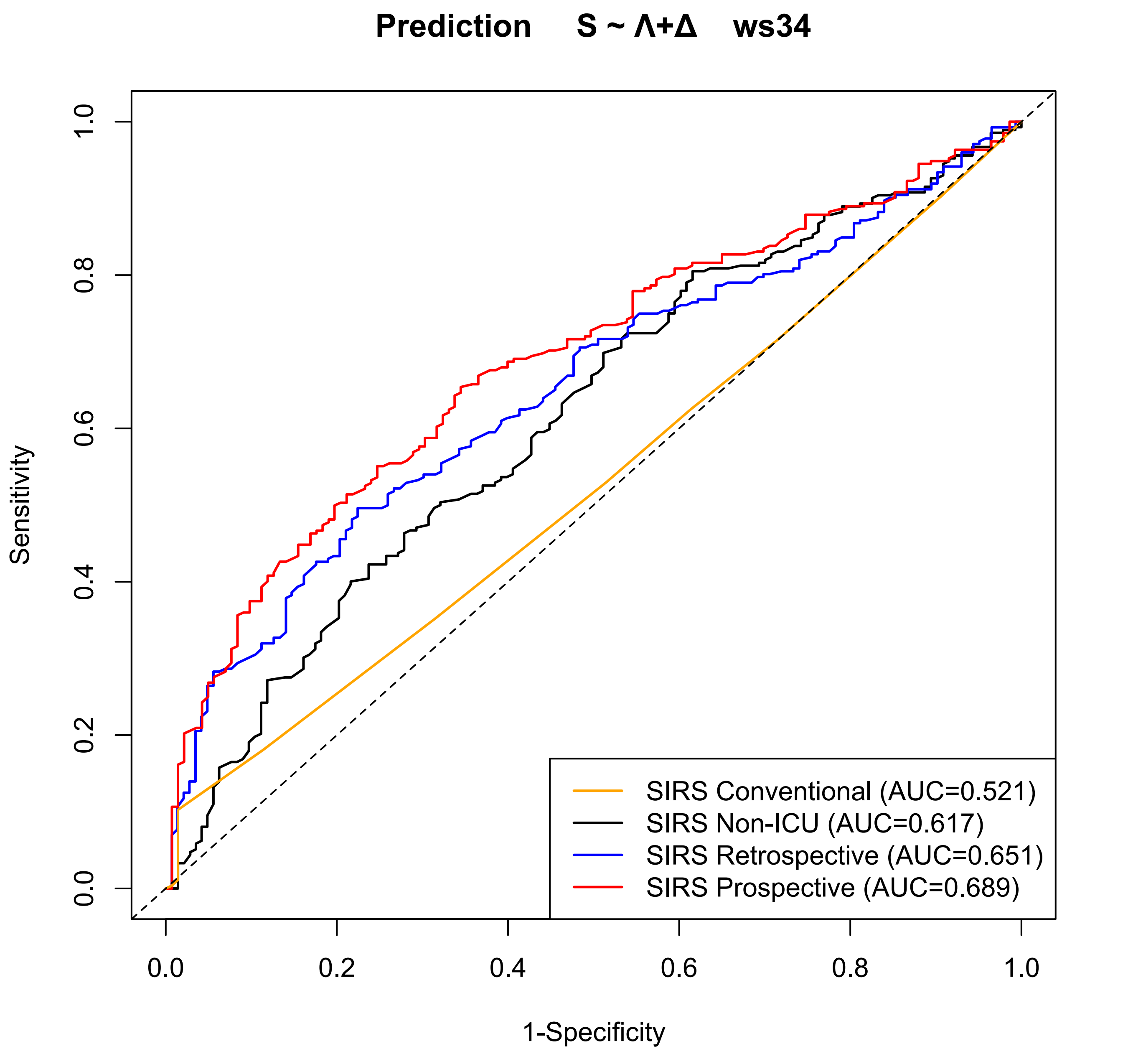

Prediction S ~ Λ+Δ ws34
Sensitivity
1-Specificity
0.0
0.2
0.4
0.6
0.8
1.0
0.0
0.2
0.4
0.6
0.8
1.0
SIRS Conventional (AUC=0.521)
SIRS Non-ICU (AUC=0.617)
SIRS Retrospective (AUC=0.651)
SIRS Prospective (AUC=0.689)

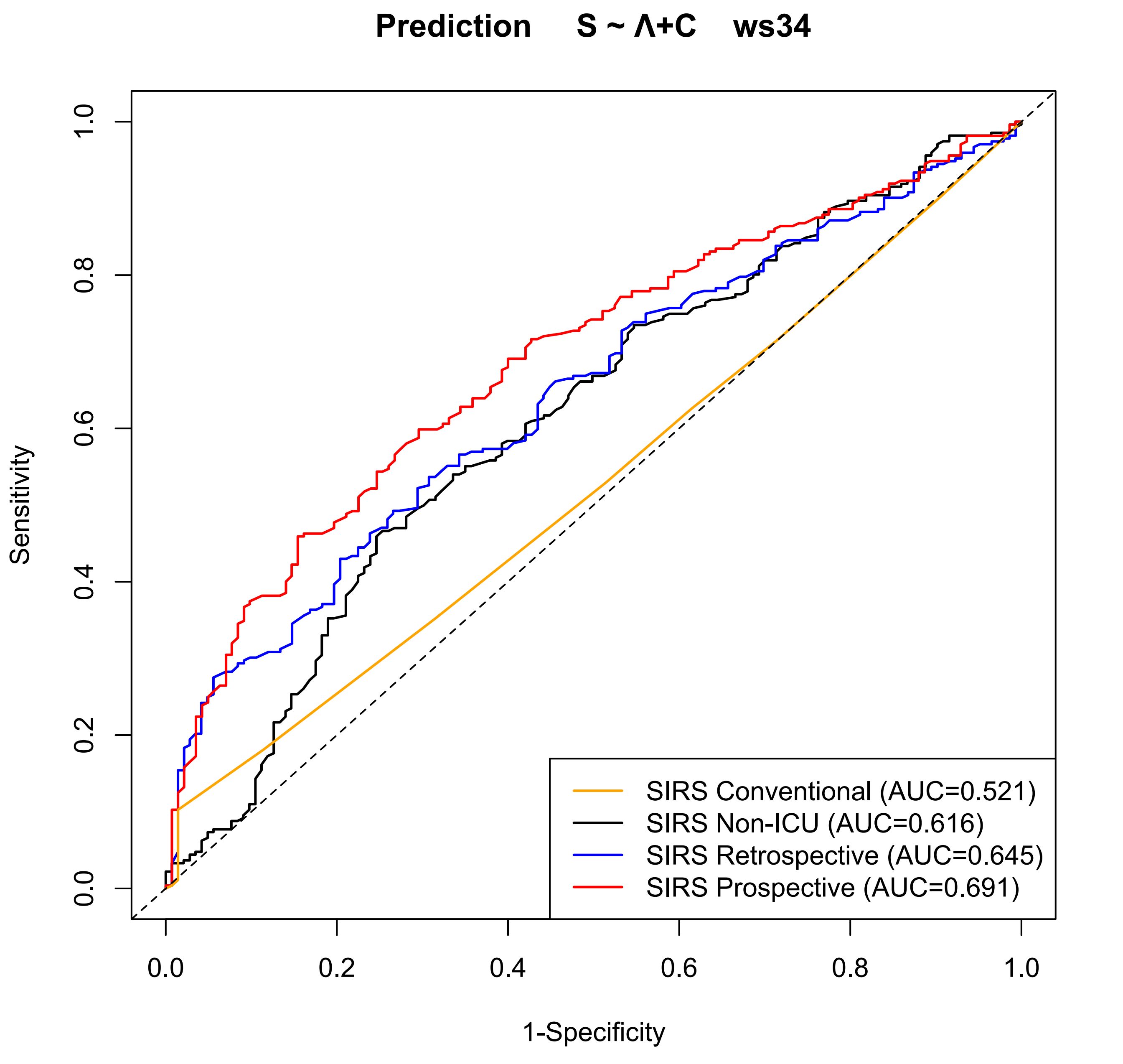

Prediction S ~ Λ+C ws34
Sensitivity
1-Specificity
0.0
0.2
0.4
0.6
0.8
1.0
SIRS Conventional (AUC=0.521)
SIRS Non-ICU (AUC=0.616)
SIRS Retrospective (AUC=0.645)
SIRS Prospective (AUC=0.691)

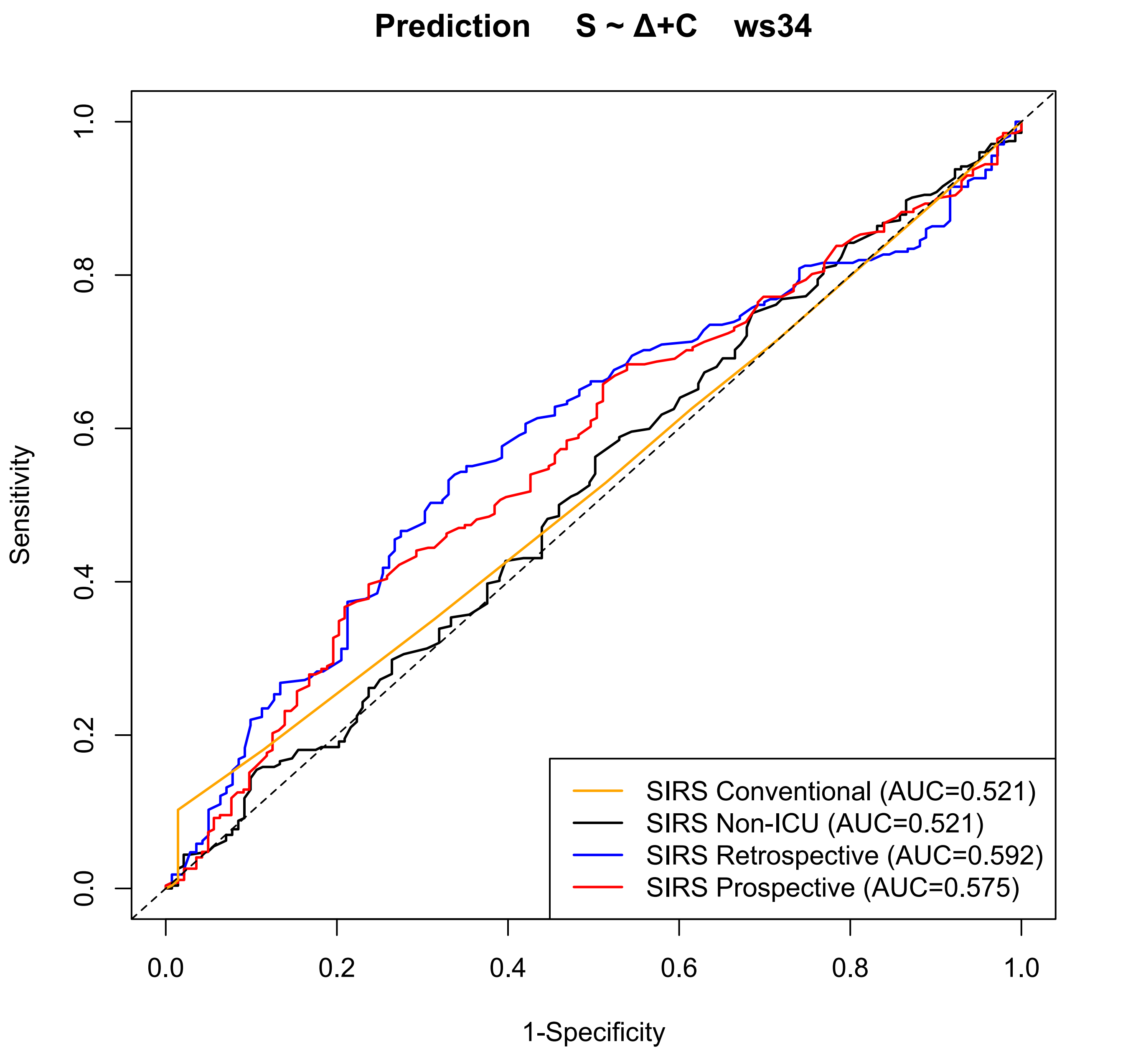
Prediction S ~ Δ+C ws34
Sensitivity
1-Specificity
0.0
0.2
0.4
0.6
0.8
1.0
0.0
0.2
0.4
0.6
0.8
1.0
SIRS Conventional (AUC=0.521)
SIRS Non-ICU (AUC=0.521)
SIRS Retrospective (AUC=0.592)
SIRS Prospective (AUC=0.575)

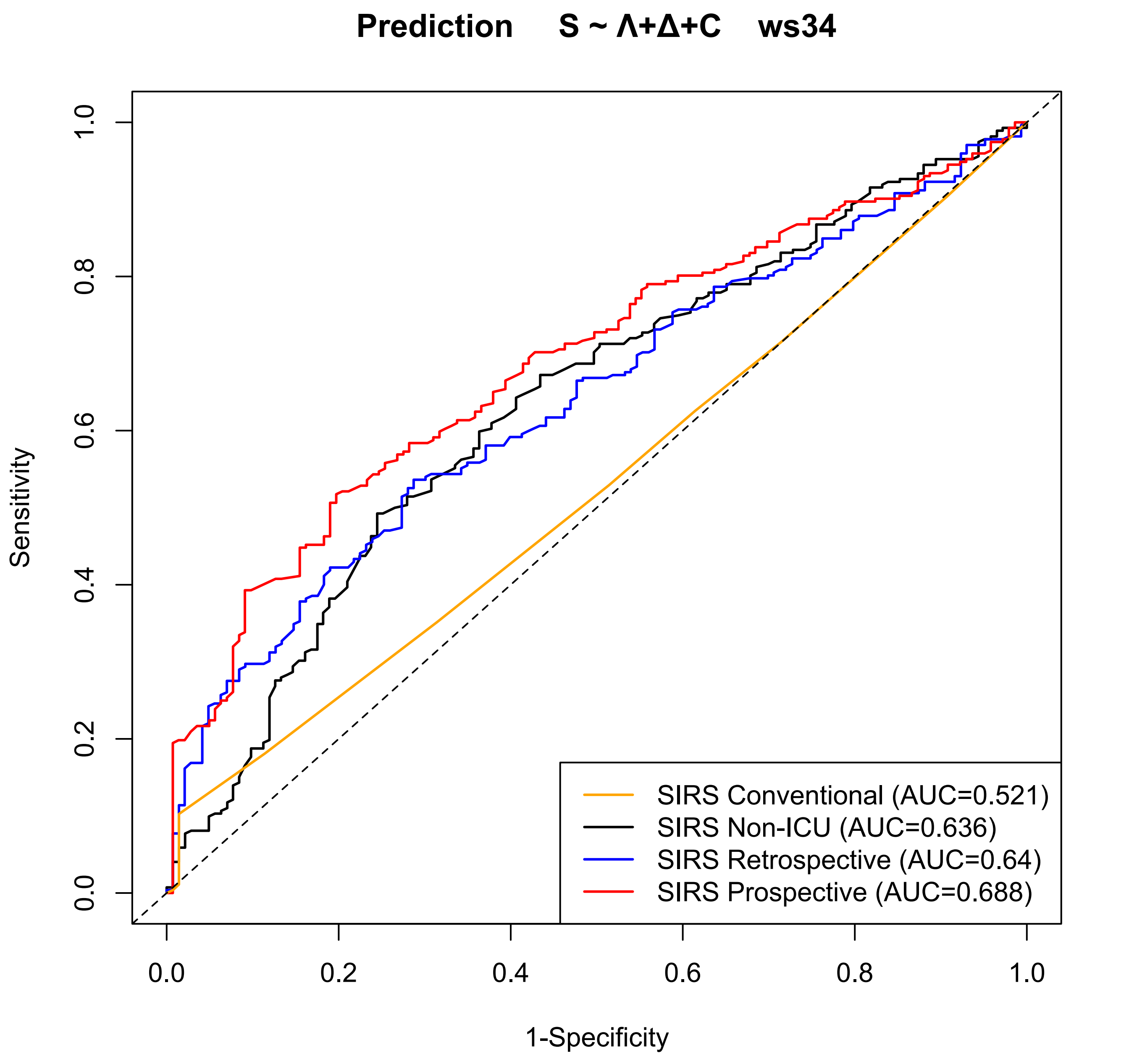

Prediction S ~ Λ+Δ+C ws34
Sensitivity
1-Specificity
0.0
0.2
0.4
0.6
0.8
1.0
SIRS Conventional (AUC=0.521)
SIRS Non-ICU (AUC=0.636)
SIRS Retrospective (AUC=0.64)
SIRS Prospective (AUC=0.688)

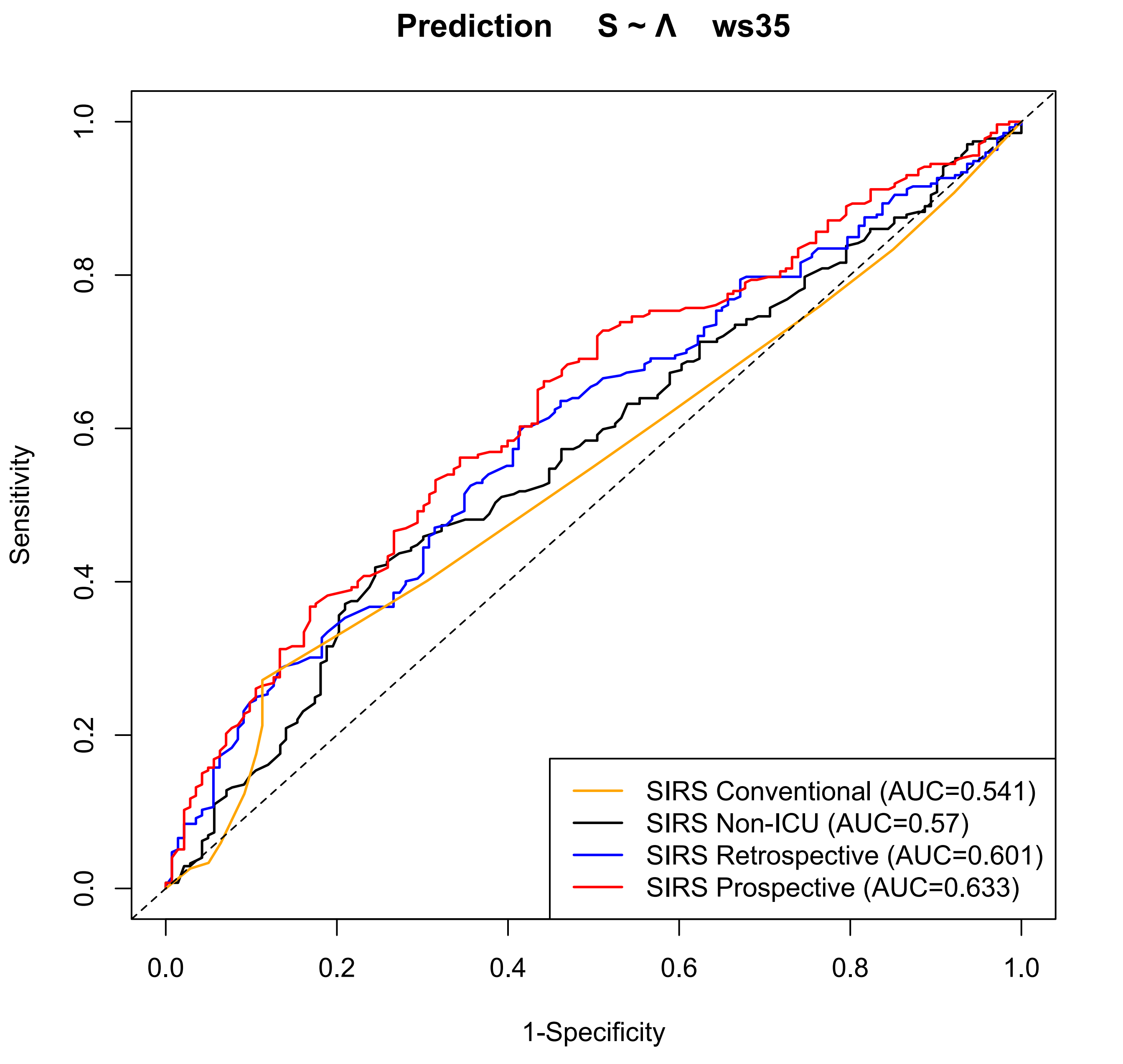

Prediction S ~ Λ ws35
Sensitivity
1-Specificity
0.0
0.2
0.4
0.6
0.8
1.0
SIRS Conventional (AUC=0.541)
SIRS Non-ICU (AUC=0.57)
SIRS Retrospective (AUC=0.601)
SIRS Prospective (AUC=0.633)

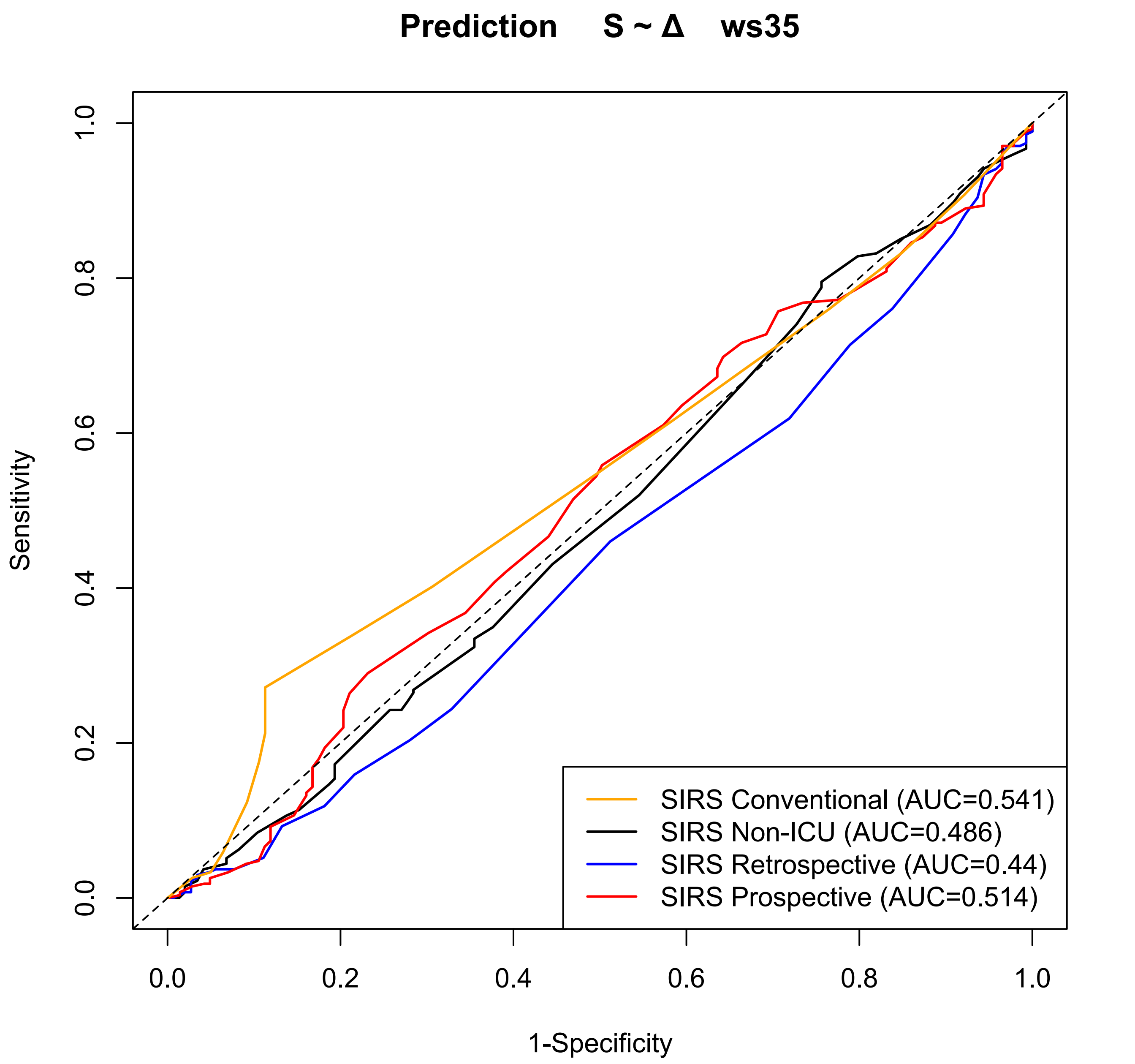
Prediction S ~ Δ ws35
Sensitivity
1-Specificity
0.0
0.2
0.4
0.6
0.8
1.0
SIRS Conventional (AUC=0.541)
SIRS Non-ICU (AUC=0.486)
SIRS Retrospective (AUC=0.44)
SIRS Prospective (AUC=0.514)

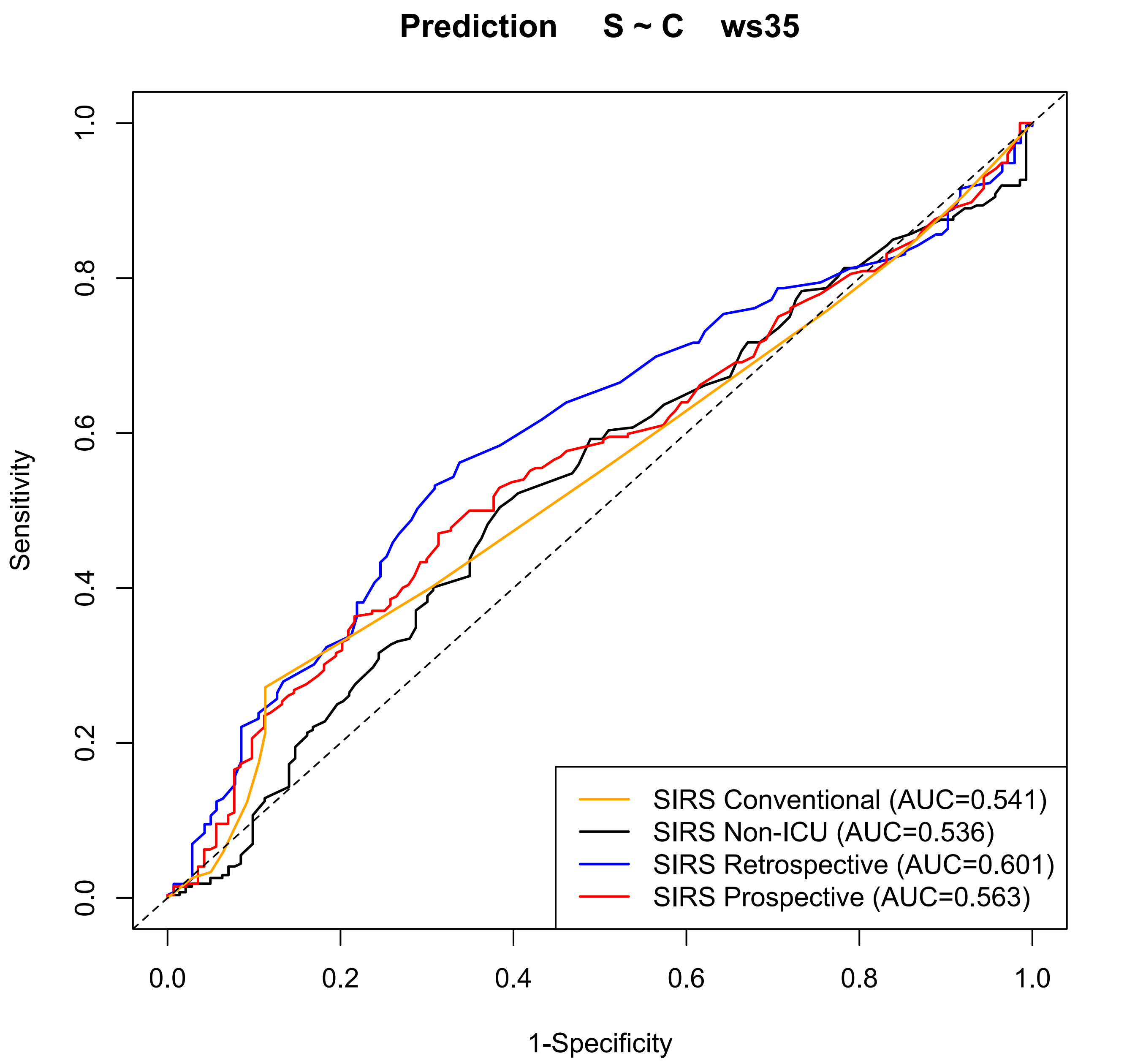

Prediction S ~ C ws35
Sensitivity
1-Specificity
0.0
0.2
0.4
0.6
0.8
1.0
SIRS Conventional (AUC=0.541)
SIRS Non-ICU (AUC=0.536)
SIRS Retrospective (AUC=0.601)
SIRS Prospective (AUC=0.563)

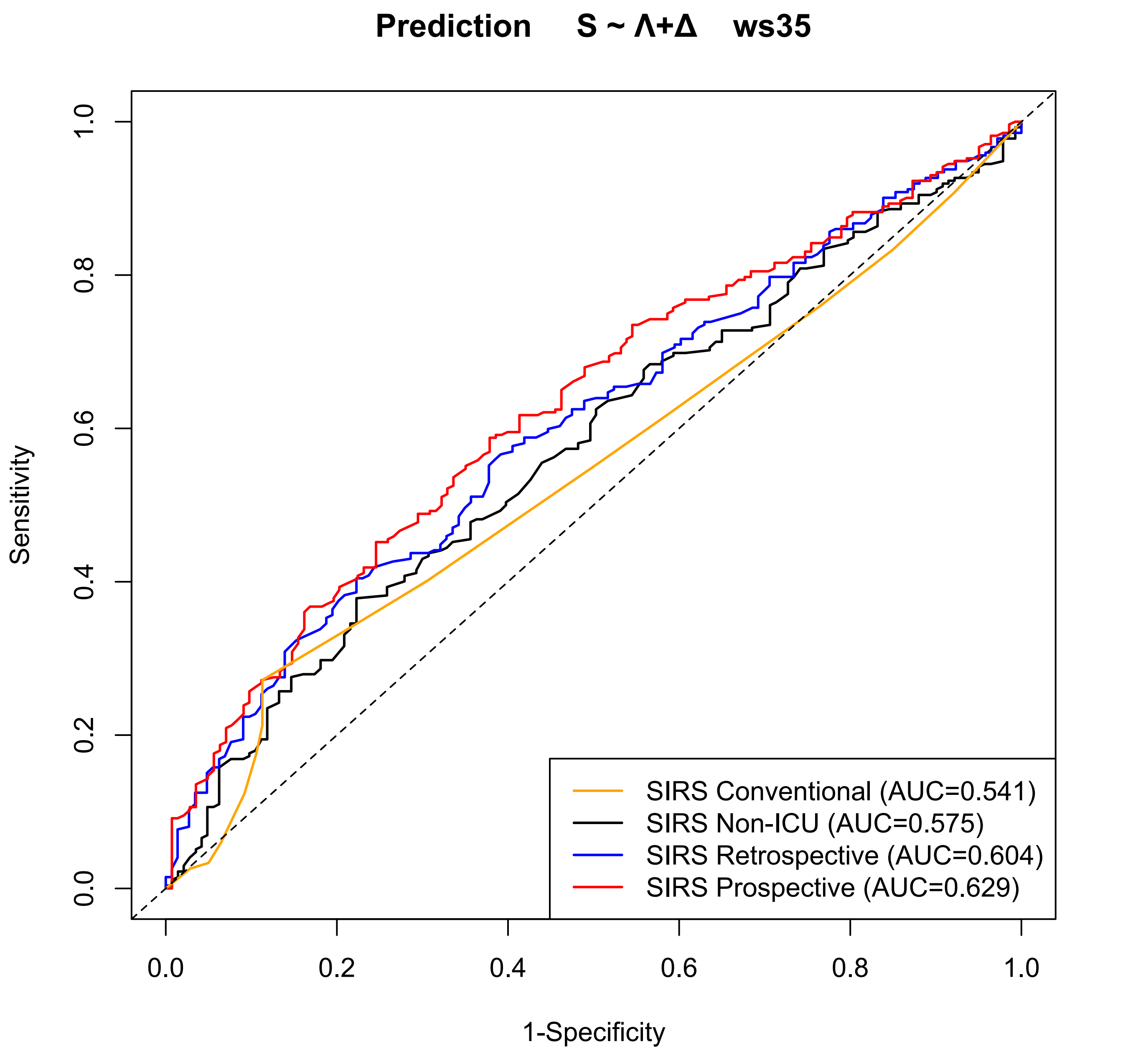

Prediction S ~ Λ+Δ ws35
Sensitivity
1-Specificity
0.0
0.2
0.4
0.6
0.8
1.0
SIRS Conventional (AUC=0.541)
SIRS Non-ICU (AUC=0.575)
SIRS Retrospective (AUC=0.604)
SIRS Prospective (AUC=0.629)

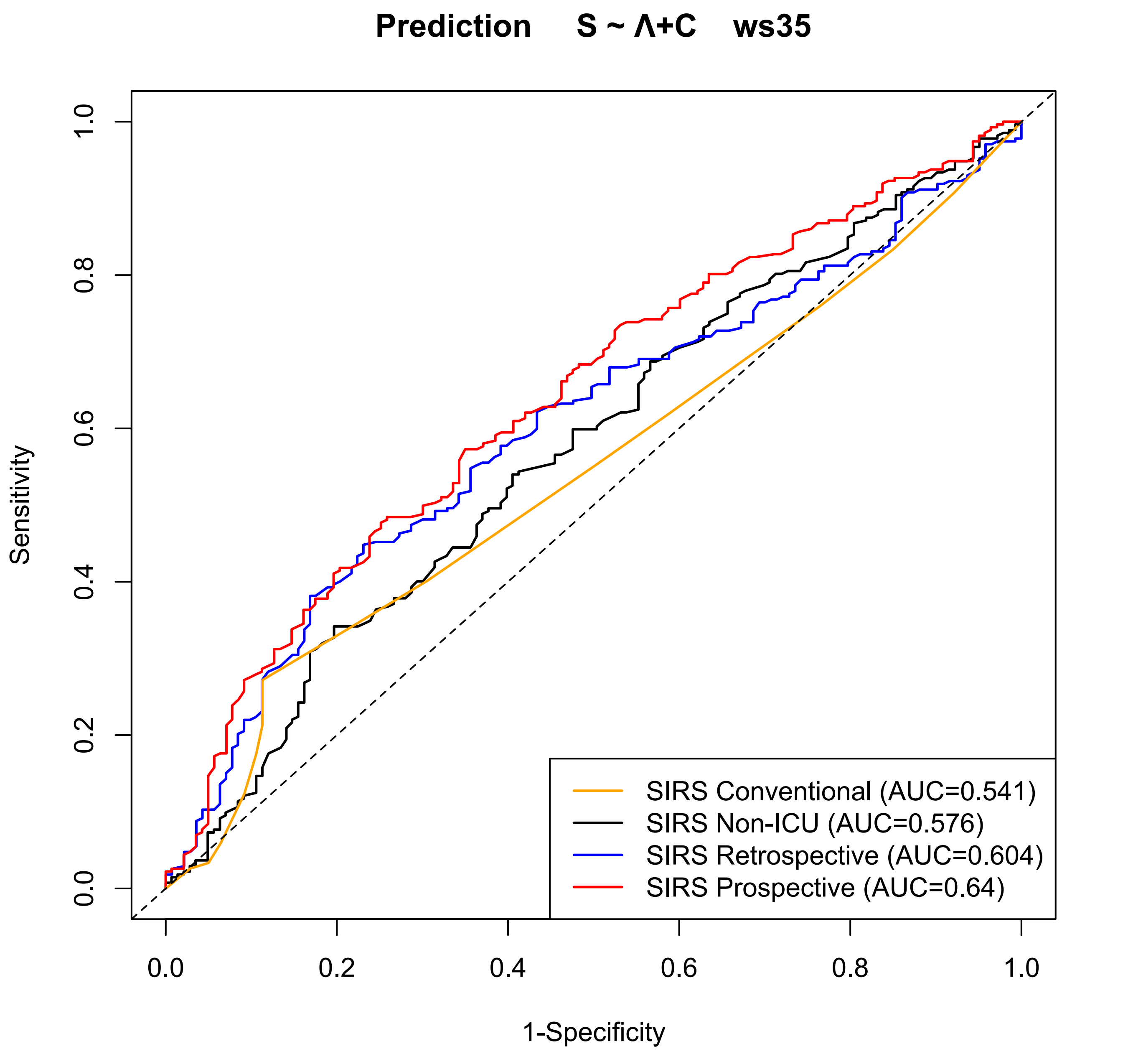

Prediction S ~ Λ+C ws35
Sensitivity
1-Specificity
0.0
0.2
0.4
0.6
0.8
1.0
SIRS Conventional (AUC=0.541)
SIRS Non-ICU (AUC=0.576)
SIRS Retrospective (AUC=0.604)
SIRS Prospective (AUC=0.64)

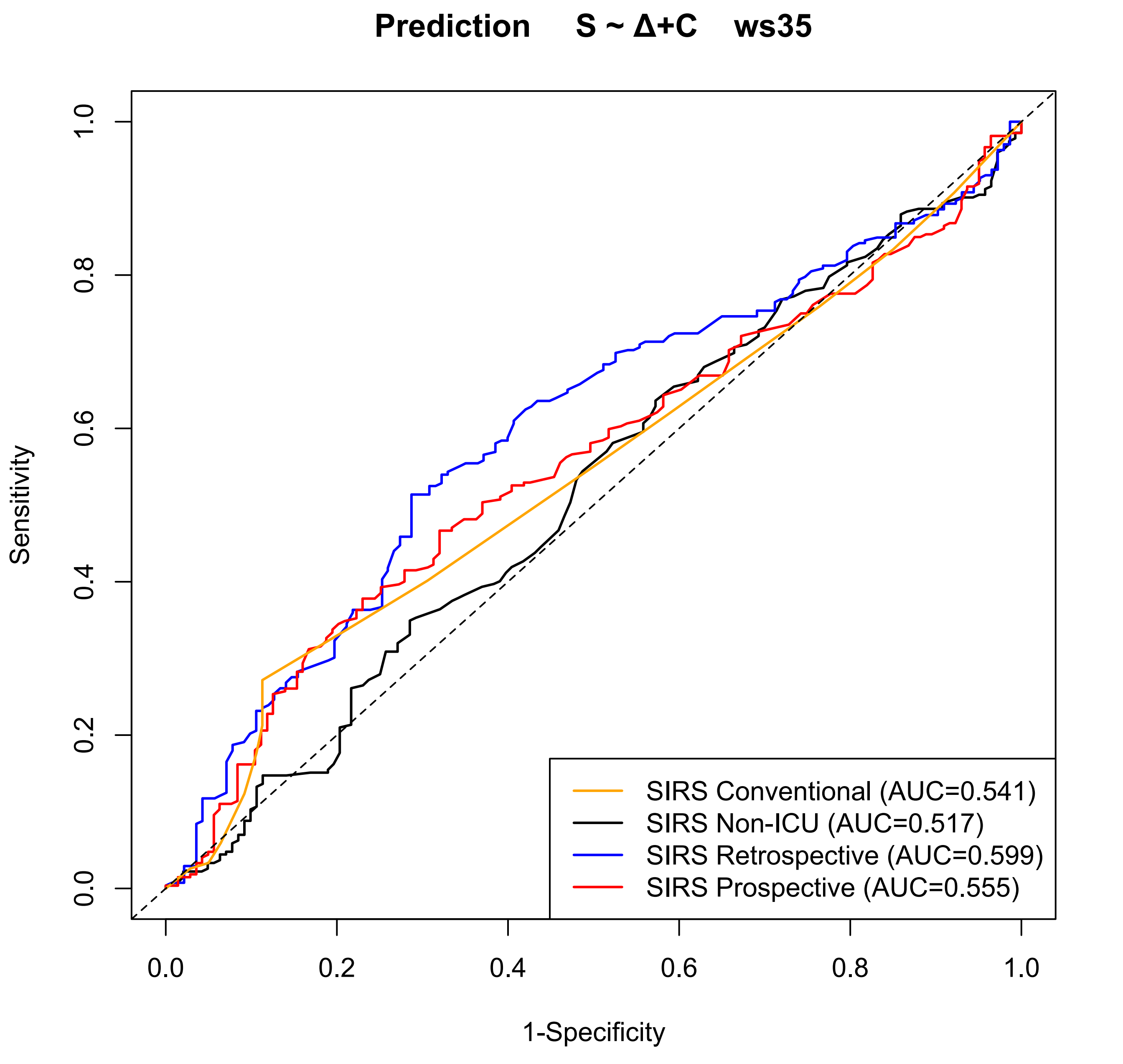

Prediction S ~ Δ+C ws35
Sensitivity
1-Specificity
0.0
0.2
0.4
0.6
0.8
1.0
SIRS Conventional (AUC=0.541)
SIRS Non-ICU (AUC=0.517)
SIRS Retrospective (AUC=0.599)
SIRS Prospective (AUC=0.555)

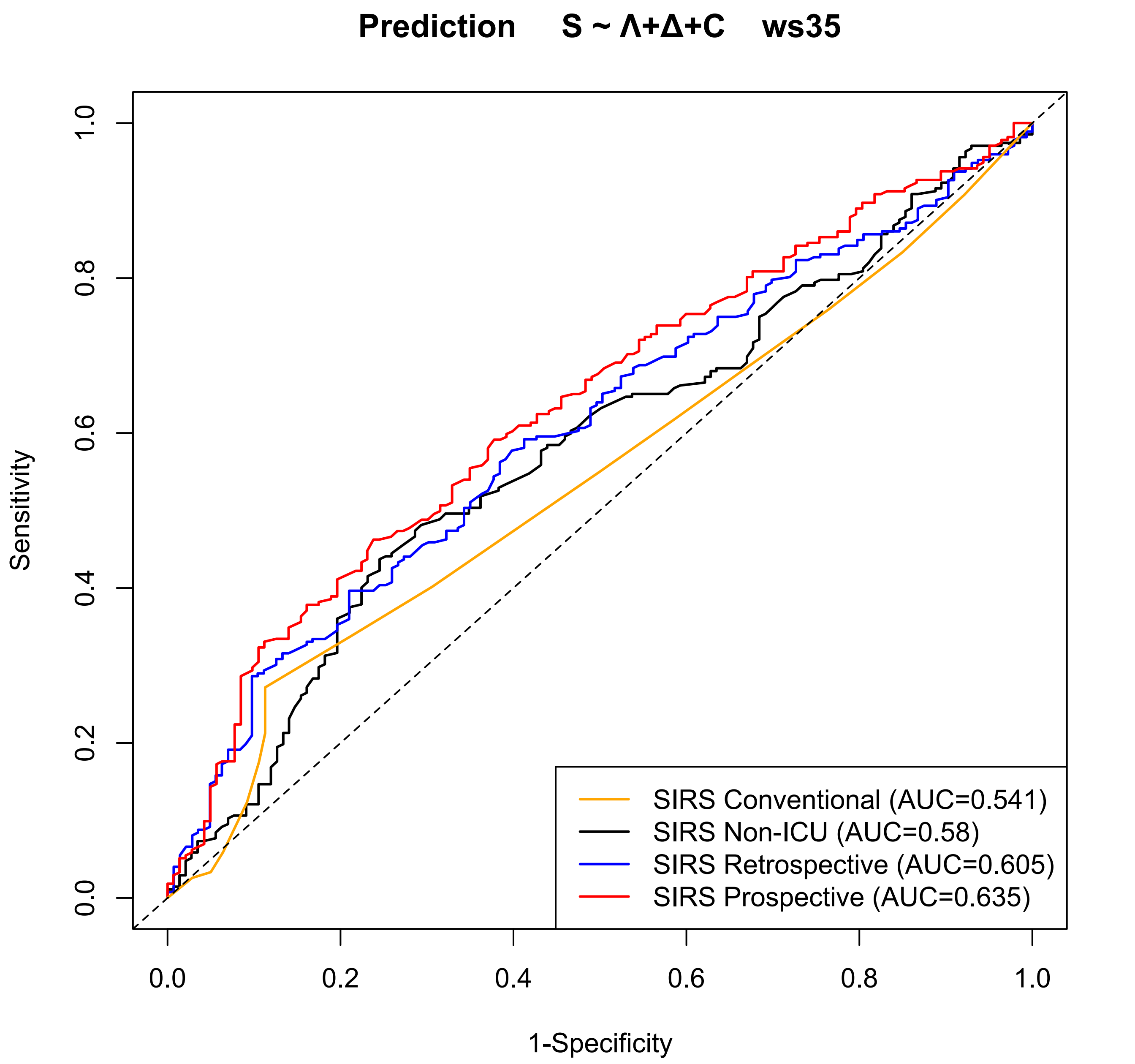
Prediction S ~ Λ+Δ+C ws35
Sensitivity
1-Specificity
0.0
0.2
0.4
0.6
0.8
1.0
SIRS Conventional (AUC=0.541)
SIRS Non-ICU (AUC=0.58)
SIRS Retrospective (AUC=0.605)
SIRS Prospective (AUC=0.635)

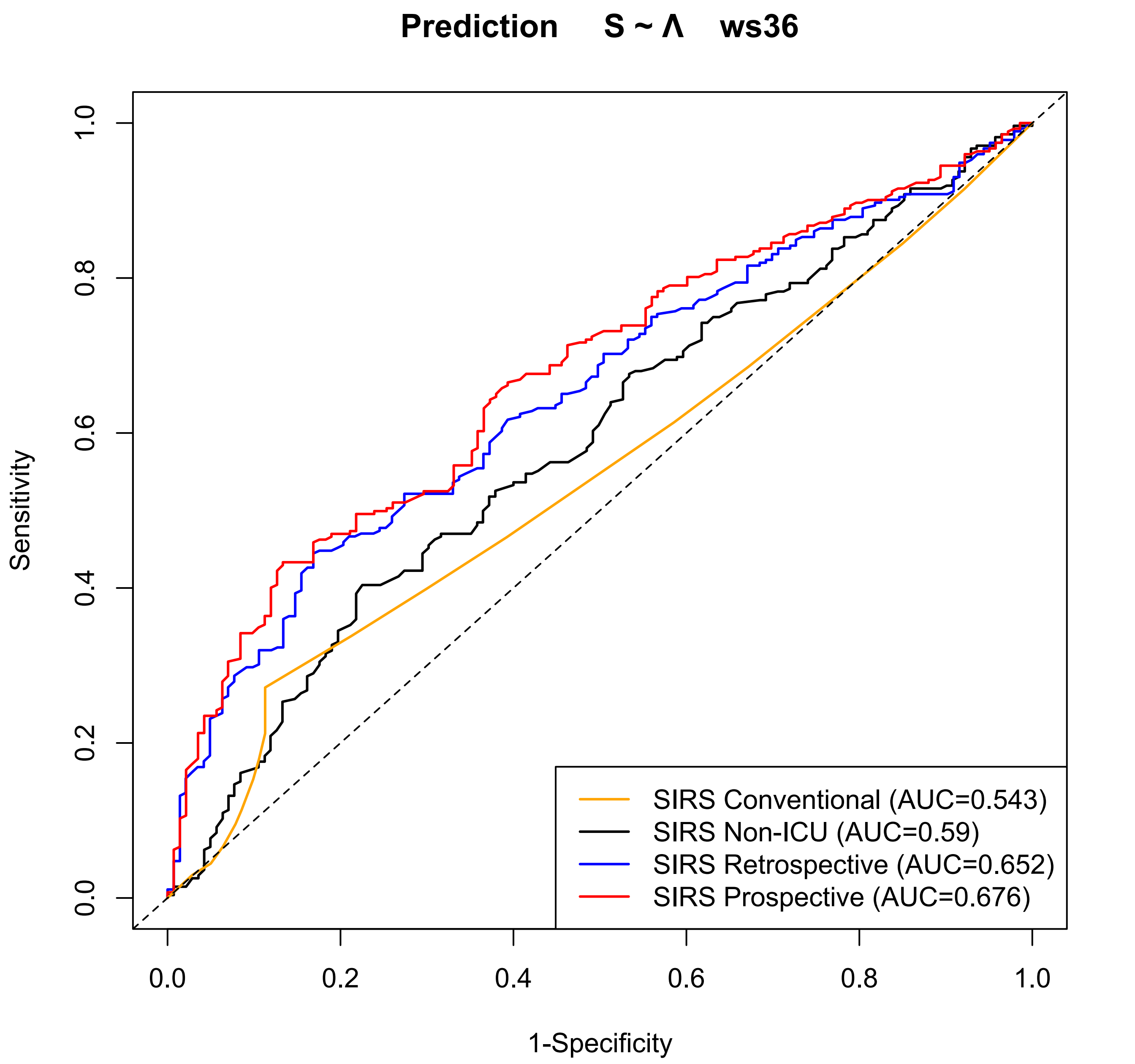
Prediction S ~ Λ ws36
Sensitivity
1-Specificity
0.0
0.2
0.4
0.6
0.8
1.0
0.0
0.2
0.4
0.6
0.8
1.0
SIRS Conventional (AUC=0.543)
SIRS Non-ICU (AUC=0.59)
SIRS Retrospective (AUC=0.652)
SIRS Prospective (AUC=0.676)

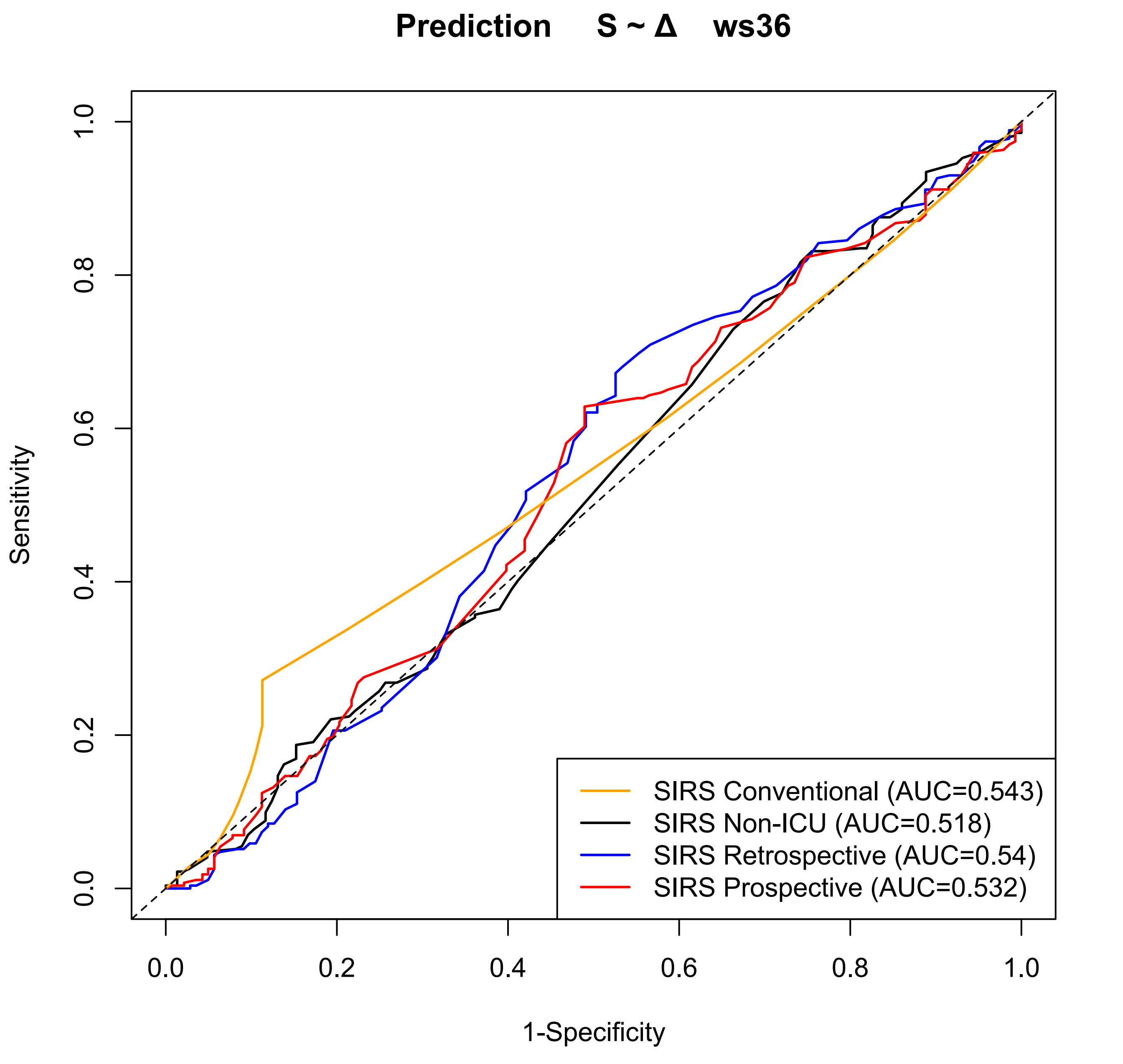

Prediction S ~ Δ ws36
Sensitivity
1-Specificity
0.0
0.2
0.4
0.6
0.8
1.0
SIRS Conventional (AUC=0.543)
SIRS Non-ICU (AUC=0.518)
SIRS Retrospective (AUC=0.54)
SIRS Prospective (AUC=0.532)

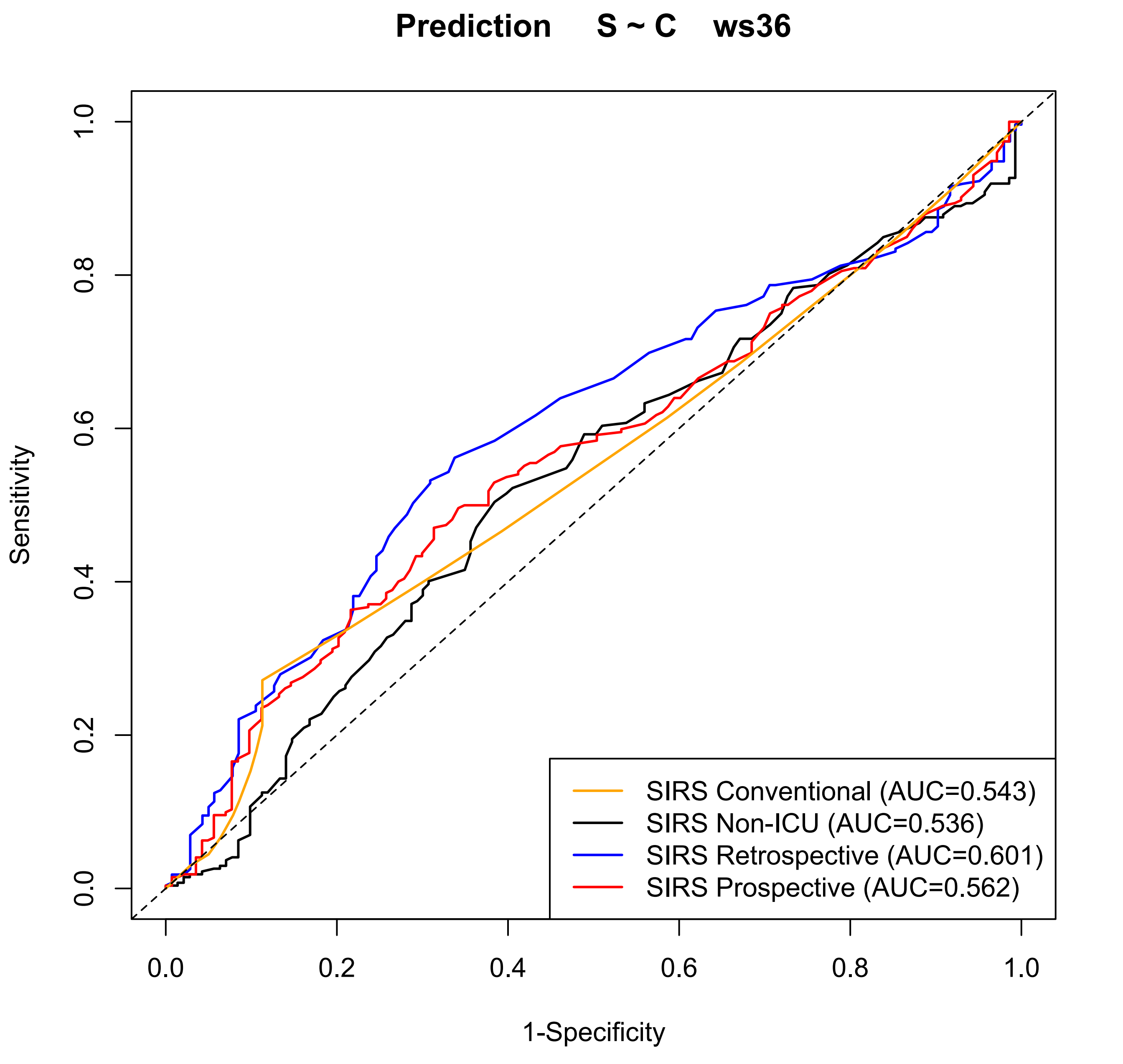
Prediction S ~ C ws36
Sensitivity
1-Specificity
0.0
0.2
0.4
0.6
0.8
1.0
SIRS Conventional (AUC=0.543)
SIRS Non-ICU (AUC=0.536)
SIRS Retrospective (AUC=0.601)
SIRS Prospective (AUC=0.562)

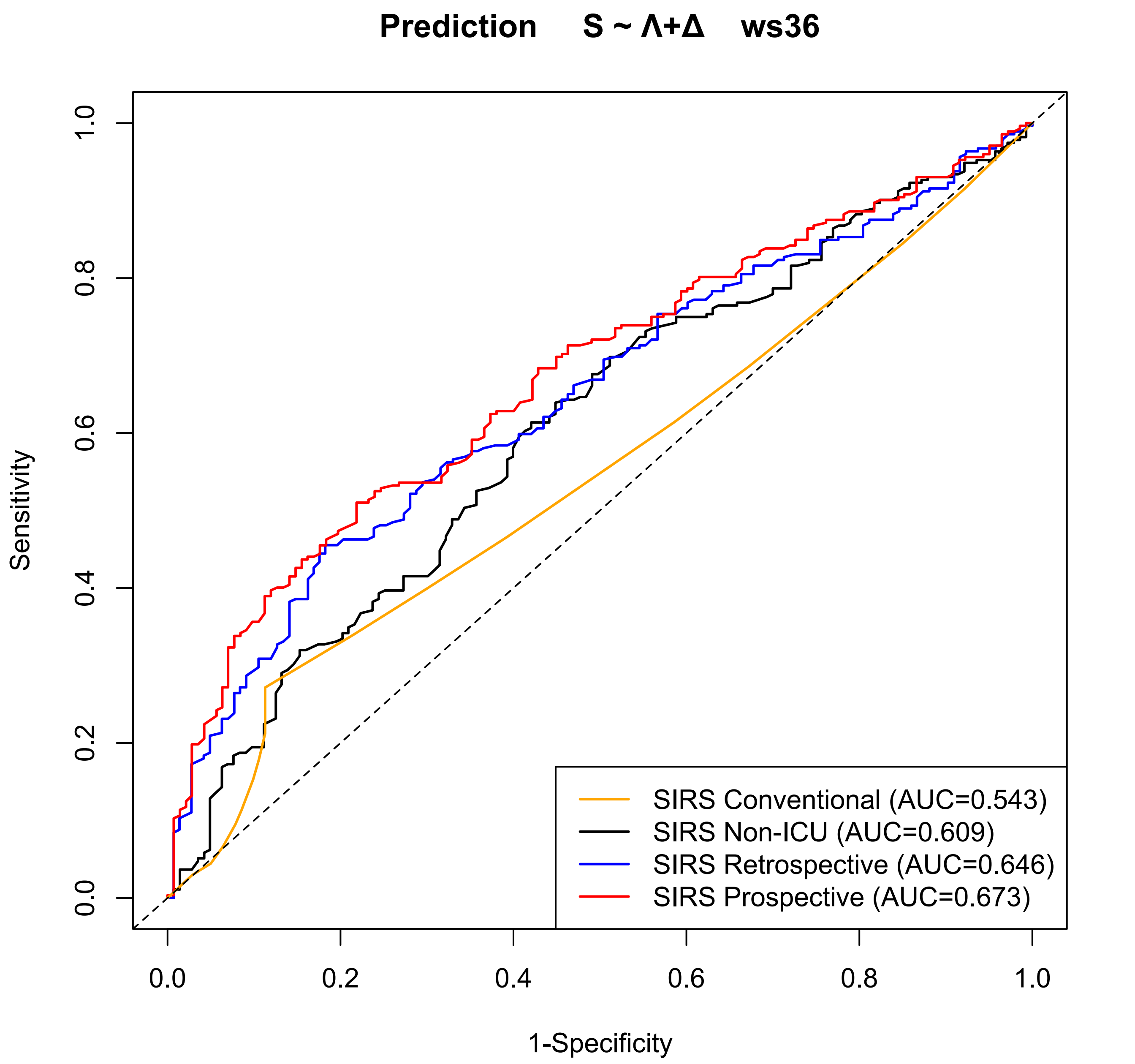
Prediction S ~ Λ+Δ ws36
Sensitivity
1-Specificity
0.0
0.2
0.4
0.6
0.8
1.0
0.0
0.2
0.4
0.6
0.8
1.0
SIRS Conventional (AUC=0.543)
SIRS Non-ICU (AUC=0.609)
SIRS Retrospective (AUC=0.646)
SIRS Prospective (AUC=0.673)

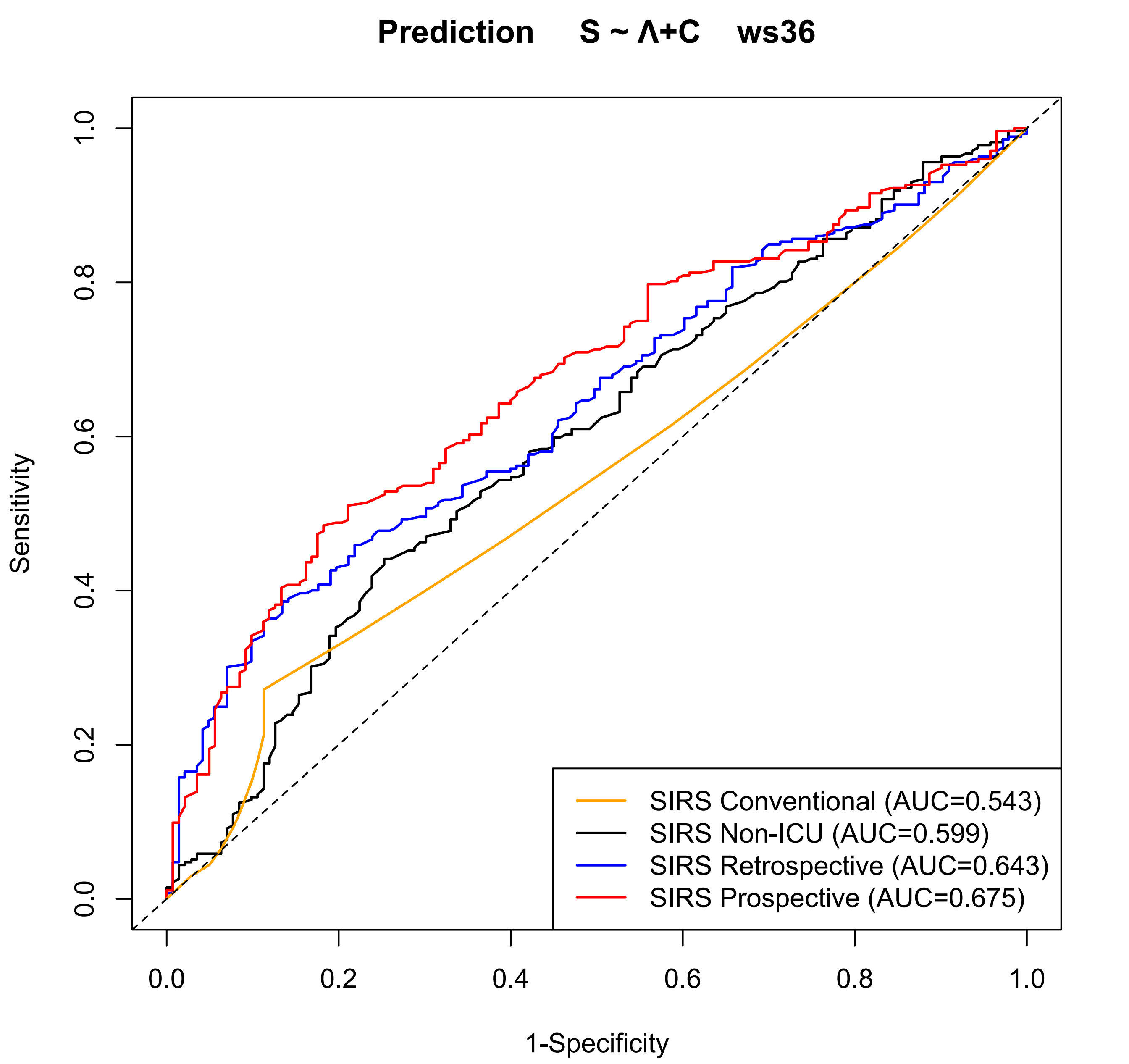

Prediction S ~ Λ+C ws36
Sensitivity
1-Specificity
0.0
0.2
0.4
0.6
0.8
1.0
0.0
0.2
0.4
0.6
0.8
1.0
SIRS Conventional (AUC=0.543)
SIRS Non-ICU (AUC=0.599)
SIRS Retrospective (AUC=0.643)
SIRS Prospective (AUC=0.675)

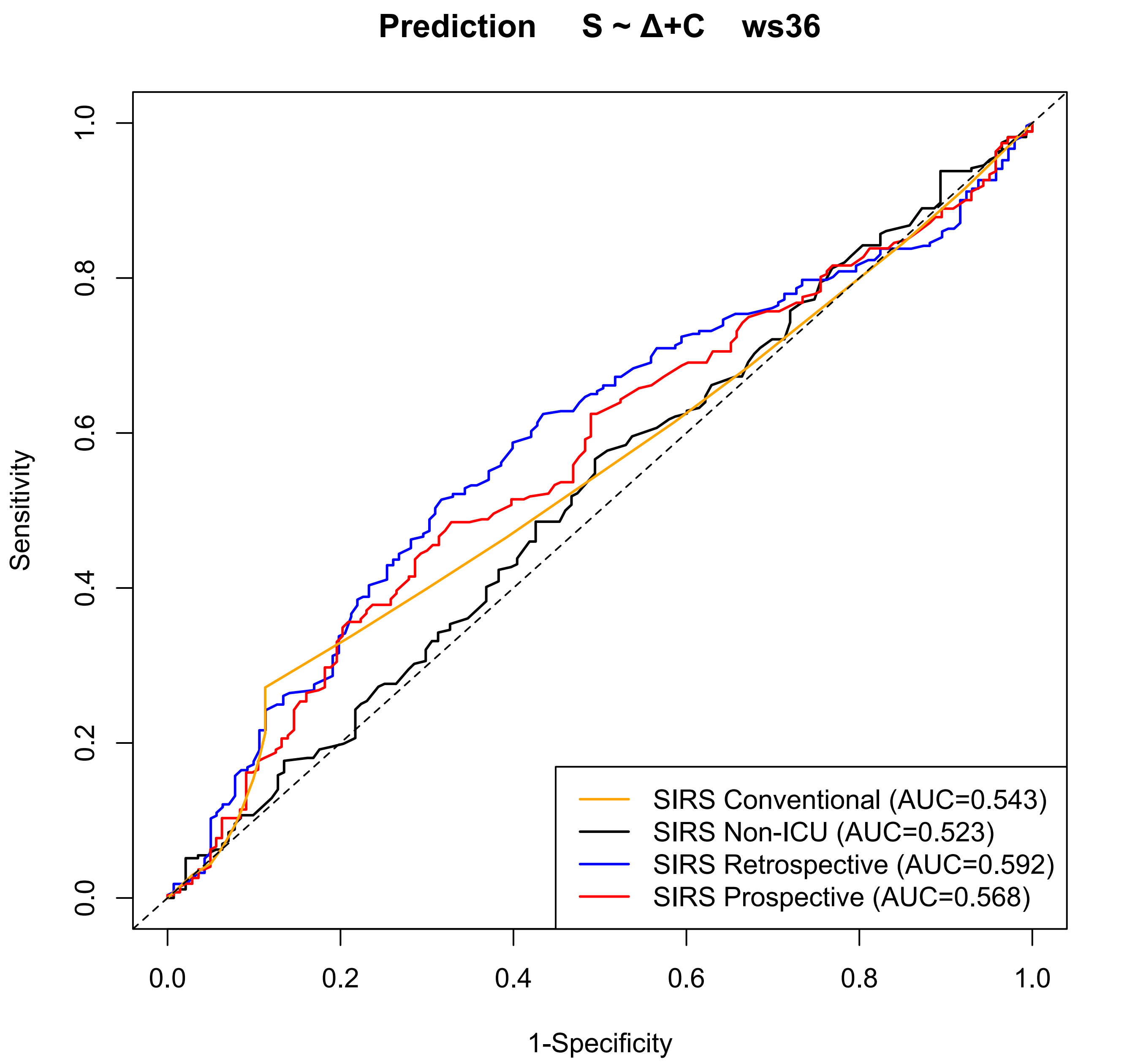

Prediction S ~ Δ+C ws36
Sensitivity
1-Specificity
0.0
0.2
0.4
0.6
0.8
1.0
SIRS Conventional (AUC=0.543)
SIRS Non-ICU (AUC=0.523)
SIRS Retrospective (AUC=0.592)
SIRS Prospective (AUC=0.568)

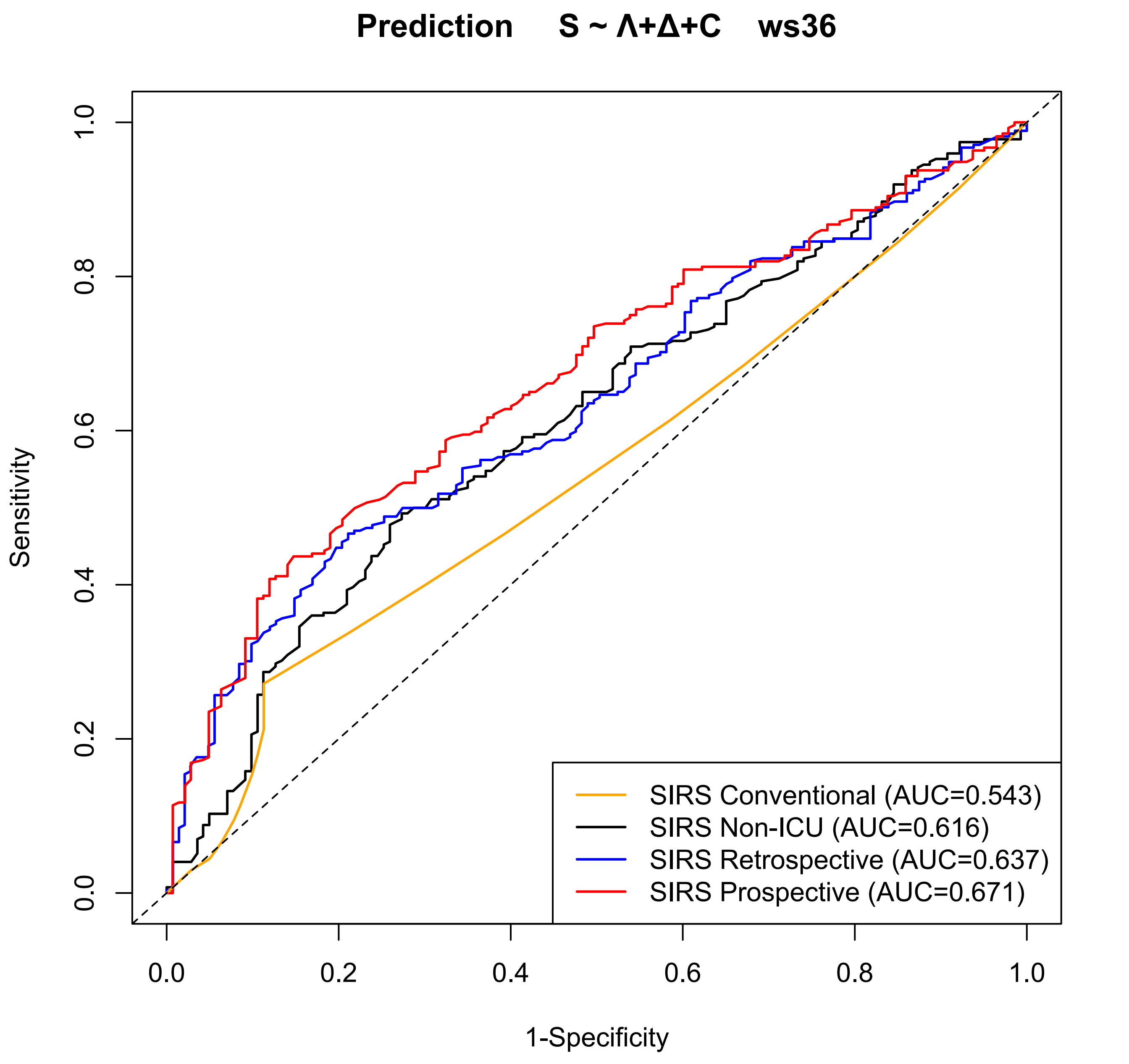

Prediction S ~ Λ+Δ+C ws36
Sensitivity
1-Specificity
0.0
0.2
0.4
0.6
0.8
1.0
0.0
0.2
0.4
0.6
0.8
1.0
SIRS Conventional (AUC=0.543)
SIRS Non-ICU (AUC=0.616)
SIRS Retrospective (AUC=0.637)
SIRS Prospective (AUC=0.671)

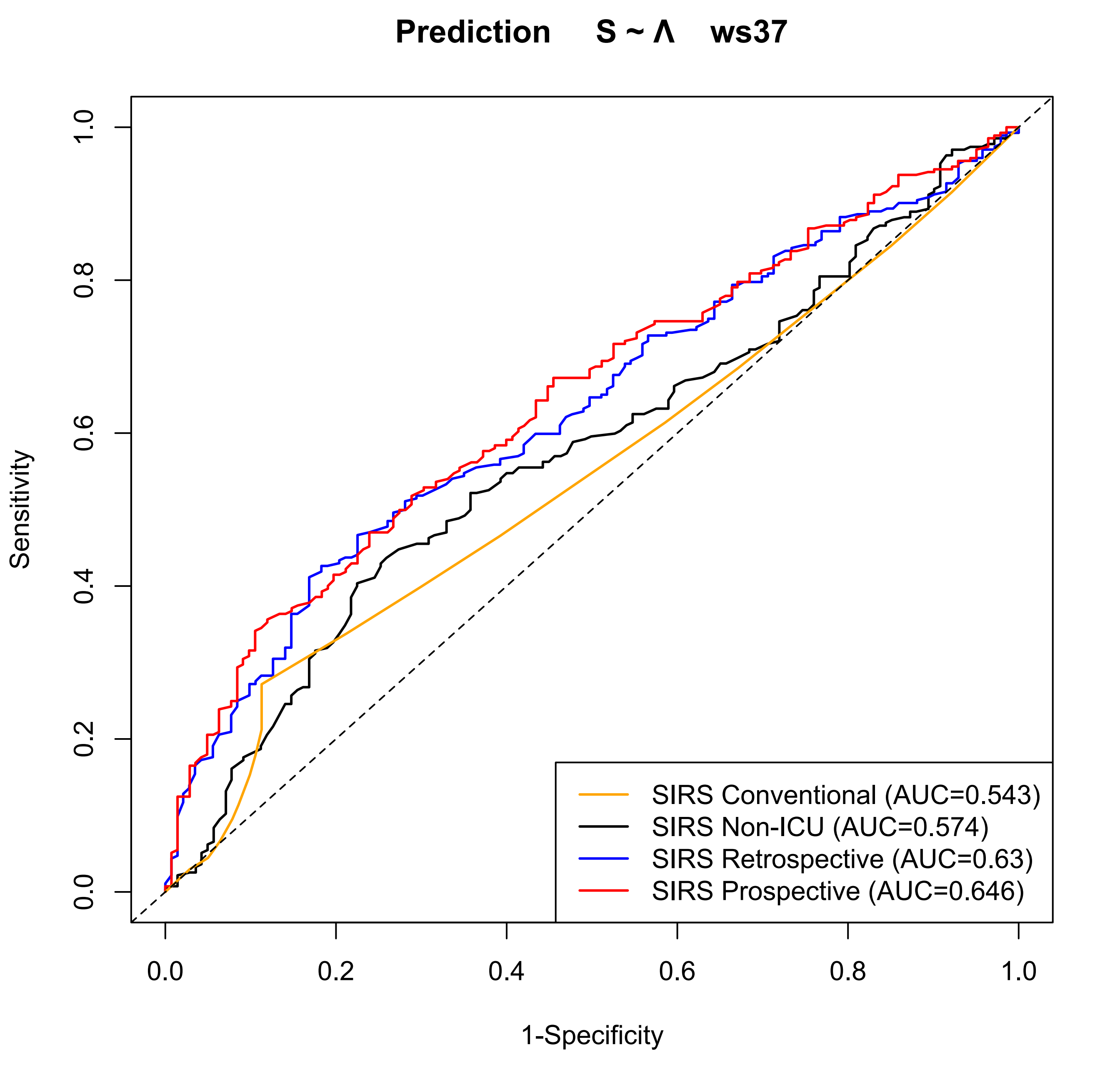
Prediction S ~ Λ ws37
Sensitivity
1-Specificity
0.0
0.2
0.4
0.6
0.8
1.0
SIRS Conventional (AUC=0.543)
SIRS Non-ICU (AUC=0.574)
SIRS Retrospective (AUC=0.63)
SIRS Prospective (AUC=0.646)

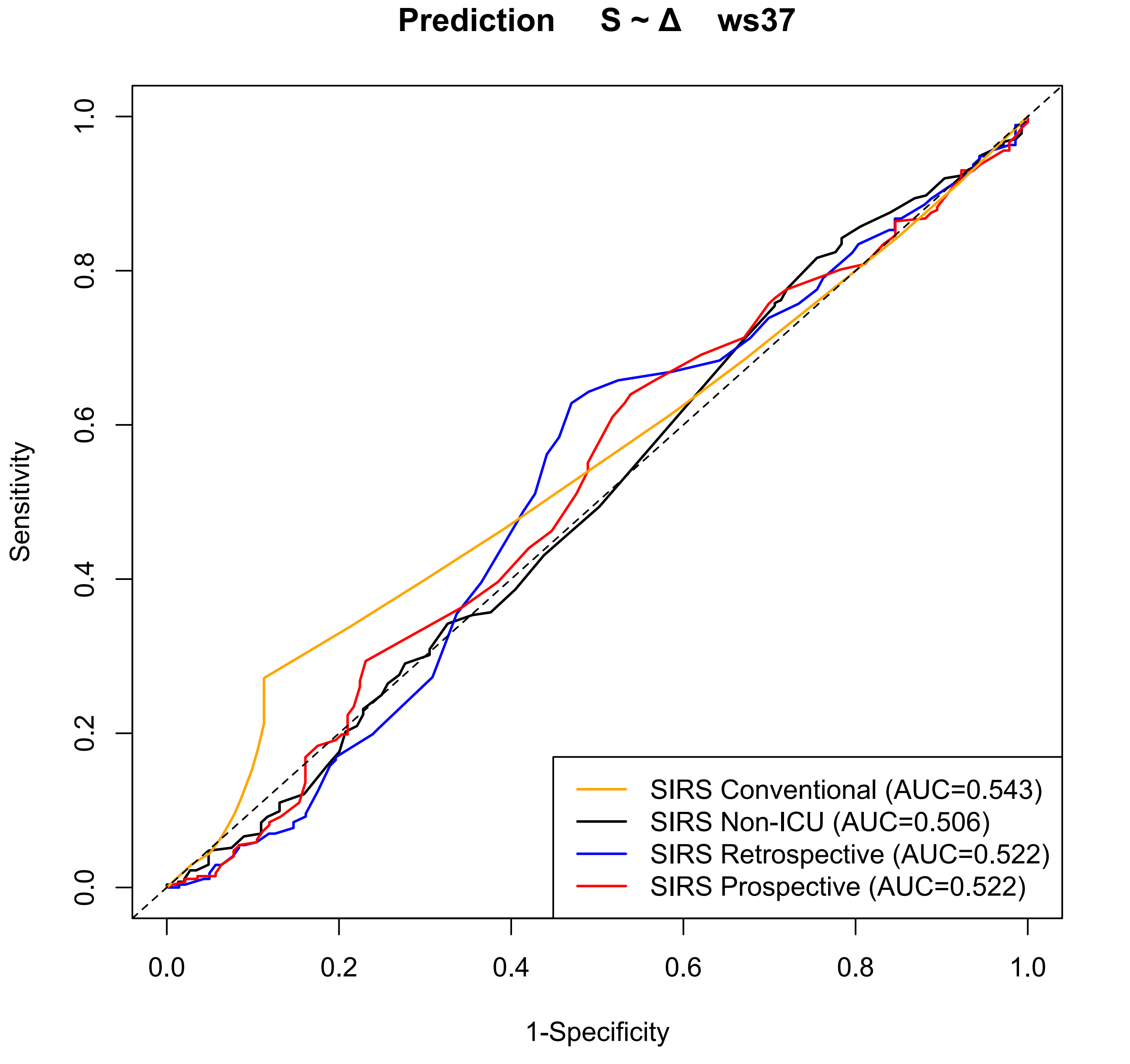

Prediction S ~ Δ ws37
Sensitivity
1-Specificity
0.0
0.2
0.4
0.6
0.8
1.0
SIRS Conventional (AUC=0.543)
SIRS Non-ICU (AUC=0.506)
SIRS Retrospective (AUC=0.522)
SIRS Prospective (AUC=0.522)

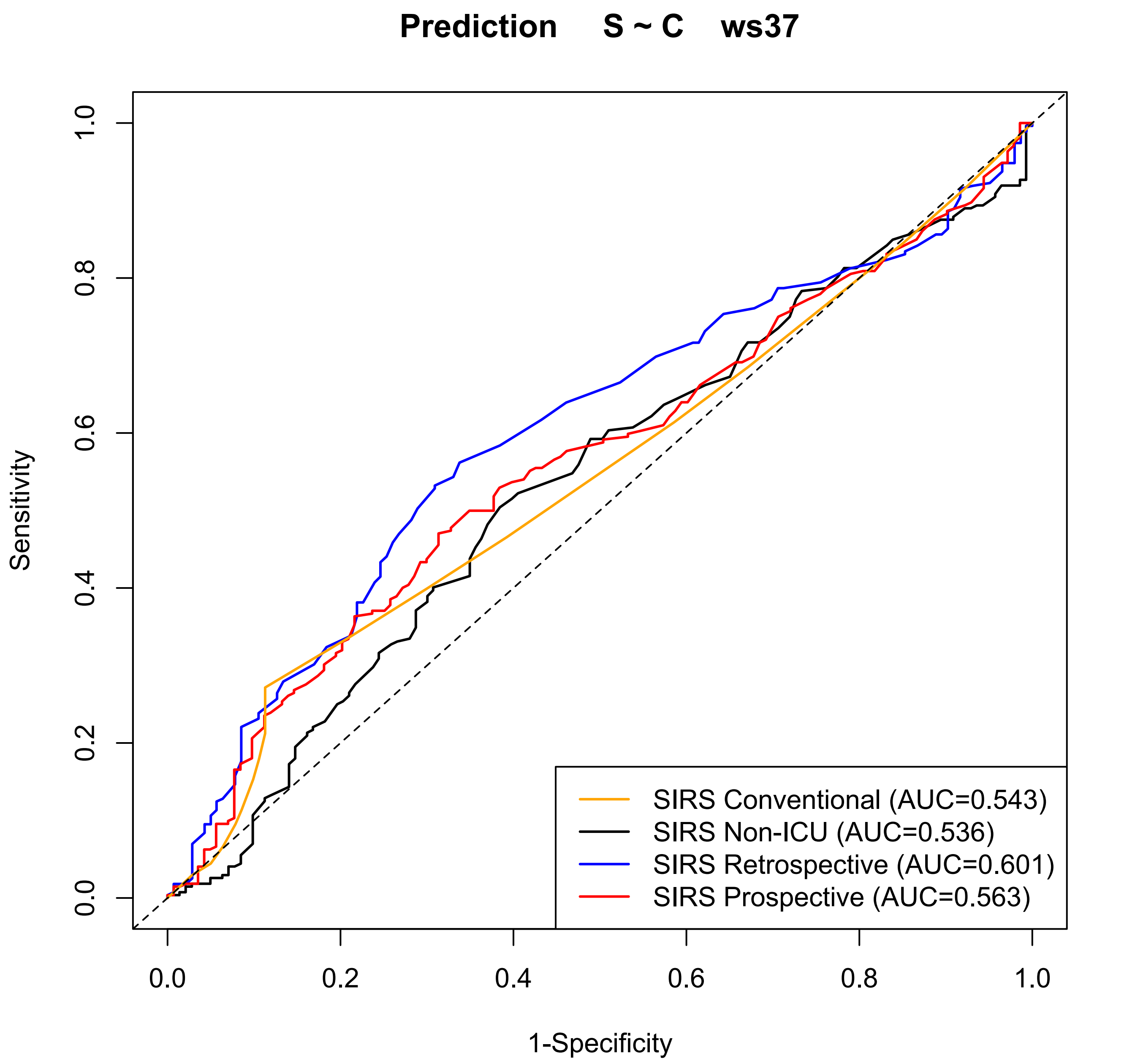

Prediction S ~ C ws37
Sensitivity
1-Specificity
0.0
0.2
0.4
0.6
0.8
1.0
SIRS Conventional (AUC=0.543)
SIRS Non-ICU (AUC=0.536)
SIRS Retrospective (AUC=0.601)
SIRS Prospective (AUC=0.563)

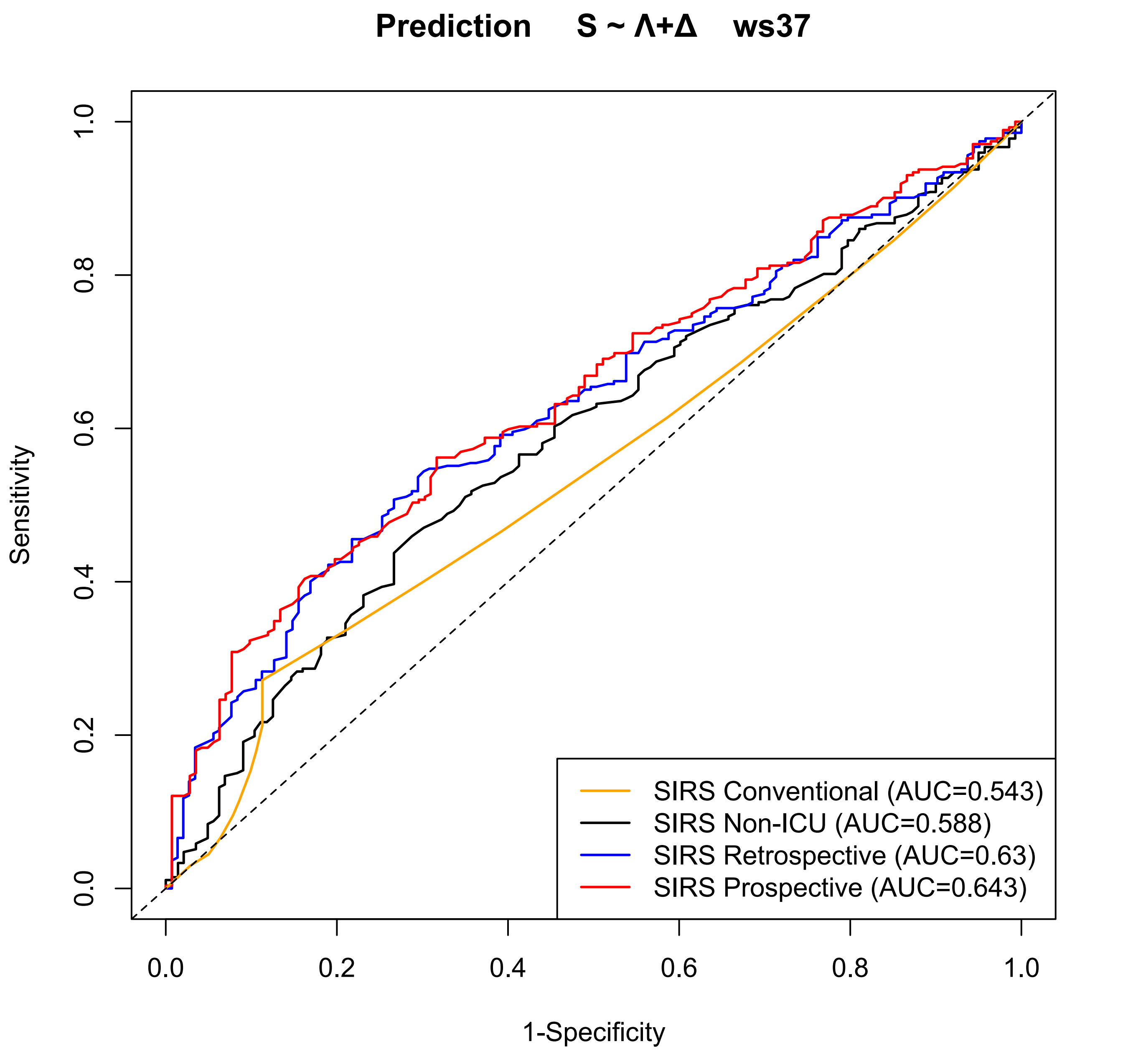

Prediction S ~ Λ+Δ ws37
Sensitivity
1-Specificity
0.0
0.2
0.4
0.6
0.8
1.0
0.0
0.2
0.4
0.6
0.8
1.0
SIRS Conventional (AUC=0.543)
SIRS Non-ICU (AUC=0.588)
SIRS Retrospective (AUC=0.63)
SIRS Prospective (AUC=0.643)

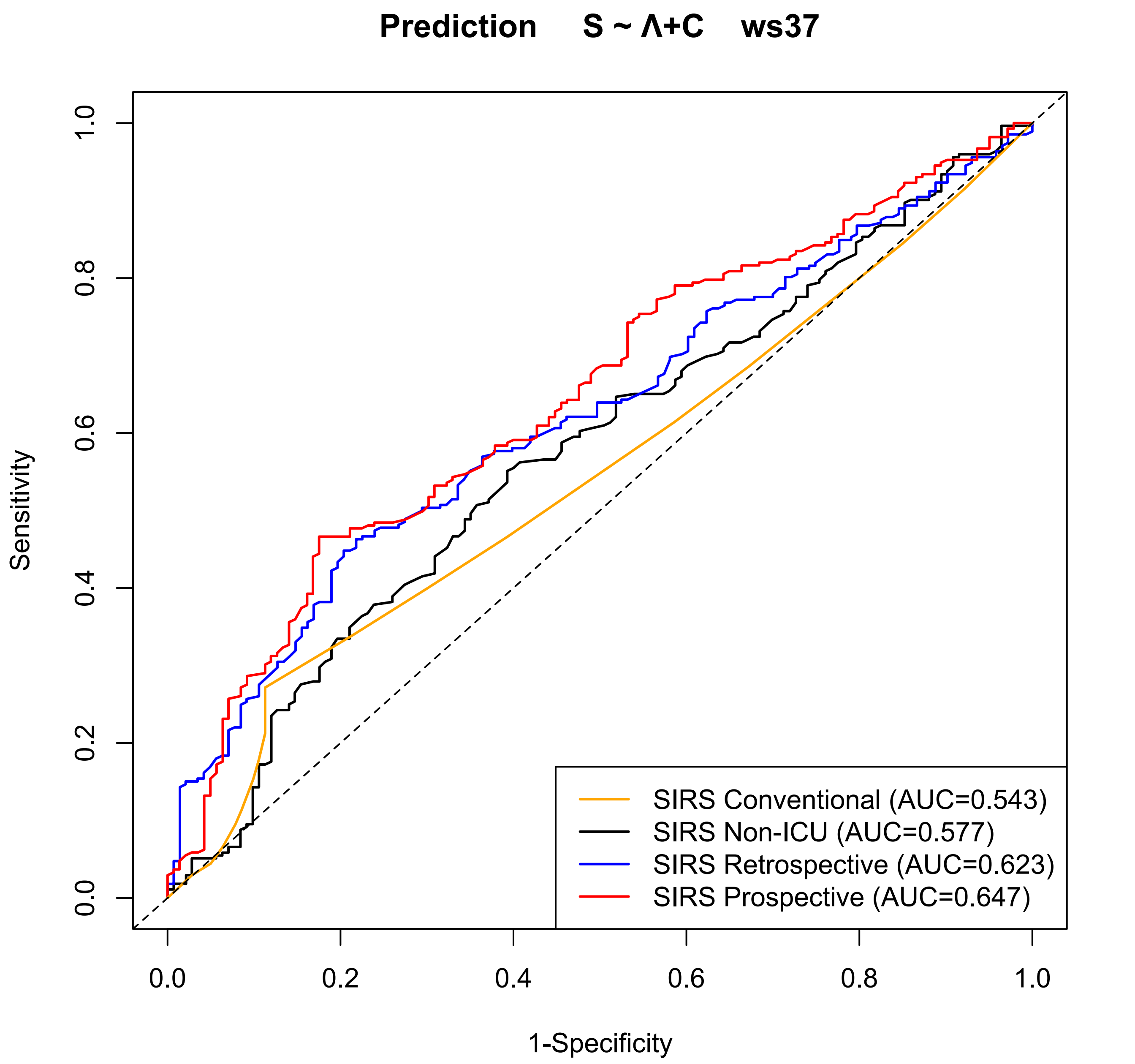
Prediction S ~ Λ+C ws37
Sensitivity
1-Specificity
0.0
0.2
0.4
0.6
0.8
1.0
SIRS Conventional (AUC=0.543)
SIRS Non-ICU (AUC=0.577)
SIRS Retrospective (AUC=0.623)
SIRS Prospective (AUC=0.647)

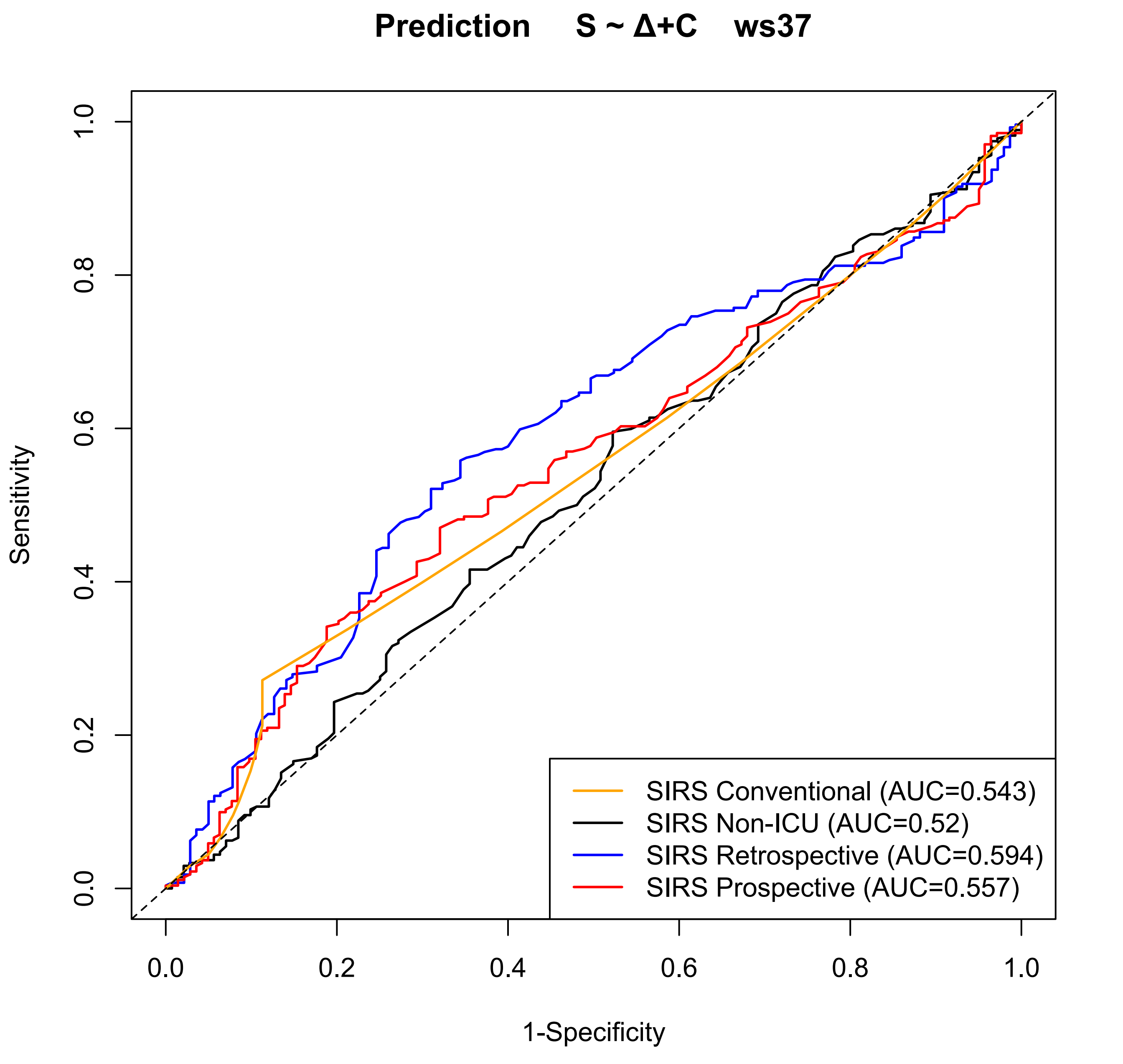

Prediction S ~ Δ+C ws37
Sensitivity
1-Specificity
0.0
0.2
0.4
0.6
0.8
1.0
SIRS Conventional (AUC=0.543)
SIRS Non-ICU (AUC=0.52)
SIRS Retrospective (AUC=0.594)
SIRS Prospective (AUC=0.557)

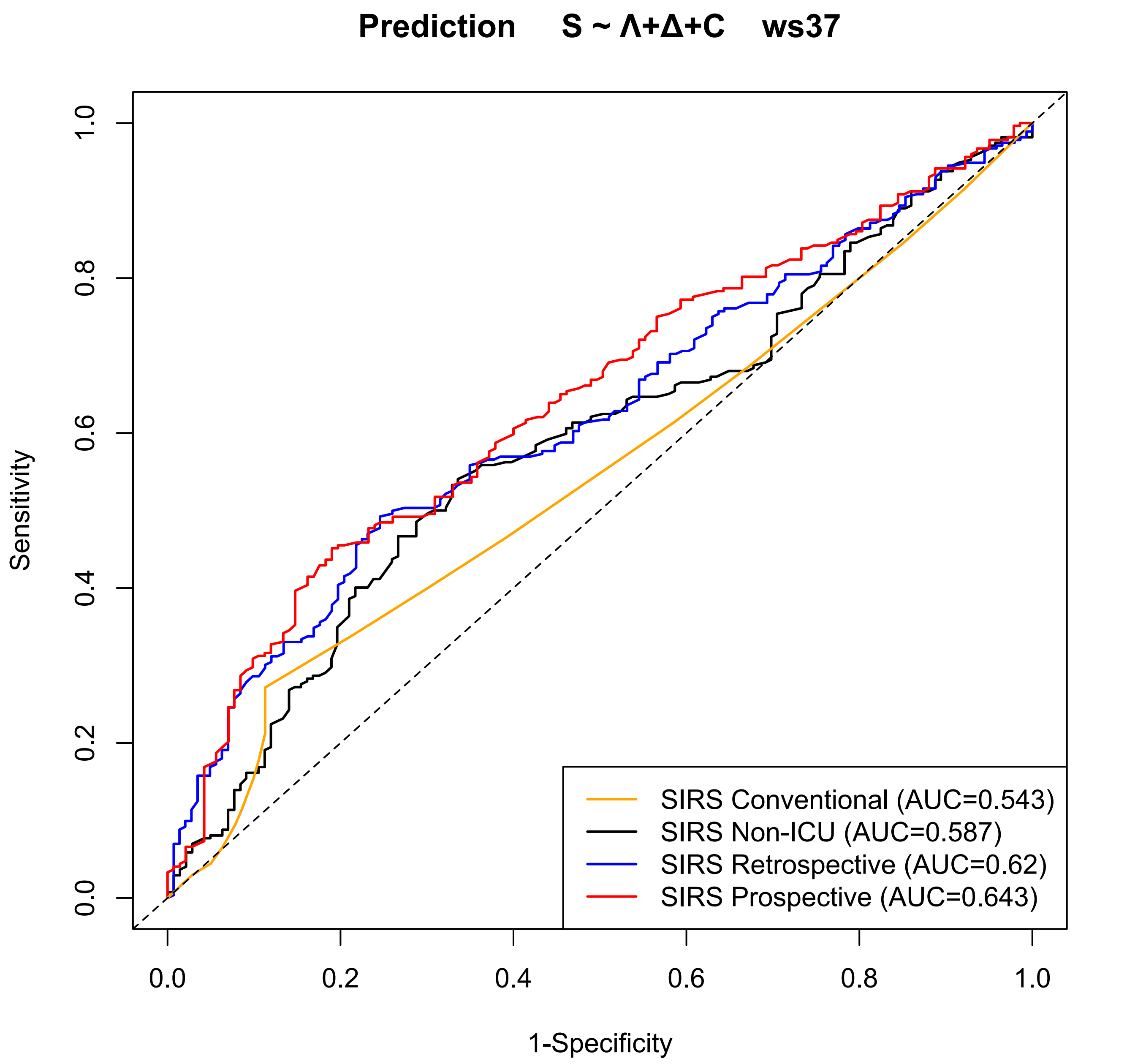

Prediction S ~ Λ+Δ+C ws37
Sensitivity
1-Specificity
0.0
0.2
0.4
0.6
0.8
1.0
0.0
0.2
0.4
0.6
0.8
1.0
SIRS Conventional (AUC=0.543)
SIRS Non-ICU (AUC=0.587)
SIRS Retrospective (AUC=0.62)
SIRS Prospective (AUC=0.643)

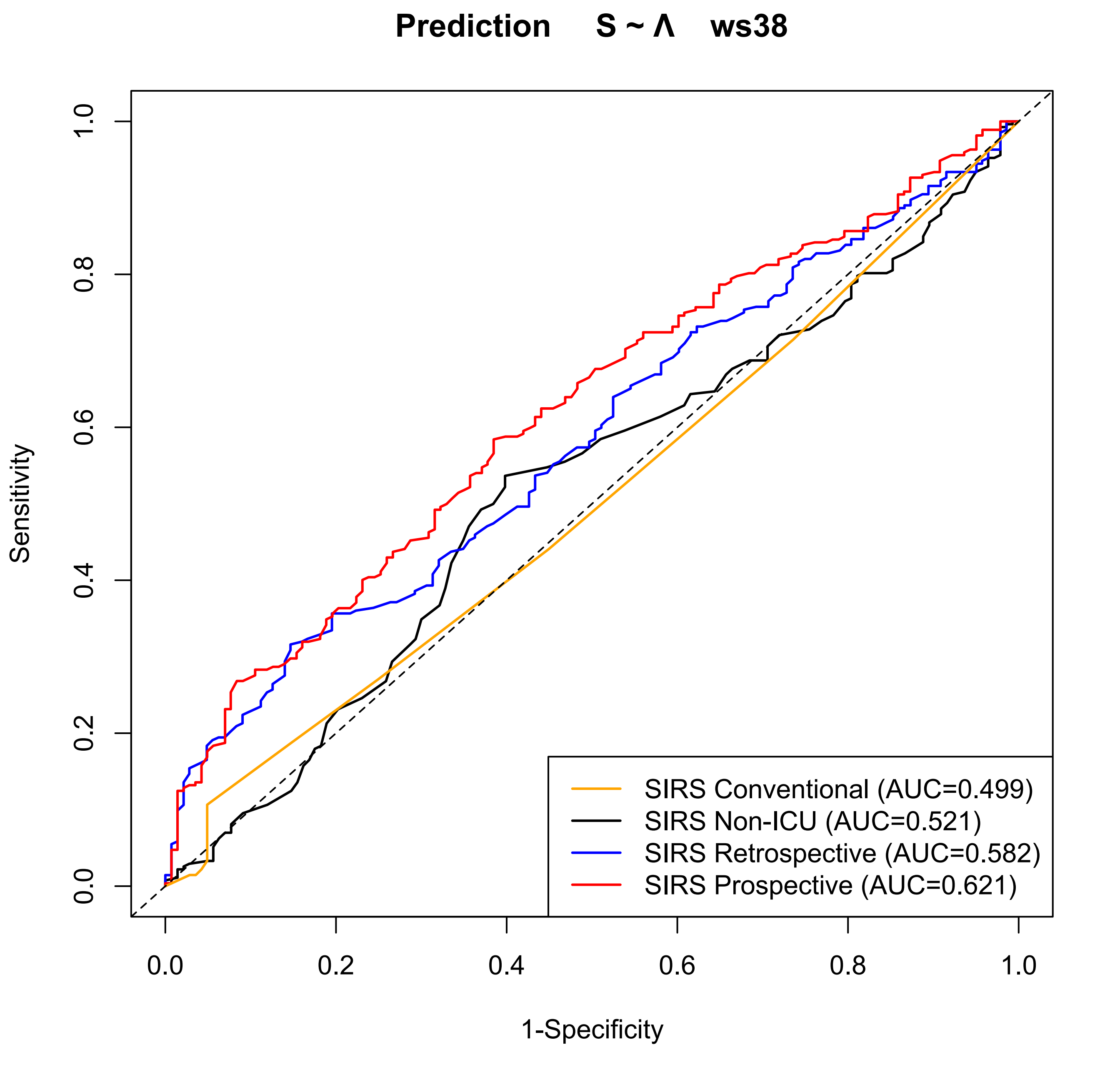

Prediction S ~ Λ ws38
Sensitivity
1-Specificity
0.0
0.2
0.4
0.6
0.8
1.0
0.0
0.2
0.4
0.6
0.8
1.0
SIRS Conventional (AUC=0.499)
SIRS Non-ICU (AUC=0.521)
SIRS Retrospective (AUC=0.582)
SIRS Prospective (AUC=0.621)

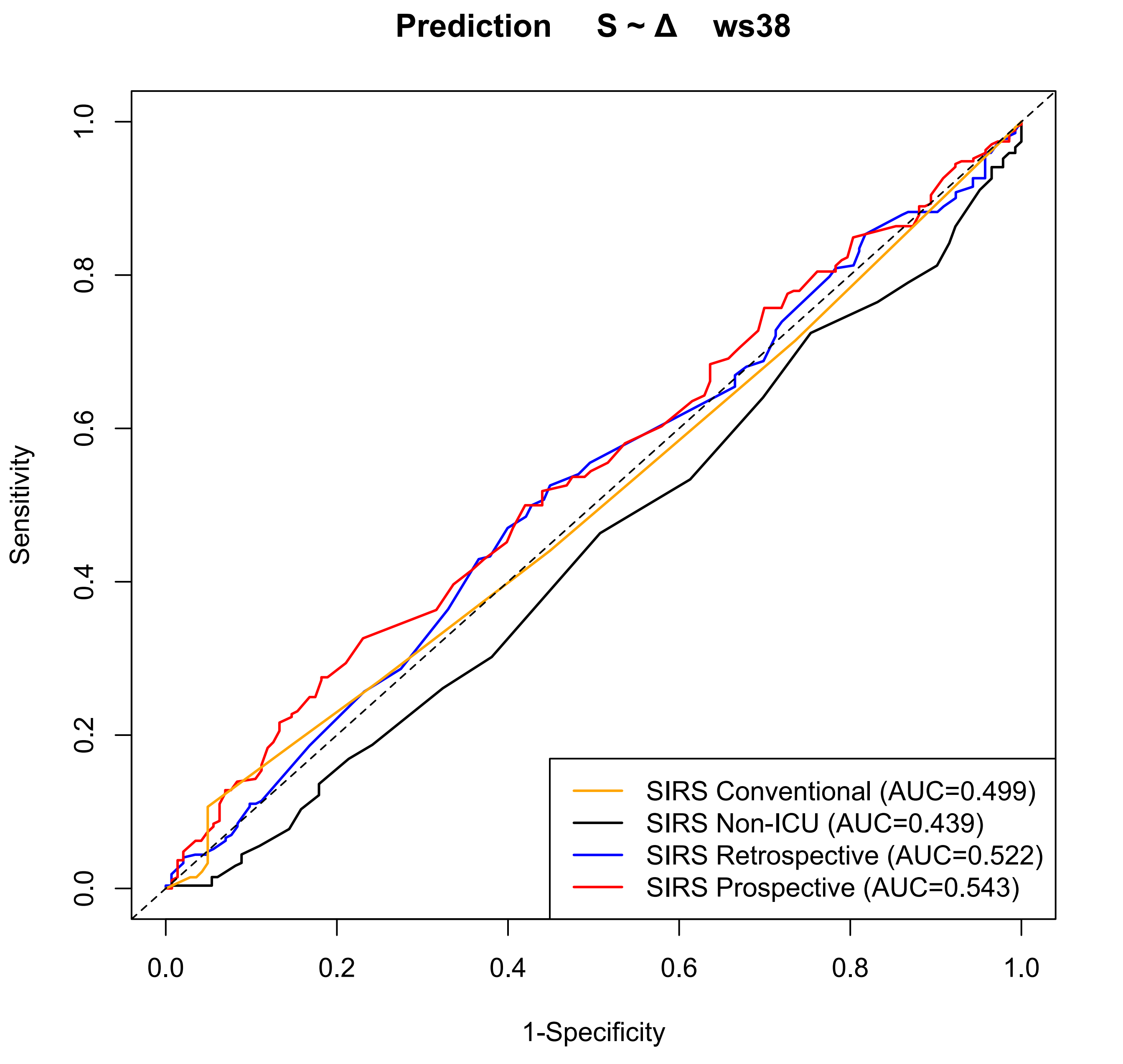
Prediction S ~ Δ ws38
Sensitivity
1-Specificity
0.0
0.2
0.4
0.6
0.8
1.0
SIRS Conventional (AUC=0.499)
SIRS Non-ICU (AUC=0.439)
SIRS Retrospective (AUC=0.522)
SIRS Prospective (AUC=0.543)

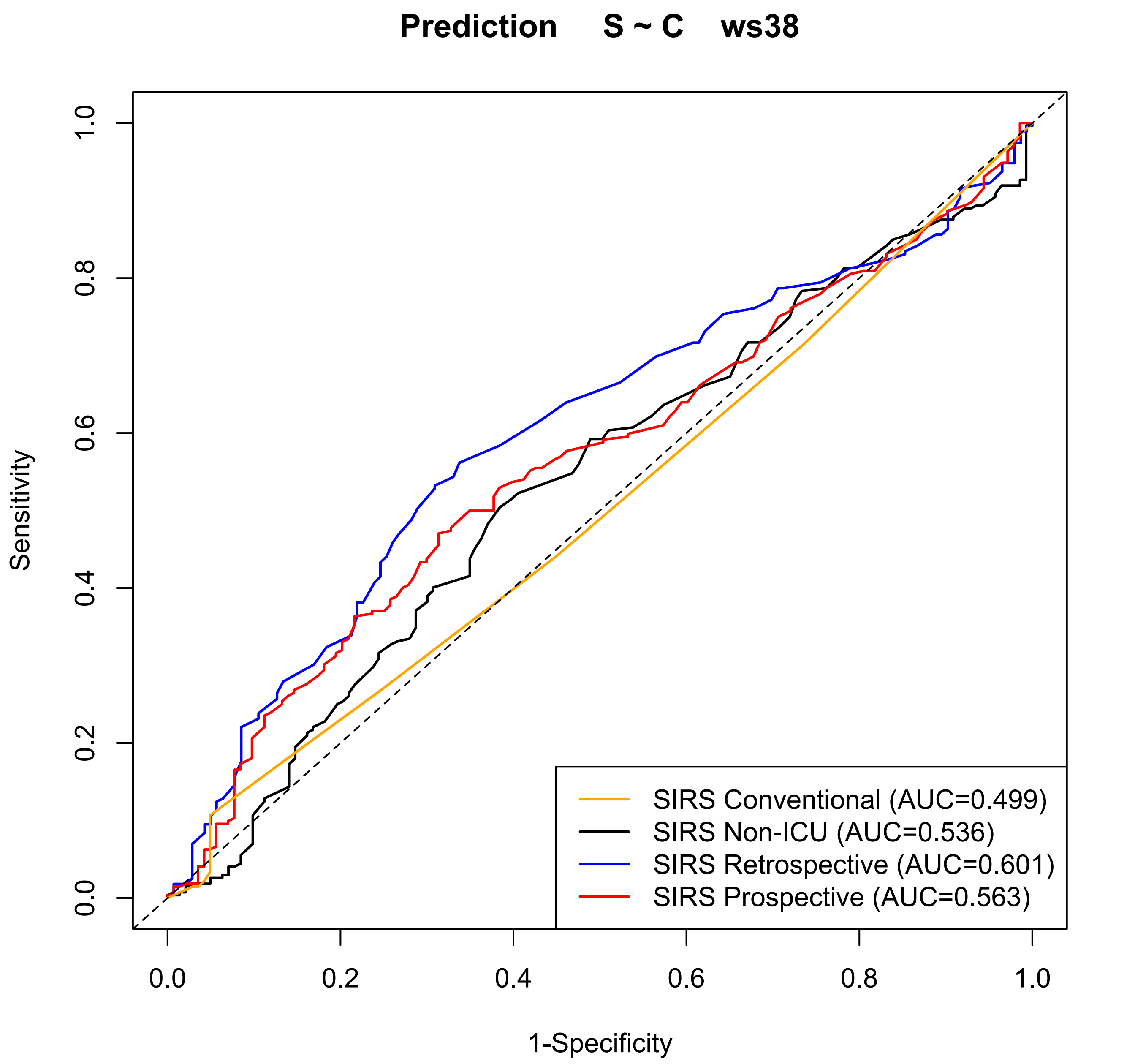

Prediction S ~ C ws38
Sensitivity
1-Specificity
0.0
0.2
0.4
0.6
0.8
1.0
SIRS Conventional (AUC=0.499)
SIRS Non-ICU (AUC=0.536)
SIRS Retrospective (AUC=0.601)
SIRS Prospective (AUC=0.563)

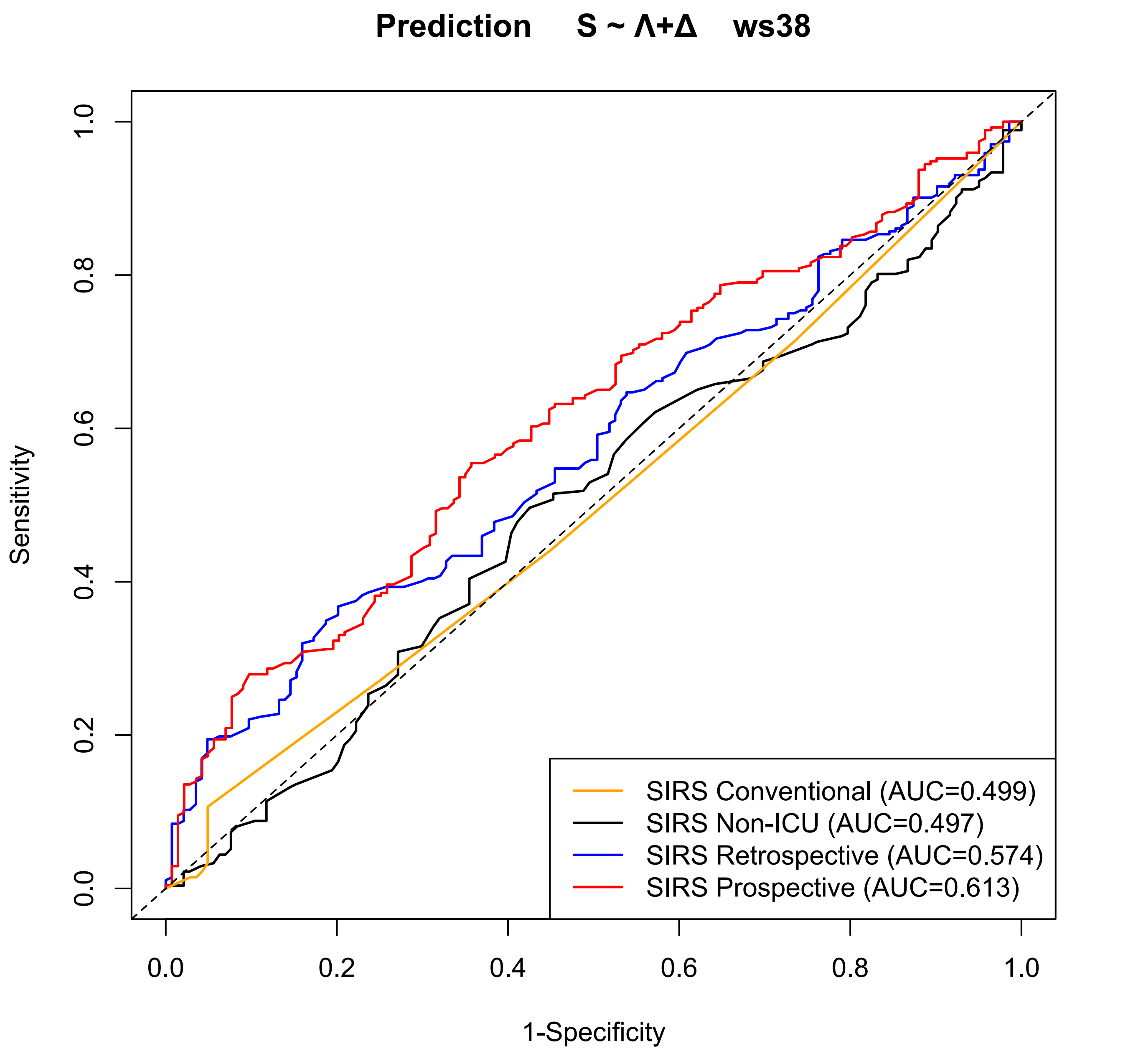
Prediction S ~ Λ+Δ ws38
Sensitivity
1-Specificity
0.0
0.2
0.4
0.6
0.8
1.0
SIRS Conventional (AUC=0.499)
SIRS Non-ICU (AUC=0.497)
SIRS Retrospective (AUC=0.574)
SIRS Prospective (AUC=0.613)

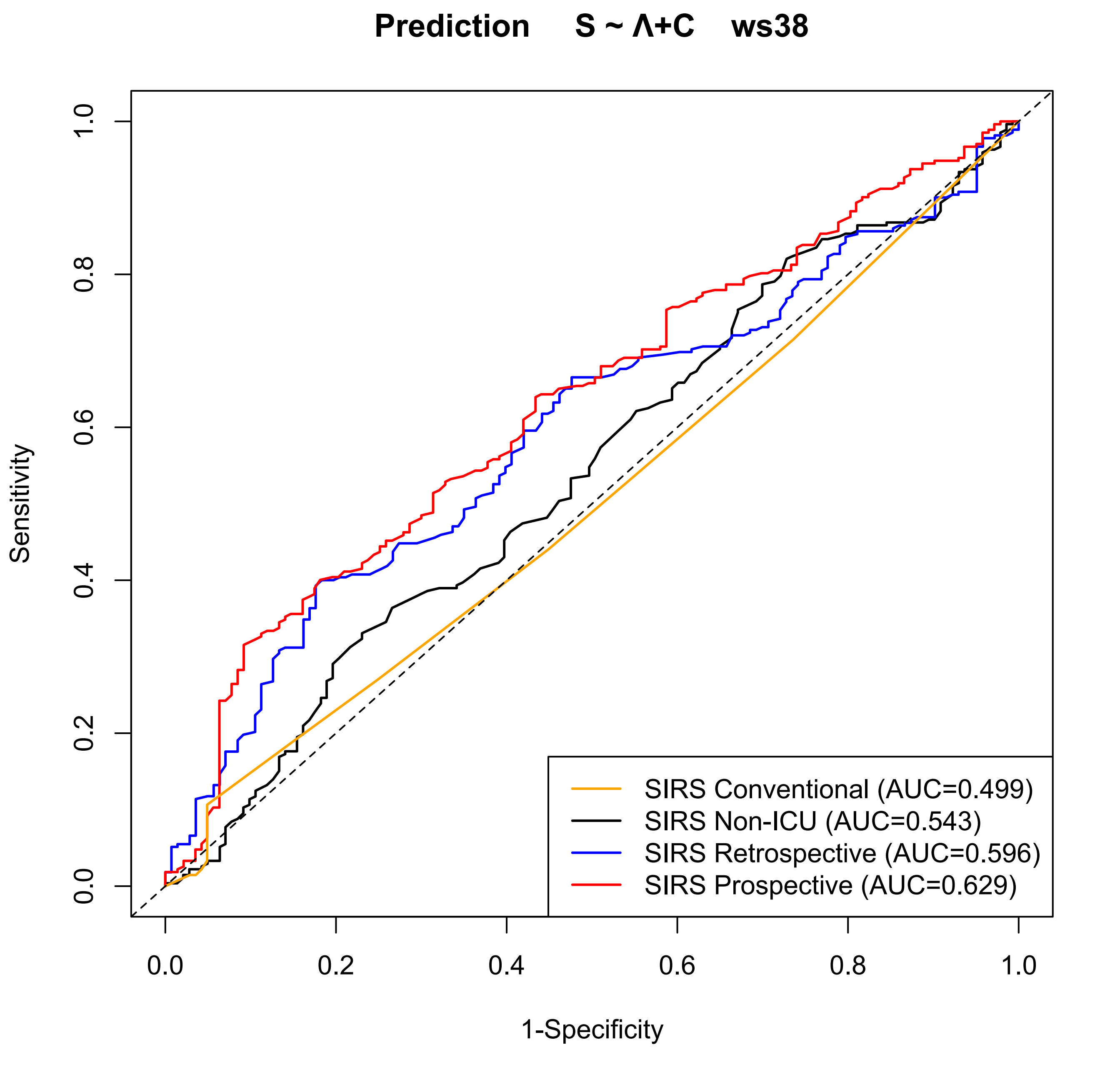

Prediction S ~ Λ+C ws38
Sensitivity
1-Specificity
0.0
0.2
0.4
0.6
0.8
1.0
SIRS Conventional (AUC=0.499)
SIRS Non-ICU (AUC=0.543)
SIRS Retrospective (AUC=0.596)
SIRS Prospective (AUC=0.629)

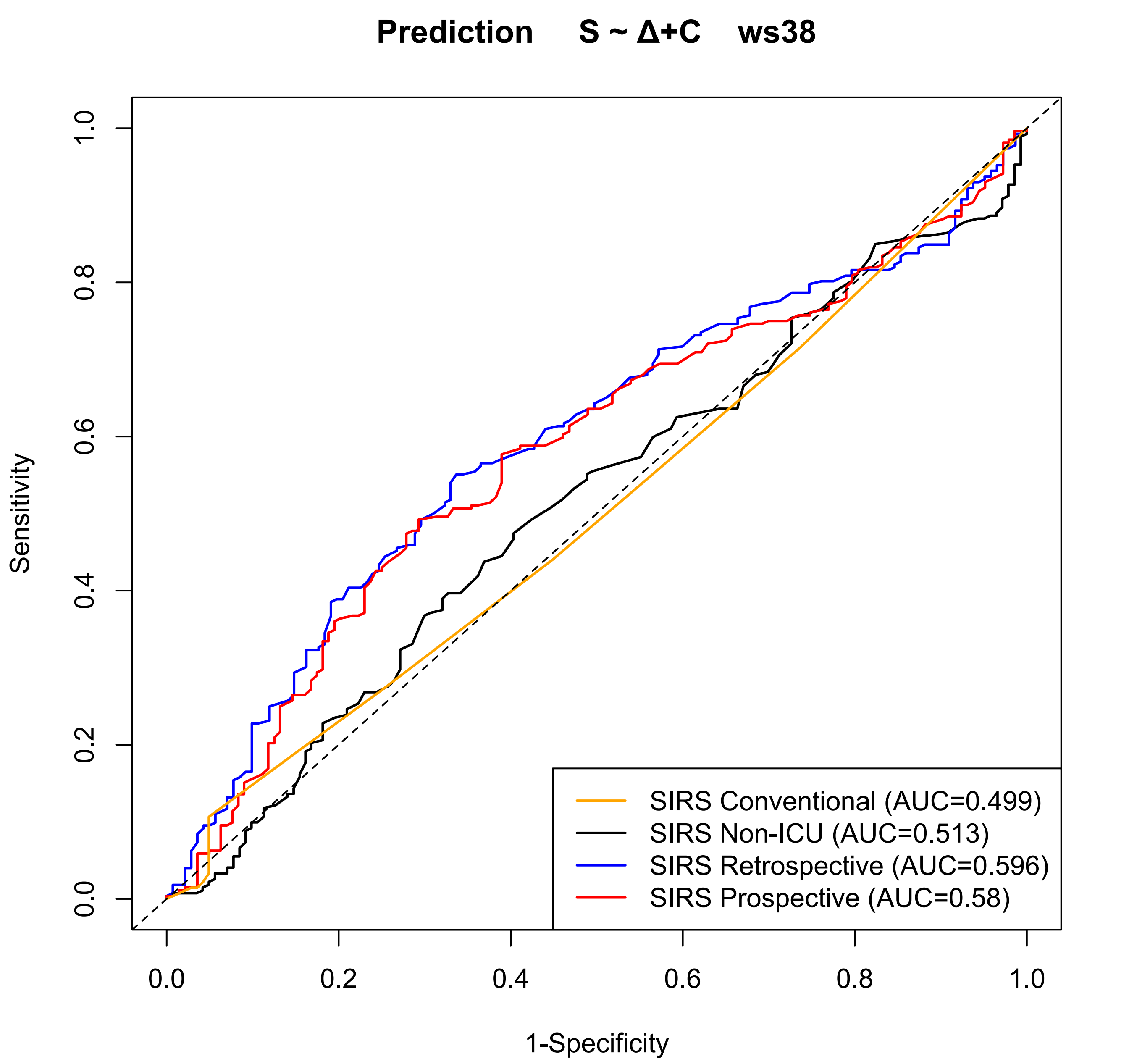

Prediction S ~ Δ+C ws38
Sensitivity
1-Specificity
0.0
0.2
0.4
0.6
0.8
1.0
SIRS Conventional (AUC=0.499)
SIRS Non-ICU (AUC=0.513)
SIRS Retrospective (AUC=0.596)
SIRS Prospective (AUC=0.58)

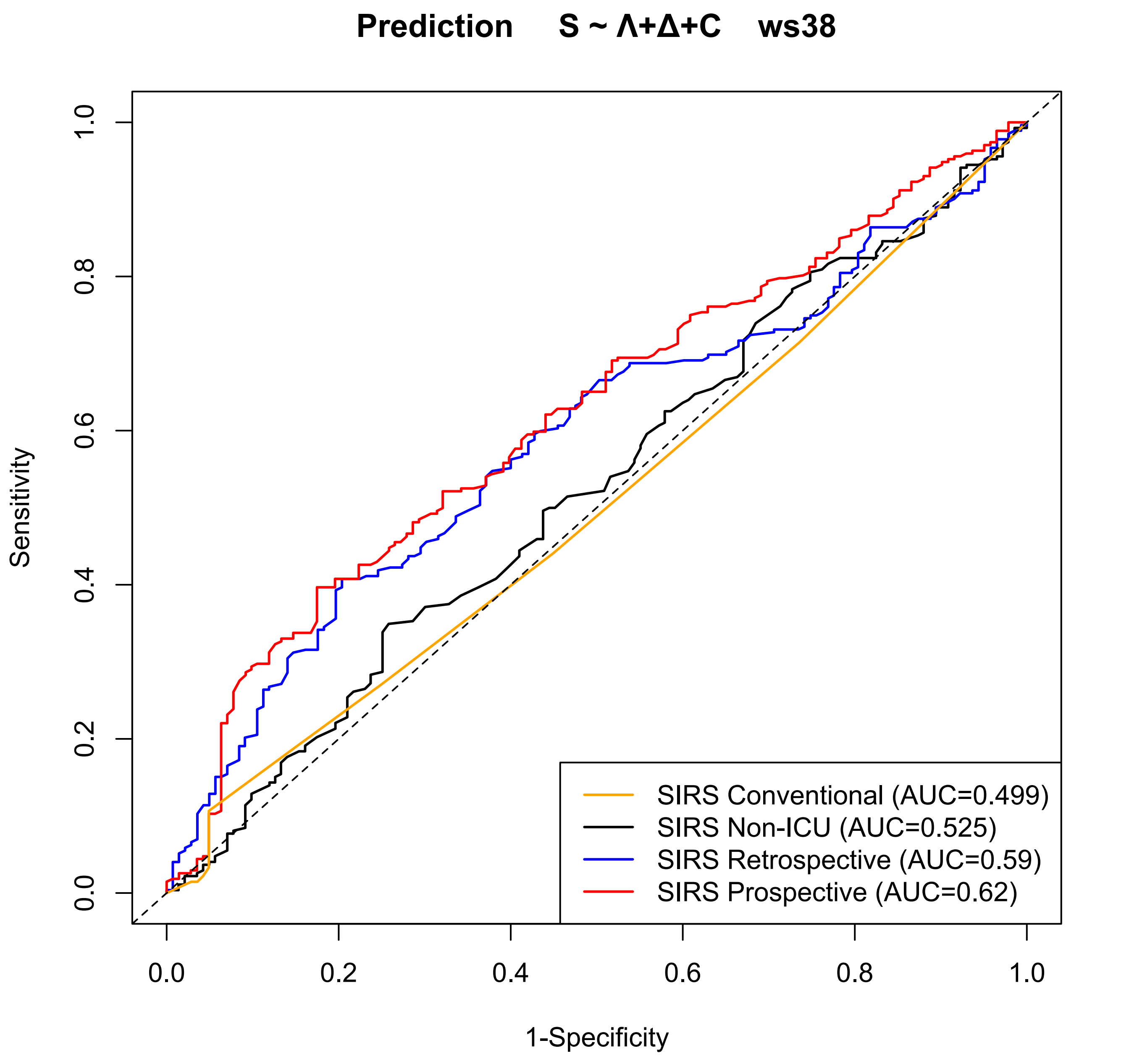

Prediction S ~ Λ+Δ+C ws38
Sensitivity
1-Specificity
0.0
0.2
0.4
0.6
0.8
1.0
SIRS Conventional (AUC=0.499)
SIRS Non-ICU (AUC=0.525)
SIRS Retrospective (AUC=0.59)
SIRS Prospective (AUC=0.62)

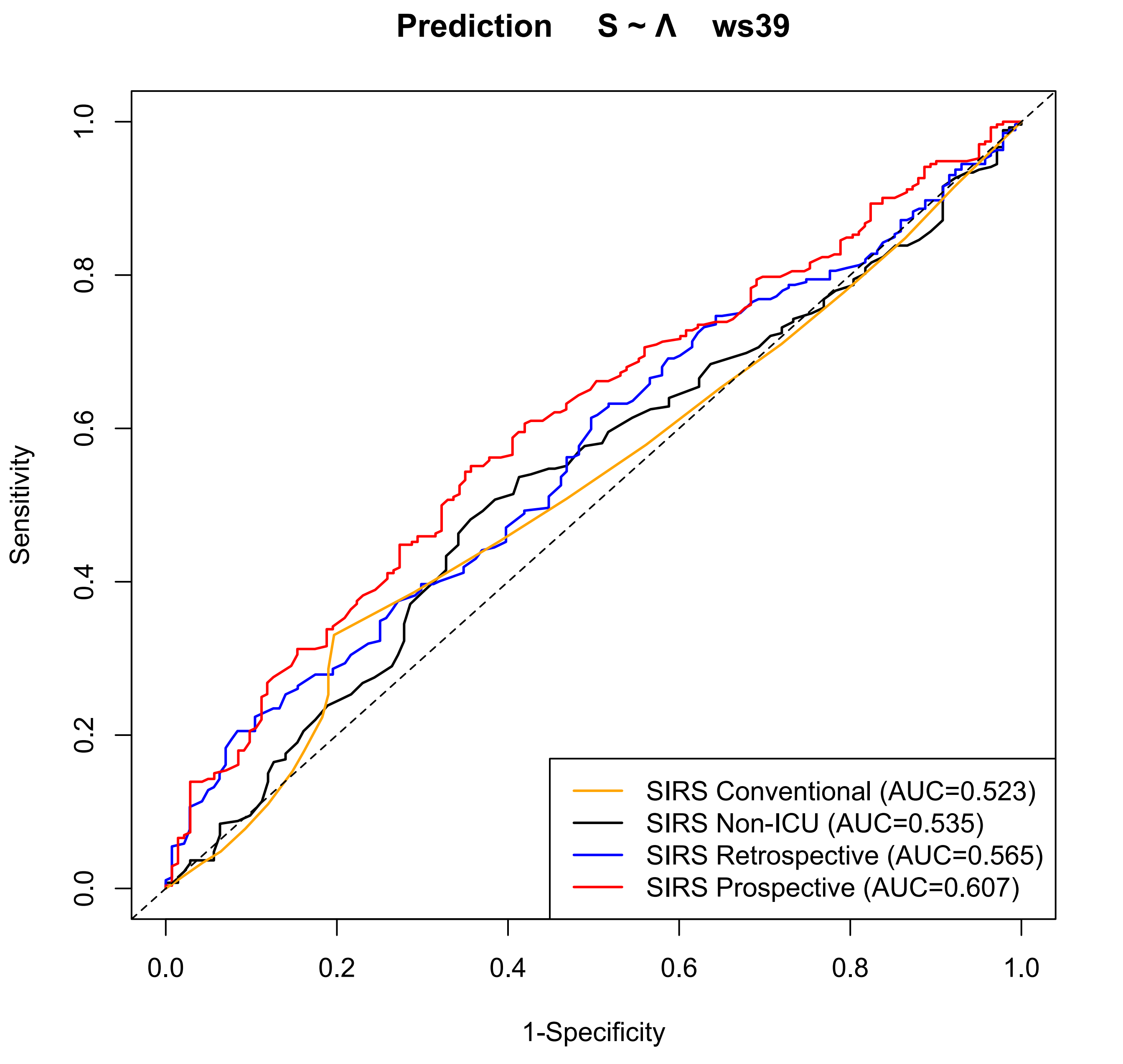

Prediction S ~ Λ ws39
Sensitivity
1-Specificity
0.0
0.2
0.4
0.6
0.8
1.0
SIRS Conventional (AUC=0.523)
SIRS Non-ICU (AUC=0.535)
SIRS Retrospective (AUC=0.565)
SIRS Prospective (AUC=0.607)

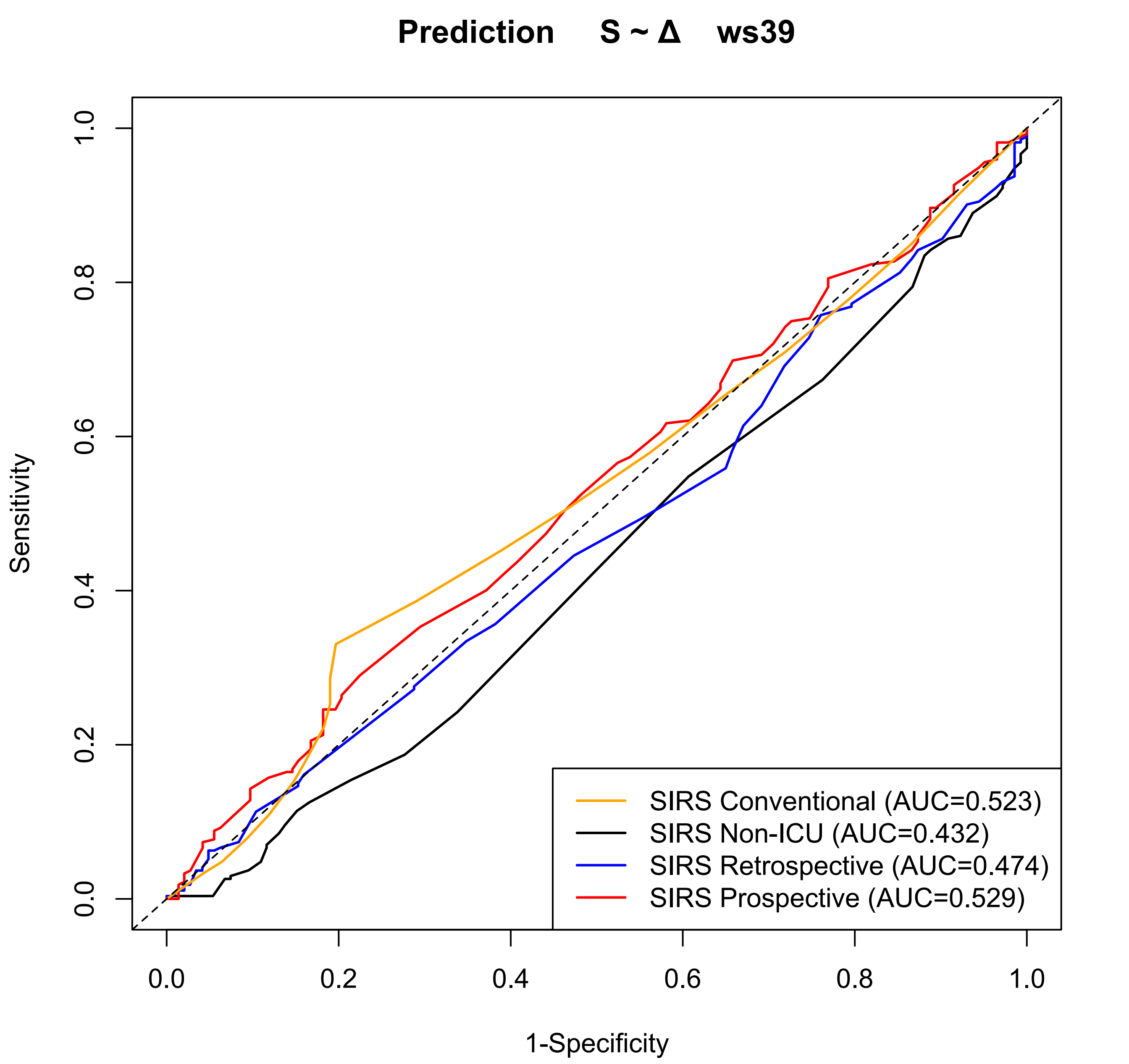

Prediction S ~ Δ ws39
Sensitivity
1-Specificity
0.0
0.2
0.4
0.6
0.8
1.0
SIRS Conventional (AUC=0.523)
SIRS Non-ICU (AUC=0.432)
SIRS Retrospective (AUC=0.474)
SIRS Prospective (AUC=0.529)

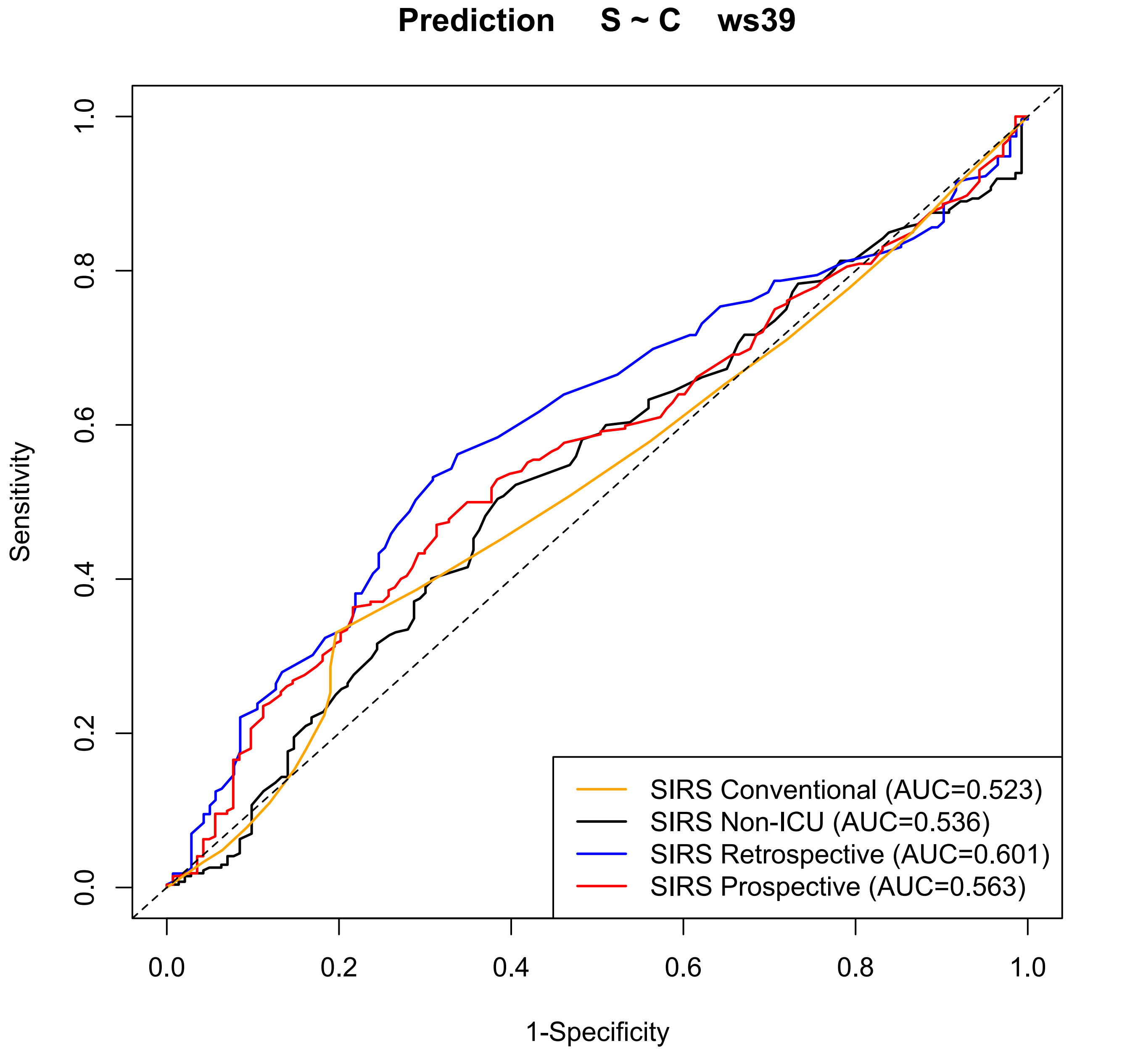

Prediction S ~ C ws39
Sensitivity
1-Specificity
0.0
0.2
0.4
0.6
0.8
1.0
SIRS Conventional (AUC=0.523)
SIRS Non-ICU (AUC=0.536)
SIRS Retrospective (AUC=0.601)
SIRS Prospective (AUC=0.563)

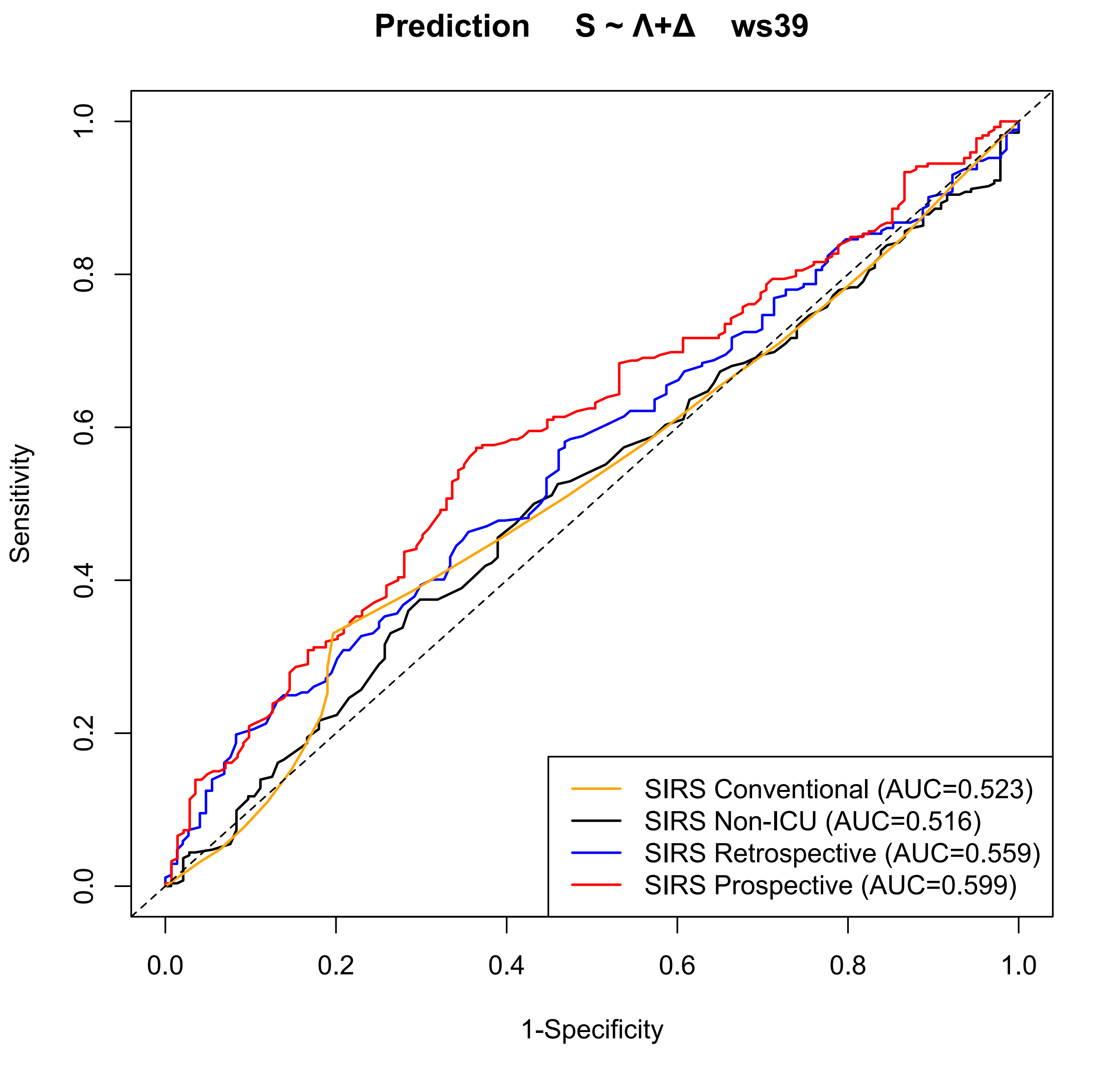

Prediction S ~ Λ+Δ ws39
Sensitivity
1-Specificity
0.0
0.2
0.4
0.6
0.8
1.0
SIRS Conventional (AUC=0.523)
SIRS Non-ICU (AUC=0.516)
SIRS Retrospective (AUC=0.559)
SIRS Prospective (AUC=0.599)

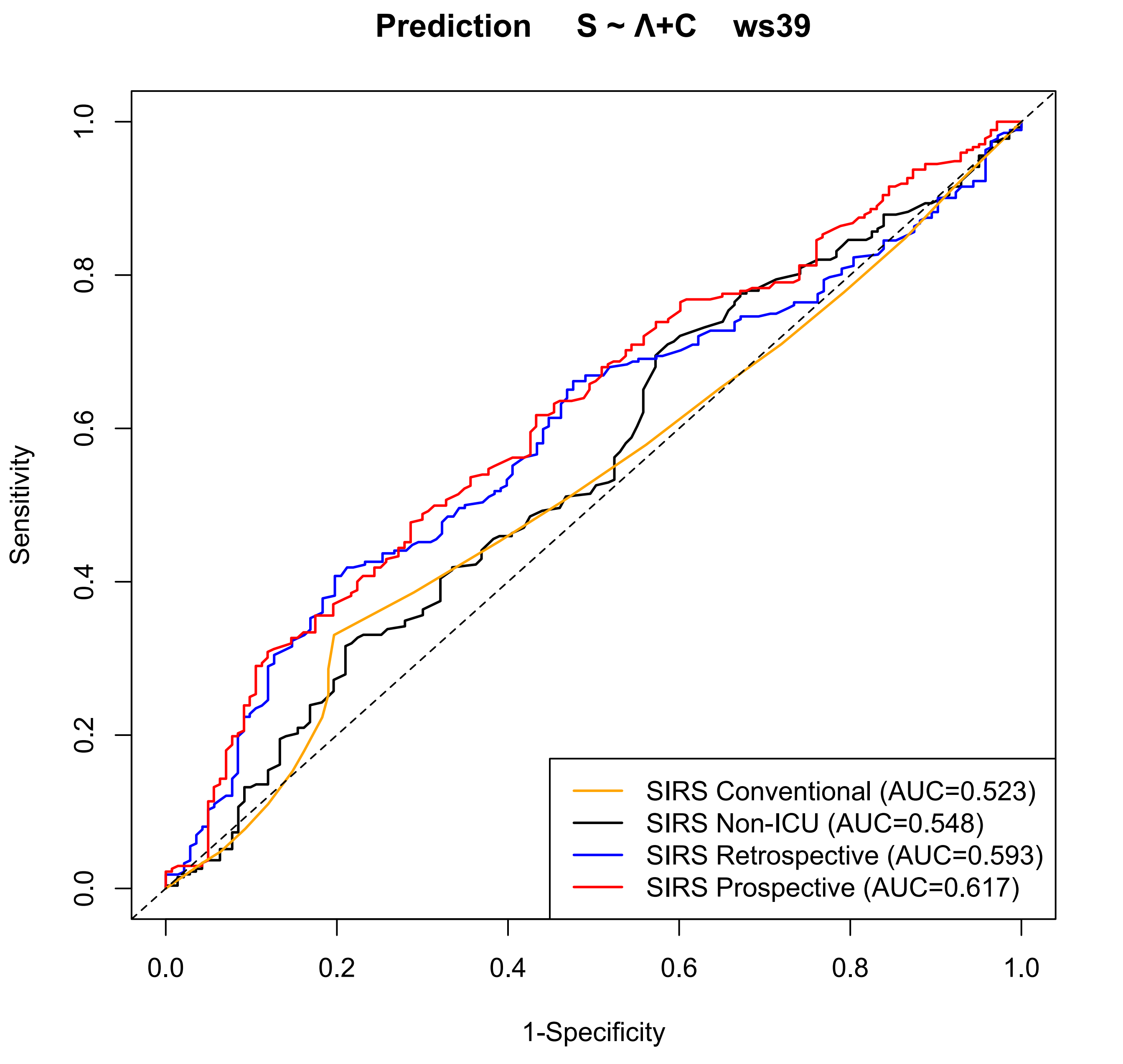

Prediction S ~ Λ+C ws39
Sensitivity
1-Specificity
0.0
0.2
0.4
0.6
0.8
1.0
SIRS Conventional (AUC=0.523)
SIRS Non-ICU (AUC=0.548)
SIRS Retrospective (AUC=0.593)
SIRS Prospective (AUC=0.617)

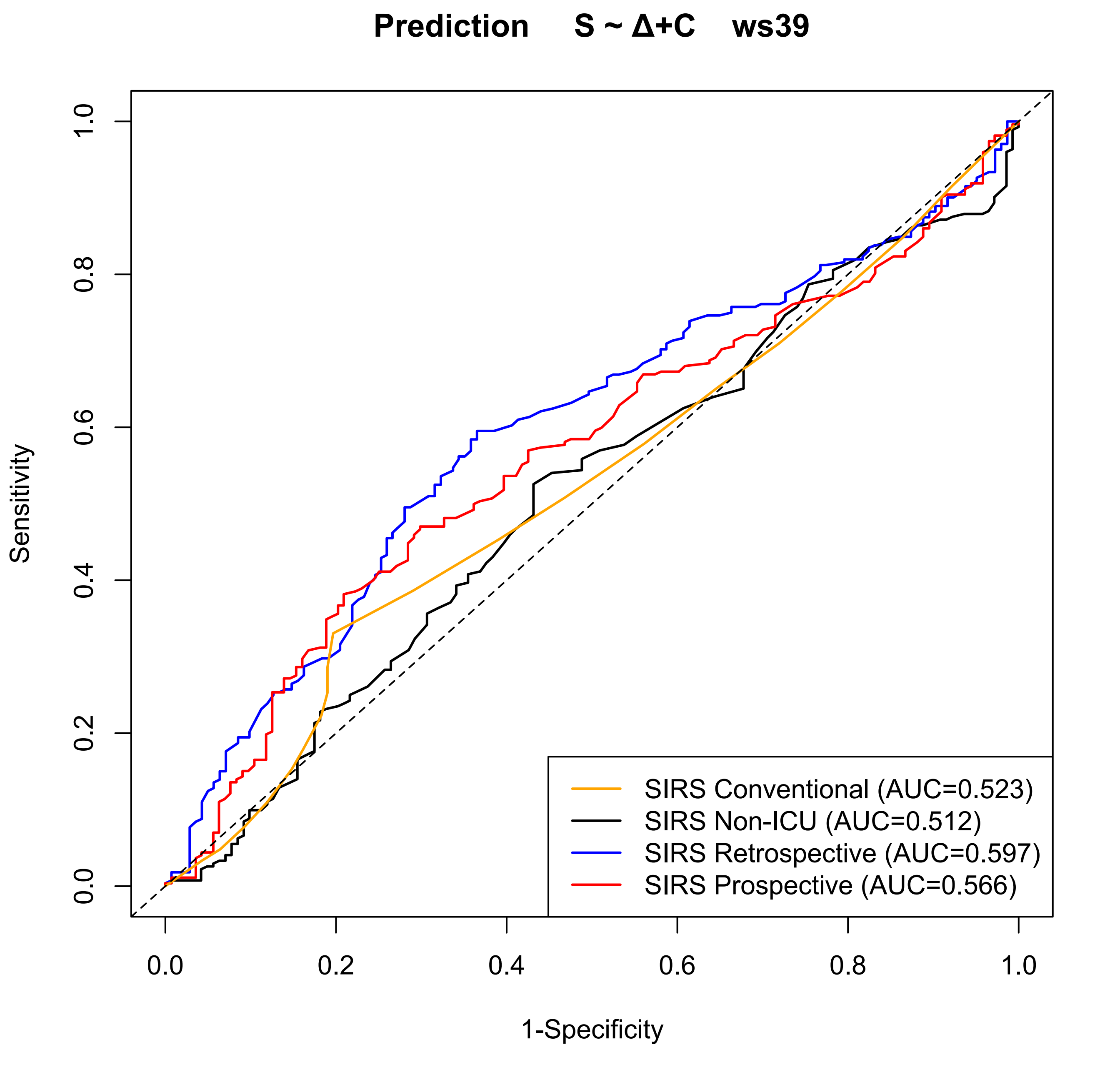

Prediction S ~ Δ+C ws39
Sensitivity
1-Specificity
0.0
0.2
0.4
0.6
0.8
1.0
0.0
0.2
0.4
0.6
0.8
1.0
SIRS Conventional (AUC=0.523)
SIRS Non-ICU (AUC=0.512)
SIRS Retrospective (AUC=0.597)
SIRS Prospective (AUC=0.566)

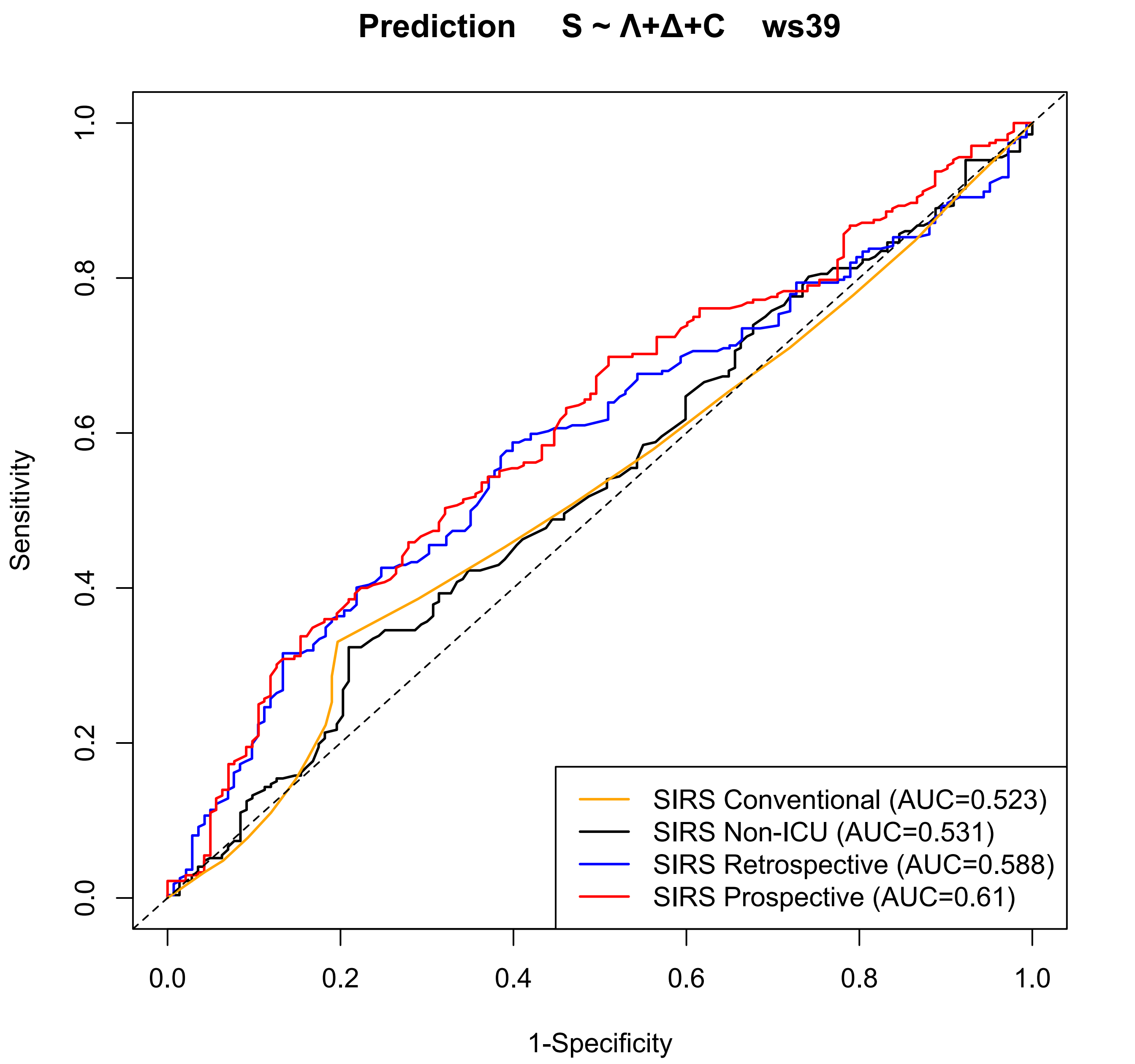
Prediction S ~ Λ+Δ+C ws39
Sensitivity
1-Specificity
0.0
0.2
0.4
0.6
0.8
1.0
SIRS Conventional (AUC=0.523)
SIRS Non-ICU (AUC=0.531)
SIRS Retrospective (AUC=0.588)
SIRS Prospective (AUC=0.61)

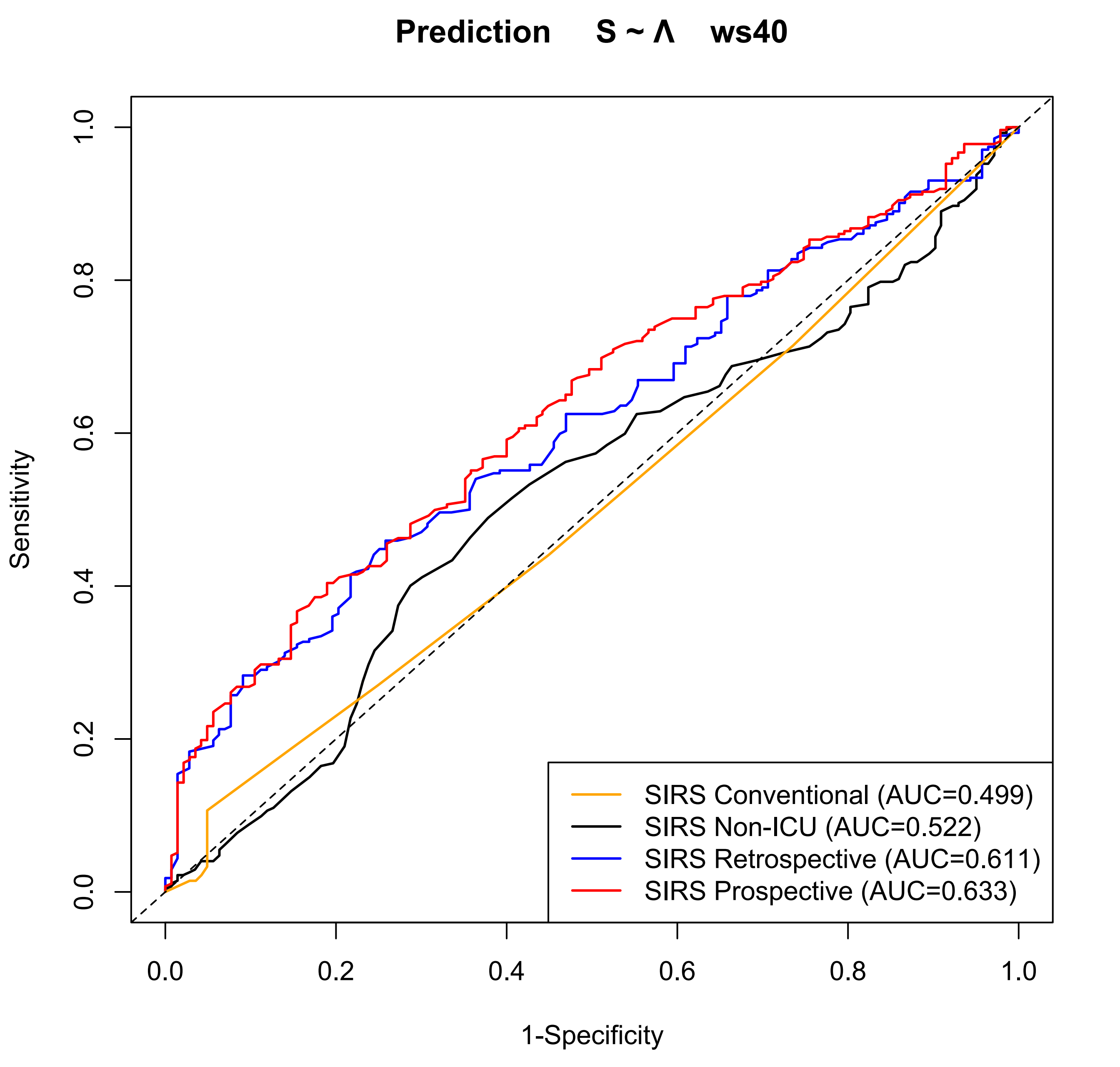
Prediction S ~ Λ ws40
Sensitivity
1-Specificity
0.0
0.2
0.4
0.6
0.8
1.0
SIRS Conventional (AUC=0.499)
SIRS Non-ICU (AUC=0.522)
SIRS Retrospective (AUC=0.611)
SIRS Prospective (AUC=0.633)

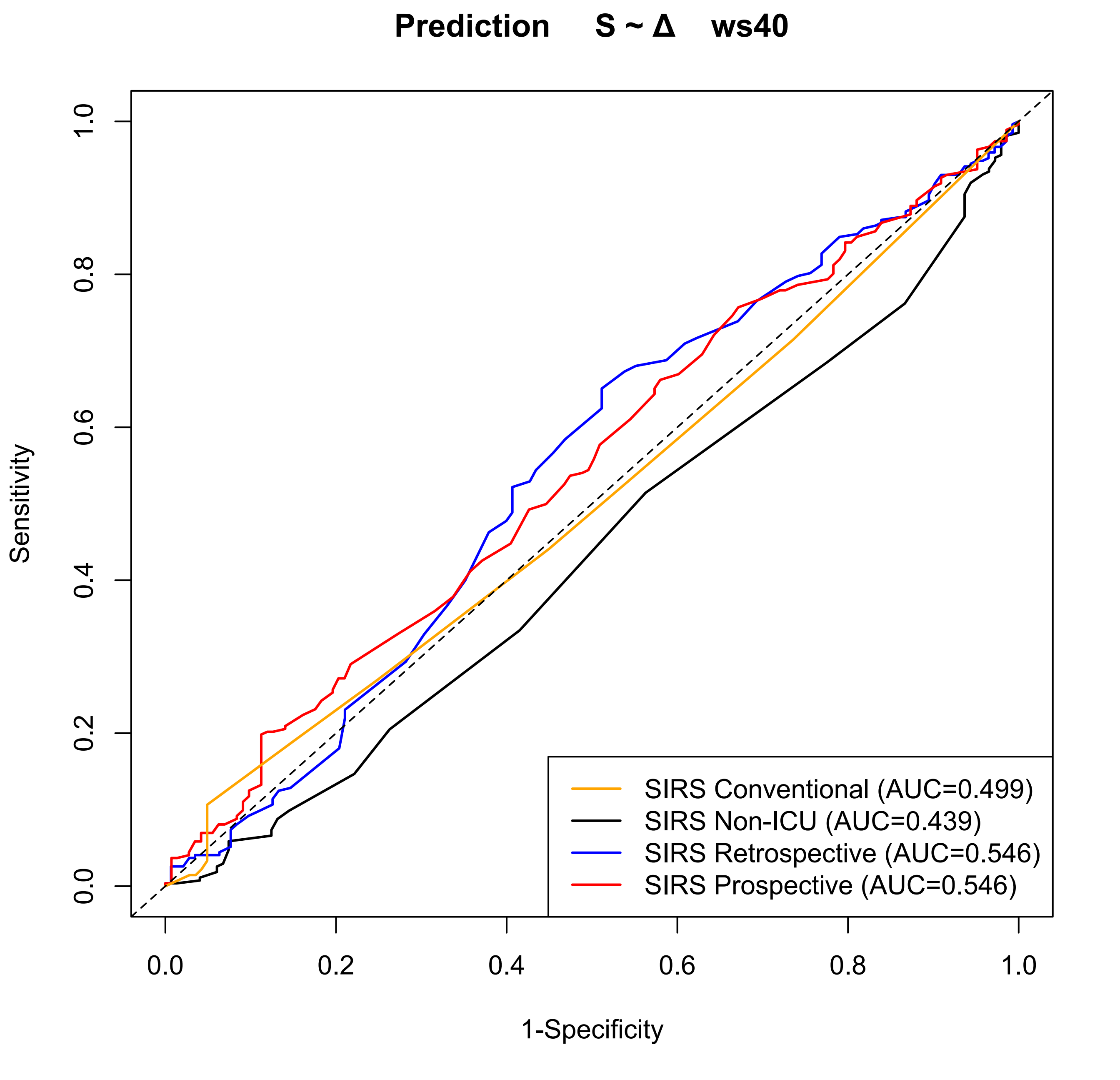
Prediction S ~ Δ ws40
Sensitivity
1-Specificity
0.0
0.2
0.4
0.6
0.8
1.0
SIRS Conventional (AUC=0.499)
SIRS Non-ICU (AUC=0.439)
SIRS Retrospective (AUC=0.546)
SIRS Prospective (AUC=0.546)

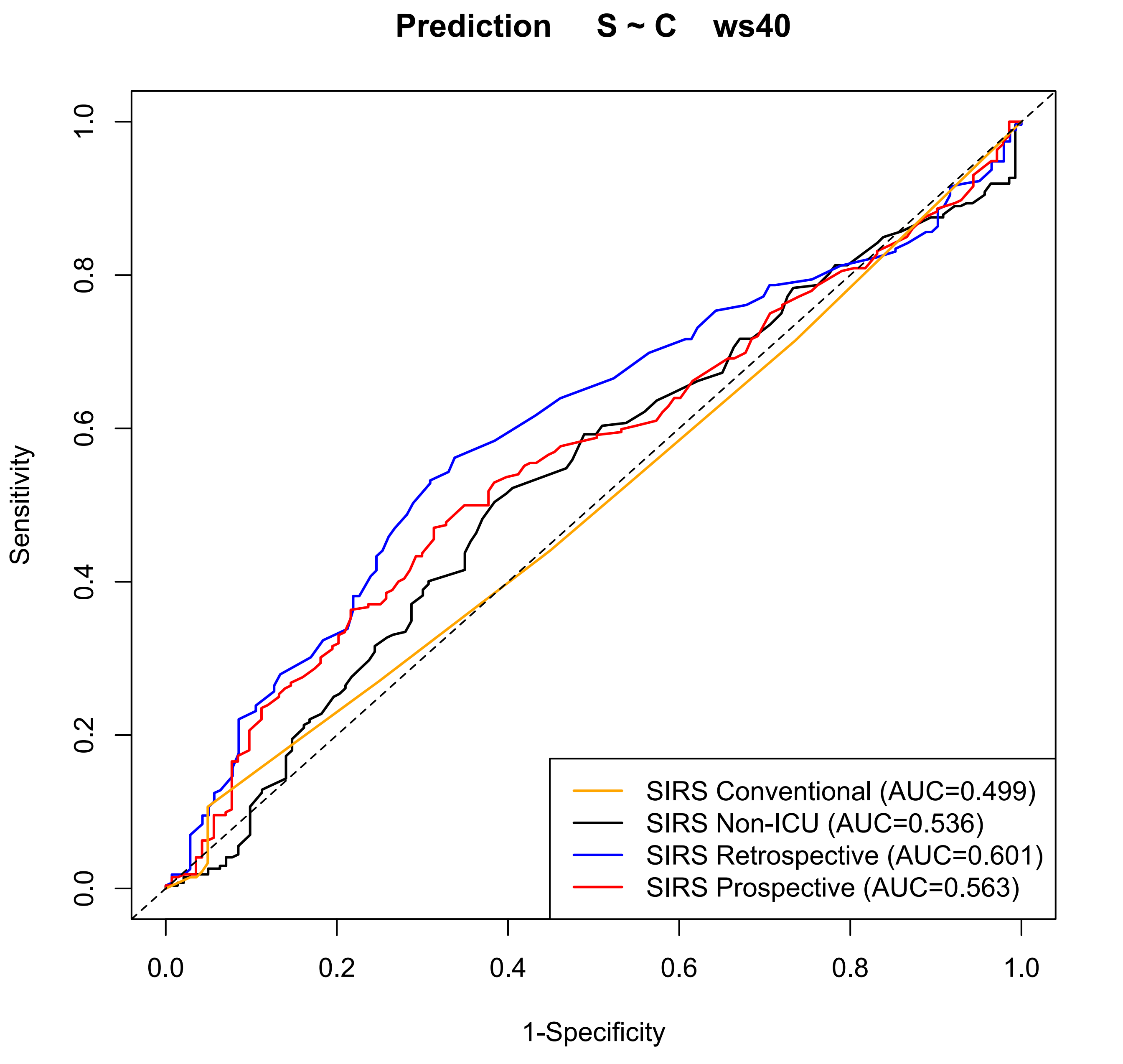

Prediction S ~ C ws40
Sensitivity
1-Specificity
0.0
0.2
0.4
0.6
0.8
1.0
0.0
0.2
0.4
0.6
0.8
1.0
SIRS Conventional (AUC=0.499)
SIRS Non-ICU (AUC=0.536)
SIRS Retrospective (AUC=0.601)
SIRS Prospective (AUC=0.563)

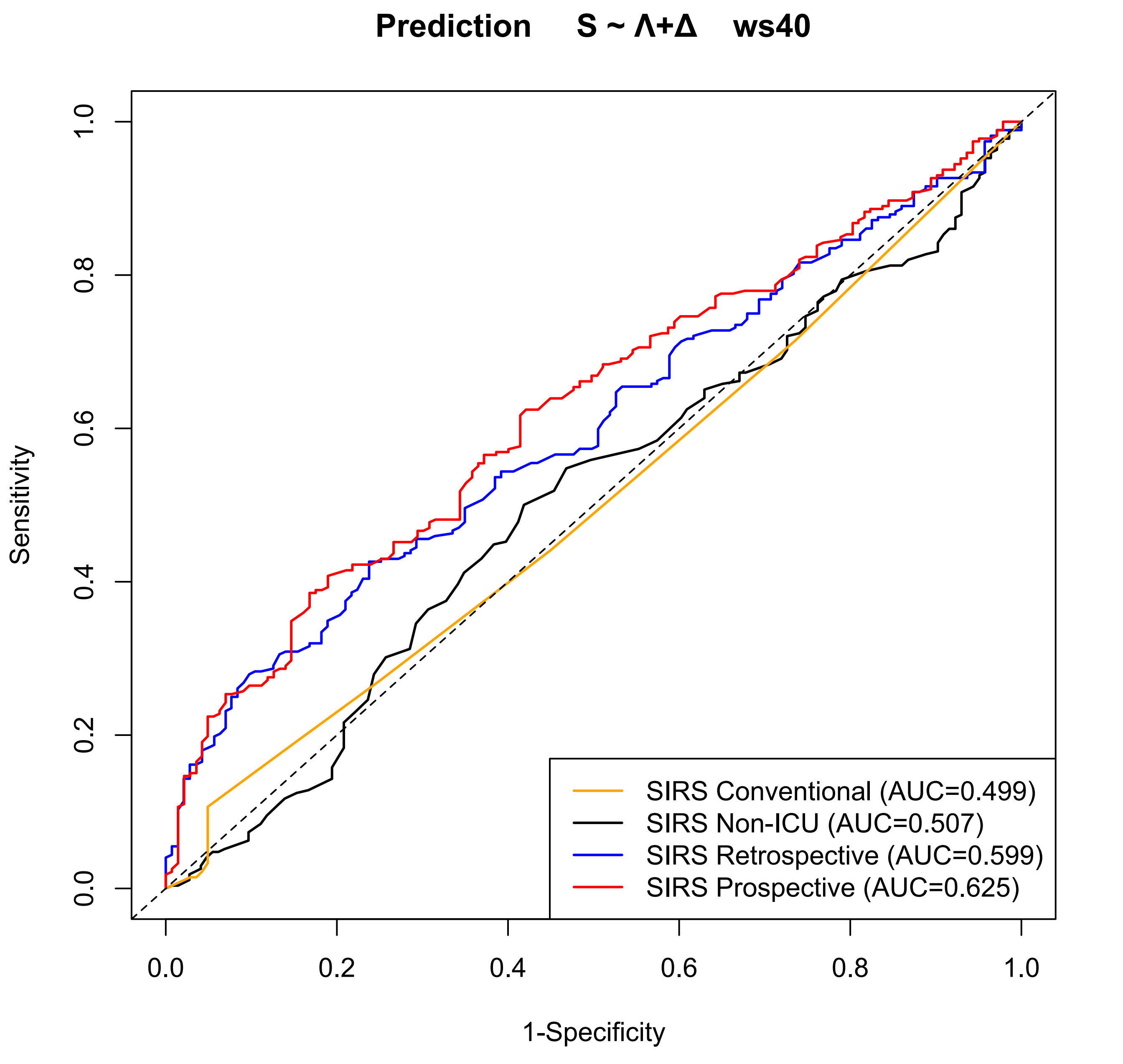

Prediction S ~ Λ+Δ ws40
Sensitivity
1-Specificity
0.0
0.2
0.4
0.6
0.8
1.0
SIRS Conventional (AUC=0.499)
SIRS Non-ICU (AUC=0.507)
SIRS Retrospective (AUC=0.599)
SIRS Prospective (AUC=0.625)

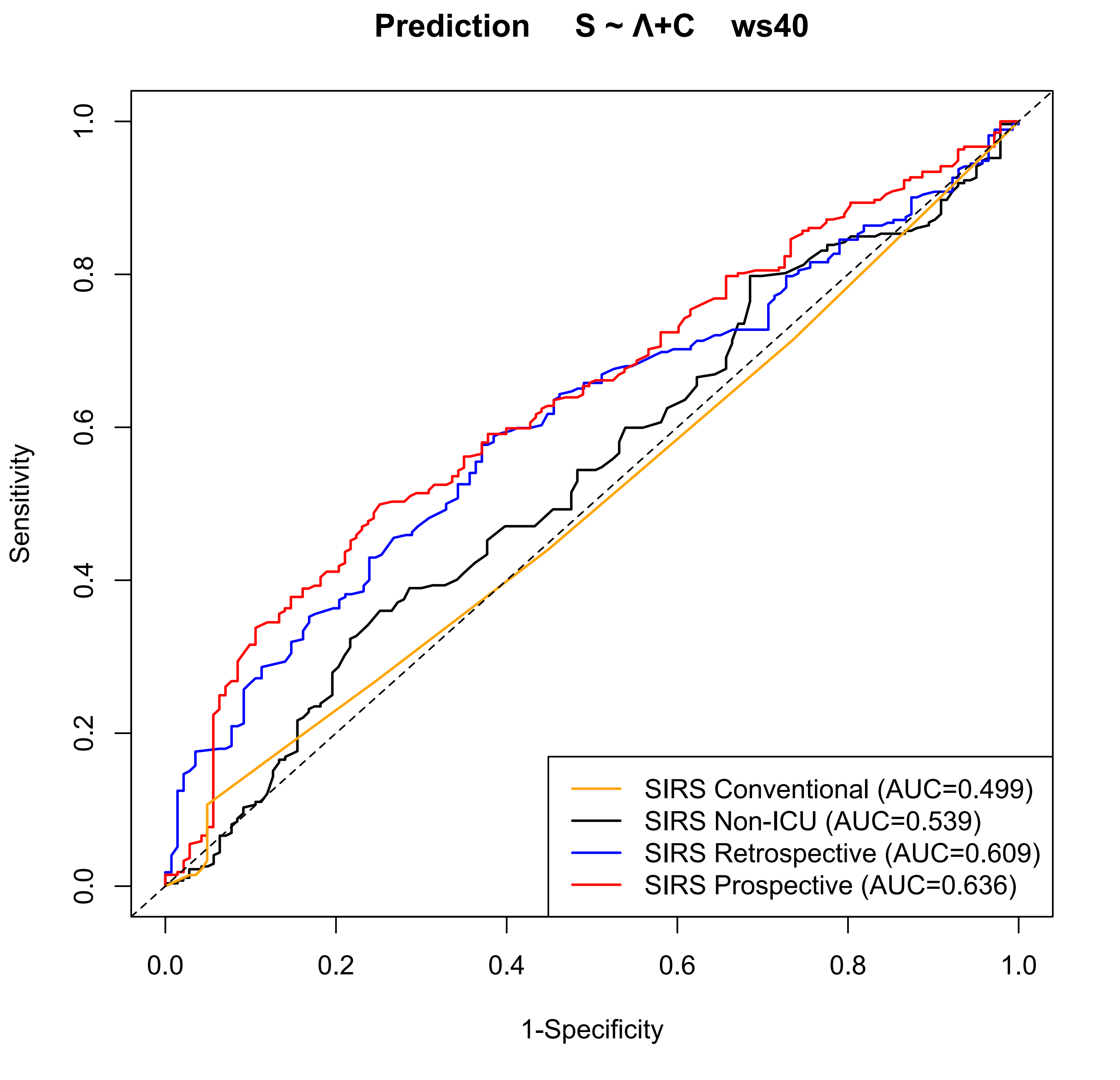
Prediction S ~ Λ+C ws40
Sensitivity
1-Specificity
0.0
0.2
0.4
0.6
0.8
1.0
SIRS Conventional (AUC=0.499)
SIRS Non-ICU (AUC=0.539)
SIRS Retrospective (AUC=0.609)
SIRS Prospective (AUC=0.636)

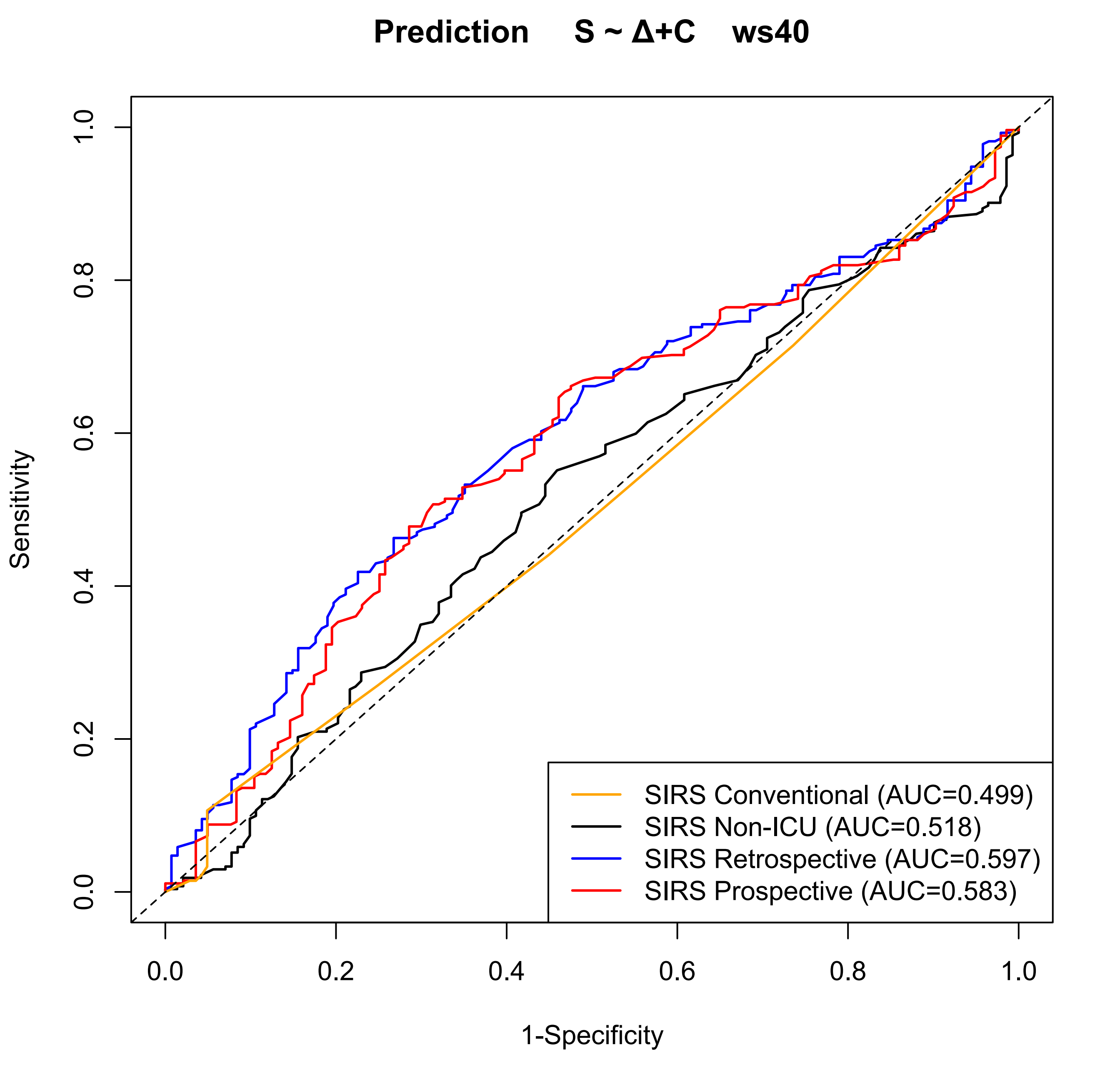
Prediction S ~ Δ+C ws40
Sensitivity
1-Specificity
0.0
0.2
0.4
0.6
0.8
1.0
SIRS Conventional (AUC=0.499)
SIRS Non-ICU (AUC=0.518)
SIRS Retrospective (AUC=0.597)
SIRS Prospective (AUC=0.583)

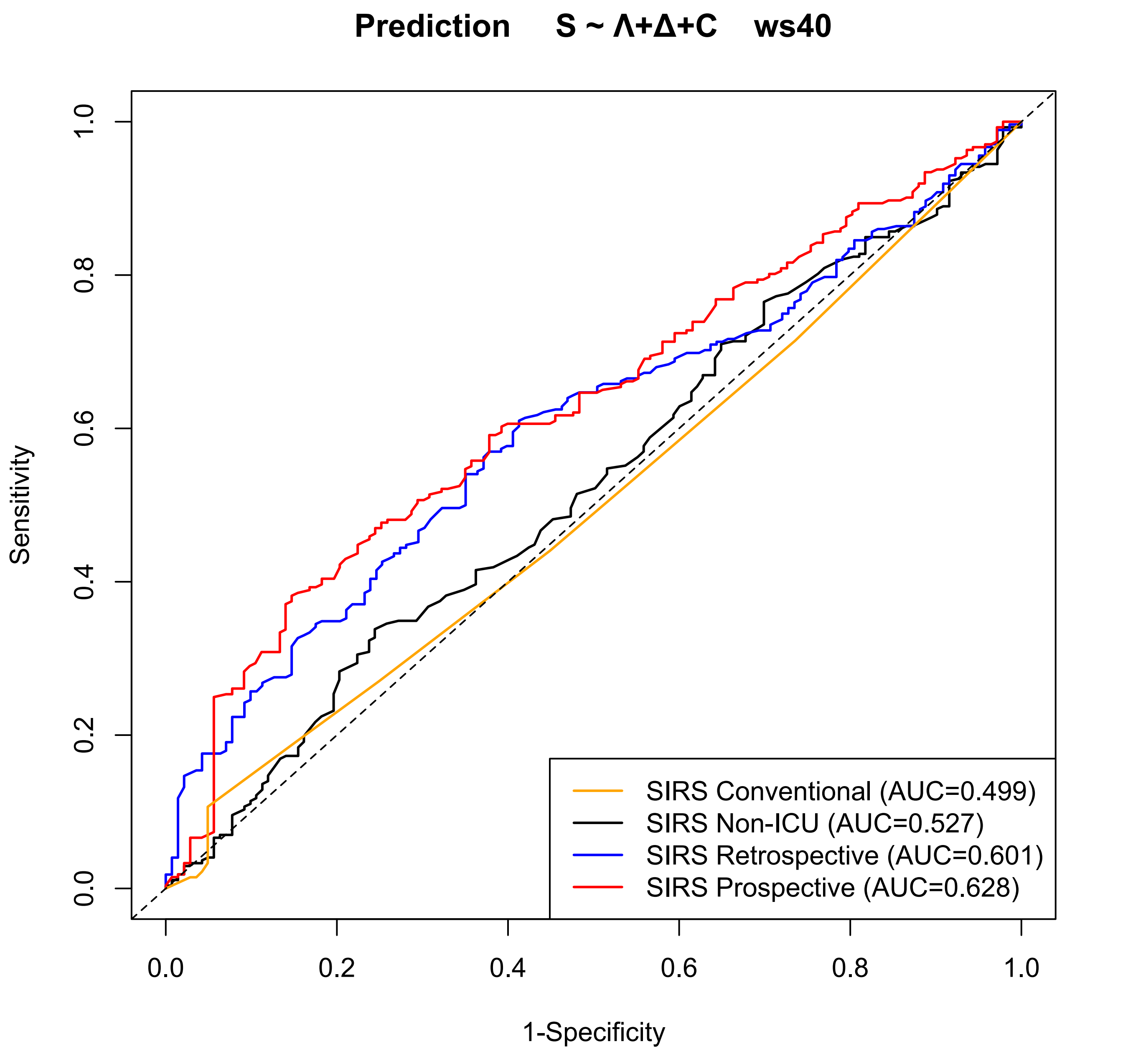
Prediction S ~ Λ+Δ+C ws40
1.0
0.8
0.6
0.4
0.2
0.0
Sensitivity
0.0
0.2
0.4
0.6
0.8
1.0
1-Specificity
SIRS Conventional (AUC=0.499)
SIRS Non-ICU (AUC=0.527)
SIRS Retrospective (AUC=0.601)
SIRS Prospective (AUC=0.628)

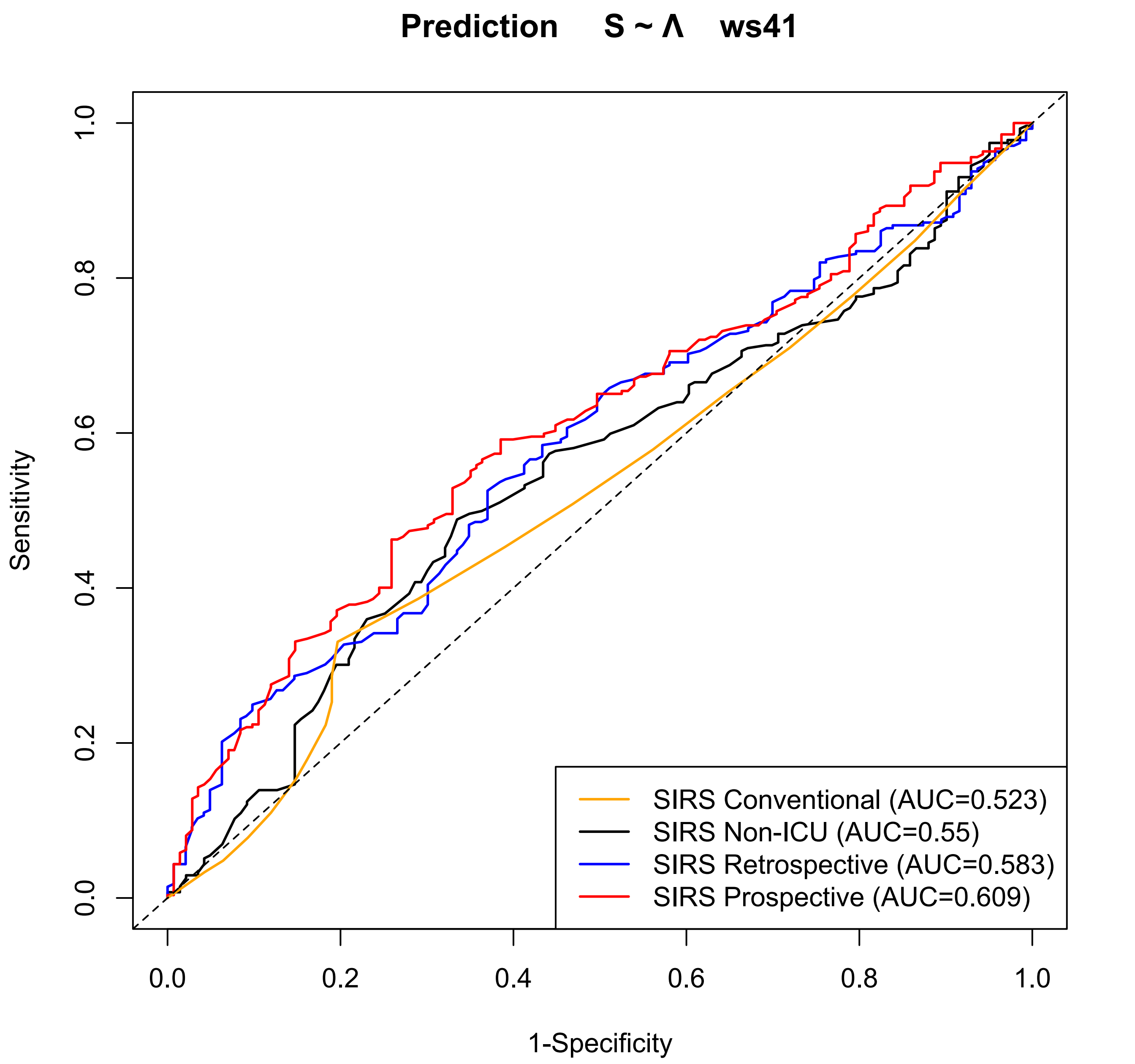
Prediction S ~ Λ ws41
Sensitivity
1-Specificity
0.0
0.2
0.4
0.6
0.8
1.0
0.0
0.2
0.4
0.6
0.8
1.0
SIRS Conventional (AUC=0.523)
SIRS Non-ICU (AUC=0.55)
SIRS Retrospective (AUC=0.583)
SIRS Prospective (AUC=0.609)

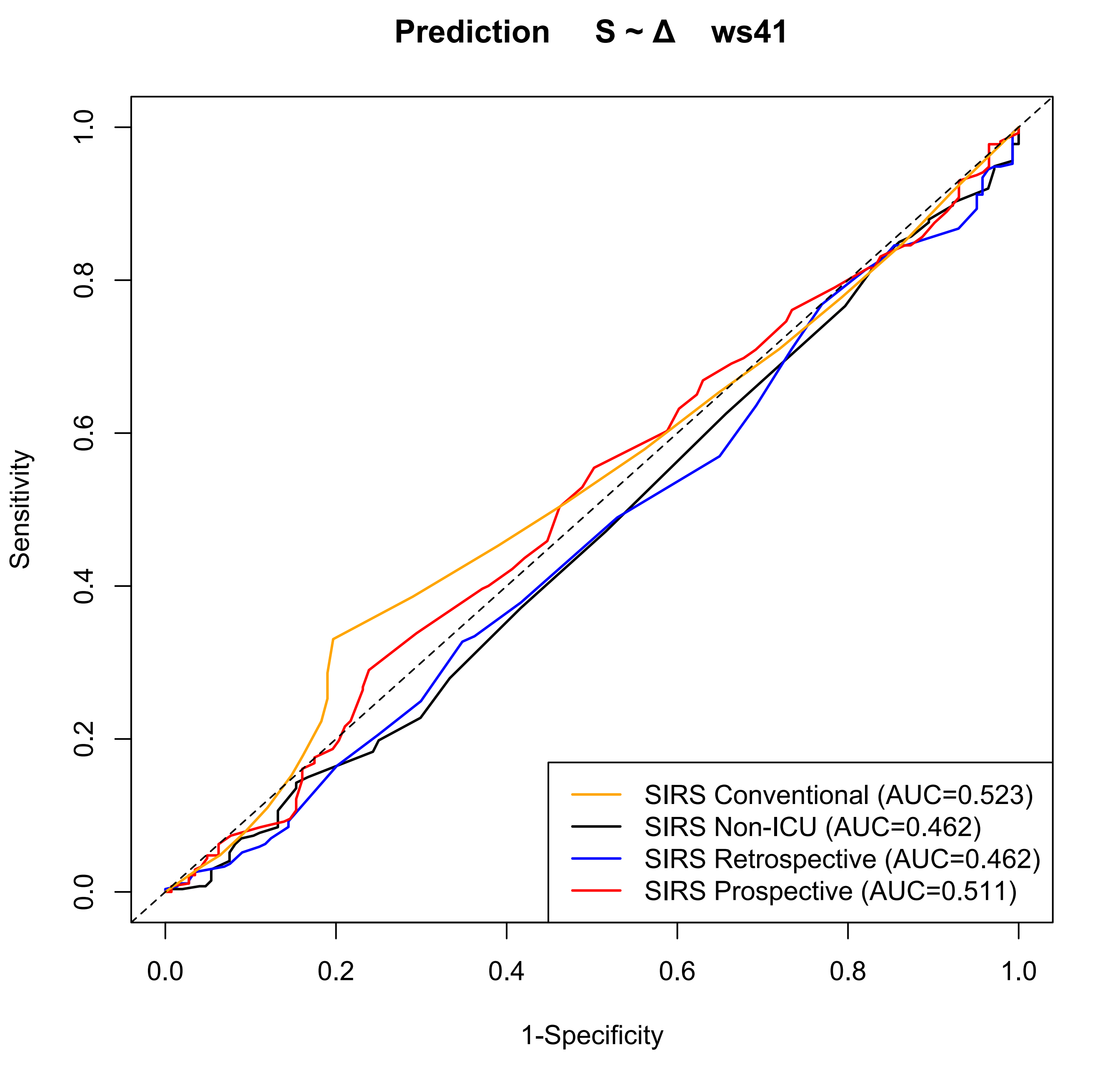
Prediction S ~ Δ ws41
Sensitivity
1-Specificity
0.0
0.2
0.4
0.6
0.8
1.0
0.0
0.2
0.4
0.6
0.8
1.0
SIRS Conventional (AUC=0.523)
SIRS Non-ICU (AUC=0.462)
SIRS Retrospective (AUC=0.462)
SIRS Prospective (AUC=0.511)

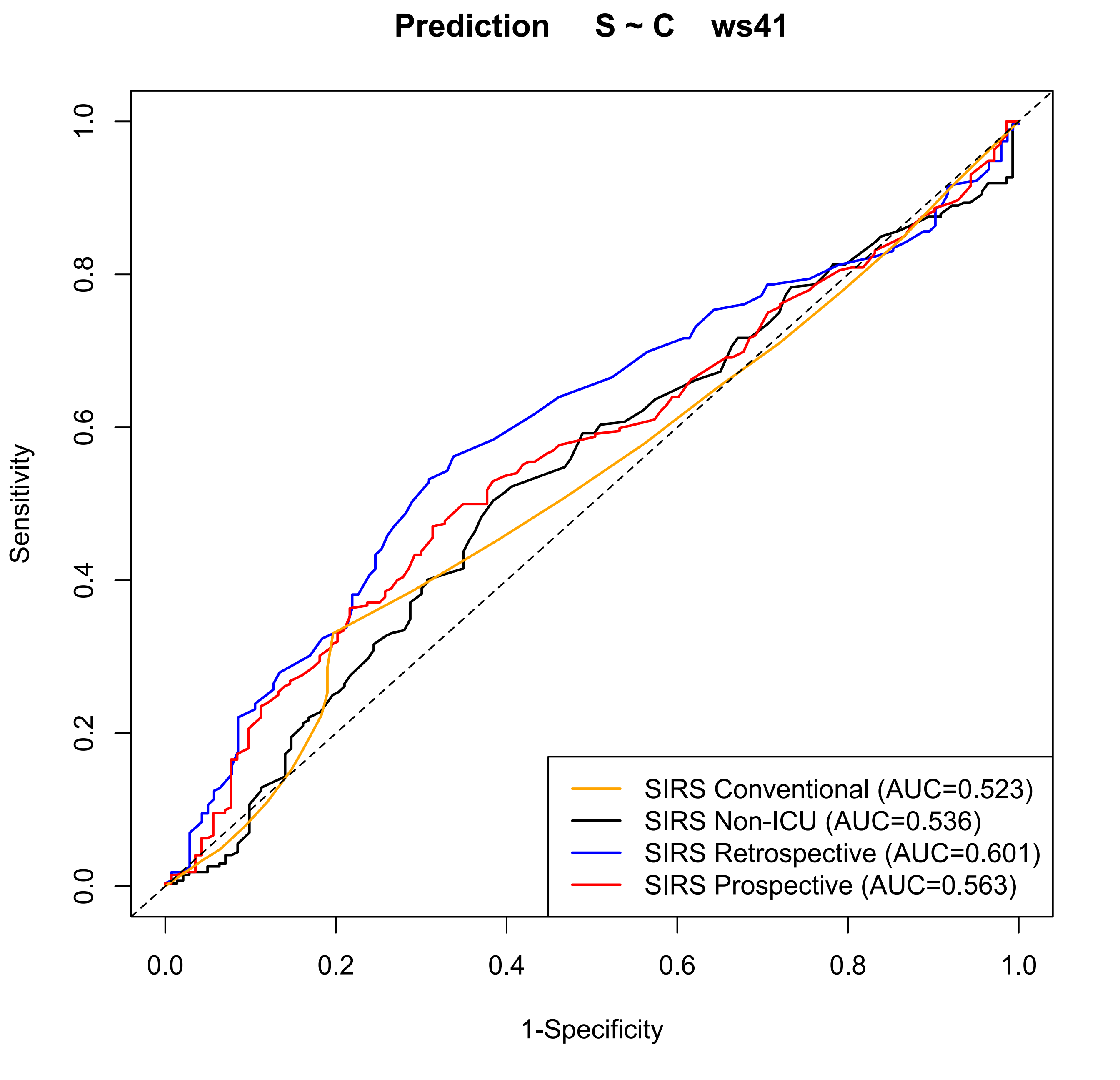

Prediction S ~ C ws41
Sensitivity
1-Specificity
0.0
0.2
0.4
0.6
0.8
1.0
SIRS Conventional (AUC=0.523)
SIRS Non-ICU (AUC=0.536)
SIRS Retrospective (AUC=0.601)
SIRS Prospective (AUC=0.563)

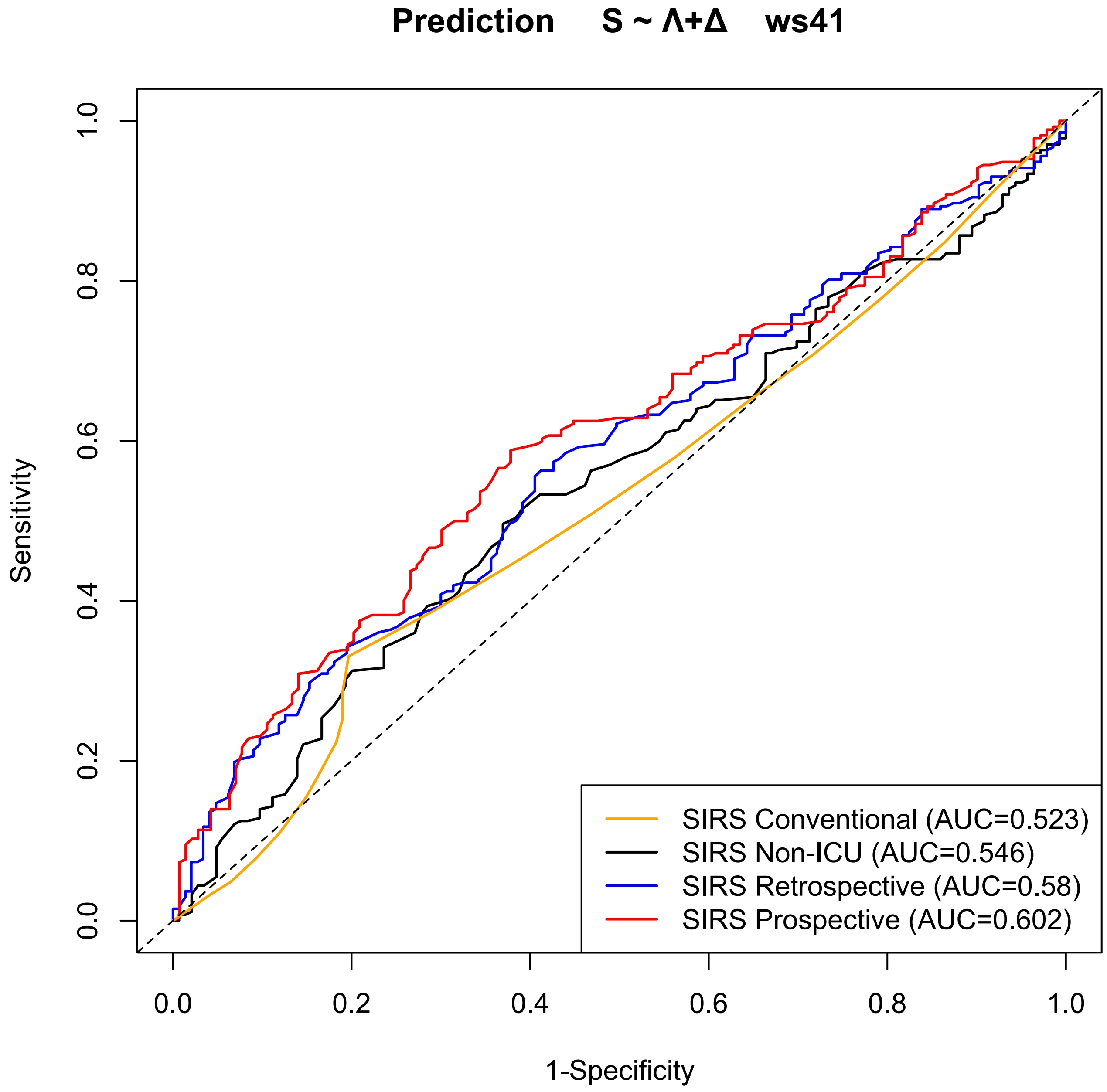

Prediction S ~ Λ+Δ ws41
Sensitivity
1-Specificity
0.0
0.2
0.4
0.6
0.8
1.0
SIRS Conventional (AUC=0.523)
SIRS Non-ICU (AUC=0.546)
SIRS Retrospective (AUC=0.58)
SIRS Prospective (AUC=0.602)

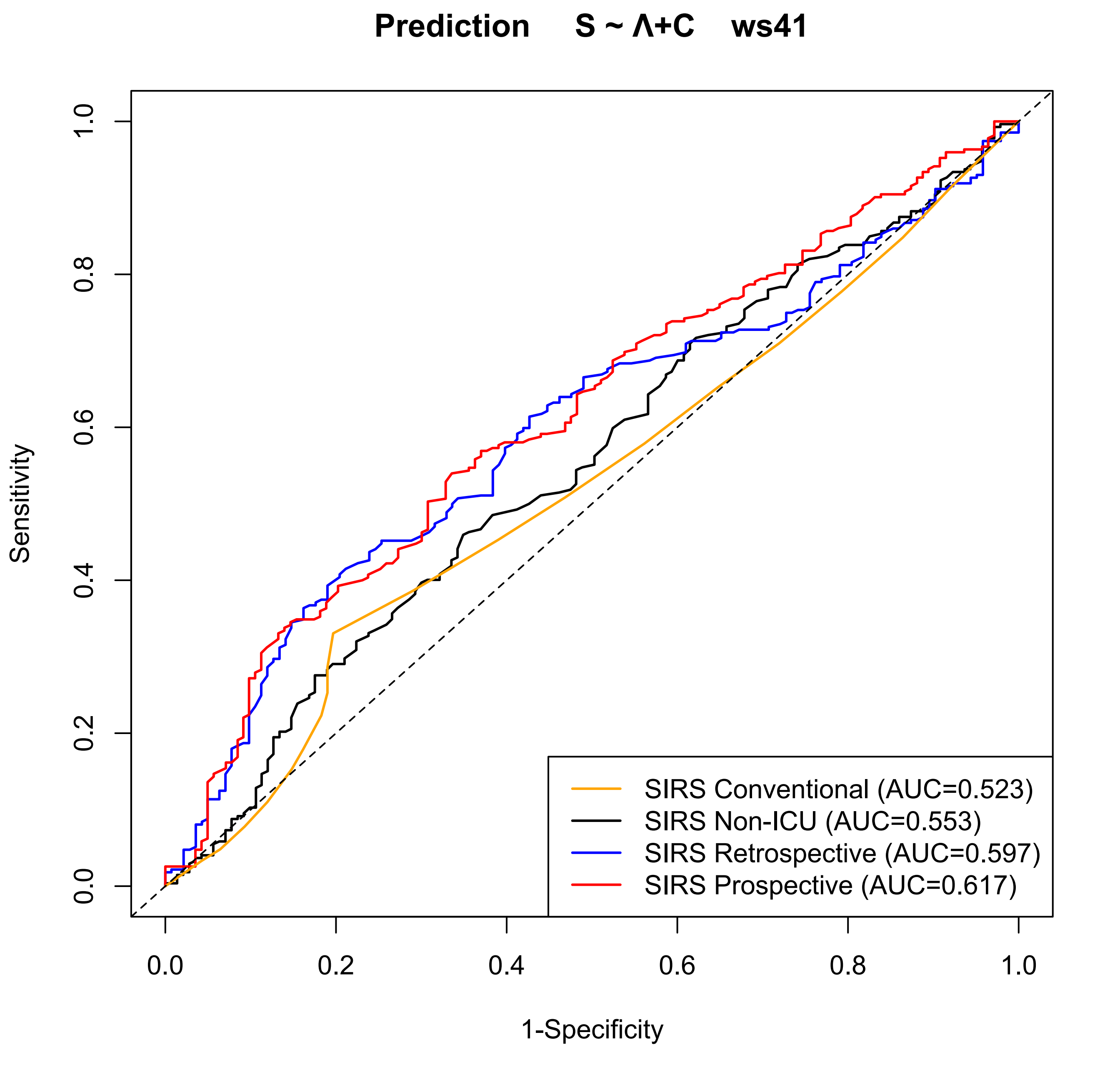

Prediction S ~ Λ+C ws41
Sensitivity
1-Specificity
0.0
0.2
0.4
0.6
0.8
1.0
0.0
0.2
0.4
0.6
0.8
1.0
SIRS Conventional (AUC=0.523)
SIRS Non-ICU (AUC=0.553)
SIRS Retrospective (AUC=0.597)
SIRS Prospective (AUC=0.617)

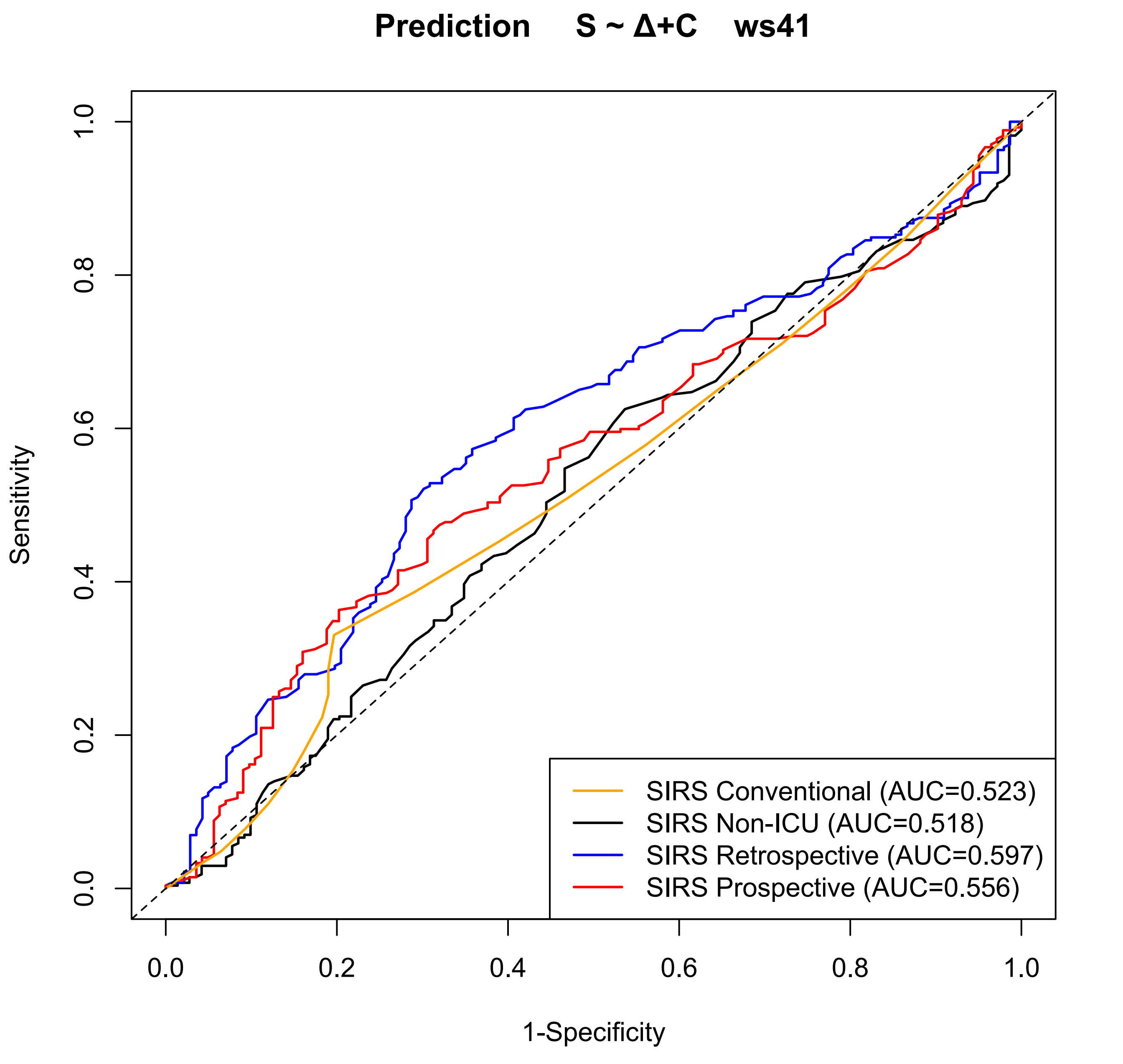

Prediction S ~ Δ+C ws41
Sensitivity
1-Specificity
0.0
0.2
0.4
0.6
0.8
1.0
SIRS Conventional (AUC=0.523)
SIRS Non-ICU (AUC=0.518)
SIRS Retrospective (AUC=0.597)
SIRS Prospective (AUC=0.556)

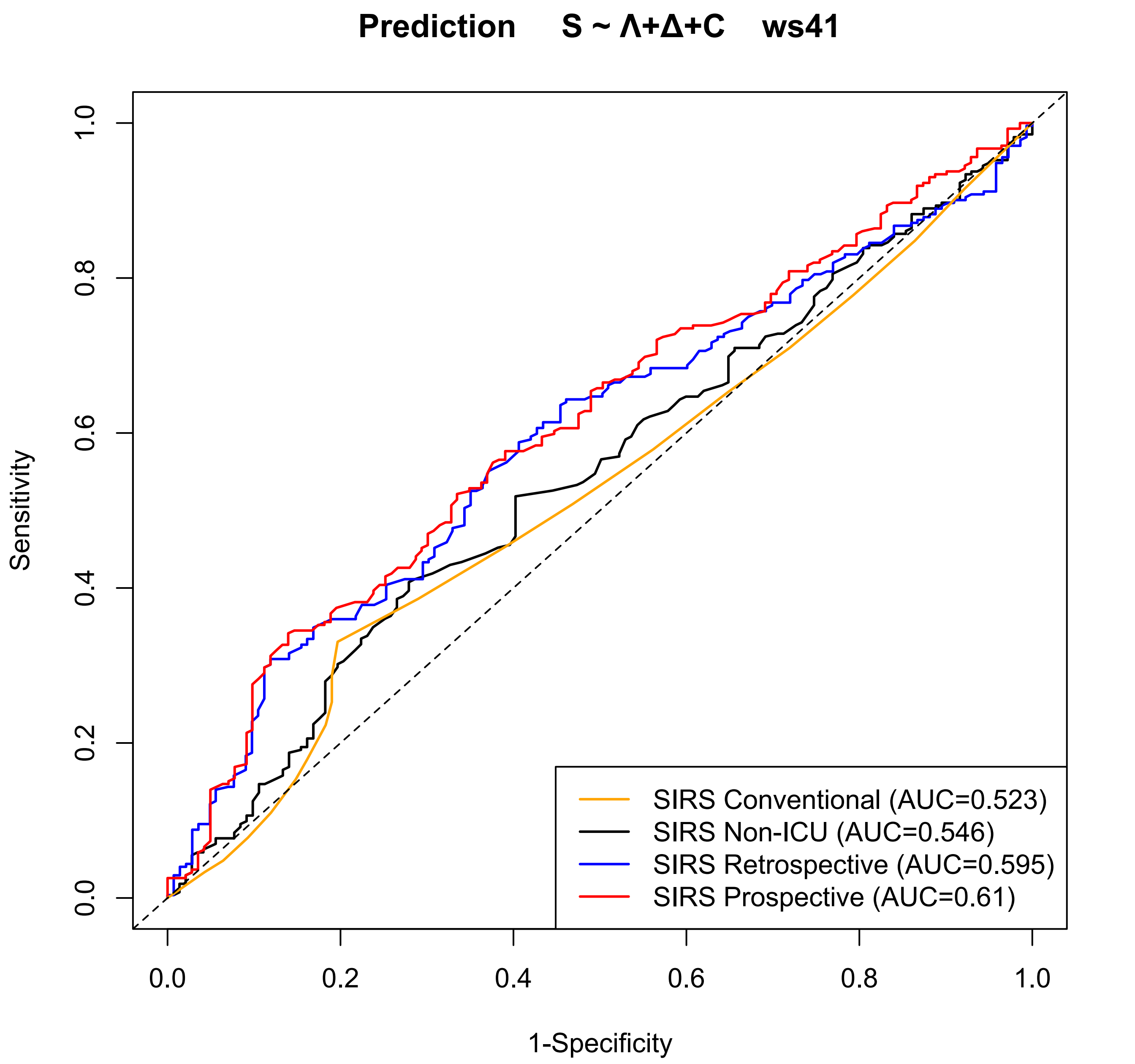

Prediction S ~ Λ+Δ+C ws41
Sensitivity
1-Specificity
0.0
0.2
0.4
0.6
0.8
1.0
SIRS Conventional (AUC=0.523)
SIRS Non-ICU (AUC=0.546)
SIRS Retrospective (AUC=0.595)
SIRS Prospective (AUC=0.61)

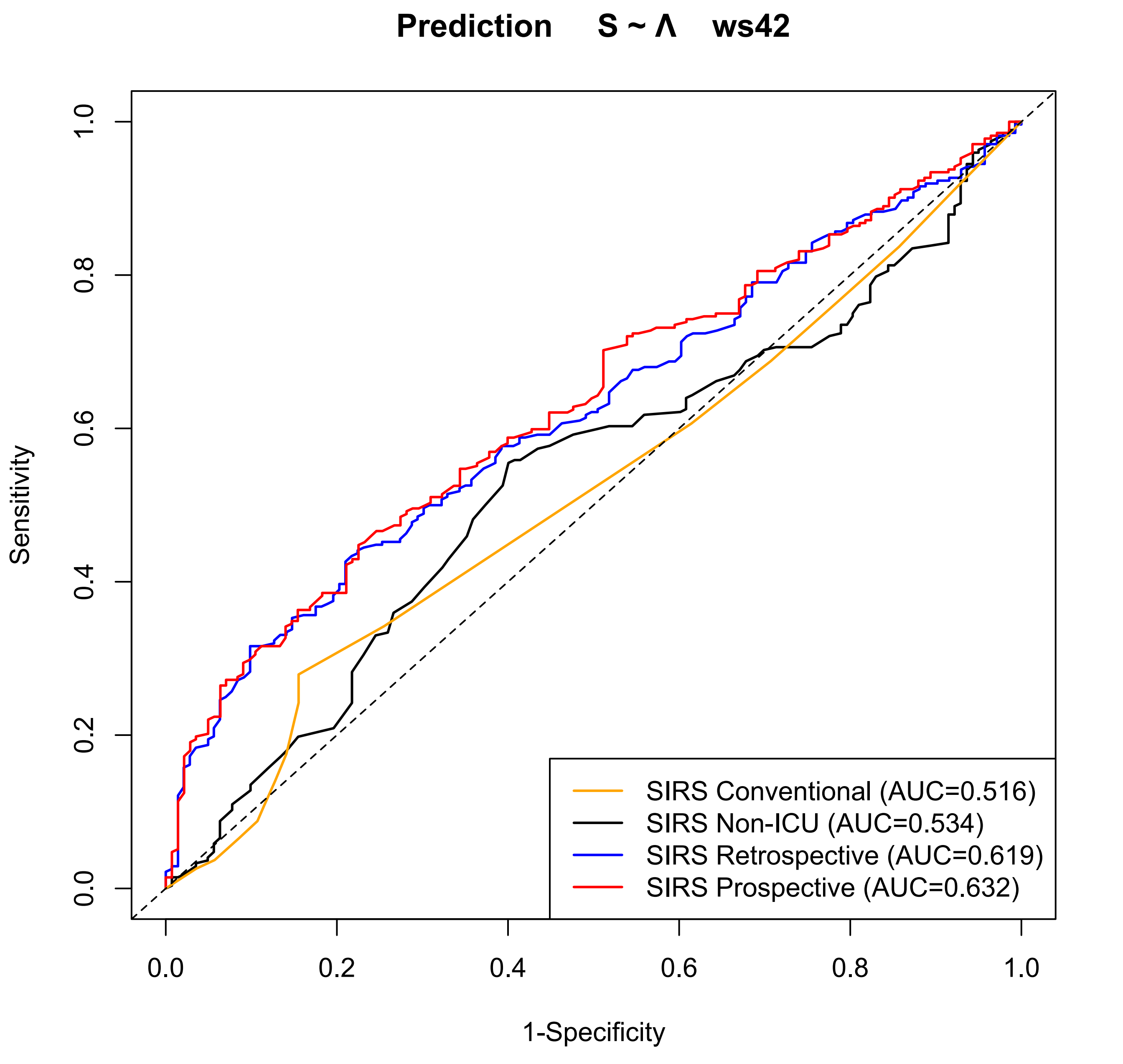

Prediction S ~ Λ ws42
Sensitivity
1-Specificity
0.0
0.2
0.4
0.6
0.8
1.0
0.0
0.2
0.4
0.6
0.8
1.0
SIRS Conventional (AUC=0.516)
SIRS Non-ICU (AUC=0.534)
SIRS Retrospective (AUC=0.619)
SIRS Prospective (AUC=0.632)

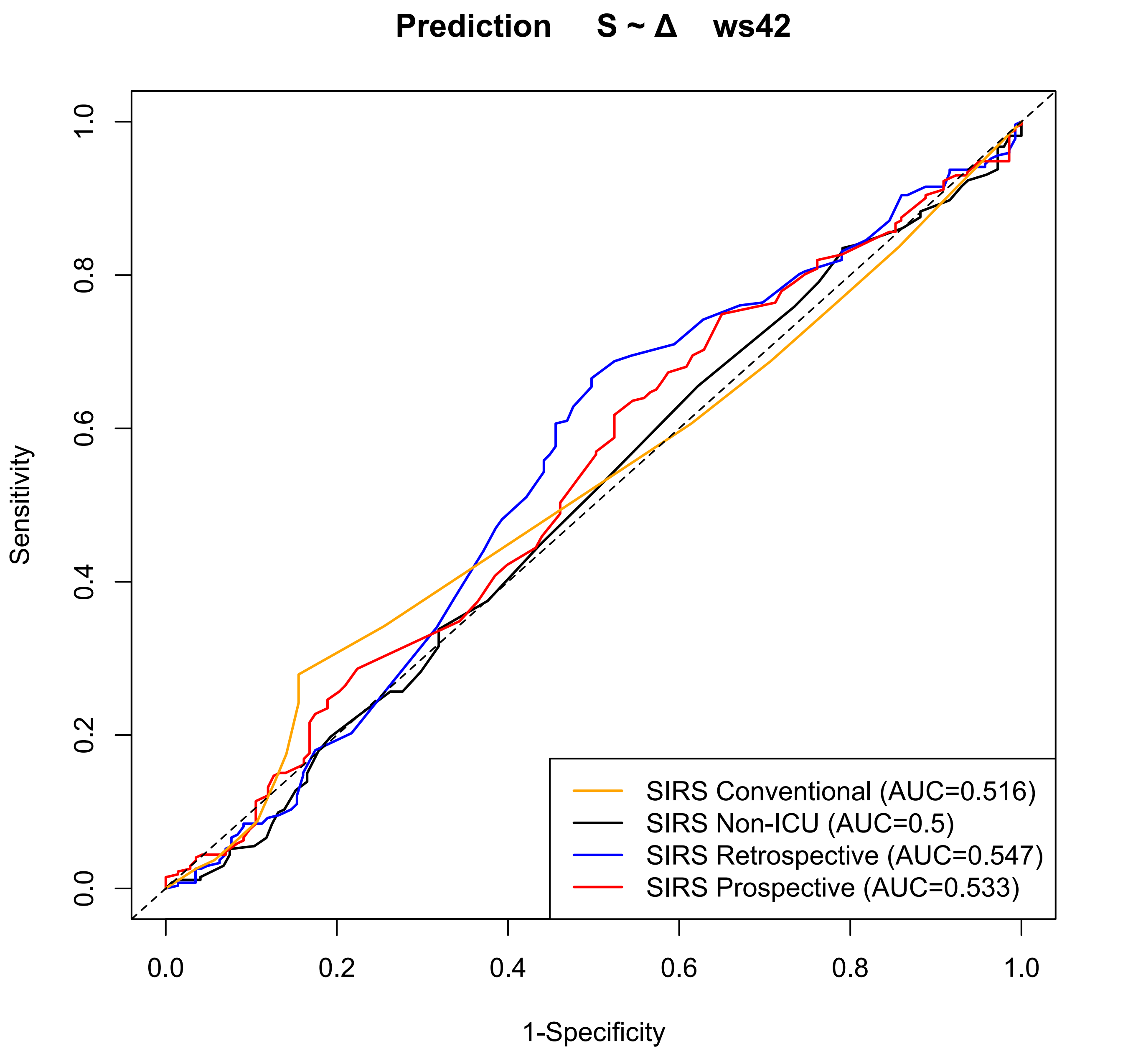

Prediction S ~ Δ ws42
Sensitivity
1-Specificity
0.0
0.2
0.4
0.6
0.8
1.0
0.0
0.2
0.4
0.6
0.8
1.0
SIRS Conventional (AUC=0.516)
SIRS Non-ICU (AUC=0.5)
SIRS Retrospective (AUC=0.547)
SIRS Prospective (AUC=0.533)

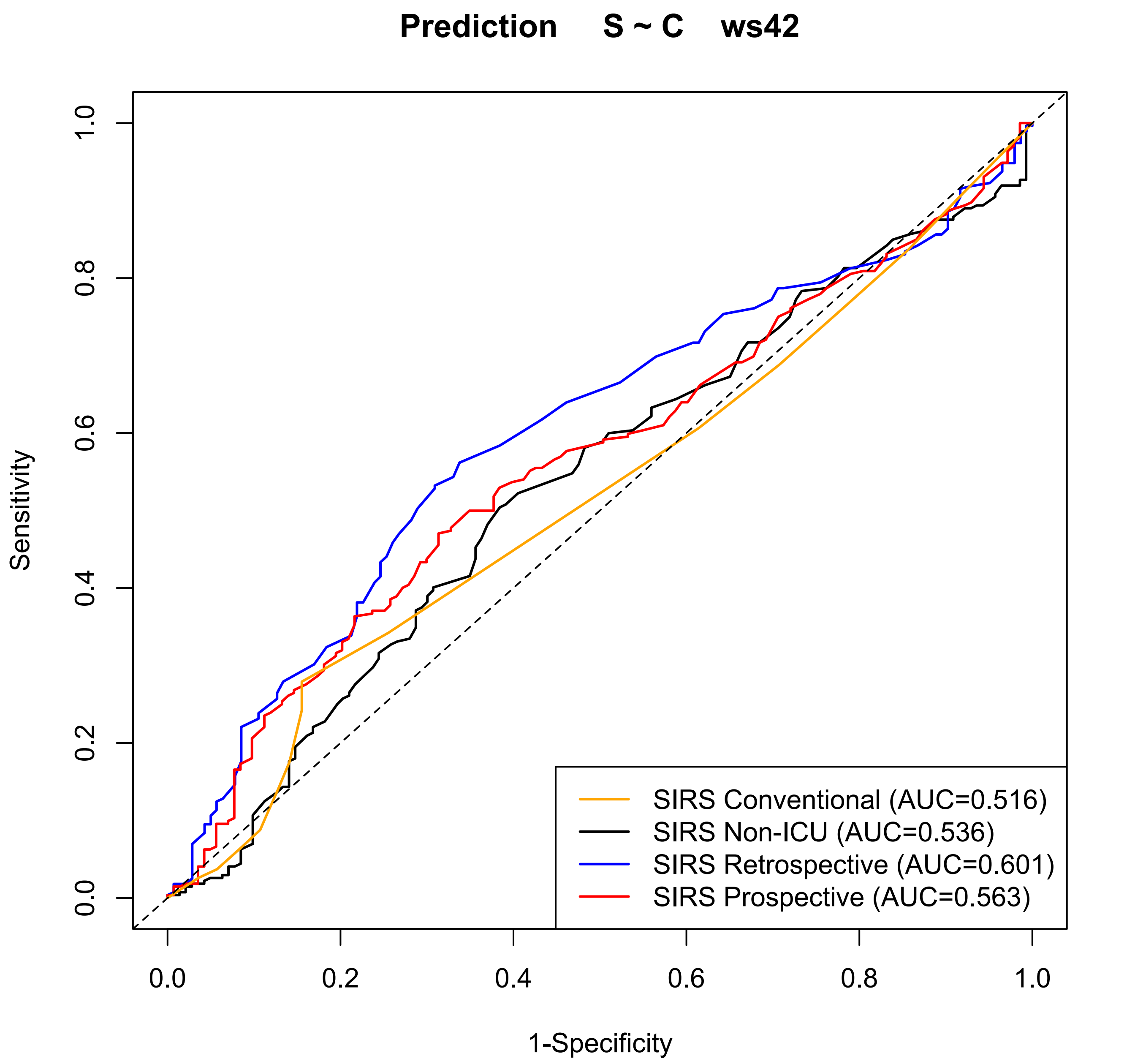

Prediction S ~ C ws42
Sensitivity
1-Specificity
0.0
0.2
0.4
0.6
0.8
1.0
SIRS Conventional (AUC=0.516)
SIRS Non-ICU (AUC=0.536)
SIRS Retrospective (AUC=0.601)
SIRS Prospective (AUC=0.563)

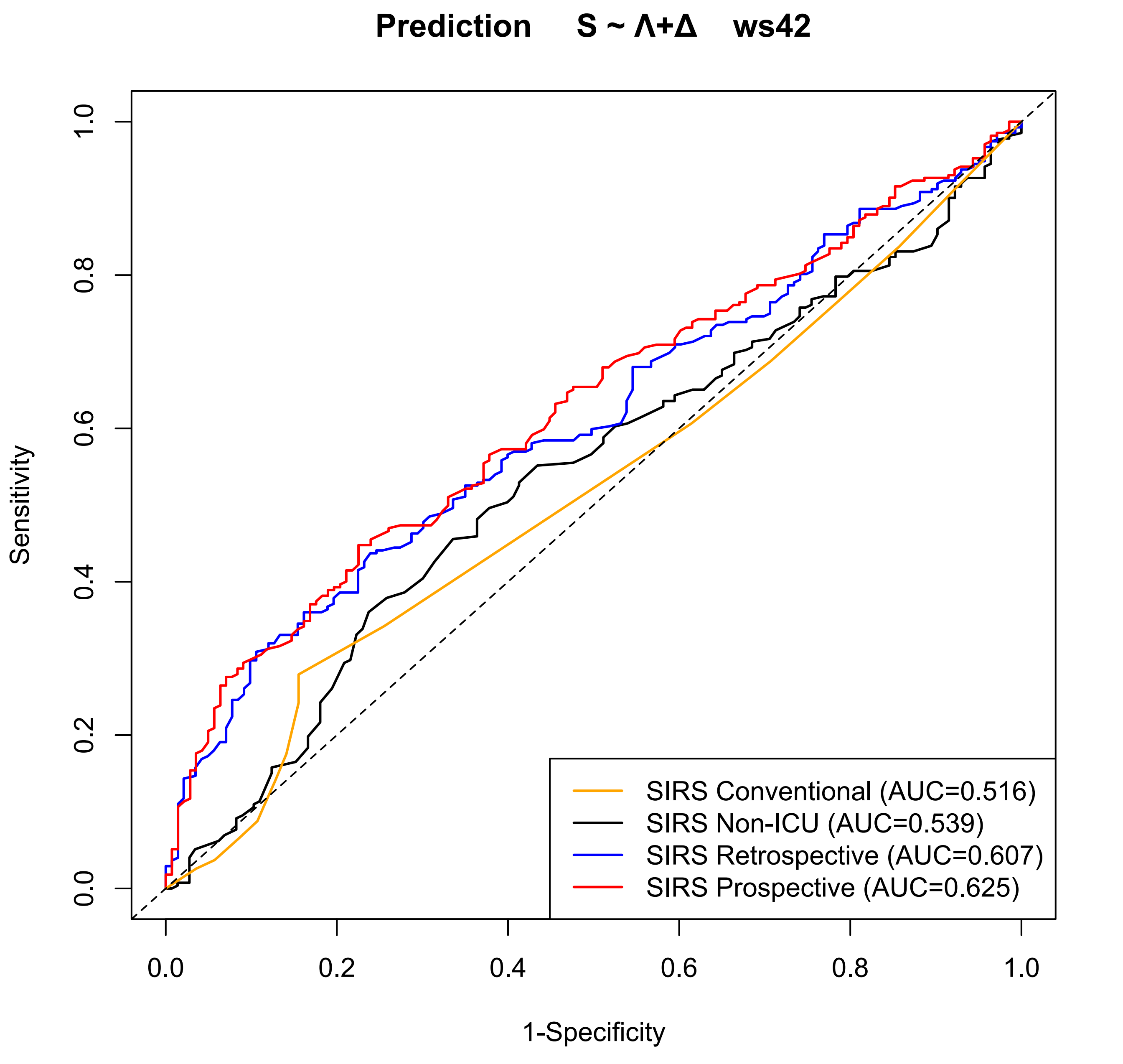

Prediction S ~ Λ+Δ ws42
Sensitivity
1-Specificity
0.0
0.2
0.4
0.6
0.8
1.0
SIRS Conventional (AUC=0.516)
SIRS Non-ICU (AUC=0.539)
SIRS Retrospective (AUC=0.607)
SIRS Prospective (AUC=0.625)

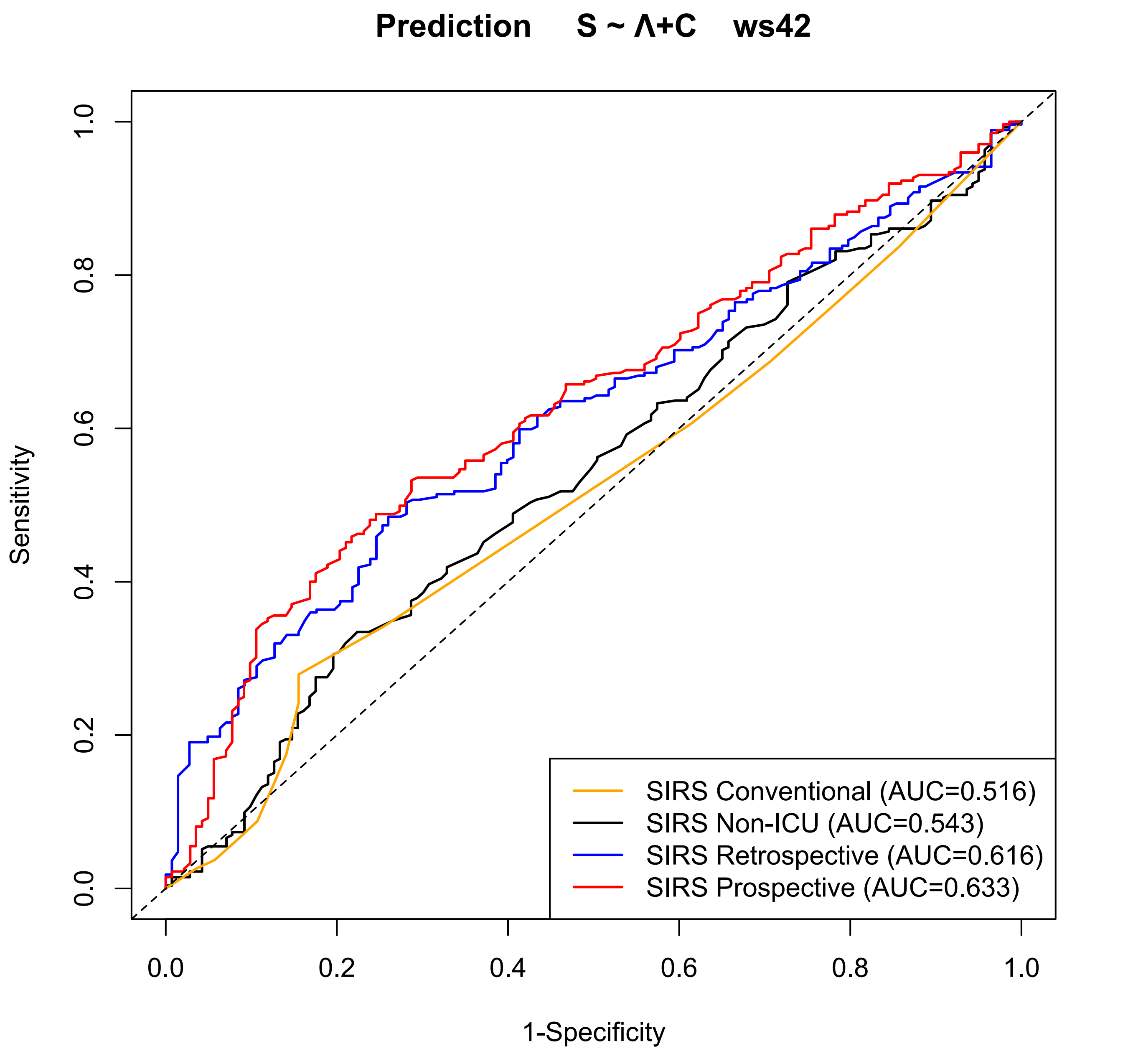

Prediction S ~ Λ+C ws42
Sensitivity
1-Specificity
0.0
0.2
0.4
0.6
0.8
1.0
SIRS Conventional (AUC=0.516)
SIRS Non-ICU (AUC=0.543)
SIRS Retrospective (AUC=0.616)
SIRS Prospective (AUC=0.633)

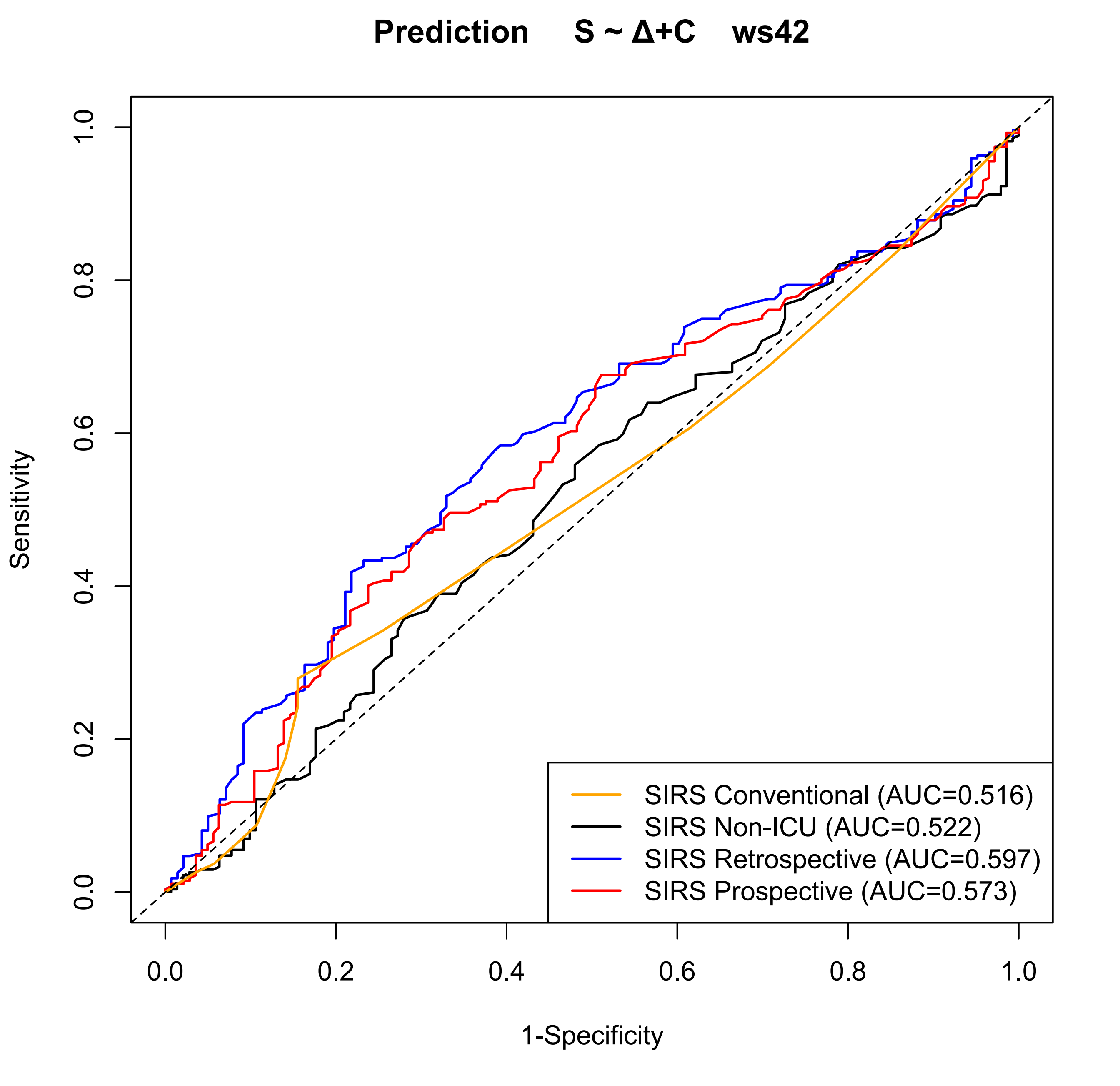

Prediction S ~ Δ+C ws42
Sensitivity
1-Specificity
0.0
0.2
0.4
0.6
0.8
1.0
SIRS Conventional (AUC=0.516)
SIRS Non-ICU (AUC=0.522)
SIRS Retrospective (AUC=0.597)
SIRS Prospective (AUC=0.573)

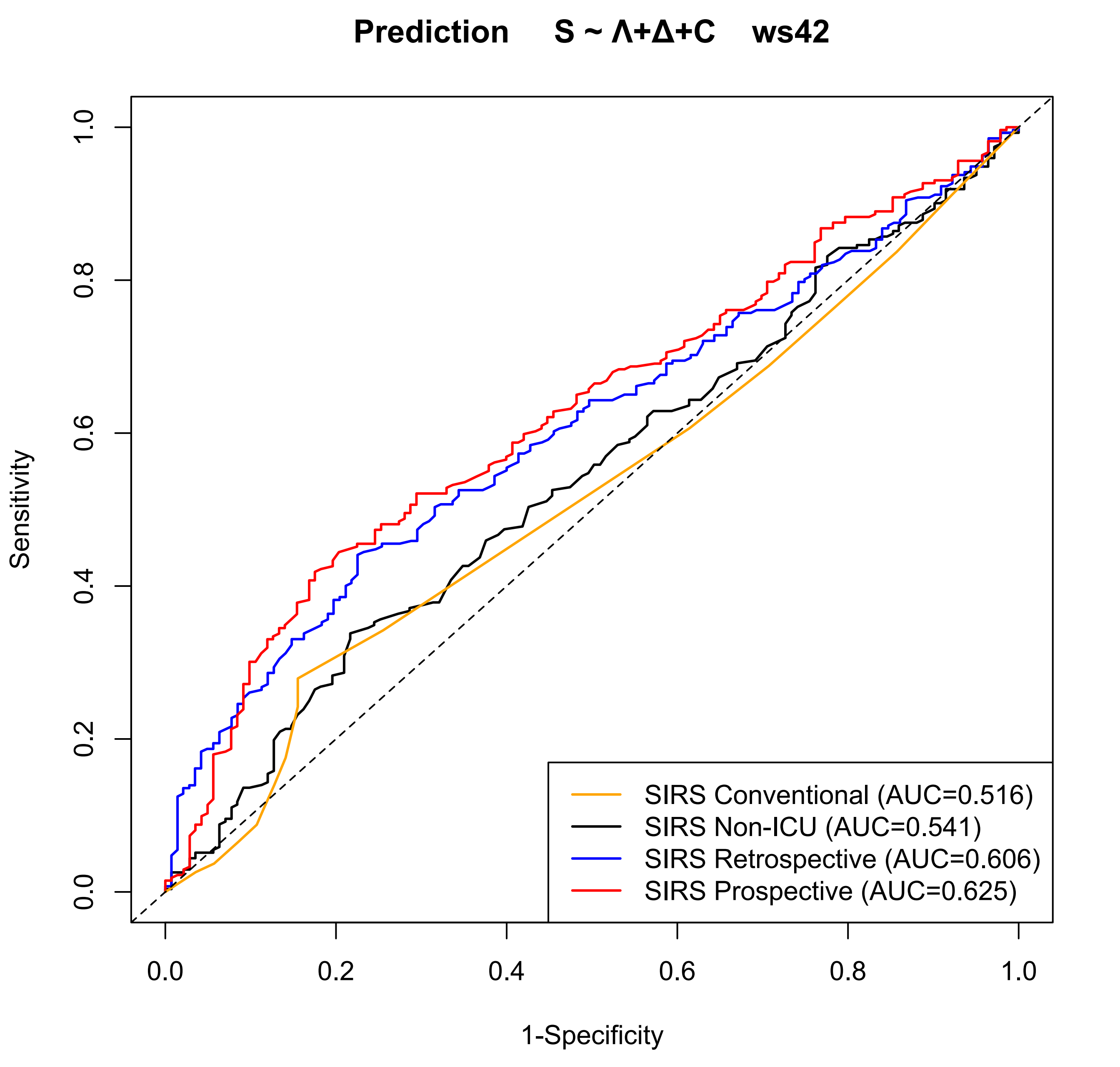
Prediction S ~ Λ+Δ+C ws42
Sensitivity
1-Specificity
0.0
0.2
0.4
0.6
0.8
1.0
SIRS Conventional (AUC=0.516)
SIRS Non-ICU (AUC=0.541)
SIRS Retrospective (AUC=0.606)
SIRS Prospective (AUC=0.625)

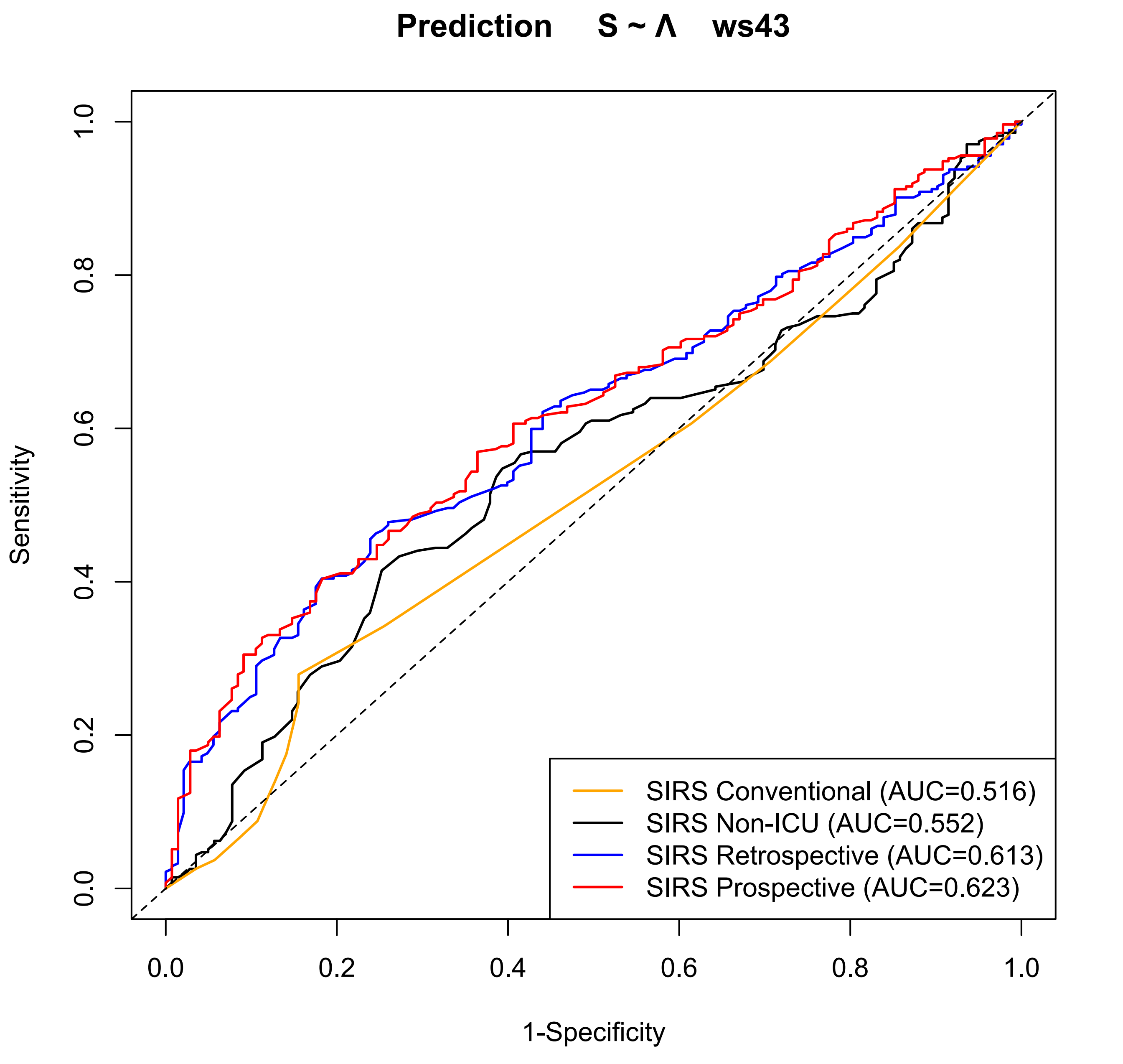

Prediction S ~ Λ ws43
Sensitivity
1-Specificity
0.0
0.2
0.4
0.6
0.8
1.0
SIRS Conventional (AUC=0.516)
SIRS Non-ICU (AUC=0.552)
SIRS Retrospective (AUC=0.613)
SIRS Prospective (AUC=0.623)

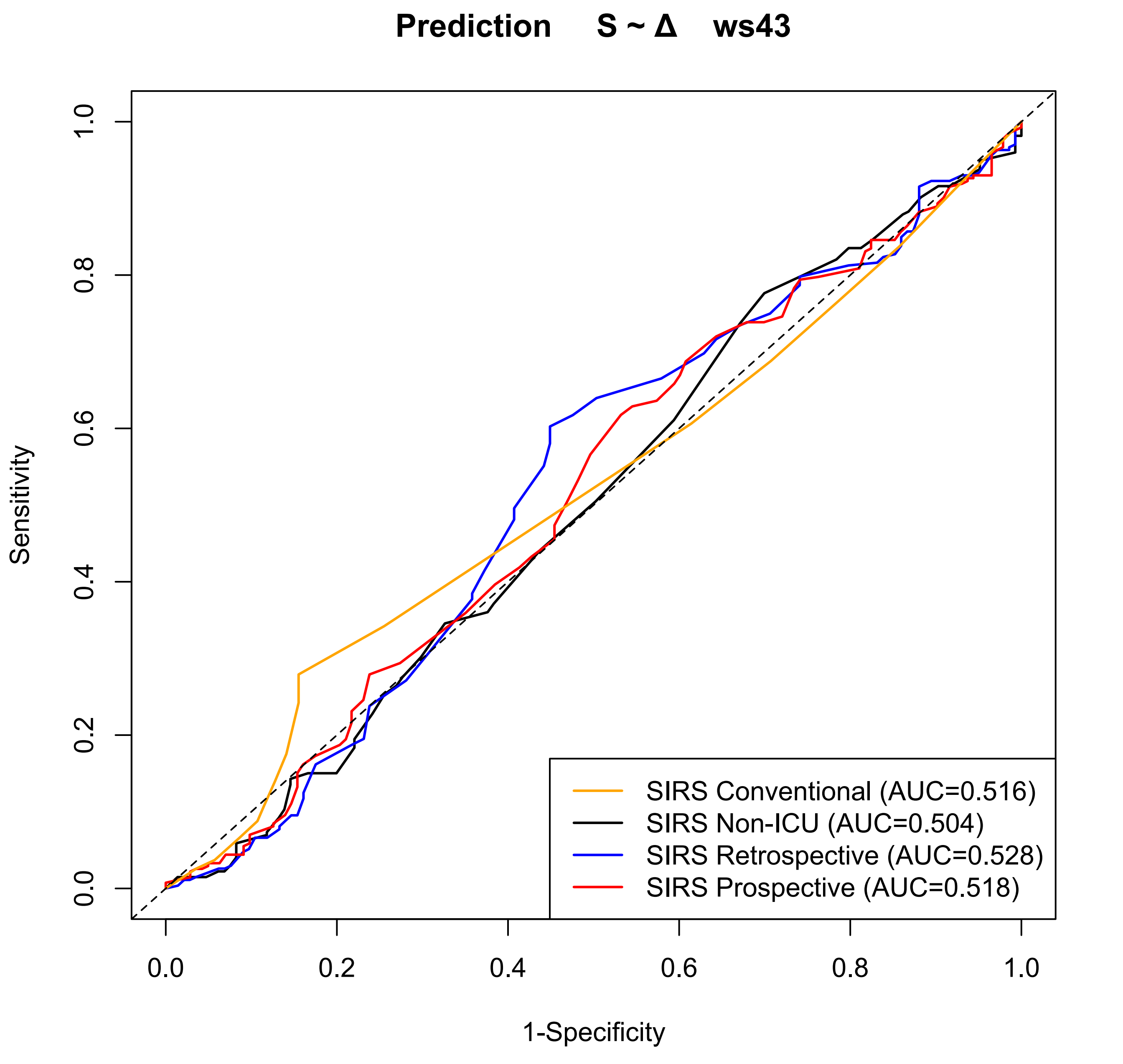

Prediction S ~ Δ ws43
Sensitivity
1-Specificity
0.0
0.2
0.4
0.6
0.8
1.0
SIRS Conventional (AUC=0.516)
SIRS Non-ICU (AUC=0.504)
SIRS Retrospective (AUC=0.528)
SIRS Prospective (AUC=0.518)

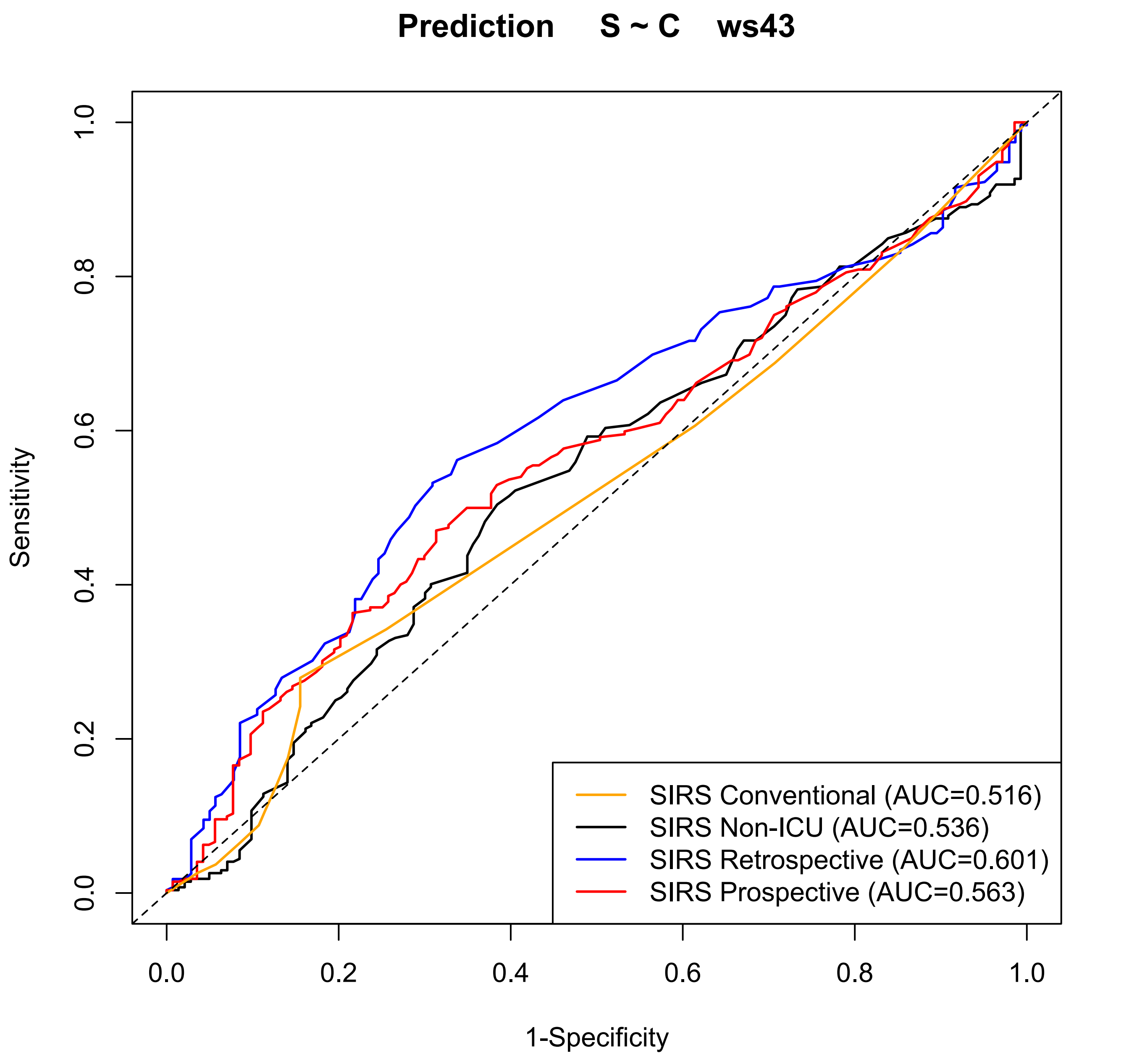
Prediction S ~ C ws43
Sensitivity
1-Specificity
0.0
0.2
0.4
0.6
0.8
1.0
SIRS Conventional (AUC=0.516)
SIRS Non-ICU (AUC=0.536)
SIRS Retrospective (AUC=0.601)
SIRS Prospective (AUC=0.563)

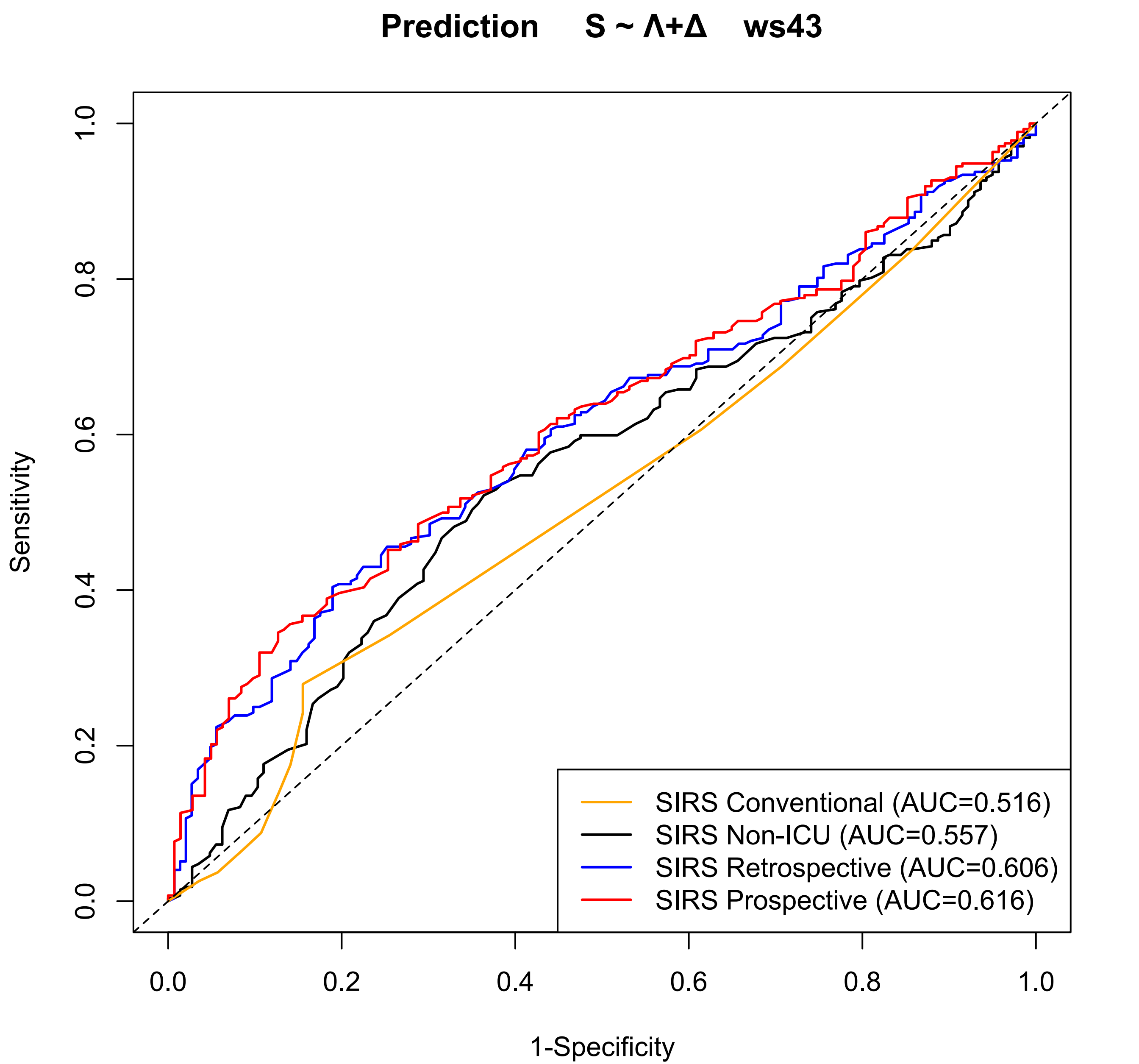

Prediction S ~ Λ+Δ ws43
Sensitivity
1-Specificity
0.0
0.2
0.4
0.6
0.8
1.0
SIRS Conventional (AUC=0.516)
SIRS Non-ICU (AUC=0.557)
SIRS Retrospective (AUC=0.606)
SIRS Prospective (AUC=0.616)

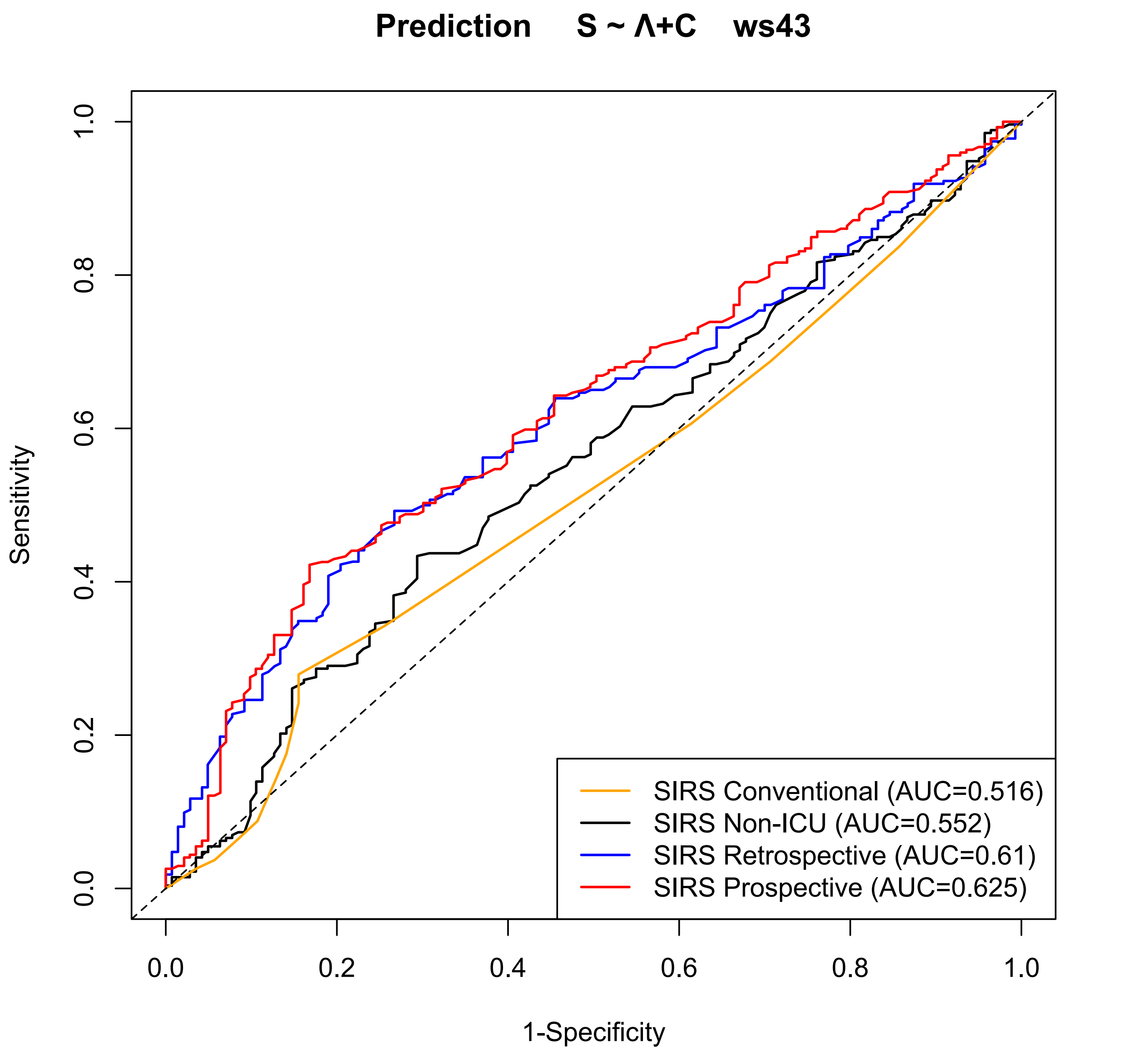

Prediction S ~ Λ+C ws43
Sensitivity
1-Specificity
0.0
0.2
0.4
0.6
0.8
1.0
SIRS Conventional (AUC=0.516)
SIRS Non-ICU (AUC=0.552)
SIRS Retrospective (AUC=0.61)
SIRS Prospective (AUC=0.625)

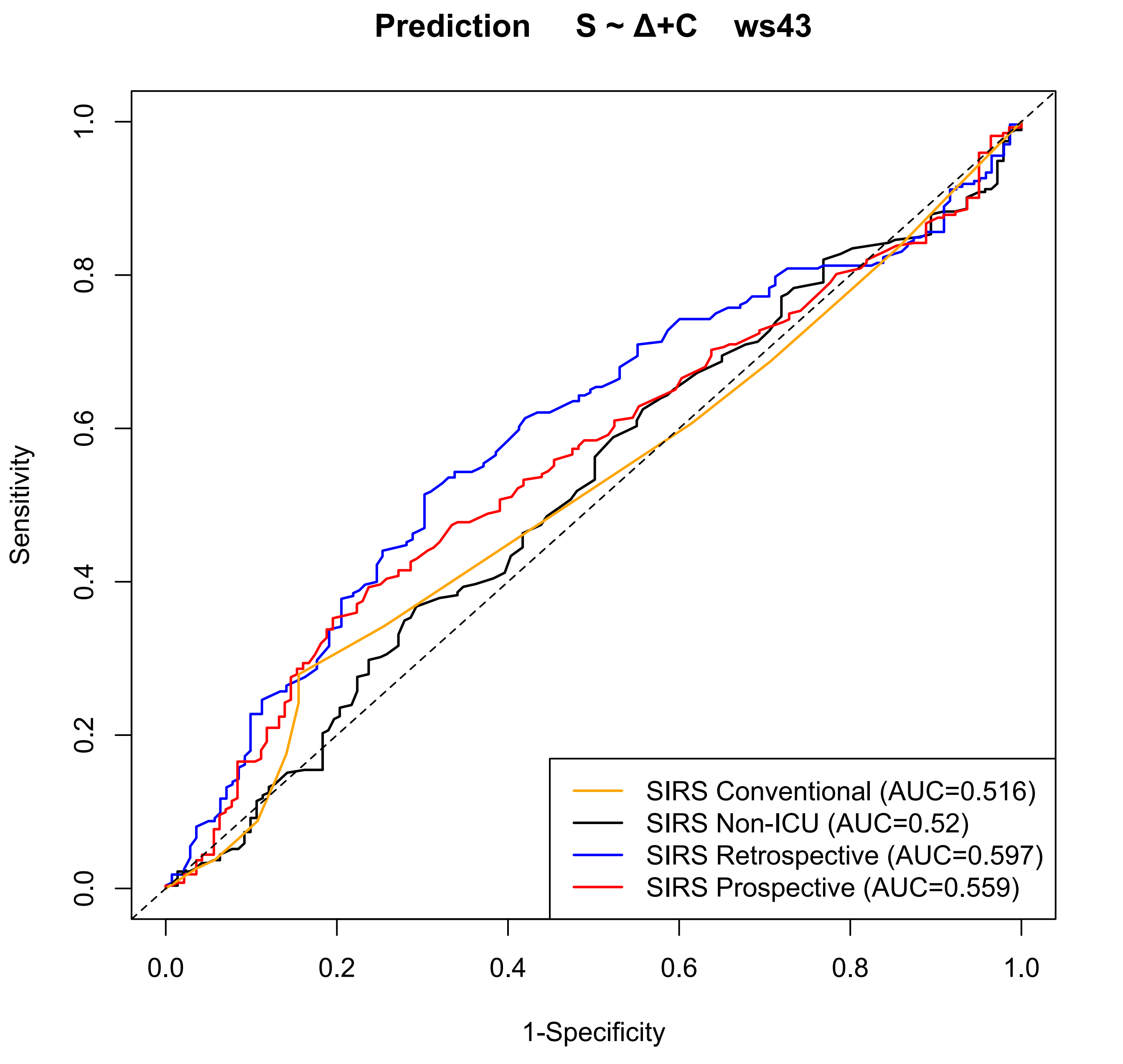

Prediction S ~ Δ+C ws43
Sensitivity
1-Specificity
0.0
0.2
0.4
0.6
0.8
1.0
SIRS Conventional (AUC=0.516)
SIRS Non-ICU (AUC=0.52)
SIRS Retrospective (AUC=0.597)
SIRS Prospective (AUC=0.559)

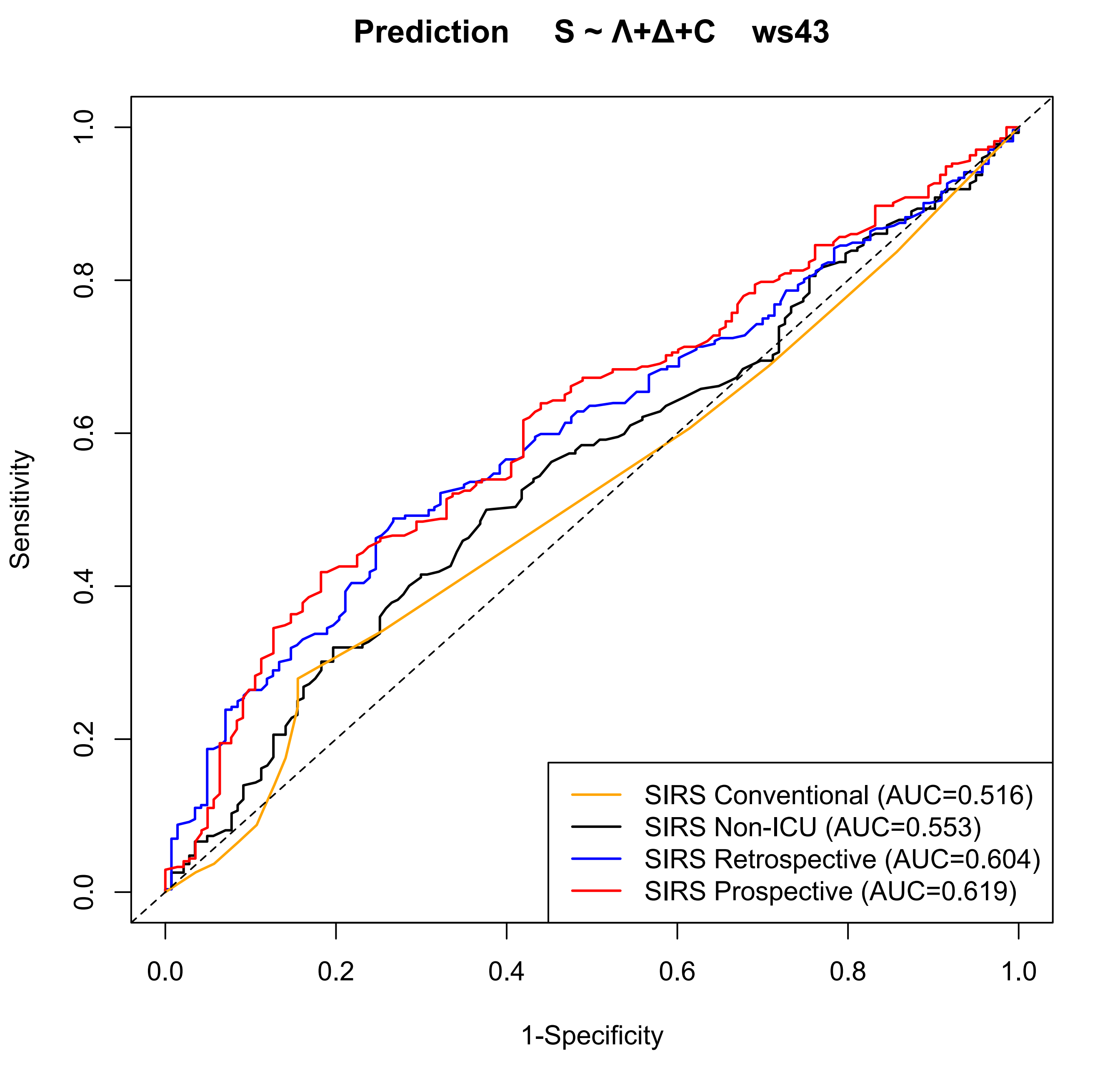

Prediction S ~ Λ+Δ+C ws43
Sensitivity
1-Specificity
0.0
0.2
0.4
0.6
0.8
1.0
SIRS Conventional (AUC=0.516)
SIRS Non-ICU (AUC=0.553)
SIRS Retrospective (AUC=0.604)
SIRS Prospective (AUC=0.619)
