# Supplementary material for: Dissecting contributions of individual systemic inflammatory response syndrome criteria from a prospective algorithm to the prediction and diagnosis of sepsis in a polytrauma cohort
Source: Front Med (Lausanne). 2023 Jul 31;10:1227031. doi: 10.3389/fmed.2023.1227031 (PMC10424878; doi:10.3389/fmed.2023.1227031)
Supplement: Supplementary file 3 [file Data_Sheet_3.pdf]

Supplementary Material 3

Dissecting contributions of individual systemic inflammatory response syndrome criteria from a prospective algorithm to the prediction and diagnosis of sepsis in a polytrauma cohort

Roman Schefzik, Bianka Hahn and Verena Schneider-Lindner

Here, we expand on the comparison of the four algorithms SIRS Conventional, SIRS Non-ICU, SIRS Retrospective and SIRS Prospective for the sepsis **diagnosis** task from Figure 7 in the main text and provide the corresponding ROC curves and AUROC values for all our 301 considered scenarios (43 weighting schemes × 7 logistic regression models). In particular, we consider the weighting schemes ws1 to ws43 and the models $S \sim \Lambda$, $S \sim \Delta$, $S \sim C$, $S \sim \Lambda + \Delta$, $S \sim \Lambda + C$, $S \sim \Delta + C$ and $S \sim \Lambda + \Delta + C$ as described in the main text.

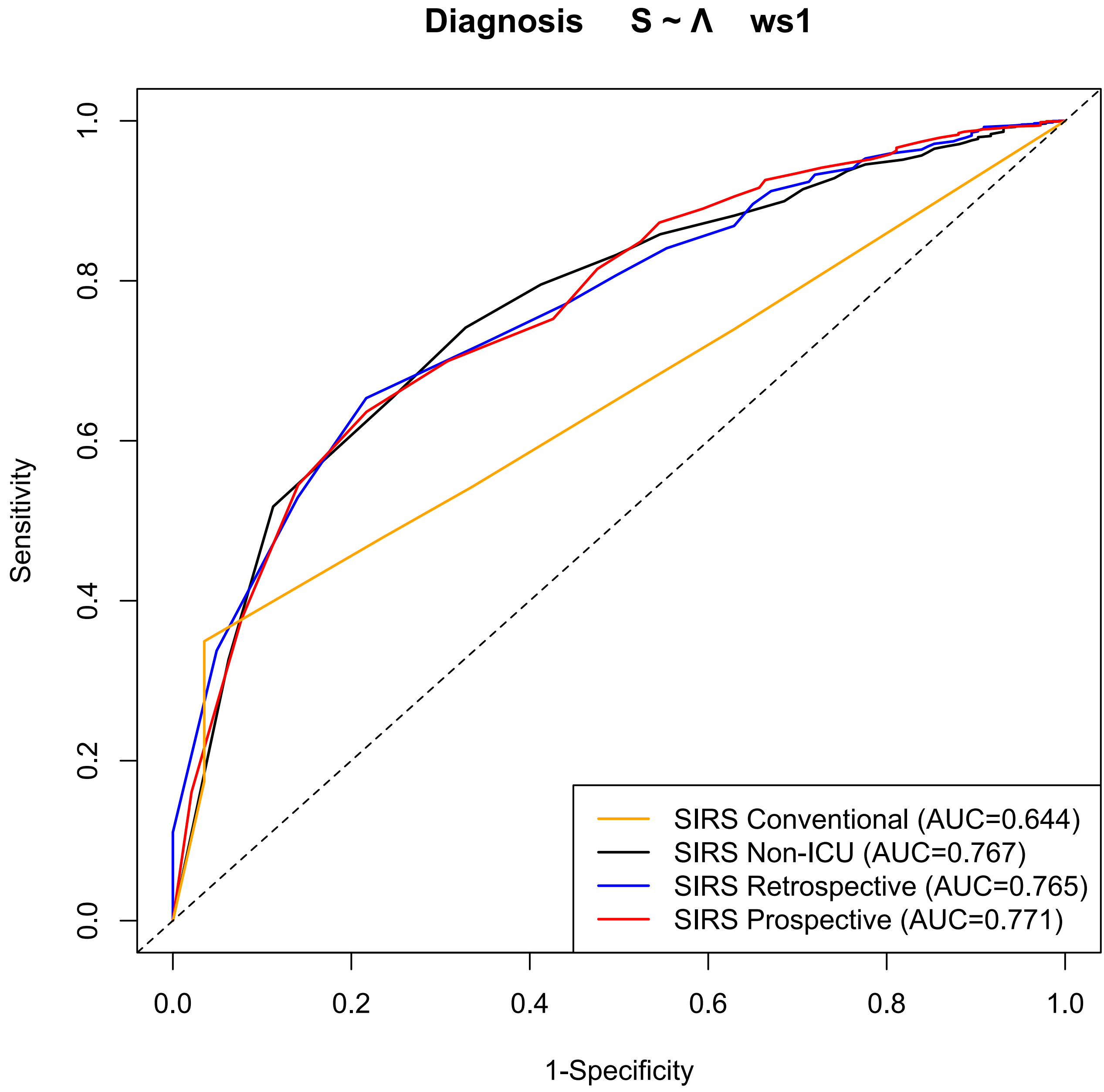

Diagnosis S ~ Λ ws1
Sensitivity
1-Specificity
0.0
0.2
0.4
0.6
0.8
1.0
SIRS Conventional (AUC=0.644)
SIRS Non-ICU (AUC=0.767)
SIRS Retrospective (AUC=0.765)
SIRS Prospective (AUC=0.771)

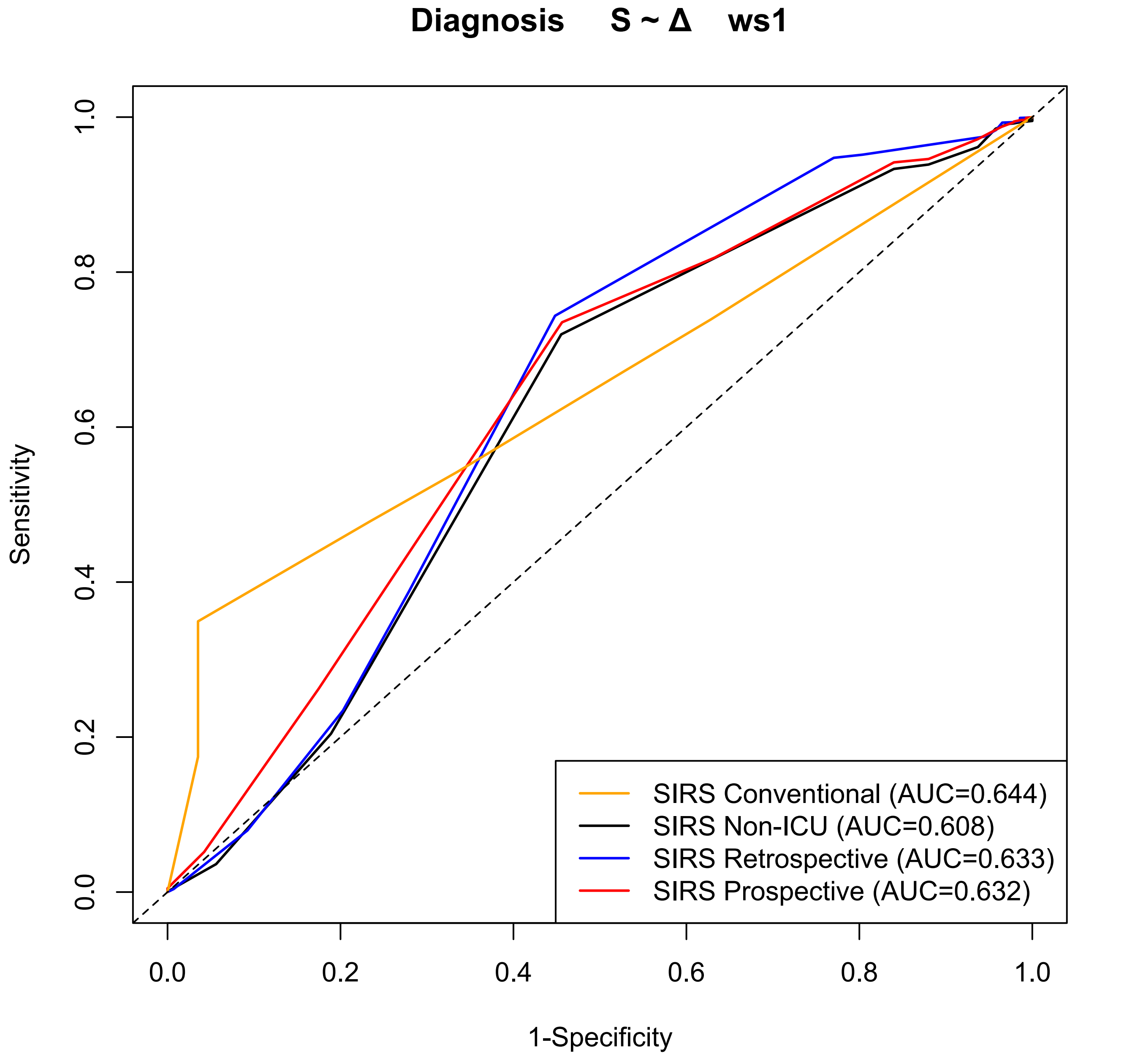
Diagnosis S ~ Δ ws1
Sensitivity
1-Specificity
0.0
0.2
0.4
0.6
0.8
1.0
SIRS Conventional (AUC=0.644)
SIRS Non-ICU (AUC=0.608)
SIRS Retrospective (AUC=0.633)
SIRS Prospective (AUC=0.632)

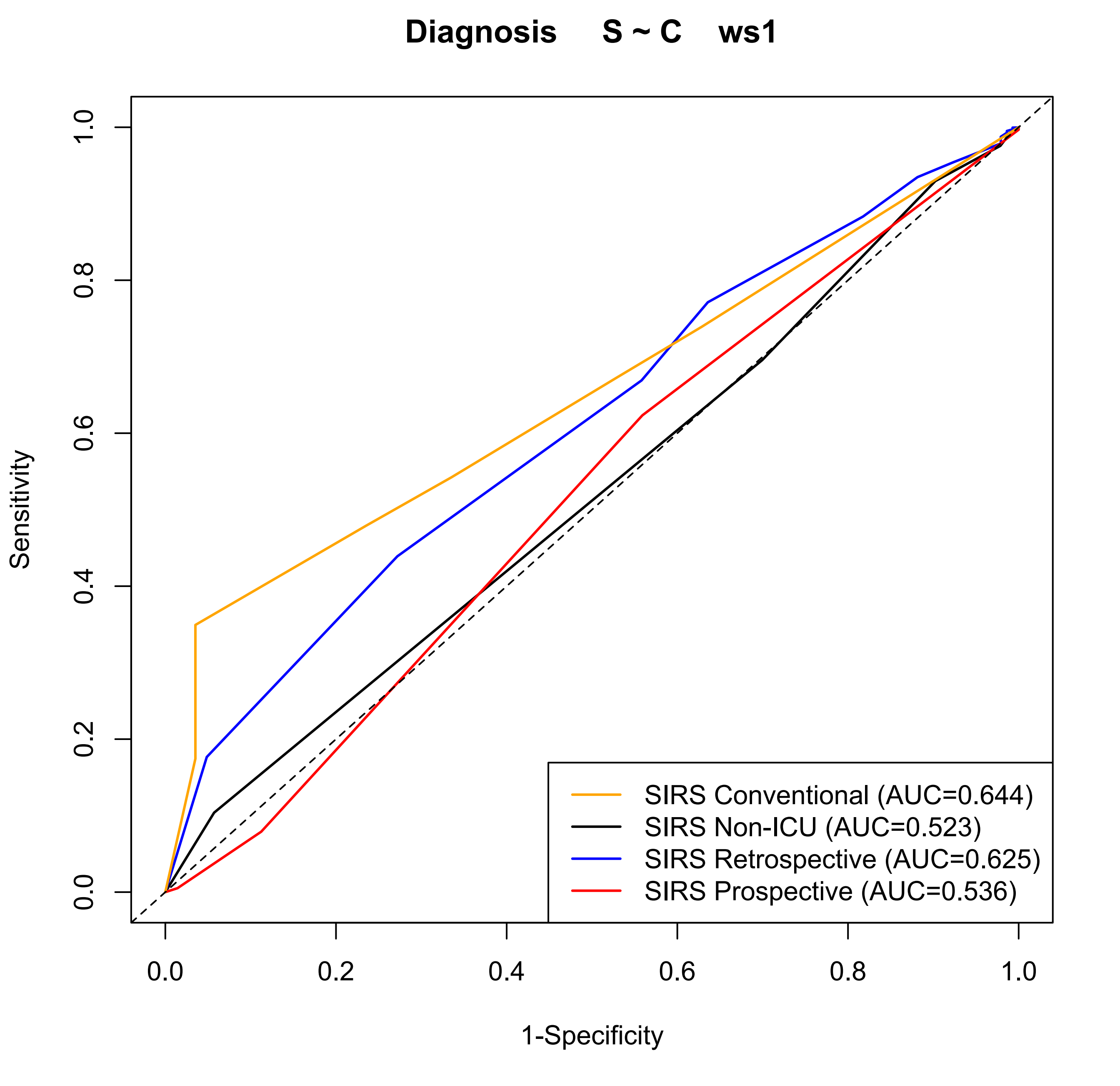

Diagnosis S ~ C ws1
Sensitivity
1-Specificity
0.0
0.2
0.4
0.6
0.8
1.0
SIRS Conventional (AUC=0.644)
SIRS Non-ICU (AUC=0.523)
SIRS Retrospective (AUC=0.625)
SIRS Prospective (AUC=0.536)

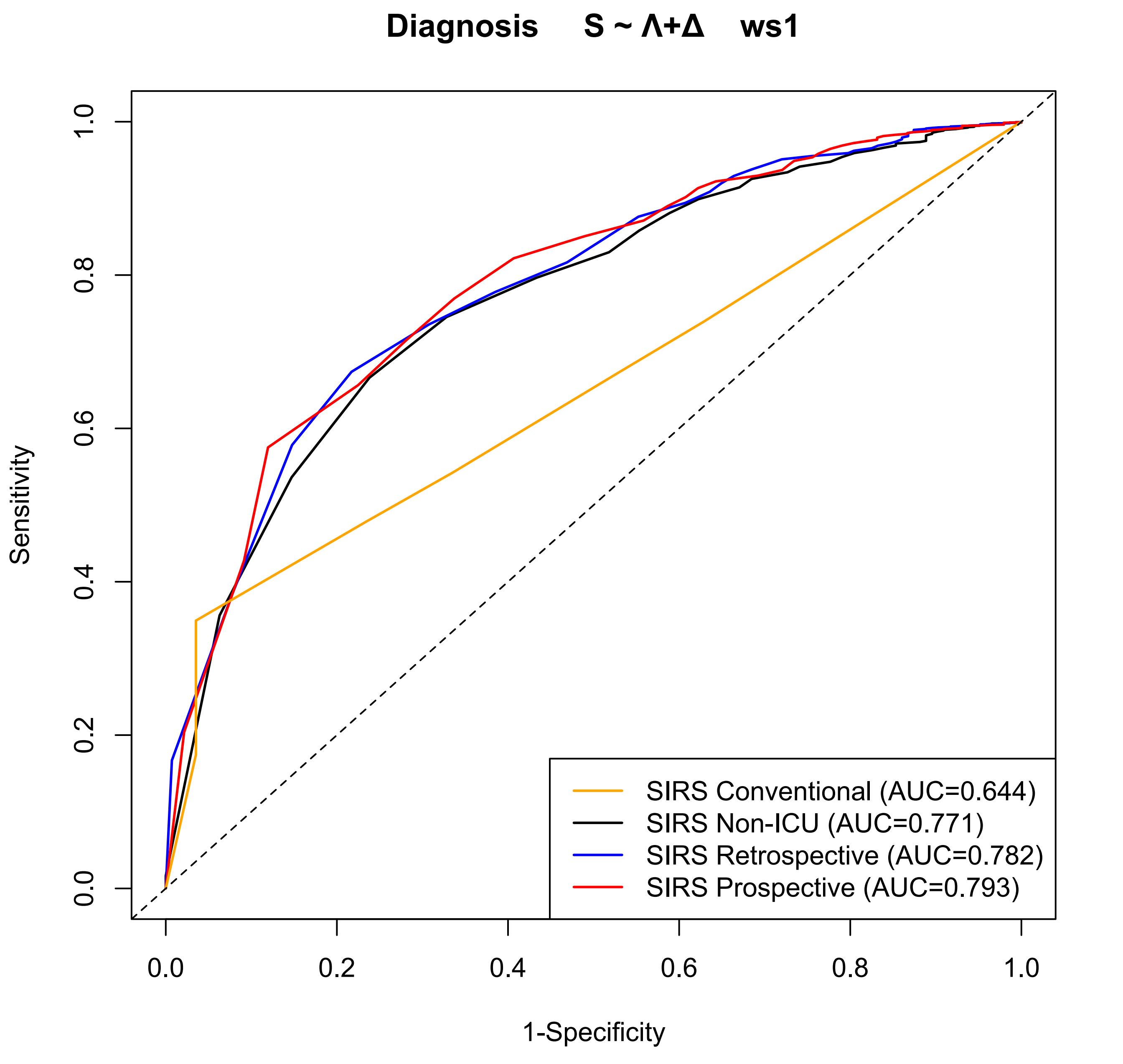

Diagnosis S ~ Λ+Δ ws1
Sensitivity
1-Specificity
0.0
0.2
0.4
0.6
0.8
1.0
SIRS Conventional (AUC=0.644)
SIRS Non-ICU (AUC=0.771)
SIRS Retrospective (AUC=0.782)
SIRS Prospective (AUC=0.793)

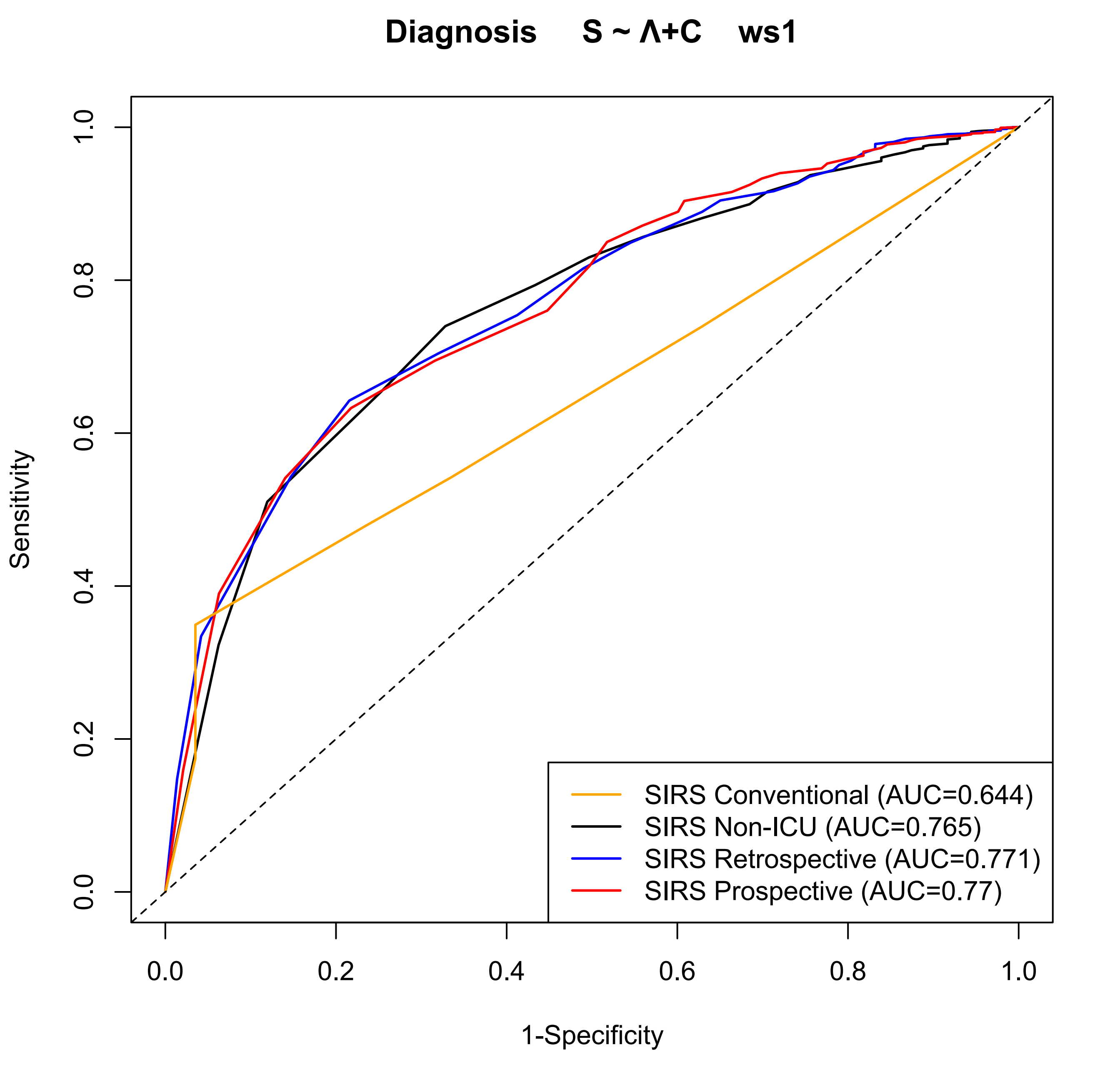

Diagnosis S ~ Λ+C ws1
Sensitivity
1-Specificity
0.0
0.2
0.4
0.6
0.8
1.0
SIRS Conventional (AUC=0.644)
SIRS Non-ICU (AUC=0.765)
SIRS Retrospective (AUC=0.771)
SIRS Prospective (AUC=0.77)

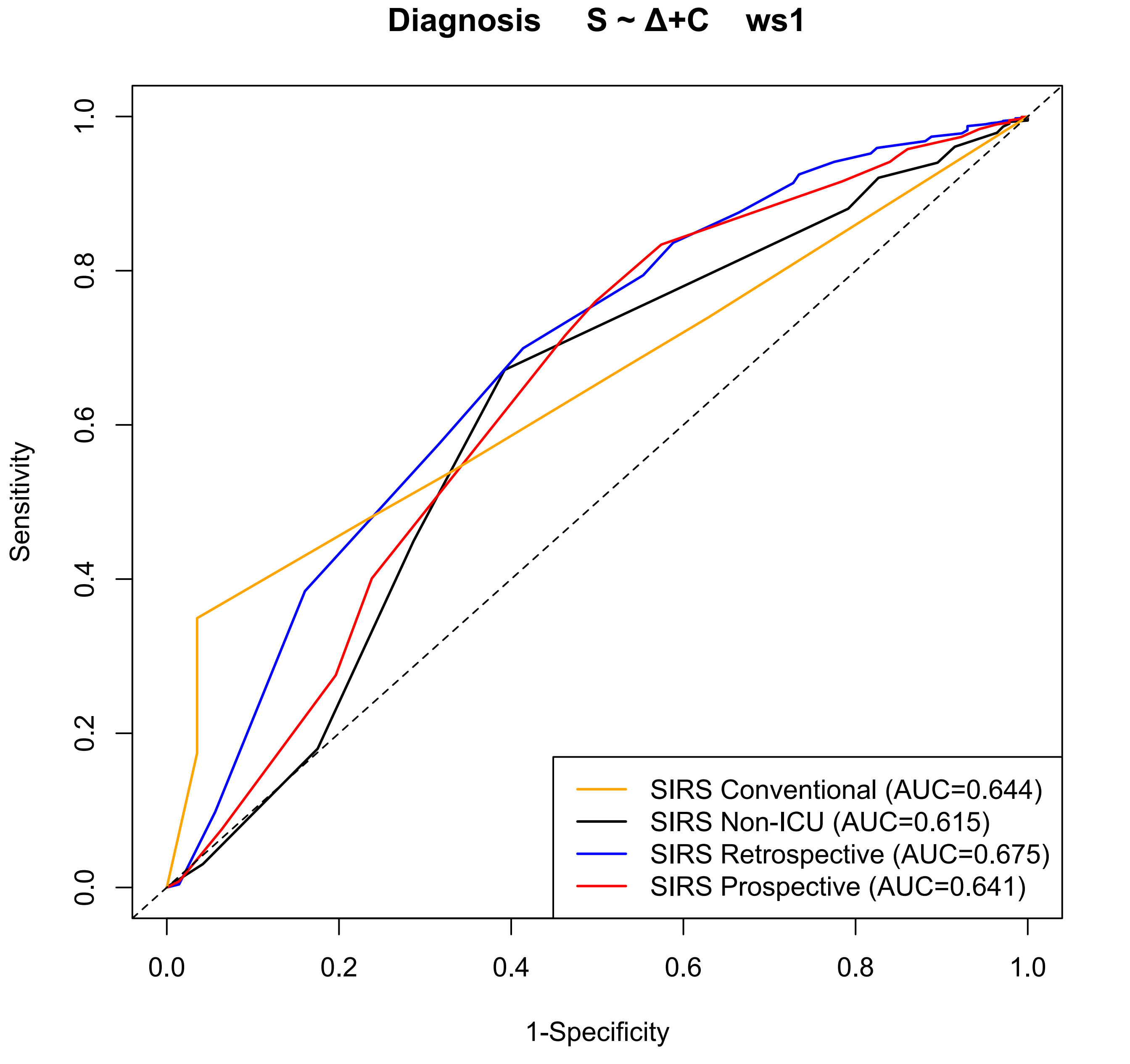
Diagnosis S ~ Δ+C ws1
Sensitivity
1-Specificity
0.0
0.2
0.4
0.6
0.8
1.0
SIRS Conventional (AUC=0.644)
SIRS Non-ICU (AUC=0.615)
SIRS Retrospective (AUC=0.675)
SIRS Prospective (AUC=0.641)

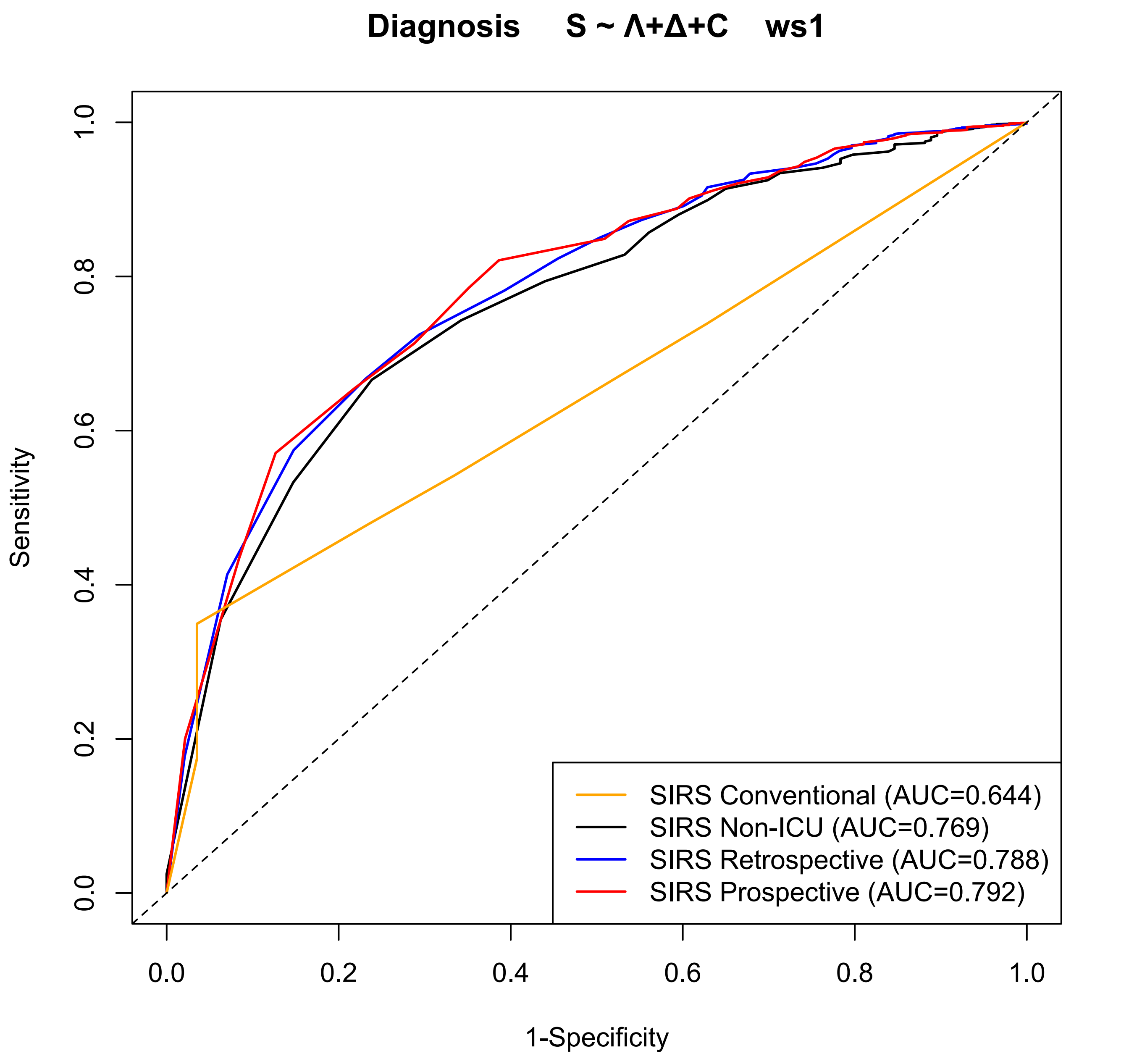

Diagnosis S ~ Λ+Δ+C ws1
Sensitivity
1-Specificity
0.0
0.2
0.4
0.6
0.8
1.0
SIRS Conventional (AUC=0.644)
SIRS Non-ICU (AUC=0.769)
SIRS Retrospective (AUC=0.788)
SIRS Prospective (AUC=0.792)

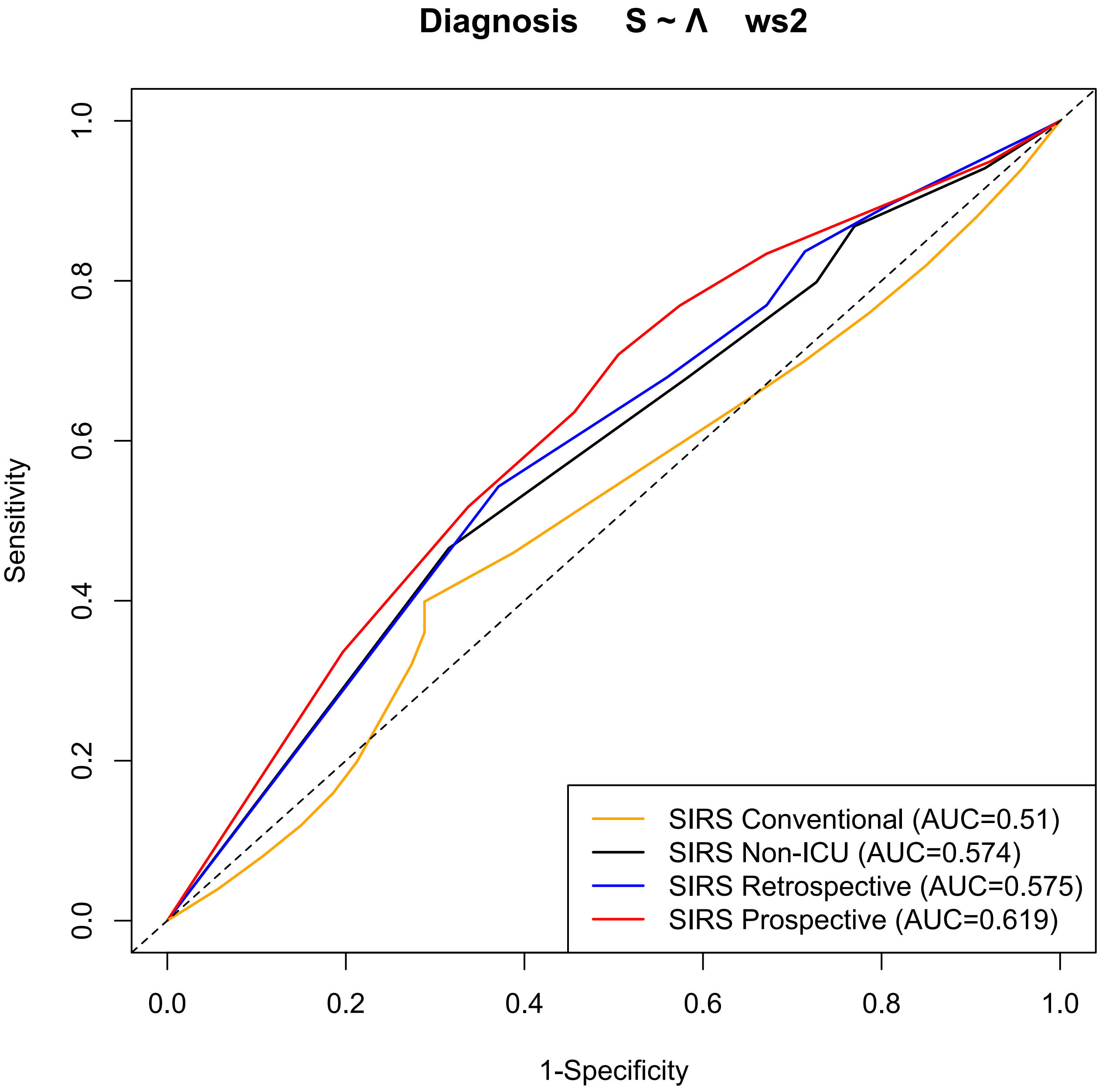

Diagnosis S ~ Λ ws2
Sensitivity
1-Specificity
0.0
0.2
0.4
0.6
0.8
1.0
SIRS Conventional (AUC=0.51)
SIRS Non-ICU (AUC=0.574)
SIRS Retrospective (AUC=0.575)
SIRS Prospective (AUC=0.619)

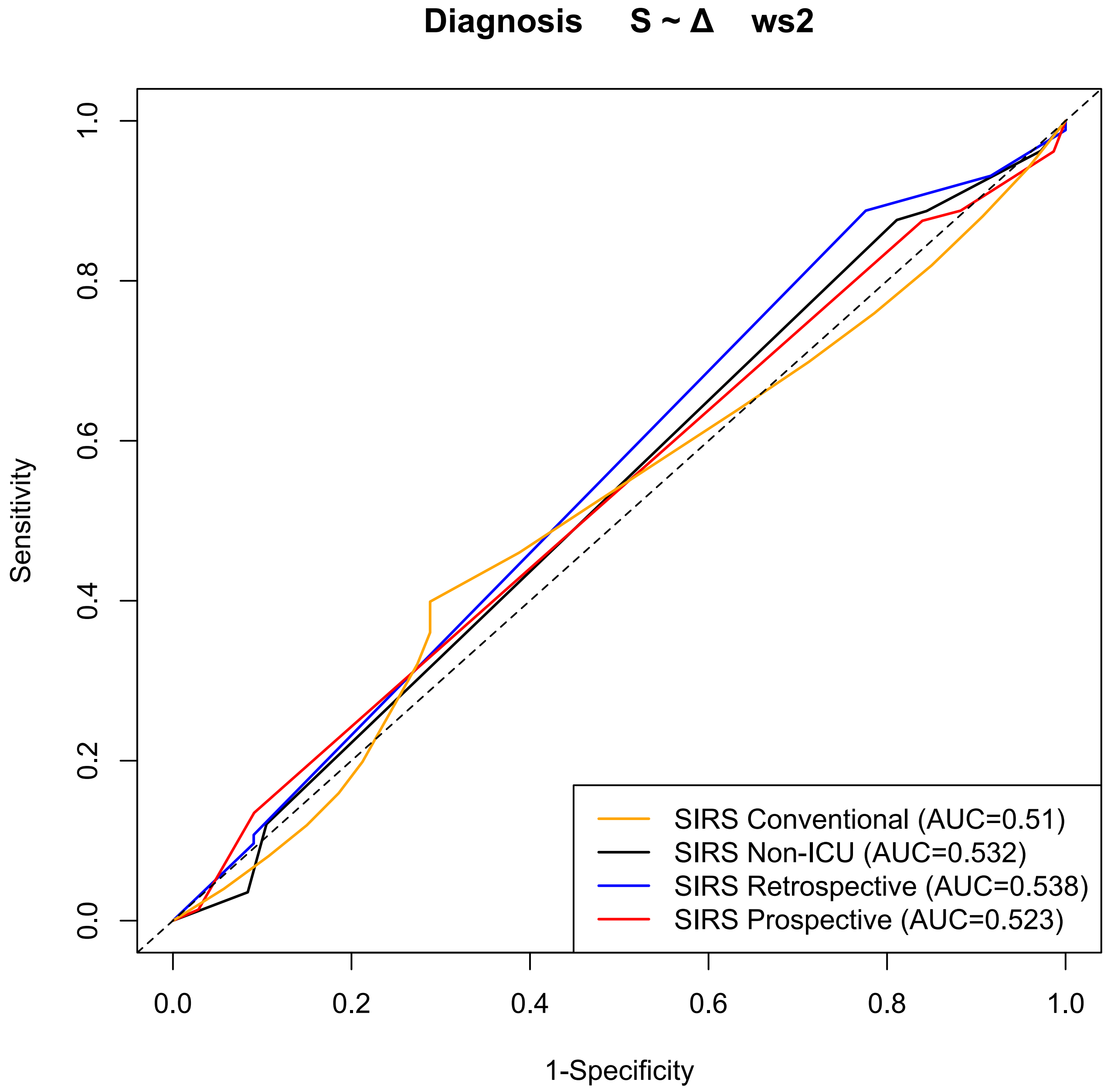

Diagnosis S ~ Δ ws2
Sensitivity
1-Specificity
0.0
0.2
0.4
0.6
0.8
1.0
SIRS Conventional (AUC=0.51)
SIRS Non-ICU (AUC=0.532)
SIRS Retrospective (AUC=0.538)
SIRS Prospective (AUC=0.523)

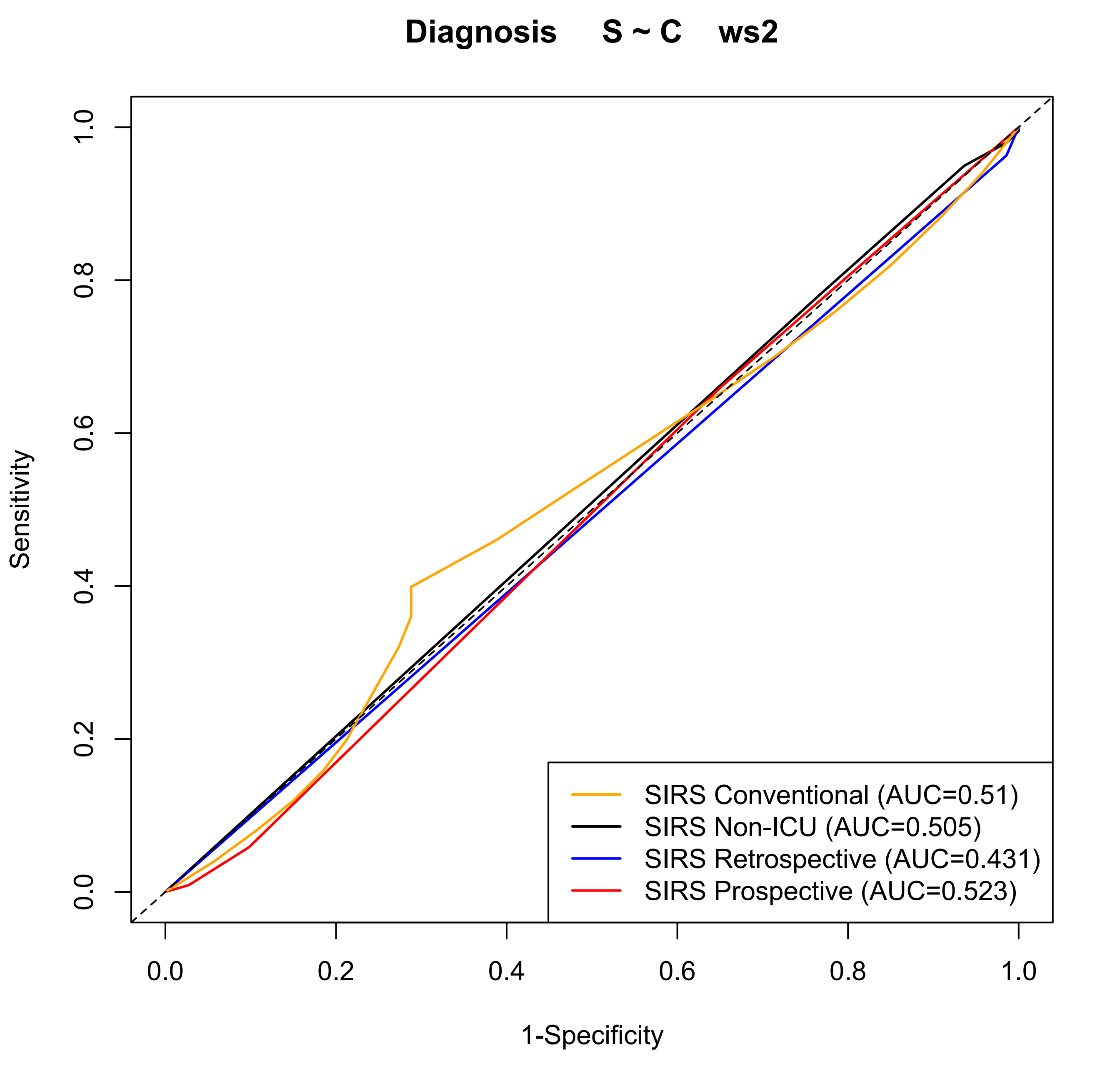
Diagnosis S ~ C ws2
Sensitivity
1-Specificity
0.0
0.2
0.4
0.6
0.8
1.0
SIRS Conventional (AUC=0.51)
SIRS Non-ICU (AUC=0.505)
SIRS Retrospective (AUC=0.431)
SIRS Prospective (AUC=0.523)

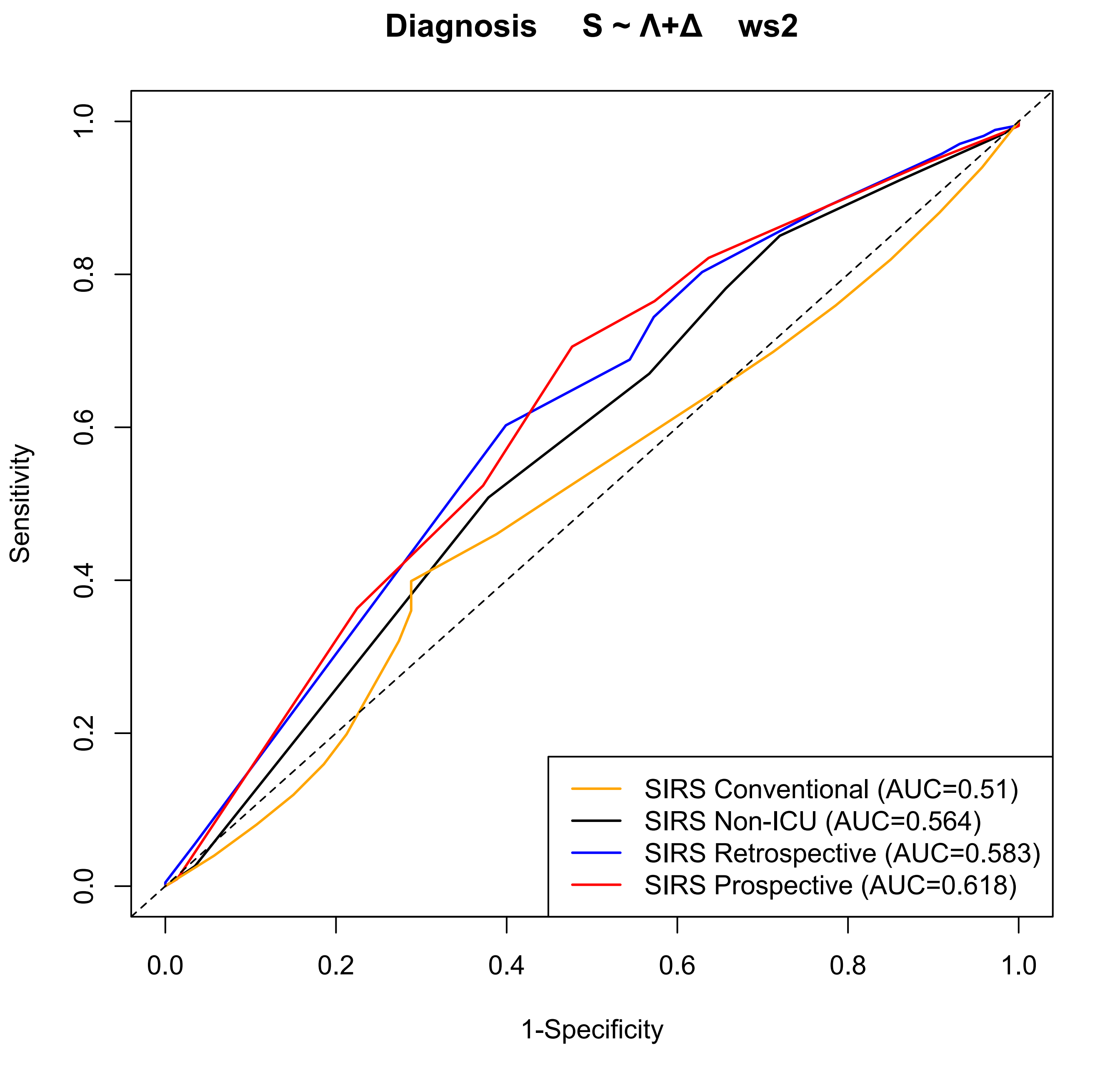
Diagnosis S ~ Λ+Δ ws2
Sensitivity
1-Specificity
0.0
0.2
0.4
0.6
0.8
1.0
SIRS Conventional (AUC=0.51)
SIRS Non-ICU (AUC=0.564)
SIRS Retrospective (AUC=0.583)
SIRS Prospective (AUC=0.618)

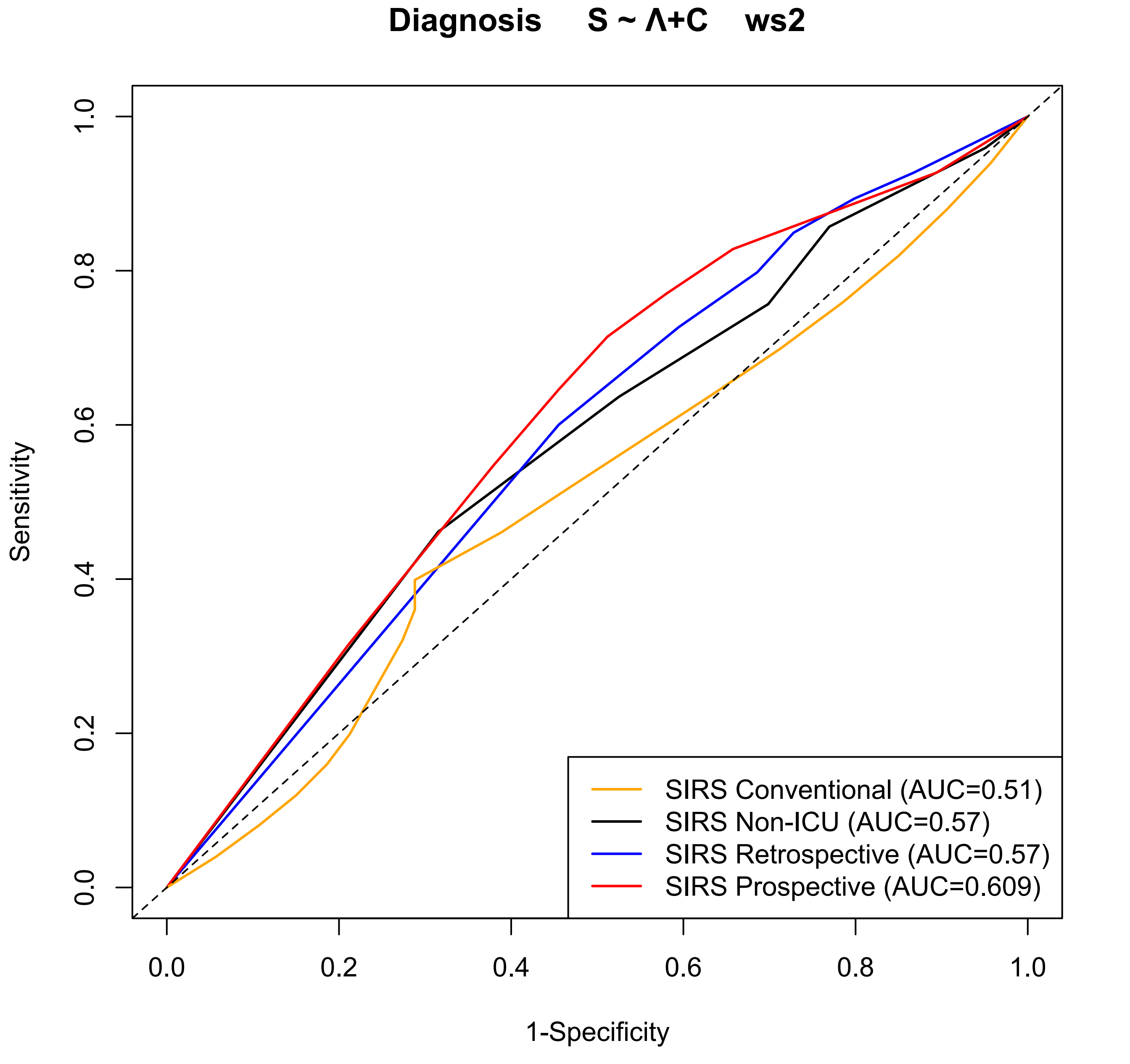
Diagnosis S ~ Λ+C ws2
Sensitivity
1-Specificity
0.0
0.2
0.4
0.6
0.8
1.0
SIRS Conventional (AUC=0.51)
SIRS Non-ICU (AUC=0.57)
SIRS Retrospective (AUC=0.57)
SIRS Prospective (AUC=0.609)

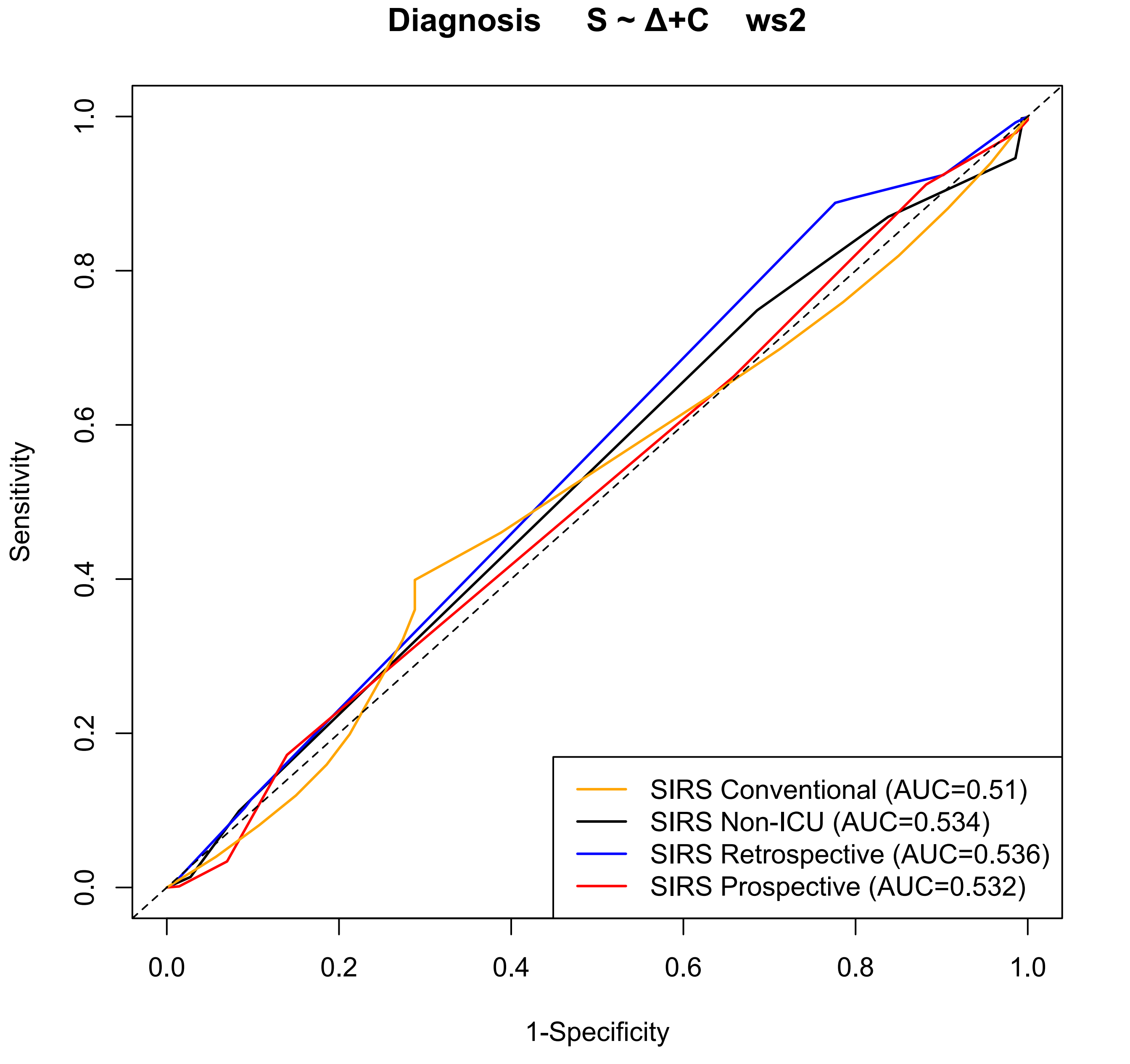

Diagnosis S ~ Δ+C ws2
Sensitivity
1-Specificity
0.0
0.2
0.4
0.6
0.8
1.0
SIRS Conventional (AUC=0.51)
SIRS Non-ICU (AUC=0.534)
SIRS Retrospective (AUC=0.536)
SIRS Prospective (AUC=0.532)

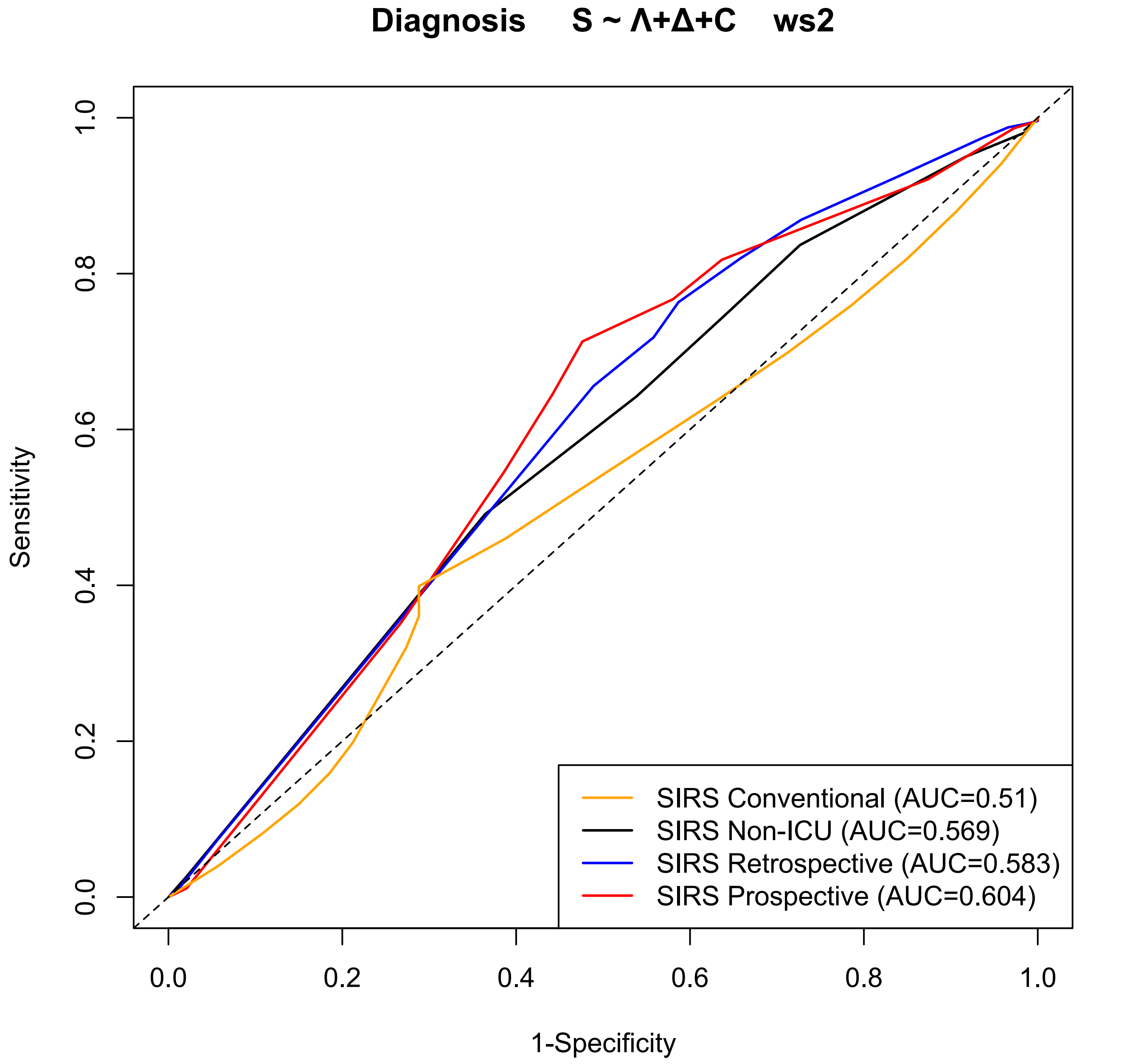
Diagnosis S ~ Λ+Δ+C ws2
Sensitivity
1-Specificity
0.0
0.2
0.4
0.6
0.8
1.0
0.0
0.2
0.4
0.6
0.8
1.0
SIRS Conventional (AUC=0.51)
SIRS Non-ICU (AUC=0.569)
SIRS Retrospective (AUC=0.583)
SIRS Prospective (AUC=0.604)

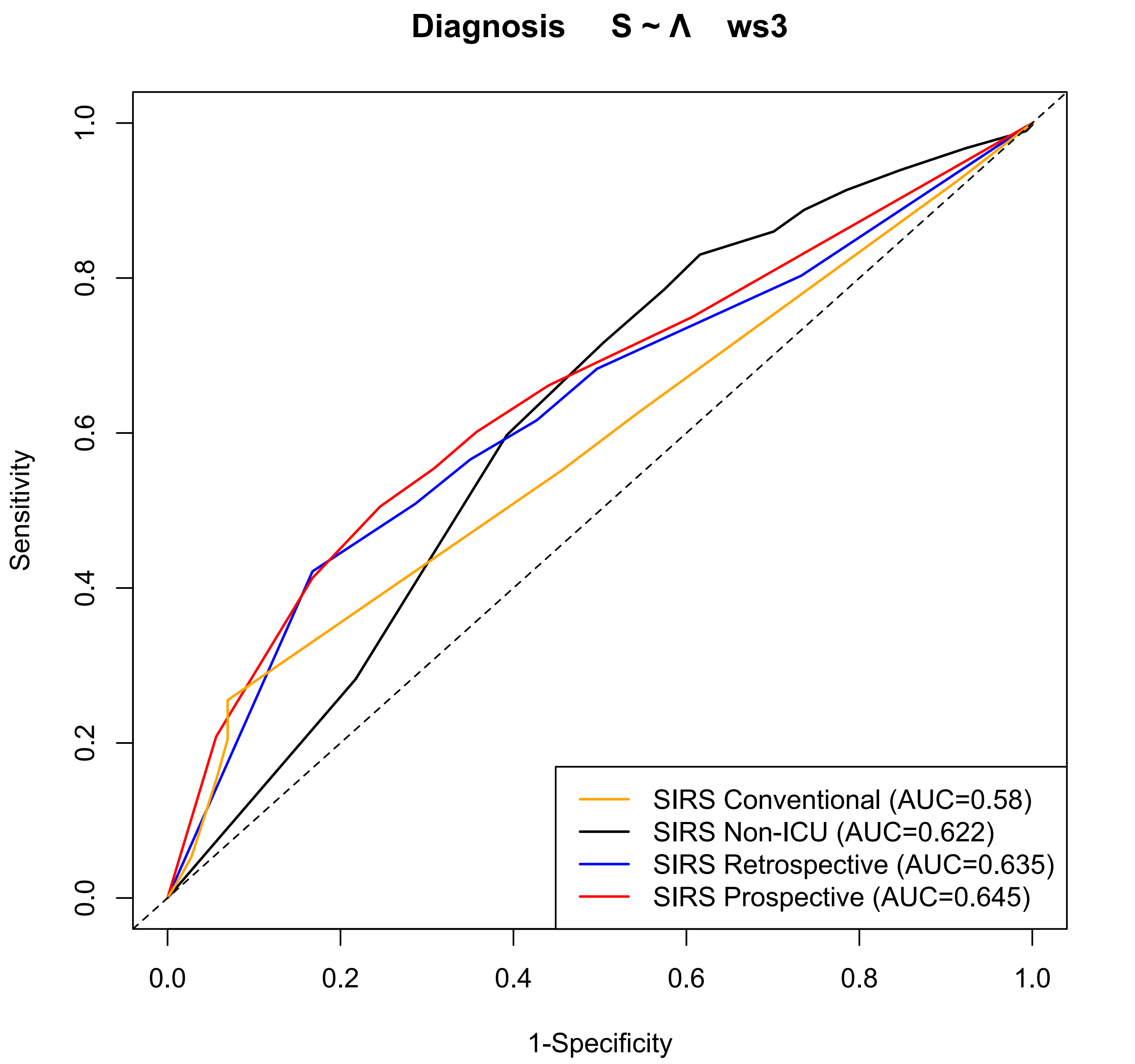
Diagnosis S ~ Λ ws3
Sensitivity
1-Specificity
0.0
0.2
0.4
0.6
0.8
1.0
SIRS Conventional (AUC=0.58)
SIRS Non-ICU (AUC=0.622)
SIRS Retrospective (AUC=0.635)
SIRS Prospective (AUC=0.645)

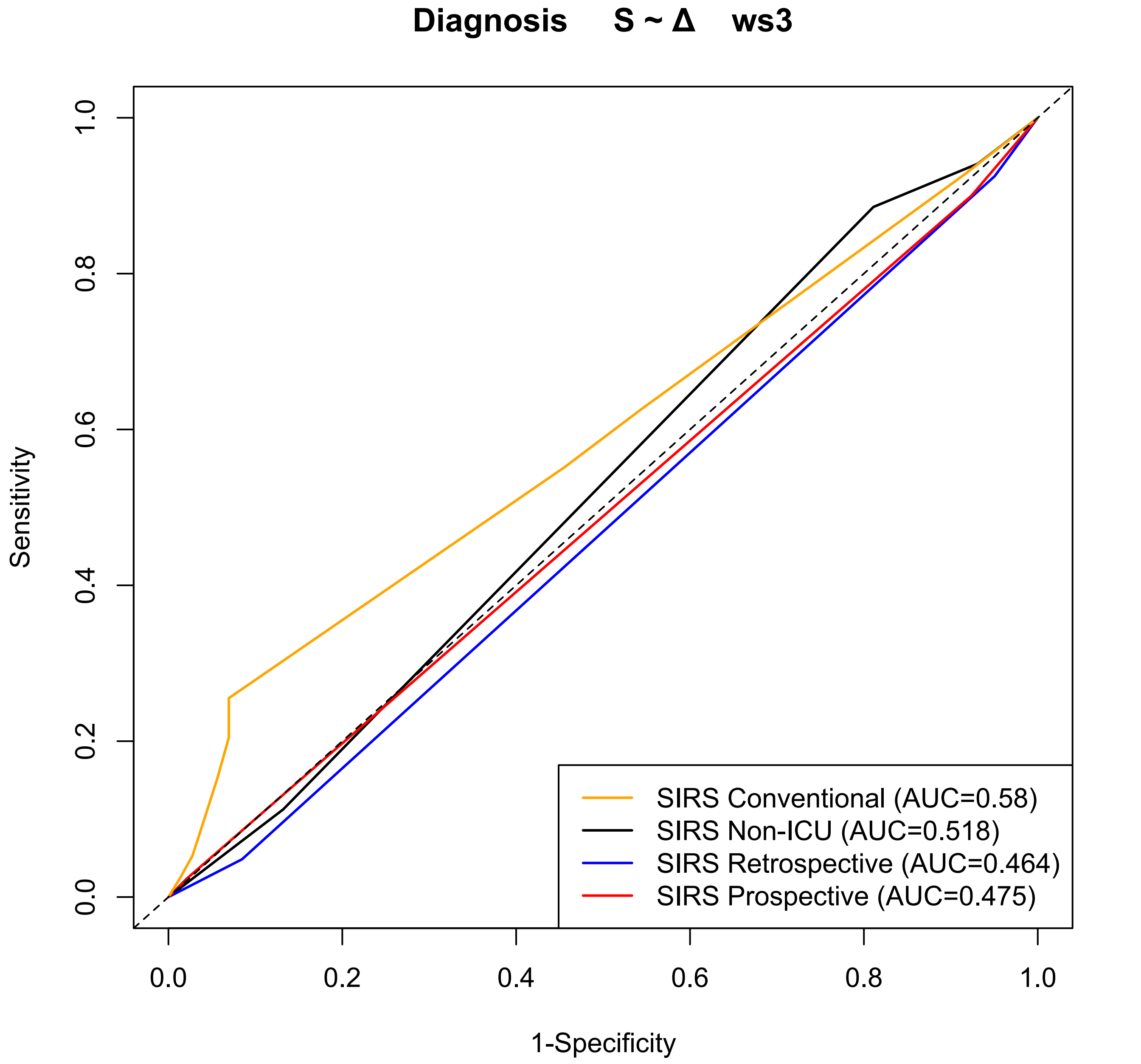

Diagnosis S ~ Δ ws3
Sensitivity
1-Specificity
0.0
0.2
0.4
0.6
0.8
1.0
0.0
0.2
0.4
0.6
0.8
1.0
SIRS Conventional (AUC=0.58)
SIRS Non-ICU (AUC=0.518)
SIRS Retrospective (AUC=0.464)
SIRS Prospective (AUC=0.475)

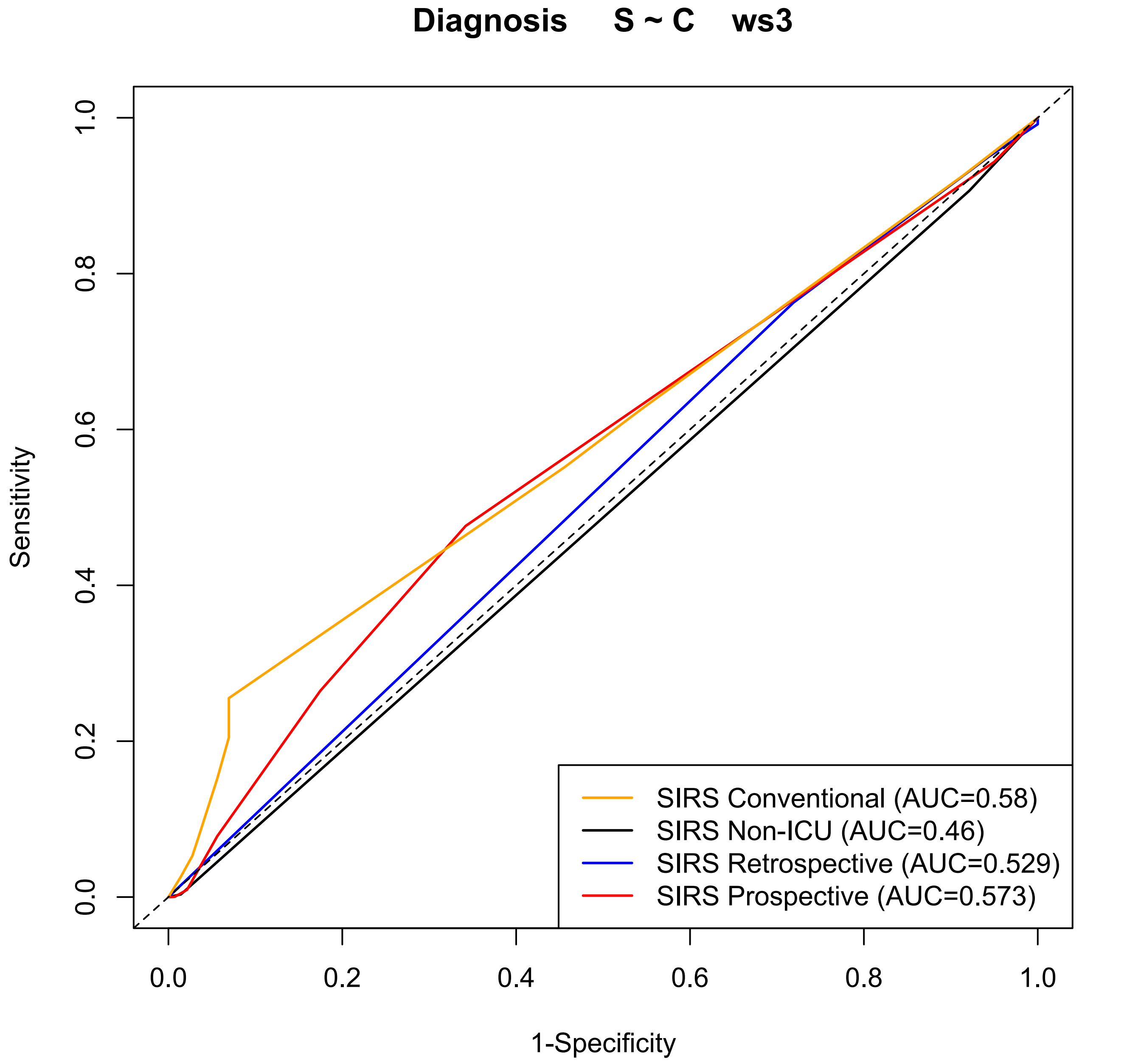

Diagnosis S ~ C ws3
Sensitivity
1-Specificity
0.0
0.2
0.4
0.6
0.8
1.0
SIRS Conventional (AUC=0.58)
SIRS Non-ICU (AUC=0.46)
SIRS Retrospective (AUC=0.529)
SIRS Prospective (AUC=0.573)

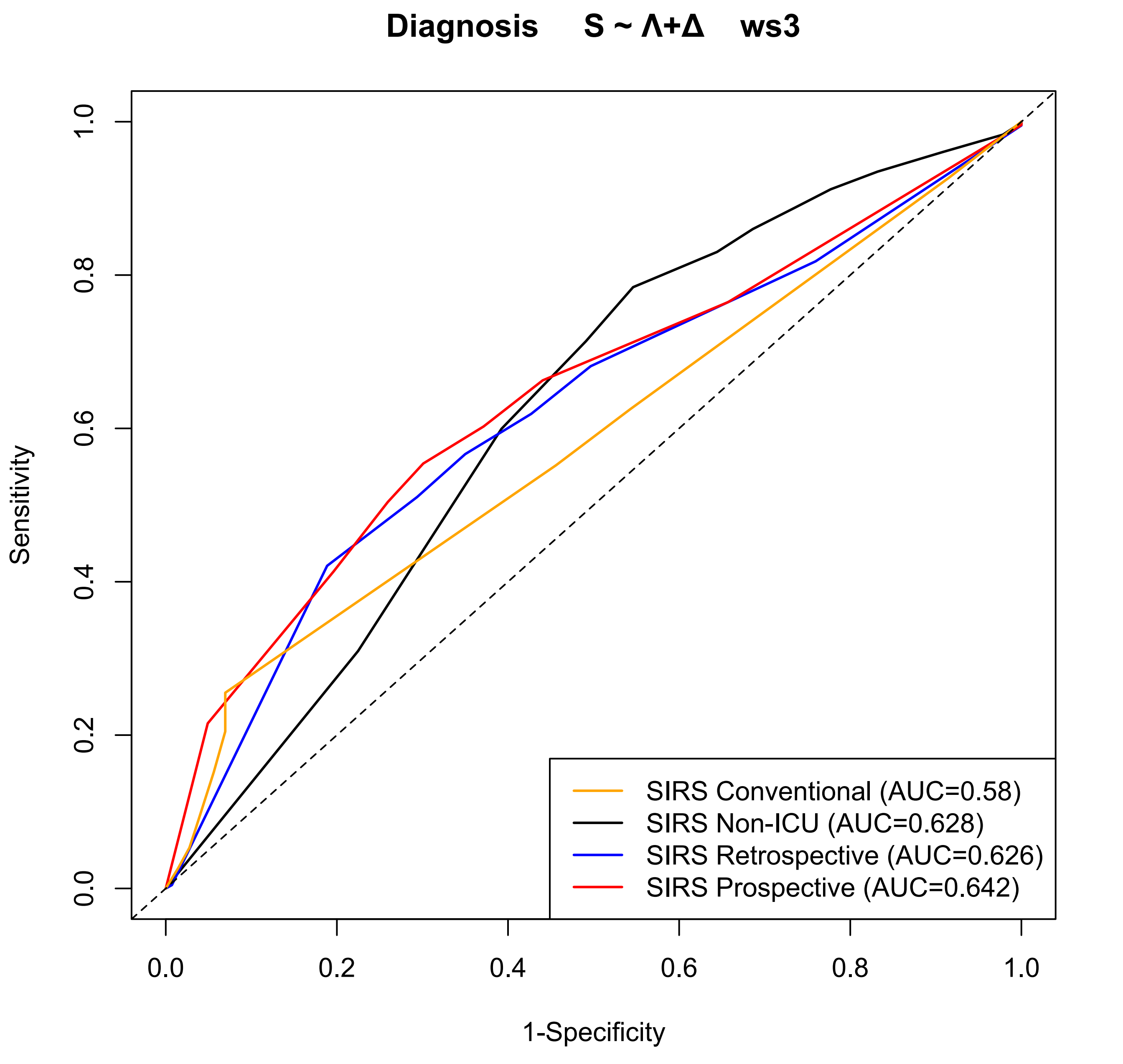
Diagnosis S ~ Λ+Δ ws3
Sensitivity
1-Specificity
0.0
0.2
0.4
0.6
0.8
1.0
SIRS Conventional (AUC=0.58)
SIRS Non-ICU (AUC=0.628)
SIRS Retrospective (AUC=0.626)
SIRS Prospective (AUC=0.642)

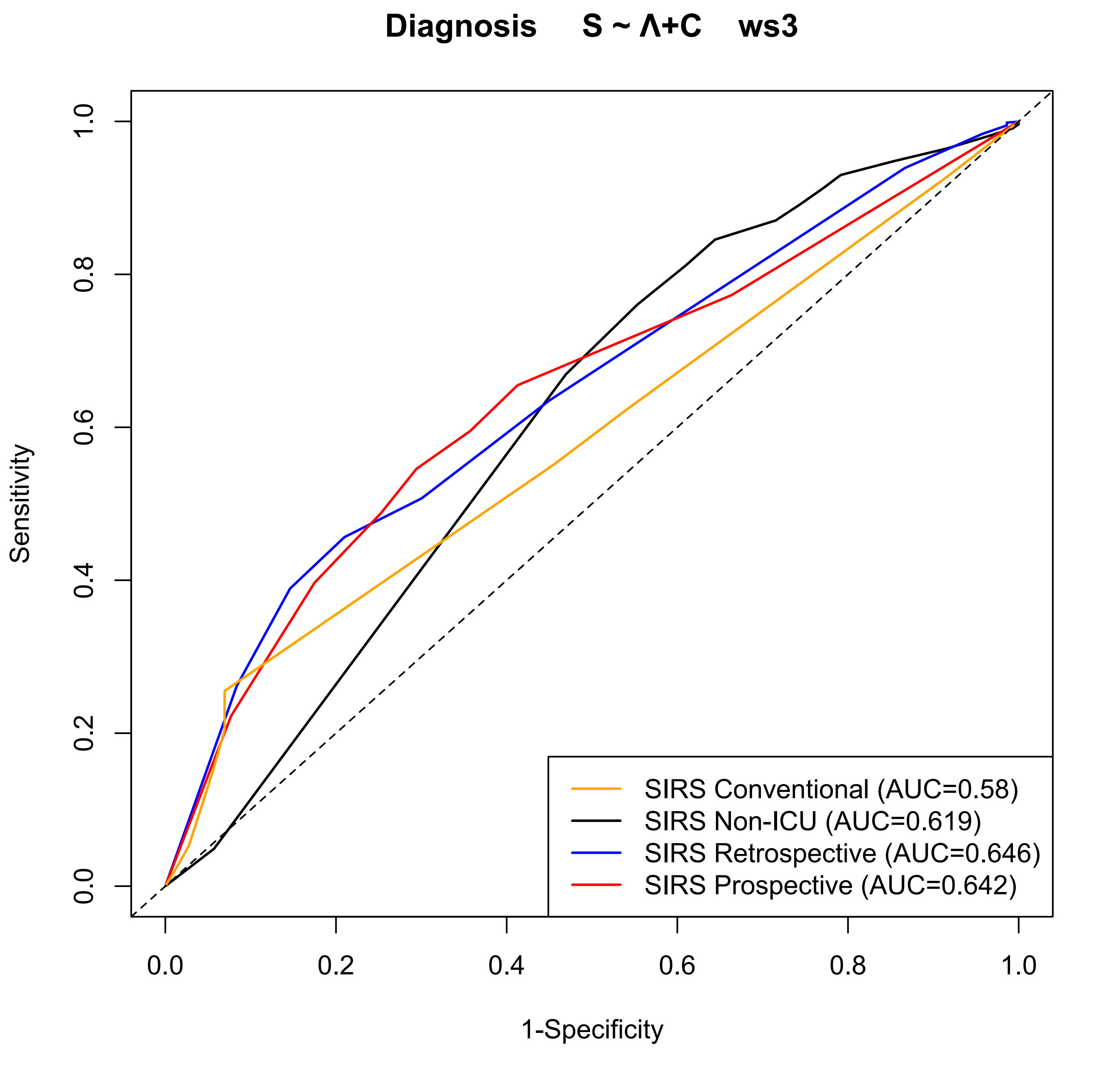
Diagnosis S ~ Λ+C ws3
Sensitivity
1-Specificity
0.0
0.2
0.4
0.6
0.8
1.0
0.0
0.2
0.4
0.6
0.8
1.0
SIRS Conventional (AUC=0.58)
SIRS Non-ICU (AUC=0.619)
SIRS Retrospective (AUC=0.646)
SIRS Prospective (AUC=0.642)

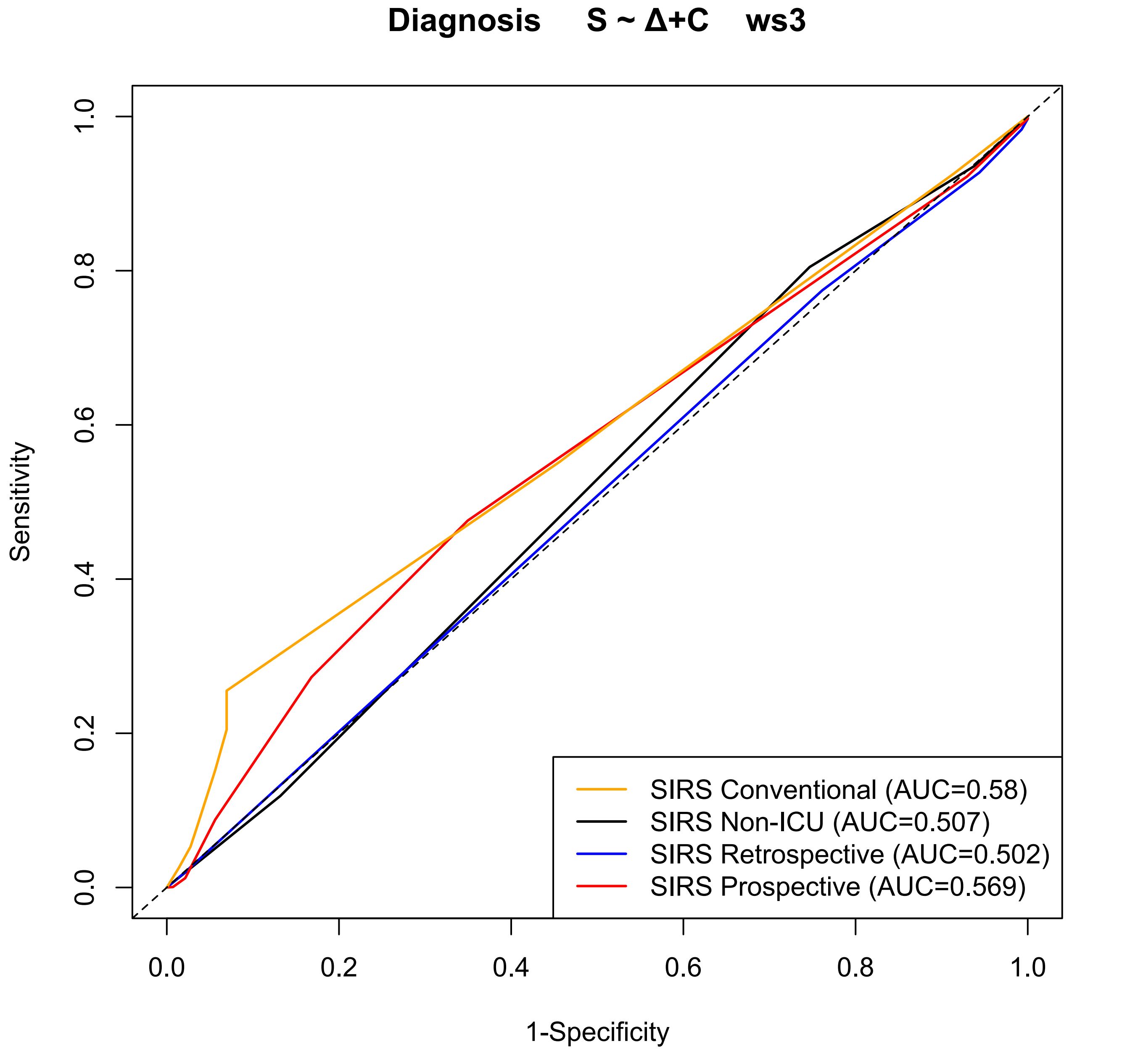
Diagnosis S ~ Δ+C ws3
Sensitivity
1-Specificity
0.0
0.2
0.4
0.6
0.8
1.0
SIRS Conventional (AUC=0.58)
SIRS Non-ICU (AUC=0.507)
SIRS Retrospective (AUC=0.502)
SIRS Prospective (AUC=0.569)

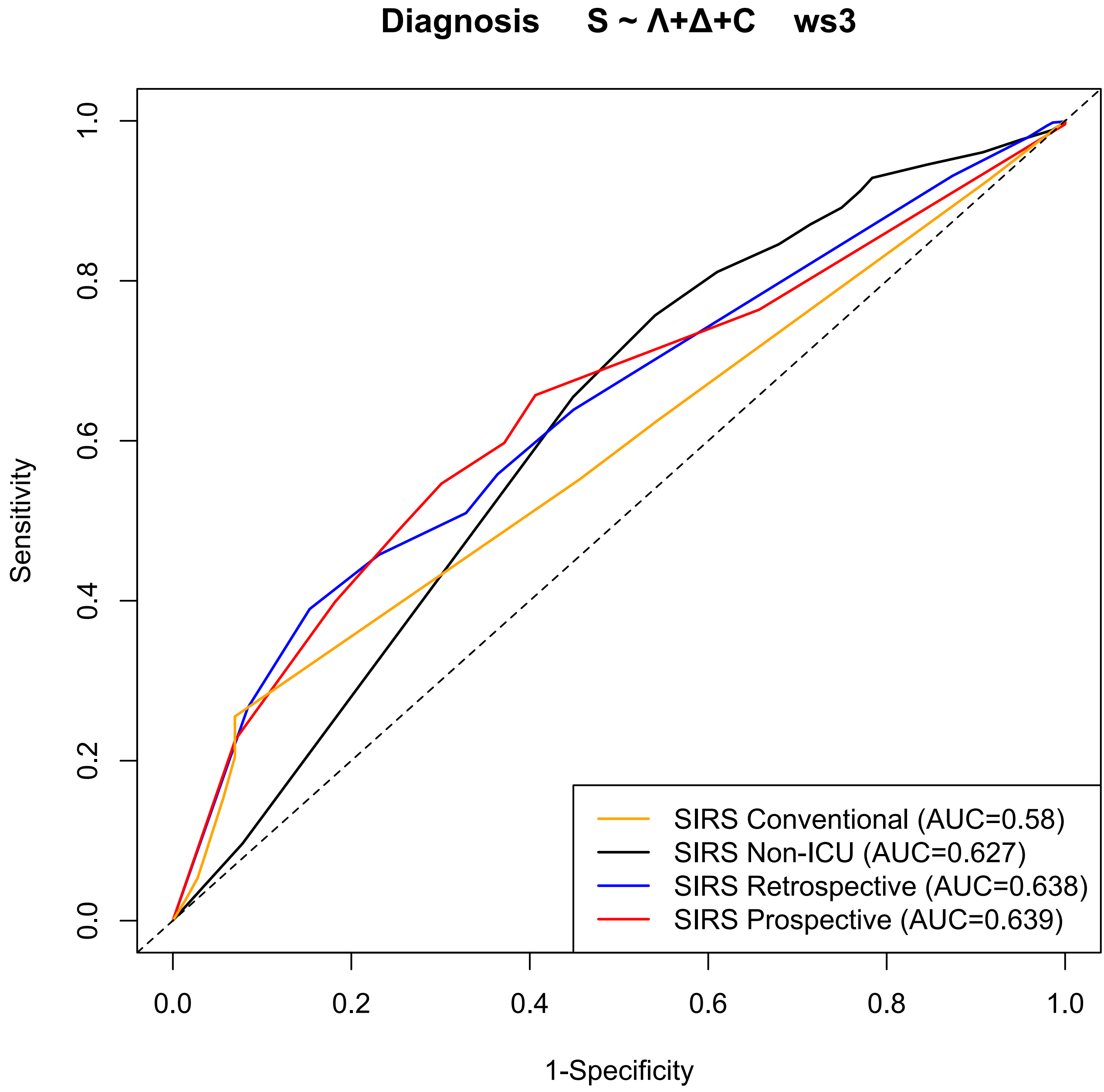
Diagnosis S ~ Λ+Δ+C ws3
Sensitivity
1-Specificity
0.0
0.2
0.4
0.6
0.8
1.0
SIRS Conventional (AUC=0.58)
SIRS Non-ICU (AUC=0.627)
SIRS Retrospective (AUC=0.638)
SIRS Prospective (AUC=0.639)

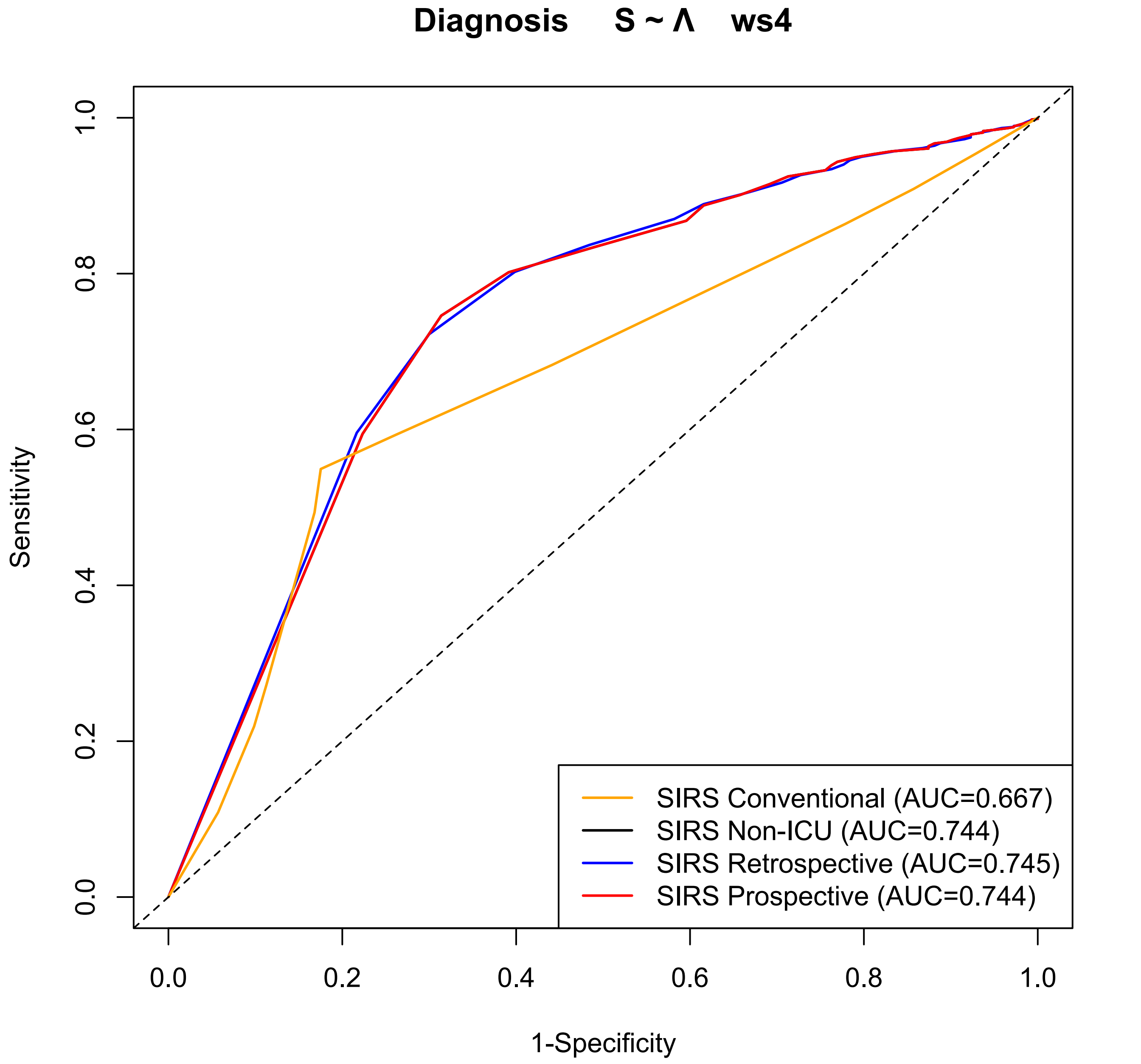
Diagnosis S ~ Λ ws4
Sensitivity
1-Specificity
0.0
0.2
0.4
0.6
0.8
1.0
SIRS Conventional (AUC=0.667)
SIRS Non-ICU (AUC=0.744)
SIRS Retrospective (AUC=0.745)
SIRS Prospective (AUC=0.744)

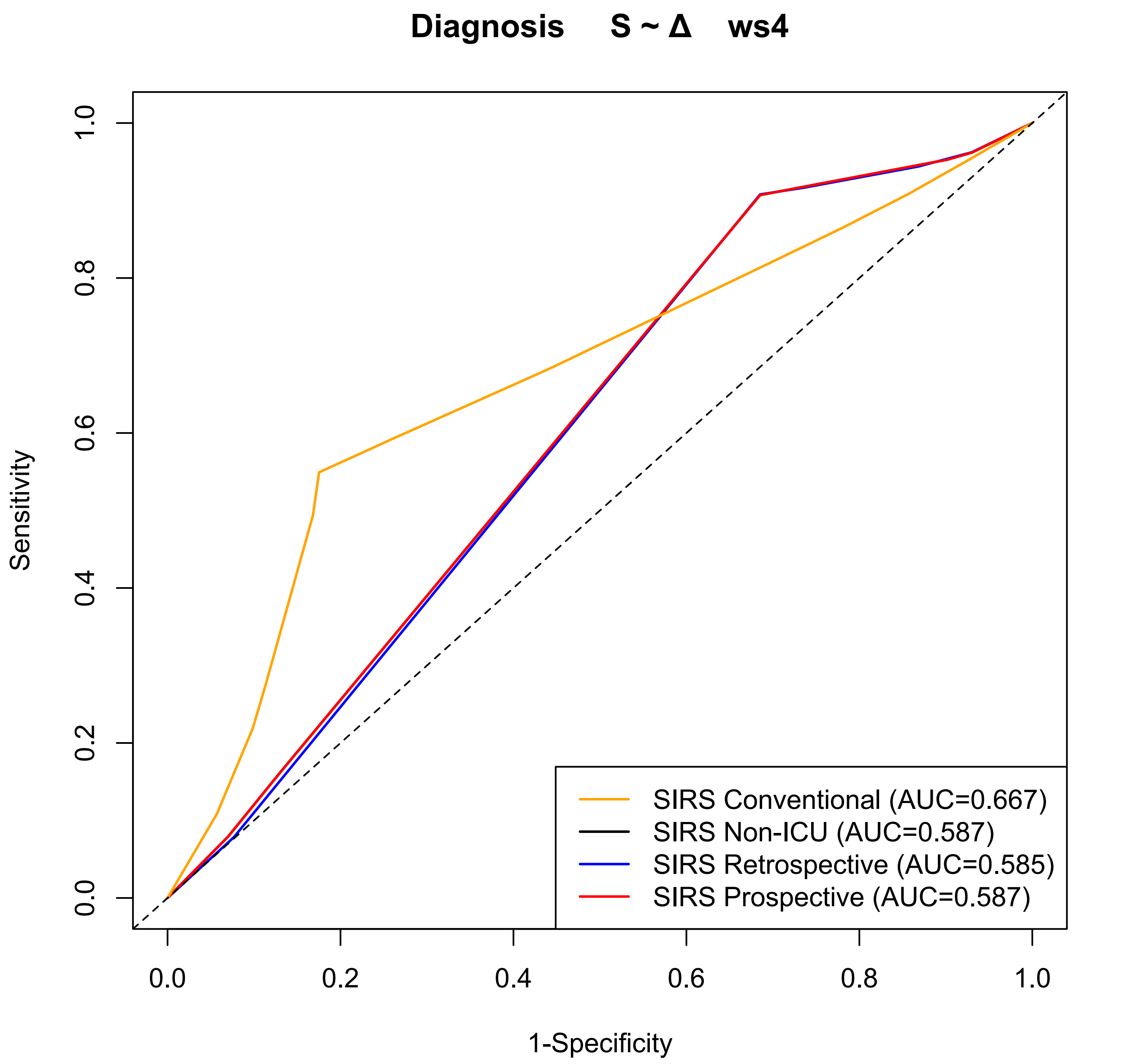

Diagnosis S ~ Δ ws4
Sensitivity
1-Specificity
0.0
0.2
0.4
0.6
0.8
1.0
SIRS Conventional (AUC=0.667)
SIRS Non-ICU (AUC=0.587)
SIRS Retrospective (AUC=0.585)
SIRS Prospective (AUC=0.587)

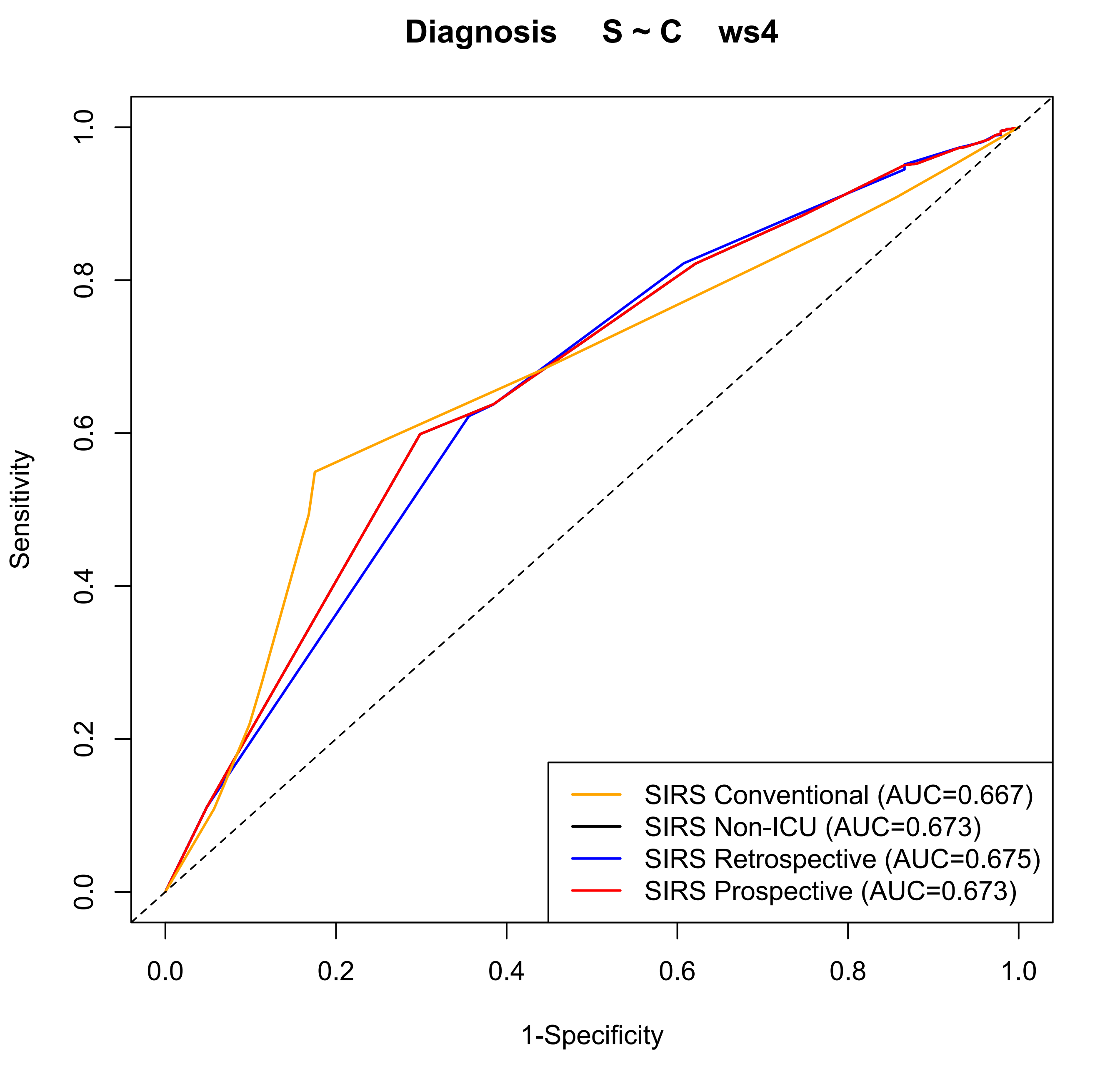

Diagnosis S ~ C ws4
Sensitivity
1-Specificity
0.0
0.2
0.4
0.6
0.8
1.0
0.0
0.2
0.4
0.6
0.8
1.0
SIRS Conventional (AUC=0.667)
SIRS Non-ICU (AUC=0.673)
SIRS Retrospective (AUC=0.675)
SIRS Prospective (AUC=0.673)

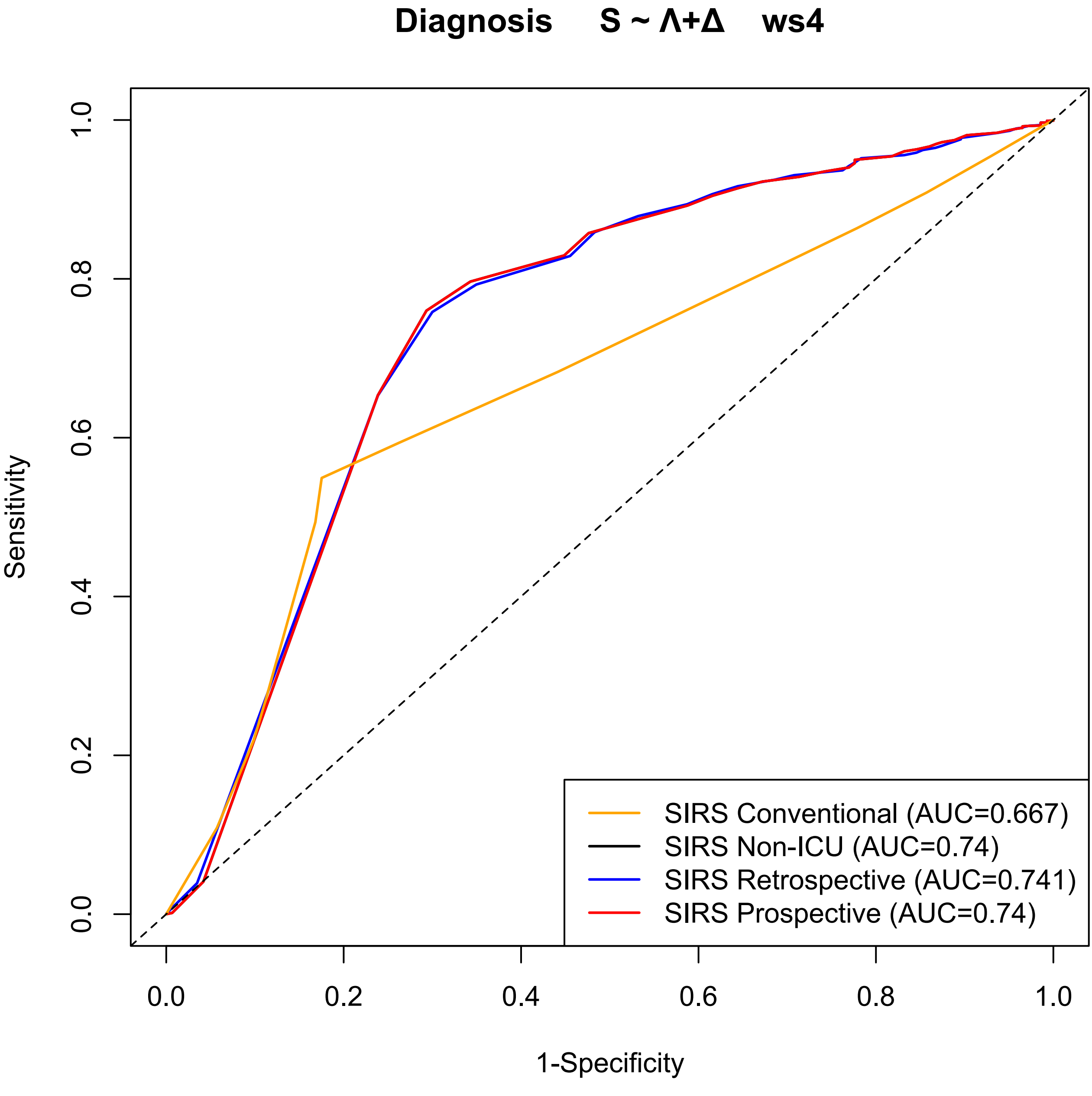
Diagnosis S ~ Λ+Δ ws4
Sensitivity
0.0
0.2
0.4
0.6
0.8
1.0
1-Specificity
0.0
0.2
0.4
0.6
0.8
1.0
SIRS Conventional (AUC=0.667)
SIRS Non-ICU (AUC=0.74)
SIRS Retrospective (AUC=0.741)
SIRS Prospective (AUC=0.74)

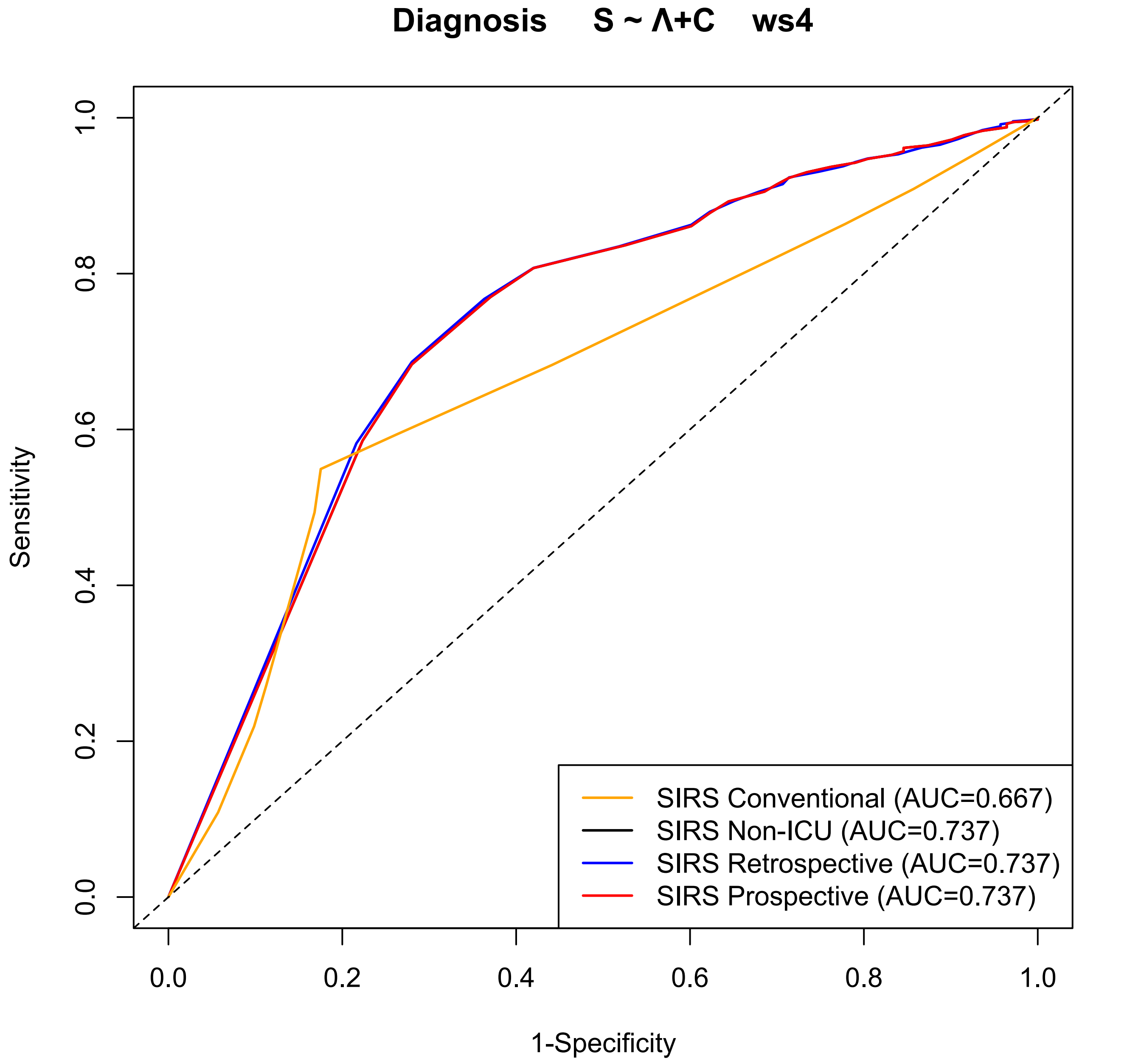

Diagnosis S ~ Λ+C ws4
Sensitivity
1-Specificity
0.0
0.2
0.4
0.6
0.8
1.0
SIRS Conventional (AUC=0.667)
SIRS Non-ICU (AUC=0.737)
SIRS Retrospective (AUC=0.737)
SIRS Prospective (AUC=0.737)

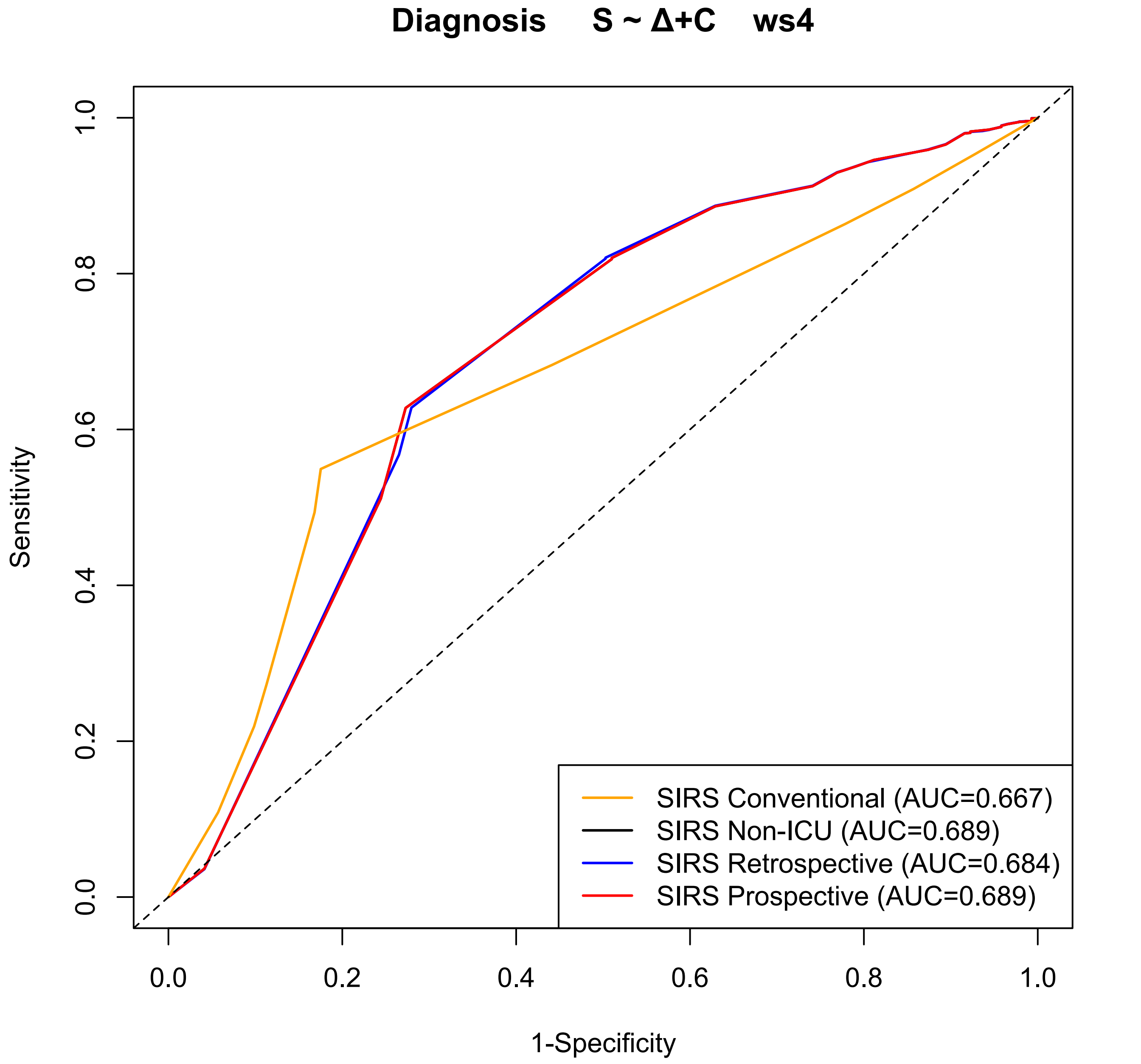

Diagnosis S ~ Δ+C ws4
Sensitivity
1-Specificity
0.0
0.2
0.4
0.6
0.8
1.0
SIRS Conventional (AUC=0.667)
SIRS Non-ICU (AUC=0.689)
SIRS Retrospective (AUC=0.684)
SIRS Prospective (AUC=0.689)

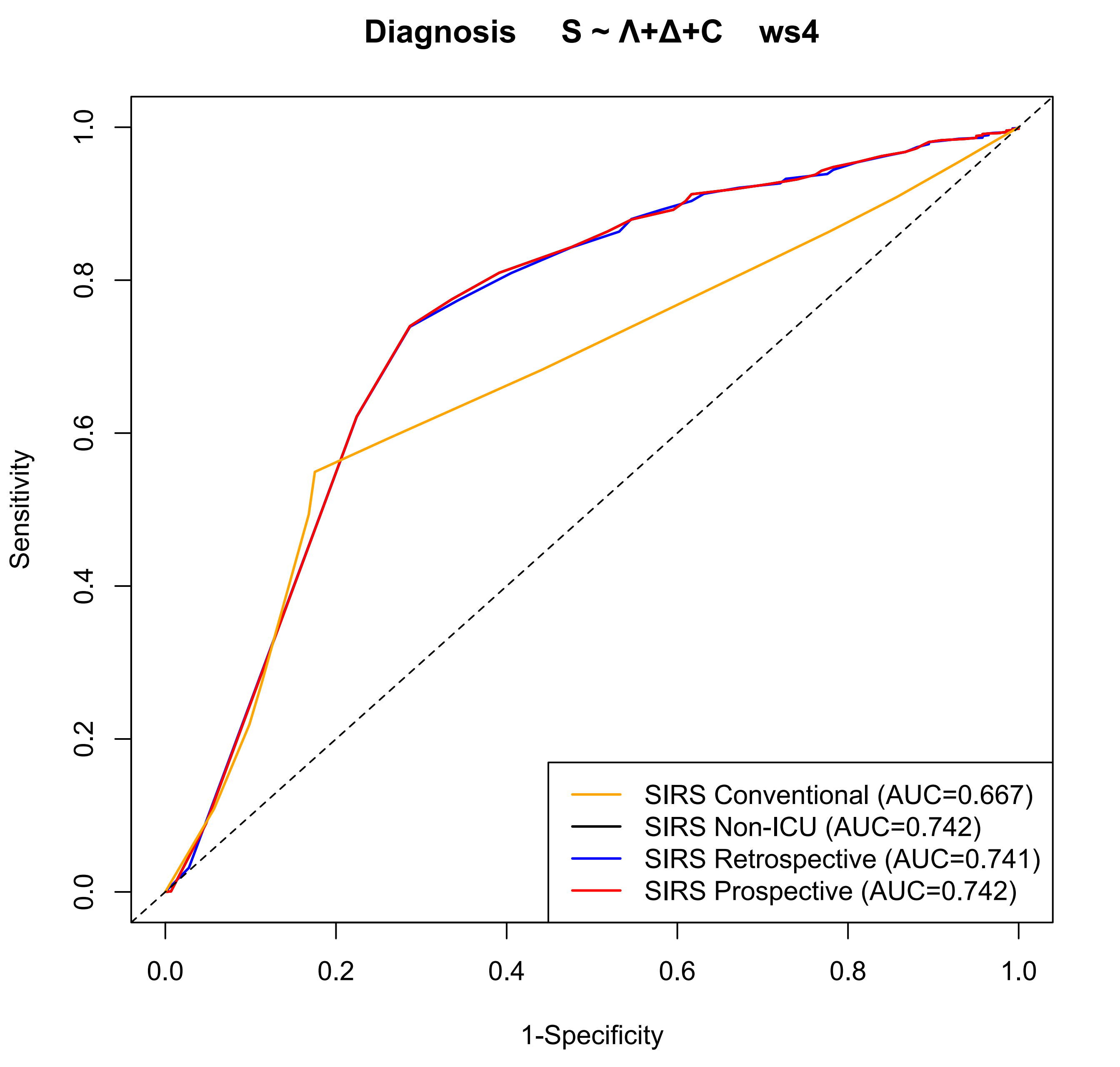
Diagnosis S ~ Λ+Δ+C ws4
Sensitivity
1-Specificity
0.0
0.2
0.4
0.6
0.8
1.0
SIRS Conventional (AUC=0.667)
SIRS Non-ICU (AUC=0.742)
SIRS Retrospective (AUC=0.741)
SIRS Prospective (AUC=0.742)

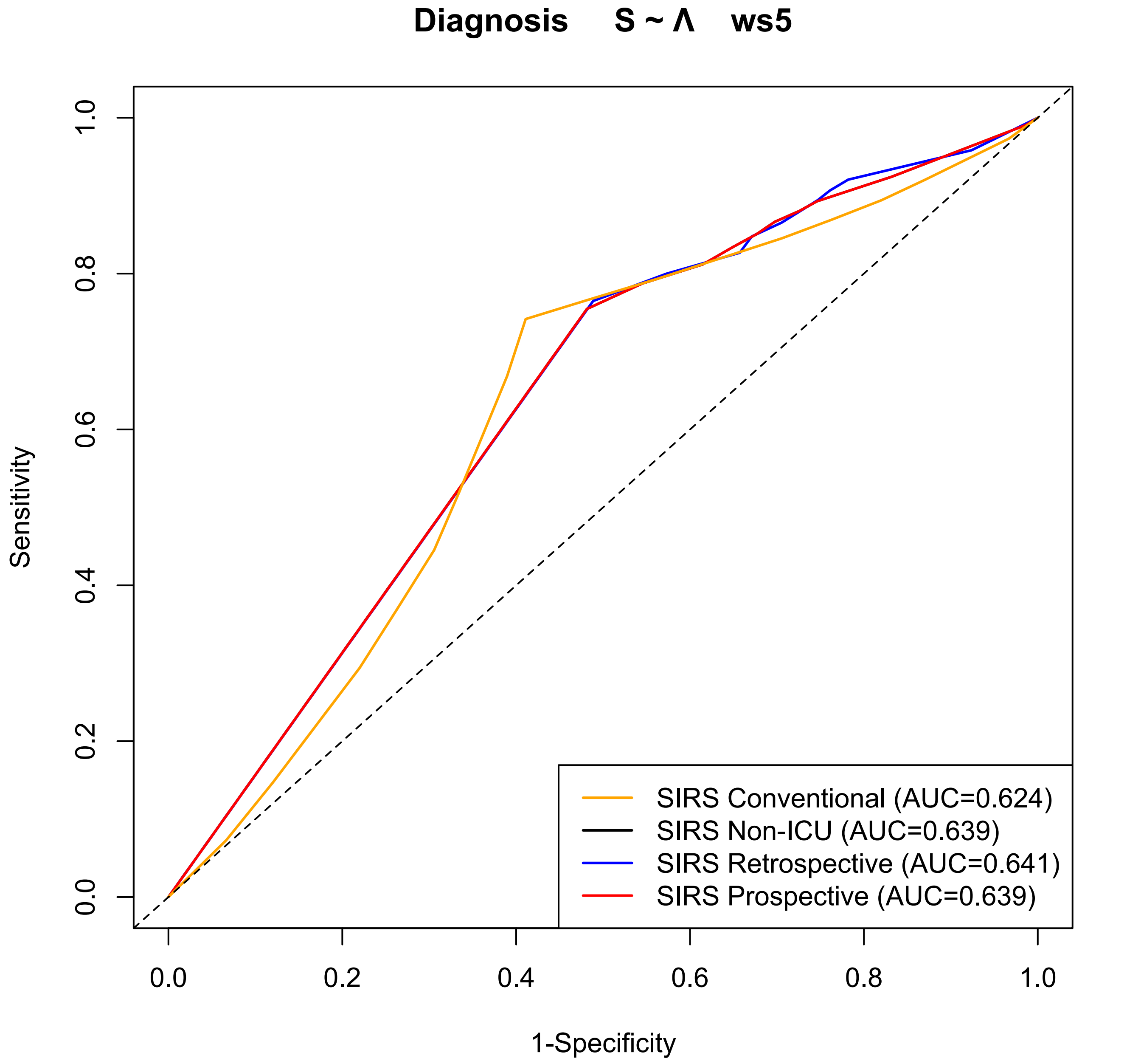

Diagnosis S ~ Λ ws5
Sensitivity
1-Specificity
0.0
0.2
0.4
0.6
0.8
1.0
SIRS Conventional (AUC=0.624)
SIRS Non-ICU (AUC=0.639)
SIRS Retrospective (AUC=0.641)
SIRS Prospective (AUC=0.639)

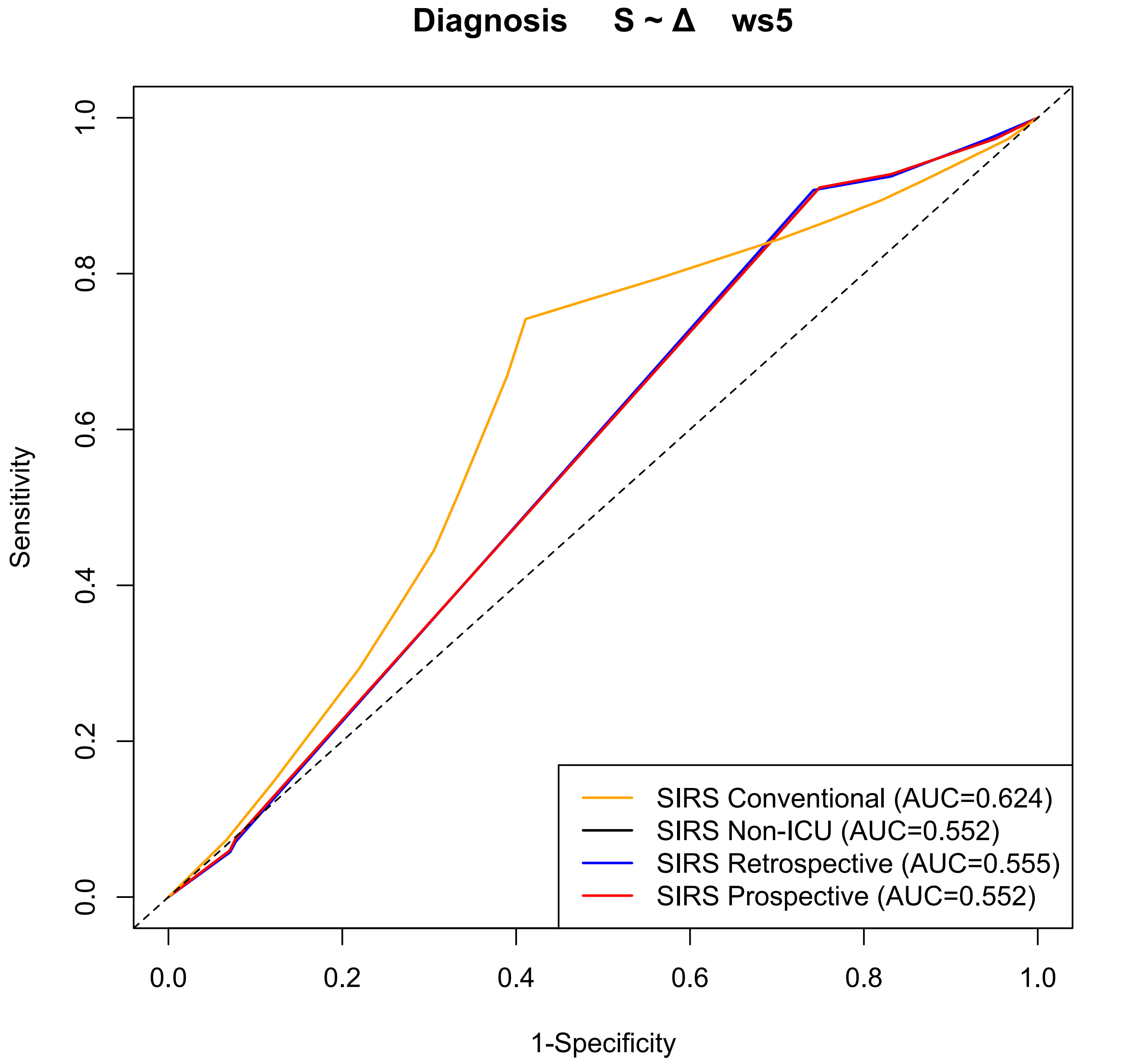
Diagnosis S ~ Δ ws5
Sensitivity
1-Specificity
0.0
0.2
0.4
0.6
0.8
1.0
SIRS Conventional (AUC=0.624)
SIRS Non-ICU (AUC=0.552)
SIRS Retrospective (AUC=0.555)
SIRS Prospective (AUC=0.552)

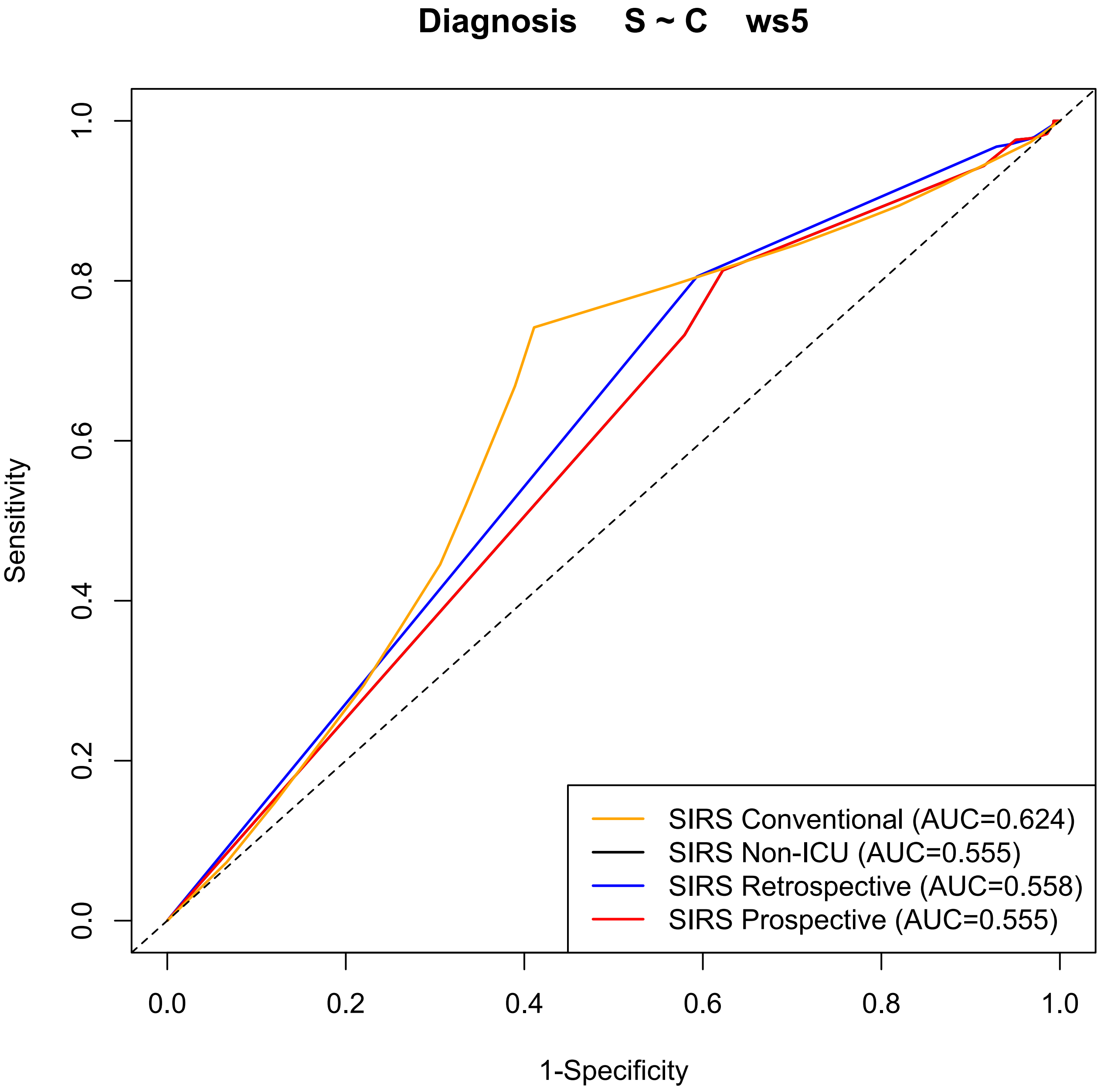
Diagnosis S ~ C ws5
Sensitivity
1-Specificity
0.0
0.2
0.4
0.6
0.8
1.0
SIRS Conventional (AUC=0.624)
SIRS Non-ICU (AUC=0.555)
SIRS Retrospective (AUC=0.558)
SIRS Prospective (AUC=0.555)

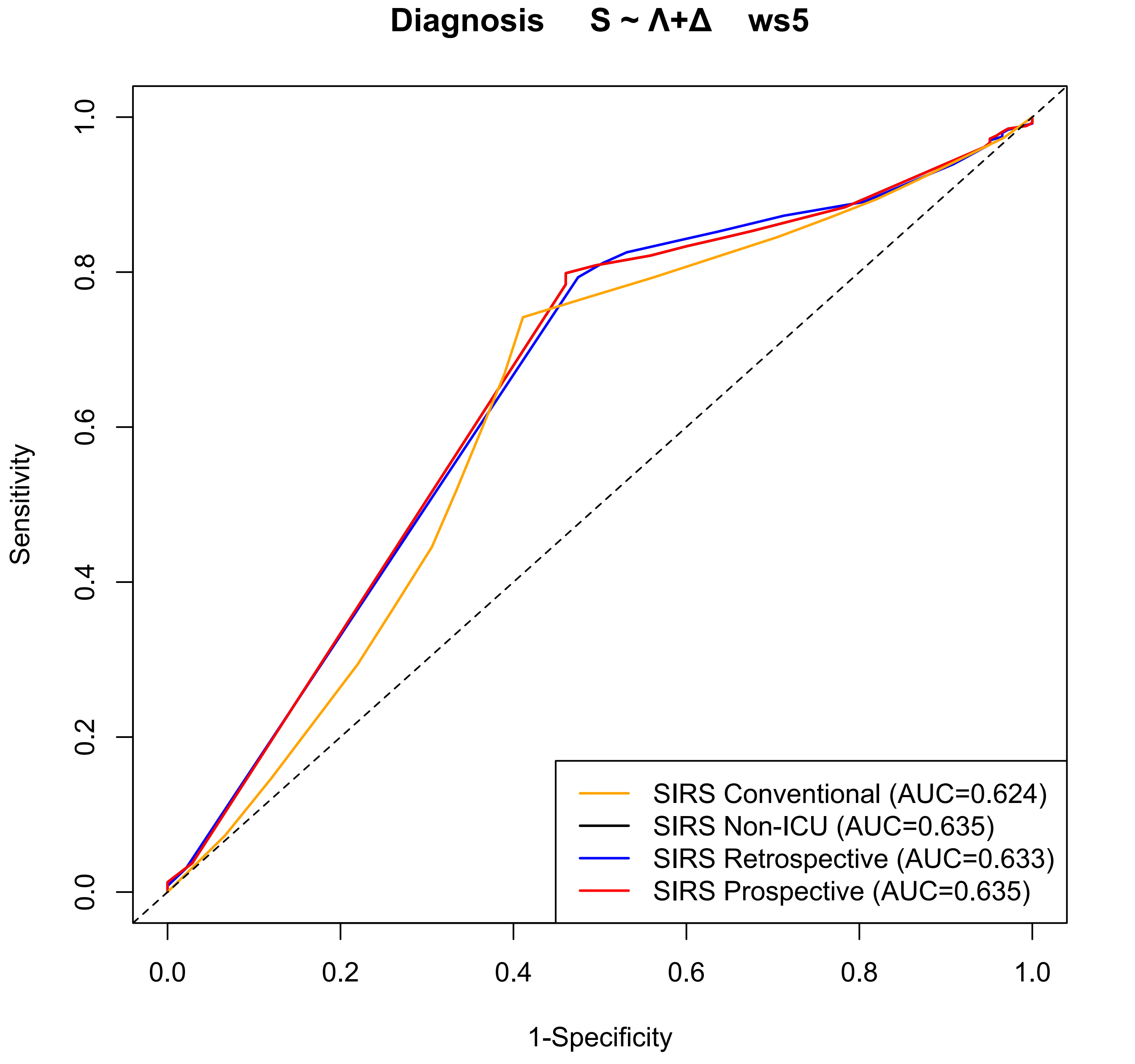
Diagnosis S ~ Λ+Δ ws5
Sensitivity
1-Specificity
0.0
0.2
0.4
0.6
0.8
1.0
SIRS Conventional (AUC=0.624)
SIRS Non-ICU (AUC=0.635)
SIRS Retrospective (AUC=0.633)
SIRS Prospective (AUC=0.635)

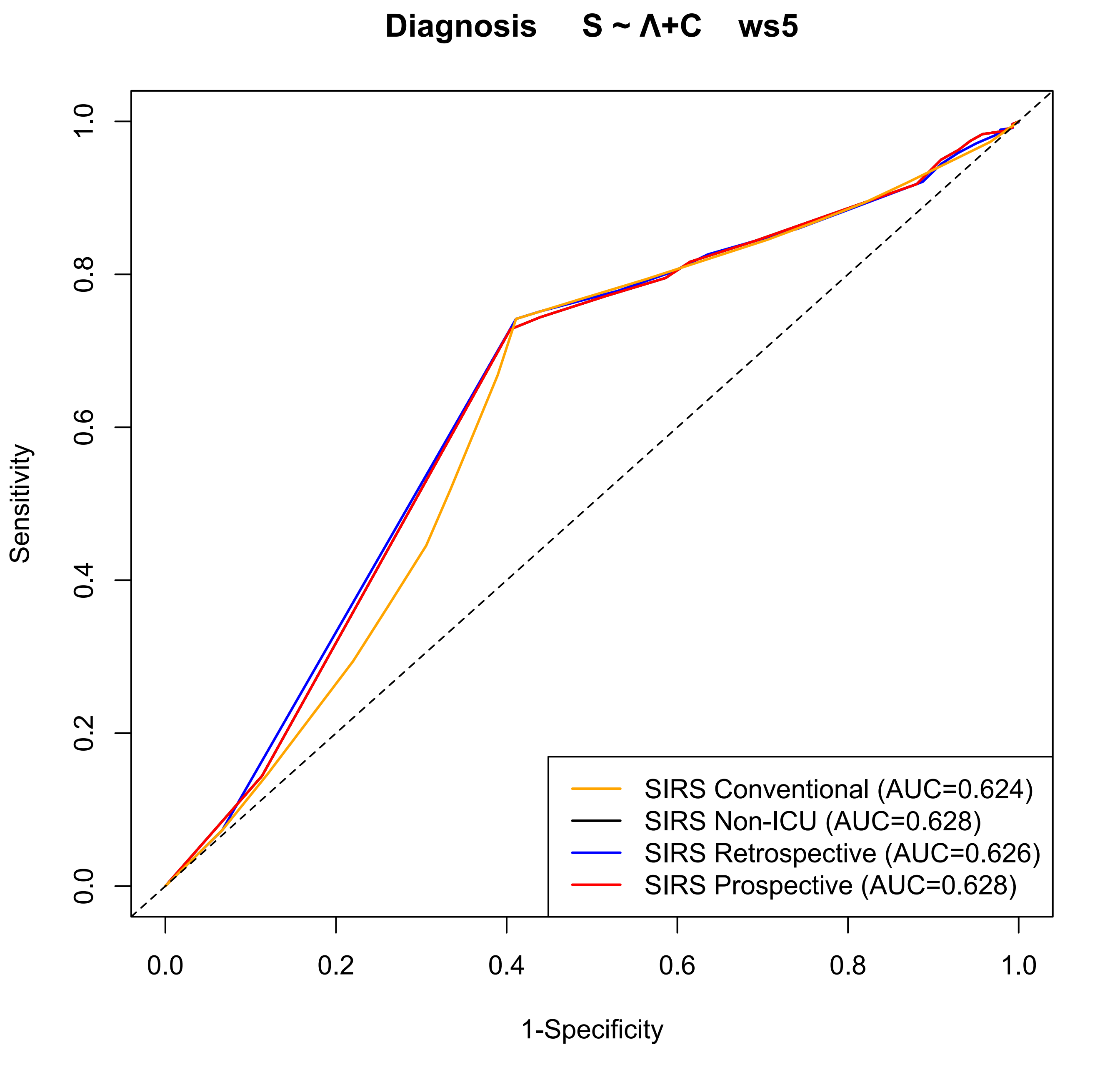
Diagnosis S ~ Λ+C ws5
Sensitivity
1-Specificity
0.0
0.2
0.4
0.6
0.8
1.0
SIRS Conventional (AUC=0.624)
SIRS Non-ICU (AUC=0.628)
SIRS Retrospective (AUC=0.626)
SIRS Prospective (AUC=0.628)

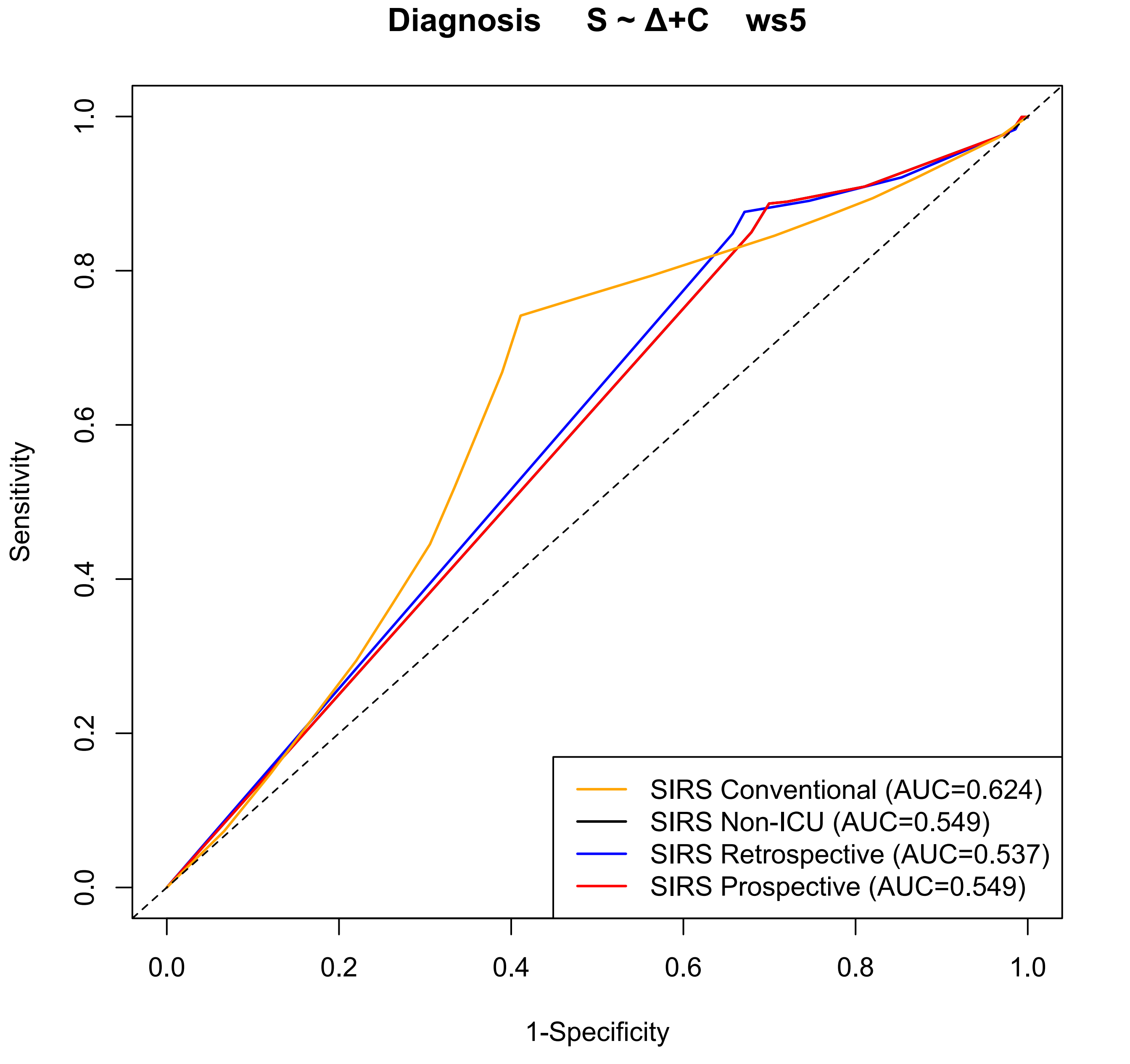

Diagnosis S ~ Δ+C ws5
Sensitivity
1-Specificity
0.0
0.2
0.4
0.6
0.8
1.0
SIRS Conventional (AUC=0.624)
SIRS Non-ICU (AUC=0.549)
SIRS Retrospective (AUC=0.537)
SIRS Prospective (AUC=0.549)

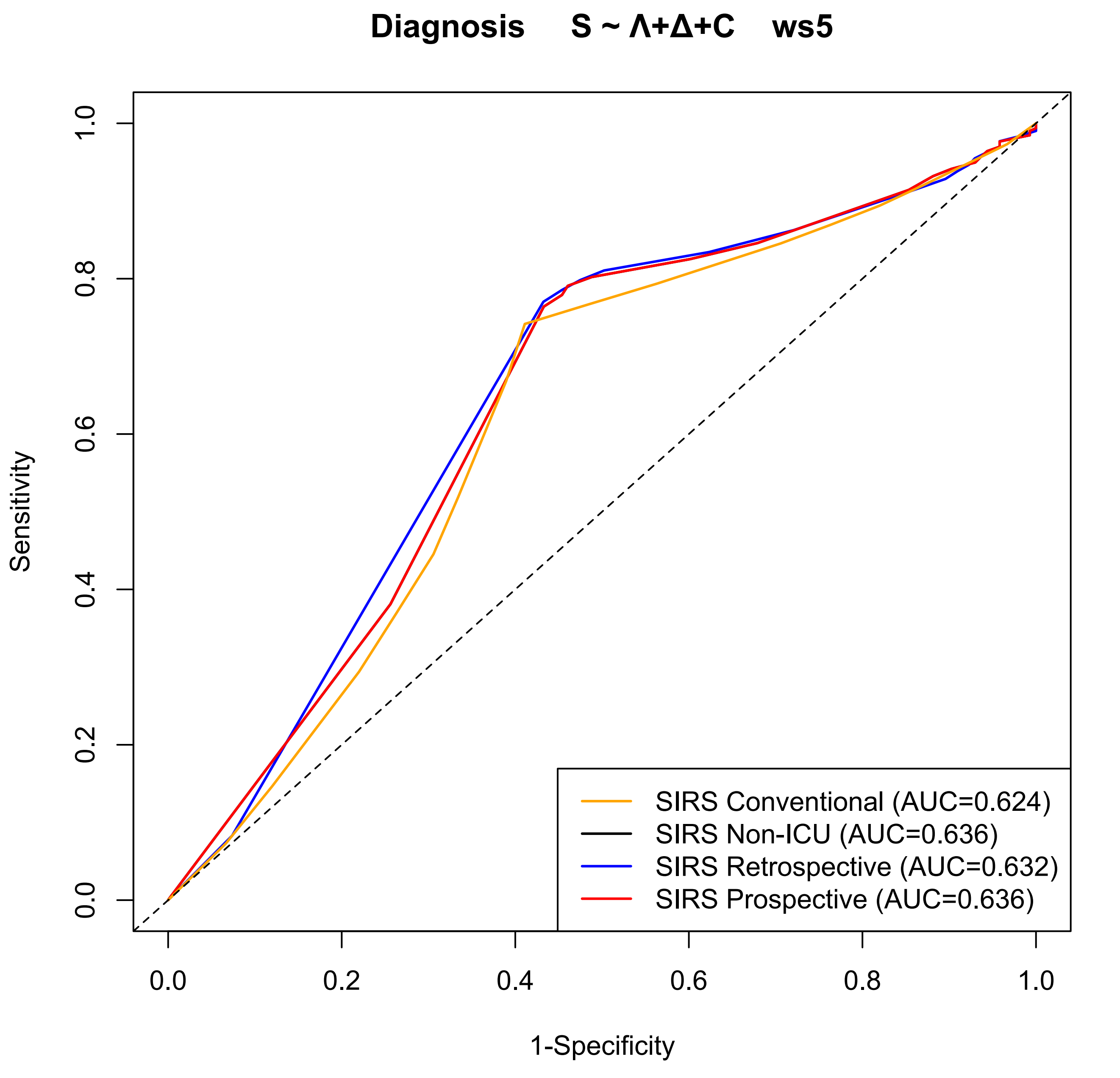

Diagnosis S ~ Λ+Δ+C ws5
Sensitivity
1-Specificity
0.0
0.2
0.4
0.6
0.8
1.0
SIRS Conventional (AUC=0.624)
SIRS Non-ICU (AUC=0.636)
SIRS Retrospective (AUC=0.632)
SIRS Prospective (AUC=0.636)

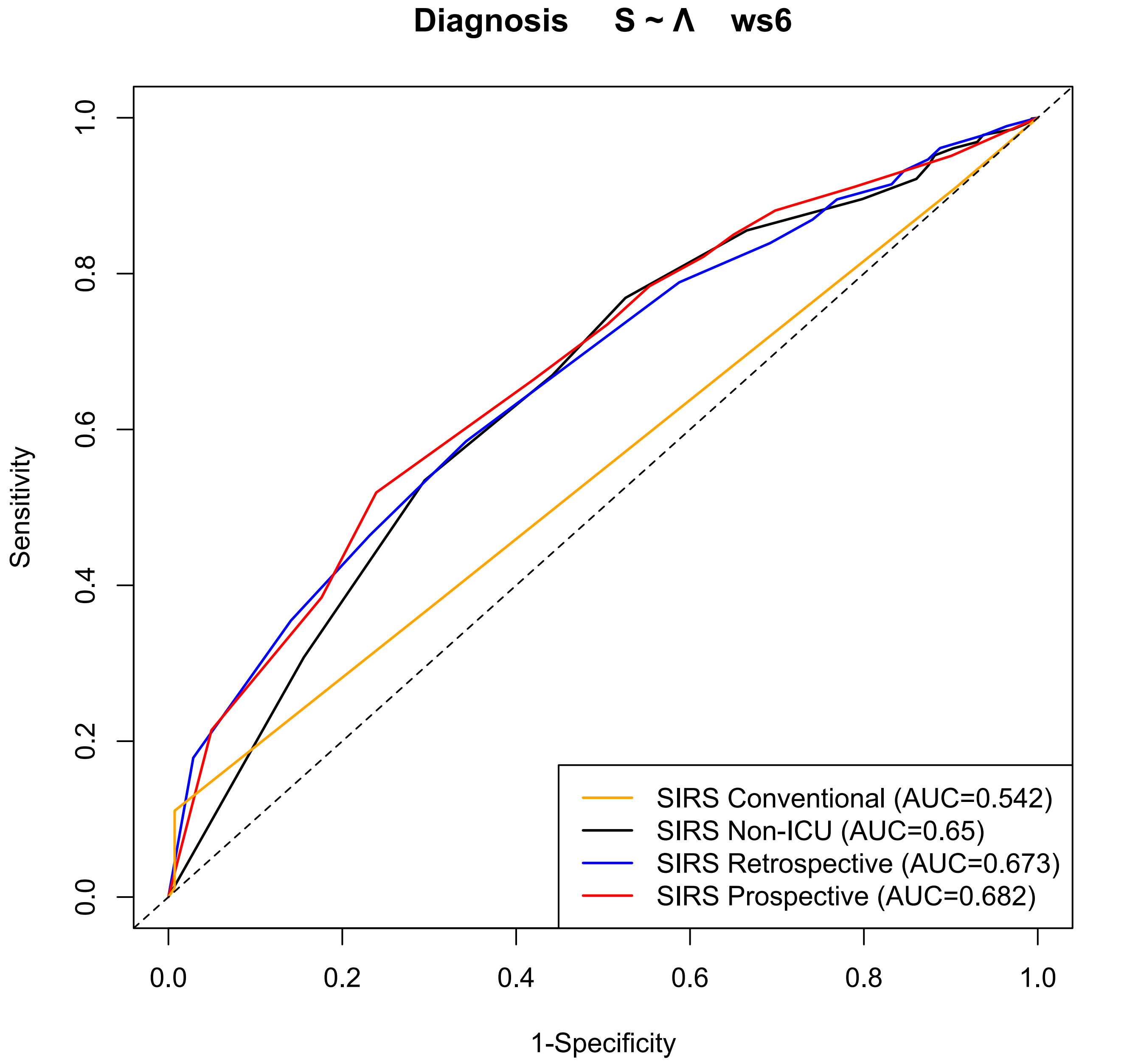
Diagnosis S ~ Λ ws6
Sensitivity
1-Specificity
0.0
0.2
0.4
0.6
0.8
1.0
SIRS Conventional (AUC=0.542)
SIRS Non-ICU (AUC=0.65)
SIRS Retrospective (AUC=0.673)
SIRS Prospective (AUC=0.682)

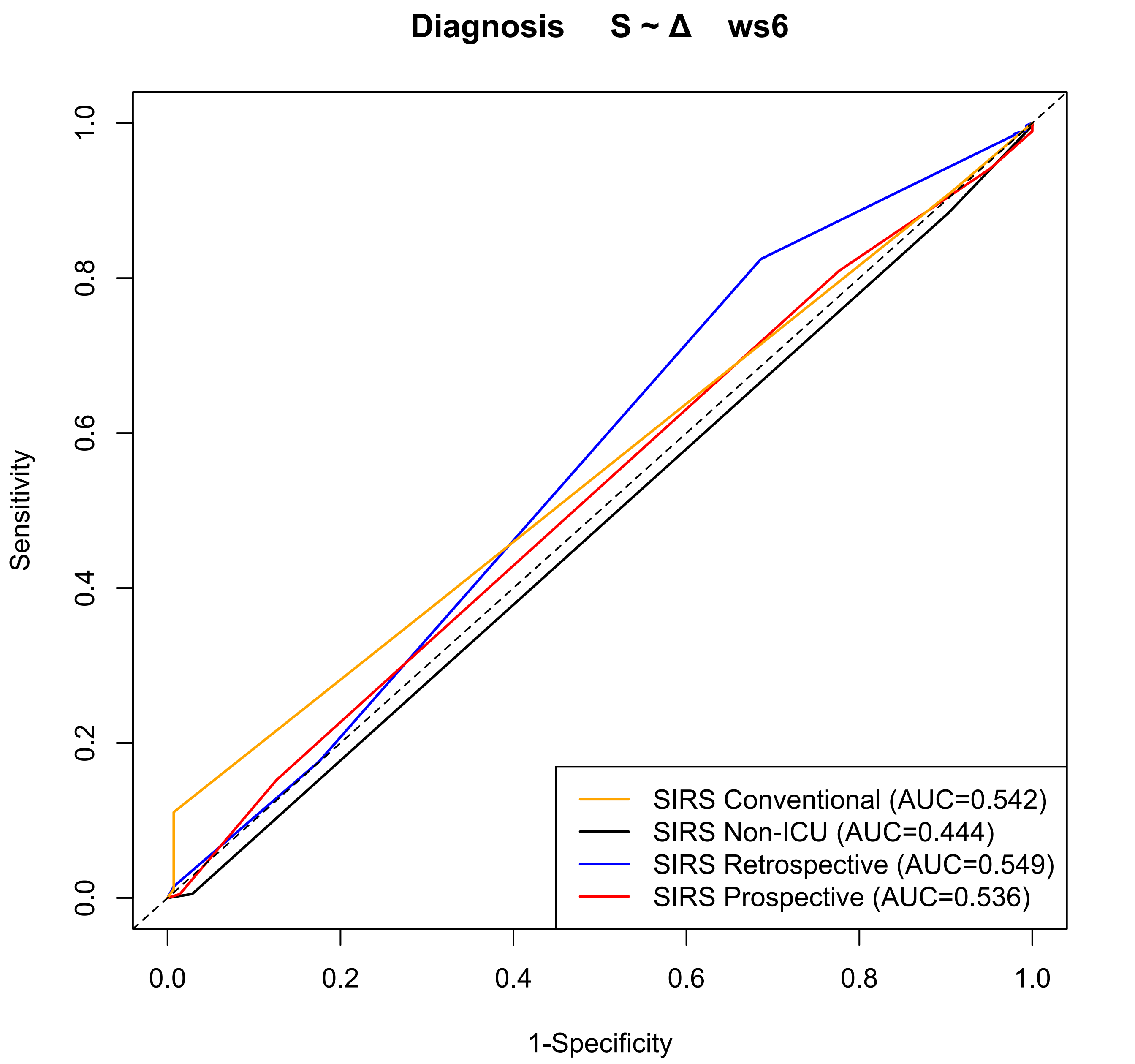

Diagnosis S ~ Δ ws6
Sensitivity
1-Specificity
0.0
0.2
0.4
0.6
0.8
1.0
0.0
0.2
0.4
0.6
0.8
1.0
SIRS Conventional (AUC=0.542)
SIRS Non-ICU (AUC=0.444)
SIRS Retrospective (AUC=0.549)
SIRS Prospective (AUC=0.536)

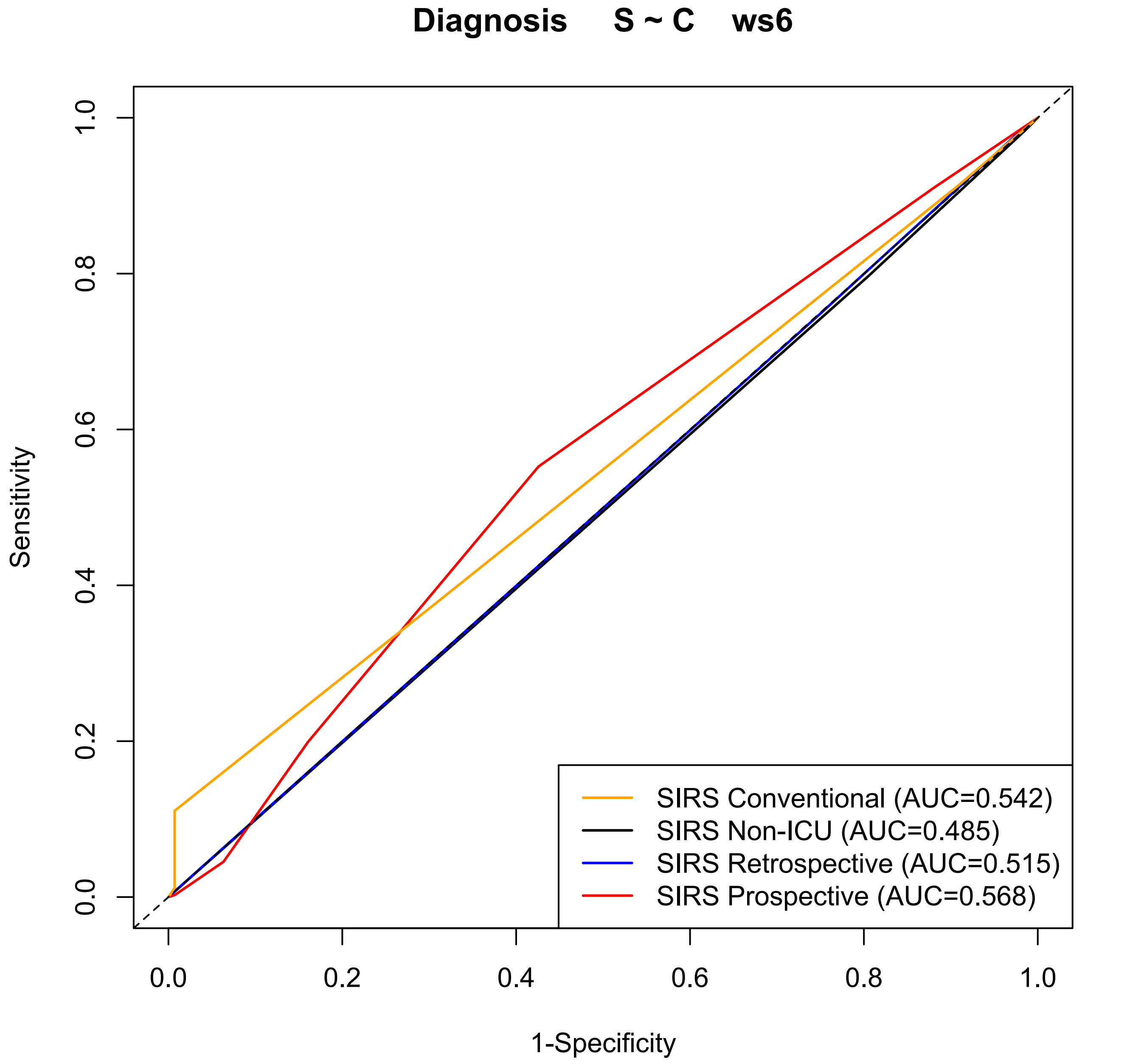
Diagnosis S ~ C ws6
Sensitivity
1-Specificity
0.0
0.2
0.4
0.6
0.8
1.0
SIRS Conventional (AUC=0.542)
SIRS Non-ICU (AUC=0.485)
SIRS Retrospective (AUC=0.515)
SIRS Prospective (AUC=0.568)

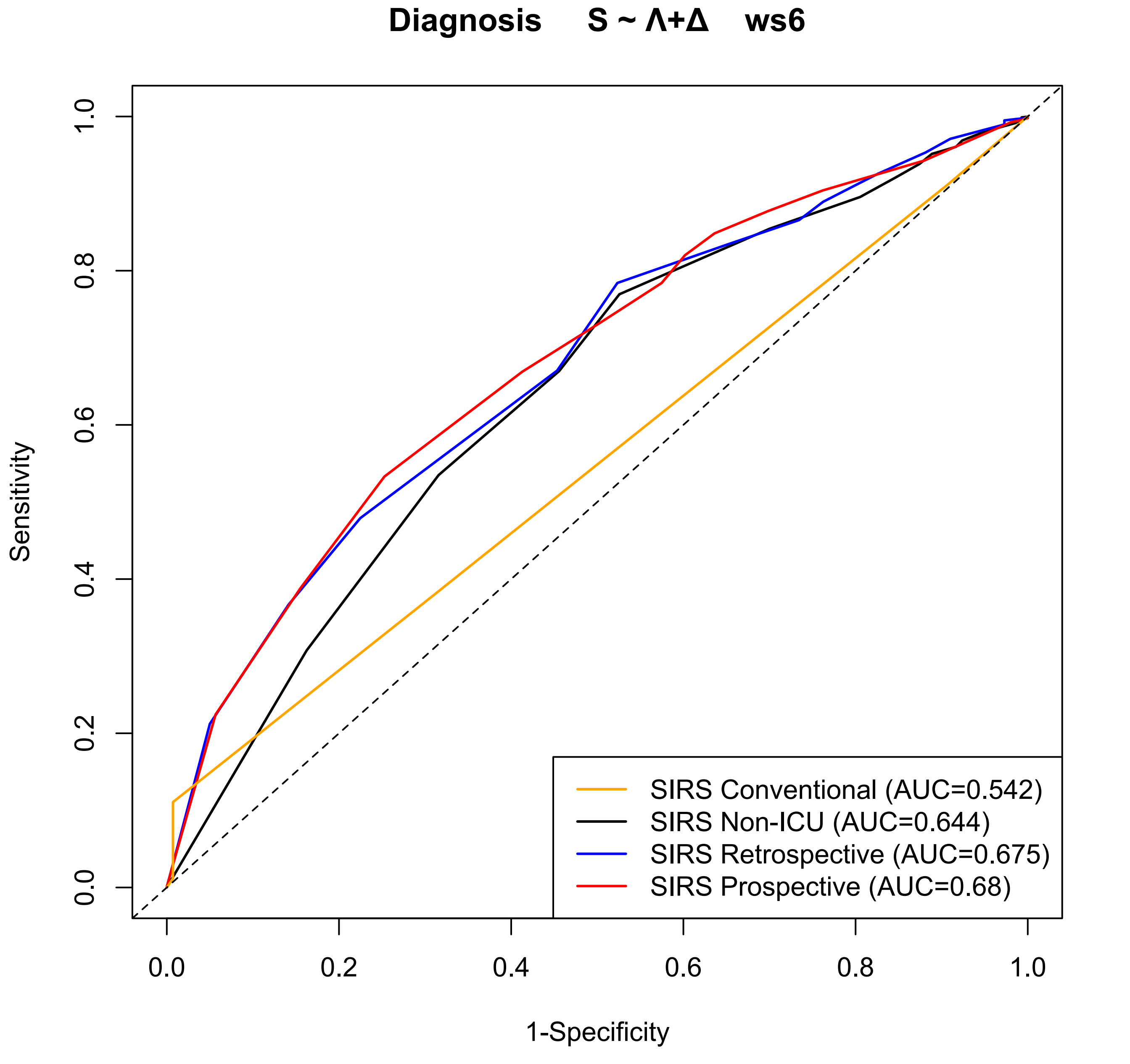

Diagnosis S ~ Λ+Δ ws6
Sensitivity
1-Specificity
0.0
0.2
0.4
0.6
0.8
1.0
0.0
0.2
0.4
0.6
0.8
1.0
SIRS Conventional (AUC=0.542)
SIRS Non-ICU (AUC=0.644)
SIRS Retrospective (AUC=0.675)
SIRS Prospective (AUC=0.68)

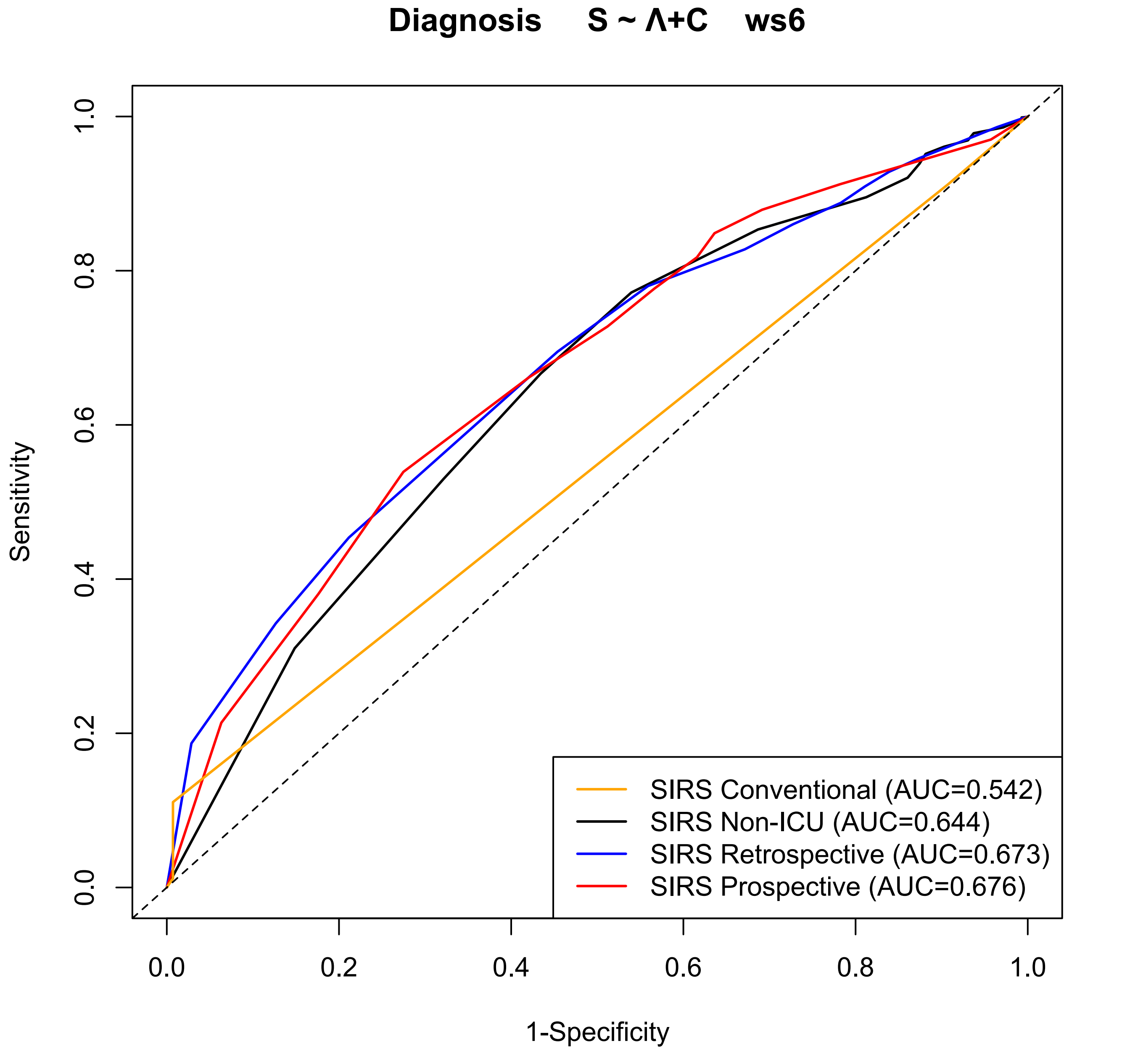
Diagnosis S ~ Λ+C ws6
Sensitivity
1-Specificity
0.0
0.2
0.4
0.6
0.8
1.0
SIRS Conventional (AUC=0.542)
SIRS Non-ICU (AUC=0.644)
SIRS Retrospective (AUC=0.673)
SIRS Prospective (AUC=0.676)

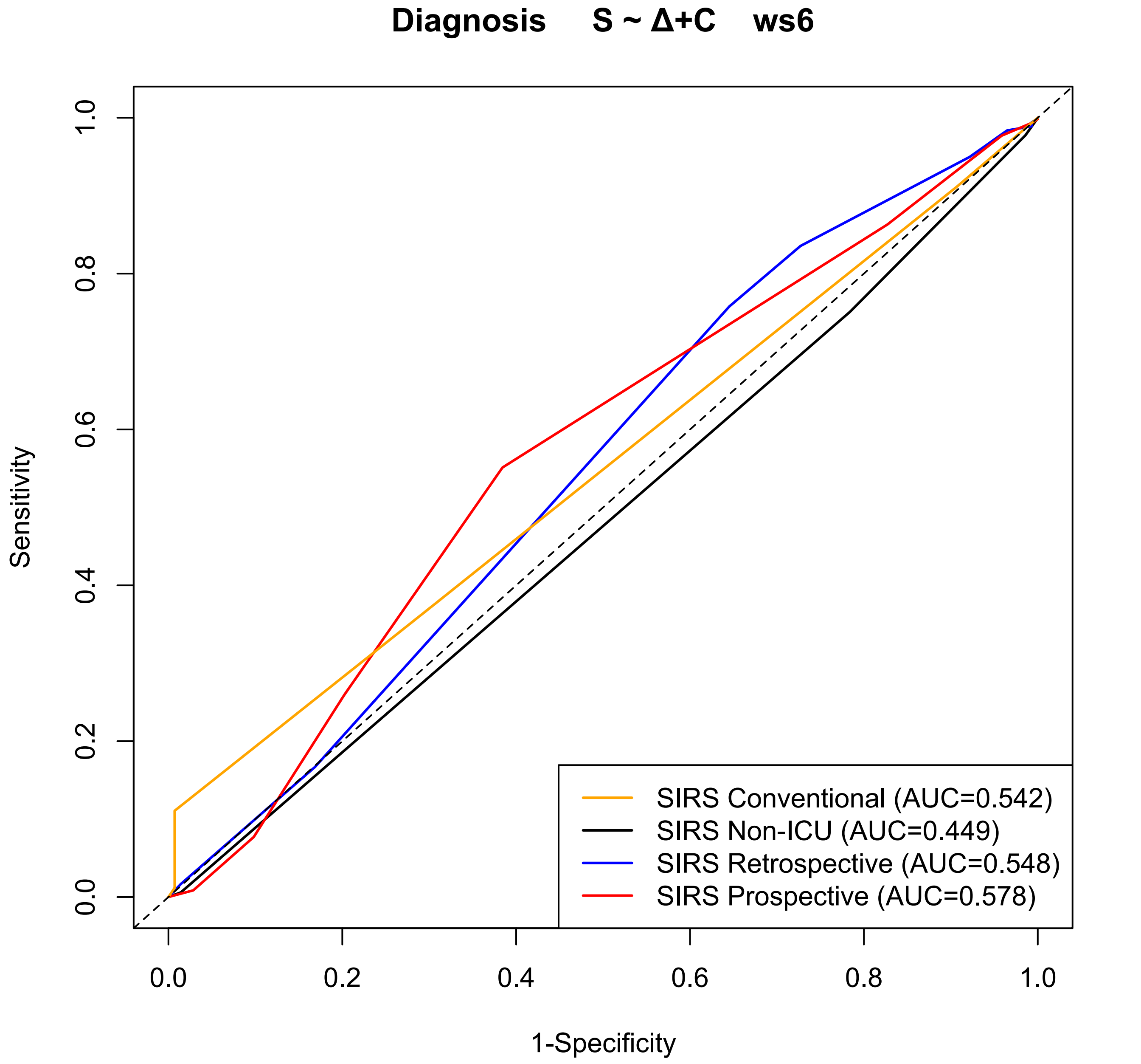

Diagnosis S ~ Δ+C ws6
Sensitivity
1-Specificity
0.0
0.2
0.4
0.6
0.8
1.0
SIRS Conventional (AUC=0.542)
SIRS Non-ICU (AUC=0.449)
SIRS Retrospective (AUC=0.548)
SIRS Prospective (AUC=0.578)

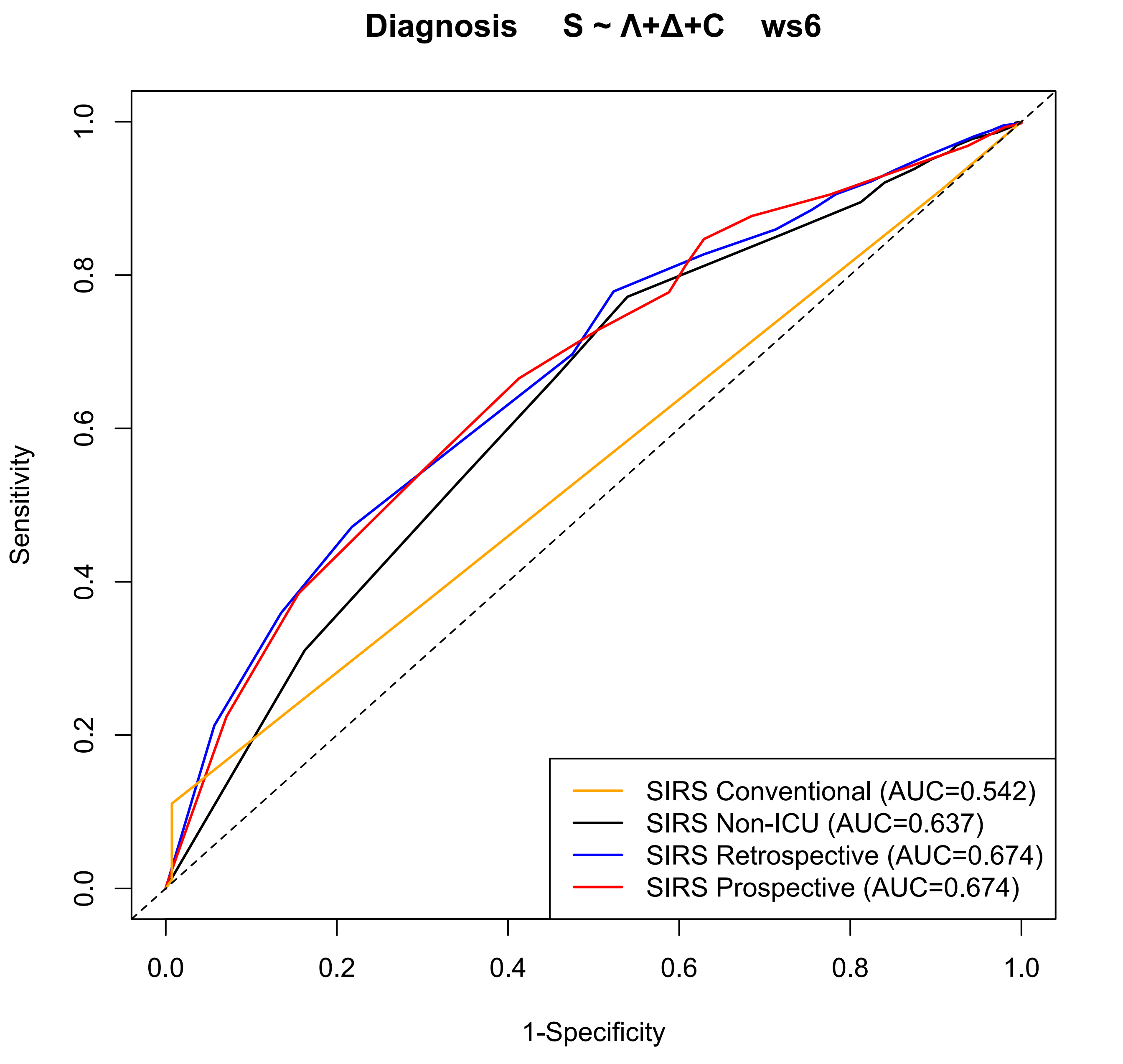

Diagnosis S ~ Λ+Δ+C ws6
Sensitivity
1-Specificity
0.0
0.2
0.4
0.6
0.8
1.0
SIRS Conventional (AUC=0.542)
SIRS Non-ICU (AUC=0.637)
SIRS Retrospective (AUC=0.674)
SIRS Prospective (AUC=0.674)

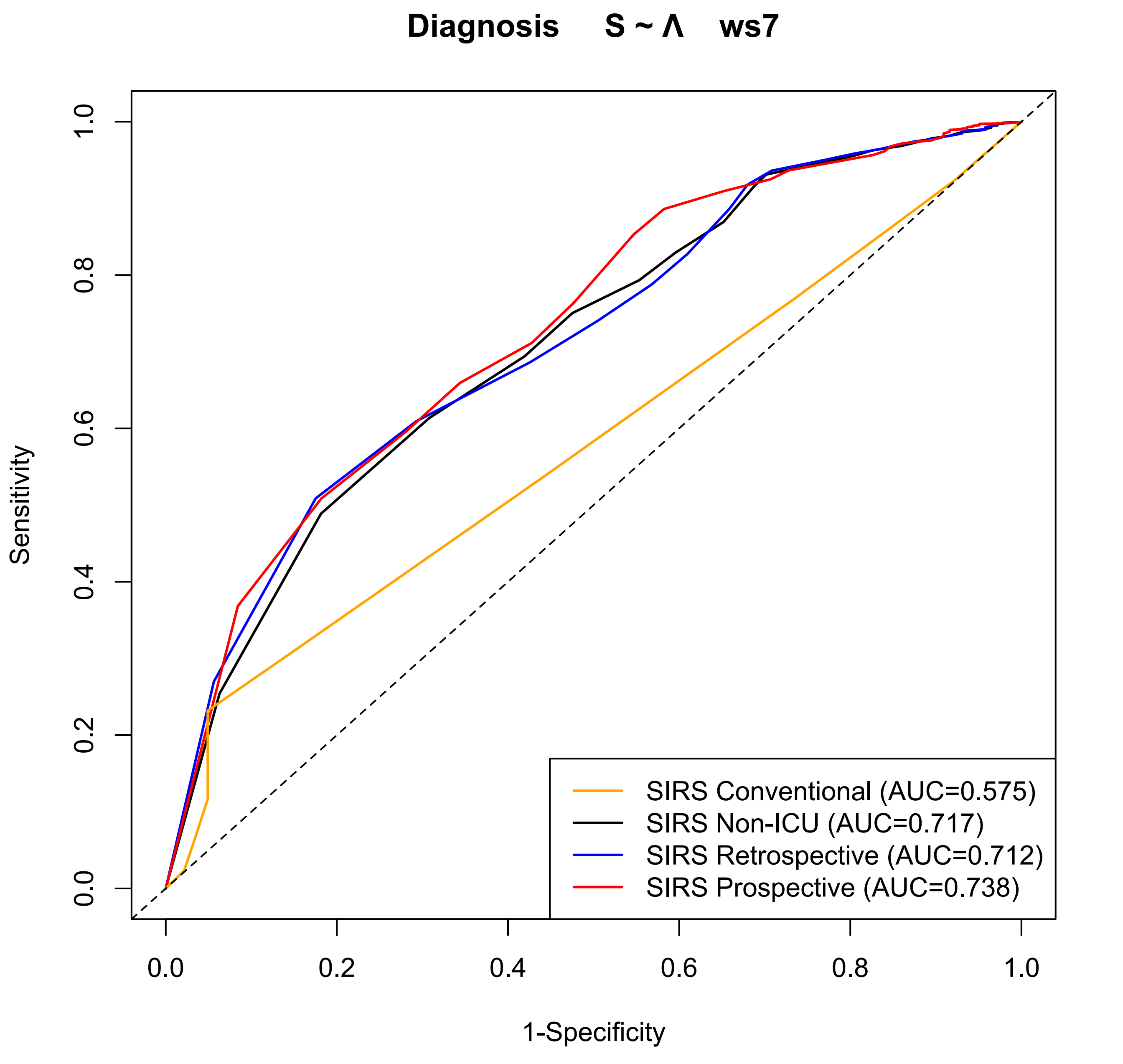

Diagnosis S ~ Λ ws7
Sensitivity
1-Specificity
0.0
0.2
0.4
0.6
0.8
1.0
SIRS Conventional (AUC=0.575)
SIRS Non-ICU (AUC=0.717)
SIRS Retrospective (AUC=0.712)
SIRS Prospective (AUC=0.738)

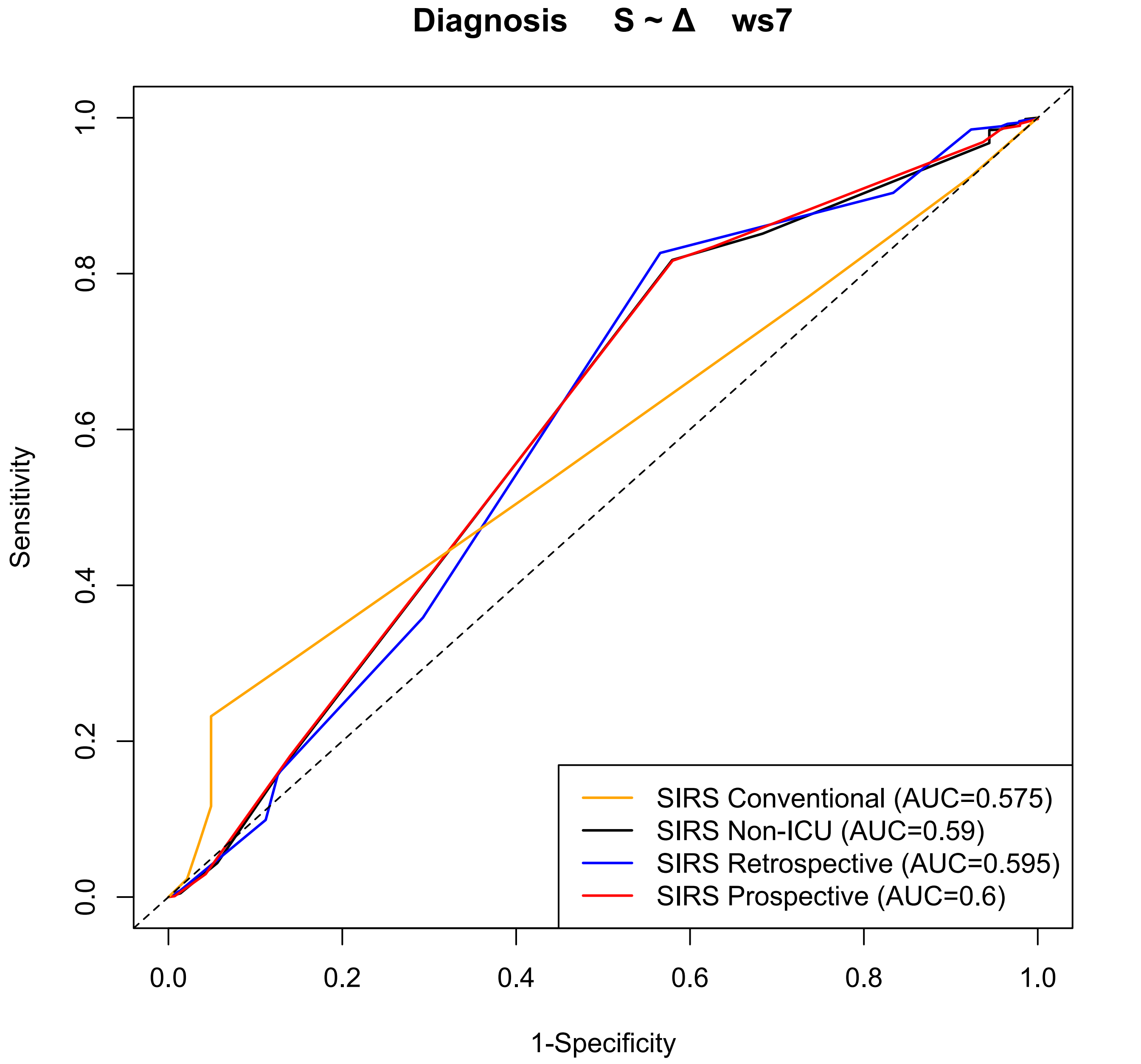
Diagnosis S ~ Δ ws7
Sensitivity
1-Specificity
0.0
0.2
0.4
0.6
0.8
1.0
0.0
0.2
0.4
0.6
0.8
1.0
SIRS Conventional (AUC=0.575)
SIRS Non-ICU (AUC=0.59)
SIRS Retrospective (AUC=0.595)
SIRS Prospective (AUC=0.6)

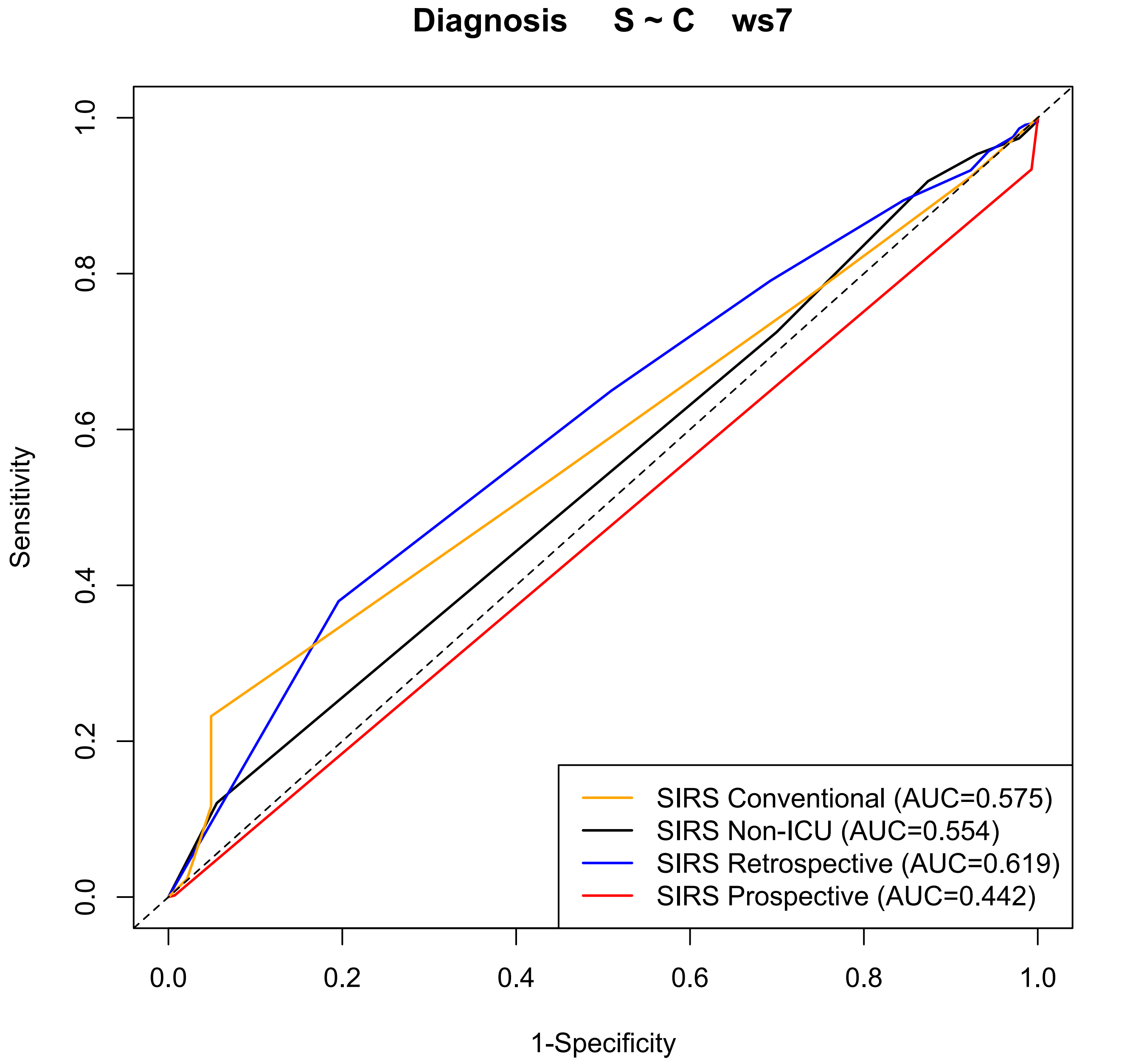

Diagnosis S ~ C ws7
Sensitivity
1-Specificity
0.0
0.2
0.4
0.6
0.8
1.0
SIRS Conventional (AUC=0.575)
SIRS Non-ICU (AUC=0.554)
SIRS Retrospective (AUC=0.619)
SIRS Prospective (AUC=0.442)

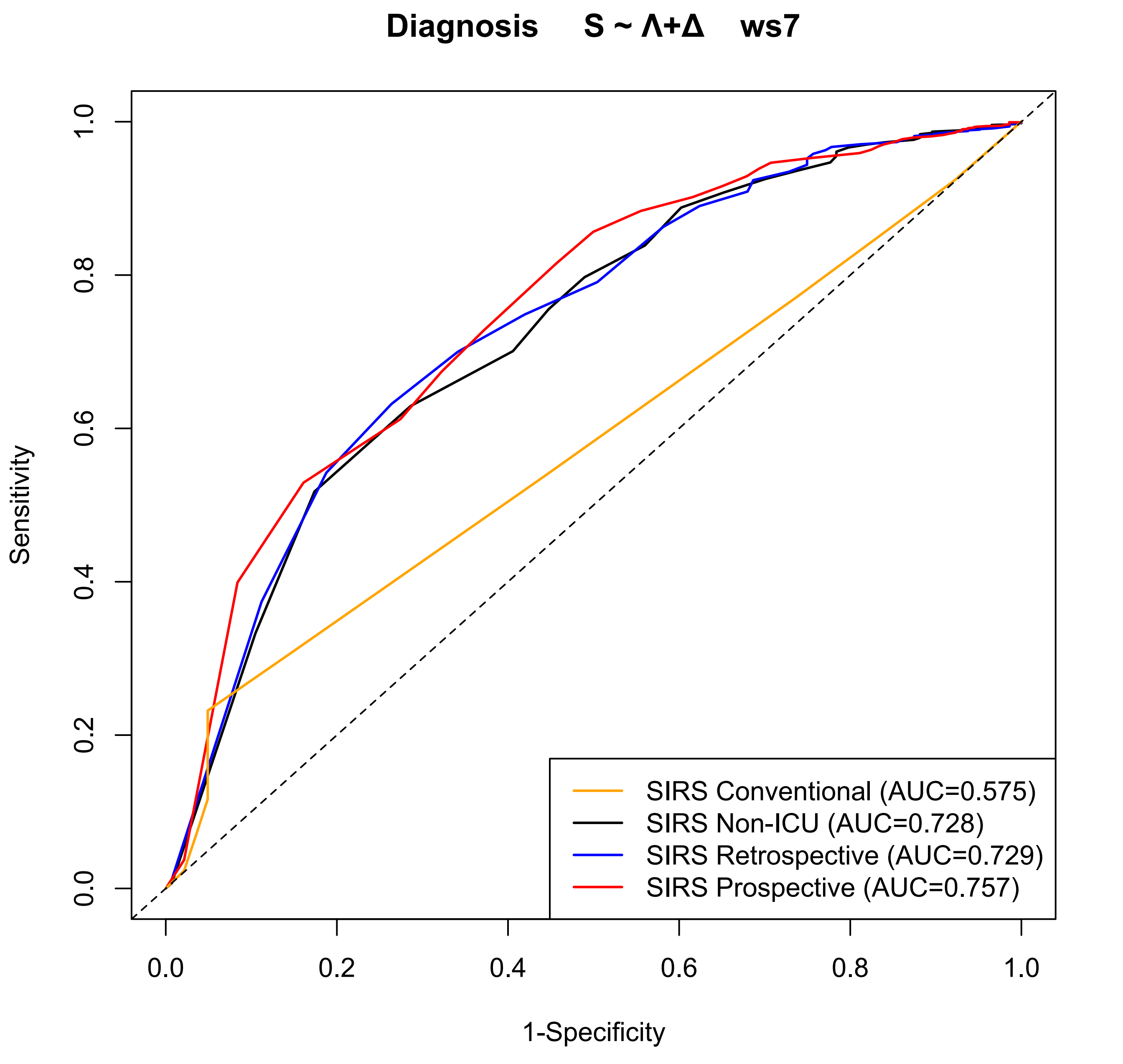

Diagnosis S ~ Λ+Δ ws7
Sensitivity
1-Specificity
0.0
0.2
0.4
0.6
0.8
1.0
SIRS Conventional (AUC=0.575)
SIRS Non-ICU (AUC=0.728)
SIRS Retrospective (AUC=0.729)
SIRS Prospective (AUC=0.757)

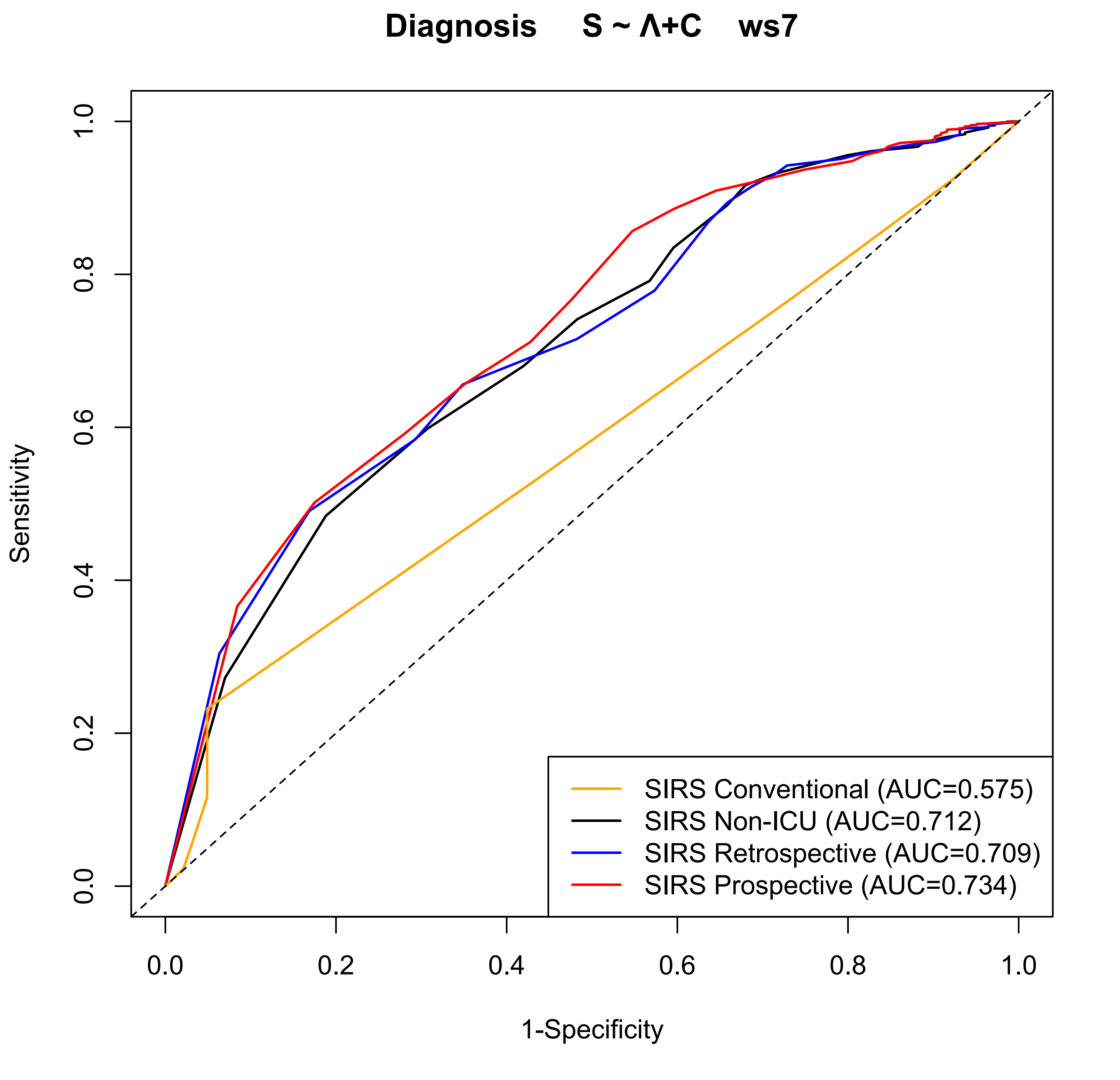
Diagnosis S ~ Λ+C ws7
Sensitivity
1-Specificity
0.0
0.2
0.4
0.6
0.8
1.0
SIRS Conventional (AUC=0.575)
SIRS Non-ICU (AUC=0.712)
SIRS Retrospective (AUC=0.709)
SIRS Prospective (AUC=0.734)

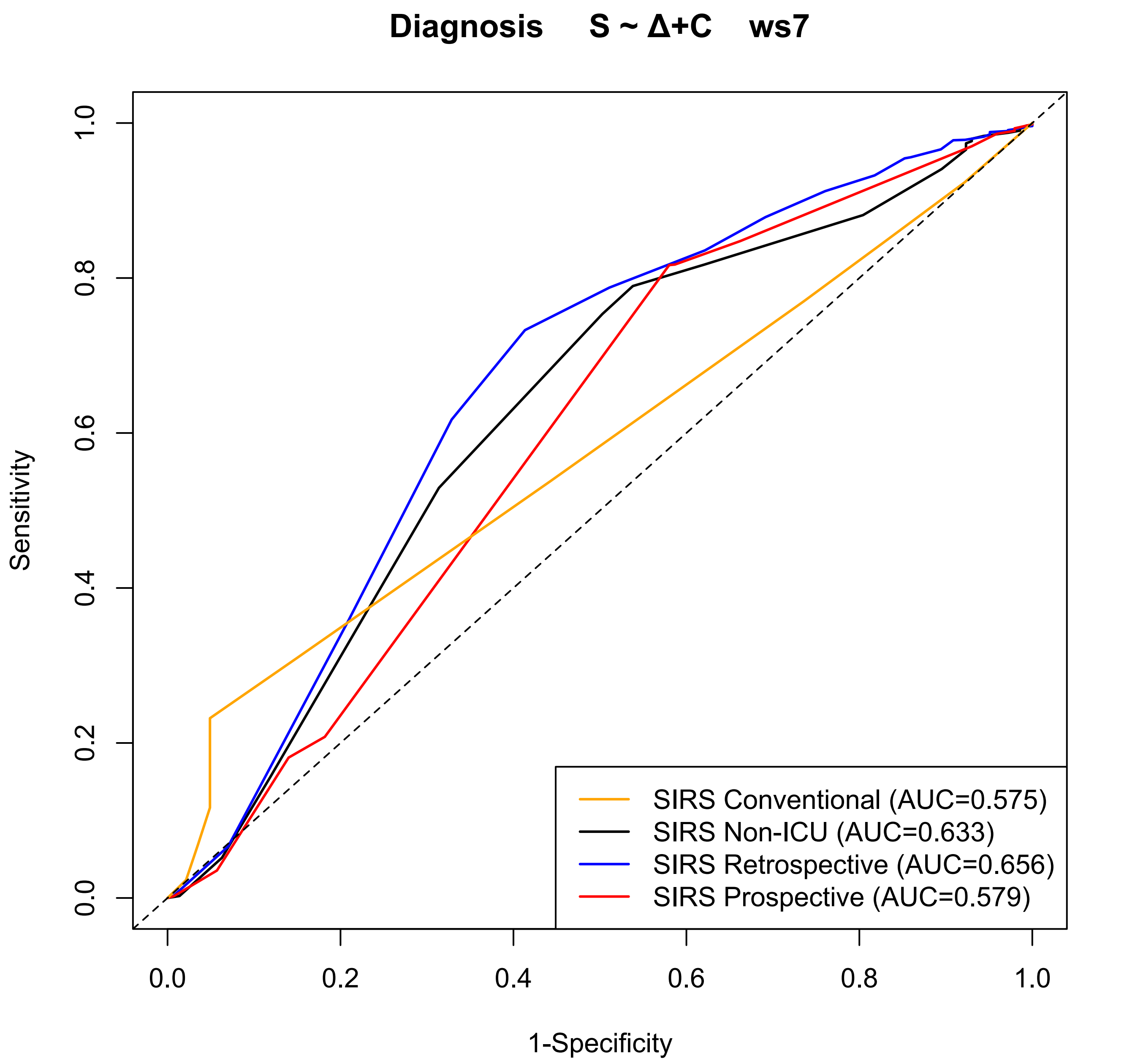
Diagnosis S ~ Δ+C ws7
Sensitivity
1-Specificity
0.0
0.2
0.4
0.6
0.8
1.0
SIRS Conventional (AUC=0.575)
SIRS Non-ICU (AUC=0.633)
SIRS Retrospective (AUC=0.656)
SIRS Prospective (AUC=0.579)

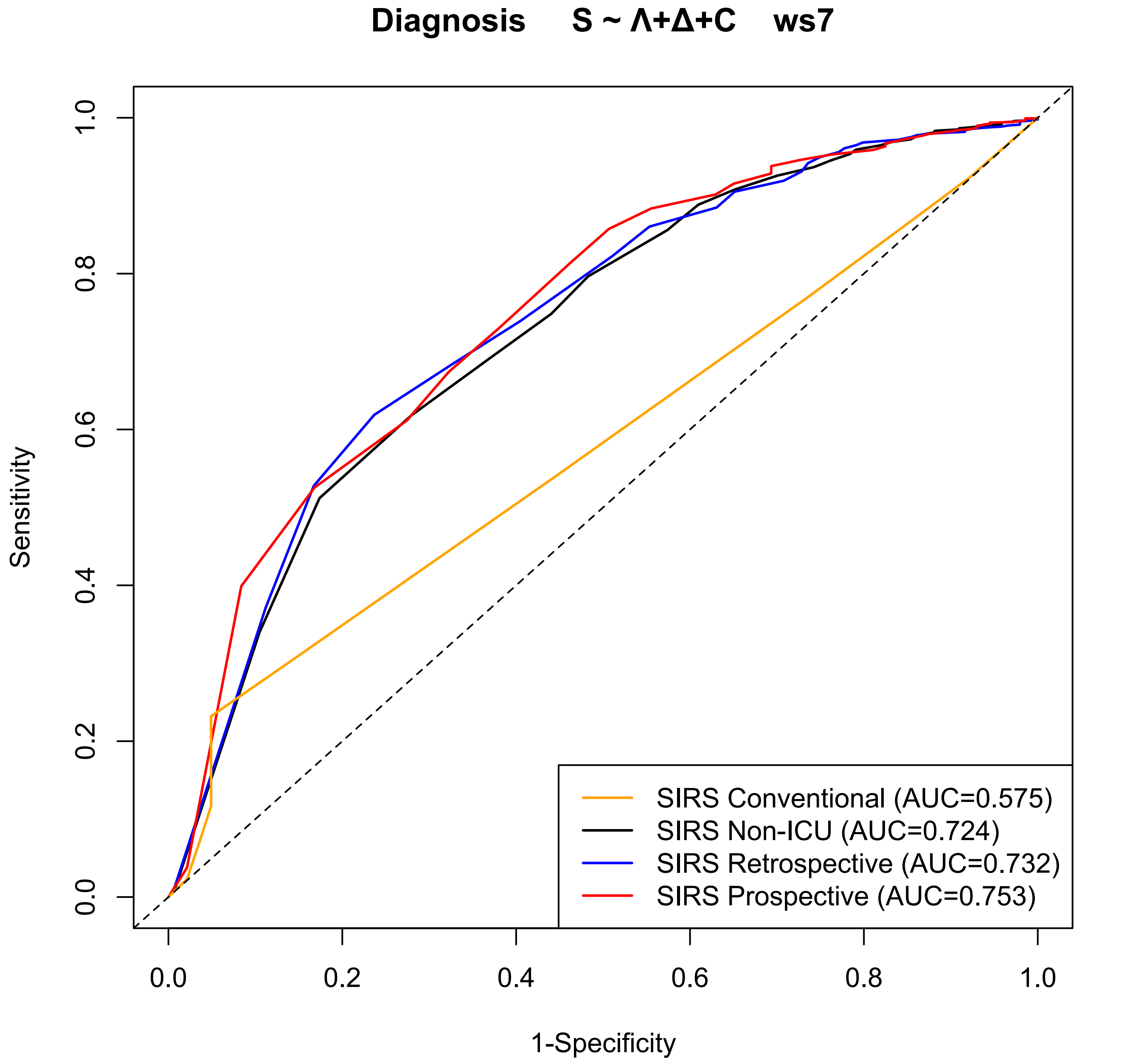

Diagnosis S ~ Λ+Δ+C ws7
Sensitivity
1-Specificity
0.0
0.2
0.4
0.6
0.8
1.0
SIRS Conventional (AUC=0.575)
SIRS Non-ICU (AUC=0.724)
SIRS Retrospective (AUC=0.732)
SIRS Prospective (AUC=0.753)

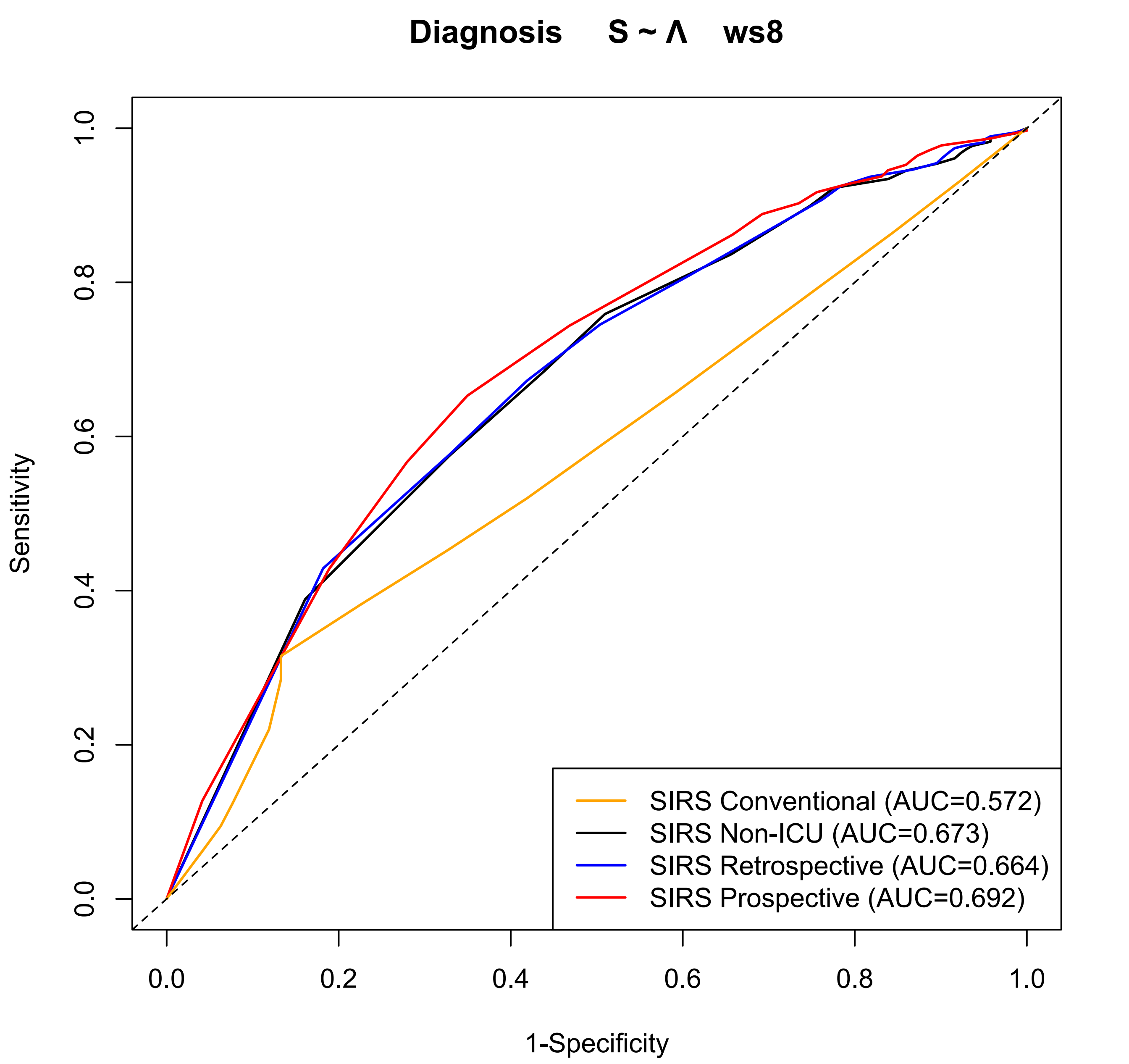
Diagnosis S ~ Λ ws8
Sensitivity
1-Specificity
0.0
0.2
0.4
0.6
0.8
1.0
SIRS Conventional (AUC=0.572)
SIRS Non-ICU (AUC=0.673)
SIRS Retrospective (AUC=0.664)
SIRS Prospective (AUC=0.692)

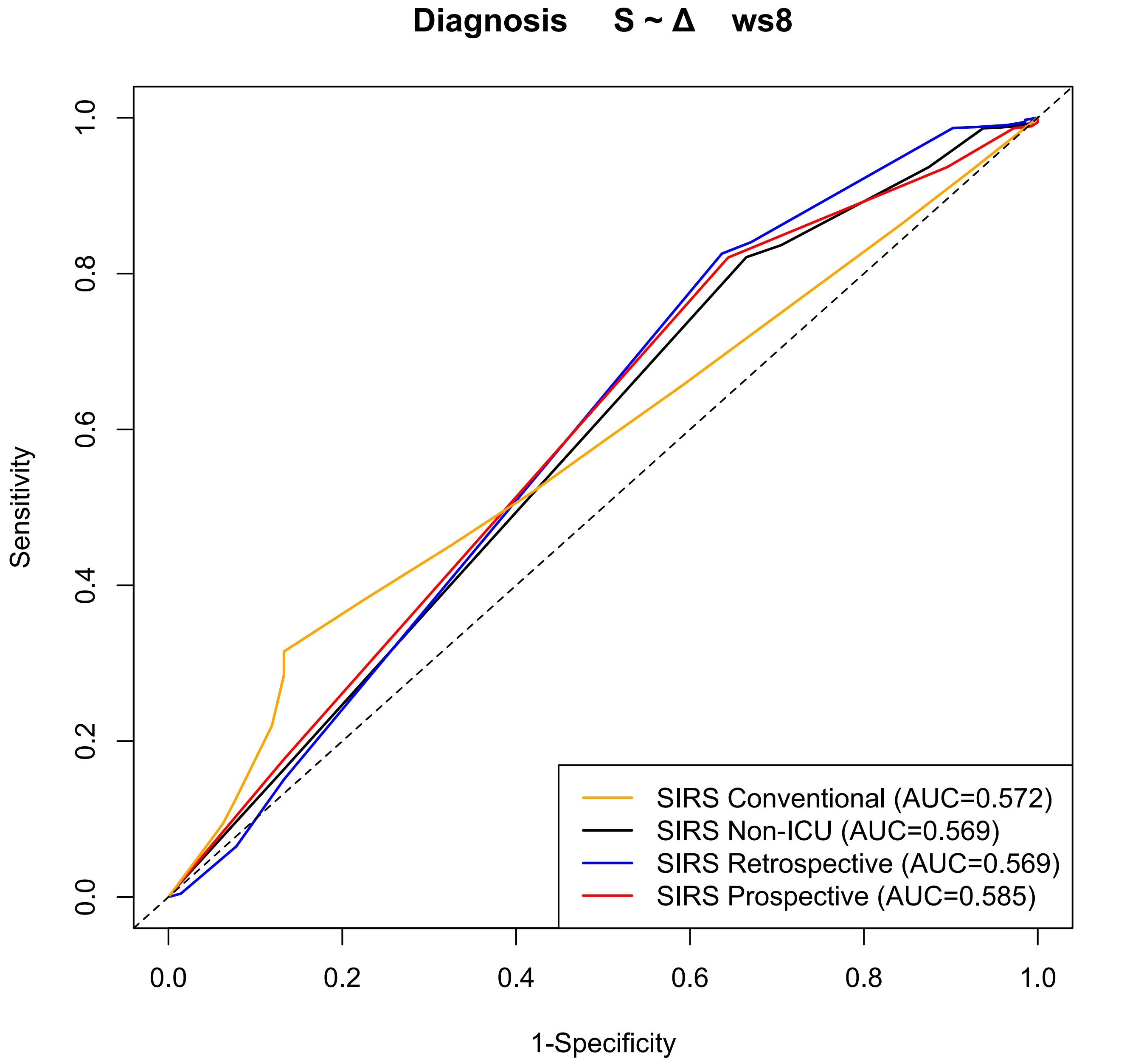
Diagnosis S ~ Δ ws8
Sensitivity
1-Specificity
0.0
0.2
0.4
0.6
0.8
1.0
SIRS Conventional (AUC=0.572)
SIRS Non-ICU (AUC=0.569)
SIRS Retrospective (AUC=0.569)
SIRS Prospective (AUC=0.585)

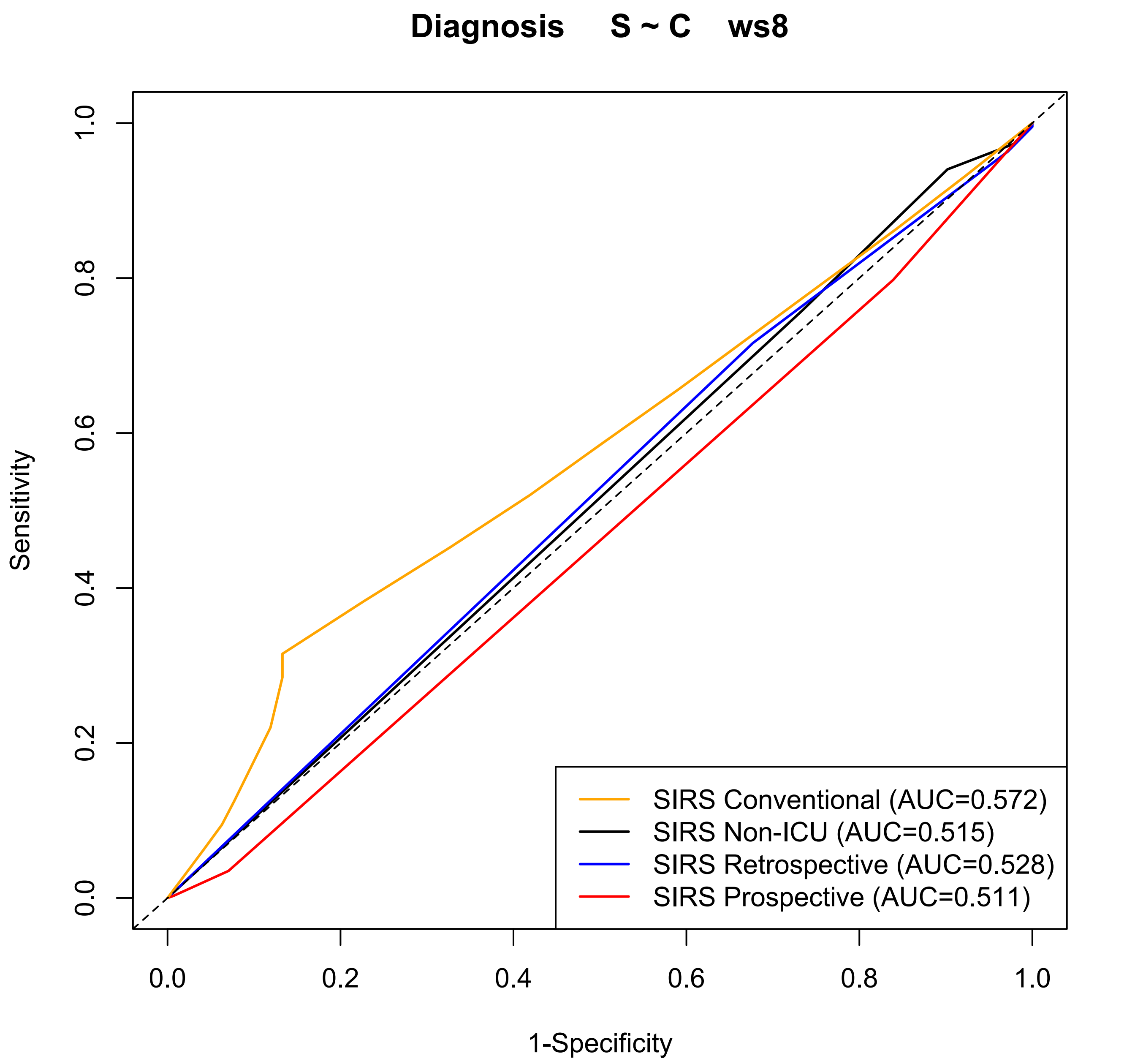

Diagnosis S ~ C ws8
Sensitivity
1-Specificity
0.0
0.2
0.4
0.6
0.8
1.0
SIRS Conventional (AUC=0.572)
SIRS Non-ICU (AUC=0.515)
SIRS Retrospective (AUC=0.528)
SIRS Prospective (AUC=0.511)

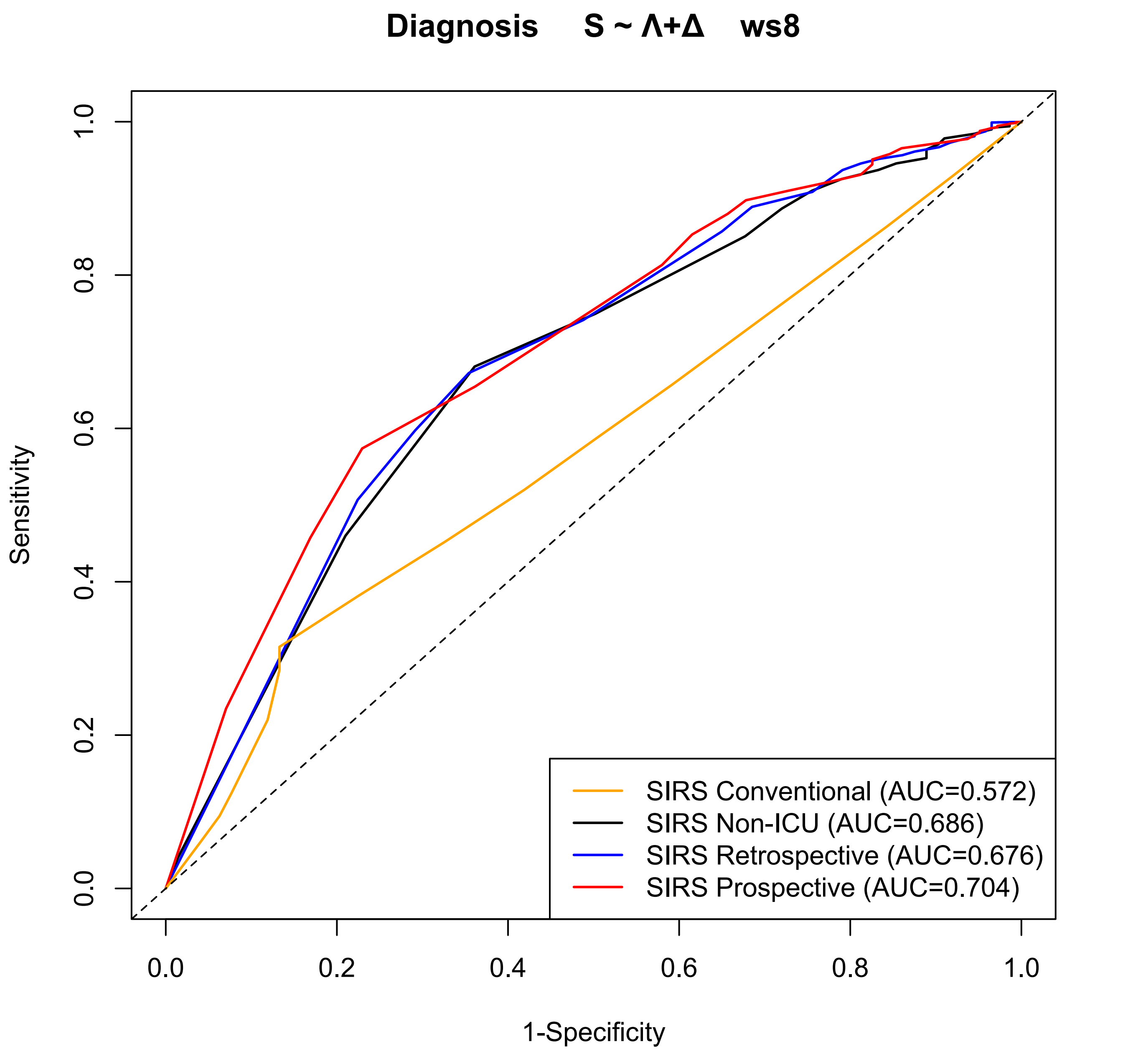

Diagnosis S ~ Λ+Δ ws8
Sensitivity
1-Specificity
0.0
0.2
0.4
0.6
0.8
1.0
SIRS Conventional (AUC=0.572)
SIRS Non-ICU (AUC=0.686)
SIRS Retrospective (AUC=0.676)
SIRS Prospective (AUC=0.704)

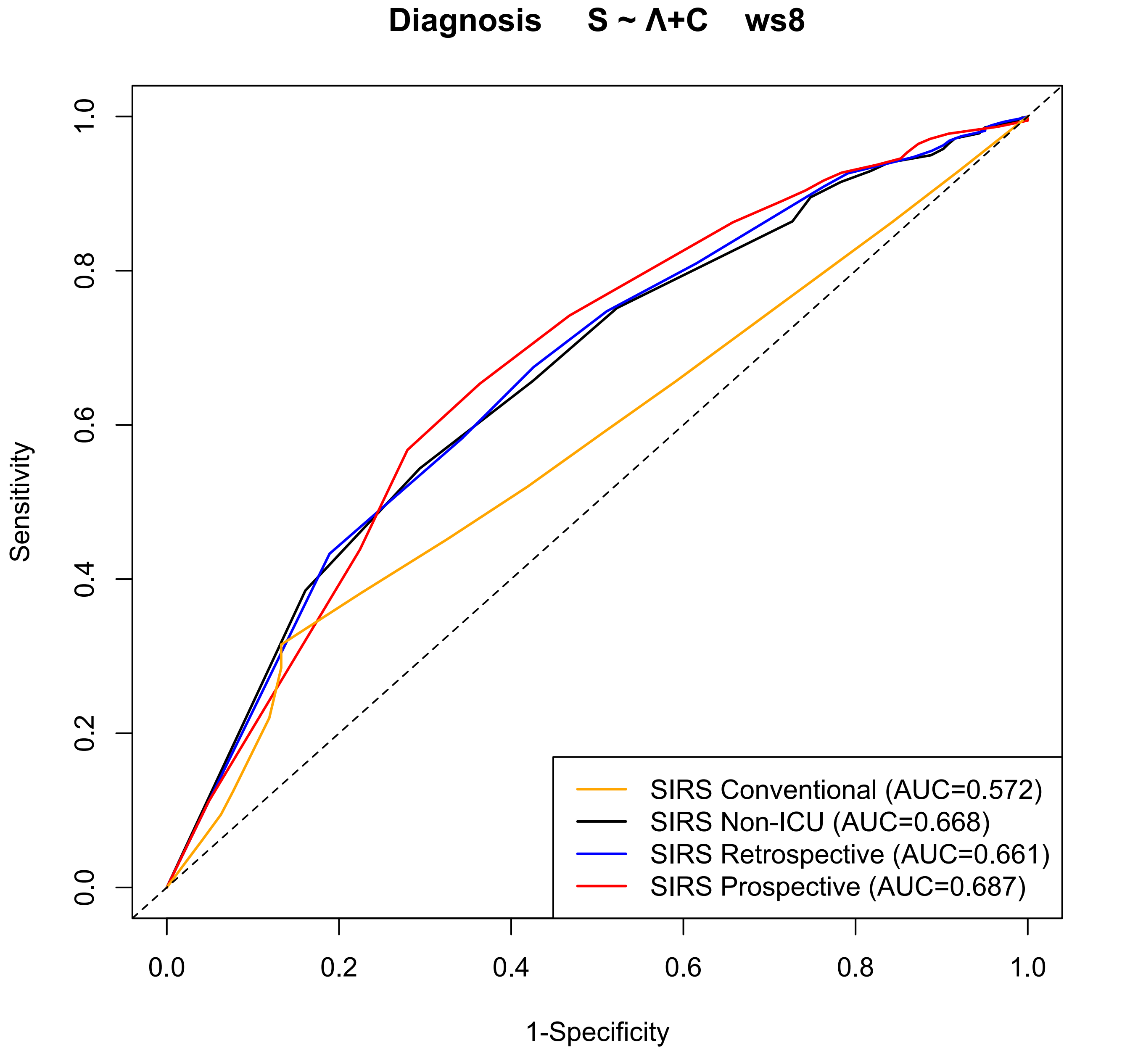

Diagnosis S ~ Λ+C ws8
Sensitivity
1-Specificity
0.0
0.2
0.4
0.6
0.8
1.0
SIRS Conventional (AUC=0.572)
SIRS Non-ICU (AUC=0.668)
SIRS Retrospective (AUC=0.661)
SIRS Prospective (AUC=0.687)

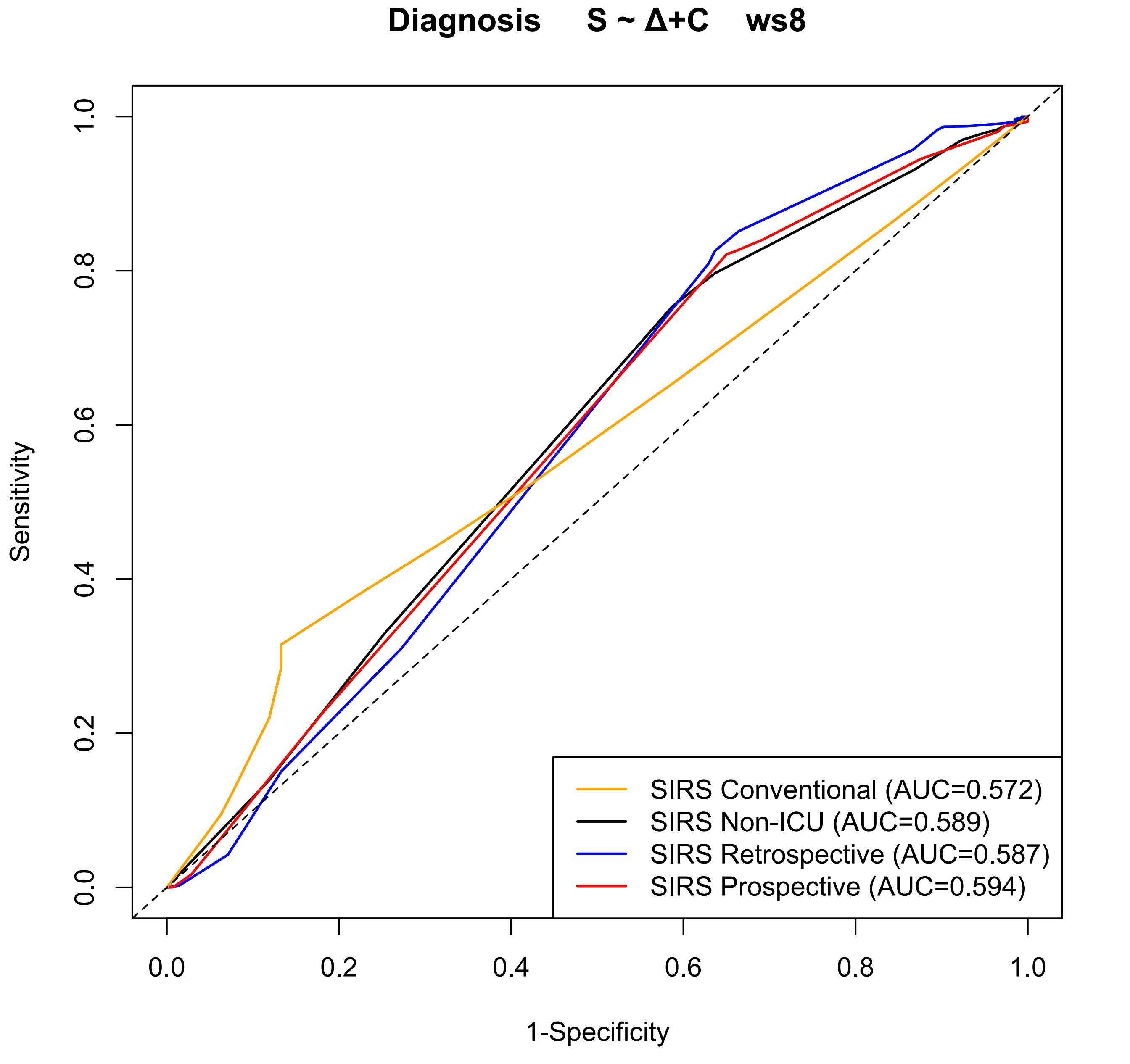
Diagnosis S ~ Δ+C ws8
Sensitivity
1-Specificity
0.0
0.2
0.4
0.6
0.8
1.0
SIRS Conventional (AUC=0.572)
SIRS Non-ICU (AUC=0.589)
SIRS Retrospective (AUC=0.587)
SIRS Prospective (AUC=0.594)

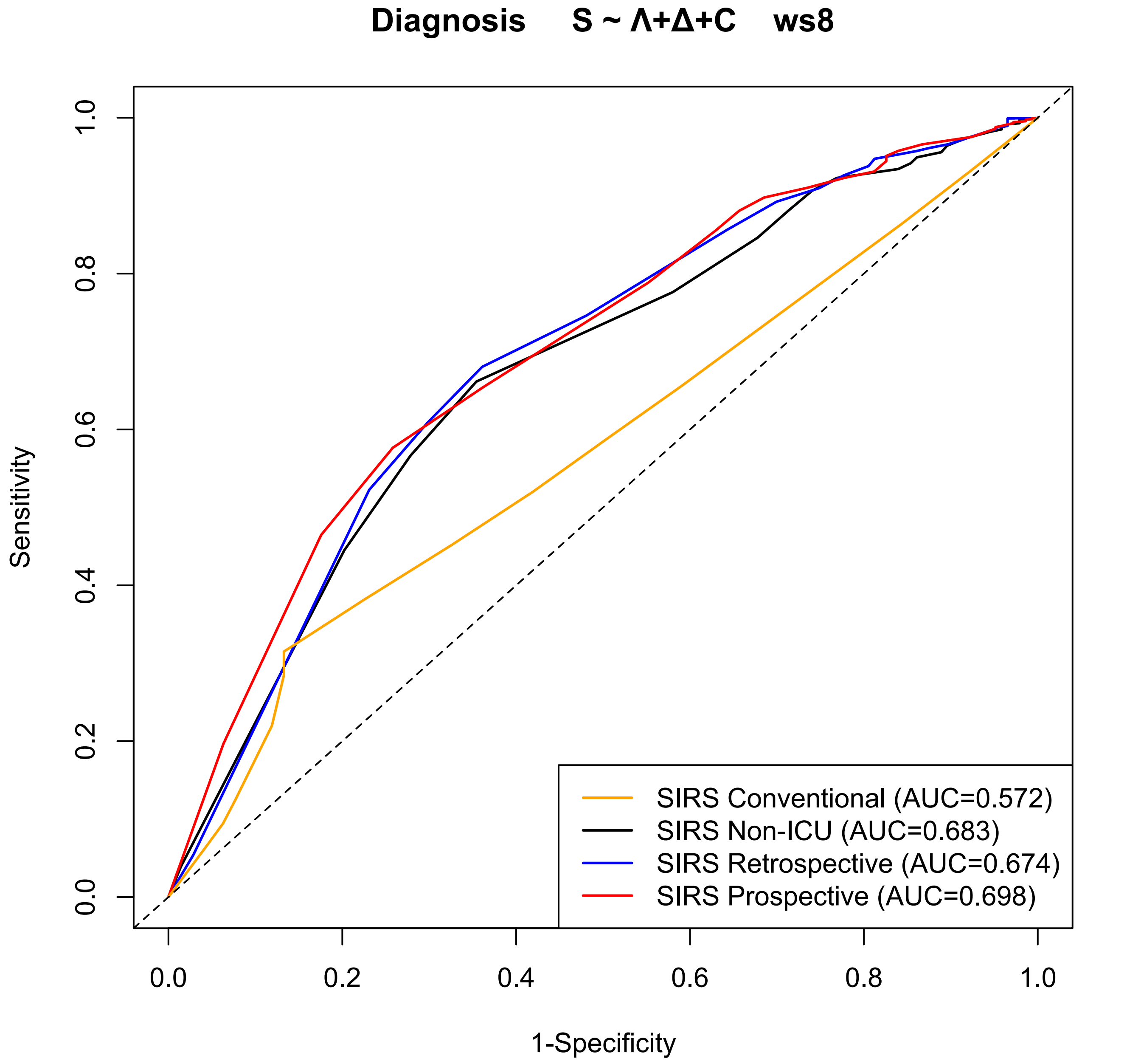
Diagnosis S ~ Λ+Δ+C ws8
Sensitivity
1-Specificity
0.0
0.2
0.4
0.6
0.8
1.0
SIRS Conventional (AUC=0.572)
SIRS Non-ICU (AUC=0.683)
SIRS Retrospective (AUC=0.674)
SIRS Prospective (AUC=0.698)

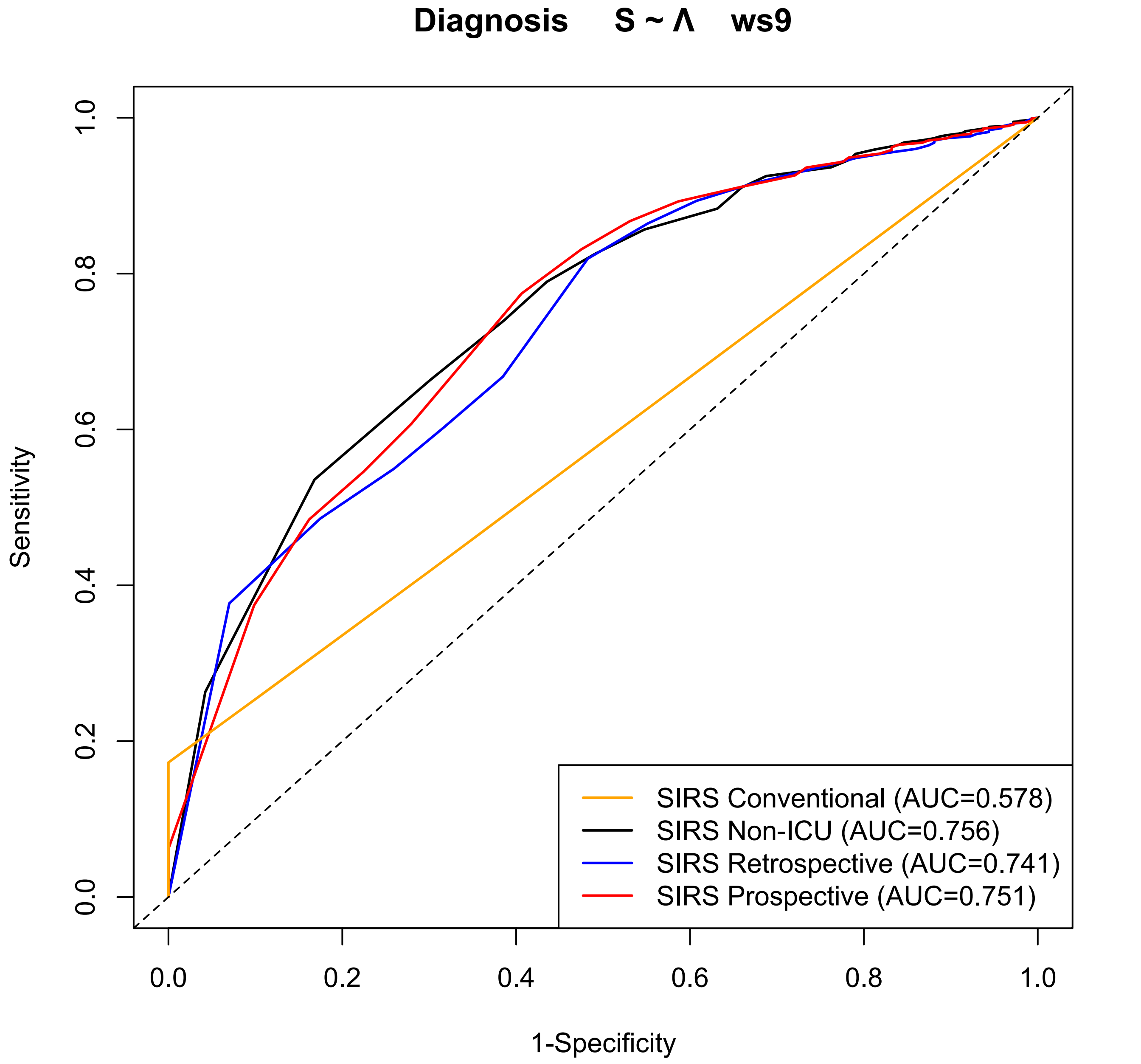
Diagnosis S ~ Λ ws9
Sensitivity
1-Specificity
0.0
0.2
0.4
0.6
0.8
1.0
SIRS Conventional (AUC=0.578)
SIRS Non-ICU (AUC=0.756)
SIRS Retrospective (AUC=0.741)
SIRS Prospective (AUC=0.751)

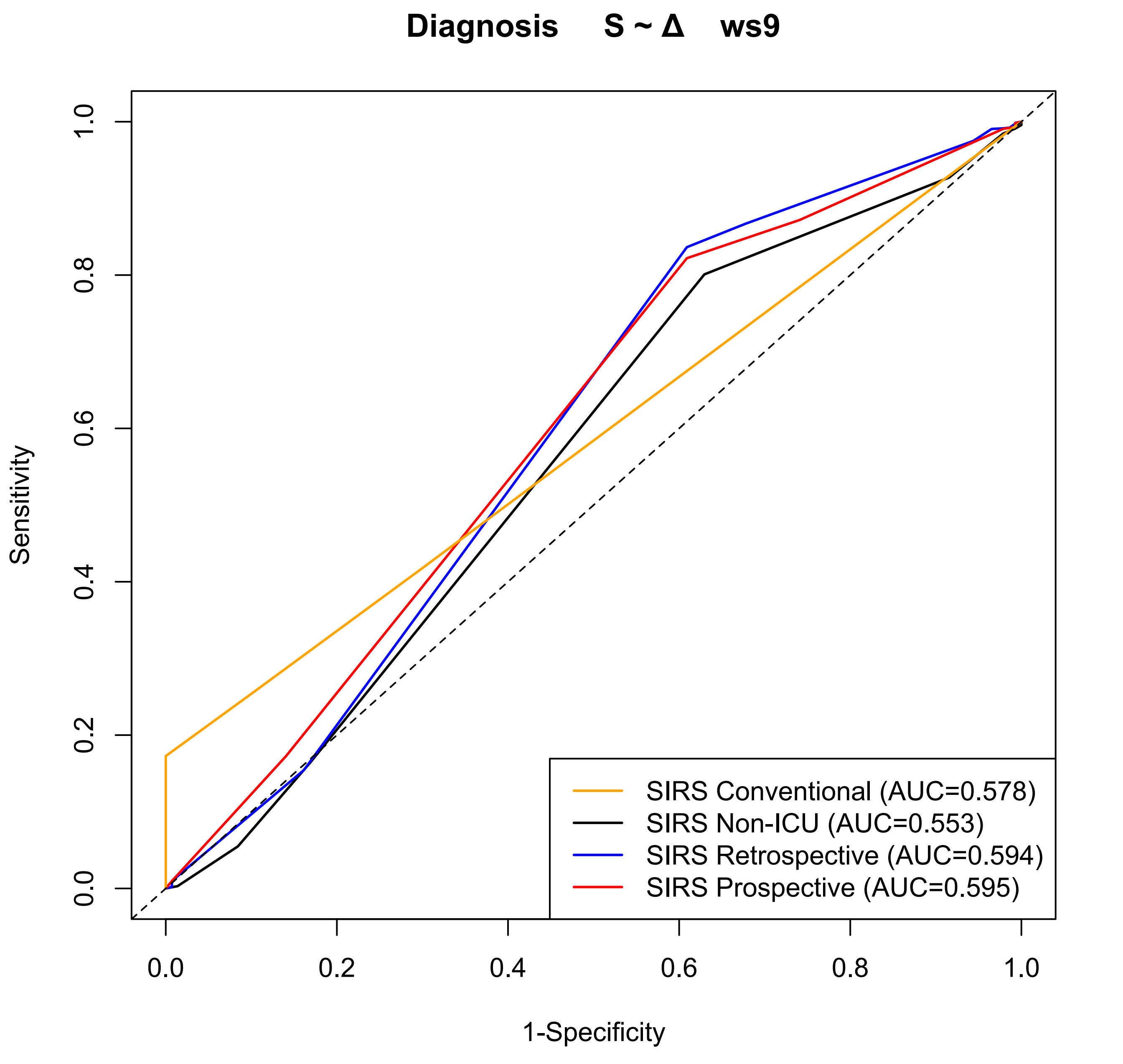
Diagnosis S ~ Δ ws9
Sensitivity
1-Specificity
0.0
0.2
0.4
0.6
0.8
1.0
SIRS Conventional (AUC=0.578)
SIRS Non-ICU (AUC=0.553)
SIRS Retrospective (AUC=0.594)
SIRS Prospective (AUC=0.595)

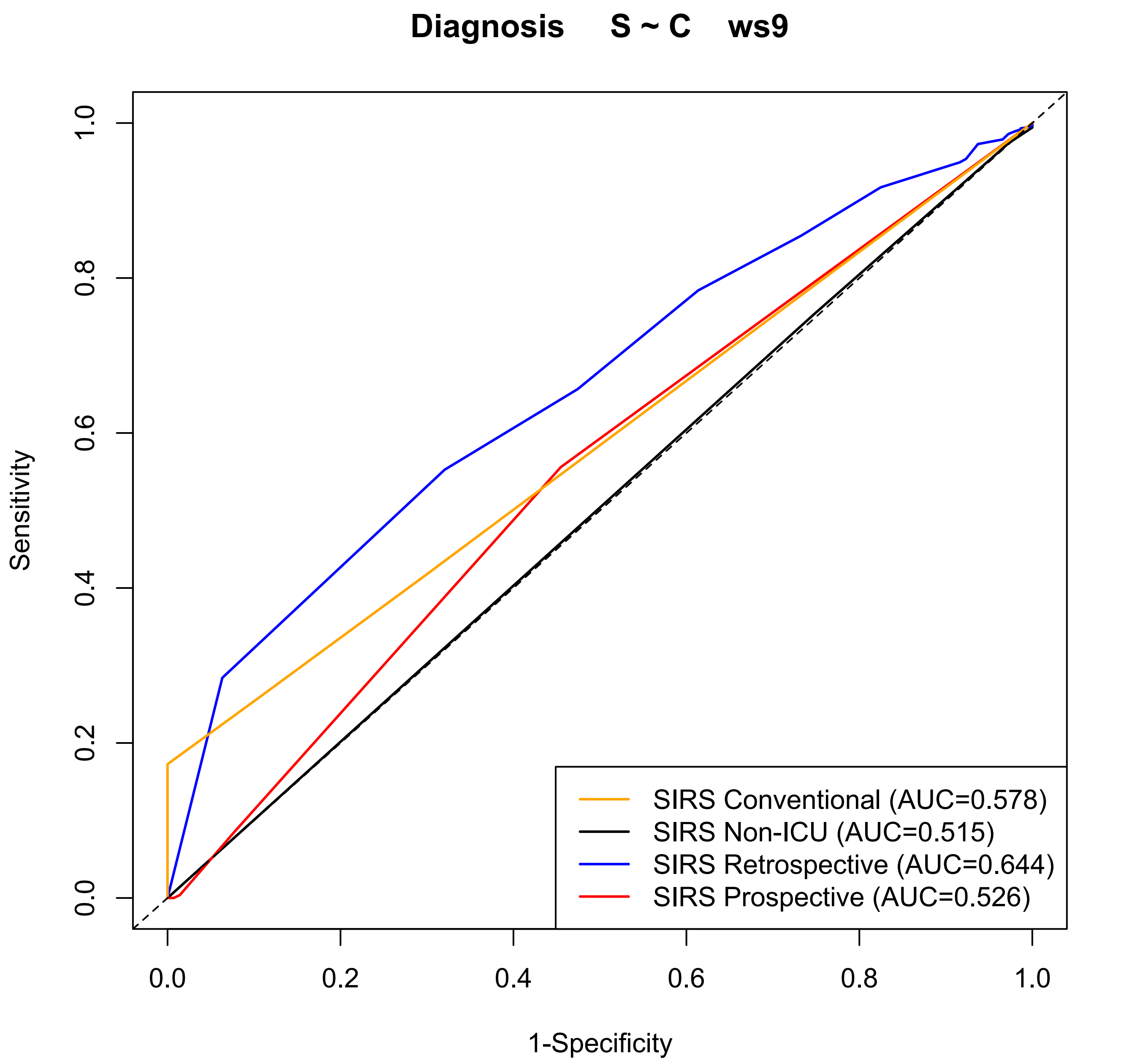

Diagnosis S ~ C ws9
Sensitivity
1-Specificity
0.0
0.2
0.4
0.6
0.8
1.0
SIRS Conventional (AUC=0.578)
SIRS Non-ICU (AUC=0.515)
SIRS Retrospective (AUC=0.644)
SIRS Prospective (AUC=0.526)

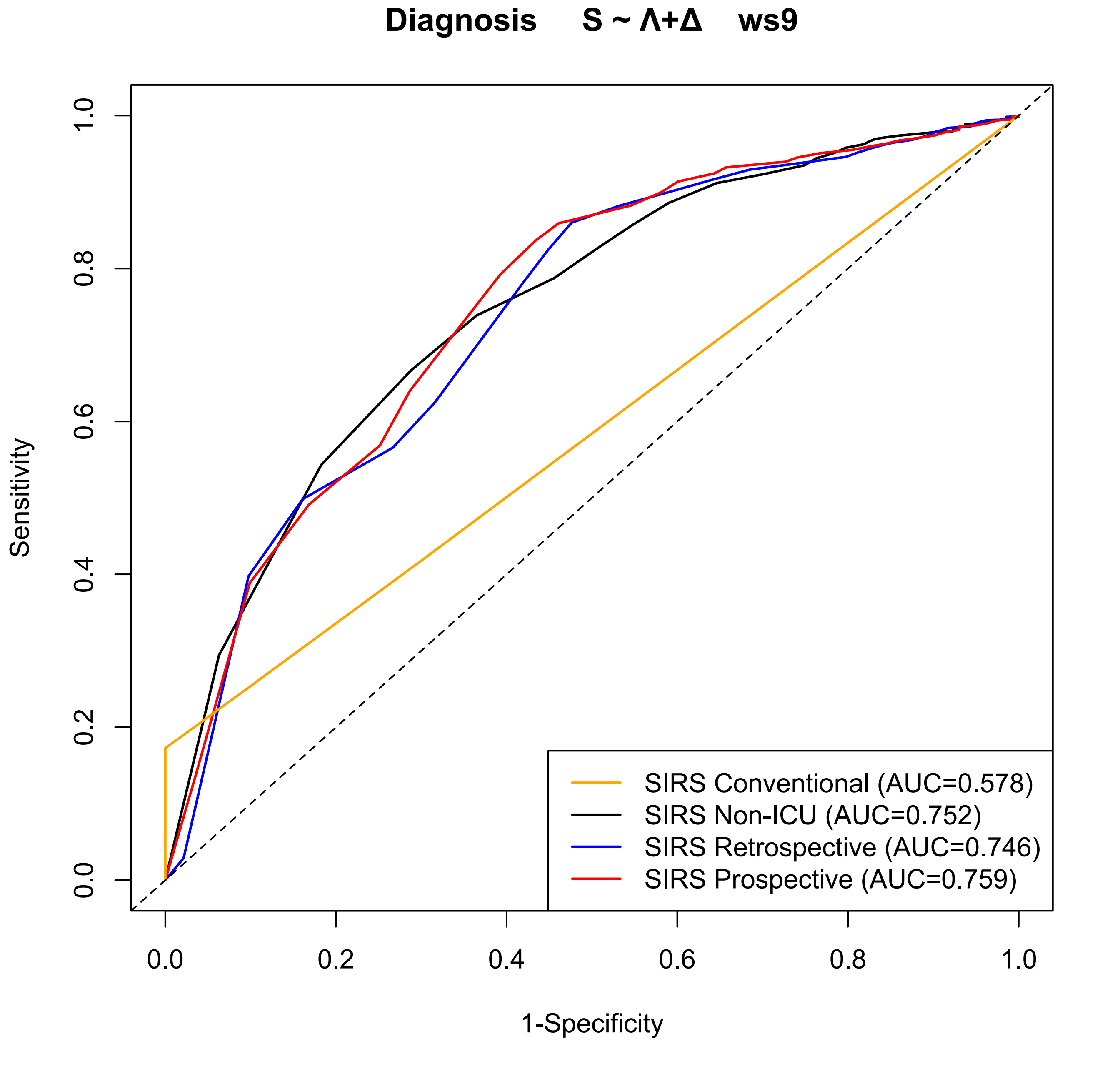
Diagnosis S ~ Λ+Δ ws9
Sensitivity
1-Specificity
0.0
0.2
0.4
0.6
0.8
1.0
SIRS Conventional (AUC=0.578)
SIRS Non-ICU (AUC=0.752)
SIRS Retrospective (AUC=0.746)
SIRS Prospective (AUC=0.759)

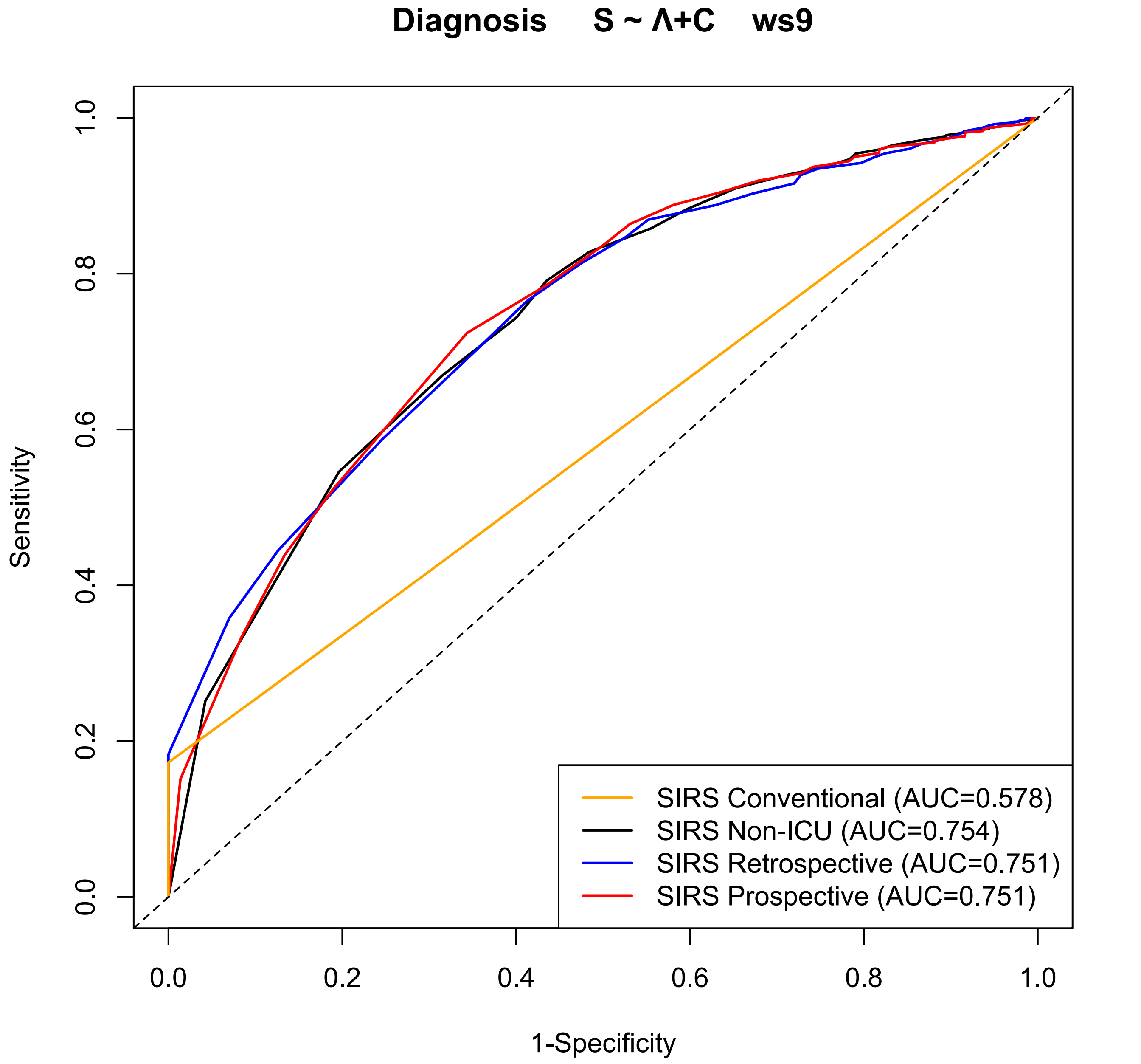
Diagnosis S ~ Λ+C ws9
Sensitivity
1-Specificity
0.0
0.2
0.4
0.6
0.8
1.0
SIRS Conventional (AUC=0.578)
SIRS Non-ICU (AUC=0.754)
SIRS Retrospective (AUC=0.751)
SIRS Prospective (AUC=0.751)

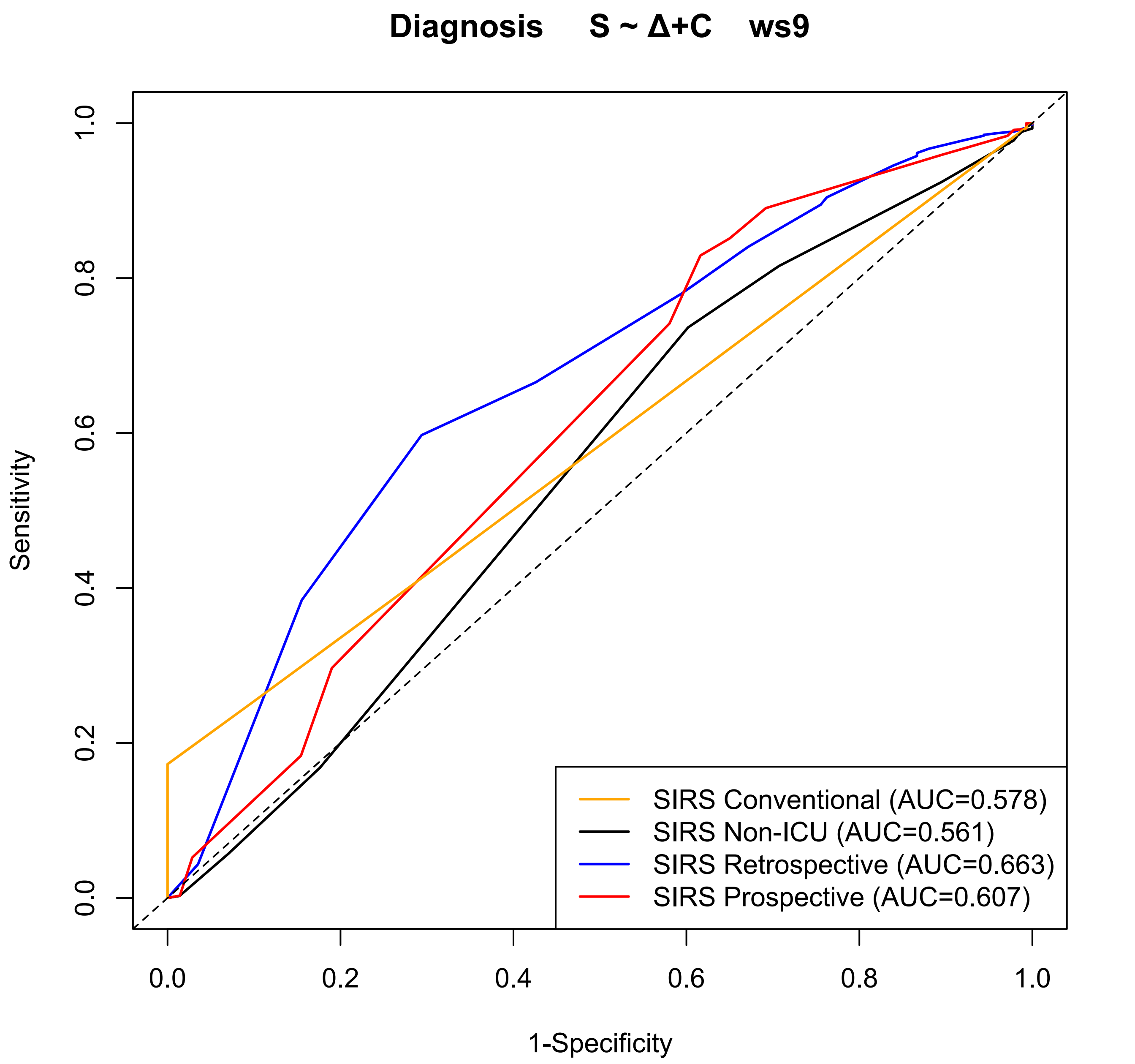
Diagnosis S ~ Δ+C ws9
Sensitivity
1-Specificity
0.0
0.2
0.4
0.6
0.8
1.0
SIRS Conventional (AUC=0.578)
SIRS Non-ICU (AUC=0.561)
SIRS Retrospective (AUC=0.663)
SIRS Prospective (AUC=0.607)

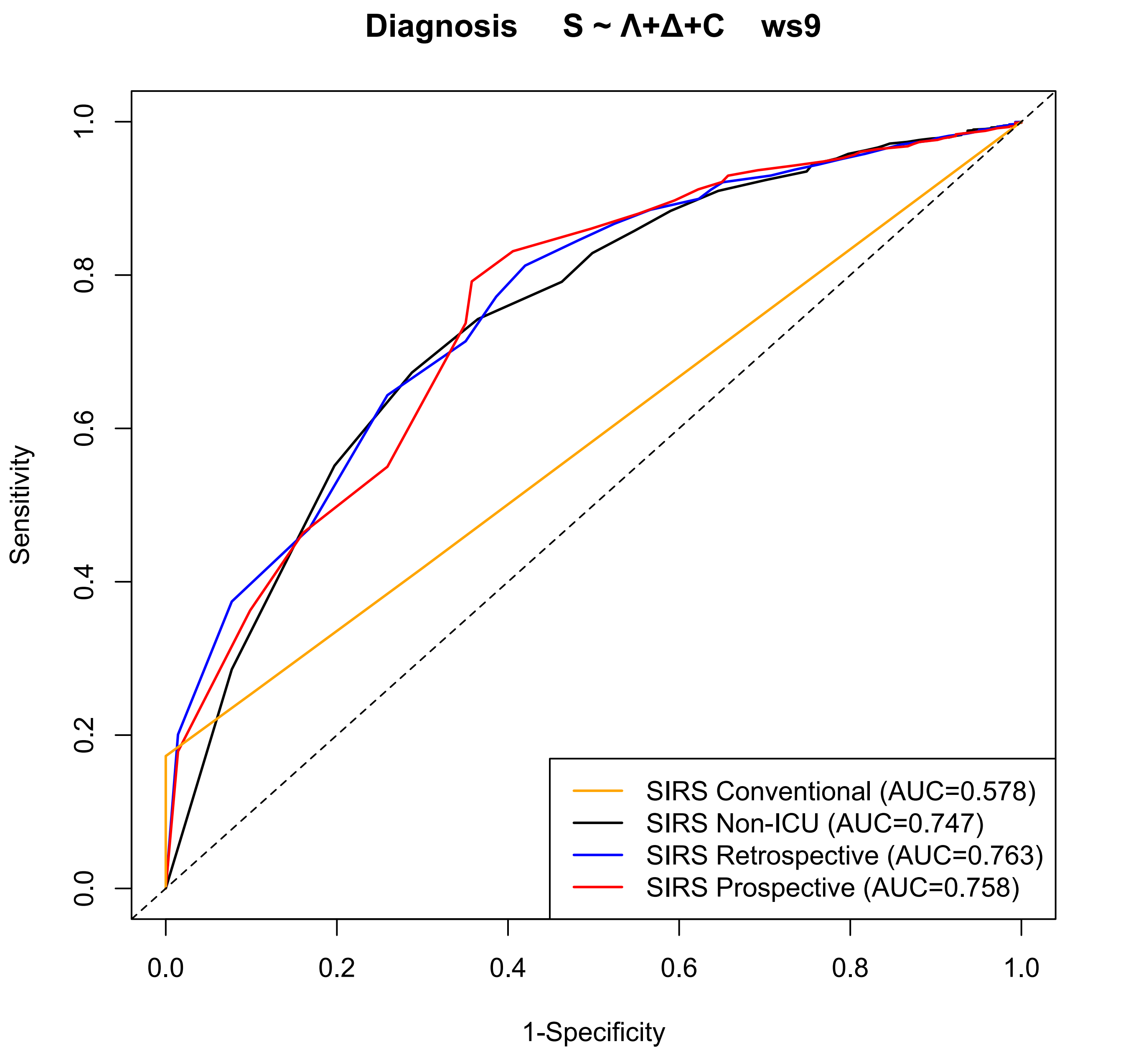
Diagnosis S ~ Λ+Δ+C ws9
Sensitivity
1-Specificity
0.0
0.2
0.4
0.6
0.8
1.0
SIRS Conventional (AUC=0.578)
SIRS Non-ICU (AUC=0.747)
SIRS Retrospective (AUC=0.763)
SIRS Prospective (AUC=0.758)

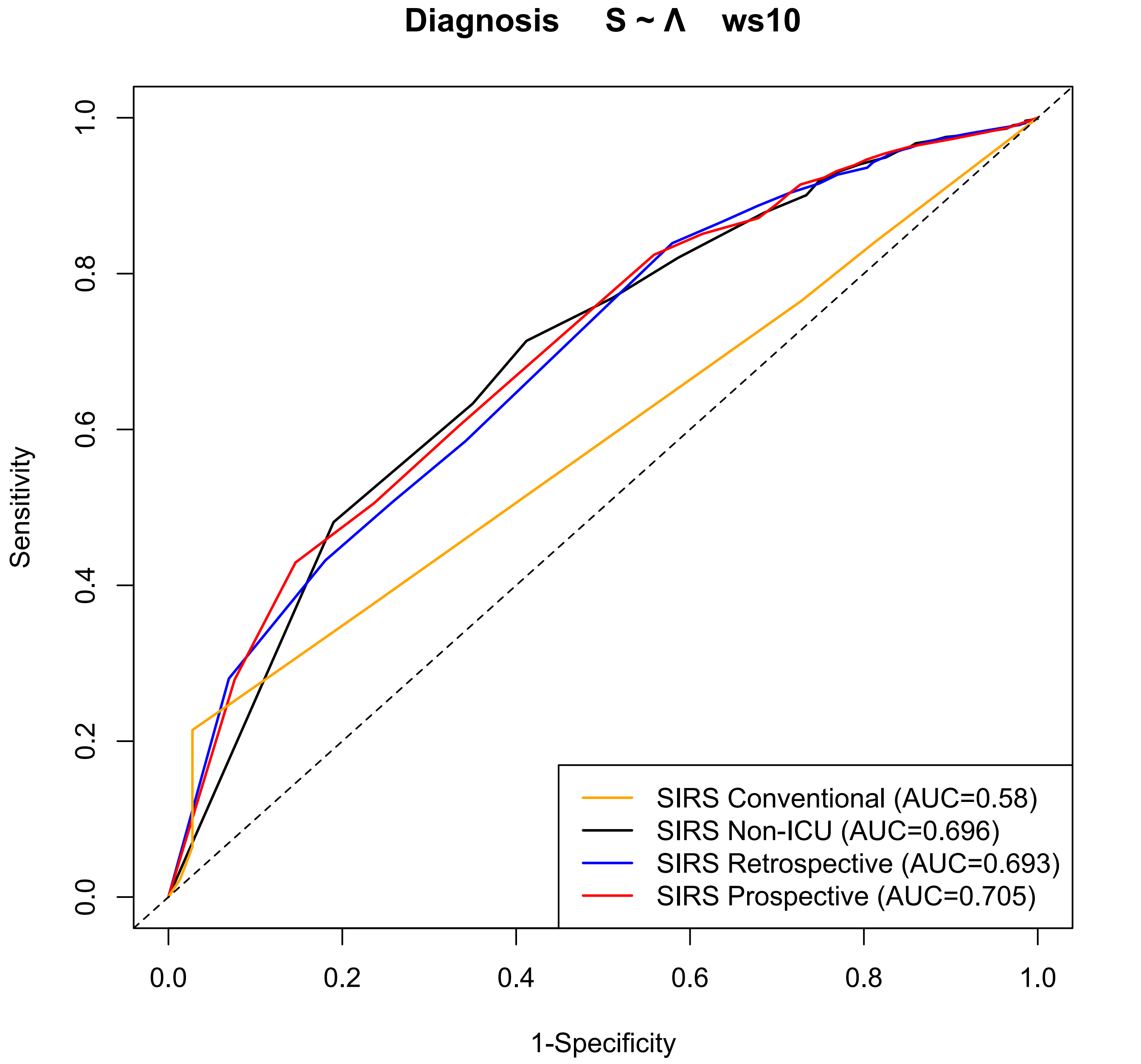

Diagnosis S ~ Λ ws10
Sensitivity
1-Specificity
0.0
0.2
0.4
0.6
0.8
1.0
SIRS Conventional (AUC=0.58)
SIRS Non-ICU (AUC=0.696)
SIRS Retrospective (AUC=0.693)
SIRS Prospective (AUC=0.705)

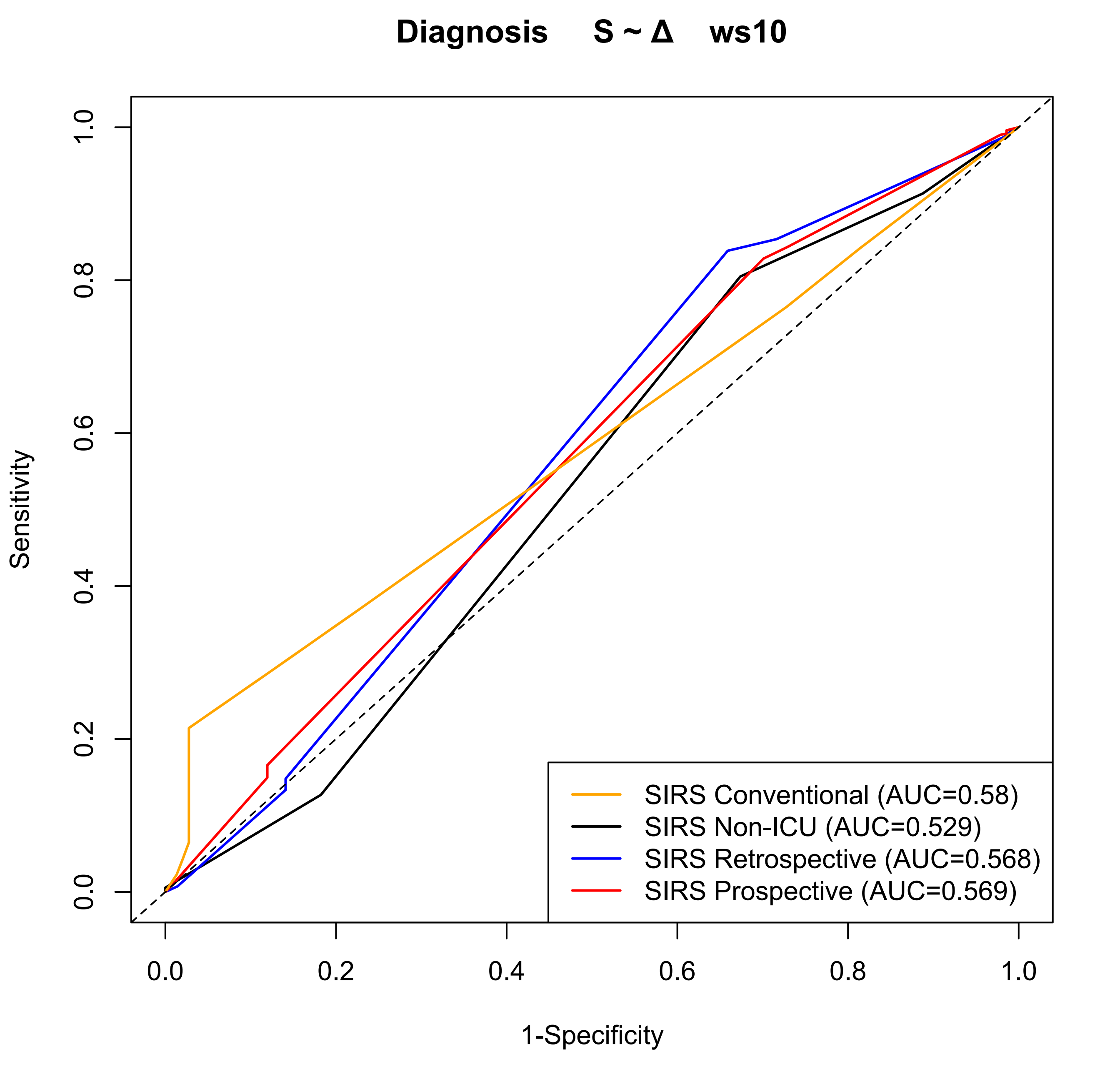

Diagnosis S ~ Δ ws10
Sensitivity
1-Specificity
0.0
0.2
0.4
0.6
0.8
1.0
SIRS Conventional (AUC=0.58)
SIRS Non-ICU (AUC=0.529)
SIRS Retrospective (AUC=0.568)
SIRS Prospective (AUC=0.569)

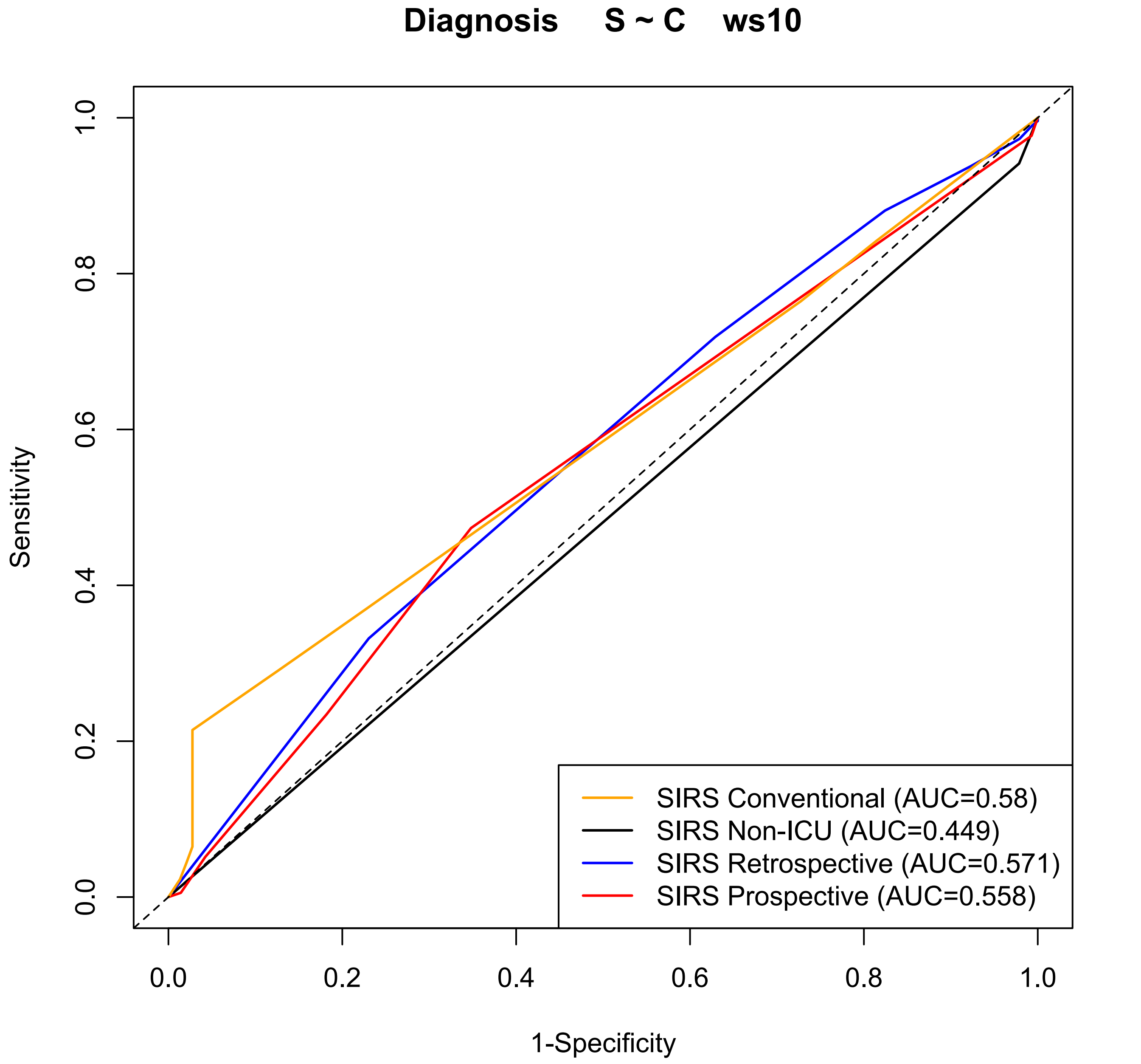

Diagnosis S ~ C ws10
Sensitivity
1-Specificity
0.0
0.2
0.4
0.6
0.8
1.0
0.0
0.2
0.4
0.6
0.8
1.0
SIRS Conventional (AUC=0.58)
SIRS Non-ICU (AUC=0.449)
SIRS Retrospective (AUC=0.571)
SIRS Prospective (AUC=0.558)

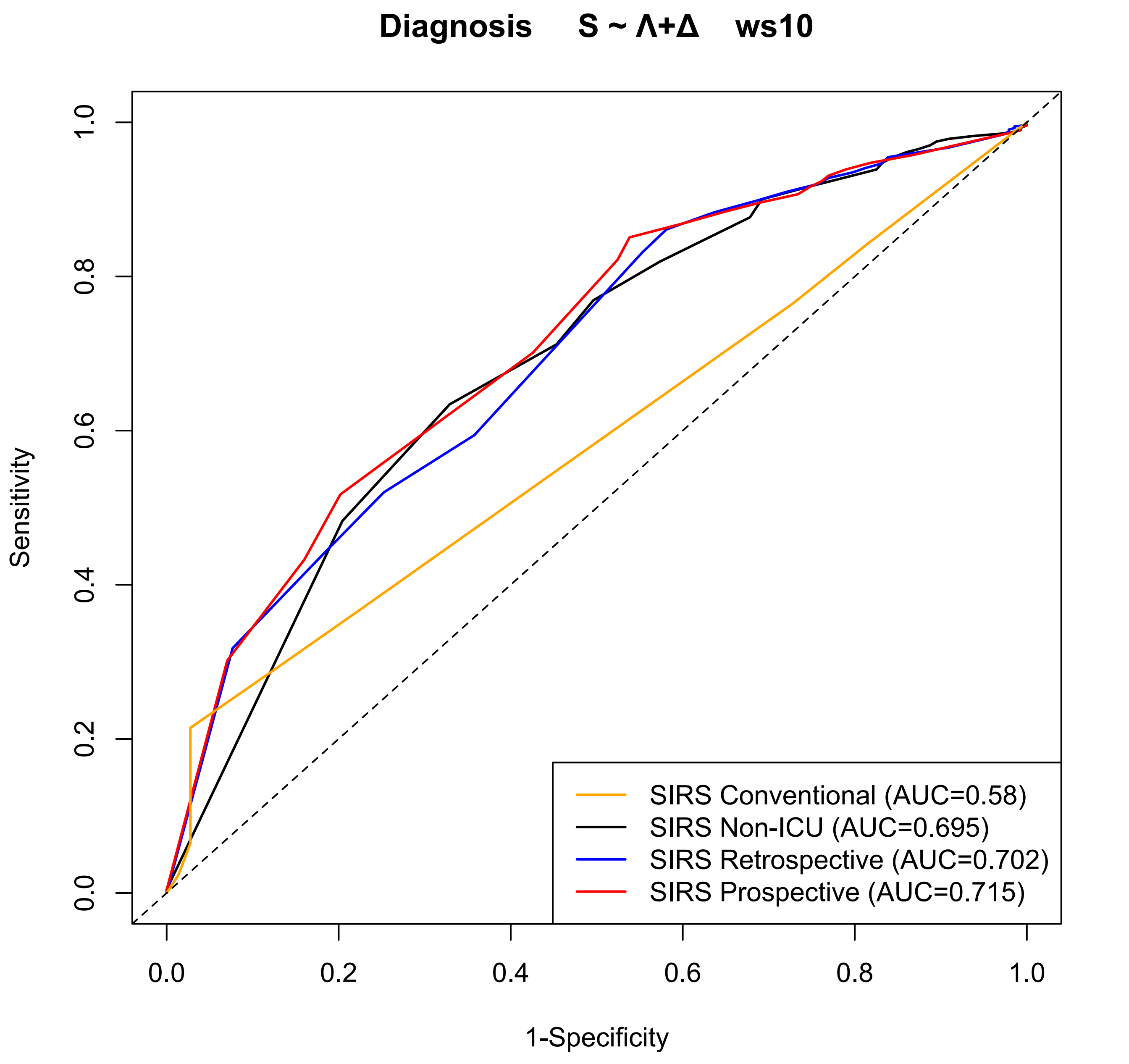
Diagnosis S ~ Λ+Δ ws10
Sensitivity
1-Specificity
0.0
0.2
0.4
0.6
0.8
1.0
SIRS Conventional (AUC=0.58)
SIRS Non-ICU (AUC=0.695)
SIRS Retrospective (AUC=0.702)
SIRS Prospective (AUC=0.715)

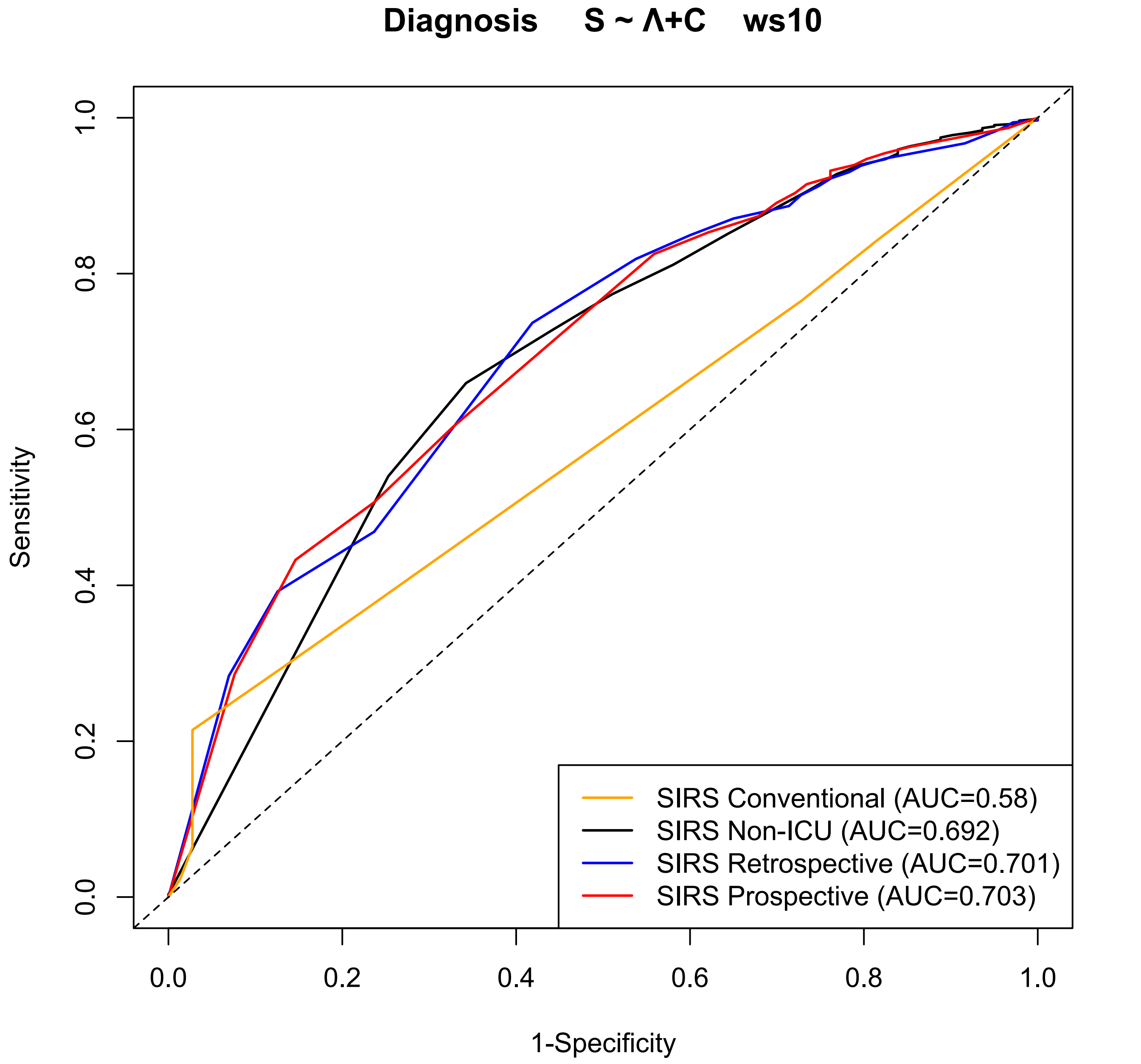

Diagnosis S ~ Λ+C ws10
Sensitivity
1-Specificity
0.0
0.2
0.4
0.6
0.8
1.0
SIRS Conventional (AUC=0.58)
SIRS Non-ICU (AUC=0.692)
SIRS Retrospective (AUC=0.701)
SIRS Prospective (AUC=0.703)

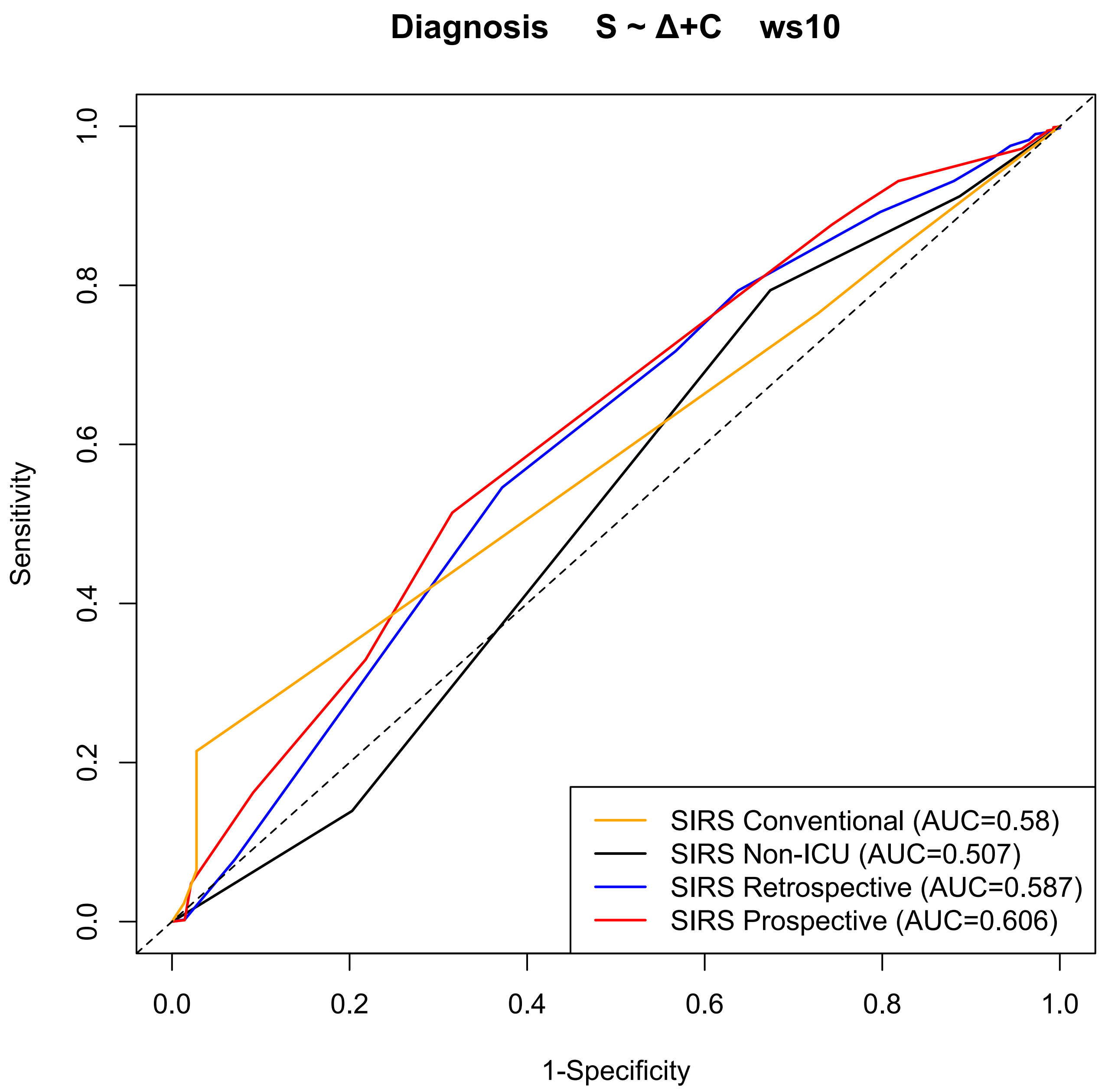
Diagnosis S ~ Δ+C ws10
Sensitivity
1-Specificity
0.0
0.2
0.4
0.6
0.8
1.0
SIRS Conventional (AUC=0.58)
SIRS Non-ICU (AUC=0.507)
SIRS Retrospective (AUC=0.587)
SIRS Prospective (AUC=0.606)

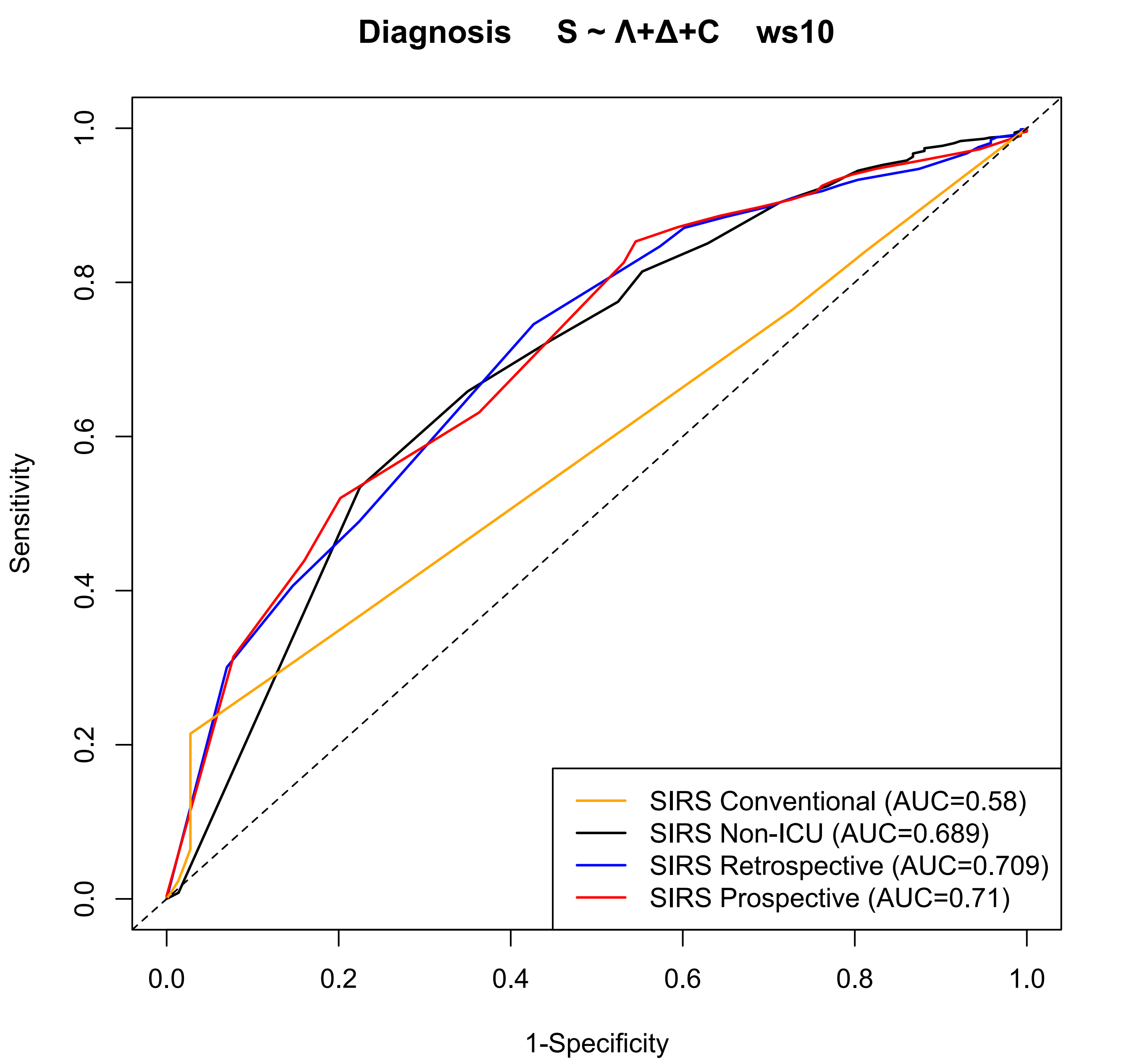

Diagnosis S ~ Λ+Δ+C ws10
Sensitivity
1-Specificity
0.0
0.2
0.4
0.6
0.8
1.0
SIRS Conventional (AUC=0.58)
SIRS Non-ICU (AUC=0.689)
SIRS Retrospective (AUC=0.709)
SIRS Prospective (AUC=0.71)

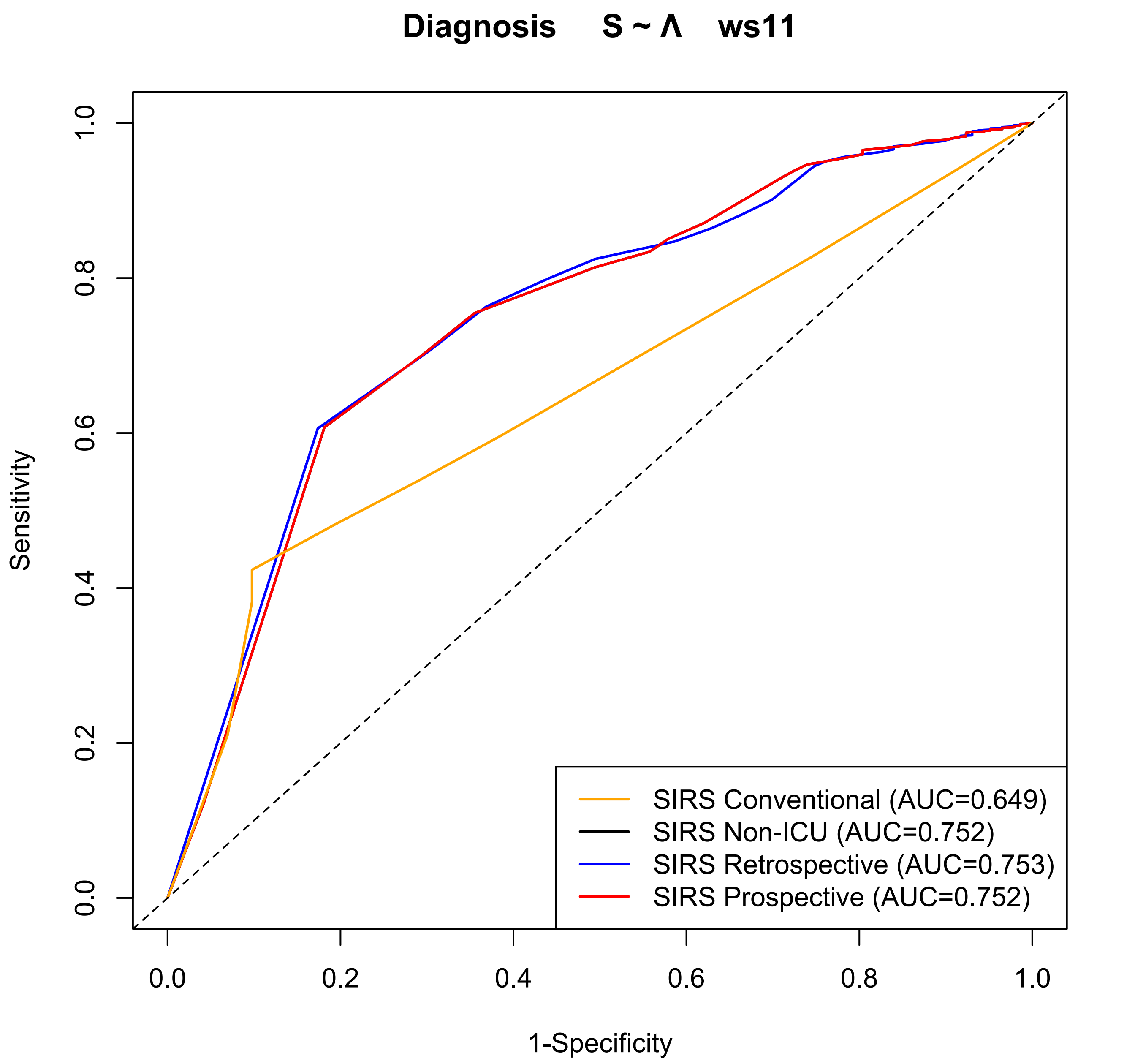
Diagnosis S ~ Λ ws11
Sensitivity
1-Specificity
0.0
0.2
0.4
0.6
0.8
1.0
SIRS Conventional (AUC=0.649)
SIRS Non-ICU (AUC=0.752)
SIRS Retrospective (AUC=0.753)
SIRS Prospective (AUC=0.752)

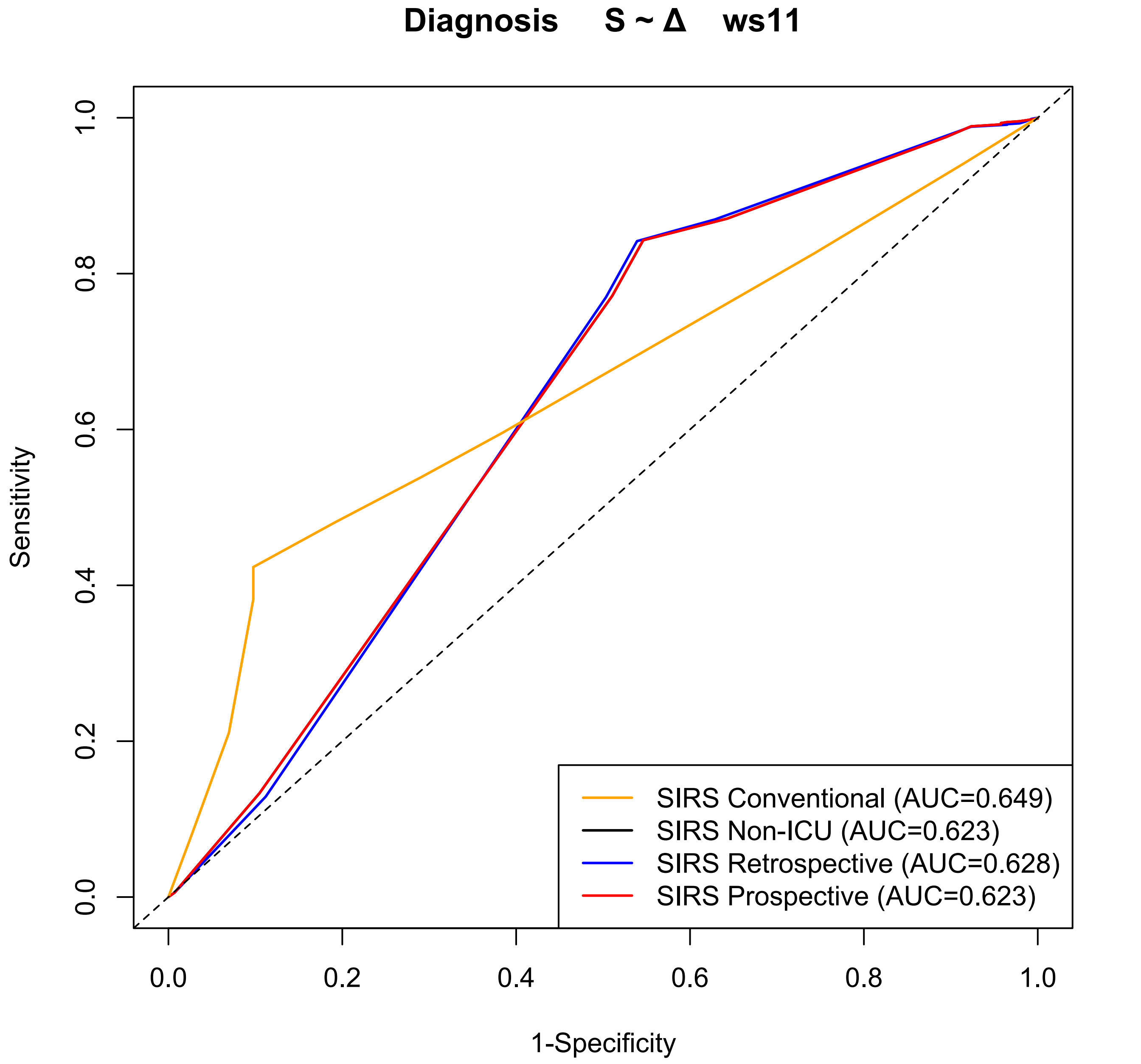

Diagnosis S ~ Δ ws11
Sensitivity
1-Specificity
0.0
0.2
0.4
0.6
0.8
1.0
SIRS Conventional (AUC=0.649)
SIRS Non-ICU (AUC=0.623)
SIRS Retrospective (AUC=0.628)
SIRS Prospective (AUC=0.623)

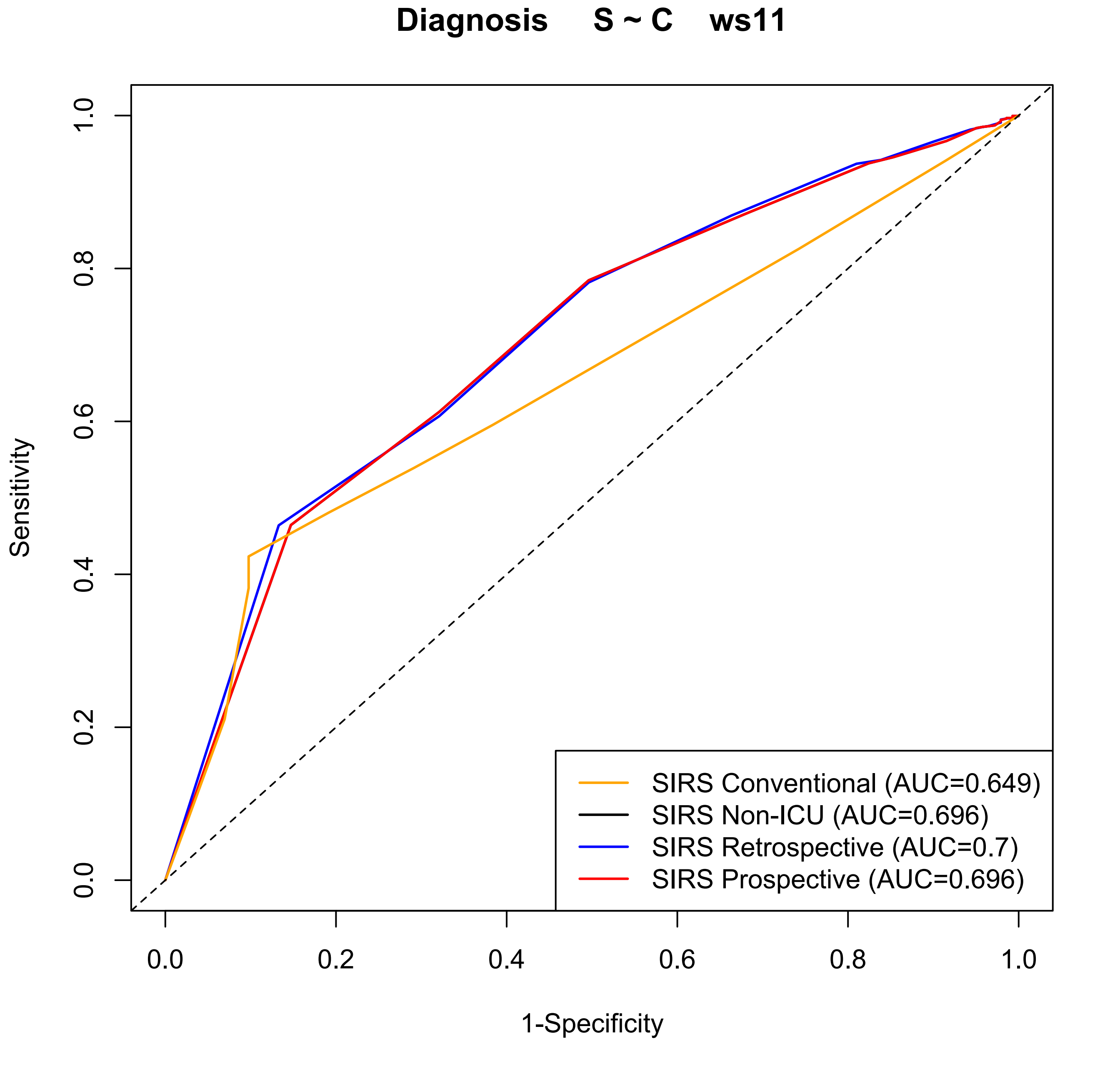
Diagnosis S ~ C ws11
Sensitivity
1-Specificity
0.0
0.2
0.4
0.6
0.8
1.0
SIRS Conventional (AUC=0.649)
SIRS Non-ICU (AUC=0.696)
SIRS Retrospective (AUC=0.7)
SIRS Prospective (AUC=0.696)

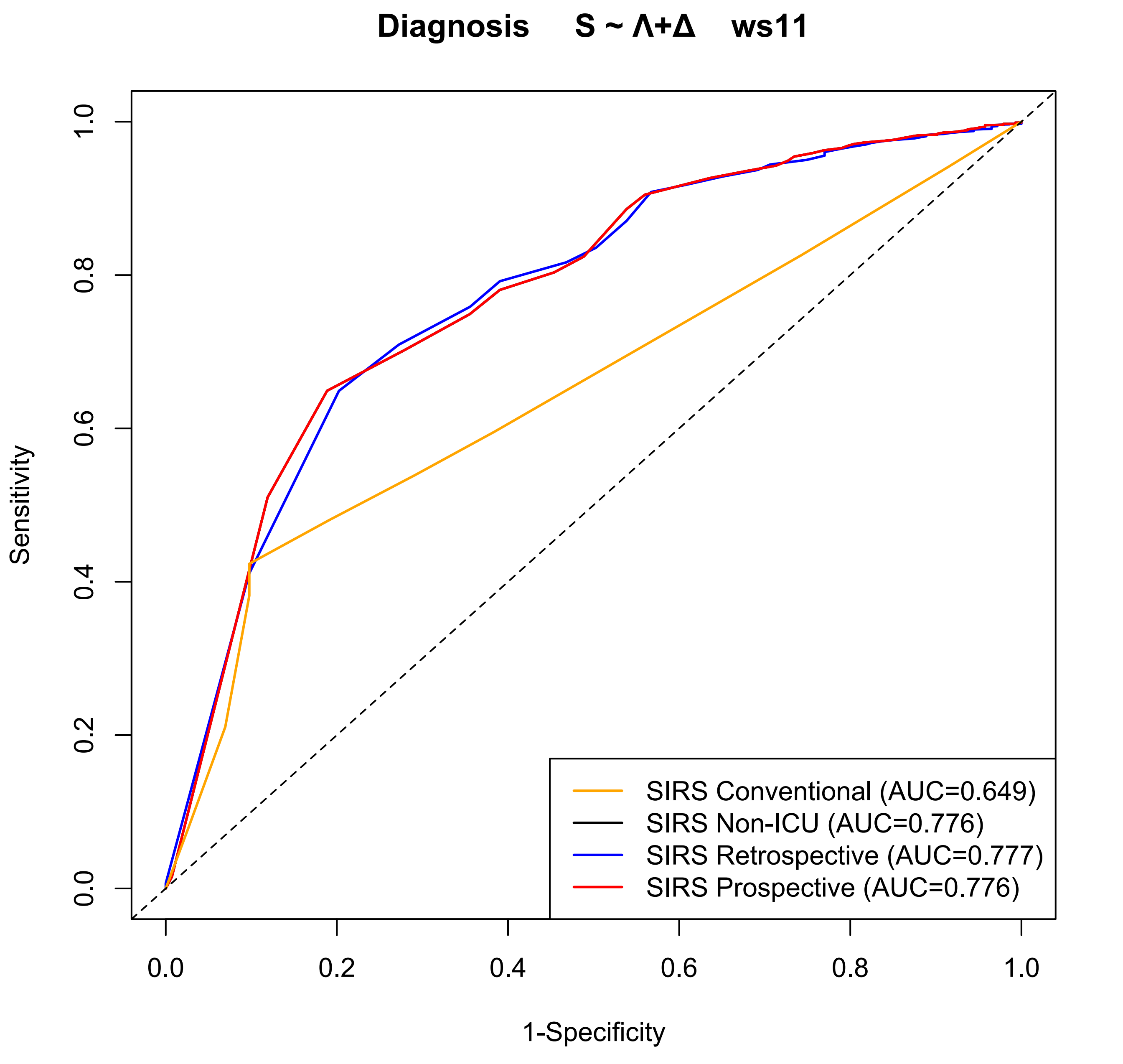

Diagnosis S ~ Λ+Δ ws11
Sensitivity
1-Specificity
0.0
0.2
0.4
0.6
0.8
1.0
SIRS Conventional (AUC=0.649)
SIRS Non-ICU (AUC=0.776)
SIRS Retrospective (AUC=0.777)
SIRS Prospective (AUC=0.776)

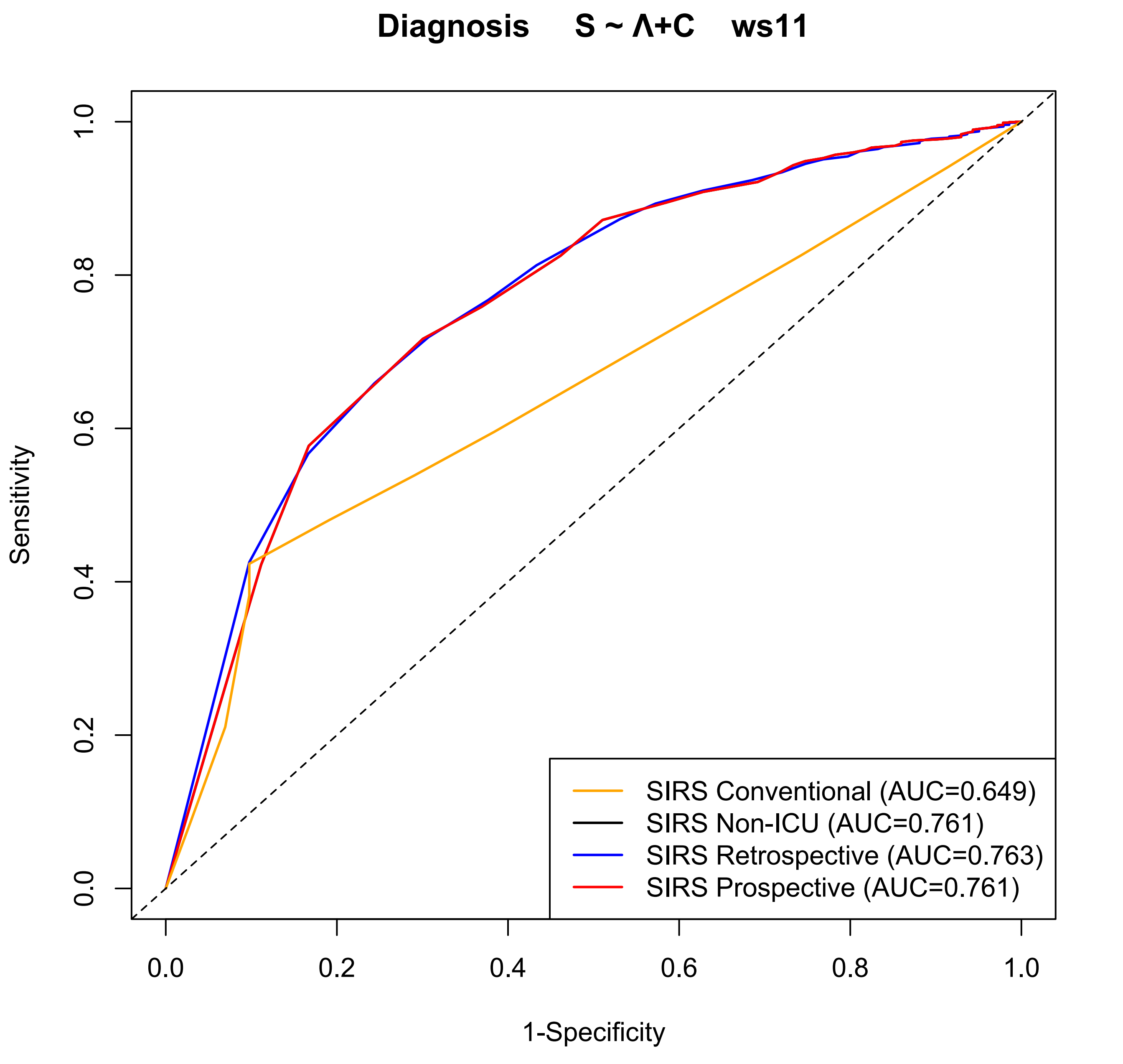
Diagnosis S ~ Λ+C ws11
Sensitivity
1-Specificity
0.0
0.2
0.4
0.6
0.8
1.0
SIRS Conventional (AUC=0.649)
SIRS Non-ICU (AUC=0.761)
SIRS Retrospective (AUC=0.763)
SIRS Prospective (AUC=0.761)

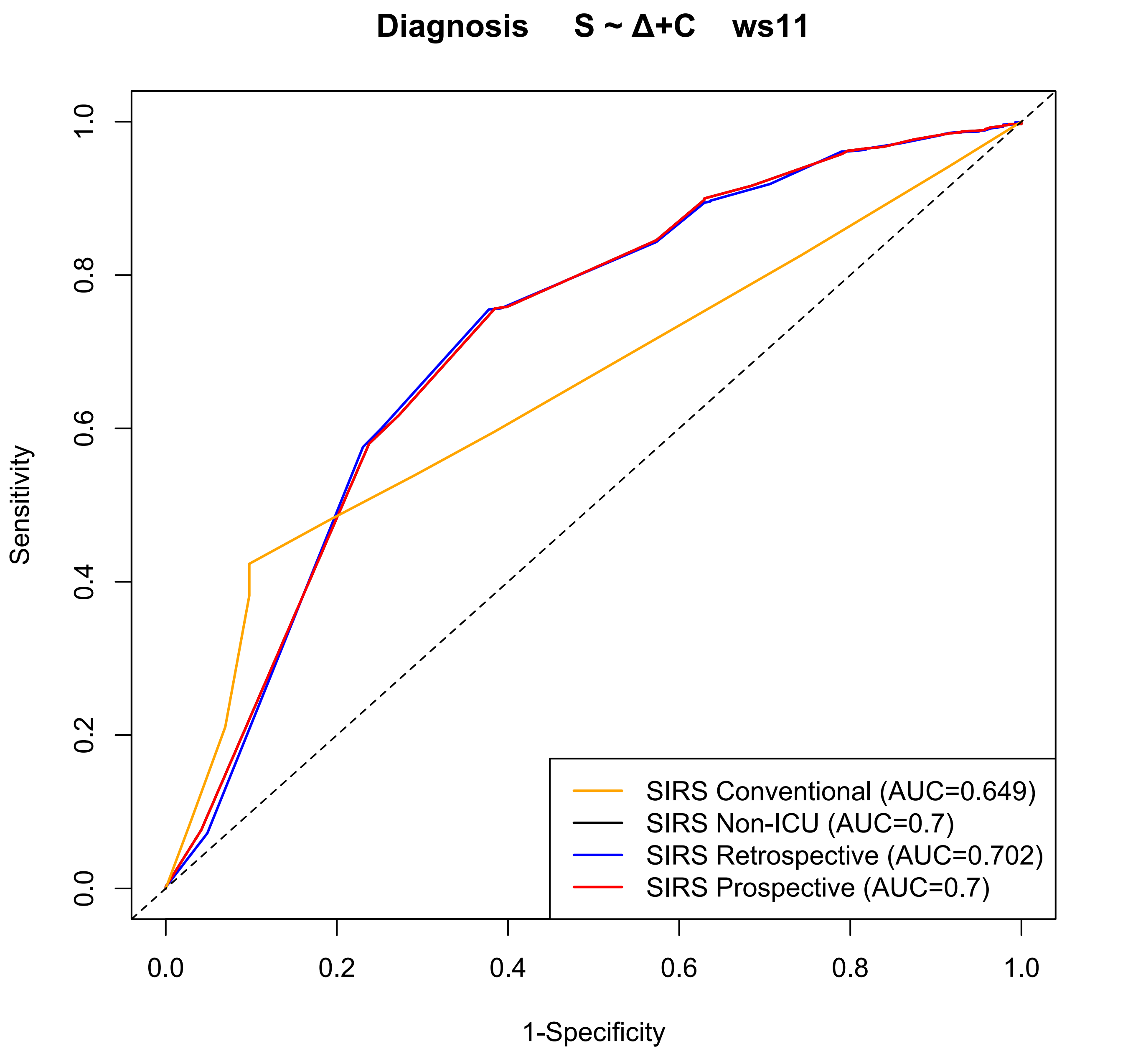

Diagnosis S ~ Δ+C ws11
Sensitivity
1-Specificity
0.0
0.2
0.4
0.6
0.8
1.0
SIRS Conventional (AUC=0.649)
SIRS Non-ICU (AUC=0.7)
SIRS Retrospective (AUC=0.702)
SIRS Prospective (AUC=0.7)

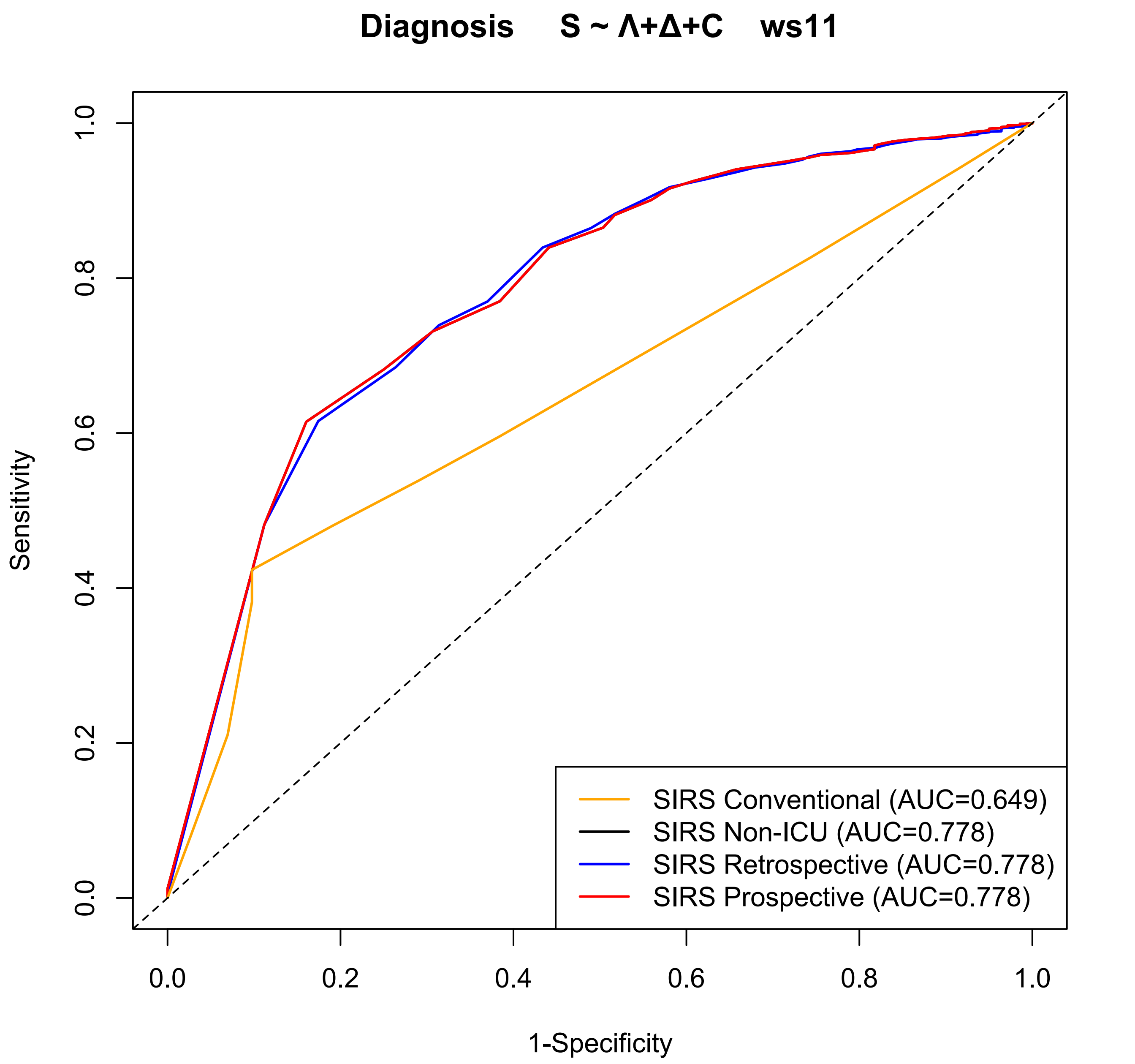

Diagnosis S ~ Λ+Δ+C ws11
Sensitivity
1-Specificity
0.0
0.2
0.4
0.6
0.8
1.0
SIRS Conventional (AUC=0.649)
SIRS Non-ICU (AUC=0.778)
SIRS Retrospective (AUC=0.778)
SIRS Prospective (AUC=0.778)

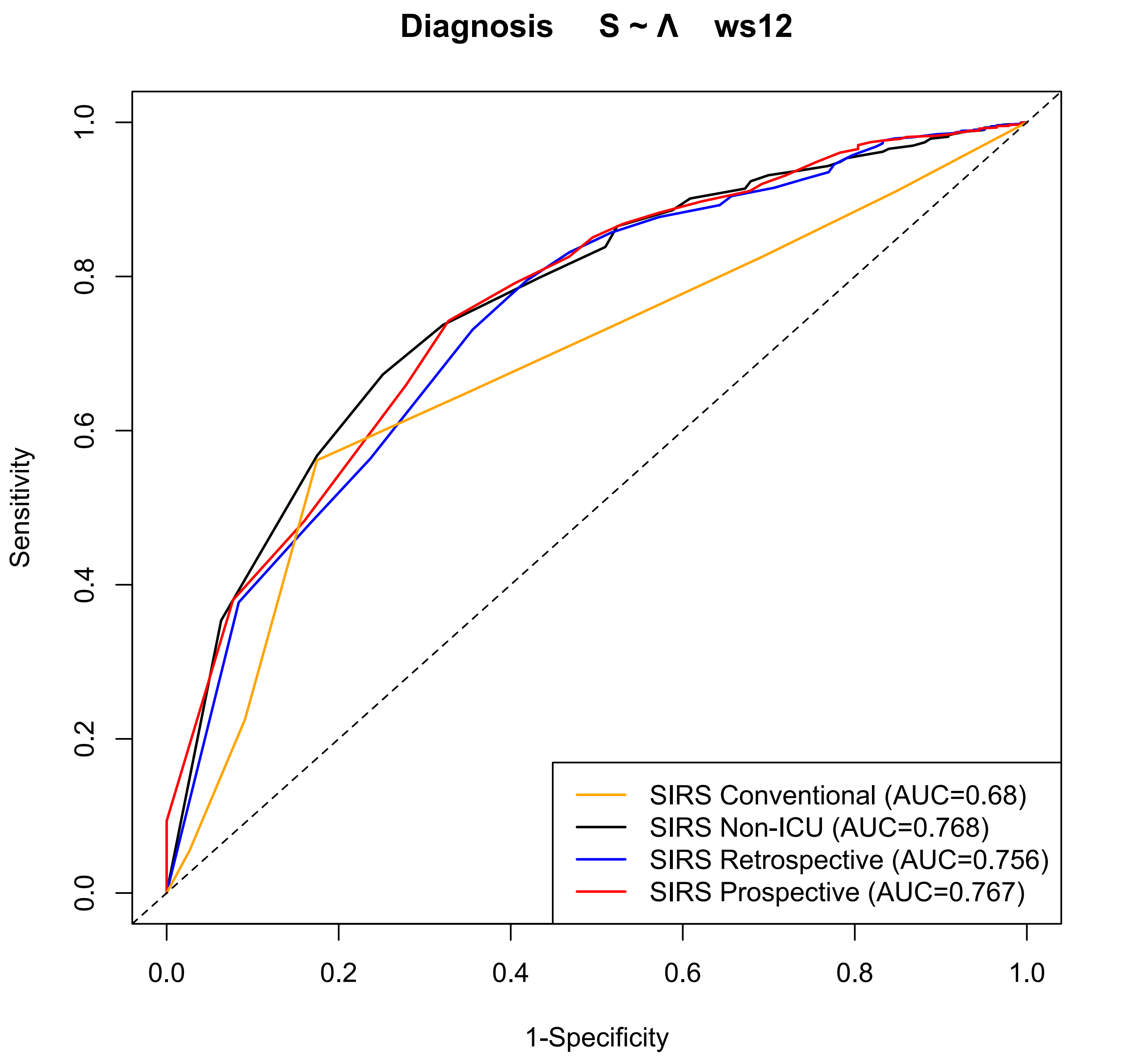

Diagnosis S ~ Λ ws12
Sensitivity
1-Specificity
0.0
0.2
0.4
0.6
0.8
1.0
0.0
0.2
0.4
0.6
0.8
1.0
SIRS Conventional (AUC=0.68)
SIRS Non-ICU (AUC=0.768)
SIRS Retrospective (AUC=0.756)
SIRS Prospective (AUC=0.767)

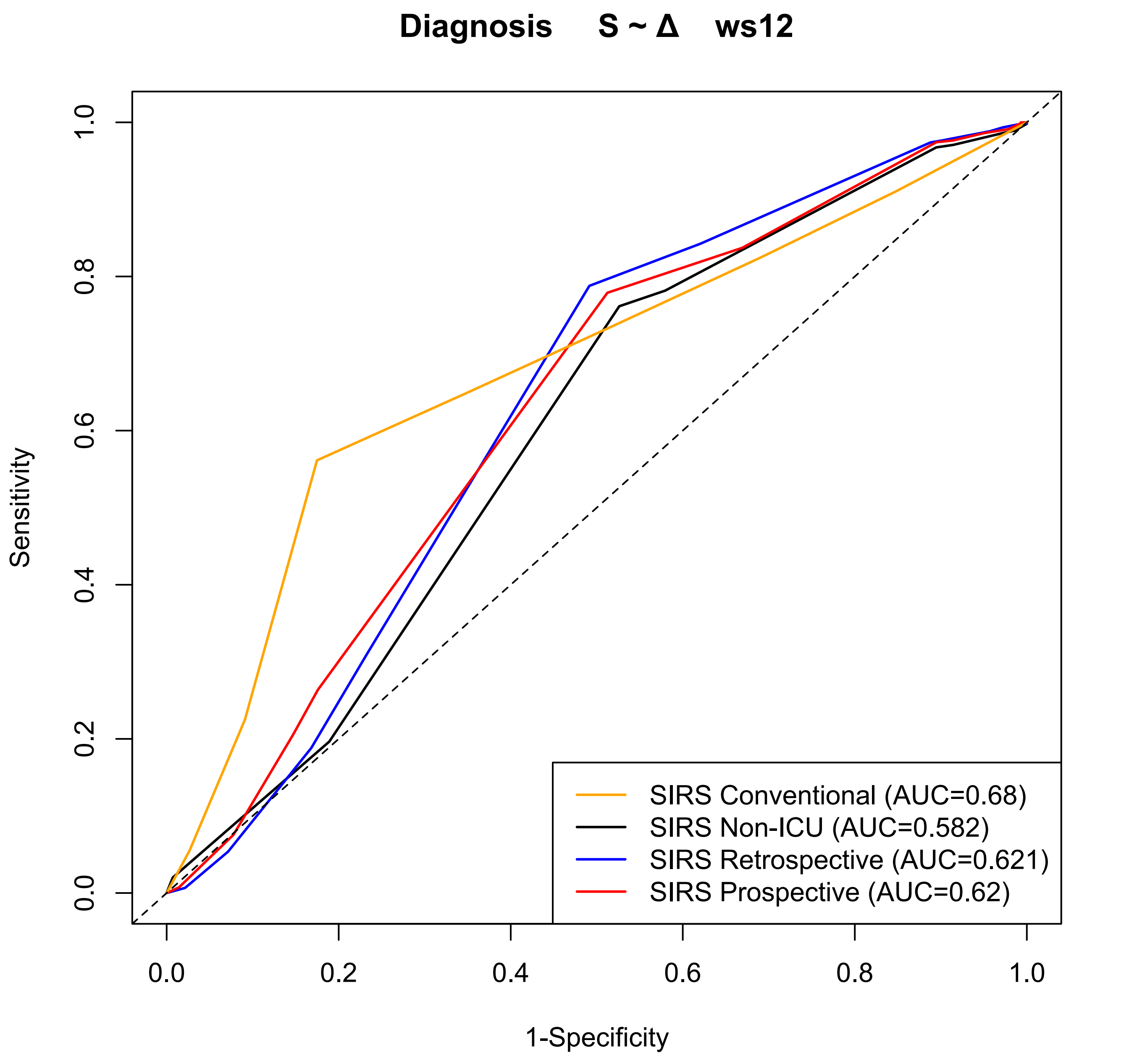

Diagnosis S ~ Δ ws12
Sensitivity
1-Specificity
0.0
0.2
0.4
0.6
0.8
1.0
SIRS Conventional (AUC=0.68)
SIRS Non-ICU (AUC=0.582)
SIRS Retrospective (AUC=0.621)
SIRS Prospective (AUC=0.62)

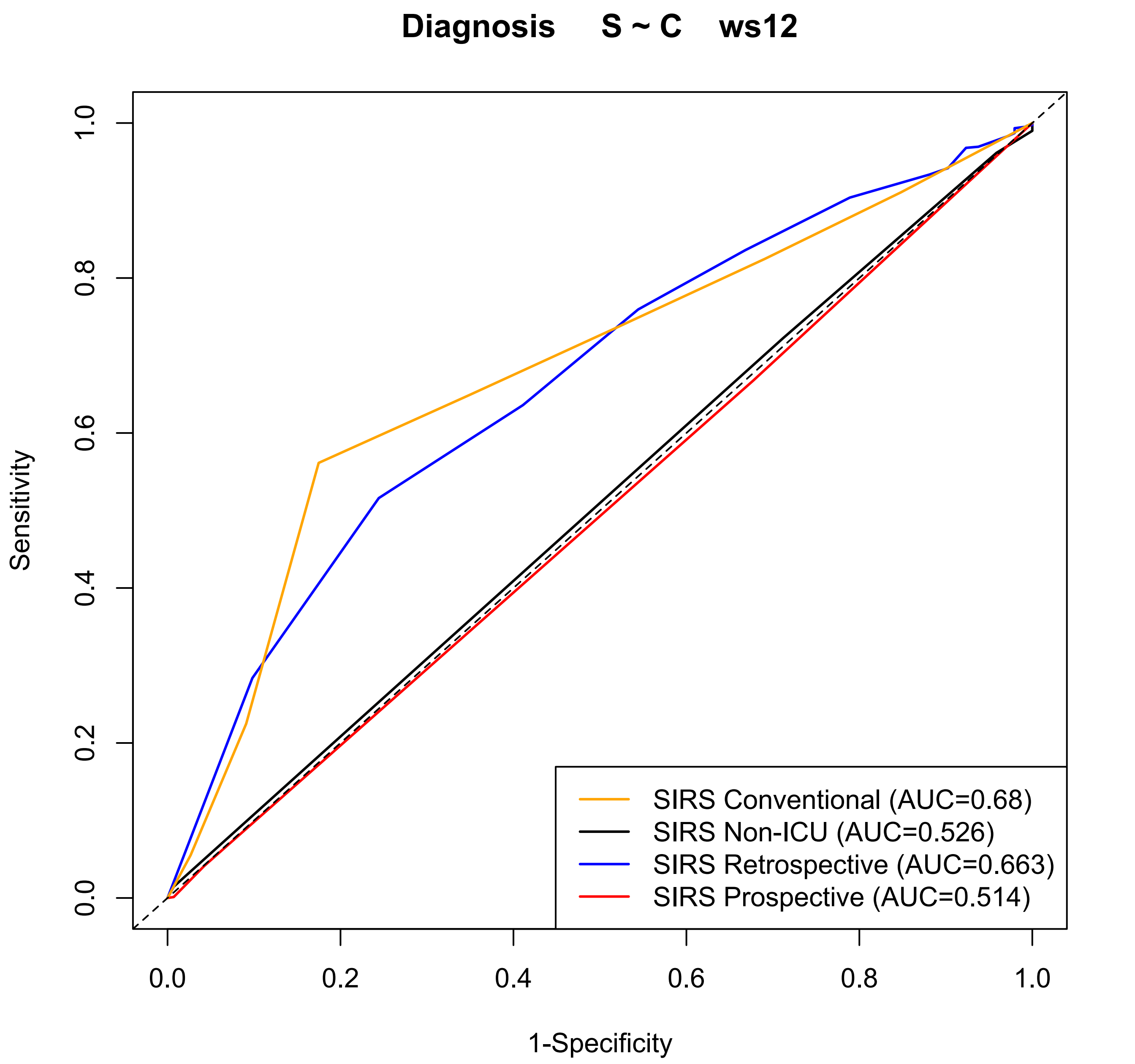
Diagnosis S ~ C ws12
Sensitivity
1-Specificity
0.0
0.2
0.4
0.6
0.8
1.0
SIRS Conventional (AUC=0.68)
SIRS Non-ICU (AUC=0.526)
SIRS Retrospective (AUC=0.663)
SIRS Prospective (AUC=0.514)

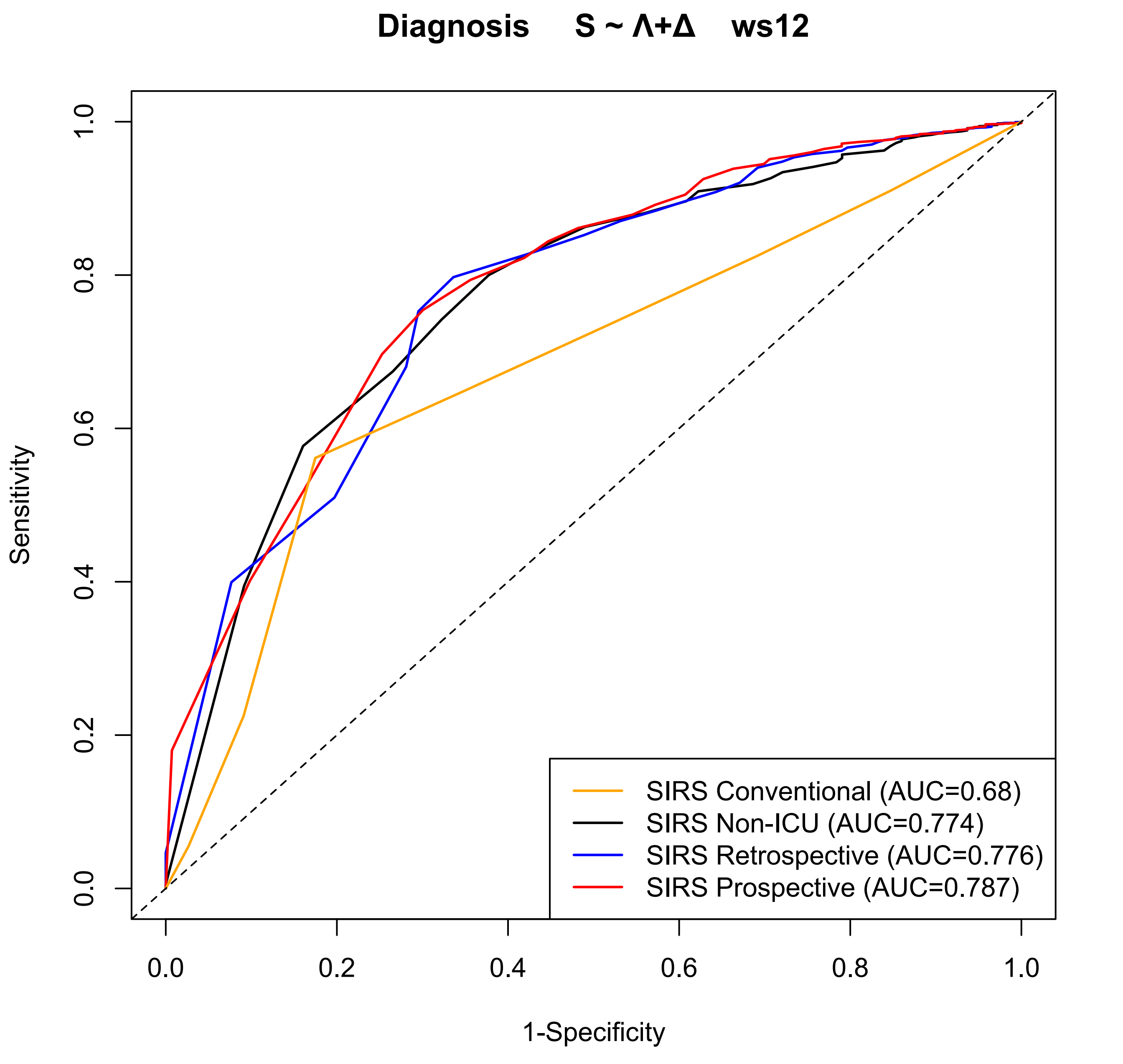
Diagnosis S ~ Λ+Δ ws12
Sensitivity
1-Specificity
0.0
0.2
0.4
0.6
0.8
1.0
SIRS Conventional (AUC=0.68)
SIRS Non-ICU (AUC=0.774)
SIRS Retrospective (AUC=0.776)
SIRS Prospective (AUC=0.787)

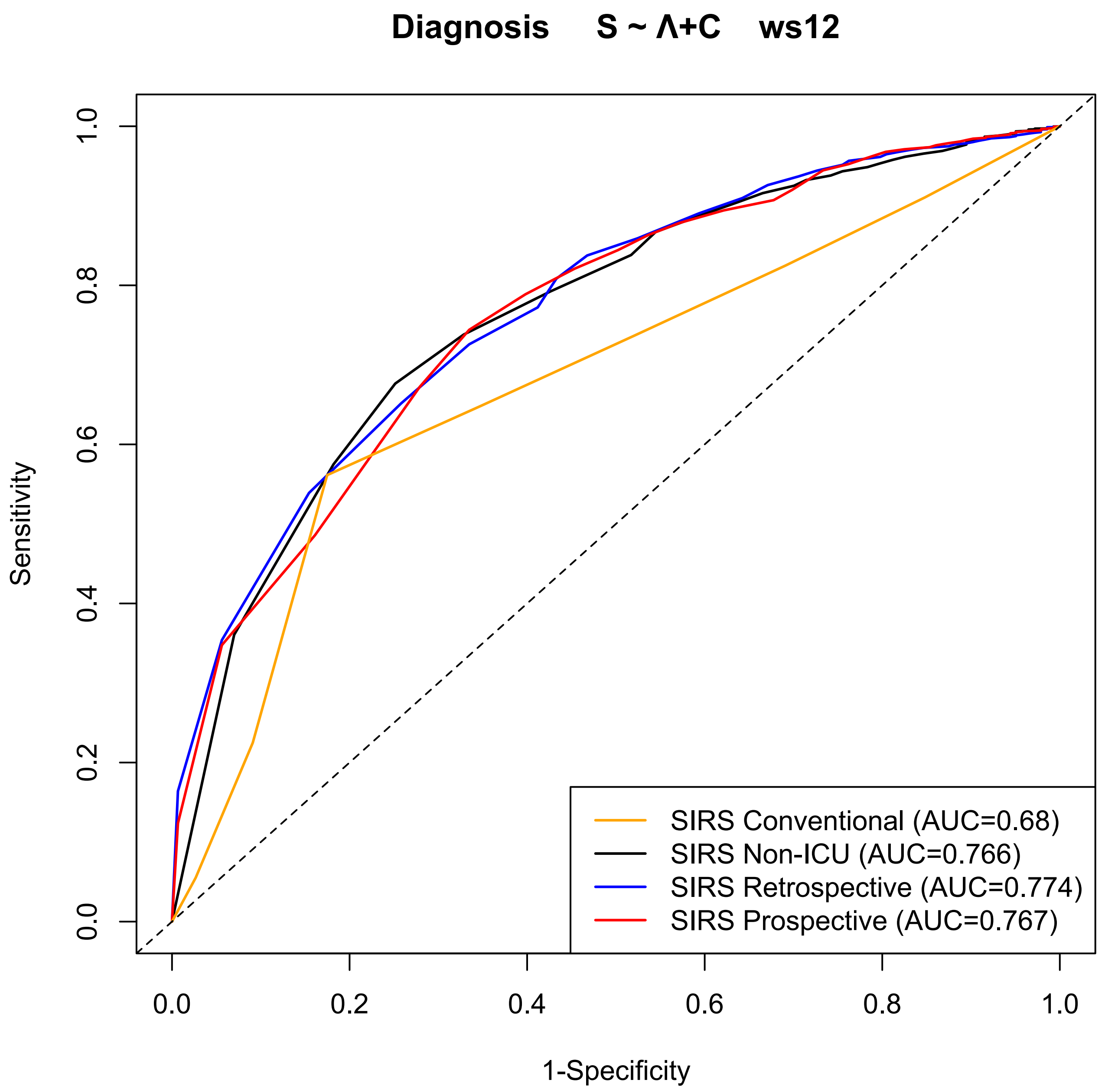
Diagnosis S ~ Λ+C ws12
Sensitivity
1-Specificity
0.0
0.2
0.4
0.6
0.8
1.0
SIRS Conventional (AUC=0.68)
SIRS Non-ICU (AUC=0.766)
SIRS Retrospective (AUC=0.774)
SIRS Prospective (AUC=0.767)

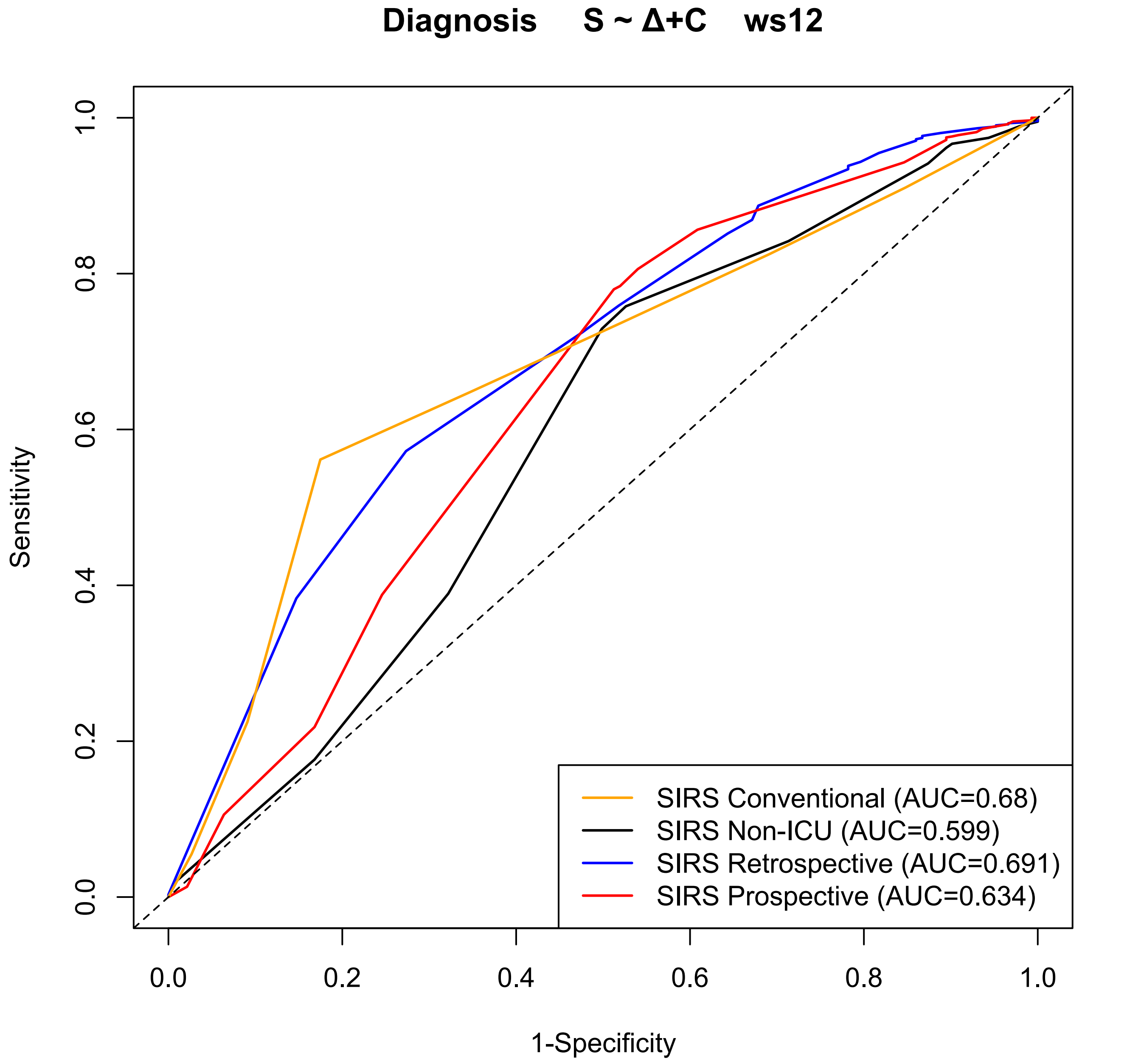

Diagnosis S ~ Δ+C ws12
Sensitivity
1-Specificity
0.0
0.2
0.4
0.6
0.8
1.0
SIRS Conventional (AUC=0.68)
SIRS Non-ICU (AUC=0.599)
SIRS Retrospective (AUC=0.691)
SIRS Prospective (AUC=0.634)

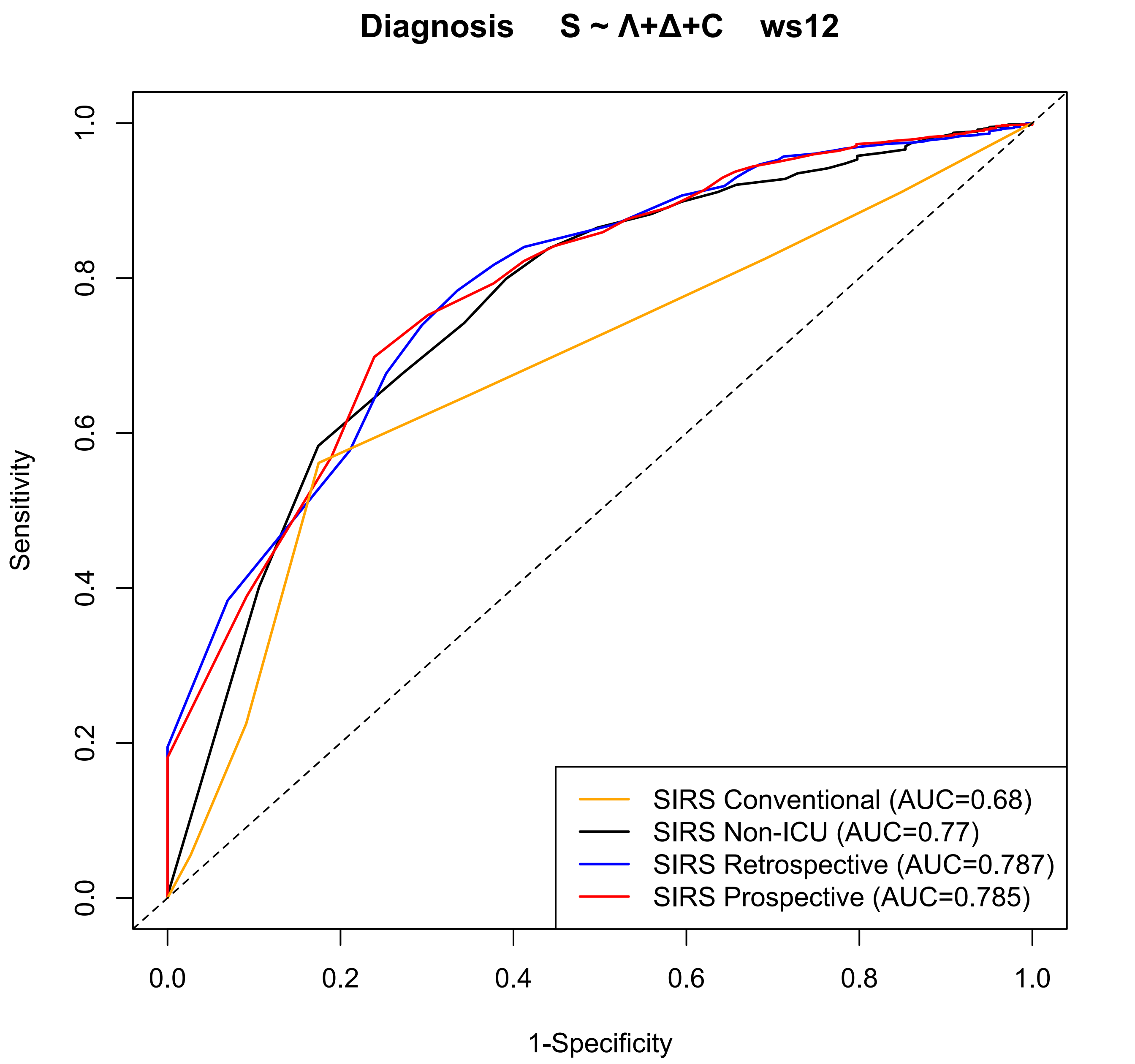

Diagnosis S ~ Λ+Δ+C ws12
Sensitivity
1-Specificity
0.0
0.2
0.4
0.6
0.8
1.0
0.0
0.2
0.4
0.6
0.8
1.0
SIRS Conventional (AUC=0.68)
SIRS Non-ICU (AUC=0.77)
SIRS Retrospective (AUC=0.787)
SIRS Prospective (AUC=0.785)

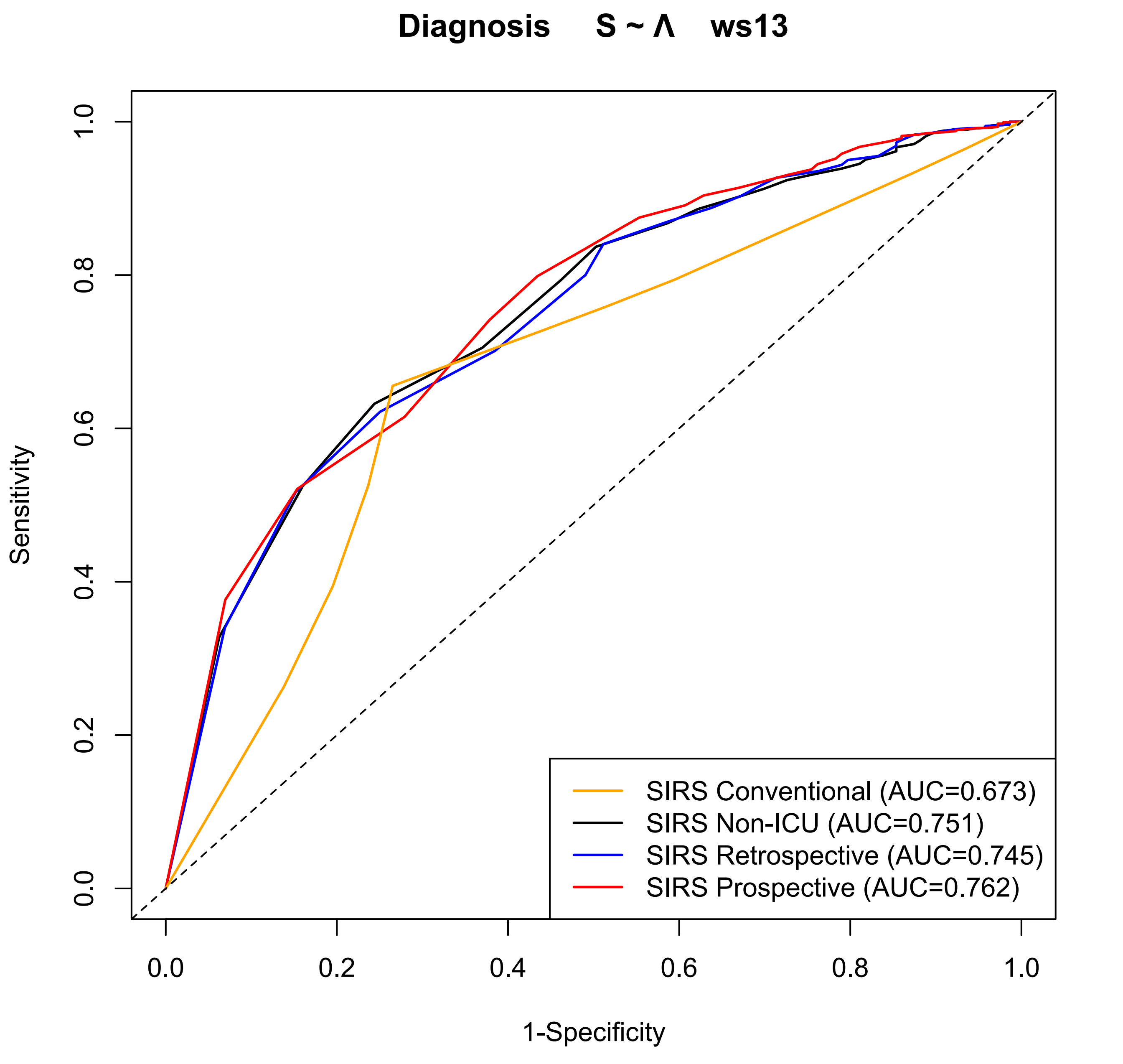

Diagnosis S ~ Λ ws13
Sensitivity
1-Specificity
0.0
0.2
0.4
0.6
0.8
1.0
SIRS Conventional (AUC=0.673)
SIRS Non-ICU (AUC=0.751)
SIRS Retrospective (AUC=0.745)
SIRS Prospective (AUC=0.762)

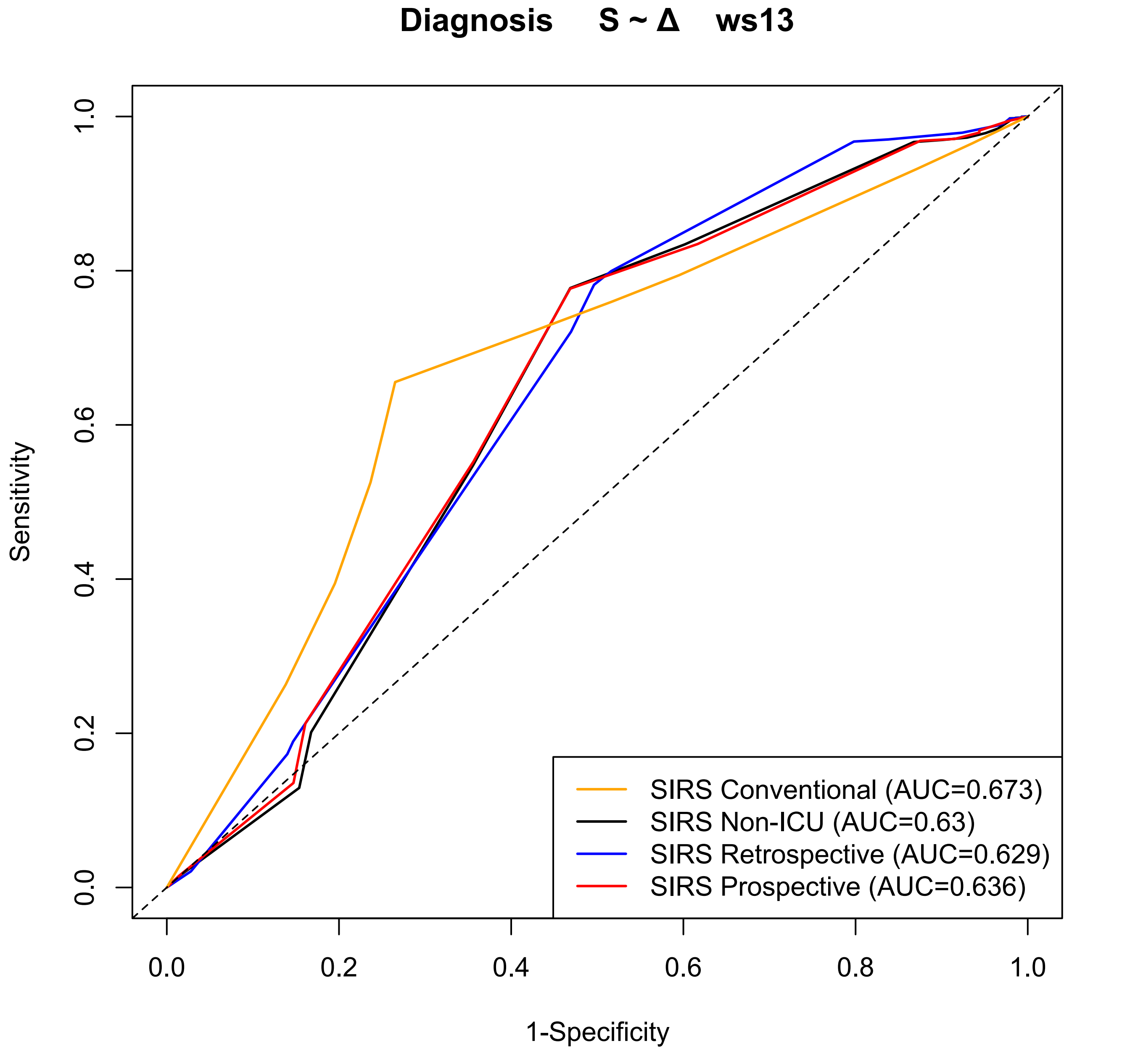
Diagnosis S ~ Δ ws13
Sensitivity
1-Specificity
0.0
0.2
0.4
0.6
0.8
1.0
SIRS Conventional (AUC=0.673)
SIRS Non-ICU (AUC=0.63)
SIRS Retrospective (AUC=0.629)
SIRS Prospective (AUC=0.636)

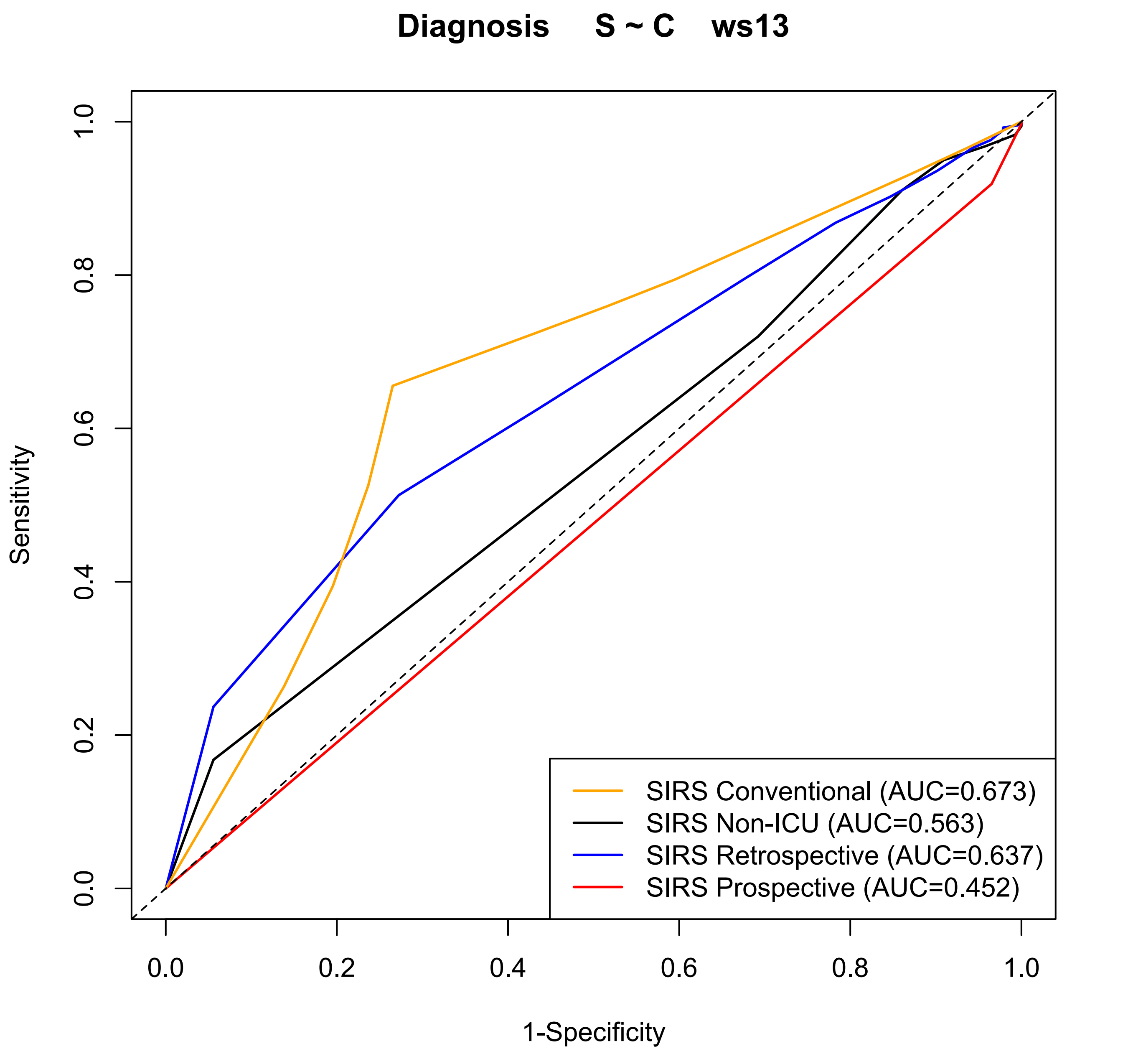

Diagnosis S ~ C ws13
Sensitivity
1-Specificity
0.0
0.2
0.4
0.6
0.8
1.0
SIRS Conventional (AUC=0.673)
SIRS Non-ICU (AUC=0.563)
SIRS Retrospective (AUC=0.637)
SIRS Prospective (AUC=0.452)

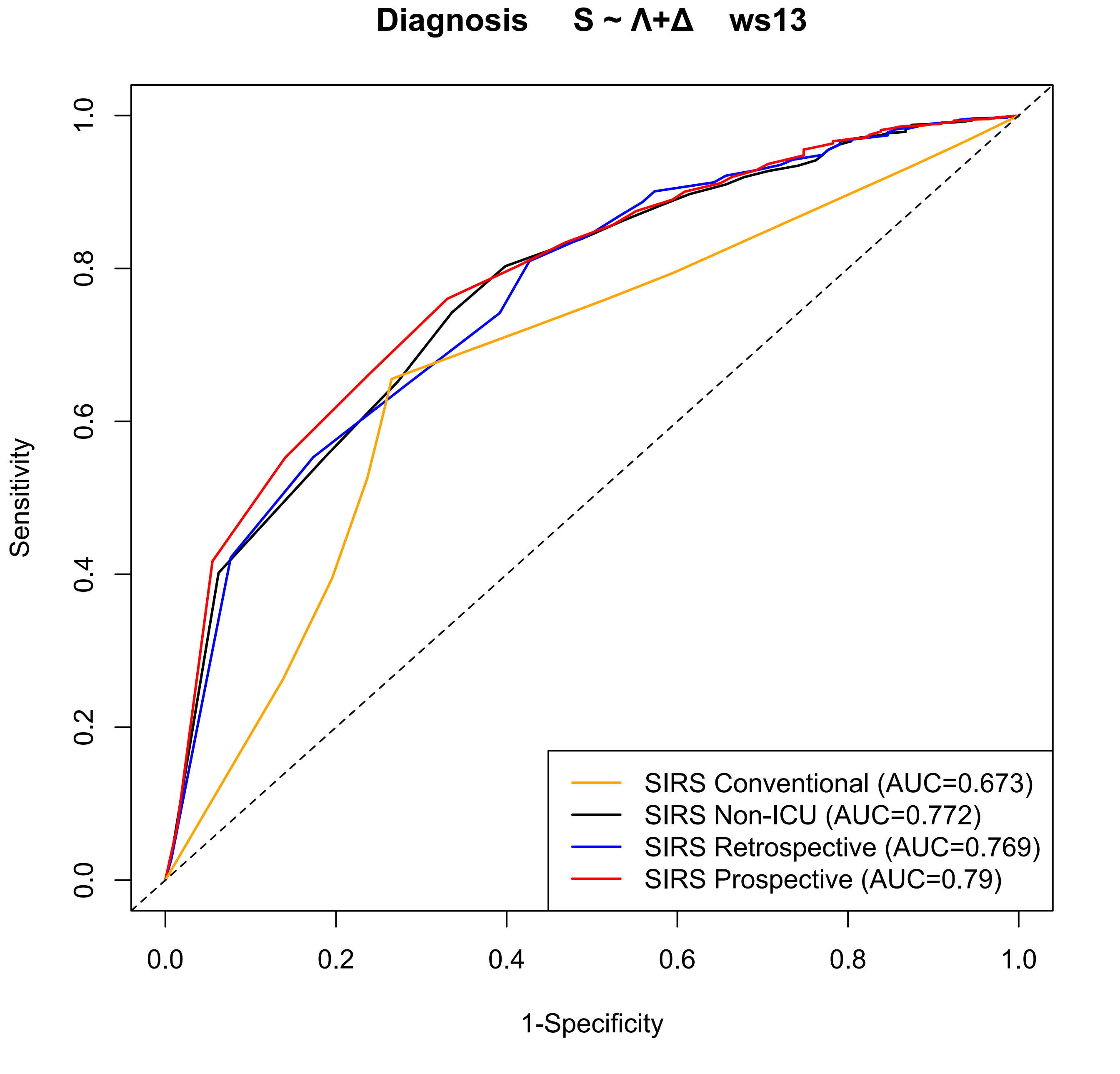

Diagnosis S ~ Λ+Δ ws13
Sensitivity
1-Specificity
0.0
0.2
0.4
0.6
0.8
1.0
SIRS Conventional (AUC=0.673)
SIRS Non-ICU (AUC=0.772)
SIRS Retrospective (AUC=0.769)
SIRS Prospective (AUC=0.79)

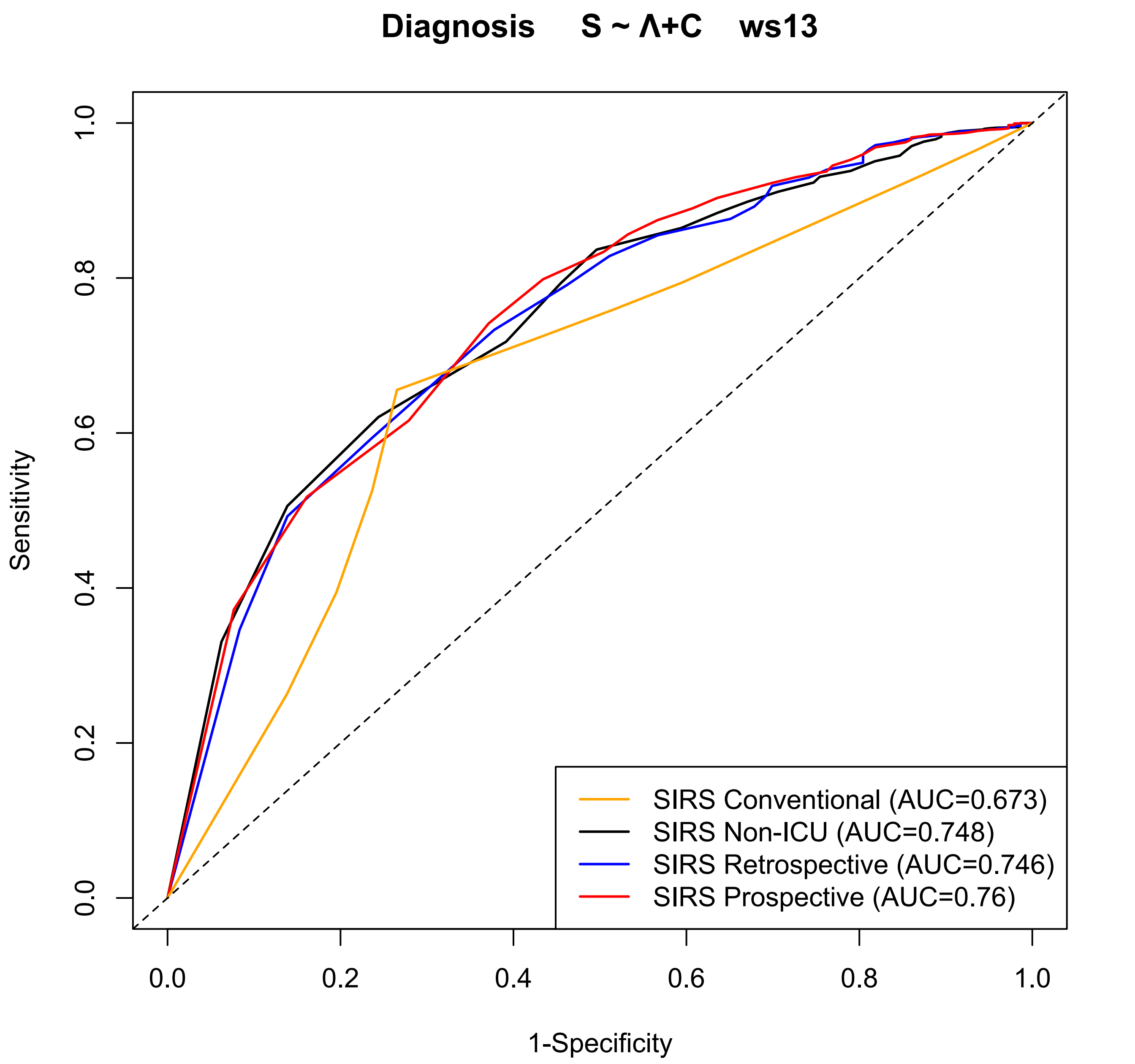

Diagnosis S ~ Λ+C ws13
Sensitivity
1-Specificity
0.0
0.2
0.4
0.6
0.8
1.0
0.0
0.2
0.4
0.6
0.8
1.0
SIRS Conventional (AUC=0.673)
SIRS Non-ICU (AUC=0.748)
SIRS Retrospective (AUC=0.746)
SIRS Prospective (AUC=0.76)

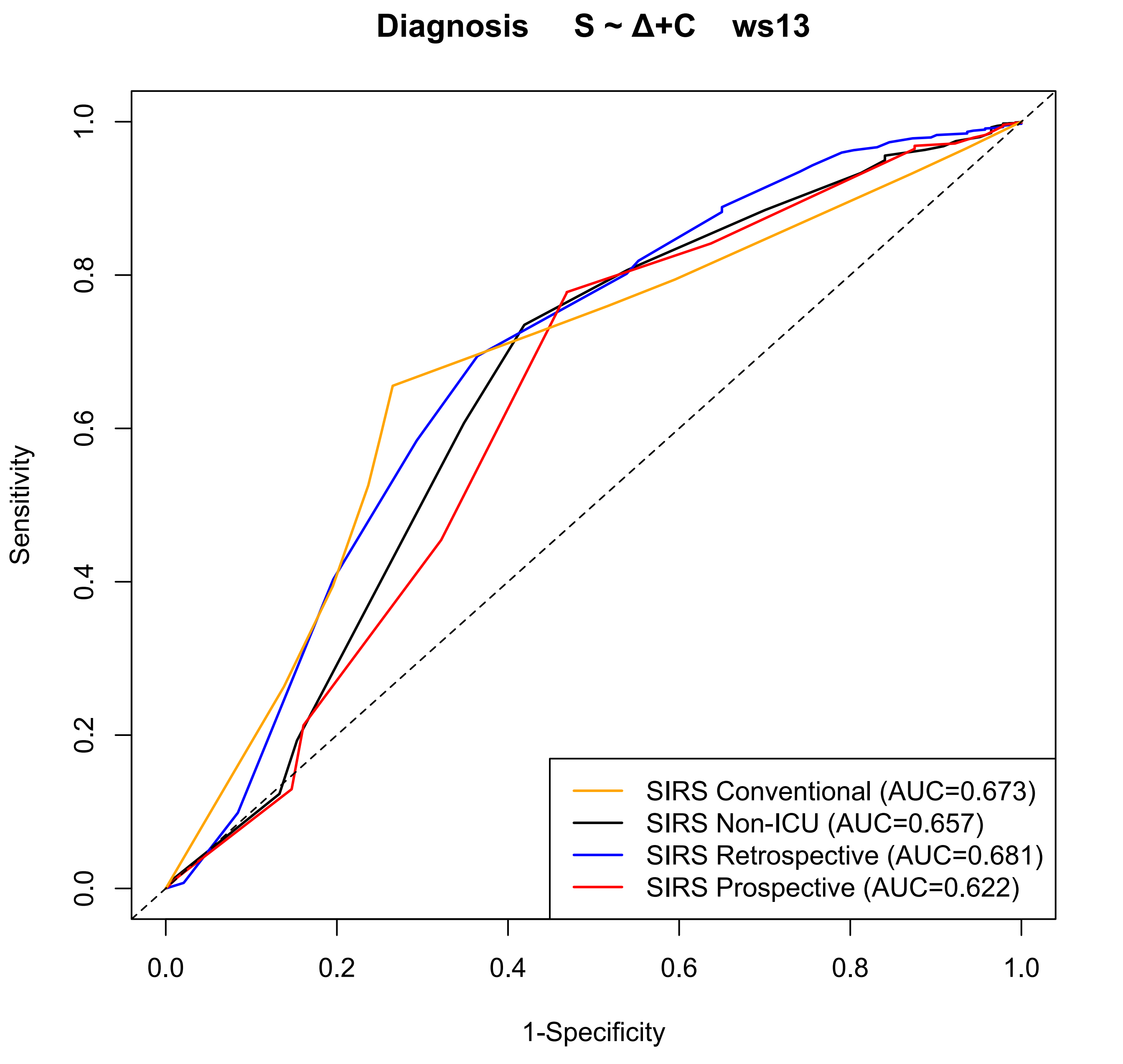

Diagnosis S ~ Δ+C ws13
Sensitivity
1-Specificity
0.0
0.2
0.4
0.6
0.8
1.0
SIRS Conventional (AUC=0.673)
SIRS Non-ICU (AUC=0.657)
SIRS Retrospective (AUC=0.681)
SIRS Prospective (AUC=0.622)

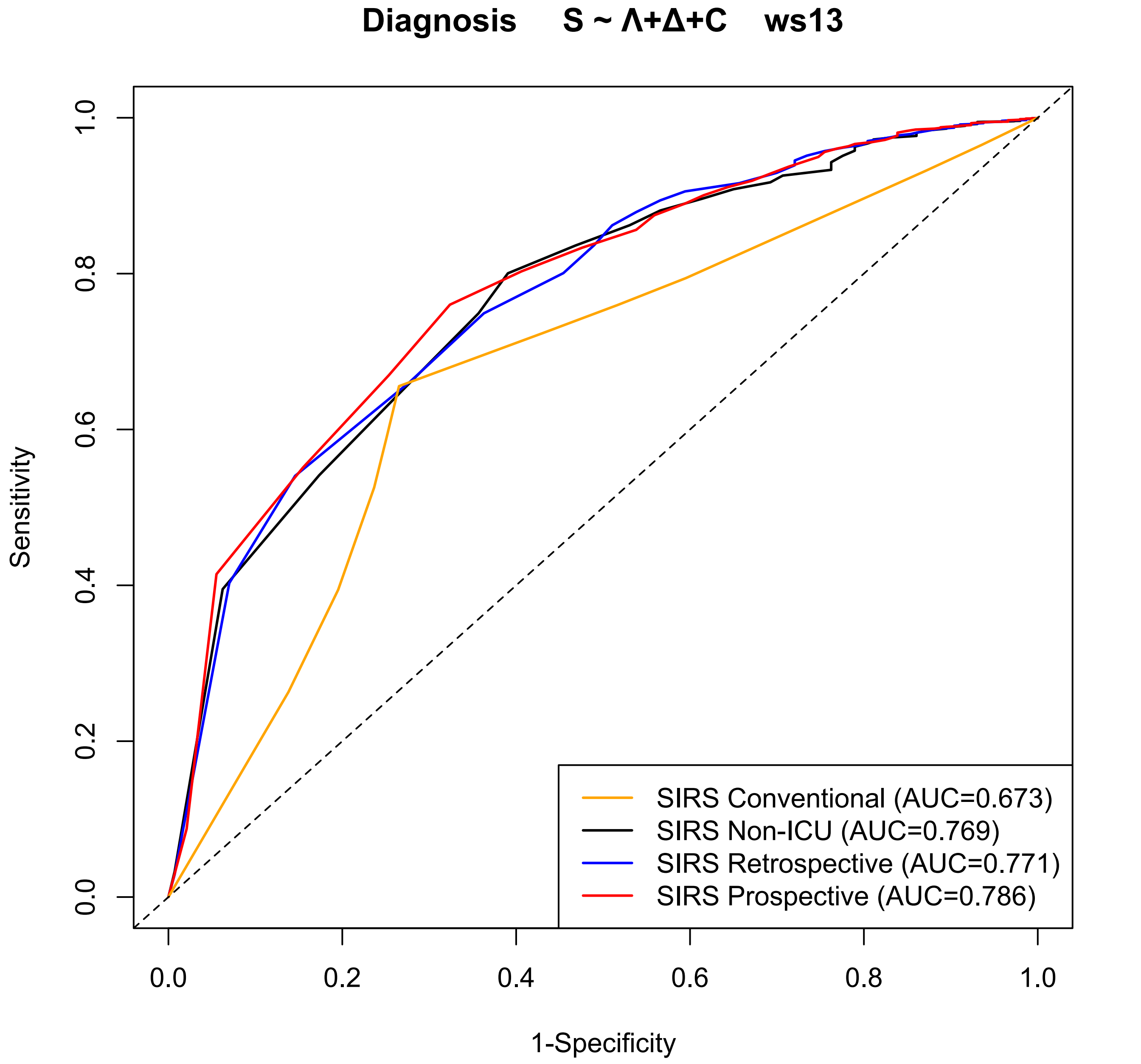
Diagnosis S ~ Λ+Δ+C ws13
Sensitivity
1-Specificity
0.0
0.2
0.4
0.6
0.8
1.0
SIRS Conventional (AUC=0.673)
SIRS Non-ICU (AUC=0.769)
SIRS Retrospective (AUC=0.771)
SIRS Prospective (AUC=0.786)

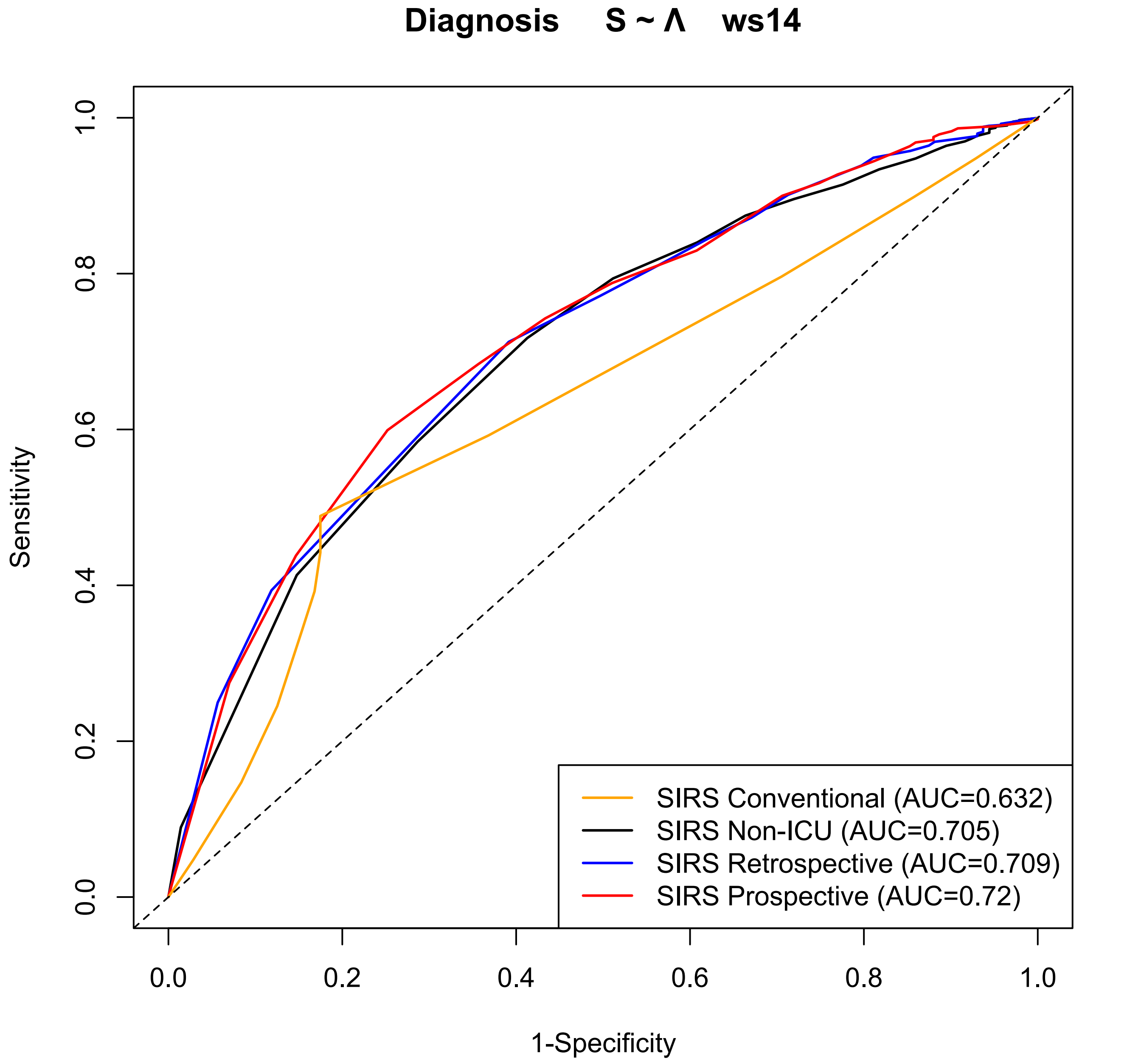

Diagnosis S ~ Λ ws14
Sensitivity
1-Specificity
0.0
0.2
0.4
0.6
0.8
1.0
SIRS Conventional (AUC=0.632)
SIRS Non-ICU (AUC=0.705)
SIRS Retrospective (AUC=0.709)
SIRS Prospective (AUC=0.72)

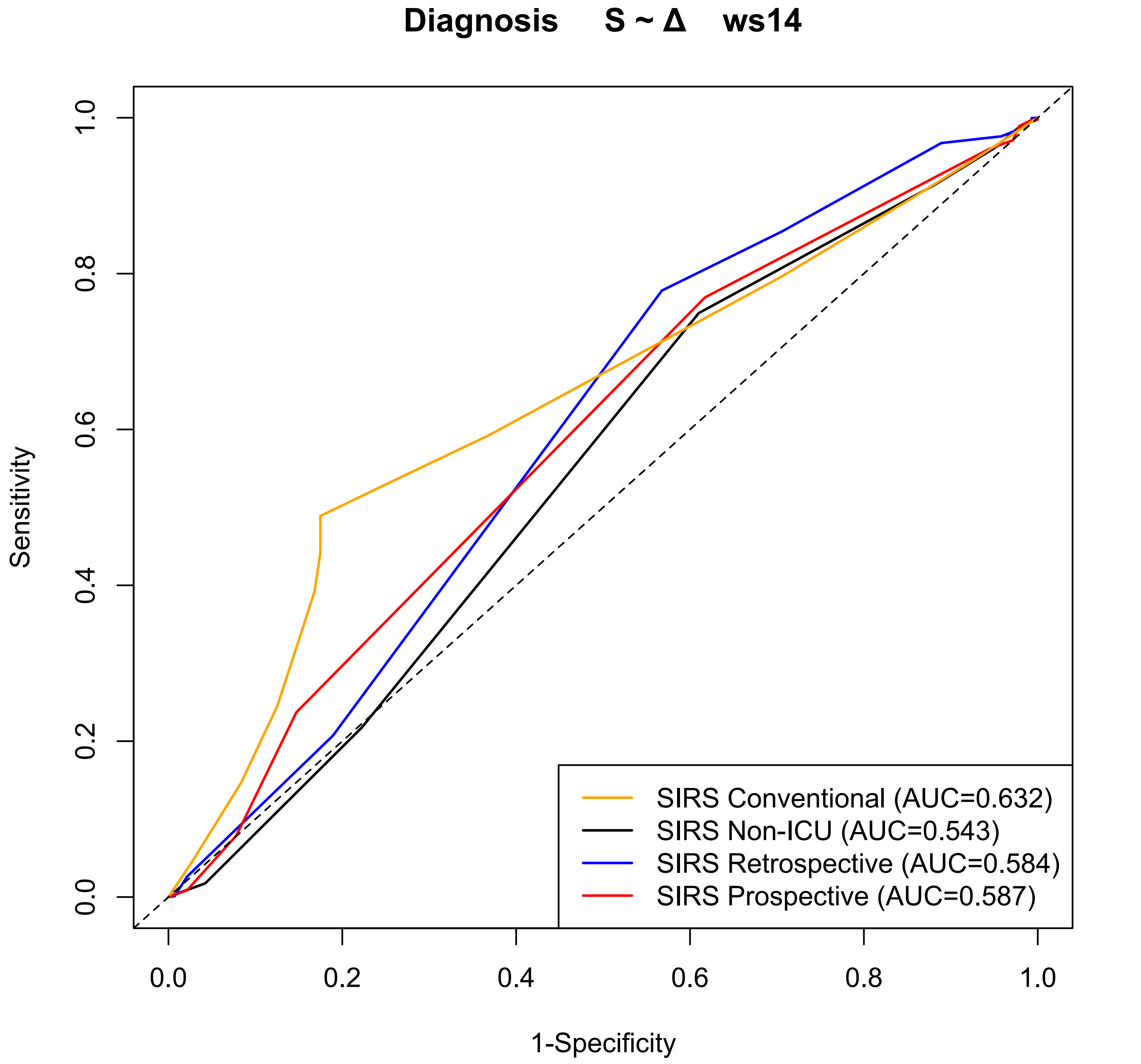

Diagnosis S ~ Δ ws14
Sensitivity
1-Specificity
0.0
0.2
0.4
0.6
0.8
1.0
SIRS Conventional (AUC=0.632)
SIRS Non-ICU (AUC=0.543)
SIRS Retrospective (AUC=0.584)
SIRS Prospective (AUC=0.587)

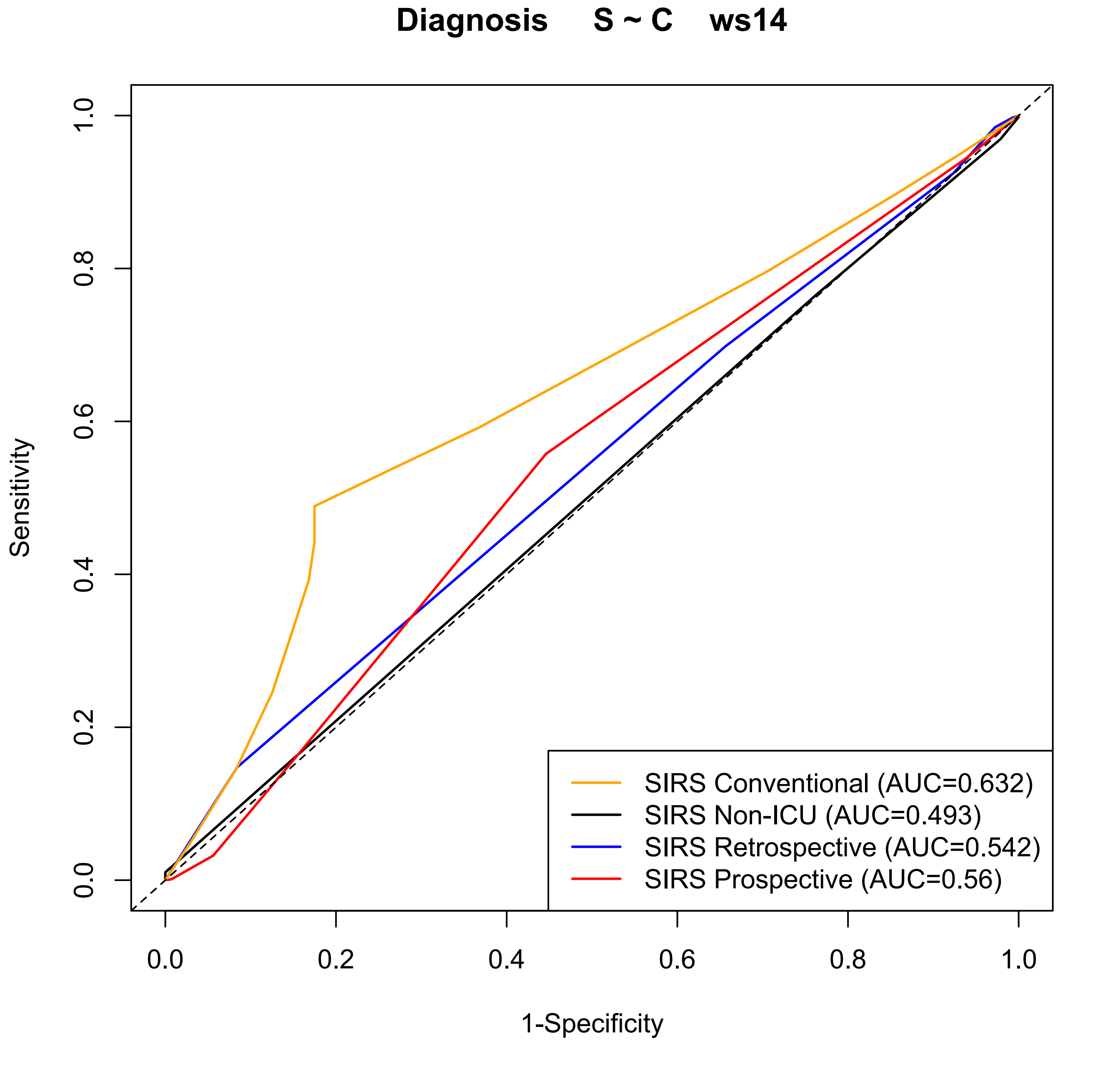
Diagnosis S ~ C ws14
Sensitivity
1-Specificity
0.0
0.2
0.4
0.6
0.8
1.0
SIRS Conventional (AUC=0.632)
SIRS Non-ICU (AUC=0.493)
SIRS Retrospective (AUC=0.542)
SIRS Prospective (AUC=0.56)

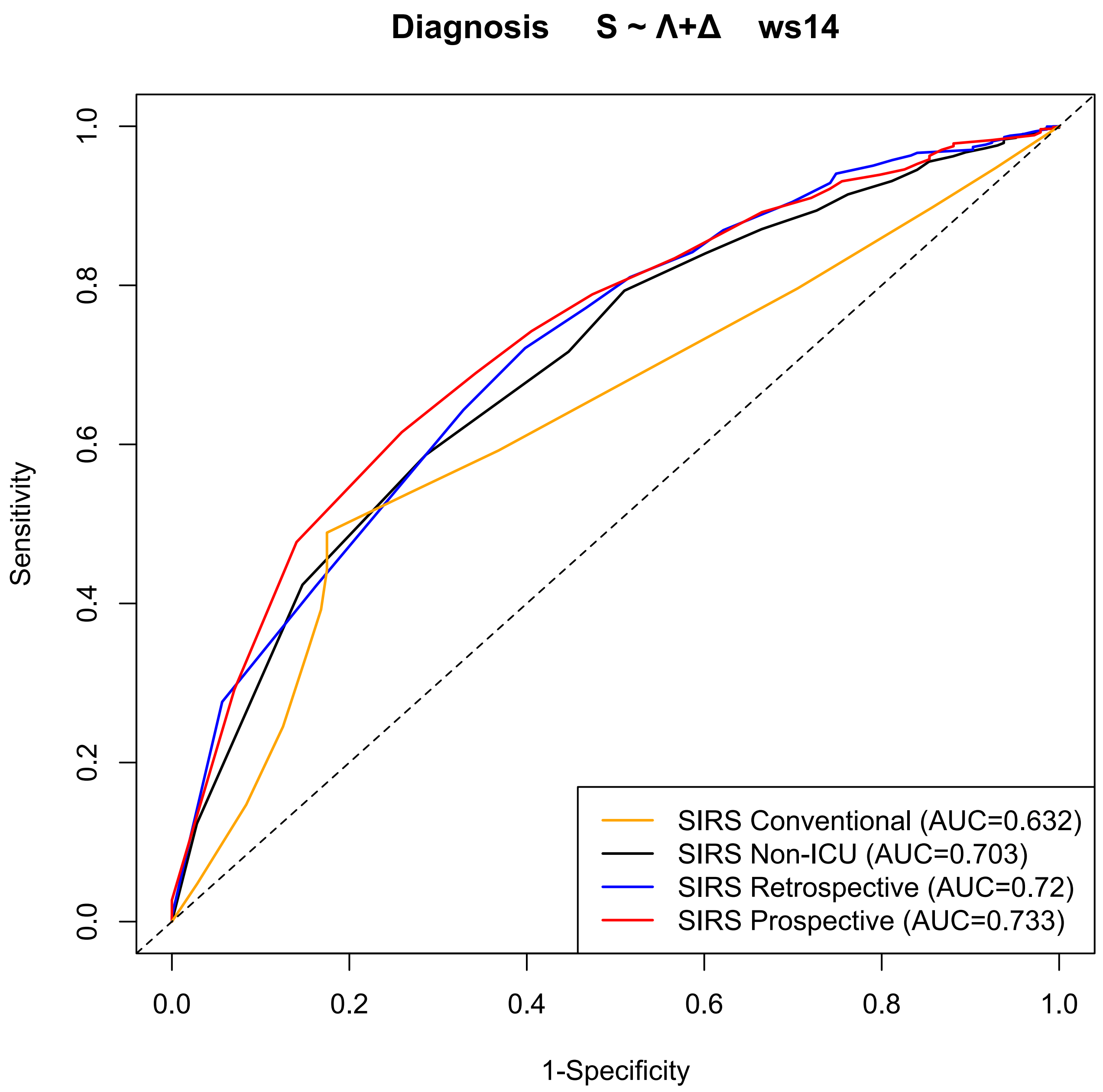

Diagnosis S ~ Λ+Δ ws14
Sensitivity
1-Specificity
0.0
0.2
0.4
0.6
0.8
1.0
SIRS Conventional (AUC=0.632)
SIRS Non-ICU (AUC=0.703)
SIRS Retrospective (AUC=0.72)
SIRS Prospective (AUC=0.733)

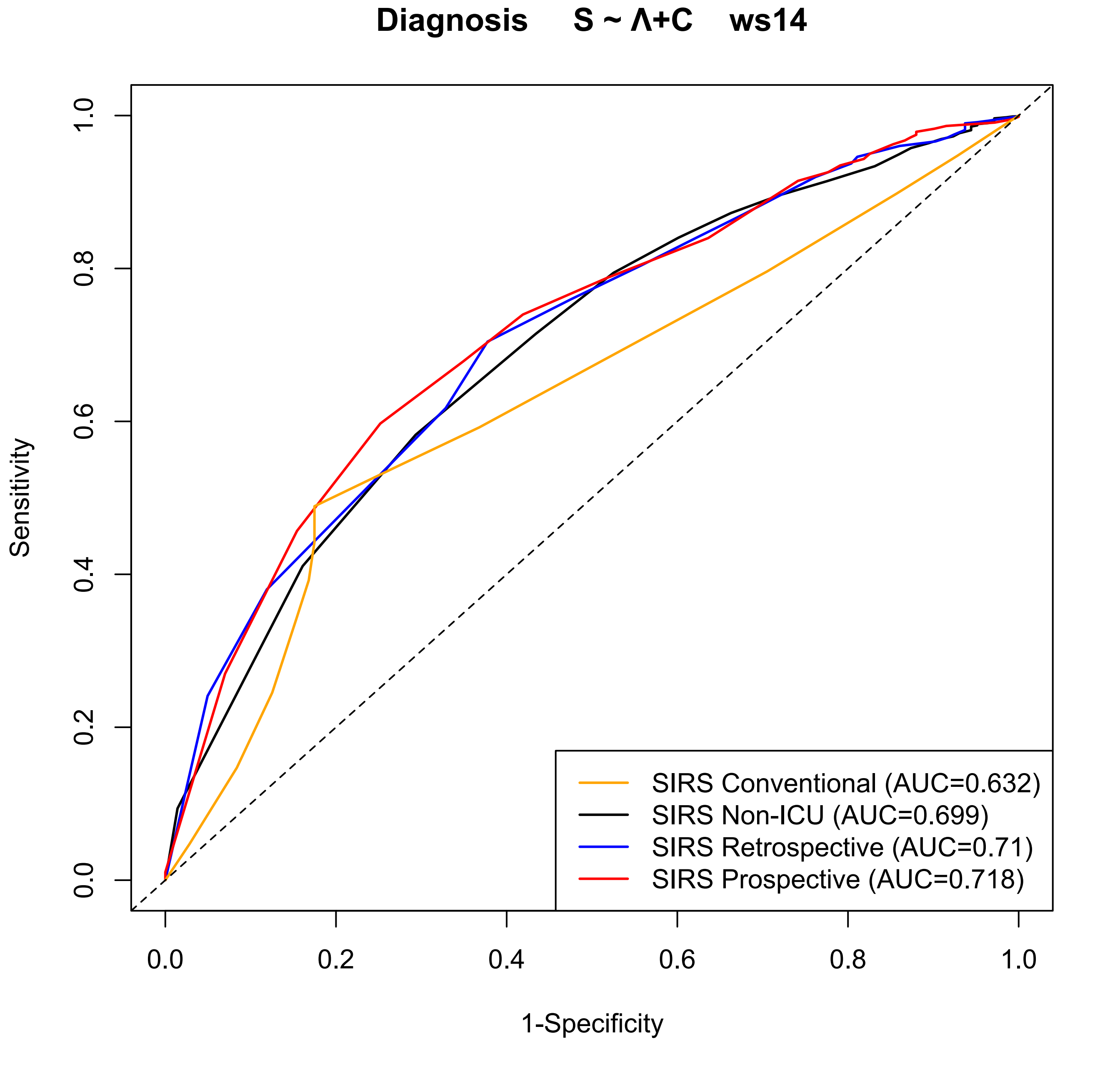
Diagnosis S ~ Λ+C ws14
Sensitivity
1-Specificity
0.0
0.2
0.4
0.6
0.8
1.0
SIRS Conventional (AUC=0.632)
SIRS Non-ICU (AUC=0.699)
SIRS Retrospective (AUC=0.71)
SIRS Prospective (AUC=0.718)

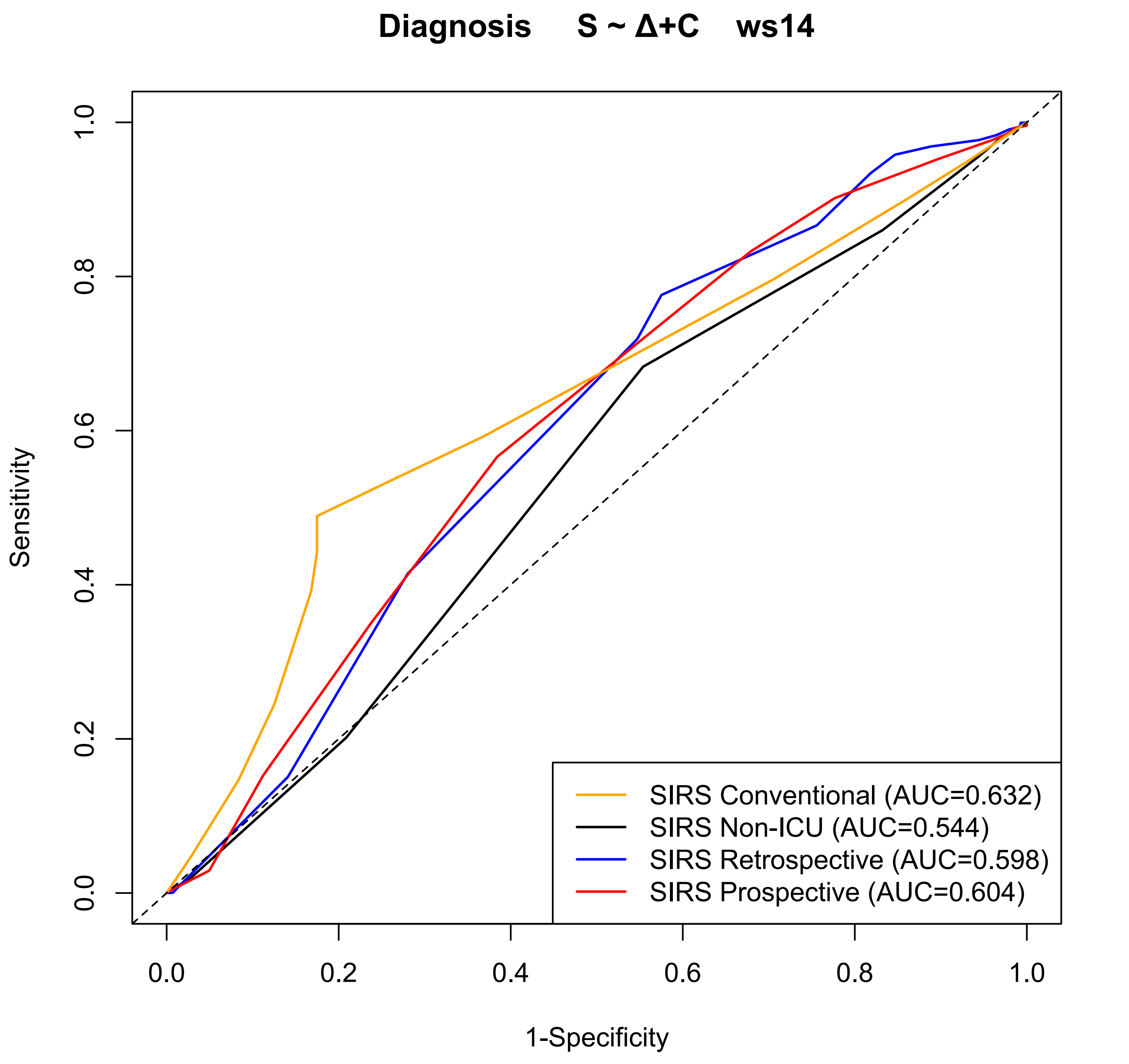

Diagnosis S ~ Δ+C ws14
Sensitivity
1-Specificity
0.0
0.2
0.4
0.6
0.8
1.0
SIRS Conventional (AUC=0.632)
SIRS Non-ICU (AUC=0.544)
SIRS Retrospective (AUC=0.598)
SIRS Prospective (AUC=0.604)

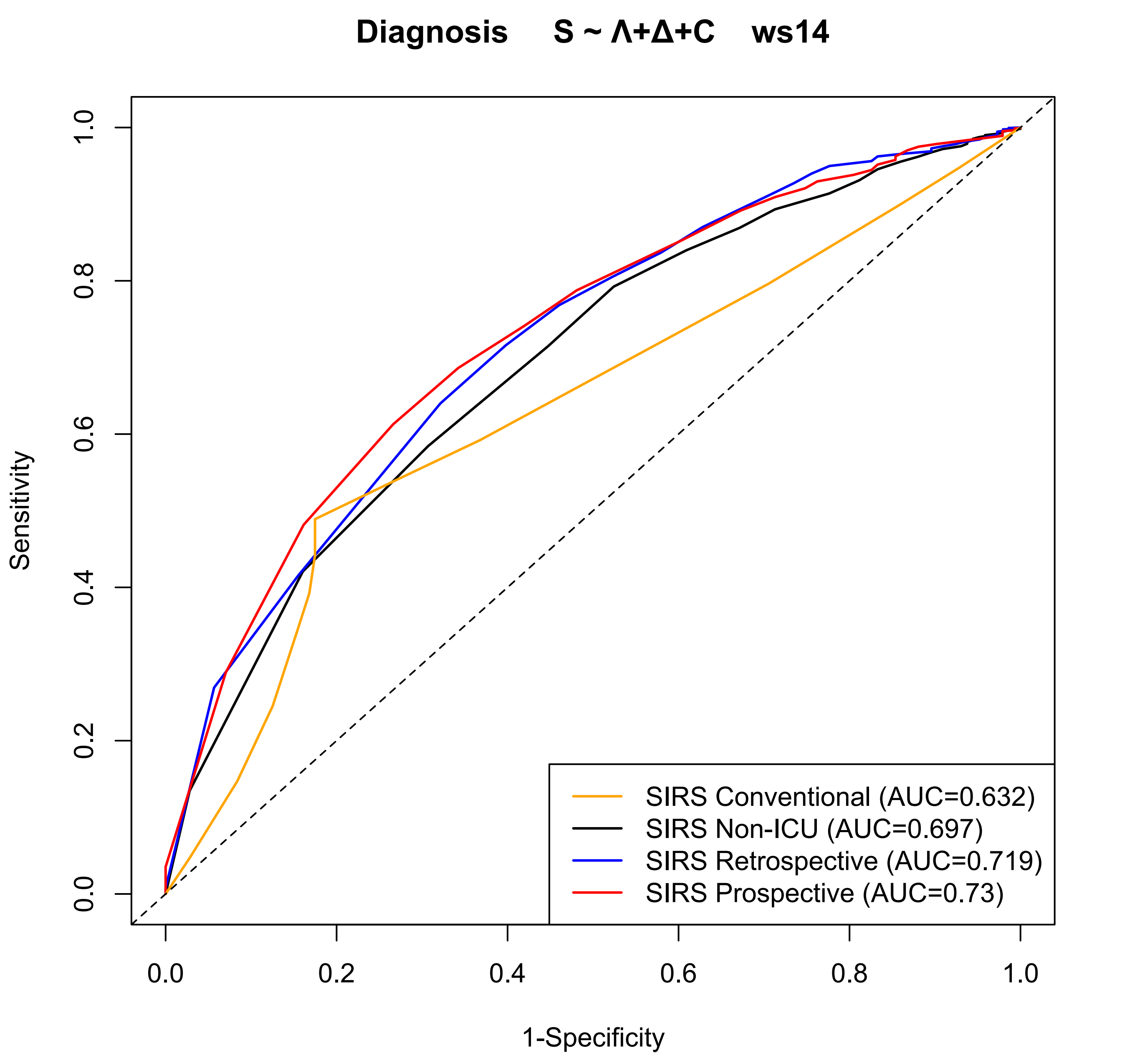

Diagnosis S ~ Λ+Δ+C ws14
Sensitivity
1-Specificity
0.0
0.2
0.4
0.6
0.8
1.0
SIRS Conventional (AUC=0.632)
SIRS Non-ICU (AUC=0.697)
SIRS Retrospective (AUC=0.719)
SIRS Prospective (AUC=0.73)

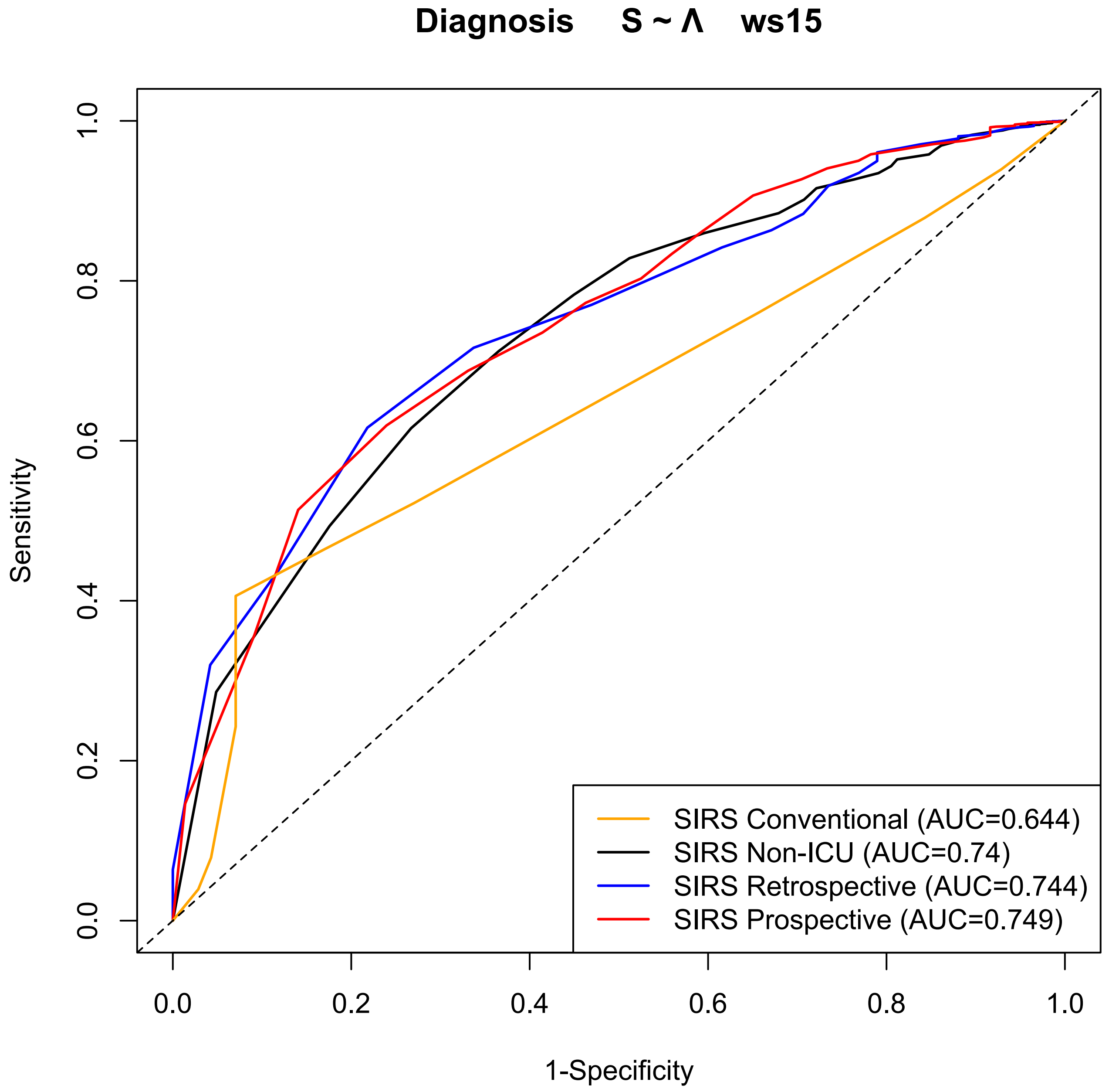
Diagnosis S ~ Λ ws15
Sensitivity
1-Specificity
0.0
0.2
0.4
0.6
0.8
1.0
SIRS Conventional (AUC=0.644)
SIRS Non-ICU (AUC=0.74)
SIRS Retrospective (AUC=0.744)
SIRS Prospective (AUC=0.749)

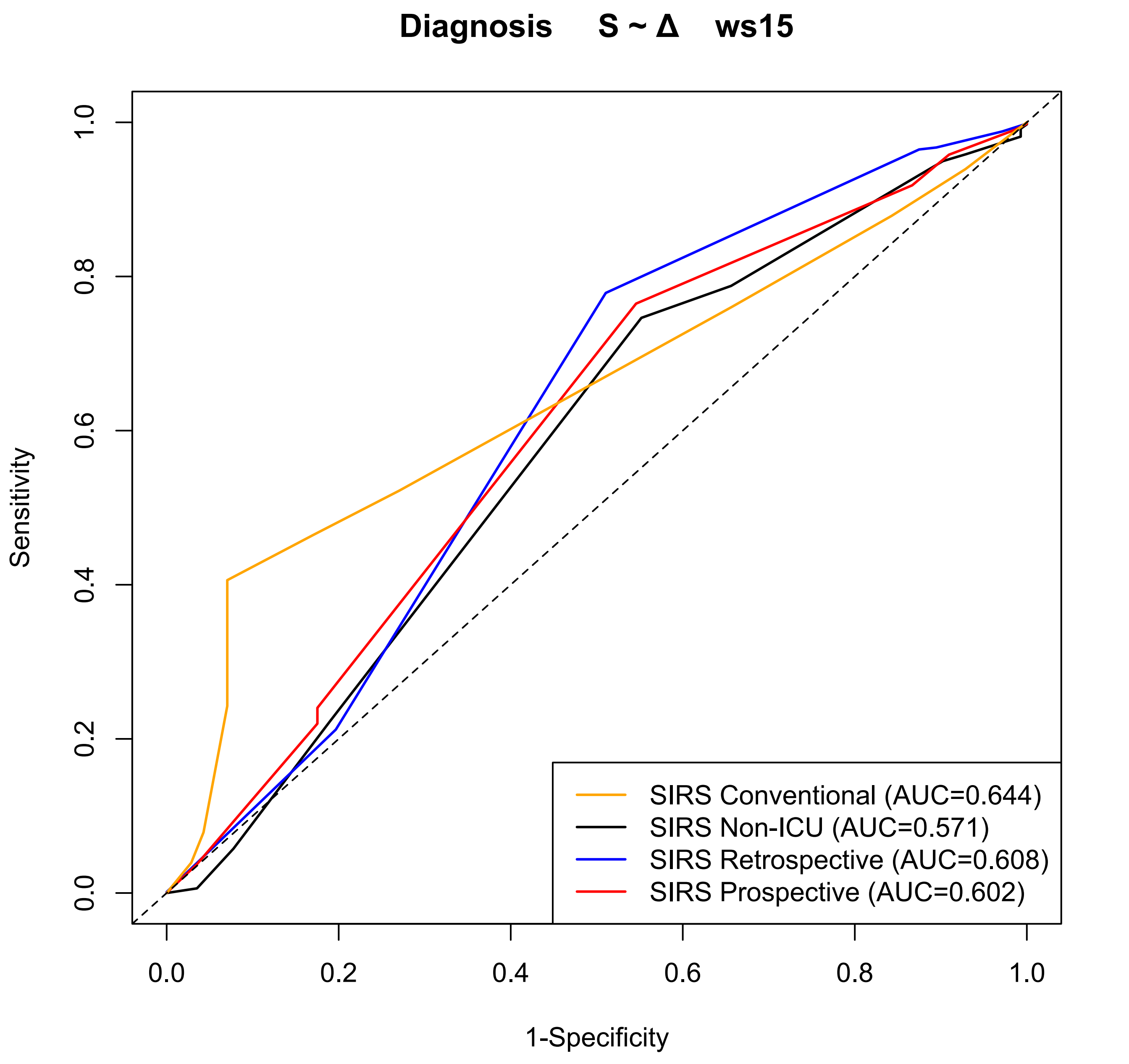

Diagnosis S ~ Δ ws15
Sensitivity
1-Specificity
0.0
0.2
0.4
0.6
0.8
1.0
SIRS Conventional (AUC=0.644)
SIRS Non-ICU (AUC=0.571)
SIRS Retrospective (AUC=0.608)
SIRS Prospective (AUC=0.602)

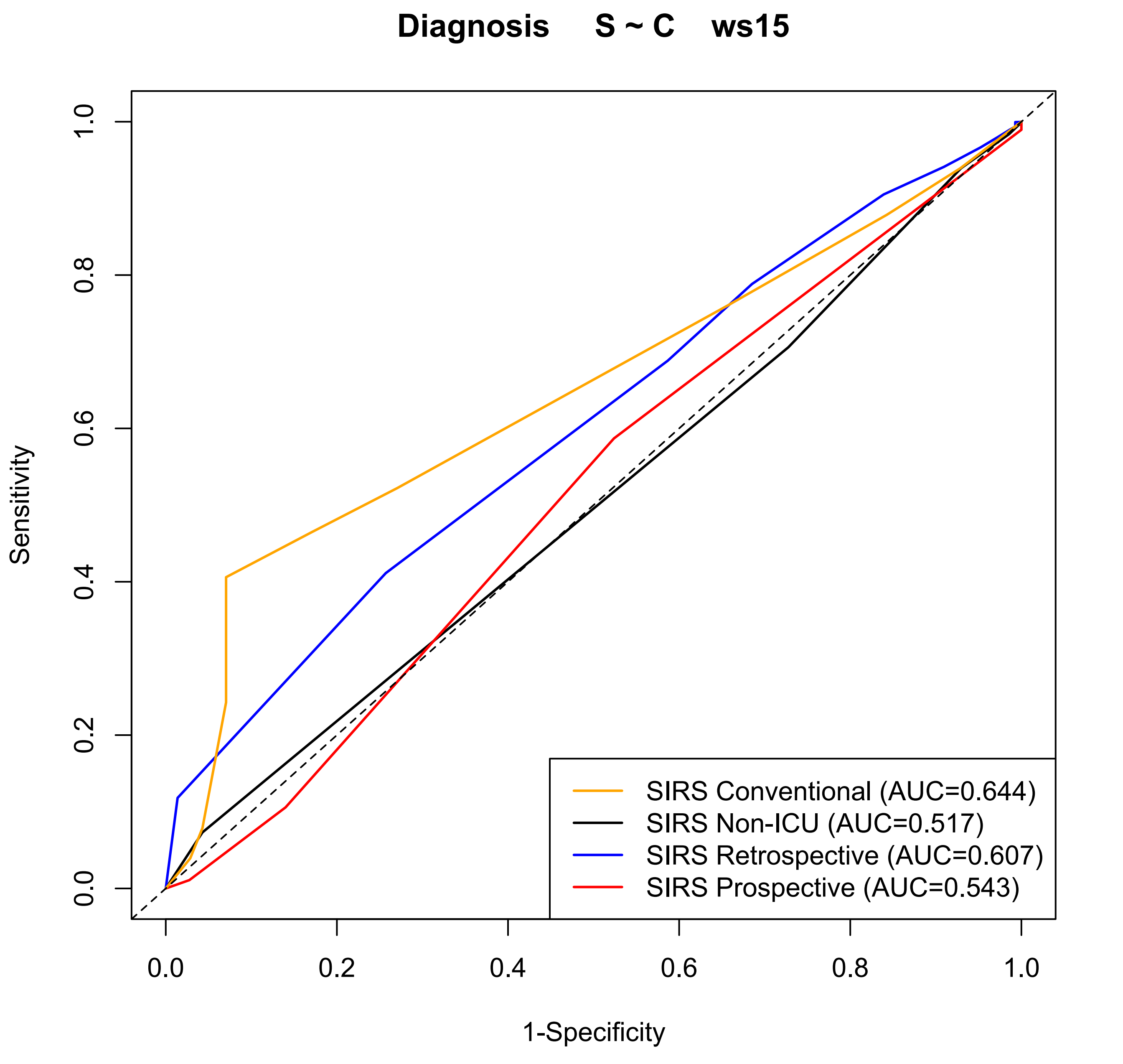

Diagnosis S ~ C ws15
Sensitivity
1-Specificity
0.0
0.2
0.4
0.6
0.8
1.0
SIRS Conventional (AUC=0.644)
SIRS Non-ICU (AUC=0.517)
SIRS Retrospective (AUC=0.607)
SIRS Prospective (AUC=0.543)

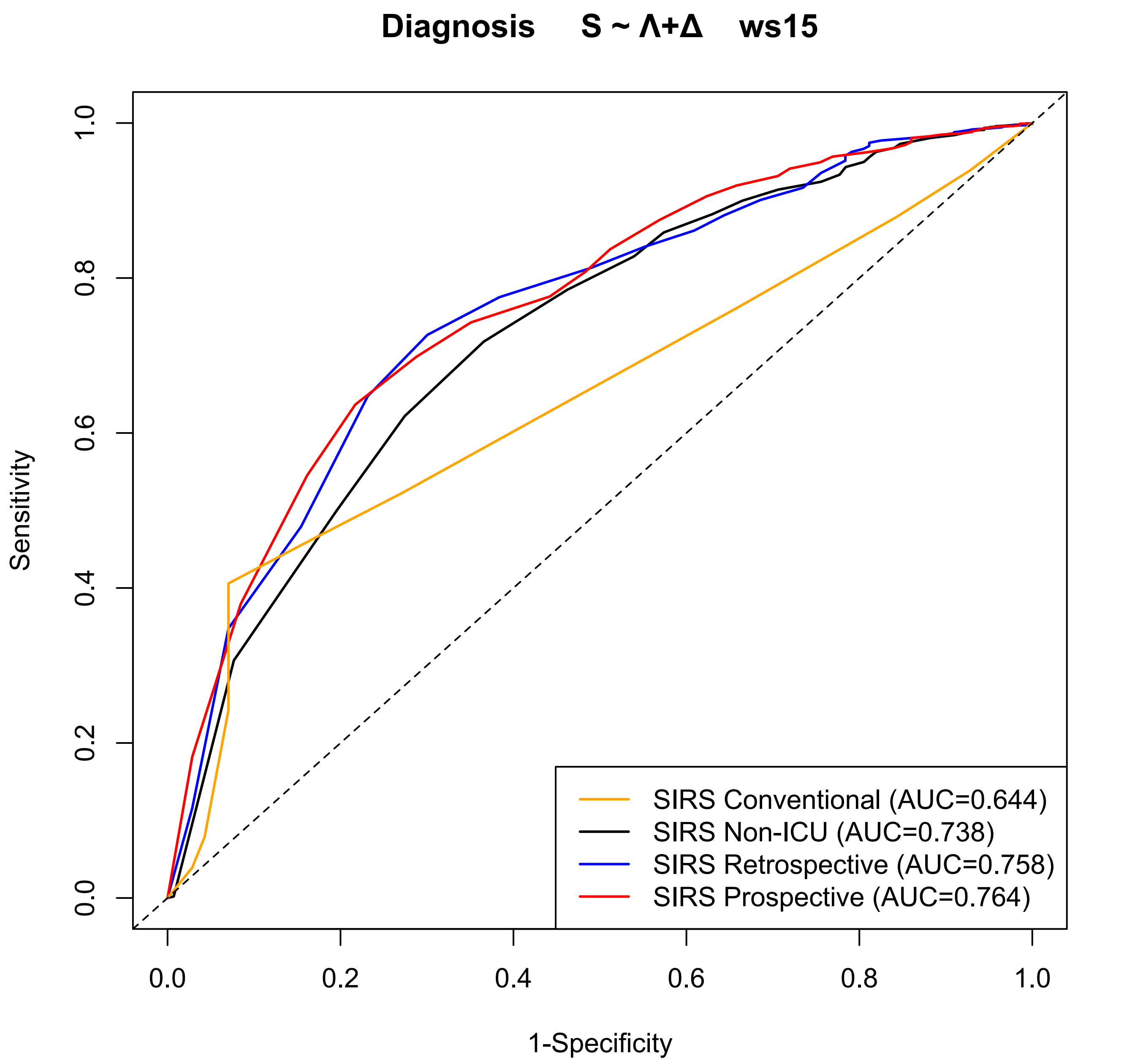
Diagnosis S ~ Λ+Δ ws15
Sensitivity
1-Specificity
0.0
0.2
0.4
0.6
0.8
1.0
SIRS Conventional (AUC=0.644)
SIRS Non-ICU (AUC=0.738)
SIRS Retrospective (AUC=0.758)
SIRS Prospective (AUC=0.764)

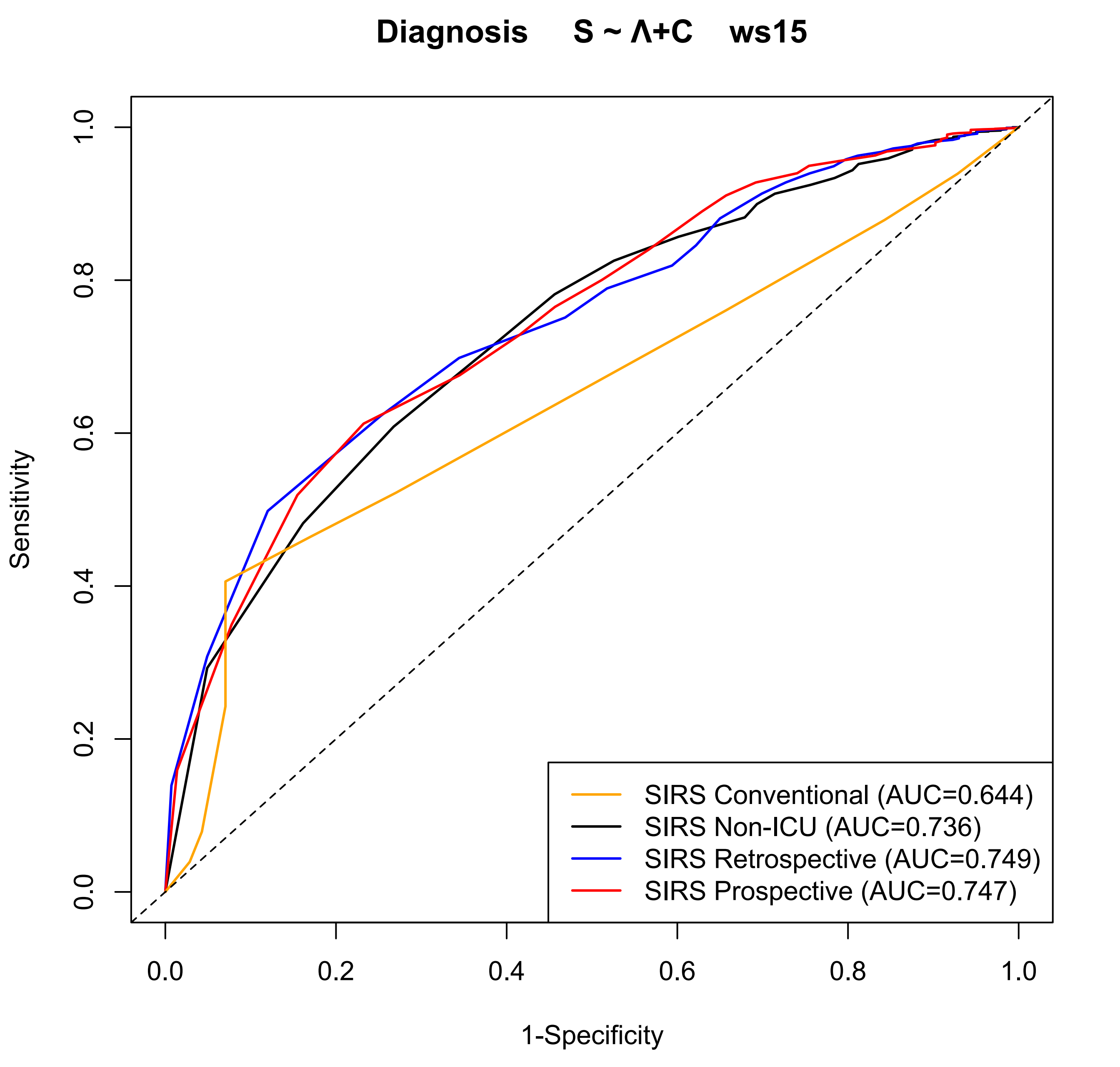
Diagnosis S ~ Λ+C ws15
Sensitivity
1-Specificity
0.0
0.2
0.4
0.6
0.8
1.0
SIRS Conventional (AUC=0.644)
SIRS Non-ICU (AUC=0.736)
SIRS Retrospective (AUC=0.749)
SIRS Prospective (AUC=0.747)

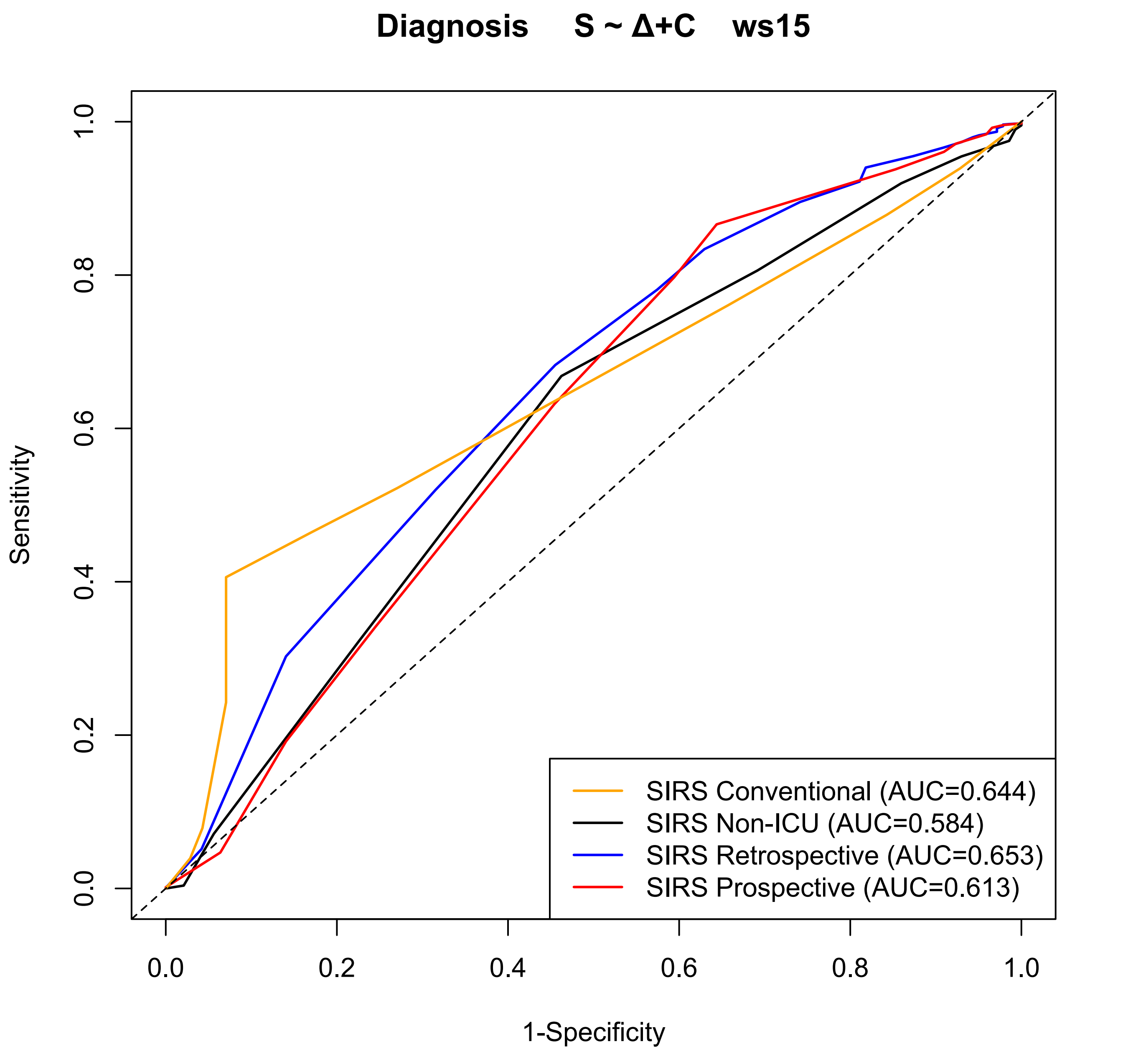

Diagnosis S ~ Δ+C ws15
Sensitivity
1-Specificity
0.0
0.2
0.4
0.6
0.8
1.0
0.0
0.2
0.4
0.6
0.8
1.0
SIRS Conventional (AUC=0.644)
SIRS Non-ICU (AUC=0.584)
SIRS Retrospective (AUC=0.653)
SIRS Prospective (AUC=0.613)

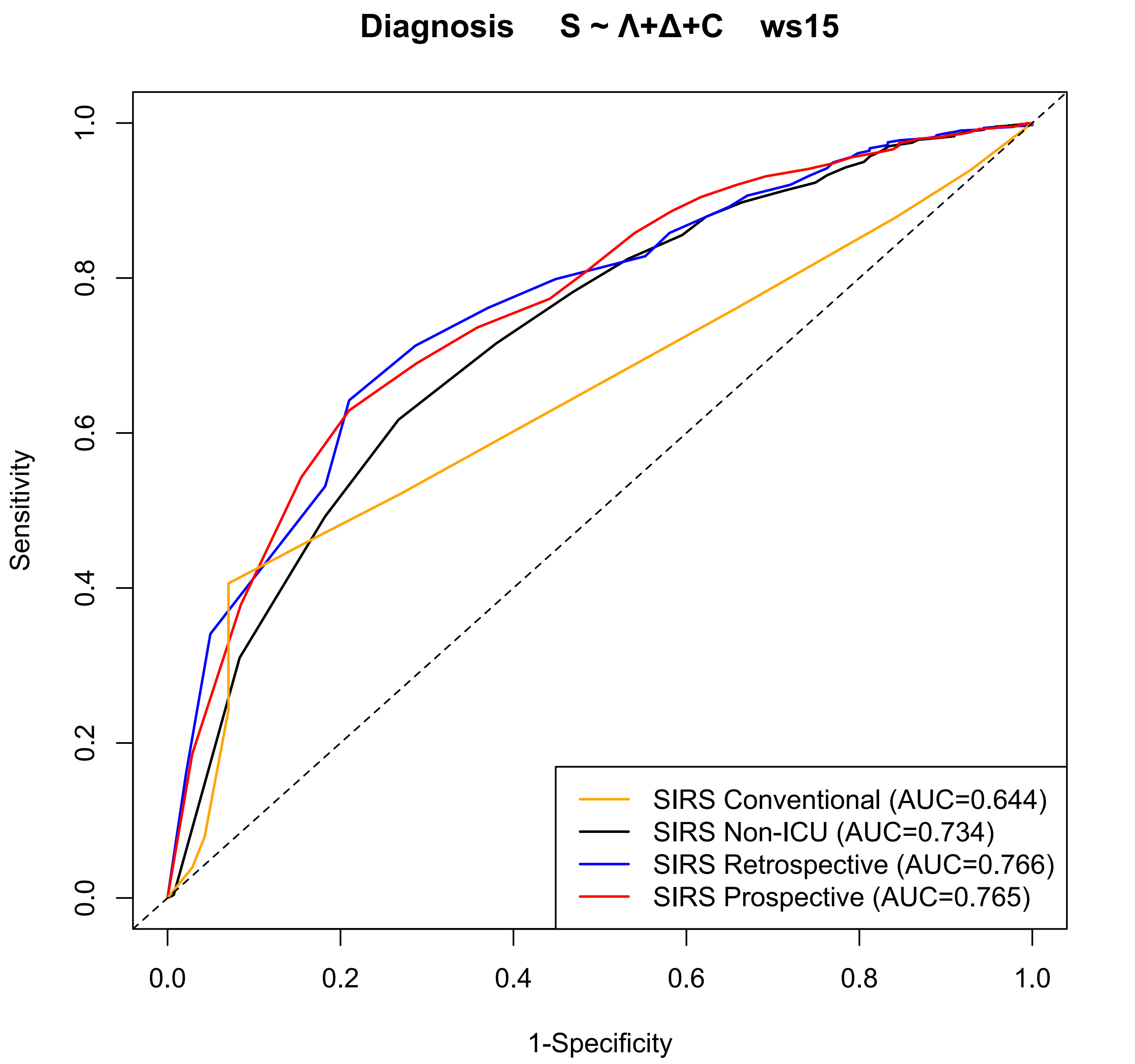

Diagnosis S ~ Λ+Δ+C ws15
Sensitivity
1-Specificity
0.0
0.2
0.4
0.6
0.8
1.0
SIRS Conventional (AUC=0.644)
SIRS Non-ICU (AUC=0.734)
SIRS Retrospective (AUC=0.766)
SIRS Prospective (AUC=0.765)

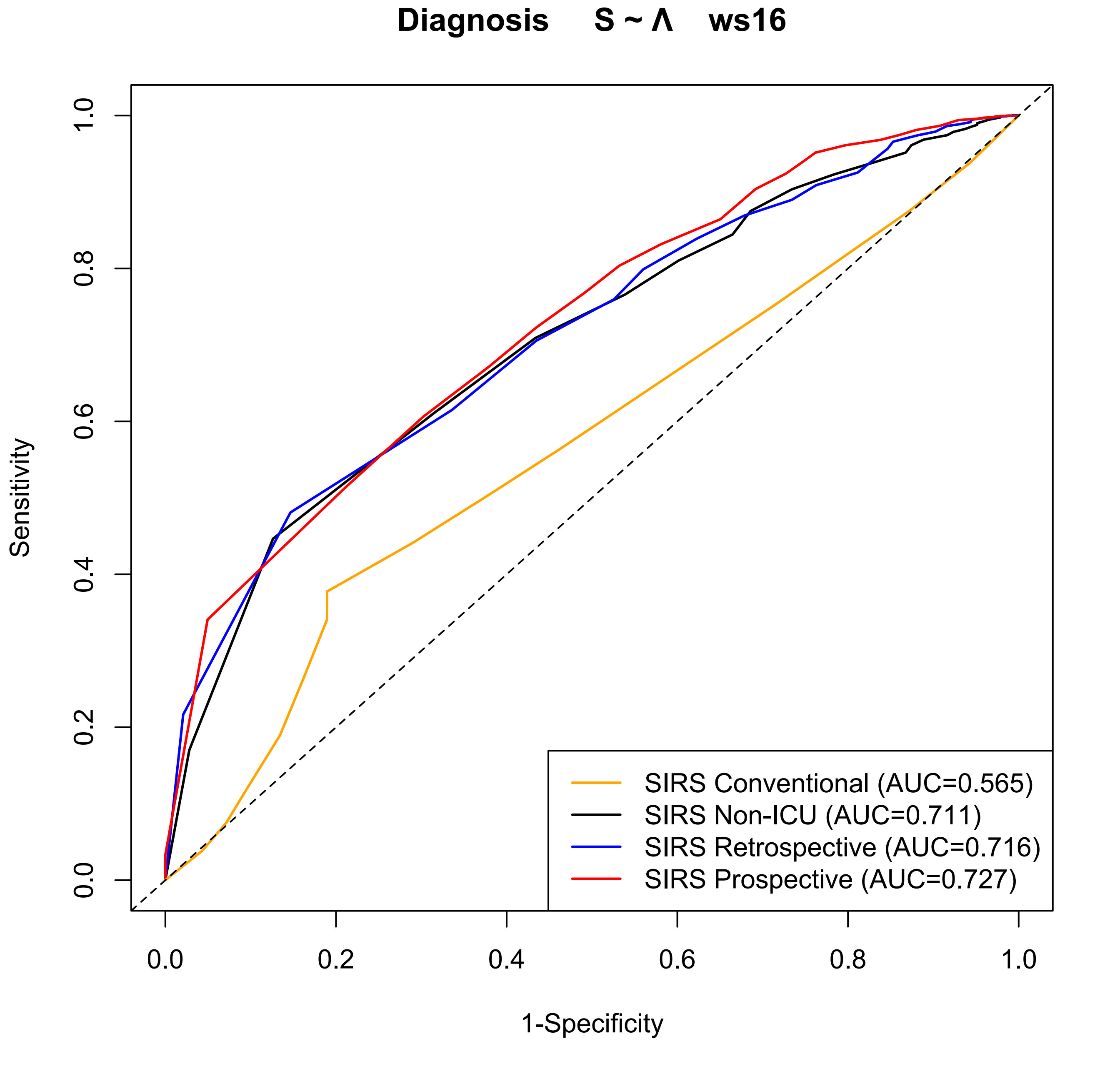

Diagnosis S ~ Λ ws16
Sensitivity
1-Specificity
0.0
0.2
0.4
0.6
0.8
1.0
0.0
0.2
0.4
0.6
0.8
1.0
SIRS Conventional (AUC=0.565)
SIRS Non-ICU (AUC=0.711)
SIRS Retrospective (AUC=0.716)
SIRS Prospective (AUC=0.727)

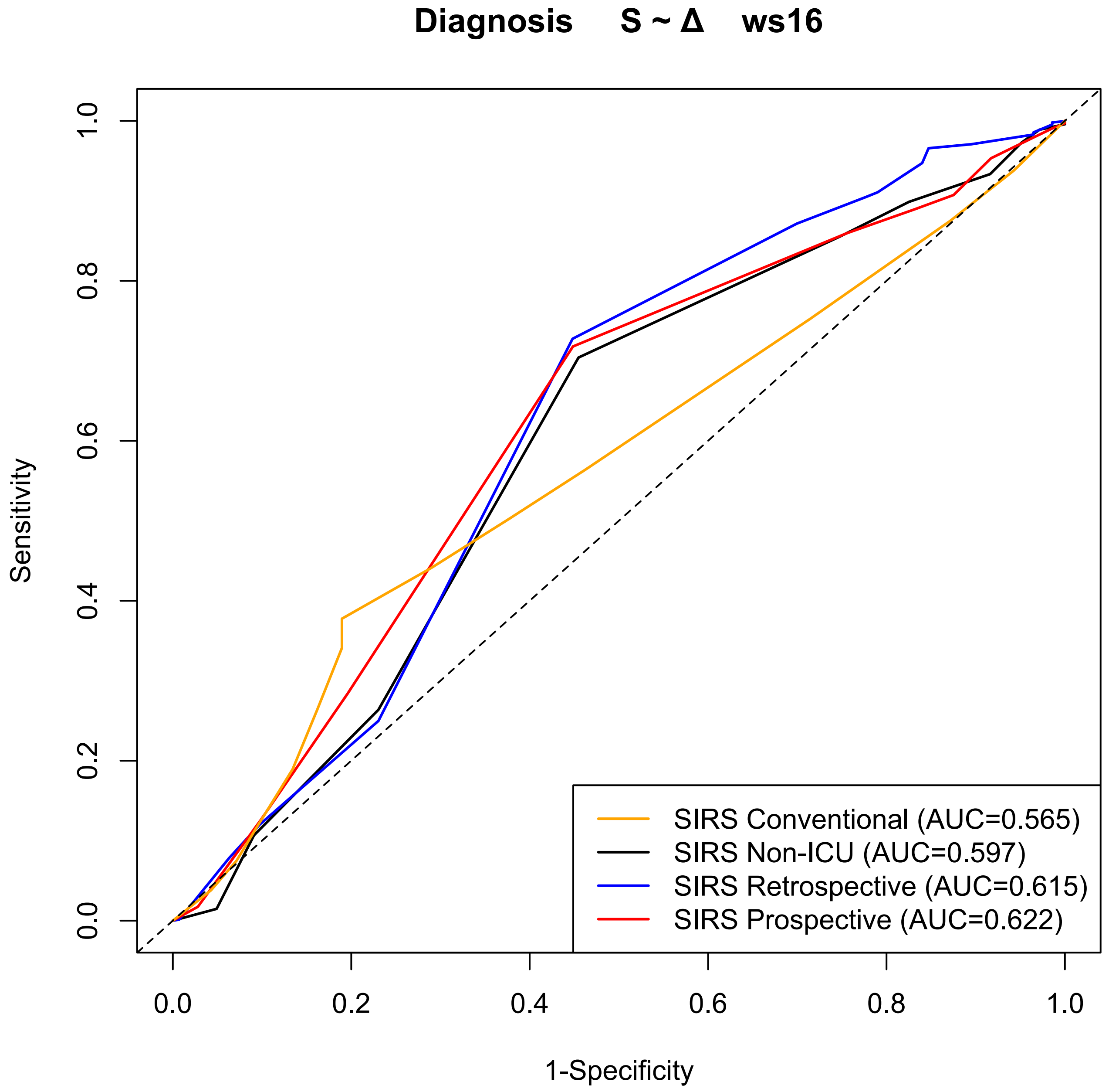

Diagnosis S ~ Δ ws16
Sensitivity
1-Specificity
0.0
0.2
0.4
0.6
0.8
1.0
SIRS Conventional (AUC=0.565)
SIRS Non-ICU (AUC=0.597)
SIRS Retrospective (AUC=0.615)
SIRS Prospective (AUC=0.622)

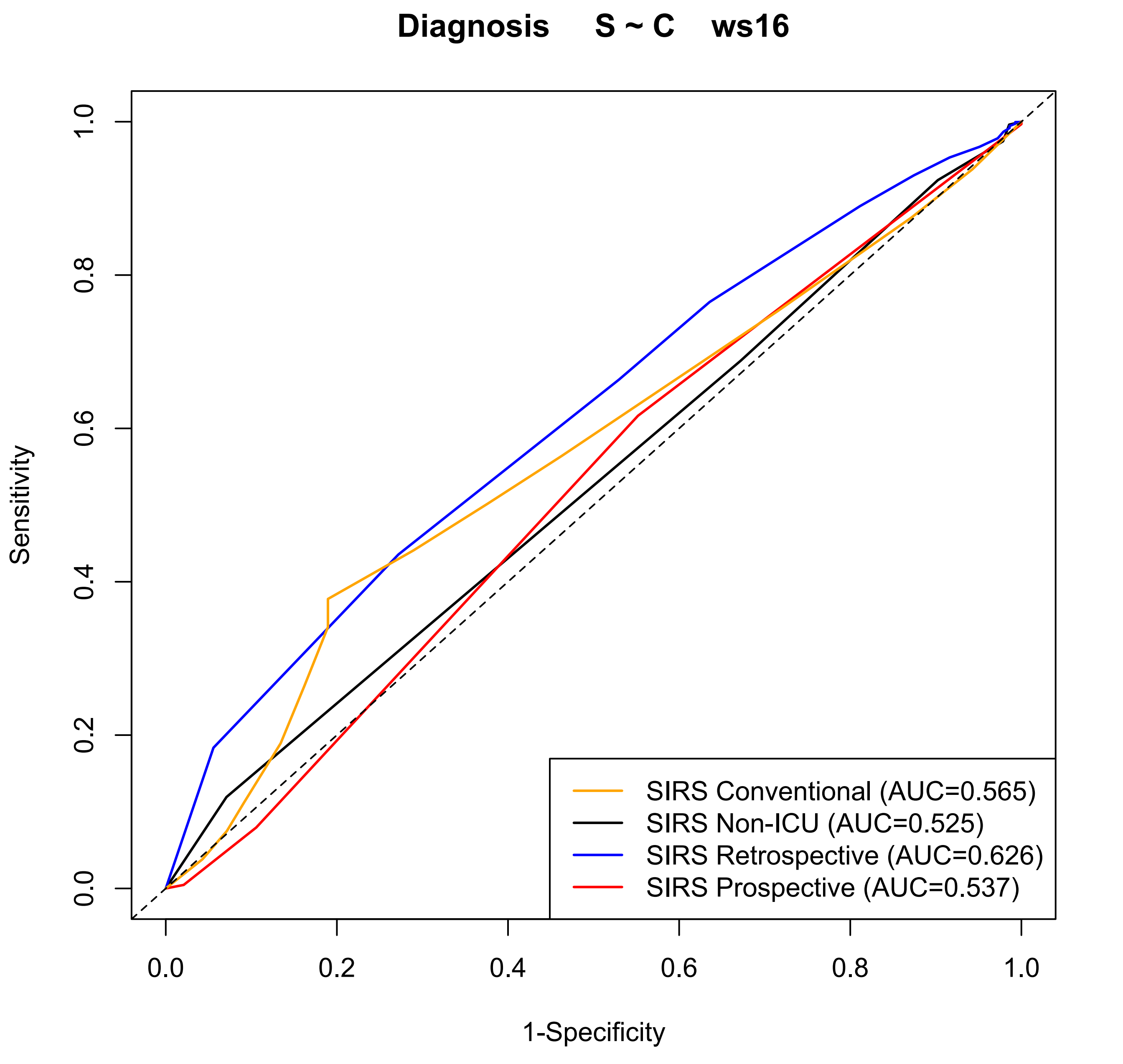
Diagnosis S ~ C ws16
Sensitivity
1-Specificity
0.0
0.2
0.4
0.6
0.8
1.0
SIRS Conventional (AUC=0.565)
SIRS Non-ICU (AUC=0.525)
SIRS Retrospective (AUC=0.626)
SIRS Prospective (AUC=0.537)

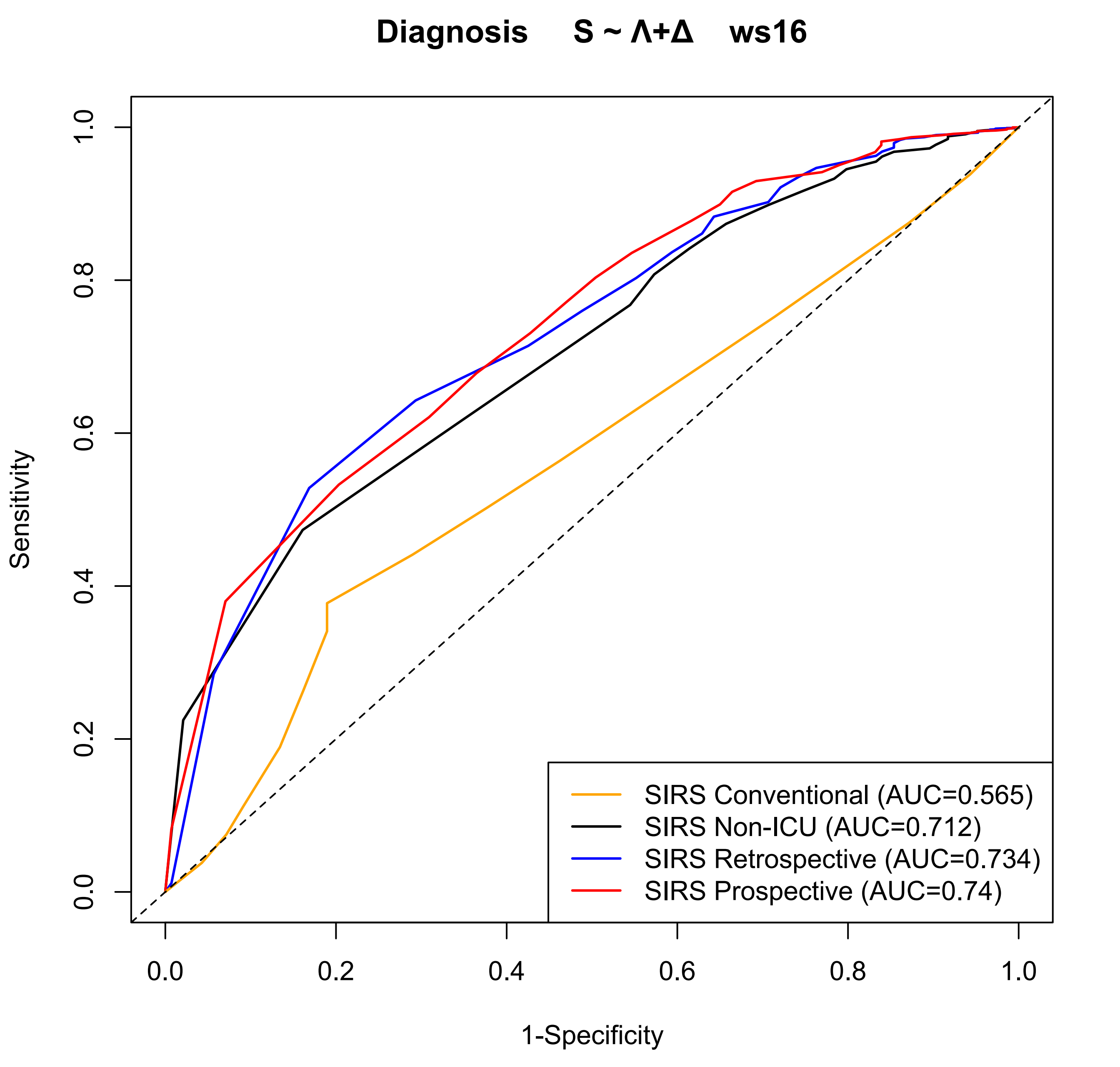
Diagnosis S ~ Λ+Δ ws16
Sensitivity
1-Specificity
0.0
0.2
0.4
0.6
0.8
1.0
SIRS Conventional (AUC=0.565)
SIRS Non-ICU (AUC=0.712)
SIRS Retrospective (AUC=0.734)
SIRS Prospective (AUC=0.74)

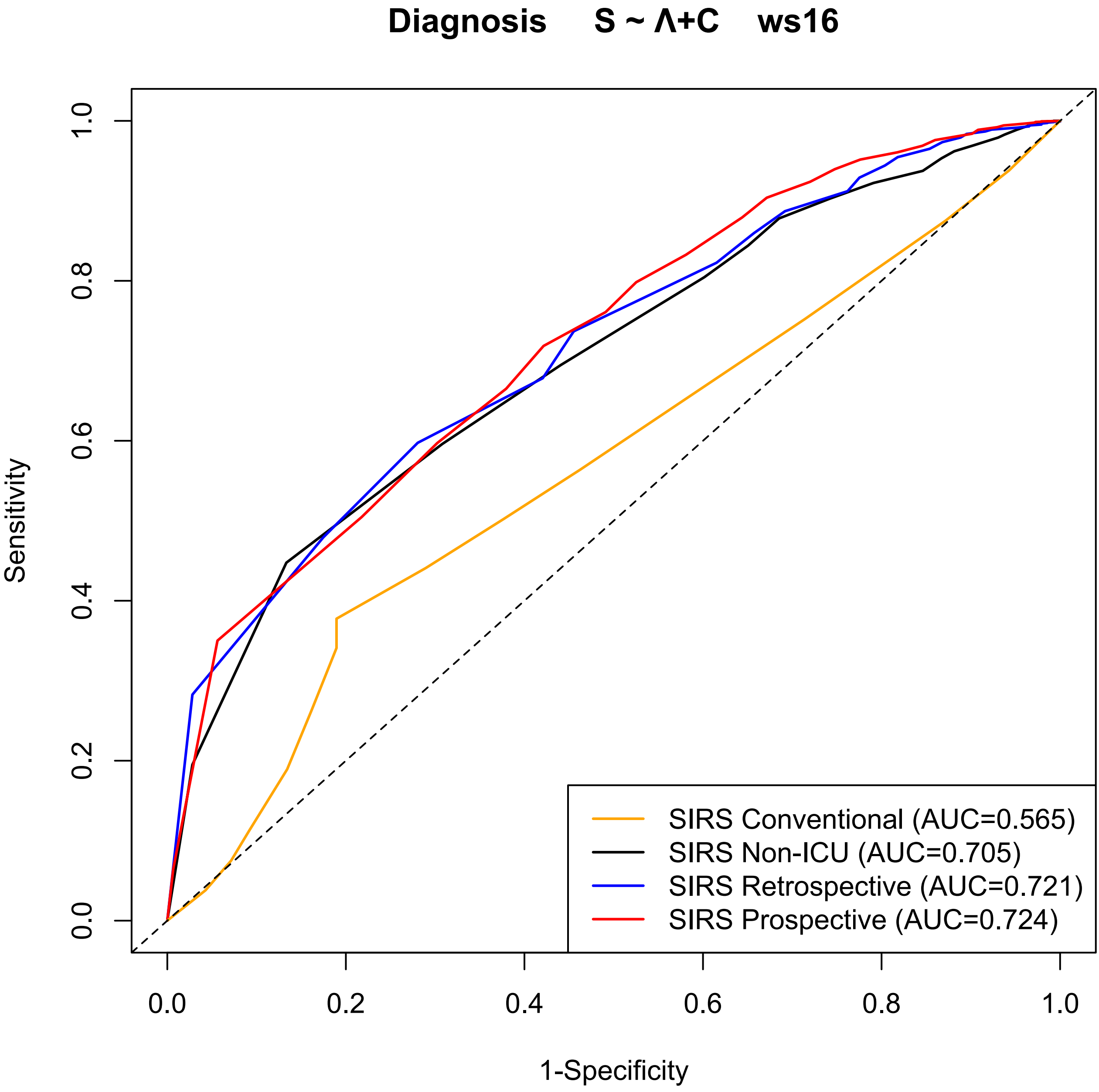

Diagnosis S ~ Λ+C ws16
Sensitivity
1-Specificity
0.0
0.2
0.4
0.6
0.8
1.0
SIRS Conventional (AUC=0.565)
SIRS Non-ICU (AUC=0.705)
SIRS Retrospective (AUC=0.721)
SIRS Prospective (AUC=0.724)

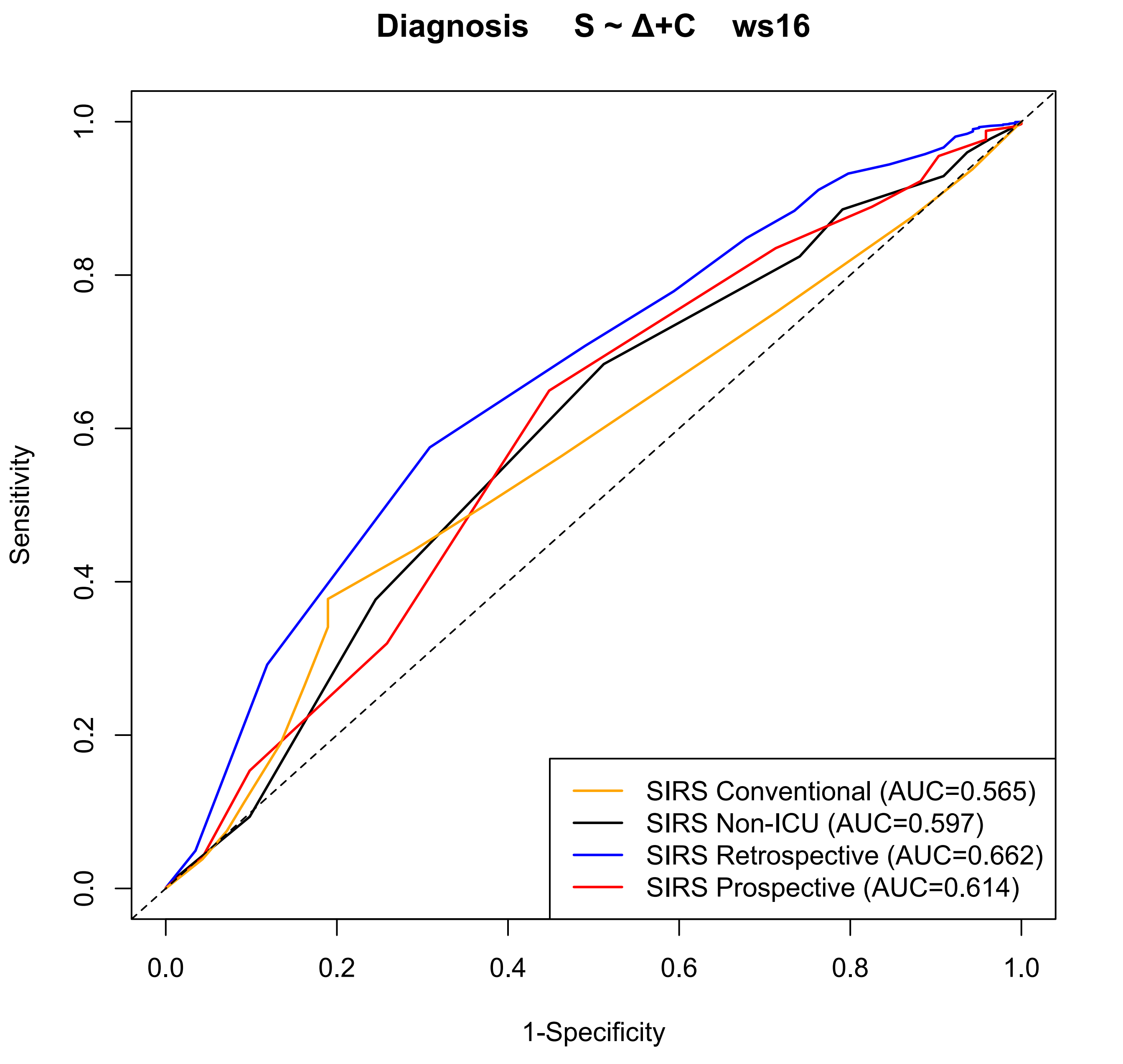
Diagnosis S ~ Δ+C ws16
Sensitivity
1-Specificity
0.0
0.2
0.4
0.6
0.8
1.0
SIRS Conventional (AUC=0.565)
SIRS Non-ICU (AUC=0.597)
SIRS Retrospective (AUC=0.662)
SIRS Prospective (AUC=0.614)

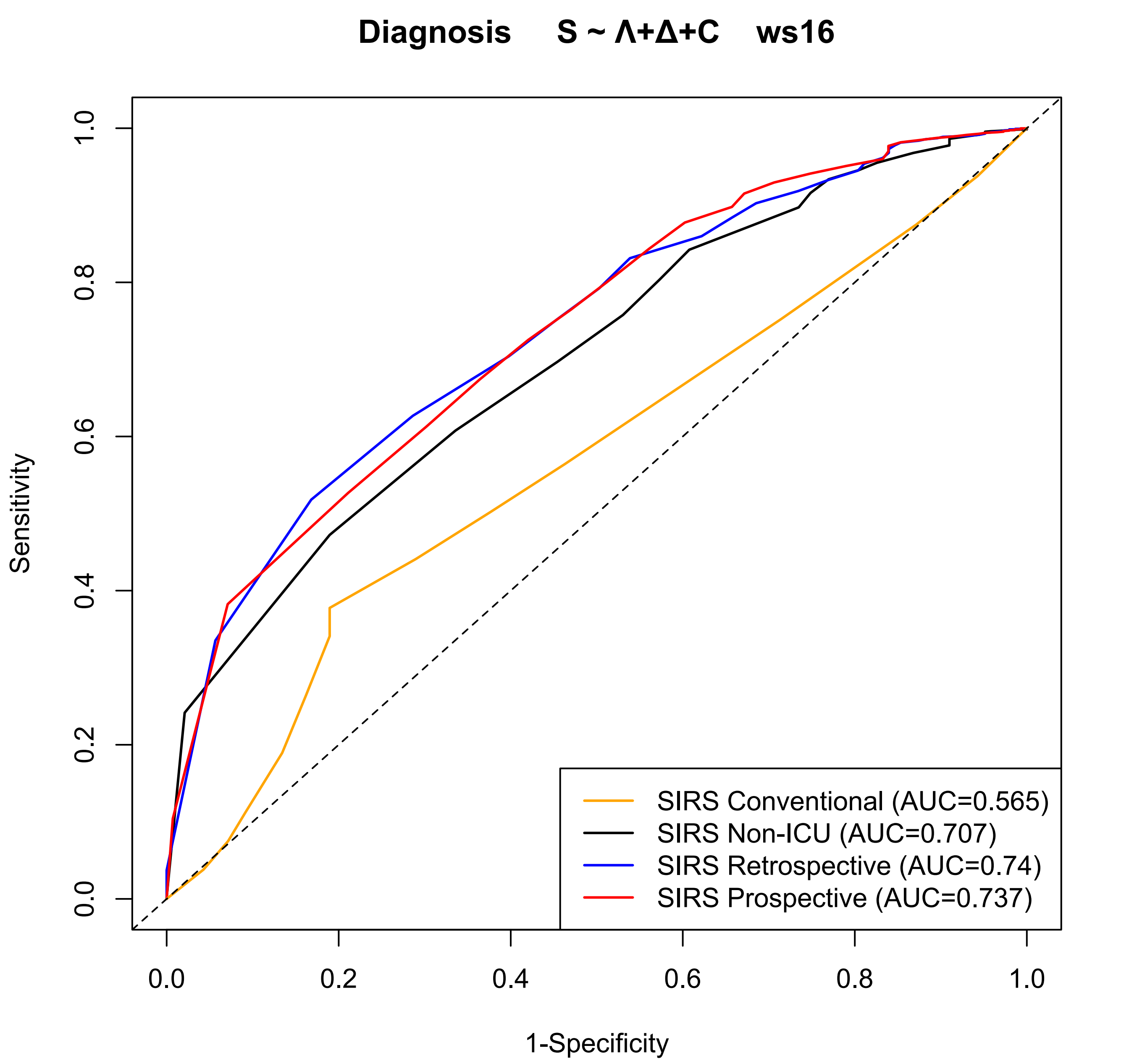

Diagnosis S ~ Λ+Δ+C ws16
Sensitivity
1-Specificity
0.0
0.2
0.4
0.6
0.8
1.0
SIRS Conventional (AUC=0.565)
SIRS Non-ICU (AUC=0.707)
SIRS Retrospective (AUC=0.74)
SIRS Prospective (AUC=0.737)

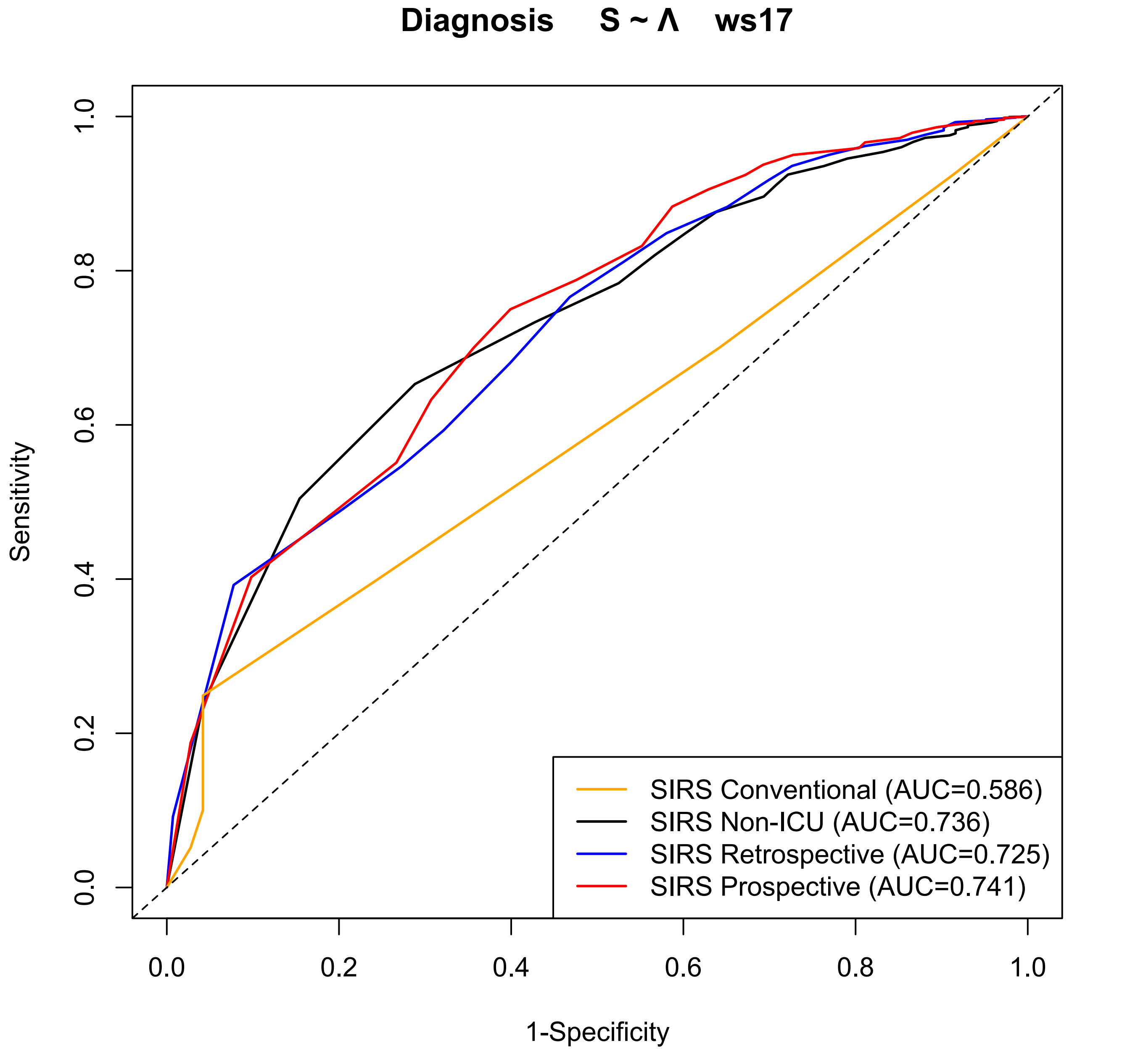
Diagnosis S ~ Λ ws17
Sensitivity
1-Specificity
0.0
0.2
0.4
0.6
0.8
1.0
SIRS Conventional (AUC=0.586)
SIRS Non-ICU (AUC=0.736)
SIRS Retrospective (AUC=0.725)
SIRS Prospective (AUC=0.741)

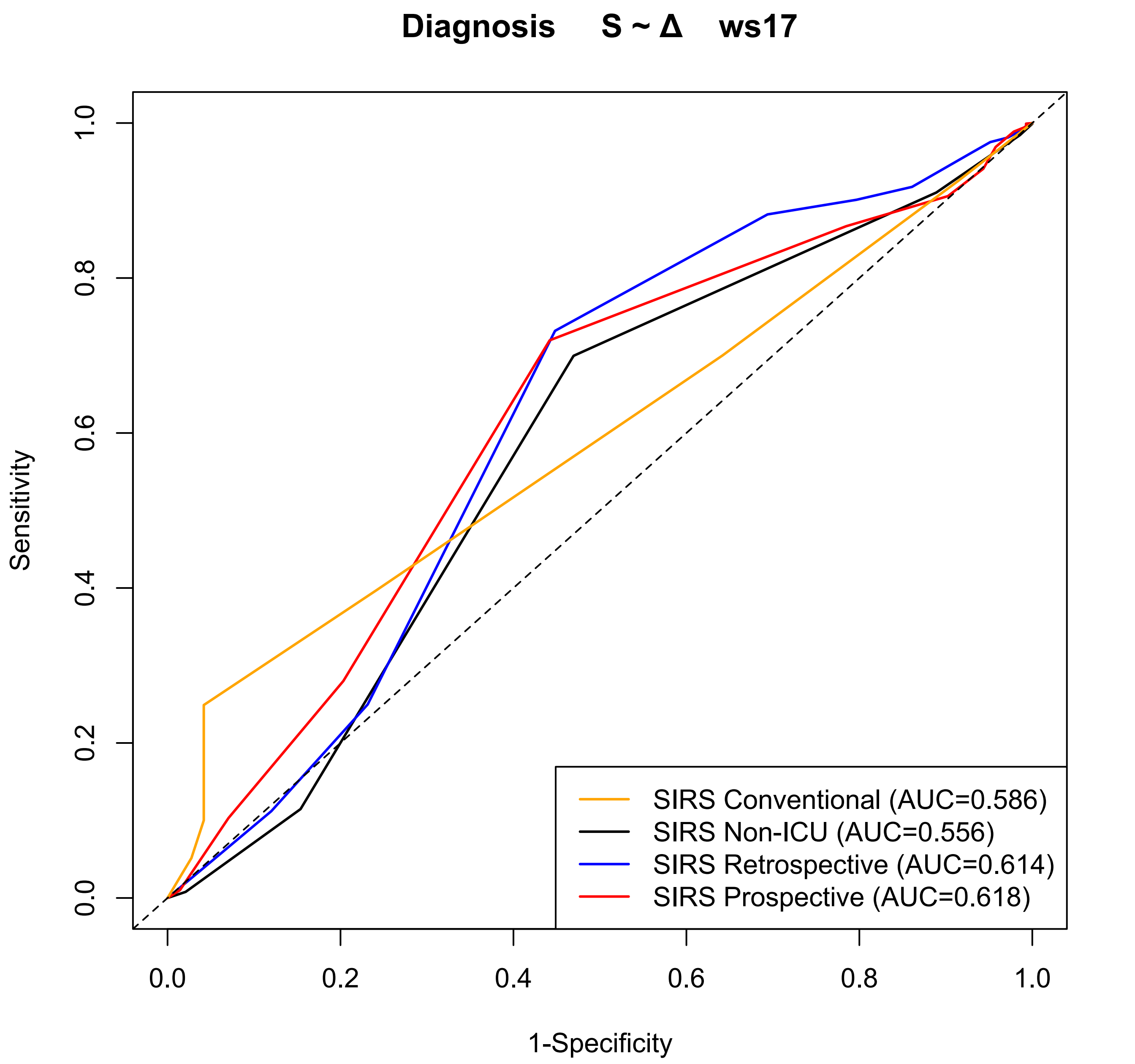
Diagnosis S ~ Δ ws17
Sensitivity
1-Specificity
0.0
0.2
0.4
0.6
0.8
1.0
SIRS Conventional (AUC=0.586)
SIRS Non-ICU (AUC=0.556)
SIRS Retrospective (AUC=0.614)
SIRS Prospective (AUC=0.618)

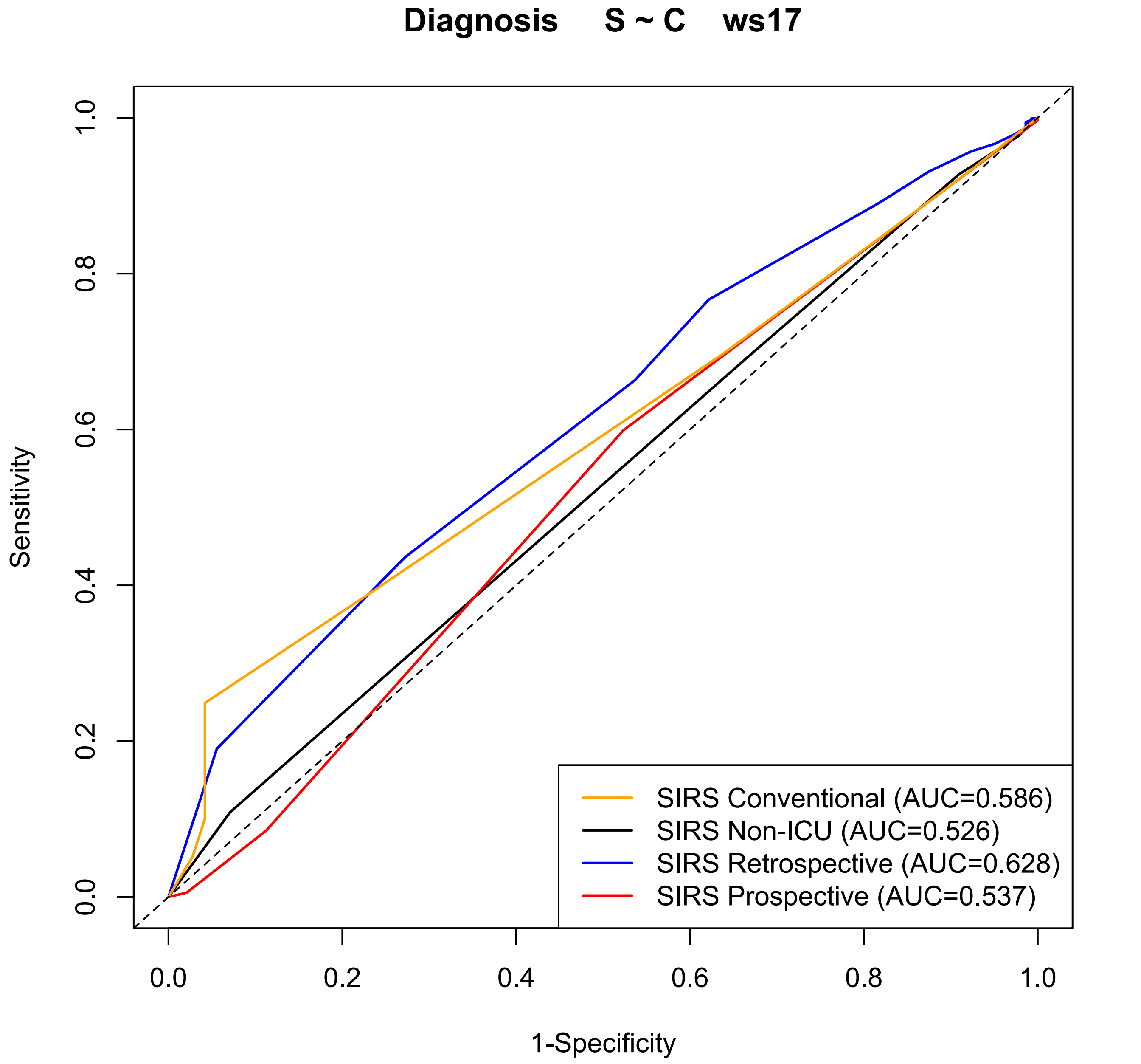

Diagnosis S ~ C ws17
Sensitivity
1-Specificity
0.0
0.2
0.4
0.6
0.8
1.0
SIRS Conventional (AUC=0.586)
SIRS Non-ICU (AUC=0.526)
SIRS Retrospective (AUC=0.628)
SIRS Prospective (AUC=0.537)

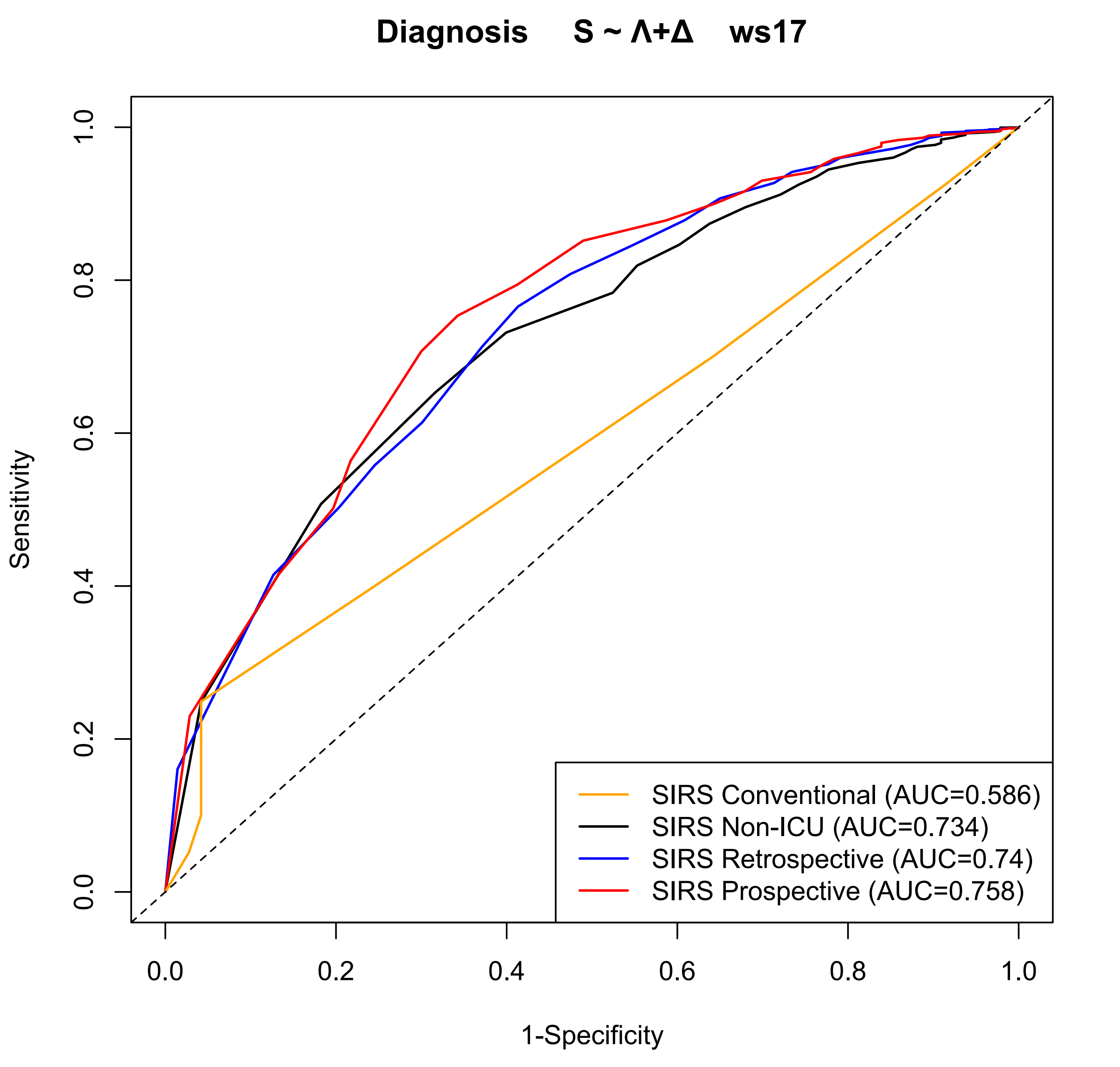

Diagnosis S ~ Λ+Δ ws17
Sensitivity
1-Specificity
0.0
0.2
0.4
0.6
0.8
1.0
SIRS Conventional (AUC=0.586)
SIRS Non-ICU (AUC=0.734)
SIRS Retrospective (AUC=0.74)
SIRS Prospective (AUC=0.758)

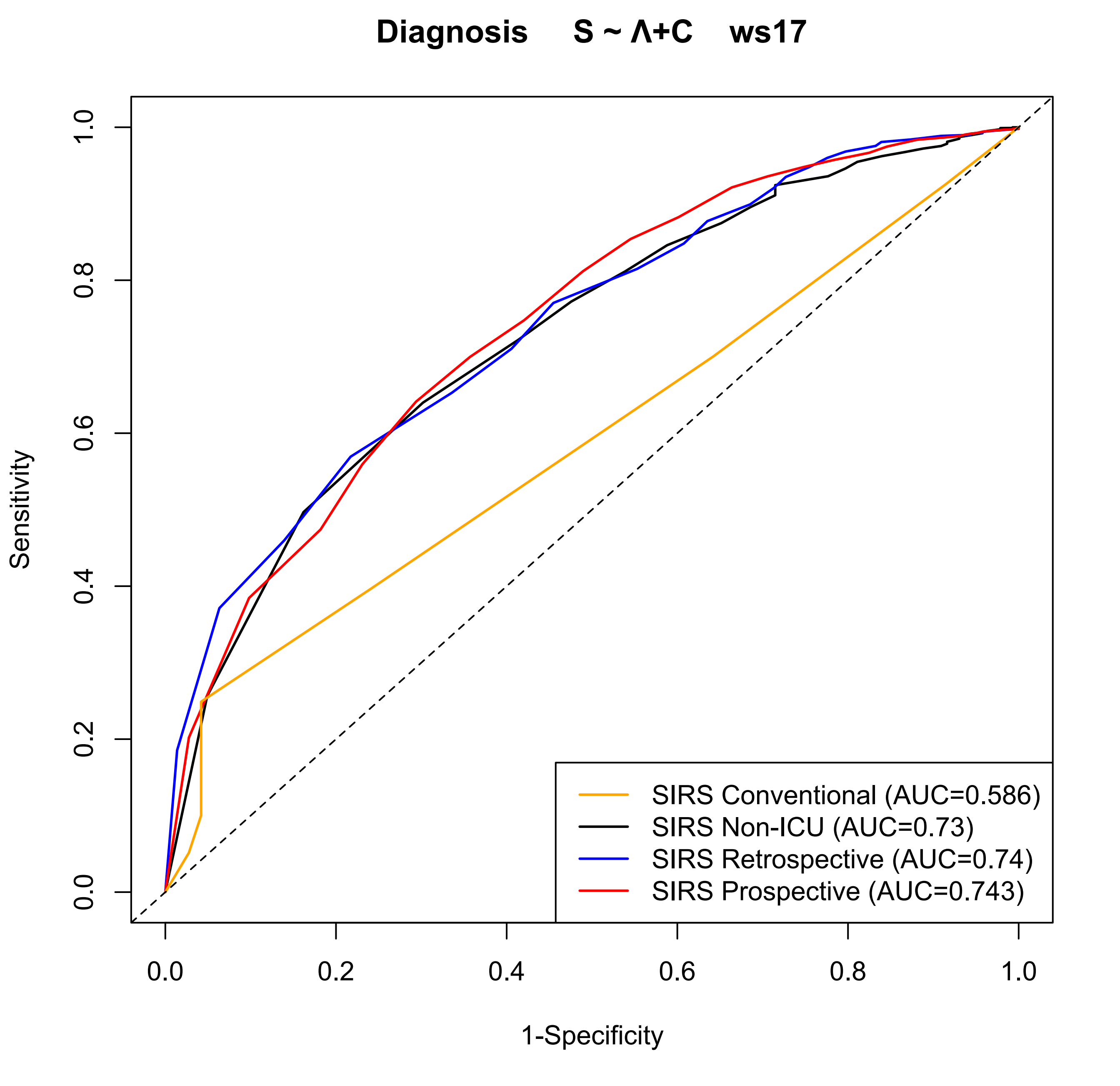
Diagnosis S ~ Λ+C ws17
Sensitivity
1-Specificity
0.0
0.2
0.4
0.6
0.8
1.0
SIRS Conventional (AUC=0.586)
SIRS Non-ICU (AUC=0.73)
SIRS Retrospective (AUC=0.74)
SIRS Prospective (AUC=0.743)

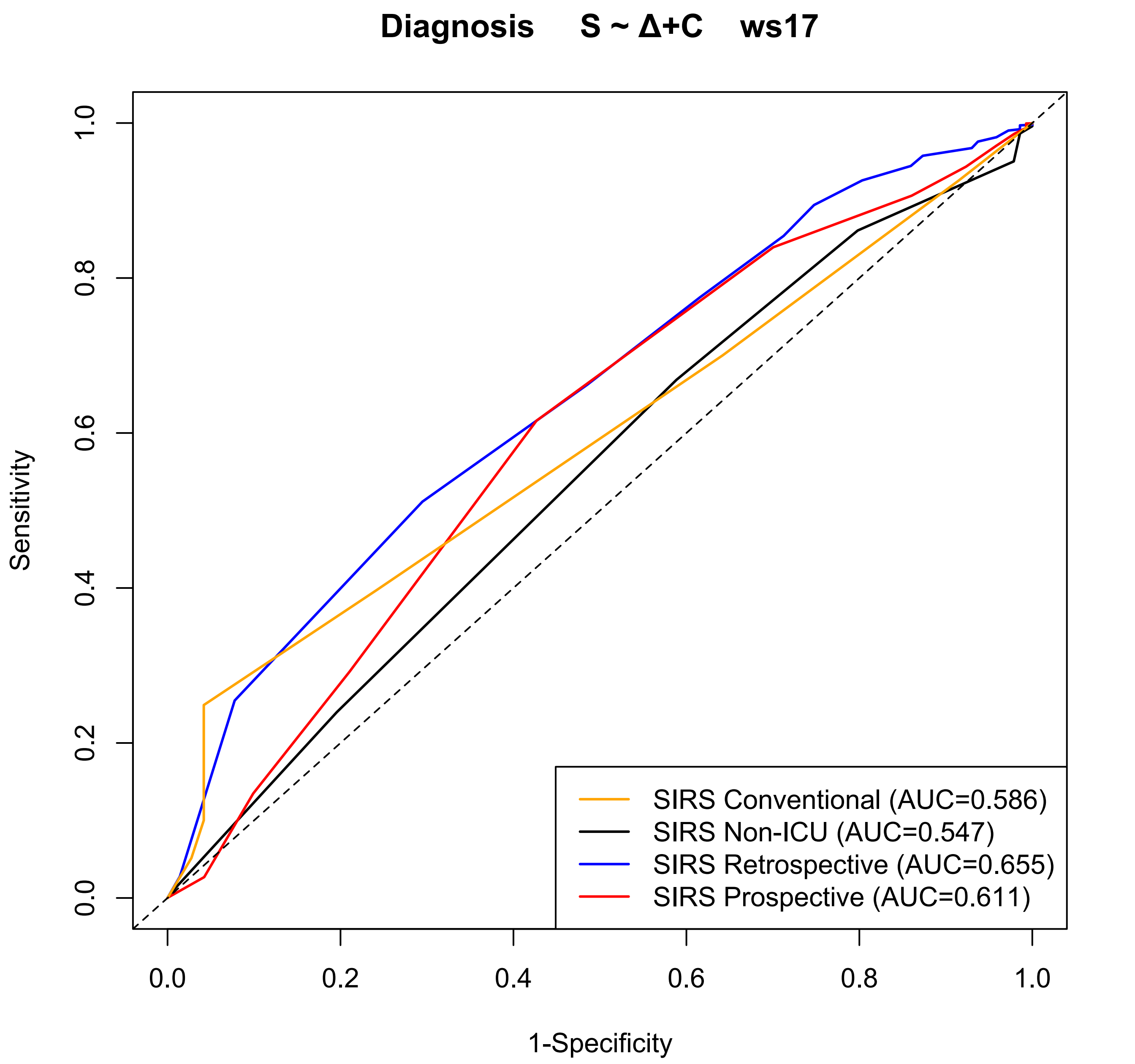
Diagnosis S ~ Δ+C ws17
Sensitivity
1-Specificity
0.0
0.2
0.4
0.6
0.8
1.0
SIRS Conventional (AUC=0.586)
SIRS Non-ICU (AUC=0.547)
SIRS Retrospective (AUC=0.655)
SIRS Prospective (AUC=0.611)

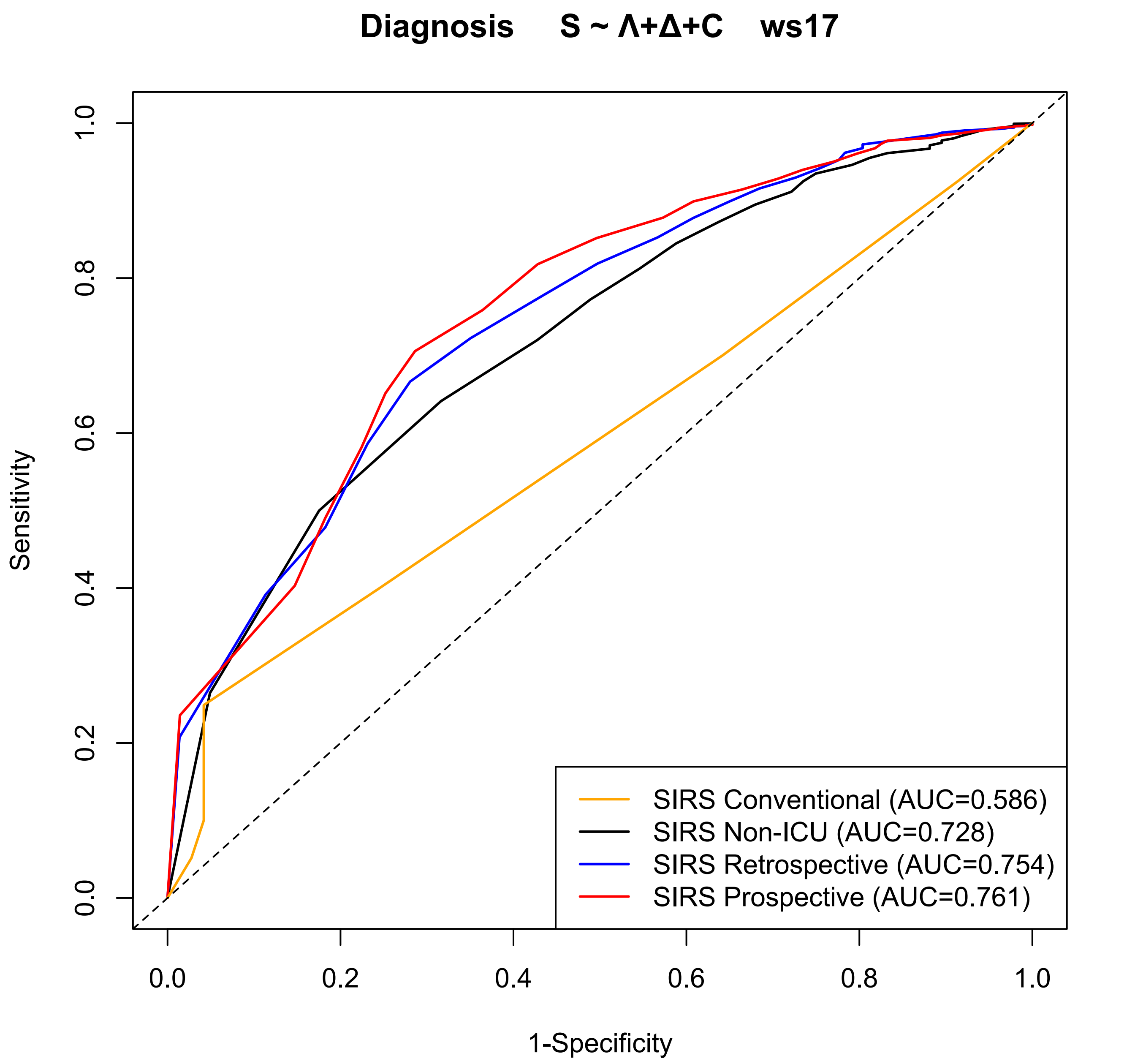
Diagnosis S ~ Λ+Δ+C ws17
Sensitivity
1-Specificity
0.0
0.2
0.4
0.6
0.8
1.0
SIRS Conventional (AUC=0.586)
SIRS Non-ICU (AUC=0.728)
SIRS Retrospective (AUC=0.754)
SIRS Prospective (AUC=0.761)

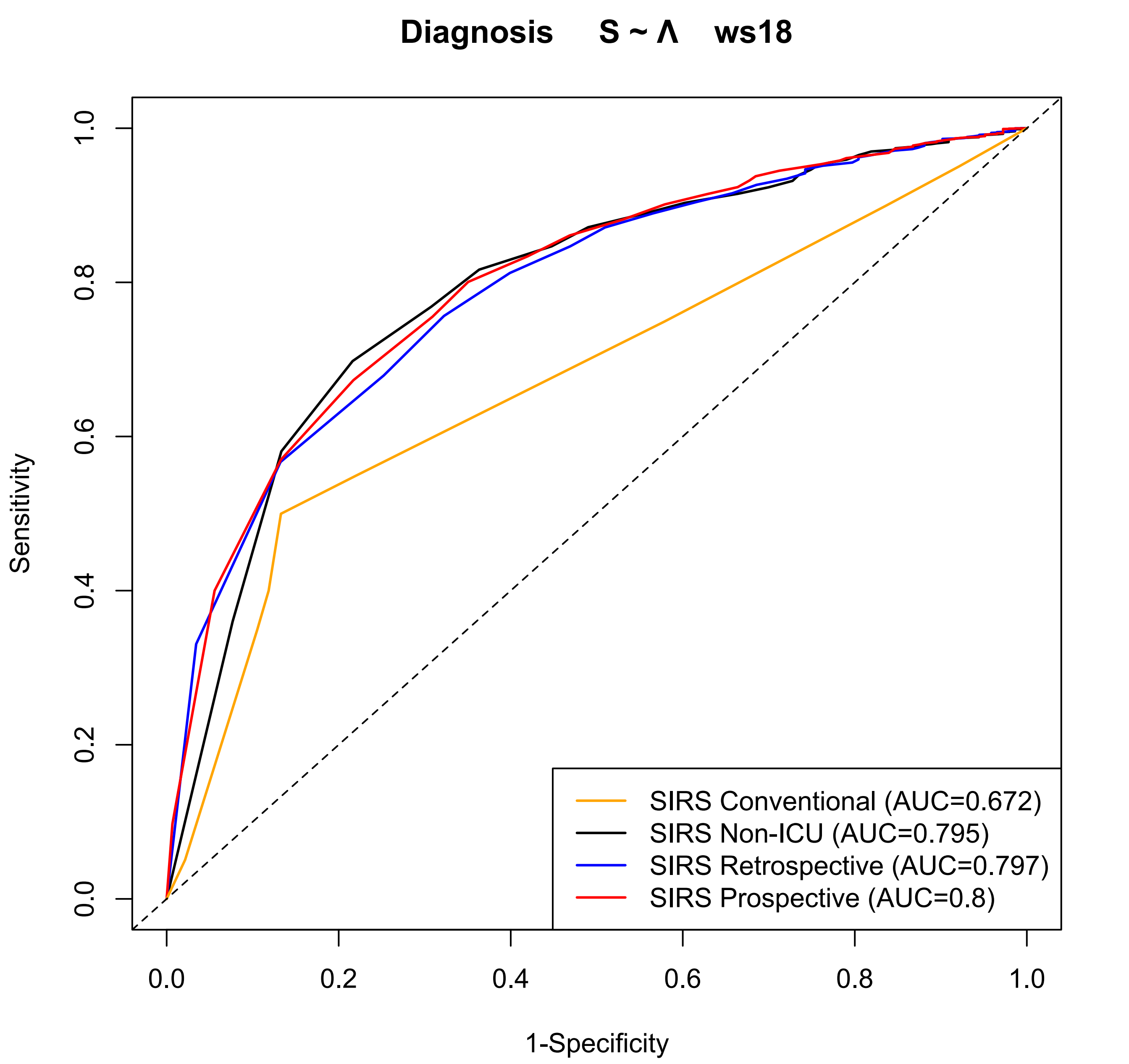
Diagnosis S ~ Λ ws18
Sensitivity
1-Specificity
0.0
0.2
0.4
0.6
0.8
1.0
0.0
0.2
0.4
0.6
0.8
1.0
SIRS Conventional (AUC=0.672)
SIRS Non-ICU (AUC=0.795)
SIRS Retrospective (AUC=0.797)
SIRS Prospective (AUC=0.8)

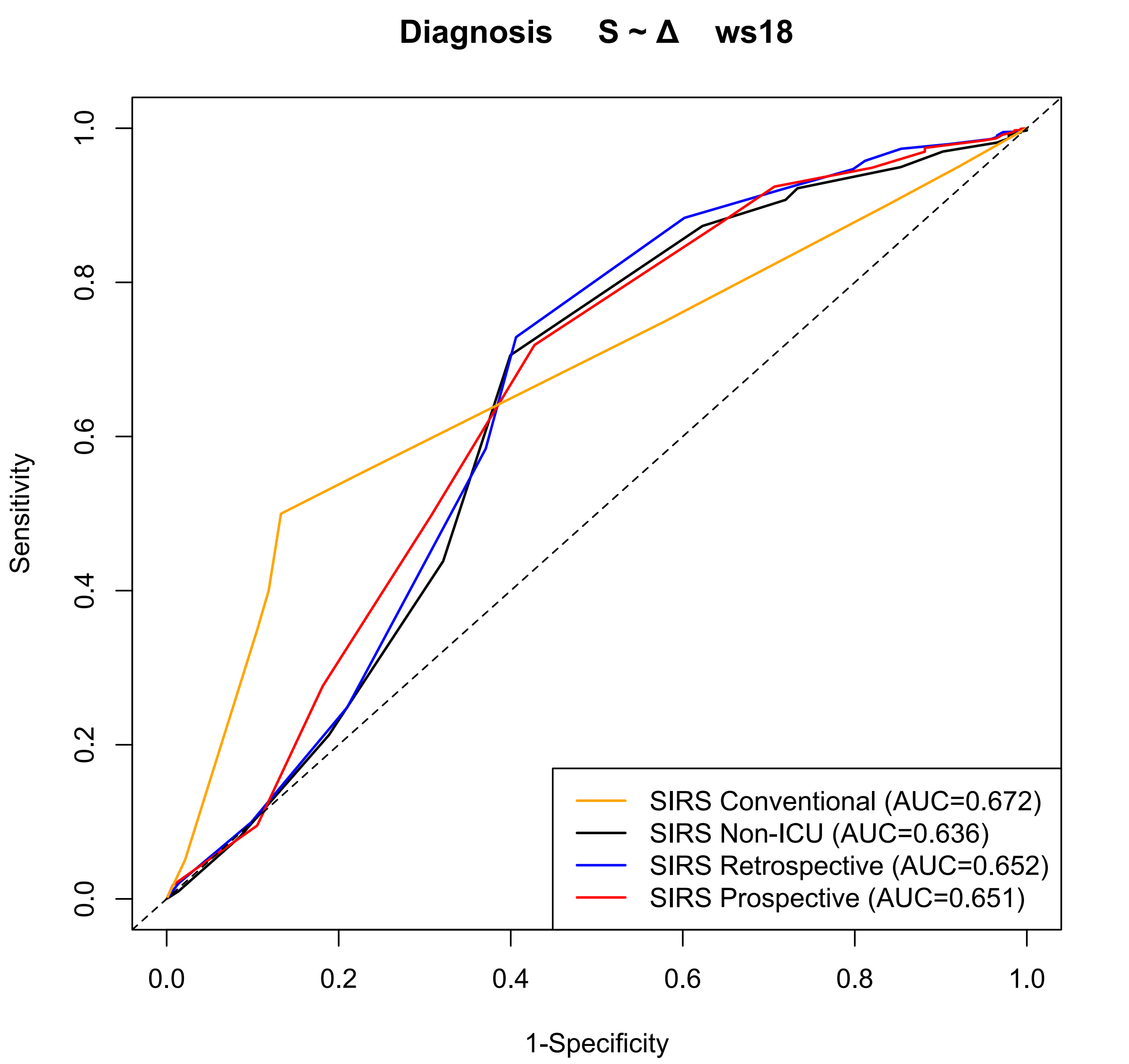

Diagnosis S ~ Δ ws18
Sensitivity
1-Specificity
0.0
0.2
0.4
0.6
0.8
1.0
SIRS Conventional (AUC=0.672)
SIRS Non-ICU (AUC=0.636)
SIRS Retrospective (AUC=0.652)
SIRS Prospective (AUC=0.651)

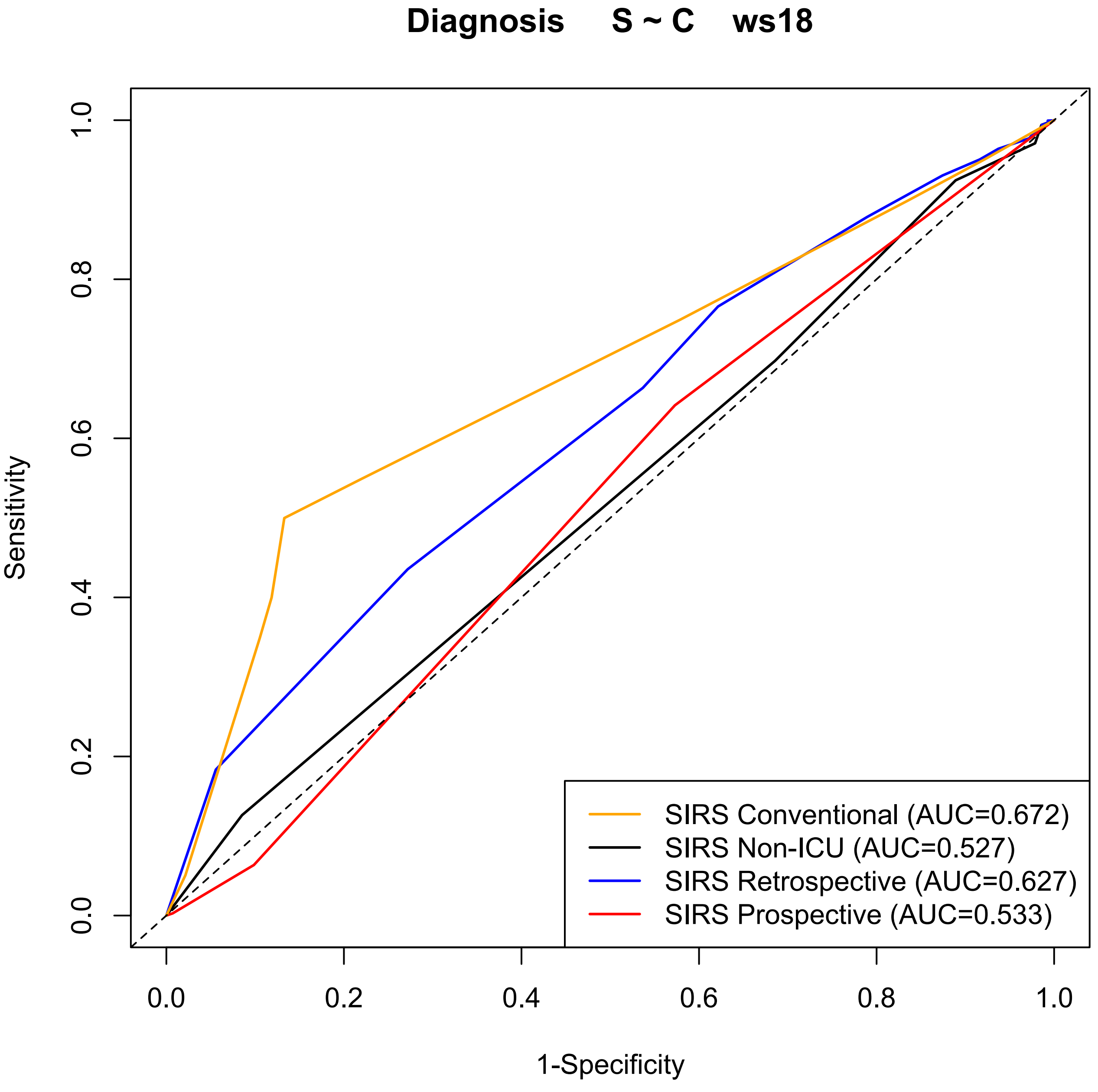

Diagnosis S ~ C ws18
Sensitivity
1-Specificity
0.0
0.2
0.4
0.6
0.8
1.0
SIRS Conventional (AUC=0.672)
SIRS Non-ICU (AUC=0.527)
SIRS Retrospective (AUC=0.627)
SIRS Prospective (AUC=0.533)

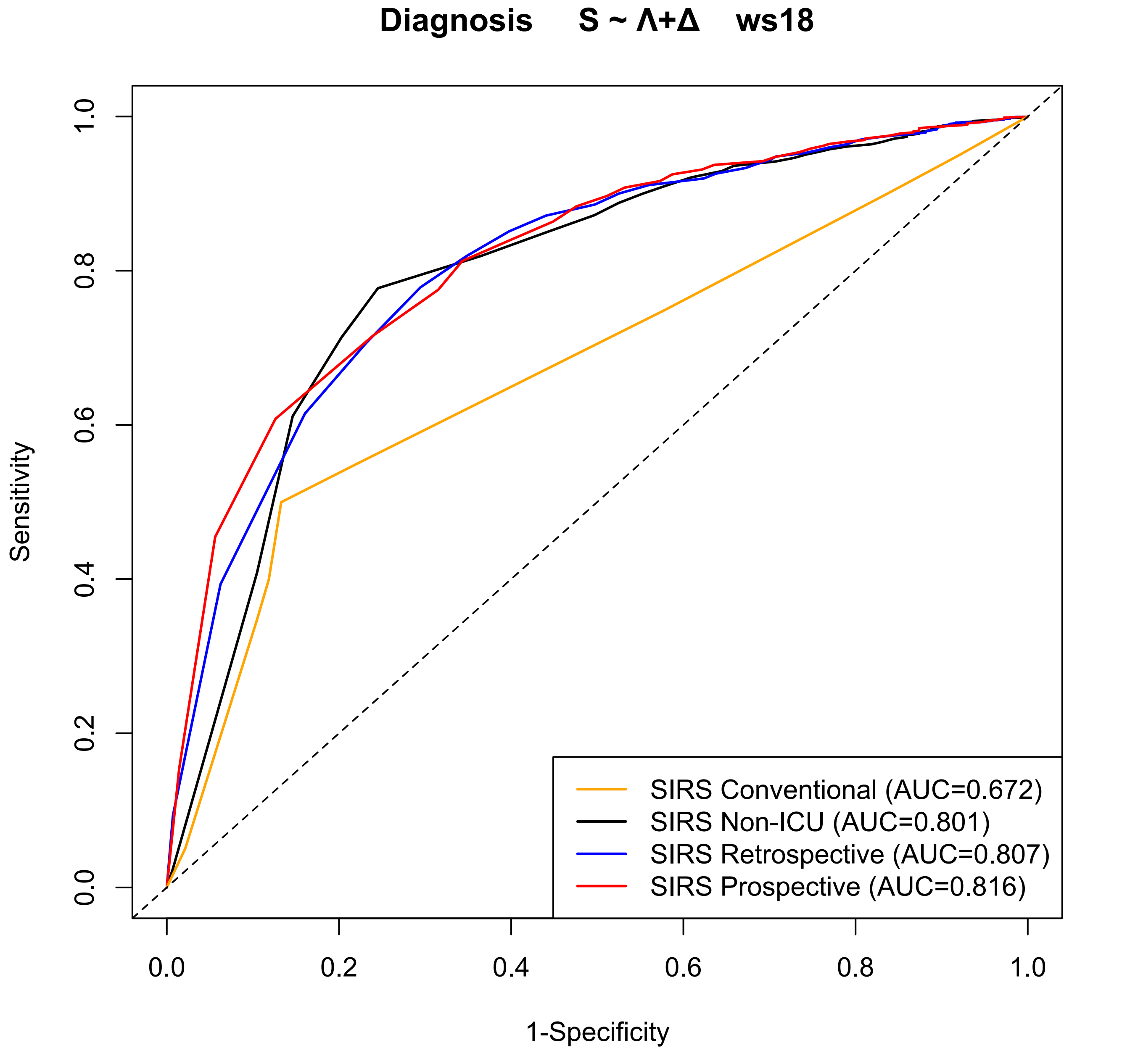

Diagnosis S ~ Λ+Δ ws18
Sensitivity
1-Specificity
0.0
0.2
0.4
0.6
0.8
1.0
SIRS Conventional (AUC=0.672)
SIRS Non-ICU (AUC=0.801)
SIRS Retrospective (AUC=0.807)
SIRS Prospective (AUC=0.816)

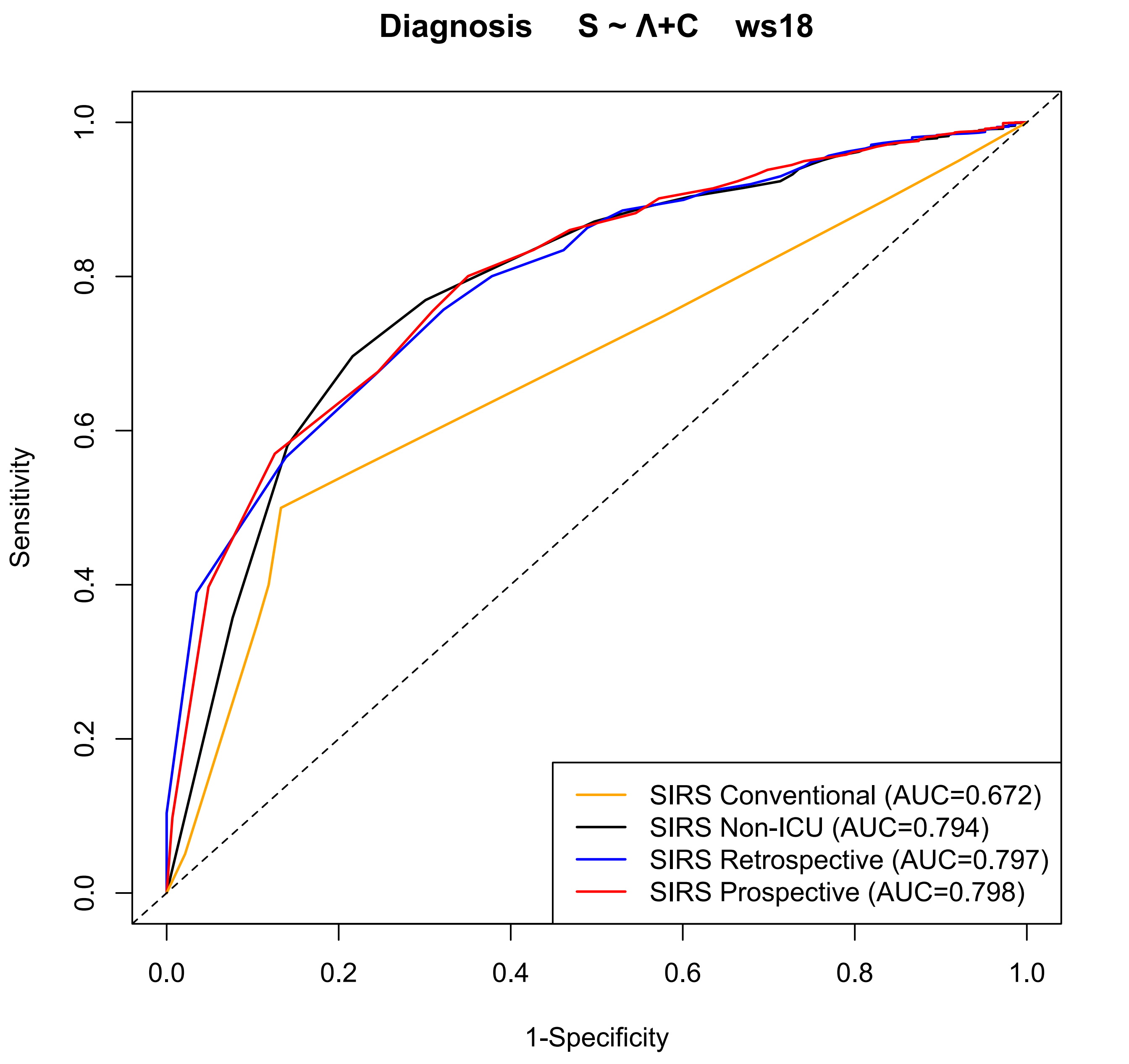

Diagnosis S ~ Λ+C ws18
Sensitivity
1-Specificity
0.0
0.2
0.4
0.6
0.8
1.0
SIRS Conventional (AUC=0.672)
SIRS Non-ICU (AUC=0.794)
SIRS Retrospective (AUC=0.797)
SIRS Prospective (AUC=0.798)

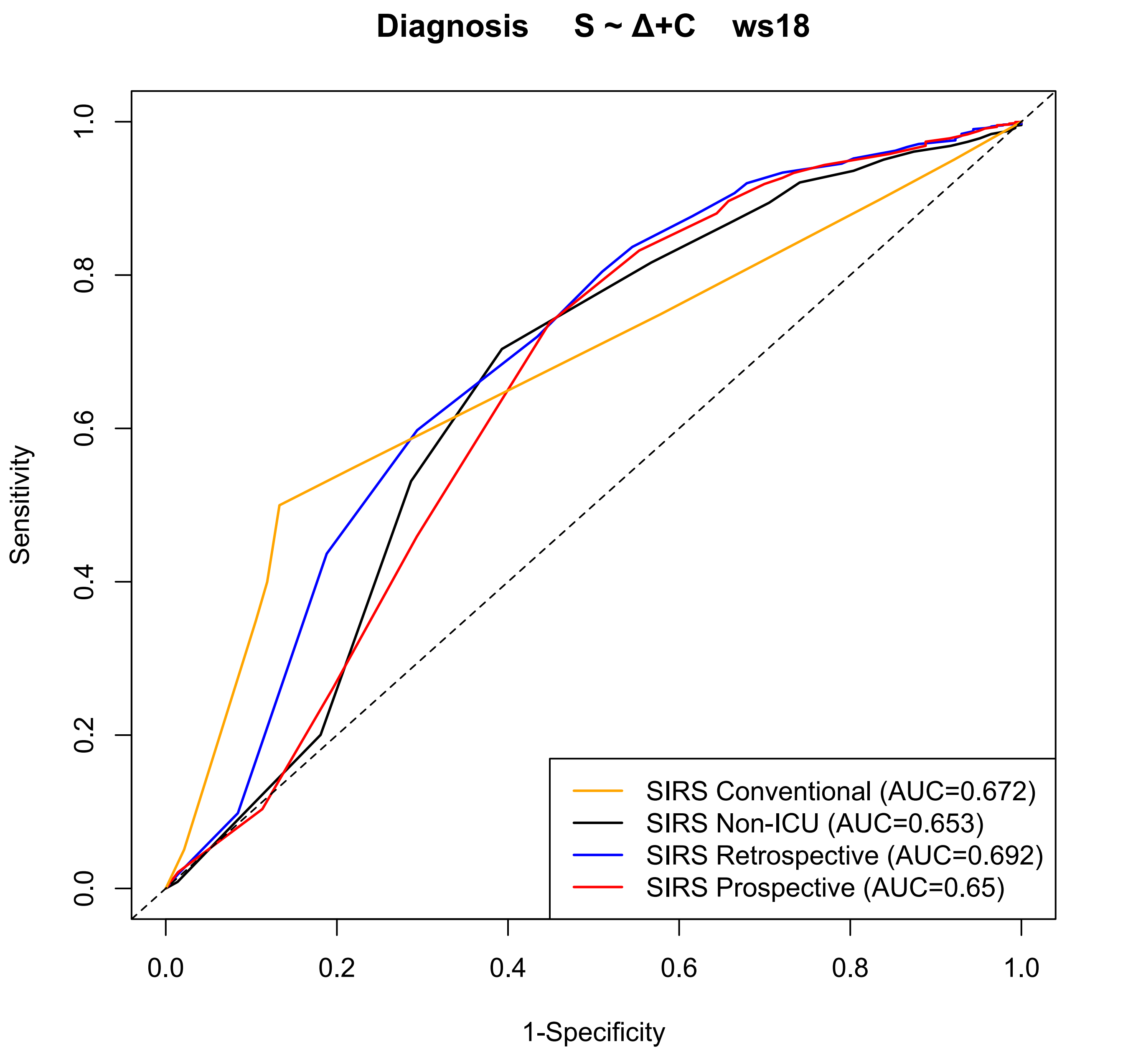
Diagnosis S ~ Δ+C ws18
Sensitivity
1-Specificity
0.0
0.2
0.4
0.6
0.8
1.0
SIRS Conventional (AUC=0.672)
SIRS Non-ICU (AUC=0.653)
SIRS Retrospective (AUC=0.692)
SIRS Prospective (AUC=0.65)

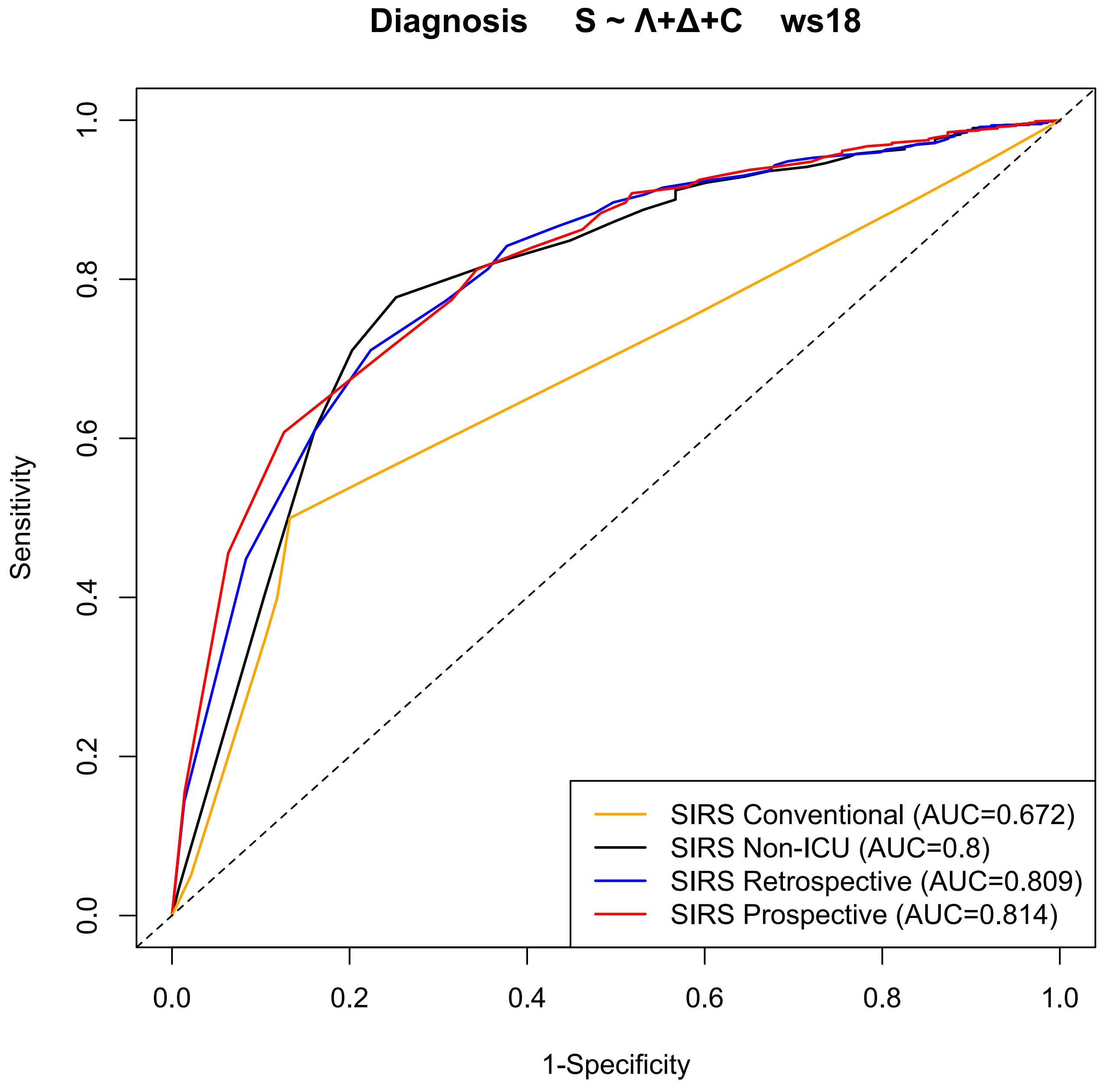

Diagnosis S ~ Λ+Δ+C ws18
Sensitivity
1-Specificity
0.0
0.2
0.4
0.6
0.8
1.0
SIRS Conventional (AUC=0.672)
SIRS Non-ICU (AUC=0.8)
SIRS Retrospective (AUC=0.809)
SIRS Prospective (AUC=0.814)

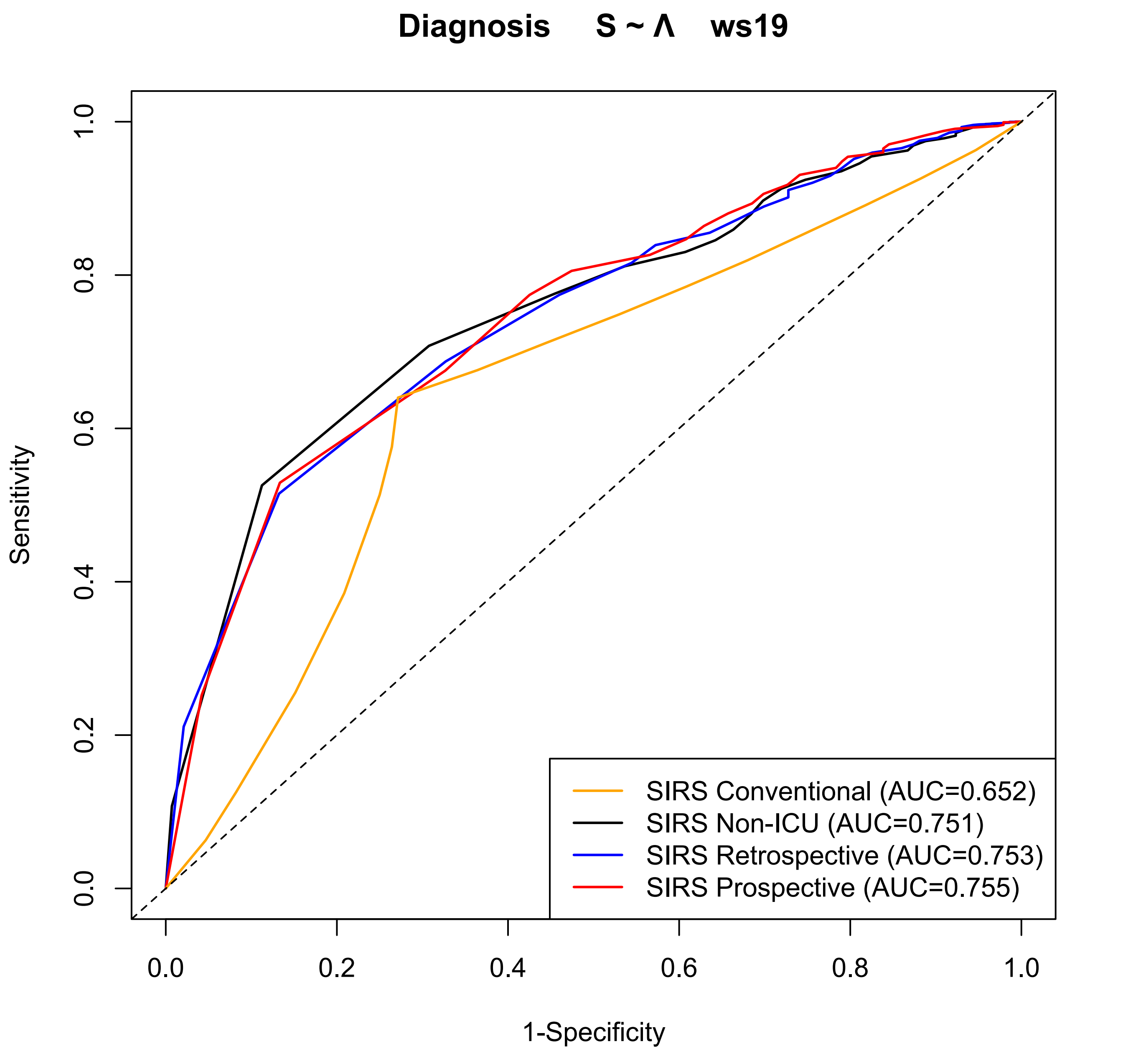
Diagnosis S ~ Λ ws19
Sensitivity
1-Specificity
0.0
0.2
0.4
0.6
0.8
1.0
SIRS Conventional (AUC=0.652)
SIRS Non-ICU (AUC=0.751)
SIRS Retrospective (AUC=0.753)
SIRS Prospective (AUC=0.755)

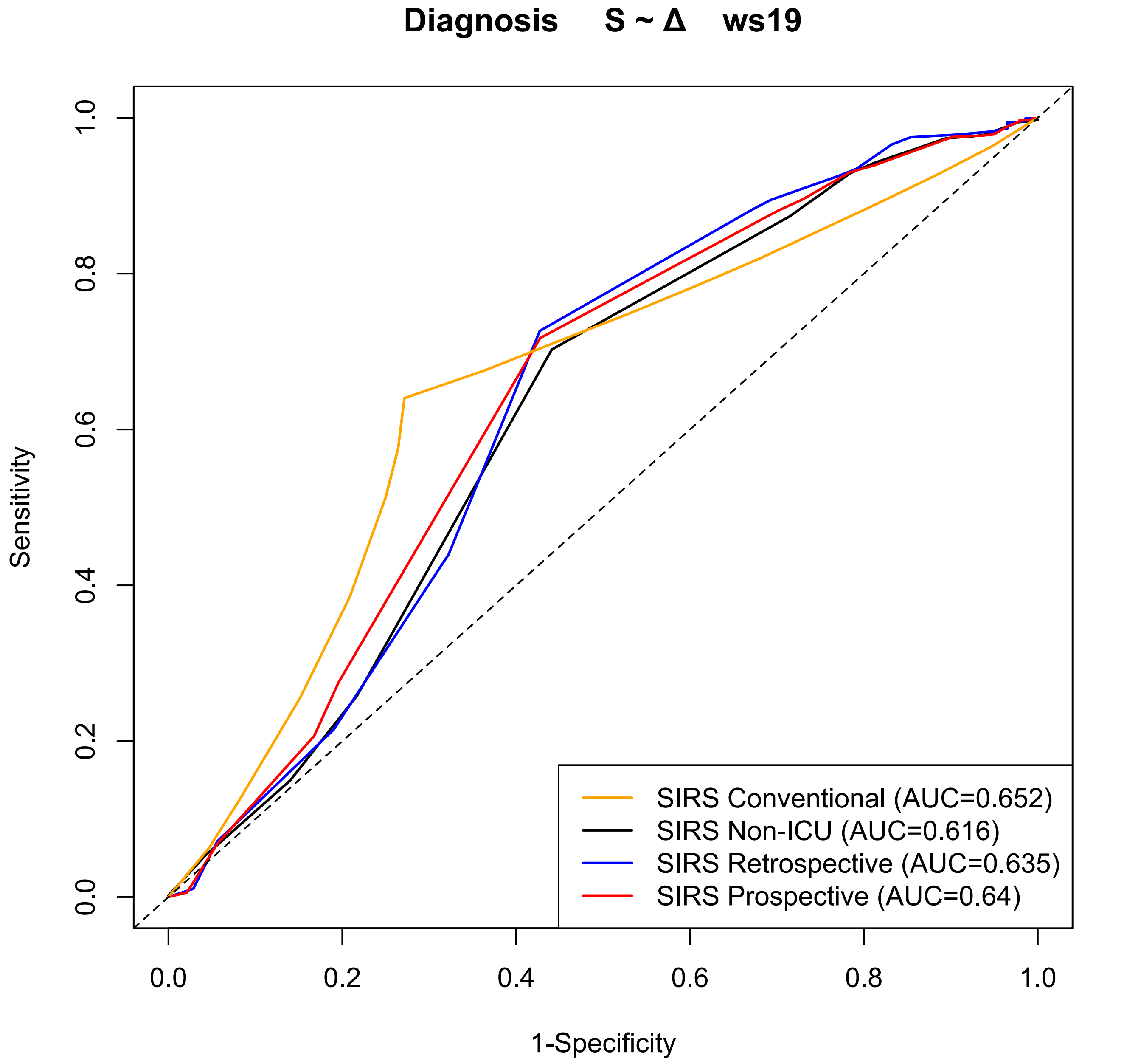
Diagnosis S ~ Δ ws19
Sensitivity
1-Specificity
0.0
0.2
0.4
0.6
0.8
1.0
SIRS Conventional (AUC=0.652)
SIRS Non-ICU (AUC=0.616)
SIRS Retrospective (AUC=0.635)
SIRS Prospective (AUC=0.64)

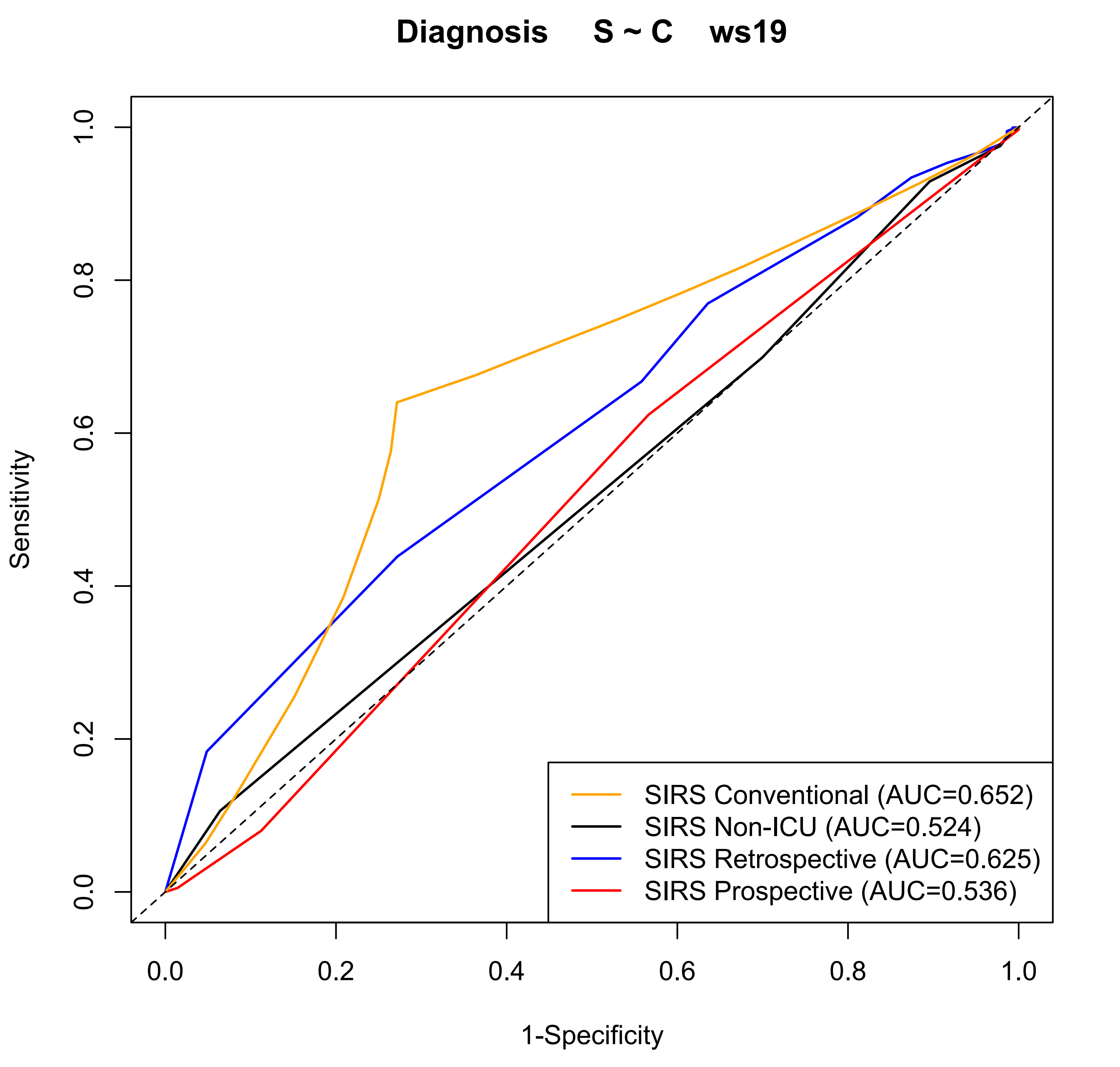

Diagnosis S ~ C ws19
Sensitivity
1-Specificity
0.0
0.2
0.4
0.6
0.8
1.0
SIRS Conventional (AUC=0.652)
SIRS Non-ICU (AUC=0.524)
SIRS Retrospective (AUC=0.625)
SIRS Prospective (AUC=0.536)

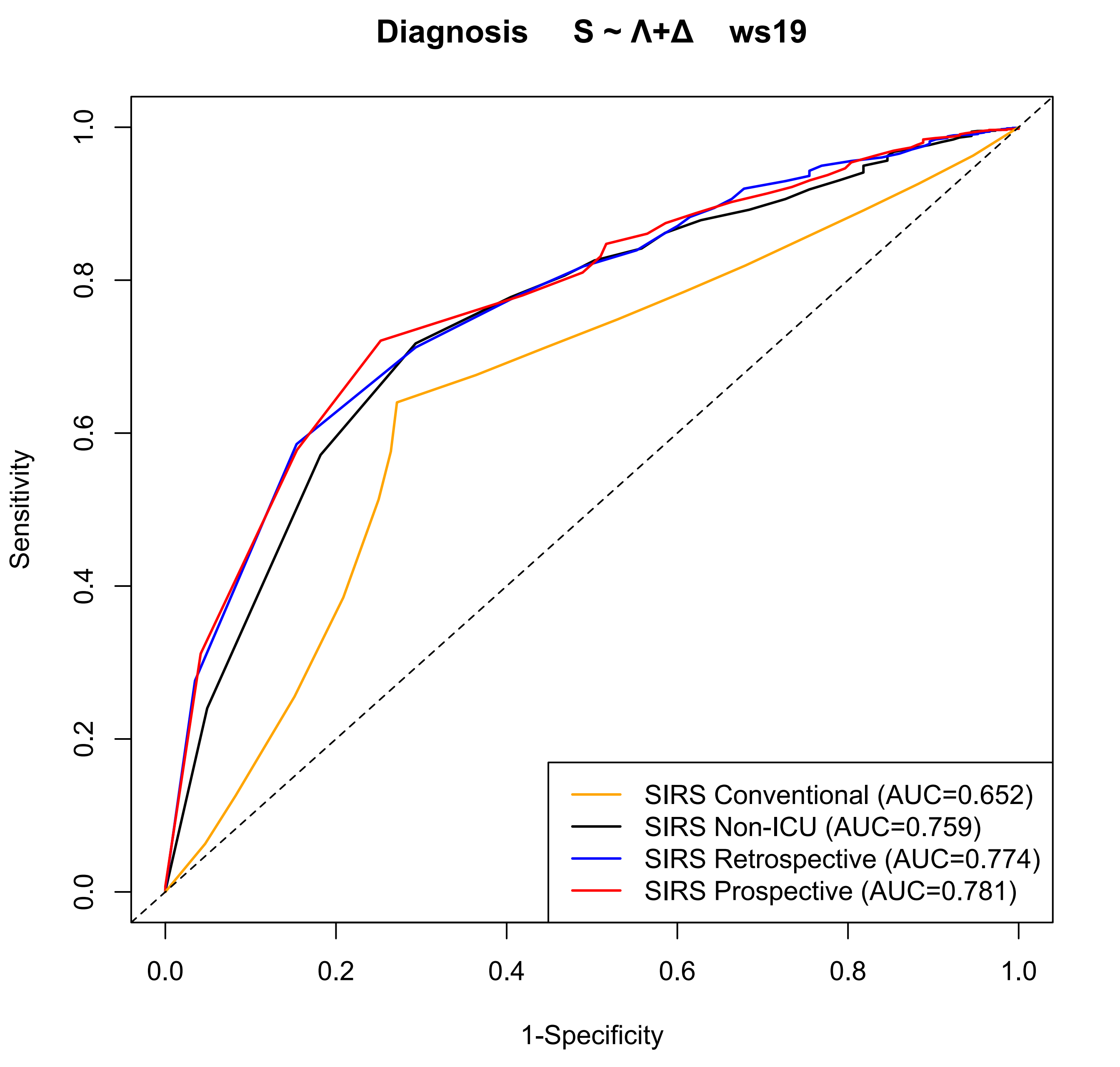
Diagnosis S ~ Λ+Δ ws19
Sensitivity
1-Specificity
0.0
0.2
0.4
0.6
0.8
1.0
0.0
0.2
0.4
0.6
0.8
1.0
SIRS Conventional (AUC=0.652)
SIRS Non-ICU (AUC=0.759)
SIRS Retrospective (AUC=0.774)
SIRS Prospective (AUC=0.781)

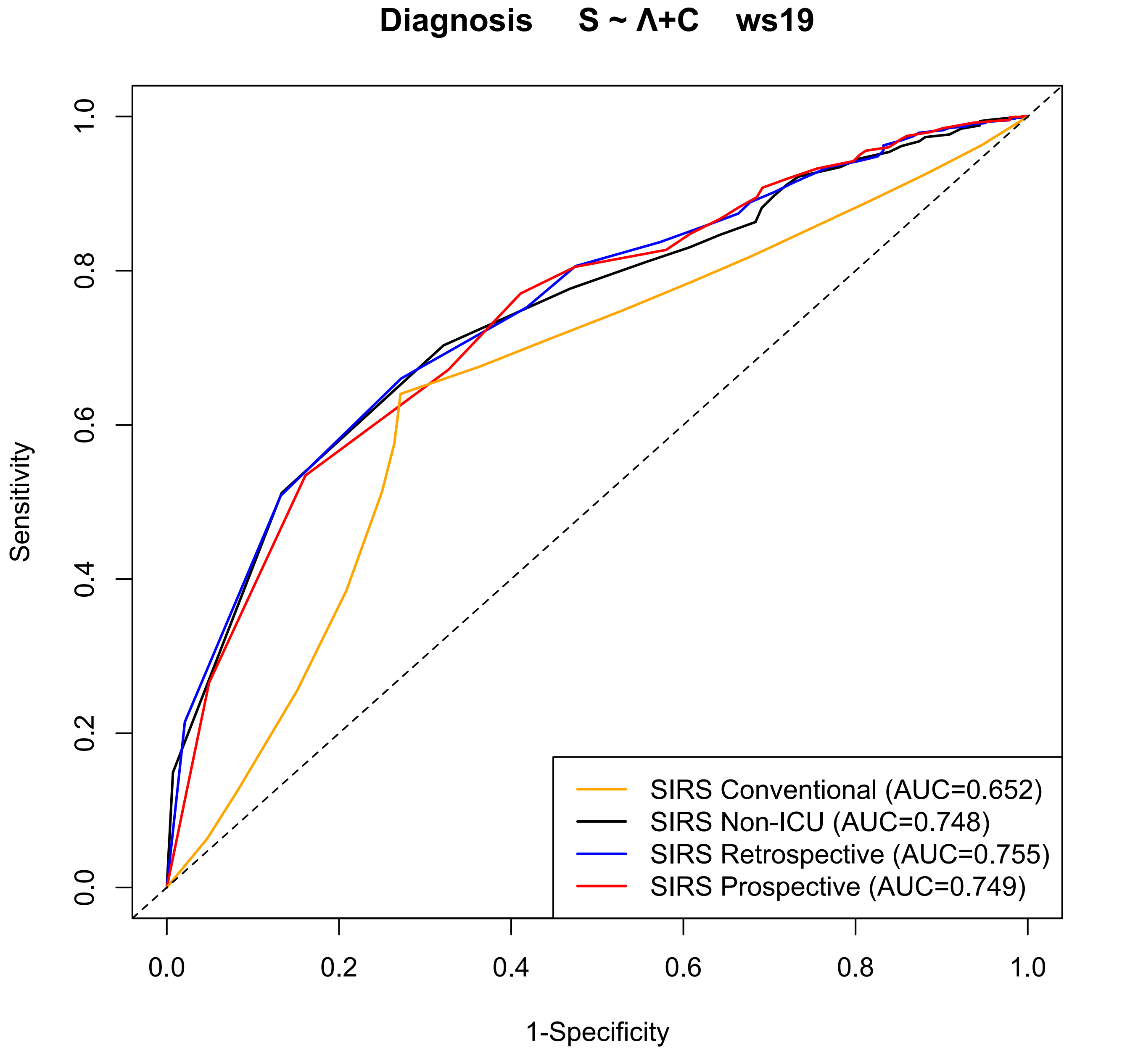
Diagnosis S ~ Λ+C ws19
Sensitivity
1-Specificity
0.0
0.2
0.4
0.6
0.8
1.0
0.0
0.2
0.4
0.6
0.8
1.0
SIRS Conventional (AUC=0.652)
SIRS Non-ICU (AUC=0.748)
SIRS Retrospective (AUC=0.755)
SIRS Prospective (AUC=0.749)

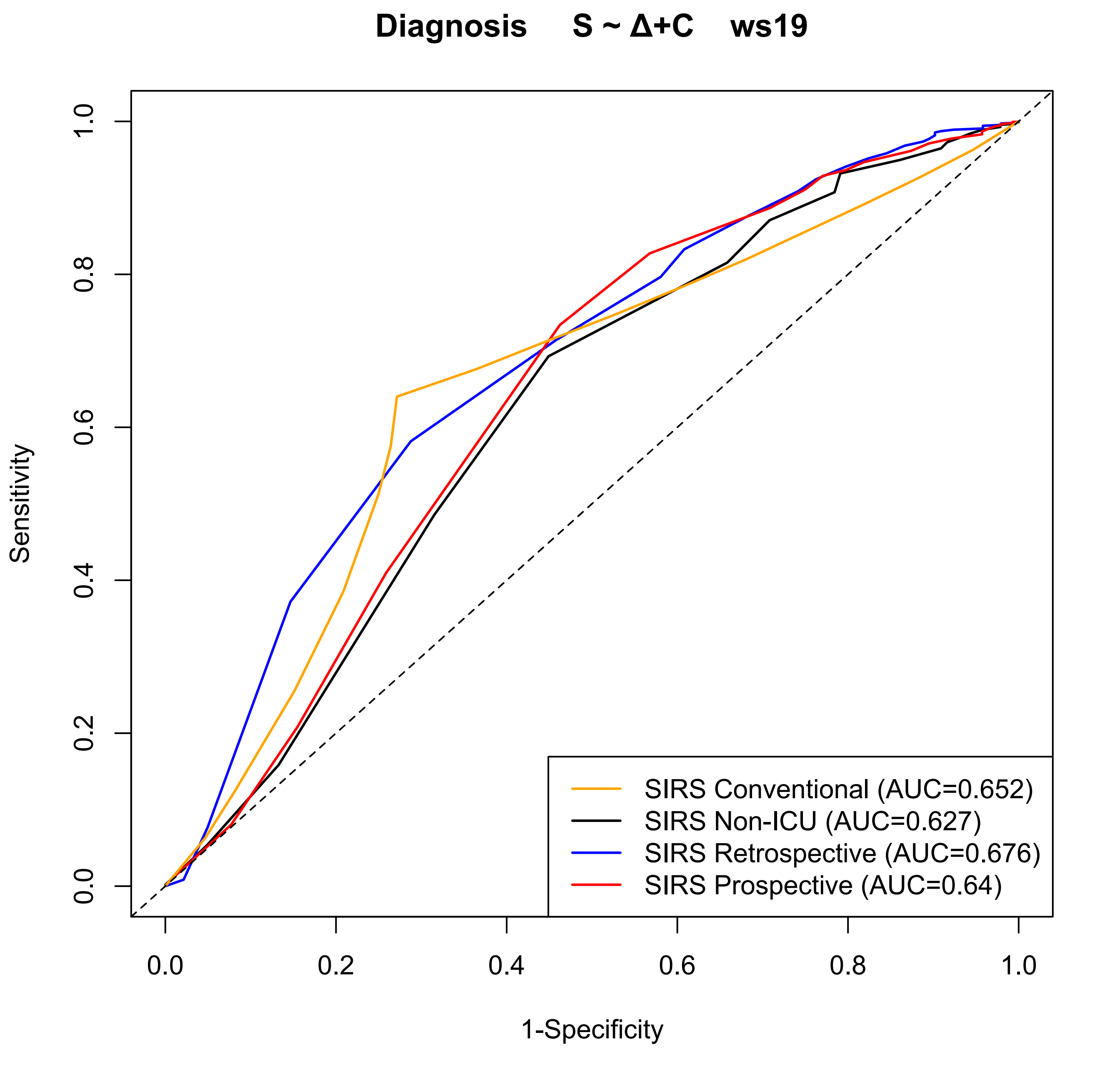

Diagnosis S ~ Δ+C ws19
Sensitivity
1-Specificity
0.0
0.2
0.4
0.6
0.8
1.0
SIRS Conventional (AUC=0.652)
SIRS Non-ICU (AUC=0.627)
SIRS Retrospective (AUC=0.676)
SIRS Prospective (AUC=0.64)

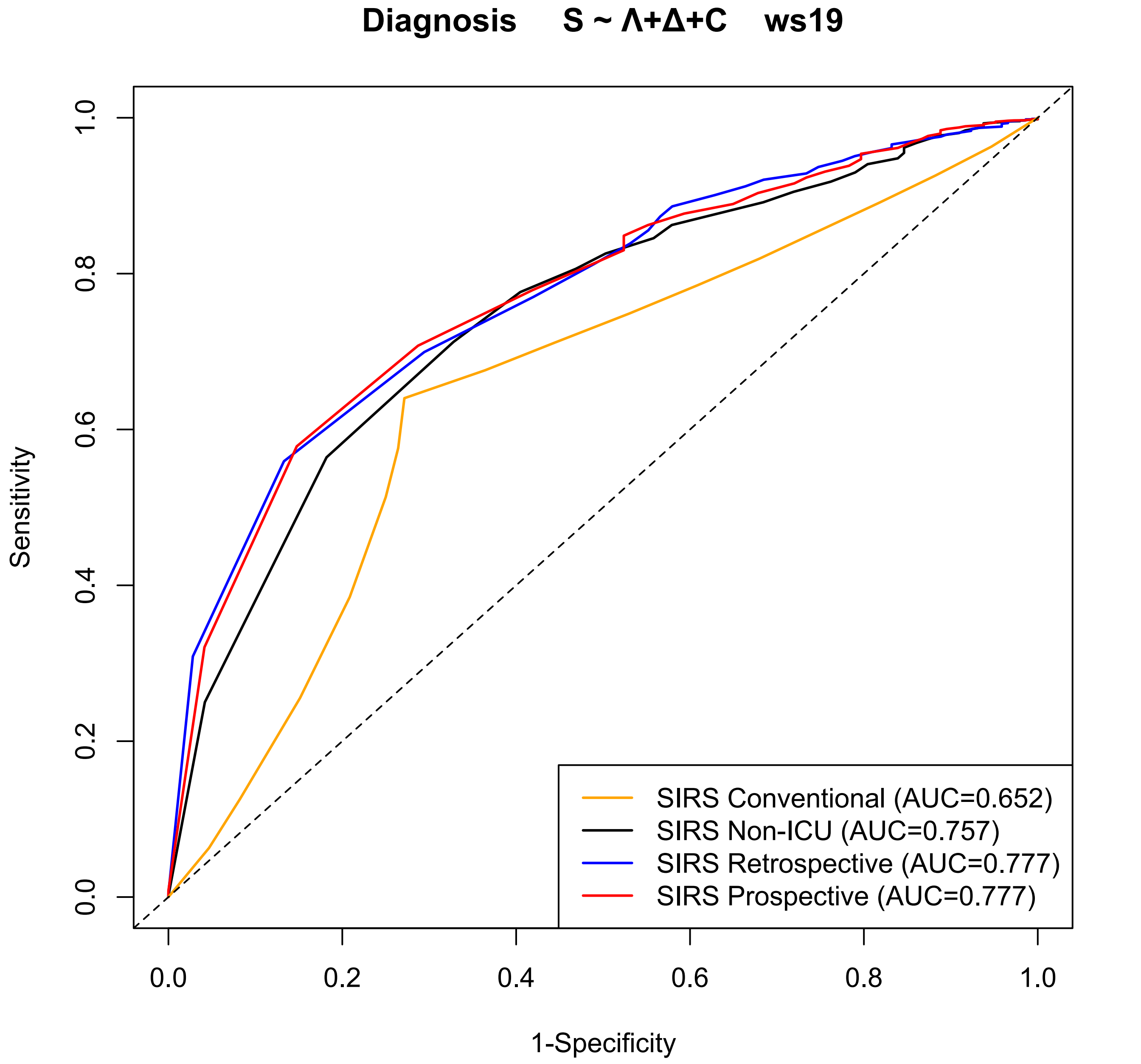
Diagnosis S ~ Λ+Δ+C ws19
Sensitivity
1-Specificity
0.0
0.2
0.4
0.6
0.8
1.0
SIRS Conventional (AUC=0.652)
SIRS Non-ICU (AUC=0.757)
SIRS Retrospective (AUC=0.777)
SIRS Prospective (AUC=0.777)

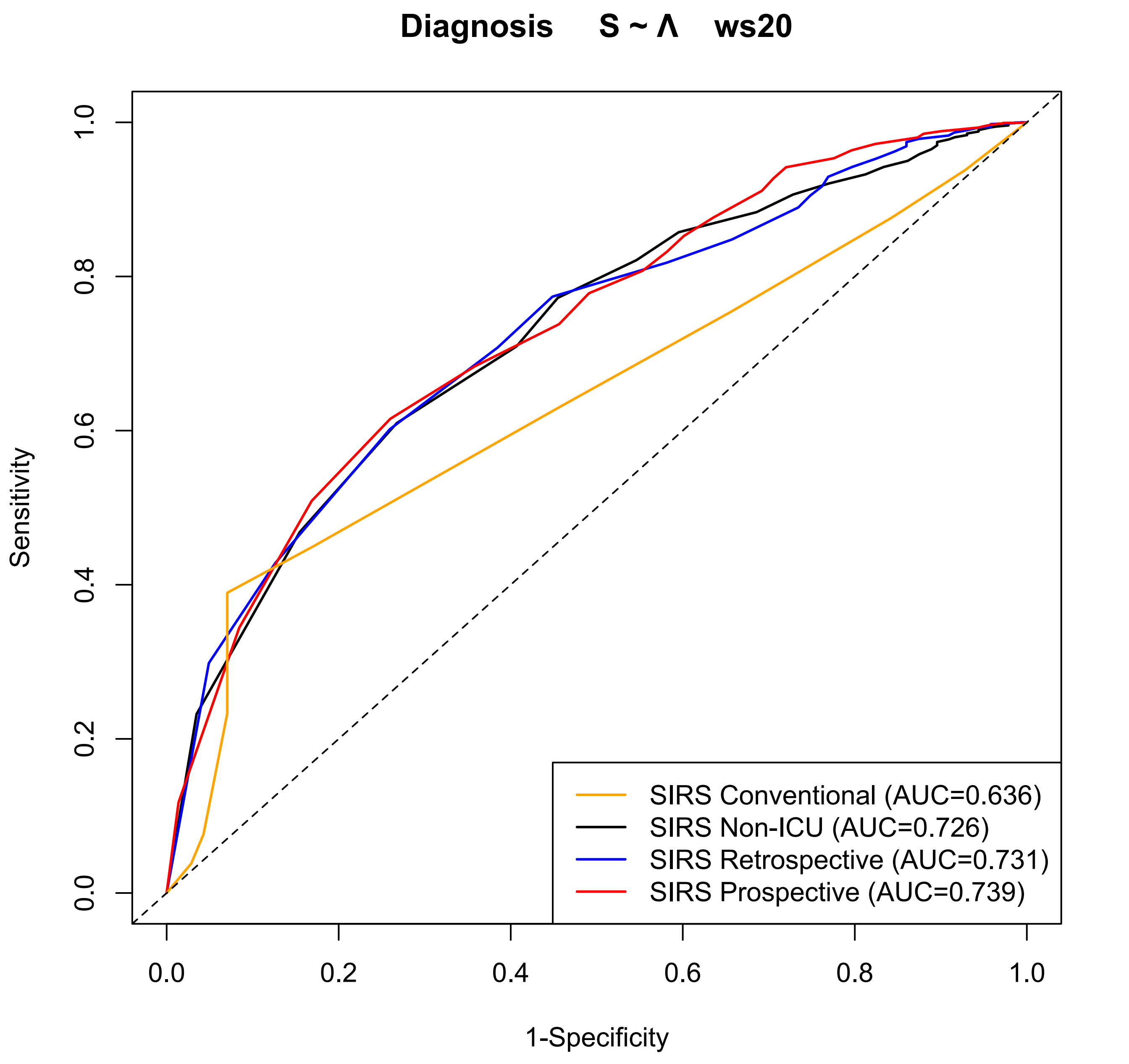

Diagnosis S ~ Λ ws20
Sensitivity
1-Specificity
0.0
0.2
0.4
0.6
0.8
1.0
SIRS Conventional (AUC=0.636)
SIRS Non-ICU (AUC=0.726)
SIRS Retrospective (AUC=0.731)
SIRS Prospective (AUC=0.739)

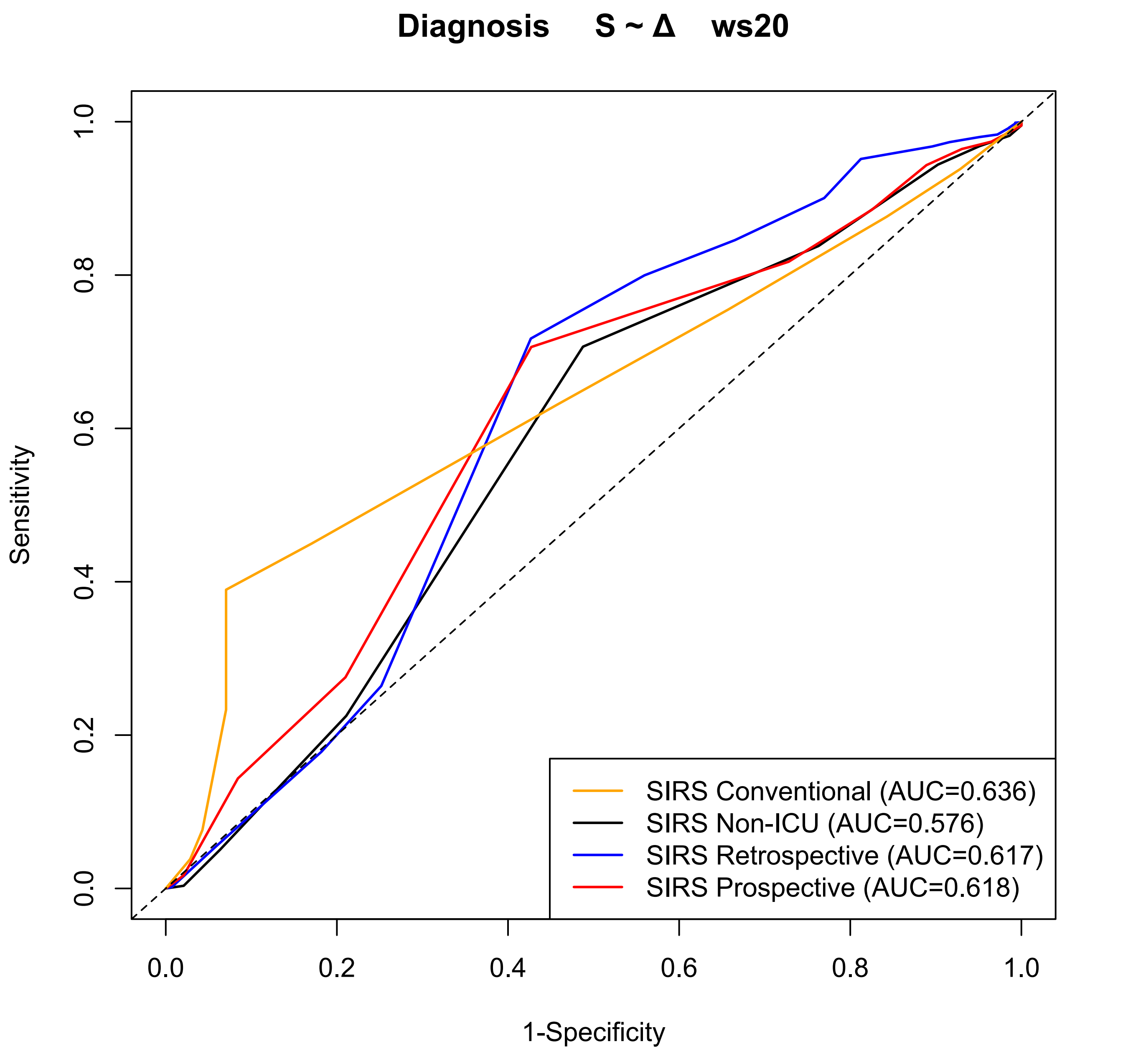

Diagnosis S ~ Δ ws20
Sensitivity
1-Specificity
0.0
0.2
0.4
0.6
0.8
1.0
SIRS Conventional (AUC=0.636)
SIRS Non-ICU (AUC=0.576)
SIRS Retrospective (AUC=0.617)
SIRS Prospective (AUC=0.618)

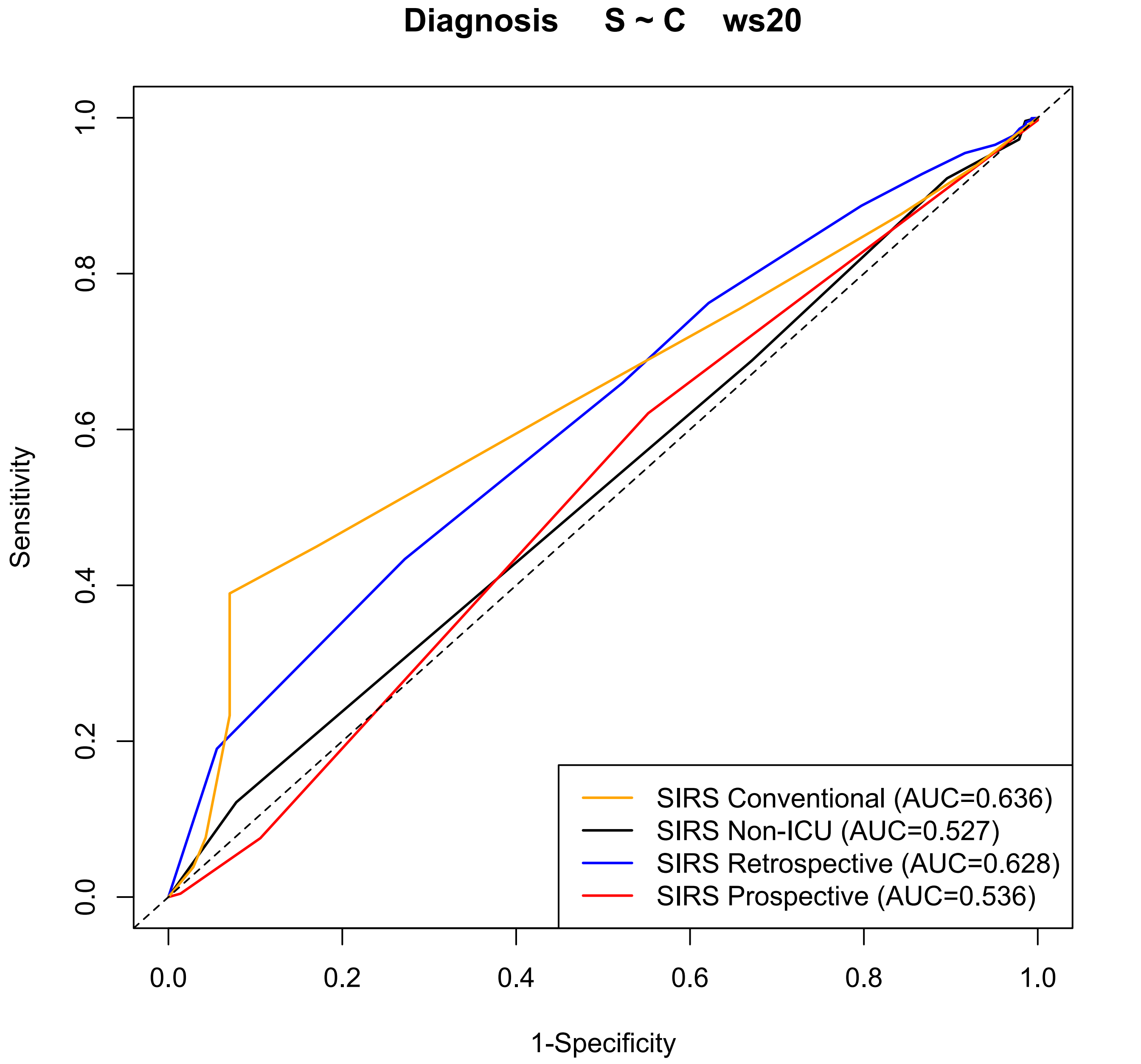
Diagnosis S ~ C ws20
Sensitivity
1-Specificity
0.0
0.2
0.4
0.6
0.8
1.0
SIRS Conventional (AUC=0.636)
SIRS Non-ICU (AUC=0.527)
SIRS Retrospective (AUC=0.628)
SIRS Prospective (AUC=0.536)

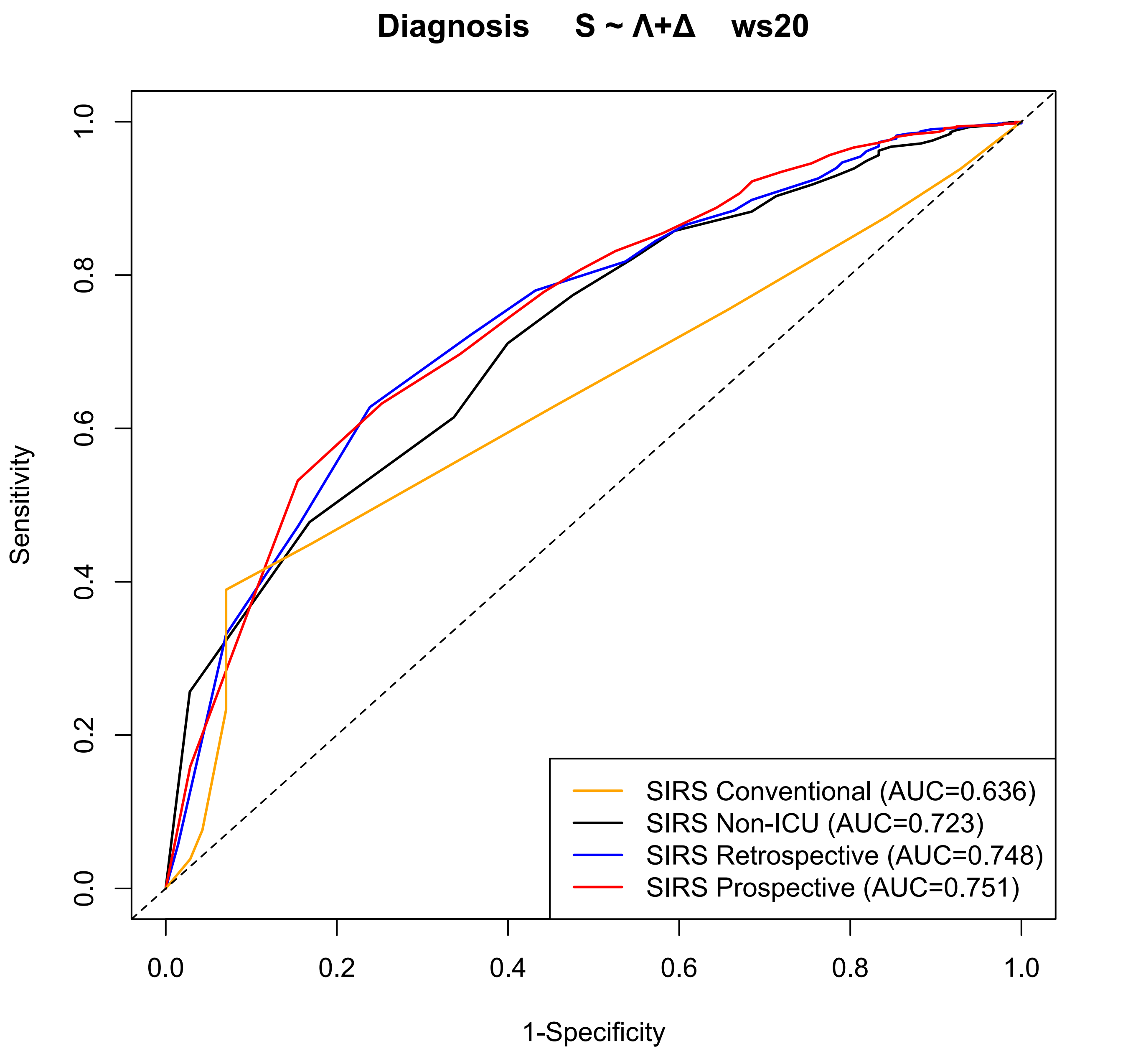

Diagnosis S ~ Λ+Δ ws20
Sensitivity
1-Specificity
0.0
0.2
0.4
0.6
0.8
1.0
0.0
0.2
0.4
0.6
0.8
1.0
SIRS Conventional (AUC=0.636)
SIRS Non-ICU (AUC=0.723)
SIRS Retrospective (AUC=0.748)
SIRS Prospective (AUC=0.751)

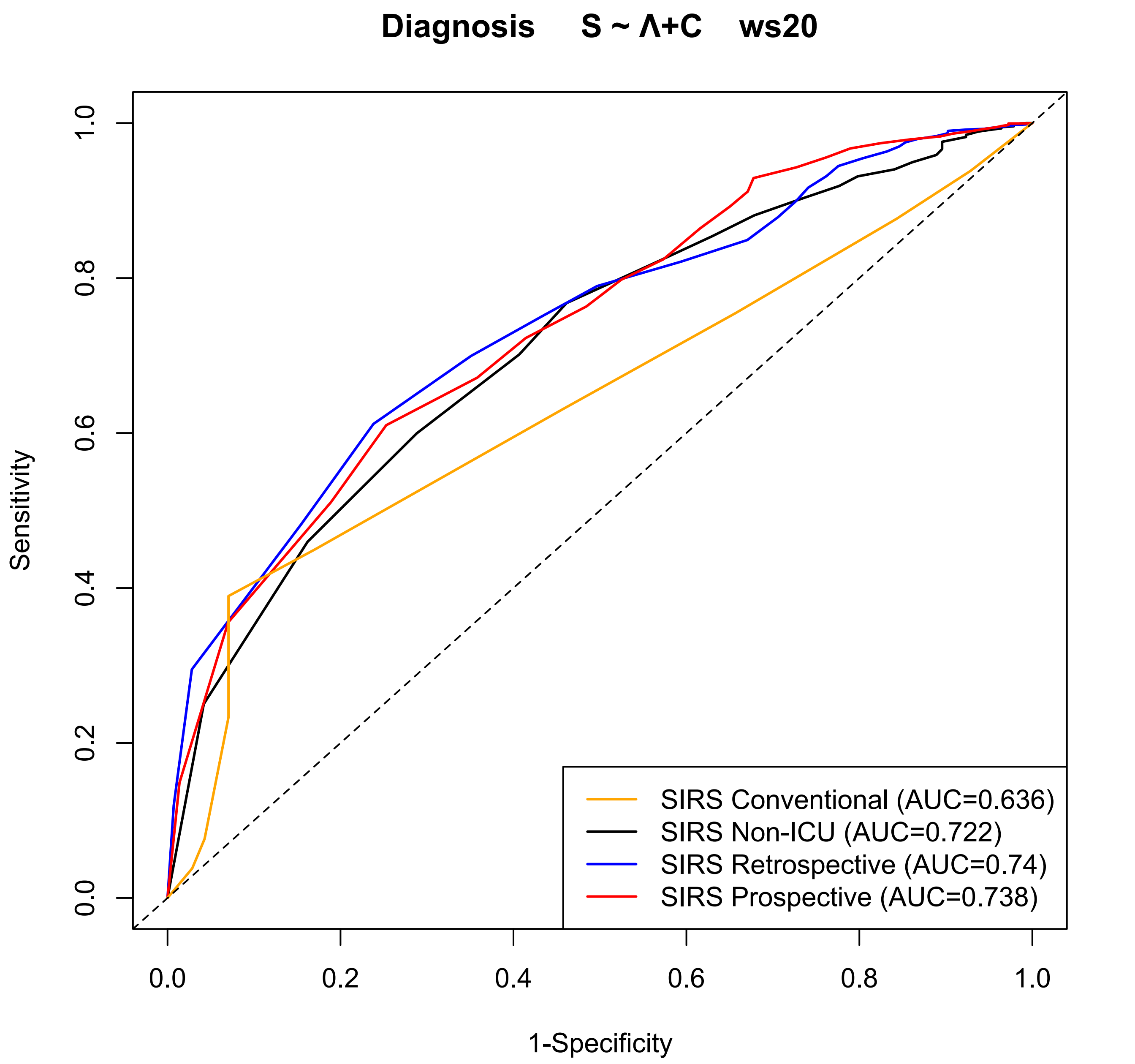
Diagnosis S ~ Λ+C ws20
Sensitivity
1-Specificity
0.0
0.2
0.4
0.6
0.8
1.0
SIRS Conventional (AUC=0.636)
SIRS Non-ICU (AUC=0.722)
SIRS Retrospective (AUC=0.74)
SIRS Prospective (AUC=0.738)

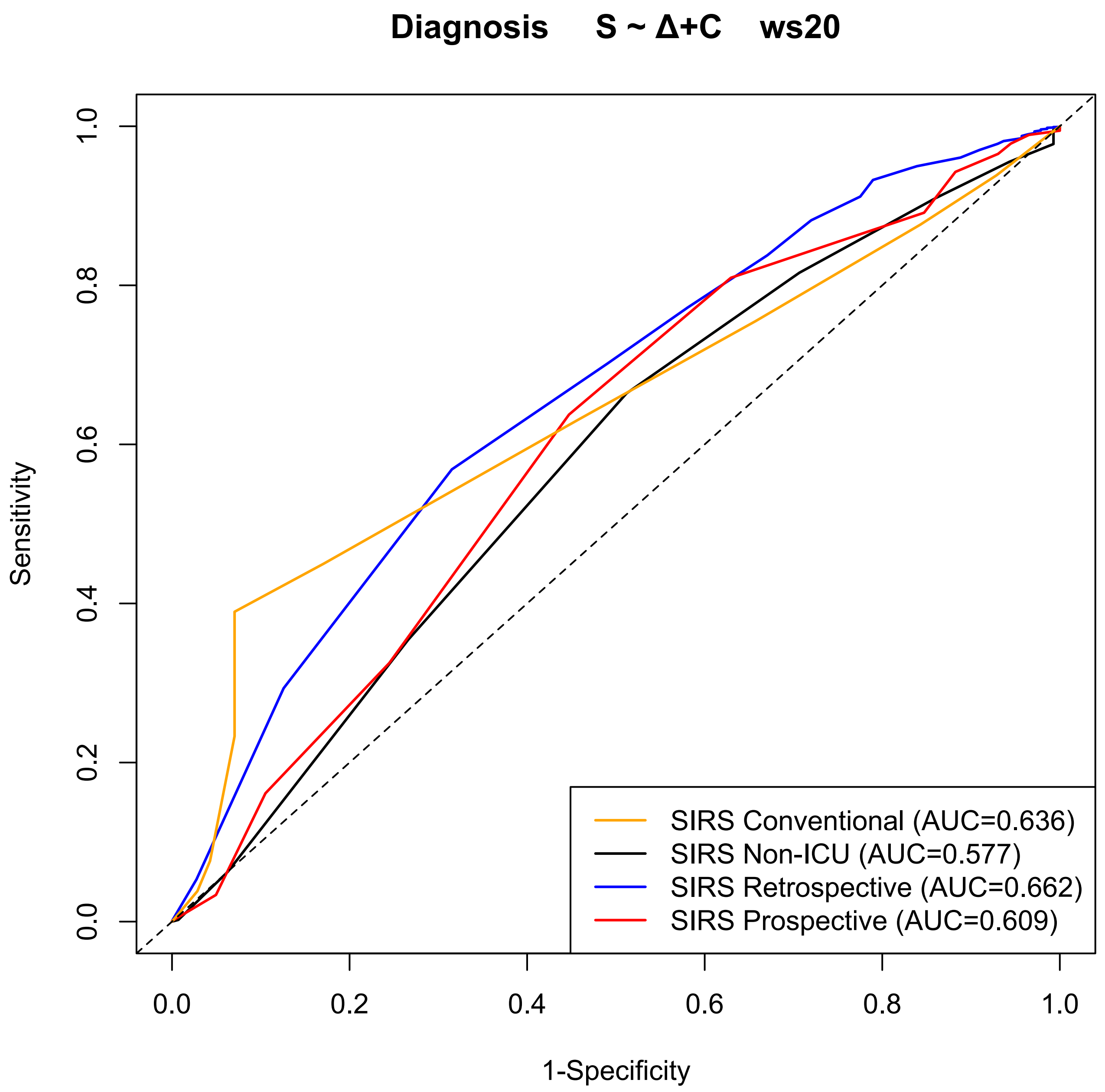

Diagnosis S ~ Δ+C ws20
Sensitivity
1-Specificity
0.0
0.2
0.4
0.6
0.8
1.0
SIRS Conventional (AUC=0.636)
SIRS Non-ICU (AUC=0.577)
SIRS Retrospective (AUC=0.662)
SIRS Prospective (AUC=0.609)

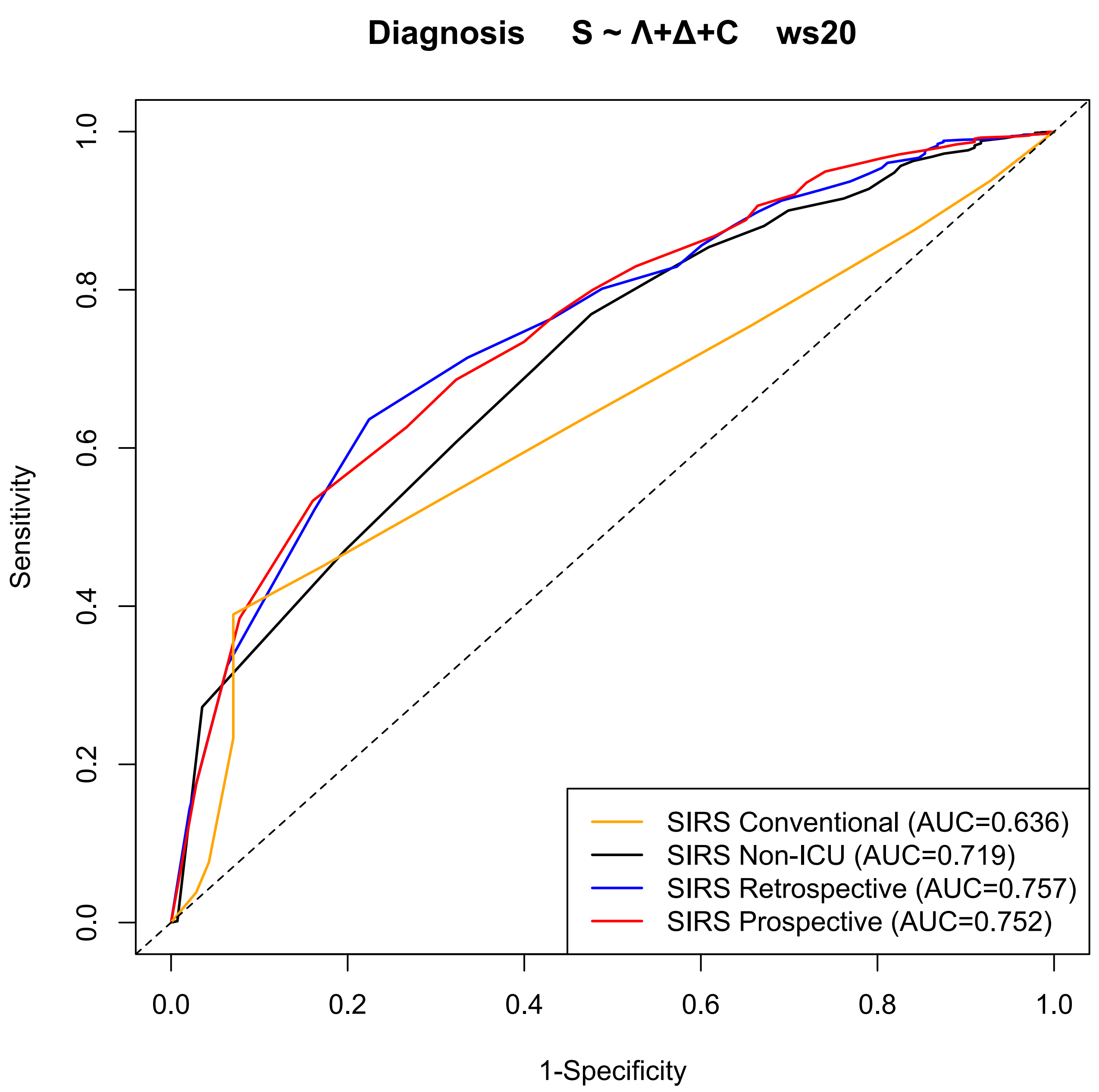

Diagnosis S ~ Λ+Δ+C ws20
Sensitivity
1-Specificity
0.0
0.2
0.4
0.6
0.8
1.0
SIRS Conventional (AUC=0.636)
SIRS Non-ICU (AUC=0.719)
SIRS Retrospective (AUC=0.757)
SIRS Prospective (AUC=0.752)

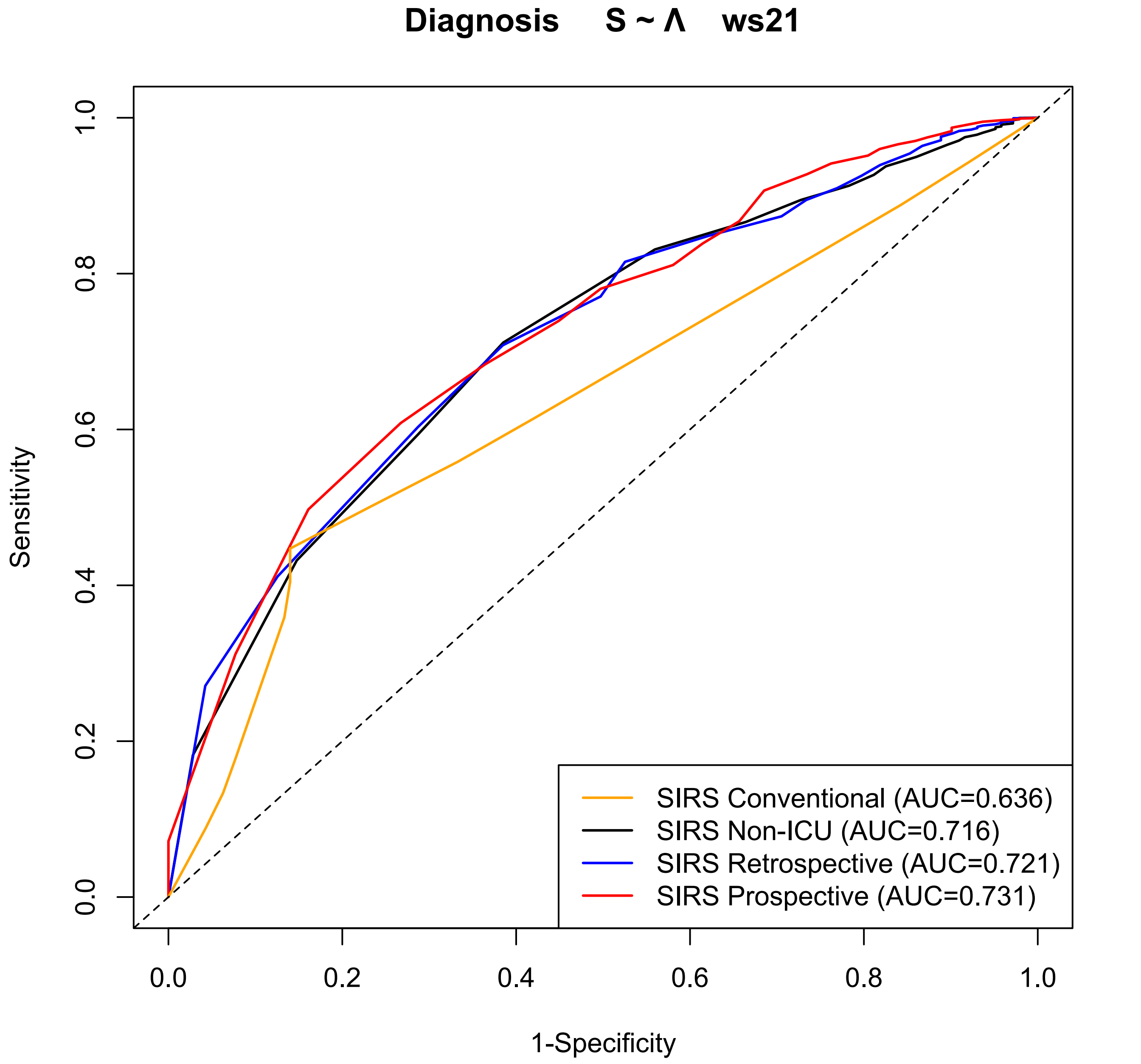
Diagnosis S ~ Λ ws21
Sensitivity
1-Specificity
0.0
0.2
0.4
0.6
0.8
1.0
SIRS Conventional (AUC=0.636)
SIRS Non-ICU (AUC=0.716)
SIRS Retrospective (AUC=0.721)
SIRS Prospective (AUC=0.731)

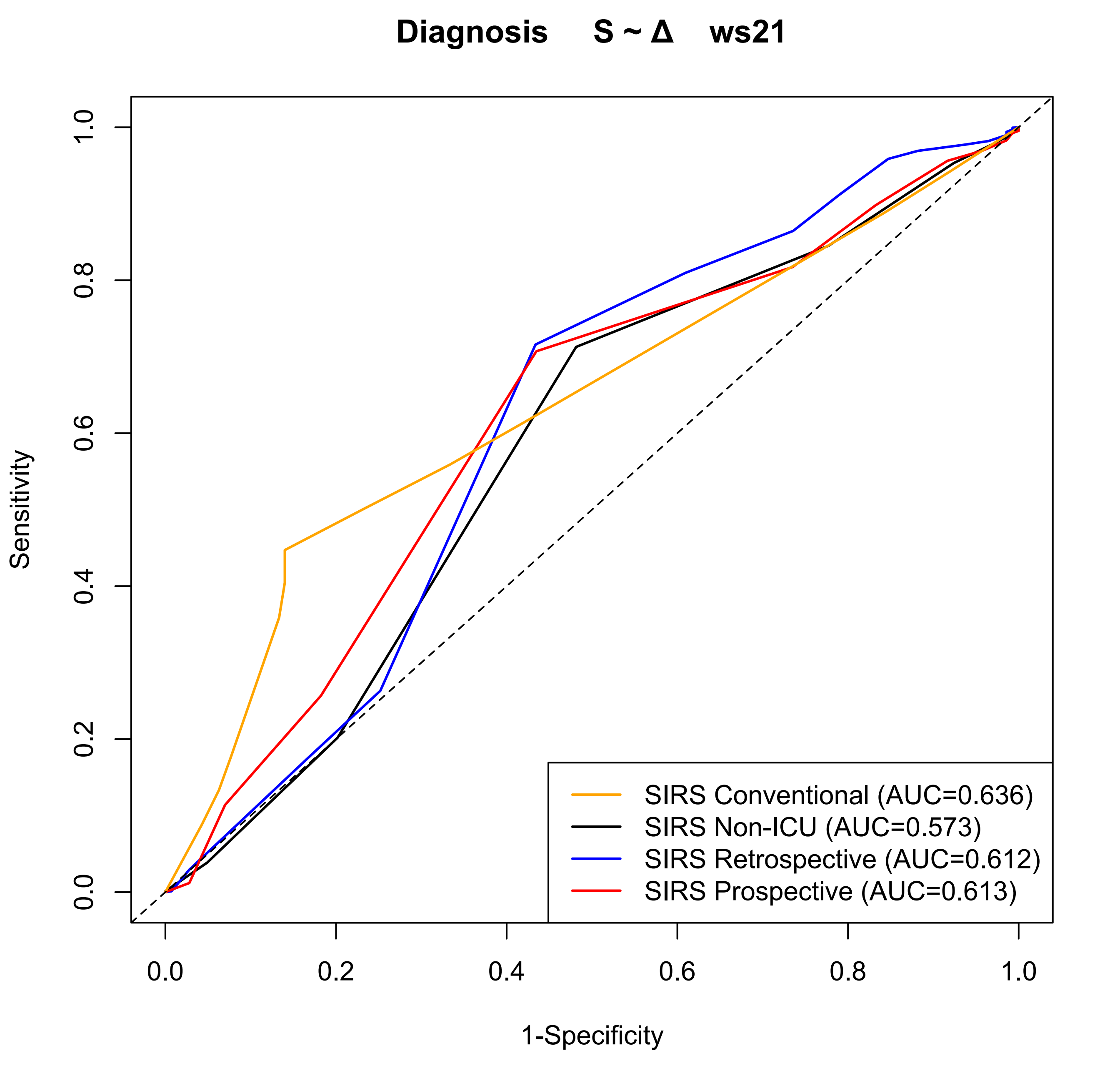
Diagnosis S ~ Δ ws21
Sensitivity
1-Specificity
0.0
0.2
0.4
0.6
0.8
1.0
SIRS Conventional (AUC=0.636)
SIRS Non-ICU (AUC=0.573)
SIRS Retrospective (AUC=0.612)
SIRS Prospective (AUC=0.613)

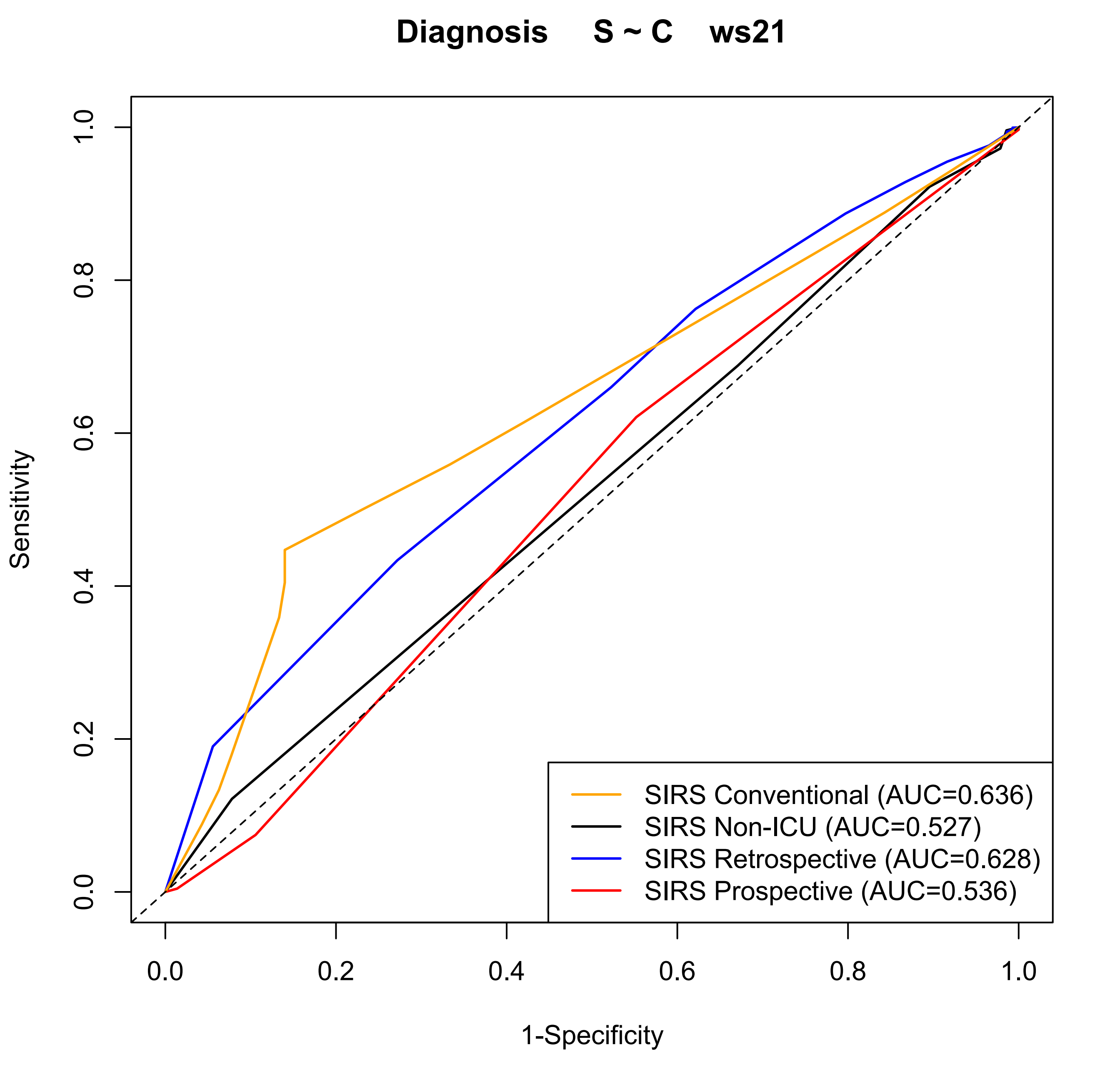
Diagnosis S ~ C ws21
Sensitivity
1-Specificity
0.0
0.2
0.4
0.6
0.8
1.0
SIRS Conventional (AUC=0.636)
SIRS Non-ICU (AUC=0.527)
SIRS Retrospective (AUC=0.628)
SIRS Prospective (AUC=0.536)

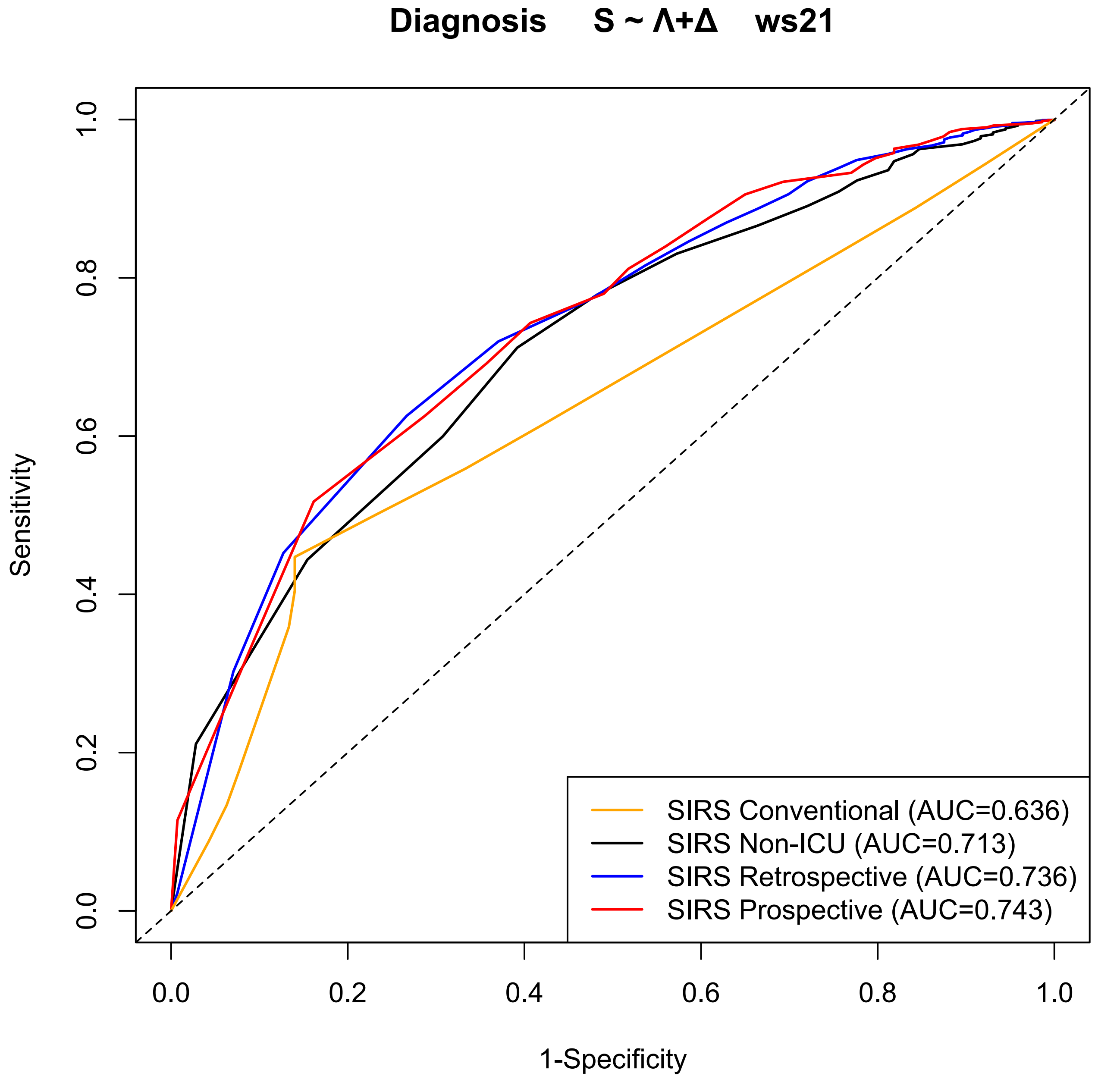

Diagnosis S ~ Λ+Δ ws21
Sensitivity
1-Specificity
0.0
0.2
0.4
0.6
0.8
1.0
0.0
0.2
0.4
0.6
0.8
1.0
SIRS Conventional (AUC=0.636)
SIRS Non-ICU (AUC=0.713)
SIRS Retrospective (AUC=0.736)
SIRS Prospective (AUC=0.743)

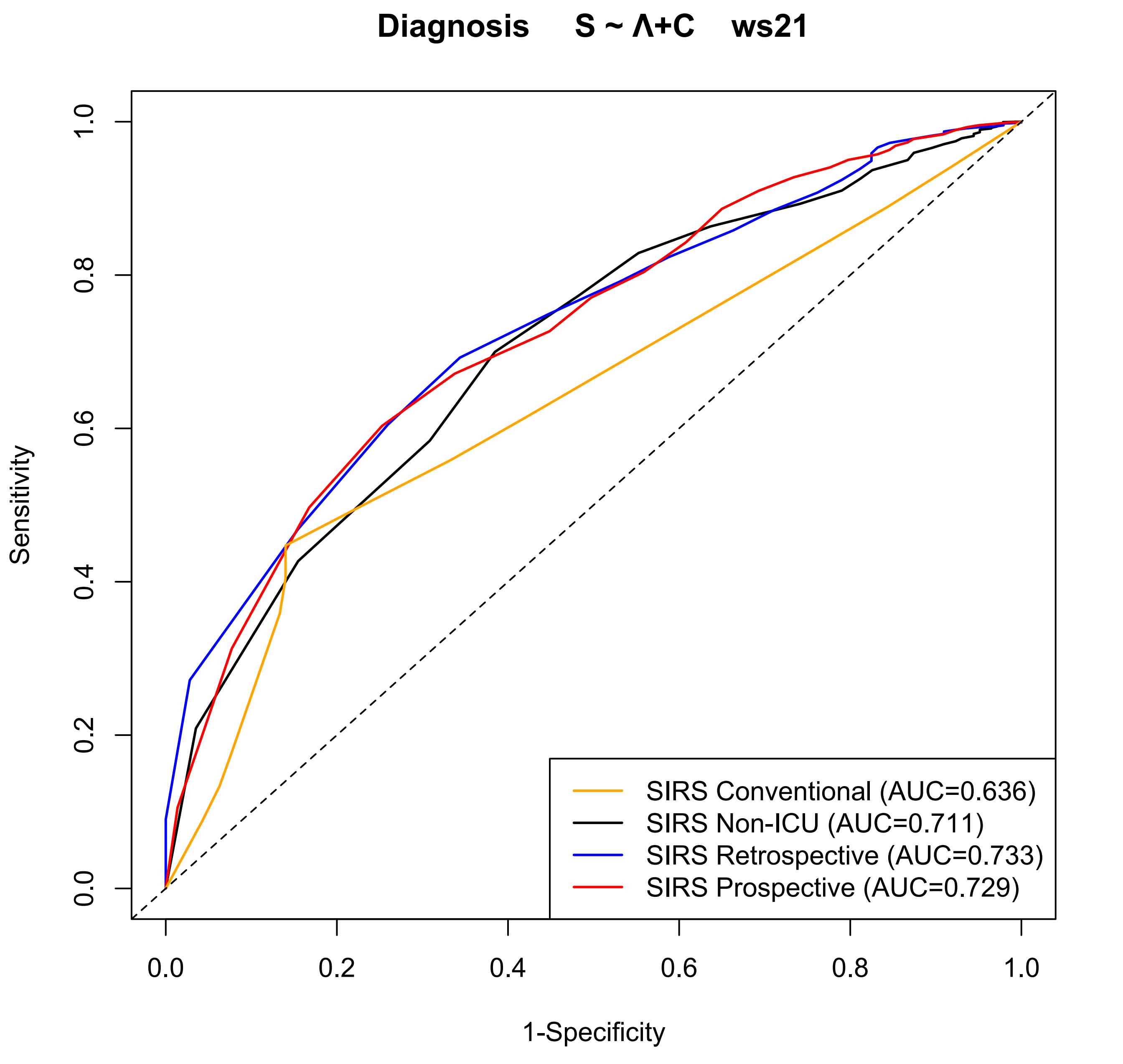

Diagnosis S ~ Λ+C ws21
Sensitivity
1-Specificity
0.0
0.2
0.4
0.6
0.8
1.0
SIRS Conventional (AUC=0.636)
SIRS Non-ICU (AUC=0.711)
SIRS Retrospective (AUC=0.733)
SIRS Prospective (AUC=0.729)

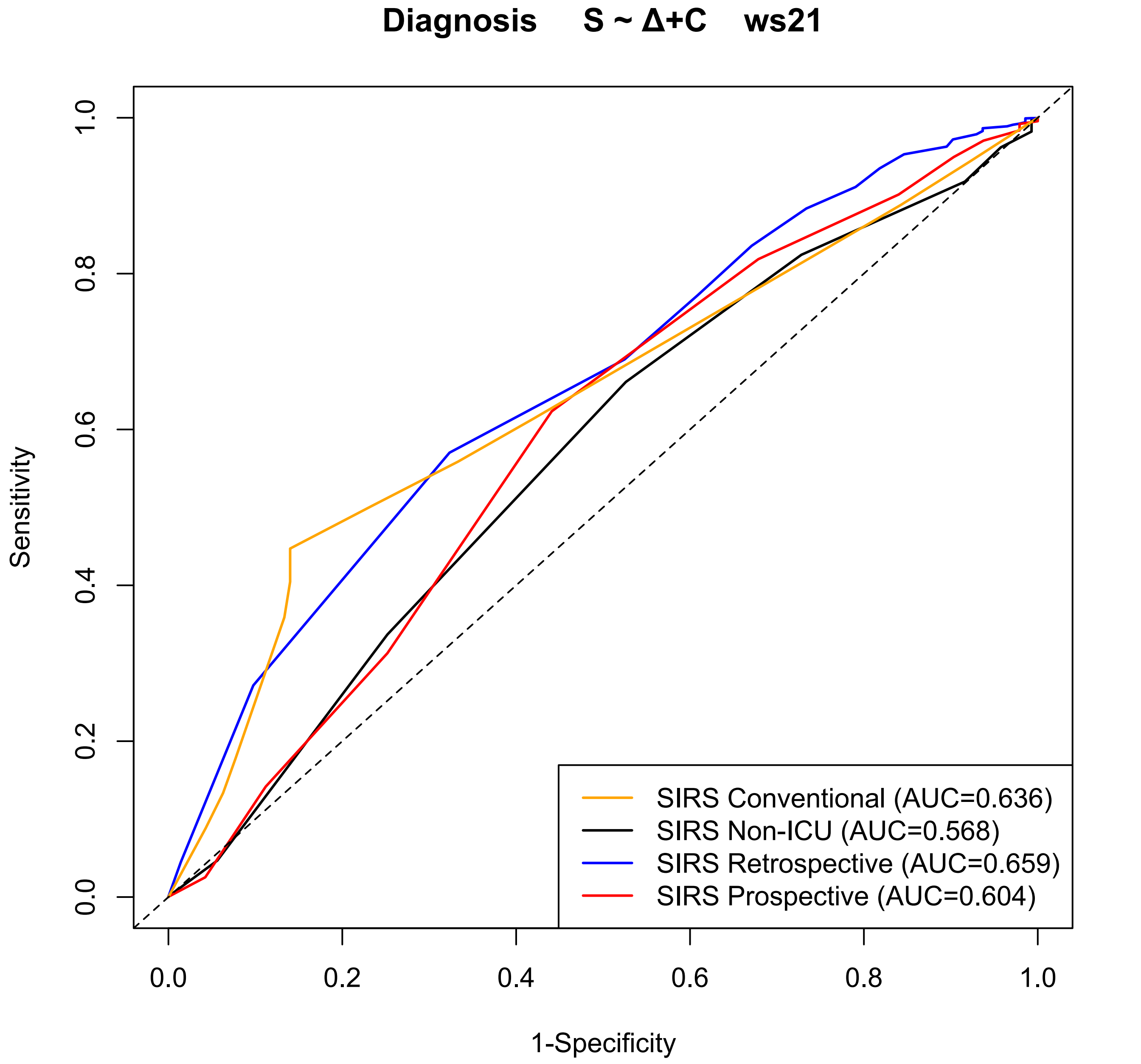

Diagnosis S ~ Δ+C ws21
Sensitivity
1-Specificity
0.0
0.2
0.4
0.6
0.8
1.0
SIRS Conventional (AUC=0.636)
SIRS Non-ICU (AUC=0.568)
SIRS Retrospective (AUC=0.659)
SIRS Prospective (AUC=0.604)

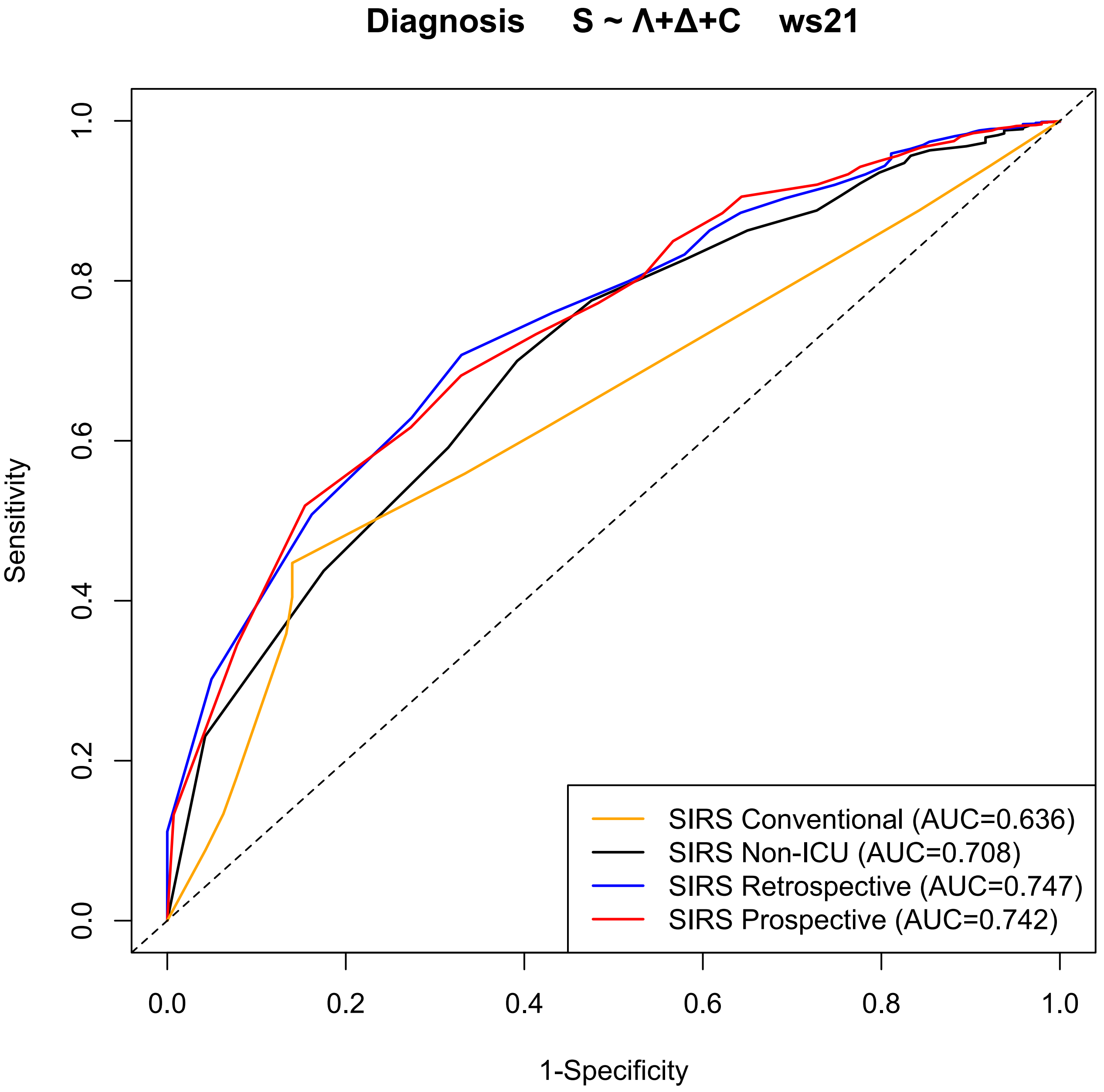
Diagnosis S ~ Λ+Δ+C ws21
Sensitivity
1-Specificity
0.0
0.2
0.4
0.6
0.8
1.0
SIRS Conventional (AUC=0.636)
SIRS Non-ICU (AUC=0.708)
SIRS Retrospective (AUC=0.747)
SIRS Prospective (AUC=0.742)

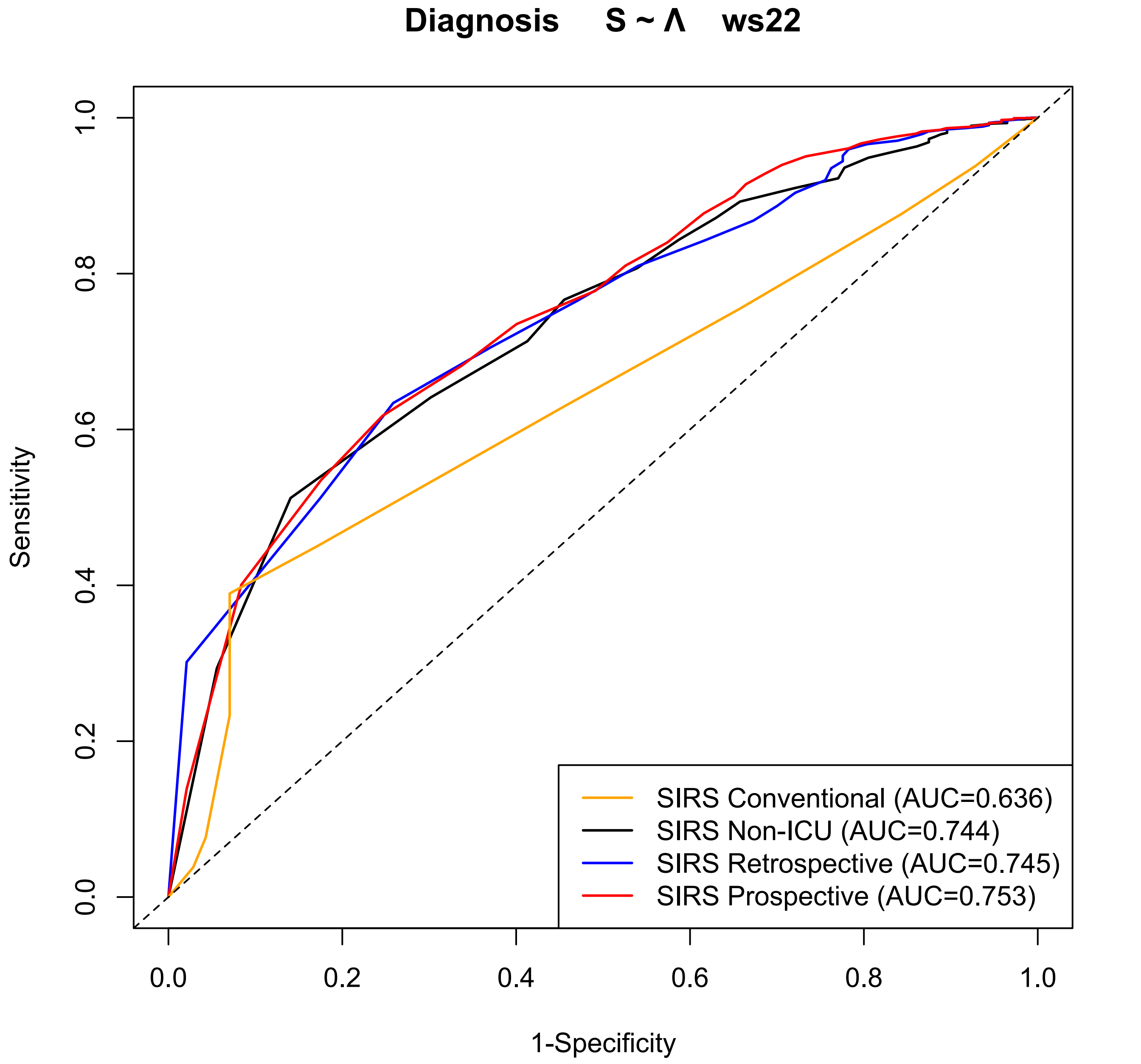

Diagnosis S ~ Λ ws22
Sensitivity
1-Specificity
0.0
0.2
0.4
0.6
0.8
1.0
SIRS Conventional (AUC=0.636)
SIRS Non-ICU (AUC=0.744)
SIRS Retrospective (AUC=0.745)
SIRS Prospective (AUC=0.753)

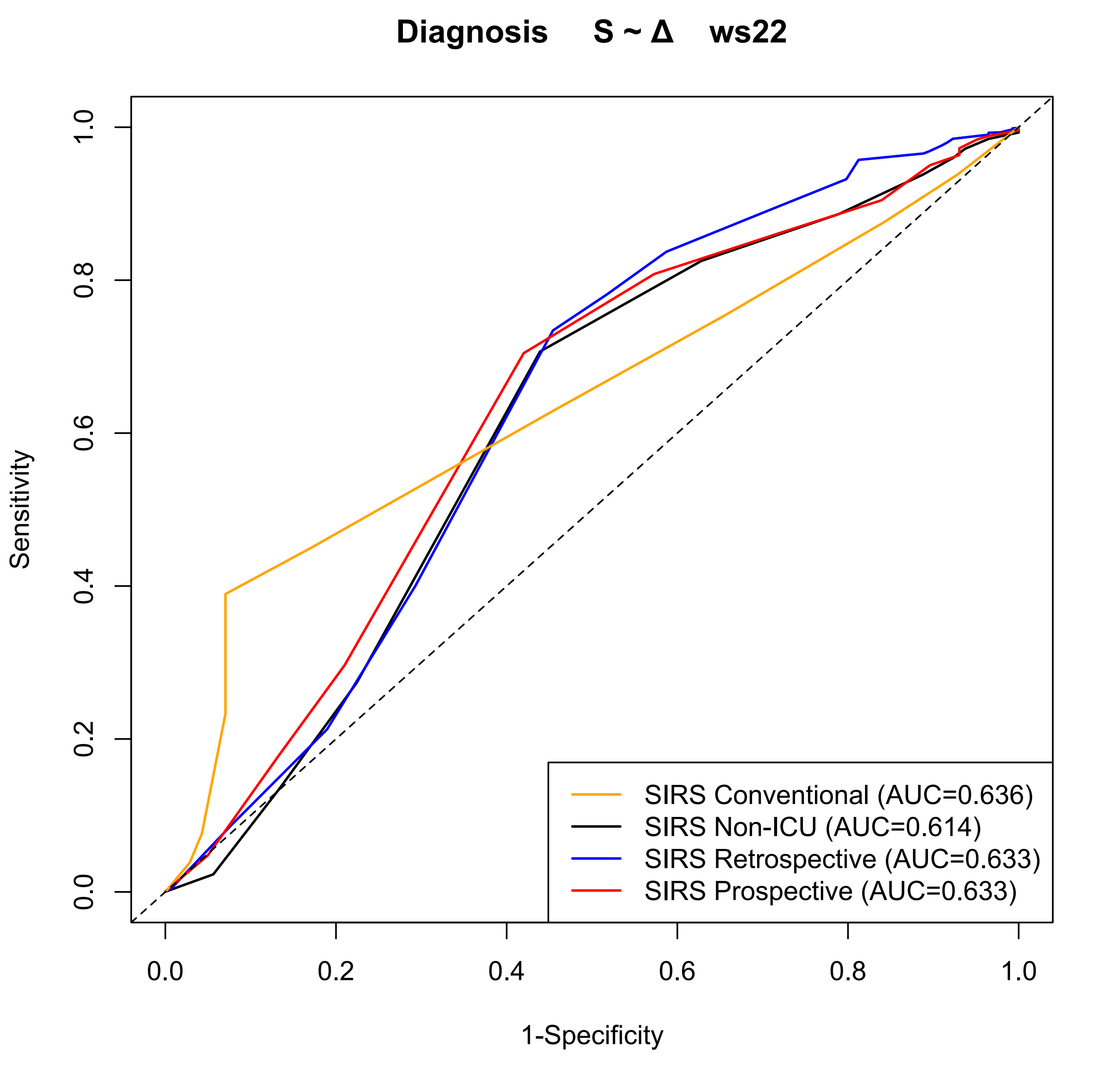

Diagnosis S ~ Δ ws22
Sensitivity
1-Specificity
0.0
0.2
0.4
0.6
0.8
1.0
SIRS Conventional (AUC=0.636)
SIRS Non-ICU (AUC=0.614)
SIRS Retrospective (AUC=0.633)
SIRS Prospective (AUC=0.633)

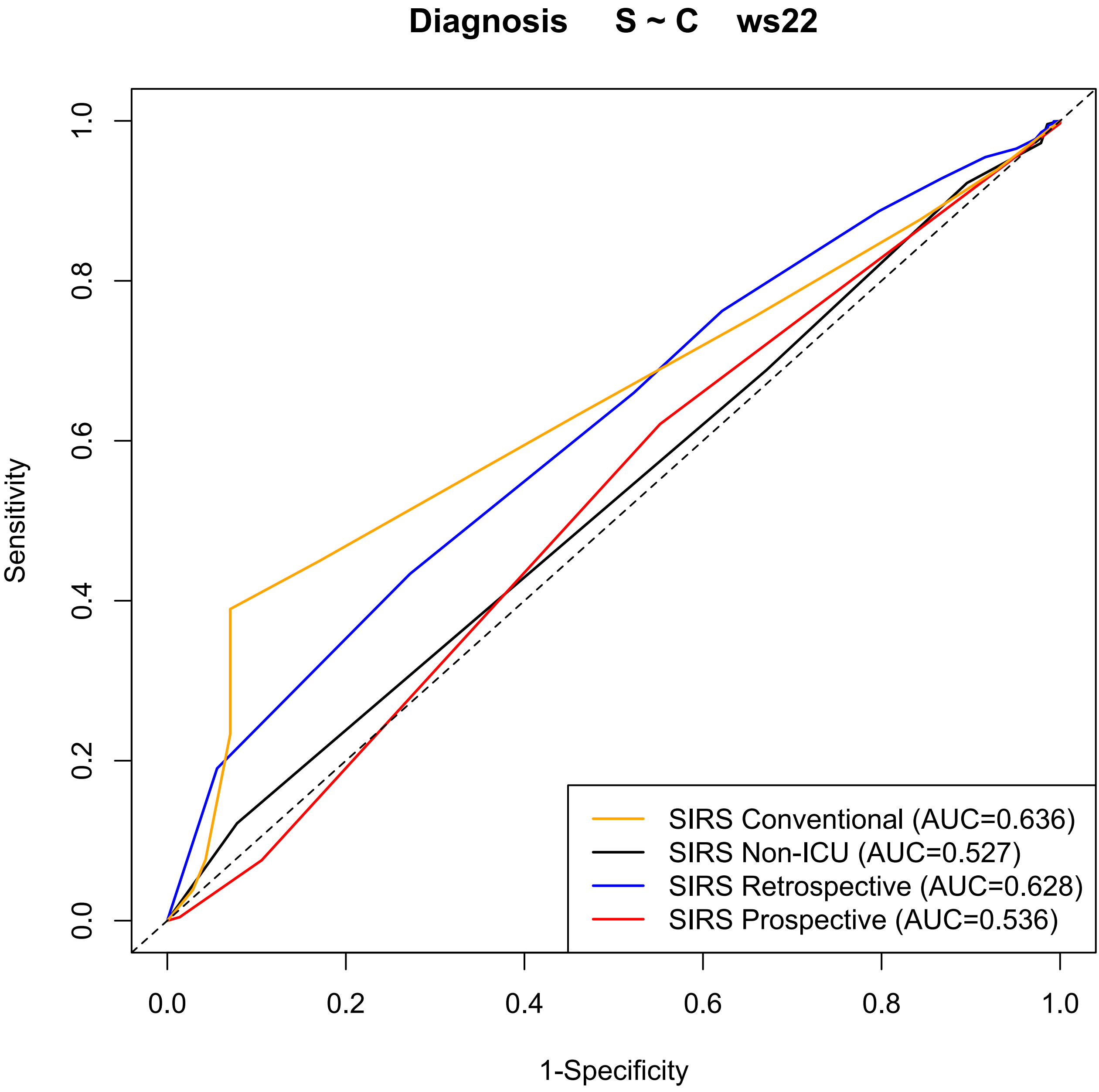
Diagnosis S ~ C ws22
Sensitivity
1-Specificity
0.0
0.2
0.4
0.6
0.8
1.0
SIRS Conventional (AUC=0.636)
SIRS Non-ICU (AUC=0.527)
SIRS Retrospective (AUC=0.628)
SIRS Prospective (AUC=0.536)

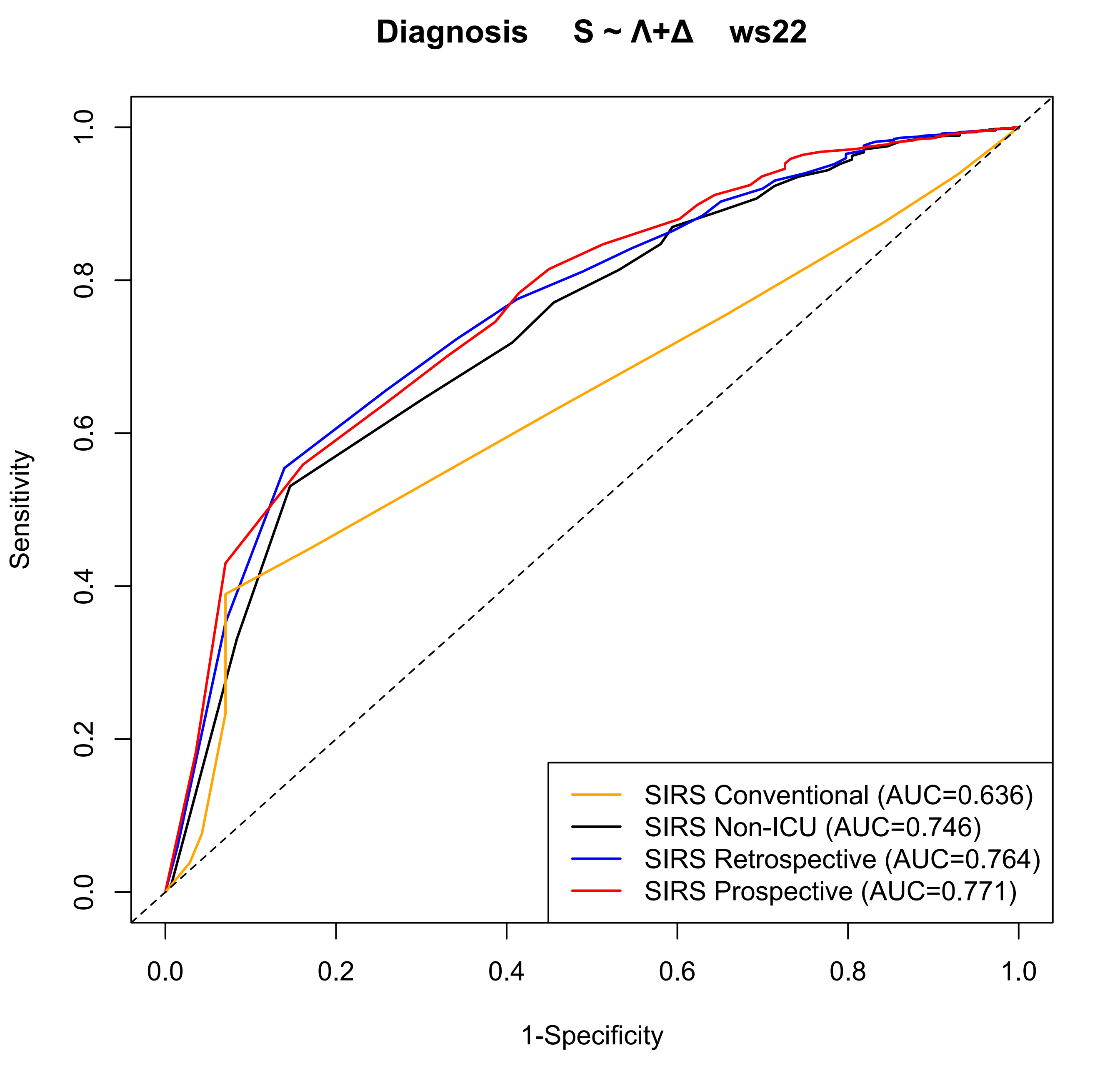
Diagnosis S ~ Λ+Δ ws22
Sensitivity
1-Specificity
0.0
0.2
0.4
0.6
0.8
1.0
SIRS Conventional (AUC=0.636)
SIRS Non-ICU (AUC=0.746)
SIRS Retrospective (AUC=0.764)
SIRS Prospective (AUC=0.771)

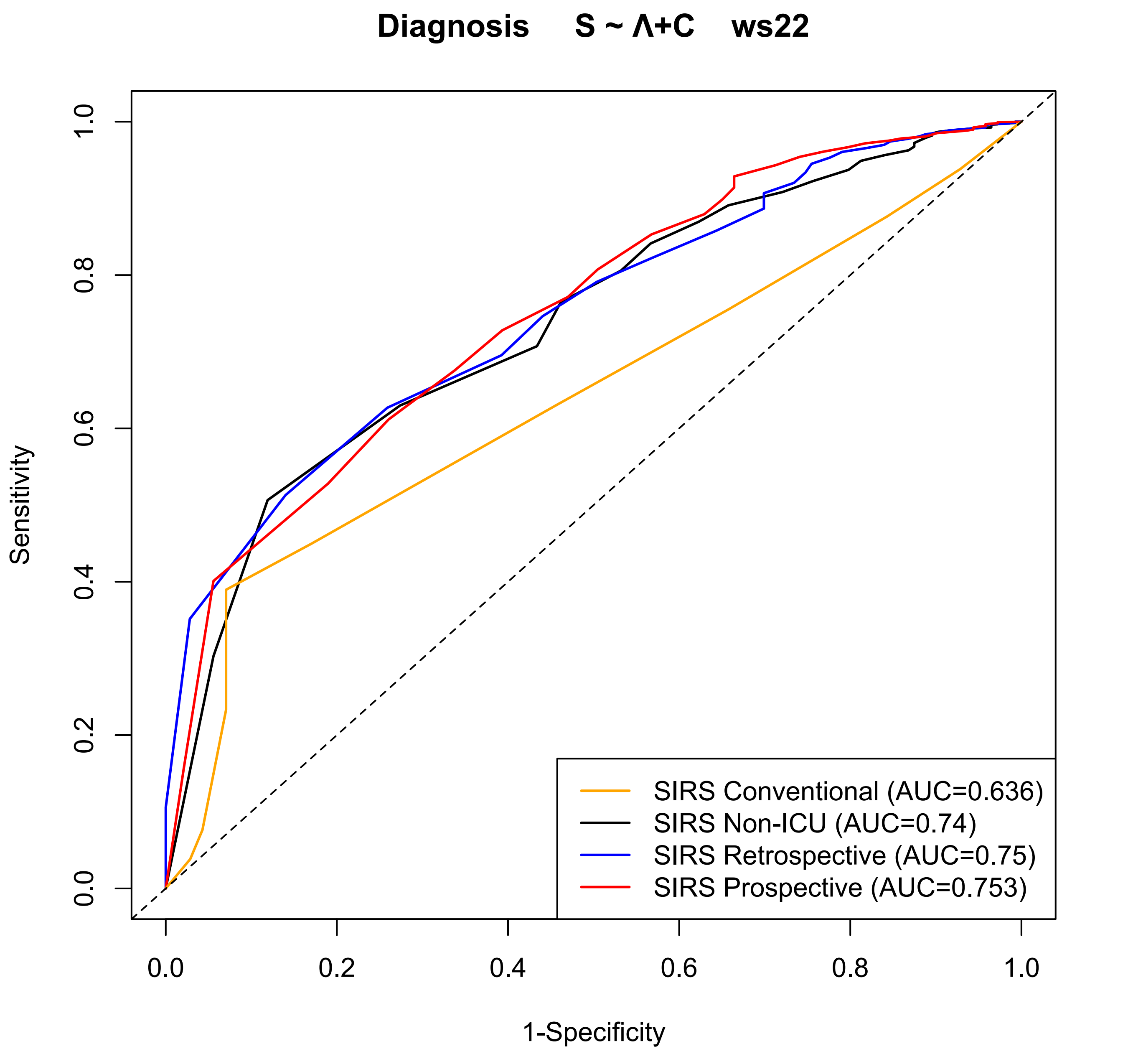
Diagnosis S ~ Λ+C ws22
Sensitivity
1-Specificity
0.0
0.2
0.4
0.6
0.8
1.0
SIRS Conventional (AUC=0.636)
SIRS Non-ICU (AUC=0.74)
SIRS Retrospective (AUC=0.75)
SIRS Prospective (AUC=0.753)

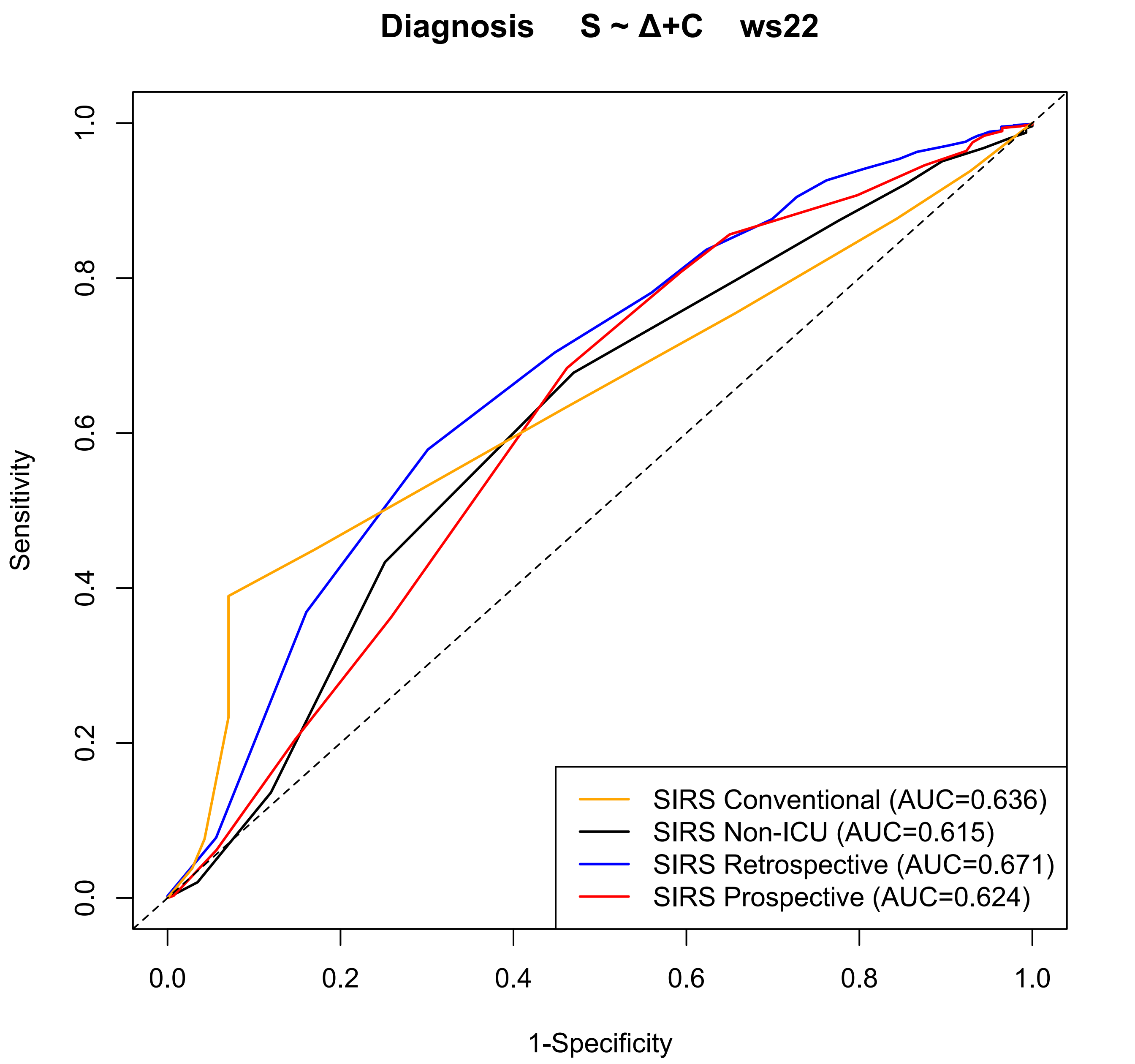
Diagnosis S ~ Δ+C ws22
Sensitivity
1-Specificity
0.0
0.2
0.4
0.6
0.8
1.0
SIRS Conventional (AUC=0.636)
SIRS Non-ICU (AUC=0.615)
SIRS Retrospective (AUC=0.671)
SIRS Prospective (AUC=0.624)

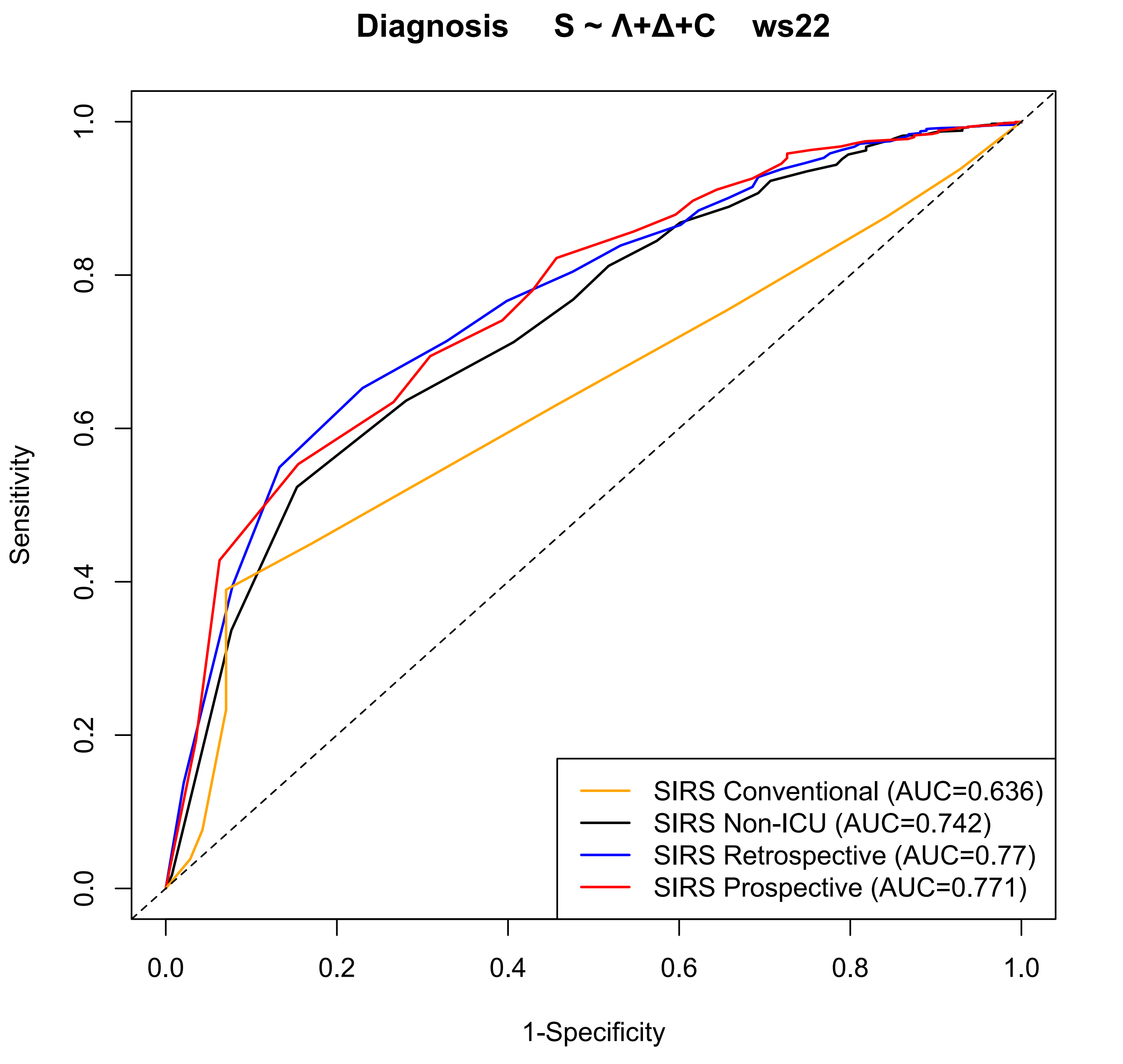

Diagnosis S ~ Λ+Δ+C ws22
Sensitivity
1-Specificity
0.0
0.2
0.4
0.6
0.8
1.0
SIRS Conventional (AUC=0.636)
SIRS Non-ICU (AUC=0.742)
SIRS Retrospective (AUC=0.77)
SIRS Prospective (AUC=0.771)

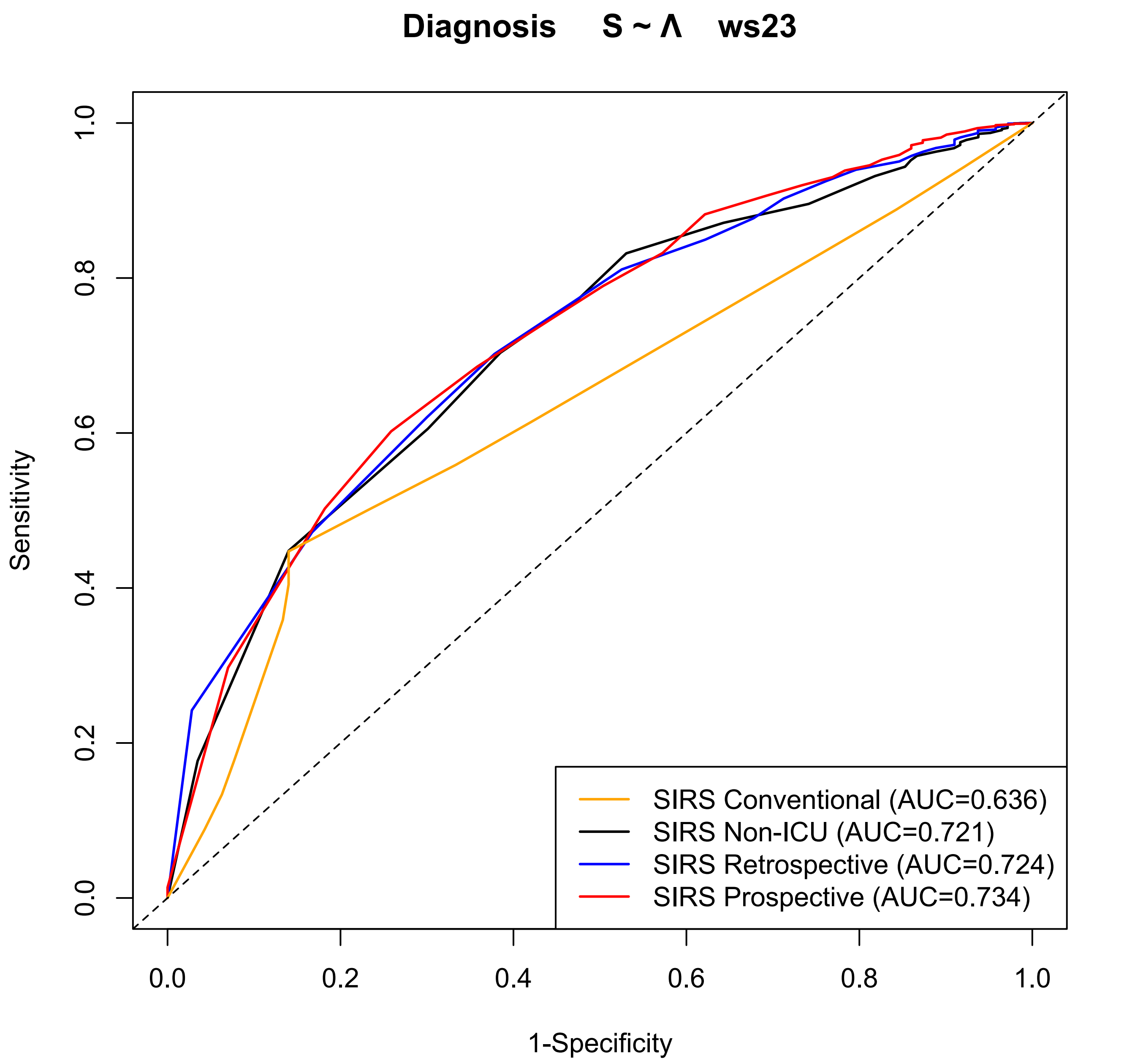
Diagnosis S ~ Λ ws23
Sensitivity
1-Specificity
0.0
0.2
0.4
0.6
0.8
1.0
SIRS Conventional (AUC=0.636)
SIRS Non-ICU (AUC=0.721)
SIRS Retrospective (AUC=0.724)
SIRS Prospective (AUC=0.734)

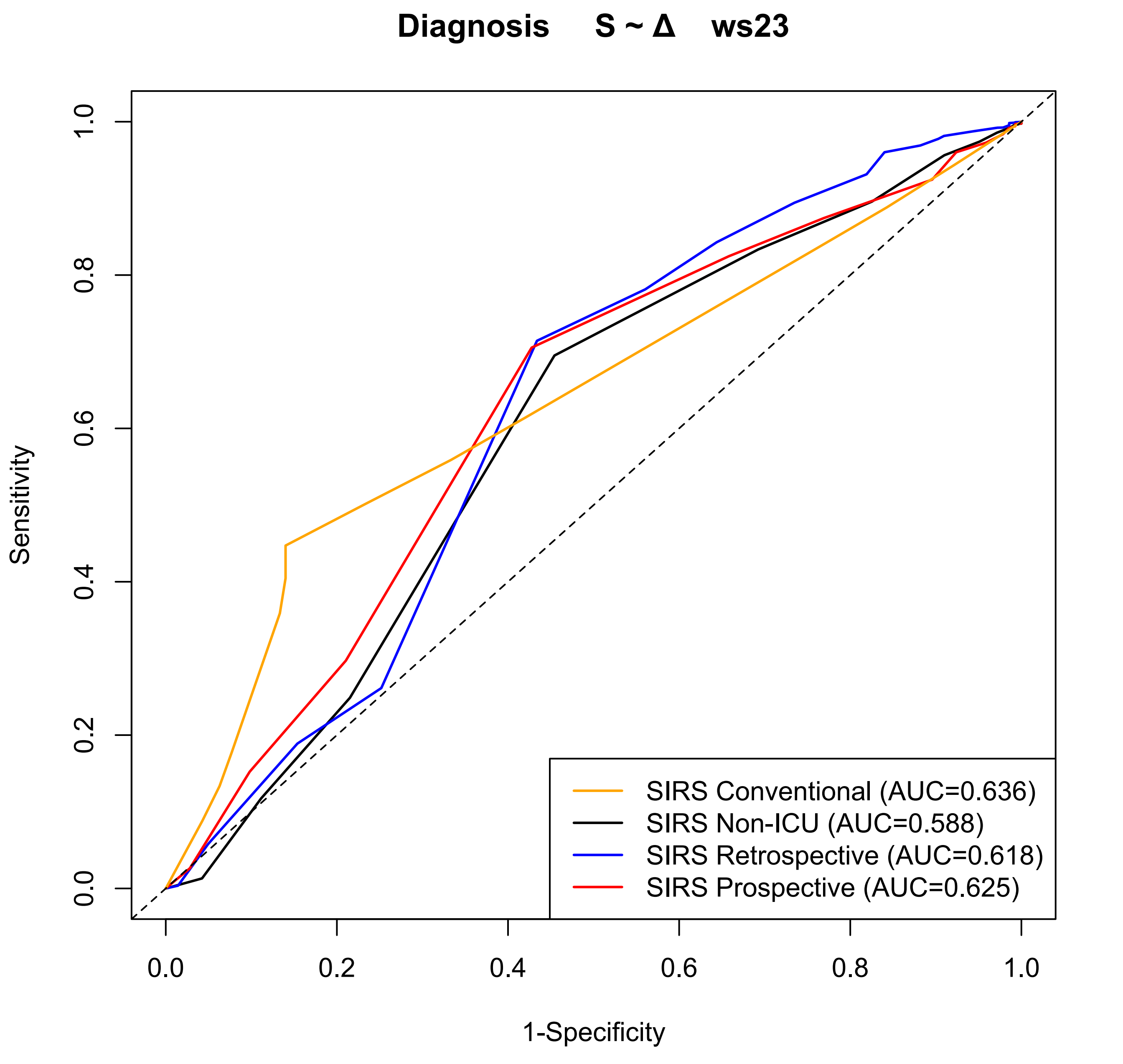
Diagnosis S ~ Δ ws23
Sensitivity
1-Specificity
0.0
0.2
0.4
0.6
0.8
1.0
SIRS Conventional (AUC=0.636)
SIRS Non-ICU (AUC=0.588)
SIRS Retrospective (AUC=0.618)
SIRS Prospective (AUC=0.625)

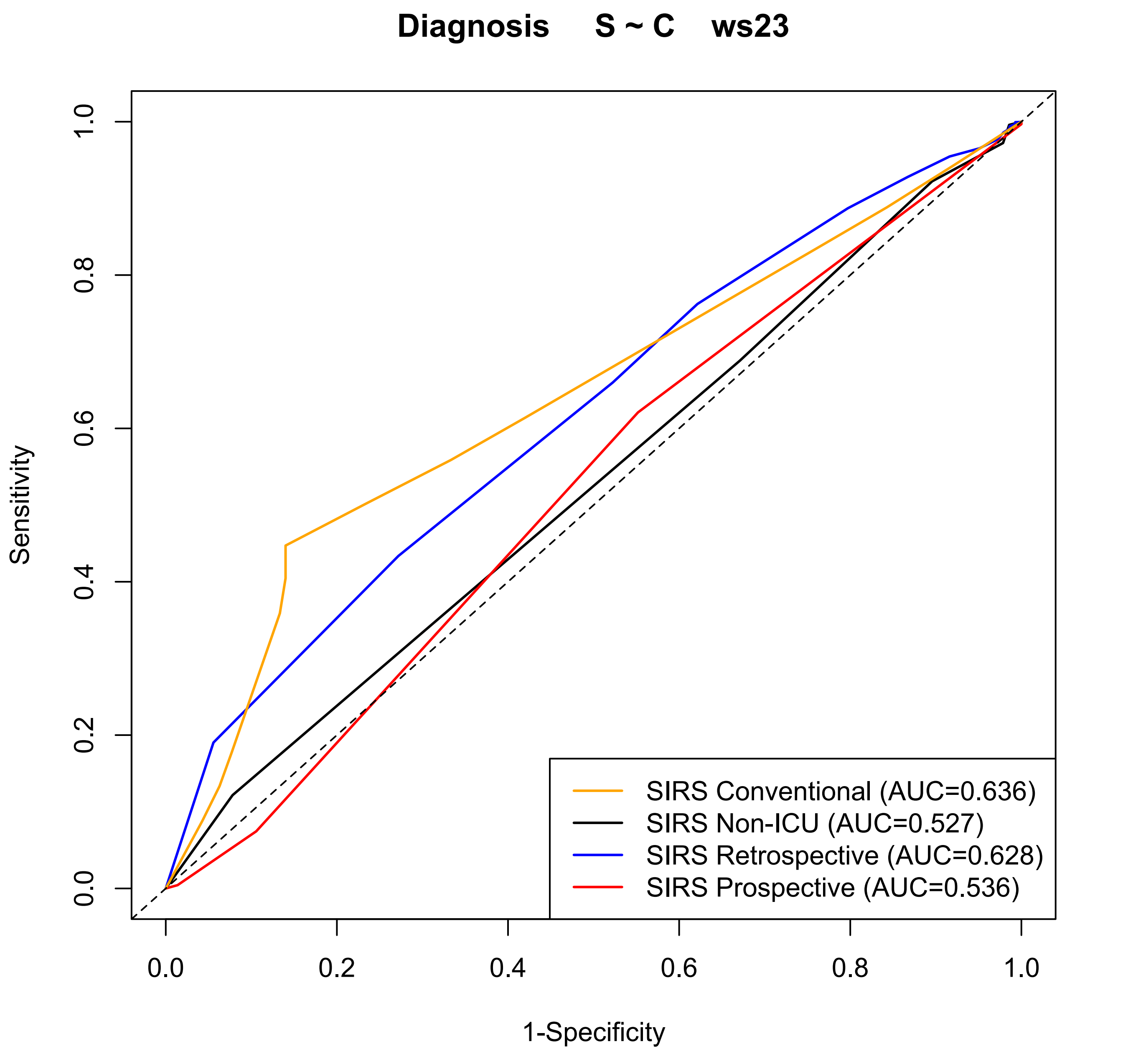
Diagnosis S ~ C ws23
Sensitivity
1-Specificity
0.0
0.2
0.4
0.6
0.8
1.0
SIRS Conventional (AUC=0.636)
SIRS Non-ICU (AUC=0.527)
SIRS Retrospective (AUC=0.628)
SIRS Prospective (AUC=0.536)

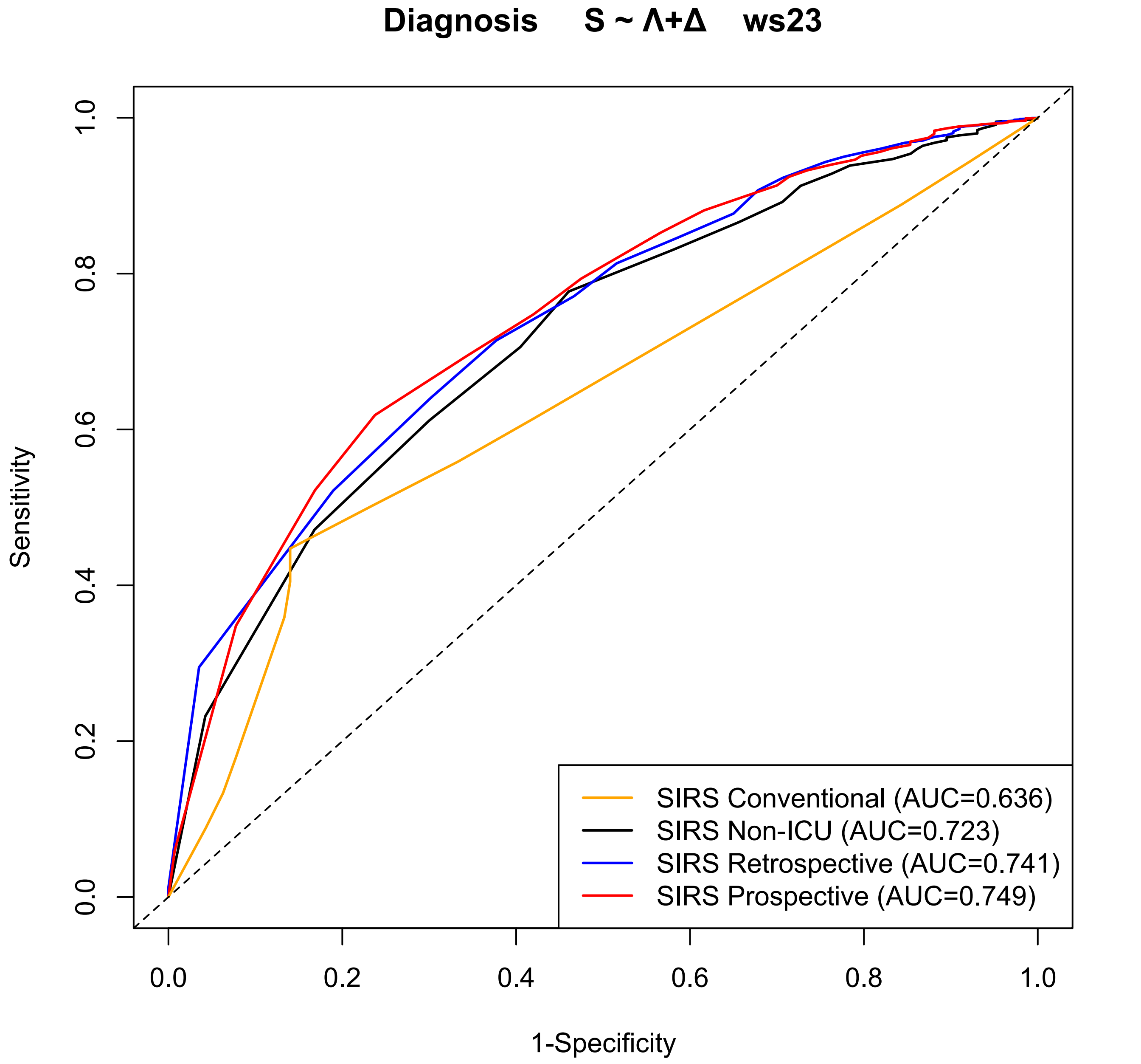

Diagnosis S ~ Λ+Δ ws23
Sensitivity
1-Specificity
0.0
0.2
0.4
0.6
0.8
1.0
SIRS Conventional (AUC=0.636)
SIRS Non-ICU (AUC=0.723)
SIRS Retrospective (AUC=0.741)
SIRS Prospective (AUC=0.749)

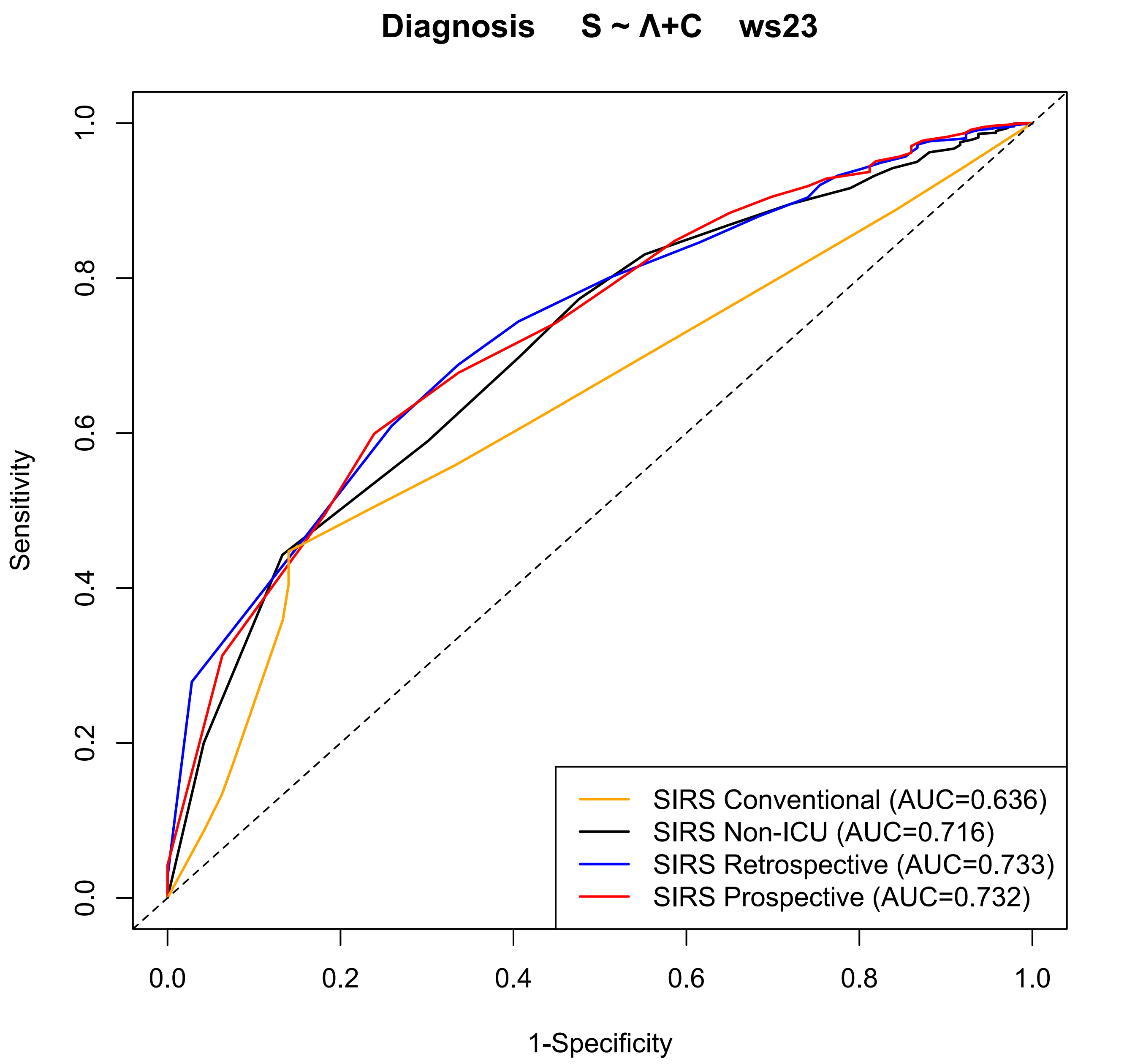
Diagnosis S ~ Λ+C ws23
Sensitivity
1-Specificity
0.0
0.2
0.4
0.6
0.8
1.0
SIRS Conventional (AUC=0.636)
SIRS Non-ICU (AUC=0.716)
SIRS Retrospective (AUC=0.733)
SIRS Prospective (AUC=0.732)

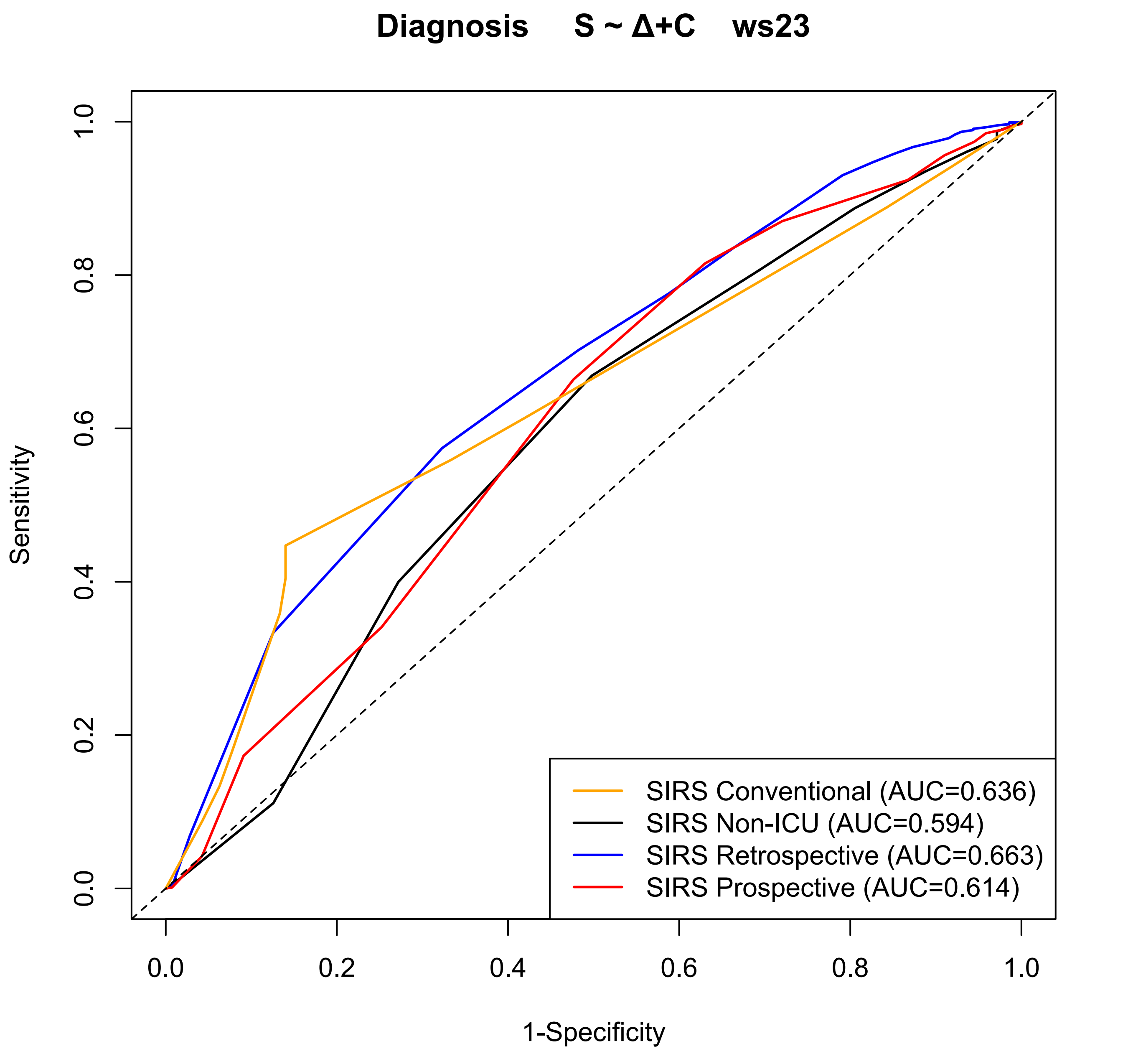

Diagnosis S ~ Δ+C ws23
Sensitivity
1-Specificity
0.0
0.2
0.4
0.6
0.8
1.0
SIRS Conventional (AUC=0.636)
SIRS Non-ICU (AUC=0.594)
SIRS Retrospective (AUC=0.663)
SIRS Prospective (AUC=0.614)

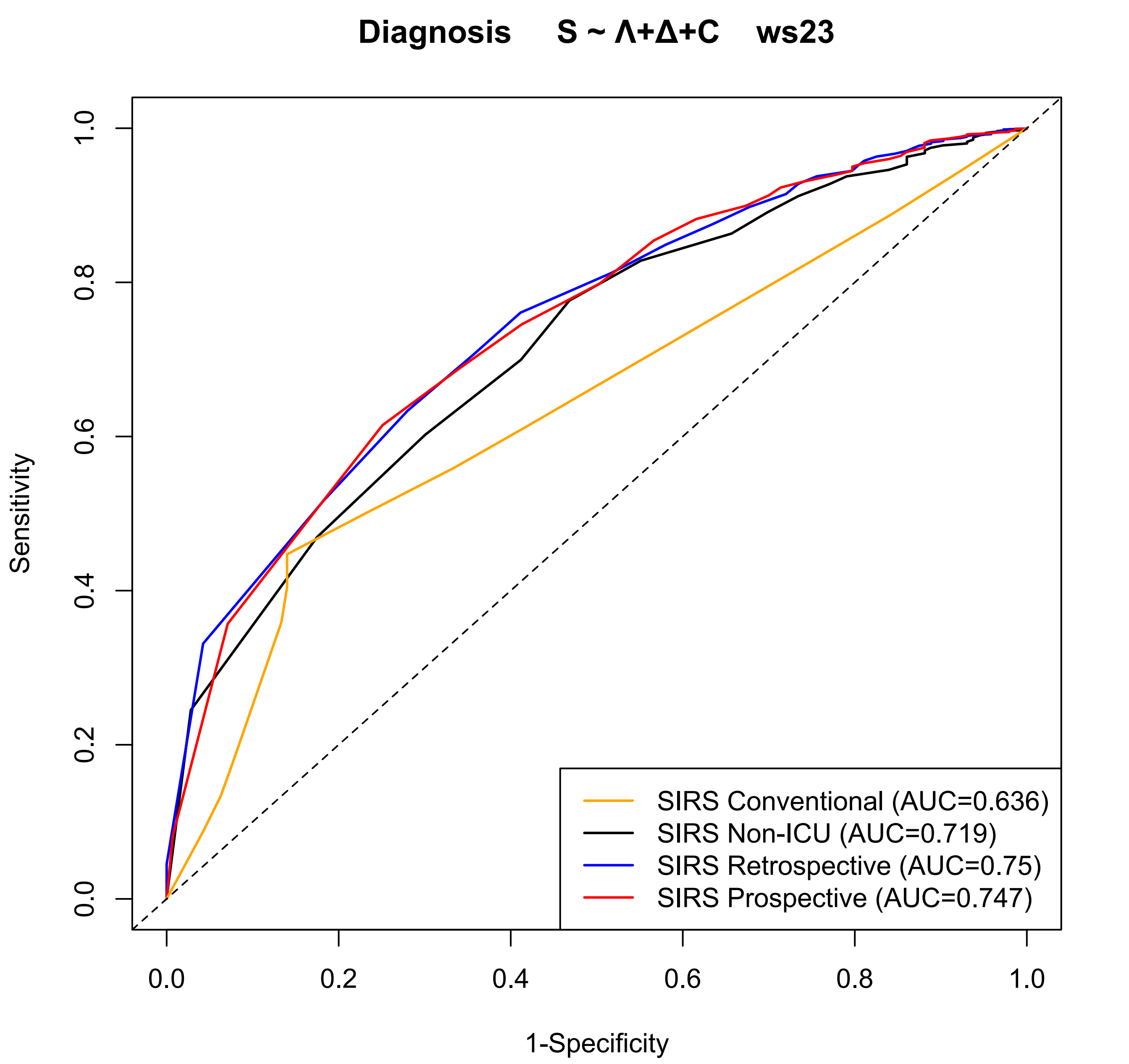
Diagnosis S ~ Λ+Δ+C ws23
Sensitivity
1-Specificity
0.0
0.2
0.4
0.6
0.8
1.0
SIRS Conventional (AUC=0.636)
SIRS Non-ICU (AUC=0.719)
SIRS Retrospective (AUC=0.75)
SIRS Prospective (AUC=0.747)

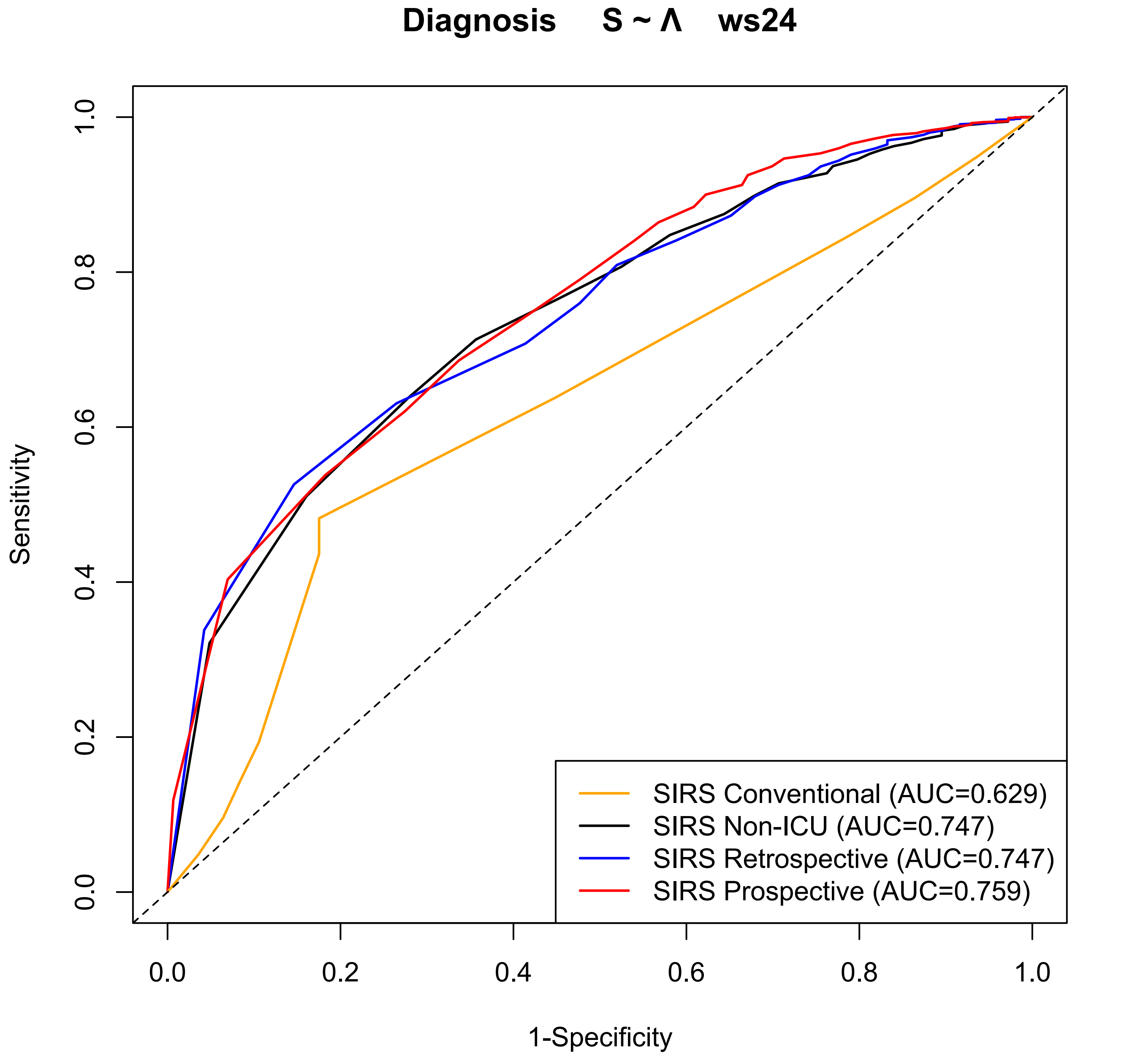
Diagnosis S ~ Λ ws24
Sensitivity
1-Specificity
0.0
0.2
0.4
0.6
0.8
1.0
SIRS Conventional (AUC=0.629)
SIRS Non-ICU (AUC=0.747)
SIRS Retrospective (AUC=0.747)
SIRS Prospective (AUC=0.759)

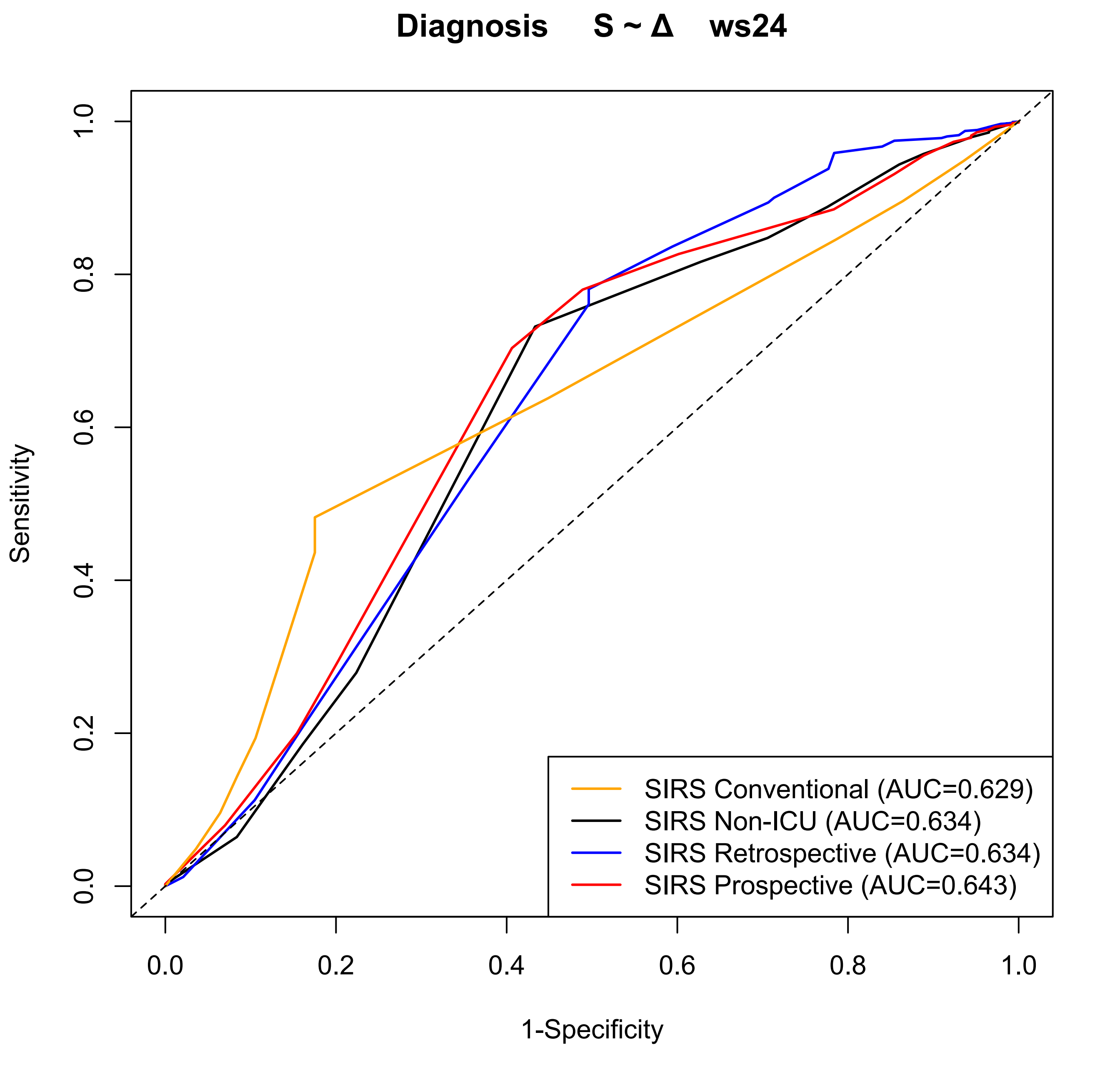

Diagnosis S ~ Δ ws24
Sensitivity
1-Specificity
0.0
0.2
0.4
0.6
0.8
1.0
SIRS Conventional (AUC=0.629)
SIRS Non-ICU (AUC=0.634)
SIRS Retrospective (AUC=0.634)
SIRS Prospective (AUC=0.643)

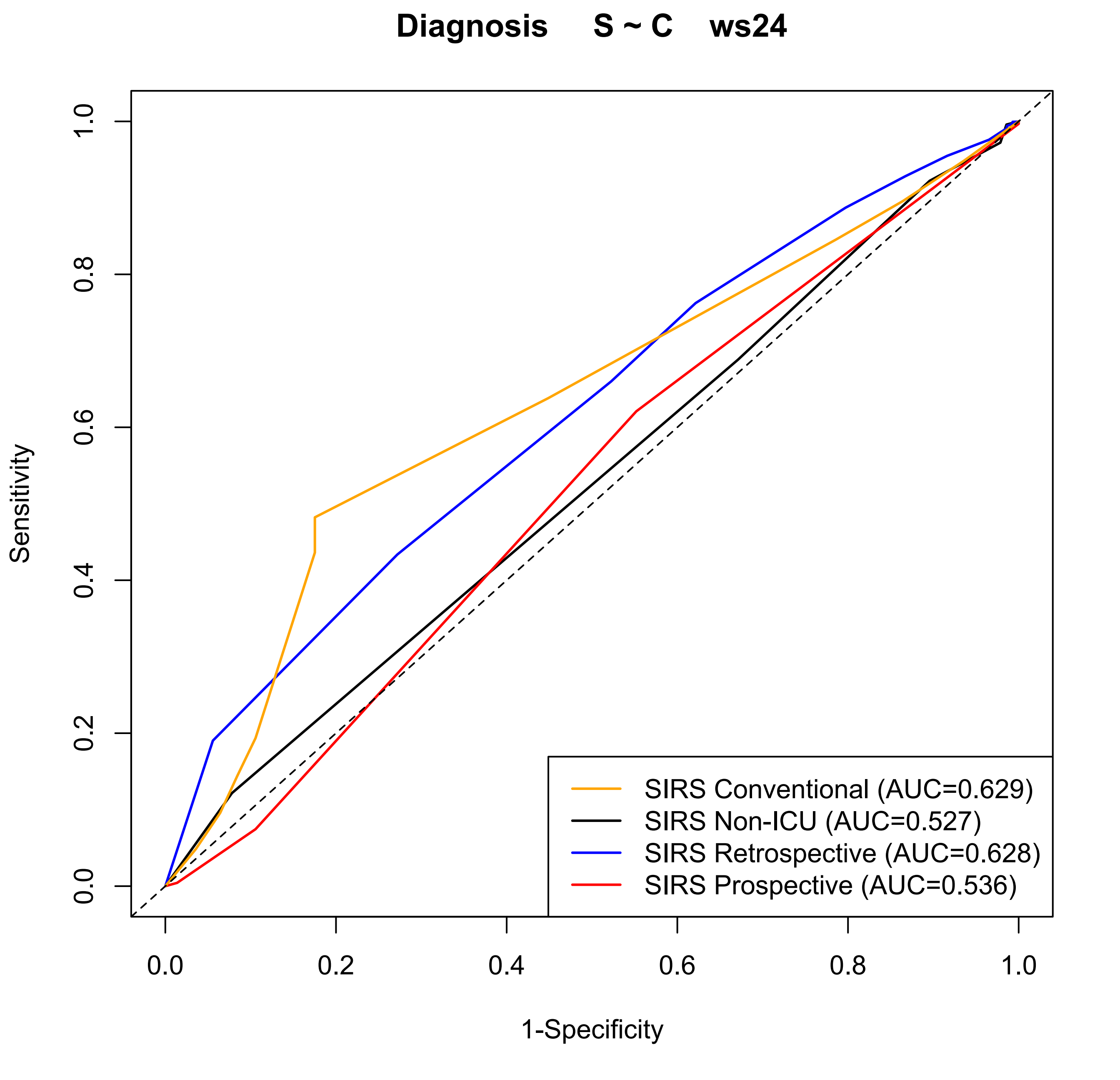

Diagnosis S ~ C ws24
Sensitivity
1-Specificity
0.0
0.2
0.4
0.6
0.8
1.0
SIRS Conventional (AUC=0.629)
SIRS Non-ICU (AUC=0.527)
SIRS Retrospective (AUC=0.628)
SIRS Prospective (AUC=0.536)

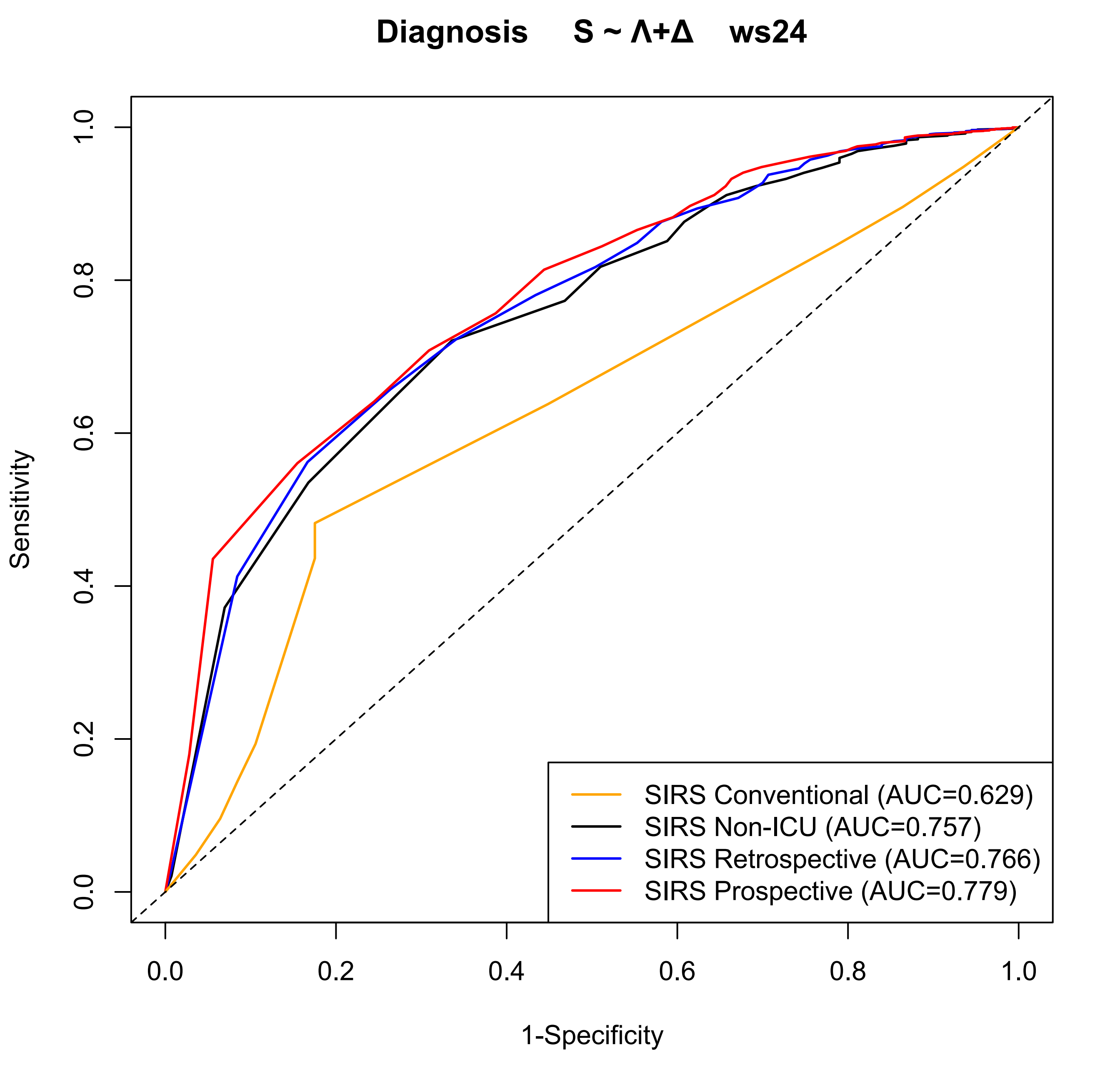
Diagnosis S ~ Λ+Δ ws24
Sensitivity
1-Specificity
0.0
0.2
0.4
0.6
0.8
1.0
0.0
0.2
0.4
0.6
0.8
1.0
SIRS Conventional (AUC=0.629)
SIRS Non-ICU (AUC=0.757)
SIRS Retrospective (AUC=0.766)
SIRS Prospective (AUC=0.779)

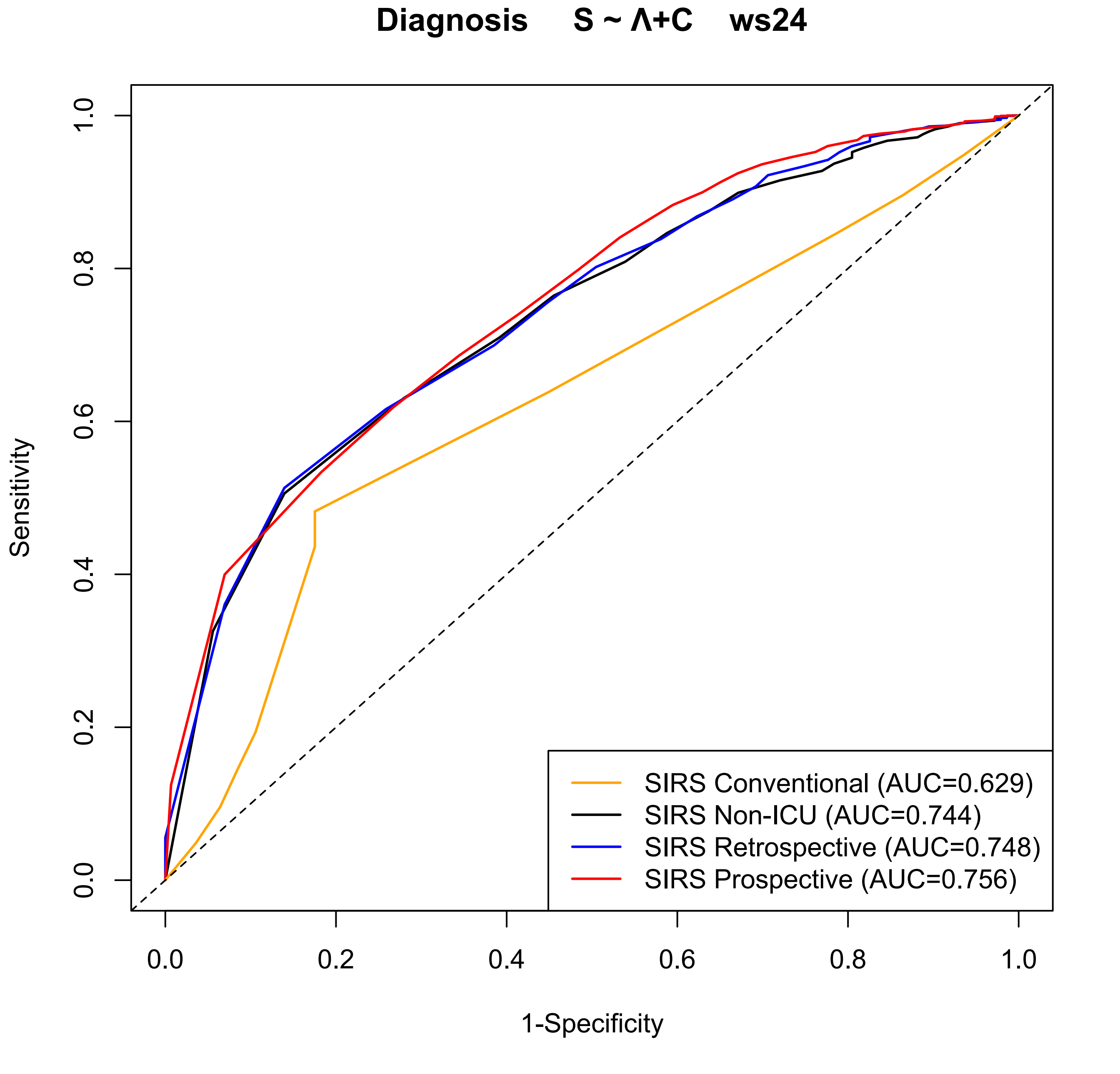

Diagnosis S ~ Λ+C ws24
Sensitivity
1-Specificity
0.0
0.2
0.4
0.6
0.8
1.0
SIRS Conventional (AUC=0.629)
SIRS Non-ICU (AUC=0.744)
SIRS Retrospective (AUC=0.748)
SIRS Prospective (AUC=0.756)

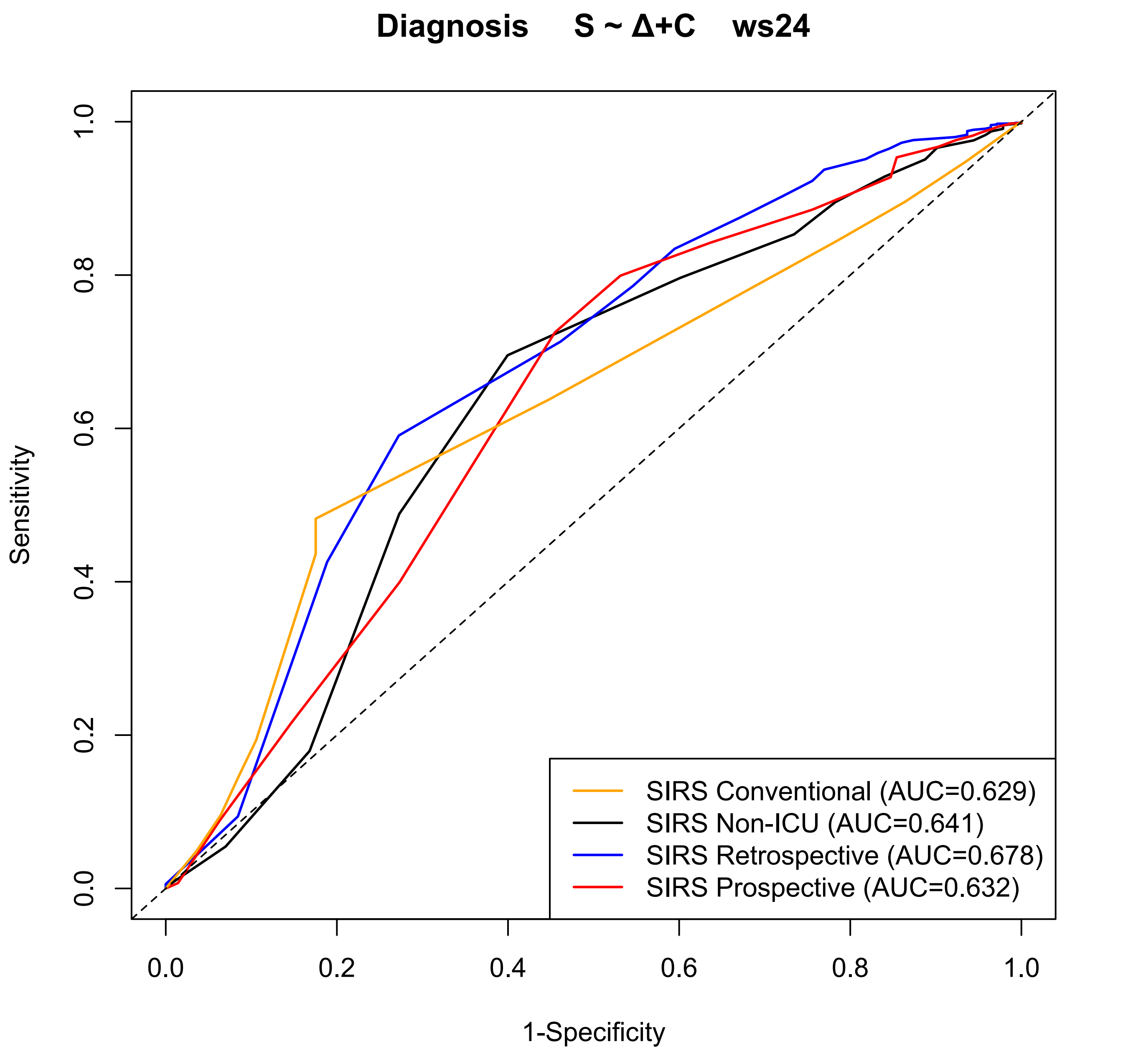

Diagnosis S ~ Δ+C ws24
Sensitivity
1-Specificity
0.0
0.2
0.4
0.6
0.8
1.0
SIRS Conventional (AUC=0.629)
SIRS Non-ICU (AUC=0.641)
SIRS Retrospective (AUC=0.678)
SIRS Prospective (AUC=0.632)

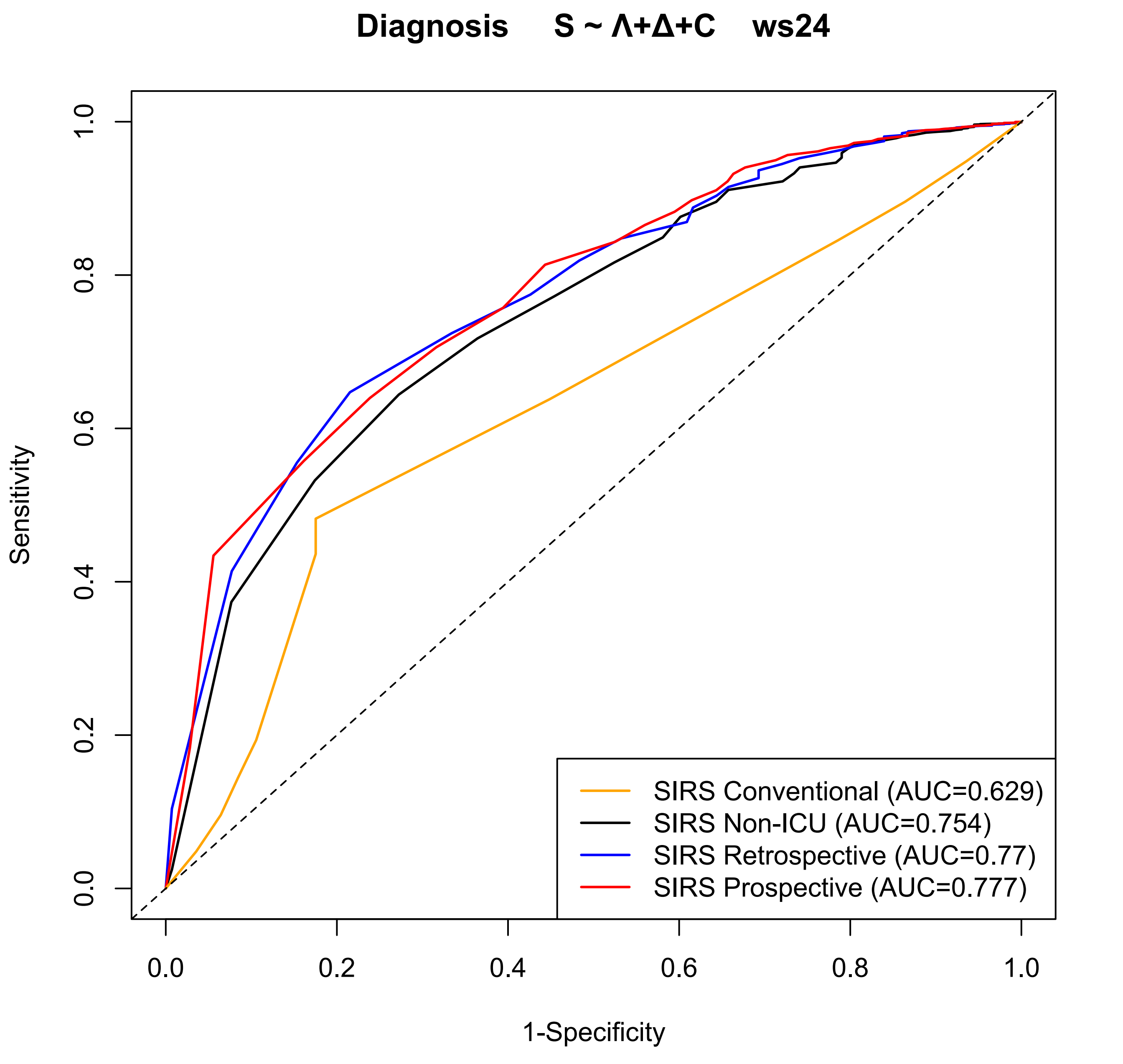

Diagnosis S ~ Λ+Δ+C ws24
Sensitivity
1-Specificity
0.0
0.2
0.4
0.6
0.8
1.0
SIRS Conventional (AUC=0.629)
SIRS Non-ICU (AUC=0.754)
SIRS Retrospective (AUC=0.77)
SIRS Prospective (AUC=0.777)

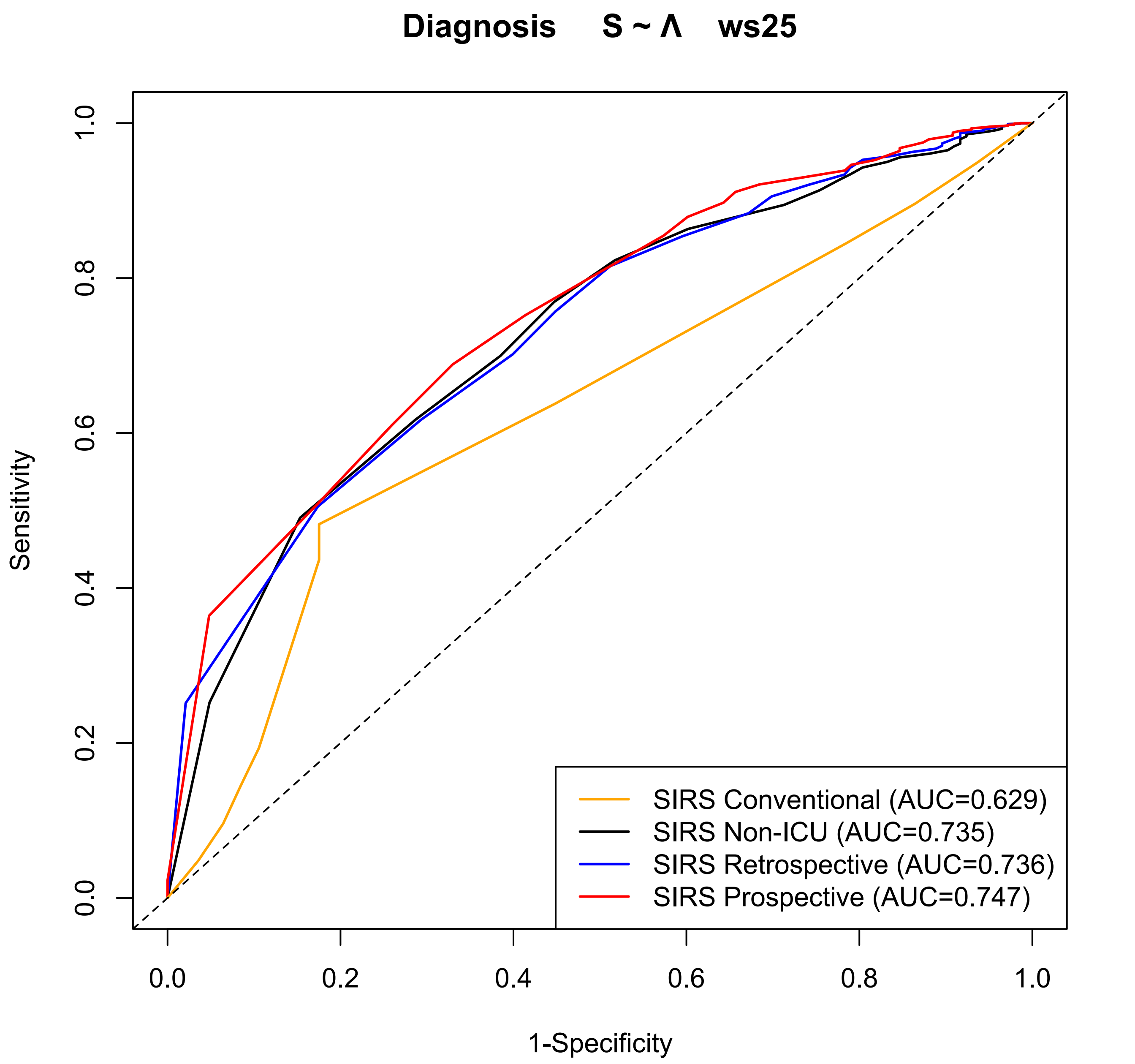

Diagnosis S ~ Λ ws25
Sensitivity
1-Specificity
0.0
0.2
0.4
0.6
0.8
1.0
SIRS Conventional (AUC=0.629)
SIRS Non-ICU (AUC=0.735)
SIRS Retrospective (AUC=0.736)
SIRS Prospective (AUC=0.747)

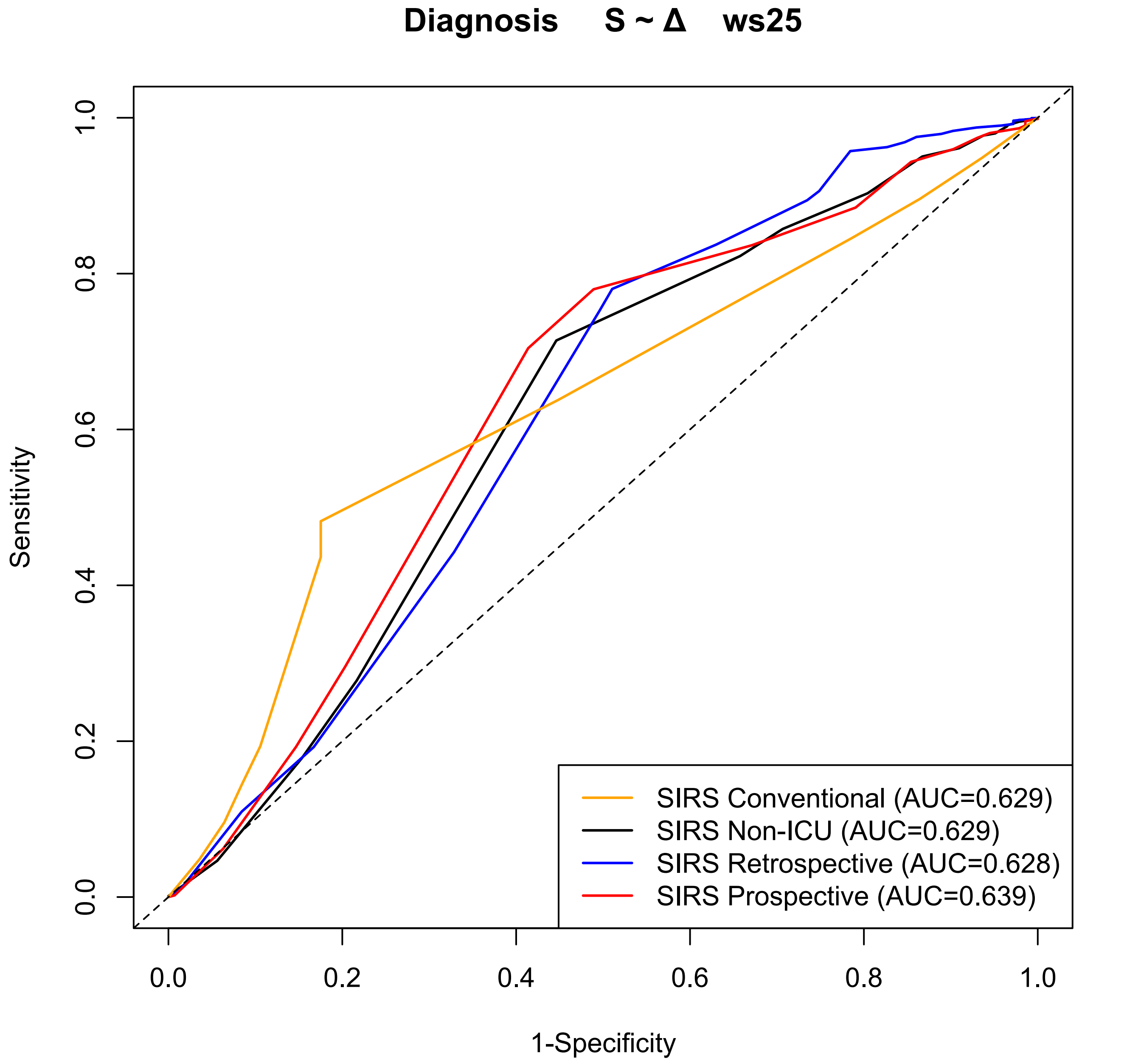

Diagnosis S ~ Δ ws25
Sensitivity
1-Specificity
0.0
0.2
0.4
0.6
0.8
1.0
SIRS Conventional (AUC=0.629)
SIRS Non-ICU (AUC=0.629)
SIRS Retrospective (AUC=0.628)
SIRS Prospective (AUC=0.639)

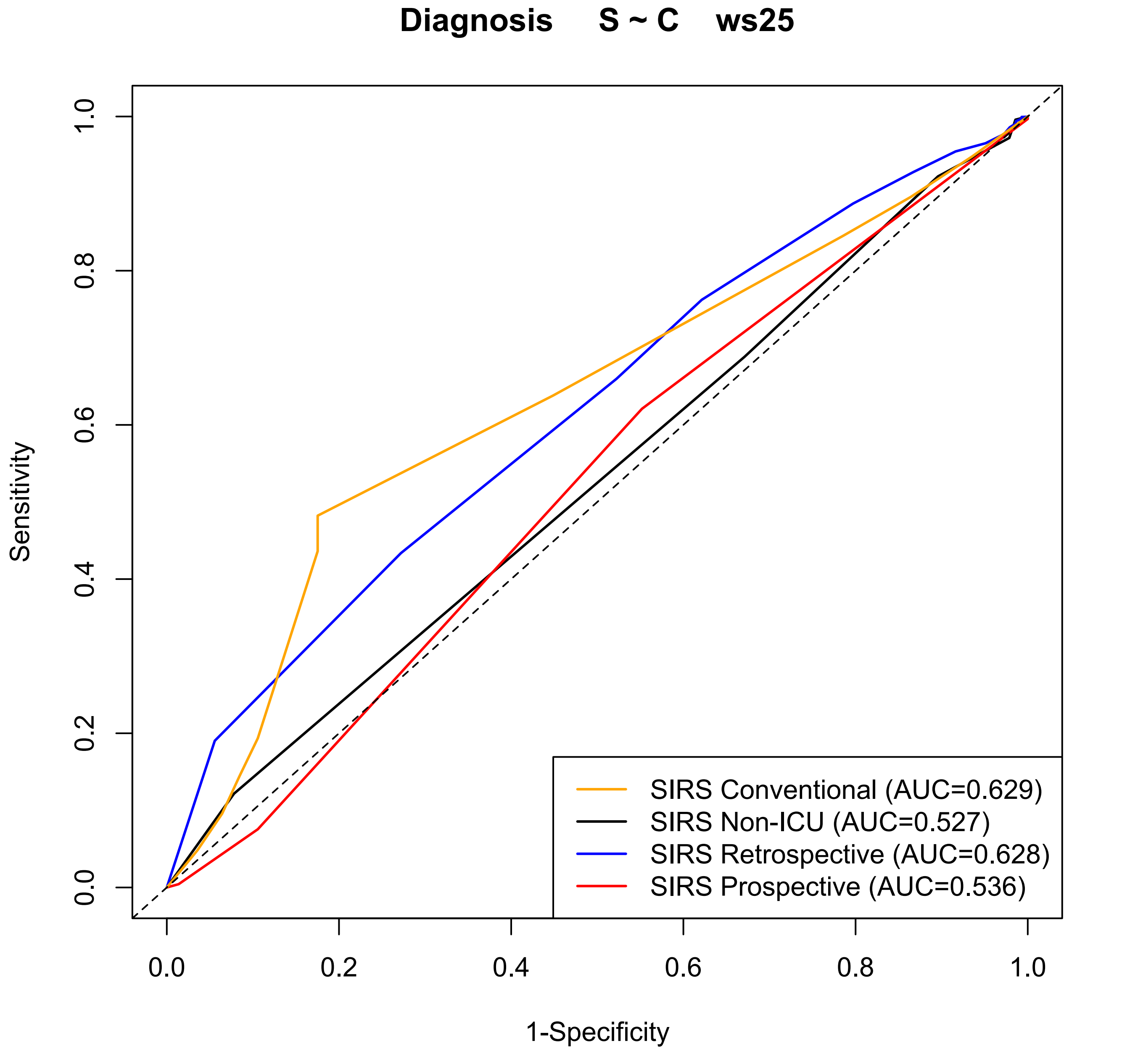
Diagnosis S ~ C ws25
Sensitivity
1-Specificity
0.0
0.2
0.4
0.6
0.8
1.0
SIRS Conventional (AUC=0.629)
SIRS Non-ICU (AUC=0.527)
SIRS Retrospective (AUC=0.628)
SIRS Prospective (AUC=0.536)

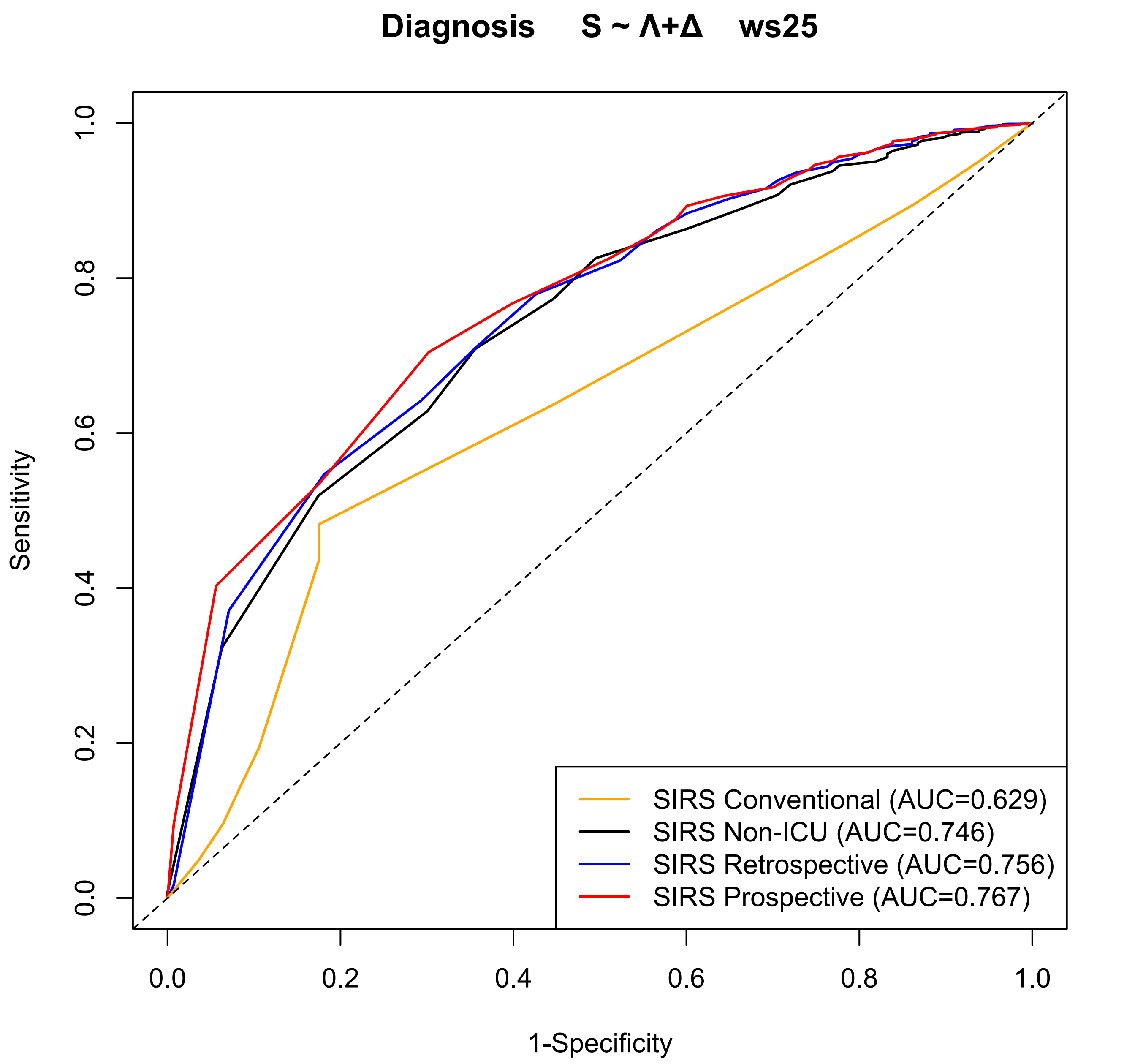
Diagnosis S ~ Λ+Δ ws25
Sensitivity
1-Specificity
0.0
0.2
0.4
0.6
0.8
1.0
SIRS Conventional (AUC=0.629)
SIRS Non-ICU (AUC=0.746)
SIRS Retrospective (AUC=0.756)
SIRS Prospective (AUC=0.767)

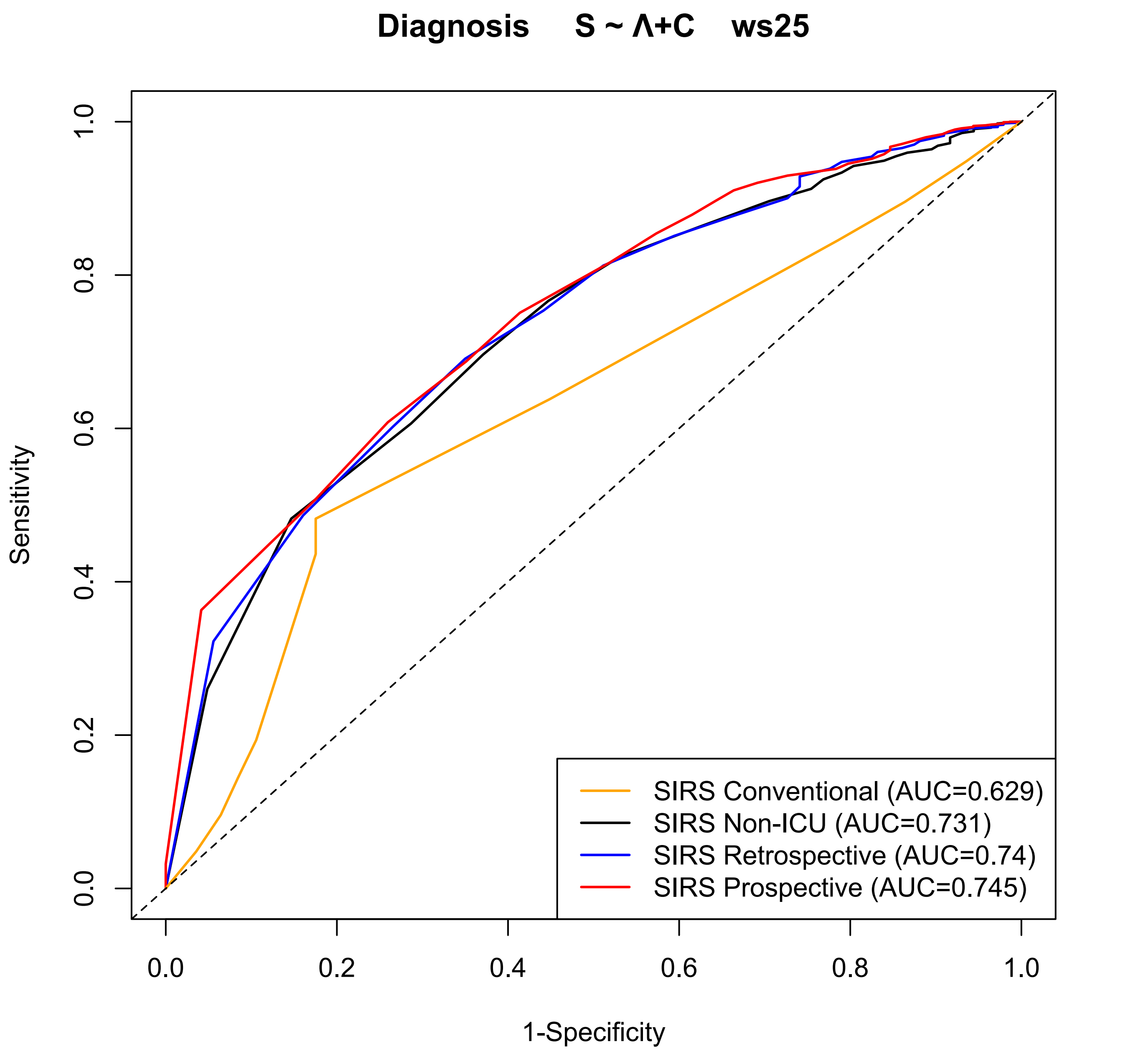
Diagnosis S ~ Λ+C ws25
Sensitivity
1-Specificity
0.0
0.2
0.4
0.6
0.8
1.0
SIRS Conventional (AUC=0.629)
SIRS Non-ICU (AUC=0.731)
SIRS Retrospective (AUC=0.74)
SIRS Prospective (AUC=0.745)

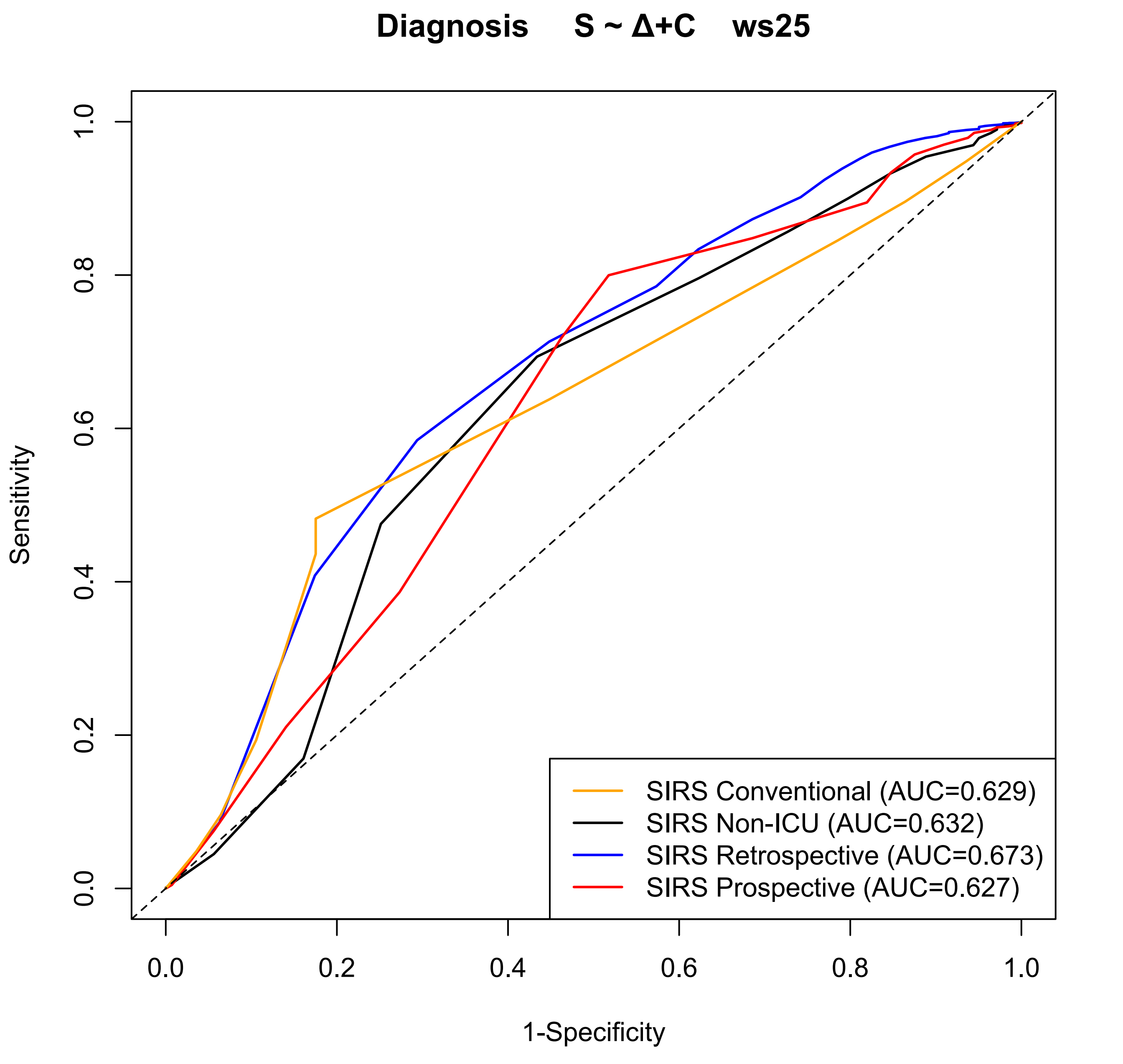
Diagnosis S ~ Δ+C ws25
Sensitivity
1-Specificity
0.0
0.2
0.4
0.6
0.8
1.0
SIRS Conventional (AUC=0.629)
SIRS Non-ICU (AUC=0.632)
SIRS Retrospective (AUC=0.673)
SIRS Prospective (AUC=0.627)

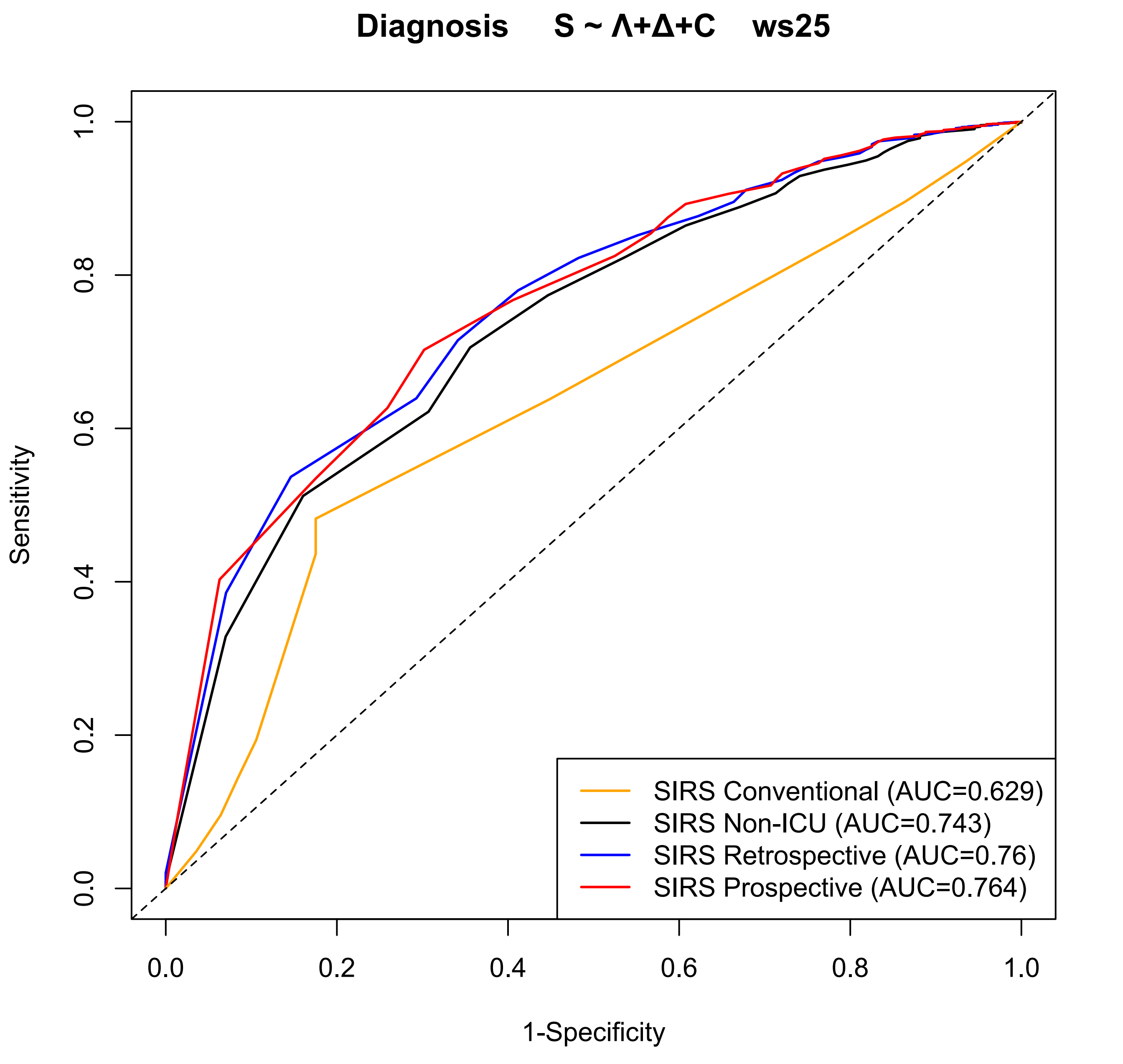
Diagnosis S ~ Λ+Δ+C ws25
Sensitivity
1-Specificity
0.0
0.2
0.4
0.6
0.8
1.0
SIRS Conventional (AUC=0.629)
SIRS Non-ICU (AUC=0.743)
SIRS Retrospective (AUC=0.76)
SIRS Prospective (AUC=0.764)

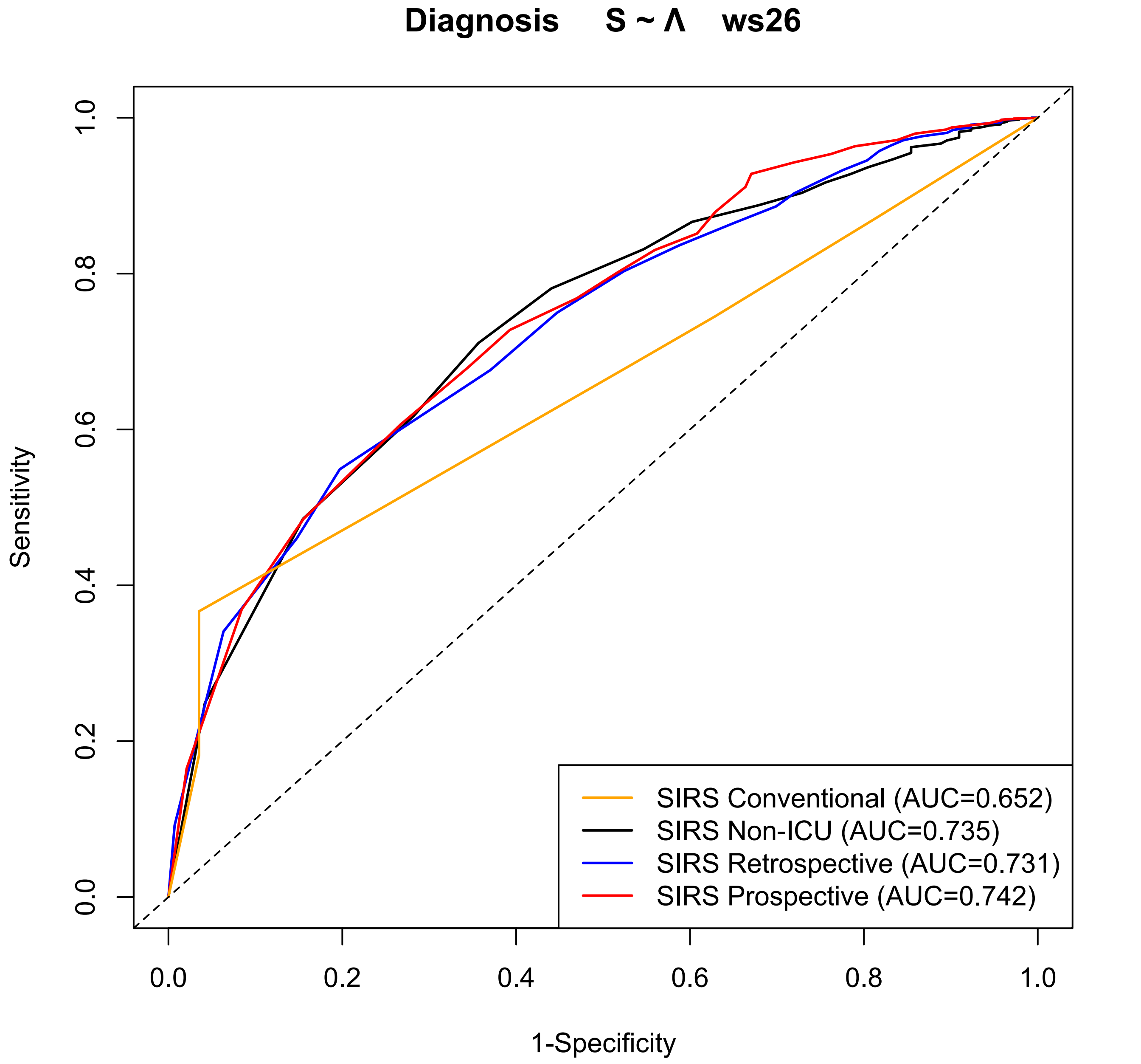

Diagnosis S ~ Λ ws26
Sensitivity
1-Specificity
0.0
0.2
0.4
0.6
0.8
1.0
0.0
0.2
0.4
0.6
0.8
1.0
SIRS Conventional (AUC=0.652)
SIRS Non-ICU (AUC=0.735)
SIRS Retrospective (AUC=0.731)
SIRS Prospective (AUC=0.742)

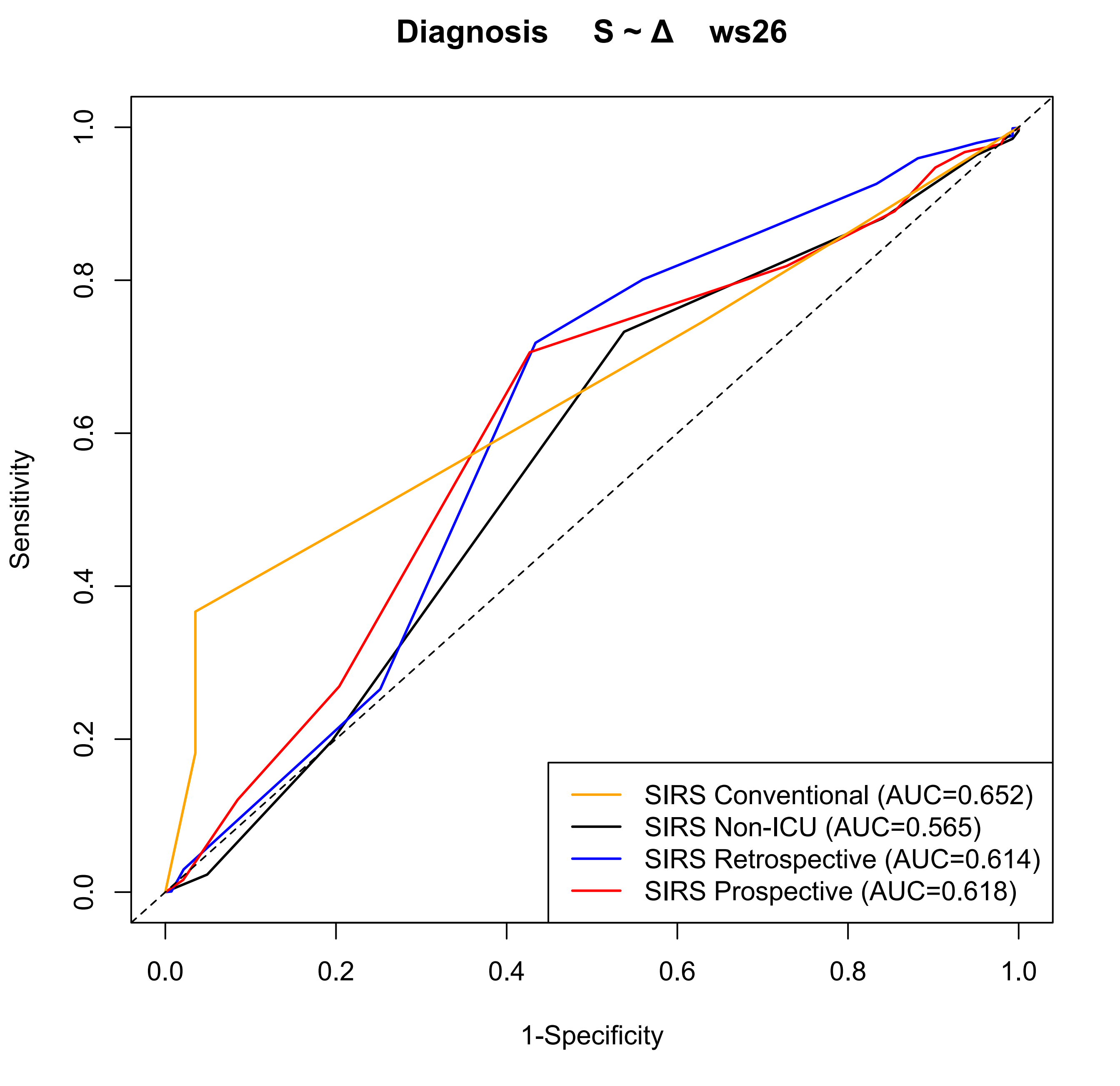

Diagnosis S ~ Δ ws26
Sensitivity
1-Specificity
0.0
0.2
0.4
0.6
0.8
1.0
SIRS Conventional (AUC=0.652)
SIRS Non-ICU (AUC=0.565)
SIRS Retrospective (AUC=0.614)
SIRS Prospective (AUC=0.618)

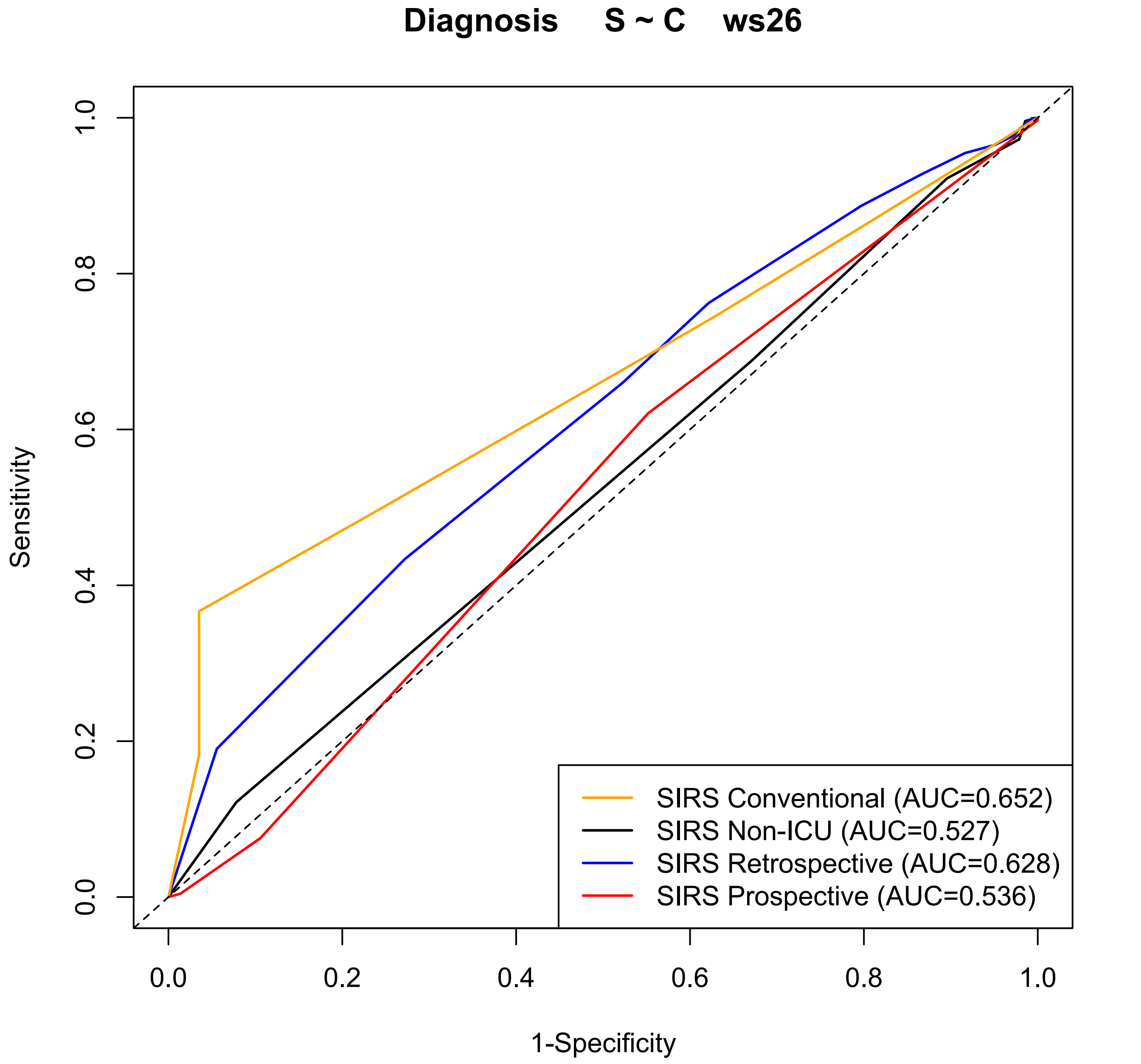
Diagnosis S ~ C ws26
Sensitivity
1-Specificity
0.0
0.2
0.4
0.6
0.8
1.0
SIRS Conventional (AUC=0.652)
SIRS Non-ICU (AUC=0.527)
SIRS Retrospective (AUC=0.628)
SIRS Prospective (AUC=0.536)

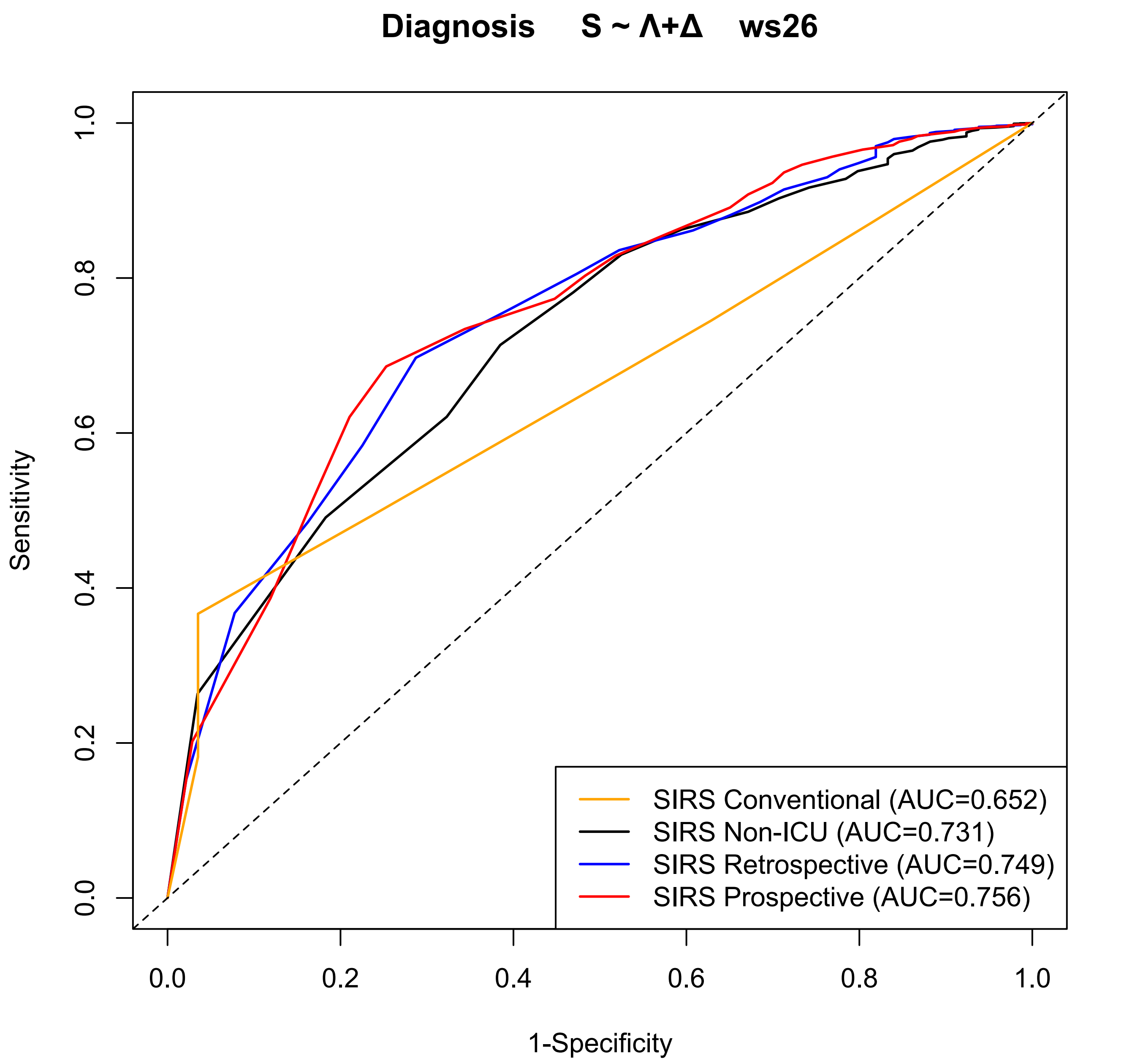

Diagnosis S ~ Λ+Δ ws26
Sensitivity
1-Specificity
0.0
0.2
0.4
0.6
0.8
1.0
SIRS Conventional (AUC=0.652)
SIRS Non-ICU (AUC=0.731)
SIRS Retrospective (AUC=0.749)
SIRS Prospective (AUC=0.756)

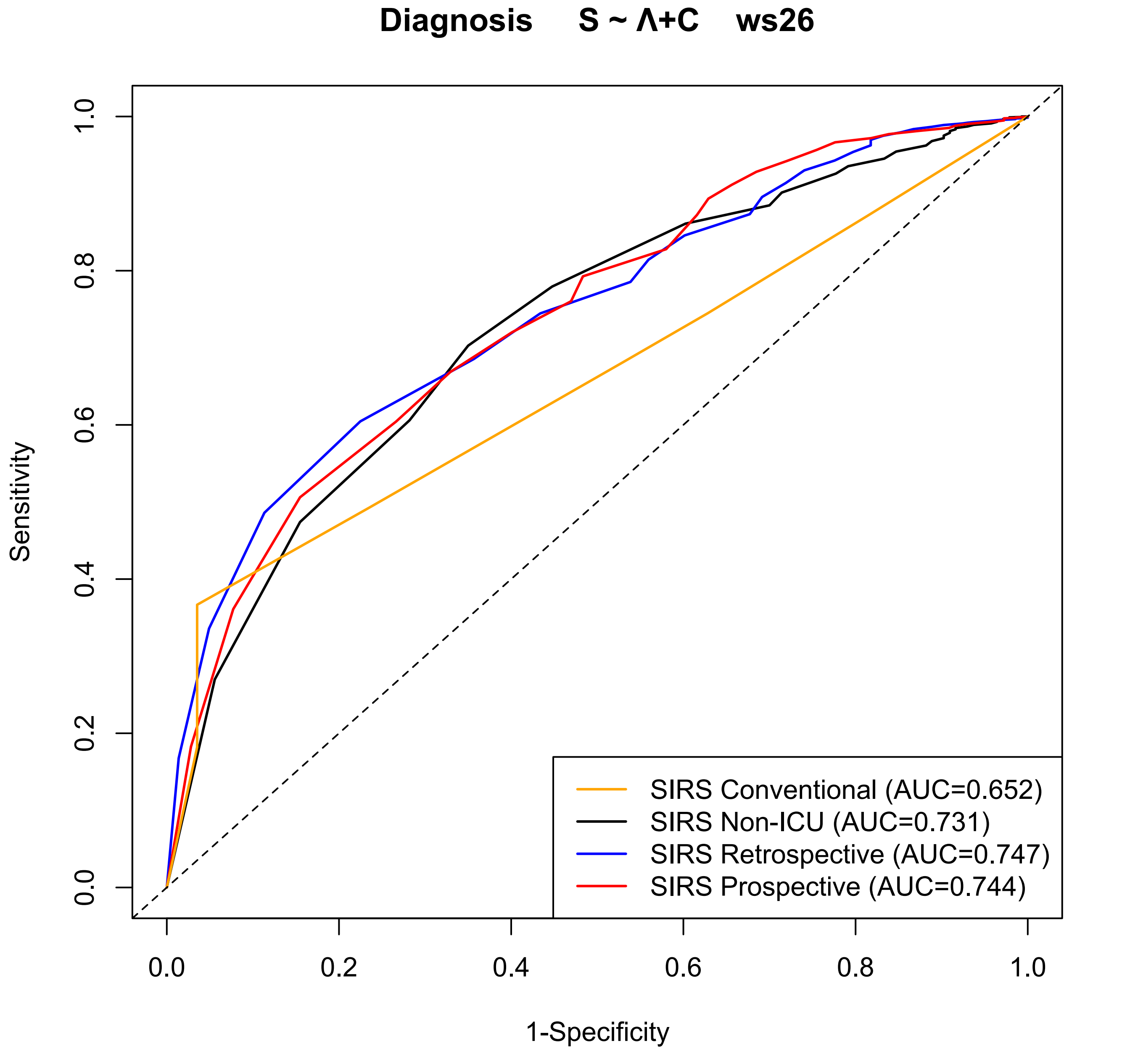
Diagnosis S ~ Λ+C ws26
Sensitivity
1-Specificity
0.0
0.2
0.4
0.6
0.8
1.0
SIRS Conventional (AUC=0.652)
SIRS Non-ICU (AUC=0.731)
SIRS Retrospective (AUC=0.747)
SIRS Prospective (AUC=0.744)

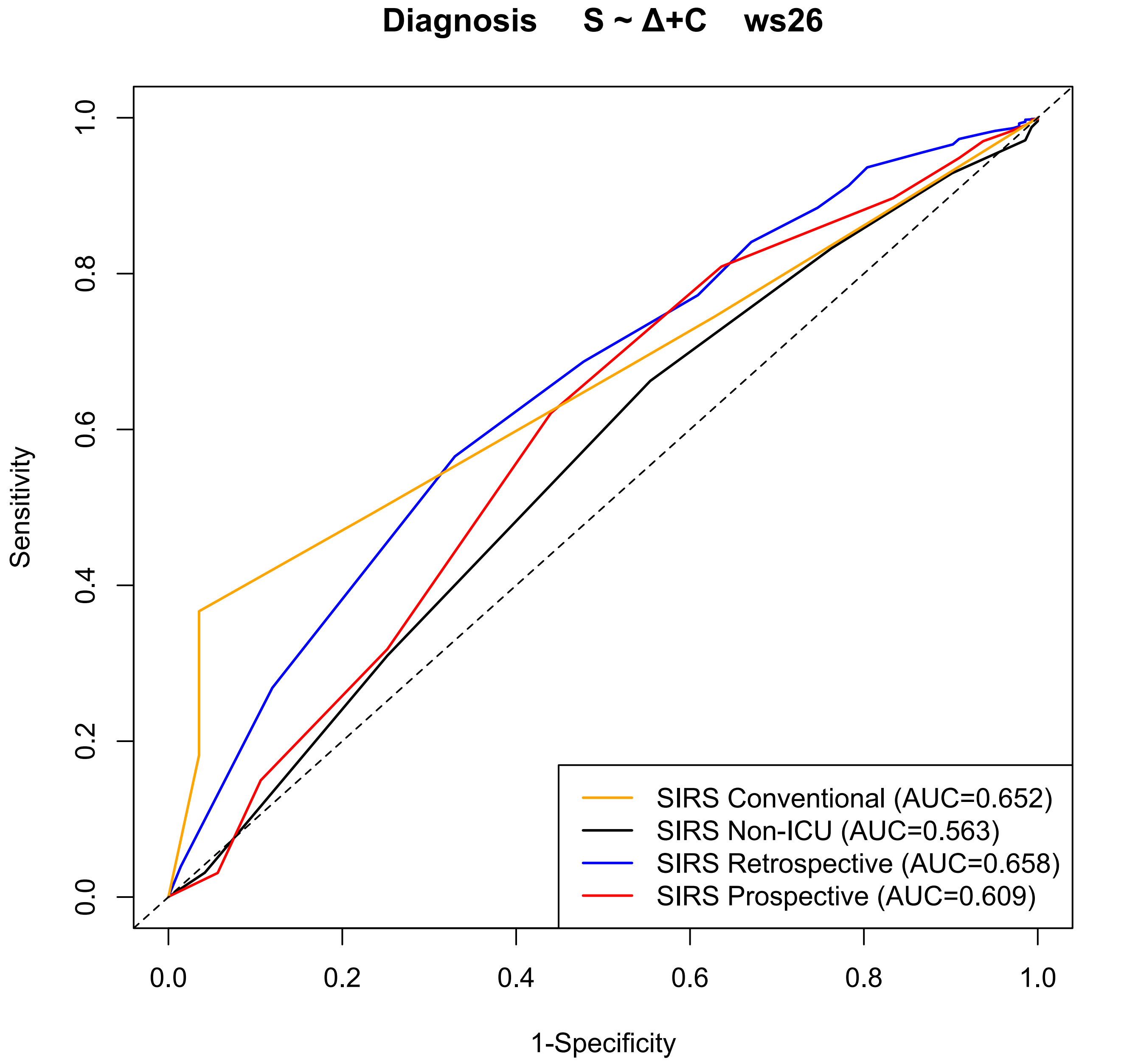

Diagnosis S ~ Δ+C ws26
Sensitivity
1-Specificity
0.0
0.2
0.4
0.6
0.8
1.0
SIRS Conventional (AUC=0.652)
SIRS Non-ICU (AUC=0.563)
SIRS Retrospective (AUC=0.658)
SIRS Prospective (AUC=0.609)

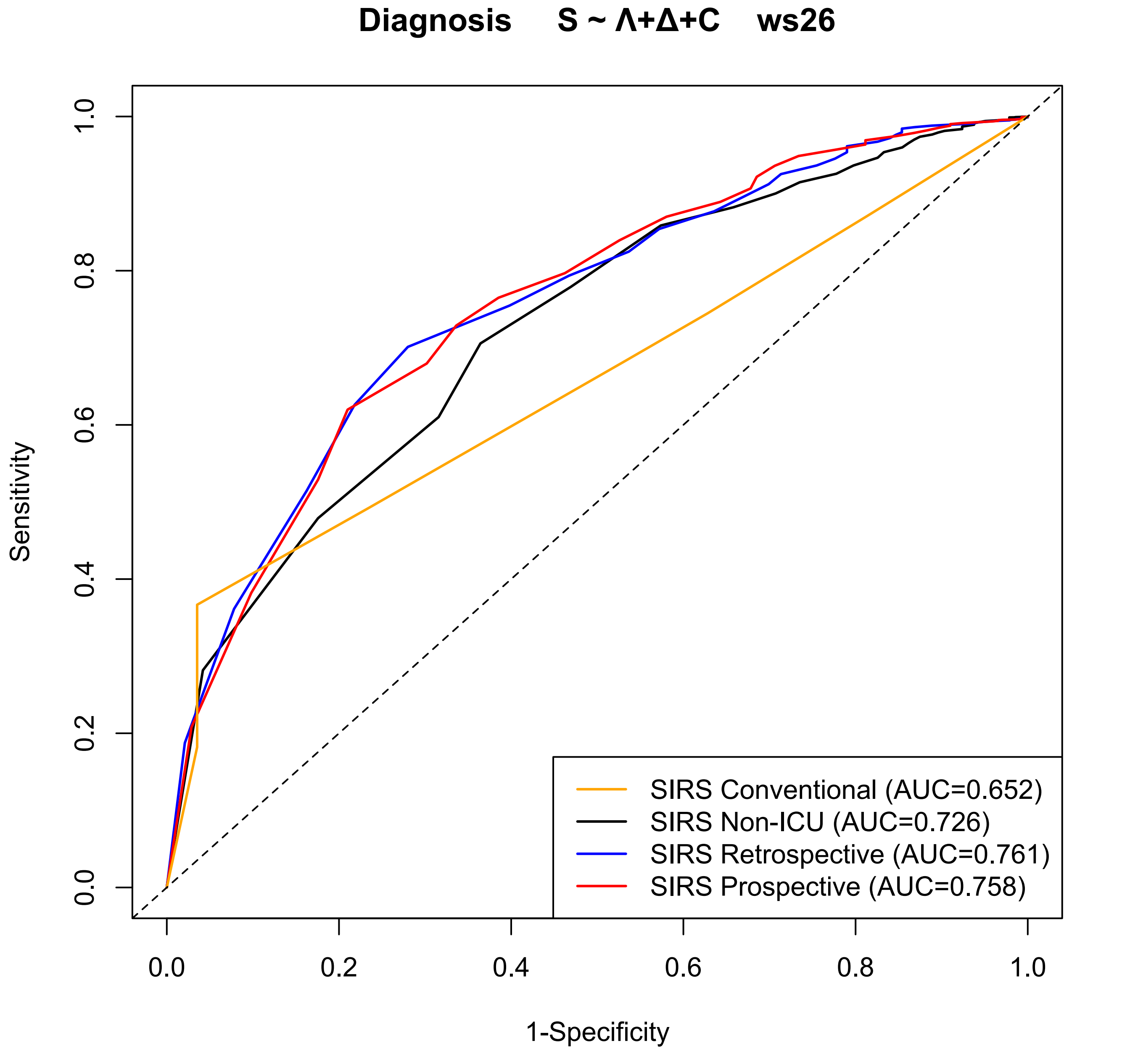
Diagnosis S ~ Λ+Δ+C ws26
Sensitivity
1-Specificity
0.0
0.2
0.4
0.6
0.8
1.0
SIRS Conventional (AUC=0.652)
SIRS Non-ICU (AUC=0.726)
SIRS Retrospective (AUC=0.761)
SIRS Prospective (AUC=0.758)

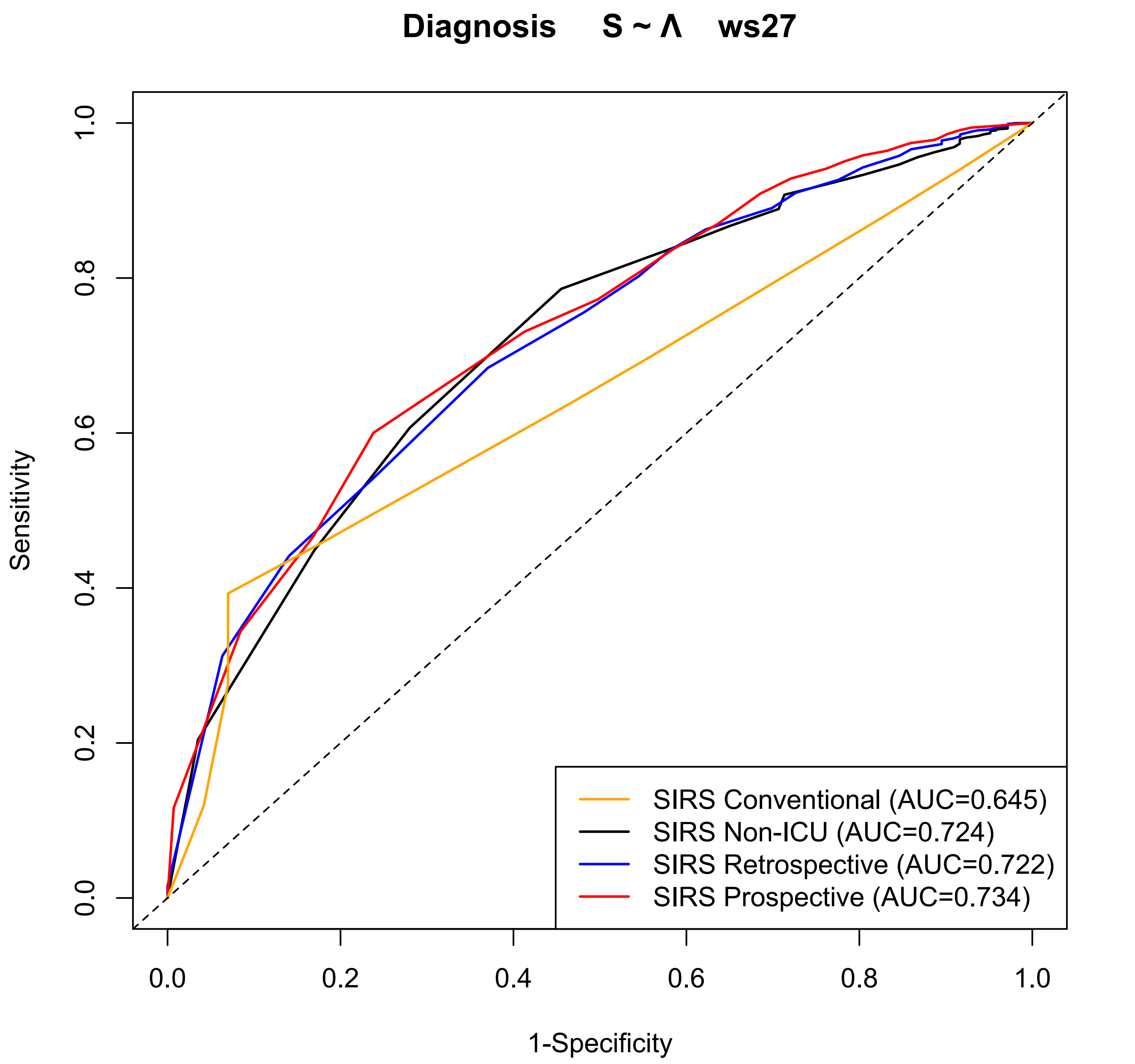
Diagnosis S ~ Λ ws27
Sensitivity
1-Specificity
0.0
0.2
0.4
0.6
0.8
1.0
0.0
0.2
0.4
0.6
0.8
1.0
SIRS Conventional (AUC=0.645)
SIRS Non-ICU (AUC=0.724)
SIRS Retrospective (AUC=0.722)
SIRS Prospective (AUC=0.734)

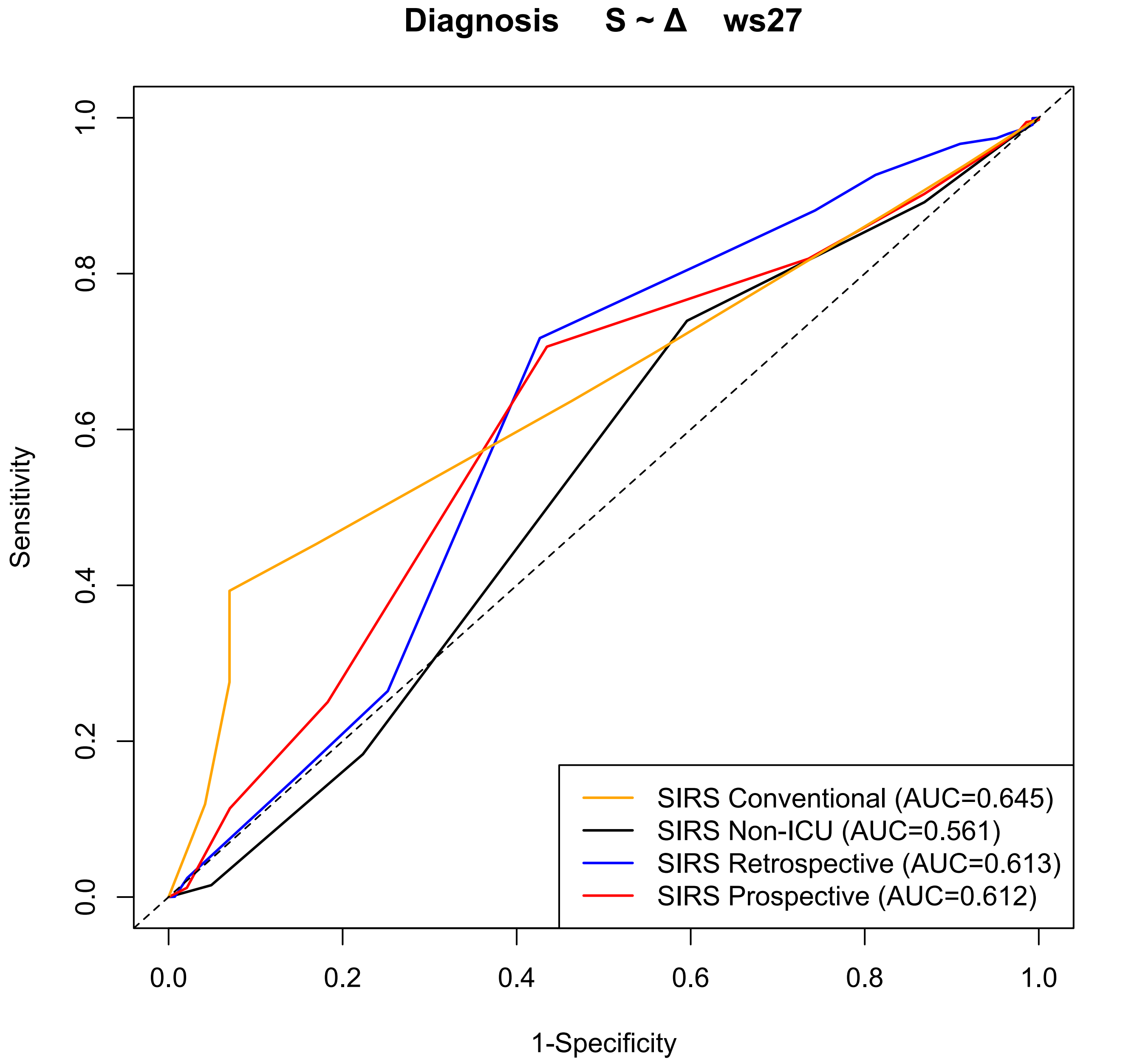

Diagnosis S ~ Δ ws27
Sensitivity
1-Specificity
0.0
0.2
0.4
0.6
0.8
1.0
SIRS Conventional (AUC=0.645)
SIRS Non-ICU (AUC=0.561)
SIRS Retrospective (AUC=0.613)
SIRS Prospective (AUC=0.612)

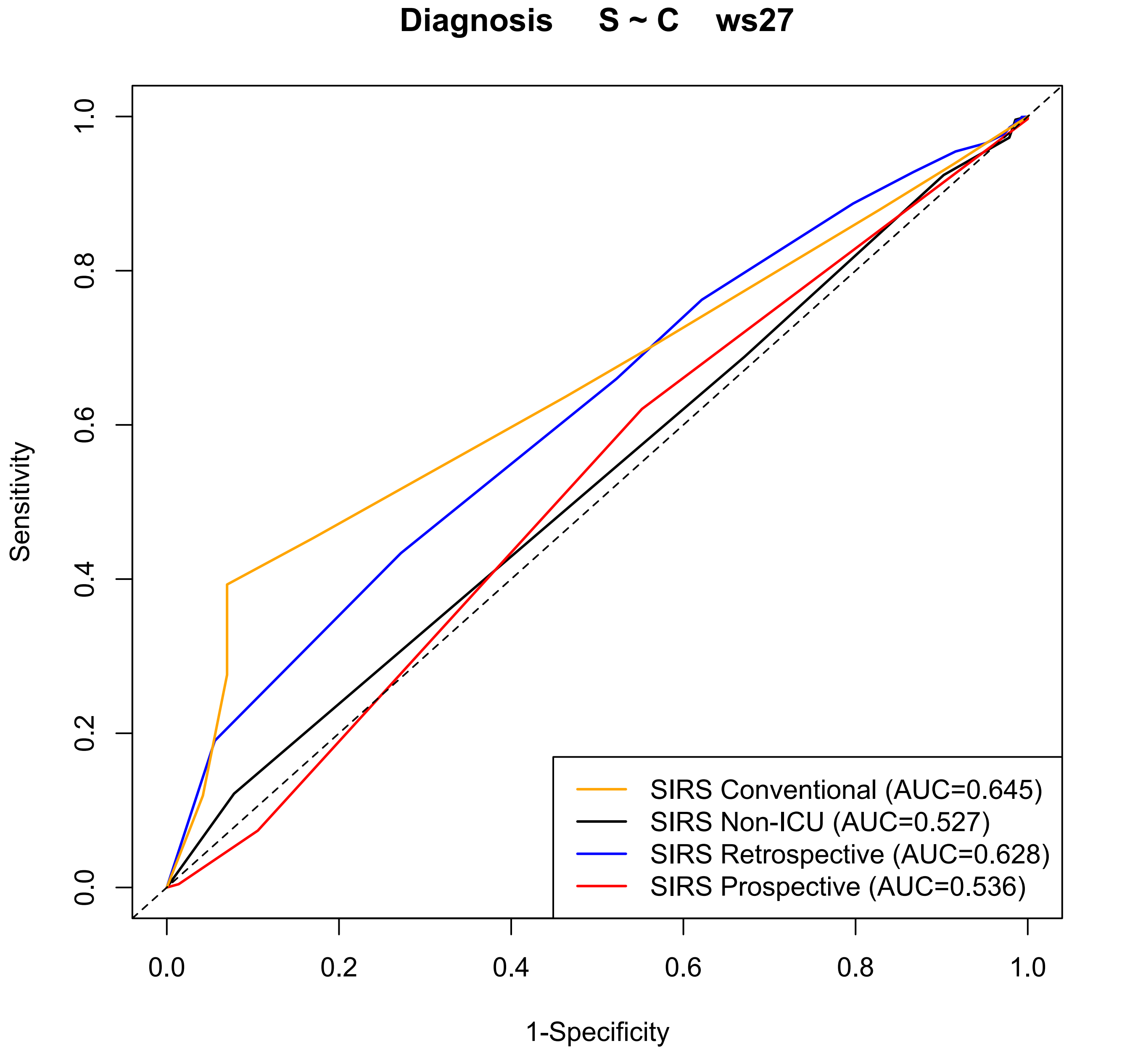
Diagnosis S ~ C ws27
Sensitivity
1-Specificity
0.0
0.2
0.4
0.6
0.8
1.0
SIRS Conventional (AUC=0.645)
SIRS Non-ICU (AUC=0.527)
SIRS Retrospective (AUC=0.628)
SIRS Prospective (AUC=0.536)

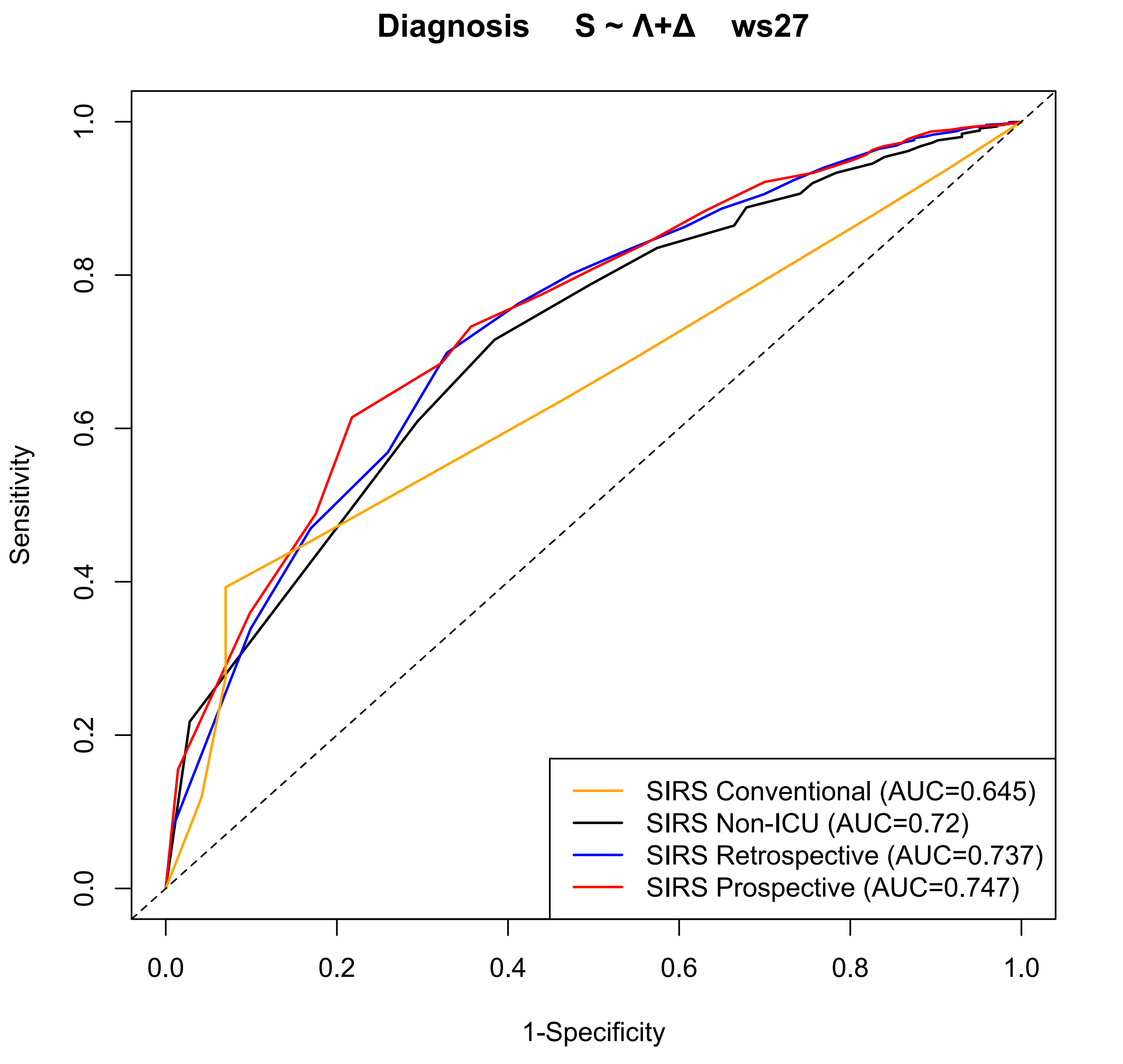

Diagnosis S ~ Λ+Δ ws27
Sensitivity
1-Specificity
0.0
0.2
0.4
0.6
0.8
1.0
SIRS Conventional (AUC=0.645)
SIRS Non-ICU (AUC=0.72)
SIRS Retrospective (AUC=0.737)
SIRS Prospective (AUC=0.747)

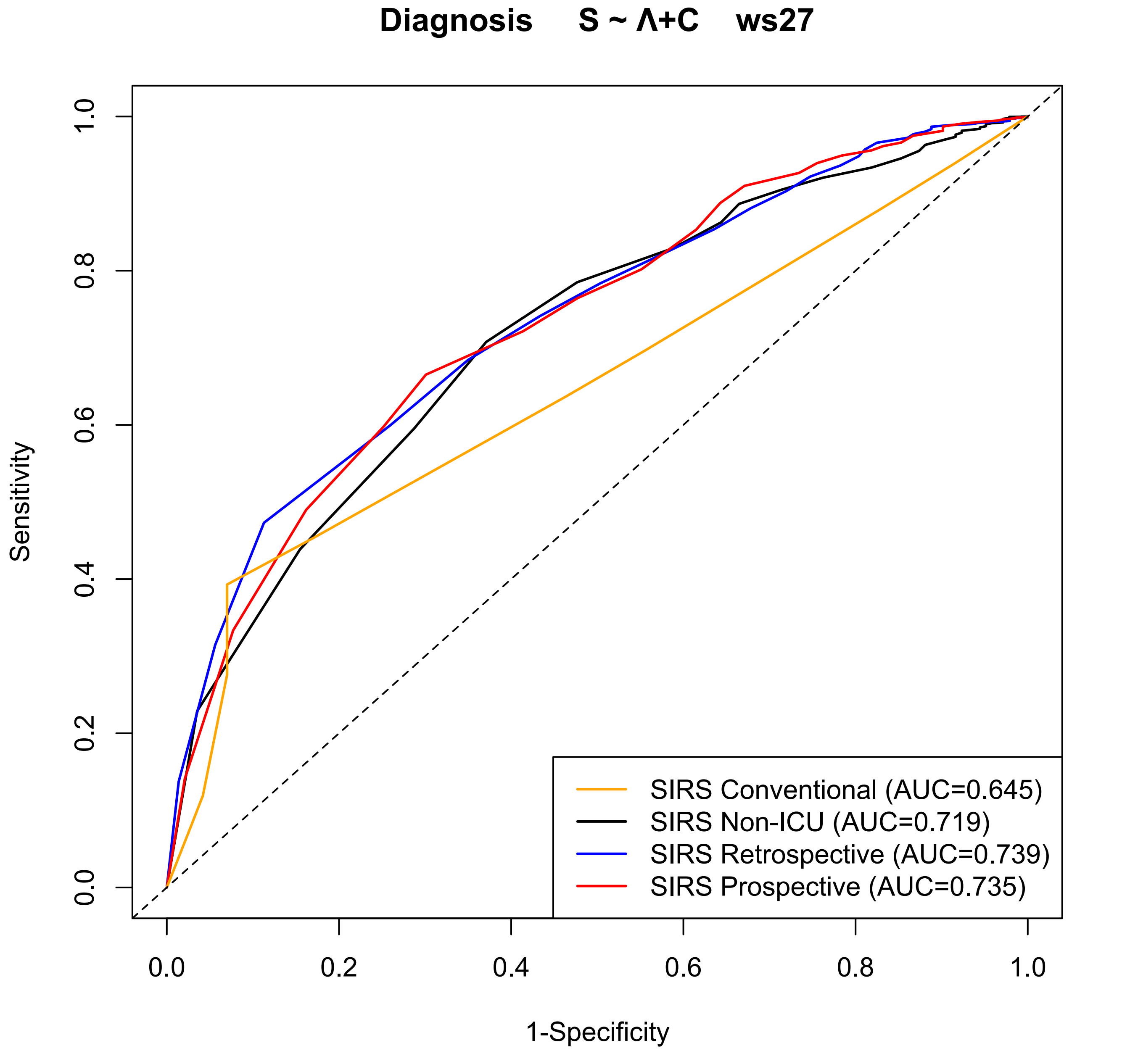

Diagnosis S ~ Λ+C ws27
Sensitivity
1-Specificity
0.0
0.2
0.4
0.6
0.8
1.0
0.0
0.2
0.4
0.6
0.8
1.0
SIRS Conventional (AUC=0.645)
SIRS Non-ICU (AUC=0.719)
SIRS Retrospective (AUC=0.739)
SIRS Prospective (AUC=0.735)

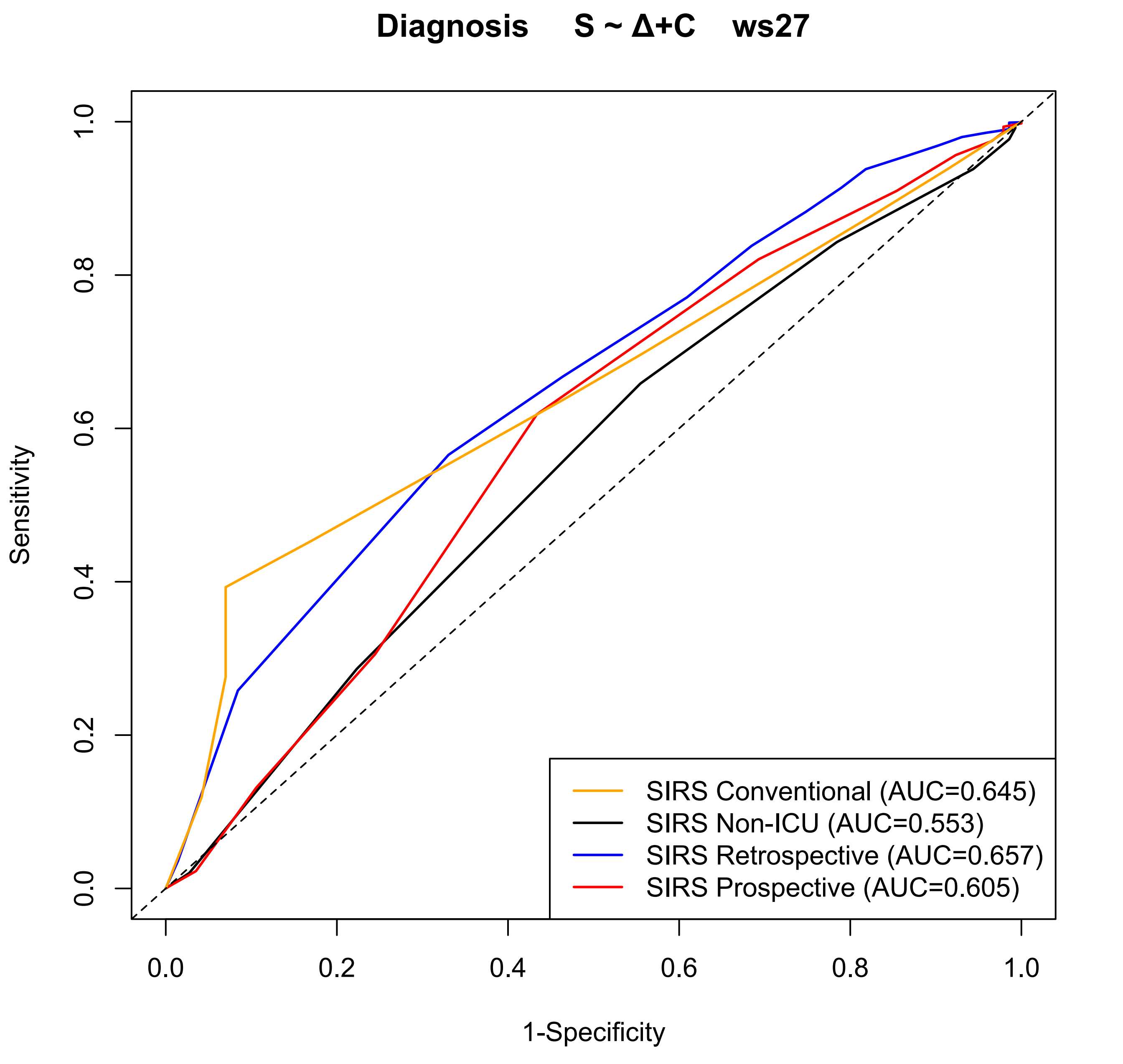
Diagnosis S ~ Δ+C ws27
Sensitivity
1-Specificity
0.0
0.2
0.4
0.6
0.8
1.0
SIRS Conventional (AUC=0.645)
SIRS Non-ICU (AUC=0.553)
SIRS Retrospective (AUC=0.657)
SIRS Prospective (AUC=0.605)

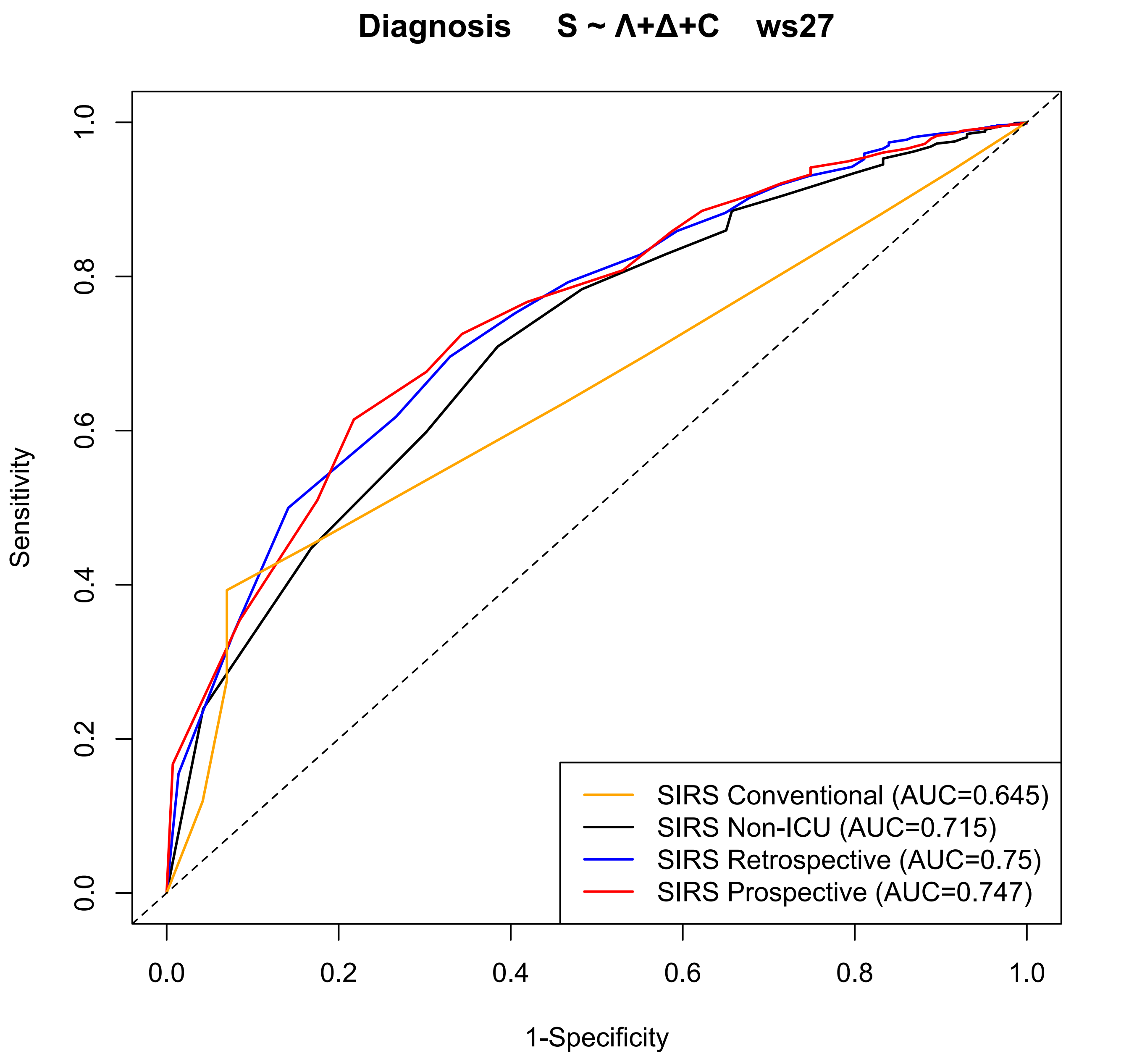

Diagnosis S ~ Λ+Δ+C ws27
Sensitivity
1-Specificity
0.0
0.2
0.4
0.6
0.8
1.0
0.0
0.2
0.4
0.6
0.8
1.0
SIRS Conventional (AUC=0.645)
SIRS Non-ICU (AUC=0.715)
SIRS Retrospective (AUC=0.75)
SIRS Prospective (AUC=0.747)

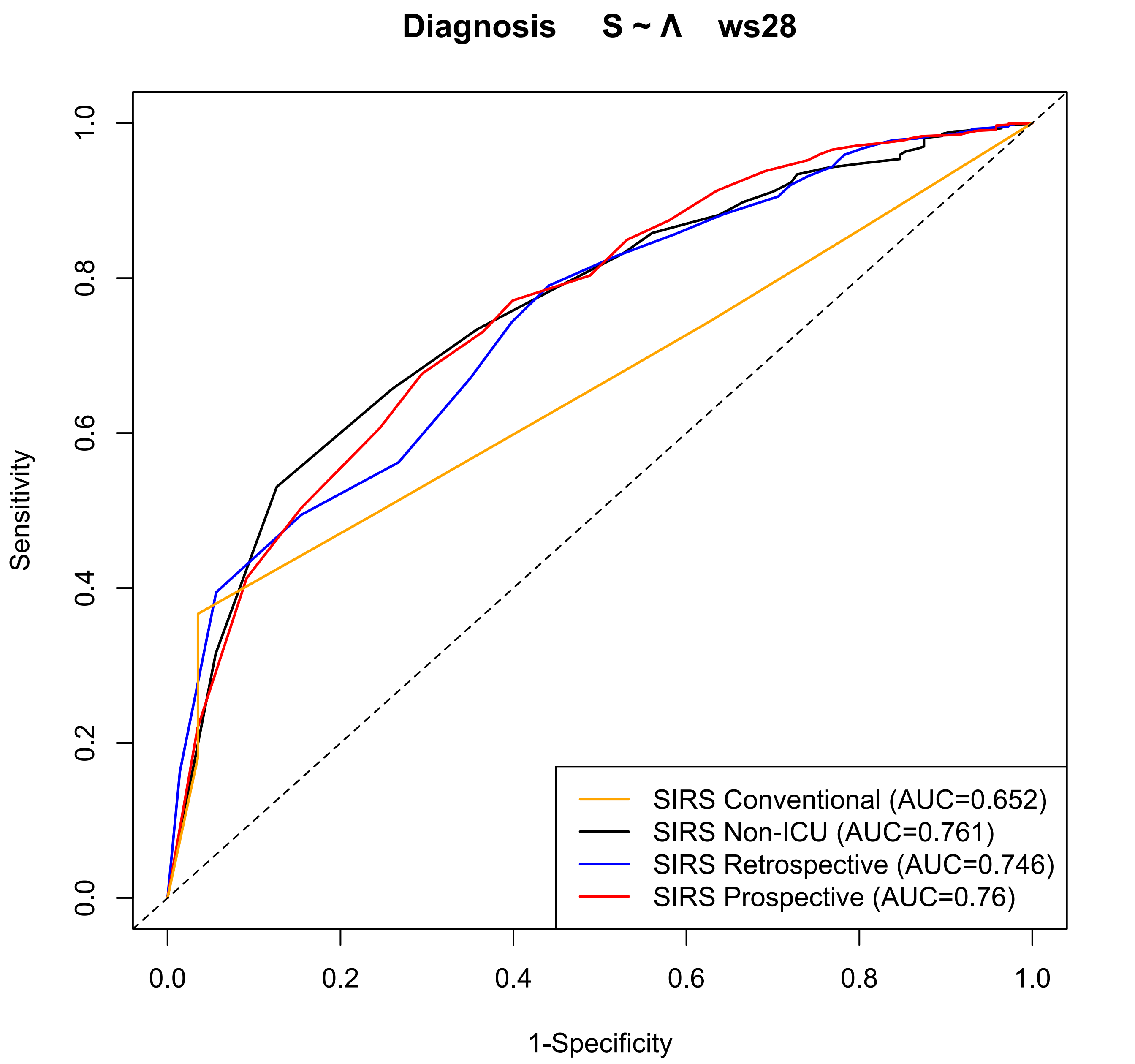

Diagnosis S ~ Λ ws28
Sensitivity
1-Specificity
0.0
0.2
0.4
0.6
0.8
1.0
SIRS Conventional (AUC=0.652)
SIRS Non-ICU (AUC=0.761)
SIRS Retrospective (AUC=0.746)
SIRS Prospective (AUC=0.76)

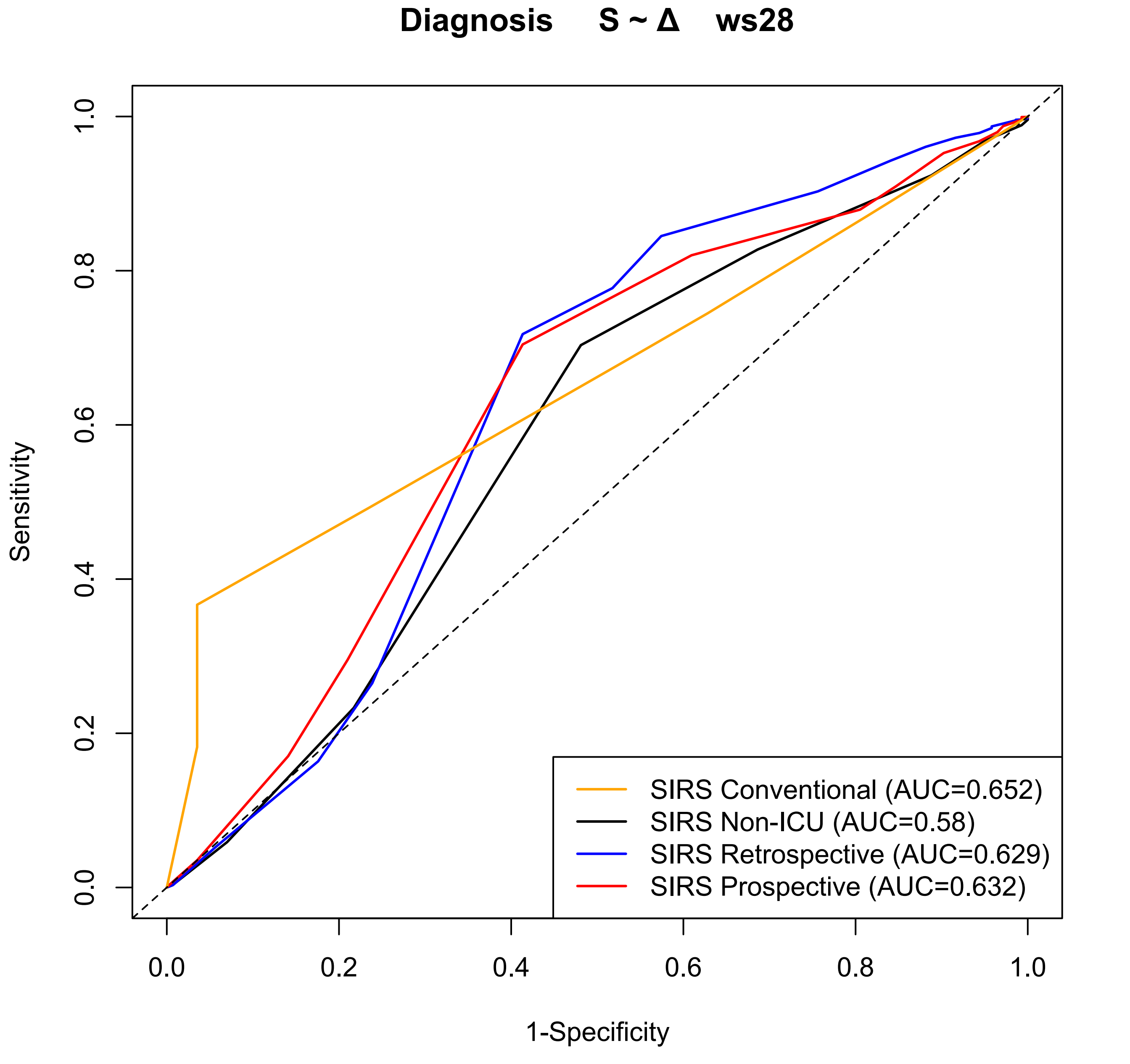
Diagnosis S ~ Δ ws28
Sensitivity
1-Specificity
0.0
0.2
0.4
0.6
0.8
1.0
SIRS Conventional (AUC=0.652)
SIRS Non-ICU (AUC=0.58)
SIRS Retrospective (AUC=0.629)
SIRS Prospective (AUC=0.632)

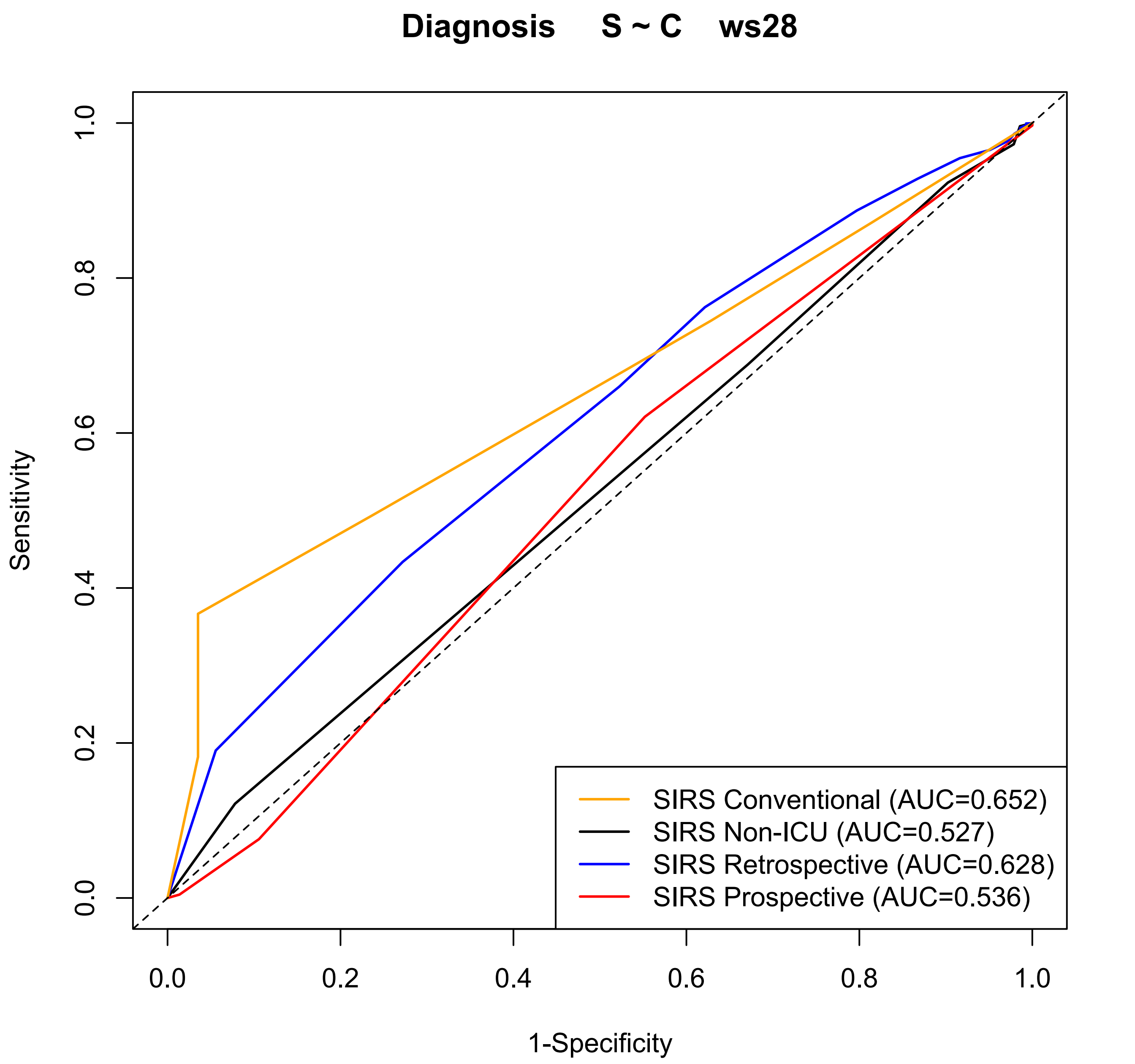
Diagnosis S ~ C ws28
Sensitivity
1-Specificity
0.0
0.2
0.4
0.6
0.8
1.0
SIRS Conventional (AUC=0.652)
SIRS Non-ICU (AUC=0.527)
SIRS Retrospective (AUC=0.628)
SIRS Prospective (AUC=0.536)

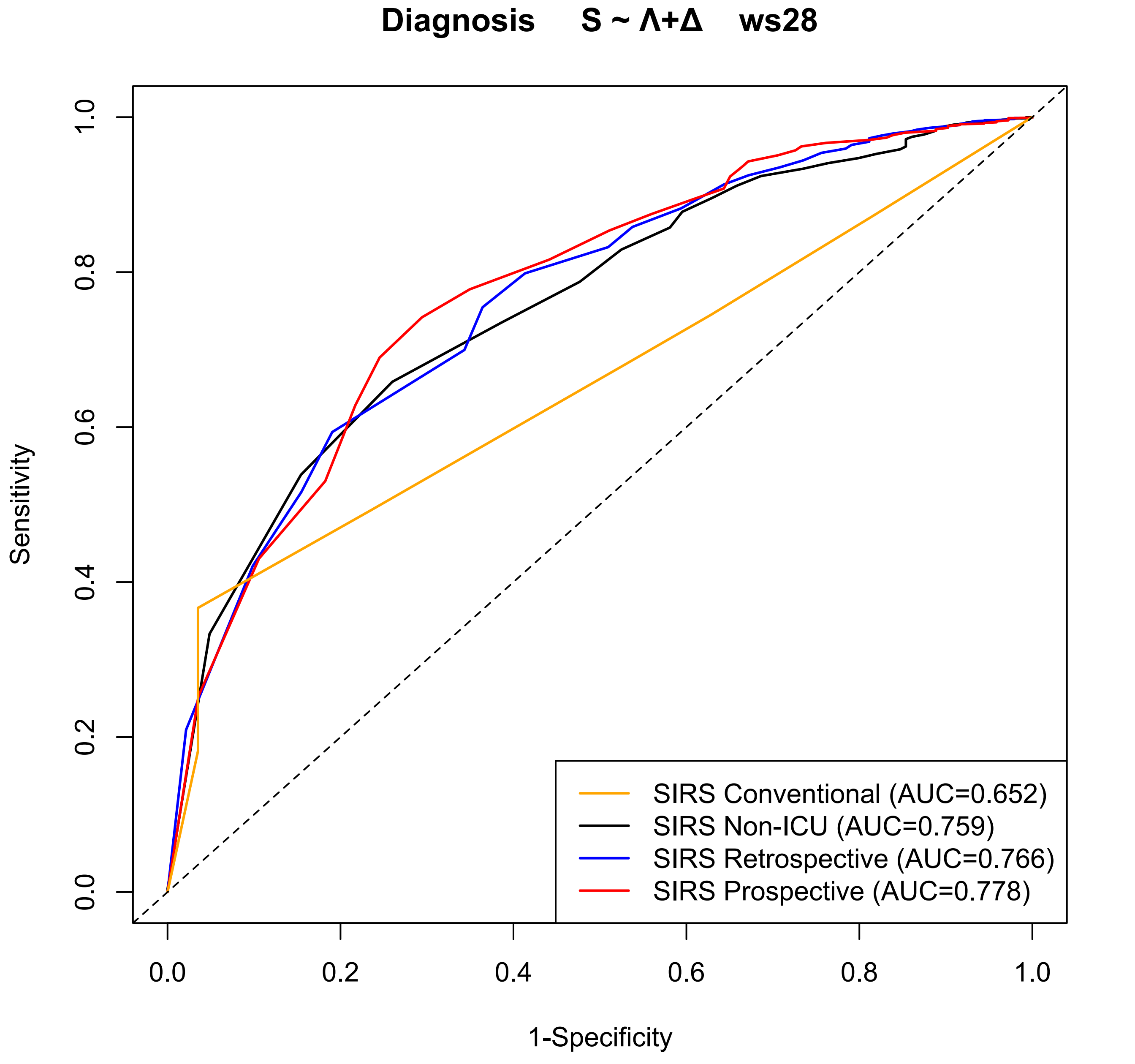
Diagnosis S ~ Λ+Δ ws28
Sensitivity
1-Specificity
0.0
0.2
0.4
0.6
0.8
1.0
SIRS Conventional (AUC=0.652)
SIRS Non-ICU (AUC=0.759)
SIRS Retrospective (AUC=0.766)
SIRS Prospective (AUC=0.778)

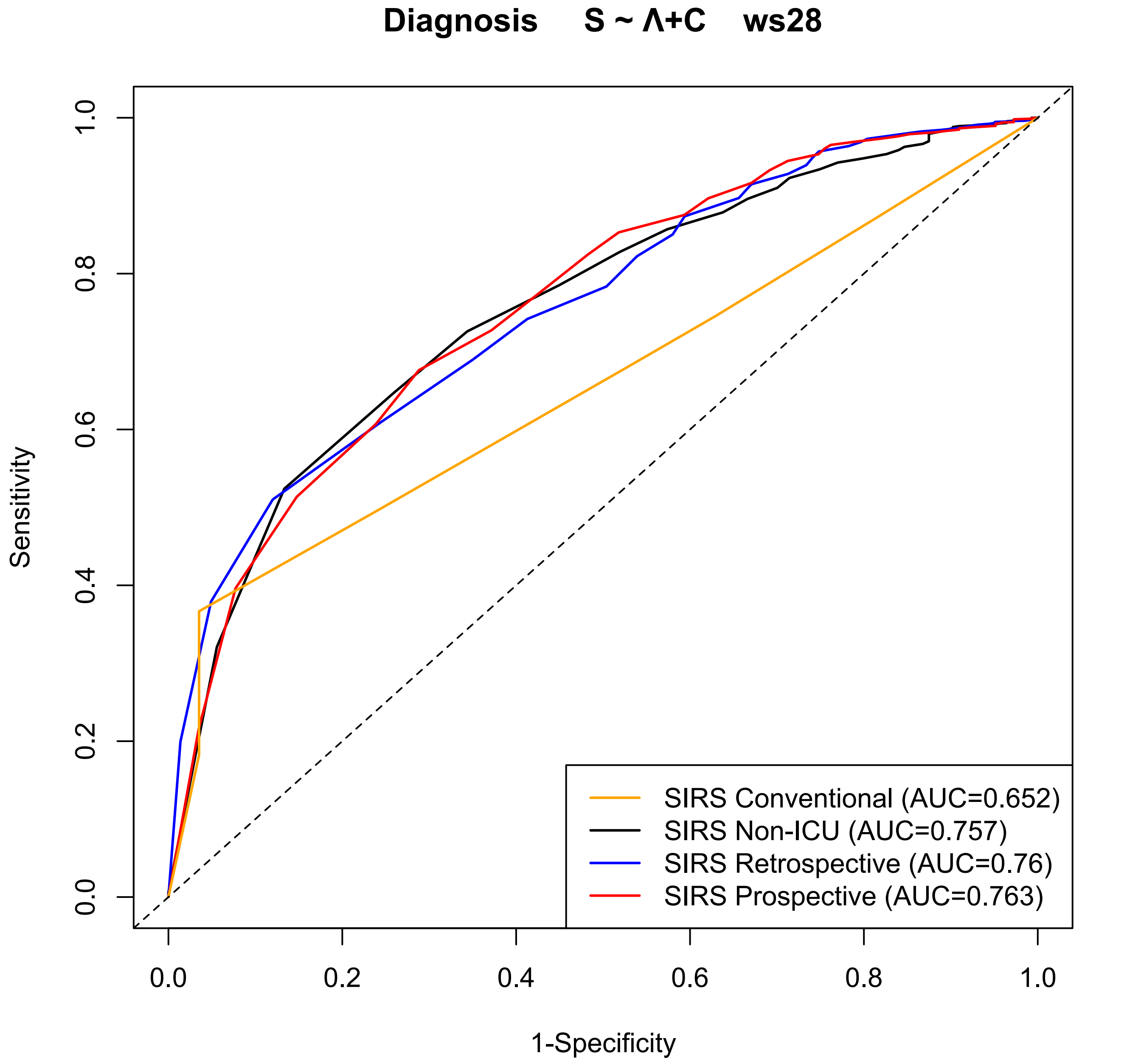

Diagnosis S ~ Λ+C ws28
Sensitivity
1-Specificity
0.0
0.2
0.4
0.6
0.8
1.0
SIRS Conventional (AUC=0.652)
SIRS Non-ICU (AUC=0.757)
SIRS Retrospective (AUC=0.76)
SIRS Prospective (AUC=0.763)

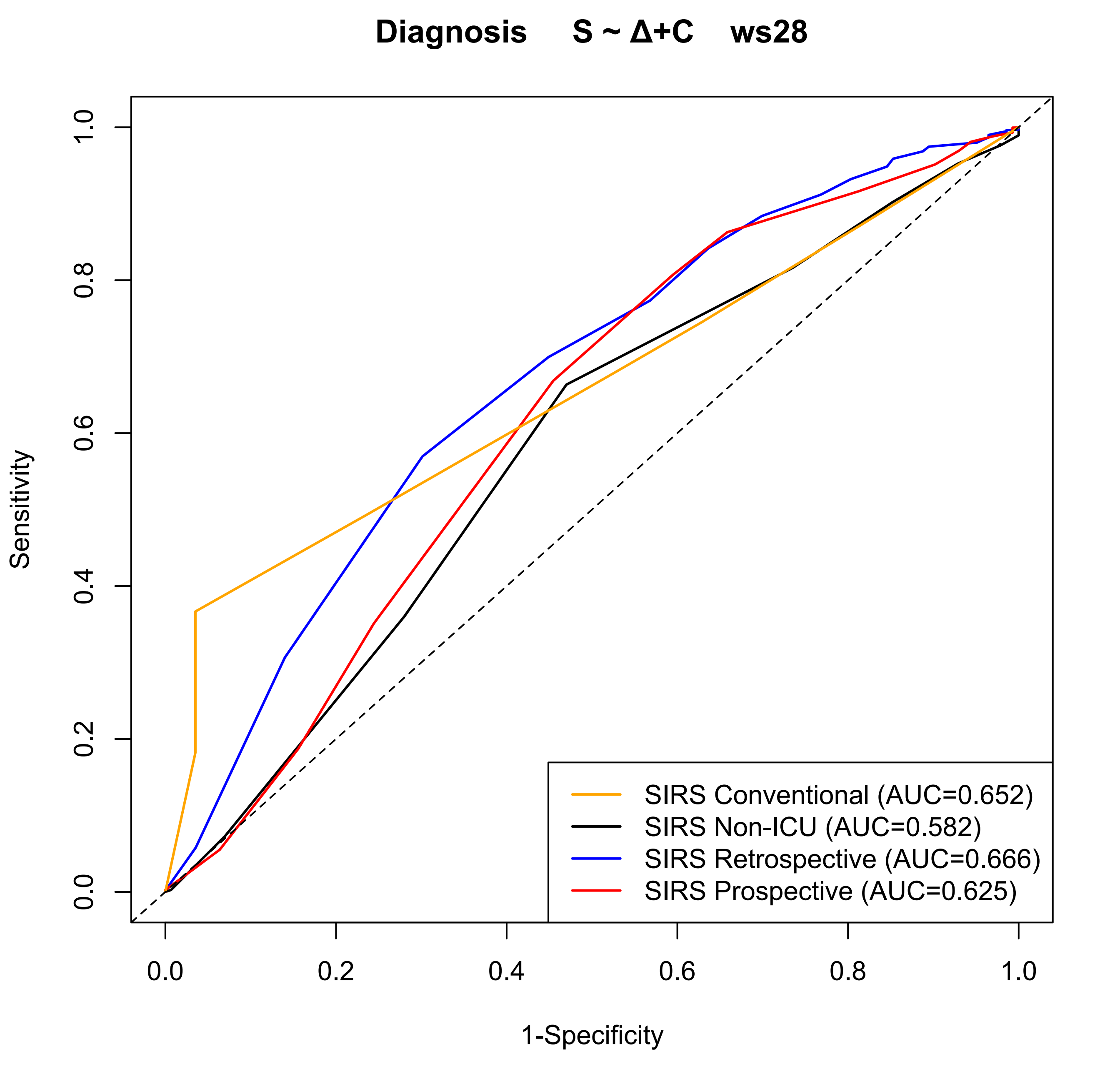

Diagnosis S ~ Δ+C ws28
Sensitivity
1-Specificity
0.0
0.2
0.4
0.6
0.8
1.0
SIRS Conventional (AUC=0.652)
SIRS Non-ICU (AUC=0.582)
SIRS Retrospective (AUC=0.666)
SIRS Prospective (AUC=0.625)

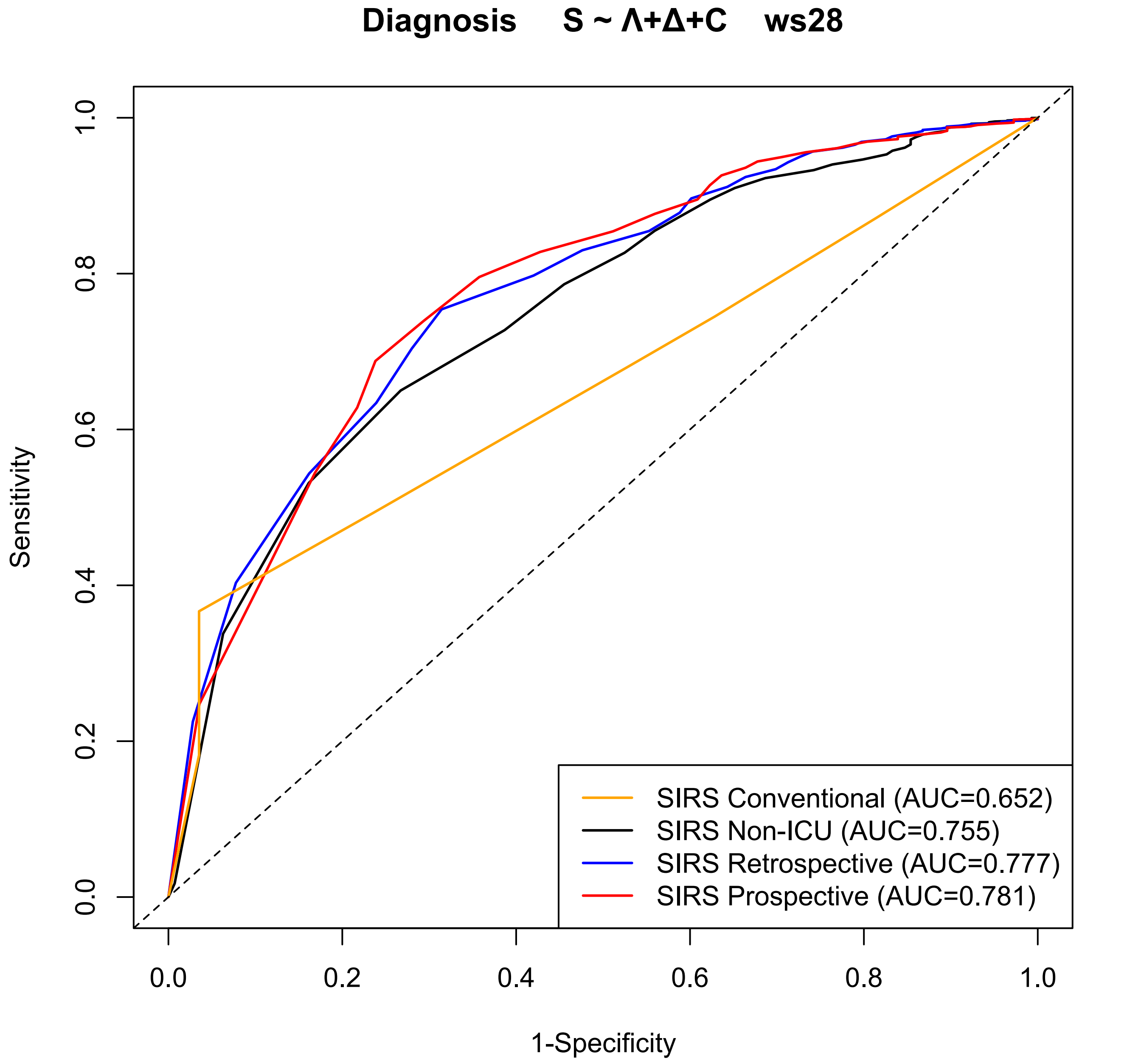

Diagnosis S ~ Λ+Δ+C ws28
Sensitivity
1-Specificity
0.0
0.2
0.4
0.6
0.8
1.0
SIRS Conventional (AUC=0.652)
SIRS Non-ICU (AUC=0.755)
SIRS Retrospective (AUC=0.777)
SIRS Prospective (AUC=0.781)

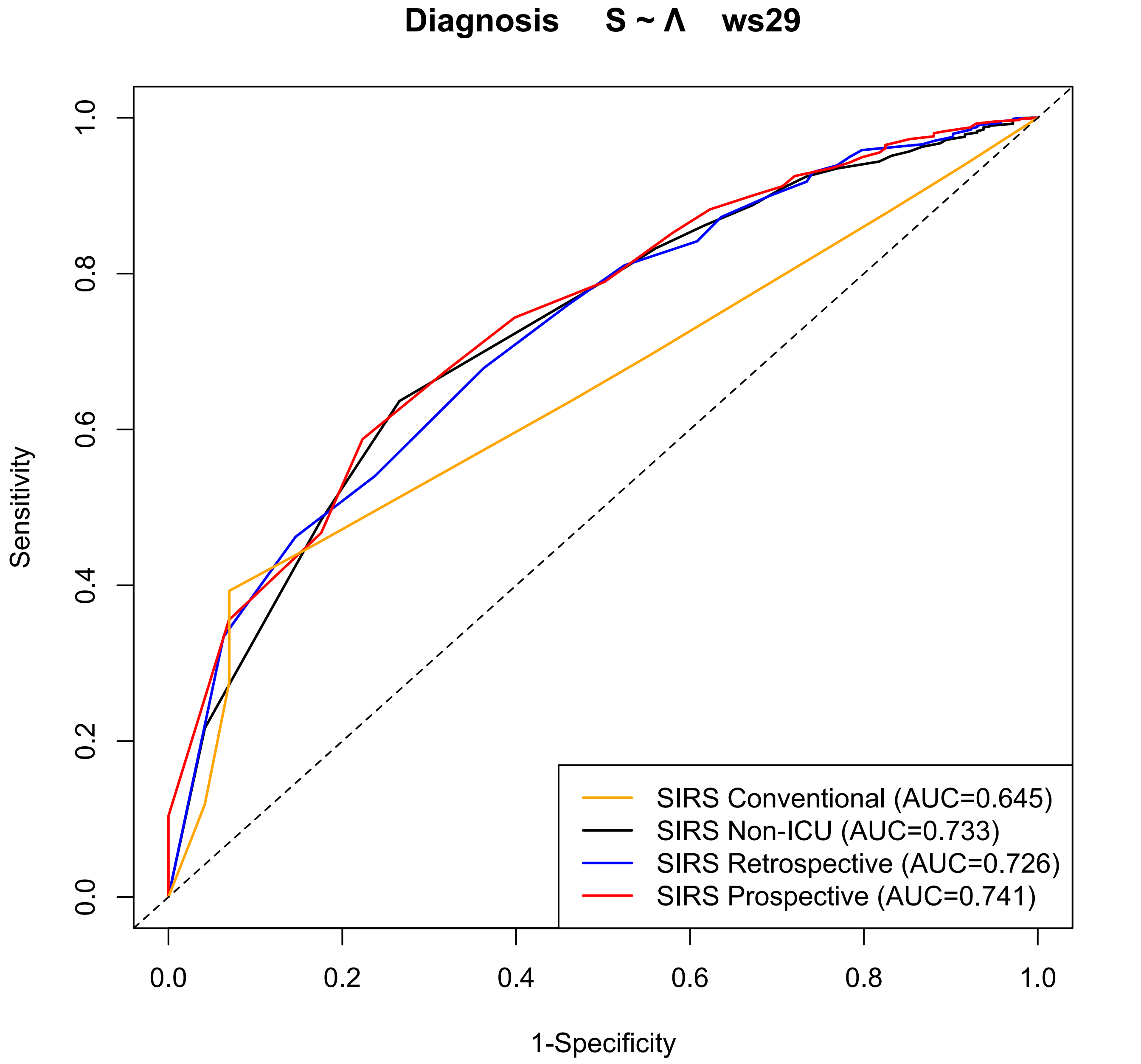
Diagnosis S ~ Λ ws29
Sensitivity
1-Specificity
0.0
0.2
0.4
0.6
0.8
1.0
SIRS Conventional (AUC=0.645)
SIRS Non-ICU (AUC=0.733)
SIRS Retrospective (AUC=0.726)
SIRS Prospective (AUC=0.741)

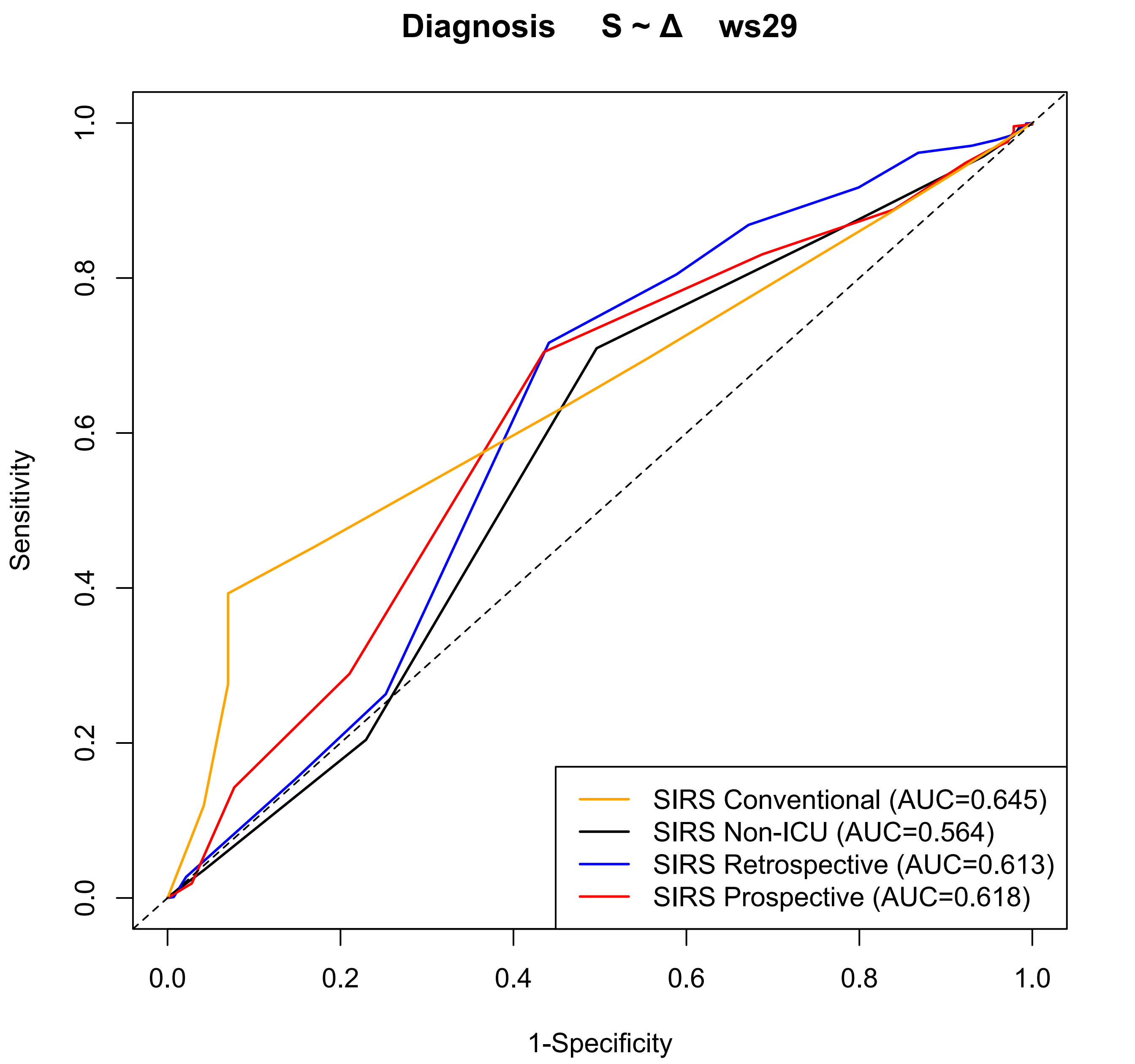

Diagnosis S ~ Δ ws29
Sensitivity
1-Specificity
0.0
0.2
0.4
0.6
0.8
1.0
SIRS Conventional (AUC=0.645)
SIRS Non-ICU (AUC=0.564)
SIRS Retrospective (AUC=0.613)
SIRS Prospective (AUC=0.618)

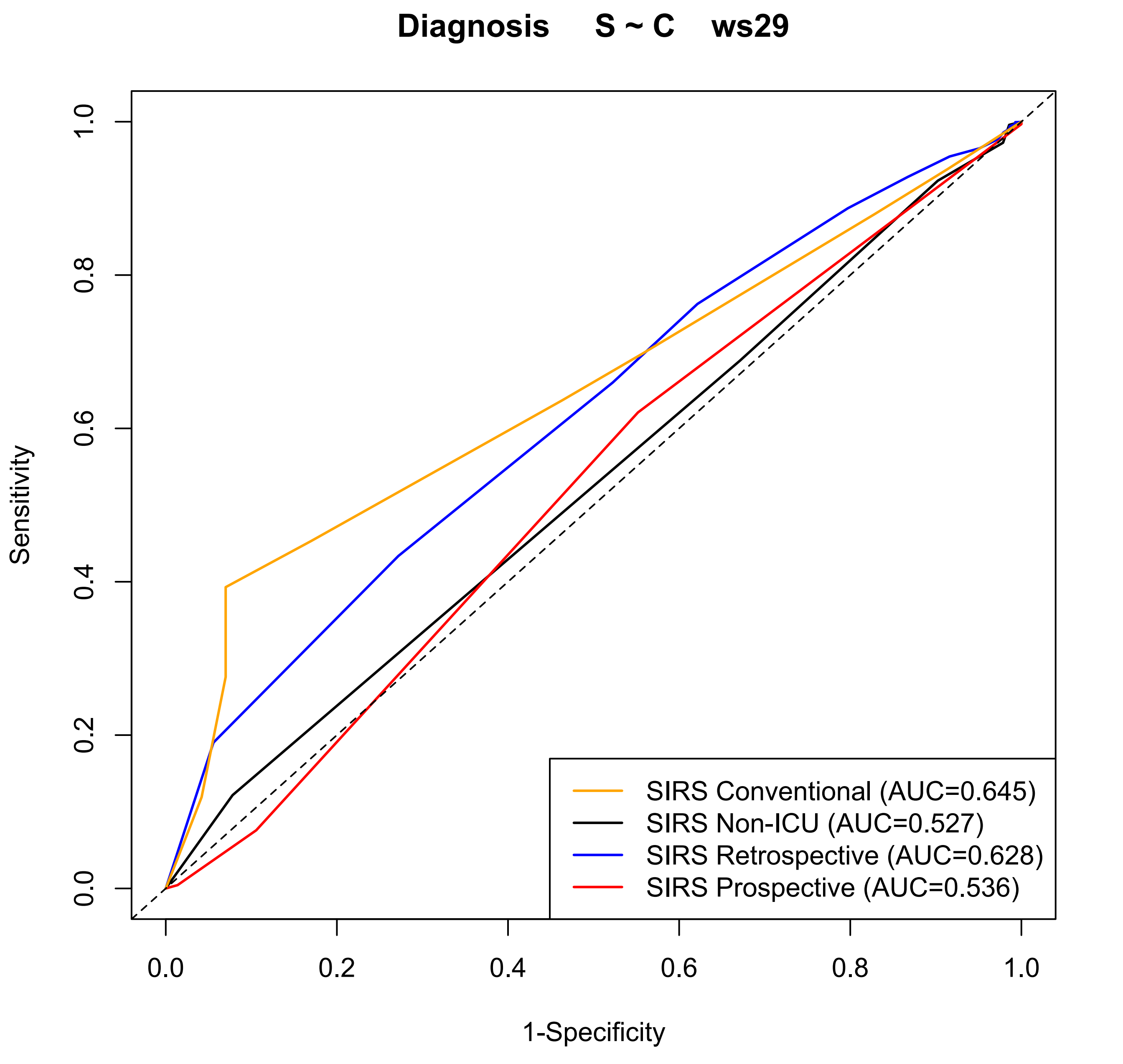
Diagnosis S ~ C ws29
Sensitivity
1-Specificity
0.0
0.2
0.4
0.6
0.8
1.0
SIRS Conventional (AUC=0.645)
SIRS Non-ICU (AUC=0.527)
SIRS Retrospective (AUC=0.628)
SIRS Prospective (AUC=0.536)

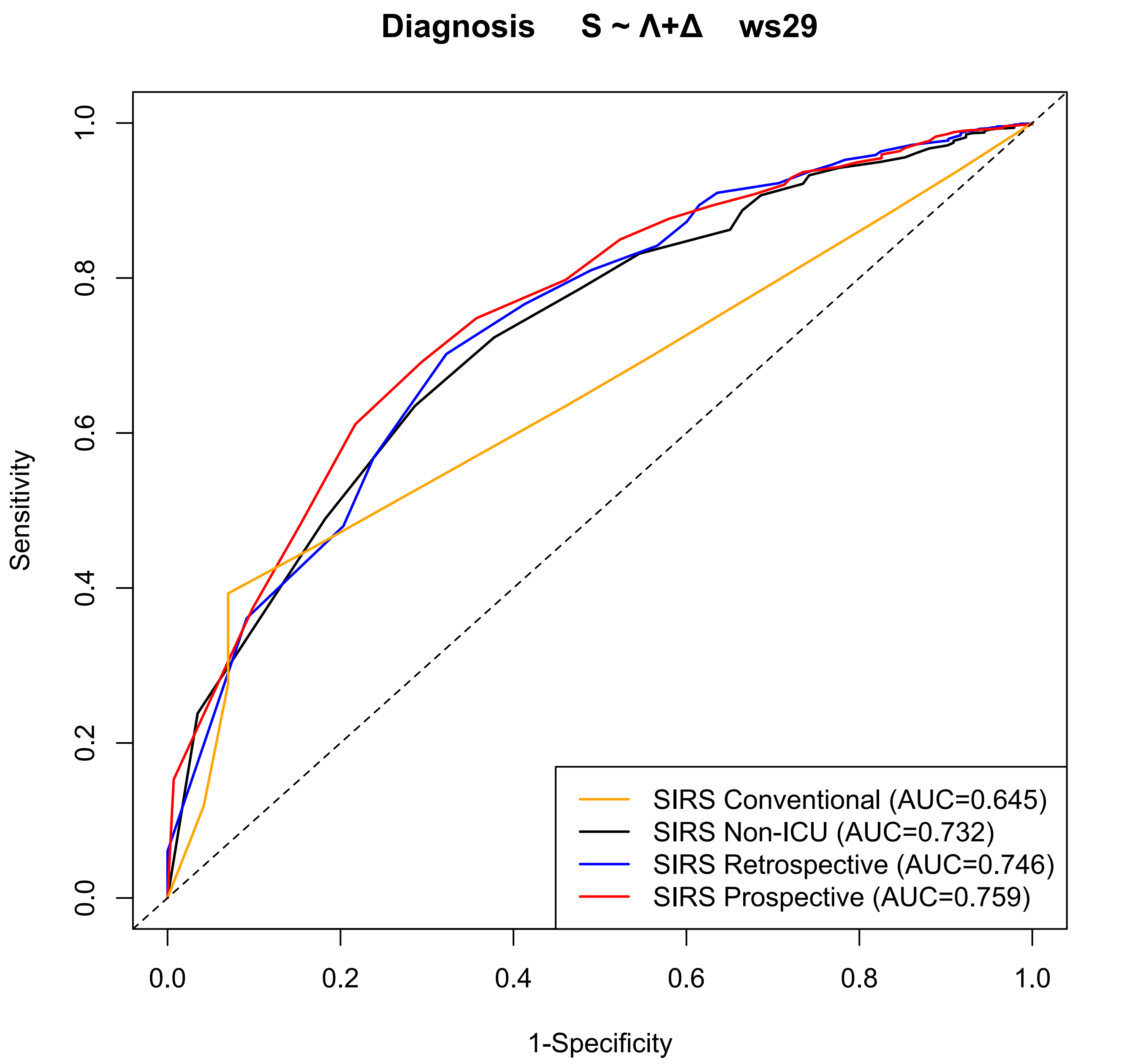
Diagnosis S ~ Λ+Δ ws29
Sensitivity
1-Specificity
0.0
0.2
0.4
0.6
0.8
1.0
SIRS Conventional (AUC=0.645)
SIRS Non-ICU (AUC=0.732)
SIRS Retrospective (AUC=0.746)
SIRS Prospective (AUC=0.759)

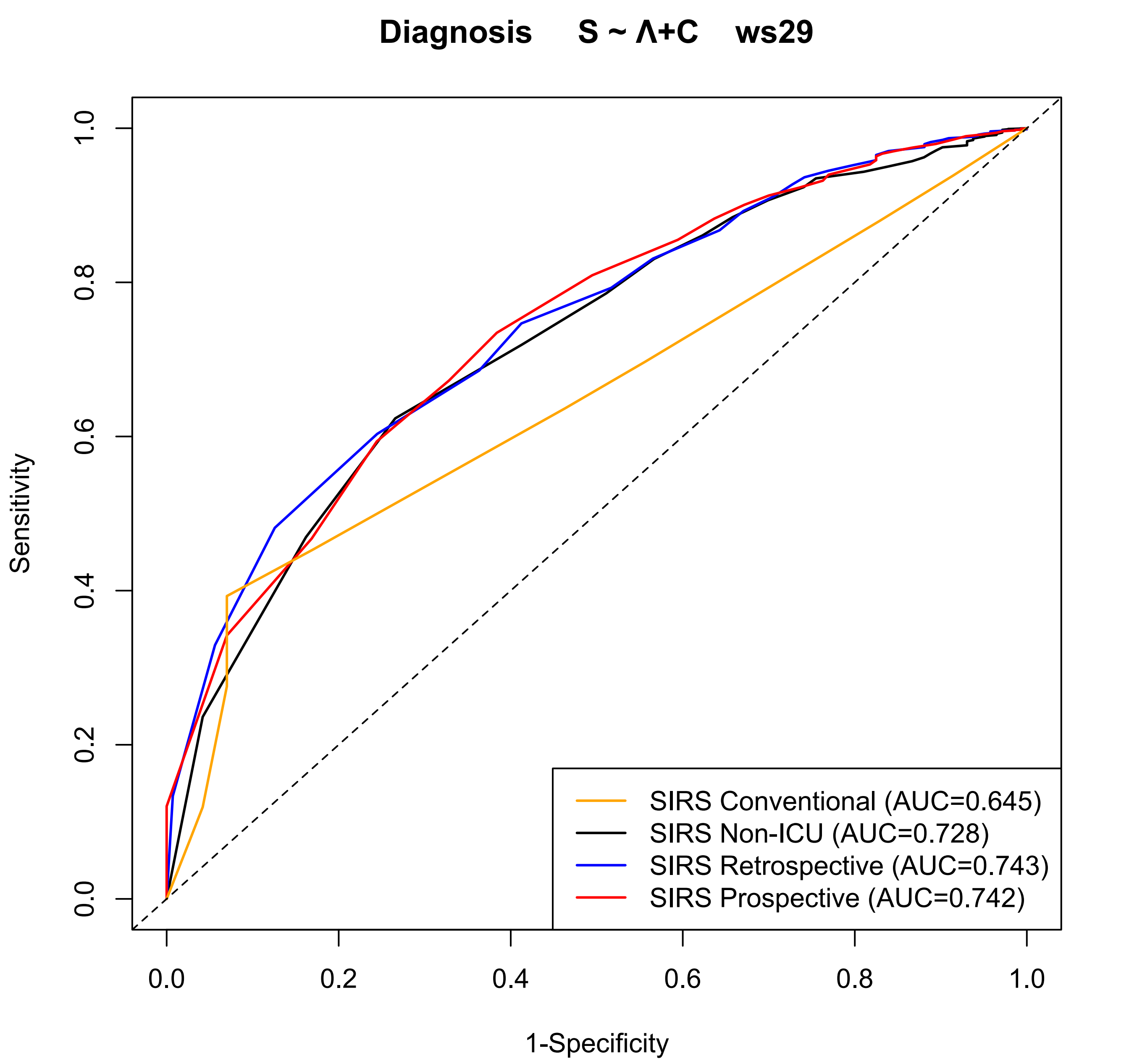
Diagnosis S ~ Λ+C ws29
Sensitivity
1-Specificity
0.0
0.2
0.4
0.6
0.8
1.0
SIRS Conventional (AUC=0.645)
SIRS Non-ICU (AUC=0.728)
SIRS Retrospective (AUC=0.743)
SIRS Prospective (AUC=0.742)

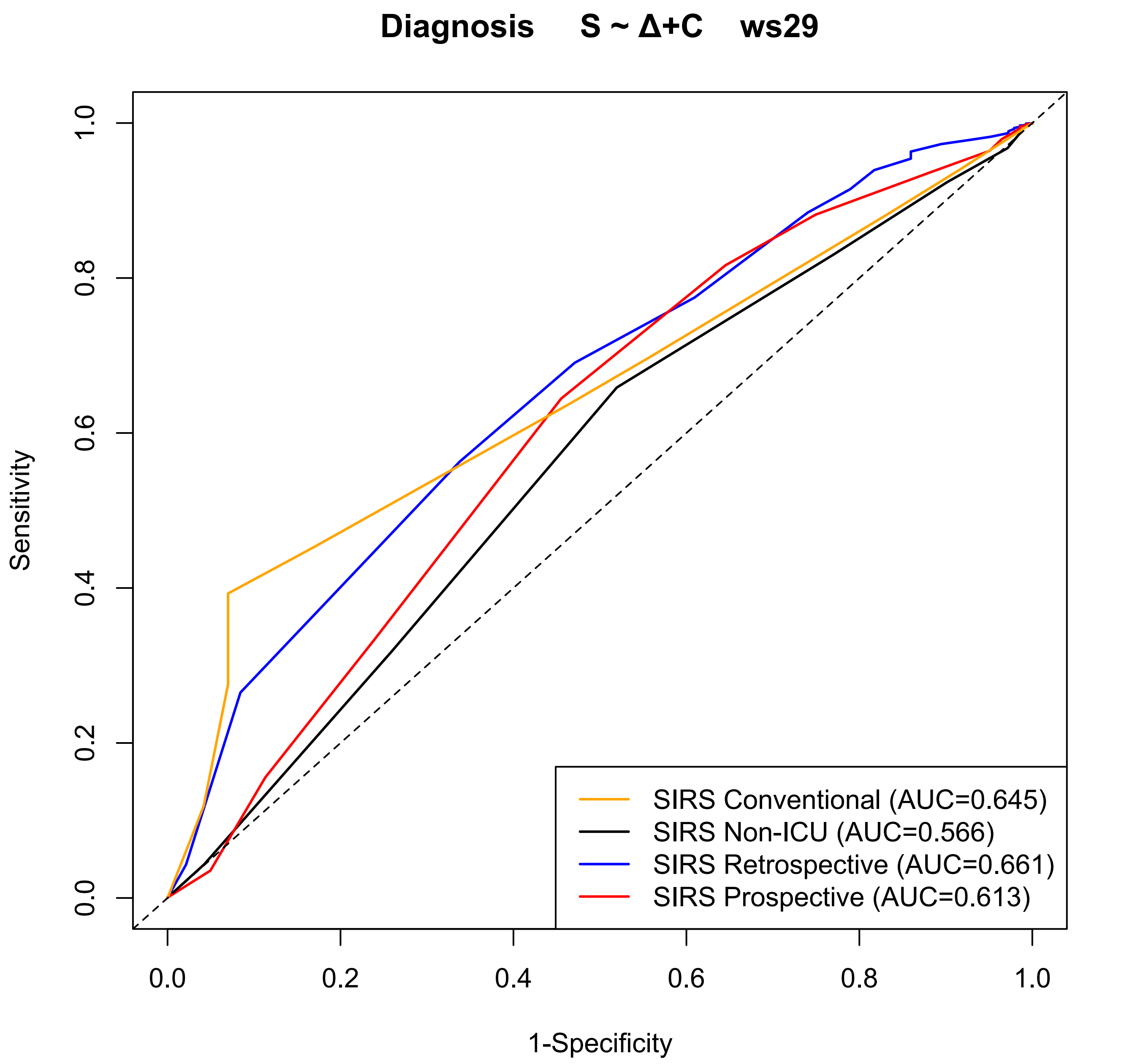
Diagnosis S ~ Δ+C ws29
Sensitivity
1-Specificity
0.0
0.2
0.4
0.6
0.8
1.0
SIRS Conventional (AUC=0.645)
SIRS Non-ICU (AUC=0.566)
SIRS Retrospective (AUC=0.661)
SIRS Prospective (AUC=0.613)

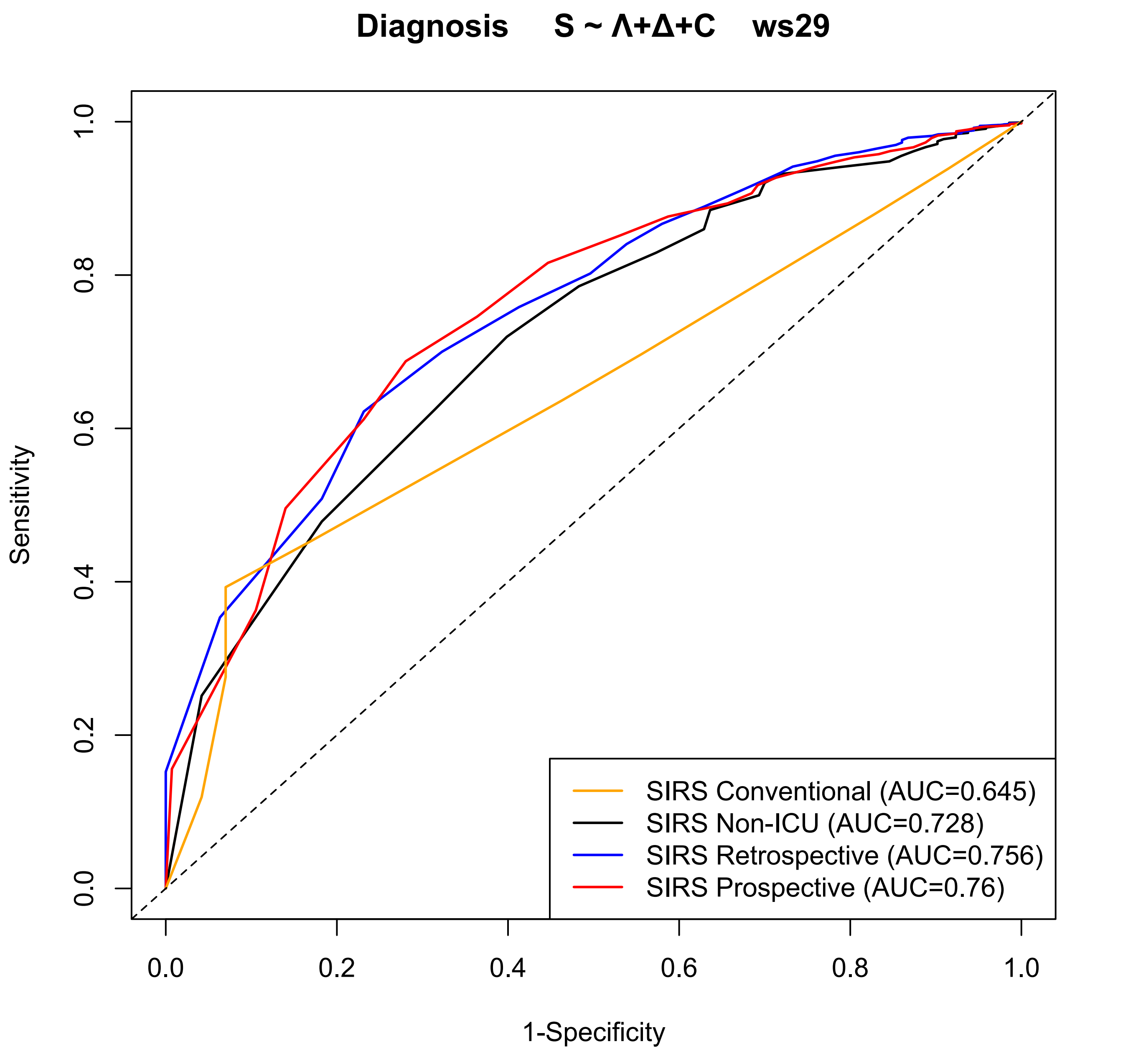
Diagnosis S ~ Λ+Δ+C ws29
Sensitivity
1-Specificity
0.0
0.2
0.4
0.6
0.8
1.0
SIRS Conventional (AUC=0.645)
SIRS Non-ICU (AUC=0.728)
SIRS Retrospective (AUC=0.756)
SIRS Prospective (AUC=0.76)

Diagnosis S ~ Λ ws30

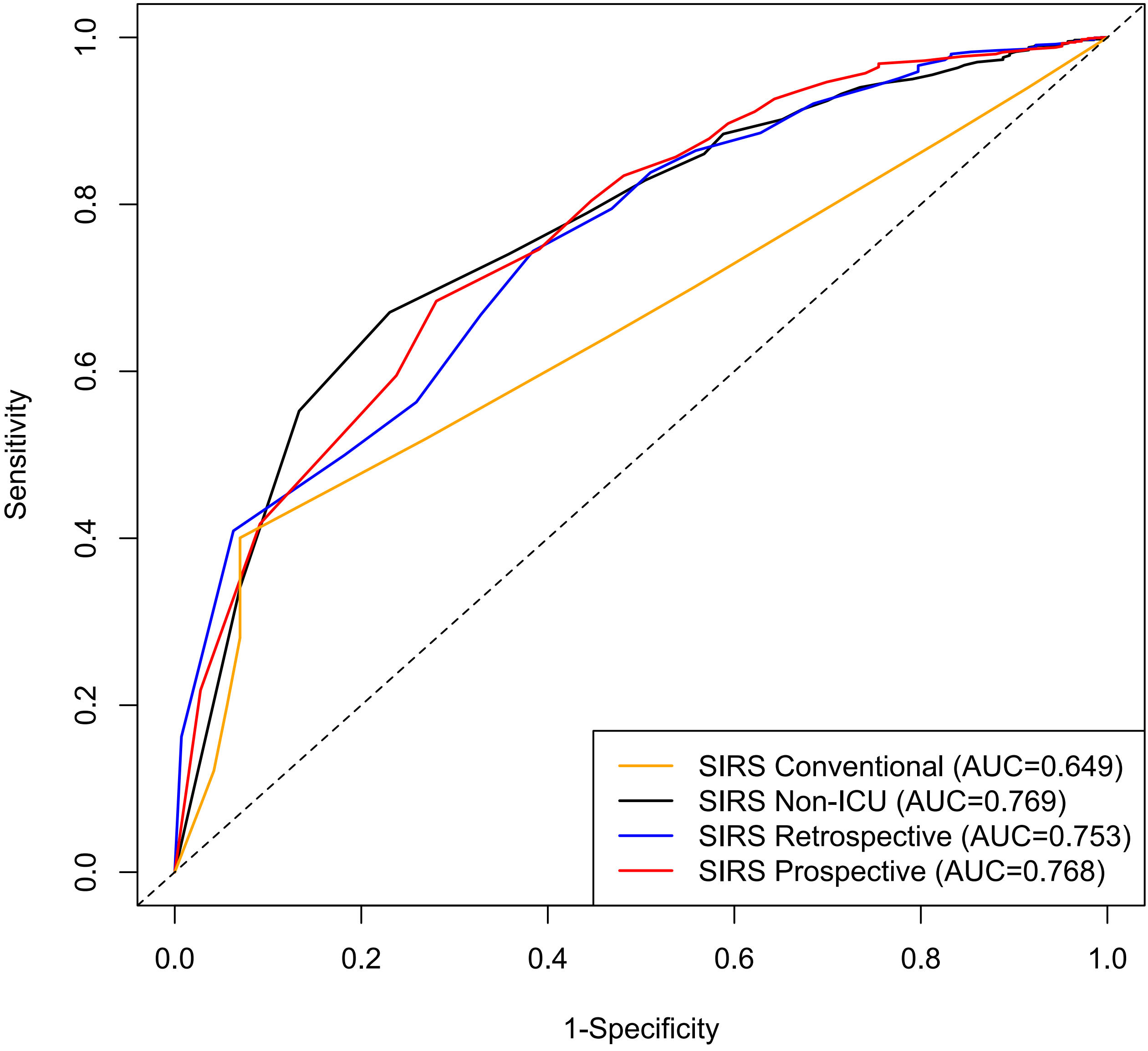

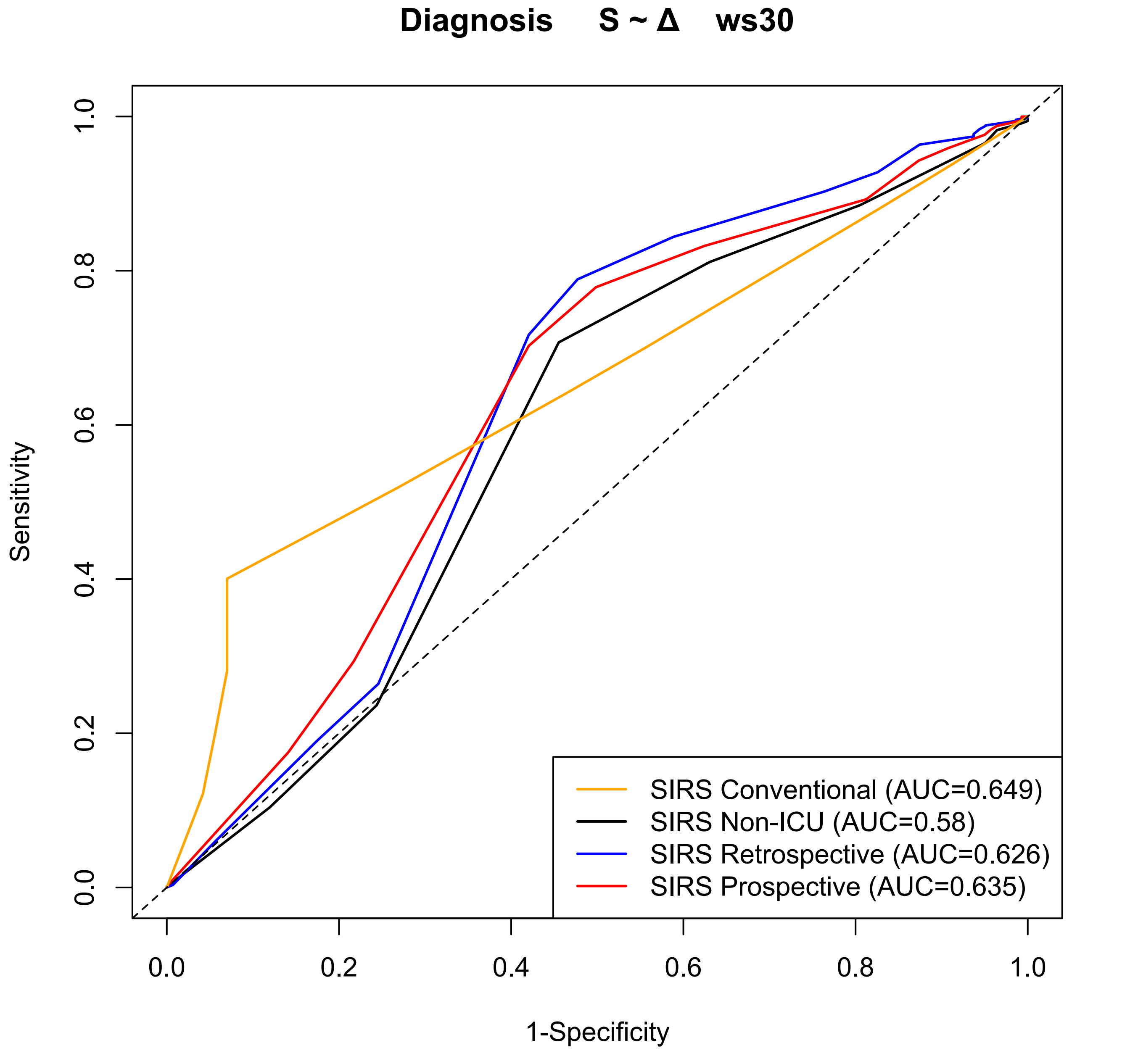
Diagnosis S ~ Δ ws30
Sensitivity
1-Specificity
0.0
0.2
0.4
0.6
0.8
1.0
SIRS Conventional (AUC=0.649)
SIRS Non-ICU (AUC=0.58)
SIRS Retrospective (AUC=0.626)
SIRS Prospective (AUC=0.635)

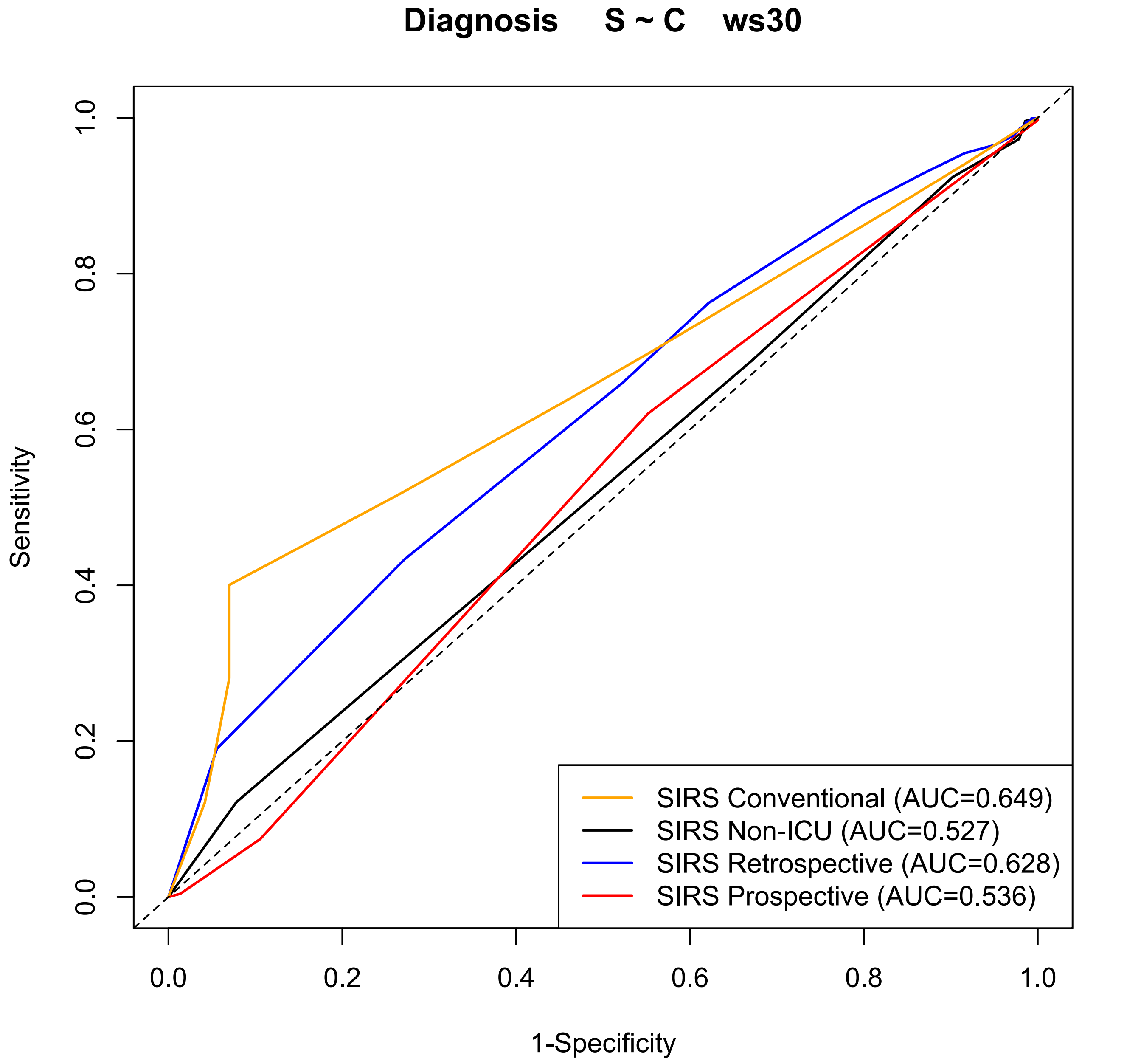

Diagnosis S ~ C ws30
Sensitivity
1-Specificity
0.0
0.2
0.4
0.6
0.8
1.0
SIRS Conventional (AUC=0.649)
SIRS Non-ICU (AUC=0.527)
SIRS Retrospective (AUC=0.628)
SIRS Prospective (AUC=0.536)

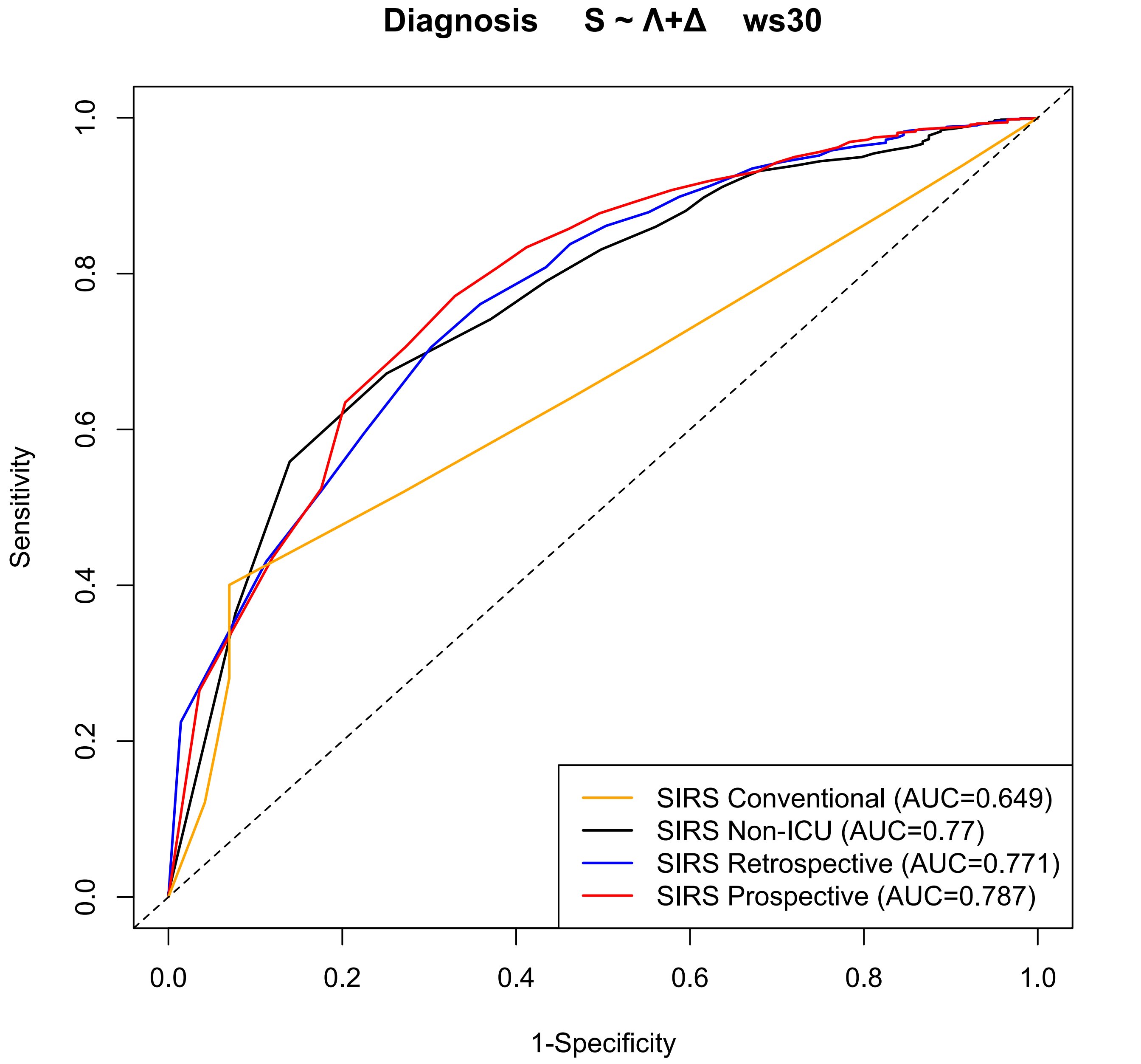

Diagnosis S ~ Λ+Δ ws30
Sensitivity
1-Specificity
0.0
0.2
0.4
0.6
0.8
1.0
SIRS Conventional (AUC=0.649)
SIRS Non-ICU (AUC=0.77)
SIRS Retrospective (AUC=0.771)
SIRS Prospective (AUC=0.787)

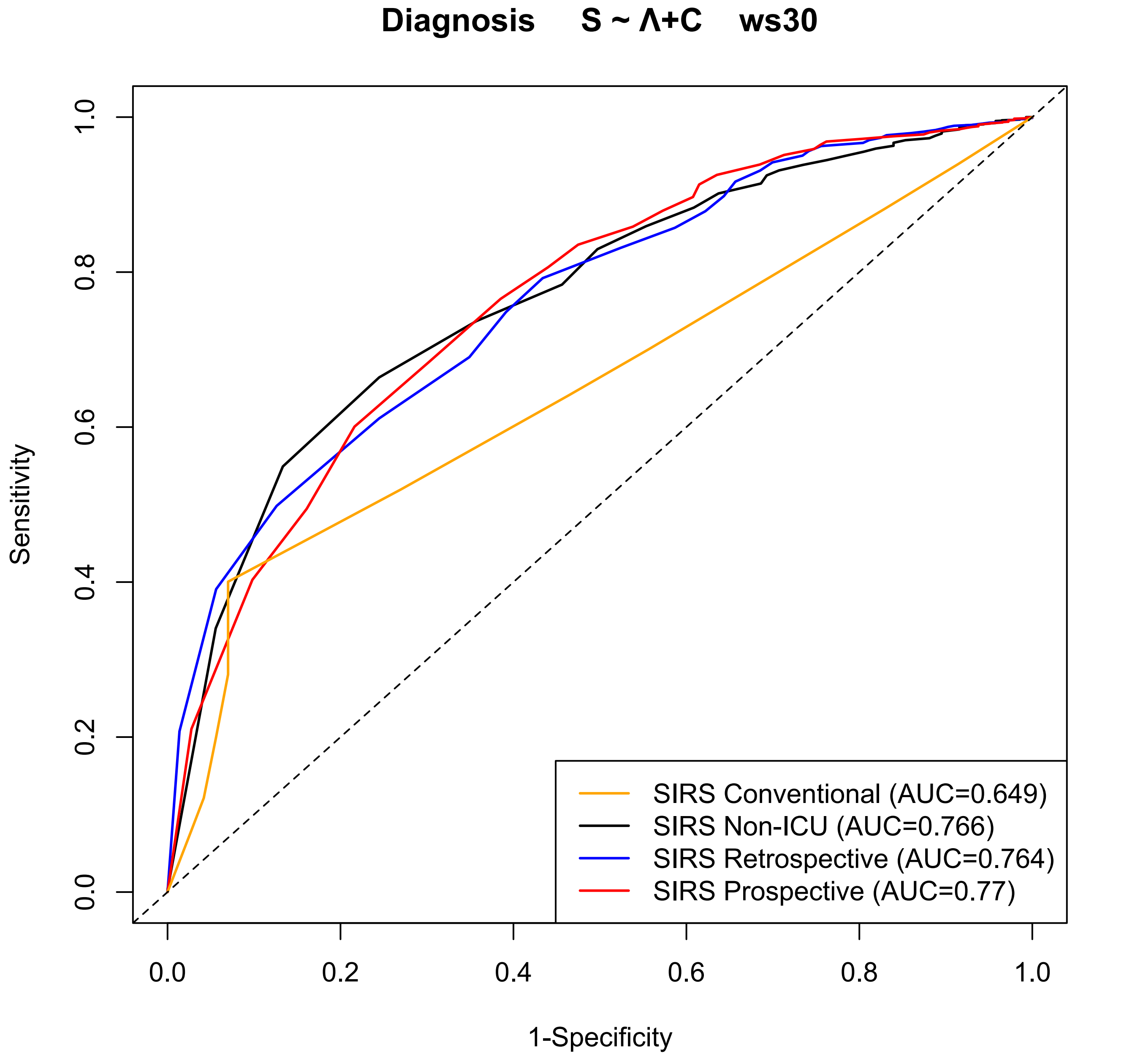
Diagnosis S ~ Λ+C ws30
Sensitivity
1-Specificity
0.0
0.2
0.4
0.6
0.8
1.0
SIRS Conventional (AUC=0.649)
SIRS Non-ICU (AUC=0.766)
SIRS Retrospective (AUC=0.764)
SIRS Prospective (AUC=0.77)

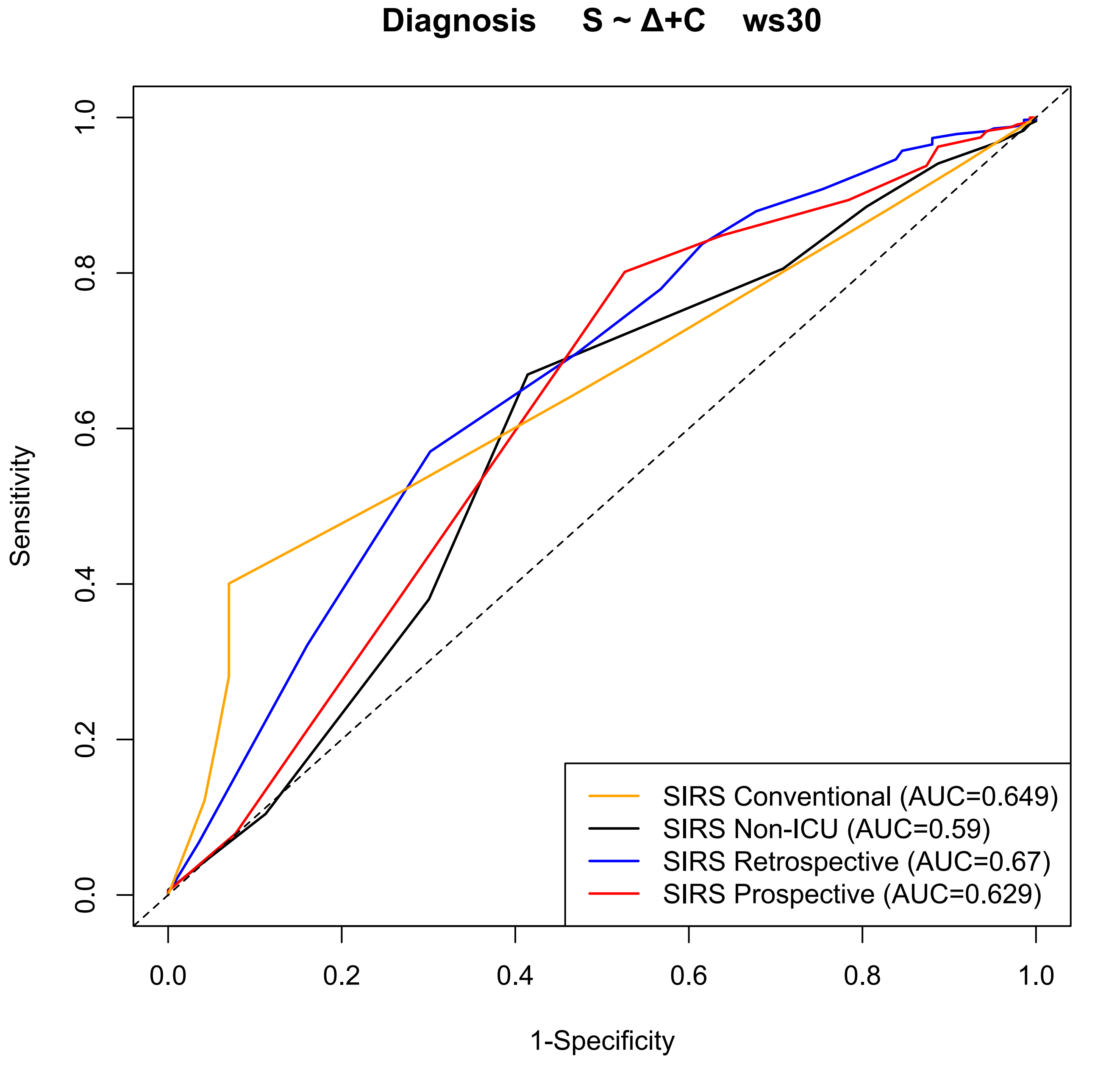

Diagnosis S ~ Δ+C ws30
Sensitivity
1-Specificity
0.0
0.2
0.4
0.6
0.8
1.0
SIRS Conventional (AUC=0.649)
SIRS Non-ICU (AUC=0.59)
SIRS Retrospective (AUC=0.67)
SIRS Prospective (AUC=0.629)

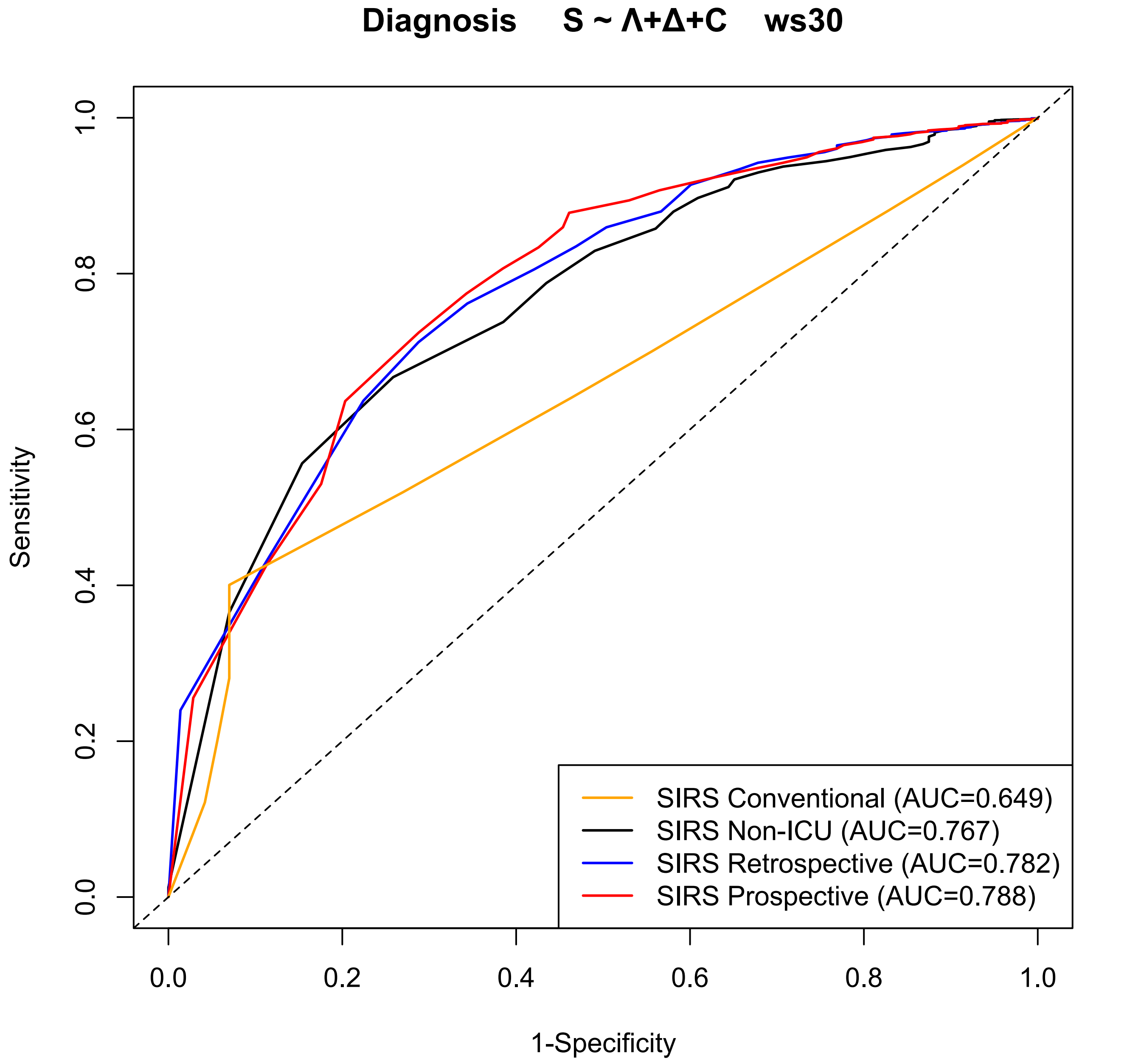

Diagnosis S ~ Λ+Δ+C ws30
Sensitivity
1-Specificity
0.0
0.2
0.4
0.6
0.8
1.0
SIRS Conventional (AUC=0.649)
SIRS Non-ICU (AUC=0.767)
SIRS Retrospective (AUC=0.782)
SIRS Prospective (AUC=0.788)

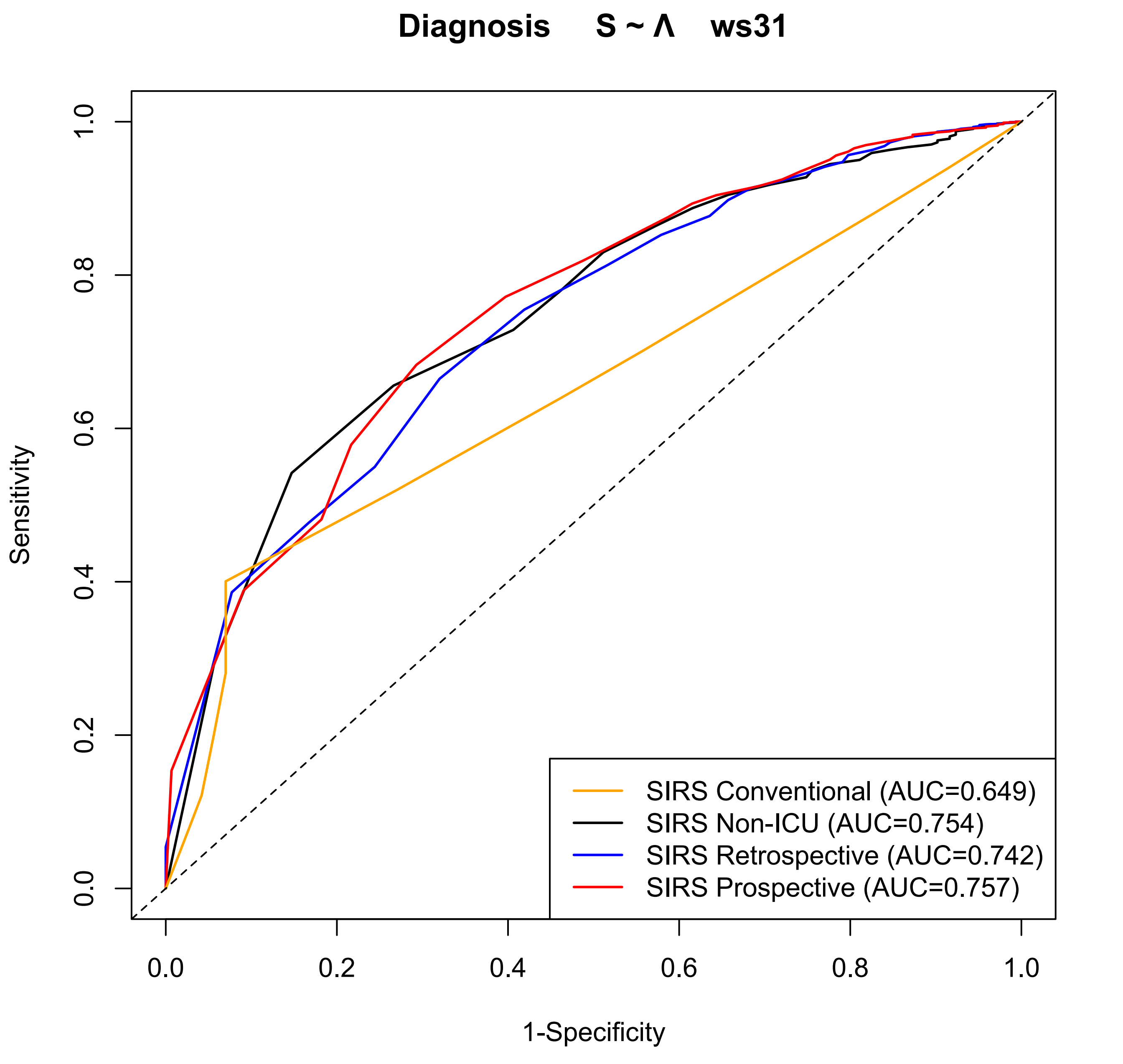

Diagnosis S ~ Λ ws31
Sensitivity
1-Specificity
0.0
0.2
0.4
0.6
0.8
1.0
SIRS Conventional (AUC=0.649)
SIRS Non-ICU (AUC=0.754)
SIRS Retrospective (AUC=0.742)
SIRS Prospective (AUC=0.757)

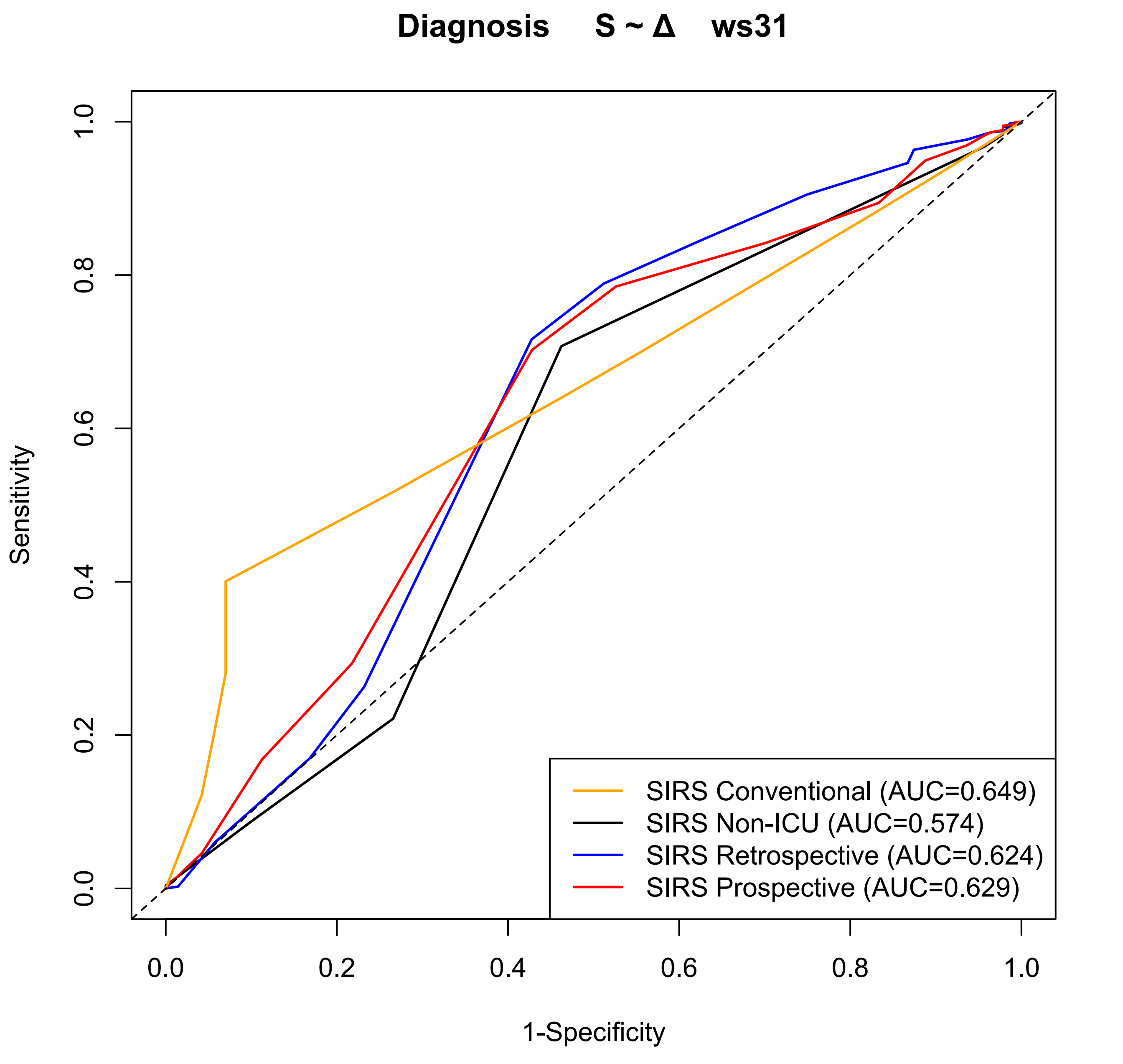

Diagnosis S ~ Δ ws31
Sensitivity
1-Specificity
0.0
0.2
0.4
0.6
0.8
1.0
SIRS Conventional (AUC=0.649)
SIRS Non-ICU (AUC=0.574)
SIRS Retrospective (AUC=0.624)
SIRS Prospective (AUC=0.629)

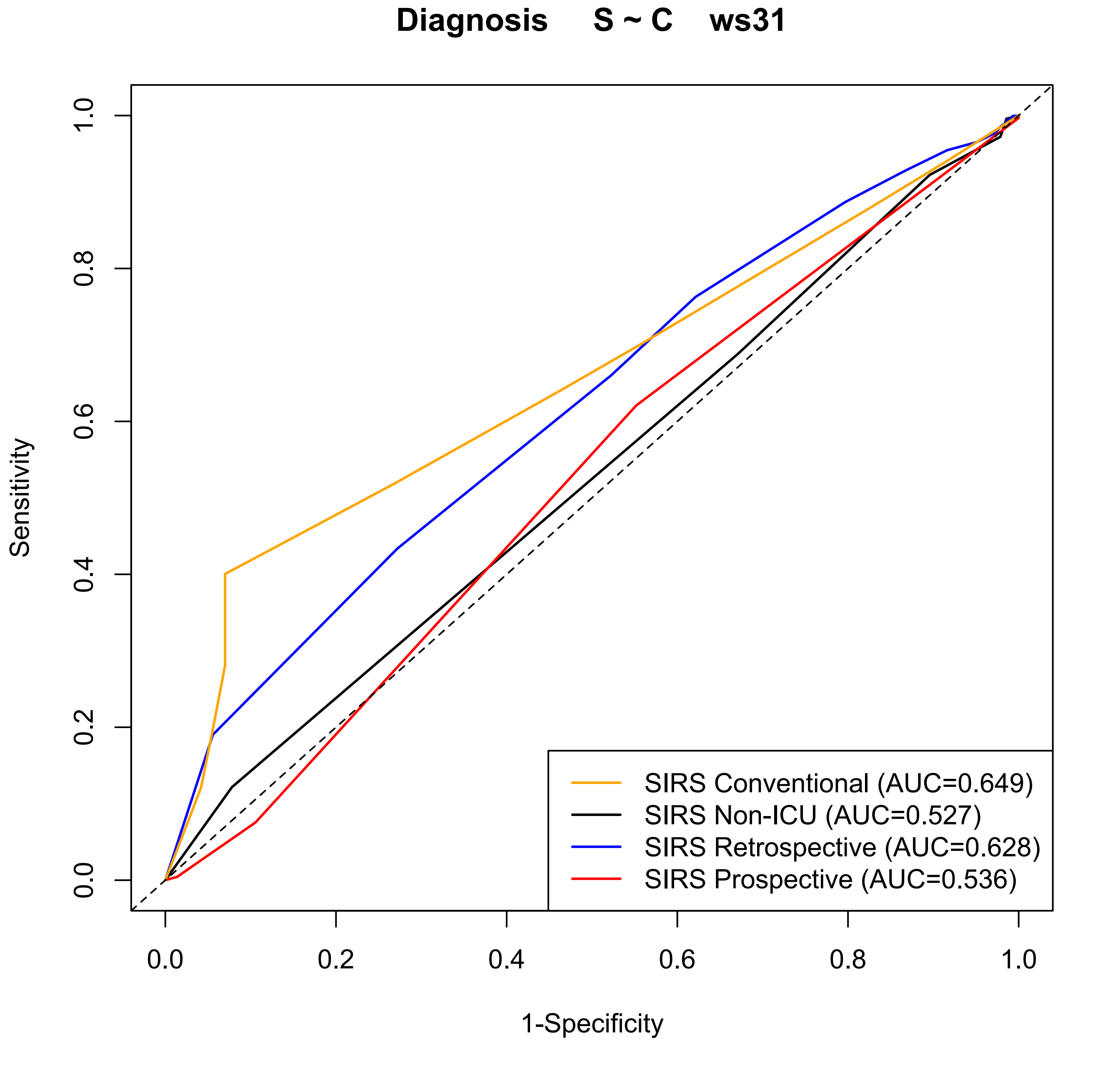

Diagnosis S ~ C ws31
Sensitivity
1-Specificity
0.0
0.2
0.4
0.6
0.8
1.0
SIRS Conventional (AUC=0.649)
SIRS Non-ICU (AUC=0.527)
SIRS Retrospective (AUC=0.628)
SIRS Prospective (AUC=0.536)

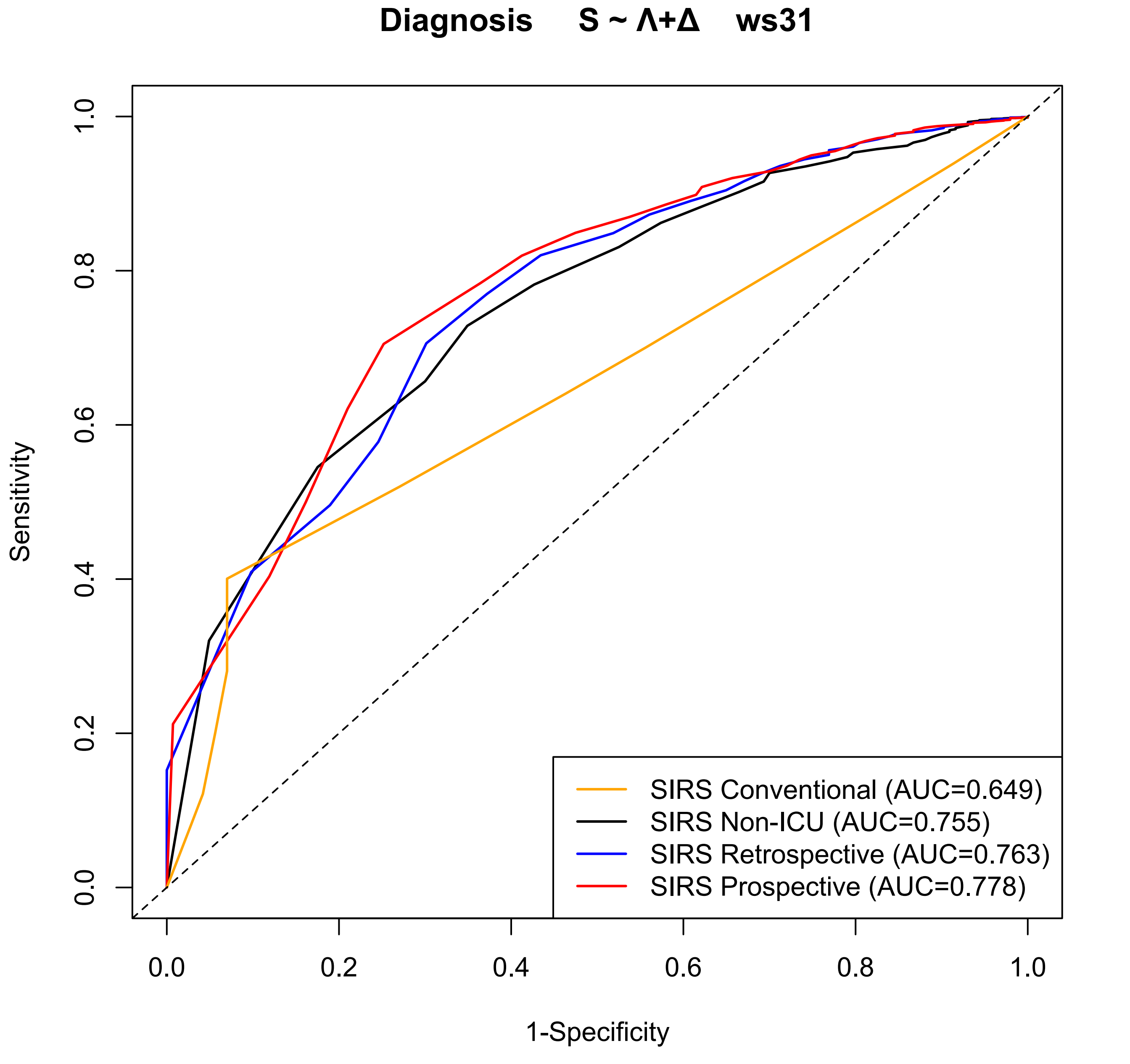
Diagnosis S ~ Λ+Δ ws31
Sensitivity
1-Specificity
0.0
0.2
0.4
0.6
0.8
1.0
SIRS Conventional (AUC=0.649)
SIRS Non-ICU (AUC=0.755)
SIRS Retrospective (AUC=0.763)
SIRS Prospective (AUC=0.778)

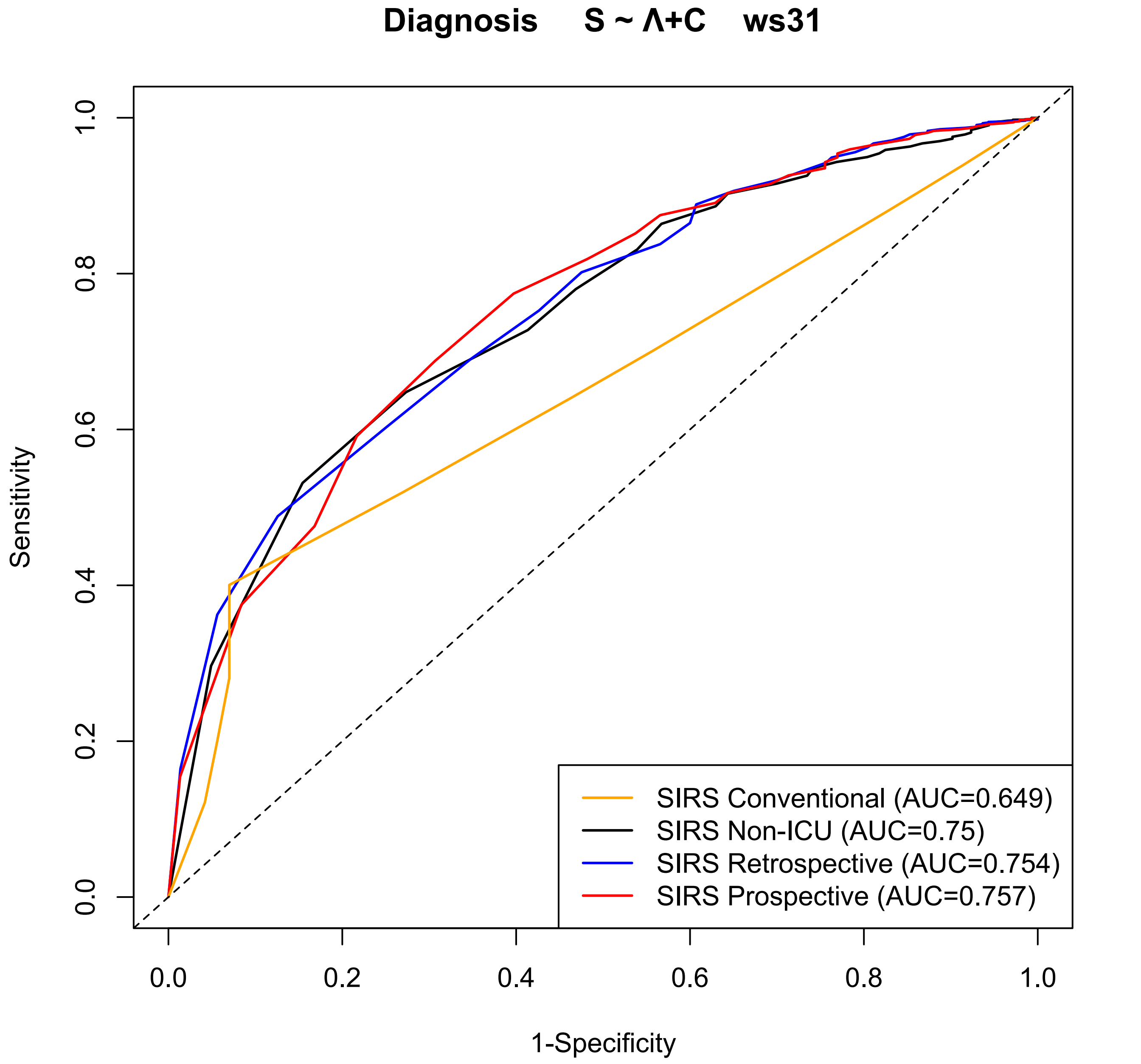
Diagnosis S ~ Λ+C ws31
Sensitivity
1-Specificity
0.0
0.2
0.4
0.6
0.8
1.0
SIRS Conventional (AUC=0.649)
SIRS Non-ICU (AUC=0.75)
SIRS Retrospective (AUC=0.754)
SIRS Prospective (AUC=0.757)

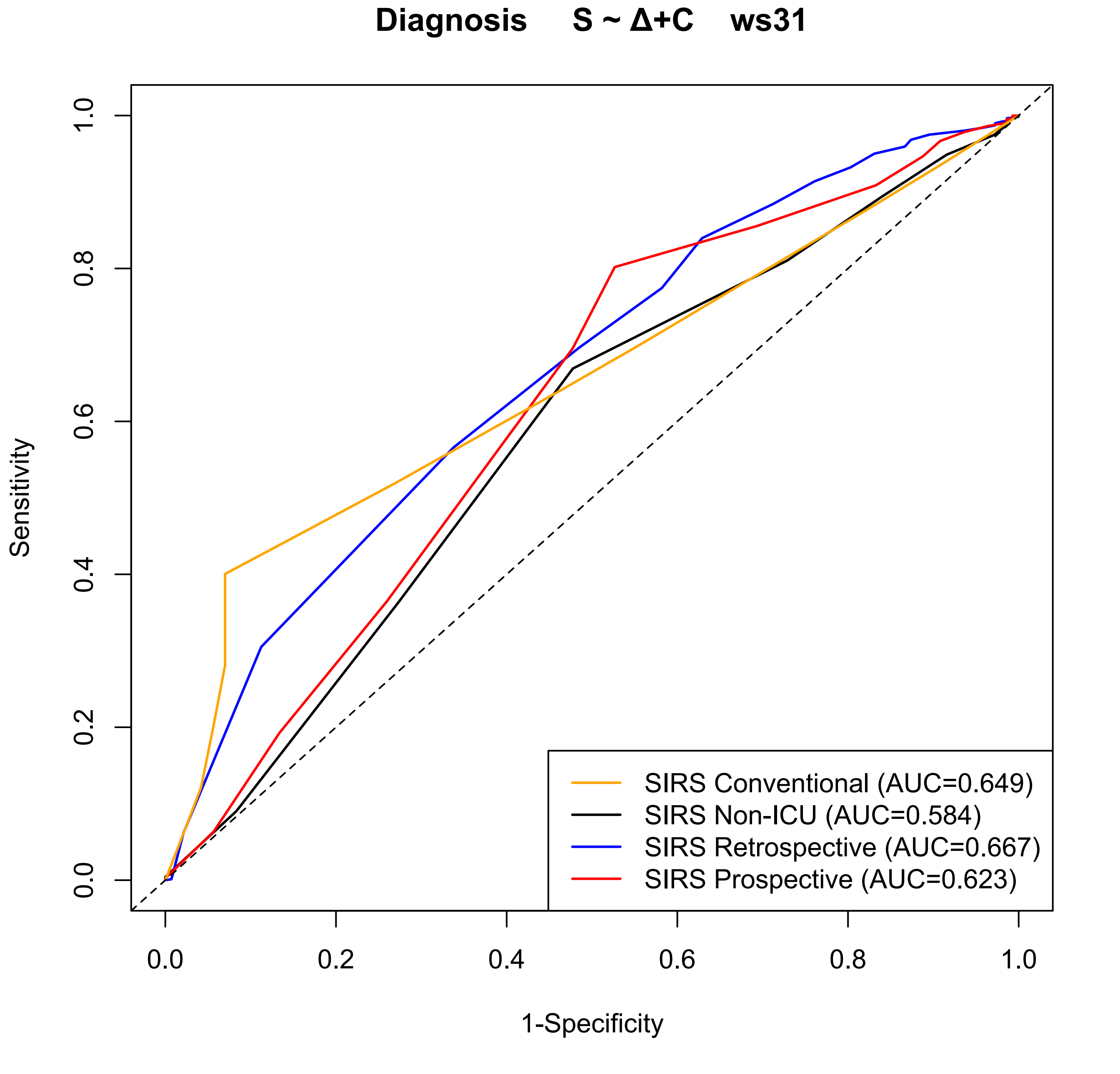

Diagnosis S ~ Δ+C ws31
Sensitivity
1-Specificity
0.0
0.2
0.4
0.6
0.8
1.0
SIRS Conventional (AUC=0.649)
SIRS Non-ICU (AUC=0.584)
SIRS Retrospective (AUC=0.667)
SIRS Prospective (AUC=0.623)

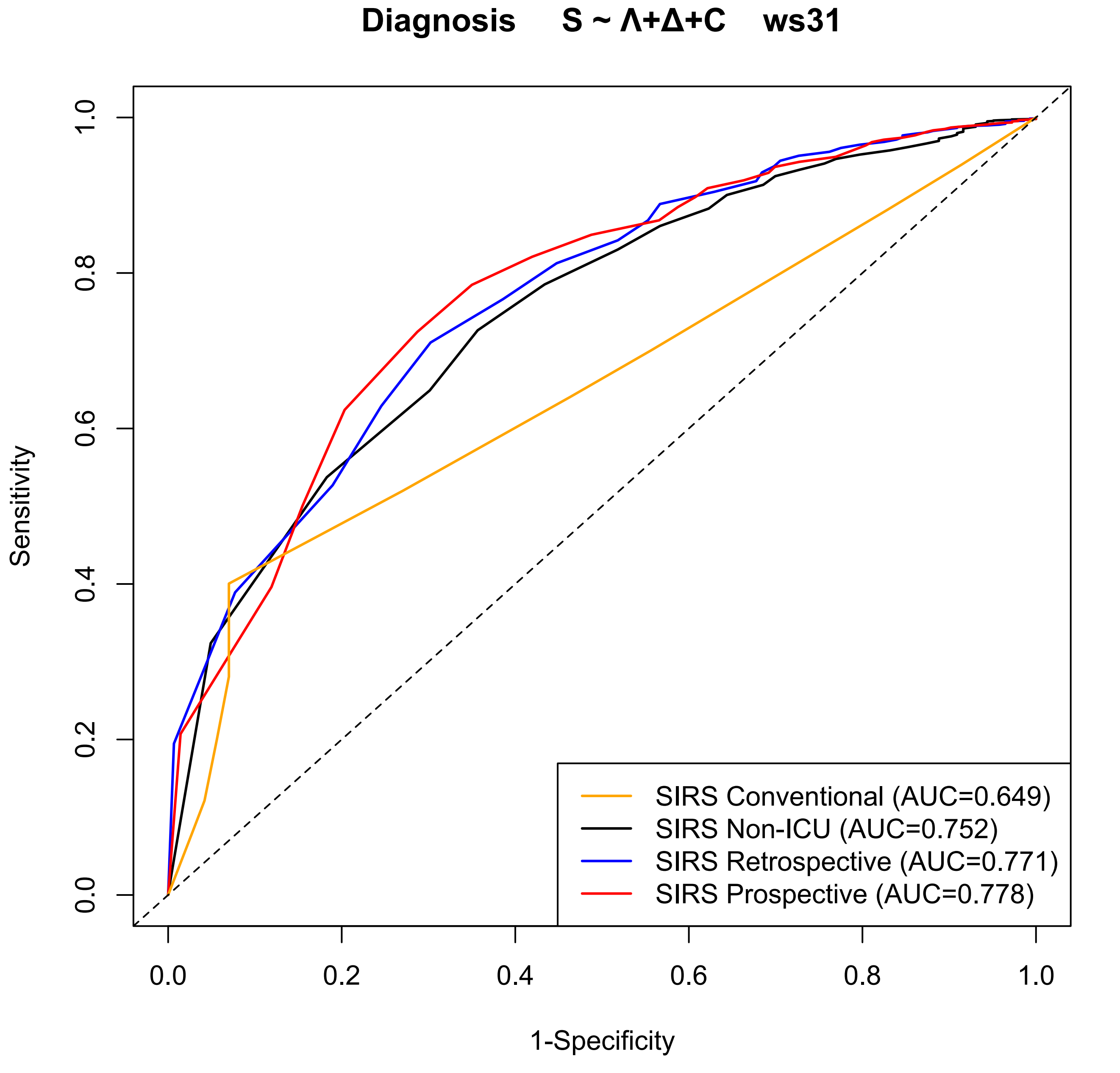
Diagnosis S ~ Λ+Δ+C ws31
Sensitivity
1-Specificity
0.0
0.2
0.4
0.6
0.8
1.0
SIRS Conventional (AUC=0.649)
SIRS Non-ICU (AUC=0.752)
SIRS Retrospective (AUC=0.771)
SIRS Prospective (AUC=0.778)

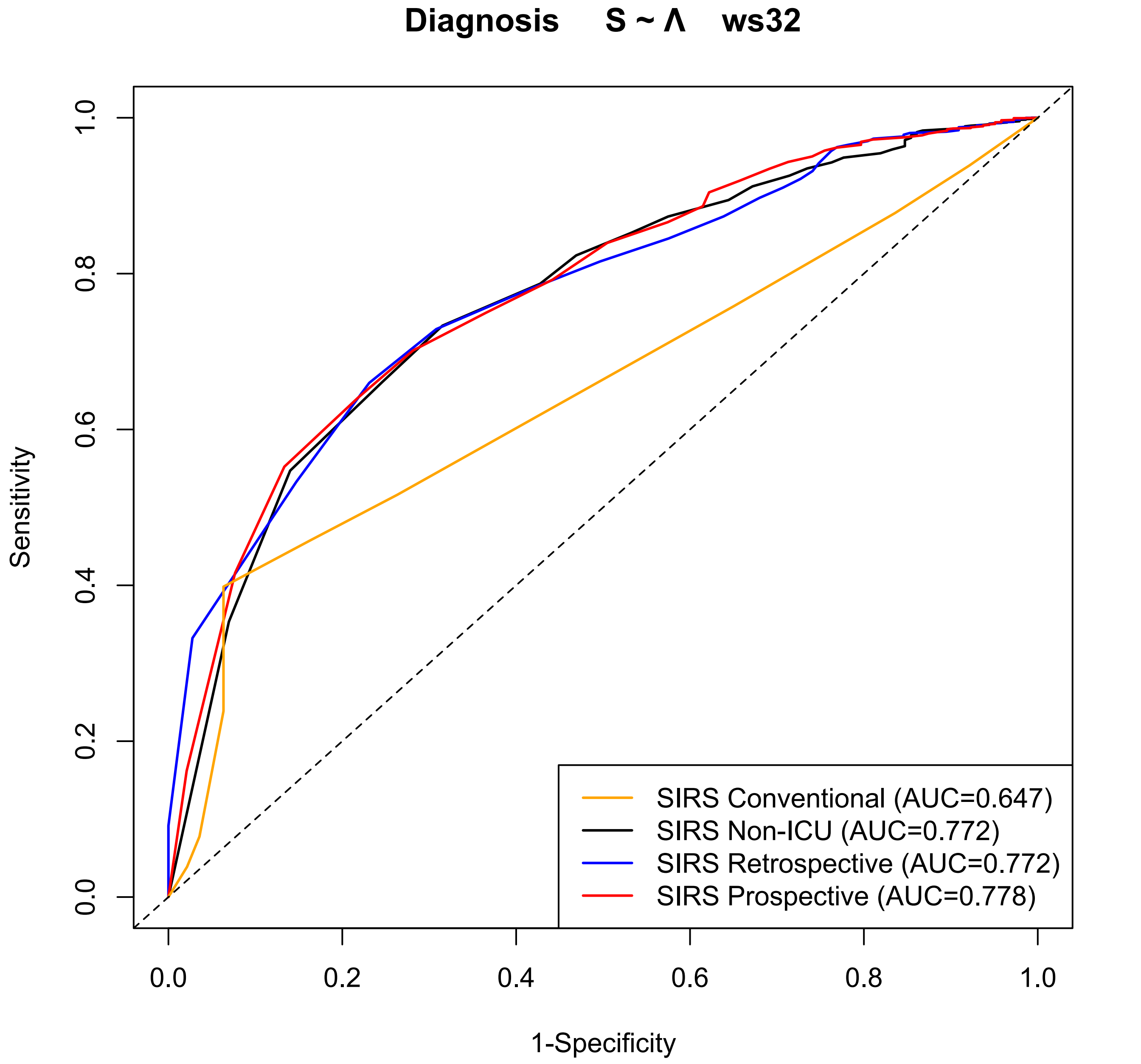

Diagnosis S ~ Λ ws32
Sensitivity
1-Specificity
0.0
0.2
0.4
0.6
0.8
1.0
SIRS Conventional (AUC=0.647)
SIRS Non-ICU (AUC=0.772)
SIRS Retrospective (AUC=0.772)
SIRS Prospective (AUC=0.778)

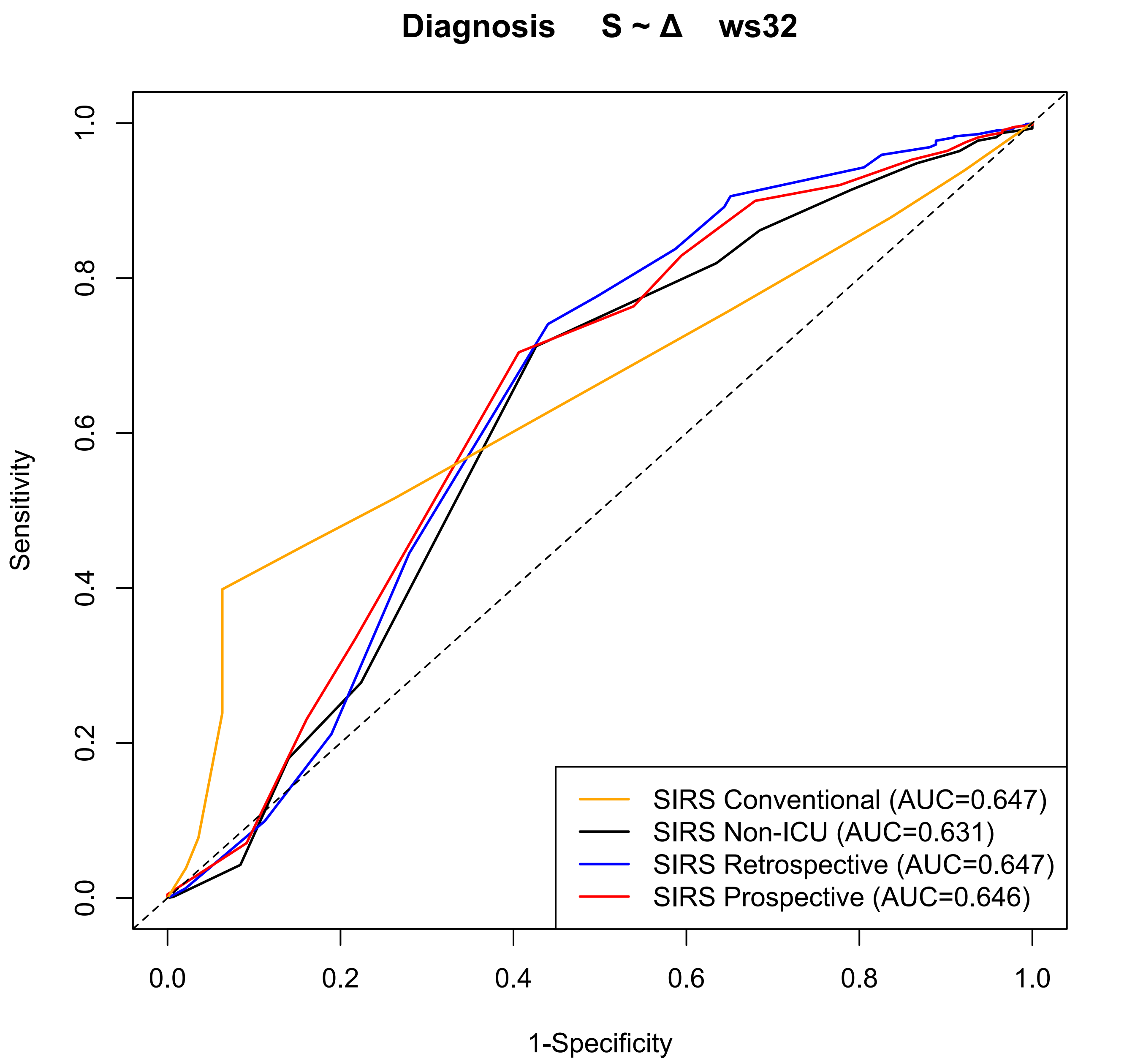

Diagnosis S ~ Δ ws32
Sensitivity
1-Specificity
0.0
0.2
0.4
0.6
0.8
1.0
SIRS Conventional (AUC=0.647)
SIRS Non-ICU (AUC=0.631)
SIRS Retrospective (AUC=0.647)
SIRS Prospective (AUC=0.646)

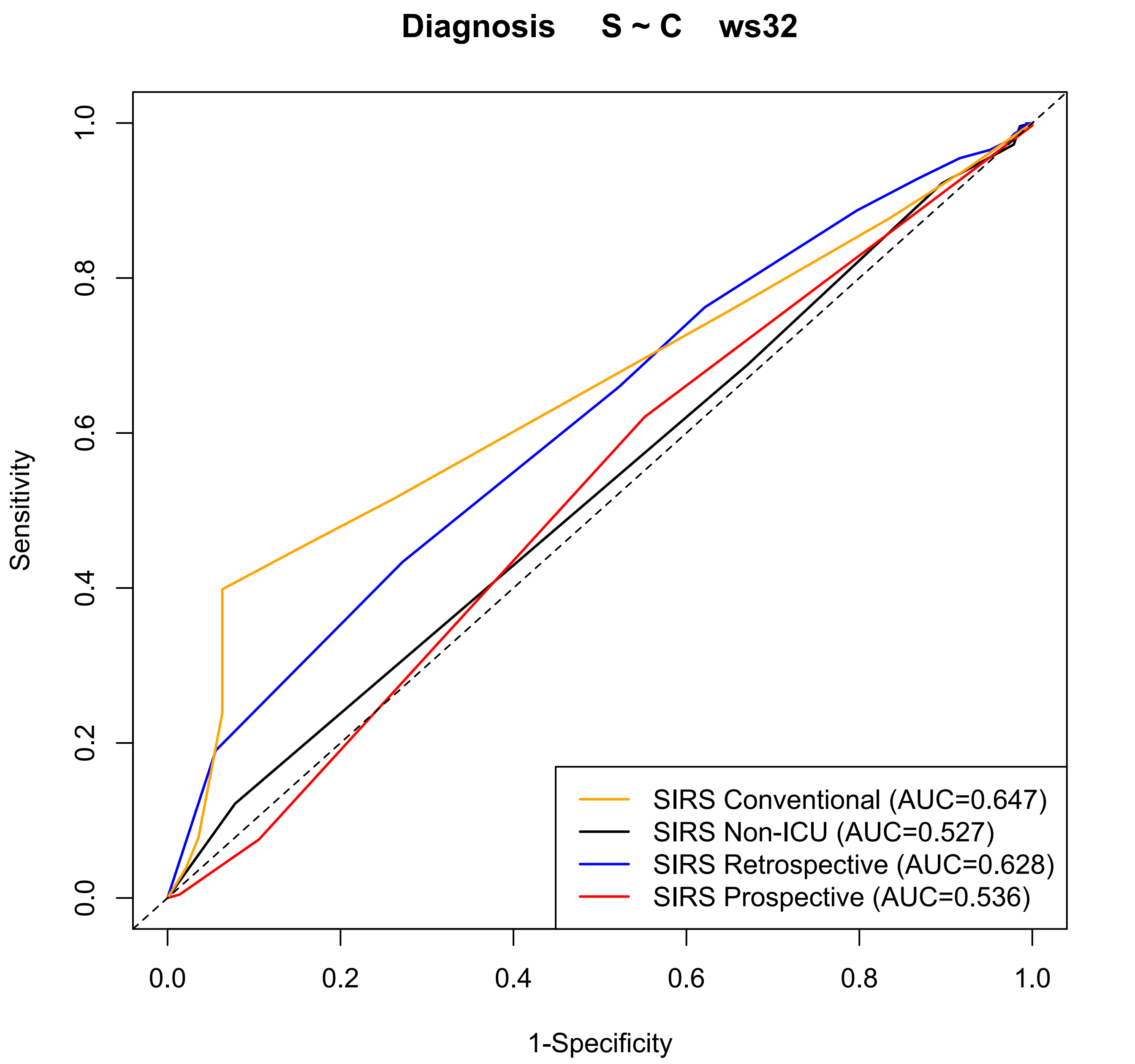

Diagnosis S ~ C ws32
Sensitivity
1-Specificity
0.0
0.2
0.4
0.6
0.8
1.0
0.0
0.2
0.4
0.6
0.8
1.0
SIRS Conventional (AUC=0.647)
SIRS Non-ICU (AUC=0.527)
SIRS Retrospective (AUC=0.628)
SIRS Prospective (AUC=0.536)

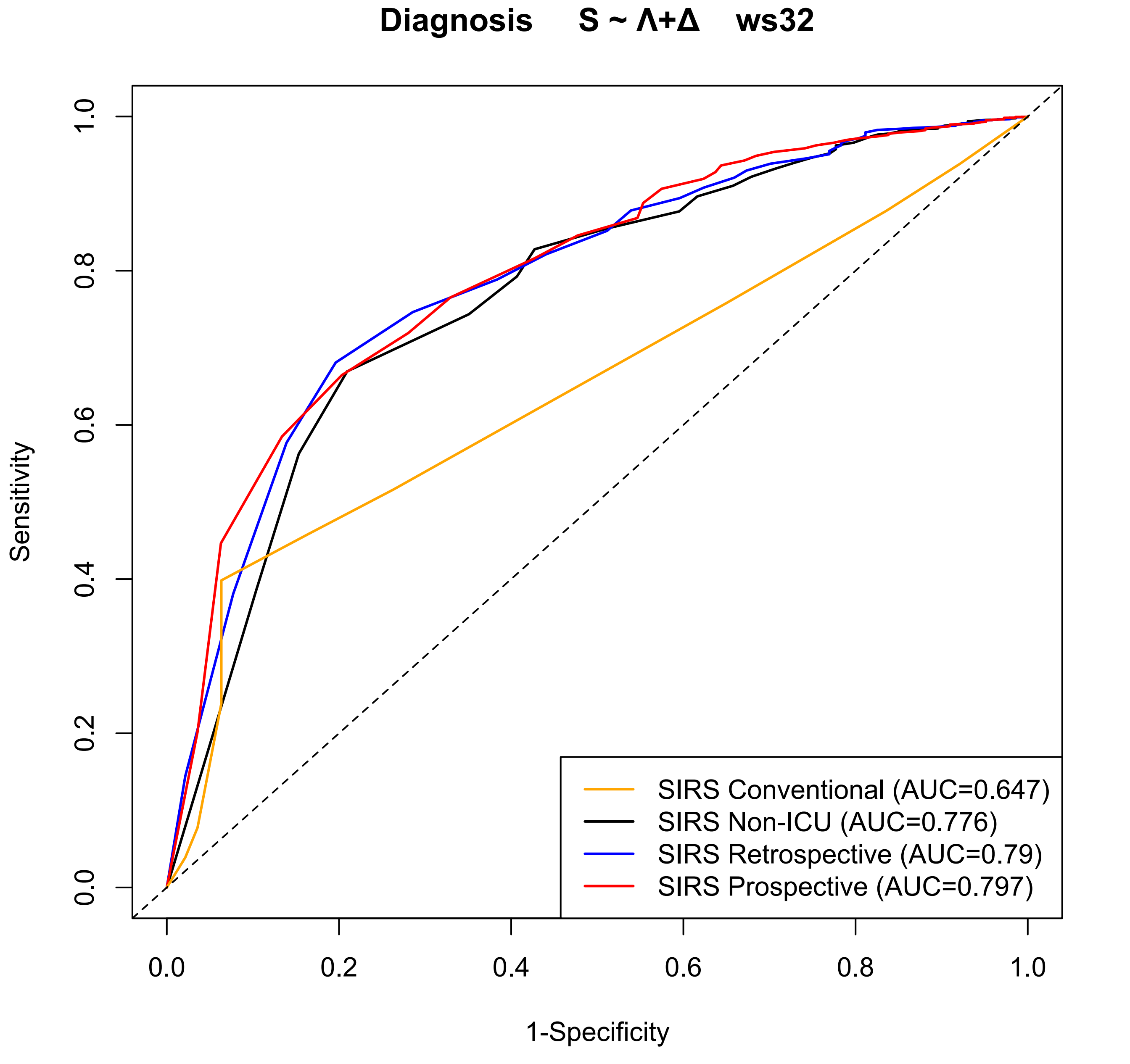
Diagnosis S ~ Λ+Δ ws32
Sensitivity
1-Specificity
0.0
0.2
0.4
0.6
0.8
1.0
SIRS Conventional (AUC=0.647)
SIRS Non-ICU (AUC=0.776)
SIRS Retrospective (AUC=0.79)
SIRS Prospective (AUC=0.797)

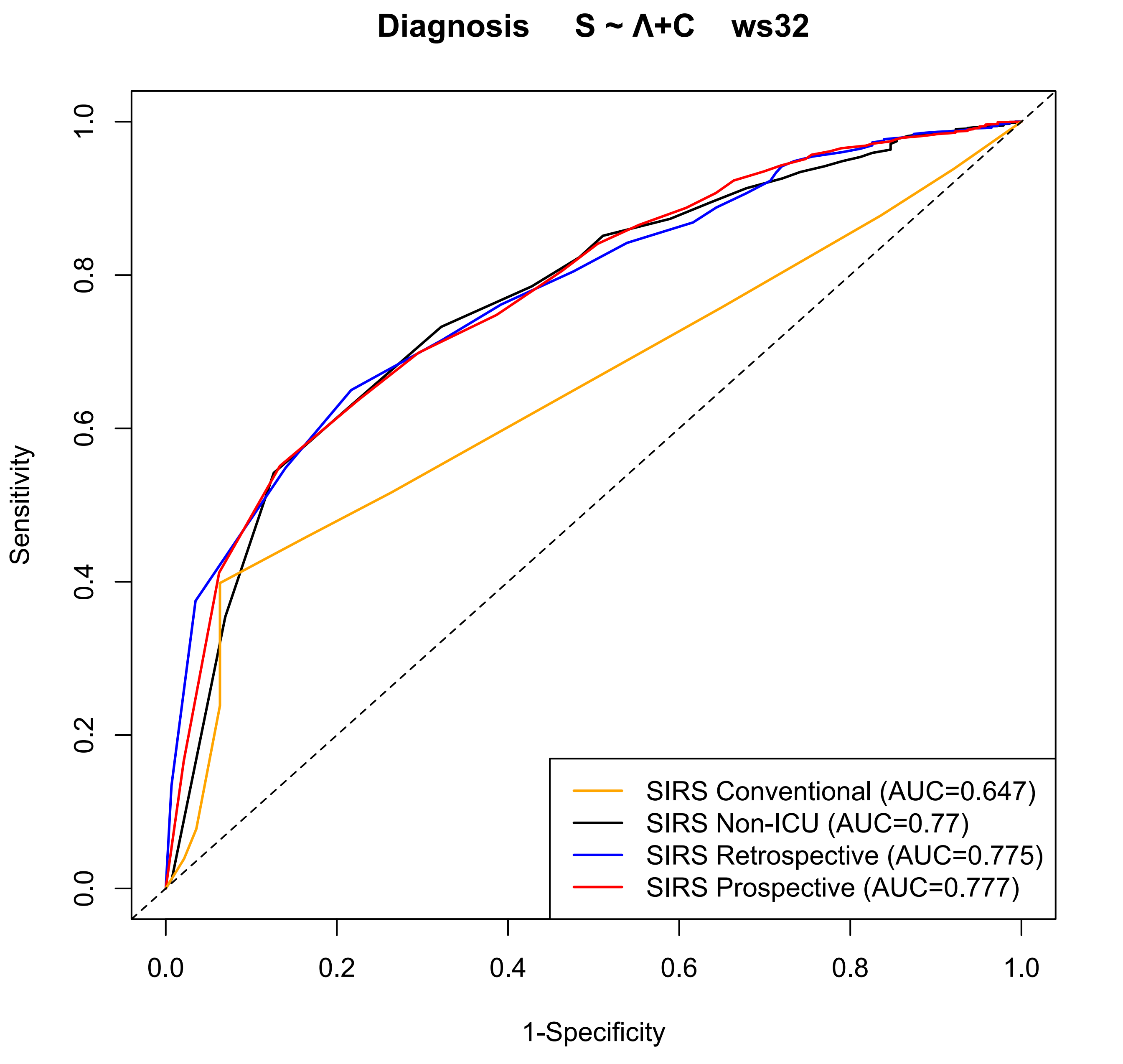

Diagnosis S ~ Λ+C ws32
Sensitivity
1-Specificity
0.0
0.2
0.4
0.6
0.8
1.0
0.0
0.2
0.4
0.6
0.8
1.0
SIRS Conventional (AUC=0.647)
SIRS Non-ICU (AUC=0.77)
SIRS Retrospective (AUC=0.775)
SIRS Prospective (AUC=0.777)

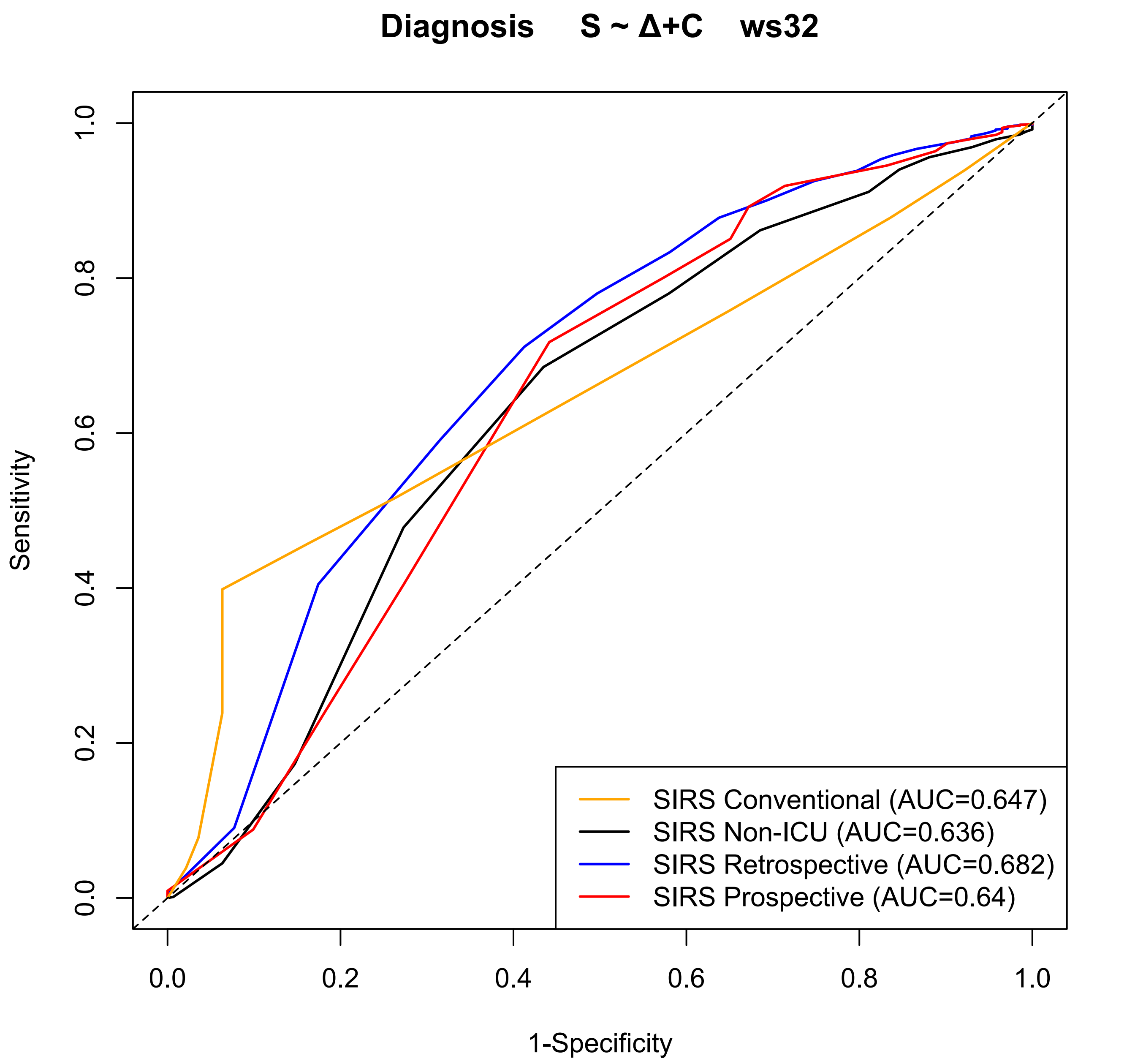

Diagnosis S ~ Δ+C ws32
Sensitivity
1-Specificity
0.0
0.2
0.4
0.6
0.8
1.0
SIRS Conventional (AUC=0.647)
SIRS Non-ICU (AUC=0.636)
SIRS Retrospective (AUC=0.682)
SIRS Prospective (AUC=0.64)

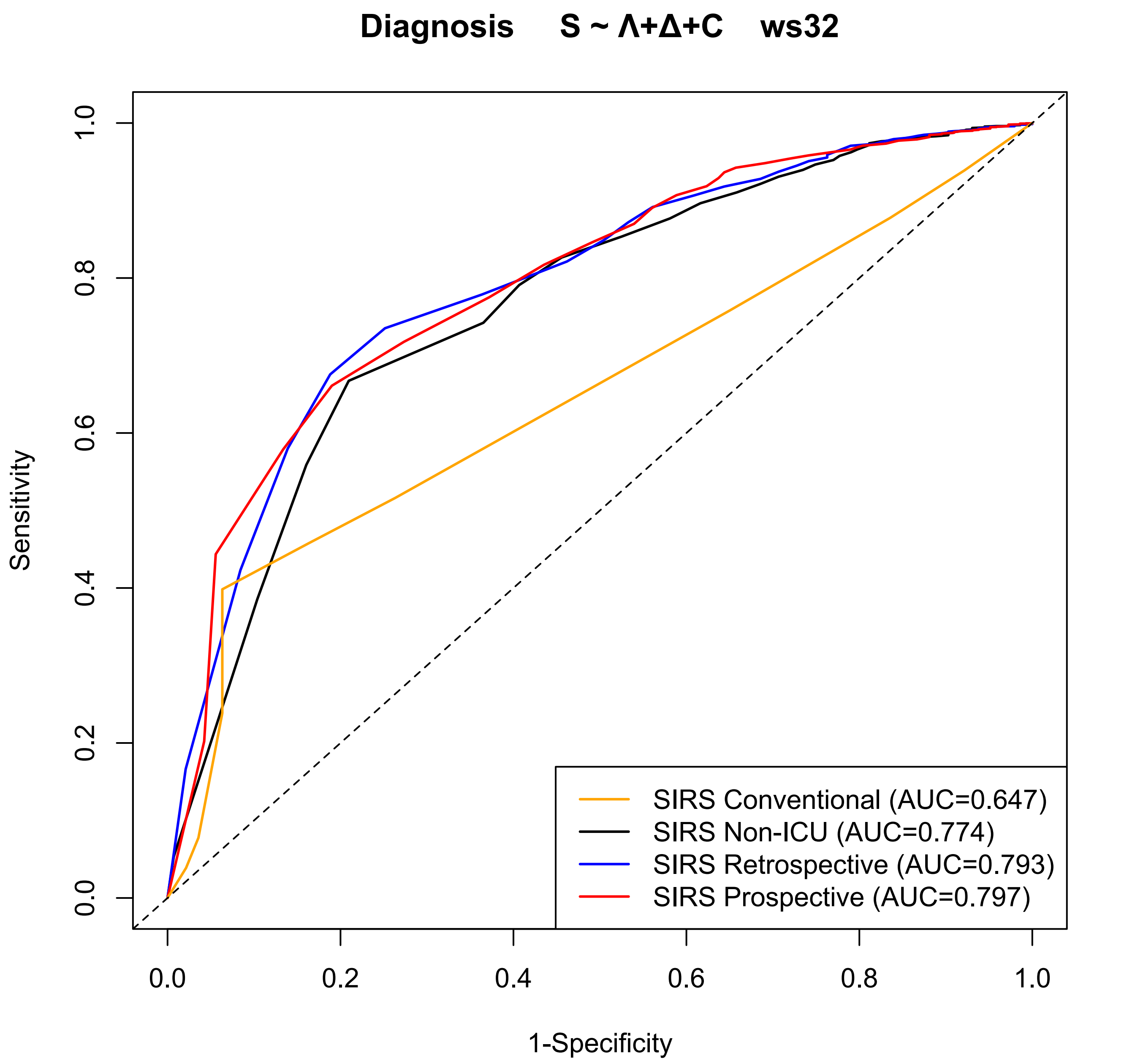

Diagnosis S ~ Λ+Δ+C ws32
Sensitivity
1-Specificity
0.0
0.2
0.4
0.6
0.8
1.0
SIRS Conventional (AUC=0.647)
SIRS Non-ICU (AUC=0.774)
SIRS Retrospective (AUC=0.793)
SIRS Prospective (AUC=0.797)

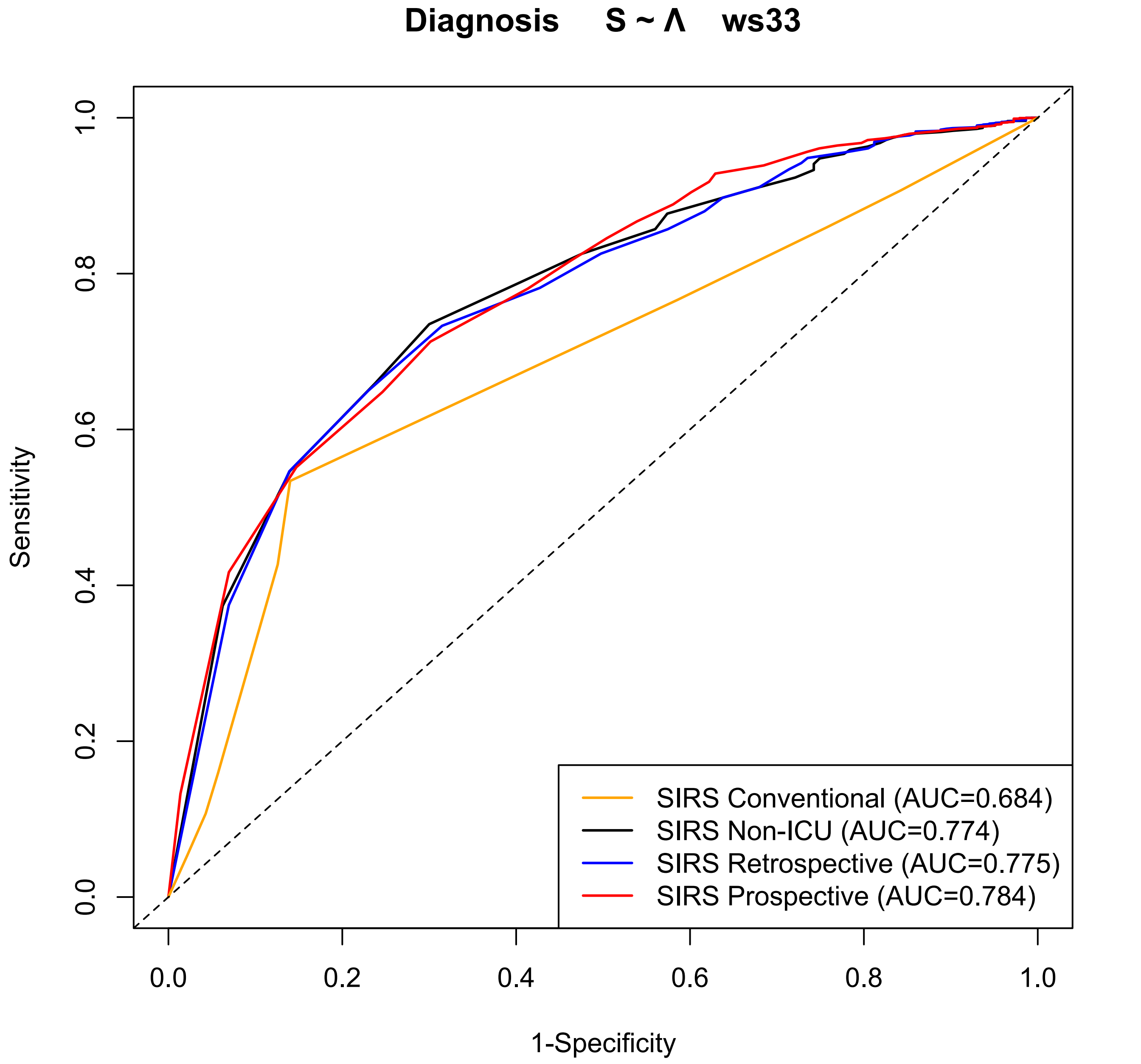
Diagnosis S ~ Λ ws33
Sensitivity
1-Specificity
0.0
0.2
0.4
0.6
0.8
1.0
0.0
0.2
0.4
0.6
0.8
1.0
SIRS Conventional (AUC=0.684)
SIRS Non-ICU (AUC=0.774)
SIRS Retrospective (AUC=0.775)
SIRS Prospective (AUC=0.784)

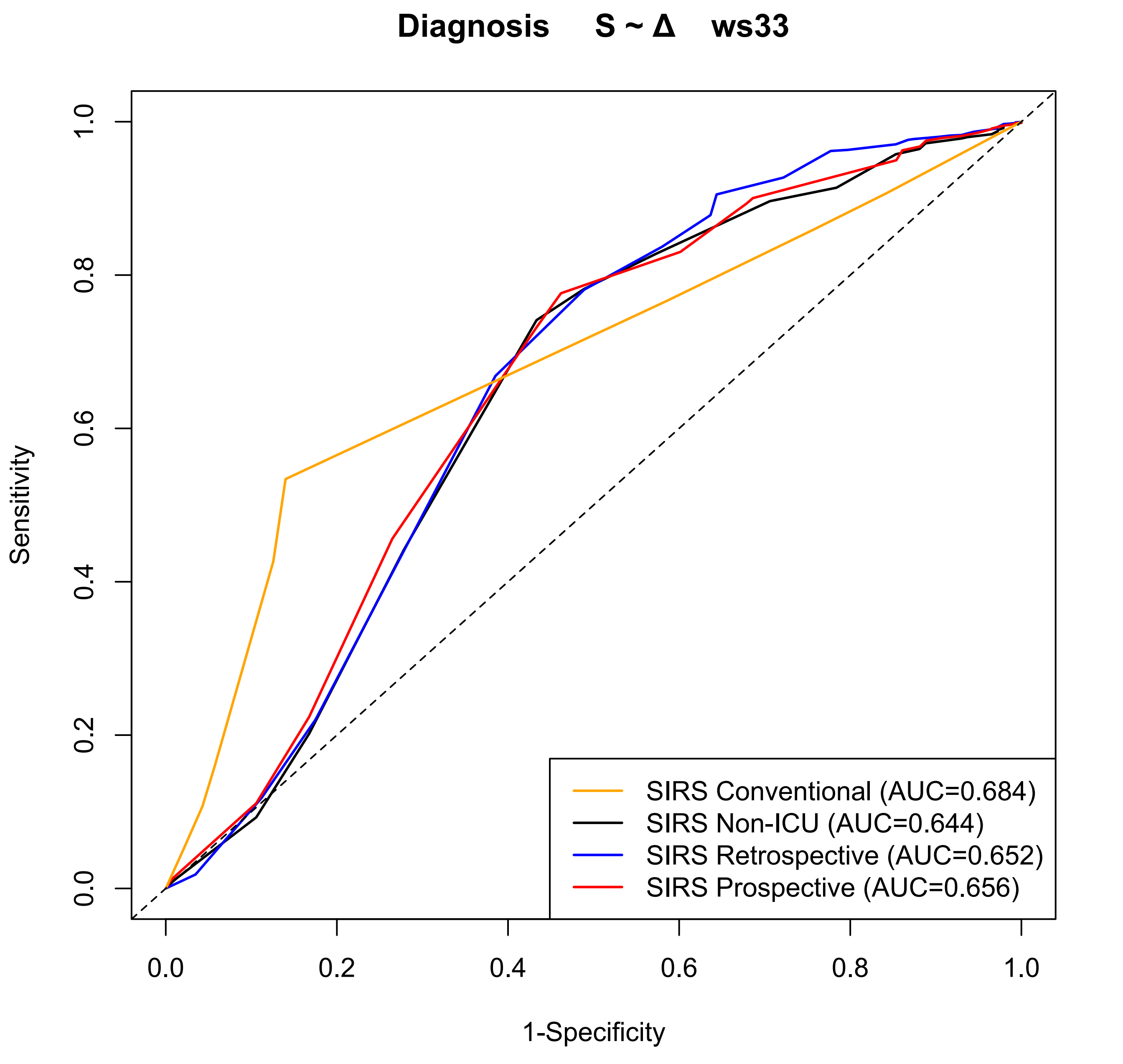

Diagnosis S ~ Δ ws33
Sensitivity
1-Specificity
0.0
0.2
0.4
0.6
0.8
1.0
0.0
0.2
0.4
0.6
0.8
1.0
SIRS Conventional (AUC=0.684)
SIRS Non-ICU (AUC=0.644)
SIRS Retrospective (AUC=0.652)
SIRS Prospective (AUC=0.656)

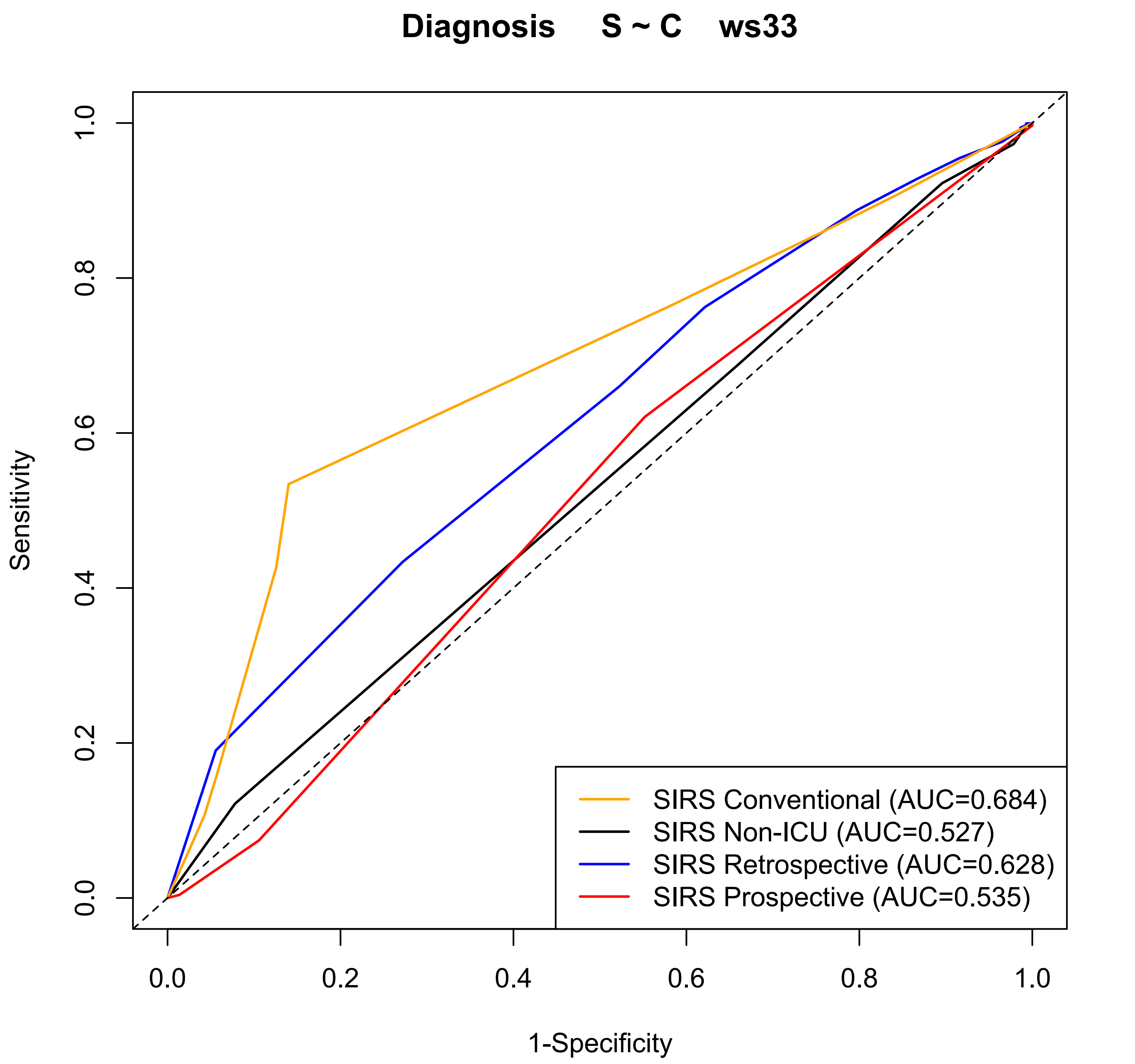

Diagnosis S ~ C ws33
Sensitivity
1-Specificity
0.0
0.2
0.4
0.6
0.8
1.0
SIRS Conventional (AUC=0.684)
SIRS Non-ICU (AUC=0.527)
SIRS Retrospective (AUC=0.628)
SIRS Prospective (AUC=0.535)

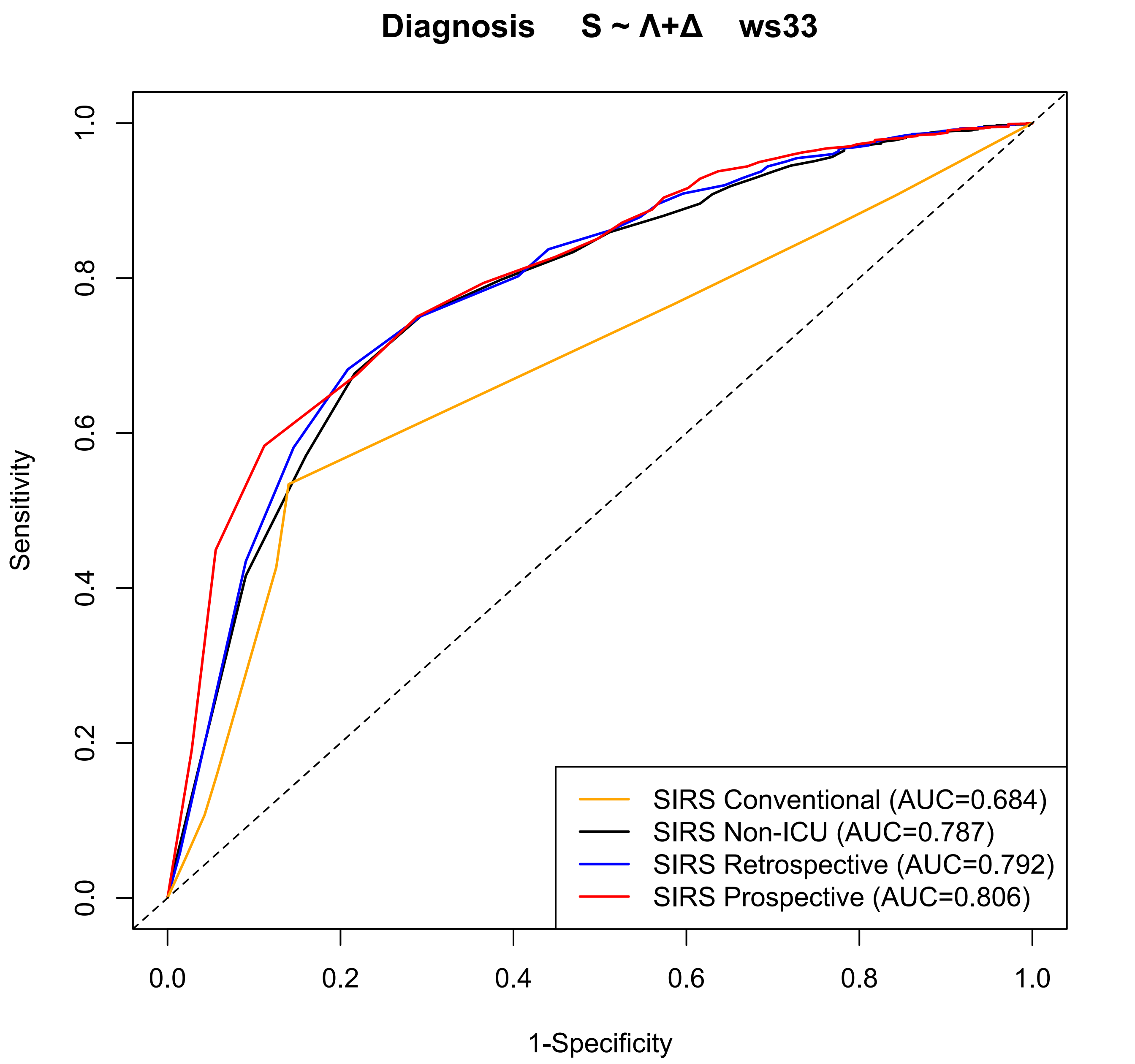
Diagnosis S ~ Λ+Δ ws33
Sensitivity
1-Specificity
0.0
0.2
0.4
0.6
0.8
1.0
0.0
0.2
0.4
0.6
0.8
1.0
SIRS Conventional (AUC=0.684)
SIRS Non-ICU (AUC=0.787)
SIRS Retrospective (AUC=0.792)
SIRS Prospective (AUC=0.806)

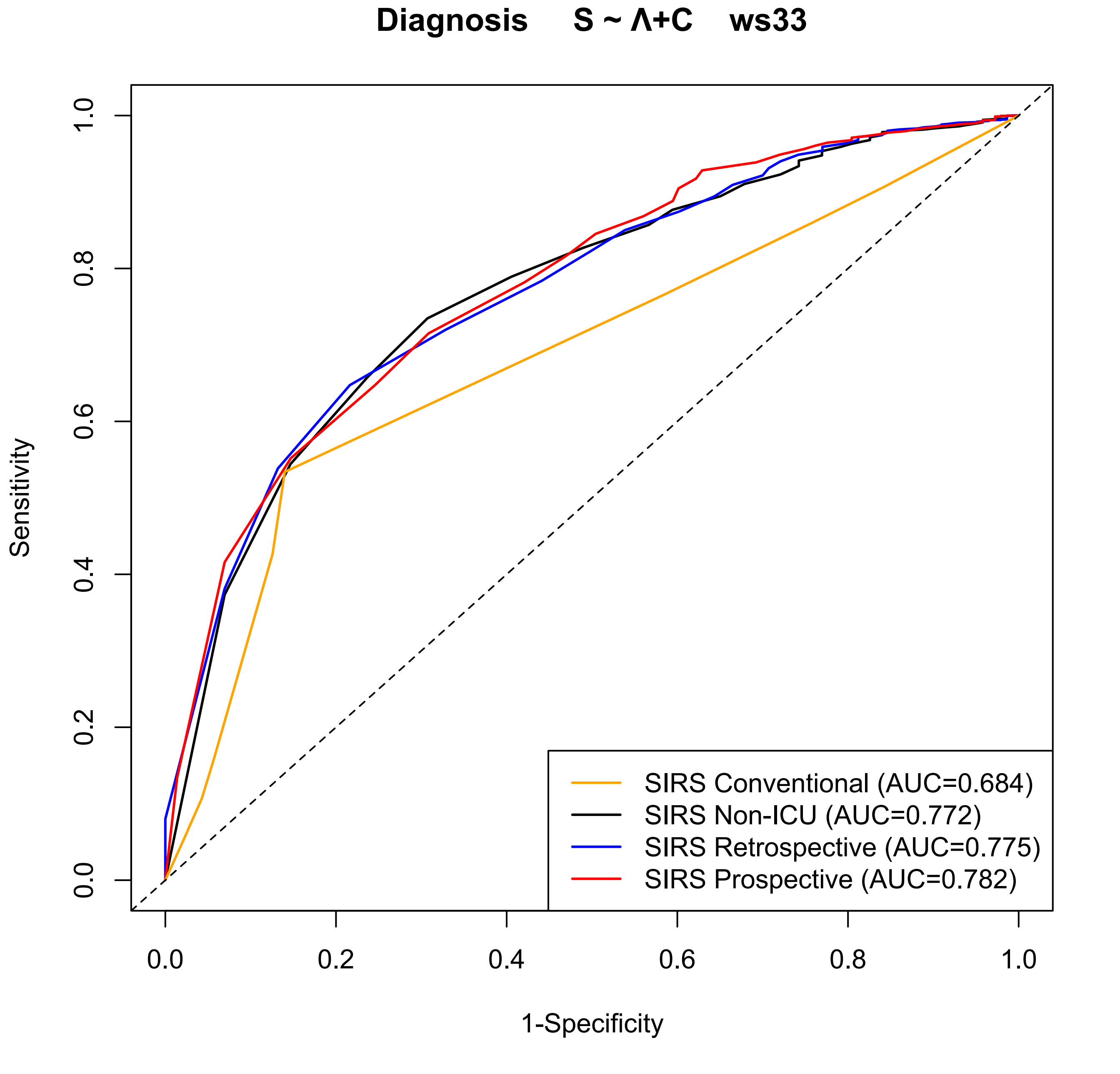
Diagnosis S ~ Λ+C ws33
Sensitivity
1-Specificity
0.0
0.2
0.4
0.6
0.8
1.0
SIRS Conventional (AUC=0.684)
SIRS Non-ICU (AUC=0.772)
SIRS Retrospective (AUC=0.775)
SIRS Prospective (AUC=0.782)

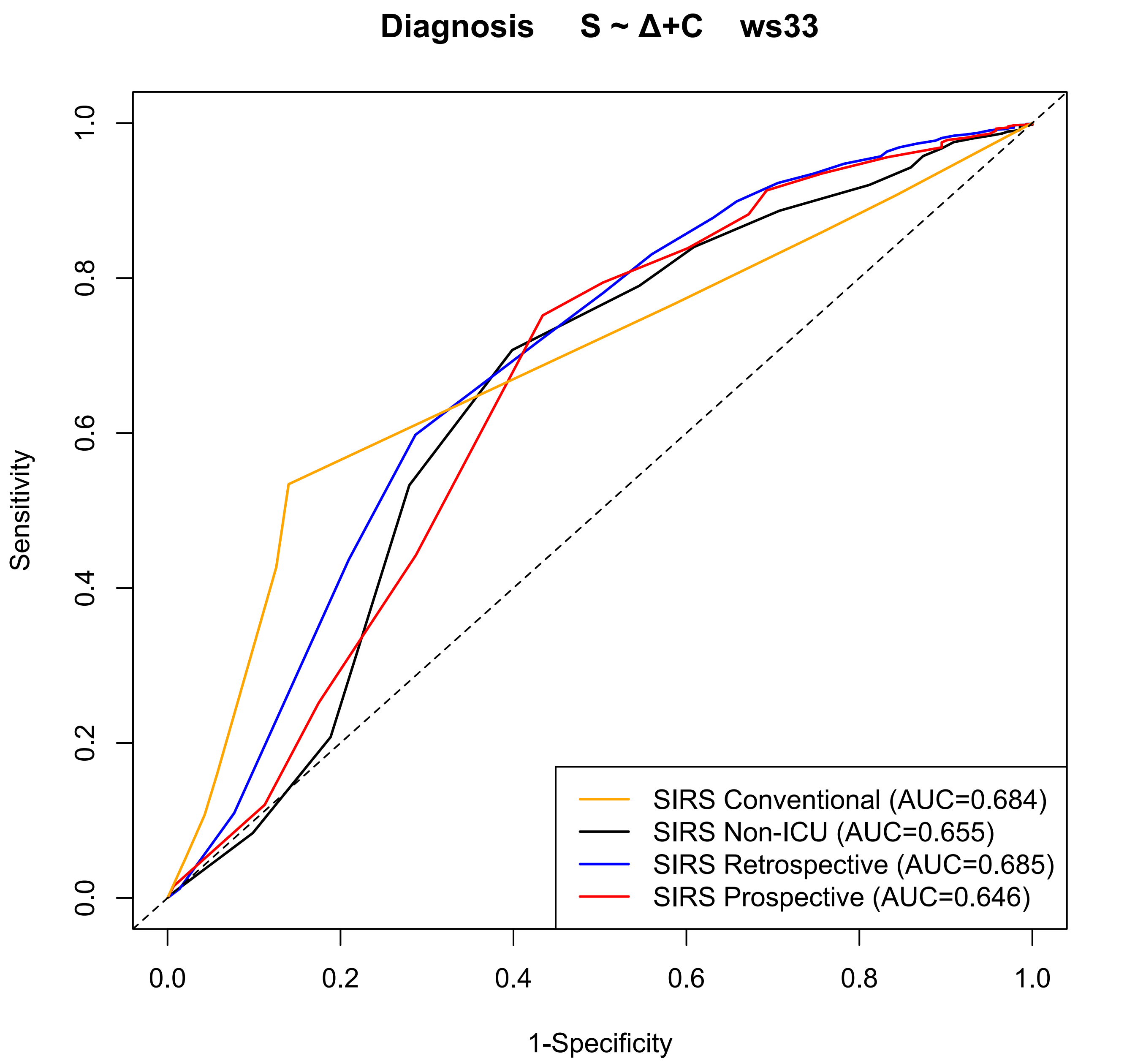
Diagnosis S ~ Δ+C ws33
Sensitivity
1-Specificity
0.0
0.2
0.4
0.6
0.8
1.0
SIRS Conventional (AUC=0.684)
SIRS Non-ICU (AUC=0.655)
SIRS Retrospective (AUC=0.685)
SIRS Prospective (AUC=0.646)

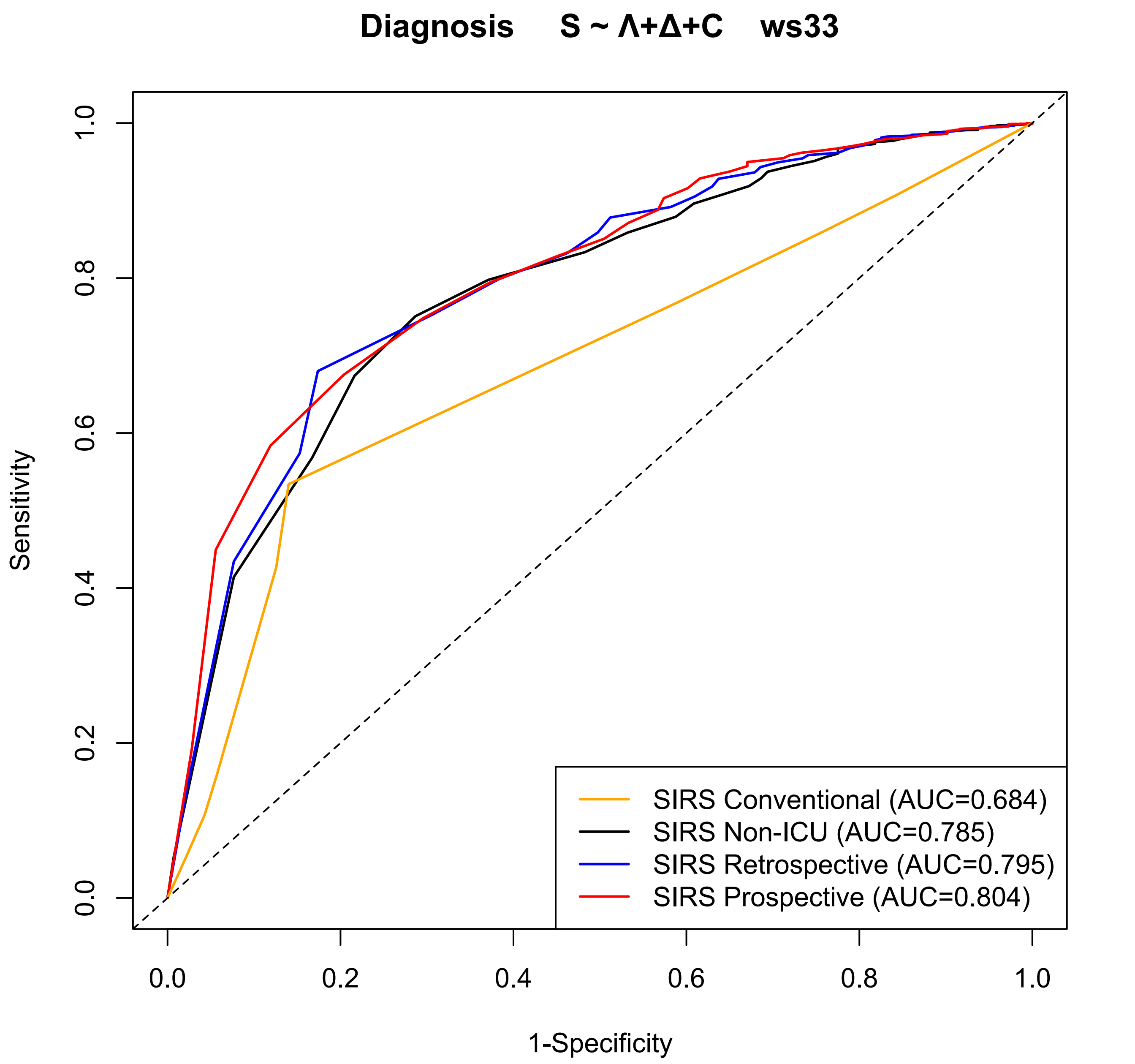

Diagnosis S ~ Λ+Δ+C ws33
Sensitivity
1-Specificity
0.0
0.2
0.4
0.6
0.8
1.0
SIRS Conventional (AUC=0.684)
SIRS Non-ICU (AUC=0.785)
SIRS Retrospective (AUC=0.795)
SIRS Prospective (AUC=0.804)

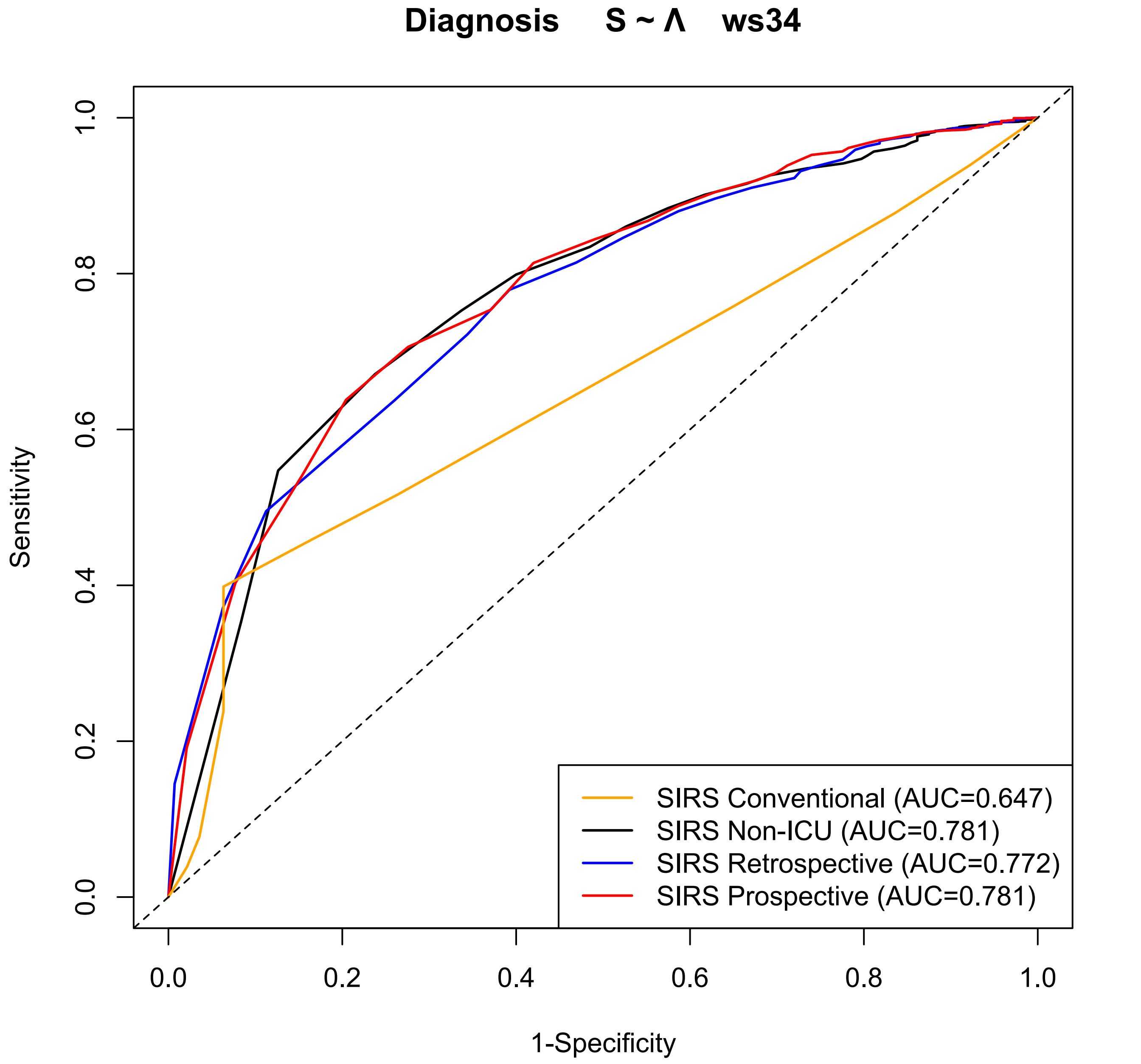
Diagnosis S ~ Λ ws34
Sensitivity
1-Specificity
0.0
0.2
0.4
0.6
0.8
1.0
SIRS Conventional (AUC=0.647)
SIRS Non-ICU (AUC=0.781)
SIRS Retrospective (AUC=0.772)
SIRS Prospective (AUC=0.781)

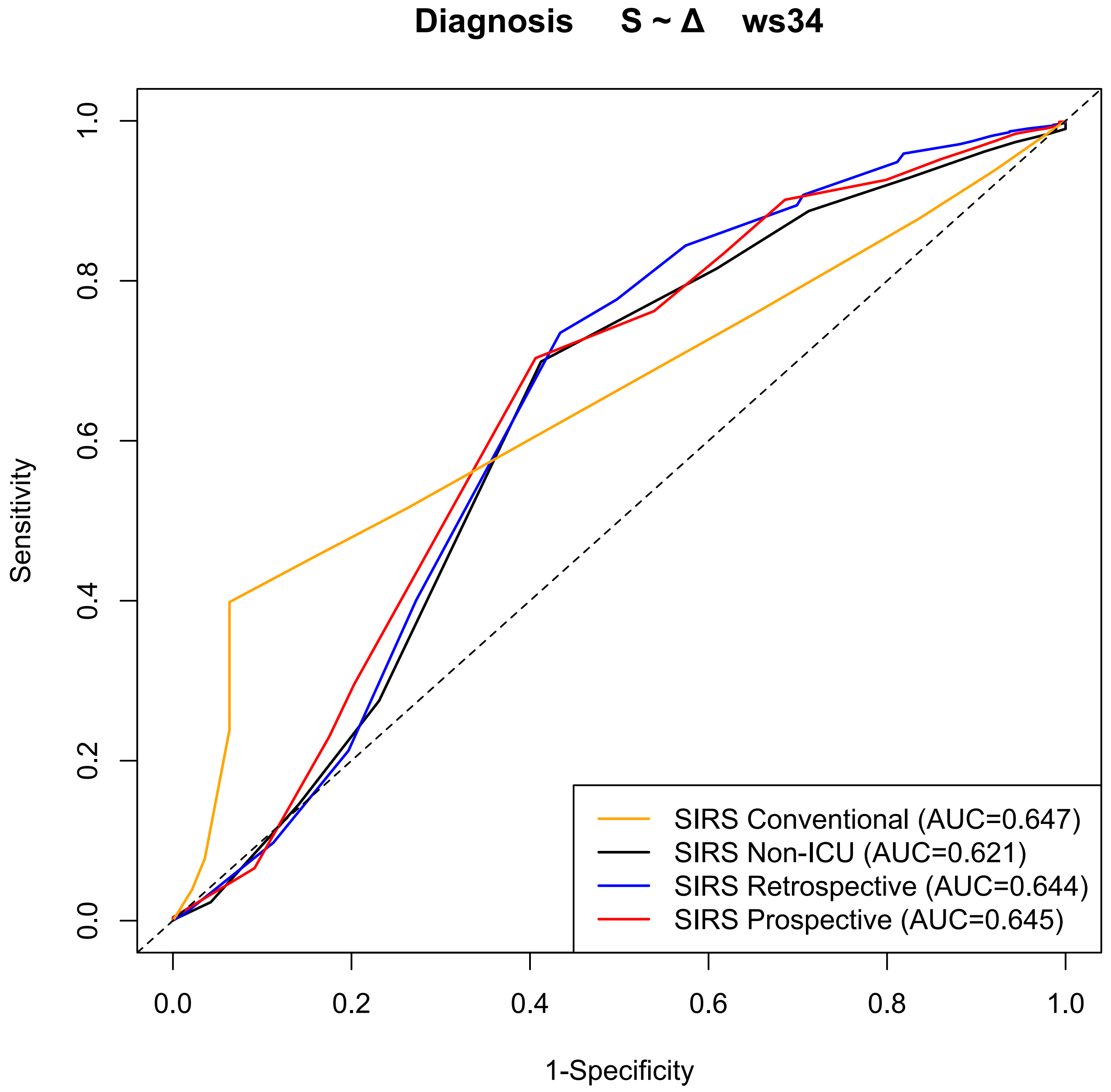

Diagnosis S ~ Δ ws34
Sensitivity
1-Specificity
0.0
0.2
0.4
0.6
0.8
1.0
SIRS Conventional (AUC=0.647)
SIRS Non-ICU (AUC=0.621)
SIRS Retrospective (AUC=0.644)
SIRS Prospective (AUC=0.645)

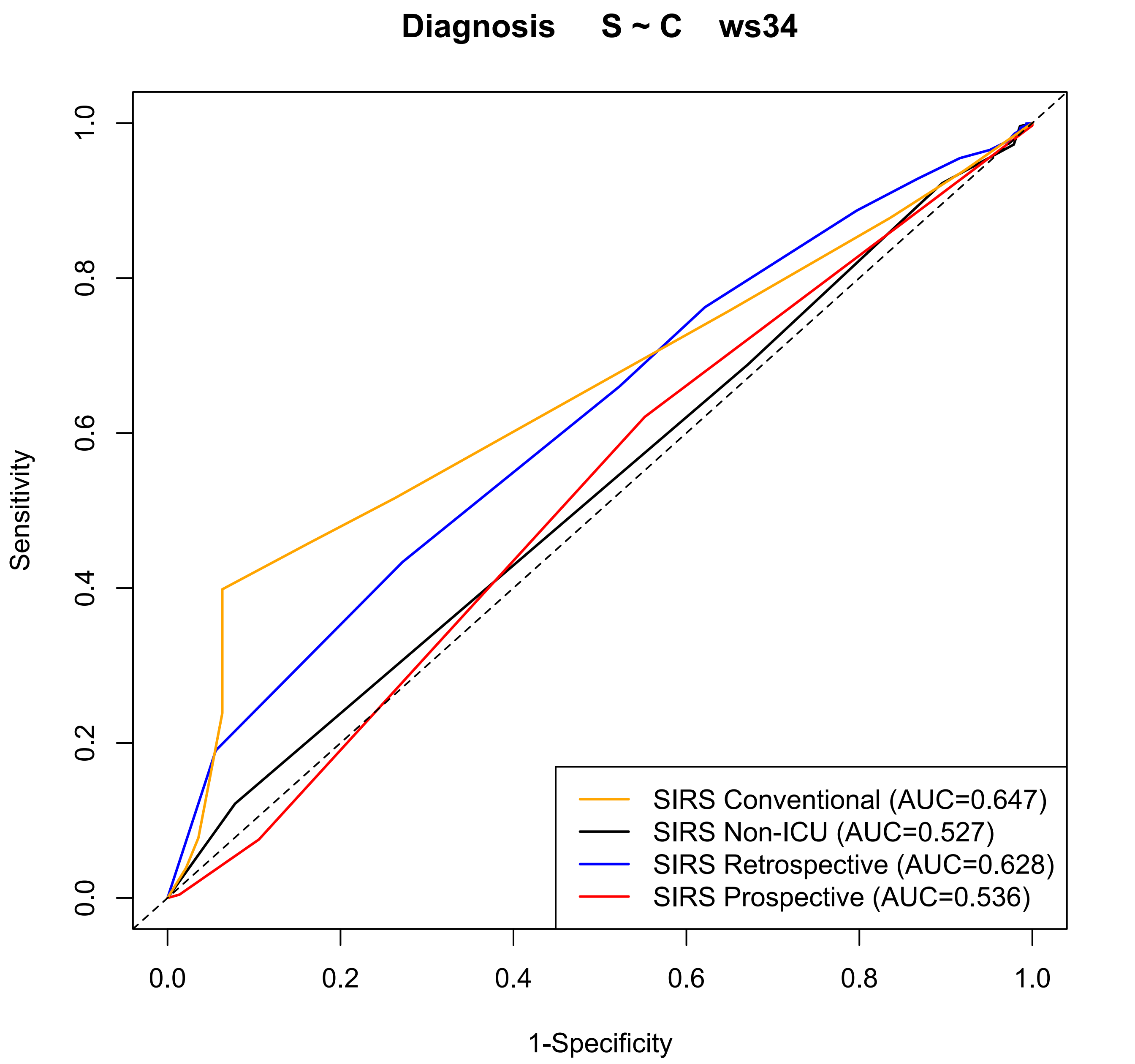
Diagnosis S ~ C ws34
Sensitivity
1-Specificity
0.0
0.2
0.4
0.6
0.8
1.0
SIRS Conventional (AUC=0.647)
SIRS Non-ICU (AUC=0.527)
SIRS Retrospective (AUC=0.628)
SIRS Prospective (AUC=0.536)

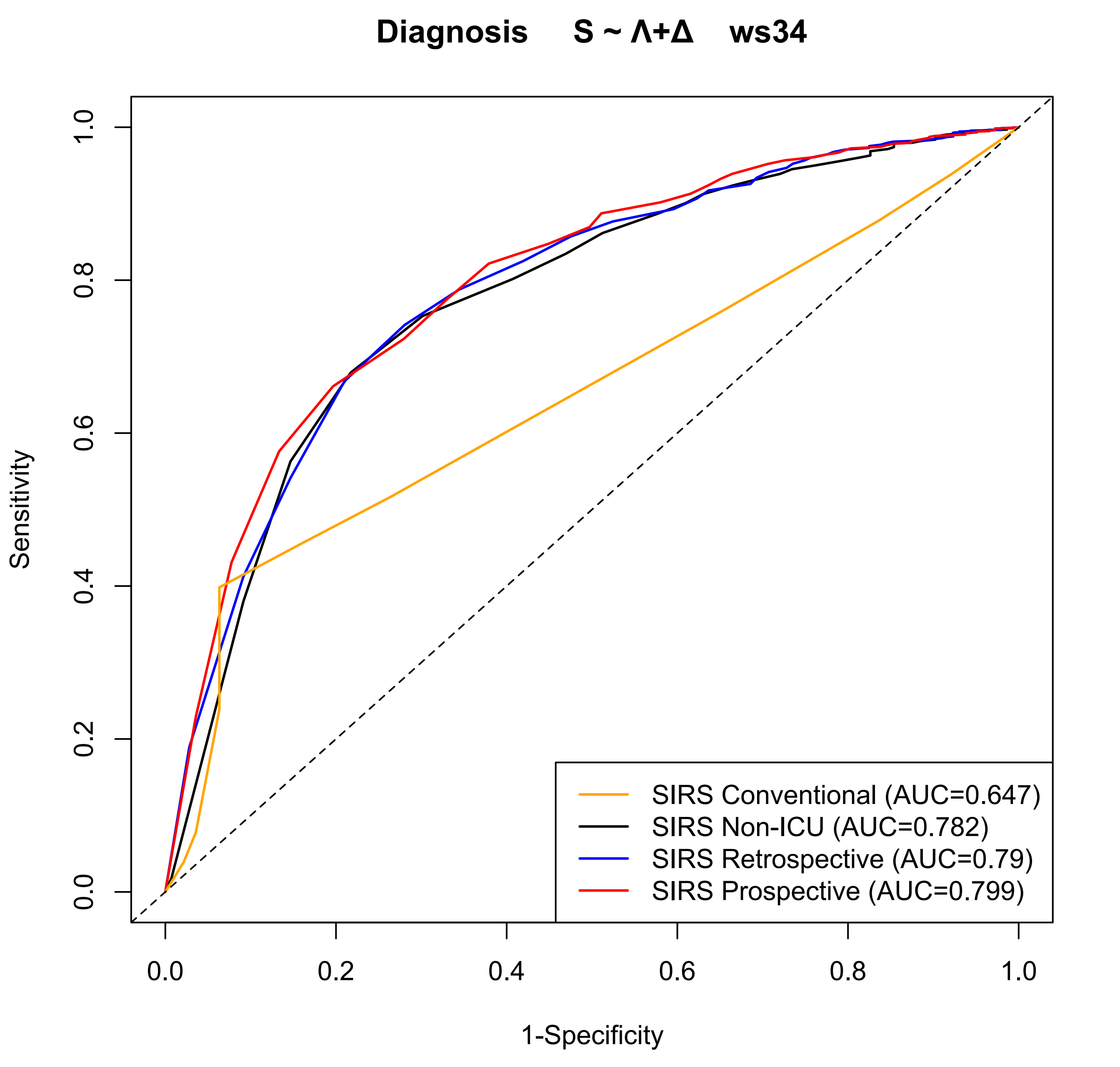
Diagnosis S ~ Λ+Δ ws34
Sensitivity
1-Specificity
0.0
0.2
0.4
0.6
0.8
1.0
0.0
0.2
0.4
0.6
0.8
1.0
SIRS Conventional (AUC=0.647)
SIRS Non-ICU (AUC=0.782)
SIRS Retrospective (AUC=0.79)
SIRS Prospective (AUC=0.799)

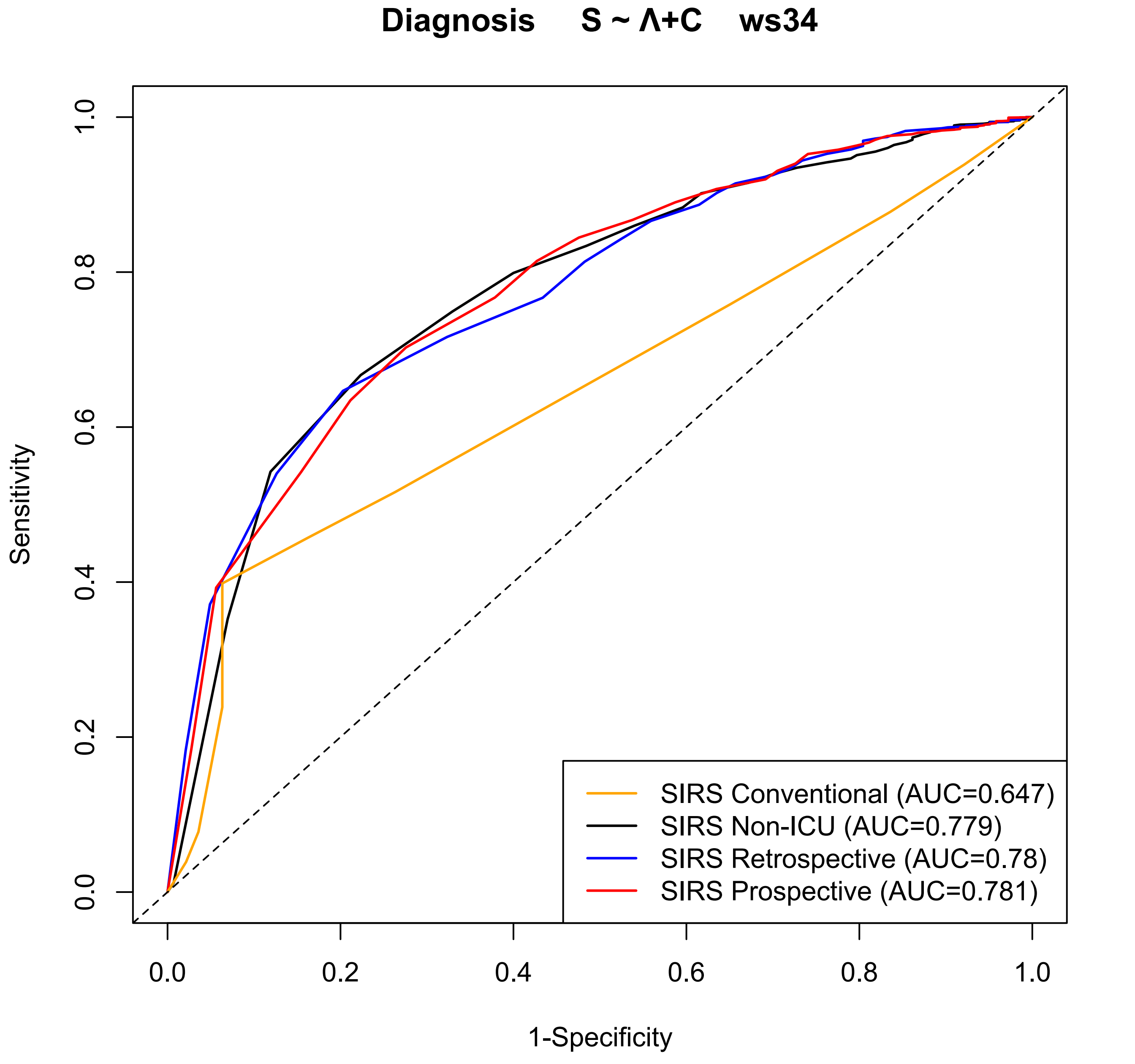

Diagnosis S ~ Λ+C ws34
Sensitivity
1-Specificity
0.0
0.2
0.4
0.6
0.8
1.0
SIRS Conventional (AUC=0.647)
SIRS Non-ICU (AUC=0.779)
SIRS Retrospective (AUC=0.78)
SIRS Prospective (AUC=0.781)

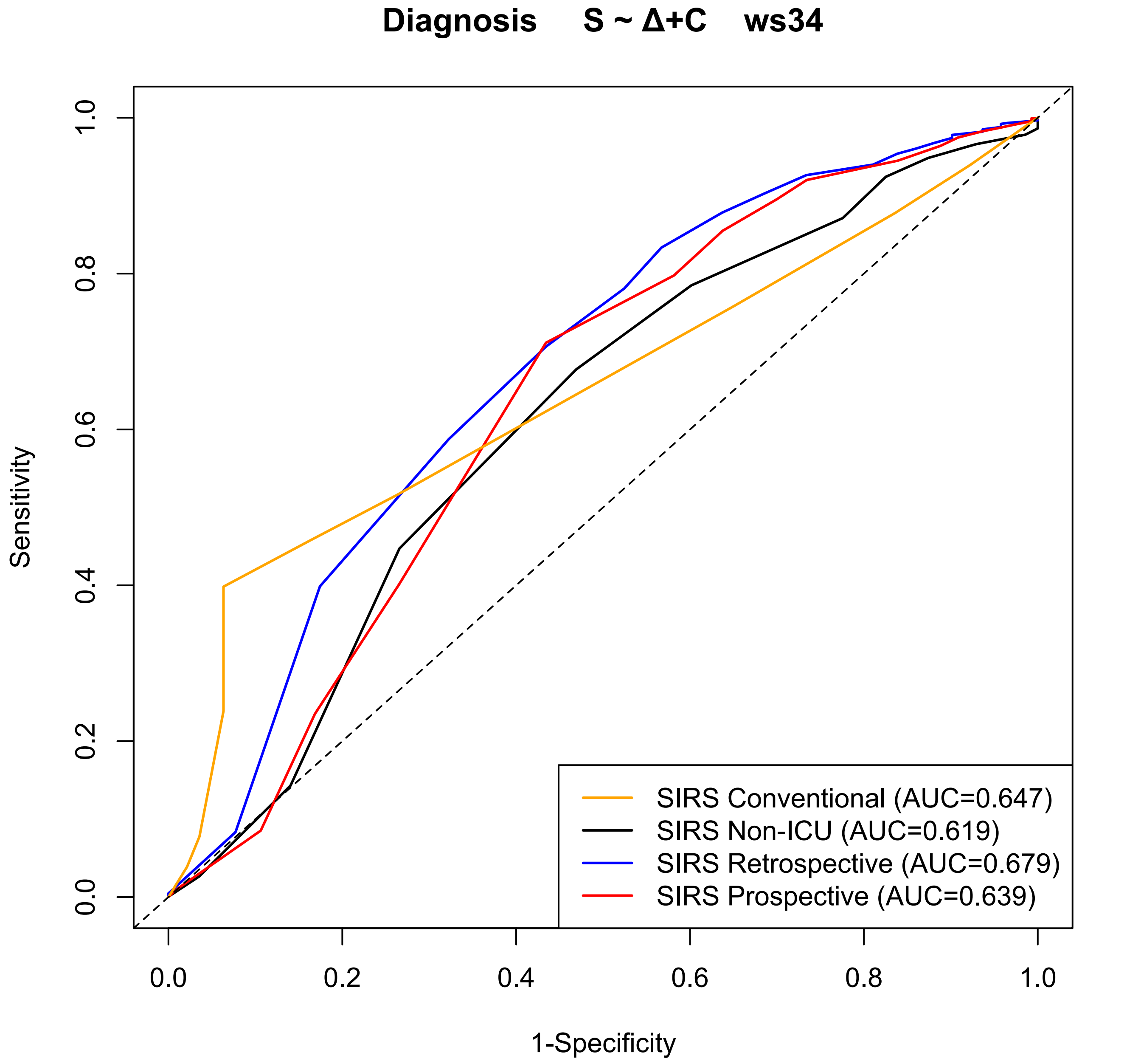

Diagnosis S ~ Δ+C ws34
Sensitivity
1-Specificity
0.0
0.2
0.4
0.6
0.8
1.0
0.0
0.2
0.4
0.6
0.8
1.0
SIRS Conventional (AUC=0.647)
SIRS Non-ICU (AUC=0.619)
SIRS Retrospective (AUC=0.679)
SIRS Prospective (AUC=0.639)

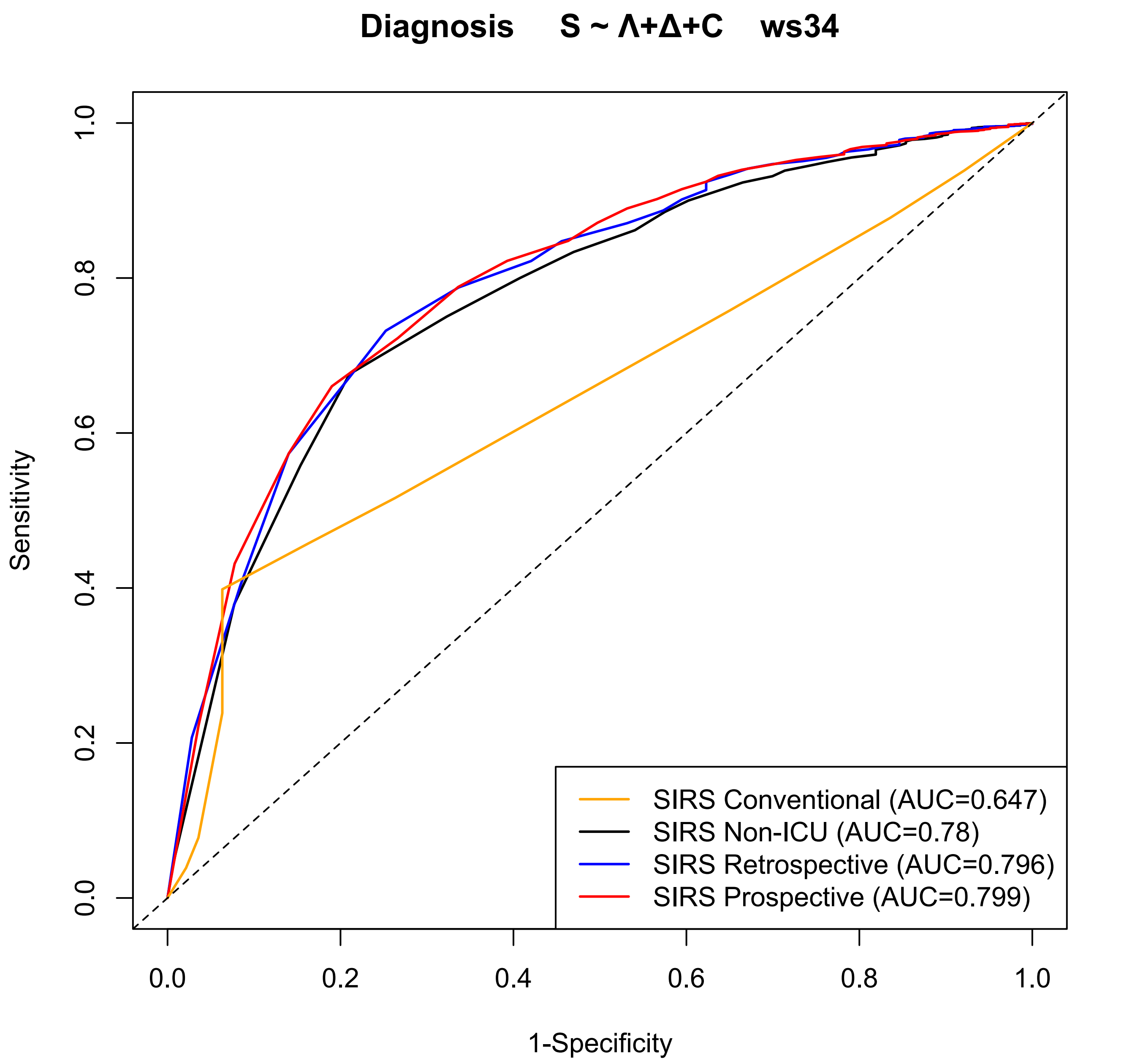

Diagnosis S ~ Λ+Δ+C ws34
Sensitivity
1-Specificity
0.0
0.2
0.4
0.6
0.8
1.0
0.0
0.2
0.4
0.6
0.8
1.0
SIRS Conventional (AUC=0.647)
SIRS Non-ICU (AUC=0.78)
SIRS Retrospective (AUC=0.796)
SIRS Prospective (AUC=0.799)

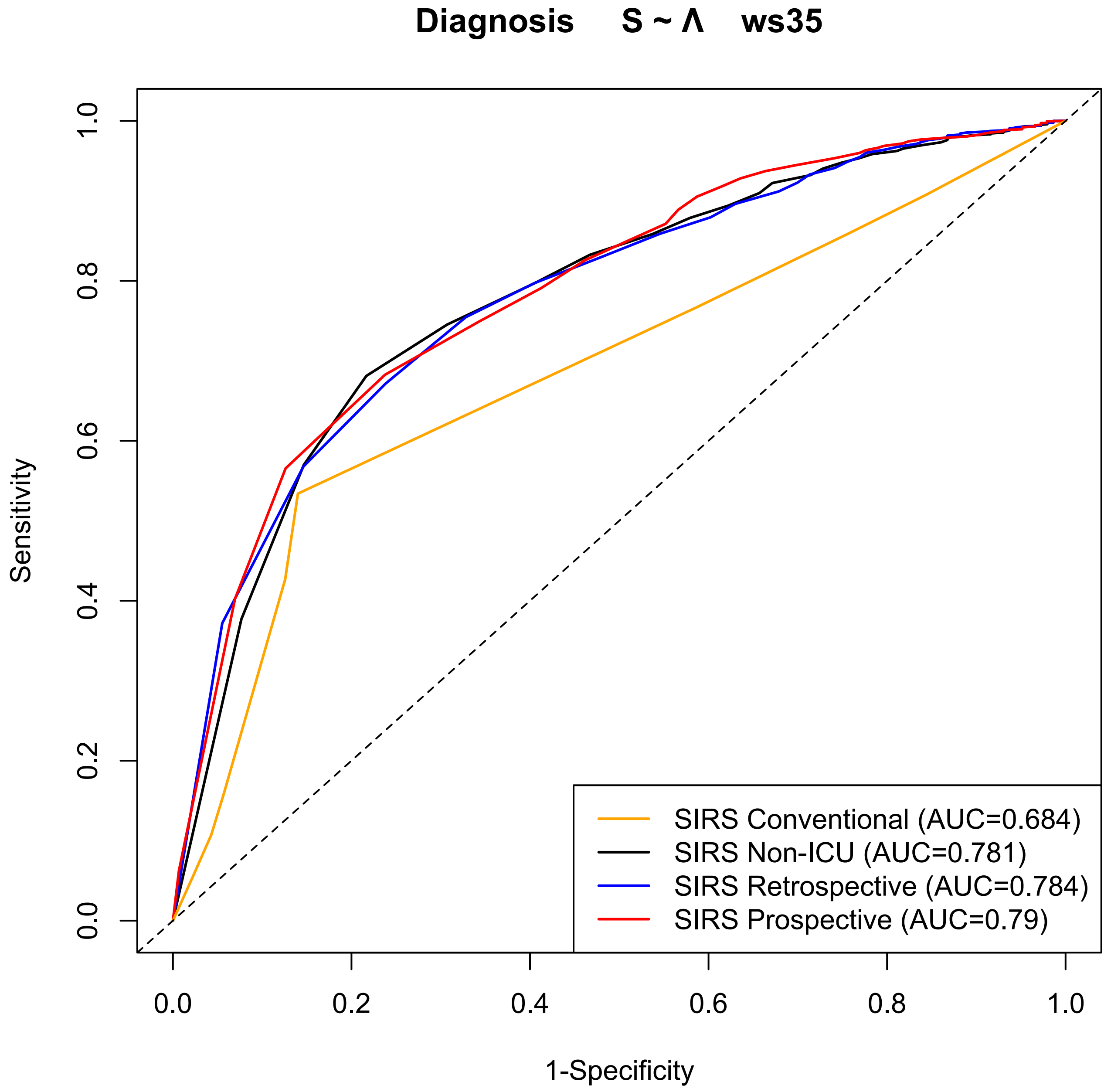

Diagnosis S ~ Λ ws35
Sensitivity
1-Specificity
0.0
0.2
0.4
0.6
0.8
1.0
SIRS Conventional (AUC=0.684)
SIRS Non-ICU (AUC=0.781)
SIRS Retrospective (AUC=0.784)
SIRS Prospective (AUC=0.79)

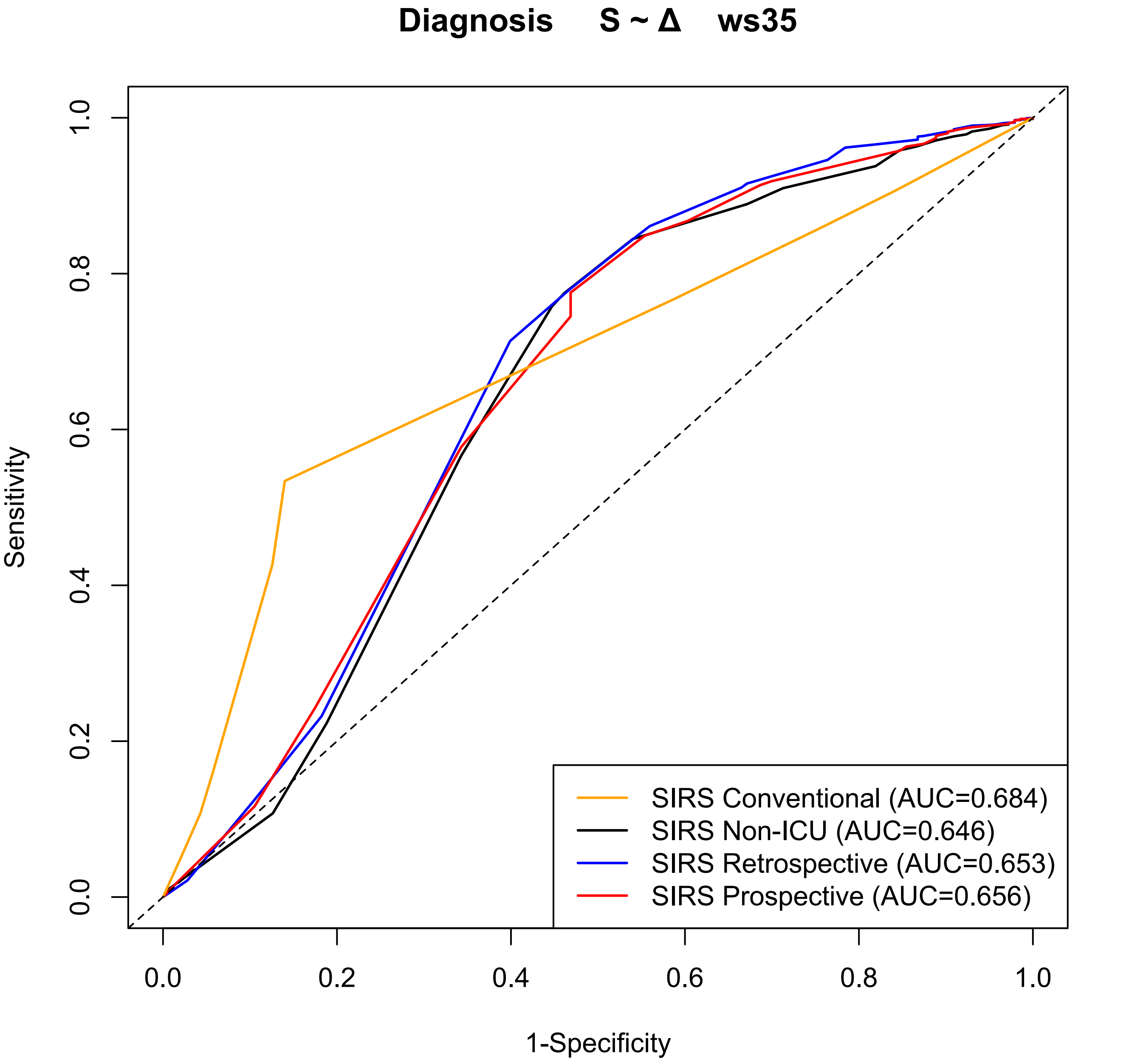

Diagnosis S ~ Δ ws35
Sensitivity
1-Specificity
0.0
0.2
0.4
0.6
0.8
1.0
SIRS Conventional (AUC=0.684)
SIRS Non-ICU (AUC=0.646)
SIRS Retrospective (AUC=0.653)
SIRS Prospective (AUC=0.656)

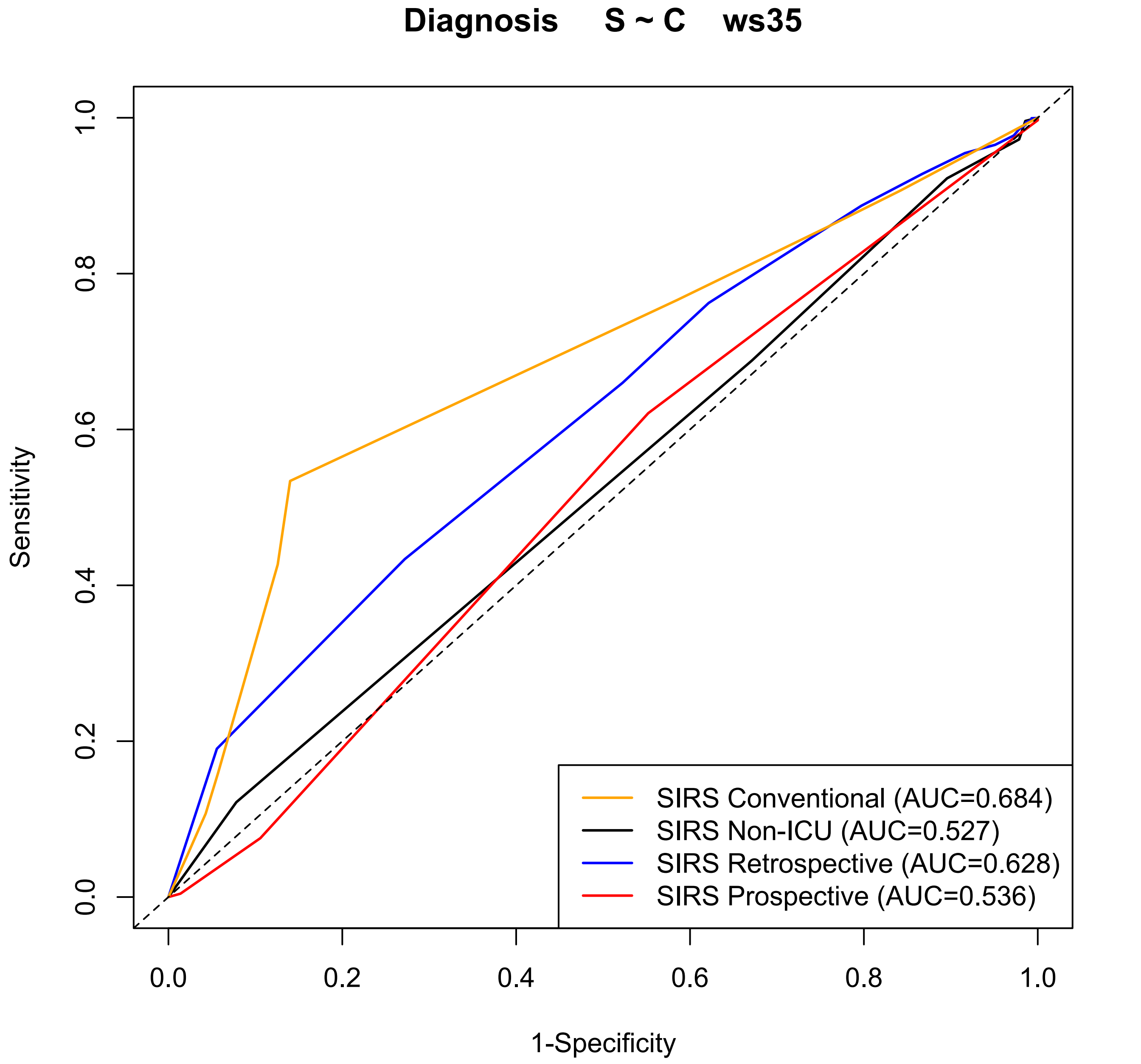

Diagnosis S ~ C ws35
Sensitivity
1-Specificity
0.0
0.2
0.4
0.6
0.8
1.0
SIRS Conventional (AUC=0.684)
SIRS Non-ICU (AUC=0.527)
SIRS Retrospective (AUC=0.628)
SIRS Prospective (AUC=0.536)

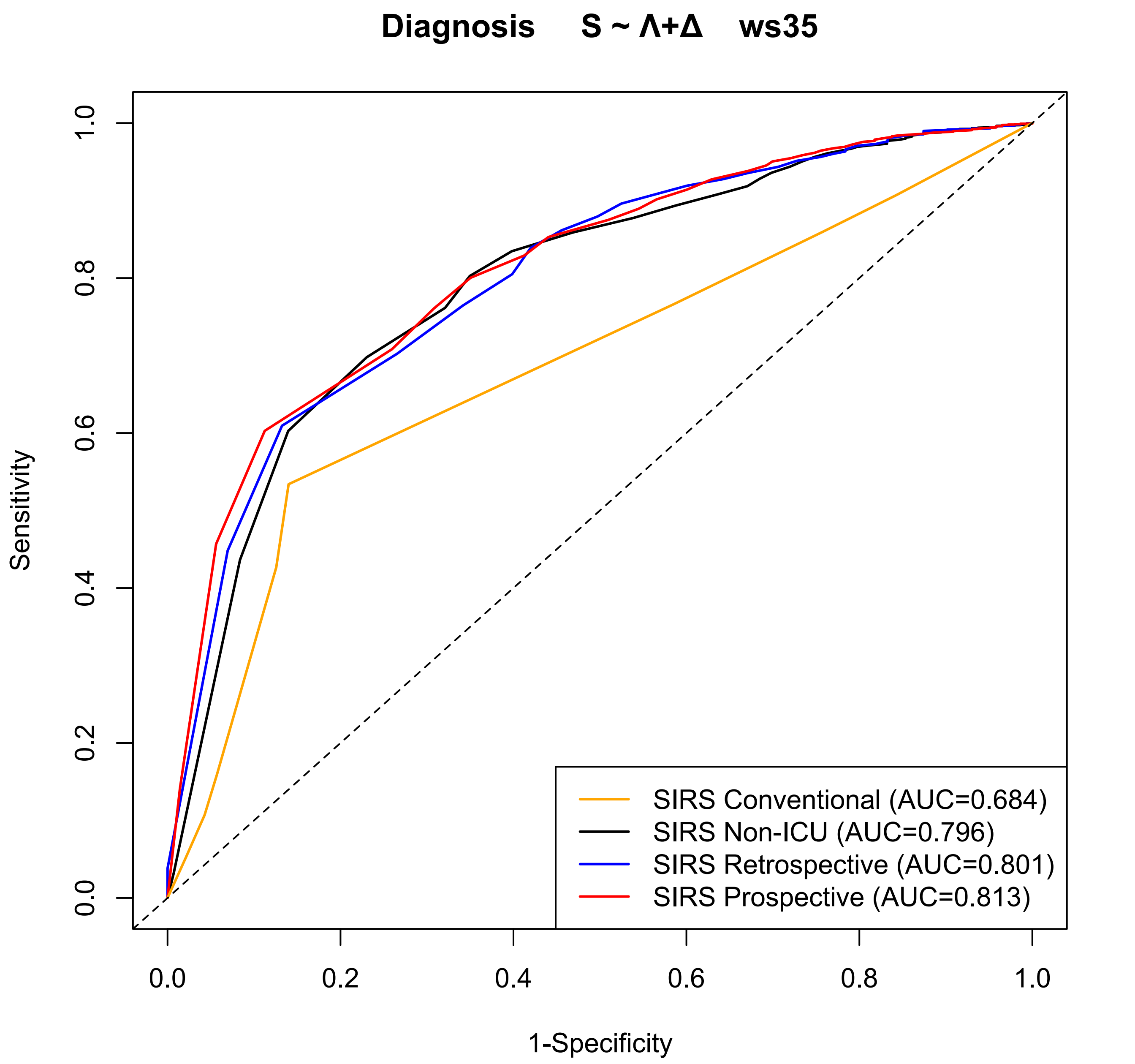
Diagnosis S ~ Λ+Δ ws35
Sensitivity
1-Specificity
0.0
0.2
0.4
0.6
0.8
1.0
SIRS Conventional (AUC=0.684)
SIRS Non-ICU (AUC=0.796)
SIRS Retrospective (AUC=0.801)
SIRS Prospective (AUC=0.813)

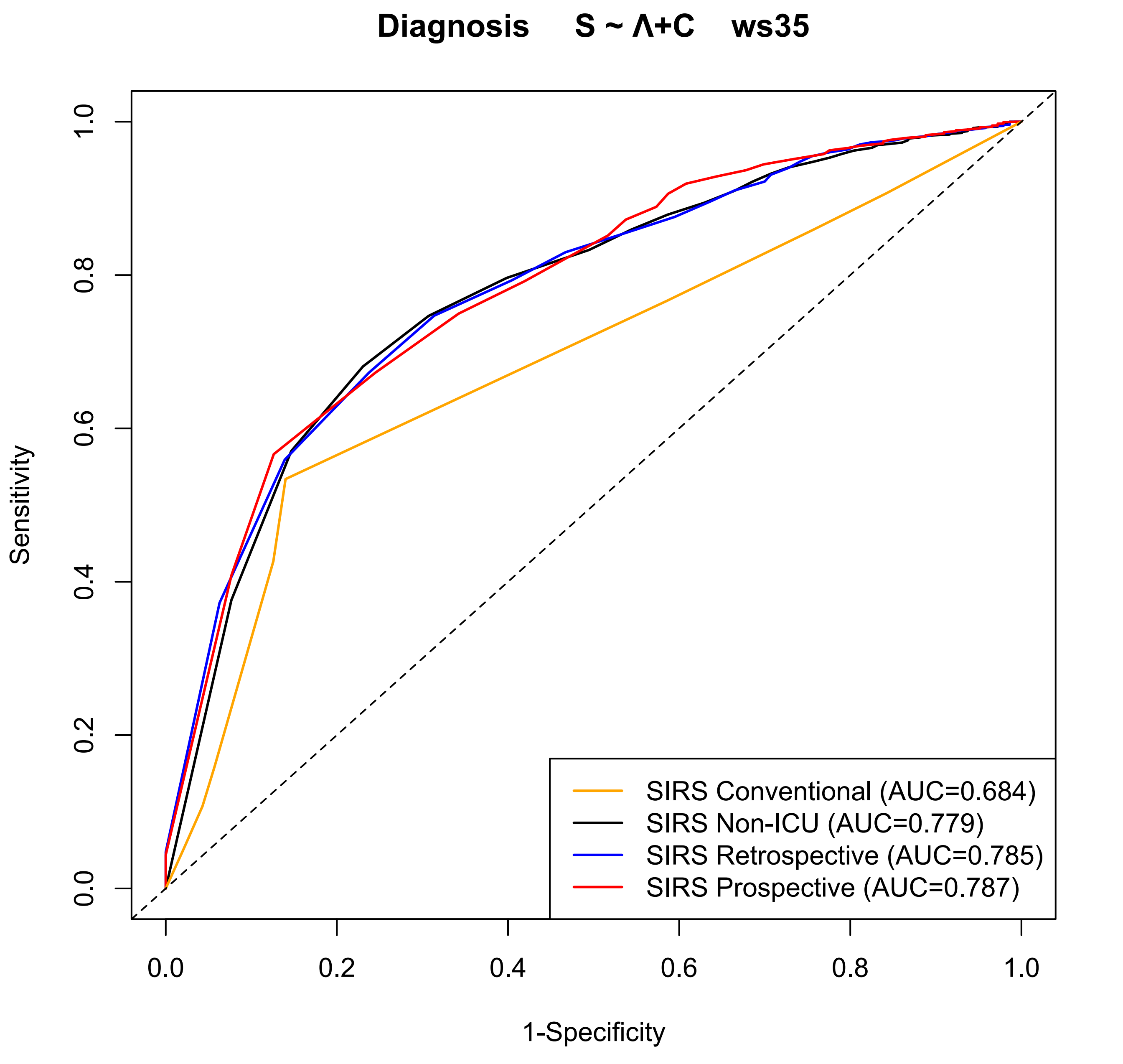
Diagnosis S ~ Λ+C ws35
Sensitivity
1-Specificity
0.0
0.2
0.4
0.6
0.8
1.0
SIRS Conventional (AUC=0.684)
SIRS Non-ICU (AUC=0.779)
SIRS Retrospective (AUC=0.785)
SIRS Prospective (AUC=0.787)

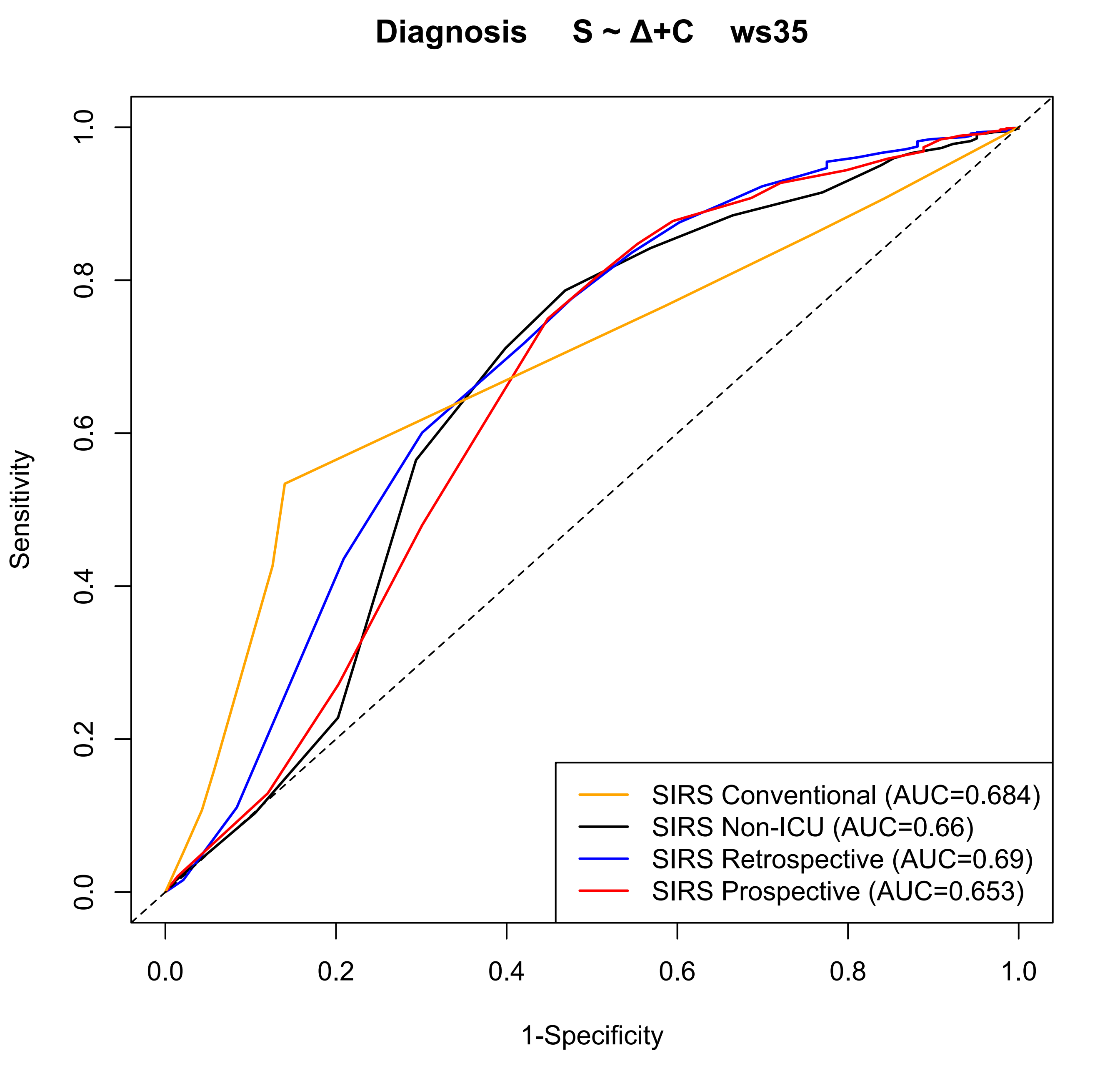
Diagnosis S ~ Δ+C ws35
Sensitivity
1-Specificity
0.0
0.2
0.4
0.6
0.8
1.0
SIRS Conventional (AUC=0.684)
SIRS Non-ICU (AUC=0.66)
SIRS Retrospective (AUC=0.69)
SIRS Prospective (AUC=0.653)

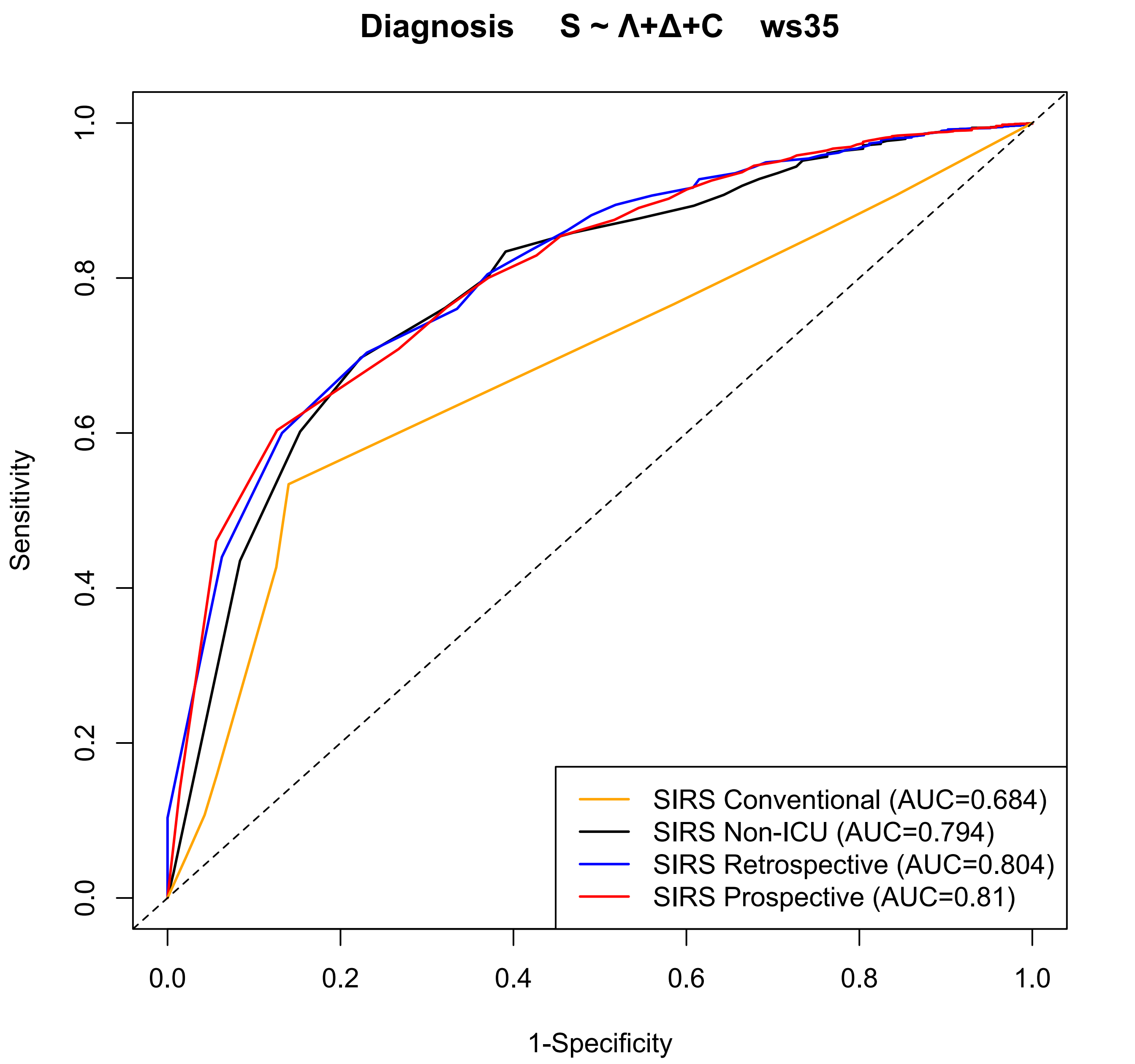
Diagnosis S ~ Λ+Δ+C ws35
Sensitivity
1-Specificity
0.0
0.2
0.4
0.6
0.8
1.0
SIRS Conventional (AUC=0.684)
SIRS Non-ICU (AUC=0.794)
SIRS Retrospective (AUC=0.804)
SIRS Prospective (AUC=0.81)

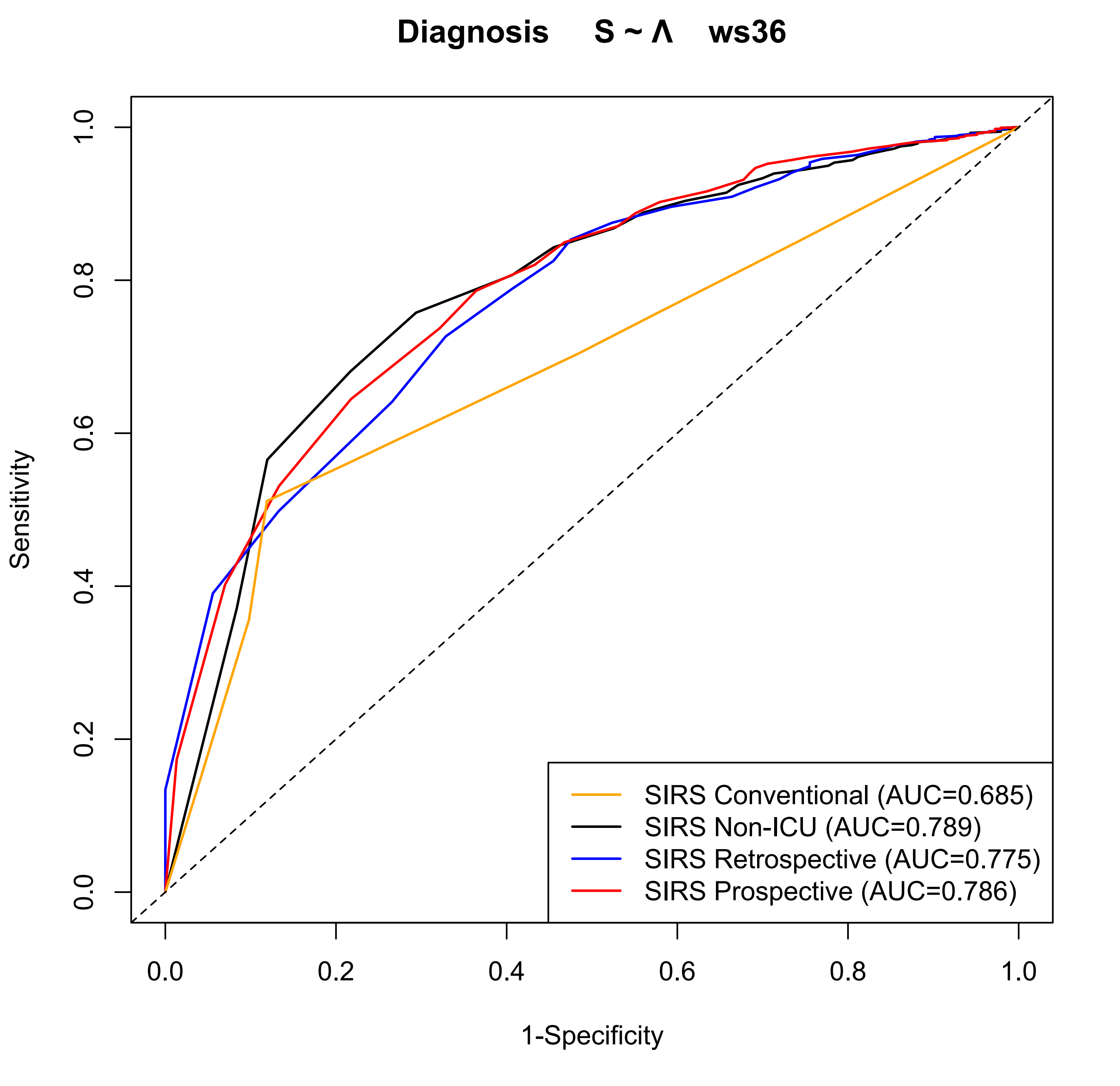

Diagnosis S ~ Λ ws36
Sensitivity
1-Specificity
0.0
0.2
0.4
0.6
0.8
1.0
SIRS Conventional (AUC=0.685)
SIRS Non-ICU (AUC=0.789)
SIRS Retrospective (AUC=0.775)
SIRS Prospective (AUC=0.786)

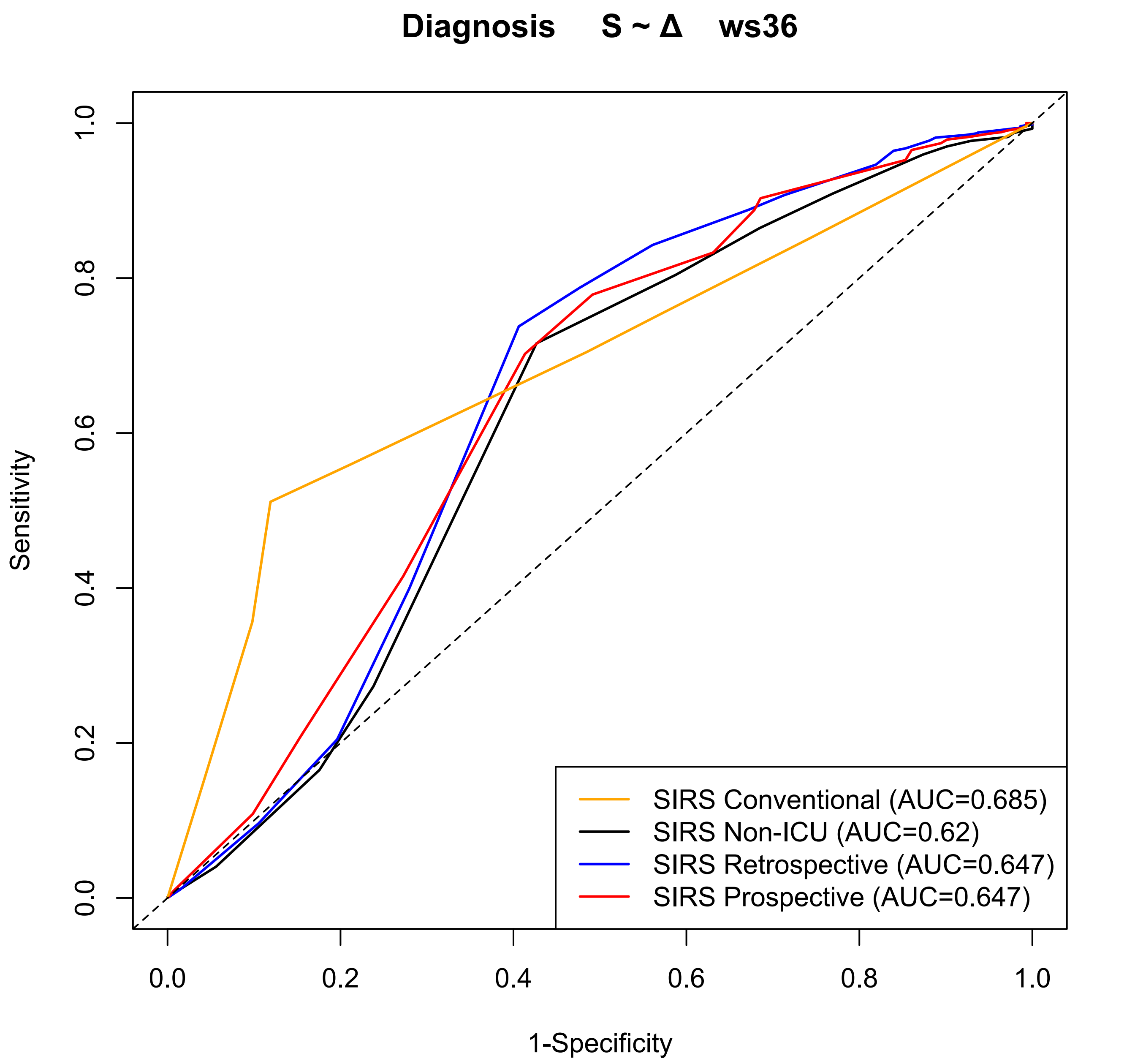
Diagnosis S ~ Δ ws36
Sensitivity
1-Specificity
0.0
0.2
0.4
0.6
0.8
1.0
SIRS Conventional (AUC=0.685)
SIRS Non-ICU (AUC=0.62)
SIRS Retrospective (AUC=0.647)
SIRS Prospective (AUC=0.647)

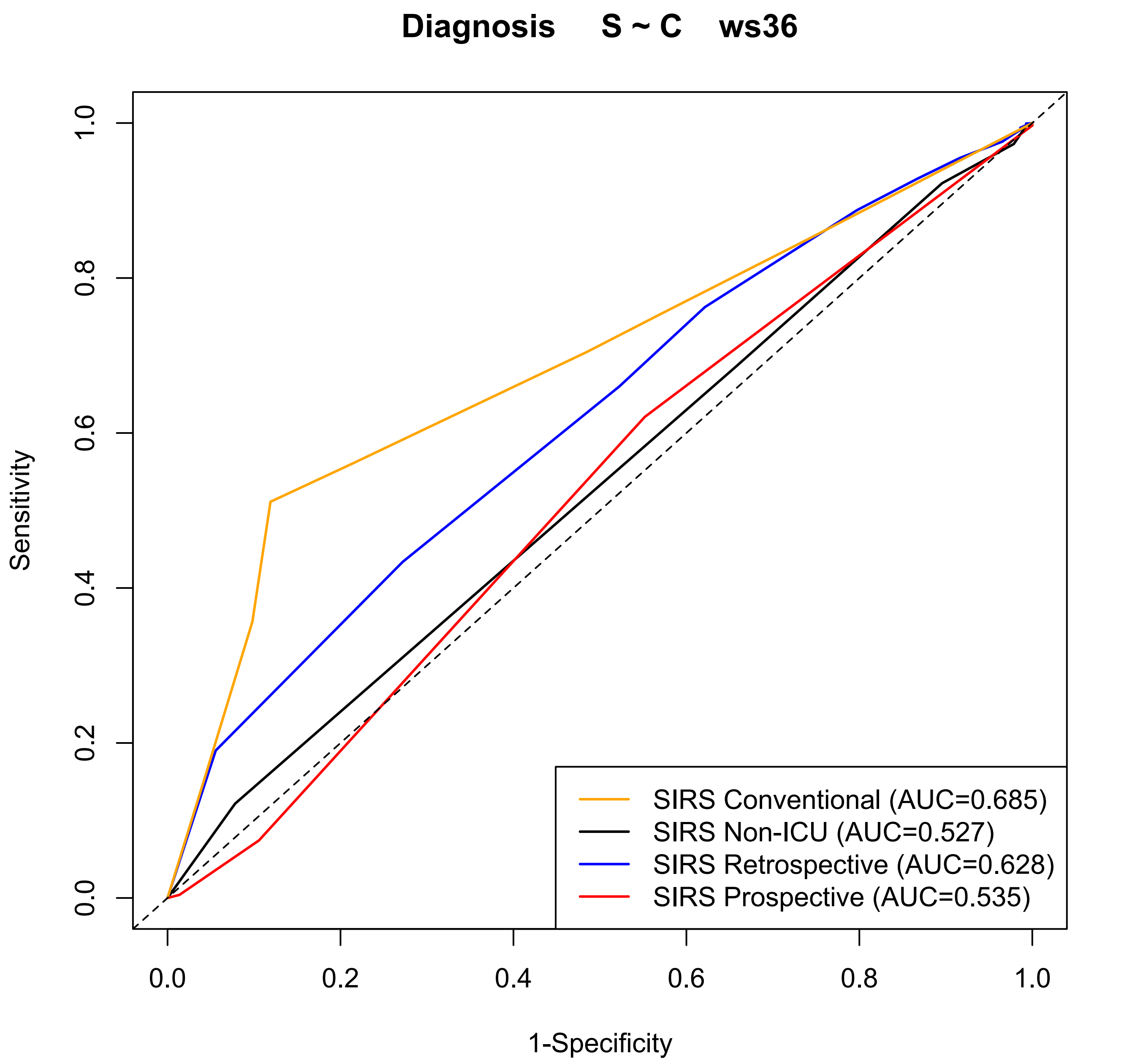
Diagnosis S ~ C ws36
Sensitivity
1-Specificity
0.0
0.2
0.4
0.6
0.8
1.0
0.0
0.2
0.4
0.6
0.8
1.0
SIRS Conventional (AUC=0.685)
SIRS Non-ICU (AUC=0.527)
SIRS Retrospective (AUC=0.628)
SIRS Prospective (AUC=0.535)

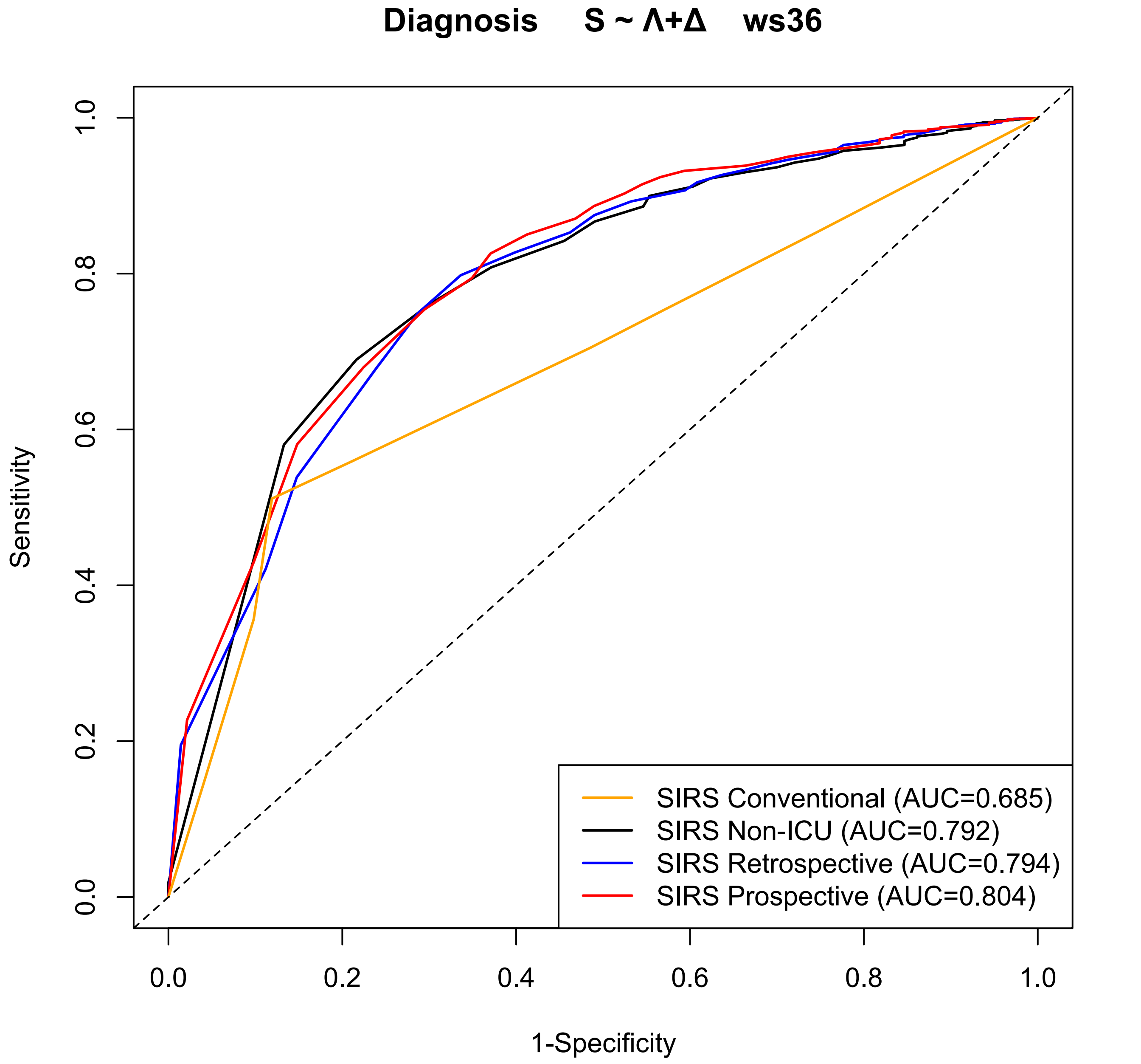

Diagnosis S ~ Λ+Δ ws36
Sensitivity
1-Specificity
0.0
0.2
0.4
0.6
0.8
1.0
SIRS Conventional (AUC=0.685)
SIRS Non-ICU (AUC=0.792)
SIRS Retrospective (AUC=0.794)
SIRS Prospective (AUC=0.804)

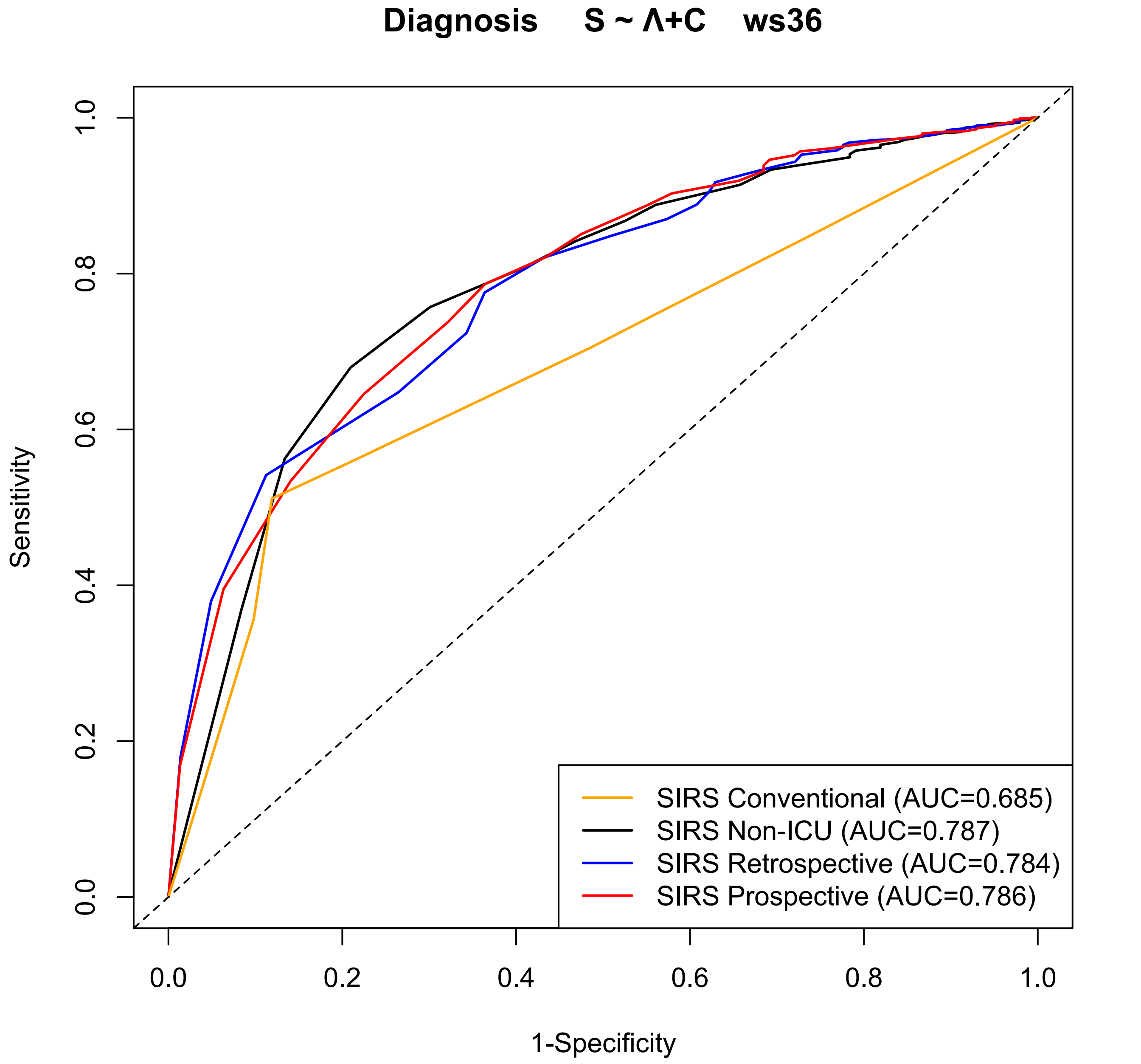
Diagnosis S ~ Λ+C ws36
Sensitivity
1-Specificity
0.0
0.2
0.4
0.6
0.8
1.0
SIRS Conventional (AUC=0.685)
SIRS Non-ICU (AUC=0.787)
SIRS Retrospective (AUC=0.784)
SIRS Prospective (AUC=0.786)

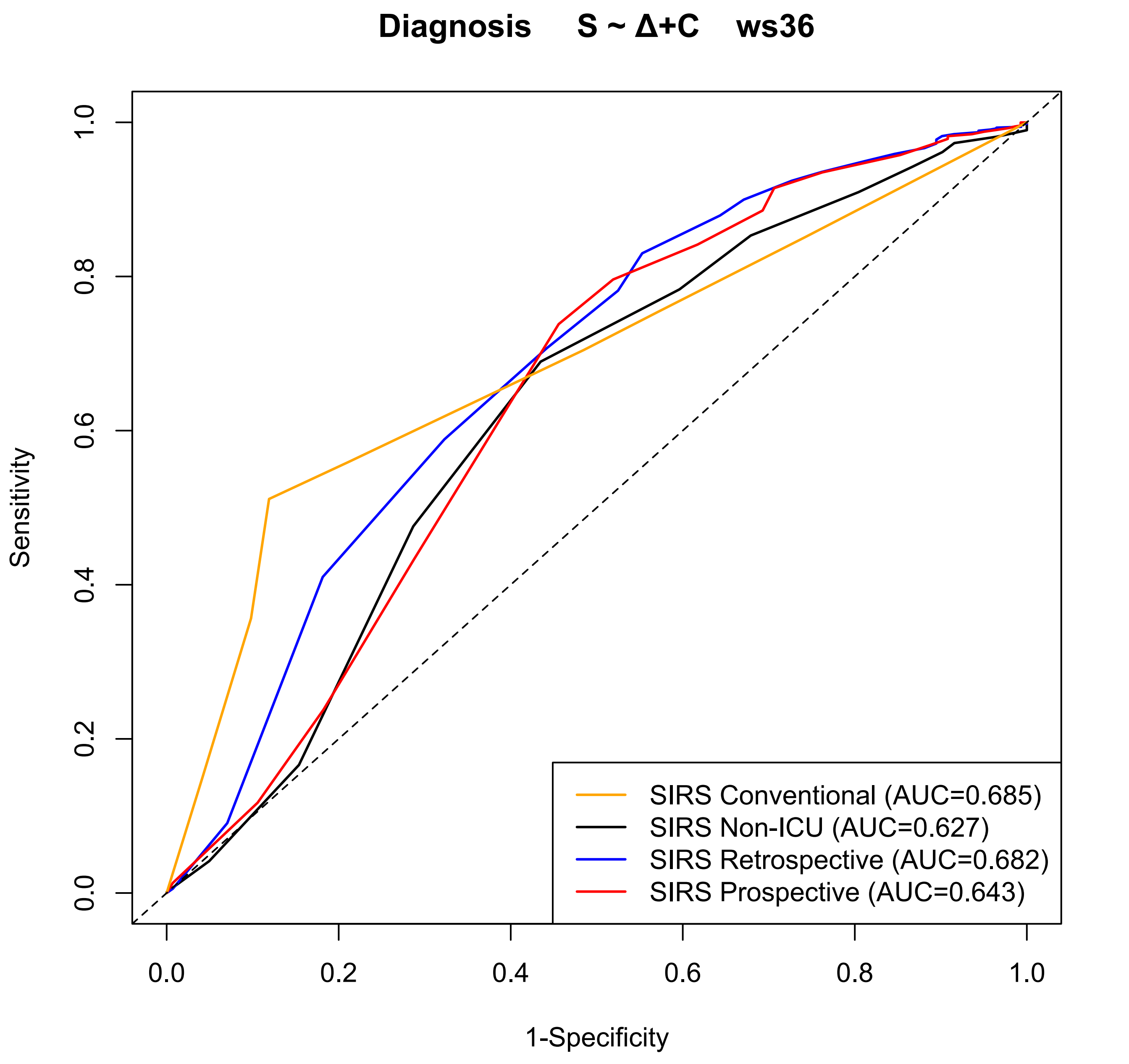

Diagnosis S ~ Δ+C ws36
Sensitivity
1-Specificity
0.0
0.2
0.4
0.6
0.8
1.0
SIRS Conventional (AUC=0.685)
SIRS Non-ICU (AUC=0.627)
SIRS Retrospective (AUC=0.682)
SIRS Prospective (AUC=0.643)

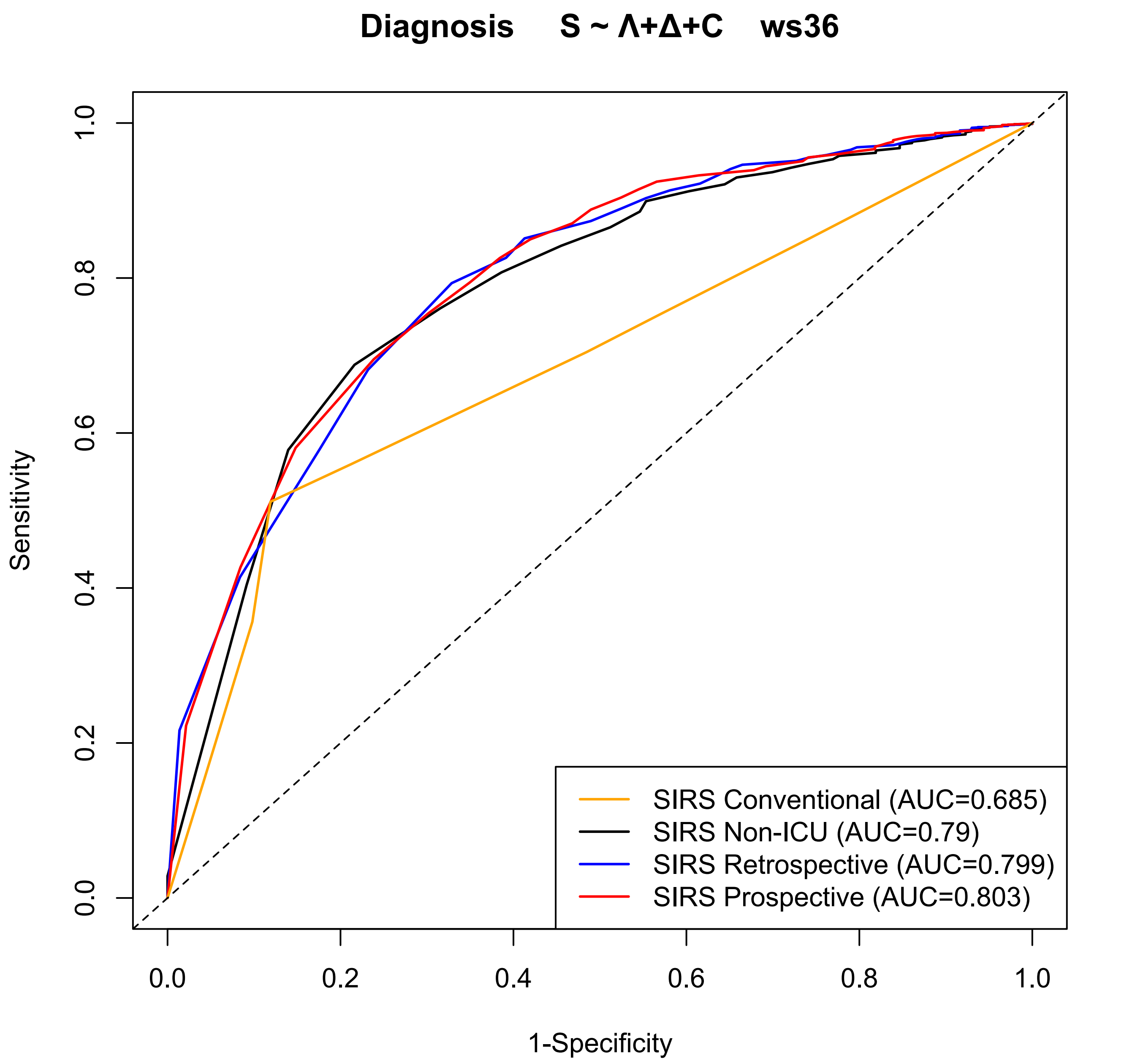

Diagnosis S ~ Λ+Δ+C ws36
Sensitivity
1-Specificity
0.0
0.2
0.4
0.6
0.8
1.0
SIRS Conventional (AUC=0.685)
SIRS Non-ICU (AUC=0.79)
SIRS Retrospective (AUC=0.799)
SIRS Prospective (AUC=0.803)

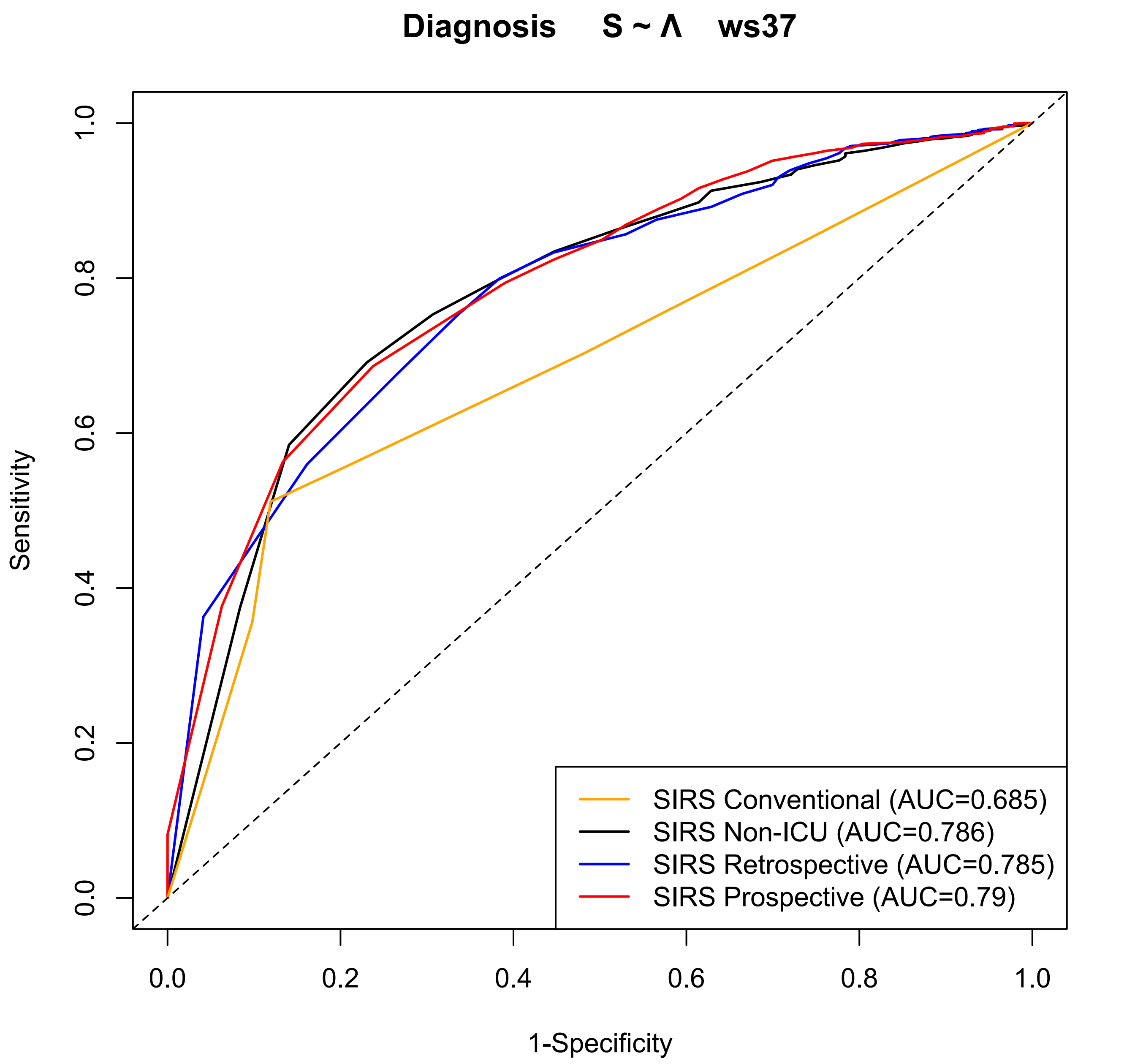
Diagnosis S ~ Λ ws37
Sensitivity
1-Specificity
0.0
0.2
0.4
0.6
0.8
1.0
0.0
0.2
0.4
0.6
0.8
1.0
SIRS Conventional (AUC=0.685)
SIRS Non-ICU (AUC=0.786)
SIRS Retrospective (AUC=0.785)
SIRS Prospective (AUC=0.79)

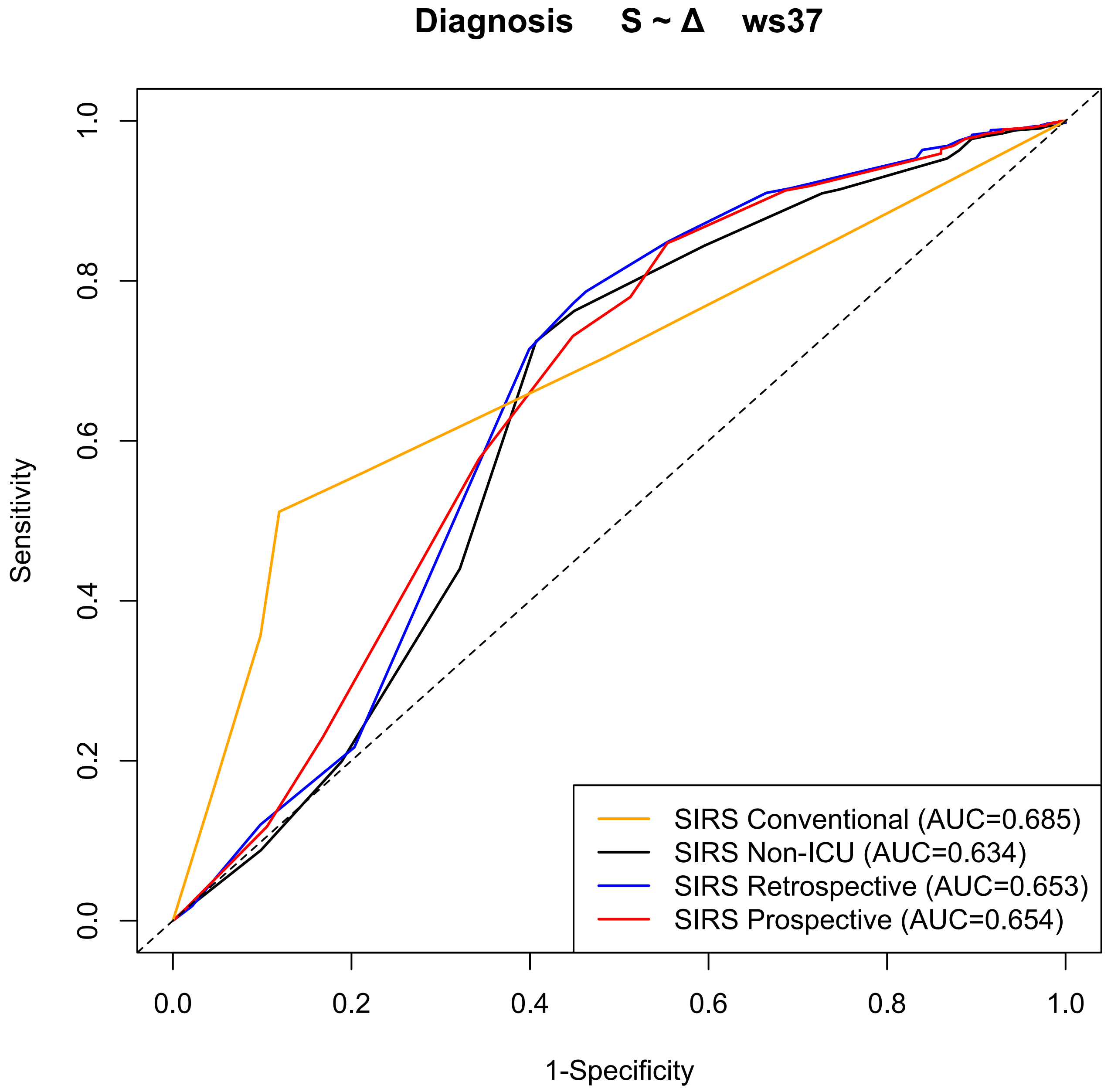

Diagnosis S ~ Δ ws37
Sensitivity
1-Specificity
0.0
0.2
0.4
0.6
0.8
1.0
0.0
0.2
0.4
0.6
0.8
1.0
SIRS Conventional (AUC=0.685)
SIRS Non-ICU (AUC=0.634)
SIRS Retrospective (AUC=0.653)
SIRS Prospective (AUC=0.654)

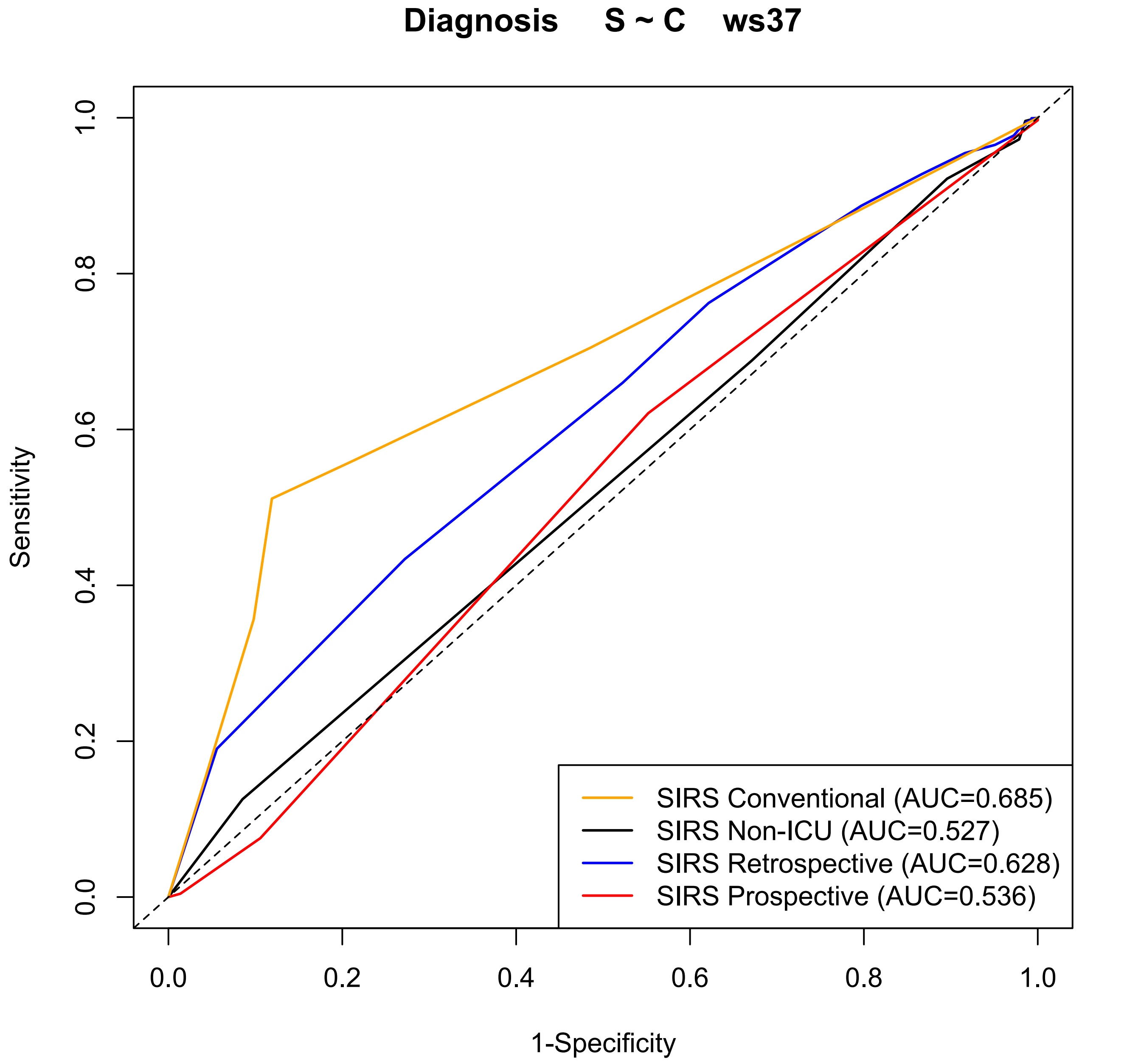
Diagnosis S ~ C ws37
Sensitivity
1-Specificity
0.0
0.2
0.4
0.6
0.8
1.0
SIRS Conventional (AUC=0.685)
SIRS Non-ICU (AUC=0.527)
SIRS Retrospective (AUC=0.628)
SIRS Prospective (AUC=0.536)

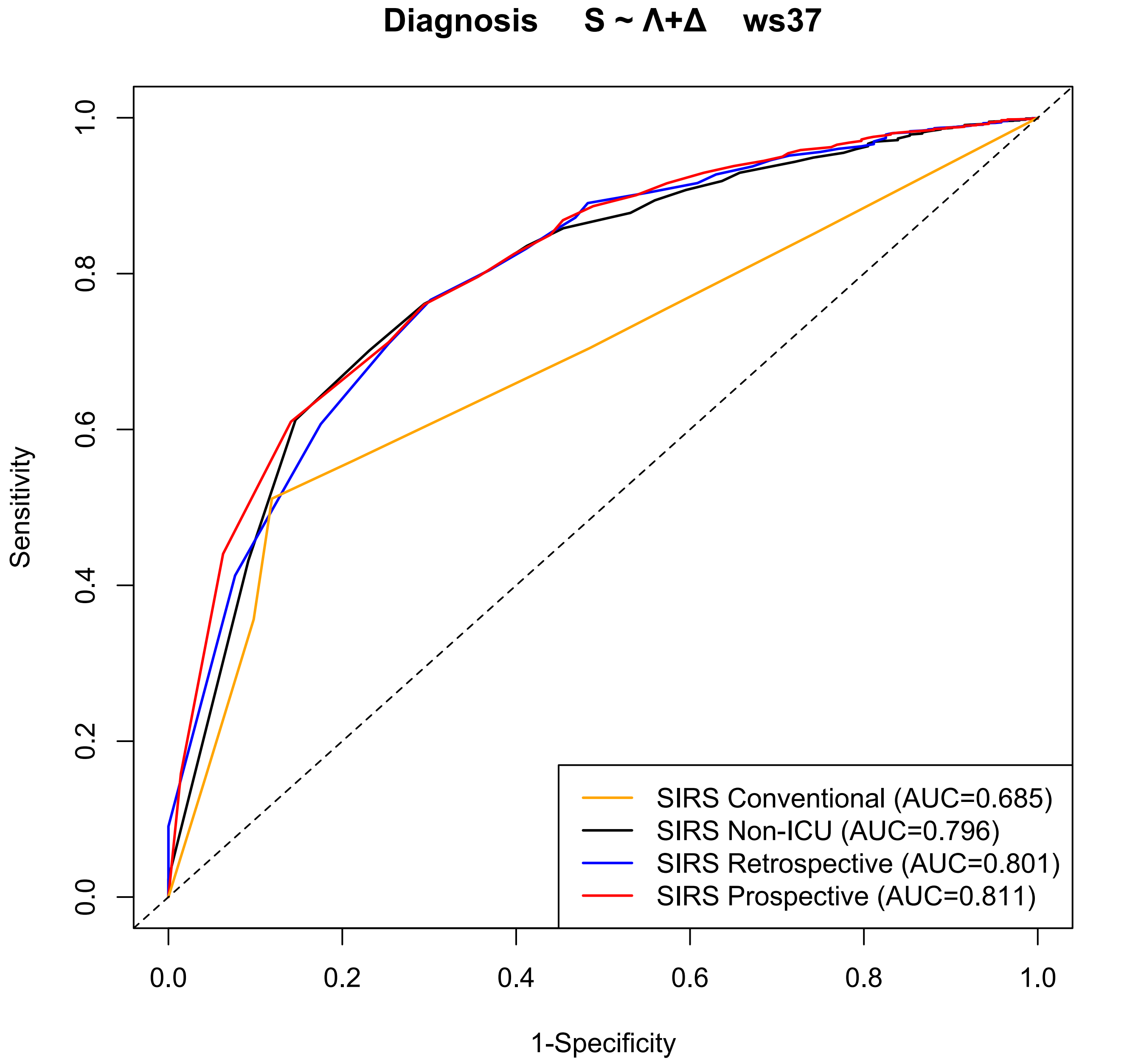
Diagnosis S ~ Λ+Δ ws37
Sensitivity
1-Specificity
0.0
0.2
0.4
0.6
0.8
1.0
SIRS Conventional (AUC=0.685)
SIRS Non-ICU (AUC=0.796)
SIRS Retrospective (AUC=0.801)
SIRS Prospective (AUC=0.811)

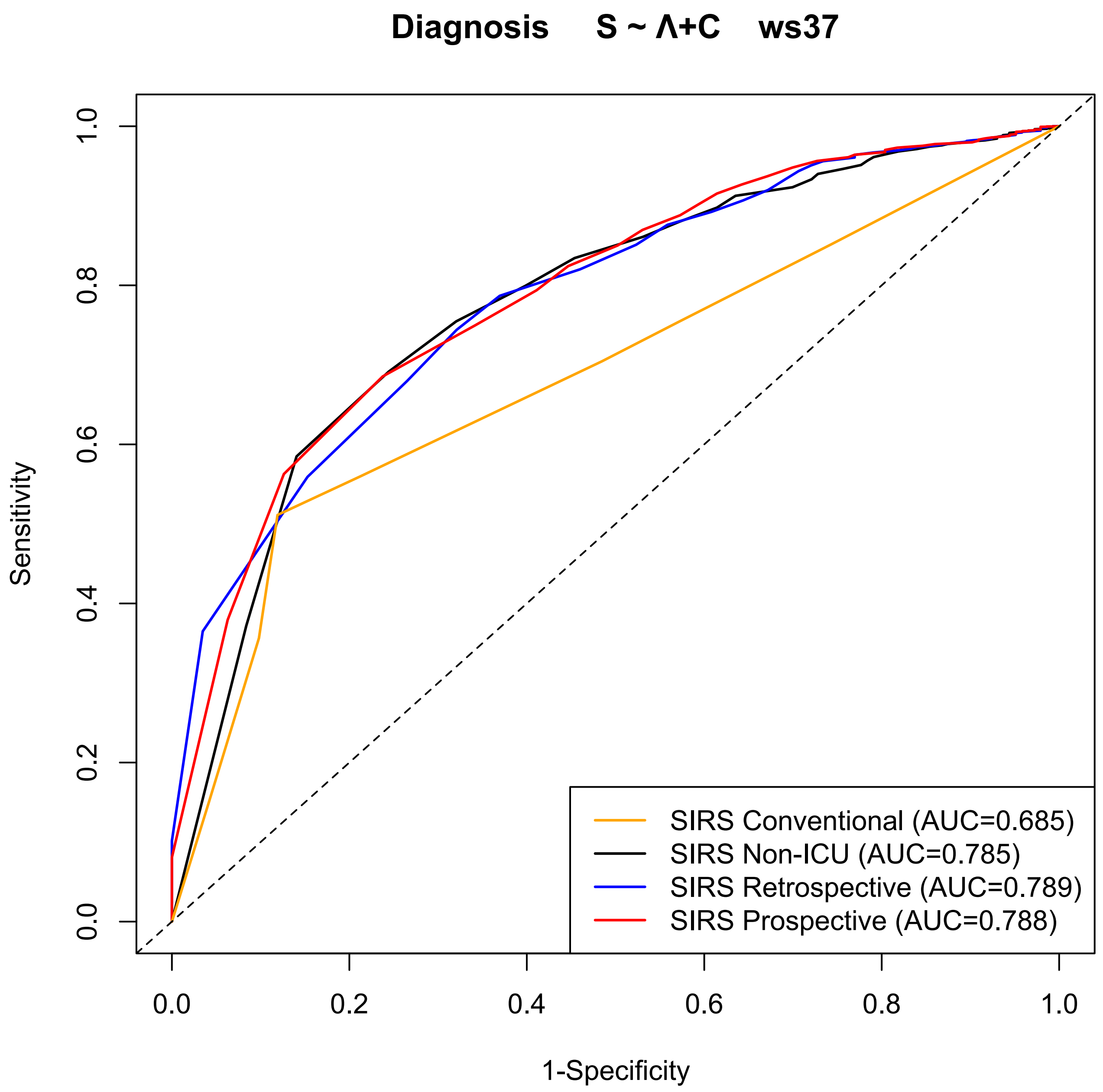
Diagnosis S ~ Λ+C ws37
Sensitivity
1-Specificity
0.0
0.2
0.4
0.6
0.8
1.0
0.0
0.2
0.4
0.6
0.8
1.0
SIRS Conventional (AUC=0.685)
SIRS Non-ICU (AUC=0.785)
SIRS Retrospective (AUC=0.789)
SIRS Prospective (AUC=0.788)

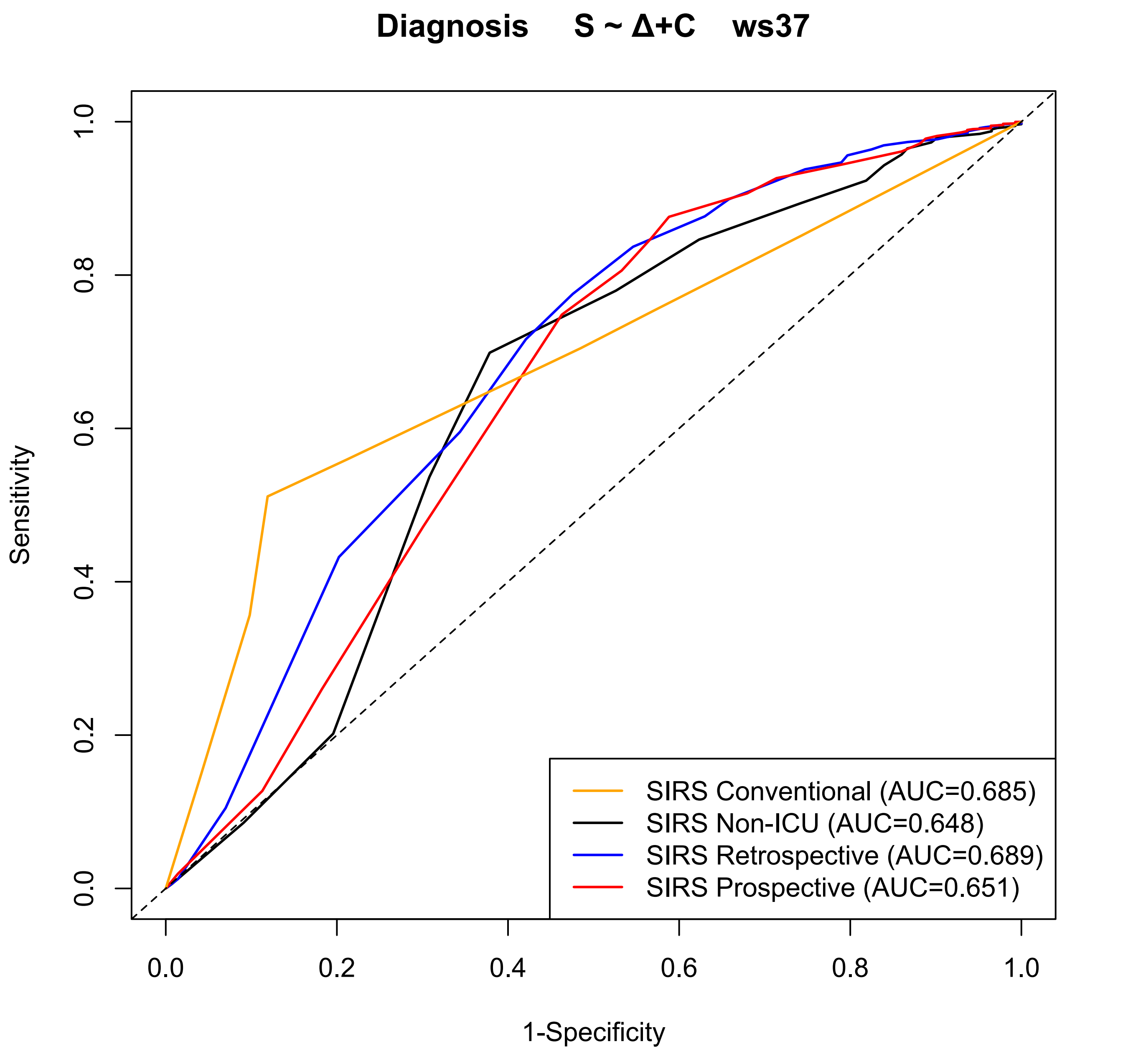
Diagnosis S ~ Δ+C ws37
Sensitivity
1-Specificity
0.0
0.2
0.4
0.6
0.8
1.0
SIRS Conventional (AUC=0.685)
SIRS Non-ICU (AUC=0.648)
SIRS Retrospective (AUC=0.689)
SIRS Prospective (AUC=0.651)

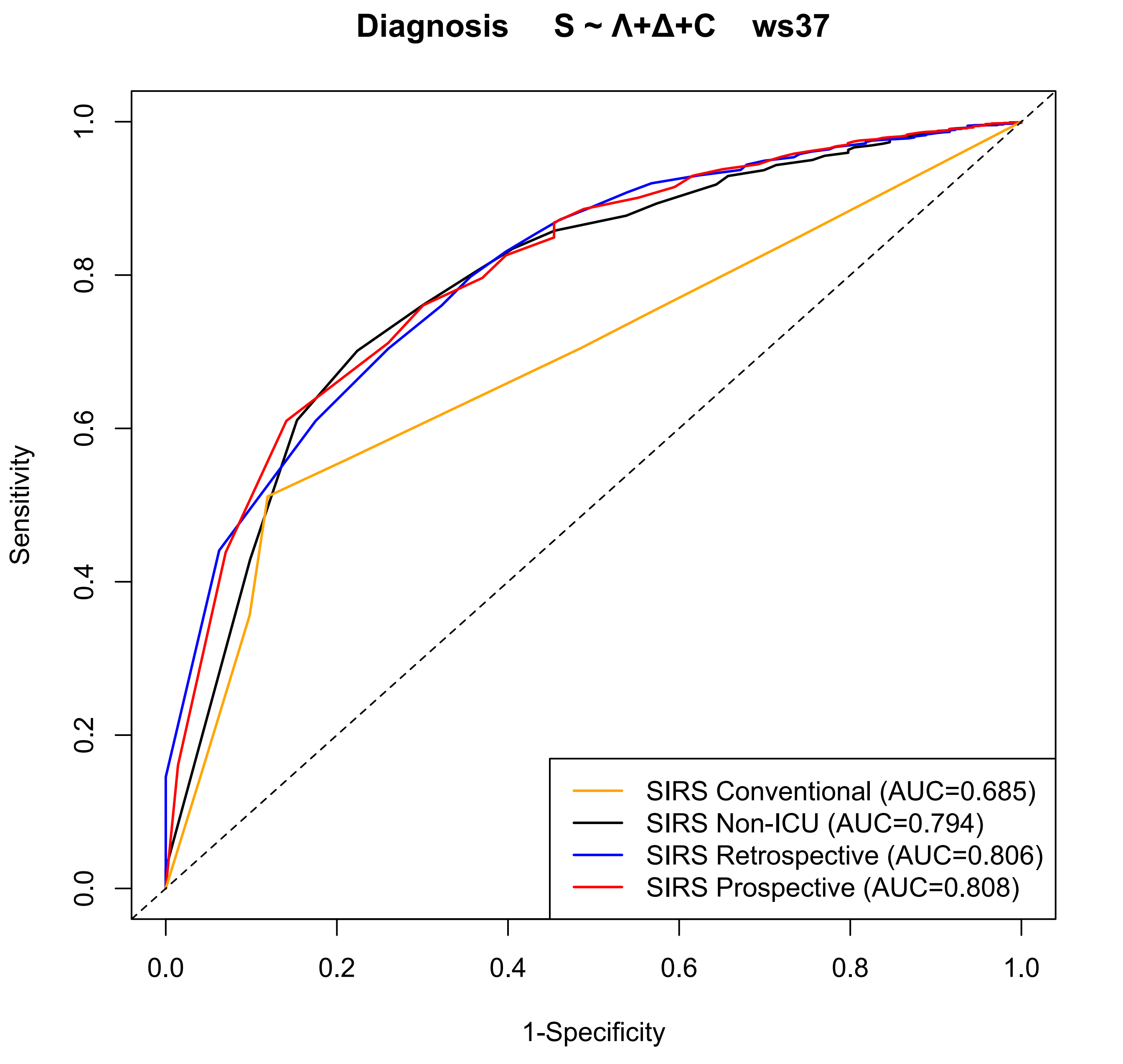

Diagnosis S ~ Λ+Δ+C ws37
Sensitivity
1-Specificity
0.0
0.2
0.4
0.6
0.8
1.0
SIRS Conventional (AUC=0.685)
SIRS Non-ICU (AUC=0.794)
SIRS Retrospective (AUC=0.806)
SIRS Prospective (AUC=0.808)

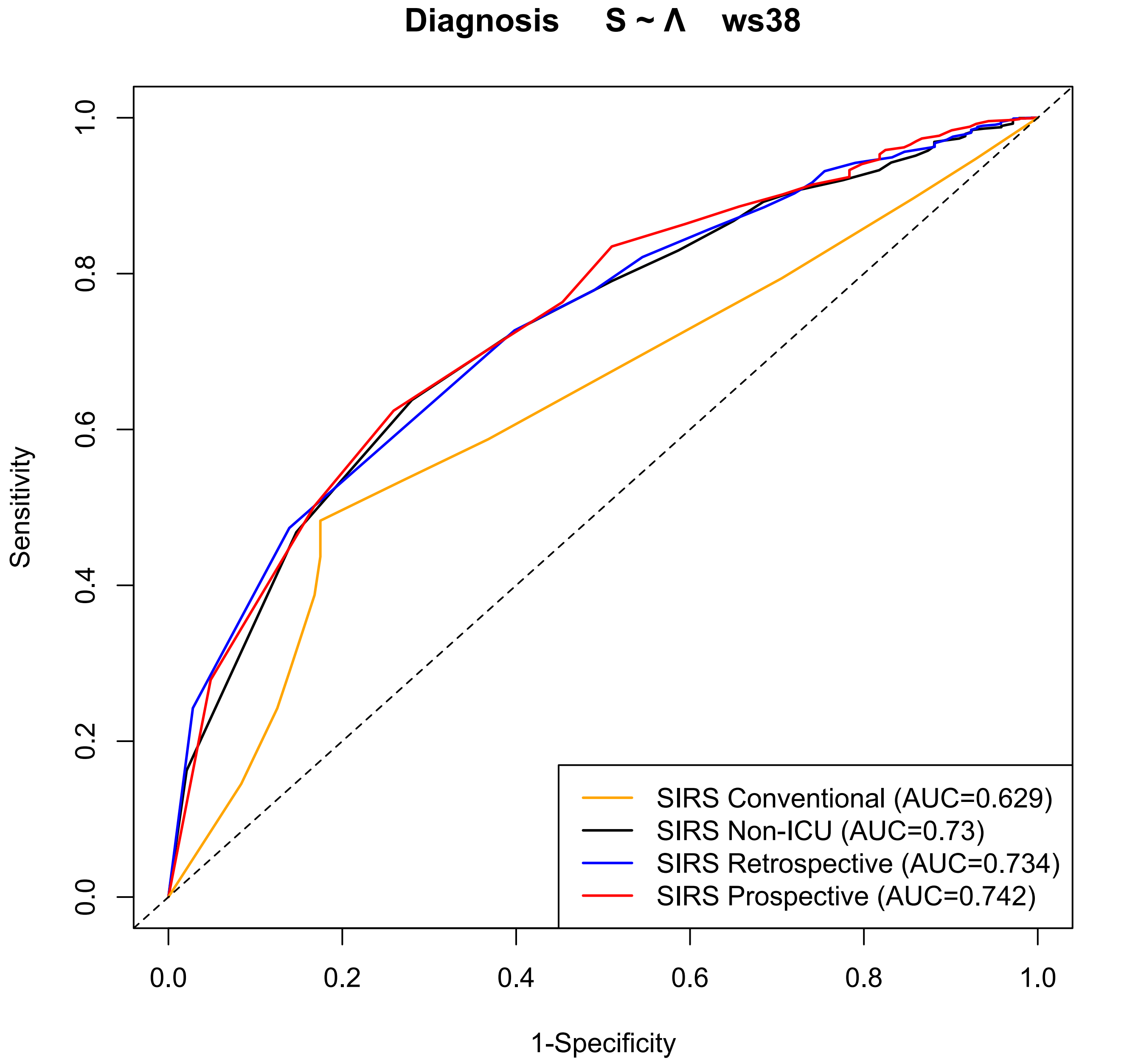
Diagnosis S ~ Λ ws38
Sensitivity
1-Specificity
0.0
0.2
0.4
0.6
0.8
1.0
SIRS Conventional (AUC=0.629)
SIRS Non-ICU (AUC=0.73)
SIRS Retrospective (AUC=0.734)
SIRS Prospective (AUC=0.742)

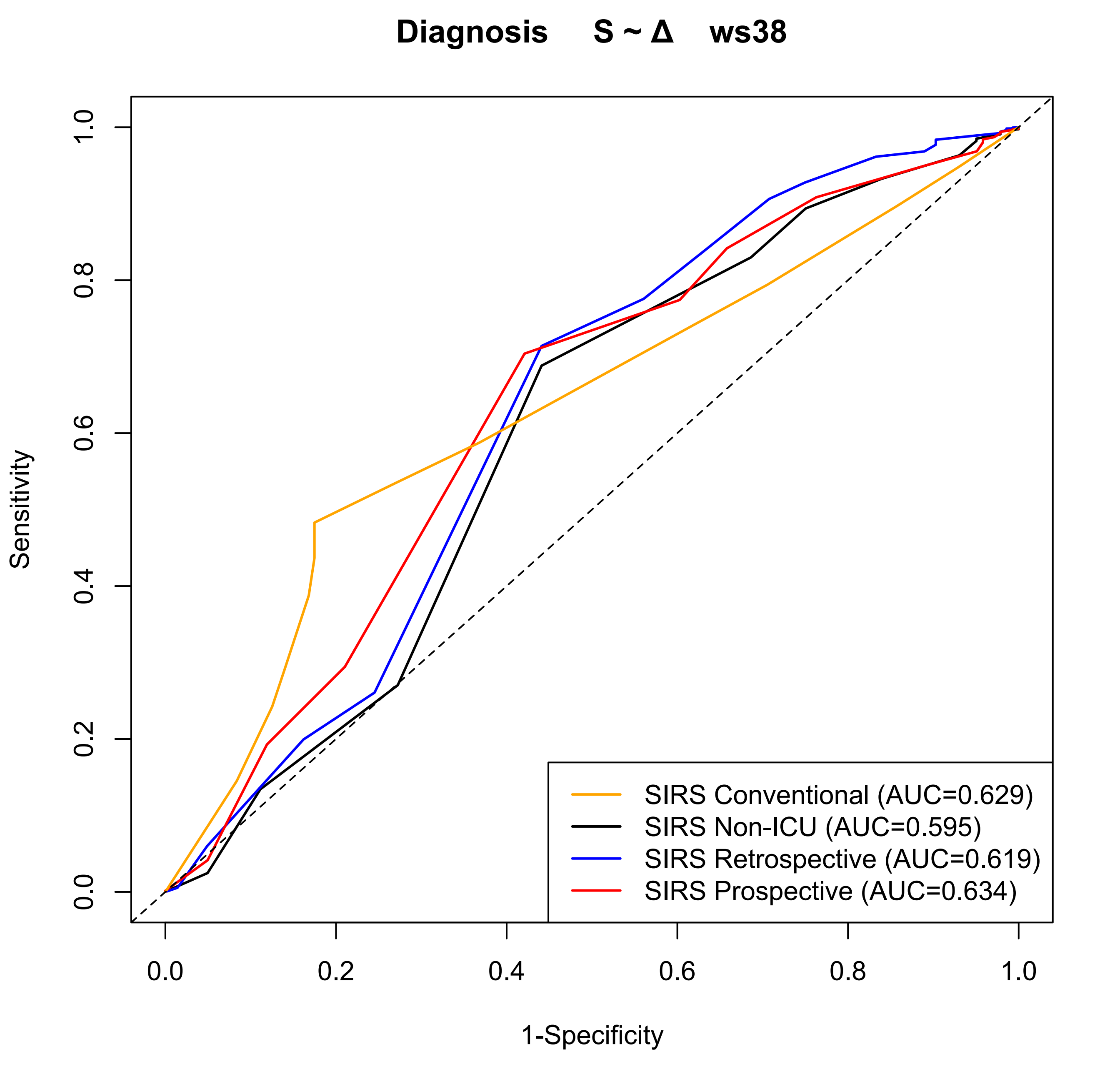
Diagnosis S ~ Δ ws38
Sensitivity
1-Specificity
0.0
0.2
0.4
0.6
0.8
1.0
SIRS Conventional (AUC=0.629)
SIRS Non-ICU (AUC=0.595)
SIRS Retrospective (AUC=0.619)
SIRS Prospective (AUC=0.634)

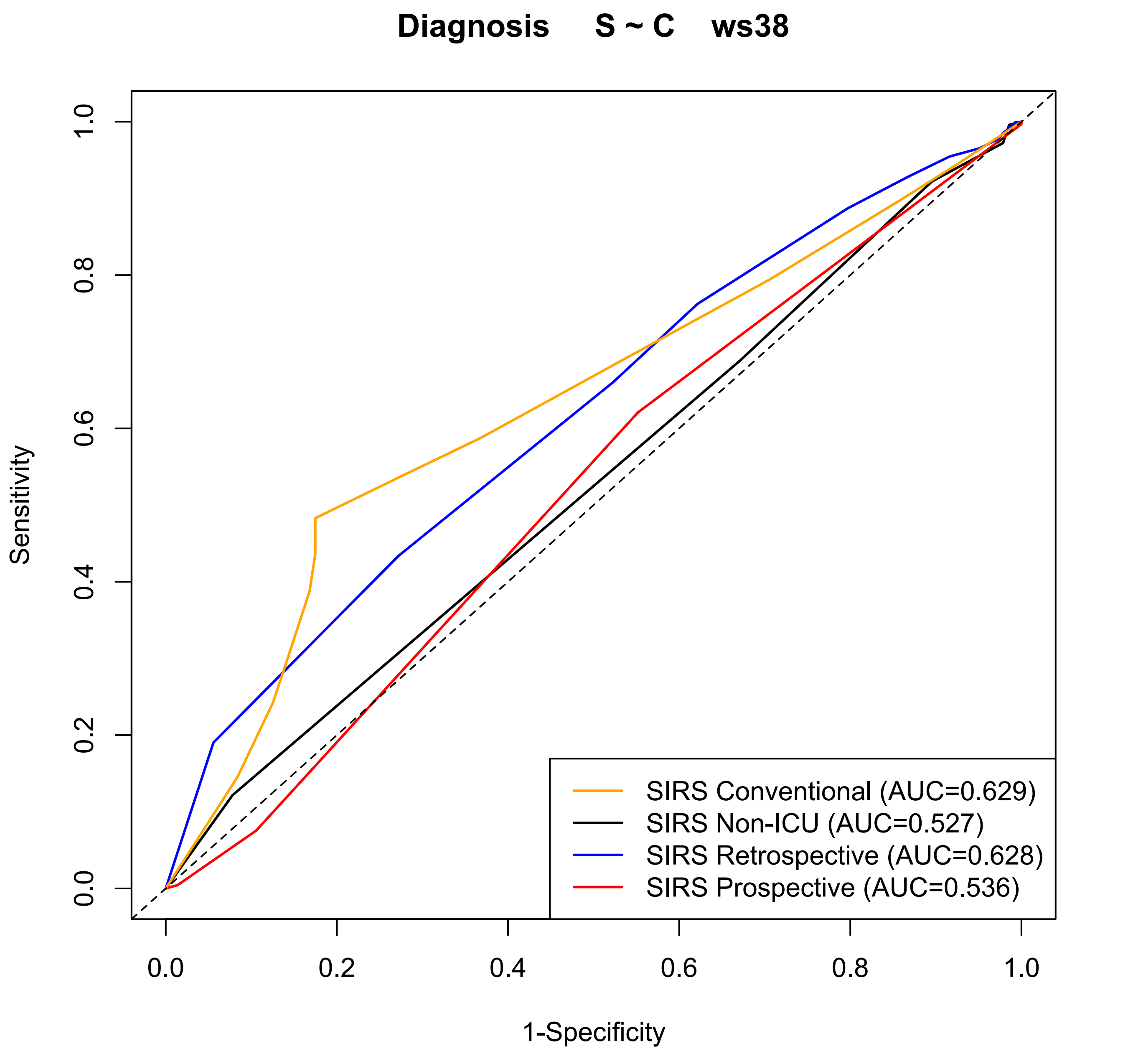

Diagnosis S ~ C ws38
Sensitivity
1-Specificity
0.0
0.2
0.4
0.6
0.8
1.0
SIRS Conventional (AUC=0.629)
SIRS Non-ICU (AUC=0.527)
SIRS Retrospective (AUC=0.628)
SIRS Prospective (AUC=0.536)

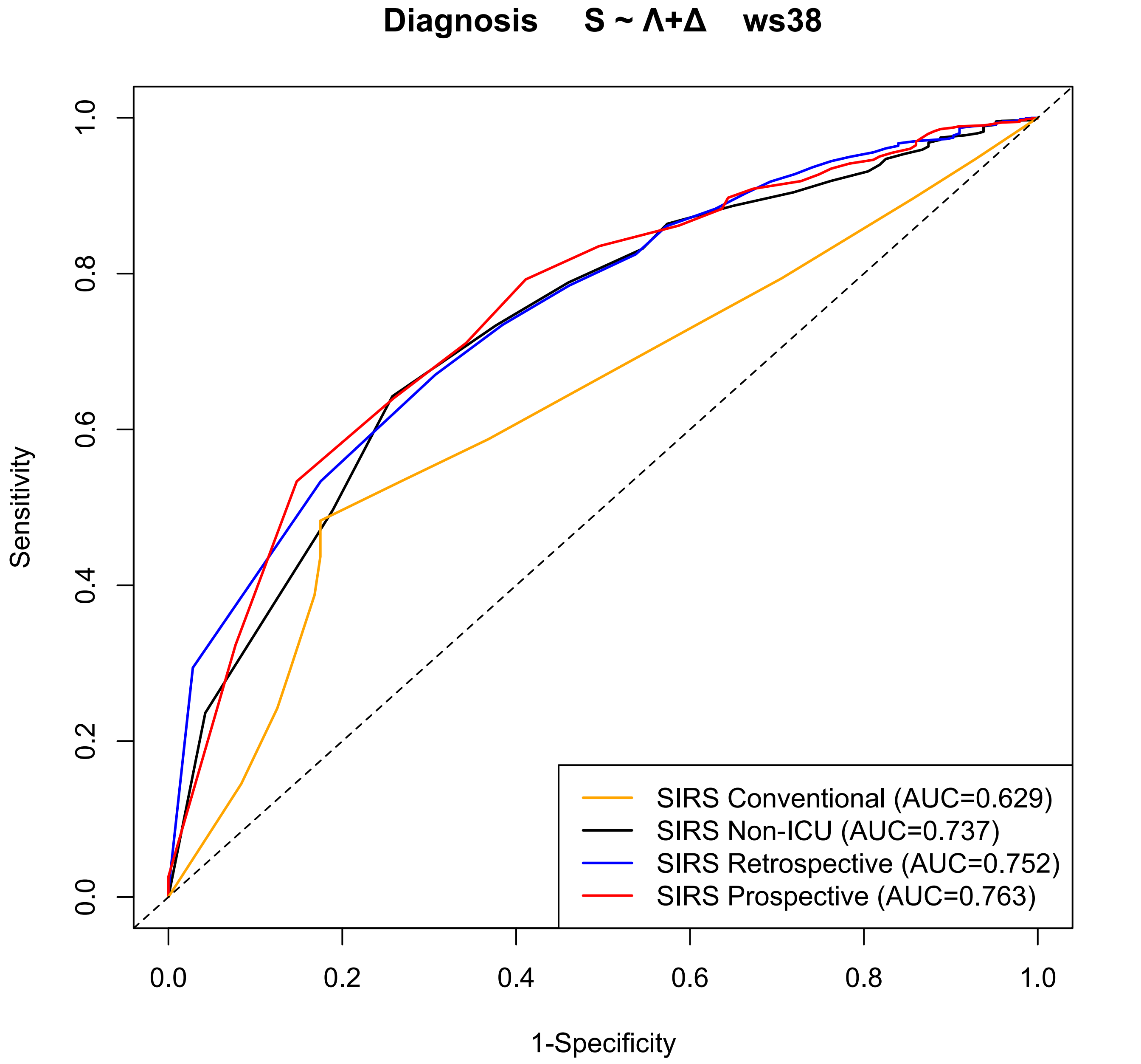
Diagnosis S ~ Λ+Δ ws38
Sensitivity
1-Specificity
0.0
0.2
0.4
0.6
0.8
1.0
SIRS Conventional (AUC=0.629)
SIRS Non-ICU (AUC=0.737)
SIRS Retrospective (AUC=0.752)
SIRS Prospective (AUC=0.763)

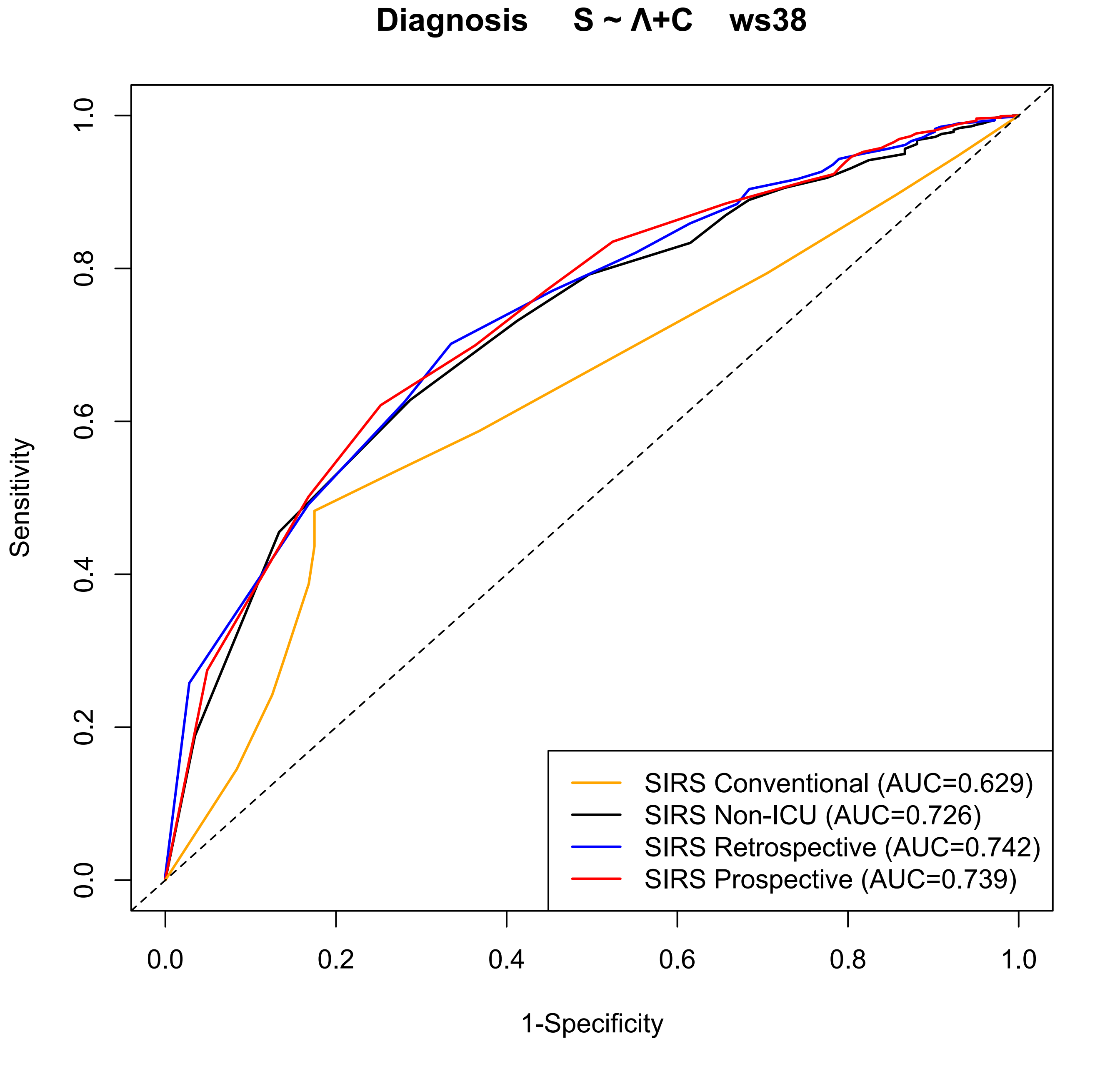

Diagnosis S ~ Λ+C ws38
Sensitivity
1-Specificity
0.0
0.2
0.4
0.6
0.8
1.0
SIRS Conventional (AUC=0.629)
SIRS Non-ICU (AUC=0.726)
SIRS Retrospective (AUC=0.742)
SIRS Prospective (AUC=0.739)

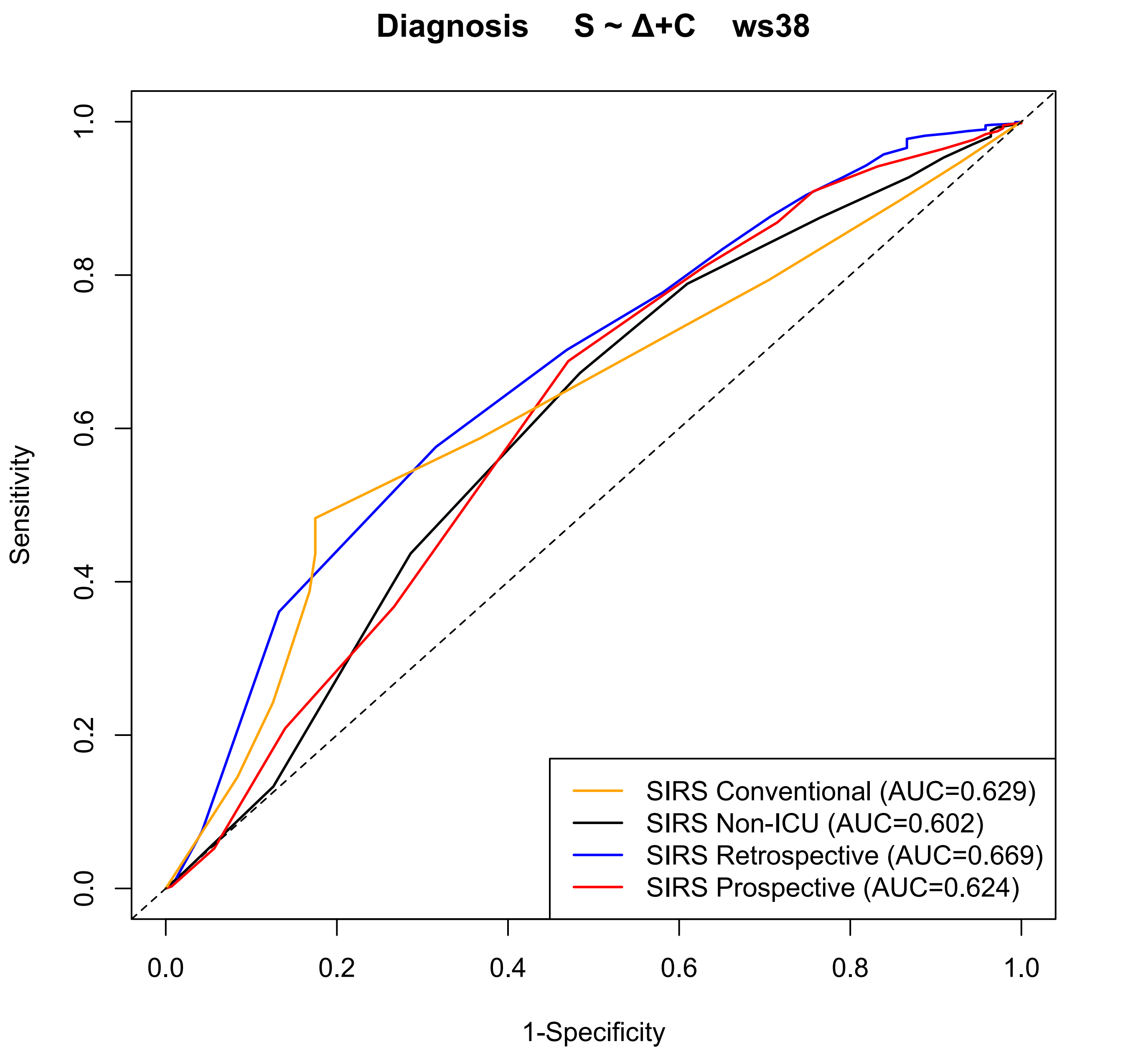

Diagnosis S ~ Δ+C ws38
Sensitivity
1-Specificity
0.0
0.2
0.4
0.6
0.8
1.0
SIRS Conventional (AUC=0.629)
SIRS Non-ICU (AUC=0.602)
SIRS Retrospective (AUC=0.669)
SIRS Prospective (AUC=0.624)

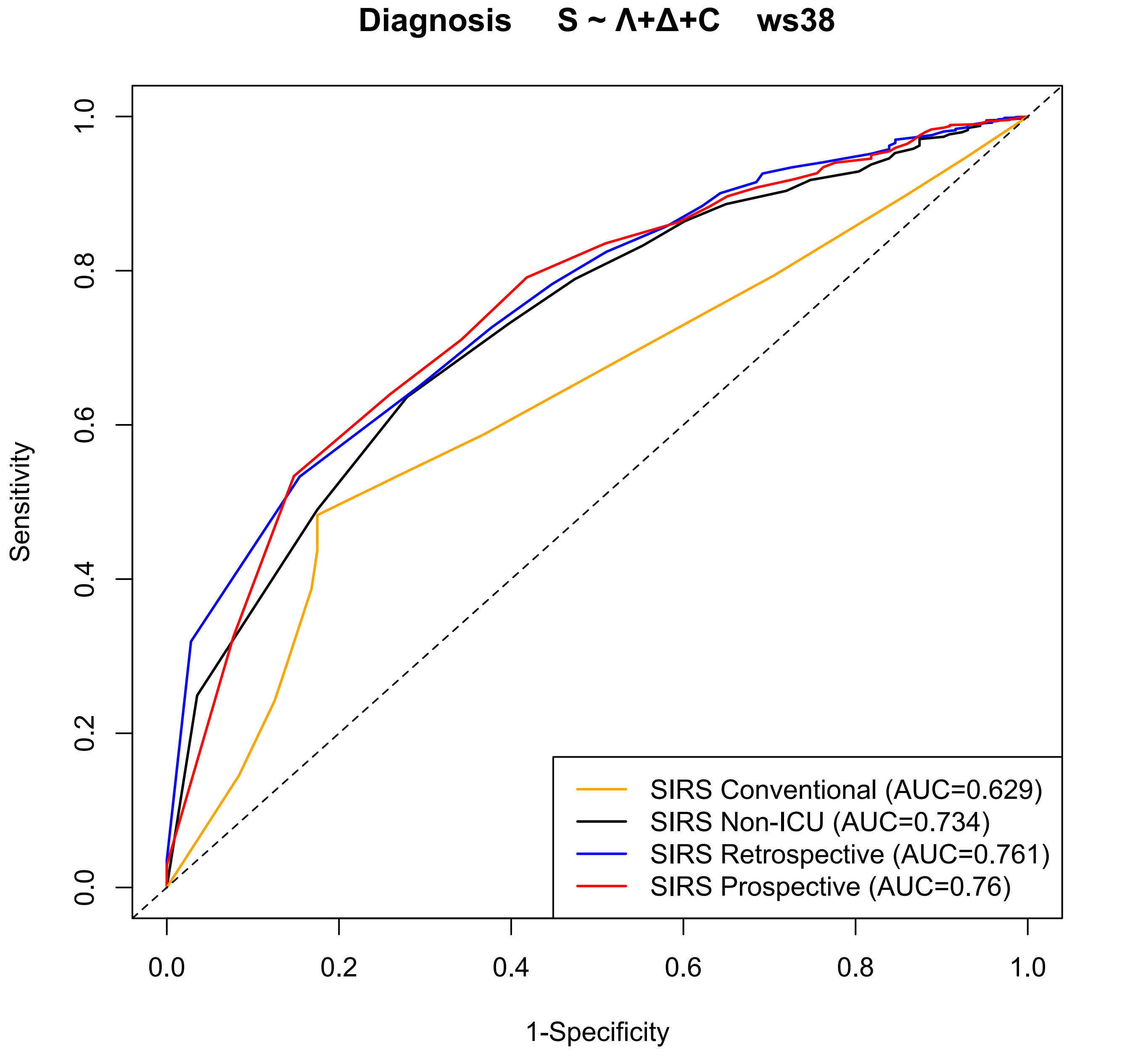

Diagnosis S ~ Λ+Δ+C ws38
Sensitivity
1-Specificity
0.0
0.2
0.4
0.6
0.8
1.0
0.0
0.2
0.4
0.6
0.8
1.0
SIRS Conventional (AUC=0.629)
SIRS Non-ICU (AUC=0.734)
SIRS Retrospective (AUC=0.761)
SIRS Prospective (AUC=0.76)

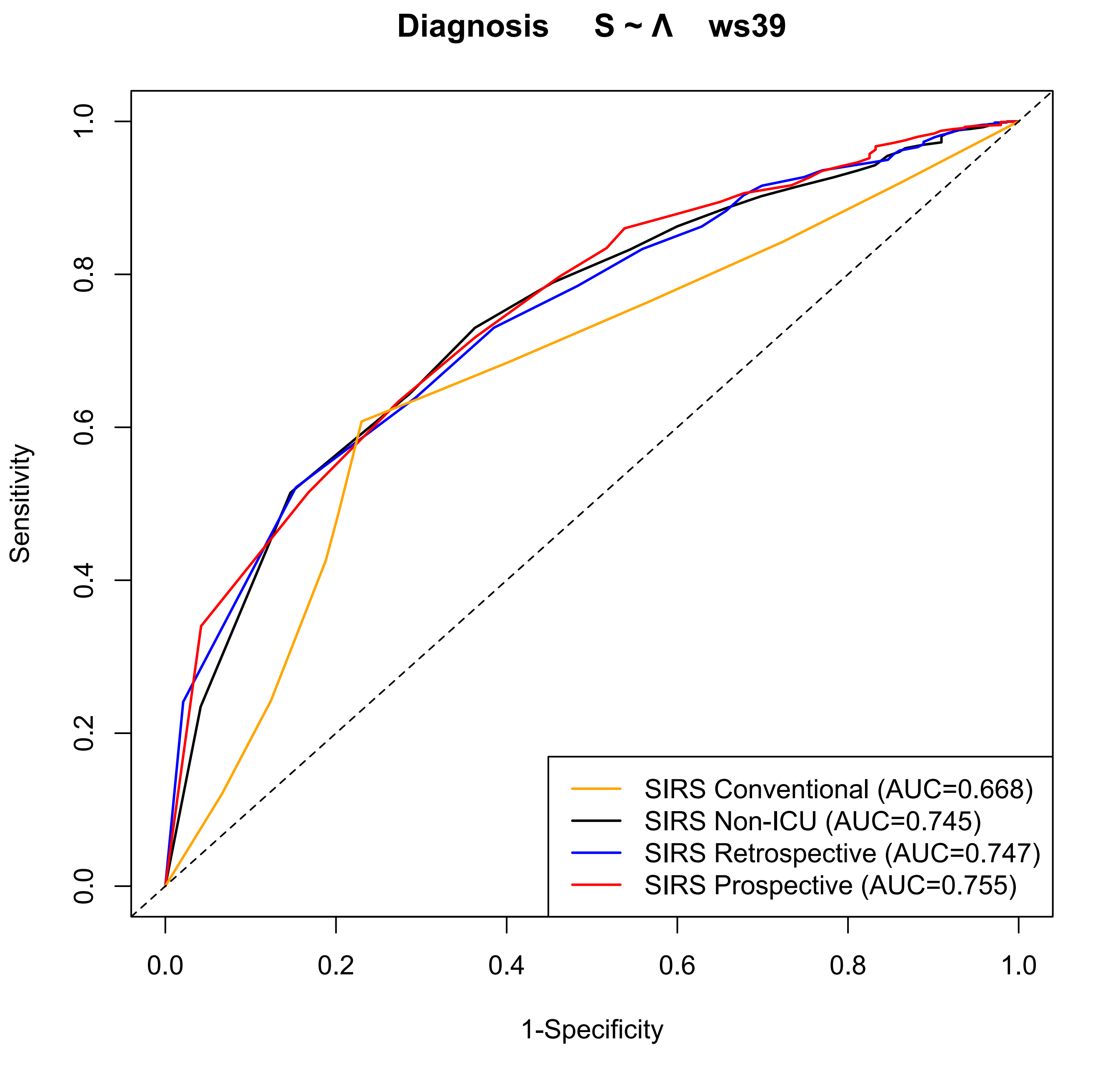

Diagnosis S ~ Λ ws39
Sensitivity
1-Specificity
0.0
0.2
0.4
0.6
0.8
1.0
SIRS Conventional (AUC=0.668)
SIRS Non-ICU (AUC=0.745)
SIRS Retrospective (AUC=0.747)
SIRS Prospective (AUC=0.755)

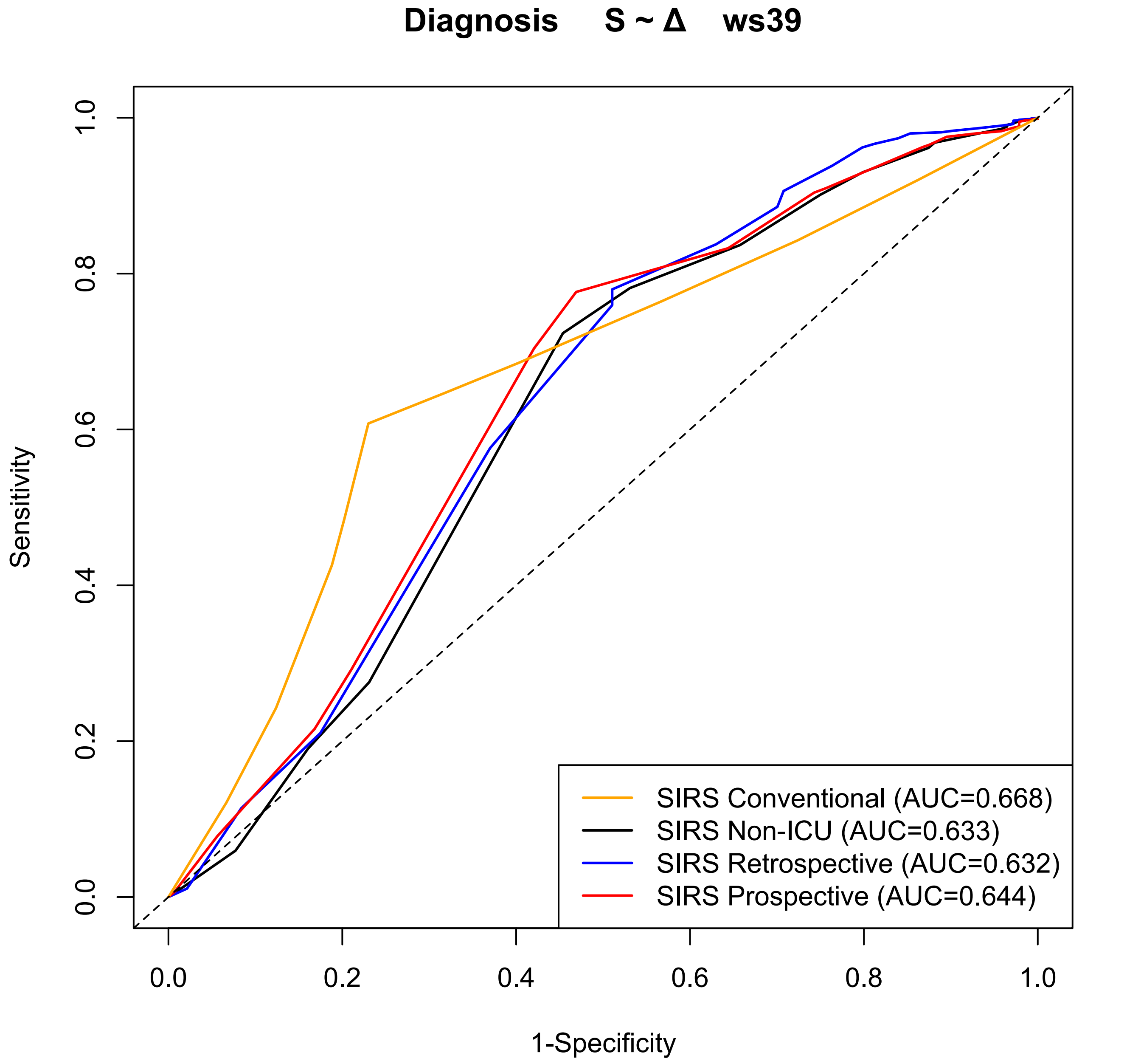

Diagnosis S ~ Δ ws39
Sensitivity
1-Specificity
0.0
0.2
0.4
0.6
0.8
1.0
SIRS Conventional (AUC=0.668)
SIRS Non-ICU (AUC=0.633)
SIRS Retrospective (AUC=0.632)
SIRS Prospective (AUC=0.644)

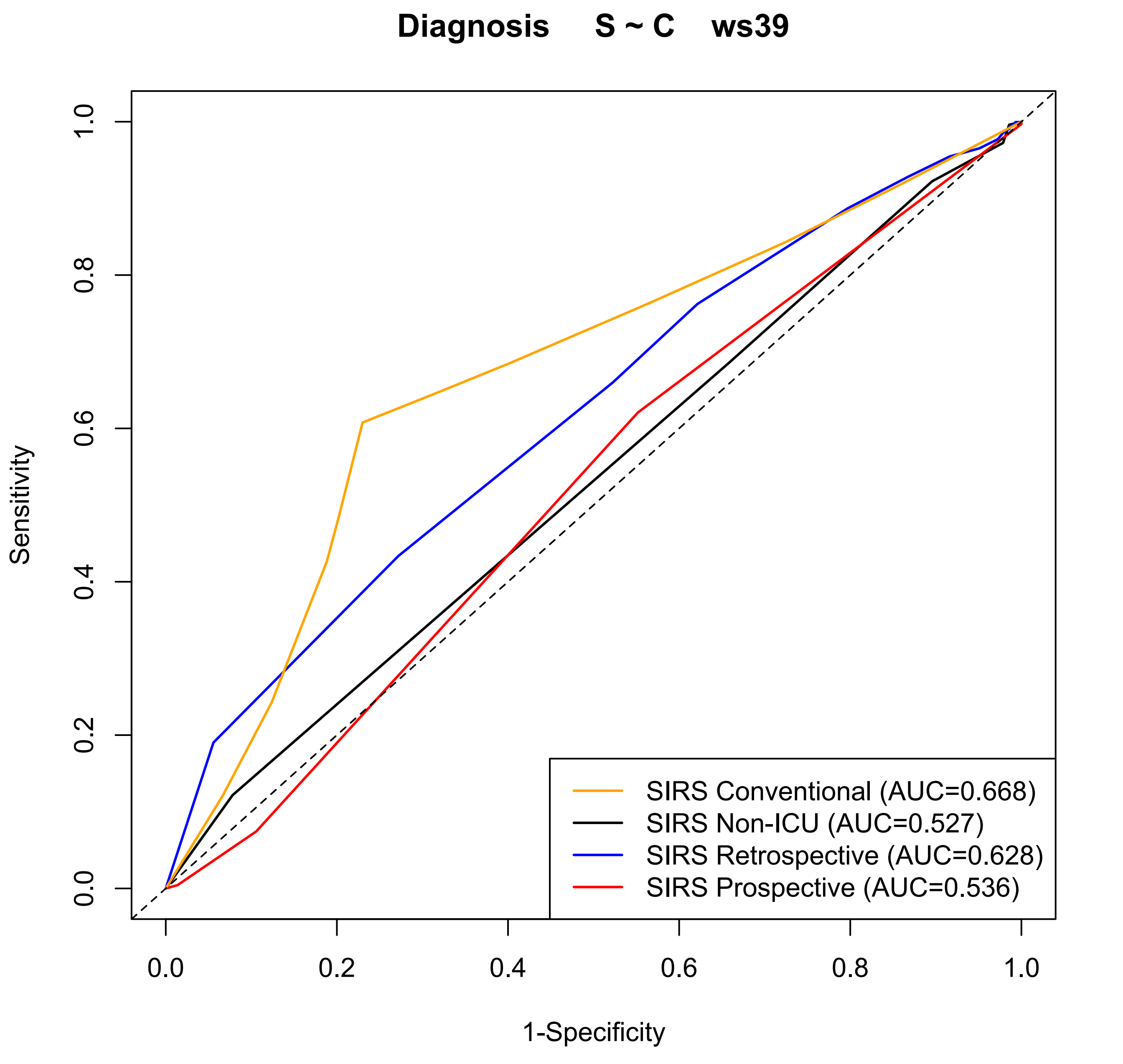

Diagnosis S ~ C ws39
Sensitivity
1-Specificity
0.0
0.2
0.4
0.6
0.8
1.0
SIRS Conventional (AUC=0.668)
SIRS Non-ICU (AUC=0.527)
SIRS Retrospective (AUC=0.628)
SIRS Prospective (AUC=0.536)

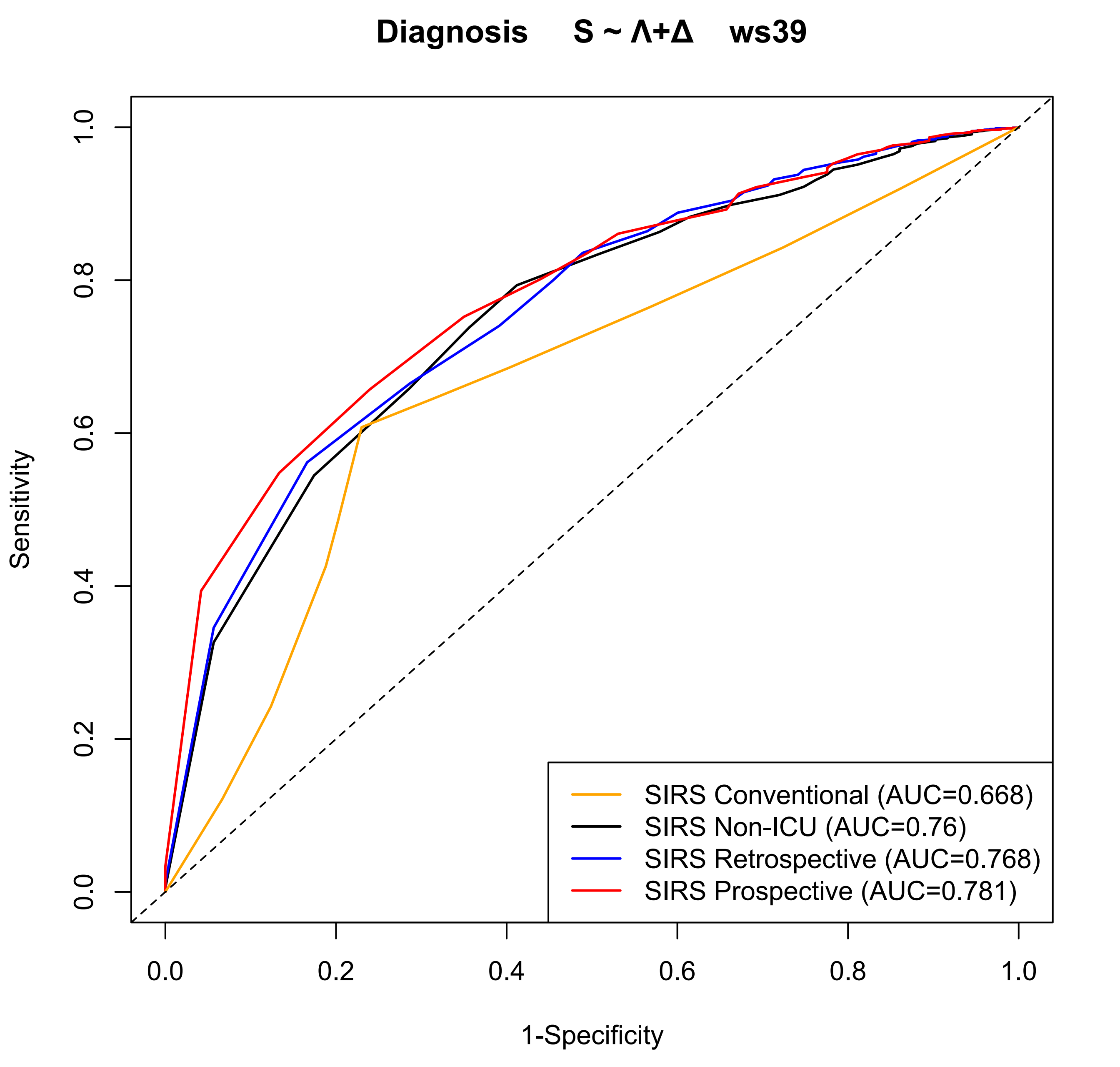
Diagnosis S ~ Λ+Δ ws39
Sensitivity
1-Specificity
0.0
0.2
0.4
0.6
0.8
1.0
SIRS Conventional (AUC=0.668)
SIRS Non-ICU (AUC=0.76)
SIRS Retrospective (AUC=0.768)
SIRS Prospective (AUC=0.781)

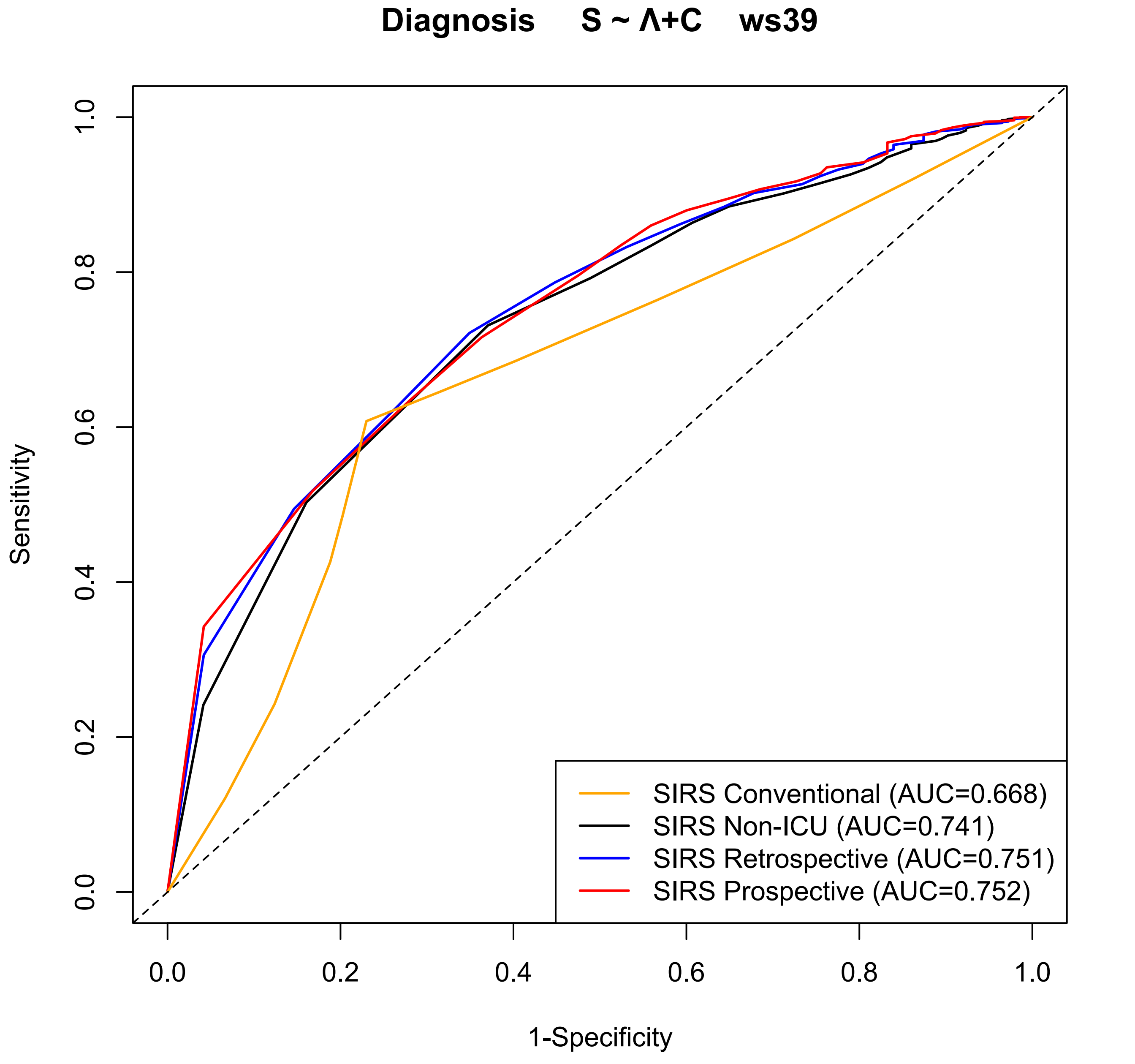

Diagnosis S ~ Λ+C ws39
Sensitivity
1-Specificity
0.0
0.2
0.4
0.6
0.8
1.0
SIRS Conventional (AUC=0.668)
SIRS Non-ICU (AUC=0.741)
SIRS Retrospective (AUC=0.751)
SIRS Prospective (AUC=0.752)

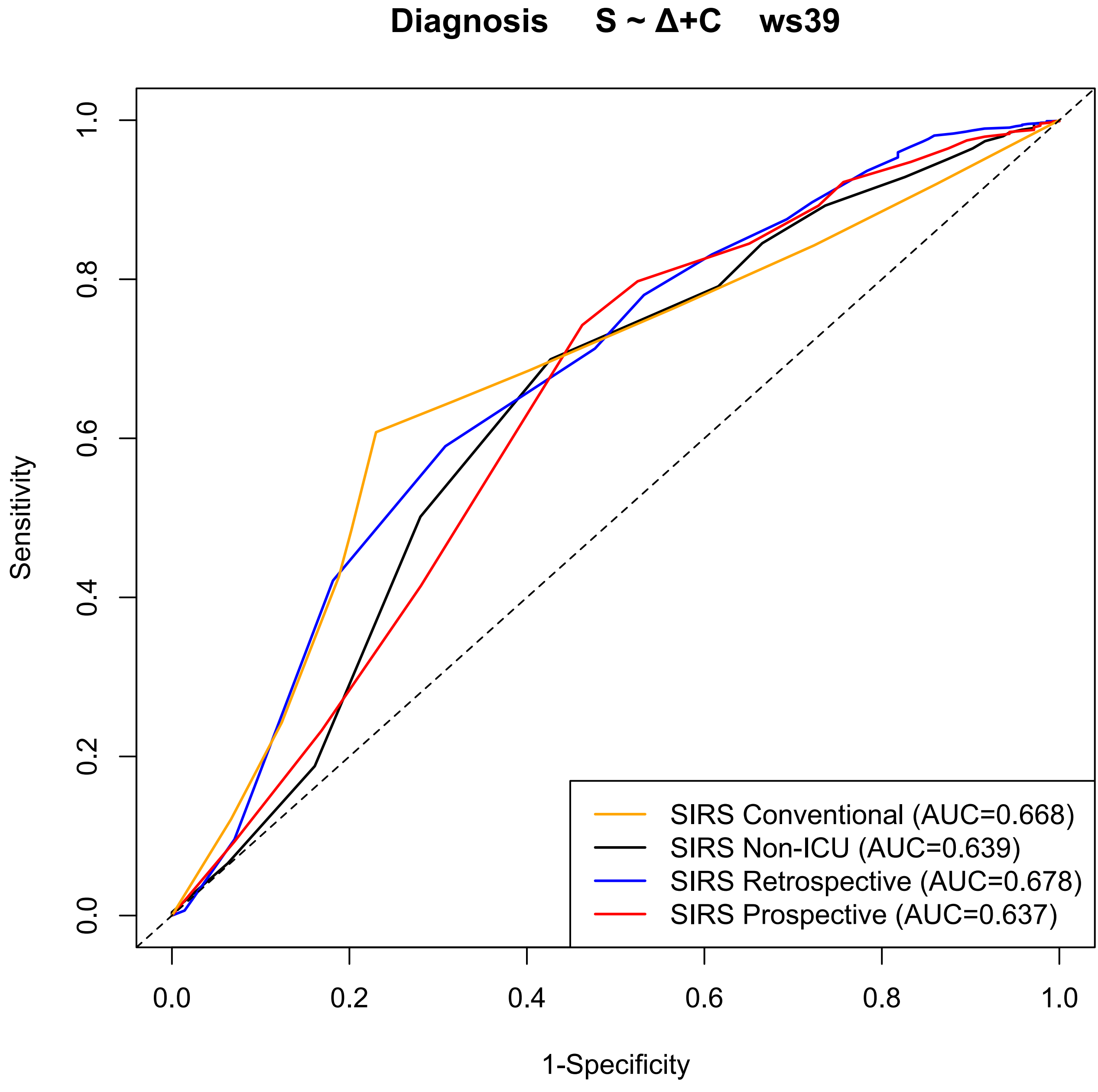

Diagnosis S ~ Δ+C ws39
Sensitivity
1-Specificity
0.0
0.2
0.4
0.6
0.8
1.0
SIRS Conventional (AUC=0.668)
SIRS Non-ICU (AUC=0.639)
SIRS Retrospective (AUC=0.678)
SIRS Prospective (AUC=0.637)

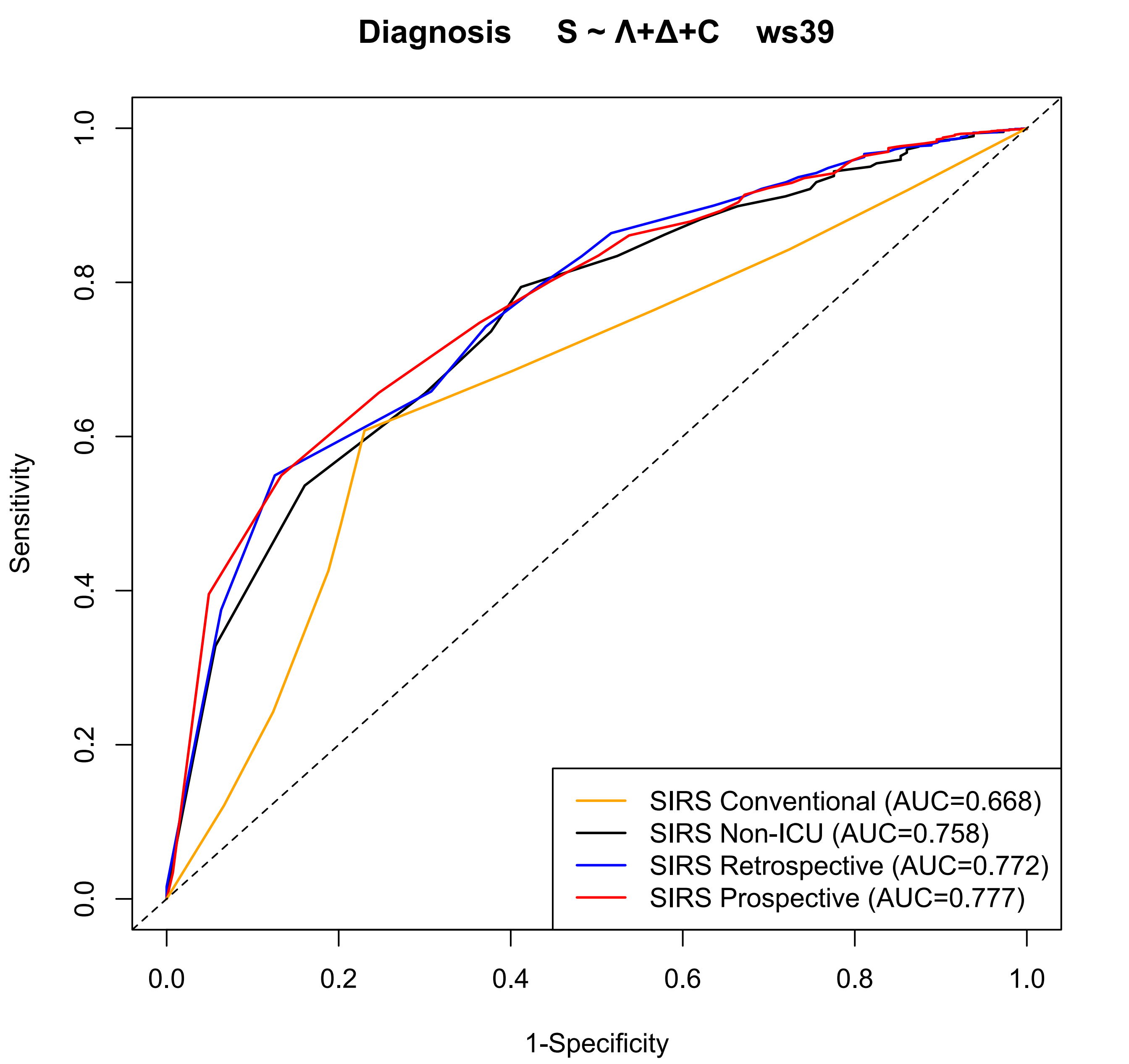
Diagnosis S ~ Λ+Δ+C ws39
Sensitivity
1-Specificity
0.0
0.2
0.4
0.6
0.8
1.0
SIRS Conventional (AUC=0.668)
SIRS Non-ICU (AUC=0.758)
SIRS Retrospective (AUC=0.772)
SIRS Prospective (AUC=0.777)

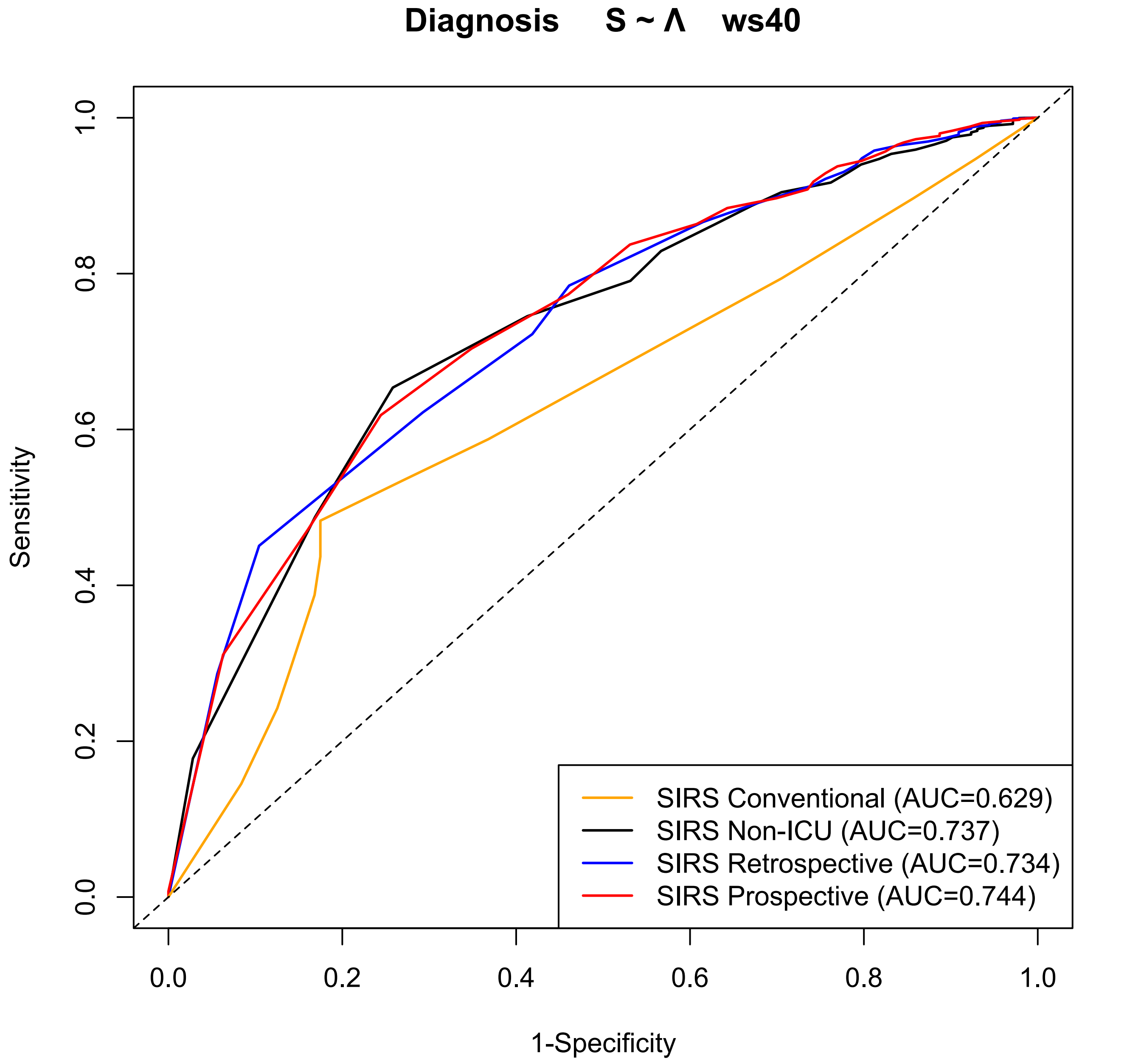
Diagnosis S ~ Λ ws40
Sensitivity
1-Specificity
0.0
0.2
0.4
0.6
0.8
1.0
SIRS Conventional (AUC=0.629)
SIRS Non-ICU (AUC=0.737)
SIRS Retrospective (AUC=0.734)
SIRS Prospective (AUC=0.744)

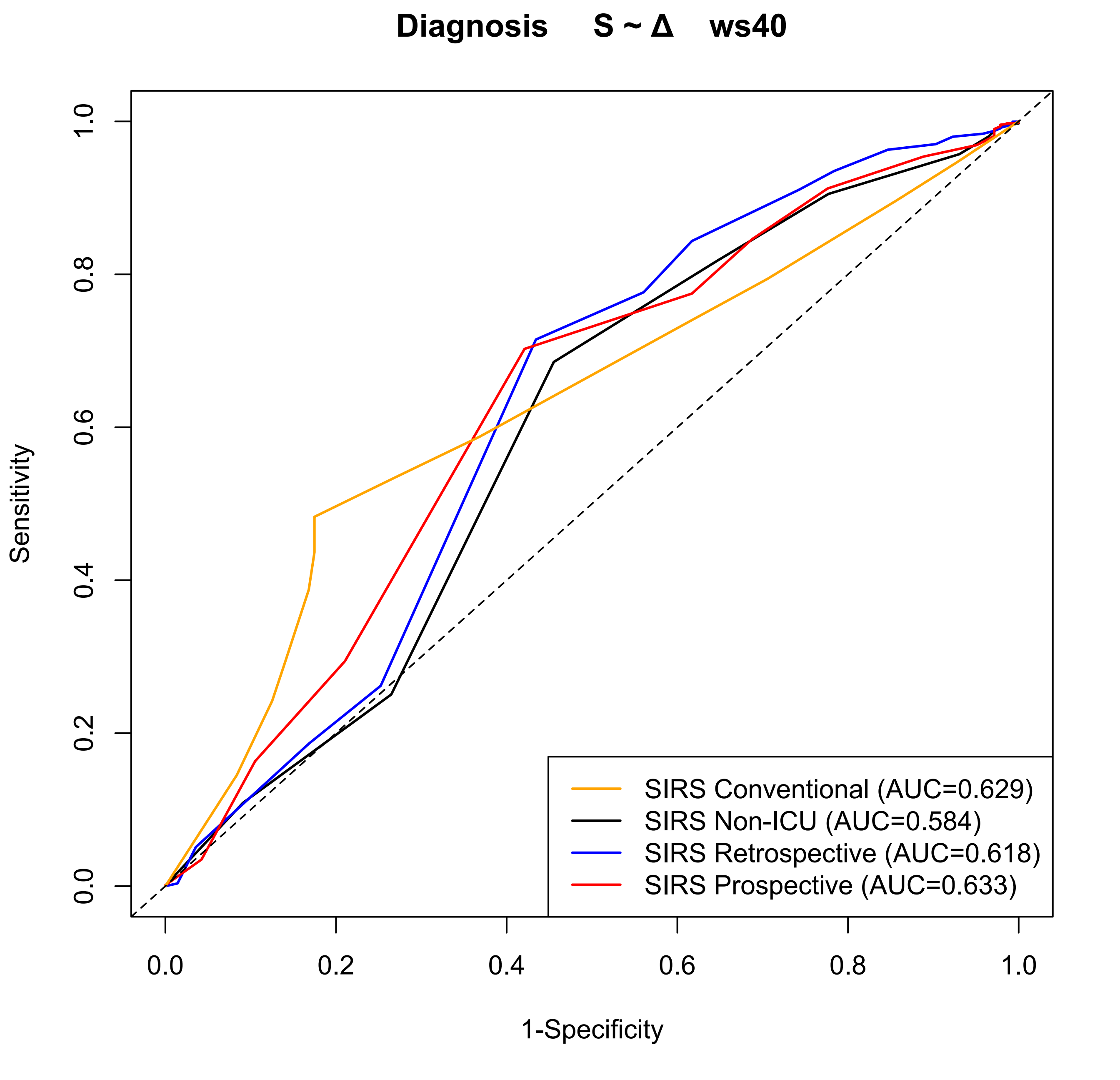

Diagnosis S ~ Δ ws40
Sensitivity
1-Specificity
0.0
0.2
0.4
0.6
0.8
1.0
SIRS Conventional (AUC=0.629)
SIRS Non-ICU (AUC=0.584)
SIRS Retrospective (AUC=0.618)
SIRS Prospective (AUC=0.633)

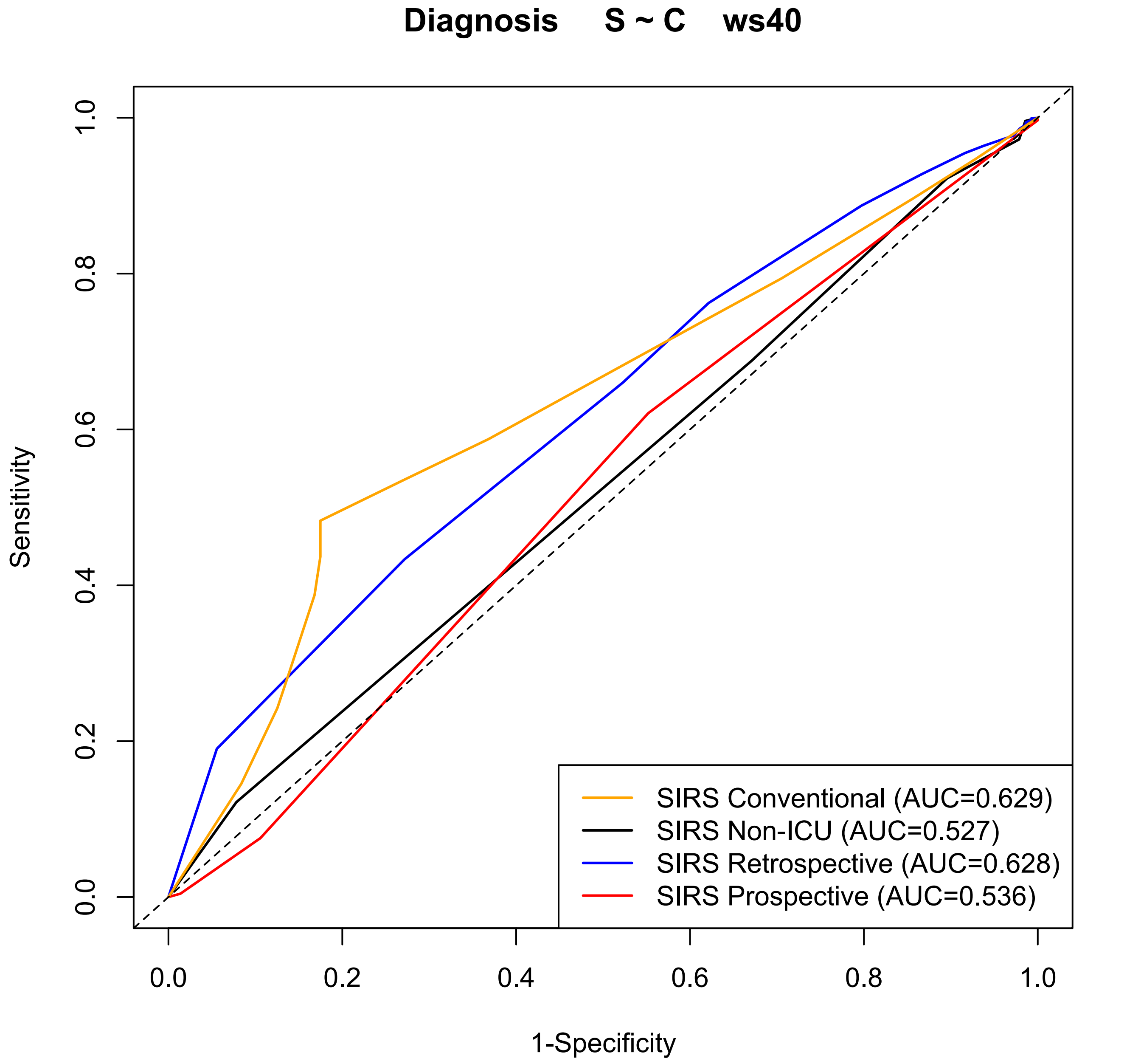
Diagnosis S ~ C ws40
Sensitivity
1-Specificity
0.0
0.2
0.4
0.6
0.8
1.0
SIRS Conventional (AUC=0.629)
SIRS Non-ICU (AUC=0.527)
SIRS Retrospective (AUC=0.628)
SIRS Prospective (AUC=0.536)

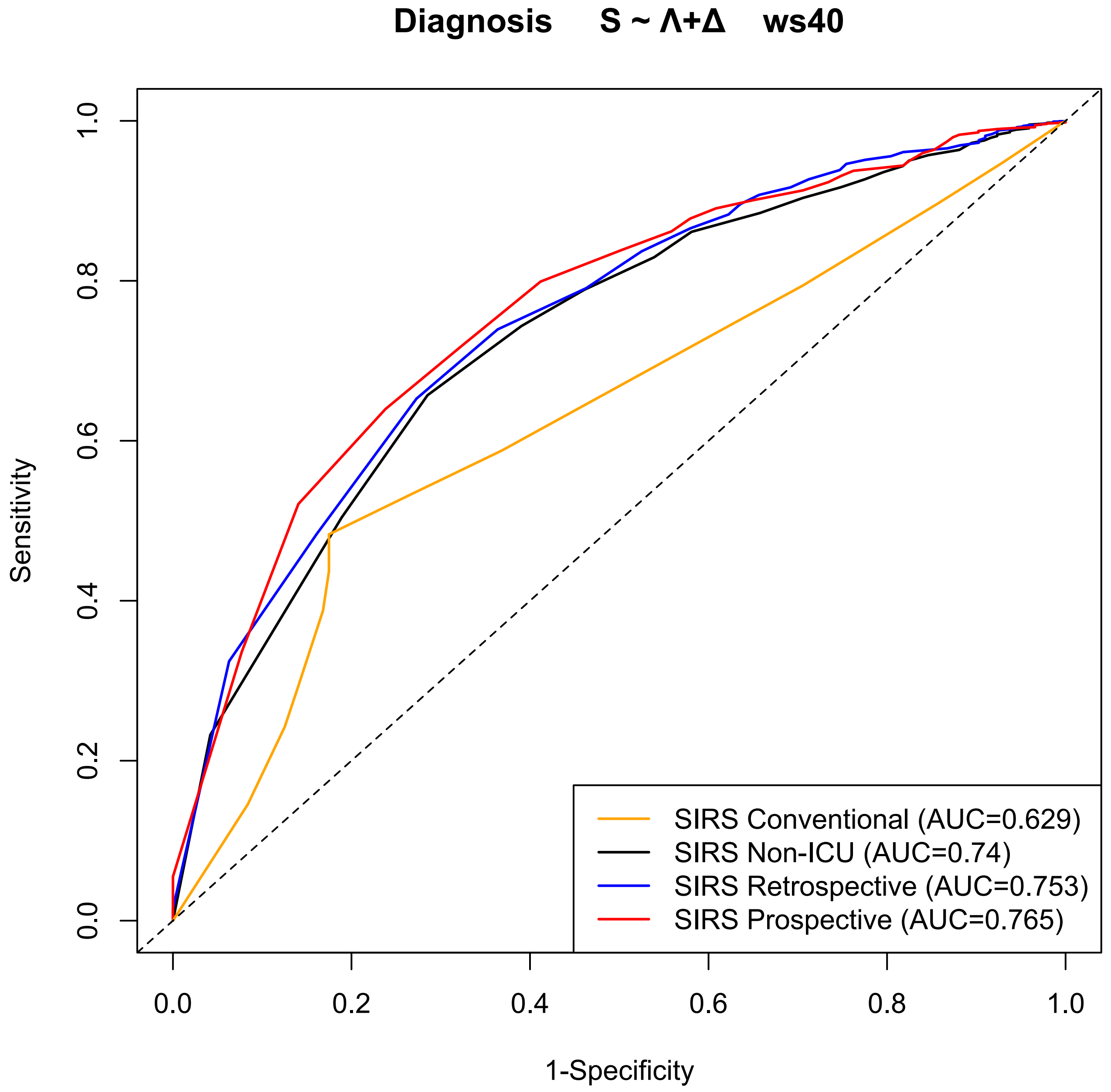
Diagnosis S ~ Λ+Δ ws40
Sensitivity
1-Specificity
0.0
0.2
0.4
0.6
0.8
1.0
SIRS Conventional (AUC=0.629)
SIRS Non-ICU (AUC=0.74)
SIRS Retrospective (AUC=0.753)
SIRS Prospective (AUC=0.765)

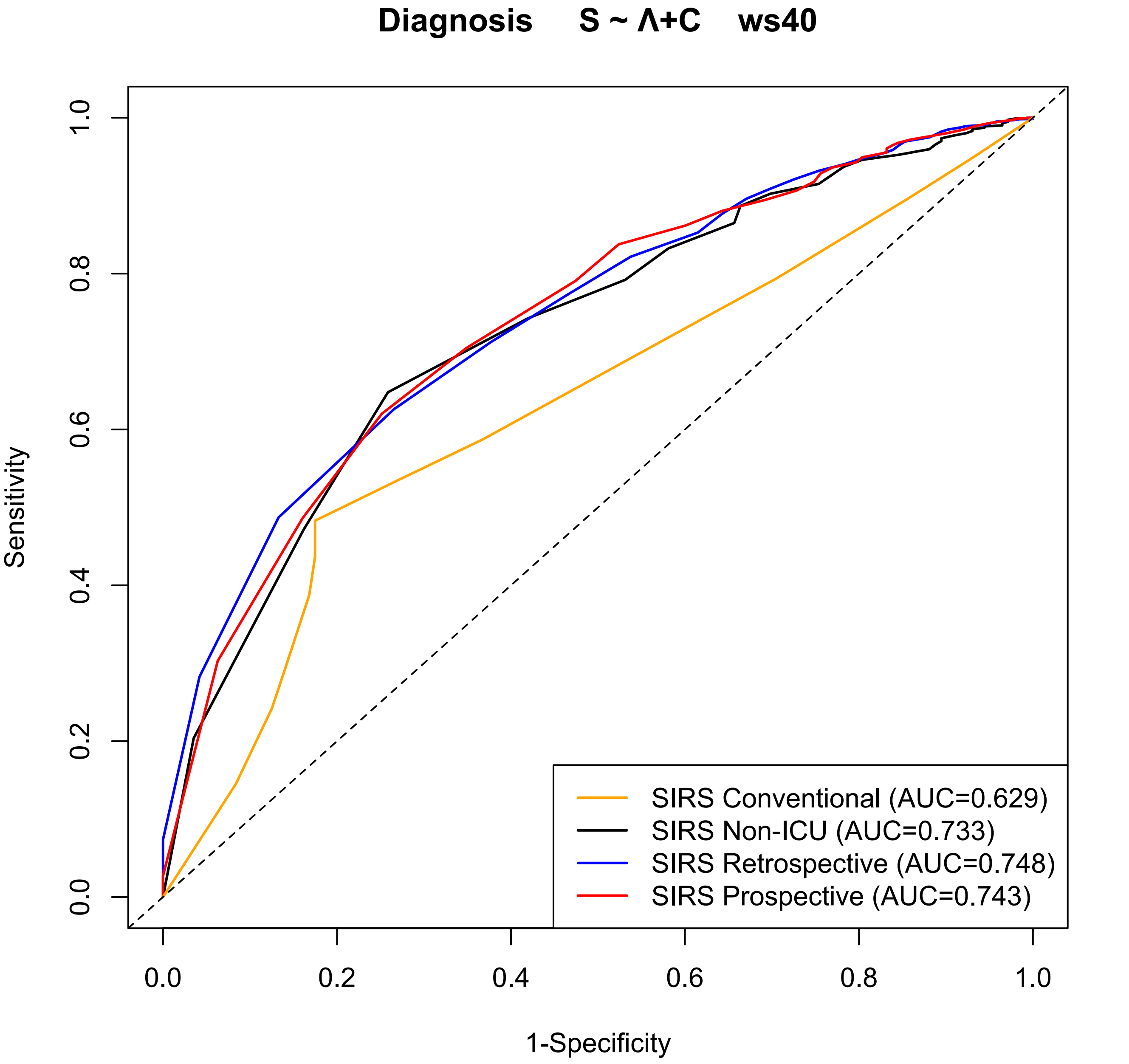
Diagnosis S ~ Λ+C ws40
Sensitivity
1-Specificity
0.0
0.2
0.4
0.6
0.8
1.0
SIRS Conventional (AUC=0.629)
SIRS Non-ICU (AUC=0.733)
SIRS Retrospective (AUC=0.748)
SIRS Prospective (AUC=0.743)

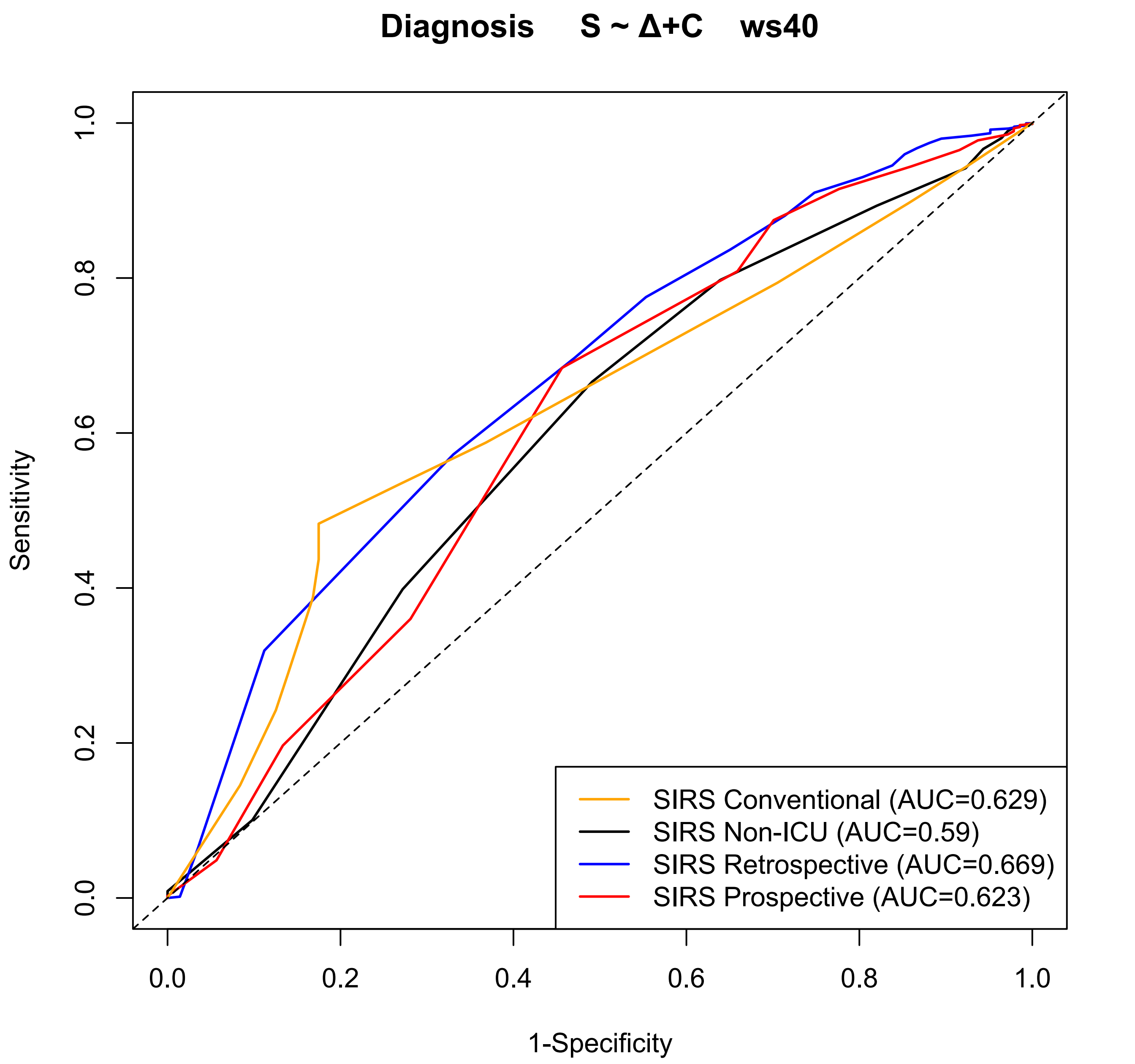

Diagnosis S ~ Δ+C ws40
Sensitivity
1-Specificity
0.0
0.2
0.4
0.6
0.8
1.0
SIRS Conventional (AUC=0.629)
SIRS Non-ICU (AUC=0.59)
SIRS Retrospective (AUC=0.669)
SIRS Prospective (AUC=0.623)

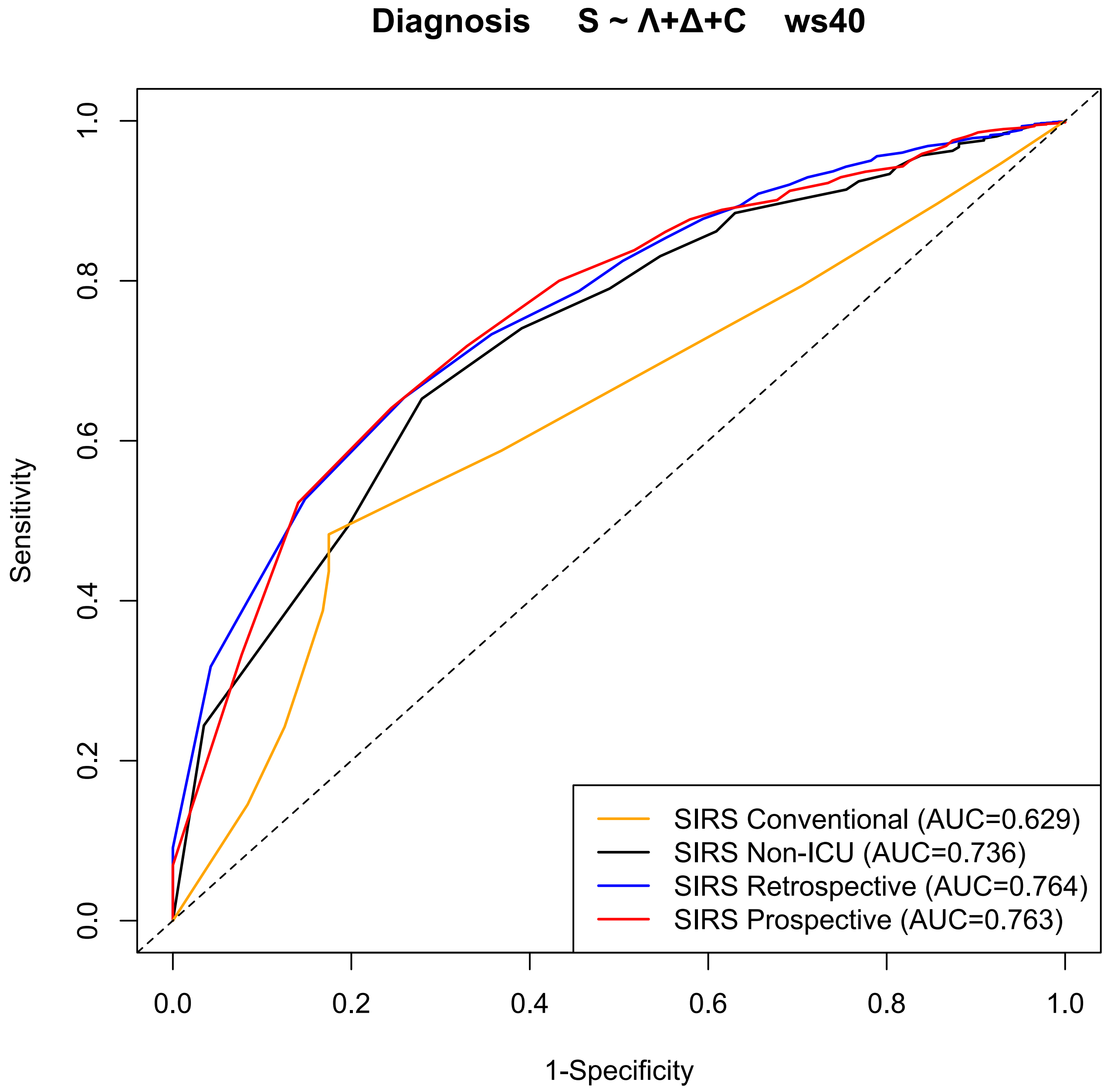

Diagnosis S ~ Λ+Δ+C ws40
Sensitivity
1-Specificity
0.0
0.2
0.4
0.6
0.8
1.0
SIRS Conventional (AUC=0.629)
SIRS Non-ICU (AUC=0.736)
SIRS Retrospective (AUC=0.764)
SIRS Prospective (AUC=0.763)

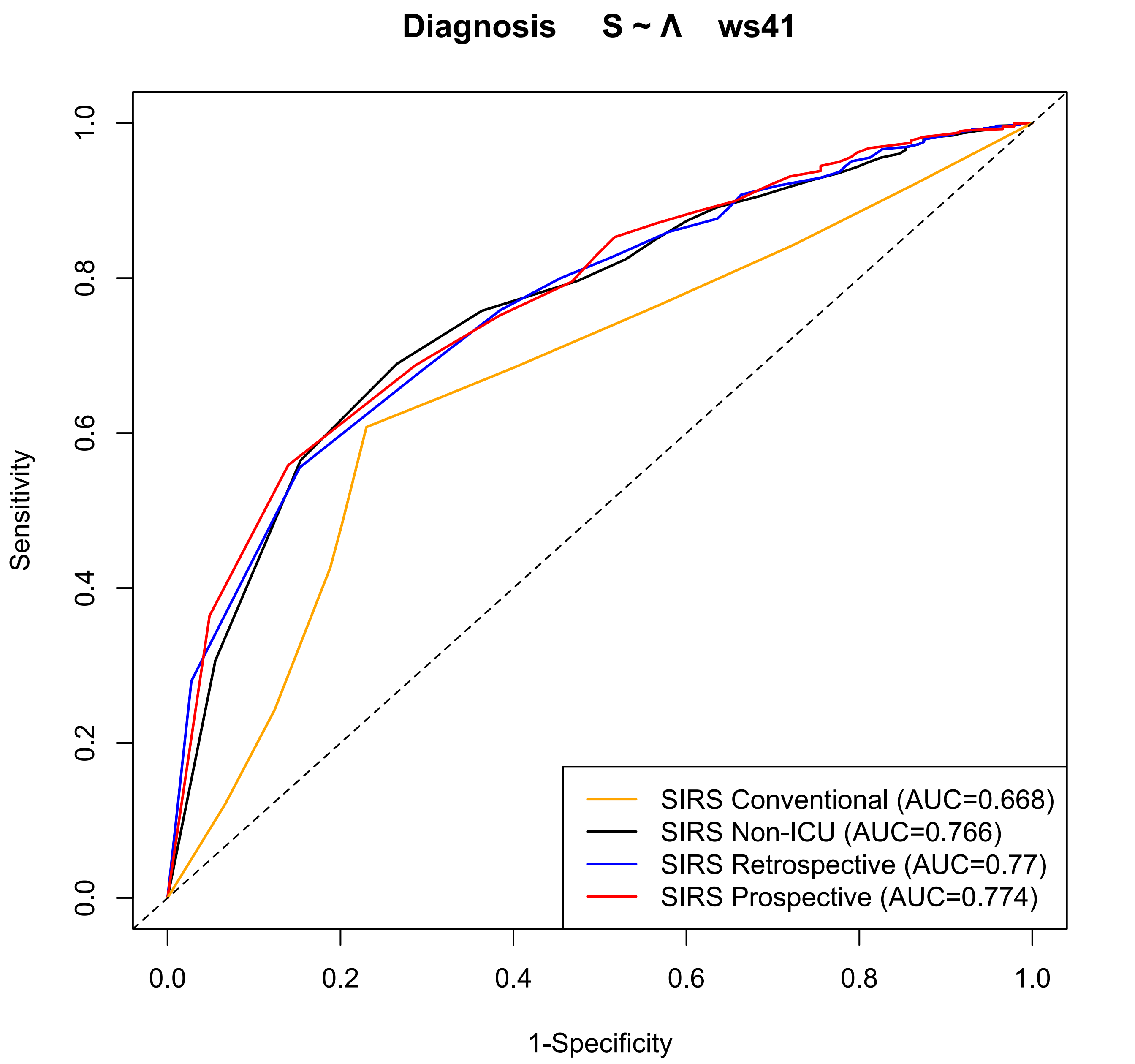
Diagnosis S ~ Λ ws41
Sensitivity
1-Specificity
0.0
0.2
0.4
0.6
0.8
1.0
SIRS Conventional (AUC=0.668)
SIRS Non-ICU (AUC=0.766)
SIRS Retrospective (AUC=0.77)
SIRS Prospective (AUC=0.774)

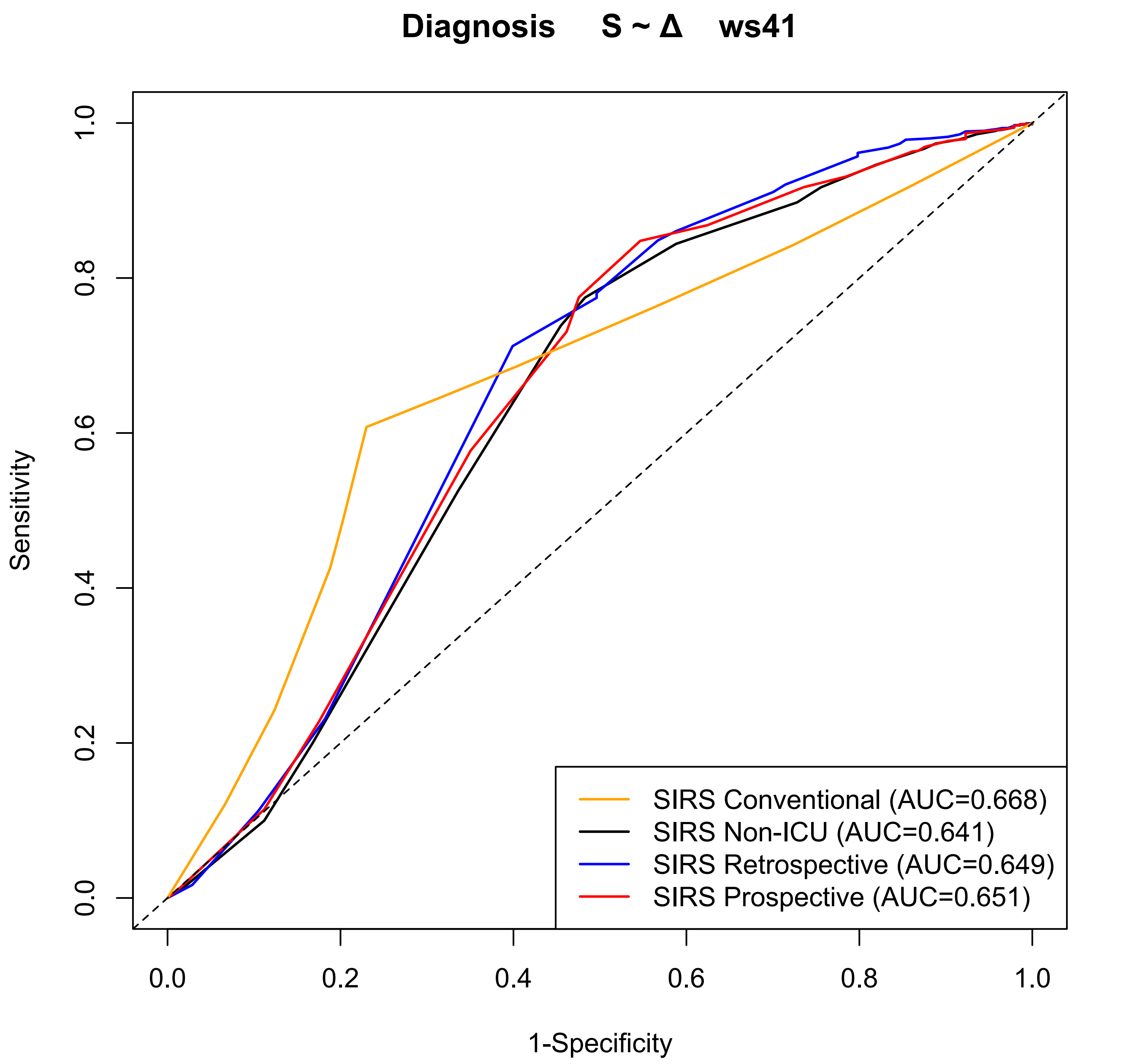

Diagnosis S ~ Δ ws41
Sensitivity
1-Specificity
0.0
0.2
0.4
0.6
0.8
1.0
SIRS Conventional (AUC=0.668)
SIRS Non-ICU (AUC=0.641)
SIRS Retrospective (AUC=0.649)
SIRS Prospective (AUC=0.651)

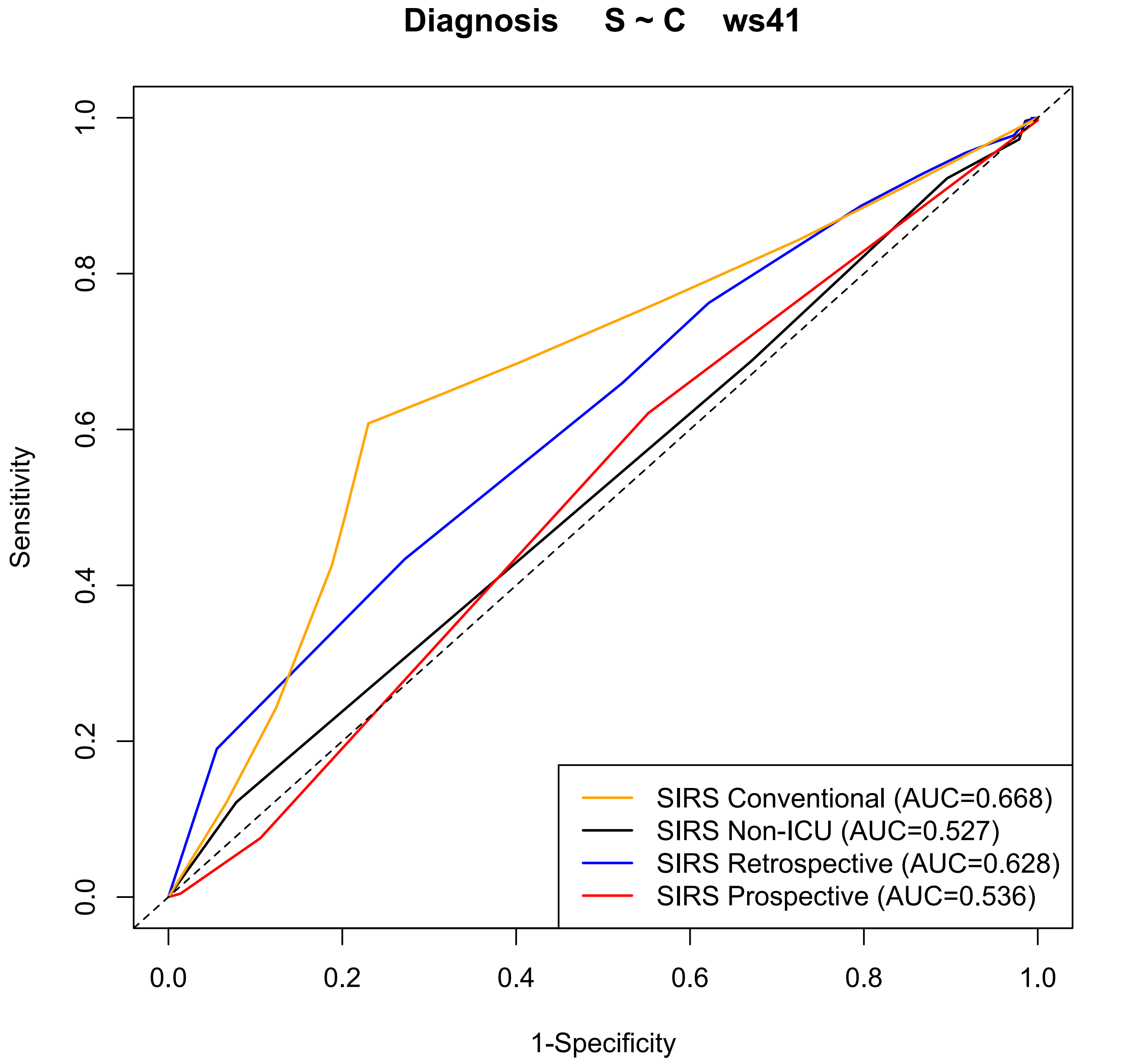

Diagnosis S ~ C ws41
Sensitivity
1-Specificity
0.0
0.2
0.4
0.6
0.8
1.0
SIRS Conventional (AUC=0.668)
SIRS Non-ICU (AUC=0.527)
SIRS Retrospective (AUC=0.628)
SIRS Prospective (AUC=0.536)

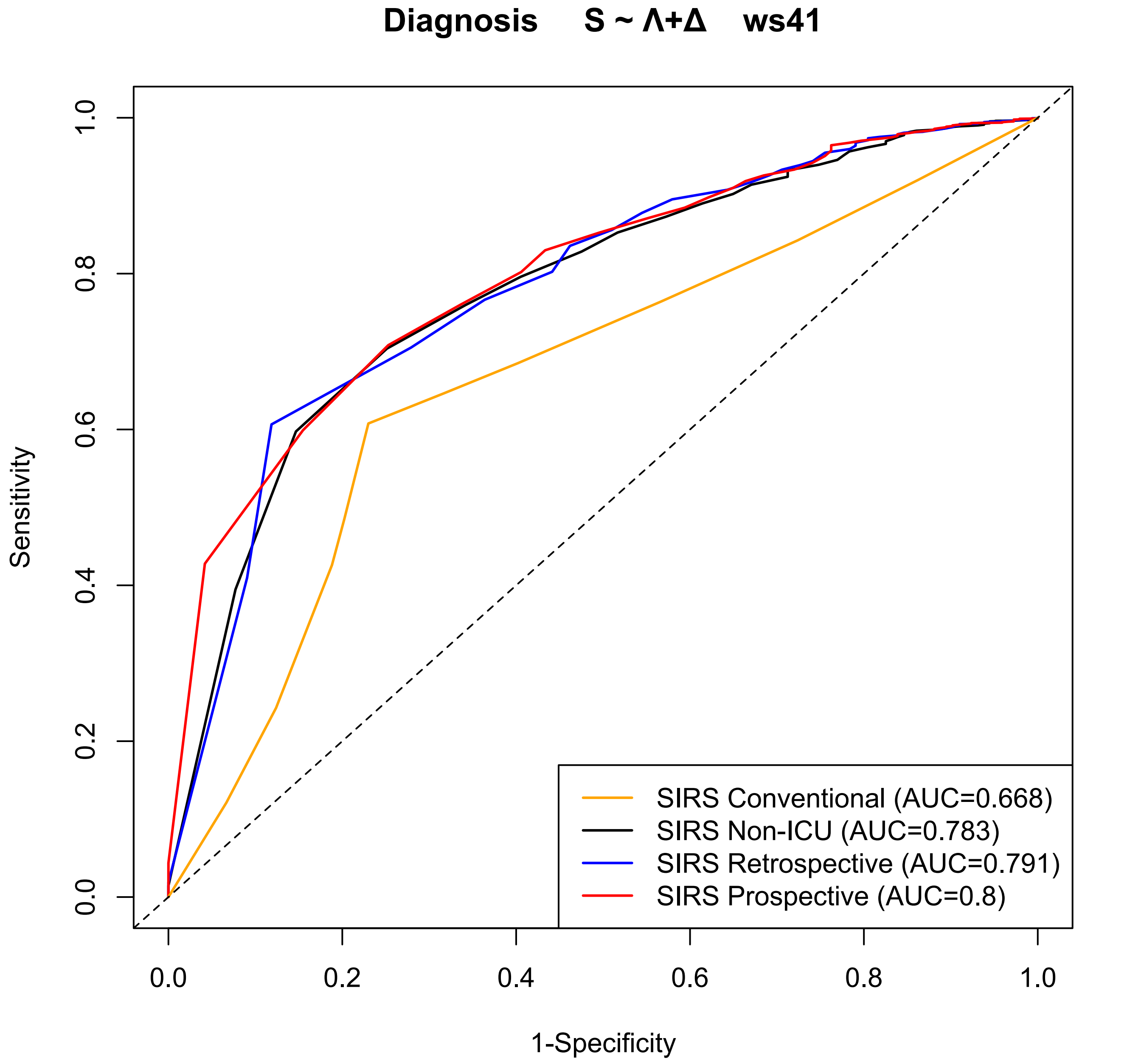
Diagnosis S ~ Λ+Δ ws41
Sensitivity
1-Specificity
0.0
0.2
0.4
0.6
0.8
1.0
SIRS Conventional (AUC=0.668)
SIRS Non-ICU (AUC=0.783)
SIRS Retrospective (AUC=0.791)
SIRS Prospective (AUC=0.8)

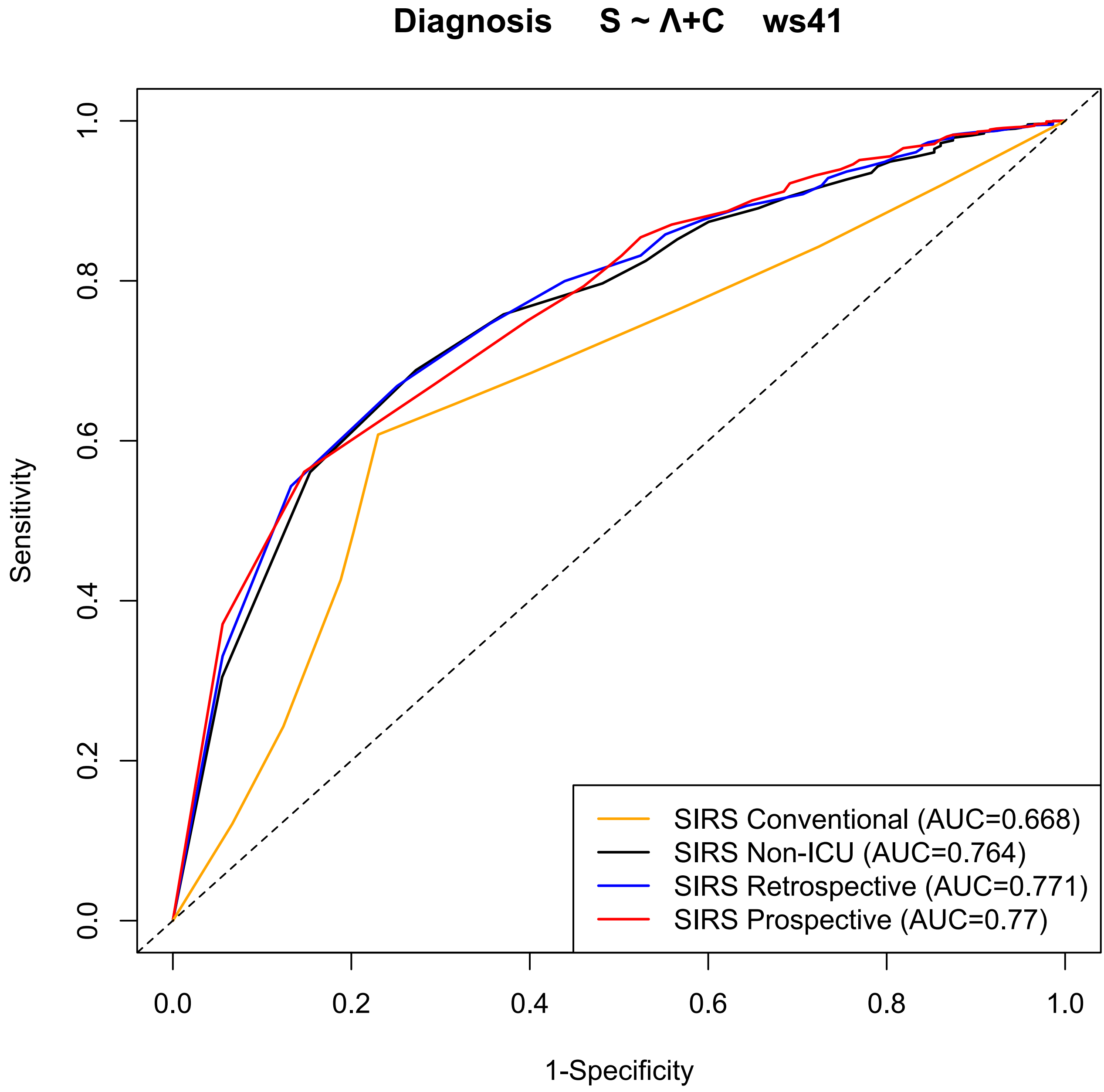

Diagnosis S ~ Λ+C ws41
Sensitivity
1-Specificity
0.0
0.2
0.4
0.6
0.8
1.0
SIRS Conventional (AUC=0.668)
SIRS Non-ICU (AUC=0.764)
SIRS Retrospective (AUC=0.771)
SIRS Prospective (AUC=0.77)

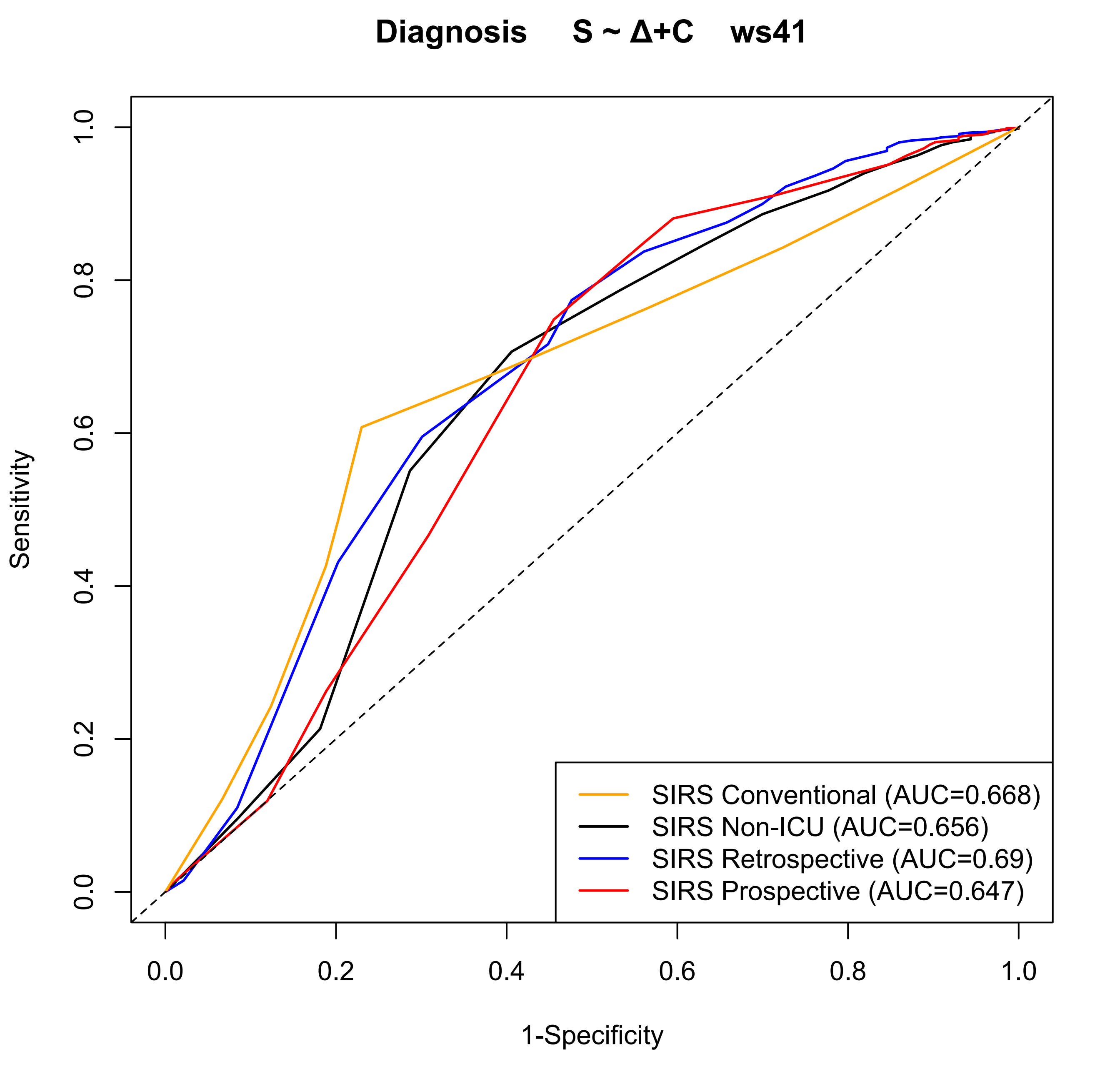
Diagnosis S ~ Δ+C ws41
Sensitivity
1-Specificity
0.0
0.2
0.4
0.6
0.8
1.0
0.0
0.2
0.4
0.6
0.8
1.0
SIRS Conventional (AUC=0.668)
SIRS Non-ICU (AUC=0.656)
SIRS Retrospective (AUC=0.69)
SIRS Prospective (AUC=0.647)

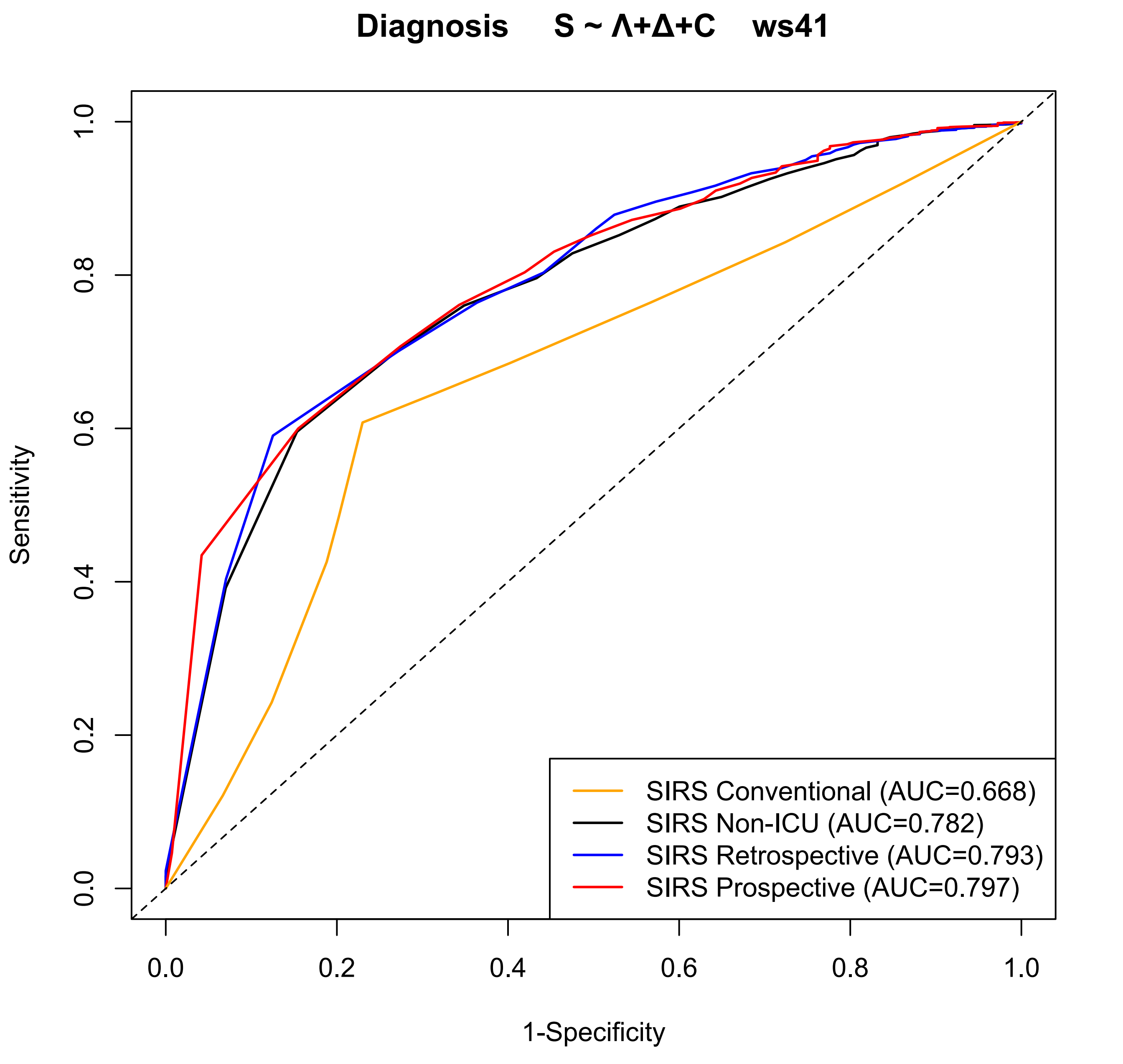

Diagnosis S ~ Λ+Δ+C ws41
Sensitivity
1-Specificity
0.0
0.2
0.4
0.6
0.8
1.0
SIRS Conventional (AUC=0.668)
SIRS Non-ICU (AUC=0.782)
SIRS Retrospective (AUC=0.793)
SIRS Prospective (AUC=0.797)

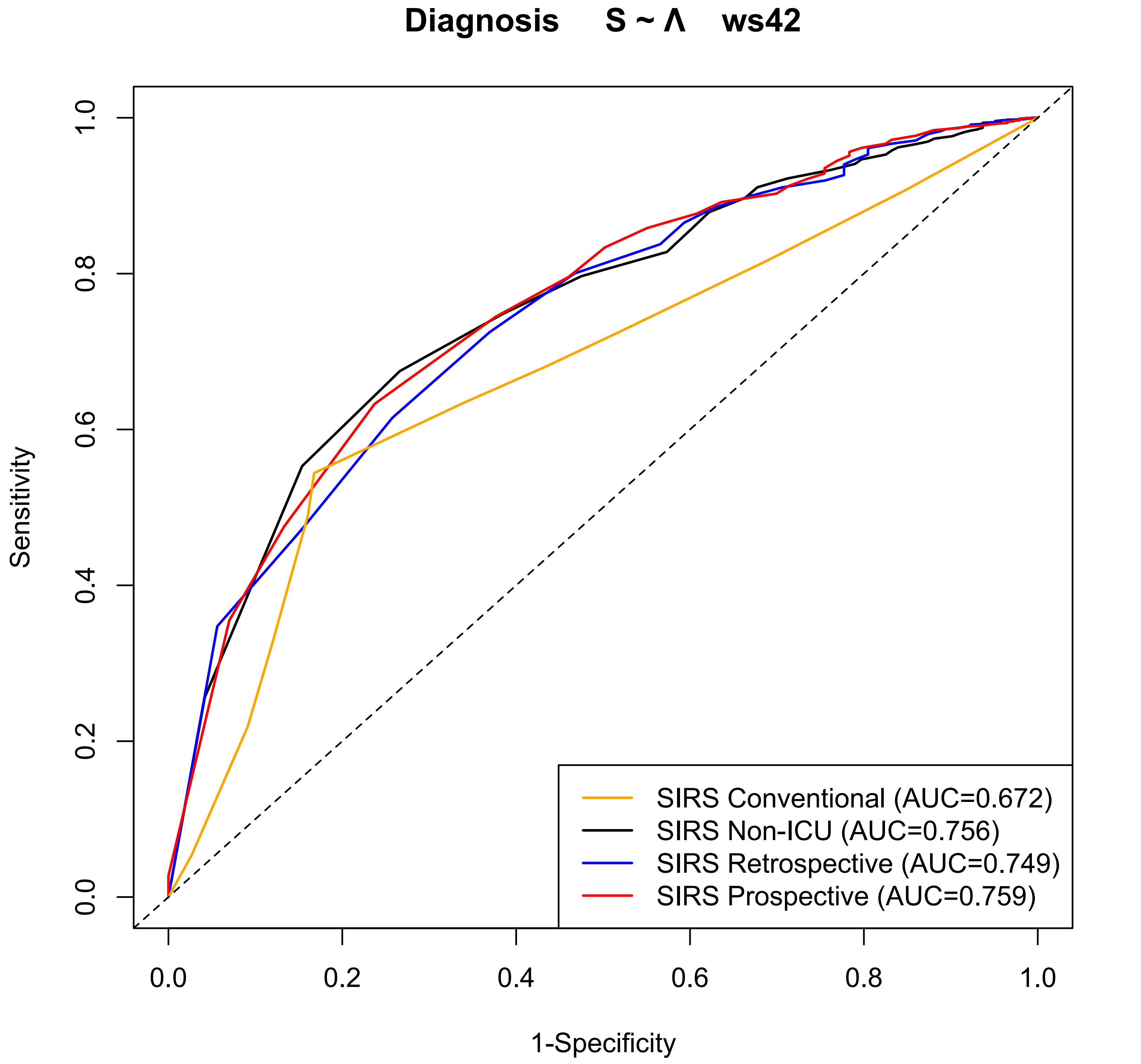
Diagnosis S ~ Λ ws42
Sensitivity
1-Specificity
0.0
0.2
0.4
0.6
0.8
1.0
SIRS Conventional (AUC=0.672)
SIRS Non-ICU (AUC=0.756)
SIRS Retrospective (AUC=0.749)
SIRS Prospective (AUC=0.759)

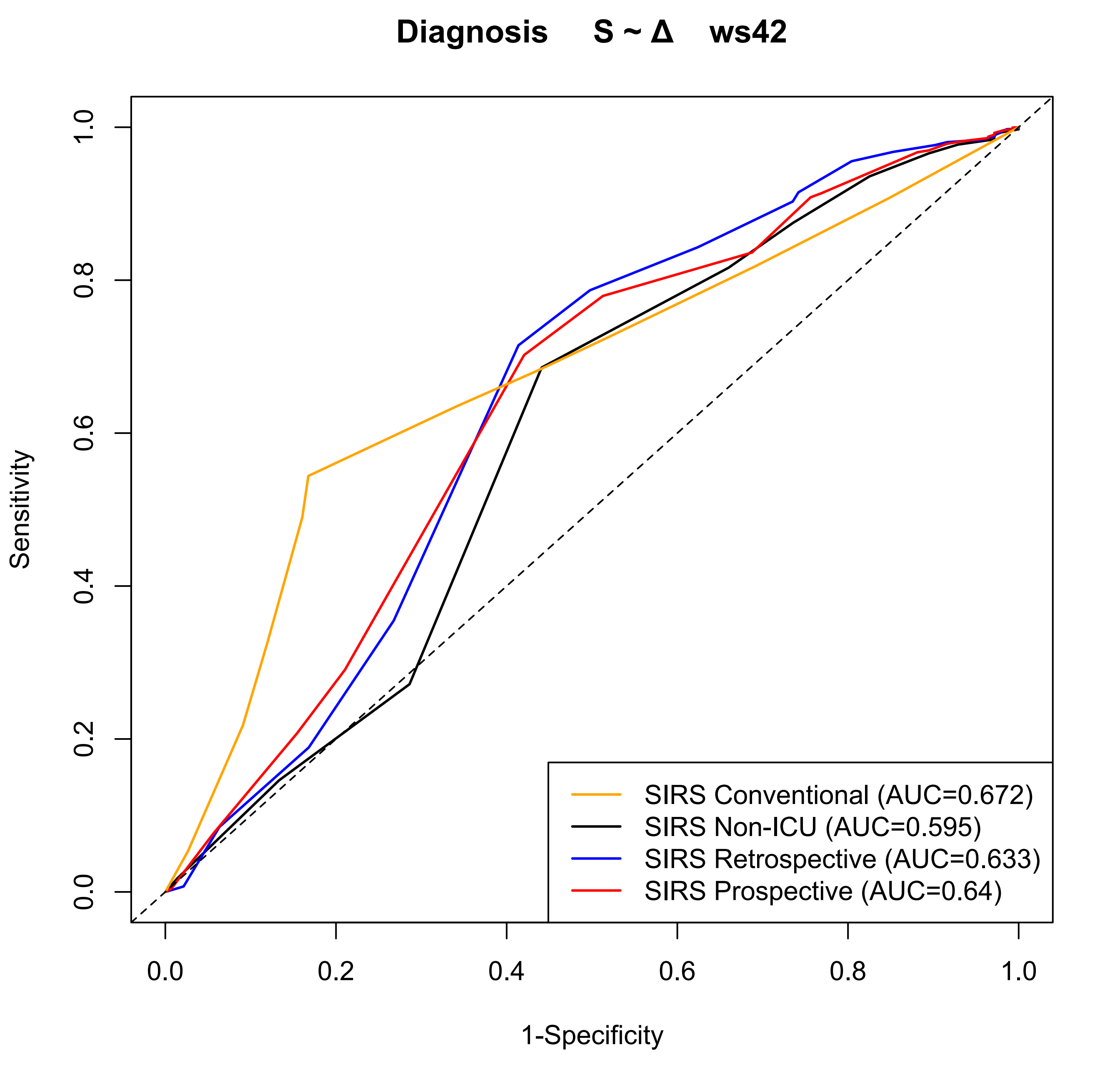

Diagnosis S ~ Δ ws42
Sensitivity
1-Specificity
0.0
0.2
0.4
0.6
0.8
1.0
SIRS Conventional (AUC=0.672)
SIRS Non-ICU (AUC=0.595)
SIRS Retrospective (AUC=0.633)
SIRS Prospective (AUC=0.64)

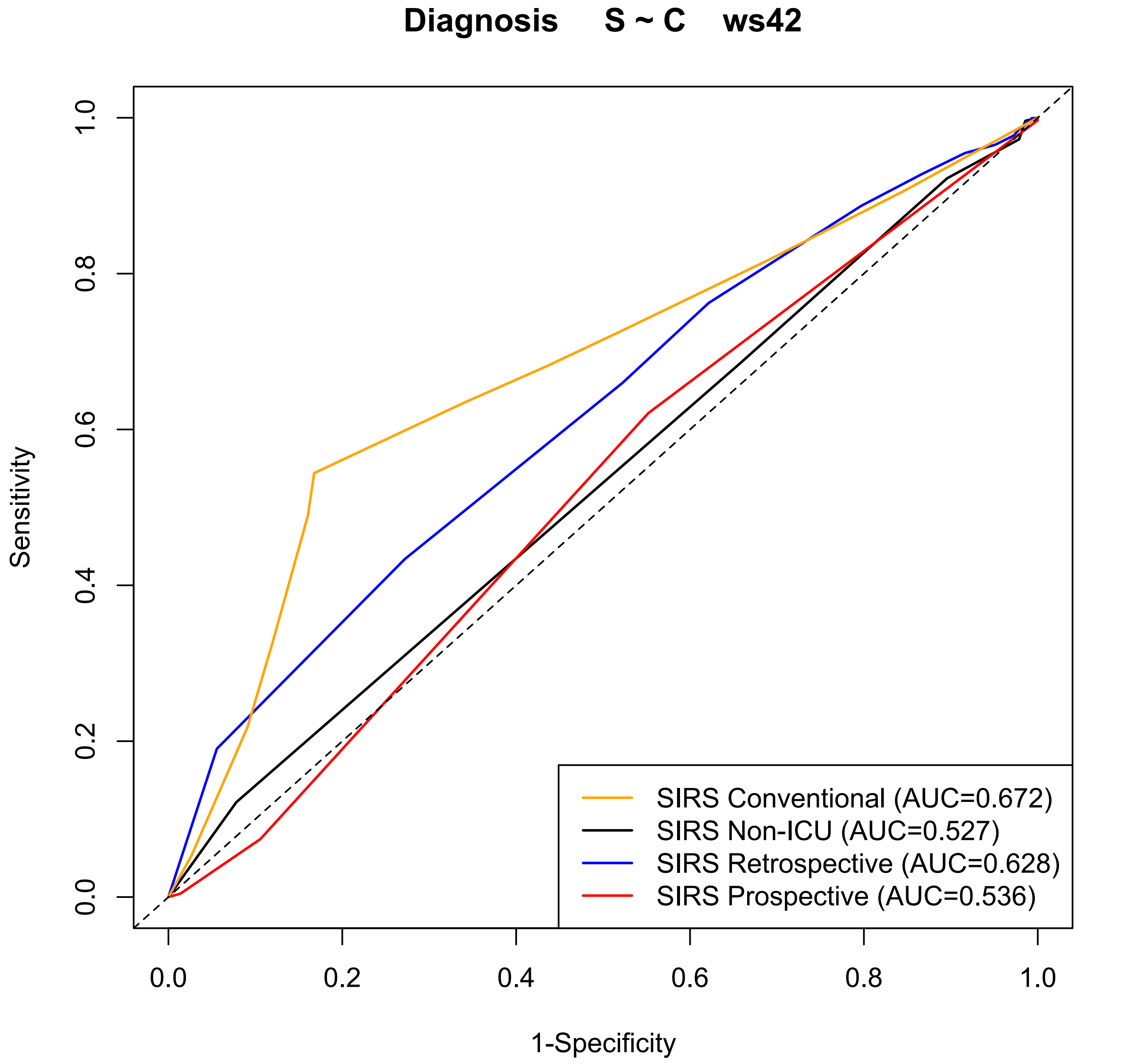

Diagnosis S ~ C ws42
Sensitivity
1-Specificity
0.0
0.2
0.4
0.6
0.8
1.0
SIRS Conventional (AUC=0.672)
SIRS Non-ICU (AUC=0.527)
SIRS Retrospective (AUC=0.628)
SIRS Prospective (AUC=0.536)

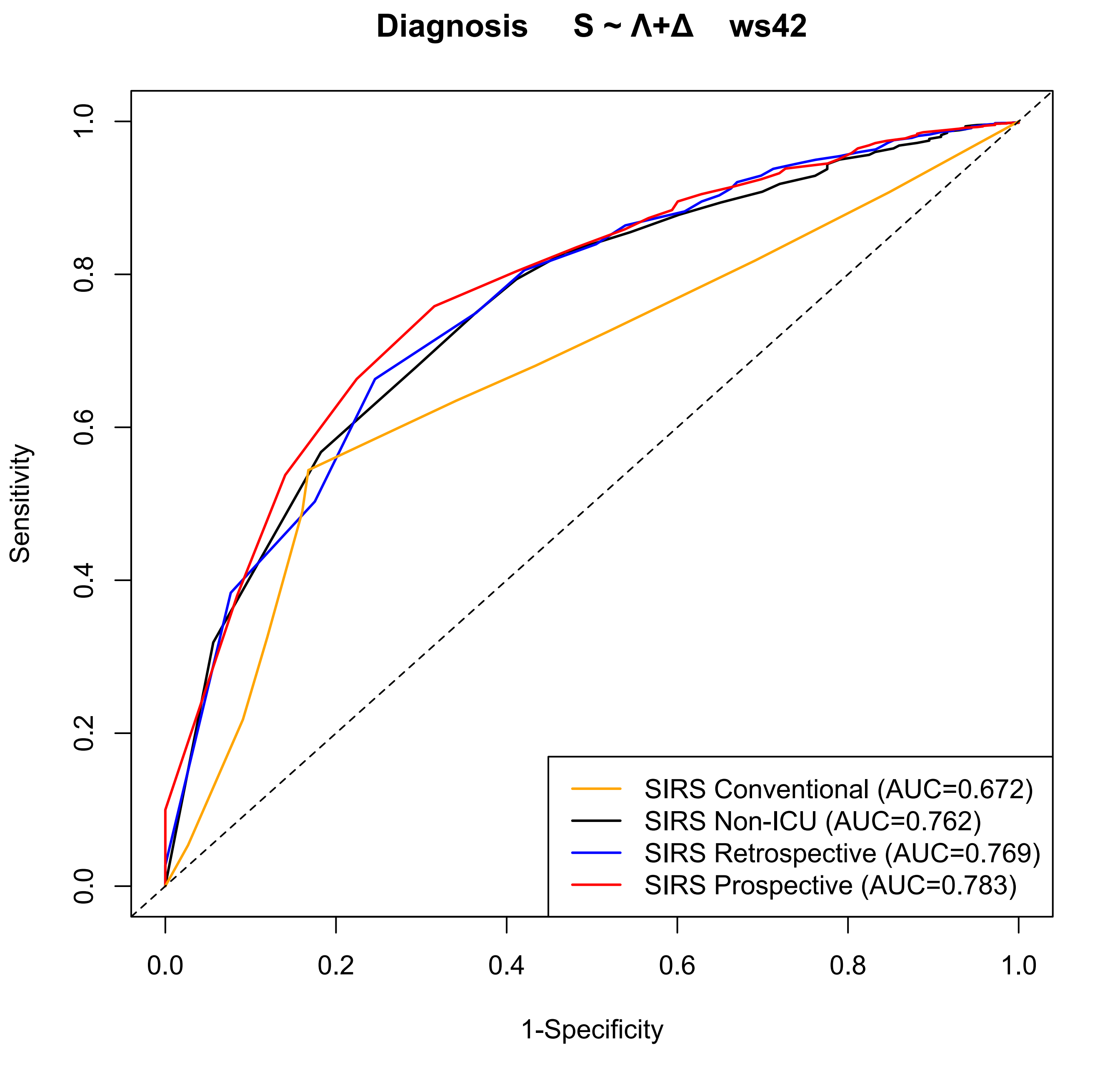

Diagnosis S ~ Λ+Δ ws42
Sensitivity
1-Specificity
0.0
0.2
0.4
0.6
0.8
1.0
0.0
0.2
0.4
0.6
0.8
1.0
SIRS Conventional (AUC=0.672)
SIRS Non-ICU (AUC=0.762)
SIRS Retrospective (AUC=0.769)
SIRS Prospective (AUC=0.783)

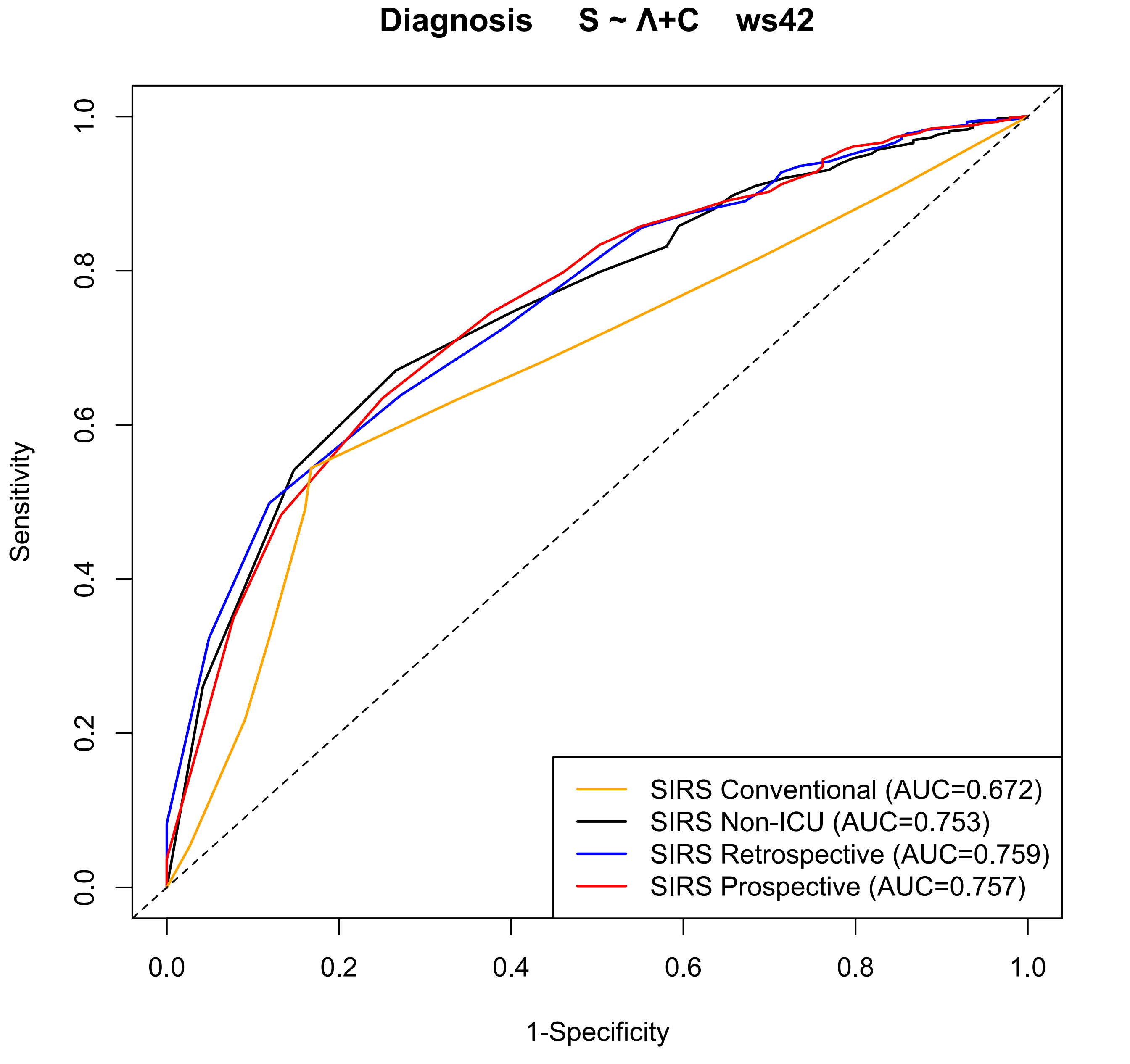
Diagnosis S ~ Λ+C ws42
Sensitivity
1-Specificity
0.0
0.2
0.4
0.6
0.8
1.0
SIRS Conventional (AUC=0.672)
SIRS Non-ICU (AUC=0.753)
SIRS Retrospective (AUC=0.759)
SIRS Prospective (AUC=0.757)

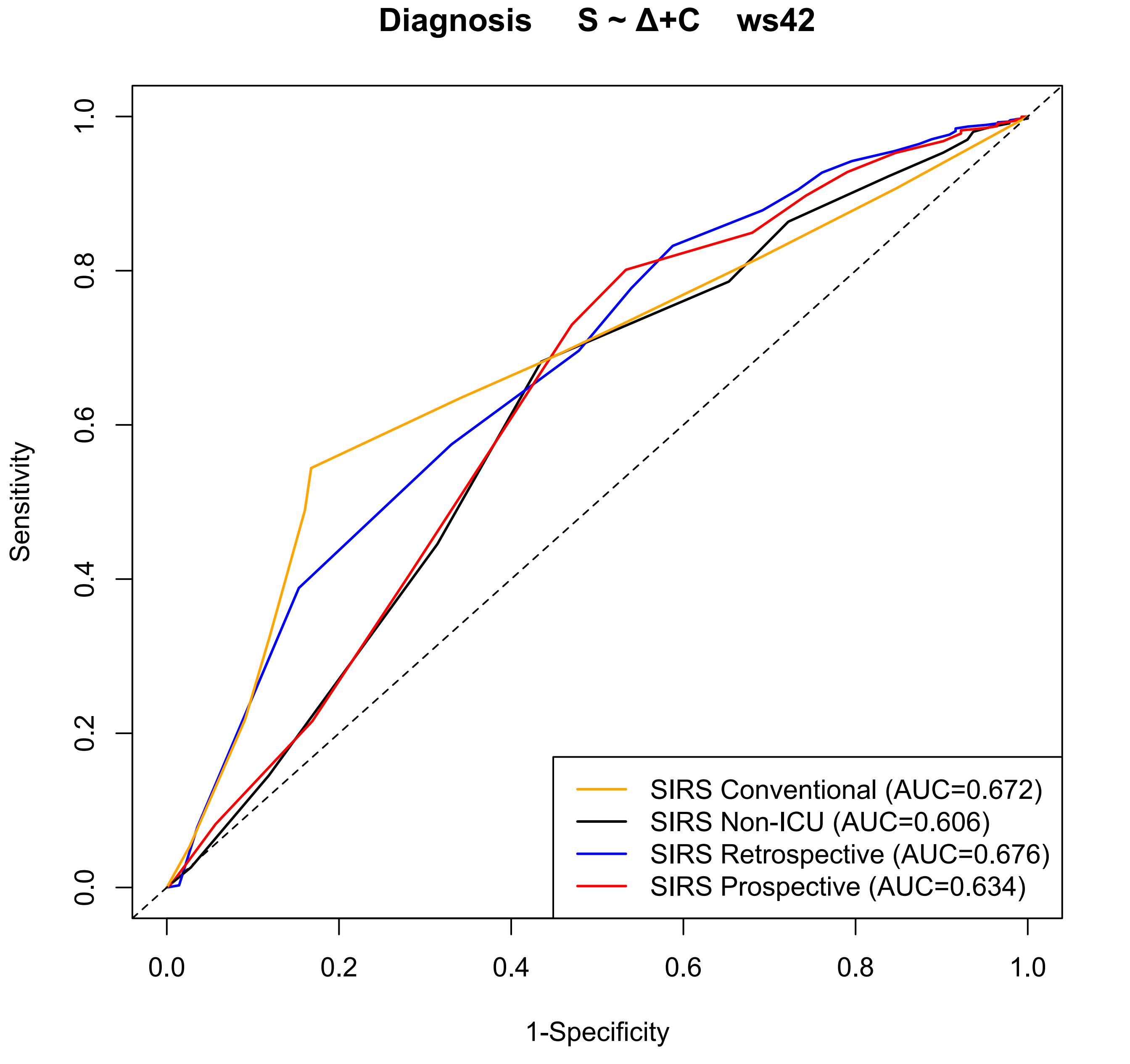

Diagnosis S ~ Δ+C ws42
Sensitivity
1-Specificity
0.0
0.2
0.4
0.6
0.8
1.0
SIRS Conventional (AUC=0.672)
SIRS Non-ICU (AUC=0.606)
SIRS Retrospective (AUC=0.676)
SIRS Prospective (AUC=0.634)

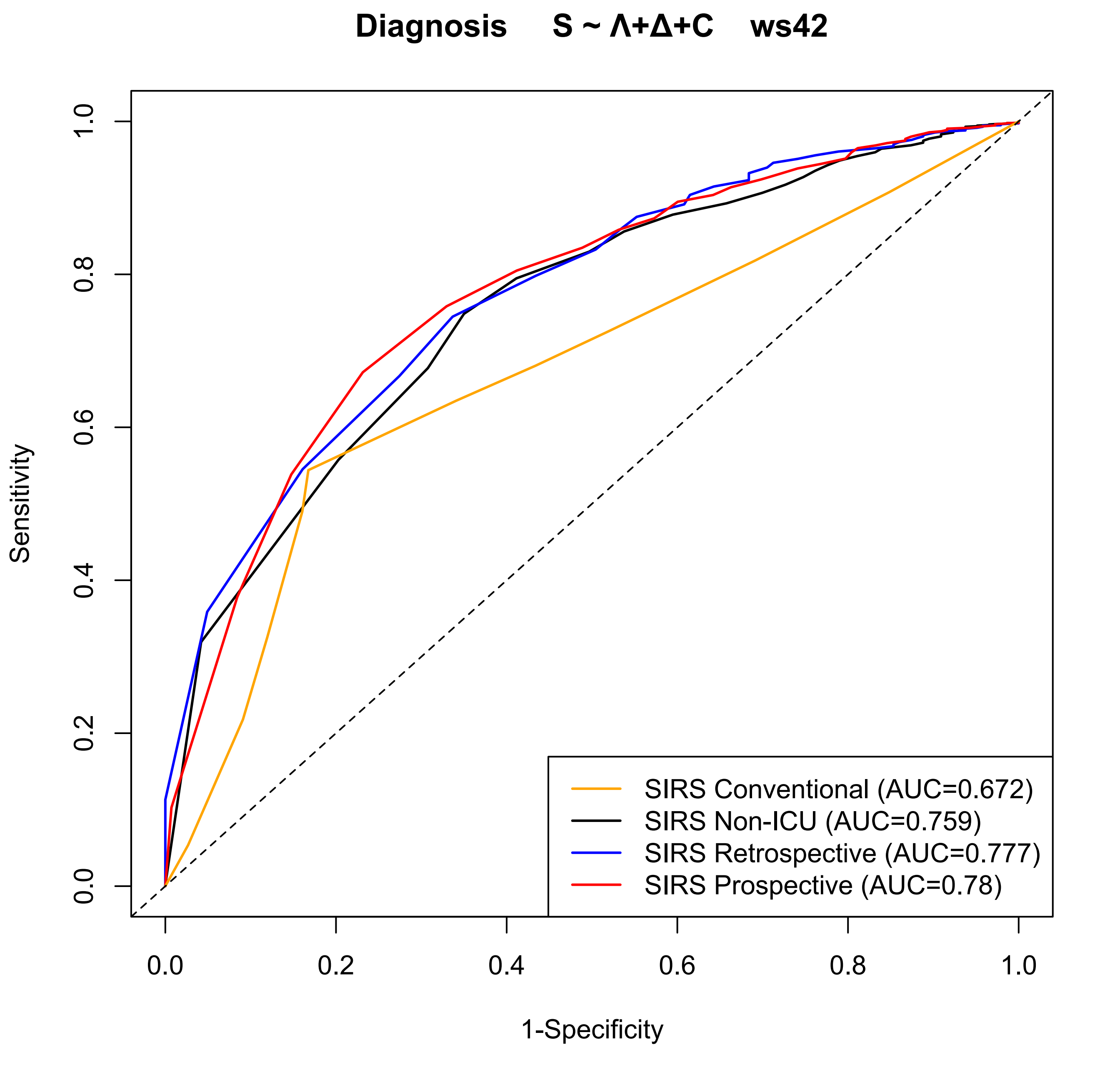
Diagnosis S ~ Λ+Δ+C ws42
Sensitivity
1-Specificity
0.0
0.2
0.4
0.6
0.8
1.0
SIRS Conventional (AUC=0.672)
SIRS Non-ICU (AUC=0.759)
SIRS Retrospective (AUC=0.777)
SIRS Prospective (AUC=0.78)

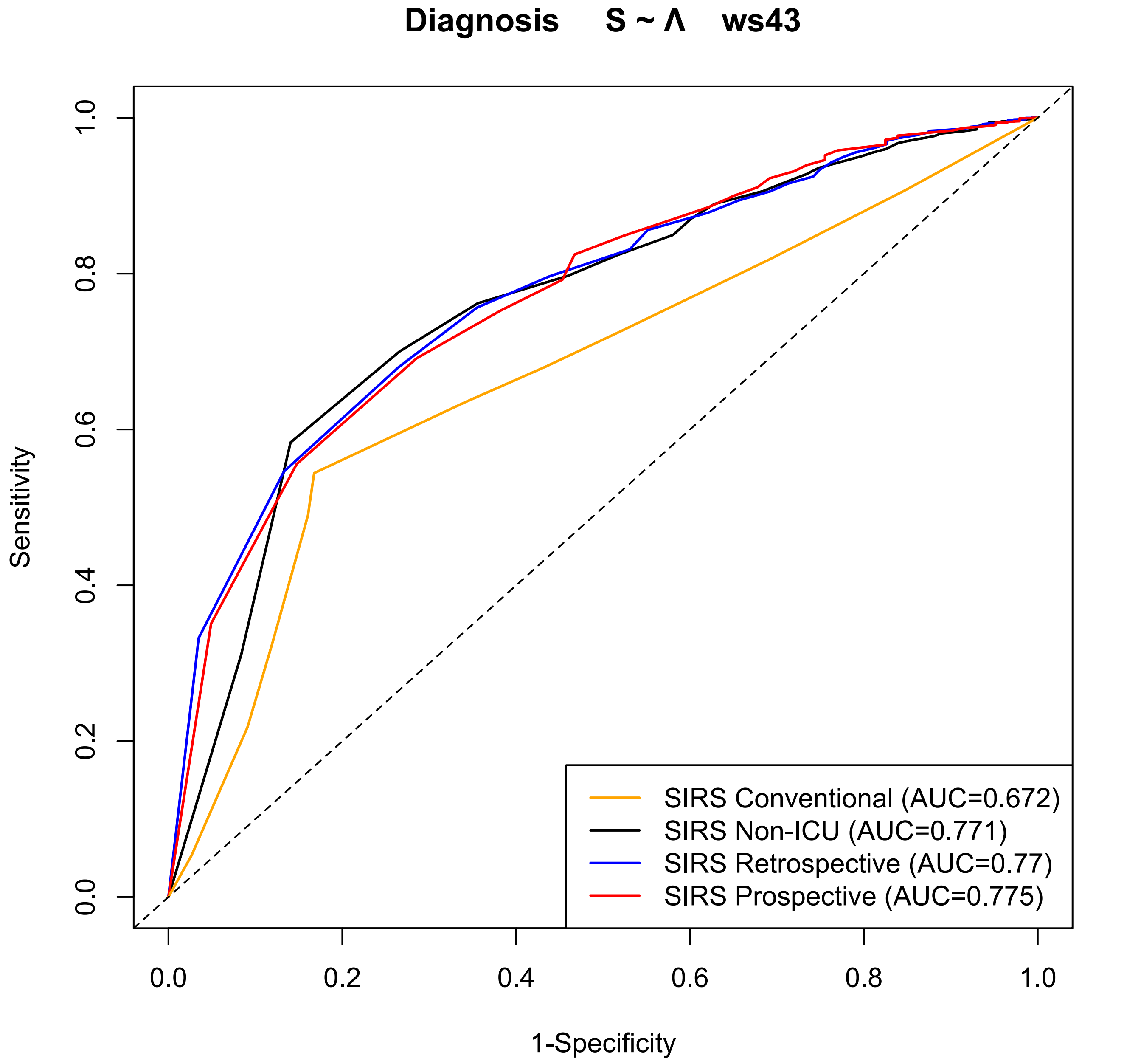

Diagnosis S ~ Λ ws43
Sensitivity
1-Specificity
0.0
0.2
0.4
0.6
0.8
1.0
SIRS Conventional (AUC=0.672)
SIRS Non-ICU (AUC=0.771)
SIRS Retrospective (AUC=0.77)
SIRS Prospective (AUC=0.775)

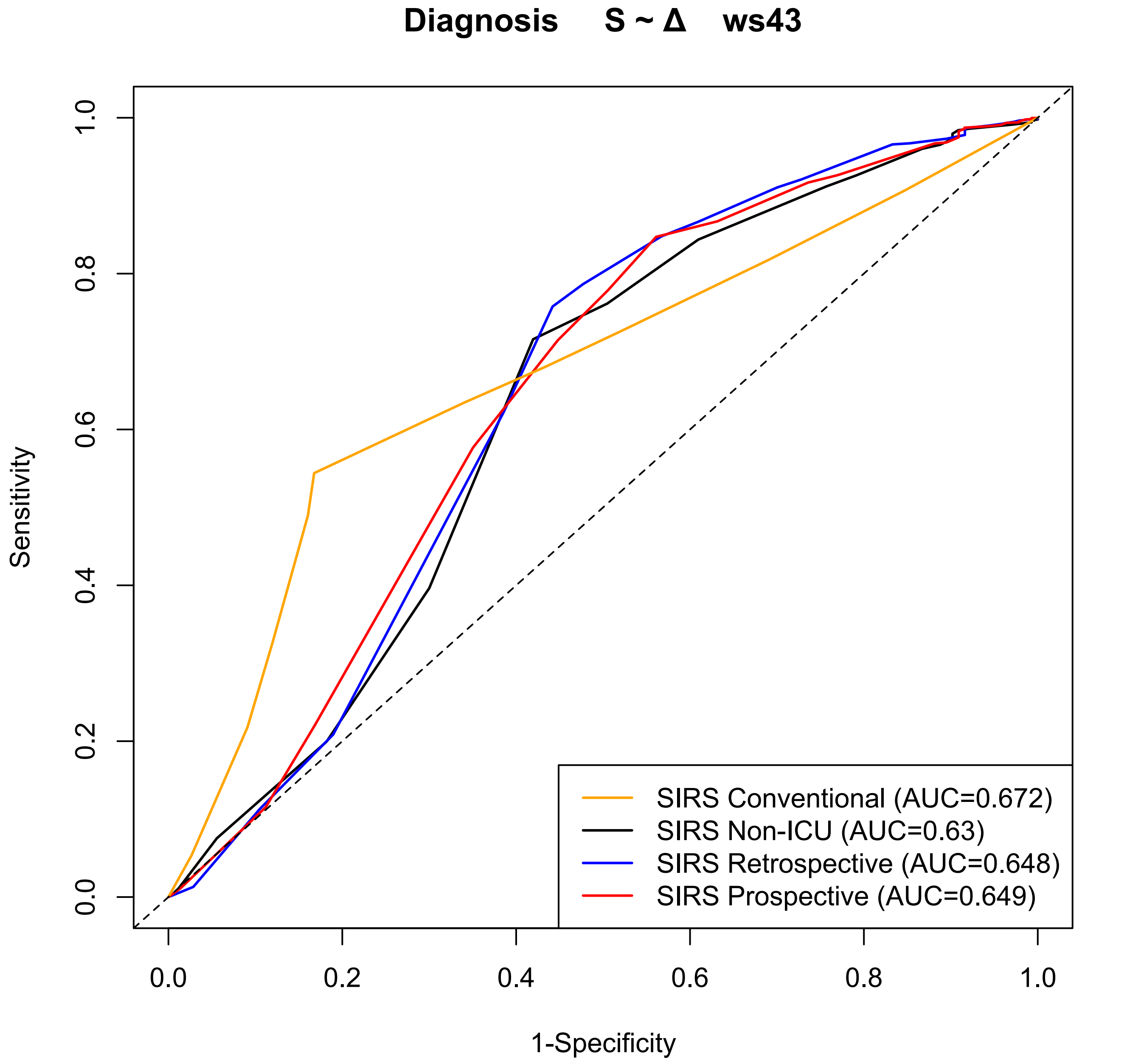
Diagnosis S ~ Δ ws43
Sensitivity
1-Specificity
0.0
0.2
0.4
0.6
0.8
1.0
SIRS Conventional (AUC=0.672)
SIRS Non-ICU (AUC=0.63)
SIRS Retrospective (AUC=0.648)
SIRS Prospective (AUC=0.649)

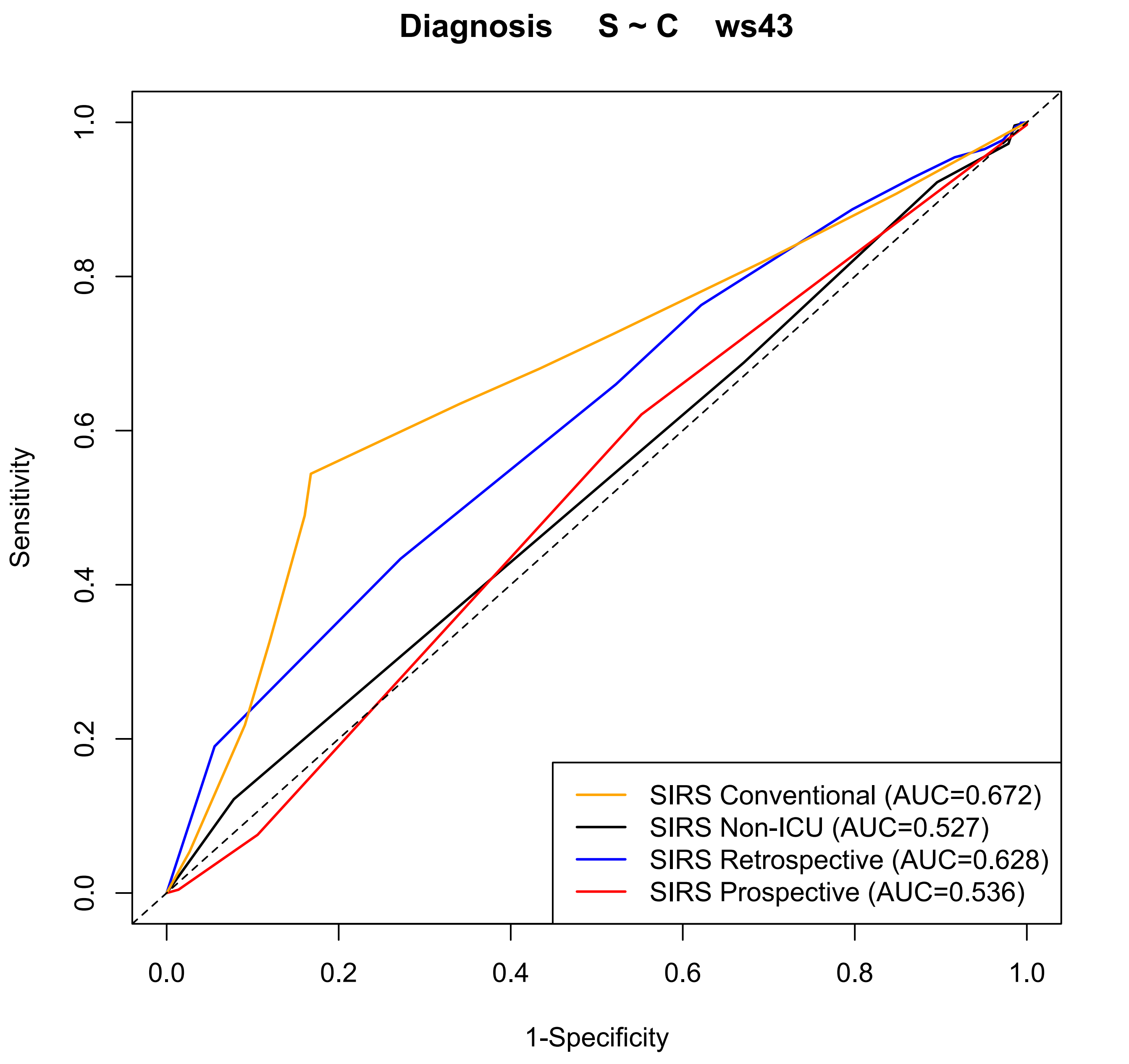

Diagnosis S ~ C ws43
Sensitivity
1-Specificity
0.0
0.2
0.4
0.6
0.8
1.0
SIRS Conventional (AUC=0.672)
SIRS Non-ICU (AUC=0.527)
SIRS Retrospective (AUC=0.628)
SIRS Prospective (AUC=0.536)

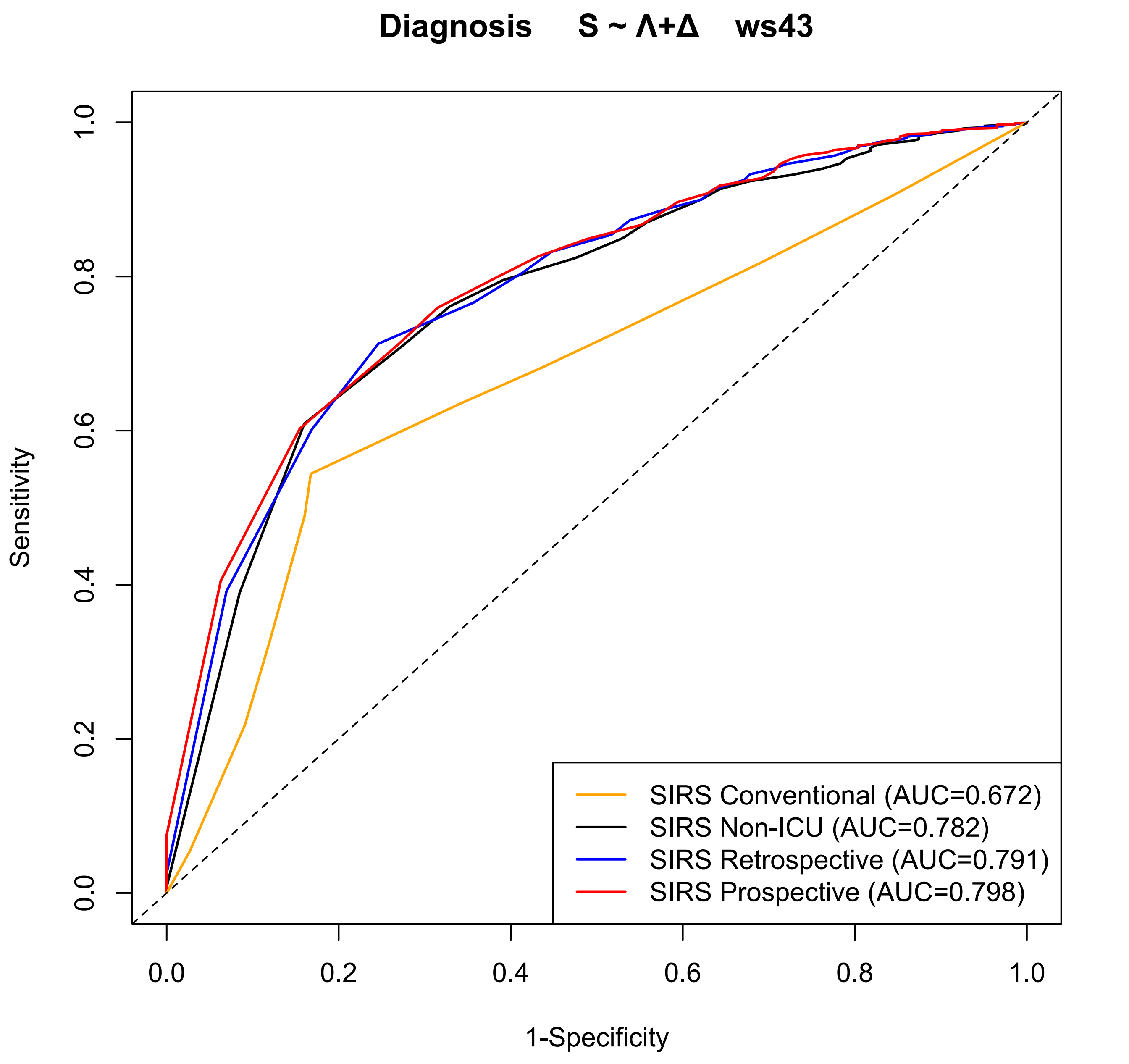

Diagnosis S ~ Λ+Δ ws43
Sensitivity
1-Specificity
0.0
0.2
0.4
0.6
0.8
1.0
SIRS Conventional (AUC=0.672)
SIRS Non-ICU (AUC=0.782)
SIRS Retrospective (AUC=0.791)
SIRS Prospective (AUC=0.798)

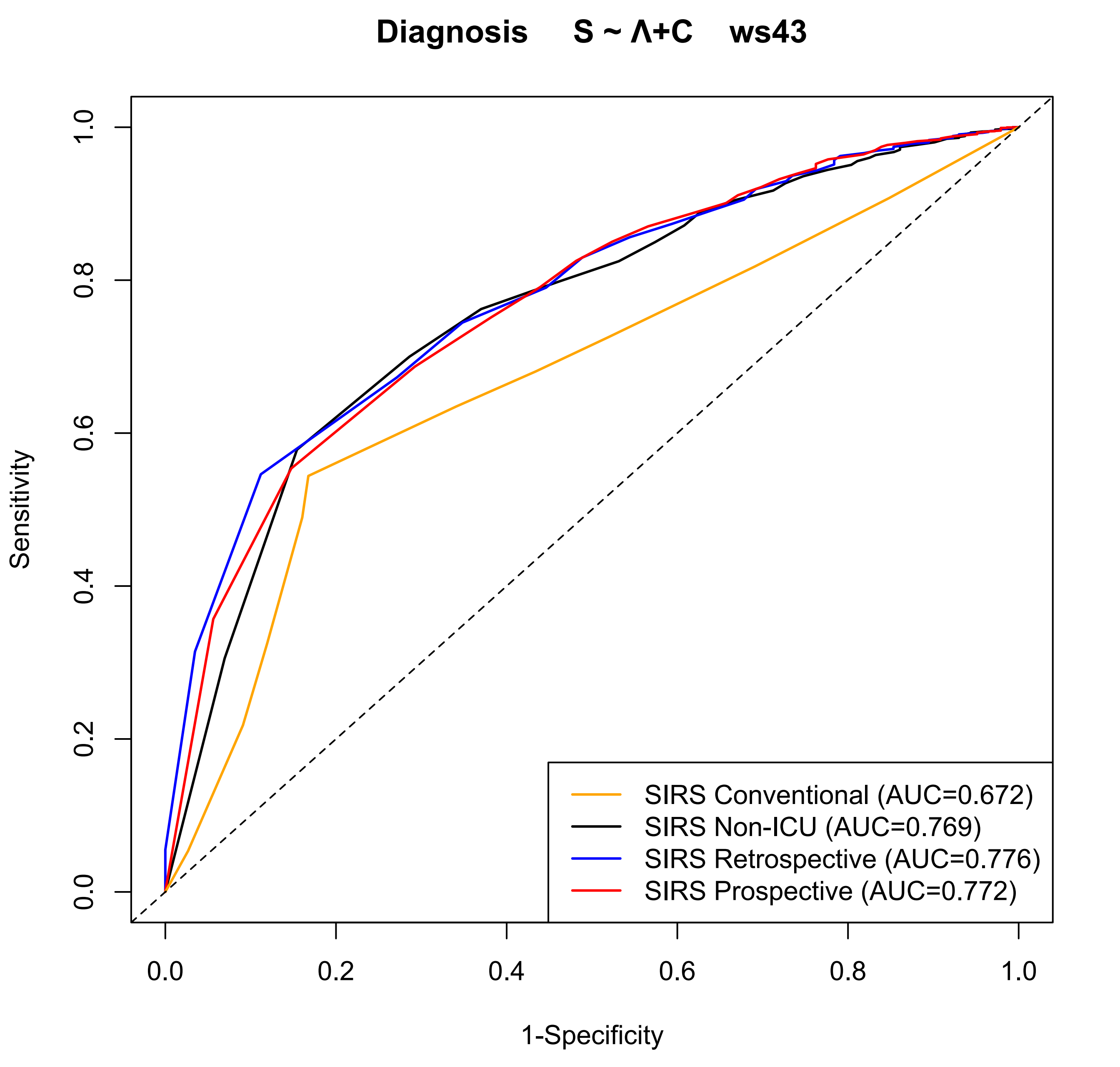
Diagnosis S ~ Λ+C ws43
Sensitivity
1-Specificity
0.0
0.2
0.4
0.6
0.8
1.0
SIRS Conventional (AUC=0.672)
SIRS Non-ICU (AUC=0.769)
SIRS Retrospective (AUC=0.776)
SIRS Prospective (AUC=0.772)

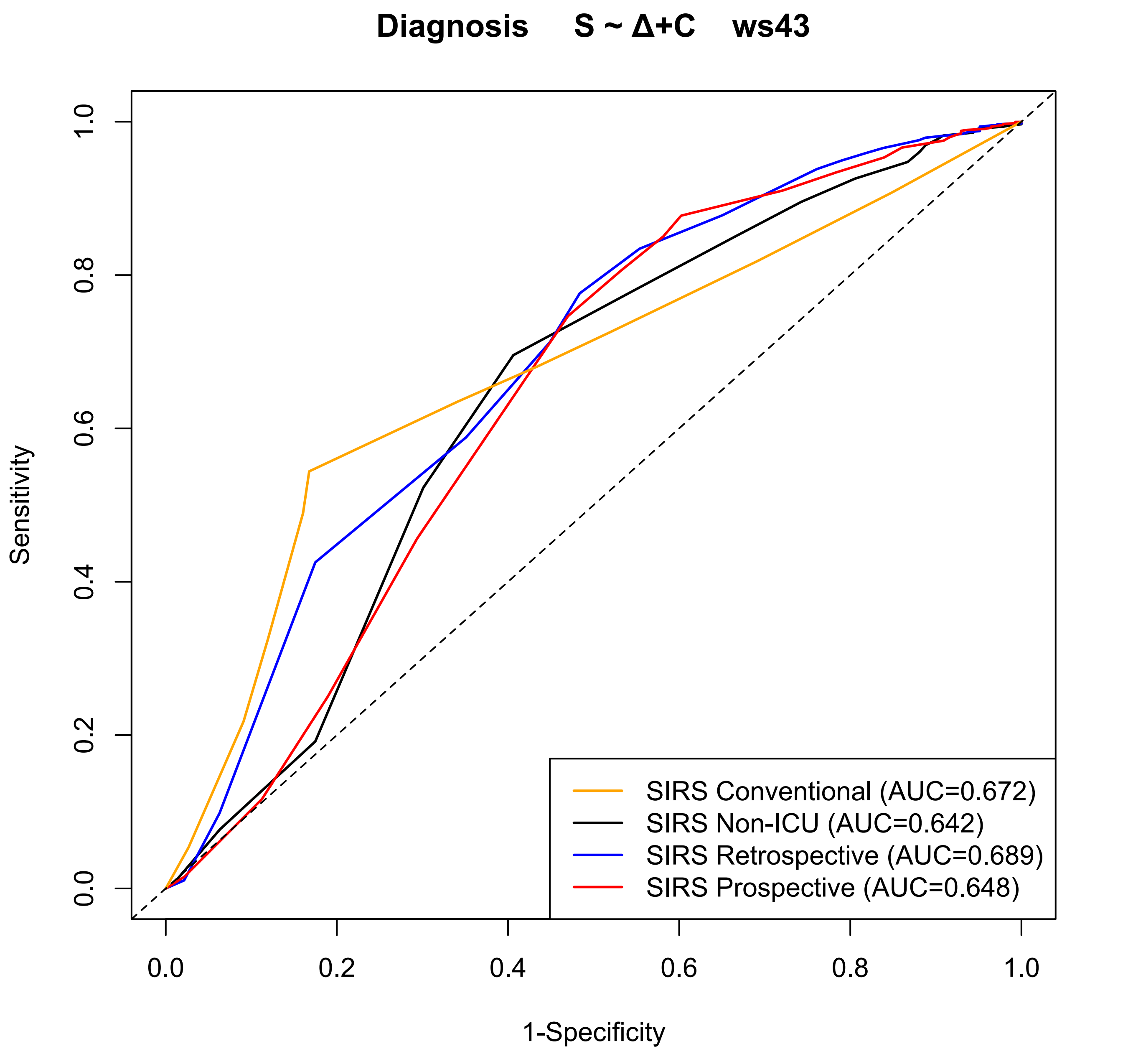

Diagnosis S ~ Δ+C ws43
Sensitivity
1-Specificity
0.0
0.2
0.4
0.6
0.8
1.0
SIRS Conventional (AUC=0.672)
SIRS Non-ICU (AUC=0.642)
SIRS Retrospective (AUC=0.689)
SIRS Prospective (AUC=0.648)

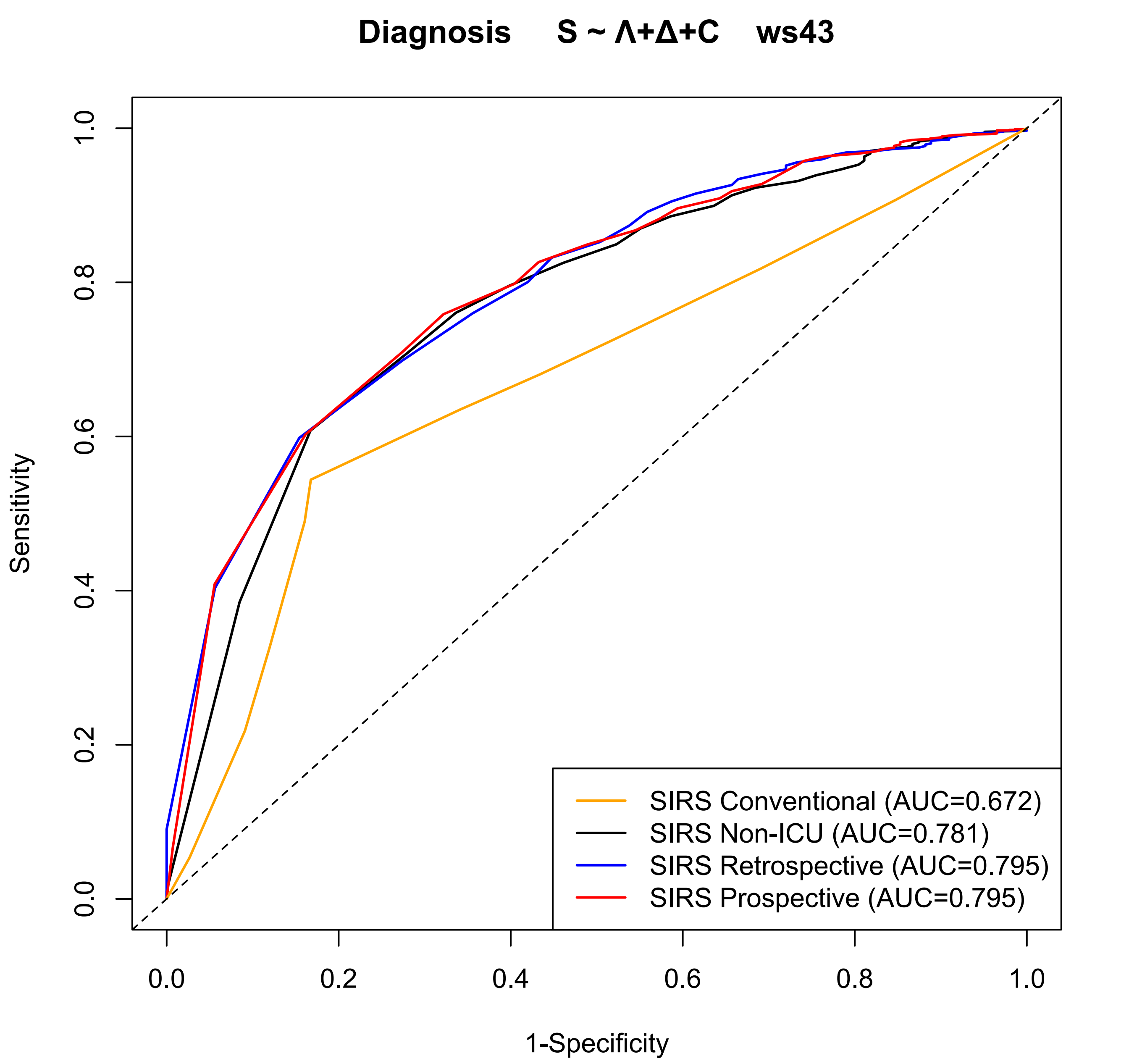

Diagnosis S ~ Λ+Δ+C ws43
Sensitivity
1-Specificity
0.0
0.2
0.4
0.6
0.8
1.0
SIRS Conventional (AUC=0.672)
SIRS Non-ICU (AUC=0.781)
SIRS Retrospective (AUC=0.795)
SIRS Prospective (AUC=0.795)
